# Association of Bone Metastatic Burden With Survival Benefit From Prostate Radiotherapy in Patients With Newly Diagnosed Metastatic Prostate Cancer: A Secondary Analysis of a Randomized Clinical Trial

**DOI:** 10.1001/jamaoncol.2020.7857

**Published:** 2021-02-18

**Authors:** Adnan Ali, Alex Hoyle, Áine M. Haran, Christopher D. Brawley, Adrian Cook, Claire Amos, Joanna Calvert, Hassan Douis, Malcolm D. Mason, David Dearnaley, Gerhardt Attard, Silke Gillessen, Mahesh K. B. Parmar, Christopher C. Parker, Matthew R. Sydes, Nicholas D. James, Noel W. Clarke

**Affiliations:** 1 Genito-Urinary Cancer Research Group, Division of Cancer Sciences, The University of Manchester, Manchester, United Kingdom; 2 FASTMAN Centre of Excellence, Manchester Cancer Research Centre, Manchester, United Kingdom; 3 Department of Surgery, The Christie NHS Foundation Trust, Manchester, United Kingdom; 4 Department of Urology, The Salford NHS Foundation Trust, Manchester, United Kingdom; 5 MRC Clinical Trials Unit at UCL, Institute of Clinical Trials and Methodology, UCL, London, United Kingdom; 6 Department of Radiology, University Hospitals Birmingham NHS Foundation Trust, Birmingham, United Kingdom; 7 Cardiff University, Cardiff, United Kingdom; 8 Royal Marsden Hospital and The Institute of Cancer Research, London, United Kingdom; 9 UCL Cancer Institute, London, United Kingdom; 10 Division of Cancer Sciences, The University of Manchester, Manchester, United Kingdom; 11 Oncology Institute of Southern Switzerland, Bellinzona, Switzerland; 12 Università della Svizzera Italiana, Lugano, Switzerland

## Abstract

**Importance:**

Prostate radiotherapy (RT) improves survival in men with low-burden metastatic prostate cancer. However, owing to the dichotomized nature of metastatic burden criteria, it is not clear how this benefit varies with bone metastasis counts and metastatic site.

**Objective:**

To evaluate the association of bone metastasis count and location with survival benefit from prostate RT.

**Design, Setting, and Participants:**

This exploratory analysis of treatment outcomes based on metastatic site and extent as determined by conventional imaging (computed tomography/magnetic resonance imaging and bone scan) evaluated patients with newly diagnosed metastatic prostate cancer randomized within the STAMPEDE trial’s metastasis M1 RT comparison. The association of baseline bone metastasis counts with overall survival (OS) and failure-free survival (FFS) was assessed using a multivariable fractional polynomial interaction procedure. Further analysis was conducted in subgroups.

**Interventions:**

Patients were randomized to receive either standard of care (androgen deprivation therapy with or without docetaxel) or standard of care and prostate RT.

**Main Outcomes and Measures:**

The primary outcomes were OS and FFS.

**Results:**

A total of 1939 of 2061 men were included (median [interquartile range] age, 68 [63-73] years); 1732 (89%) had bone metastases. Bone metastasis counts were associated with OS and FFS benefit from prostate RT. Survival benefit decreased continuously as the number of bone metastases increased, with benefit most pronounced up to 3 bone metastases. A plot of estimated treatment effect indicated that the upper 95% CI crossed the line of equivalence (hazard ratio [HR], 1) above 3 bone metastases without a detectable change point. Further analysis based on subgroups showed that the magnitude of benefit from the addition of prostate RT was greater in patients with low metastatic burden with only nonregional lymph nodes (M1a) or 3 or fewer bone metastases without visceral metastasis (HR for OS, 0.62; 95% CI, 0.46-0.83; HR for FFS, 0.57; 95% CI, 0.47-0.70) than among patients with 4 or more bone metastases or any visceral/other metastasis (HR for OS, 1.08; 95% CI, 0.91-1.28; interaction *P* = .003; HR for FFS, 0.87; 95% CI, 0.76-0.99; interaction *P* = .002).

**Conclusions and Relevance:**

In this exploratory analysis of a randomized clinical trial, bone metastasis count and metastasis location based on conventional imaging were associated with OS and FFS benefit from prostate RT in M1 disease.

**Trial Registration:**

ClinicalTrials.gov Identifier: NCT00268476; ISRCTN.com Identifier: ISRCTN78818544

## Key Points

**Question:**

Are bone metastatic burden and site associated with survival benefit from the addition of prostate radiotherapy (RT) to standard-of-care systemic therapy in newly diagnosed metastatic prostate cancer?

**Findings:**

This exploratory analysis of 1939 participants in a randomized clinical trial shows that survival benefit following prostate RT gradually diminished with increasing bone metastasis number, with survival benefit most pronounced in patients with up to 3 bone metastases. Prostate RT was associated with greater overall and failure-free survival in patients with only nonregional lymph node metastasis (M1a) or 3 or fewer bone metastases without visceral metastasis.

**Meaning:**

In patients with prostate cancer, bone metastatic burden and metastasis location may be useful in predicting survival benefit from prostate RT.

## Teaser

This exploratory analysis of a randomized clinical trial evaluates the association of bone metastasis count and location with survival benefit from prostate radiotherapy in patients with newly diagnosed metastatic prostate cancer.

## Introduction

Two randomized clinical trials, HORRAD and STAMPEDE, confirmed that prostate radiotherapy (RT) improves survival in newly diagnosed, low-metastatic-burden prostate cancer.^1,2,3^ These results have established a broad consensus for addition of prostate RT to standard of care (SOC) for first-line treatment in men with newly diagnosed, low-metastatic-burden disease.^4,5,6,7^ However, controversy exists on how to define low metastatic burden.^7^ Most criteria dichotomize into low-burden or high-burden subgroups using combined factors with differing thresholds based on bone metastasis counts; these have previously been identified as prognostic in patients with prostate cancer treated with systemic therapy.^8,9,10,11,12^ Therefore, the threshold effects of bone metastatic burden for selecting men with newly diagnosed metastatic prostate cancer (mPCa) who might benefit from prostate RT have not been evaluated systematically. Also, owing to the historical nature of setting criteria for metastatic burden, the role of prostate RT in treatment for men presenting with only nonregional lymph node (NRLN) or visceral metastases has not been reported. Herein, we use data from the STAMPEDE trial’s M1 RT comparison^1^ to explore the association of bone metastatic burden and the influence of isolated or concomitant nodal or visceral metastatic sites with treatment outcome following RT.

## Methods

### Study Participants

Patients randomly allocated to the STAMPEDE trial’s M1 RT comparison were eligible for study. The first efficacy results from this comparison have been published previously.^1^ Briefly, patients with newly diagnosed mPCa and no contraindication to RT were randomized 1:1 to SOC or SOC plus prostate RT. Patients underwent baseline staging imaging per study protocol prior to randomization. Metastatic sites at baseline were evaluated by conventional imaging (bone scan and computed tomography/magnetic resonance imaging). Pretreatment bone scans were centralized, and metastasis counts were analyzed retrospectively. Reviewers were blinded to treatment allocation and outcomes as previously reported.^1^ The SOC comprised lifelong androgen deprivation therapy (ADT), with up-front docetaxel permitted in patients randomized after December 17, 2015. Where used, docetaxel was planned as six 3-week cycles of 75 mg/m^2^ with or without prednisolone, 10 mg, daily. Patients allocated to RT received either 55 Gy in 20 daily fractions over 4 weeks or 36 Gy in 6 weekly fractions over 6 weeks. The schedule was nominated before randomization. All patients provided written informed consent. The trial is registered at ClinicalTrials.gov (NCT00268476) and ISRCTN.com (ISRCTN78818544) and had full regulatory, national ethics committee, and local site approval. Full details of the STAMPEDE trial can be found at http://www.stampedetrial.org, and the trial protocol is in Supplement 1.

### Outcomes

The STAMPEDE trial comparison’s primary definitive and intermediate outcome measures were overall survival (OS) and failure-free survival (FFS), and we focus on these outcome measures. Overall survival was defined as the time from randomization to death from any cause, and FFS as the time from randomization to the first of: biochemical failure; progression locally, in lymph nodes or in distant metastases; or death from prostate cancer.^1^ Biochemical failure was defined as a rise in prostate-specific antigen (PSA) level of 50% greater than the lowest reported PSA level within 24 weeks of enrollment and greater than 4 ng/mL (to convert to μg/L, multiply by 1.0); patients without a decrease of 50% were considered to have biochemical failures at time zero. Patients without the event of interest were censored at the time last known to be event free. Secondary outcomes are described in eMethods in Supplement 2. The outcomes data set frozen for the STAMPEDE M1 RT comparison was used for survival analyses.^1^

### Statistical Analysis

All analyses conducted herein are exploratory. To evaluate whether treatment effect varied by bone metastasis count, a multivariable fractional polynomial interaction (MFPI) algorithm using a nested Cox regression model was performed. Cox models were adjusted for minimization factors used at randomization: age (<70 or ≥70 years), N stage (N0/N+/NX), World Health Organization performance status (0 or 1-2), nonsteroidal anti-inflammatory drug or aspirin use (either or no), and planned docetaxel use (yes/no), along with metastatic site (only NRLN, bone ± NRLN or any visceral/other metastasis). Models with first-degree fractional polynomial, second-degree fractional polynomial, and linear functions of bone metastasis counts were evaluated, and the interaction model with the lowest Bayesian information criterion and Akaike information criterion was chosen (see eMethods in Supplement 2). A *P* value from a likelihood ratio test of the interaction between treatment group and bone metastasis count is presented. The MFPI model–estimated treatment effect as a function of bone metastasis count was plotted graphically on the HR scale with 95% CI. Further details regarding the MFPI have been published previously.^13,14^ Sensitivity analysis was also undertaken using Cox models adjusted for selected clinically relevant baseline variables: age, pre-ADT PSA level, World Health Organization performance status, T stage, Gleason score, N stage, planned docetaxel use, nominated RT schedule, and metastatic sites.

As a check for interactions identified using MFPI procedures, we conducted 3 further analyses. We evaluated treatment effects within nonoverlapping subpopulations based on bone metastasis count. If there were insufficient numbers of patients within subpopulations based on bone metastasis count, we collapsed them to achieve groups of reasonable size. We then conducted further analysis within subgroups based on bone metastasis count cutoff and metastatic sites. Four subgroups were created based on these parameters: only NRLN metastasis (M1a), 3 or fewer and 4 or more bone metastases (±NRLN), and any visceral/other metastasis. Balance regarding baseline characteristics between treatment arms was evaluated within each subgroup. Kaplan-Meier (KM) estimates were used to plot survival curves, and relative treatment effects were evaluated using Cox models within the subgroups. Finally, based on information obtained from the previous steps, a metastatic burden classification was devised and evaluated as detailed in eMethods in Supplement 2. An HR below 1 favored the prostate RT group. Median follow-up was determined through reverse-censoring on death. Statistical analyses were performed using Stata, version 15.1 (StataCorp).

## Results

### Patient Cohort

**Figure 1.**
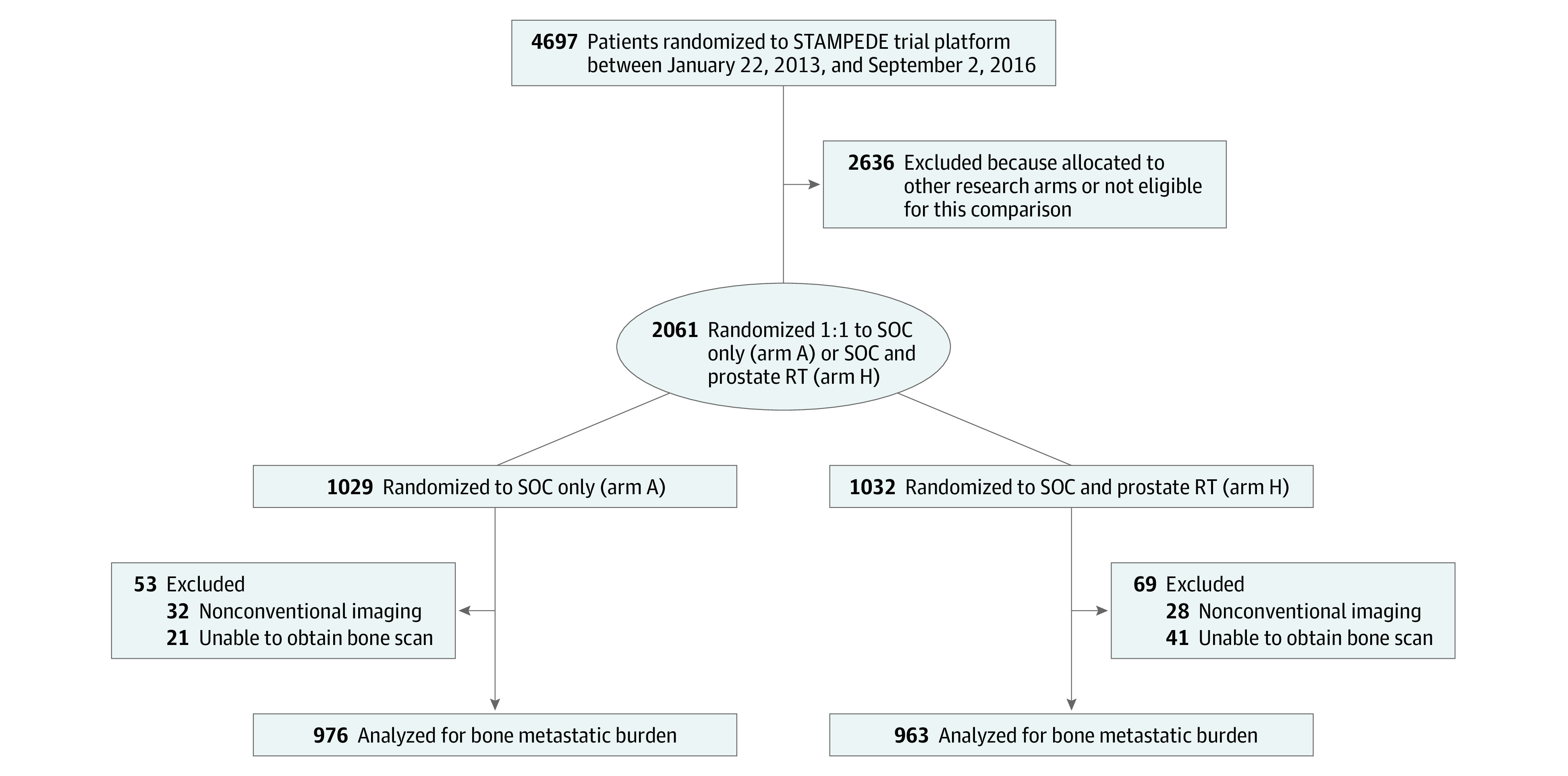
Flowchart Showing Inclusion of Patients for Bone Metastatic Burden Analysis RT indicates radiotherapy; SOC, standard of care.

Following exclusion of patients undergoing nonconventional imaging (n = 60) and where baseline bone scans could not be centralized (n = 62), baseline bone scans from 1939 of 2061 (94%) patients with newly diagnosed mPCa randomized between January 22, 2013, and September 2, 2016, in the STAMPEDE M1 RT comparison were included (Figure 1). Baseline characteristics were balanced between the SOC and the SOC plus RT groups (eTable 1 in Supplement 2) and were representative of the M1 RT comparison (eTable 2 in Supplement 2). The median (interquartile range [IQR]) age was 68 (63-73) years and the median (IQR) PSA level pre-ADT was 98 (33-313) ng/mL. Of the 1939 patients included, 1587 (82%) had bone metastases with or without additional NRLN metastasis, 181 (9%) had only NRLN metastasis (M1a), and 171 (9%) had visceral/other metastasis. Median (IQR) follow-up was 37 (24-48) months.

### Bone Metastasis Count–Treatment Interaction

**Figure 2.**
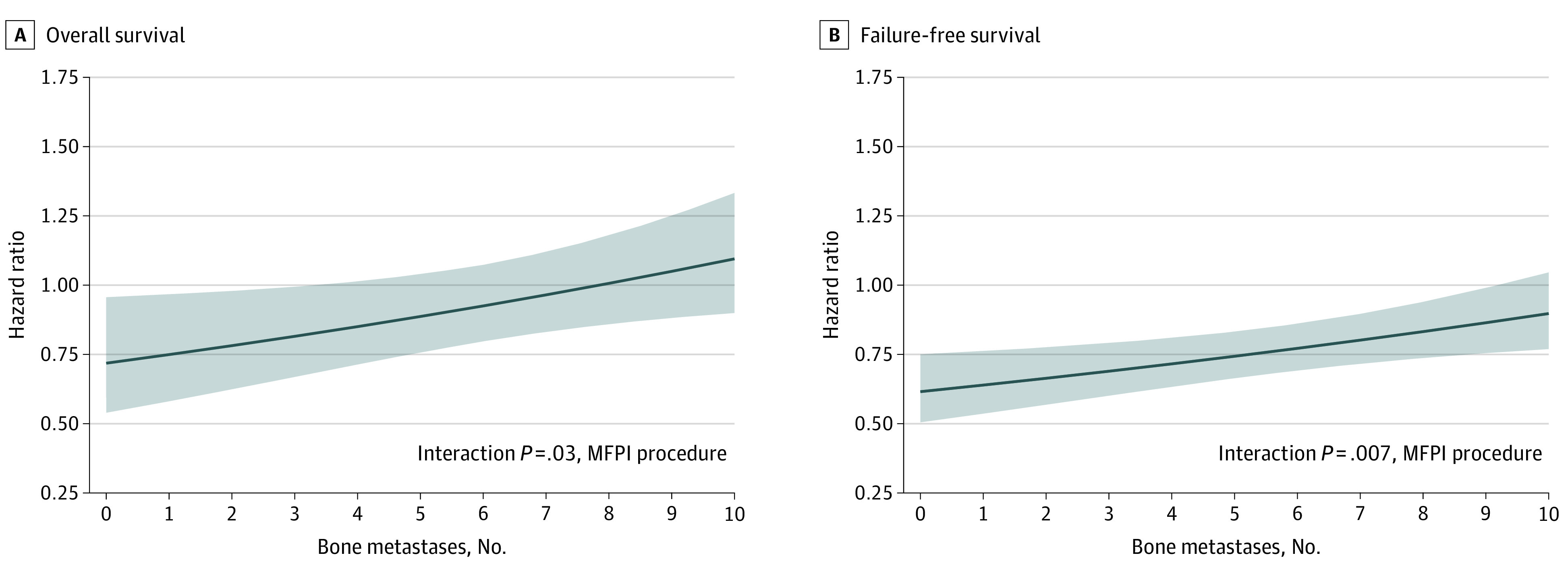
Treatment Effect Plots for Bone Metastasis Count Estimated treatment effect (solid line) with pointwise 95% CI (shaded area) is shown for overall survival (A) and failure-free survival (B). The horizontal gray line at hazard ratio 1.00 denotes equivalence of treatment effects, with values below 1.00 favoring prostate radiotherapy. MFPI indicates multivariable fractional polynomial interaction.

Using the MFPI procedure, the linear model had the lowest Bayesian information criterion and Akaike information criterion for both OS and FFS outcomes (eResults and eTable 3 in Supplement 2). There was evidence of heterogeneity of treatment effect on survival for bone metastasis count. A plot of estimated treatment effect indicated that the survival benefit from prostate RT decreased gradually with increasing bone metastasis counts. Good evidence of survival benefit with addition of prostate RT was seen up to 3 bone metastases, with the upper 95% CI crossing the line of equivalence (HR, 1) just after this (Figure 2A). Evaluation of relative treatment effect in nonoverlapping subpopulations based on bone metastasis counts also showed an HR less than 1 in subpopulations with 3 or fewer bone metastases (eFigure 1 in Supplement 2). In subpopulations of patients with 1, 2, and 3 bone metastases, prostate RT was associated with an absolute improvement of 8.5%, 6.2%, and 5.8% in 3-year KM estimated survival, respectively (eFigure 2A in Supplement 2). Beyond 4 bone metastases, the estimated survival benefit from prostate RT decreased continuously, with the point estimate crossing the line of equivalence at 8 bone metastases. Although the treatment effect plot suggested some survival benefit in patients with 4 to 7 bone metastases, this was not evident in the analysis based on subgroups and subpopulations (eTable 4, eFigure 1, and eFigure 2 in Supplement 2). Similarly, for FFS, there was good evidence of a treatment interaction with bone metastasis count, with the upper 95% CI crossing the line of equivalence at around 9 bone metastases (Figure 2B). Prostate RT was associated with absolute improvements of 21.5%, 10.1%, 14.2% and 8.84% in KM estimated 3-year FFS rates in subpopulations of patients with 1, 2, 3, and 4 bone metastases, respectively (eFigure 2B in Supplement 2). A sensitivity analysis evaluating the interaction of bone metastasis count with treatment in a multivariable Cox model adjusted for age, pre-ADT PSA level, T stage, Gleason score, N stage, metastatic sites, planned docetaxel use, and nominated RT schedule also yielded similar results for OS and FFS.

**Figure 3.**
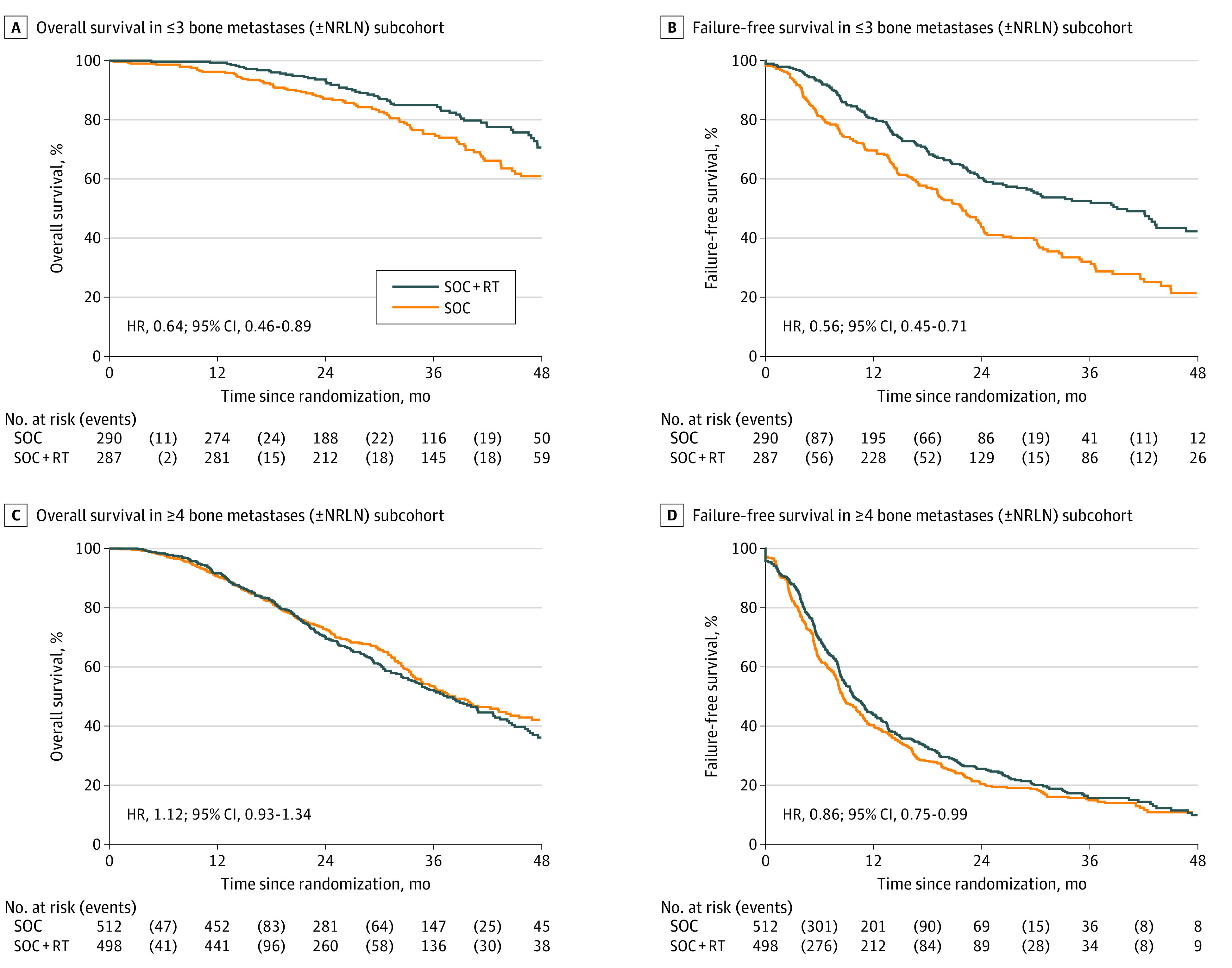
Kaplan-Meier Curves for Overall and Failure-Free Survival by Treatment in 1587 Patients With Bone Metastases Overall and failure-free survival by treatment in 1587 patients with bone metastases with or without nonregional lymph node metastasis (NRLN) metastasis stratified by ≤3 (A and B) and ≥4 (C and D) bone metastases. RT indicates radiotherapy; SOC, standard of care.

We further checked the interaction of bone metastasis count with treatment outcomes as identified previously by evaluating treatment effects in the 1587 patients with bone metastases with or without NRLN, split into 2 subgroups defined by bone metastasis count. A cut point of 3 bone metastases was chosen based on the threshold identified from the prior MFPI results (for baseline characteristics in subgroups, see eTable 5 in Supplement 2). In the 577 patients with 3 or fewer bone metastases with or without NRLN and no visceral metastasis, prostate RT improved survival (HR, 0.64; 95% CI, 0.46-0.89; 3-year KM estimated survival, 85% with SOC plus RT and 75% with SOC). There was no evidence of survival benefit from prostate RT in patients with 4 or more bone metastases with or without NRLN (HR, 1.12; 95% CI, 0.93-1.34) (Table and Figure 3). A sensitivity analysis conducted in 1287 patients with only bone metastases after excluding patients with any NRLN or visceral/other metastasis also confirmed this (eTable 6 and eFigure 3 in Supplement 2).

**Table.** Summary of Estimated Treatment Effects for Overall and Failure-Free Survival in Subgroups

	Events/patients, No./No.		HR (95% CI)^a^	3-y KM survival, %		Interaction by bone metastasis subgroups *P* value
	SOC	SOC + RT		SOC	SOC + RT	
Overall survival						
Only NRLN metastasis	28/89	21/92	0.60 (0.33-1.09)	73	80	
Bone metastases (±NRLN)	303/802	291/785	0.96 (0.82-1.13)	61	64	.006
≤3 bone metastases	81/290	58/287	0.64 (0.46-0.89)	75	85	
≥4 bone metastases	222/512	233/498	1.12 (0.93-1.34)	53	52	
Any visceral or other metastasis	37/85	35/86	0.89 (0.55-1.42)	53	56	
Failure-free survival						
Only NRLN metastasis	54/89	46/92	0.63 (0.42-0.94)	29	51	
Bone metastases (±NRLN)	598/802	532/785	0.75 (0.67-0.85)	22	30	.004
≤3 bone metastases	184/290	135/287	0.56 (0.45-0.71)	33	53	
≥4 bone metastases	414/512	397/498	0.86 (0.75-0.99)	15	16	
Any visceral or other metastasis	68/85	64/86	0.98 (0.68-1.39)	19	20	

Abbreviations: HR, hazard ratio; KM, Kaplan-Meier; NRLN, nonregional lymph node metastasis; RT, radiotherapy; SOC, standard of care.

^a^ HRs and 95% CIs are from Cox proportional hazards models adjusted for age (<70 or ≥70 years), N stage (N0, N+ or NX), World Health Organization performance status (0 or 1-2), nonsteroidal anti-inflammatory drug or aspirin use (uses either or no), and docetaxel use (yes or no). Cox models evaluating treatment effects in the only-NRLN metastasis subgroup were adjusted for all variables as stated above except N stage.

### Only NRLN or Any Visceral/Other Metastasis

**Figure 4.**
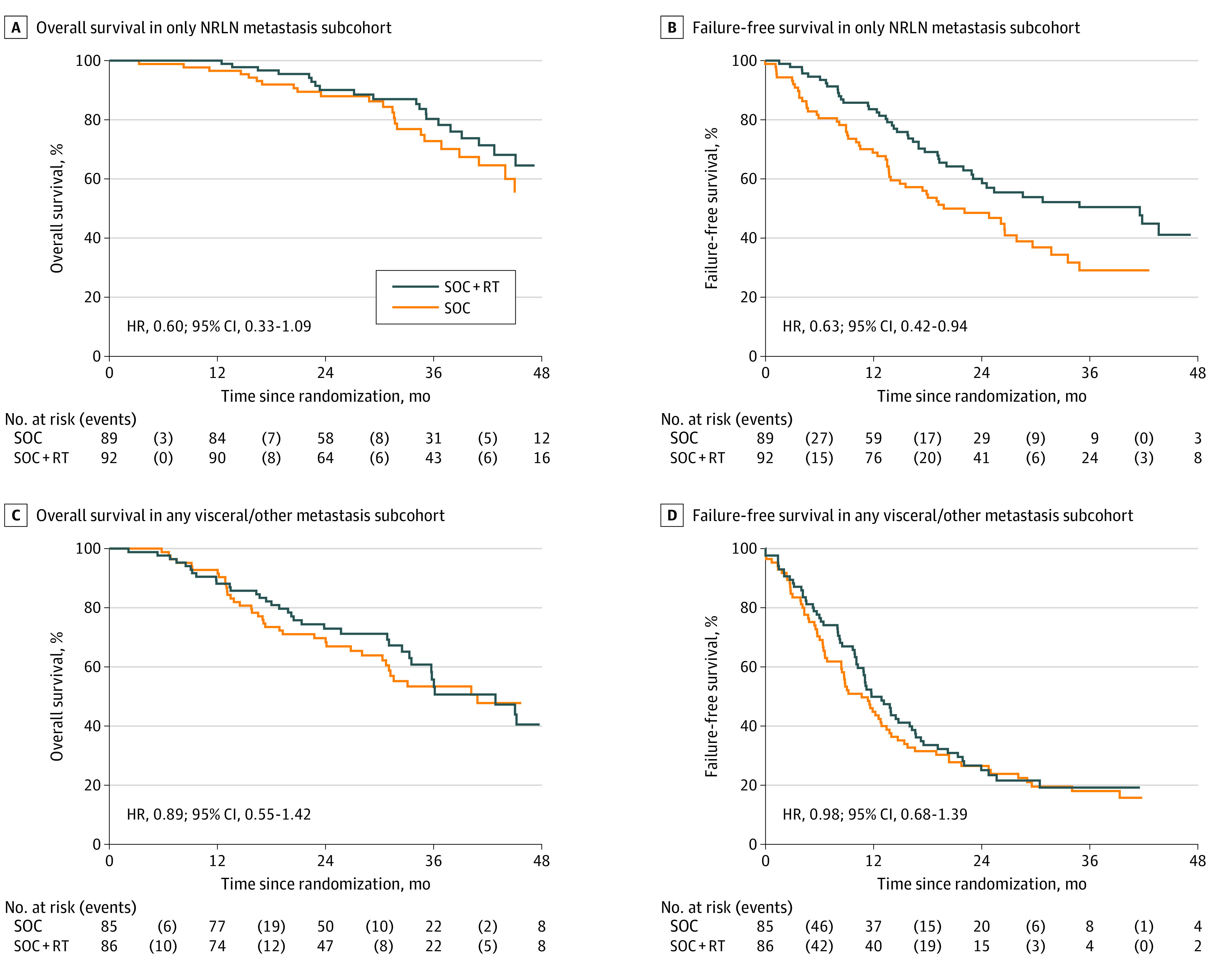
Kaplan-Meier Curves for Overall Survival (OS) and Failure-Free Survival (FFS) by Treatment in Patients With Only Nonregional Lymph Node (NRLN) Metastasis (M1a) and Any Visceral or Other Metastasis Overall and failure-free survival by treatment in patients with only NRLN metastasis (M1a) (A and B) and any visceral or other metastasis (C and D). RT indicates radiotherapy; SOC, standard of care.

Further analysis was undertaken in 181 patients with only NRLN (M1a) and 171 patients with any visceral/other metastasis (for baseline characteristics, see eTables 7 and 8 in Supplement 2). In the subgroup of 181 patients with only NRLN metastasis (M1a), there was a strong indication of survival benefit from prostate RT (HR, 0.60; 95% CI, 0.33-1.09). The absolute improvement in 3-year survival with prostate RT was 7%, from 73% (SOC) to 80% (SOC plus RT) (Table and Figure 4). There was good evidence of improvement in FFS from prostate RT with only NRLN metastasis (HR, 0.63; 95% CI, 0.42-0.94; absolute improvement in 3-year KM estimated FFS of 22%, from 29% with SOC to 51% with SOC plus RT). Similar analysis in patients with any visceral/other metastasis showed no evidence of benefit from adding prostate RT on OS or FFS (OS HR, 0.89; 95% CI, 0.55-1.42; FFS HR, 0.98; 95% CI, 0.68-1.39) (Table and Figure 4).

### Metastatic Burden Classification

Based on the aforementioned results, a metastatic burden classification was devised, wherein low burden was defined as patients with only NRLN or 3 or fewer bone metastases with or without NRLN regardless of axial or extra axial location and without any visceral/other metastasis. All others fell in to a high-burden category. Prostate RT improved OS and FFS in patients with low-metastatic-burden disease (OS HR, 0.62; 95% CI, 0.46-0.83, *P* = .001; FFS HR, 0.57; 95% CI, 0.47-0.70, *P* < .001) (eTable 9 and eFigure 4 in Supplement 2). There was heterogeneity in the treatment effect of RT on survival (interaction *P* = .003) and FFS (interaction *P* = .002; supporting data reported in eTable 9 in Supplement 2) with a clearer effect for patients with low-burden than high-burden disease. Additionally, benefit for prostate RT on OS and FFS within patients with low-burden disease was consistent across age, pre-ADT PSA level, World Health Organization performance status, Gleason score, tumor stage, regional nodal stage, nominated RT schedule, or planned docetaxel use (all interaction *P* > .10; supporting data reported in eFigure 5 and eFigure 6 in Supplement 2). Extended results evaluating secondary outcome measures are presented in eResults in Supplement 2.

## Discussion

We have used a systematic approach herein to consolidate the notion that bone metastasis number based on conventional bone scan is predictive of survival benefit from adding prostate RT to SOC in newly diagnosed mPCa. This benefit is most pronounced up to 3 bone metastases; at or below this there is good evidence that the addition of prostate RT to SOC systemic therapy improves OS and FFS in these men. We also present evidence that men with M1a disease have improved FFS.

How the treatment effect of prostate RT changes with baseline bone metastasis count is of clinical relevance for patient selection and future trial designs. The first analysis of the STAMPEDE M1 RT comparison^1^ identified that prostate RT was more effective in the prespecified low-burden subgroup based on the CHAARTED definition.^10^ However, it was not clear how this treatment effect varied based on bone metastasis counts and whether a higher threshold could be selected. In this study, we explored this issue systematically, showing that survival benefit from prostate RT is most pronounced in patients with up to 3 bone metastases. Overall, OS and FFS benefits are supported by evidence for up to 3 bone metastases, but benefit is less certain between 4 and 7 bone metastases and is not clearly evident above 7. This association between bone metastatic number and benefit from local treatment also emphasizes the importance of accurate metastatic burden assessment to select patients for prostate RT. Another trial, HORRAD,^3^ in a subgroup of 160 patients with less than 5 bone metastases, showed some evidence of OS benefit for combining prostate RT with ADT compared with ADT alone (HR, 0.68; 95% CI, 0.42-1.10). However, as bone metastasis counts in HORRAD were categorized into 1 to 4, 5 to 15 and more than 15, a lower cutoff of 3 bone metastases was not considered,^3^ highlighting the importance of evaluating such effects on a continuous scale.^15^ Additionally, some studies published previously have suggested that patients with any number of bone metastases confined to the vertebral column are considered low burden.^10,12^ However, in our study of nearly 2000 patients, less than 2% of patients had 4 or more bone metastases solely within the vertebral column. We could not explore treatment effects in such patients given the small numbers.

Further exploratory analysis based on metastatic sites indicated survival benefit from prostate RT in patients with only NRLN metastasis (M1a) but not in those with visceral/other metastasis. There are currently no other randomized clinical trial data on the role of prostate RT in patients with M1a disease. We also showed a substantial prostate RT effect on FFS in M1a disease; the absolute improvement was 22% at 3 years. This is consistent with a previously reported nonrandomized analysis from STAMPEDE studying RT in N+M0 patients.^16^ Also, as NRLN metastatic burden has been shown to be prognostic,^17^ the role of metastatic NRLN metastasis counts as a predictive factor warrants additional investigation. Primary-site RT should therefore be considered as SOC in these men, who in the present study constituted 9% of the overall primary M1 caseload. By contrast, there was no evidence of benefit on FFS or OS in patients with visceral/other metastasis. Taken together, our study reinforces the predictive role of nonosseous metastatic sites within the metastatic burden criteria.

Currently, the definition of low metastatic burden is not agreed upon internationally; it includes a range of definitions based on metastasis number (<3 to <10), various sites (bone, lymph node, and/or visceral metastasis), and different imaging modalities.^2,7,9,10,12,18^ All such criteria are based on the prognostic relevance of metastatic sites and their extent. In our study, we built upon these prognostic criteria to evaluate systematically the predictive nature of metastatic burden based on conventional imaging using bone scan and computed tomography/magnetic resonance imaging. We show that metastatic burden criteria are not just prognostic; they are predictive of survival benefit when primary-site prostate RT is used. The subgroup of patients with only M1a or 3 or fewer bone metastases (±NRLN) and without any visceral/other metastasis had an 8% estimated absolute survival benefit at 3 years, whereas patients with bone metastasis counts greater than this or with visceral/other metastasis had no such benefit.

Various biologically plausible reasons exist whereby prostate RT could delay metastatic progression and improve survival in patients with low metastatic burden.^19^ Phylogenetic analysis of metastases in mPCa highlights complex metastatic cascades, wherein both primary-to-metastatic and metastasis-to-metastasis progression is identified.^20^ Based on this, we can hypothesize that treating the primary in low-burden disease may disrupt metastatic dissemination from the primary and delay metastatic progression. By contrast, with high burden, metastasis-to-metastasis progression could be the dominant mode of dissemination; treating the primary in this setting has minimal benefit. This hypothesis is supported by the observed heterogeneity in metastatic progression-free survival in the current study and the HORRAD trial.^2^ In our study, an absolute improvement of 7% in 3-year metastatic progression-free survival is observed with addition of prostate RT in patients with low-burden disease. Furthermore, a 2020 study^21^ using the same high-burden and low-burden criteria as devised herein demonstrated that patients with low-burden disease had a lower fraction of the genome altered, with lower genomic instability and fewer oncogenic alterations in the NOTCH, cell cycle, and epigenetic modifier pathways.

### Limitations

Several caveats to this exploratory analysis require mention, including its retrospective nature. Although the data on quantitative bone metastatic burden were available for most of the patients, some patients had to be excluded because their scans could not be centralized. There was also a lack of information on quantitative lymph node and visceral metastasis, which may also be predictive. However, a sensitivity analysis conducted in patients with only bone metastasis did not alter the predictive nature of the bone metastatic burden. We are also conscious that counting bone metastasis is not an accurate representation of bone metastatic volume. We explored this further in a separate study^22^ by evaluating bone scans using the automated bone scan index, which yielded similar results. Additionally, around 20% of patients in our study received docetaxel as their SOC. Currently, there is no evidence for combined use of RT and docetaxel nor of the effect of combining them with ADT in M1 disease. The value of prostate RT combined with abiraterone and docetaxel is currently being tested in the PEACE-1 trial (NCT01957436). Other ongoing trials are evaluating local treatment in combination with newer systemic therapies or metastasis-directed treatments. These trials can further validate metastatic burden as a predictor of survival benefit from local treatment. Finally, it is unclear how to translate these data to staging with newer imaging modalities using ^68^gallium-labeled ligands of the prostate-specific membrane antigen (known as ^68^Ga-PSMA) or whole-body magnetic resonance imaging, given that these emerging imaging modalities are more sensitive in detecting occult metastases. We emphasize that caution is required in extrapolating these data and cutoffs onto newer sensitive imaging modalities. These will need similar detailed studies to ascertain their true individual clinical relevance relative to their utility in predicting treatment outcome. This will be best evaluated prospectively within well-powered randomized clinical trials.

## Conclusions

Bone metastatic burden based on conventional imaging is predictive of OS and FFS benefit when prostate RT is added to SOC in newly diagnosed mPCa. This beneficial effect is most pronounced in patients with up to 3 bone metastases, below which addition of prostate RT to SOC improves survival in patients without visceral or other metastasis. The criteria for low metastatic burden based on conventional imaging, predictive of survival benefit from prostate RT in men with newly diagnosed mPCa, should now also include men with M1a disease.

## Notes

Supplement 1. Trial Protocol

Supplement 2. **eMethods.** **eResults.** **eTable 1.** Baseline characteristics of 1939 patients by treatment included in this study. **eTable 2.** Baseline characteristics of patients randomized in the STAMPEDE M1|RT comparison and the patients included in this study, by treatment. **eTable 3.** Bayesian Information Criterion (BIC) and Akaike Information Criterion (AIC) for models evaluating interaction of treatment with linear, FP1 and FP2 functions of bone metastasis count. **eTable 4.** Summary of estimated treatment effects for overall and failure-free survival, for all 1939 patients in subgroups based on ≤3, 4 to 7 and >7 bone metastases. **eTable 5.** Baseline characteristics of 1587 patients with bone metastases (±NRLN) and without visceral metastasis stratified by ≤3 and ≥4 bone metastasis, by treatment. **eTable 6.** Summary of estimated treatment effects for each outcome measure, for all 1272 patients with only bone metastasis and in subgroups based on ≤3 and ≥4 bone metastases. **eTable 7.** Baseline characteristics of 181 patients with only non-regional lymph node metastasis (M1a), by treatment. **eTable 8.** Baseline characteristics of 171 patients with any visceral or other metastasis, by treatment arms. **eTable 9.** Summary of treatment effects for each outcome measure by the newly devised metastatic burden criteria. **eFigure 1.** Hazard ratios and 95% confidence intervals for overall and failure-free survival in non-overlapping sub-populations based on bone metastases count for 1939 patients. **eFigure 2.** Kaplan-Meier estimated 3-year (A) overall and (B) failure free survival in non-overlapping sub-populations based on bone metastases count for 1939 patients. **eFigure 3.** Kaplan-Meier curves for overall and failure-free survival by treatment in 1272 patients with only bone metastasis and no non-regional lymph node/visceral/other metastasis stratified by (A,B) ≤3 and (C,D) ≥4 bone metastases. **eFigure 4.** Kaplan-Meier curves for overall and failure-free survival in the newly devised (A,B) low and (C,D) high metastatic burden subgroups. **eFigure 5.** Effect of prostate radiotherapy on overall survival across baseline factors in low and high metastatic burden subgroups. **eFigure 6.** Effect of prostate radiotherapy on failure-free survival across baseline factors in low and high metastatic burden subgroups. **eReferences.**

Supplement 3. Data Sharing Statement
