# Supplementary material for: Association of Bone Metastatic Burden With Survival Benefit From Prostate Radiotherapy in Patients With Newly Diagnosed Metastatic Prostate Cancer: A Secondary Analysis of a Randomized Clinical Trial
Source: JAMA Oncol. 2021 Feb 18;7(4):555–63. doi: 10.1001/jamaoncol.2020.7857 (PMC7893550; doi:10.1001/jamaoncol.2020.7857)
Supplement: Supplement 2. — eMethods. eResults. eTable 1. Baseline characteristics of 1939 patients by treatment included in this study. eTable 2. Baseline characteristics of patients randomized in the STAMPEDE M1|RT comparison and the patients included in this study, by treatment. eTable 3. Bayesian Information Criterion (BIC) and Akaike Information Criterion (AIC) for models evaluating interaction of treatment with linear, FP1 and FP2 functions of bone metastasis count. eTable 4. Summary of estimated treatment effects for overall and failure-free survival, for all 1939 patients in subgroups based on ≤3, 4 to 7 and >7 bone metastases. eTable 5. Baseline characteristics of 1587 patients with bone metastases (±NRLN) and without visceral metastasis stratified by ≤3 and ≥4 bone metastasis, by treatment. eTable 6. Summary of estimated treatment effects for each outcome measure, for all 1272 patients with only bone metastasis and in subgroups based on ≤3 and ≥4 bone metastases. eTable 7. Baseline characteristics of 181 patients with only non-regional lymph node metastasis (M1a), by treatment. eTable 8. Baseline characteristics of 171 patients with any visceral or other metastasis, by treatment arms. eTable 9. Summary of treatment effects for each outcome measure by the newly devised metastatic burden criteria. eFigure 1. Hazard ratios and 95% confidence intervals for overall and failure-free survival in non-overlapping sub-populations based on bone metastases count for 1939 patients. eFigure 2. Kaplan-Meier estimated 3-year (A) overall and (B) failure free survival in non-overlapping sub-populations based on bone metastases count for 1939 patients. eFigure 3. Kaplan-Meier curves for overall and failure-free survival by treatment in 1272 patients with only bone metastasis and no non-regional lymph node/visceral/other metastasis stratified by (A,B) ≤3 and (C,D) ≥4 bone metastases. eFigure 4. Kaplan-Meier curves for overall and failure-free survival in the newly devised (A,B) low and (C,D) high metast [file jamaoncol-e207857-s002.pdf]

Supplemental Online Content

Ali SA, Hoyle A, Haran AM, et al. Association of bone metastatic burden with survival benefit from prostate radiotherapy in patients with newly diagnosed metastatic prostate cancer: a secondary analysis of a randomized clinical trial. *JAMA Oncol*. Published online February 18, 2021. doi:10.1001/jamaoncol.2020.7857

eMethods.

eResults.

eTable 1. Baseline characteristics of 1939 patients by treatment included in this study.

eTable 2. Baseline characteristics of patients randomized in the STAMPEDE M1|RT comparison and the patients included in this study, by treatment.

eTable 3. Bayesian Information Criterion (BIC) and Akaike Information Criterion (AIC) for models evaluating interaction of treatment with linear, FP1 and FP2 functions of bone metastasis count.

eTable 4. Summary of estimated treatment effects for overall and failure-free survival, for all 1939 patients in subgroups based on ≤3, 4 to 7 and >7 bone metastases.

eTable 5. Baseline characteristics of 1587 patients with bone metastases (±NRLN) and without visceral metastasis stratified by ≤3 and ≥4 bone metastasis, by treatment.

eTable 6. Summary of estimated treatment effects for each outcome measure, for all 1272 patients with only bone metastasis and in subgroups based on ≤3 and ≥4 bone metastases.

eTable 7. Baseline characteristics of 181 patients with only non-regional lymph node metastasis (M1a), by treatment.

eTable 8. Baseline characteristics of 171 patients with any visceral or other metastasis, by treatment arms.

eTable 9. Summary of treatment effects for each outcome measure by the newly devised metastatic burden criteria.

eFigure 1. Hazard ratios and 95% confidence intervals for overall and failure-free survival in non-overlapping sub-populations based on bone metastases count for 1939 patients.

eFigure 2. Kaplan-Meier estimated 3-year (A) overall and (B) failure free survival in non-overlapping sub-populations based on bone metastases count for 1939 patients.

eFigure 3. Kaplan-Meier curves for overall and failure-free survival by treatment in 1272 patients with only bone metastasis and no non-regional lymph node/visceral/other metastasis stratified by (A,B) ≤3 and (C,D) ≥4 bone metastases.

eFigure 4. Kaplan-Meier curves for overall and failure-free survival in the newly devised (A,B) low and (C,D) high metastatic burden subgroups.

eFigure 5. Effect of prostate radiotherapy on overall survival across baseline factors in low and high metastatic burden subgroups.

eFigure 6. Effect of prostate radiotherapy on failure-free survival across baseline factors in low and high metastatic burden subgroups.

eReferences.

This supplemental material has been provided by the authors to give readers additional information about their work.

eMethods.

Secondary outcome measures

Secondary outcome measures evaluated included progression-free survival (PFS) defined as FFS without biochemical events, metastatic progression-free survival (mPFS) defined as time from randomisation to new metastases or progression of existing metastases or death and prostate cancer-specific survival (PCSS). Patients without the event of interest were censored at the time last known to be event-free. The outcomes dataset frozen for the published STAMPEDE "M1|RT comparison" was used for survival analyses.[1]

Statistical analyses

To evaluate treatment and bone metastasis interaction on a continuous scale, the multivariable fractional polynomial interaction (MFPI) approach was utilized. The MFPI algorithm is an extension of the MFP algorithm based on fractional polynomial analysis of continuous predictors.[2, 3] This approach for detecting interactions between treatment and a continuous variable avoids the assumption of linearity and arbitrary categorization.[4, 5] It aims to use all information from a continuous variable while allowing for possible non-linearity using first (FP1) and second degree (FP2) fractional polynomial transformations of the continuous variable. Interaction of treatment with linear, FP1 and FP2 functions of bone metastasis counts were evaluated using nested Cox models adjusted for minimisation factors used at randomisation: age (<70 or ≥70), N stage (N0, N+ or NX), WHO PS (0 or 1-2), NSAID or aspirin use (uses either or no) and planned docetaxel use (yes or no) along with metastatic site (only NRLN metastasis, bone±NRLN metastasis or any visceral/other metastasis). Despite the benefits of the fractional polynomial approach, overfitting of interaction terms with FP2 transformations is a real concern.[6] Such overfitting can be avoided by selecting a simplified model based on Bayesian Information Criteria (BIC) and Akaike Information Criteria (AIC).[6-8] To guard against such overfitting, model with the lowest BIC was preferred. Model comparison was performed using the BIC adapted for censored data which corrects for number of events rather than sample size.[9] Differences between BIC (ΔBIC) values for two models were interpreted as per the BIC evidence grades presented by Raftery.[10] Selection based on BIC was verified using the Akaike information criteria (AIC).[8] A p-value from a likelihood ratio test of the interaction between treatment group and bone metastasis count is presented. The MFPI model-estimated treatment effect as a function of bone metastasis count was plotted graphically on the HR scale with 95%CI. Further details regarding the MFPI have been published previously.[2, 6]

In the newly devised low and high metastatic burden subgroups, we evaluated treatment effects for primary and secondary outcome measures. Adjusted Cox proportional hazards regression models were used to estimate relative treatment effects. Flexible parametric models fitted using (5,5) degrees of freedom with adjustment variables as specified above were used to generate 3-year survival estimates. Restricted mean survival time (RMST) were evaluated using a t-star of 59 months as determined by the Royston and Parmar method.[11] Fine and Gray regression models were used for competing risk analysis of prostate cancer-specific survival.[12] Consistency of treatment effect within the low and high burden subgroups was explored across selected baseline characteristics of clinical relevance: patient age (<70 or ≥70), pre-ADT PSA (quartiles), WHO performance status (0 or 1–2), Gleason sum score (≤7, 8–10 or unknown), tumour stage (≤T2, T3 or T4), regional nodal status (N0, N1 or NX), nominated RT schedule (36 Gy/6f/6 weeks or 55 Gy/20f/4 weeks) and planned docetaxel use (No or Yes). A HR below 1 favoured the prostate radiotherapy group. Statistical analyses were performed using Stata v15.1 (StataCorp, College Station, TX, USA).

© 2021 Ali SA et al. *JAMA Oncology.*

eResults.

For models evaluating the interaction between treatment and bone metastasis counts, **eTable 3** shows the BIC and AIC statistics for linear, FP1 and FP2 models. For both OS and FFS, the linear model had the lowest BIC. Also, the BIC criteria suggest that both FP1 and FP2 models were overfit (ΔBIC >6) compared to the linear model. Since both the AIC and the BIC are smallest for the linear model, each criterion would select this model for OS and FFS.

The effect of treatment was heterogeneous across the newly devised metastatic burden subgroups for secondary outcome measures (interaction p-values: PFS=0.004; mPFS=0.009; PCSS=0.005) with good evidence of benefit noted for all outcome measures in patients with low metastatic burden **(eTable 9 in supplement)**. In the low metastatic burden subgroup, there was good evidence that prostate RT improved PFS (HR=0.72, 95%CI 0.57 – 0.92), mPFS (HR=0.74, 95%CI 0.58 – 0.94) and PCSS (sub-HR=0.60, 95%CI 0.43 – 0.86). The absolute improvement in 3-year PFS, mPFS and PCSS was 9%, 7% and 9% respectively in patients with low metastatic burden.

© 2021 Ali SA et al. *JAMA Oncology.*

eTable 1. Baseline characteristics of 1939 patients by treatment included in this study.

	SOC (n=976)		SOC+RT (n=963)	
	n	%	n	%
**Age at randomisation**				
**Median**	68		68	
**IQR**	63-73		63-73	
**PSA (ng/mL) before ADT**				
**Median**	98		98	
**IQR**	31-315		33-312	
**WHO performance status**				
**0**	695	71	689	72
**1 to 2**	281	29	274	29
**Primary tumour stage**				
**≤T2**	89	9	94	10
**T3**	555	57	563	59
**T4**	246	25	232	24
**TX**	86	9	74	8
**Gleason score**				
**≤7**	161	17	165	17
**8 to 10**	781	80	757	79
**Unknown**	34	4	41	4
**Regional node status**				
**N0**	332	34	329	34
**N1**	582	60	569	59
**NX**	62	6	65	7
**Nominated RT schedule**				
**36Gy in 6f over 6 weeks**	447	46	459	48
**55Gy in 20f over 4 weeks**	529	54	504	52
**Planned Docetaxel use**				
**No**	804	82	792	82
**Yes**	172	18	171	18
**Sites of metastases**				
**Bone**	872	89	860	89
**NRLN**	276	28	277	29
**Lung**	36	4	41	4
**Liver**	22	2	18	2
**Other**	34	3	32	3
**Number of bone metastases**				
**≤3**	417	43	409	42
**4 to 9**	204	21	203	21
**≥10**	355	36	351	36
**Abbreviations: SOC – standard-of-care, RT – radiotherapy, IQR- inter-quartile range, PSA – prostate specific antigen, ADT – androgen deprivation therapy, NRLN- Non-regional lymph nodes.**				

© 2021 Ali SA et al. *JAMA Oncology.*

eTable 2. Baseline characteristics of patients randomized in the STAMPEDE M1|RT comparison and the patients included in this study, by treatment.

	M1|RT comparison (n=2061)				Included (n=1939)			
	SOC (n=1029)		SOC+RT (n=1032)		SOC (n=976)		SOC+RT (n=963)	
	n	%	n	%	n	%	n	%
**Age at randomisation**								
**Median**	68		68		68		68	
**IQR**	63 - 73		63-73		63-73		63-73	
**PSA (ng/ml) before ADT**								
**Median**	98		97		98		98	
**IQR**	30 - 316		33 - 313		31-315		33-312	
**WHO performance status**								
**0**	732	71	734	71	695	71	689	72
**1 to 2**	297	29	298	29	281	29	274	29
**Primary tumour stage**								
**≤T2**	96	9	103	10	89	9	94	10
**T3**	585	57	603	58	555	57	563	59
**T4**	260	25	246	24	246	25	232	24
**TX**	88	9	80	8	86	9	74	8
**Gleason score**								
**≤7**	173	17	172	17	161	17	165	17
**8 to 10**	820	80	810	78	781	80	757	79
**Unknown**	36	3	50	5	34	4	41	4
**Regional node status**								
**N0**	345	34	344	33	332	34	329	34
**N1**	620	60	620	60	582	60	569	59
**NX**	64	6	68	7	62	6	65	7
**Nominated RT schedule**								
**36Gy in 6f over 6 weeks**	482	47	497	48	447	46	459	48
**55Gy in 20f over 4 weeks**	547	53	535	52	529	54	504	52
**Planned Docetaxel use**								
**No**	845	82	849	82	804	82	792	82
**Yes**	184	18	183	18	172	18	171	18
**Sites of metastases**								
**Bone**	919	89	917	89	872	89	860	89
**NRLN**	294	29	304	29	276	28	277	29
**Lung**	42	4	48	5	36	4	41	4
**Liver**	23	2	19	2	22	2	18	2
**Other**	35	3	33	3	34	3	32	3
**Abbreviations: SOC – standard of care, RT – radiotherapy, IQR- inter-quartile range, PSA – prostate specific antigen, ADT – androgen deprivation therapy, NRLN- Non-regional lymph nodes.**								

© 2021 Ali SA et al. *JAMA Oncology.*

eTable 3. Bayesian Information Criterion (BIC) and Akaike Information Criterion (AIC) for models evaluating interaction of treatment with linear, FP1 and FP2 functions of bone metastasis count.

Outcome evaluated	Model class	BIC	AIC
**Overall survival**			
	**Linear**	9782.17	9768.46
	**FP1**	9788.88	9770.59
	**FP2**	9801.80	9769.79
**Failure-free survival**			
	**Linear**	17810.99	17795.45
	**FP1**	17818.17	17797.45
	**FP2**	17838.25	17801.99

© 2021 Ali SA et al. *JAMA Oncology.*

eTable 4. Summary of estimated treatment effects for overall and failure-free survival, for all 1939 patients in subgroups based on ≤3, 4 to 7 and >7 bone metastases.

	Events/patients		HR (95%CI) [a]	3-year KM survival %	
	SOC	SOC+RT		SOC	SOC+RT
**Overall survival**					
≤3 bone metastases	123/417	89/409	0.65 (0.49-0.85)	73%	83%
≥4 and ≤7 bone metastases	53/180	63/168	1.39 (0.96-2.00)	69%	62%
>7 bone metastases	192/379	195/386	1.03 (0.84-1.25)	46%	47%
**Failure-free survival**					
≤3 bone metastases	266/417	199/409	0.60 (0.50-0.72)	31%	51%
≥4 and ≤7 bone metastases	121/180	121/168	0.89 (0.68-1.15)	25%	23%
>7 bone metastases	333/379	322/386	0.85 (0.73-1.00)	11%	13%

[a] Hazard ratios and 95%CI are from Cox proportional hazards models adjusted for age (<70 or ≥70), N stage (N0, N+ or NX), WHO performance status (0 or 1-2), NSAID or aspirin use (uses either or no), docetaxel use (yes or no) and metastatic site (only NRLN, bone±NRLN or any visceral/other).

Abbreviations: SOC – standard of care, RT – radiotherapy, HR – hazard ratio, CI – confidence interval, KM – Kaplan-Meier.

© 2021 Ali SA et al. *JAMA Oncology.*

eTable 5. Baseline characteristics of 1587 patients with bone metastases (±NRLN) and without visceral metastasis stratified by ≤3 and ≥4 bone metastases, by treatment.

	≤3 Bone metastases (± NRLN) (n=577)				≥ 4 Bone metastases (± NRLN) (n=1010)			
	SOC (n=290)		SOC+RT (n=287)		SOC (n=512)		SOC+RT (n=498)	
	n	%	n	%	n	%	n	%
**Age at randomisation**								
**Median**	68		69		68		68	
**IQR**	64-73		63-73		63-73		63-73	
**PSA (ng/ml) before ADT**								
**Median**	44		46		183		182	
**IQR**	15-100		20-98		58-581		53-638	
**WHO performance status**								
**0**	212	73	223	78	348	68	341	69
**1 to 2**	78	27	64	22	164	32	157	32
**Primary tumour stage**								
**≤T2**	39	13	26	9	40	8	50	10
**T3**	182	63	177	62	273	53	277	56
**T4**	55	19	71	25	135	26	120	24
**TX**	14	5	13	5	64	13	51	10
**Gleason score**								
**≤7**	65	22	63	22	78	15	74	15
**8 to 10**	218	75	216	75	418	82	405	81
**Unknown**	7	2	8	3	16	3	19	4
**Regional node status**								
**N0**	136	47	127	44	175	34	177	36
**N1**	141	49	151	53	293	57	271	54
**NX**	13	5	9	3	44	9	50	10
**Nominated RT schedule**								
**36Gy in 6f over 6 weeks**	126	43	119	42	235	46	270	54
**55Gy in 20f over 4 weeks**	164	57	168	59	277	54	228	46
**Planned Docetaxel use**								
**No**	246	85	243	85	417	81	407	82
**Yes**	44	15	44	15	95	19	91	18
**Sites of metastases**								
**Bone**	290	100	287	100	512	100	498	100
**NRLN**	44	15	46	16	120	53	105	47
**Lung**	0	0	0	0	0	0	0	0
**Liver**	0	0	0	0	0	0	0	0
**Other**	0	0	0	0	0	0	0	0
**Number of bone metastases**								
**≤3**	290	100	287	100	0	0	0	0
**4 to 9**	0	0	0	0	185	36	183	37
**≥10**	0	0	0	0	327	64	315	63
**Abbreviations: NRLN- Non-regional lymph nodes., SOC – standard of care, RT – radiotherapy, IQR- inter-quartile range, PSA – prostate specific antigen, ADT – androgen deprivation therapy.**								

© 2021 Ali SA et al. *JAMA Oncology.*

eTable 6. Summary of estimated treatment effects for each outcome measure, for all 1272 patients with only bone metastasis and in subgroups based on ≤3 and ≥4 bone metastases.

	Events/patients		HR (95% CI)[a]	3 year KM survival %		Interaction p value
	SOC	SOC+ RT		SOC	SOC+ RT	
**Overall survival**						
Only bone metastasis	230/638	229/634	0.94 (0.79 - 1.13)	63%	67%	0.044
≤3 bone metastases	63/246	48/241	0.67 (0.46 - 0.97)	77%	86%	
≥4 bone metastases	167/392	181/393	1.07 (0.87 - 1.32)	54%	56%	
**Failure-free survival**						
Only bone metastasis	463/638	420/634	0.75 (0.65 - 0.85)	24%	33%	0.013
≤3 bone metastases	150/246	108/241	0.56 (0.44 -0.72)	36%	56%	
≥4 bone metastases	313/392	312/393	0.85 (0.72 - 0.99)	16%	17%	

[a] Hazard ratios and 95%CI are from Cox proportional hazards models adjusted for minimisation factors used at randomisation: age (<70 or ≥70), WHO performance status (0 or 1-2), N stage (N0, N+ or NX), NSAID or aspirin use (uses either or no) and docetaxel use (yes or no).

Abbreviations: SOC – standard of care, RT – radiotherapy, HR – hazard ratio, CI – confidence interval, KM – Kaplan-Meier, NRLN – non-regional lymph node.

© 2021 Ali SA et al. *JAMA Oncology.*

eTable 7. Baseline characteristics of 181 patients with only non-regional lymph node metastasis (M1a), by treatment.

	Only NRLN (n=181)			
	SOC (n=89)		SOC+RT (n=92)	
	n	%	n	%
**Age at randomisation**				
**Median**	66		68	
**IQR**	63-72		63-72	
**PSA (ng/ml) before ADT**				
**Median**	64		92	
**IQR**	27-151		33-197	
**WHO performance status**				
**0**	73	82	68	74
**1 to 2**	16	18	24	26
**Primary tumour stage**				
**≤T2**	6	7	9	10
**T3**	56	63	65	71
**T4**	26	29	15	16
**TX**	1	1	3	3
**Gleason score**				
**≤7**	6	7	16	17
**8 to 10**	80	90	69	75
**Unknown**	3	3	7	8
**Regional node status**				
**N0**	4	4	5	5
**N1**	83	93	87	95
**NX**	2	2	0	0
**Nominated RT schedule**				
**36Gy in 6f over 6 weeks**	45	51	34	37
**55Gy in 20f over 4 weeks**	44	49	58	63
**Planned Docetaxel use**				
**No**	72	81	75	82
**Yes**	17	19	17	18
**Sites of metastases**				
**NRLN**	89	100	92	100
**Bone**	0	0	0	0
**Lung**	0	0	0	0
**Liver**	0	0	0	0
**Other**	0	0	0	0

Abbreviations: NRLN- Non-regional lymph node, SOC – Standard of care, RT – radiotherapy, IQR- inter-quartile range, PSA – prostate specific antigen, ADT – androgen deprivation therapy.

© 2021 Ali SA et al. *JAMA Oncology.*

eTable 8. Baseline characteristics of 171 patients with any visceral or other metastasis, by treatment arms.

	Any visceral/other metastasis (n=171)			
	SOC (n=85)		SOC+RT (n=86)	
	n	%	n	%
**Age at randomisation**				
**Median**	68		69	
**IQR**	63-72		62-74	
**PSA (ng/ml) before ADT**				
**Median**	131		124	
**IQR**	48-421		36-373	
**WHO performance status**				
**0**	62	73	57	66
**1 to 2**	23	27	29	34
**Primary tumour stage**				
**≤T2**	4	5	9	10
**T3**	44	52	44	51
**T4**	30	35	26	30
**TX**	7	8	7	8
**Gleason score**				
**≤7**	12	14	12	14
**8 to 10**	65	76	67	78
**Unknown**	8	9	7	8
**Regional node status**				
**N0**	17	20	20	23
**N1**	65	76	60	70
**NX**	3	4	6	7
**Nominated RT schedule**				
**36Gy in 6f over 6 weeks**	41	48	36	42
**55Gy in 20f over 4 weeks**	44	52	50	58
**Planned Docetaxel use**				
**No**	69	81	67	78
**Yes**	16	19	19	22
**Sites of metastases**				
**Bone**	70	82	75	87
**NRLN**	23	27	34	40
**Lung**	36	42	41	48
**Liver**	22	26	18	20
**Other**	34	40	32	37
**Number of bone metastases**				
**≤3**	38	45	30	35
**4 to 9**	19	22	20	23
**≥10**	28	33	36	42
Abbreviations: SOC – Standard of care, RT – radiotherapy, IQR- inter-quartile range, PSA – prostate specific antigen, ADT – androgen deprivation therapy, NRLN- Non-regional lymph nodes.				

© 2021 Ali SA et al. *JAMA Oncology.*

eTable 9. Summary of treatment effects for each outcome measure by the newly devised metastatic burden criteria. Low metastatic burden is defined as patients with only NRLN metastasis or ≤3 bone metastases (±NRLN) and without any visceral or other metastasis.

	Events/Patients		HR (95%CI) [a]	Interaction by metastatic burden p-value	Restricted mean survival time (months)[b]			3-year survival[b]	
	SOC	SOC+RT			SOC	SOC+RT	Difference (95% CI)	SOC	SOC+RT
**Overall survival**									
Low burden	109/379	79/379	0.62 (0.46 - 0.83)	0.003	45.1	49.6	4.5 (1.9 - 7.0)	74%	82%
High burden	259/597	268/584	1.08 (0.91 - 1.28)		39.2	37.9	-1.3 (-3.8 - 1.2)	55%	54%
**Failure-free survival**									
Low burden	238/379	181/379	0.57 (0.47 - 0.70)	0.002	27.9	37.1	9.2 (5.9 - 12.4)	34%	52%
High burden	482/597	461/584	0.87 (0.76 - 0.99)		17.4	19.1	1.7 (-0.3 - 3.6)	17%	19%
**Progression-free survival**									
Low burden	157/379	129/379	0.72 (0.57 - 0.92)	0.004	39.3	43.7	4.4 (1.3 - 7.5)	57%	66%
High burden	366/597	385/584	1.10 (0.95 - 1.27)		28.6	26.6	-2.0 (-4.6 - 0.6)	36%	31%
**Metastatic progression-free survival**									
Low burden	144/379	120/379	0.74 (0.58 - 0.94)	0.009	40.9	44.8	3.9 (1.1 - 6.7)	61%	68%
High burden	351/597	370/584	1.11 (0.96 - 1.28)		29.9	27.8	-2.1 (-4.7 - 0.5)	38%	33%
**Prostate cancer-specific survival[b]**									
Low burden	81/379	56/379	0.60 (0.43 - 0.86)	0.005	48.5	52.3	3.8 (1.4 - 6.2)	79%	88%
High burden	229/597	240/584	1.10 (0.92 - 1.32)		40.9	39.4	-1.6 (-3.9 - 0.7)	58%	57%

Hazard ratios and restricted means survival time differences are for prostate radiotherapy relative to control.

Low burden is defined as patients with only NRLN metastasis or ≤3 bone metastases (+/-NRLN) and no visceral or other metastasis.
[a] Hazard ratios and 95%CI are from Cox proportional hazards models adjusted for age (<70 or ≥70), N stage (N0, N+ or NX), WHO performance status (0 or 1-2), NSAID or aspirin use (uses either or no), docetaxel use (yes or no) and stratified by time period.
[b] Survival probabilities and restricted mean survival time estimates are taken from flexible parametric models (t-star, 59 months).
[c] Sub-distribution hazard ratios and 95%CI are from Fine and Grey Competing risk regression adjusted for variables as stated above.
Abbreviations: SOC – standard of care, RT – radiotherapy, HR – hazard ratio, CI – confidence interval, NRLN-non-regional lymph node metastasis.

© 2021 Ali SA et al. *JAMA Oncology.*

eFigure 1. Hazard ratios and 95% confidence intervals for overall and failure-free survival in non-overlapping sub-populations based on bone metastases counts for 1939 patients.

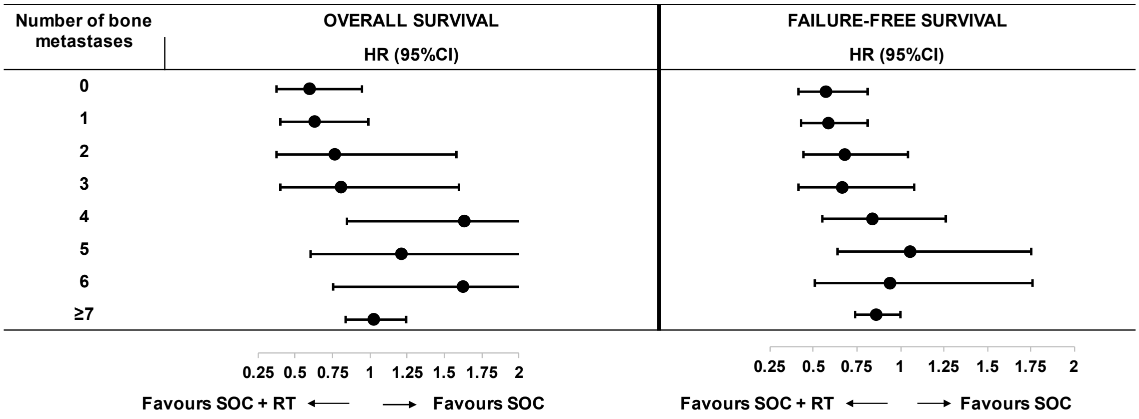

© 2021 Ali SA et al. *JAMA Oncology.*

eFigure 2. Kaplan-Meier estimated 3-year (A) overall and (B) failure free survival in non-overlapping sub-populations based on bone metastasis counts for 1939 patients.

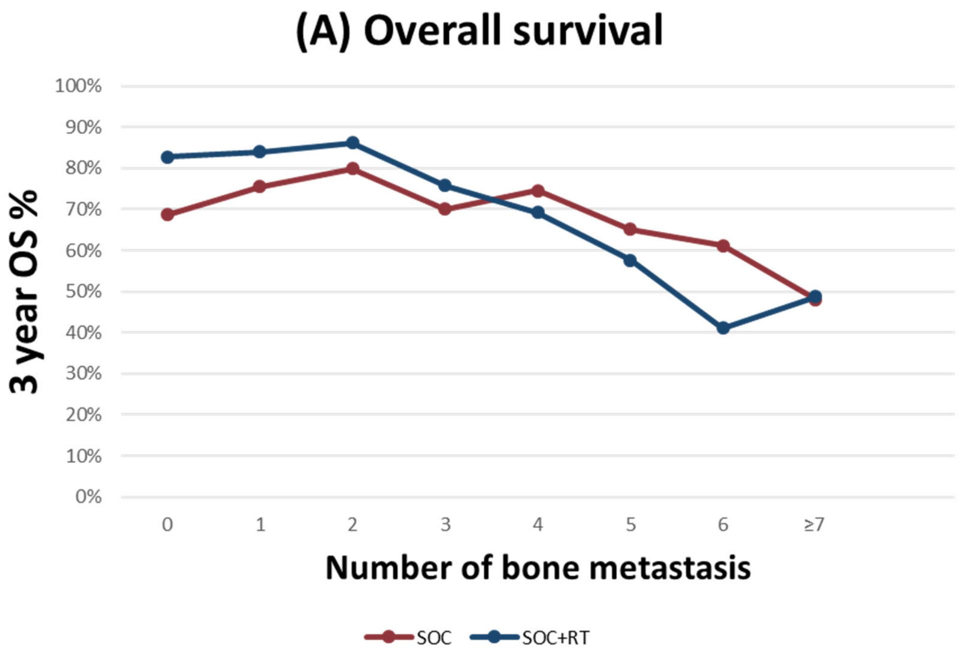

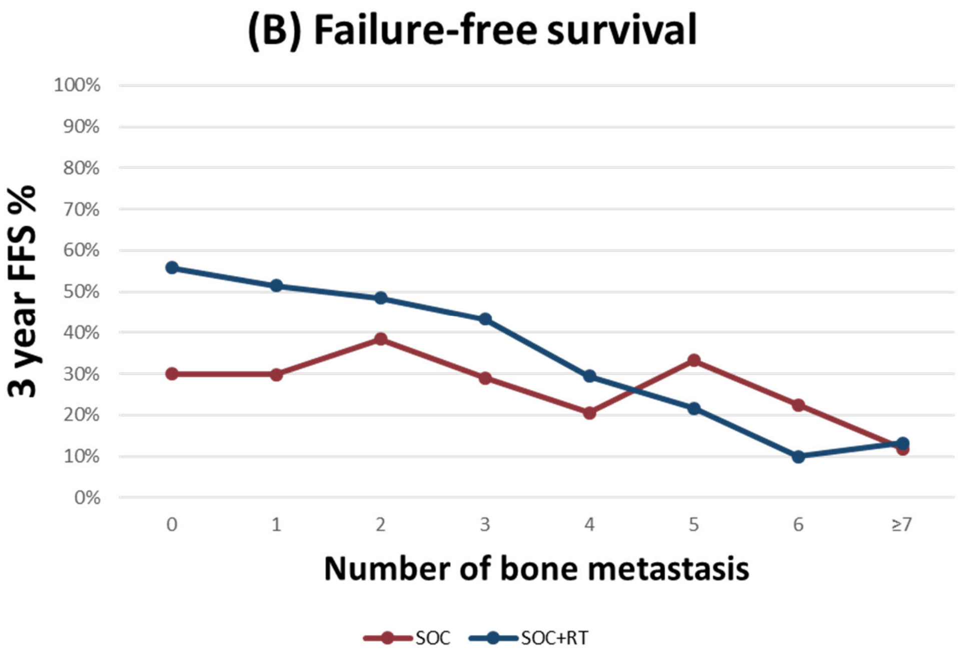

© 2021 Ali SA et al. *JAMA Oncology*.

eFigure 3. Kaplan-Meier curves for overall and failure-free survival by treatment in 1272 patients with only bone metastasis and no non-regional lymph node/visceral/other metastasis stratified by (A,B) ≤3 and (C,D) ≥4 bone metastases. SOC- standard of care, RT-radiotherapy.

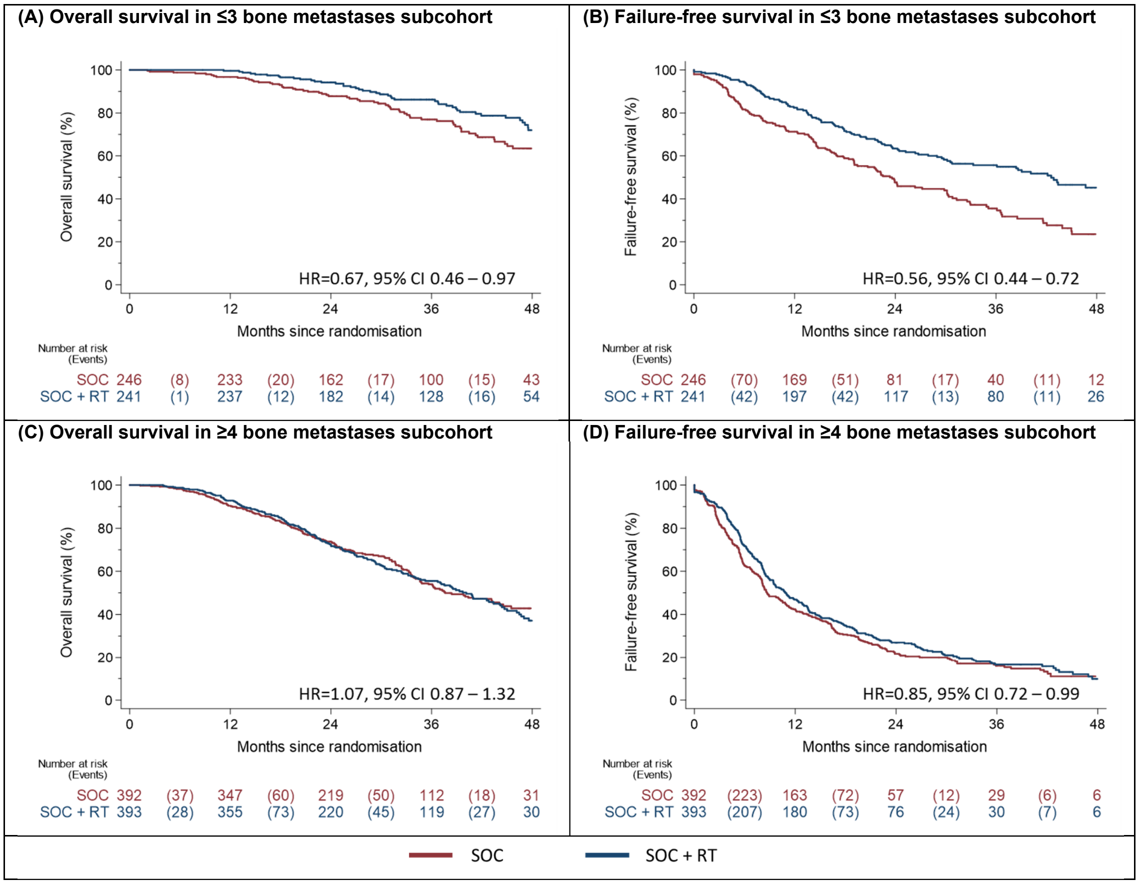

© 2021 Ali SA et al. *JAMA Oncology*.

eFigure 4. Kaplan-Meier curves for overall and failure-free survival in the newly devised (A,B) low and (C,D) high metastatic burden subgroups. Low metastatic burden is defined as patients with only NRLN metastasis or ≤3 bone metastases (±NRLN) and without any visceral or other metastasis. SOC- standard of care, RT-radiotherapy.

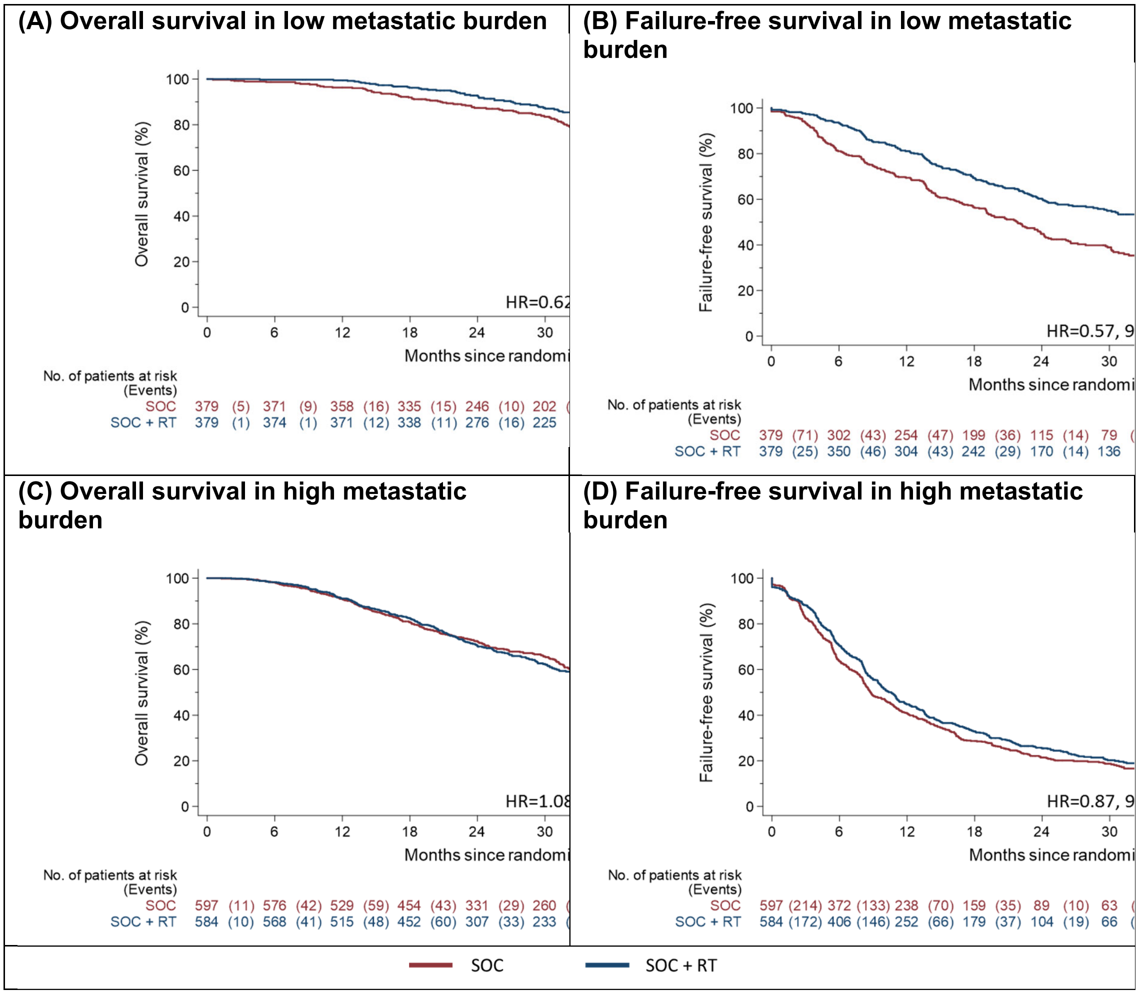

© 2021 Ali SA et al. *JAMA Oncology.*

eFigure 5. Effect of prostate radiotherapy on overall survival across baseline factors in low and high metastatic burden subgroups. Low burden is defined as patients with only NRLN metastasis or ≤3 bone metastases (±NRLN) and without any visceral or other metastasis. Solid vertical line indicates a hazard ratio of 1, dotted line indicates the hazard ratios for overall survival within low and high metastatic burden subgroups.

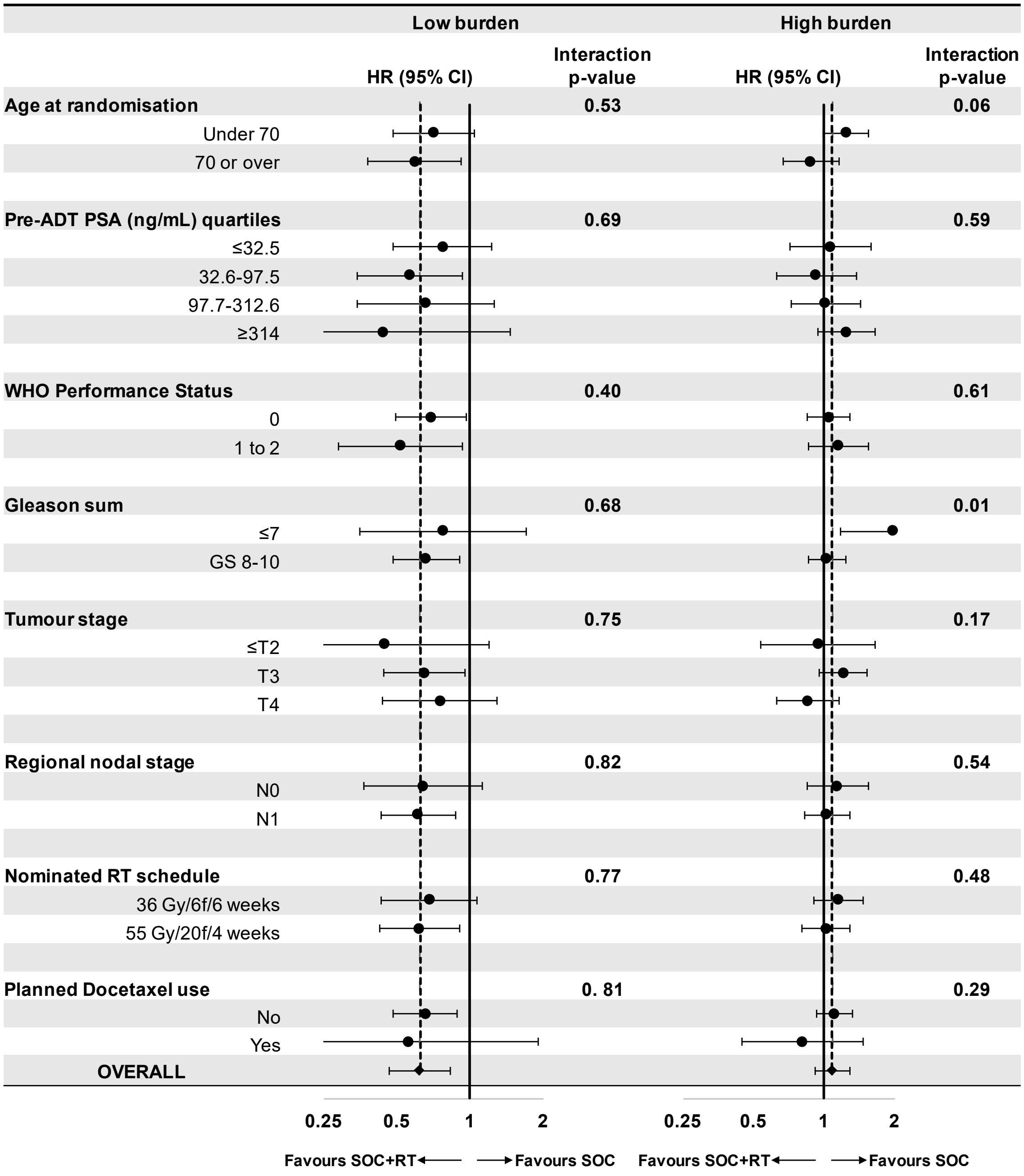

© 2021 Ali SA et al. *JAMA Oncology.*

eFigure 6. Effect of prostate radiotherapy on failure-free survival across baseline factors in low and high metastatic burden subgroups. Low burden is defined as patients with only NRLN metastasis or ≤3 bone metastases (±NRLN) and no visceral or other metastasis. Solid vertical line indicates a hazard ratio of 1, dotted line indicates the hazard ratios for failure-free survival within low and high metastatic burden subgroups.

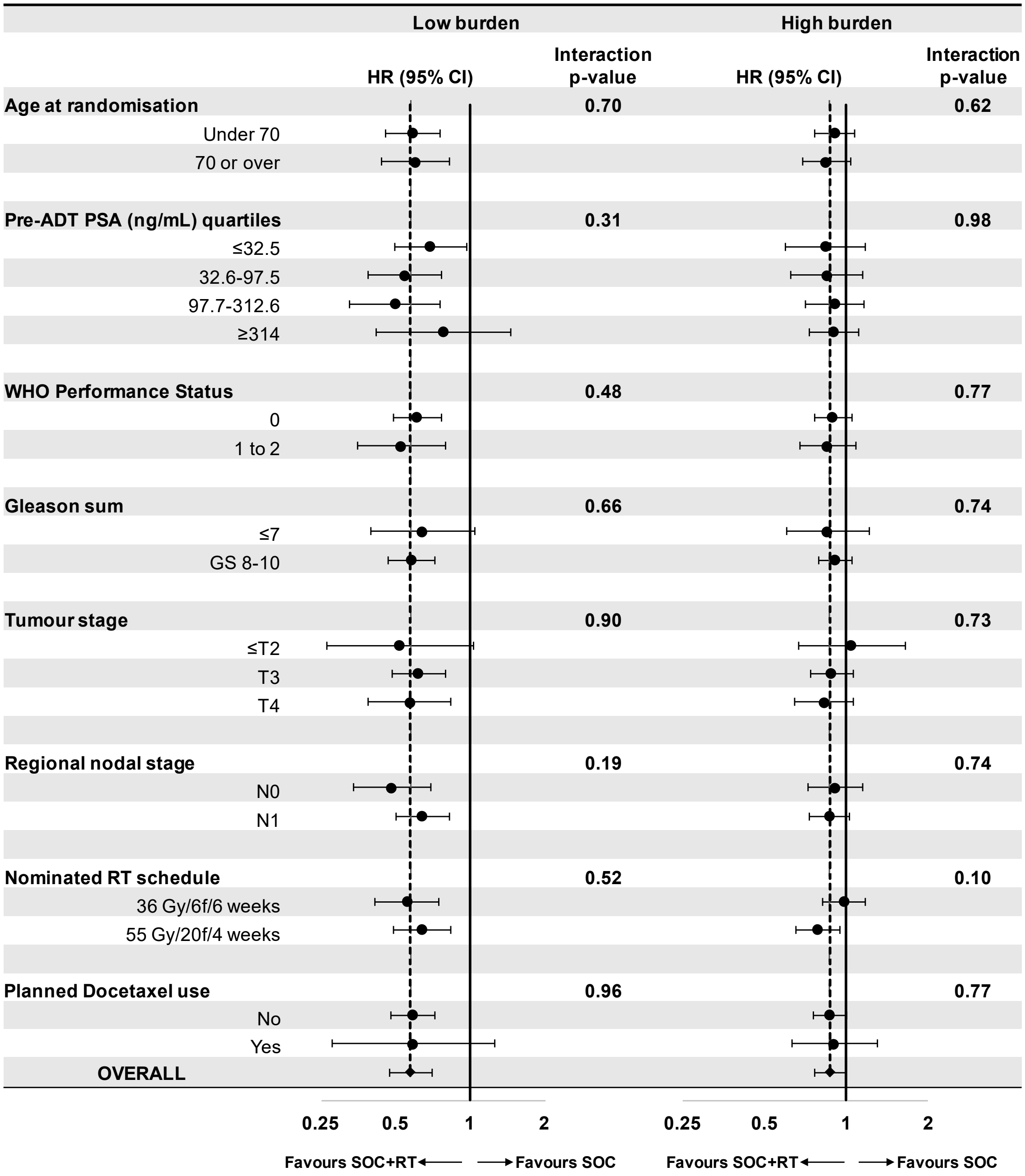

© 2021 Ali SA et al. *JAMA Oncology.*

eReferences.

1. Parker CC, James ND, Brawley CD, et al. Radiotherapy to the primary tumour for newly diagnosed, metastatic prostate cancer (STAMPEDE): a randomised controlled phase 3 trial. *Lancet*. 2018;392(10162):2353-2366.

2. Royston P, Sauerbrei W. A new approach to modelling interactions between treatment and continuous covariates in clinical trials by using fractional polynomials. *Stat Med*. 2004;23(16):2509-25.

3. Royston P, Altman DG. Regression Using Fractional Polynomials of Continuous Covariates: Parsimonious Parametric Modelling. *Journal of the Royal Statistical Society Series C (Applied Statistics)*. 1994;43(3):429-467.

4. Farewell VT, Tom BDM, Royston P. The Impact of Dichotomization on the Efficiency of Testing for an Interaction Effect in Exponential Family Models. *Journal of the American Statistical Association*. 2004;99(467):822-831.

5. Altman DG, Lausen B, Sauerbrei W, Schumacher M. Dangers of using "optimal" cutpoints in the evaluation of prognostic factors. *J Natl Cancer Inst*. 1994;86(11):829-35.

6. Royston P, Sauerbrei W. *Multivariable model-building : a pragmatic approach to regression analysis based on fractional polynomials for modelling continuous variables*. New York: John Wiley; 2008.

7. Schwarz G. Estimating the Dimension of a Model. *Ann Statist*. 1978;6(2):461-464.

8. Akaike H. A new look at the statistical model identification. *IEEE transactions on automatic control*. 1974;19(6):716-723.

9. Volinsky CT, Raftery AE. Bayesian information criterion for censored survival models. *Biometrics*. 2000;56(1):256-62.

10. Raftery AE. Bayesian Model Selection in Social Research. *Sociological Methodology*. 1995;25:111-163.

11. Royston P, Parmar MK. The use of restricted mean survival time to estimate the treatment effect in randomized clinical trials when the proportional hazards assumption is in doubt. *Stat Med*. 2011;30(19):2409-21.

12. Fine JP, Gray RJ. A Proportional Hazards Model for the Subdistribution of a Competing Risk. *Journal of the American Statistical Association*. 1999;94(446):496-509.

© 2021 Ali SA et al. *JAMA Oncology.*
