# Supplementary material for: Association of Bone Metastatic Burden With Survival Benefit From Prostate Radiotherapy in Patients With Newly Diagnosed Metastatic Prostate Cancer: A Secondary Analysis of a Randomized Clinical Trial
Source: JAMA Oncol. 2021 Feb 18;7(4):555–63. doi: 10.1001/jamaoncol.2020.7857 (PMC7893550; doi:10.1001/jamaoncol.2020.7857)
Supplement: Supplement 3. — Data Sharing Statement [file jamaoncol-e207857-s003.pdf]

Data Sharing Statement

Ali. Association of Bone Metastatic Burden With Survival Benefit From Prostate Radiotherapy in Patients With Newly Diagnosed Metastatic Prostate Cancer. *JAMA Oncol*. Published February 18, 2021. doi:10.1001/jamaoncol.2020.7857

Data
Data available: No

Additional Information
Explanation for why data not available: Patients enrolled in the trial did not provide informed consent for the data to be shared publicly. Therefore, data from the trial will not be shared publicly.
