# Supplementary material for: Association of Bone Metastatic Burden With Survival Benefit From Prostate Radiotherapy in Patients With Newly Diagnosed Metastatic Prostate Cancer: A Secondary Analysis of a Randomized Clinical Trial
Source: JAMA Oncol. 2021 Feb 18;7(4):555–63. doi: 10.1001/jamaoncol.2020.7857 (PMC7893550; doi:10.1001/jamaoncol.2020.7857)
Supplement: Supplement 1. — Trial Protocol [file jamaoncol-e207857-s001.pdf]

STAMPEDE

Systemic Therapy in Advancing or Metastatic Prostate Cancer: Evaluation of Drug Efficacy

A multi-arm multi-stage randomised controlled trial

Version: 9.0
Date: 22-Oct-2012

MRC CTU ID: PR08
ISRCTN #: ISRCTN78818544
NCT #: NCT00268476
EUDRACT #: 2004-000193-31
CTA #: 00316/0026/001-0001
MREC #: 04/MRE07/35

Authorised by:
Name: PROFESSOR NICHOLAS D JAMES
Role: CHIEF INVESTIGATOR

Name: MATTHEW SYDES
Role: TRIAL STATISTICIAN

GENERAL INFORMATION

This document was constructed using the MRC CTU Protocol Template Version 4.0. It describes the STAMPEDE trial, coordinated by the Medical Research Council (MRC) Clinical Trials Unit (CTU), and provides information about procedures for entering patients into it. The protocol should not be used as an aide-memoire or guide for the treatment of other patients. Every care has been taken in drafting this protocol, but corrections or amendments may be necessary. These will be circulated to the registered investigators in the trial, but sites entering patients for the first time are advised to contact the Cancer Group, MRC CTU, London, UK, to confirm they have the most up-to-date version.

COMPLIANCE

The trial will be conducted in compliance with the approved protocol, the Declaration of Helsinki 1996, the principles of Good Clinical Practice (GCP), Commission Directive 2005/28/EC with the implementation in national legislation in the UK by Statutory Instrument 2004/1031 and subsequent amendments, the UK Data Protection Act (DPA number: Z5886415), and the National Health Service (NHS) Research Governance Framework for Health and Social Care (RGF). International sites will comply with the principles of GCP as laid down by the ICH topic E6 (Note for Guidance on GCP), Commission Directive 2005/28/EC (the European Directive 2001/20/EC [where applicable]) and applicable national regulations.

SPONSOR

Medical Research Council, 2nd Floor, David Phillips Building, Polaris House, North Star Avenue, Swindon, SN2 1FL, UK

FUNDING

Clinical Trials Advisory Awards Committee (on behalf of Cancer Research UK, Medical Research Council, and other charities) together with educational grants from Novartis, Aventis and Janssen Pharma NV.

AUTHORISATIONS AND APPROVALS

The following persons are authorised to sign the final protocol and protocol amendments for the sponsor: Professor N James (Chief Investigator) and Matthew Sydes (Trial Statistician).

TRIAL REGISTRATION

This trial has been registered with the ClinicalTrials.gov Clinical Trials Register, where it is identified as NCT00268476.

RANDOMISATIONS
To randomise, call MRC CTU, Monday to Friday 0900-1700
excluding public holidays or dates when notice has been given by the CTU.
Tel: +44 (0) 20 7670 4777

SAE REPORTING
Fax to 020 7670 4818 within 24 hours of becoming aware of the event

TRIAL ADMINISTRATION

COORDINATING SITE

MRC Clinical Trials Unit
Aviation House
Kingsway
London
WC2B 6NH
UK

Switchboard: 020 7670 4700
Fax: 020 7670 4818
Email: stampede@ctu.mrc.ac.uk

MRC CTU STAFF

Trial Manager:	Francesca Schiavone	Tel:	0207 670 4632
Trial Manager:	Charlene Green	Tel:	0207 670 4882
Data Manager:	Hannah Gardner	Tel:	0207 670 4809
Data Manager:	Dominic Hague	Tel:	0207 670 4947
Data Manager:	Katie Ward	Tel:	0207 670 4794
Statistician:	Gordana Jovic	Tel:	0207 670 4647
Senior Trial Statistician:	Matt Sydes	Tel:	0207 670 4798
Director of MRC CTU:	Max Parmar	Tel:	0207 670 4729
Trial Surgeon:	Alastair Ritchie		

Chief Investigator

Professor Nicholas James	Tel:	0121 414 4097/4474
School of Cancer Sciences	Fax:	0121 414 3263
University of Birmingham	Email:	n.d.James@bham.ac.uk
Edgbaston, Birmingham		j.d.alonzo@bham.ac.uk(sec)
B15 2TT		
UK		

Co-Investigators

Prof Noel Clarke, Urologist	Manchester, UK
Prof David Dearnaley, Oncologist	Sutton, UK
Prof Johann de Bono, Oncologist	Sutton, UK
Prof Malcolm Mason, Oncologist	Cardiff, UK
Dr Chris Parker, Oncologist	Sutton, UK
Dr Martin Russell, Oncologist	Glasgow, UK
Prof George Thalmann, Urologist	Bern, Switzerland

Patient Representative

Robin Millman, Patient

Health Economics

Prof Mark Sculpher	York, UK

Molecular Genetics

Prof John Masters, Pathologist	London, UK

For full details of all trial committees, please see **Appendix M**.

CONTENTS

GENERAL INFORMATION I

CONTENTS 4

ABBREVIATIONS 8

1 SUMMARY 11
1.1 LAY SUMMARY 11
1.2 ABSTRACT AND SUMMARY OF TRIAL DESIGN 12
1.3 TRIAL DOCUMENTATION 16

2 BACKGROUND 17
2.1 INTRODUCTION AND SETTING 17
2.1.1 Long-term Androgen Deprivation Therapy 17
2.1.2 Role of Radiotherapy for Patients With M0 Disease 17
2.2 RATIONALE 17
2.3 DESIGN 18
2.4 RESEARCH TREATMENT AND BIOSPHOSPHONATES 18
2.5 RESEARCH TREATMENT: CHEMOTHERAPY 19
2.6 RESEARCH TREATMENT: CYCLOOXYGENASE-2 INHIBITORS 19
2.7 RESEARCH TREATMENT: STEROID SYNTHESIS INHIBITORS 20
2.8 RESEARCH TREATMENT: RADIOTHERAPY TO THE PROSTATE FOR PATIENTS WITH NEWLY-DIAGNOSED METASTATIC DISEASE 20
2.9 RESEARCH TREATMENT: COMBINATIONS 21
2.9.1 Bisphosphonate And Chemotherapy 21
2.9.2 Bisphosphonate And Cyclooxygenase-2 Inhibitors 21

3 SELECTION OF INSTITUTIONS AND INVESTIGATORS 23
3.1 RADIOTHERAPY ACCREDITATION 23

4 SELECTION OF PATIENTS 24
4.1 PATIENT INCLUSION CRITERIA 24
4.1.1 High-Risk Newly Diagnosed Non-Metastatic Node-Negative Disease 24
4.1.2 Newly Diagnosed Metastatic Or Node-Positive Disease 24
4.1.3 Previously Treated With Radical Surgery And/Or Radiotherapy, Now Relapsing 24
4.1.4 For All Patients 24
4.2 PATIENT EXCLUSION CRITERIA 25
4.3 SELECTION CRITERIA FOR COMPARISON OF RESEARCH (M1) RT FOR METASTATIC DISEASE 26
4.4 SCREENING PROCEDURES 26
4.4.1 Investigations Prior To Randomisation 26
4.4.2 Androgen Deprivation Therapy Prior To Randomisation 26
4.4.3 Hypercalcaemia At Randomisation 27
4.4.4 NSAIDs And Cox-2 Inhibitors At Randomisation 27
4.4.5 Starting Trial Treatment 27
4.4.6 Concomitant Medications 27
4.5 ADDITIONAL DETAILS FOR PATIENTS JOINING SUB-STUDIES 28

5 RANDOMISATION AND ENROLMENT ... 29
5.1 CO-ENROLMENT GUIDELINES ... 29

6 TREATMENT OF PATIENTS ... 30
6.1 TRIAL TREATMENT ... 30
6.1.1 Arm A: ADT Alone Or ADT + Standard-of-Care (M0) RT (Control Arm) ... 30
6.1.2 Arm B: ADT + Zoledronic Acid ... 31
6.1.3 Arm C: ADT + Docetaxel ... 31
6.1.4 Arm D: ADT + Celecoxib ... 31
6.1.5 Arm E: ADT + Docetaxel + Zoledronic Acid ... 31
6.1.6 Arm F: ADT + Zoledronic Acid + Celecoxib ... 32
6.1.7 Arm G: ADT + Abiraterone ... 32
6.1.8 ARM H: ADT + Prostate Radiotherapy in M1 patients ... 33
6.2 ADMINISTRATION AND DOSE MODIFICATIONS ... 33
6.2.1 Zoledronic Acid ... 33
6.2.2 Docetaxel ... 33
6.2.3 Celecoxib ... 34
6.2.4 Abiraterone ... 34
6.2.5 Research (M1) Prostate Radiotherapy ... 34
6.3 TRIAL PRODUCTS ... 36
6.4 MEASURES OF COMPLIANCE/ADHERENCE ... 36
6.5 TREATMENT DATA COLLECTION ... 36
6.6 ADMINISTRATION OF STANDARD RADIOTHERAPY TO NON-METASTATIC PATIENTS ... 36
6.6.1 Treatment Details ... 36
6.7 NON-TRIAL TREATMENT ... 38
6.7.1 Medications Permitted ... 38
6.7.2 Data On Concomitant Medication ... 38

7 ASSESSMENTS AND PROCEDURES ... 39
7.1 FLOW CHART/SCHEDULE FOR FOLLOW-UP ... 39
7.1.1 Psa Measurements ... 39
7.1.2 Assessment Of Treatment Failure (Definition Of Progression) ... 39
7.1.3 Additional Safety Assessment ... 39
7.1.4 Data Collection and Non-Administration Of Standard Radiotherapy ... 39
7.1.5 Data Collection Palliative Radiotherapy ... 40
7.1.6 Data Collection Research (M1) Radiotherapy ... 40
7.1.7 Follow-Up Schedules ... 40
7.2 FOLLOW-UP ... 40
7.3 TRIAL CLOSURE ... 42

8 STOPPING OF TREATMENT OR FOLLOW UP ... 44
8.1 STOPPING RESEARCH INTERVENTIONS ... 44
8.2 PATIENT TRANSFERS ... 44
8.3 WITHDRAWAL FROM THE TRIAL COMPLETELY ... 44

9 STATISTICAL CONSIDERATIONS ... 46
9.1 METHOD OF RANDOMISATION ... 46
9.2 OUTCOME MEASURES ... 46
9.3 SAMPLE SIZE: PRINCIPLES AND ASSUMPTIONS ... 47
9.4 SAMPLE SIZE ISSUES AND TRIAL STAGES: ORIGINAL RESEARCH ARMS (B-F) ... 48

9.4.1 Pilot Phase: Original Research Arms (B-F) 48
9.4.2 Activity Stages I-Iii: Original Research Arms (B-F) 48
9.4.3 Efficacy Stage Iv: Original Research Arms (B-F) 49
9.4.4 Sample Size For Original Research Arms (B-F) 49
9.5 SAMPLE SIZE ISSUES AND TRIAL STAGES: ADDITIONAL RESEARCH ARM G 49
9.5.1 Pilot Phase: Additional Research Arm G 49
9.5.2 Activity Stages I-Iii: Additional Research Arm G 49
9.5.3 Efficacy Stage Iv: Additional Research Arm G 50
9.5.4 Sample Size For Additional Research Arm G 50
9.6 SAMPLE SIZE ISSUES AND TRIAL STAGES: ADDITIONAL RESEARCH ARM H 50
9.6.1 Pilot Phase: Additional Research Arm H 50
9.6.2 Activity Stages I-III: Additional Research Arm H 50
9.6.3 Efficacy Stage Iv: Additional Research Arm H 50
9.6.4 Sample Size For Additional Research Arm H 50
9.7 FURTHER NOTES ON TRIAL DESIGN 53
9.7.1 Overall Sample Size 53
9.7.2 Factorial Design 53
9.8 INTERIM MONITORING AND ANALYSES 53
9.9 OUTLINE ANALYSIS PLAN 53
9.9.1 Pilot / Safety Phases 54
9.9.2 Activity And Efficacy Stages 54

10 MONITORING AND QUALITY ASSURANCE 55
10.1 MONITORING AT MRC CTU 55
10.2 DIRECT ACCESS TO DATA 55
10.3 VISITS TO INVESTIGATOR SITES 55
10.4 CONFIDENTIALITY 55

11 SAFETY REPORTING 56
11.1 DEFINITIONS 56
11.1.1 Trial-Specific Exemptions 57
11.2 INSTITUTION/INVESTIGATOR RESPONSIBILITIES 57
11.2.1 Investigator Assessment 57
11.2.2 Notification Procedure 58
11.3 MRC CTU RESPONSIBILITIES 59

12 ETHICAL CONSIDERATIONS AND APPROVAL 60
12.1 ETHICAL CONSIDERATIONS 60
12.2 ETHICAL APPROVAL 60

13 REGULATORY APPROVAL 62

14 INDEMNITY 63

15 FINANCE 64

16 TRIAL COMMITTEES 65
16.1 TRIAL MANAGEMENT GROUP (TMG) 65
16.2 TRIAL STEERING COMMITTEE (TSC) 65
16.3 INDEPENDENT DATA MONITORING COMMITTEE (IDMC) 65

17 ANCILLARY STUDIES....67
17.1 QUALITY OF LIFE....67
17.2 HEALTH ECONOMICS....67
17.3 TRANSLATIONAL SUB-STUDIES....67
17.3.1 DNA Analysis....67
17.3.2 Tissue Microarray....68

18 PUBLICATION....69

19 PROTOCOL AMENDMENTS....70
19.1 PROTOCOL....70
19.1.1 Amendments Made To Sections In Protocol Version 1.0 (May 2004)....70
19.1.2 Amendments Made To Sections In Protocol Version 1.1 (May 2005)....70
19.1.3 Amendments Made To Sections In Protocol Version 2.0 (Jun 2005)....70
19.1.4 Amendments Made To Section In Protocol Version 3.0 (Jul 2006)....71
19.1.5 Amendments Made To Protocol Version 4.0 (Dec 2007)....72
19.1.6 Amendments Made To Protocol Version 5.0 (Aug 2008)....72
19.1.7 Amendments Made To Protocol Version 6.0 (Jul 2009)....73
19.1.8 Amendments Made To Protocol Version 7.0 (Jul 2011)....73
19.1.9 Amendments Made To Protocol Version 7.1 (Jul 2011)....74
19.1.10 Amendments Made To Protocol Version 8.0 (Sep 2011)....75

20 REFERENCES....77

ABBREVIATIONS

Abbreviation	Expansion
ACE	Angiotensin-Converting Enzyme
ACTH	Adrenocorticotropic hormone
ADT	Androgen deprivation therapy
AS	Activity Stage
bid	Twice a day (bis in die)
BP	Blood pressure
BSA	Body surface area
CERES	Consumers for Ethics in Research
CF	Consent Form
CI	Chief Investigator
CI	Confidence interval
COSTART	Coding Symbols for a Thesaurus of Adverse Reaction Terms
Cox 2	Cyclooxygenase 2
CRF	Case Report Form
CRUK	Cancer Research UK
CRPC	Castrate Refractory Prostate Cancer
CT	Computerised tomography
CTA	Clinical Trials Authorisation
CTAAC	Clinical Trials Advisory and Awards Committee
CTC	Common Toxicity Criteria
CTU	Clinical Trials Unit
CTV	Clinical Tumour Volume
CXR	Chest X-ray
DDX	Doctors and Dentists Exemption
DNA	Deoxyribonucleic Acid
DPA	Data Protection Act
ERC	Endpoint Review Committee
ES	Efficacy Stage
ICH	International Conference on Harmonization
ECG	Electro cardiogram
FBC	Full Blood Count
FFS	Failure-Free Survival

Abbreviation	Expansion
GCP	Good Clinical Practice
GP	General Practitioner
GRO	General Register Office
HE	Health Economics
HES	Hospital Episode Statistics
hr	Hour
HR	Hazard Ratio
HRPC	Hormone Refractory Prostate Cancer
HT	Hormone Therapy
IDMC	Independent Data Monitoring Committee
IM	Intramuscular
IMRT	Intensity Modulated Radiation Therapy
ISRCTN	International Standard Randomised Controlled Trial Number
IU	International Units
IV	Intravenous
LD	Longest diameter
LFTs	Liver Function Tests
LHRH	Luteinising Hormone Releasing Hormone
LREC	Local Research Ethics Committee
MHRA	Medicine and Healthcare Products Regulatory Agency
m	Month
min	Minutes
MRC	Medical Research Council
MREC	Multi-Centre Research Ethics Committee
MRI	Magnetic resonance imaging
NCI	National Cancer Institute (USA)
NCRN	National Cancer Research Network
NHS	National Health Service
NSAID	Non-Steroidal Anti-inflammatory Drugs
ONS	Office for National Statistics
OS	Overall Survival
PI	Principal Investigator
PIS	Patient Information Sheet
po	per orum (orally)

Abbreviation	Expansion
PSA	Prostate Specific Antigen
pts	Patients
PTV	Planned Tumour Volume
QALY	Quality-adjusted Life Years
qds	quater die sumendus (4 times each day)
QL	Quality of Life
R&D	Research and Development
RECIST	Response Evaluation Criteria In Solid Tumours
SAE	Serious Adverse Event
SAR	Serious Adverse Reaction
sc	Sub-cutaneous (under skin)
SNP	Single Nucleotide Polymorphism
SSA	Site Specific Assessment
STAMPEDE	Systemic Therapy in Advancing and Metastatic Prostate Cancer: Evaluation of Drug Efficacy
SUSAR	Suspected Unexpected Serious Adverse Reactions
SWOG	South West Oncology Group
TMG	Trial Management Group
TURP	Trans-Urethral Resection of Prostate
TSC	Trial Steering Committee
ULN	Upper Limit of Normal
U+E	Urea and Electrolytes
WHO	World Health Organisation

1 SUMMARY

1.1 LAY SUMMARY

Prostate cancers depend upon the male hormone testosterone for their growth. Lowering testosterone levels (either by removing all or part of both testes, or by giving anti-hormone treatment) slows the growth of prostate cancers. This type of treatment is called hormone treatment and is often used when prostate cancers have spread outside the prostate gland. Although hormone treatment is usually successful at stopping the cancer growing for a period of time, the cancer will begin to grow again in most men.

There are increasing numbers of treatments available for advanced prostate cancer. These treatments are usually used in prostate cancer when hormone treatment is no longer effective and the cancer has started to grow again. The aim of this trial, which is called STAMPEDE, is to assess four of these treatments, given earlier in the course of the disease in combination with hormone treatment.

The treatments assessed during the trial are:

1. Zoledronic acid: Prostate cancer cells can spread to bones and weaken them. Zoledronic acid is a drug that reduces bone destruction and hardens bones. This may make them more resistant to attack by cancer cells.

2. Docetaxel: A drug that stops cells replicating that is currently being used to treat a range of cancers including lung, breast and ovarian cancer as well as prostate cancer. Docetaxel prolongs survival in men with relapsed metastatic prostate cancer.

3. Celecoxib: An aspirin-like drug that is used to treat arthritis. It slows down the growth of cancer cells in the laboratory. We wished to see if it had the same effect on cancer cells in patients. Recruitment to new patients for the evaluation of this drug is finished as a planned interim analysis failed to demonstrate sufficient activity.

4. Abiraterone (included from protocol version 8.0)**:** An inhibitor of steroid hormone synthesis that blocks prostate cancer cells from generating their own male hormones. This is thought to be a major way in which prostate cancer cells resume growth following castration based therapies. The agent prolongs survival when given to men following failure of docetaxel chemotherapy.

5. Prostate radiotherapy (included from protocol version 9.0)**:** treatment with high-energy x-rays targeted to the prostate gland. This treatment is now mandatory for patients with cancer that is confined to the prostate gland as large trials have shown it improves survival times. We are not certain whether we should give radiotherapy to the prostate if the cancer has already spread.

STAMPEDE will look at the effect of combining one or two of the treatments described above with hormone treatment. A computer program will be used to allocate which treatment the patient receives, using a chance process. The trial will look at the effects of the combined treatments on quality of life and find out whether the new treatment combinations increase the time when the cancer is not growing and ultimately results in patients living longer. The study will also look at which treatment provides the greater value for money for the health service. More than 5,000 patients will join the trial with answers becoming available over 7 to 12 years.

1.2 ABSTRACT AND SUMMARY OF TRIAL DESIGN

STAMPEDE is a multi-centre, randomised controlled trial for patients with locally advanced or metastatic prostate cancer who are about to commence Androgen Deprivation Therapy (ADT). Patients can have either newly diagnosed disease, or have been previously treated with radical radiotherapy or surgery but now have a rising prostate specific antigen (PSA) (further details on eligibility see **Section 4**). The trial will assess the effects of adding different agents, both as single agents and in combinations, to androgen deprivation therapy. The investigational agents are (i) a bisphosphonate, zoledronic acid, (ii) a cytotoxic chemotherapeutic agent, docetaxel and (iii) a cyclooxygenase (Cox-2) inhibitor, celecoxib. (iv) a novel androgen deprivation therapy drug called abiraterone, a steroid synthesis inhibitor. Recruitment to the celecoxib arms (D and F) is now closed. An additional arm containing abiraterone was added in protocol version 8.0. A further comparison arm involving prostate radiotherapy for patients with metastatic disease is added in the current protocol version 9.0. The trial has multiple arms; the control arm of the trial is androgen deprivation therapy (ADT) only, achieved through the use of luteinising hormone releasing hormone (LHRH) analogues or LHRH antagonists, or bilateral orchidectomy according to local practice. The other trial arms are summarised in **Figure 1 to 5**.

Figure 1: Recruiting arms of the STAMPEDE Trial to Apr-2011

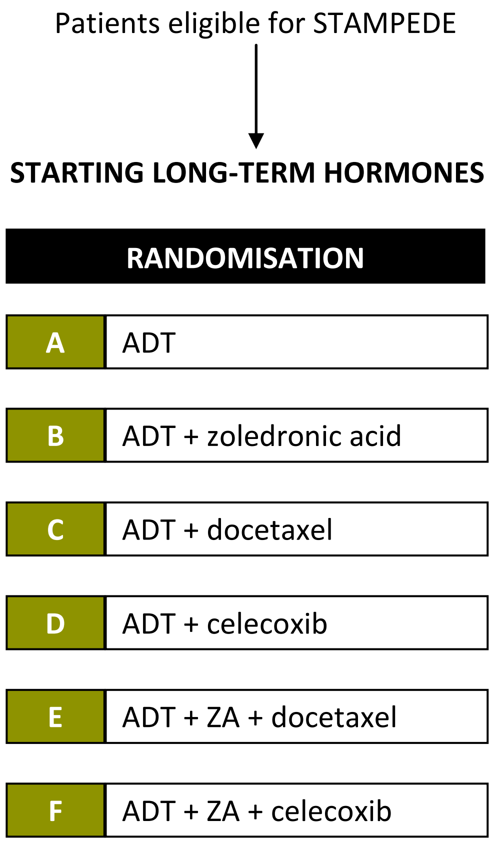

Figure 2: Recruiting arms of the STAMPEDE Trial from Apr-2011 to Nov-2011 (v7.0)

Patients eligible for STAMPEDE

↓

STARTING LONG-TERM HORMONES

RANDOMISATION

**A**	ADT
**B**	ADT + zoledronic acid
**C**	ADT + docetaxel
**E**	ADT + ZA + docetaxel

Figure 3: Recruiting arms of the STAMPEDE Trial from Nov-2011 to Jan-2013 (v9.0)

Patients eligible for STAMPEDE

↓

STARTING LONG-TERM HORMONES[1]

RANDOMISATION

**A**	ADT
**B**	ADT + zoledronic acid
**C**	ADT + docetaxel
**E**	ADT + ZA + docetaxel
**G**	ADT + abiraterone

[1] All suitable pts with newly diagnosed locally advanced disease should also have RT to the prostate

Figure 4: Recruiting arms of the STAMPEDE trial from protocol version 9.0

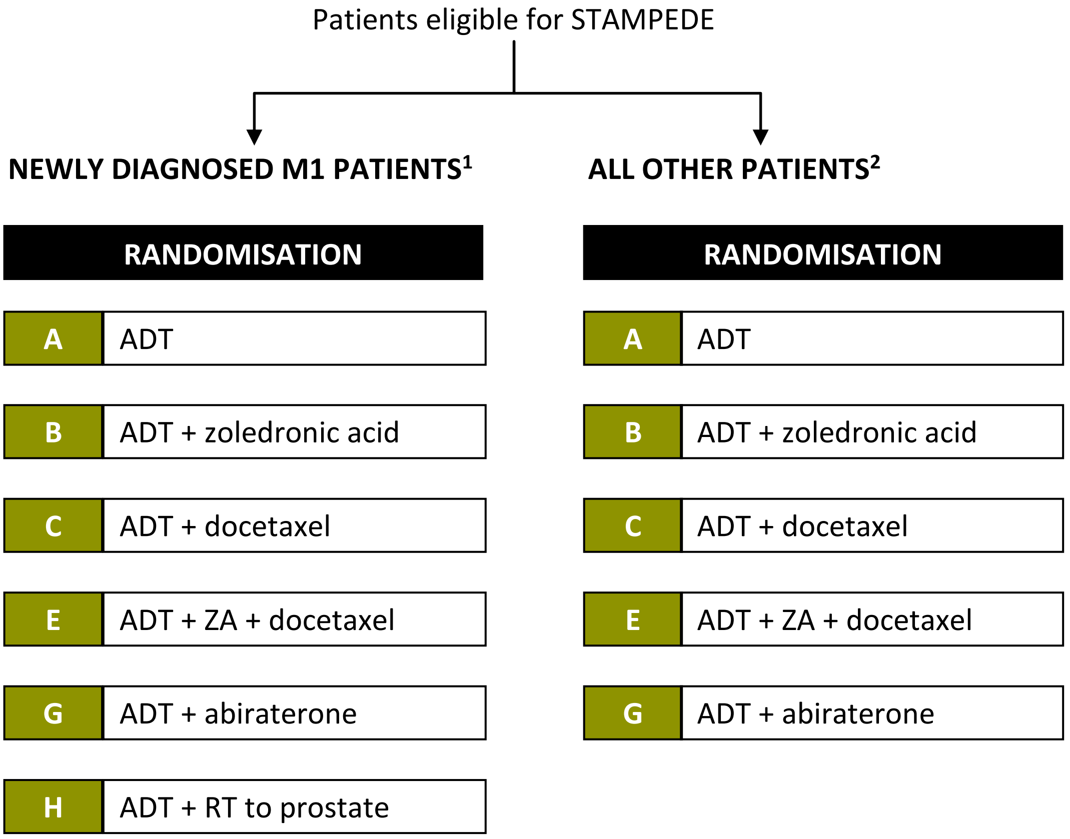

[1] Except pts with a contra-indication to RT
[2] All suitable pts with newly diagnosed locally advanced disease should also have RT to the prostate

Figure 5: Arms of the STAMPEDE Trial from protocol version 9.0 after original research arms complete accrual

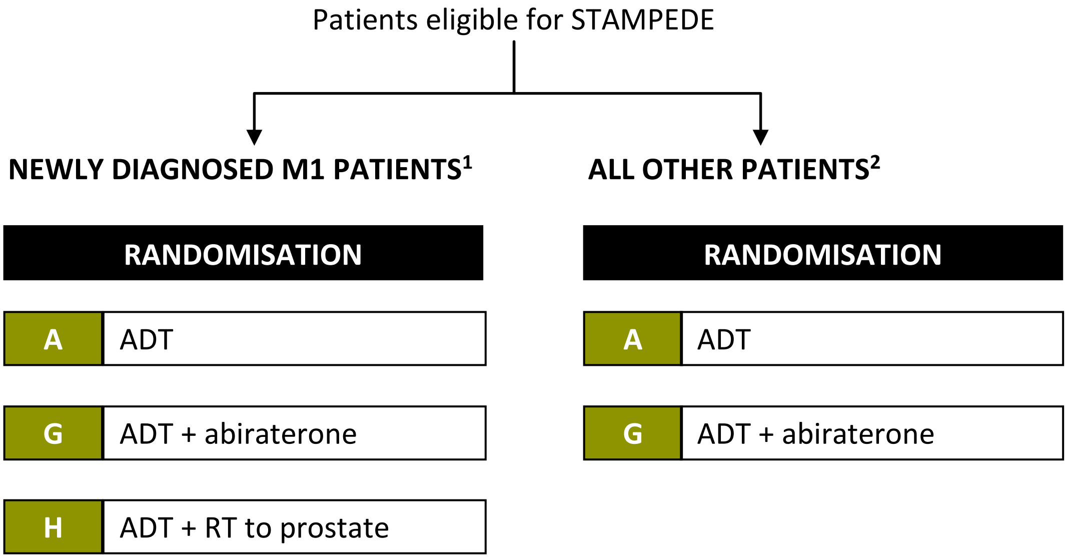

[1] Except pts with a contra-indication to RT
[2] All suitable pts with newly diagnosed locally advanced disease should also have RT to the prostate1

For each comparison of research arm against control, the trial will be conducted in five stages: a Pilot Phase, Activity Stages I to III and Efficacy Stage IV. The primary outcome measure of the Pilot Phase is the safety, with 30 patients recruited to each research arm. Research arms will only continue to recruitment in the next stage if they have been shown to be both safe and feasible, although patient data from all patients and all stages will be included in the final analyses. In Activity Stages I-III the primary outcome measure is failure-free survival (FFS). Further patients will be recruited until a certain number of FFS events have been observed in the control arm (see **Section 9** for further detail). Some evidence of activity will be required for a research arm to proceed to further recruitment in each stage and guidelines are in place. In Efficacy Stage IV, patients will be recruited when a certain number of primary outcome measure events have been reported. This is when around 403 deaths have been reported in the control arm for the original research comparisons and 267 (control arm deaths) for both the abiraterone comparison and the local-RT-for-M1-disease comparison. The exact number of patients and duration of the trial will depend on the observed accrual rate, observed event rate and the number of patients accruing at each stage.

Recruitment to arms D (ADT + celecoxib) and F (ADT + zoledronic acid + celecoxib) was stopped in Apr-2011 after the second planned activity analysis when the IDMC and TSC considered the lack-of-benefit guidelines.(1) Refer to **Section 9.3**. for further information regarding the guidelines for stopping accrual to research arms during the activity stages of the trial.

In version 8.0 of the protocol a new arm G (ADT + abiraterone) was added. Arm H (ADT+ prostate radiotherapy) will be added in protocol version 9.0. The trial stages remain as at trial inception but will be staggered with respect to the stages for the original arms A-F.

Patients will be assessed 6 weekly for the first 24 weeks after randomisation and then every 12 weeks up to 2 years, then 6-monthly until 5 years and annually, thereafter. The first 700 patients on trial completed questionnaires aimed at assessing the effects of the investigational treatments on

their quality of life (QL) and on their use of health care resources (Health Economics (HE) study). From protocol version 8.0, the QL and HE study has been re-opened to all new patients.

In addition, there are translational sub-studies. Patients willing to participate will be asked to donate a droplet of blood at randomisation which will be stored for either DNA and protein analysis in order to try to identify markers that are associated with response to therapy, side-effects or susceptibility to prostate cancer.

Patients will also be asked to give permission to use some of their stored material for further studies on the causes and nature of prostate cancer. In selected centres patients were asked to participate in a bone mineral density sub-study. This sub-study has now stopped recruitment. There are separate patient information sheets for the QL and HE study and the translational sub-studies (For further details of ancillary studies, see Section 17).

1.3 TRIAL DOCUMENTATION

Table 1 presents a summary of the required trial documentation for participating centres and Table 2 presents a summary of the timings of the case report forms (CRFs) for your randomised patients.

Table 1: Summary of trial documentation required ahead of initial accreditation

TRIAL DOCUMENTATION	TIMING
R&D approval (including IRMER approval)	Before centre participation
Investigator Statement	Before centre participation
Signature list & delegation of responsibilities	Before centre participation
Trial personnel contact details	Before centre participation
PIS, GP & CF on local paper	Before centre participation
Signed Clinical Trial Agreement between Trust and Sponsor	Before centre participation

Table 2: Summary of trial documentation required ahead of re-accreditation (protocol v9.0 only)

TRIAL DOCUMENTATION	TIMING
R&D approval (including IRMER approval)	Before centre re-activation
PIS, GP & CF on local paper	Before centre re-activation
RTQA accreditation	Before centre re-activation

2 BACKGROUND

2.1 INTRODUCTION AND SETTING

Prostate cancer is a major health problem world-wide and accounts for nearly one fifth of all newly diagnosed male cancers. In the UK, approximately 35,000 men are diagnosed with prostate cancer each year and in 2008 almost 10,000 men died from the disease.(2)

2.1.1 Long-term Androgen Deprivation Therapy

The initial (first line) treatment for locally advanced or metastatic prostate cancer is androgen deprivation therapy (ADT) achieved either surgically with bilateral orchidectomy, or medically with LHRH agonists or antagonist or oral anti-androgens alone. (3) Oral anti-androgens were permitted in the trial but were used by very few patients and are no longer permitted for new patients within the trial from version 8.0.

ADT produces responses in up to 95% of patients but it is not curative and disease recurs in virtually all patients treated with ADT as sole therapy, with a median time to progression of 18-24 months. (3) Such disease is referred to as hormone or increasingly as castrate refractory prostate cancer (HRPC or CRPC).

2.1.2 Role of Radiotherapy for Patients With M0 Disease

Two randomised trials, SPCG7 (4) and NCIC PR.3 / MRC PR07 (5-7) have tested the question of whether androgen deprivation therapy alone or combined with radiotherapy is the best treatment for high-risk patients with no evidence of spread outside the pelvis. Both trials demonstrate an improvement in overall and disease specific survival from the addition of radiotherapy to androgen deprivation therapy. The size of this overall survival benefit is substantial (hazard ratio 0.68 in SPCG7 and HR 0.77 in PR07). With substantial benefit demonstrated in two mature, large, well conducted randomised trials, we now recommend that radiotherapy be considered standard for patients with no nodal or metastatic spread. Patients in this category will now only be allowed to enter the trial if standard radiotherapy is planned, with the exception of those for whom radiotherapy is contra-indicated who should be discussed with the Trials Unit prior to inclusion. For patients with node positive, M0 disease there are no clear data on whether radiotherapy is or is not indicated. The NCIC PR.3 / MRC PR07 trial included patients with unknown nodal status who received whole pelvic radiotherapy. Given the large overall benefit observed in this trial, the STAMPEDE TMG recommends that pelvic nodal radiotherapy be considered for patients with node positive, non-metastatic disease at the discretion of the treating clinician.

2.2 RATIONALE

There are increasing numbers of treatments which are used post relapse of first-line androgen deprivation therapy in patients with CRPC, but little evidence as to which is associated with the best response or how they may be combined or sequenced or whether any of them might have a role in first-line treatment. Such treatments include further hormonal manipulations (8), bisphosphonates, (9) cytotoxic chemotherapy (10), new hormone therapies (11) and palliative radiotherapy. The traditional approach to the testing and introduction of new treatments for prostate cancer is to use them in patients with castrate refractory disease. An alternative approach is to investigate new drugs and new approaches to treatment, as first-line therapy in patients starting androgen

deprivation therapy. At this point patients should be fitter and better able to tolerate treatment than when they have CRPC and there is the possibility of having a larger and longer lasting effect.

2.3 DESIGN

STAMPEDE (also known as MRC PR08) is an innovative, multi-arm multi-stage, multi-centre, randomised controlled trial. It initially assessed the effects of a bisphosphonate (zoledronic acid), a cytotoxic chemotherapeutic agent (docetaxel) and a cyclooxygenase (Cox-2) inhibitor (celecoxib), as single agents or combinations, in patients commencing androgen deprivation therapy for advancing or metastatic prostate cancer. Each comparison is divided into five stages such that, for each investigational arm, safety and activity data are generated in the first four stages; an investigational arm will only proceed to the fifth and final stage of recruitment, where it will be assessed for its effect on overall survival, if it has been shown to be sufficiently safe and active. It is important to note, however, that patient data from all arms and all stages will be included in the final analyses of the primary outcome measure, even if the investigational arm did not proceed to the final stage.

Planned interim analysis failed to demonstrate sufficient activity for celecoxib (James, 2012 –ref to be added to list) and this agent has now been removed from the trial recruitment; patients remaining on celecoxib treatment reverted to standard care. Protocol version 8.0 added a new drug abiraterone to the study as an additional arm (see **Section 2.7**). Protocol version 9.0 adds a new comparison arm involving prostate radiotherapy for patients with metastatic disease (see **Section 2.8**).

2.4 RESEARCH TREATMENT AND BIOSPHOSPHONATES

The bisphosphonates are a class of drug that act by reducing osteoclast formation, inhibiting osteoclast activity and inducing osteoclast apoptosis. They are effective at controlling hypercalcaemia and preventing skeletal complications associated with malignant disease. (12, 13) Zoledronic acid is a highly potent, third generation bisphosphonate; studies comparing the efficacy of zoledronic acid to other bisphosphonates suggest that zoledronic acid has a 40-850 fold higher potency than clodronate in preclinical models of bone resorption. (14). It has also been shown to be more effective than pamidronate (90mg) in controlling malignant hypercalcaemia. (15) In addition, zoledronic acid has also demonstrated direct anti-cancer activity, including inhibition of proliferation of breast cancer and prostate cancer cells in vitro. (16)

In randomised controlled trials of 1,648 patients, 4mg zoledronic acid was more effective than pamidronate in reducing the risk of skeletal complications in patients with bone metastases from breast cancer. (17, 18) Also, in metastatic prostate cancer, zoledronic acid has been shown to reduce the rate of skeletal-related events compared to placebo in a trial involving 429 men. (19) In April 2002, zoledronic acid received approval from the Committee for Proprietary Medicinal Products for the prevention of skeletal-related events (for example, fractures) in patients with any advanced malignancies involving bone.

The MRC PR05 prostate cancer trial showed that a first generation bisphosphonate (clodronate) commenced at the time of androgen deprivation therapy initiation, delayed time to progression in patients with bony metastatic disease and there was some evidence that it may also improve survival. (20) There is, therefore, a good rationale for investigating a more potent bisphosphonate in patients with prostate cancer who are about to commence ADT therapy.

2.5 RESEARCH TREATMENT: CHEMOTHERAPY

There is increasing evidence of the clinical efficacy of chemotherapy in prostate cancer. (10) Two randomised phase III trials in patients with metastatic hormone refractory prostate cancer (HRPC) using a docetaxel-containing regimen have been completed: the SWOG 9916 study (21) and the TAX-327 study. (22) Both studies show that the use of a docetaxel-based regimen improved survival for patients with metastatic HRPC and had significantly greater PSA response rates compared to the mitoxantrone plus prednisolone arm.

In the TAX-327 trial, (22) 1,006 patients with metastatic HRPC were randomized to receive either mitoxantrone 12 mg/m^2 with prednisone 10mg daily (Arm C) or docetaxel 75mg/m^2 3-weekly for 10 cycles with prednisone (Arm A) or docetaxel 30 mg/m^2/wk x 5 of 6 weeks x 5 cycles with prednisone (Arm B). Median overall survival was 16.5 months for patients treated with mitoxantrone versus 18.9 months for the 3-weekly docetaxel regimen (hazard ratio 0.76 (0.62-0.94)). There was also improvements for 3-weekly docetaxel in pain (22% vs 35%, p = 0.01) and PSA response (32% vs 45%, p=0.0005).

In June 2006 in the UK docetaxel was given NICE (National Institute for Health and Clinical Excellence) approval for use in hormone (now more commonly termed castrate) refractory prostate cancer patients.

2.6 RESEARCH TREATMENT: CYCLOOXYGENASE-2 INHIBITORS

Note: recruitment completed to both celecoxib-containing arms in Apr-2011 at the end of Activity Stage II of this comparison

Cyclooxygenase-2 (Cox-2) is an isoenzyme induced by a variety of mitogens, cytokines and growth factors that are associated with a range of process including inflammation, (23) and carcinogenesis.(24, 25) There is a growing body of evidence that inhibition of Cox-2 may play an important role in the prevention of cancer and the delay of progression in established cancer. A number of case-control studies have shown a reduction in risk of prostate cancer associated with the use of non-steroidal anti-inflammatory drugs (NSAID), which include inhibition of Cox-2 amongst their mode of action. (26) Pathological studies show Cox-2 is upregulated in carcinomas (27) and one study suggested that NSAID use may delay progression from subclinical to clinical prostate cancer. (28)

Celecoxib, a Cox-2 inhibitor, is better tolerated than other NSAIDs and there is evidence that it is active as a chemoprevention agent. (29) It also has important antineoplastic properties such as the ability to inhibit angiogenic factors and induce apoptosis in human cancer cells including prostate cancer. (30)

Evidence has suggested that an anti-cancer effect is only seen at higher doses of celecoxib than required for an anti-inflammatory effect. (31) Therefore, the dose of 800mg/day for STAMPEDE patients has been chosen. Although there is some high profile evidence of a small absolute increase in CVS toxicity risk associated with higher doses of celecoxib, (32) most current cancer trials are using a dose of 800mg/day as it is believed that a higher dose will result in a greater increase in cancer effect.

There is also some evidence of a schedule effect on CVS toxicity. It has been observed that CVS toxicity becomes evident after one year of taking celecoxib. (32) Therefore, a maximum duration of one year has been set for celecoxib use in this trial. Any potential risks of course have to be weighed against any potential benefits of celecoxib in the delay of progression in established prostate cancer.

Given case-control data suggesting effects on prostate cancer, pathological expression of Cox-2 in prostate cancer and in vitro data suggesting that inhibition of Cox-2 inhibits growth and invasiveness, further investigation in prostate cancer is warranted.

2.7 RESEARCH TREATMENT: STEROID SYNTHESIS INHIBITORS

Recent evidence suggests that an important mechanism for escape from tumour control by androgen ablation is the intracellular conversion of steroid precursors to androgenic steroids by prostate cancer cells. A key enzyme in this process is CYP17, which therefore represents a logical target for therapy in CRPC. (11) Abiraterone acetate is a selective inhibitor of CYP17 and is highly active in patients developing resistance to standard androgen ablation therapies. (33-35) Recruitment to a phase III study comparing abiraterone acetate to placebo in CRPC patients post-docetaxel, completed accrual in 2009 and reported initial results in 2010 with an improvement in overall survival of around 4 months and a hazard ratio of 0.65. (36) The drug has now received a marketing authorisation in the USA and in the EU from September 2011. A second trial in pre-chemotherapy CRPC patients completed recruitment April 2010 and preliminary results are positive. (37) Side-effects with abiraterone acetate are modest with the main adverse effects being elevated transaminases (usually mild), hypokalaemia and hypertension due to secondary hyperaldosteronism (preventable by low doses of glucocorticoids) and fluid retention. In order to prevent secondary hyperaldosteronism, it is recommended that prednisolone (or prednisone) 10mg daily be administered in the CRPC setting. Within more recent studies in earlier stage patients, lower doses (typically 5mg of prednisone/prednisolone) are being used due to concerns about long-term exposure to glucocorticoid side effects. More recent evidence even suggests that for most patients, no glucocorticoids may be needed. (38) Within the STAMPEDE trial, we propose to use a prednisone/prednisolone dose of 5mg daily.

We hypothesise that the agent may be more active still when given up-front in combination with first-line androgen deprivation therapy by preventing or delaying the development of castrate refractory disease.

2.8 RESEARCH TREATMENT: RADIOTHERAPY TO THE PROSTATE FOR PATIENTS WITH NEWLY-DIAGNOSED METASTATIC DISEASE

Therapy directed against the primary tumour in the presence of metastatic disease has been evaluated rigorously in only one malignancy to date: renal cell carcinoma. Two cooperative groups ran randomised trials enrolling patients with previously untreated metastatic RCC whose primary tumours were amenable to surgical resection. Patients were randomized to receive the standard systemic therapy of the day, interferon-alpha, either alone or with radical nephrectomy. The combination of nephrectomy and interferon was shown to significantly improve median survival from 7 to 17 months in one trial(39) and from 8 to 11 months in the other.(40) The mechanism by which nephrectomy improves survival remains obscure. In preclinical models, the primary tumour has been found to secrete molecules that prime the microenvironment in which metastases can develop. An implication of this work is that therapy directed at the primary tumour, by abrogating this endocrine signalling, could retard the formation and the growth of distant metastases.

The results of two large-scale randomised trials of prostate radiotherapy are also provocative. The Scandinavian SPCG-7 trial and the MRC PR07 trial randomised men with locally advanced prostate cancer, who were at high risk of possessing occult metastatic disease, to either androgen deprivation therapy (ADT) alone or ADT plus prostate radiotherapy.(4, 41) The addition of radiotherapy dramatically improved 10-year outcomes: mortality from prostate cancer was halved. Interestingly, the benefit of radiotherapy started to emerge as early as three years from the time of randomisation. This seems improbably early if the benefit of local treatment is mediated via the

prevention of subsequent disease dissemination. Rather, it is more consistent with the possibility that local treatment has a beneficial impact on the rate of progression of existing micrometastatic disease.

We hypothesise that local therapy to the primary site may retard distant disease progression and prolong survival in patients with metastatic prostate cancer.

2.9 RESEARCH TREATMENT: COMBINATIONS

2.9.1 BISPHOSPHONATE AND CHEMOTHERAPY

Zoledronic acid and docetaxel have different mechanisms of action. In addition to its skeletal protection activity, zoledronic acid has shown direct activity against prostate cancer cells, both in vitro and in vivo. (16) There is also in vitro and in vivo evidence to suggest synergy between zoledronic acid and chemotherapy in breast cancer cells and anti-angiogenic effects in patients. (42, 43)

Toxicities of the two agents are complementary and administration in combination is expected to be feasible and safe. These aspects were evaluated in the initial Pilot Phase of the trial. Since both agents show considerable promise as single agents and there is in vitro evidence of synergy, we believe there is a strong rationale for evaluating these two agents in combination.

2.9.2 BISPHOSPHONATE AND CYCLOOXYGENASE-2 INHIBITORS

Note: recruitment stopped to both celecoxib-containing arms in Apr-2011 at the end of Activity Stage II.

An alternative approach to combination therapy is to target the principal site of relapse and a key mode of progression and this is the rationale for combining zoledronic acid with a Cox-2 inhibitor. Bisphosphonates have already been shown to delay bone disease progression in hormone refractory disease. (20) Cox-2 appears to play a crucial role in the molecular phenotype of advanced prostate cancer as outlined above, and this effect is likely to be apparent in both soft tissue and in bone. Toxicities of the two agents are likely to be complementary and there is no strong a priori reason to anticipate unacceptable toxicity. The Pilot Phase of the trial will evaluate tolerability and safety of the combination. Targeting both bone progression and the underlying molecular changes leading to progression can be expected to have synergistic benefits in terms of delaying development of hormone refractory disease.

Figure 6: Use of RT in STAMPEDE

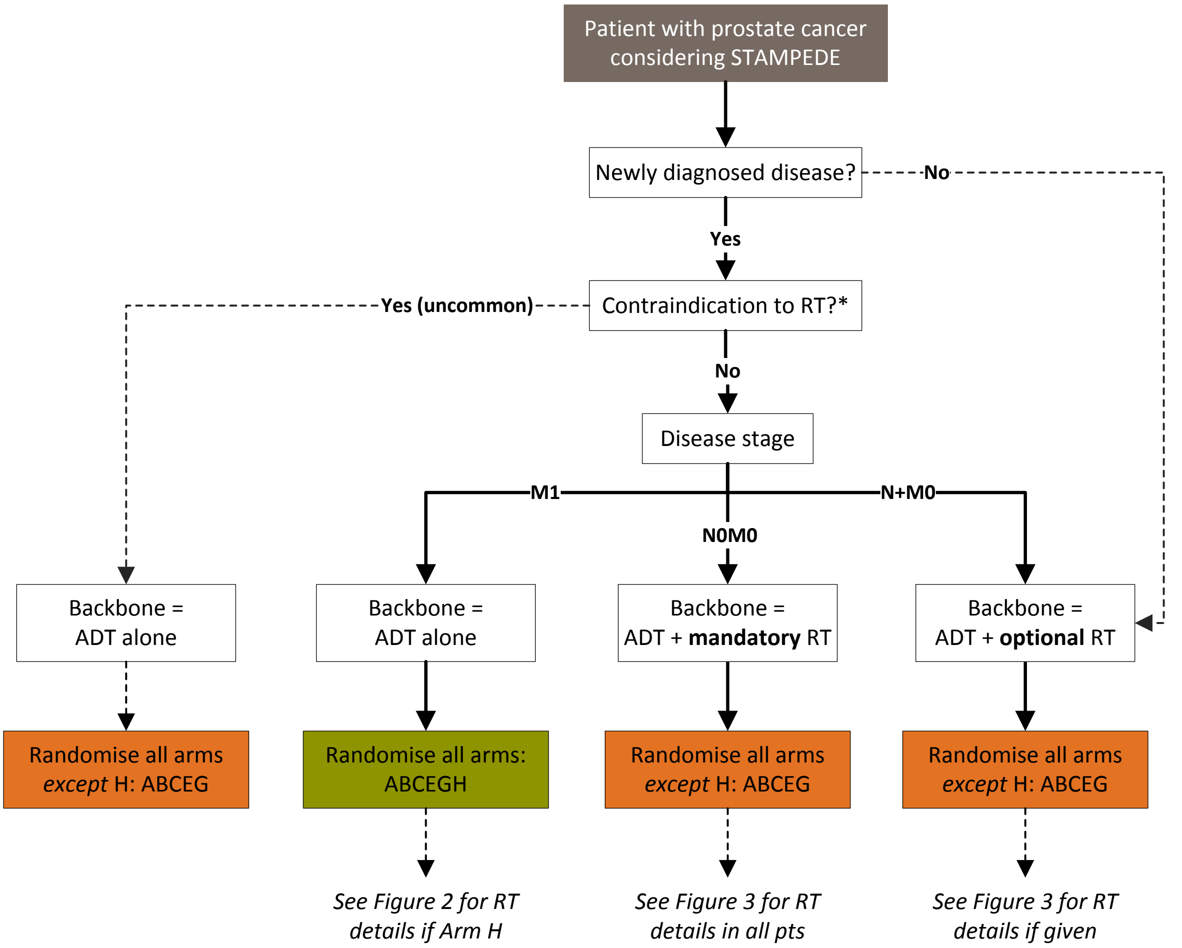

*It is expected that only around 1% of patients will have a contraindication to RT e.g. inflammatory bowel disease. These cases should be discussed with the trials unit prior to randomisation (see **Section 2.7**).

3 SELECTION OF INSTITUTIONS AND INVESTIGATORS

Centres who wish to participate in the STAMPEDE trial should be registered with the Medical Research Council Clinical Trials Unit (MRC CTU) for this purpose. Before any patients are randomised the MRC CTU must receive a completed and signed Investigator Statement. The STAMPEDE investigator statement is signed by the Principal Investigator for that institution (**Appendix M**). R&D approval for the site, along with a fully-signed model agreement are also required before recruitment can begin.

In addition and in compliance with the principles of GCP all institutions participating in the trial will complete a delegation log and forward this to the MRC CTU. Each person working on the STAMPEDE trial must sign off a section of this log indicating their responsibilities. The MRC CTU must be notified of any changes to trial personnel and/or their responsibilities. An up-to-date copy of this log must be stored in the Investigator Site file at the institution and also at the MRC CTU.

The Clinical Trial Authorisation (CTA) for the STAMPEDE trial requires that the Medicines and Healthcare Products Regulatory Agency (MHRA) be supplied with the names and addresses of all participating investigators/institutions. Trial staff at the MRC CTU will perform this task; hence, it is vital to receive full contact details for all investigators prior to their entering patients.

Finally, before a patient is entered into the trial written informed consent must be obtained. Approved patient information sheets and informed consent forms are supplied as templates.

Only a limited number of centres participated in the initial Pilot Phase of the original trial; this was to ensure that safety and feasibility data were collected expediently. Subsequent stages of the trial are open to any centre that wishes to participate and has fulfilled the requirements described above.

3.1 RADIOTHERAPY ACCREDITATION

The introduction of the RT comparison in v9.0 introduces the need for RTQA accreditation in sites giving radiotherapy. The details of RTQA accreditation is in **Appendix K**. However, centres that have been RTQA accredited for another multi-centre prostate radiotherapy trial in the UK (e.g. MRC RT01, RADICALS or CHHIP) will be automatically granted STAMPEDE RTQA accreditation.

4 SELECTION OF PATIENTS

4.1 PATIENT INCLUSION CRITERIA

Patients must fulfil both of the criteria in **Section 4.1.1** **or** one criterion in **Section 4.1.2** **or** at least one criteria in **Section 4.1.3**. Additionally, all patients must fulfil the criteria in **Section 4.1.4**.

4.1.1 High-Risk Newly Diagnosed Non-Metastatic Node-Negative Disease

Both:

- At least two of: Stage T3/4, PSA≥40ng/ml or Gleason sum score 8-10
- Intention to treat with radical radiotherapy (unless there is a contra-indication; exemption can sought in advance of consent, after discussion with MRC CTU)

OR

4.1.2 Newly Diagnosed Metastatic Or Node-Positive Disease

At least one of:

- Stage T_{any} N+ M0
- Stage T_{any} N_{any} M+

OR

4.1.3 Previously Treated With Radical Surgery And/Or Radiotherapy, Now Relapsing[1]

At least one of:

- PSA ≥4ng/ml and rising with doubling time less than 6 months
- PSA ≥20ng/ml
- N+
- M+

AND

4.1.4 For All Patients

I. Histologically confirmed prostate adenocarcinoma
II. Intention to treat with long-term androgen deprivation therapy
III. Fit for all protocol treatment[2] and follow-up, WHO performance status 0-2[3]
IV. Have completed the appropriate investigations prior to randomisation
V. Adequate haematological function: neutrophil count >$1.5x10^9$/l and platelets >$100x10^9$/l
VI. Estimated creatinine clearance >30ml/min
VII. Serum potassium ≥3.5mmol/L
VIII. Written informed consent
IX. Willing and expected to comply with follow-up schedule
X. Using effective contraceptive method if applicable

[1] Courses of hormone therapy for localised disease must have been completed at least 12 months previously and have been no longer than 12 months in duration. It can have been given as adjuvant or neoadjuvant therapy.
[2] Medical contraindications to the trial medications are given in **Appendix G**
[3] For WHO performance status definitions see **Appendix A**

4.2 PATIENT EXCLUSION CRITERIA[4]

Patients must not fulfil any of the criteria, below.

I. Prior systemic therapy for locally advanced or metastatic prostate cancer except as listed in **Section 4.1.3**.
II. Metastatic brain disease or leptomeningeal disease
III. Abnormal liver functions consisting of any of the following:
  - Serum bilirubin ≥1.5 x ULN (except for patients with Gilbert's disease, for whom the upper limit of serum bilirubin is 51.3µmol/l or 3mg/dl)
  - Aspartate aminotransferase (AST) or alanine aminotransferase (ALT) ≥2.5 x ULN
IV. Any other previous or current malignant disease which, in the judgement of the responsible physician, is likely to interfere with STAMPEDE treatment or assessment
V. Patients with active peptic ulceration, gastrointestinal bleeding, inflammatory bowel disease
VI. Symptomatic peripheral neuropathy grade ≥2 (NCI CTC)[5]
VII. Any surgery (e.g. TURP) performed within the past 4 weeks
VIII. Patients with significant cardiovascular disease such that, in the investigator's opinion, the patient is unfit for any of the study treatments. This might include:
  - Severe/unstable angina
  - Myocardial infarction less than 6 months prior to randomisation
  - Arterial thrombotic events less than 6 months prior to randomisation
  - Clinically significant cardiac failure requiring treatment (NYHA II-IV)6
  - Cerebrovascular disease (e.g. stroke or transient ischaemic episode) less than 2 years prior to randomisation
  - Patients with uncontrolled hypertension defined as systolic BP greater or equal than 160 mmHg or diastolic BP greater or equal than 95 mmHg
IX. Patients who have been scheduled to have major dental extractions within the next 2 years
X. Patients receiving treatment with drugs known to induce CYP3A4 (including phenytoin, carbamazepine, Phenobarbital)[7]
XI. Prior exposure to abiraterone
XII. Prior chemotherapy for prostate cancer
XIII. Prior therapy with zoledronic acid other than short-term treatment for hypercalcaemia
XIV. Prior exposure to policy of long-term hormone therapy before randomisation (unless as described in **Section 4.4.2**)

[4] The exclusion criteria for patients who have been on a Cox-2-inhibitor for 6+ months has been removed
[5] See **Appendix I** for common toxicity grading
[6] NYHA classifications can be found in **Appendix A**
[7] A full list is included in **Appendix G**

4.3 SELECTION CRITERIA FOR COMPARISON OF RESEARCH (M1) RT FOR METASTATIC DISEASE

All patients meeting criteria in Section 4.1 and 4.2 are eligible for the trial, but not all can be allocated to the research (M1) radiotherapy arm. The selection criteria for this "RT to the prostate" comparison are:

- Newly diagnosed prostate cancer
- Demonstrable M1 disease
- No contraindication to radiotherapy e.g. no previous pelvic radiotherapy,
- No previous radical prostatectomy

Patients meeting these criteria will have a chance to be allocated to Arms A and H.

4.4 SCREENING PROCEDURES

4.4.1 Investigations Prior To Randomisation

All patients should have the following examinations performed. The latest available scans should be used:

- CT or MRI of pelvis and abdomen
- Bone Scan
- Chest X-ray (only if chest was not included in CT)
- ECG
- PSA Test

The following blood tests within 8 weeks (56 days) prior to randomisation:

- Testosterone (if available)
- Urea and Electrolytes
- Liver function tests
- Serum creatinine
- Serum corrected calcium
- Phosphates
- Magnesium
- Albumin
- Total cholesterol
- HDL cholesterol
- Systolic blood pressure
- Diastolic blood pressure

Patients who initially fail to meet the eligibility criteria can be re-screened at a later date.

Prior to randomisation:

- Check details of any prior treatments for prostate cancer
- Check any contraindications to radiotherapy

4.4.2 Androgen Deprivation Therapy Prior To Randomisation

It is preferable that patients are not started on hormones prior to randomisation. However, if androgen deprivation therapy has already started, the primary therapy should have not have started more than 12 weeks before randomisation, and the baseline PSA measurement must be taken

before this was initiated (please report the latest PSA measurement taken before the start of androgen deprivation therapy).

Short periods (not exceeding 2 weeks duration) of prior anti-androgens to cover tumour flare are allowed but will not be counted in the 12 week time period mentioned above; but a PSA measurement must be taken before this is initiated.

Note that long-term anti-androgen monotherapy is not permitted in the trial for newly recruited patients from version 8.0 (see **Section 6.1**); patients may change treatment to join the trial, provided that they have not had more than 12 weeks of androgen deprivation therapy prior to randomisation. Further details on hormone therapies allowed prior to randomisation are discussed in **Appendix L**.

Relapsing patients previously treated with radical surgery or radiotherapy must have completed a policy of hormone therapy at least 12 months previously and have been no longer than 12 months in duration, given as adjuvant or neo-adjuvant therapy.

Note that baseline testosterone measurements will not be required in patients who have already commenced hormone manipulation prior to randomisation.

4.4.3 Hypercalcaemia At Randomisation

For patients who are hypercalcaemic prior to randomisation and require treatment, it is recommended that they are treated with a bisphosphonate and that the treatment should be discontinued when they are stabilised.

4.4.4 NSAIDs And Cox-2 Inhibitors At Randomisation

Note: recruitment completed to both celecoxib-containing arms in Apr-2011 at the end of Activity Stage II

For patients who are currently on a Cox-2-inhibitor and who meet the inclusion criteria, please ensure that treatment is discontinued before randomisation. If the patient is allocated to an arm, which does not include celecoxib (arms A, B, C or E), it is advised that the Cox-2 be replaced with a suitable NSAID.

For patients who are taking an NSAID prior to randomisation and are allocated a celecoxib arm (Arm D or F), a clinical decision should be taken as to whether the patient should continue taking the NSAID alongside the celecoxib. This decision should take into account the risk of gastrointestinal problems, and consideration should be given to the co-administration of a proton pump inhibitor

4.4.5 Starting Trial Treatment

Trial treatment should be commenced as soon as possible after randomisation. Investigators should aim that this is at least within 4 weeks post randomisation and within 12 weeks of starting androgen Deprivation Therapy (see **Section 6**).

Radiotherapy for patients allocated to Arm H should be commenced within 4 weeks from randomisations and continued according to the predefined scheduled unless toxicity is reported. Any delays in starting research radiotherapy should be discussed with the STAMPEDE team and recorded as appropriate in the relevant CRF.

4.4.6 Concomitant Medications

All concomitant medications should be recorded including any vitamin and mineral supplements the patient is taking, regular consumption of NSAID and/or aspirin and use of other bisphosphonates (see **Section 4.3.1**). Of particular interest in this regards, are herbal preparations such as PC-SPES,

Prostasol, Saw Palmetto and St John's Wort. All concomitant medications should be continued throughout the trial unless the responsible clinician decides otherwise.

4.5 ADDITIONAL DETAILS FOR PATIENTS JOINING SUB-STUDIES

An additional droplet of blood must be taken if the patient has given their consent to participate in the DNA analysis sub-study.

The local pathologist will also be asked to give the remaining tumour sample for tissue micro array analysis to be carried out, if the patient has given consent for his remaining samples to be used for further analyses. Full details of all sub-studies and instructions relating to the handling of the blood sample are given in **Section 17** and **Appendix D**.

5 RANDOMISATION AND ENROLMENT

Patients will be allocated to any of the open research arms for which they are suitable. Patients with non-metastatic disease or who have had previous local therapy to the prostate or who have a contraindication to radiotherapy (see **Section 4.3.1**) will not be allocated to Arm H.

To enter a patient the randomisation form should be completed carefully and the MRC CTU contacted by phone:

RANDOMISATIONS
To randomise, call MRC CTU, Monday to Friday 0900-1700
excluding public holidays or dates when notice has been given by the CTU.
Tel: +44 (0) 20 7670 4777

A trial number and treatment will be allocated and given over the phone or by return fax. In addition, a letter confirming these details will be sent. The trial number will be the primary way in which the patient will be identified and should be used in all correspondence.

5.1 CO-ENROLMENT GUIDELINES

Ideally, patients should not be participating in any other clinical trial of prostate cancer treatment when they enter STAMPEDE and should not enter any other trials until the patient has had a failure-free survival (FFS) event reported. After this point, the patient may be entered into further, second-line treatment studies. The primary outcome measure of STAMPEDE is overall survival. Participation in post-progression studies should be reported on the Co-enrolment CRF.

6 TREATMENT OF PATIENTS

6.1 TRIAL TREATMENT

Patients will be randomised to the control arm (Arm A) or one of the research arms. All patients will receive androgen deprivation therapy (ADT) to achieve castration levels of testosterone. The method of ADT is a local choice but must be specified for each patient prior to randomisation. The recommended methods of ADT are given in **Section 6.1.1**. All trial treatments should commence as soon as practically possible after randomisation. Patients having a bilateral orchidectomy should commence any additional treatment with 4 weeks of the operation unless there is a strong clinical reason not to do so. Note that from protocol version 8.0 onwards, bicalutamide monotherapy is no longer a permitted as a trial therapy for new patients (but patients may switch to a permitted therapy to join the trial – see **Section 4.3.2**).

6.1.1 Arm A: ADT Alone Or ADT + Standard-of-Care (M0) RT (Control Arm)

The standard of care for this patient group is **androgen deprivation therapy** (see **Section 6.1.1.1**). For some patient groups, this should now be supplemented with standard radiotherapy (see **Section 6.1.1.2**).

6.1.1.A Hormone Therapy

The recommended methods of ADT are bilateral orchidectomy, LHRH analogues and LHRH antagonists. Anti-androgens alone are not permissible as hormone therapy for patients participating in STAMPEDE, but their use is recommended in the short-term to prevent tumour “flare” which may occur after commencing LHRH analogues. Anti-androgen prophylaxis of tumour flare is not required when using LHRH antagonists. At the time of randomisation, centres will be asked to specify the method of ADT for each patient. Other methods of ADT should be discussed with the Chief Investigator or the Trial Surgeon. The planned duration of ADT should be at least 2 years.

Bilateral orchidectomy: Operations should be performed by appropriately trained surgeons. A total or subcapsular orchidectomy may be performed.

LHRH agonists: LHRH agonists/analogues used according to local practice. The prophylactic use of anti-androgens to prevent tumour “flare” is recommended.

LHRH antagonists: LHRH antagonists used according to local practice. The use of prophylactic use of anti-androgens to prevent tumour “flare” is not necessary.

6.1.1.B Standard-of-care (M0) RT

N0M0 patients: Investigators should give standard radiotherapy (RT) to patients with node negative, non-metastatic disease (N0M0), in accordance with the recent data from the PR07 and SPCG trials. If there is an intention to omit radiotherapy (e.g contraindications) in patients with N0M0 disease this must be discussed with the Trials Office before consent. See **Section 6.6** for further details of radiotherapy administration.

N+M0 patients: the benefit of radiotherapy in this group is at present uncertain with no firm data to either support or refute its use. However, the PR07 trial included some node positive patients as cross sectional imaging was not a part of the baseline assessment in this trial, which did include whole pelvis radiotherapy. For patients with node positive, non-metastatic disease, radiotherapy is

therefore recommended in suitable cases. Investigators will be asked to state their intention with regard to planned radiotherapy in this group at randomisation. Intention to give radiotherapy (or not) for node positive patients must be stated at randomisation to ensure that there is no bias towards particular combinations of systemic therapy with radiotherapy.

Standard radiotherapy is not a core part of the trial, therefore we intend to collect minimal data about the radiotherapy administered. It is accepted that some patients will develop progressive disease before radiotherapy can be administered and if this occurs the reasons for non-delivery of treatment must be recorded on the radiotherapy form.

6.1.2 Arm B: ADT + Zoledronic Acid

Androgen deprivation therapy (+/- standard-of-care M0 RT) as described in **Section 6.1.1**.

Zoledronic Acid: 4mg 15min IV infusion every 3 weeks, for 6 treatments followed by zoledronic acid 4mg 15min IV infusion every 4 weeks up to a maximum of 2 years from the start of the treatment or until disease (including PSA) progression (see **Section 7.2**). Patients should also receive an oral supplement of 500mg calcium and 400IU vitamin D daily. These doses are available as a combination tablet. See **Section 6.2.1** for further information.

6.1.3 Arm C: ADT + Docetaxel

Androgen deprivation therapy (+/-standard-of-care M0 RT) as described in **Section 6.1.1**.

Docetaxel: 75mg/m2 Day 1 as 1hr IV infusion, plus prednisolone 5mg bid daily for 21 days. The cycle should be repeated every 3 weeks for a maximum of 6 cycles. The recommended administration schedule, anti-emetic regimen and dose modifications for docetaxel are given in **Appendix F**. See **Section 6.2.2** for further information.

6.1.4 Arm D: ADT + Celecoxib

Note: recruitment completed to both celecoxib-containing arms in Apr-2011 at the end of its Activity Stage II

Androgen deprivation therapy as described in Section 6.1.1.

Celecoxib 400mg bid until the sooner of 1 year or disease (including PSA) progression (see Section 7.2). See Section 6.2.3 for further information.

6.1.5 Arm E: ADT + Docetaxel + Zoledronic Acid

Androgen deprivation therapy (+/- standard-of-care M0 RT) as described in **Section 6.1.1**.

Docetaxel: 75mg/m2 Day 1 as 1hr IV infusion, plus prednisolone 5mg bid daily for 21 days. The cycle should be repeated every 3 weeks for a maximum of 6 cycles. The recommended administration schedule, anti-emetic regimen and dose modifications for docetaxel are given in **Appendix F**. See **Section 6.2.2** for further information.

Zoledronic Acid: 4mg 15min IV infusion every 3 weeks, for 6 treatments followed by zoledronic acid 4mg 15min IV infusion every 4 weeks up to a maximum of 2 years from the start of the treatment or until disease (including PSA) progression (see **Section 7.2**). Patients should also receive an oral

supplement of 500mg calcium and 400IU vitamin D daily. These doses are available as a combination tablet. See Section 6.2.1 for further information.

Co-administration of docetaxel and zoledronic acid: Docetaxel 75mg/m^2 Day 1 as 1hr IV infusion, plus prednisolone 5mg bid daily followed by zoledronic acid 4mg 15min IV infusion. There is evidence to suggest that the co-administration of docetaxel and zoledronic acid is sequence dependent (39). Consequently, docetaxel should be administered before zoledronic acid

6.1.6 Arm F: ADT + Zoledronic Acid + Celecoxib

Note: recruitment completed to both celecoxib-containing arms in Apr-2011 at the end of Activity Stage II

Androgen deprivation therapy as described in Section 6.1.1.

Zoledronic Acid 4mg 15min IV infusion every 3 weeks, for 6 treatments followed by zoledronic acid 4mg 15min IV infusion every 4 weeks up to a maximum of 2 years from the start of the treatment or until disease (including PSA) progression (see Section 7.2). Patients should also receive an oral supplement of 500mg calcium and 400IU vitamin D daily (Calcichew). These doses are available as a combination tablet. See Section 6.2.1 for further information.

Celecoxib 400mg bid until the sooner of 1 year or disease (including PSA) progression (see Section 7.2). See Section 6.2.3 for further information.

6.1.7 Arm G: ADT + Abiraterone

Androgen deprivation therapy (+/- standard-of-care M0 RT) as described in Section 6.1.1.

Abiraterone will be administered as a single 1000mg daily oral dose (4 tablets to be taken together once a day) together with prednisolone or prednisone 5mg daily to prevent secondary ACTH excess.

In patients with M1 disease, treatment with abiraterone will continue from randomisation until clinical disease progression, consistent with the COU-AA-301 trial (36) i.e., abiraterone would be given for these patients until a composite of PSA progression (as defined in Appendix J), radiological progression (appearance of new lesions or progression of existing lesions) and clinical progression (defined as new cancer-related symptoms). It is accepted that these flexible criteria for stopping treatment with abiraterone are open to the investigator's interpretation and discretion. Patients might continue treatment beyond the first failure-free survival (FFS) event (see Table 1 in Section 9.2); the first FFS event must be reported as per the other arms.

In patients with N0M0 disease or N+M0 disease undergoing radical radiotherapy, treatment would continue for 2 years or disease progression as defined for M1 patients, whichever is the sooner. ADT can be discontinued in this group at 2 years at the discretion of the local investigator (see Section 6.1.1.A).

For patients with N+M0 disease not planned for radical radiotherapy, treatment will continue as for patients with M1 disease until disease progression.

See Section 6.2.4 for further information for all groups.

6.1.8 ARM H: ADT + PROSTATE RADIOTHERAPY IN M1 PATIENTS

Androgen deprivation therapy as described in Section 6.1.1.

Radiotherapy will commence as soon as practicable and within eight weeks after randomization. Treatment will be according to the guidelines in Section 6.2.5. Two radiotherapy dose-fractionation schedules are permitted:

- 36Gy in 6 fractions of 6Gy, administered weekly over 6 consecutive weeks
- 55Gy in 20 fractions of 2.75Gy, administered daily, five days per week, over 4 consecutive weeks

Details of the recommendations for outlining, CTV and PTV are in Section 6.2.5.

6.2 ADMINISTRATION AND DOSE MODIFICATIONS

6.2.1 ZOLEDRONIC ACID

Zoledronic acid will be administered by IV infusion in accordance with the instructions in the summary of product characteristics at a target dose of 4mg (adjusted for renal function, see below) every 3 weeks for the first 6 cycles and every 4 weeks, thereafter.

Serum Creatinine Measurements: Serum creatinine should be measured at baseline and within 48 hours prior to every administration of zoledronic acid. It is permissible to have serum creatinine levels measured on Fridays prior to the administration of zoledronic acid on the following Monday.

Serum Electrolytes and FBC: Serum electrolytes including calcium, phosphate and magnesium should also be measured prior to each infusion. FBC should be measured at least 3 monthly. Zoledronic acid should be discontinued if there is any evidence of hypersensitivity to the drug. In patients with mild to moderate renal impairment, lower doses of zoledronic acid are recommended according to standard dose reduction schedules for administration of this drug. In rare cases, zoledronic acid treatment has been associated with the development of osteonecrosis of the jaw, particularly following dental extractions. If a patient develops osteonecrosis of the jaw then the zoledronic acid should be immediately and permanently discontinued. For full details of zoledronic acid administration and does reductions see Appendix F. Contraindications, special precautions, interactions and side effects are listed in Appendix G.

6.2.2 DOCETAXEL

The use of docetaxel should be confined to units specialised in the administration of cytotoxic chemotherapy and it should only be administered under the supervision of a physician qualified in the use of anticancer chemotherapy.

Docetaxel will be administered by IV infusion in accordance with the instructions in the summary of product characteristics at a dose of 75mg/m^2 (up to a maximum dose of 160mg) on Day 1 of the study treatment period and then every 3 weeks thereafter for a maximum of 6 doses. Patients with a body surface area (BSA) greater than 2.13m^2 should be dosed as though they have a BSA of 2.13m^2. No ideal weight should be used for BSA calculations. Prednisolone or prednisone 5mg bid will be given until completion of chemotherapy. Additional dexamethasone should be given pre- and post-docetaxel infusion to suppress allergic reactions.

Please note that liver function test (LFTs) should be carried out within a week before the first cycle of docetaxel if an anti-androgen has been administered. This is due to an increased risk of

neutropenia associated with docetaxel use following anti-androgen administration. Treatment should be delayed if LFTs are abnormal.

For full details of premedication schedule, recommended anti-emetic regimen and dose modifications for docetaxel see **Appendix F**. Contraindications, special precautions, interactions and side effects are listed in **Appendix G**.

Docetaxel in combination with prednisone or prednisolone is indicated for the treatment of patients with hormone refractory metastatic prostate cancer. (21, 22)

6.2.3 CELECOXIB

Note: recruitment completed to both celecoxib-containing arms in Apr-2011 at the end of Activity Stage II. No new patients should be receiving this agent now within the trial.

Celecoxib should be administered in accordance with the instructions in the summary of product characteristics at a dose of 400mg bid orally. Rarely this drug is poorly tolerated and in this instance should be discontinued; particular care should be taken with patients with a history of gastrointestinal disease and patients with significant risk factors for cardiovascular events (see Appendix G). Patients with confirmed severe cardiovascular history should not be in STAMPEDE (see exclusion criteria, Section 4.2). Contraindications, special precautions, interactions and side effects are listed in Appendix G. Dose reductions are not anticipated.

6.2.4 ABIRATERONE

Abiraterone absorption is increased by food. The tablets should be taken at least 2 hours after food, swallowed whole with some water. No food should be eaten for 1 hour afterwards. Prednisolone (prednisone in Switzerland) should be taken as a single dose with food in the morning.

If clinical symptoms or signs suggestive of hepatotoxicity develop, serum transaminases, in particular serum alanine aminotransferase (ALT), should be measured immediately. If a rise in transaminases or bilirubin is confirmed, action should be taken as detailed in **Appendix G**.

6.2.4.A Steroid Dose Modifications

Prednisolone or prednisone will be started at 5mg once daily, to prevent secondary mineralocorticoid excess. Prednisone/prednisolone dose increase of up to 10mg/day is permitted to manage mineralocorticoid-related toxicities (e.g., hypokalaemia, hypertension) which are refractory to standard management. Patients experiencing serious Cushing symptoms (e.g., weight gain, muscle loss) can decrease or discontinue (temporarily or permanently) steroids at the investigator's discretion. It should be noted that weight gain and muscle loss are also associated with androgen deprivation therapy.

6.2.5 RESEARCH (M1) PROSTATE RADIOTHERAPY

A treatment planning CT scan will be acquired with the patient supine, with empty rectum and comfortably full bladder.

Megavoltage equipment is required with effective photon energies ≥6MV. Minimum source-to-axis distance is 100cm. Field arrangement is at the clinician's discretion: acceptable treatment techniques (field arrangement) include a 3-field (anterior, right lateral, and left lateral), 4-field (anterior, posterior, right lateral, and left lateral), or 6-field (right and left anterior oblique, right and

left posterior oblique, and right and left lateral) or equivalent coplanar technique with multi-leaf collimation for all fields to adequately protect normal structures.

The Clinical Target Volume (CTV) will consist of the prostate gland alone as visualized on the treatment-planning CT scan. The base of the seminal vesicles may also be included if they are macroscopically involved. Inclusion of pelvic lymph nodes in the CTV is not permitted. The Planning Target Volume will have a 0.8 cm margin posteriorly and 1.0 cm margin in all other directions around the CTV to account for prostate gland motion and uncertainty in daily treatment setup.

Critical normal tissues should be delineated on the treatment-planning CT scan by the treating clinician:

- Rectum – inferior limit: level of ischial tuberosities; superior limit: sigmoid flexure
- Bladder – entirety

Two radiotherapy dose-fractionation schedules are permitted. In either case, radiotherapy is prescribed such that at least 95% of the PTV receives the prescribed dose:

- 36Gy in 6 fractions of 6Gy, administered weekly over 6 consecutive weeks
- 55Gy in 20 fractions of 2.75Gy, administered daily, five days per week, over 4 consecutive weeks

Dose-volume objectives for each dose-fractionation schedule are shown in **Tables 3** and **4** below. Values have been calculated using the formula BED = D[1+d/(alpha-beta ratio)] assuming an alpha-beta ratio of 3 for rectum and bladder. These are provided for guidance only.

Table 3: Rectal dose volume objectives

55Gy/20F	36Gy/6F	Max Vol (%)
52.5 Gy	33.3 Gy	50%
43.5 Gy	27.8 Gy	60%
26.1 Gy	16.7 Gy	80%

Table 4: Bladder dose-volume objectives

55Gy/20F	36Gy/6F	Max Vol (%)
52.2	33.3	50%
43.5	27.8	25%

Portal imaging to verify accuracy of treatment delivery may be done according to the participating centre's local guidelines. Image-guidance technology (e.g., gold seed intraprostatic fiducial markers, cone-beam CT scanning) will be permitted according to clinician preference but is not required. Further illustration on the research radiotherapy arm schedule is shown in **Figure 7**.

Figure 7: Diagram for deciding approach to research (M1) RT

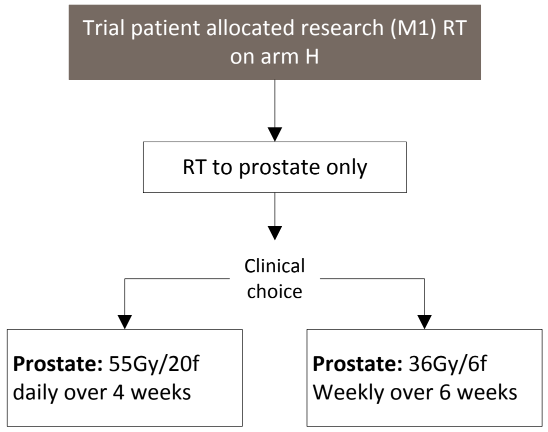

6.3 TRIAL PRODUCTS

Details of the procedures for obtaining the drugs within the trial, dispensing and disposal of unused drug are given in **Appendix E**.

Arrangements for free or discounted drugs are given in the Finance section (**Section 15**).

6.4 MEASURES OF COMPLIANCE/ADHERENCE

Date of treatment, dose, delays and reasons for delays or dose modifications of all study infusions (zoledronic acid and docetaxel) will be recorded. The estimated number of abiraterone tablets taken in a given time period will also be recorded as well as any dose reductions.

6.5 TREATMENT DATA COLLECTION

Data will be recorded on case report forms (CRFs); the top copy/original should be sent to the MRC CTU for data entry and a copy kept at the local centre. Up-to-date versions of all CRFs can be found on the trial website (http://www.stampedetrial.org/) and centres will be notified of any changes throughout the course of the trial The type of data to be recorded is detailed in the Assessments and Procedures section (**Section 7**).

6.6 ADMINISTRATION OF STANDARD RADIOTHERAPY[8] TO NON-METASTATIC PATIENTS

6.6.1 TREATMENT DETAILS

Standard radiotherapy will be given to appropriate patients in each of the trial arms, following a period of neo-adjuvant ADT therapy, as is generally standard in UK practice. For patients receiving

[8] **Note:** this text has been transferred into the protocol from the Appendices in version 8.0, and updated

docetaxel, this period needs to be a minimum of 6 months after randomisation to ensure that chemotherapy is completed and toxicity resolved before RT begins. To ensure consistency of timing of administration of standard radiotherapy in all arms, this same 6 months period is recommended for all patients. For patients with negative nodes on axial imaging, clinicians may choose between irradiating prostate and seminal vesicles alone or including the pelvic nodes in addition. Additional staging tests such as pelvic node sampling may be considered in making this decision. Conformal or intensity modulated radiotherapy should be used in all patients. Where patients have good clinical evidence that nodes are free of tumour or patients for whom nodal radiotherapy is contra-indicated (e.g. significant bowel disease), treatment may be given to the prostate gland and seminal vesicles only. The recommended dose is 74Gy in 37 fractions to the prostate and seminal vesicles or the equivalent using hypofractionated schedules. These recommendations are summarised **Figure 3**. Alternative dosing schedules are permitted but must be agreed with the STAMPEDE Trial Management Group.

6.6.1.A Standard-of-care RT Timing in M0 patients

Radiotherapy should be given around 6 to 9 months after randomisation in all trial arms and, if receiving docetaxel, the patient must have recovered from any docetaxel toxicity before RT can begin.

6.6.1.B Type Of standard-of-care RT in M0 patients

Conformal or intensity modulated radiotherapy.

6.6.1.C Standard Clinical Target Volume in M0 patients

- **CTV1:** Prostate plus seminal vesicles
- **CTV2:** Regional lymph nodes to include internal iliac and the inferior part of the common iliac nodes as used in EORTC trial 22961 (44)
- **PTV1:** CTV1 plus 10-15 mm according to local practice
- **PTV2:** CTV2 plus 10-20mm according to local practice

6.6.1.D Standard-of-care RT Dose in M0 patients

Prostate dose of 74Gy in 2Gy fractions or equivalent, with optional dose to the pelvic nodes of 46-50Gy in 2Gy fractions or equivalent using IMRT to deliver the treatment over 37 fractions, suggested dose is 55Gy in 37 fractions with IMRT. Higher doses may be considered if the department is experienced in using IMRT for nodal radiotherapy, particularly as data emerges from the PIVOTAL trial of nodal IMRT in high-risk node negative patients where a nodal dose of 60Gy in 37 fractions is being evaluated. Alternative schedules should be agreed with the STAMPEDE Trial Management Group.

Figure 8: Diagram for deciding recommended approach to standard-of-care (M0) RT in non-metastatic patients

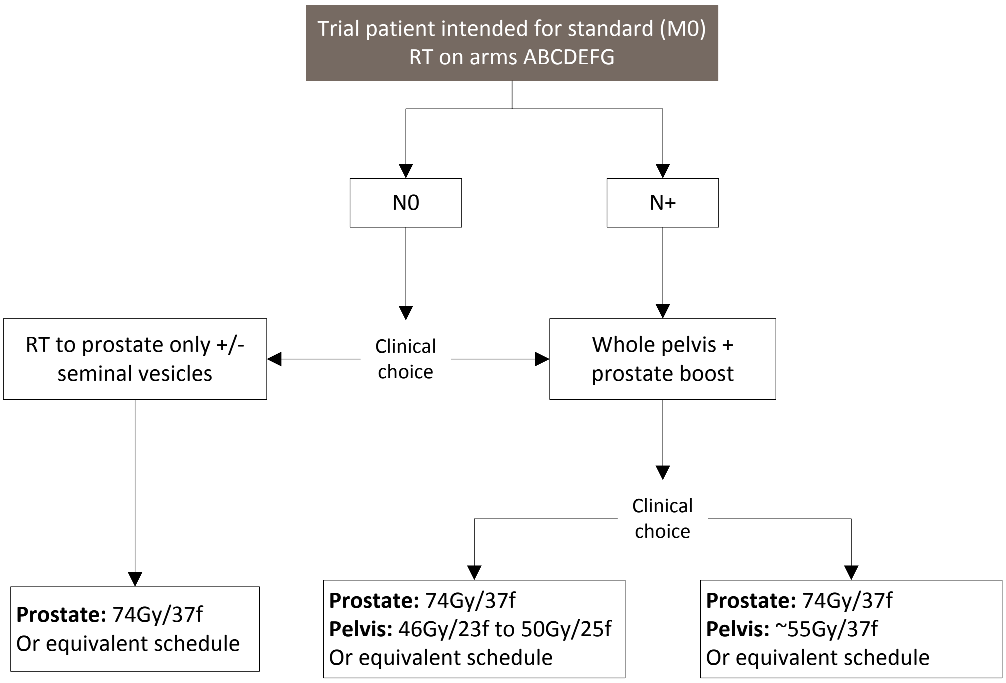

6.7 NON-TRIAL TREATMENT

6.7.1 MEDICATIONS PERMITTED

Any additional treatment that the responsible physician feels is appropriate is permitted.

6.7.2 DATA ON CONCOMITANT MEDICATION

All concomitant medication will be recorded on the baseline form prior to randomisation and on any subsequent Serious Adverse Event forms. This should include aspirin that may be taken on a regular basis for cardiovascular disease, the use of any Non-Steroidal Anti-inflammatory Drugs (NSAID) as well as any vitamin or mineral supplements the patient is taking.

7 ASSESSMENTS AND PROCEDURES

7.1 FLOW CHART/SCHEDULE FOR FOLLOW-UP

A detailed follow-up schedule is given in **Table 5**.

7.1.1 PSA MEASUREMENTS

All patients should have PSA measured pre-androgen deprivation therapy and at weeks 6, 12, 18 and 24 and every 12 weeks, thereafter, up to 2 years post randomisation. Following this, PSA should be measured every 6 months until 5 years and annually, thereafter. For patients who do not have a scheduled hospital visit, it would be acceptable for arrangements to be made for blood samples to be drawn in a GP's surgery.

7.1.2 ASSESSMENT OF TREATMENT FAILURE (DEFINITION OF PROGRESSION)

It is not proposed to routinely assess patients for response. However, in order that objective progression can be assessed, it is necessary to have imaging taken at time of best response as judged by the treating clinician. All patients should have baseline radiological examinations as detailed in **Section 4.3.1**. In addition it is recommended that all patients should have scans or X-rays repeated at 24 weeks (and whenever clinically appropriate) if they were abnormal at baseline, particularly if they have a low PSA value on entry in to the trial making biochemical assessment of treatment failure difficult. The following events would constitute a disease progression and should be reported on a progression form:

- Biochemical failure – must be reported alongside castrate levels of testosterone if the patient has received intermittent ADT (see **Appendix J**).
- Local progression
- Lymph node progression
- Progression in distant metastases
- Development of new metastases

Please note that skeletal-related events (SREs) may be indicative of disease progression but can have other causes such as osteoporotic fracture. All SREs should be investigated further to establish whether or not the patient has progressed, in which case a progression form should be completed.

7.1.3 ADDITIONAL SAFETY ASSESSMENT

Due to the risk of liver toxicity and secondary hyperaldosteronism with abiraterone, patients will require 2 weekly U+Es, LFTs and blood pressure measurement for the first 12 weeks. It is not proposed to collect the detail of these measurements unless results are abnormal; in this instance, they should be reported as AEs (on the next Follow-up CRFs) and as an SAEs (see **Section 11**) if appropriate.

Medical review and PSA measurements follow the pattern in the control arm: visits at weeks 6, 12, 18 and 24 and every 12 weeks, thereafter, up to 2 years post randomisation. Following this, PSA should be measured every 6 months until 5 years and annually, thereafter. For patients who do not have a scheduled hospital visit, it would be acceptable for arrangements to be made for blood samples to be drawn either in a GP's surgery or in the patient's home.

7.1.4 DATA COLLECTION AND NON-ADMINISTRATION OF STANDARD RADIOTHERAPY

There are CRFs to be completed for patients receiving primary radiotherapy whether this is standard radiotherapy for M0 patients on any arm or prostate radiotherapy for Arm H patients. All

radiotherapy and acute side effects details will be recorded on the Radiotherapy Form; any late side effects will be recorded on the Follow up form.

If it is decided not to give the planned radiotherapy (for example, due to early metastatic progression or patient refusal), this should be stated on the Standard Radiotherapy form together with the reason for non-administration of the treatment.

7.1.5 Data Collection Palliative Radiotherapy

For patients who receive palliative radiotherapy as part of first line treatment, a Palliative Radiotherapy CRF should be completed. Details of salvage RT for relapse and palliative treatment will be requested and completed only on the Progression Form.

7.1.6 Data Collection Research (M1) Radiotherapy

There are arm specific CRFs for patients randomised to arm H. Adverse events such as hip fractures, TURPs, skeletal-related events will be collected retrospectively via the Hospital Episode Statistics (HES) database.

7.1.7 Follow-Up Schedules

An individualised form with a follow-up schedule will be provided for each randomised patient. For patients who are receiving LHRH analogues, it is assumed that any additional treatment will commence within two weeks of randomisation. For patients who are due to have an orchidectomy it is recognised that surgery will have to be scheduled and the scheduling of any additional treatments may be affected by post-operative recovery. It is recommended that all patients who had abnormal radiological investigations at baseline or present with a low PSA on entry into the STAMPEDE trial should have them repeated 24 weeks after randomisation.

7.2 FOLLOW-UP

Every effort should be made to follow-up all patients who have been randomised. Patients should, if possible, remain under the care of an oncologist or urologist for the duration of the trial. If care of a patient is returned to the GP, it is the responsibility of the consultant who obtained the patient's consent to participate in the trial to ensure that the data collection forms are completed. If the patient moves from the local area, arrangements should be made for trial follow-up to be undertaken by their new local centre. Details of other participating centres can be obtained from the MRC CTU. The consent of patients should be obtained for their names to be flagged for survival information through national registries, for example NHS Information Centre/Office of National Statistics (ONS) in England/Wales and General Register Office in Scotland, Hospital Episode Statistics (HES). If the clinician moves, appropriate arrangements should be made to arrange for trial follow-up to continue at the centre.

Table 5: Summary of timing of case report forms

CASE REPORT FORMS	TIMING OF ASSESSMENT AND CRF
Bone Density Risk Factor	At randomisation
Randomisation	At randomisation
Baseline	At randomisation
Cardiovascular Assessment	At randomisation
Pathology	At randomisation. When pathology sample has been taken and sent to UCL laboratory.
Pre-18 Week Bisphosphonate	Treatment administered every 3. Form holds data for 2 cycles. Form to be sent after 2nd cycle given.
Post-18 Week Bisphosphonate Treatment	Treatment administered every 4. Form holds data for 3 cycles. Form to be sent after 3rd cycle given.
Docetaxel Treatment	Treatment administered every 3 weeks Form holds 2 cycles. Form to be sent after 2nd cycle given.
Standard-of-Care (M0) RT	When standard radiotherapy is completed or if planned RT is no longer to be given (all patients planned for RT in Arms A to G)
Research (M1) RT	When research radiotherapy (Arm H) is completed or at 3 months (Arm A)
Follow-Up	Every 6 weeks for 6 months, then every 12 weeks until 2 years. Every 6 months until 5 years and annually thereafter. (See **Table 6** for more information.)
Palliative Radiotherapy	If applicable, when the palliative radiotherapy course is completed.
End of Treatment	When each treatment is completed (either at end of scheduled treatment or at early cessation of treatment).
Progression & Additional Treatment	At the first occurrence of each type of progression and whenever a patient that has progressed receives additional treatment.
Serious Adverse Event	Following any Serious Adverse Event
Skeletal-related Event	Whenever a patient experiences a skeletal-related event
Death	At Death
Patient Transfer	When a patient is transferred to a different hospital for the administration of trial treatment and follow up

Note: Quality of Life Study is only for first 700 patients entered into the trial and those who were recruited after the implementation of version 8.0 of the protocol. MRC CTU will inform centres of which of their patients this applies to.

Table 6: Data required on follow-up forms

Timing of follow-up	PSA	Evidence of Progression	Androgen deprivation therapy	Abiraterone treatment	Unscheduled Visits	Toxicities
Before progression	✓	✓	✓	✓	✓	✓
After Progression	-	✓	✓	✓	✓	✓

7.3 TRIAL CLOSURE

For the purpose of complying with UK Clinical Regulations introduced on May 2004, the trial will be considered 'closed' when the follow-up point for the primary analysis of the final comparison has been reached. However, further observational follow-up of all patients enrolled in the trial will continue until all randomised patients have died. This will initially be via the hospital, but in the longer term may employ national registers.

Table 7: Schedule for completion of treatment and outcome forms by arm.

TIMING FROM RANDOMISATION			TREATMENT FORMS			OUTCOME FORMS	
YEARS	MONTHS	WEEKS	ZOL. ACID	DOCETAXEL	RT	FOLLOW-UP$^{\Psi}$	QL + HE$^{¥}$
6-Weekly							
-	-	6	B,E,F (†)	C,E (†)	-	All arms	All arms
-	-	12	B,E,F (†)	C,E (†)	M1: A,H	All arms	All arms
-	-	18	B,E,F (†)	C,E (†)	-	All arms	All arms
-	-	24	B,E,F (‡)	-	-	All arms	All arms
12-Weekly							
-	-	36	B,E,F (‡)	-	-	All arms	All arms
-	-	48	B,E,F (‡)	-	M0: A,B,C,E,G	All arms	All arms
-	-	60	B,E,F (‡)	-	-	All arms	All arms
-	-	72	B,E,F (‡)	-	-	All arms	All arms
-	-	84	B,E,F (‡)	-	-	All arms	All arms
-	-	86	B,E,F (‡)	-	-	All arms	All arms
6-Monthly							
2	24	104	B,E,F (‡)	-	-	All arms	All arms
	30	130	-	-	-	All arms	All arms
3	36	156	-	-	-	All arms	All arms
	42	182	-	-	-	All arms	All arms
4	48	208	-	-	-	All arms	All arms
	54	234	-	-	-	All arms	All arms
5	60	260	-	-	-	All arms	All arms
Annual							
6	-	-	-	-	-	All arms	All arms
7	-	-	-	-	-	All arms	All arms
Etc	-	-	-	-	-	All arms	All arms

Key:
A = ADT alone
B = ADT + zoledronic acid
C = ADT + docetaxel
D = ADT + celecoxib
E = ADT + zoledronic acid + docetaxel
F = ADT + zoledronic acid + celecoxib
G = ADT + abiraterone
H = ADT + M1 research RT to the prostate

Notes:
$^{\Psi}$ See **Table 6** for information required at follow-up
† Form records data for two cycles
‡ Form records data for three cycles
¥ 1st 700 patients and those recruited from protocol version 8.0 onwards only

Note: recruitment completed to both celecoxib-containing arms in Apr-2011 at the end of Activity Stage II

Radiotherapy, Late RT Toxicity, Palliative Radiotherapy Progression, SAE, End of Treatment, Co-enrolment and Death forms to be completed as required.

8 STOPPING OF TREATMENT OR FOLLOW UP

Patients should be given every encouragement to adhere to protocol treatment and follow-up schedule, in order to reduce biases. However, a patient has the right to withdraw consent for participation in any aspect of this trial at any time.

8.1 STOPPING RESEARCH INTERVENTIONS

A patient may stop trial treatment for the following reasons:

- Progression whilst on therapy (trial treatment must be discontinued in this instance). For patients randomised to Arm G, please refer to **Section 6.1.7** for criteria to stop treatment
- Unacceptable toxicity
- Intercurrent illness which prevents further treatment
- Withdrawal of consent for treatment
- Any alteration in the patient's condition which justifies the discontinuation of treatment in the clinician's opinion
- Intention to commence a new anti-cancer treatment due to evidence of relapse.

The reason should be recorded on the treatment and/or follow-up forms as well as the End of Treatment form. In the case of abiraterone, the disease event for stopping abiraterone may be after the first reportable failure-free survival event (see **Section 6.1.7**). Unless a patient states otherwise, it should be assumed that consent is given to continue to record trial data.

8.2 PATIENT TRANSFERS

For patients moving from the area, every effort should be made for the patient to be followed-up at another participating trial centre and for this trial centre to take over responsibility for the patient. To document the transfer process the main contact person at both the current and receiving hospitals should complete and sign the Patient Transfer Confirmation form. A fully completed form must be returned to the CTU prior to the patient transfer and ideally any data queries for the patient should be completed prior to transfer.

On receipt of the completed transfer form a member of the STAMPEDE team will confirm the database has been updated and request confirmation of the name of the patient's new Clinician. Photocopies of the following documents may then be sent to the new hospital to complete the transfer and copies must be also retained at the original site for monitoring purposes:

- Consent form
- Completed CRFs
- Any documentation relating to the patient's participation in STAMPEDE (patient names must be removed from any documentation).

8.3 WITHDRAWAL FROM THE TRIAL COMPLETELY

If a patient explicitly withdraws consent to have any data recorded their decision must be respected and the MRC CTU must be informed in writing. All communication surrounding the withdrawal

should be noted in the patient's records and no further STAMPEDE CRFs should be completed for that patient.

Early stopping of follow-up should not be undertaken lightly and the site must consider the implications for the trial and the patient in reaching such a decision.

Patients can change their minds about withdrawal at any time and re-consent to participate in the trial. Follow-up data should be collected only from the point of when consent was re-instated.

9 STATISTICAL CONSIDERATIONS

9.1 METHOD OF RANDOMISATION

Patients will be randomised centrally using a computerised algorithm developed and maintained by the MRC CTU. Randomisation will be performed using the method of minimisation over a number of clinically important stratification factors with an additional random element. To decrease determinability, the factors are not listed here but can be found in the Statistical Design Document. From the outset, the trial had 1 control arm (A) and 5 research arms (B, C, D, E and F); from version 8.0, an additional research arm (G) was introduced.

As the control arm is the comparator arm for all the research arms, it is intended to recruit twice as many patients to the control arm as to each of the original research arms as this is an efficient design. Therefore, the initial randomisation ratio will be 2A:1B:1C:1D:1E:1F. From version 7.0, accrual to the celecoxib-containing arms was halted and the allocation ratio was 2A:1B:C1:D0:E1:F0.

The allocation weighting for the additional arm G (version 8.0 onwards) will be 2, meaning that as many patients will be randomised to arm G as the control arm A: the randomisation ratio will be 2:2 (equivalent to 1:1 control:abiraterone). This gives an overall allocation ratio of A2:B1:C1:D0:E1:F0:G2. When recruitment has been completed to the ongoing original research arms B, C and E (which will be around 2 years before completion of accrual to G), the allocation ratio will be A2:B0:C0:E0:D0:F0:G2 (or 2:2). This is more efficient for this comparison than the 2:1 allocation ratio employed for the original research arms because of the minimal co-recruitment period.

Version 9.0 introduces a RT comparison for men with metastatic disease which is irrelevant to a subset of men joining STAMPEDE. This can only be achieved by splitting the randomisation system so that patients with newly diagnosed M1 disease and no contraindication will be randomised A2:B1:C1:D0:E1:F0:G2:H2 and other men will be randomised A2:B1:C1:D0:E1:F0:G2:H0. Note that the allocation ratio for each pairwise comparison in unaffected, only the rate at which comparisons accrue.

9.2 OUTCOME MEASURES

The overall, definitive primary outcome measure for the trial for each comparison is overall survival (all cause mortality). The design of the trial is such that it is important to have additional intermediate outcome measures to assess each research arm as the trial progresses. These are listed in **Table 8**. The intermediate primary outcome measure is failure-free survival. The reasons for different emphases in each recruitment stage are explained in **Section 9.3**.

Table 8: Trial Outcome Measures by Comparison Stage

TRIALS STAGE	PRIMARY OUTCOME MEASURES	SECONDARY OUTCOME MEASURES
Pilot phase	Safety*	Feasibility
Activity Stage (AS) I-III	Failure-free survival (FFS)†	Overall survival (OS)<br>Toxicity<br>Skeletal-related events
Efficacy Stage (ES) IV	Overall survival	Quality of life<br>Cost effectiveness<br>Failure-free survival†<br>Toxicity<br>Skeletal-related events

*Based on toxicity
†Including biochemical failure (see **Appendix J**)

9.3 SAMPLE SIZE: PRINCIPLES AND ASSUMPTIONS

The overall design for this study is a multi-arm multi-stage, multi-centre randomised controlled trial. There are fives stages for each research arm: a Pilot Phase, Activity Stages I-III and Efficacy Stage IV. Full details of the methodology underlying the trial design are given by Royston et al. (45, 46) The sample size calculations were performed using the `stage2` (version 1.2.0, March 2002) and `stagen` (version 1.1.1, 18 May 2004) programs, both implemented in Stata (Stata Corp, TX) and updated using the later `nstage` program (version 1.0.3, 13-jun-2007; version 2.1.0, 28-jun-2009). (47)

The trial is designed under the assumptions in **Table 9**, and additionally, we assume a slightly higher proportion of non-metastatic than metastatic patients such that the median FFS is two years and median OS four years.

Table 9: Hazard ratio assumptions under null and alternative hypotheses

SIZE OF HR	PILOT	AS I-III	ES IV
Under null hypothesis (H0)	n/a	HR(FFS) = 1.00	HR(OS) = 1.00
Under alternative hypothesis (H1)	n/a	HR(FFS) = 0.75	HR(OS) = 0.75

The HR of 0.75 for any research arm relative to control would translate into an absolute improvement in FFS of 10%, from approximately 50% to 60% at two years and OS of 10%, from approximately 50% to 60% at four years. A beneficial difference of this size would be clinically worthwhile and, indeed, experience tells us it may be unrealistic to expect a larger difference. Therefore, we have adequately powered the trial to detect a HR of 0.75 for overall survival. This design gives 95% power at Activity Stages I-III and 90% power at Efficacy Stage IV for each comparison. Further details of the sample size calculations are summarised in **Sections 9.4** and **9.5** and detailed in a separate Statistical Design Document which is available on request.

Note that, from version 8.0, standard-of-care M0 RT was introduced to the majority of patients with N0 M0 disease. This is likely improve the outcomes for this group. Further agents are starting to be

licensed for patients with castration-refractory disease which may also improve survival rates. Improved FFS rates would delay the intermediate analyses; improved survival rates would delay the definitive analyses. The Statistical Design Document includes models where median survival is estimated at 5 years rather than 4 years. The trial is powered to detect a difference in relative improvement and the analyses will be performed when a pre-planned number of events has been reported in the control arm, rather than after a certain number of patients have been recruited or a certain amount of time elapsed. **Sections 9.4** and **9.5** provide more detail.

9.4 SAMPLE SIZE ISSUES AND TRIAL STAGES: ORIGINAL RESEARCH ARMS (B-F)

9.4.1 PILOT PHASE: ORIGINAL RESEARCH ARMS (B-F)

It was anticipated that 210 patients will be recruited to the Pilot Phase from a limited number of centres over a one year period. Approximately 60 patients would be randomised to the control arm and 30 patients to each of the five research arms, each of which would be assessed for safety and feasibility. If recruitment proved unfeasible or any of the research arms proved unsafe or not feasible to administer (e.g., poorly tolerated or unexpected toxicity) recruitment to these arms would have been discontinued. There were already considerable safety data on the use of docetaxel and zoledronic acid in patients with malignancies including prostate cancer, and on the use of Cox-2 inhibitors (including celecoxib), although mainly from patients with musculoskeletal disorders. There were fewer data on the combination arms, but it was thought very unlikely that any of the research arms would be discontinued during the Pilot Phase. When 210 patients have been on the trial for a minimum of 18 weeks, the independent Data Monitoring Committee (IDMC) would review the data from the Pilot Phase. Recruitment continued to the trial during this period as equipoise remained. Recruitment continued beyond this point. Safety data continues to be assessed throughout the trial.

9.4.2 ACTIVITY STAGES I-III: ORIGINAL RESEARCH ARMS (B-F)

In the sample size calculations, we assume that all research arms successfully pass through the Pilot Phase to Efficacy Stage I and that patients will be recruited at a rate of approximately 500 per year. This is faster than in the Pilot Phase because the trial will recruit from additional centres, both in the UK and internationally. The analysis of Activity Stages I, II and III are planned for when around 113, 216 and 334 failure-free survival events have been observed in the control arm, respectively.

The Activity Stage analyses will comprise pairwise comparisons of FFS between the control arm and each of the 5 research arms (i=B, C, D, E, F). Let HR_i(true) represent the hazard ratio (HR) of the ith research arm to the control arm, and HRi(observed) the observed value. Discontinuation of accrual of further patients will be considered for the ith research regimen at each of Activity Stages I-III according to the guidelines in **Table 10**.

Table 10: Guidelines for stopping accrual to the ith original research arm

ACTIVITY STAGE	NUMBER OF CONTROL ARM EVENTS	CONSIDER DISCONTINUATION IF HR_i(OBSERVED) IS...
I	~113	>1.00
II	~216	>0.92
III	~334	>0.89

9.4.3 Efficacy Stage IV: Original Research Arms (B-F)

The analysis of Efficacy Stage IV for the original research arms will be performed when around 403 deaths have been observed in the control arm. This would give 90% power to detect the targeted hazard ratio of 0.75 at one-sided significance level of 0.025. The actual length of this stage, balancing continued accrual with just follow-up, depends on the number of arms passing through to further recruitment from Activity Stages I-III and the observed accrual and event rates.

9.4.4 Sample Size For Original Research Arms (B-F)

Assuming an accrual rate of 500 patients/year, between 2800 and 3600 patients are planned to be entered into the original research comparisons of the trial over a period of 5½ and 7 years. The exact number of patients to be entered depends on the observed accrual rate and the observed event rate, which is, in itself, dependent on the mix of patients joining the trial from the broad spectrum of eligibility. The primary analysis on overall survival requires around 403 deaths to be observed on the control arm. Accrual will continue until the main analysis can be foreseen so that the overall duration of the trial is as short as possible (longer accrual facilitates this) and so that few, if any, patients remain on treatment when the main results are released. The statistical team will monitor and project the timelines using the `artpep` command in Stata.

9.5 SAMPLE SIZE ISSUES AND TRIAL STAGES: ADDITIONAL RESEARCH ARM G

9.5.1 Pilot Phase: Additional Research Arm G

A similar approach will be followed for the additional research arm G as detailed for the original research arms in **Section 9.4.1**. The IDMC will review safety data, in the context of data from the control arm, when the first 30 patients allocated to arm G have been on trial for 18 weeks. Furthermore, an additional review of safety will be performed when 30 patients with newly diagnosed non-metastatic disease have been allocated to arm G and have been on trial for 18 weeks.

9.5.2 Activity Stages I-III: Additional Research Arm G

The same principles will be applied to the new comparison as to the previous comparisons. The notable difference will be in the accrual rate to this comparison which is anticipated to be higher. There are two reasons for this. First, STAMPEDE started to recruit slowly in only a limited number of pilot sites. As more sites have been activated, including internationally, accrual has increased. At the time of version 8.0 of the protocol, monthly accrual to the study was averaging around 60 patients/month (over 700 patients/year). Second, there is an equal allocation ratio for the abiraterone arm compared to the control arm. It is this different allocation ratio which means that the number of control arm events required to trigger the intermediate analyses is different for the assessment of abiraterone to the assessment of the original research arms. This is shown in **Table 11**.

Table 11: Guidelines for stopping accrual to the additional research arm G

Activity Stage	Number of control arm events	Consider discontinuation if HR_G(observed) is…
I	~75	>1.00
II	~142	>0.92
III	~221	>0.89

9.5.3 Efficacy Stage IV: Additional Research Arm G

The analysis of Efficacy Stage IV for the additional comparison will be performed when around 267 deaths have been observed in the control arm. This would give 90% power to detect the targeted hazard ratio of 0.75 at one-sided significance level of 0.025.

9.5.4 Sample Size For Additional Research Arm G

Consideration will be given to ceasing further randomisations to arm G if it is not showing sufficient evidence of activity, just as for research arms B to F. Up to around 1500 patients will join the abiraterone comparison with half allocated to the research arm. Providing the accrual rate remains above 50 patients/months, accrual will be halted when 1500 patients have been recruited or after 3 years, whichever is the sooner. The total number of patients joining this comparison depends on the same issues as for the original comparisons (notably, observed accrual and event rates) but also the length of time that the original research arms co-recruit alongside the additional research arm. It is assumed that this will be for approximately 1 year. The sample size calculations and projected durations are fairly robust to changes in the co-recruitment with the original research arms and future co-recruitment of any further research arms which the Trial Management Group may introduce. This is detailed in the Statistical Design Document.

9.6 SAMPLE SIZE ISSUES AND TRIAL STAGES: ADDITIONAL RESEARCH ARM H

9.6.1 Pilot Phase: Additional Research Arm H

A similar approach will be followed for the additional research arm H as detailed for the original research arms in **Section 9.4.1**. The IDMC will review safety data, in the context of data from the control arm, when the first 30 patients allocated to arm H have been on trial for around 18 weeks.

9.6.2 Activity Stages I-III: Additional Research Arm H

The same principles will be applied to the new comparison as to the previous comparisons and an equal allocation ratio of control arm patients to patients allocated arm H will be employed, as for Arm G. The number of control arm events required to trigger the intermediate analyses will be the same as for the abiraterone comparison (see **Table 13**).

9.6.3 Efficacy Stage IV: Additional Research Arm H

The analysis of Efficacy Stage IV for the additional comparison will be performed when around 267 deaths have been observed in the control arm. This would give 90% power to detect the targeted hazard ratio of 0.75 at one-sided significance level of 0.025.

9.6.4 Sample Size For Additional Research Arm H

Consideration will be given to ceasing further randomisations to arm H if it is not showing sufficient evidence of activity on the intermediate primary outcome measure (FFS), just as for the other research arms. This research comparison is relevant to around 60% of patients joining STAMPEDE. At the point of the scientific approval, accrual was averaging around 80 patients per month to the trial. If accrual to the trial was slower at 70 patients per month, then accrual to this comparison could be between 18 and 42 patients per month, depending on which other trial arms are open to recruitment at the time.

We are targeting a 25% relative improvement in overall survival following local radiotherapy to the prostate in this patient group. This is the same size of effect targeted with the other research arms in STAMPEDE. This relative improvement can be further justified in the light of MRC PR07 which

demonstrated an improvement of this magnitude for adding radiotherapy to ADT in locally advanced disease, with a hazard ratio for overall survival of 0.77 (95% CI 0.61 to 0.98). In that trial, fewer than half of the deaths were from prostate cancer, whereas in newly diagnosed metastatic patients nearly all men will die of their disease. Therefore, it is relevant to note the relative benefit of radiotherapy in PR07 in terms of prostate cancer-specific survival, where the hazard ratio was 0.54 (95% CI 0.27 to 0.78). Long-term survival-based data, with a median follow-up of ~10 years, were presented orally at the American Society of Clinical Oncology 2012 which confirmed these findings.(7)

We anticipate that around 1250 patients are required over 4 years to observe 267 control arm deaths after 5.25 years. In addition to the factors listed in **Section 2.1.2**, this assumes that (i) recruitment is constantly 70 pts/m to the trial overall, (ii) the original research arms stop accrual within 6 months after activation of the RT arm, (iii) the abiraterone arm stops accrual around 24 months after activation of the RT arm, and (iv) a further new research arm with an equal allocation ratio is introduced 18 months after activation of the RT arm.

With variations on these factors, between 1000 and 1400 patients are required over 2.75 to 4.50 years to address survival within 4.50 to 6.50 years. These sample scenarios will be documented in the Trial Master File.

All patients joining the trial will be starting long-term ADT for the first time. The focus of this comparison will be on the newly diagnosed, metastatic patients (and no contraindications to RT), which is the largest subgroup of patients in the trial and the group of patients at highest risk. Patients with non-metastatic disease will be excluded from this particular comparison as there are already randomised data demostrating the survival benefit from radiotherapy in patients with locally advanced disease. Radiotherapy is now mandatory in node negative patients; it is also recommended in the node-positive, non-metastatic (N+ M0) group.

For the control arm of the whole trial, we assumed that median failure-free survival is 18, 24 or 30 months and constructed sample size scenarios around each of these options; the event rate would depend on the patient mix. We now know that 60% of patients have M1 disease at trial entry and we have reported that FFS at 24 months is 51% across the whole of the control arm.(1)

For the sample size calculation for this new planned comparison, we have based our estimates on the subgroup of patients with newly diagnosed M1 disease in the control arm. Therefore, we estimate median FFS to be 1 year and estimate that median overall survival will be 3.5 years.

Figure 9: Progress Of STAMPEDE Through The Trial Stages

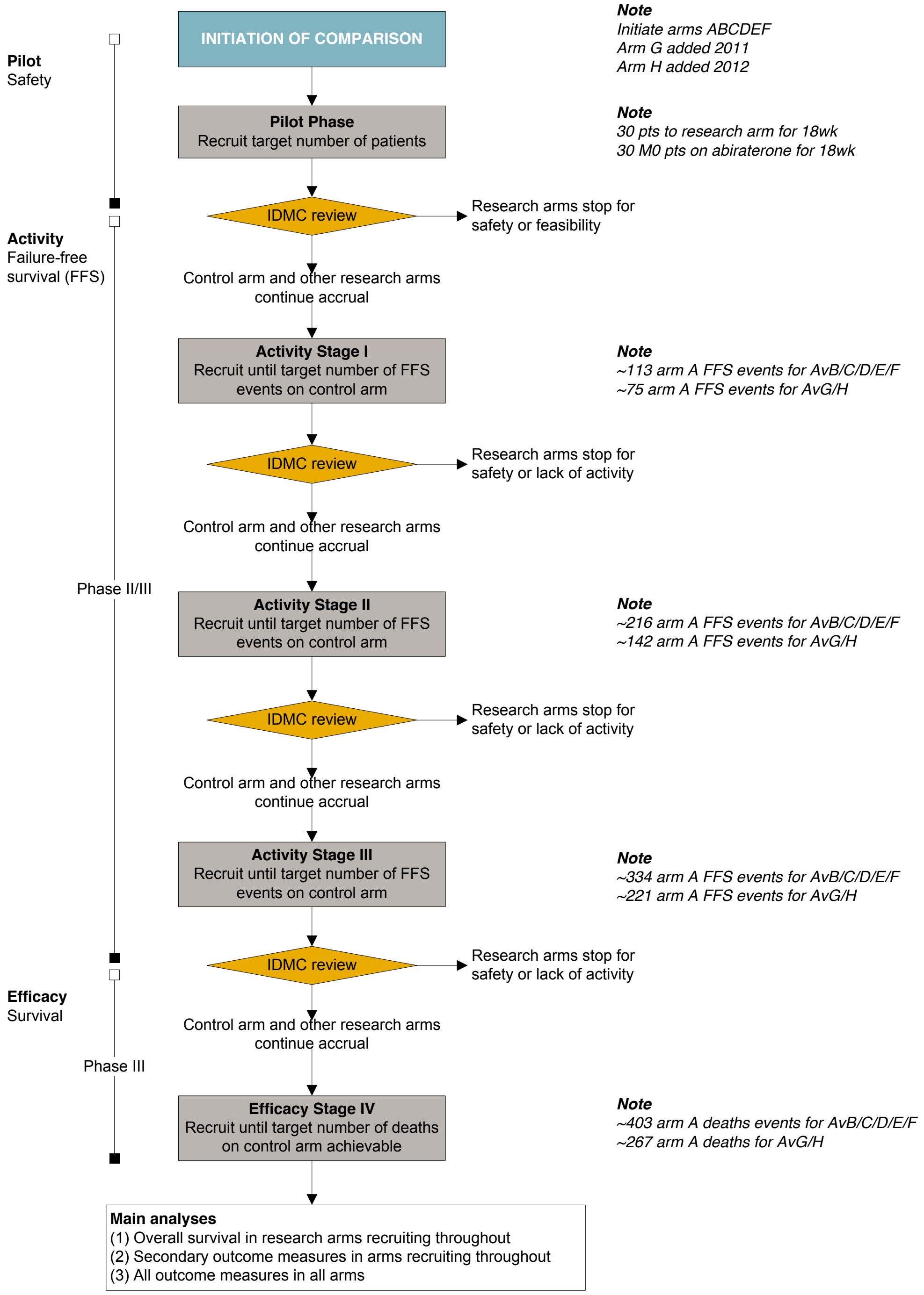

Key

FFS: Failure-free survival
HR: Hazard ratio
IDMC: Independent Data Monitoring Committee
Pts: Patients

Notes

Exact accrual depends on many factors including accrual rate, event rate and arms recruiting in each stage

9.7 FURTHER NOTES ON TRIAL DESIGN

9.7.1 Overall Sample Size

Given the adaptive nature of the study, there is no formal overall sample size target, but the numbers of patients required for each comparison are detailed in **Sections 9.4** and **9.5**. It is expected that more then 5000 patients will likely be recruited overall.

9.7.2 Factorial Design

We note here that we have not employed a factorial design in this trial because we anticipate the possibility of synergy between ADT, zoledronic acid and docetaxel and between ADT, zoledronic acid and celecoxib. It would not be possible to assess any such interactions reliably in a factorial trial (see the Statistical Design Document for further details).

9.8 INTERIM MONITORING AND ANALYSES

The accumulating data will be reviewed at regular intervals (approximately annually) by an Independent Data Monitoring Committee (IDMC), including pre-specified formal intermediate analyses of activity data (see also **Section 16**). These analyses will be performed by the trial team at the MRC CTU. Only patients randomised contemporaneously will be included in the comparison of each research arm against control ie patients allocated to the control arm prior to version 8.0 will not contribute to the comparison of abiraterone (Arm A vs Arm G).

The IDMC will be asked to give advice on whether the accumulating data from the trial with the guidelines for discontinuation of accrual for Activity Stages I-III, together with results from any other relevant trials, justifies continuing recruitment of further patients or further follow-up. A decision to discontinue recruitment, in all patients or in selected subgroups will be made only if the result is likely to convince a broad range of clinicians including those entering patients into the trial and the general clinical community. The intermediate stopping guidelines apply to the intermediate primary outcome measure. To stop accrual early for benefit in any comparison would require convincing data in terms of the definitive primary outcome measure, overall survival. For example, this could be $p<0.001$ as proposed by Haybittle-Peto.(48, 49) The use of such a guideline for stopping for benefit has a minimal impact on the operating characteristics.

If a decision is made to continue without change, the IDMC will advise on the frequency of future reviews of the data on the basis of accrual and event rates. The IDMC will make recommendations to the Trial Steering Committee (TSC, see **Section 16**) as to whether the trial should continue in its present form. While the trial is ongoing the accumulating data will generally remain confidential, unless the TSC and IDMC agree that the data should be made public.

9.9 OUTLINE ANALYSIS PLAN

Analyses will be performed on an intention-to-treat basis. The standard unadjusted log-rank approach will be applied to analyses of FFS and OS. The impact of potential confounders including the stratification factors used at randomisation will be considered in a Cox proportional hazard model. Flexible parametric models will be used to calculate the absolute differences between the arms to show treatment differences over time and to estimate restricted mean “survival” times (RMST). The estimated difference in restricted means survival time will be used preferentially to compare treatment arms if the proportional hazards assumptions required for hazard ratios cannot be supported. The χ^2 test or Mann-Whitney test will be implemented for categorical data

comparisons, including toxicity, as appropriate. The primary outcome measures (see **Section 9.2**) will be considered for all arms of the trial at each phase, but the main emphasis will be placed on the comparison of the research arms that have continued to recruit throughout the trial.

9.9.1 Pilot / Safety Phases

The Pilot Phase randomised patients between all the trial arms so that the results from these patients can be included in the main trial. Feasibility is considered in terms of the acceptability of the trial randomisation and reported toxicities and adherence to trial medication. Centres participating in the Pilot Phase for the original research arms were required to keep an anonymised log of all patients assessed for trial eligibility (see protocol version 2.0) so that the number of patients who did not participate in the study and the number of eligible patients who choose to not participate in the study could be summarised (reasons for non-participation were collected where the patients was willing). The anonymised logs will not be needed for new research arms like Arm G introduced in version 8.0 or Arm H introduced in version 9.0.

For the patients who are randomised, we shall describe the incidence of expected and unexpected severe toxicities and adverse events/reactions (see **Section 11**) to decide whether to continue with research arms beyond the Pilot Phase. As indicated above, we do not anticipate that recruitment to the research arms will be discontinued after the Pilot Phase, as there is considerable experience with zoledronic acid and docetaxel when combined with ADT, while Cox-2 inhibitors generally have a good toxicity profile. Although there are limited data on the combinations, we do not expect severe toxicity.

9.9.2 Activity And Efficacy Stages

The approach to analysis of these stages is summarised within the sample size calculations (see Section 9.4.3). Each research arm will be compared in a pairwise fashion against the control arm.

Full details are available in the Statistical Analysis Plan.

10 MONITORING AND QUALITY ASSURANCE

10.1 MONITORING AT MRC CTU

Data provided to the MRC CTU will be checked for missing or unusual values (range checks) and consistency over time. If missing or questionable data are identified, staff at the MRC CTU will request that the data be clarified. The exact procedures for data clarification and the amendment of CRFs will be described in the trial specific SOPs and instructions will be sent to all STAMPEDE institutions as soon as they have been approved to participate in the trial. The MRC CTU will also send reminders for any overdue data.

10.2 DIRECT ACCESS TO DATA

Collaborating institutions should be aware that direct access to patient data by MRC CTU staff may be required for trial-related monitoring or audit. Patient consent for this will be obtained as part of the general trial consent process.

10.3 VISITS TO INVESTIGATOR SITES

A selection of institutions will be visited at least once during the course of the STAMPEDE trial. The MRC CTU will give the responsible investigator adequate notice of the monitoring visit to allow adequate time, space and staff for these visits. The standard operating procedures (SOP) for monitoring are available from the MRC CTU.

After the monitoring visit the monitor will complete a site visit report. This report will be circulated to the TMG for comment. Once the TMG have reviewed the report and agreed on any recommendations the monitor will finalise the report and send a copy to the Principal Investigator (PI) at the site. A copy will also be sent to the CI for the trial and another copy will be kept in the MRC CTU STAMPEDE trial master file.

10.4 CONFIDENTIALITY

All information collected during the course of the research will be kept strictly confidential. In addition, all procedures for handling, processing, storage and destruction of data are compliant with the Data Protection Act 1998. No individual patients will be identified when the results of the trial are published.

Patients will be asked for permission for information about their health status to be obtained from the Office of National Statistics (ONS) or via the NHS Strategic Tracing Service or similar by the Medical Research Council, if necessary. In addition, patients will be asked for permission to inform their GP of their involvement in the STAMPEDE trial.

11 SAFETY REPORTING

ICH GCP requires that both investigators and sponsors follow specific procedures when reporting adverse events/reactions in clinical trials. These procedures are described in this section of the protocol. Further information on the expected toxicities for the trial interventions (docetaxel, zoledronic acid, abiraterone and radiotherapy) can be found in **Appendix G**.

11.1 DEFINITIONS

The safety reporting definitions from ICH GCP apply in this trial protocol. These definitions are given in **Table 12**.

Table 12: Event Terms and Definitions

TERM	DEFINITION
Adverse Event (**AE**)	Any untoward medical occurrence in a patient or clinical trial subject to whom a medicinal product has been administered including occurrences which are not necessarily caused by or related to that product.
Adverse Reaction (**AR**)	Any untoward and unintended response to an investigational medicinal product related to any dose administered.
Unexpected Adverse Reaction (**UAR**)	An adverse reaction, the nature or severity of which is not consistent with the information about the medicinal product in question set out in the summary of product characteristics (or Investigator brochure) for that product.
Serious Adverse Event (**SAE**) or Serious Adverse Reaction (**SAR**) or Suspected Unexpected Serious Adverse Reaction (**SUSAR**)	Respectively any adverse event, adverse reaction or unexpected adverse reaction that:<br>• results in death<br>• is life-threatening*<br>• requires hospitalisation or prolongation of existing hospitalisation**<br>• results in persistent or significant disability or incapacity<br>• consists of a congenital anomaly or birth defect<br>• Other important medical condition***

Clarifications and Exceptions

*The term 'life-threatening' in the definition of 'serious' refers to an event in which the patient was at risk of death at the time of the event; it does not refer to an event which hypothetically might have caused death if it were more severe.

**Hospitalisation is defined as an inpatient admission, regardless of length of stay, even if the hospitalisation is a precautionary measure for continued observation. Hospitalisations for a pre-existing condition (including elective procedures that have not worsened) do not constitute an SAE.

***Medical judgement should be exercised in deciding whether an AE/AR is serious in other situations. Important AE/ARs that are not immediately life-threatening or do not result in death or hospitalisation but may jeopardise the subject or may require intervention to prevent one of the other outcomes listed in the definition above, should also be considered serious.

Pregnancy occurring in a STAMPEDE patient's partner during the patient's participation in the trial, must be reported to the MRC CTU within the same timelines as an SAE and classified as an 'other important medical condition' on the SAE form. The outcome of a pregnancy should be followed up carefully and any abnormal outcome to the mother or child should be reported.

11.1.1 Trial-Specific Exemptions

Disease progression or death as a result of disease progression are not considered to be SAEs and should be reported on the STAMPEDE Progression Form or Death Form.

The following situations that fulfill the definition of an SAE are excluded from expedited notification on an SAE form and should be reported only on the STAMPEDE follow-up form:

- Elective hospitalisation and surgery for treatment of locally advanced or metastatic prostate cancer or its complications
- Elective hospitalisation to simplify treatment or procedures
- Elective hospitalisation for pre-existing conditions that have not been exacerbated by trial treatment

11.2 INSTITUTION/INVESTIGATOR RESPONSIBILITIES

All non-serious AEs/ARs, whether expected or not, should be recorded in the toxicity (symptoms) section of the Follow-up CRF and sent to the MRC CTU within one month of the form being due. SAEs/SARs should be notified to the MRC CTU as described below.

The severity (i.e. intensity) of all AEs/ARs (serious and non-serious) in this trial should be should be graded using Common Terminology Criteria for Adverse Events (CTCAE) v3.0 (ctep.cancer.gov/reporting/index.html). A flowchart is given in **Appendix I** to help explain the notification procedures. Any questions concerning this process should be directed to the MRC CTU in the first instance.

11.2.1 Investigator Assessment

11.2.1.A Seriousness

When an AE/AR occurs the investigator responsible for the care of the patient must first assess whether the event is serious using the definitions given in **Table 12**. If the event is serious and not exempt from expedited reporting, then an SAE form must be completed and the MRC CTU notified.

11.2.1.B Causality

The Investigator must assess the causality of all serious events/reactions in relation to the trial therapy using the definitions in **Table 13**. There are 5 categories: unrelated, unlikely, possible, probable and definitely related. If the causality assessment is unrelated or unlikely to be related the event is classified as a SAE. If the causality is assessed as either possible, probable or definitely related then the event is classified as a SAR.

Table 13: Assigning type of SAE through causality

RELATIONSHIP	DESCRIPTION	EVENT TYPE
Unrelated	There is no evidence of any causal relationship	Unrelated SAE
Unlikely	There is little evidence to suggest there is a causal relationship (e.g. the event did not occur within a reasonable time after administration of the trial medication). There is another reasonable explanation for the event (e.g. the patient's clinical condition, other concomitant treatment).	Unrelated SAE
Possible	There is some evidence to suggest a causal relationship (e.g. because the event occurs within a reasonable time after administration of the trial medication). However, the influence of other factors may have contributed to the event (e.g. the patient's clinical condition, other concomitant treatments).	SAR
Probable	There is evidence to suggest a causal relationship and the influence of other factors is unlikely.	SAR
Definitely	There is clear evidence to suggest a causal relationship and other possible contributing factors can be ruled out.	SAR

11.2.1.C Expectedness

If the event is a SAR the Investigator must assess the expectedness of the event. Please see Appendix G (Table G.2) for a list of expected toxicities associated with the drugs being used in this trial. If a SAR is assessed as being unexpected it becomes a SUSAR.

11.2.1.D Notification

Investigators must notify the MRC CTU of all SAEs occurring from the time of randomisation until 30 days after the last protocol treatment administration. SAEs occurring in patients randomised to Arm A must be reported until 2 years and 2 months or progression (whichever is sooner). SARs and SUSARs must be notified to the MRC CTU indefinitely (i.e. no matter when they occur after randomisation).

11.2.2 NOTIFICATION PROCEDURE

The SAE form must be completed by the Investigator (consultant named on the signature list and delegation of responsibilities log who is responsible for the patient's care), with due care being paid to the grading, causality and expectedness of the event as outlined above. In the absence of the responsible investigator the form should be completed and signed by a member of the site trial team. The responsible investigator should subsequently check the SAE form, make changes as appropriate, sign and then re-fax to the MRC CTU as soon as possible. The initial report shall be followed by detailed, written reports as appropriate.

Send the SAE form by fax to the MRC CTU. Fax Number: + 44 (0) 20 7670 4818. The STAMPEDE trial team will confirm receipt of the SAE report to the main point of contact via email

Follow-up: Patients must be followed-up until clinical recovery is complete and laboratory results have returned to normal or baseline, or until the event has stabilised. Follow-up should continue after completion of protocol treatment if necessary. Follow-up information should be noted on a

further SAE form by ticking the box marked 'follow-up' and faxing to the MRC CTU as information becomes available. Extra, annotated information and/or copies of test results may be provided separately. The patient must be identified by trial number, date of birth and initials only. The patient's name should not be used on any correspondence.

11.3 MRC CTU RESPONSIBILITIES

Medically qualified staff at the MRC CTU and/or the Chief Investigator (or a medically qualified delegate) will review all SAE reports received. The causality assessment given by the local Investigator at the hospital cannot be overruled and in the case of disagreement, both opinions will be provided in any subsequent reports.

The MRC CTU is undertaking the duties of trial sponsor and is responsible for the reporting of SUSARs and other SARs to the regulatory authorities (MHRA and competent authorities of other European member states and any other countries in which the trial is taking place) and the research ethics committees as appropriate.

The MRC CTU will also keep all investigators informed of any safety issues that arise during the course of the trial.

SAE REPORTING
Fax to 020 7670 4818 within 24 hours of becoming aware of the event

12 ETHICAL CONSIDERATIONS AND APPROVAL

12.1 ETHICAL CONSIDERATIONS

This is a randomised trial therefore neither the patients nor their physicians will be able to choose the patients' treatment. Treatment will be allocated randomly using a computer-based algorithm. This is to ensure that the groups of patients receiving each of the different treatments are similar.

Androgen deprivation therapy alone is the standard treatment for these forms of prostate cancer. Patients will be randomised to one or two of the newer treatments in combination with hormone treatment. The trial employs an unequal allocation ratio for efficiency, these are explained in detail in the patient information sheet.

The newer combined treatment options are being assessed in a detailed and systematic fashion in this trial. There is some evidence to suggest that the newer treatment options may have advantages over standard treatment (androgen deprivation therapy) alone with regards clinical outcome, but this is not confirmed and toxicity may be increased. This trial will follow a large group of men who have been randomly allocated to either the standard treatment (androgen deprivation therapy alone) or the newer combined treatment options in order to measure the benefits of the new treatments. The patients will also be followed-up for toxicity and safety issues, so that any benefits can be weighed against any negative aspects.

Patients participating in the trial will have some additional hospital visits and some extra blood samples taken compared to patients who are not participating in the trial, with the amount varying according to the allocated treatment. Sometimes the blood samples can be taken when the patient is attending hospital for treatment, anyway. On some of the trial arms, the patient may have to make additional visits to the hospital for the blood sample to be taken, although in some cases it may be possible for the blood sample to be taken in the GP's surgery. The additional visits and blood samples are to ensure that follow-up of patients is comparable in all the treatment groups. The blood samples will also be used for genetic and serum marker studies, where this information will be considered with clinical data. Blood samples will be link-anonymised. There will be no feedback to individual patients.

If new information emerges during the course of the trial which may affect the treatment or follow-up of patients who have joined the trial, information will be provided through by the trial team to all Principal Investigators. PIs have therefore the duty to inform patients in their care of any new information emerged using any appropriate channel (e.g. letter, communication at follow up clinic, etc).

12.2 ETHICAL APPROVAL

The protocol has a Favourable Opinion from an appropriate Research Ethics Committee, according to national guidelines. Additionally, each site must also obtain management permission for research (R&D approval) from the relevant host organisations before patients can be entered into the trial. The patient's informed consent to participate in the trial should be obtained after a full explanation has been given of the treatment options, including the conventional and generally accepted methods of treatment. Patient information sheets and patient consent forms are given in **Appendix B**.

The right of the patient to refuse to participate in the trial without giving reasons must be respected. After the patient has entered the trial, the clinician must remain free to give alternative treatment to that specified in the protocol, at any stage, if he feels it to be in the best interest of the patient. However, the reason for doing so should be recorded and the patient will remain within the trial for the purpose of follow-up and data analysis according to the treatment option to which he has been allocated. Similarly, the patient must remain free to withdraw at any time from the protocol treatment without giving reasons and without prejudicing his further treatment.

A statement of MRC policy on ethical considerations in clinical trials of cancer therapy, including the question of informed consent, is available from the MRC Head Office web site (http://www.mrc.ac.uk).

13 REGULATORY APPROVAL

This trial has been approved in the UK by the MHRA and will be conducted under a CTA (Ref: 00316/0026/001-0001) in the UK.

The trial has been approved in Switzerland by Swissmedic (Ref: 2009 DR 3235).

14 INDEMNITY

The MRC adheres to the principles of the Research Governance Framework for Health and Social Care (Department of Health, England). The MRC is not insured but it has indemnity arrangements in place such that public funding is provided to meet claims.

The likely scenarios in which the MRC might face claims for damages are set out below.

As Sponsor, the MRC accepts that it might face claims for damages where the MRC, or any of its employees, or any person formally acting with MRC's authority, have a negligent or have failed to adhere to the relevant guidelines/guidance, legislation or procedure on good practice in relation to medical research and that negligence or failure has caused or materially contributed to the personal injury suffered by the individual making the claim.

The MRC may consider an ex- gratia payment when a significant adverse reaction in the form of a personal injury has occurred which is likely to have been caused by, or materially contributed to, by participation to the research study.

Where studies are carried out in a hospital, the hospital continues to have a duty of care to a patient being treated within the hospital, whether or not the patient is participating in an MRC-supported study. MRC does not accept liability for any breach in the hospital's duty of care, or any negligence on the part of employees of hospitals. This applies whether the hospital is a NHS Trust or not.

The Swiss Group for Clinical Cancer Research (SAKK) have provided trial-specific insurance to provide indemnity for Swiss sites against claims relating to non-negligent harm.

15 FINANCE

STAMPEDE is funded by the Clinical Trials Advisory Awards Committee (CTAAC) on behalf of Cancer Research UK; it is also funded by the MRC through the MRC Clinical Trials Unit. The trial has National Cancer Research Network (NCRN) approval and, therefore, local NCRN funds may be available at each centre to support entry of patients into this trial.

Zoledronic acid is manufactured by Novartis. Novartis have agreed to provide an educational grant to support the conduct of this study. Novartis have also agreed to supply the study drug, zoledronic acid free of charge for patients participating in the study.

Docetaxel is manufactured by Sanofi-Aventis Pharma. They have agreed to supply the study drug, docetaxel at a discounted rate for patients that are participating in the trial and to provide an educational grant to support the conduct of the study. The Department of Health has agreed to provide a central subvention as follow: £1,787 per patient randomised to arms C and E of the trial and prescribed docetaxel. This amount is payable in respect of a hospital trust randomising more than 3 patients. For more details contact the STAMPEDE Trial Manager.

Celecoxib is manufactured by Pfizer. They agreed to supply free drug and provide funds to distribute drug to participating sites.

Abiraterone is manufactured by Janssen Pharma PV (pharmaceutical companies of Johnson & Johnson). They have agreed to provide free drug and funds to distribute drug to participating sites and to help support the conduct and management of the trial.

16 TRIAL COMMITTEES

16.1 TRIAL MANAGEMENT GROUP (TMG)

A Trial Management Group (TMG) has been formed comprising the Chief Investigator, other co-investigators and members of the MRC CTU. The membership of the TMG may be expanded if other groups of trialists wish to participate. It will also be amended during the trial if other circumstances require e.g. retirement.

The TMG will be responsible for the day-to-day running and management of the trial and will meet by teleconference at least 3 monthly and in person as needed. The TMG members are detailed in **Appendix K**.

Further details of TMG functioning are provided in the TMG charter (available on request)

16.2 TRIAL STEERING COMMITTEE (TSC)

A Trial Steering Committee (TSC) has been formed to provide overall supervision for the trial and provide advice through its independent chair. The ultimate decision for the continuation of the trial lies with the TSC. The TSC will meet regularly.

Further details of TSC functioning are provided in the TSC charter (available on request)

16.3 INDEPENDENT DATA MONITORING COMMITTEE (IDMC)

An Independent Data Monitoring Committee (IDMC) has been formed. The IDMC will be the only group who sees the confidential, accumulating data to the trial. Reports to the IDMC will be produced by the MRC CTU. The IDMC will meet within 6 months of the trial opening with the frequency of meetings dictated by the IDMC. The IDMC will consider data in accordance with the analysis plan (see **Section 9.5**) and will be advisory to the TSC. The IDMC can recommend premature closure or reporting of the trial, or that recruitment to any research arm be discontinued.

From version 8.0 onwards, any recommendation from the IDMC to stop recruitment to one or more trial arms will be acted upon immediately, pending ratification from the TSC. As this period between meetings should be very short, sites would not be notified until after the TSC have made a decision. IDMC recommendations based on emerging safety issues would be discussed with sites promptly.

Further details of IDMC functioning and the procedures for interim analysis and monitoring are provided in the IDMC charter (available on request).

Figure 10: Diagram of relationships between trial committees

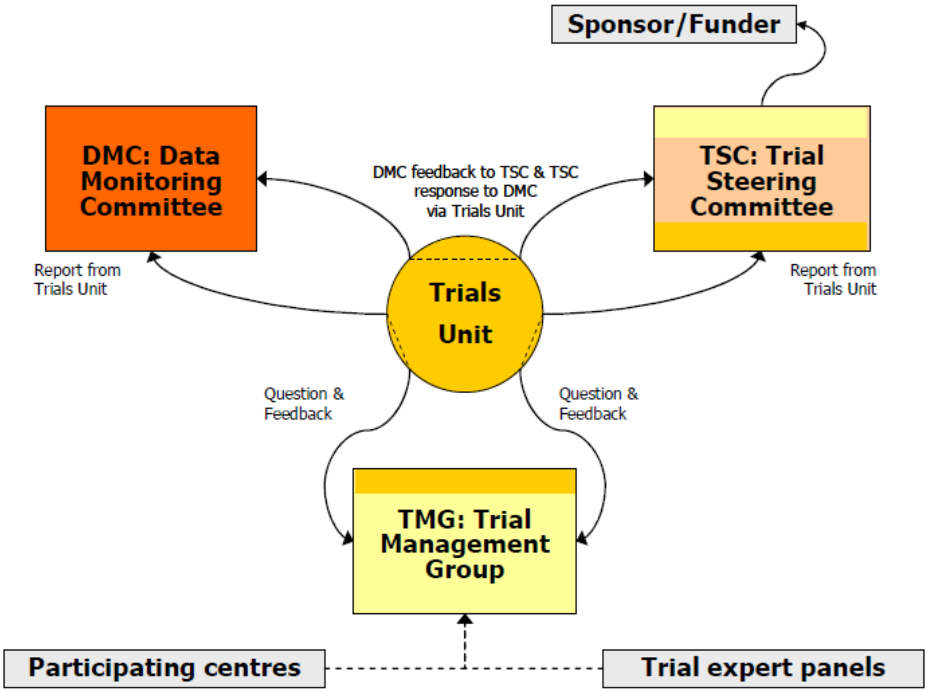

17 ANCILLARY STUDIES

17.1 QUALITY OF LIFE

A quality of life (QL) study is being performed to assess the impact of each treatment arm on the quality of patient's lives and participation in this study was limited to the first 700 patients recruited (this was reached in September 2008) patients. The QL study re-opened from the implementation of version 8.0 of the protocol. The EORTC QLQ-C30 with the prostate-specific module QLQ PR25 will be used. Key items for assessment are pain reduction for patients with metastatic disease and urinary symptoms for patients with locally advanced disease. In addition specific hypotheses will be generated for each of the research arms. The EuroQol (EQ-5D) (50) will be used in the study as a generic measure of health-related quality of life which can be linked to public preferences. These data will be used to calculate quality-adjusted life-years as part of the economic evaluation (see **Section 17.2**). Patients who were recruited into the QL study, should continue on the study throughout the trial. Questionnaires should be self-administered, although it is recommended that a key person (e.g. research nurse) at each centre be responsible for the data collection to optimise compliance and completeness of the data.

The QL and the HE questionnaires should be completed without conferring with friends or relatives and all questions should be answered even if the patient feels them to be irrelevant.

The responsible person should check each questionnaire for its completeness, ensuring that the correct date of completion and patient identifiers are present. The research nurse should approach patients at appropriate clinical visits to complete a questionnaire. If no clinical visit is scheduled for the patient (with a window of 4 weeks around the expected date) the nurse should organise the completion of the questionnaire, by post or by a visit to the patient at home (or in a hospice).

17.2 HEALTH ECONOMICS

A health economics (HE) sub-study will be performed. Core resource use information will be collected, using CRFs on days in hospital (by speciality) and outpatient visits. Data being collected on concomitant medication will also be used in the economic analysis. Information on patients' use of primary care and community-based services will be collected as additional questions in the QL questionnaire. Costs will be calculated on the basis of representative UK unit costs at the point of analysis. Health outcomes will be assessed in terms of quality-adjusted life years (QALYs). Quality adjustments will be based on patients' responses to the EQ-5D health status measure which will be administered at baseline and each point of follow-up as part of the QL questionnaire. A cost-effectiveness analysis will compare all regimens that continue to recruit into their Activity Stage IV.

17.3 TRANSLATIONAL SUB-STUDIES

17.3.1 DNA Analysis

Blood samples from as many patients as possible will be collected for future research. With patient consent, an additional droplet of blood sample will be collected and stored for DNA and protein analysis in order to try to identify molecular features of clinical significance.

Blood samples should be sent directly to the central laboratory on the FTA elute cards provided. Patient information sheets and consent forms which highlight this research are given in **Appendix B**, while details of specimen collection, posting and contact details are given in **Appendix D**.

17.3.2 Tissue Microarray

Patient consent will be sought to utilise paraffin embedded tissue for the construction of tissue microarrays from needle cores. One needle biopsy will be selected for microarray and the remaining tissue will be returned to the originating histopathologist. Given the entry criteria for the trial, the majority of patients will have extensive disease in the diagnostic needle core biopsies, in contrast to men with localised, low grade disease. Consequently, removal of one core is unlikely to compromise any subsequent histopathological assessment. Details regarding transfer of samples will be issued at the time of construction of the micro array.

18 PUBLICATION

The results from different centres will be analysed together and published as soon as possible. Individual clinicians must not publish data concerning their patients that are directly relevant to questions posed by the study until the TMG has published its report. The TMG together with the STAMPEDE collaborators will form the basis of the writing committee and decide on the nature of publications. Any release, of efficacy or safety data, presentation or publication will be agreed with the TSC according to the terms of their charter.

All publications will acknowledge the participating centres and clinicians, and these will be detailed in an appendix to the main report. Papers will have named authors determined by the TMG according to the following principles:

- To be as inclusive as possible where this is practicable
- To ensure that there is justification for anyone to be named as an author
- Reasons for nomination for authorship may include: trial design; grant holding; day-to-day trial oversight (TMG membership); analysis; discussion and interpretation of data; representation for key groups; active participation at large recruiting sites. It should be accepted that the people qualifying for authorship will vary over time. In addition, key positions will vary depending on the nature of the publication: clinical lead for clinical papers, statistician lead for methodology papers, translational papers may be lead by authors not on the main TMG if appropriate (e.g., the bone sub-study). In the event of any dispute related to authorship or data release, the TSC will be responsible for making the executive decision.

In the manuscript, a full list of sites and the number of patients recruited will be provided. In the presentations, this list of sites will also be shown. The term “the STAMPEDE investigators” will clearly be stated and relevant names included in the presentation credits.

19 PROTOCOL AMENDMENTS

19.1 PROTOCOL

19.1.1 AMENDMENTS MADE TO SECTIONS IN PROTOCOL VERSION 1.0 (MAY 2004)

Administrative changes such as typos, word change etc.

Name additions/changes to:

TMG members

TSC members

IDMC members

'General Information' Section – additional information re. Abridged version of protocol

Section 1.2 – Figure 1, Celecoxib duration amended

Section 1.3 – Figure 2, addition of cardiovascular assessment form, name and timings amended

Section 2.3 – Docetaxel information updated

Section 2.4 – Additional text re dose and duration justification for Celecoxib use.

Section 3 – Title change and content updated

Section 4.2 – New exclusion criteria added

Section 4.3.1 – New investigations added and additional text re testosterone measurements and additional text re. prior celecoxib treatment

Section 6.1.4 – Celecoxib duration amended

Section 6.1.5 – Additional text re. Co-administration of docetaxel and bisphosphonates

Section 6.1.6 – Celecoxib duration amended

Section 6.2.2 – additional docetaxel information

Section 6.2.3 – addition of CV event history

Section 11 – Safety reporting updated

Section 12.1 – Additional text re. the collection of blood for genetic and serum marker studies

Section 15 – Additional information re. Central Subvention for docetaxel arms

19.1.2 AMENDMENTS MADE TO SECTIONS IN PROTOCOL VERSION 1.1 (MAY 2005)

Section 6.2 Administration and Dose Modifications, subsection 6.2.1 Zoledronic Acid

19.1.3 AMENDMENTS MADE TO SECTIONS IN PROTOCOL VERSION 2.0 (JUN 2005)

General Information section – SAE reporting fax number and timeframe added.

Section 1.2 – Addition of anti-androgen use for M0 patients as a method of HT

Section 1.2 – Increase in amount of blood needed & addition tissue sample request.

Section 1.3 Trial Documentation updated to include new table detailing trial documentation ahead of accreditation, the inclusion of the radiotherapy forms and correct case report form timings

Section 2.1 – Addition of anti-androgen use for M0 patients as a method of HT

Section 4.1.3 – Inclusion criteria Vii "Normal testosterone prior to hormone treatment" removed.

Section 4.1.3 - φnote has been omitted and moved to section 4.2 (see number 8)

Section 4.2 – Exclusion criteria added to exclude patients with active peptic ulceration, gastrointestinal bleeding and inflammatory bowel disease.

Section 4.2 – Exclusion Criteria added to exclude patients with planned major dental work

Section 4.3.1 - All blood test timelines changed from 14 days to 28 days.

Section 4.3.1 – Hormone Therapy pre-randomisation deadline extended from 4 weeks to 12 weeks.

Section 4.3.1 – Additional information regarding the use of NSAIDs and cox-2-inhibitors before coming on to the STAMPEDE study and once commenced on study treatment

Section 4.3.2 – Updated to ask for all vitamins and minerals the patient is taking to be recorded.

Section 4.3.3 – Updated to include the extra blood required and the request for consent of patients' tissue samples.

Section 6.1.1 – Addition of anti-androgen use for M0 patients as a method of HT

Section 6.1.6 – Addition of the calcium & vitamin name "calcichew".

Section 6.6.2 – asking also to collect vitamins and minerals under concomitant medication.

Section 6.6.3 – New section to inform investigators that patient's, who they wish to give radiotherapy to, are also eligible for STAMPEDE

Section 6.6.4 – New section to detail what data is being collected on the radiotherapy given to patients.

Section 7.1; figure 4 – Addition of radiotherapy form and in note, addition of AA alone

Section 7.1.2 – omission of repeated scans and x-rays at 24 weeks, also omitted in note under figure 4.

Chapter 11 – Safety reporting section updated

Section 17.3 – Increase in amount of blood needed & additional tissue sample request.

19.1.4 Amendments Made To Section In Protocol Version 3.0 (Jul 2006)

Front Cover - NCRN logo added for accuracy

Front Cover - Clafication that protocol developed with NCRI rather than on behalf of

Front Cover - Clarification the it is a 6 arm trial

General Information section - MRC CTU staff section updated

Section 1.2 – Statistics section updated.

Section 1.2 - Additional research paragragh updated to refelct additional studies and for clarification of terms

Section 1.2 - Blood collection volume changed to reflect new technique used

Section 1.3 (figure 3) - Table showing case report form scheduyle updated to reflect clarification of follow-up schedule and addition of new CRF (End of Treatment)

Section 2.2 - AS changed to HT (clarification of terms)

Section 2.3 - Updated in information in reagrd to use of docetaxel added to reflect up to date practice

Section 2.5 - Sub-headings numbered for consistancy

Section 3.0 - Information in reagrd to the Pilot Phase now written in past tense as Pilot Phase has now been completed

Section 4.1.1 - Inclusion criteria extended so that patinets who fulfil 2 out of the three of the first inclusion criteria can be eligible.

Section 4.3.1 - Change in time scales by which baseline investigations need to be completed.

Section 4.3.1 - Clarification that chest X-ray is only required if chest is not included in the CT

Section 4.3.1 - Removal of 12 week timeline for basline PSA test ot be performed. (Stipulation that it must be performed before start of HT)

Section 4.3.2 – Information added in regard to time allowed from randomisation to start of treatment

Section 4.3.3 - Additional research paragragh updated to refelct additional studies and for clarification of terms

Section 4.3.3 - Blood collection volume changed to reflect new technique used

Sections 6.1.2-6.1.6 - Androgen Supression replaced with hormone therapy for consistemcy of terms

Section 6.2.2 - '(Taxotere)' Removed for consistancy

Section 6.2.2 _ information added in regard to the need to closely monitor liver function prior to docetaxel adminstration

Section 7.1 - Page number reference updated

Section 7.1.1 - PSA measurement timings updated to accurately reflect follow-up schedule

Section 7.3 (Table 4) - Table and key updated to accurately refelct follow-up schedule and to include information about new CRFs and removal of withdrawal CRF

Section 8 - Rewording for clarifcation of definition of trial withdrawal

Section 8.1 - Instruction that withdrawal from trial treatment should be recorded on End of Treatment Form rather than withdrawal form

Section 8.1 - Information updated to emphasise that trial treatment must be discontinued following a progression

Section 8.2- Information added in regard to patient transfers

Section 8.3 - Instruction that withdrawal from trial completely must be notified in writing to the MRC CTU rather than included on withdrawal form

Section 9 and Summary – Target event numbers updated to reflect the slightly revised numbers obtained by using –nstage- which is the new, recommended program for MAMS trials

Sections 11.1 and 11.2 - Form numbers removed to allow for future changes in numbering

Section 11.2 – Reference to toxicity grading website added

Section 11.2.1 - Reference to table in appendix G added

Section 12.2 - 'Suggested' removed from 'Suggested patient information sheets'

Section 13 - CTA reference added

Section 17.3 - Information added to reflect new blood collection method for DNA analysis and in regard to additional translational studies for which funding has recently been approved

19.1.5 Amendments Made To Protocol Version 4.0 (Dec 2007)

General Information Section - Randomisation and SAE reporting details sections clarified

Section 1.2 and throughout protocol - Efficacy Stages 1-111 renamed to Activity Stages 1-111 for accuracy and clarity

Section 1.2 - Follow schedule corrected

Section 4.1.2 - Inclusion criteria widened to include high risk relapsing patients, that would not have met the previous PSA based criteria

Section 4.1.3 - Note added to reference location of WHO performance status definitions

Section 4.2 - Notes added to reference locations of toxicity gradings and NYHA classifications

Section 4.3.1 - Timings of baseline scan information changed to accurately reflect most common current practice

Section 6.1.1 - Information about use of LHRH antagonists to ensure that the protocol accurately reflects current and future practice

Section 6.1.1 - Information about suggested duration of hormone therapy added to ensure that the protocol accurately reflects current practice

Section 6.2.2 - Additional information added about the timing of liver function tests prior to docetaxel administration added for clarity

Section 6.6.4 - Information on radiotherapy data collection added

Section 7.1.1 - Erroneous information about the timing of PSA measurements removed

Figure 3 - Moved to new section in protocol for clarity and extended to include current information on data collection

Figure 3b - Added to describe how extent of data collection during follow-up should change, post treatment and post progression

Figure 4 - Notes added to explain the changes in data collected at follow-up and to information that the quality of life study will be applicable to the first 700 patients randomised only

Figure 4 - Note added to include palliative radiotherapy CRF

Section 11.3 - SAE reporting information updated

Section 19 - Protocol amendments list updated

19.1.6 Amendments Made To Protocol Version 5.0 (Aug 2008)

1. General Information Section – Randomisation phone line number updated – non UK extension added
2. Section 3 – Information about QL study removed to reflect closure of QL study after first 700 patients
3. Section 4.2 – Exclusion criteria clarified to explain that only patients with severe poor cardiovascular history should be excluded
4. Section 4.3.1 – Information on co-administration of NSAIDS with celecoxib changed based on clinical advice.
5. Section 5 - Randomisation phone line number updated – non UK extension added
6. Section 6.2.1. – Information added to clarify that patients who develop an osteonecrosis of the jaw should stop zoledronic acid treatment
7. Section 6.2.3 – ‘severe’ text added to acurately reflect which patients should be excluded based on their cardiovascular history

8. Section 7.1.2 – Defination of disease progression extended for clarity

9. Figure 3 – Updated to include reference to newly created skeltal related event form

10. Figure 4 – Previous error in table amended to show that the 4th Zoledronic Acid form that is submitted contains information about 3 cycles rather than 2 as previously indicated

11. Table 4 – 'Other important medical condition' added to definition of serious in the SAE section, to accuratly reflect SAE form and current practice

12. Section 11.1 – Information added on reporting or pregnancies

13. Section 17 - Information about QL study removed to reflect closure of QL study after first 700 patients

19.1.7 Amendments Made To Protocol Version 6.0 (Jul 2009)

1. General Information Section – Trial Pharmacist removed and changes of:

Co-Investigator

Patient Representatives

Trial Manager

Data Manager

General Information Section - Coordinating Centre – address change

General Information Section – change of Sponsor address

Section 1.1 – ratio of patients randomised to the investigational arms updated

Section 1.2 – figure 1b added to clarify trial design from Apr-2011 onwards

Section 1.2 – paragraph added to explain trial changes after the second activity analysis

Section 1.2 – wording added to clarify that QL data only collected for first 700 patients randomised

Section 1.3 – SSA Favourable Opinion removed from list of trial documentation required ahead of site accreditation

Section 2.1 – Amount of men diagnosed with prostate cancer annually updated

Section 2.4 –note added to explain completion of recruitment to celecoxib- containing arms

Section 2.5.2 - note added to explain completion of recruitment to celecoxib- containing arms

Section 3 – SSA Favourable Opinion removed

Section 4.2 – Exclusion criterion xiii greyed out

Section 4.3.1 – paragraph removed regarding potential randomisation to celecoxib-containing arms

Section 5 – Randomisation instructions expanded to exclude public holidays or dates when notice has been given by the CTU

Section 6.1.4 – formatting changed to grey font to reflect recruitment completion for arm D

Section 6.1.6 - formatting changed to grey font to reflect recruitment completion for arm F

Section 6.2.3 – recruitment note added

Section 6.6.3 – radiotherapy statement changed to reflect data from recent trials

Section 7.1.2 – removal of reference to SRE- specific CRF

Section 7.3 – Figure 3 - Addition of Bone Density Risk Factor Form and BMD sub-study assessment forms to summary of timing table

Section 7.3 – Figure 4 – Weeks added to timings of assessments post 2 years

Section 7.3- Figure 4 – note added to explain recruitment completion for arms D and F

Section 12.1 – Wording changed to reflect change to randomisation allocation ratio

Section 12.1 – Addition of statement regarding new information emerging during the trial

Section 12.2 – Reference to SSA removed

Section 16.3 – Statement added regarding actioning IDMC recommendation ahead of TSC ratification

19.1.8 Amendments Made To Protocol Version 7.0 (Jul 2011)

1. General Information Section- SAE reporting fax number corrected

2. Section 11- SAE reporting fax number corrected

19.1.9 AMENDMENTS MADE TO PROTOCOL VERSION 7.1 (JUL 2011)

Throughout protocol – numbering has been updated in some sections new accommodate new information that has been added.

General Information Section – contact details updated

General Information Section – Funding information updated to include involvement from additional company

General Information Section – Wording on compliance and regulations updated to reflect current MRC CTU standard wording

General Information Section – Abbreviations list updated

Section 1.1 – The number of investigational agents being studied updated from three to four

Section 1.1 – Information regarding celecoxib updated to reflect that recruitment to these arms was discontinued in Apr-2011

Section 1.1 – Information about new IMP, Abiraterone inserted

Section 1.1 – Sample size and trial duration information updated to reflect changes brought about by additional trial arm

Section 1.2 – Summary information updated to reflect the discontinuation of recruitment to celecoxib arms and the addition of abiraterone

Figures 1a, b and c - Updated to reflect the discontinuation of recruitment to celecoxib arms and the addition of abiraterone

Section 1.2 – Information on trial stages updated to reflect changes brought about by additional trial arm

Section 1.2 – Information updated regarding the re-opening of the quality of life sub-study from implementation of protocol version 8.0

Section 2.1 – Wording related to hormone therapy updated for clarity

Section 2.1 - Updated to reflect the discontinuation of recruitment to celecoxib arms and the addition of abiraterone

Section 2.2 – Updated references added

Section 2.3 – Updated references added

Section 2.5 – Section added to give background information on new IMP, abiraterone

Section 2.6.1 – Updated references added

Section 2.7 – Section added to give information regarding radiotherapy which is to be given as part of standard care following recently published trial data.

Section 3 – Wording updated regarding selection of investigators to reflect current MRC CTU practice

Section 4.1 – Inclusion criteria updated with new criterion regarding radiotherapy use

Section 4.1 - Inclusion criteria updated with new criterion regarding contraceptive use

Section 4.1 – Wording of inclusion and exclusion criteria updated for clarity

Section 4.1 – Exclusion criteria updated with new criterion regarding acceptable liver function for trial entry

Section 4.1 – Exclusion criteria updated with specifics related to blood pressure levels

Section 4.1 - Exclusion criteria updated with new criterion regarding concomitant medications

Section 4.1 - Exclusion criteria updated with new criterion regarding prior treatment with abiraterone

Section 4.1 - Exclusion criteria updated with new criterion regarding prior treatment with chemotherapy

Section 4.1 - Exclusion criteria updated with new criterion regarding prior treatment with zoledronic acid

Section 4.3 – Wording updated to reflect that patients who initially fail screening can be re-screened at a later date

Section 4.3.2 – Wording updated regarding prior anti-androgen and LHRH use updated for clarity

Section 5.1 – Co-enrolment guidelines information updated to describe newly created co-enrolment CRF

Section 6.1 – Trial treatment information updated to reflect the fact that anti-androgens alone will be no longer permitted as hormone therapy

Section 6.1.1 – Updated to describe patients for whom radiotherapy should be given as standard practice

Section 6.1.1 a and b - Sections added to give information regarding radiotherapy treatment

Section 6.1.1-6.1.6 – References to further sections updated

Section 6.1.7 – Section added to describe abiraterone treatment

Section 6.2.4 - Section added to describe abiraterone treatment

Section 6.6 - Section added to give information regarding radiotherapy treatment

Section 7.1.1 – Reference to blood being taken at patient's home removed as this does not occur in practice

Section 7.1.2 – Wording updated regarding the reporting of biochemical failures for clarity

Section 7.1.2 – Wording updated regarding skeletal-related events for clarity

Section 7.1.3 – Section added to describe additional assessments required related to abiraterone treatment

Section 7.1.4 – Section added to provide information on when treatment should commence

Figure 4 – Updated for clarity regarding return of BMD sub-study forms, the addition the co-enrolment CRF and the description of the re-opening of the QoL Sub-study.

Figure 5 – Updated with reference to abiraterone and co-enrolment form

Section 7.3 - Wording on trial closure updated to reflect current MRC CTU standard wording

Section 8.1 – Additional criteria for definition of progression added for clarity

Section 8.1 – Definition of progression for abiraterone patients added.

Section 9 – Statistical information updated to describe the addition of the new trial arm

Section 11 – Safety reporting wording updated for clarity

Section 11 – SAE reporting fax number updated

Section 12 – Ethical information updated to describe the unequal randomisation allocation ratio

Section 12 – Ethical information updated to describe that the visit schedule will vary according to trial arm

Section 12.2 – Wording updated to reflect international participation in the trial

Section 13 – Wording updated to reflect international participation in the trial

Section 14 – Wording updated to reflect international participation in the trial

Section 15 - Updated to reflect the discontinuation of recruitment to celecoxib arms and the addition of abiraterone

Section 16 – Reference to trial committee charters added for information

Section 17.1 – Information added to reflect re-opening of quality of life sub-study

Section 17.2 – Timing of health economics analysis updated to previous error

Section 18 – Information on publication policy expanded for clarity

Section 19 – Information regarding amendments to protocol appendices moved to the separate appendices document

Section 20 – References extensively updated

19.1.10 Amendments Made To Protocol Version 8.0 (Sep 2011)

Throughout protocol – numbering, sections headings, tables, figures and bibliographical references have been updated in some sections to accommodate new information that has been added

Throughout protocol – Androgen Deprivation Therapy has replaced Hormone Therapy as deemed more representative of the type of hormone therapy used in the study

General Information Section – New staff members of the MRC CTU and Co-Investigators added and contact details updated

General Information Section – Abbreviations list updated

Section 1.1 – Information regarding the new research radiotherapy treatment inserted

Section 1.1 – Information regarding docetaxel updated

Section 1.2 – Wording updated to reflect the addition of the new research comparison arm

Section 1.3 – Additional criteria for the re-accreditation of participating centres (for protocol version 9.0 only)

Section 2.1.1 – Wording updated to clarify the use of anti-androgen in trial patients

Section 2.1.2 – Information added to describe the rationale for the RT comparison arm

Section 2.8 – Information added to describe research RT treatment to prostate for patients with newly diagnosed metastatic disease

Section 3.1 – Information added to describe RT Quality Assurance procedures and centre accreditation

Section 4.1.1 to 4.1.3 – Wording updated to clarify inclusion criteria for all patients groups (newly diagnosed non-metastatic, metastatic and relapsing patients)

Section 4.2 – Clarification added on cardiovascular exclusion criteria

Section 4.2 – New exclusion criterion added concerning patients with prior exposure to hormone therapy

Section 4.2 – New exclusion criterion added to reflect the addition of the new RT comparison arm

Section 4.4.1 – Clarification added regarding pre-randomisation checks

Section 4.4.2 – Clarification added regarding permissible hormone therapy duration prior to randomisation

Section 4.4.5 – Information added regarding starting research radiotherapy treatment

Section 4.4.6 – Information updated on concomitant medications

Section 5 – Clarification regarding randomisation allocation added to reflect the addition of the new RT research arm

Section 6.1.8 – Information added to describe the administration of research radiotherapy

Section 6.2.1 – Clarification added regarding the measurement of serum creatinine levels prior to the administration of zoledronic acid

Section 6.2.3 – Clarification regarding the completion of recruitment to the celecoxib containing arms

Section 6.25 – Information added regarding the administration of research radiotherapy treatment

Section 6.6 – Clarification incorporated to describe the administration of standard-of-care radiotherapy

Section 7.1.4 – Information added regarding data collection and non administration of standard radiotherapy

Section 7.2 – Section updated to include new treatment specific CRFs and timing of CRFs

Section 8.1 – Clarification added for the criteria to stop treatment for patients randomised to arm G

Section 8.2 – Section expanded to include additional details on study patient transfer to different centres

Section 8.3 – Additional sentence inserted to reinforce the importance of compliance with follow up assessments

Section 9.1 – Additional paragraph inserted to clarify the method of randomisation and allocation distribution in the light of the introduction of the new RT arm

Section 9.4 – Wording updated to clarify the assessment of safety data

Section 9.5.4 – Wording updated concerning the end of randomisations to arm G

Section 9.6 to 9.6.4 – Section added describing sample size issues and trial stages for arm H

Section 9.8 – Clarification on intermediate stopping guidelines

Section 9.9 – Clarification on the outline analysis plan

Section 11 – Information on safety reporting updated to reflect the addition of the research RT comparison arm

Section 11 – Clarification added regarding arm A safety reporting timelines

Section 12.1 – Clarification added regarding the Principal Investigator’s responsibilities

Section 14 – Indemnity section updated to reflect current MRC policy

Section 16 – Clarification regarding TMG membership

Section 17.3 – Section on Bone Mineral Density sub-study removed

Section 19 – Information regarding amendments to protocol appendices moved to the separate appendices document

Section 20 – References updated

20 REFERENCES

1. James ND, Sydes MR, Mason MD, Clarke NW, Anderson J, Dearnaley DP, et al. Celecoxib plus hormone therapy versus hormone therapy alone for hormone-sensitive prostate cancer: first results from the STAMPEDE multiarm, multistage, randomised controlled trial. The lancet oncology. 2012;13(5):549-58. Epub 26-Mar-2012.

2. Cancer Research UK. CancerStats Key Facts: Prostate Cancer. Cancer Research UK. 2011.

3. Sharifi N, Gulley JL, Dahut WL, Sharifi N, Gulley JL, Dahut WL. An update on androgen deprivation therapy for prostate cancer. Endocrine-Related Cancer. 2010;17(4):R305-15.

4. Widmark A, Klepp O, Solberg A, Damber JE, Angelsen A, Fransson P, et al. Endocrine treatment, with or without radiotherapy, in locally advanced prostate cancer (SPCG-7/SFUO-3): an open randomised phase III trial. Lancet. 2009;373(9660):301-8. Epub 2008/12/19.

5. Warde PR, Mason MD, Sydes MR, Gospodarowicz MK, Swanson GP, Kirkbride P, et al. Intergroup randomized phase III study of androgen deprivation therapy (ADT) plus radiation therapy (RT) in locally advanced prostate cancer (CaP) (NCIC-CTG, SWOG, MRC-UK, INT: T94-0110; NCT00002633). J Clin Oncol. 2010;28(18s Supplement: Proceedings of ASCO 2010):Abstr CRA4504.

6. Warde P, Mason M, Ding K, Kirkbride P, Brundage M, Cowan R, et al. Survival Benefit with Combined Androgen Deprivation and Radiation Therapy in Locally Advanced Prostate Cancer – Results of a Phase III Trial. The Lancet. 2011 - in press.

7. Mason M, Sydes M, Parulekar W, Parmar M, Anderson J, Barber J, et al. Final analysis of intergroup randomized phase III study of androgen deprivation therapy (ADT) + radiation therapy (RT) in locally advanced prostate cancer (CaP) (NCIC-CTG, SWOG, MRC-UK, INT: T94-0110). National Cancer Research Institute (NCRI) Cancer Conference 2012. 2012;2012.

8. Scher HI, Kelly WK. Flutamide withdrawal syndrome: its impact on clinical trials in hormone-refractory prostate cancer. Journal of Clinical Oncology. 1993;11(8):1566-72.

9. Saad F, Colombel M. Management of castration-resistant prostate cancer: bisphosphonates and emerging therapies. Expert Review of Anticancer Therapy. 2010;10(12):1991-2002.

10. Sartor O, Halstead M, Katz L, Sartor O, Halstead M, Katz L. Improving outcomes with recent advances in chemotherapy for castrate-resistant prostate cancer. Clinical Genitourinary Cancer. 2011;8(1):23-8.

11. Attard G, Richards J, de Bono JS, Attard G, Richards J, de Bono JS. New strategies in metastatic prostate cancer: targeting the androgen receptor signaling pathway. Clinical Cancer Research. 2011;17(7):1649-57.

12. Lipton A. Improving progression-free and overall survival in patients with cancer: a potential role for bisphosphonates. Expert Opinion on Pharmacotherapy. 2011;12(5):749-62.

13. Lipton A, Small E, Saad F, Gleason D, Gordon D, Smith M, et al. The new bisphosphonate, Zometa (zoledronic acid), decreases skeletal complications in both osteolytic and osteoblastic lesions: a comparison to pamidronate. [Review] [55 refs]. Cancer Investigation. 2002;20 Suppl 2:45-54.

14. Green JR, Muller K, Jaeggi KA. Preclinical pharmacology of CGP 42'446, a new, potent, heterocyclic bisphosphonate compound. Journal of Bone & Mineral Research. 1994;9(5):745-51.

15. Major P, Lortholary A, Hon J, Abdi E, Mills G, Menssen HD, et al. Zoledronic acid is superior to pamidronate in the treatment of hypercalcemia of malignancy: a pooled analysis of two randomized, controlled clinical trials. J Clin Oncol. 2001;19(2):558-67. Epub 2001/02/24.

16. Costa L, Harper P, Coleman RE, Lipton A, Costa L, Harper P, et al. Anticancer evidence for zoledronic acid across the cancer continuum. Critical Reviews in Oncology-Hematology. 2011;77 Suppl 1:S31-7.

17. Rosen LS, Gordon D, Kaminski M, Howell A, Belch A, Mackey J, et al. Long-term efficacy and safety of zoledronic acid compared with pamidronate disodium in the treatment of skeletal complications in patients with advanced multiple myeloma or breast carcinoma: a randomized, double-blind, multicenter, comparative trial. Cancer. 2003;98(8):1735-44. Epub 2003/10/10.

18. Rosen LS, Gordon DH, Dugan W, Jr., Major P, Eisenberg PD, Provencher L, et al. Zoledronic acid is superior to pamidronate for the treatment of bone metastases in breast carcinoma patients with at least one osteolytic lesion. Cancer. 2004;100(1):36-43. Epub 2003/12/24.

19. Saad F, Gleason D, Murray R, Tchekmedyian S, Venner P, Lacombe L, et al. A Randomized, Placebo-Controlled Trial of Zoledronic Acid in Patients With Hormone-Refractory Metastatic Prostate Carcinoma Journal of the National Cancer Institute. 2002;94(19).

20. Dearnaley DP, Sydes MR, Mason MD, Stott M, Powell CS, Robinson AC, et al. A double-blind, placebo-controlled, randomized trial of oral sodium clodronate for metastatic prostate cancer (MRC PR05 Trial). J NatlCancer Inst. 2003;95(17):1300-11.

21. Petrylak DP, Tangen CM, Hussain MH, Lara PN, Jr., Jones JA, Taplin ME, et al. Docetaxel and estramustine compared with mitoxantrone and prednisone for advanced refractory prostate cancer. N Engl J Med. 2004;351(15):1513-20. Epub 2004/10/08.

22. Tannock IF, de Wit R, Berry WR, Horti J, Pluzanska A, Chi KN, et al. Docetaxel plus prednisone or mitoxantrone plus prednisone for advanced prostate cancer. N Engl J Med. 2004;351(15):1502-12. Epub 2004/10/08.

23. Hawkey CJ. COX-2 inhibitors.[comment]. Lancet. 1999;353(9149):307-14.

24. Taketo MM. Cyclooxygenase-2 inhibitors in tumorigenesis (Part II). Journal of the National Cancer Institute. 1998;90(21):1609-20.

25. Taketo MM. Cyclooxygenase-2 inhibitors in tumorigenesis (Part I). Journal of the National Cancer Institute. 1998;90(20):1529-36.

26. Nelson JE, Harris RE. Inverse association of prostate cancer and non-steroidal anti-inflammatory drugs (NSAIDs): results of a case-control study. Oncology Reports. 2000;7(1):169-70.

27. Eberhart CE, Coffey RJ, Radhika A, Giardiello FM, Ferrenbach S, DuBois RN. Up-regulation of cyclooxygenase 2 gene expression in human colorectal adenomas and adenocarcinomas. Gastroenterology. 1994;107(4):1183-8.

28. Roberts RO, Jacobson DJ, Girman CJ, Rhodes T, Lieber MM, Jacobsen SJ. A population-based study of daily nonsteroidal anti-inflammatory drug use and prostate cancer. [see comments.]. Mayo Clinic Proceedings. 2002;77(3):219-25.

29. Basler JW, Piazza GA, Basler JW, Piazza GA. Nonsteroidal anti-inflammatory drugs and cyclooxygenase-2 selective inhibitors for prostate cancer chemoprevention. Journal of Urology. 2004;171(2 Pt 2):S59-62; discussion S-3.

30. Masferrer JL, Leahy KM, Koki AT, Zweifel BS, Settle SL, Woerner BM, et al. Antiangiogenic and antitumor activities of cyclooxygenase-2 inhibitors. Cancer Research. 2000;60(5):1306-11.

31. Steinbach G, Lynch PM, Phillips RK, Wallace MH, Hawk E, Gordon GB, et al. The effect of celecoxib, a cyclooxygenase-2 inhibitor, in familial adenomatous polyposis. New England Journal of Medicine. 2000;342(26):1946-52.

32. Solomon SD, McMurray JJ, Pfeffer MA, Wittes J, Fowler R, Finn P, et al. Cardiovascular risk associated with celecoxib in a clinical trial for colorectal adenoma prevention. New England Journal of Medicine. 2005;352(11):1071-80.

33. Danila DC, Morris MJ, de Bono JS, Ryan CJ, Denmeade SR, Smith MR, et al. Phase II multicenter study of abiraterone acetate plus prednisone therapy in patients with docetaxel-treated castration-resistant prostate cancer. J Clin Oncol. 2010;28(9):1496-501. Epub 2010/02/18.

34. Reid AH, Attard G, Danila DC, Oommen NB, Olmos D, Fong PC, et al. Significant and sustained antitumor activity in post-docetaxel, castration-resistant prostate cancer with the CYP17 inhibitor abiraterone acetate. J Clin Oncol. 2010;28(9):1489-95. Epub 2010/02/18.

35. Ryan CJ, Smith MR, Fong L, Rosenberg JE, Kantoff P, Raynaud F, et al. Phase I clinical trial of the CYP17 inhibitor abiraterone acetate demonstrating clinical activity in patients with castration-resistant prostate cancer who received prior ketoconazole therapy. Journal of Clinical Oncology. 2010;28(9):1481-8.

36. de Bono JS, Logothetis CJ, Molina A, Fizazi K, North S, Chu L, et al. Abiraterone and increased survival in metastatic prostate cancer. New England Journal of Medicine. 2011;364(21):1995-2005.

37. Ryan CJ, Smith MR, de Bono JS, Molina A, Logothetis C, de Souza PL, et al. Interim analysis (IA) results of COU-AA-302, a randomized, phase III study of abiraterone acetate (AA) in chemotherapy-naive patients (pts) with metastatic castration-resistant prostate cancer (mCRPC). J Clin Oncol. 2012;30(Suppl):Abstr LBA4518.

38. Attard G, Reid AH, de Bono JS, Attard G, Reid AHM, de Bono JS. Abiraterone acetate is well tolerated without concomitant use of corticosteroids. Journal of Clinical Oncology. 2010;28(29):e560-1; author reply e2.

39. Flanigan RC, Salmon SE, Blumenstein BA, Bearman SI, Roy V, McGrath PC, et al. Nephrectomy followed by interferon alfa-2b compared with interferon alfa-2b alone for metastatic renal-cell cancer. N Engl J Med. 2001;345(23):1655-9. Epub 2002/01/05.

40. Mickisch GH, Garin A, van Poppel H, de Prijck L, Sylvester R. Radical nephrectomy plus interferon-alfa-based immunotherapy compared with interferon alfa alone in metastatic renal-cell carcinoma: a randomised trial. Lancet. 2001;358(9286):966-70. Epub 2001/10/05.

41. Warde P, Mason M, Ding K, Kirkbride P, Brundage M, Cowan R, et al. Combined androgen deprivation therapy and radiation therapy for locally advanced prostate cancer: a randomised, phase 3 trial. The Lancet. 2011;378:2104-11. Epub 03-Nov-2011.

42. Neville-Webbe HL, Holen I, Coleman RE. The anti-tumour activity of bisphosphonates. Cancer Treatment Reviews. 2002;28(6):305-19.

43. Witters LM, Crispino J, Fraterrigo T, Green J, Lipton A. Effect of the combination of docetaxel, zoledronic acid, and a COX-2 inhibitor on the growth of human breast cancer cell lines. American journal of clinical oncology. 2003;26(4):S92-7. Epub 2003/08/07.

44. Kouloulias V, Giraud J-Y, Davis BJ, Dusserre A, Zurlo A, Bolla M. Quality assurance of the 22961 EORTC trial. A phase III study of the optimal combination of hormonal adjuvant treatment by LHRH analogue and radiation therapy for the management of locally advanced prostate cancer: the dummy run. 2004(0167-8140 (Print)).

45. Royston P, Parmar MKB, Qian W. Novel designs for multi-arm clinical trials with survival outcomes with an application in ovarian cancer. Statistics in Medicine. 2003;22(14):2239-56.

46. Royston P, Barthel FMS, Parmar MKB, Choodari-Oskooei B, Isham V. Designs for clinical trials with time-to-event outcomes based on stopping guidelines for lack of benefit. Trials. 2011;12(1):81.

47. Royston P. nstage: MAMS trial sample size calculator. MRC Clinical Trials Unit, London2009.

48. Haybittle JL. Repeated assessment of results in clinical trials of cancer treatment. The British journal of radiology. 1971;44(526):793-7. Epub 1971/10/01.

49. Peto R, Pike MC, Armitage P, Breslow NE, Cox DR, Howard SV, et al. Design and analysis of randomized clinical trials requiring prolonged observation of each patient. I. Introduction and design. British journal of cancer. 1976;34(6):585-612. Epub 1976/12/01.

50. Dolan P, Gudex C, Kind P, Williams A. Discussion Paper 138: A social tarif for EuroQol: results from a UK General Population Survey. . Centre for Health Economics. University of York, 1995.

STAMPEDE

Systemic Therapy in Advancing or Metastatic Prostate Cancer: Evaluation of Drug Efficacy

A multi-arm multi-stage randomised controlled trial

Version: 10.0
Date: 02-Apr-2013

MRC CTU ID:	PR08
ISRCTN #:	ISRCTN78818544
NCT #:	NCT00268476
EUDRACT #:	2004-000193-31
CTA #:	00316/0026/001-0001
MREC #:	04/MRE07/35

Authorised by:

Name: PROFESSOR NICHOLAS D JAMES
Role: CHIEF INVESTIGATOR

Name: MATTHEW SYDES
Role: TRIAL STATISTICIAN

GENERAL INFORMATION

This document was constructed using the MRC CTU Protocol Template Version 4.0. It describes the STAMPEDE trial, coordinated by the Medical Research Council (MRC) Clinical Trials Unit (CTU), and provides information about procedures for entering patients into it. The protocol should not be used as an aide-memoire or guide for the treatment of other patients. Every care has been taken in drafting this protocol, but corrections or amendments may be necessary. These will be circulated to the registered investigators in the trial, but sites entering patients for the first time are advised to contact the Cancer Group, MRC CTU, London, UK, to confirm they have the most up-to-date version.

COMPLIANCE

The trial will be conducted in compliance with the approved protocol, the Declaration of Helsinki 1996, the principles of Good Clinical Practice (GCP), Commission Directive 2005/28/EC with the implementation in national legislation in the UK by Statutory Instrument 2004/1031 and subsequent amendments, the UK Data Protection Act (DPA number: Z5886415), and the National Health Service (NHS) Research Governance Framework for Health and Social Care (RGF). International sites will comply with the principles of GCP as laid down by the ICH topic E6 (Note for Guidance on GCP), Commission Directive 2005/28/EC (the European Directive 2001/20/EC [where applicable]) and applicable national regulations.

SPONSOR

Medical Research Council, 2nd Floor, David Phillips Building, Polaris House, North Star Avenue, Swindon, SN2 1FL, UK

FUNDING

Clinical Trials Advisory Awards Committee (on behalf of Cancer Research UK, Medical Research Council, and other charities) together with educational grants from Novartis, Sanofi-Aventis, Pfizer and Janssen Pharma NV.

AUTHORISATIONS AND APPROVALS

The following persons are authorised to sign the final protocol and protocol amendments for the sponsor: Professor Nicholas James (Chief Investigator) and Matthew Sydes (Trial Statistician).

TRIAL REGISTRATION

This trial has been registered with the ClinicalTrials.gov Clinical Trials Register, where it is identified as NCT00268476.

RANDOMISATIONS
To randomise, call MRC CTU, Monday to Friday 0900-1700
excluding public holidays or dates when notice has been given by the CTU.
Tel: +44 (0) 20 7670 4777

SAE REPORTING
Fax to 020 7670 4818 within 24 hours of becoming aware of the event

TRIAL ADMINISTRATION

COORDINATING SITE

MRC Clinical Trials Unit<br>Aviation House<br>Kingsway<br>London<br>WC2B 6NH<br>UK	Switchboard:<br>Fax:<br>Email:	020 7670 4700<br>020 7670 4818<br>stampede@ctu.mrc.ac.uk

MRC CTU STAFF

Trial Manager:	Francesca Schiavone	Tel:	0207 670 4632
Trial Manager:	Charlene Green	Tel:	0207 670 4882
Data Manager:	Hannah Gardner	Tel:	0207 670 4809
Data Manager:	Dominic Hague	Tel:	0207 670 4947
Data Manager:	Katie Ward	Tel:	0207 670 4794
Statistician:	Gordana Jovic	Tel:	0207 670 4647
Senior Trial Statistician:	Matt Sydes	Tel:	0207 670 4798
Director of MRC CTU:	Max Parmar	Tel:	0207 670 4729
Trial Surgeon:	Alastair Ritchie		

CHIEF INVESTIGATOR

Professor Nicholas James
School of Cancer Sciences
University of Birmingham
Edgbaston, Birmingham
B15 2TT
UK

Tel: 0121 414 4097/4474

Fax: 0121 414 3263

Email: n.d.James@bham.ac.uk
j.d.alonzo@bham.ac.uk(sec)

CO-INVESTIGATORS

Prof Noel Clarke, Urologist	Manchester, UK
Prof David Dearnaley, Oncologist	Sutton, UK
Prof Johann de Bono, Oncologist	Sutton, UK
Prof Malcolm Mason, Oncologist	Cardiff, UK
Dr Chris Parker, Oncologist	Sutton, UK
Dr Martin Russell, Oncologist	Glasgow, UK
Prof George Thalmann, Urologist	Bern, Switzerland

Patient Representative

Robin Millman, Patient

Health Economics

Prof Mark Sculpher	York, UK

Molecular Genetics

Prof John Masters, Pathologist	London, UK

For full details of all trial committees, please see **Appendix M**.

CONTENTS

GENERAL INFORMATION I

CONTENTS 4

ABBREVIATIONS 8

1 SUMMARY 11
1.1 LAY SUMMARY 11
1.2 ABSTRACT AND SUMMARY OF TRIAL DESIGN 12
1.3 TRIAL DOCUMENTATION 16

2 BACKGROUND 17
2.1 INTRODUCTION AND SETTING 17
2.1.1 Long-term Androgen Deprivation Therapy 17
2.1.2 Role of Radiotherapy for Patients With M0 Disease 17
2.2 RATIONALE 17
2.3 DESIGN 18
2.4 RESEARCH TREATMENT AND BIOSPHOSPHONATES 18
2.5 RESEARCH TREATMENT: CHEMOTHERAPY 19
2.6 RESEARCH TREATMENT: CYCLOOXYGENASE-2 INHIBITORS 19
2.7 RESEARCH TREATMENT: STEROID SYNTHESIS INHIBITORS 20
2.8 RESEARCH TREATMENT: RADIOTHERAPY TO THE PROSTATE FOR PATIENTS WITH NEWLY-DIAGNOSED METASTATIC DISEASE 20
2.9 RESEARCH TREATMENT: COMBINATIONS 21
2.9.1 Bisphosphonate And Chemotherapy 21
2.9.2 Bisphosphonate And Cyclooxygenase-2 Inhibitors 21

3 SELECTION OF INSTITUTIONS AND INVESTIGATORS 23
3.1 RADIOTHERAPY ACCREDITATION 23

4 SELECTION OF PATIENTS 24
4.1 PATIENT INCLUSION CRITERIA 24
4.1.1 High-Risk Newly Diagnosed Non-Metastatic Node-Negative Disease 24
4.1.2 Newly Diagnosed Metastatic Or Node-Positive Disease 24
4.1.3 Previously Treated With Radical Surgery And/Or Radiotherapy, Now Relapsing 24
4.1.4 For All Patients 24
4.2 PATIENT EXCLUSION CRITERIA 25
4.3 SELECTION CRITERIA FOR COMPARISON OF RESEARCH (M1) RT FOR METASTATIC DISEASE 26
4.4 SCREENING PROCEDURES 26
4.4.1 Investigations Prior To Randomisation 26
4.4.2 Androgen Deprivation Therapy Prior To Randomisation 26
4.4.3 Hypercalcaemia At Randomisation 27
4.4.4 NSAIDs And Cox-2 Inhibitors At Randomisation 27
4.4.5 Starting Trial Treatment 27
4.4.6 Concomitant Medications 27
4.5 ADDITIONAL DETAILS FOR PATIENTS JOINING SUB-STUDIES 28

5 RANDOMISATION AND ENROLMENT 29
5.1 CO-ENROLMENT GUIDELINES 29

6 TREATMENT OF PATIENTS 30
6.1 TRIAL TREATMENT 30
6.1.1 Arm A: ADT Alone Or ADT + Standard-of-Care (M0) RT (Control Arm) 30
6.1.2 Arm B: ADT + Zoledronic Acid 31
6.1.3 Arm C: ADT + Docetaxel 31
6.1.4 Arm D: ADT + Celecoxib 31
6.1.5 Arm E: ADT + Docetaxel + Zoledronic Acid 31
6.1.6 Arm F: ADT + Zoledronic Acid + Celecoxib 32
6.1.7 Arm G: ADT + Abiraterone 32
6.1.8 ARM H: ADT + Prostate Radiotherapy in M1 patients 33
6.2 ADMINISTRATION AND DOSE MODIFICATIONS 33
6.2.1 Zoledronic Acid 33
6.2.2 Docetaxel 33
6.2.3 Celecoxib 34
6.2.4 Abiraterone 34
6.2.5 Research (M1) Prostate Radiotherapy 34
6.3 TRIAL PRODUCTS 36
6.4 MEASURES OF COMPLIANCE/ADHERENCE 36
6.5 TREATMENT DATA COLLECTION 36
6.6 ADMINISTRATION OF STANDARD RADIOTHERAPY TO NON-METASTATIC PATIENTS 36
6.6.1 Treatment Details 36
6.7 NON-TRIAL TREATMENT 38
6.7.1 Medications Permitted 38
6.7.2 Data On Concomitant Medication 38

7 ASSESSMENTS AND PROCEDURES 39
7.1 FLOW CHART/SCHEDULE FOR FOLLOW-UP 39
7.1.1 Psa Measurements 39
7.1.2 Assessment Of Treatment Failure (Definition Of Progression) 39
7.1.3 Additional Safety Assessment 39
7.1.4 Data Collection and Non-Administration Of Standard Radiotherapy 39
7.1.5 Data Collection Palliative Radiotherapy 40
7.1.6 Data Collection Research (M1) Radiotherapy 40
7.1.7 Follow-Up Schedules 40
7.2 FOLLOW-UP 40
7.3 TRIAL CLOSURE 42

8 STOPPING OF TREATMENT OR FOLLOW UP 44
8.1 STOPPING RESEARCH INTERVENTIONS 44
8.2 PATIENT TRANSFERS 44
8.3 WITHDRAWAL FROM THE TRIAL COMPLETELY 44

9 STATISTICAL CONSIDERATIONS 46
9.1 METHOD OF RANDOMISATION 46
9.2 OUTCOME MEASURES 46
9.3 SAMPLE SIZE: PRINCIPLES AND ASSUMPTIONS 47
9.4 SAMPLE SIZE ISSUES AND TRIAL STAGES: ORIGINAL RESEARCH ARMS (B-F) 48

9.4.1 Pilot Phase: Original Research Arms (B-F) ... 48
9.4.2 Activity Stages I-Iii: Original Research Arms (B-F) ... 48
9.4.3 Efficacy Stage Iv: Original Research Arms (B-F) ... 49
9.4.4 Sample Size For Original Research Arms (B-F) ... 49
9.5 SAMPLE SIZE ISSUES AND TRIAL STAGES: ADDITIONAL RESEARCH ARM G ... 49
9.5.1 Pilot Phase: Additional Research Arm G ... 49
9.5.2 Activity Stages I-Iii: Additional Research Arm G ... 49
9.5.3 Efficacy Stage Iv: Additional Research Arm G ... 50
9.5.4 Sample Size For Additional Research Arm G ... 50
9.6 SAMPLE SIZE ISSUES AND TRIAL STAGES: ADDITIONAL RESEARCH ARM H ... 50
9.6.1 Pilot Phase: Additional Research Arm H ... 50
9.6.2 Activity Stages I-III: Additional Research Arm H ... 50
9.6.3 Efficacy Stage Iv: Additional Research Arm H ... 50
9.6.4 Sample Size For Additional Research Arm H ... 51
9.7 FURTHER NOTES ON TRIAL DESIGN ... 53
9.7.1 Overall Sample Size ... 53
9.7.2 Factorial Design ... 53
9.8 INTERIM MONITORING AND ANALYSES ... 53
9.9 OUTLINE ANALYSIS PLAN ... 53
9.9.1 Pilot / Safety Phases ... 54
9.9.2 Activity And Efficacy Stages ... 54

10 MONITORING AND QUALITY ASSURANCE ... 55
10.1 MONITORING AT MRC CTU ... 55
10.2 DIRECT ACCESS TO DATA ... 55
10.3 VISITS TO INVESTIGATOR SITES ... 55
10.4 CONFIDENTIALITY ... 55

11 SAFETY REPORTING ... 56
11.1 DEFINITIONS ... 56
11.1.1 Trial-Specific Exemptions ... 57
11.2 INSTITUTION/INVESTIGATOR RESPONSIBILITIES ... 57
11.2.1 Investigator Assessment ... 57
11.2.2 Notification Procedure ... 58
11.3 MRC CTU RESPONSIBILITIES ... 59

12 ETHICAL CONSIDERATIONS AND APPROVAL ... 60
12.1 ETHICAL CONSIDERATIONS ... 60
12.2 ETHICAL APPROVAL ... 60

13 REGULATORY APPROVAL ... 62

14 INDEMNITY ... 63

15 FINANCE ... 64

16 TRIAL COMMITTEES ... 65
16.1 TRIAL MANAGEMENT GROUP (TMG) ... 65
16.2 TRIAL STEERING COMMITTEE (TSC) ... 65
16.3 INDEPENDENT DATA MONITORING COMMITTEE (IDMC) ... 65

17 ANCILLARY STUDIES ... 67
17.1 QUALITY OF LIFE ... 67
17.2 HEALTH ECONOMICS ... 67
17.3 TRANSLATIONAL SUB-STUDIES ... 67
17.3.1 DNA Analysis ... 67
17.3.2 Tissue Microarray ... 68

18 PUBLICATION ... 69

19 PROTOCOL AMENDMENTS ... 70
19.1 PROTOCOL ... 70
19.1.1 Amendments Made To Sections In Protocol Version 1.0 (May 2004) ... 70
19.1.2 Amendments Made To Sections In Protocol Version 1.1 (May 2005) ... 70
19.1.3 Amendments Made To Sections In Protocol Version 2.0 (Jun 2005) ... 70
19.1.4 Amendments Made To Section In Protocol Version 3.0 (Jul 2006) ... 71
19.1.5 Amendments Made To Protocol Version 4.0 (Dec 2007) ... 72
19.1.6 Amendments Made To Protocol Version 5.0 (Aug 2008) ... 72
19.1.7 Amendments Made To Protocol Version 6.0 (Jul 2009) ... 73
19.1.8 Amendments Made To Protocol Version 7.0 (Jul 2011) ... 73
19.1.9 Amendments Made To Protocol Version 7.1 (Jul 2011) ... 74
19.1.10 Amendments Made To Protocol Version 8.0 (Sep 2011) ... 75

20 REFERENCES ... 77

ABBREVIATIONS

Abbreviation	Expansion
ACE	Angiotensin-Converting Enzyme
ACTH	Adrenocorticotropic hormone
ADT	Androgen deprivation therapy
AS	Activity Stage
bid	Twice a day (bis in die)
BP	Blood pressure
BSA	Body surface area
CERES	Consumers for Ethics in Research
CF	Consent Form
CI	Chief Investigator
CI	Confidence interval
COSTART	Coding Symbols for a Thesaurus of Adverse Reaction Terms
Cox 2	Cyclooxygenase 2
CRF	Case Report Form
CRUK	Cancer Research UK
CRPC	Castrate Refractory Prostate Cancer
CT	Computerised tomography
CTA	Clinical Trials Authorisation
CTAAC	Clinical Trials Advisory and Awards Committee
CTC	Common Toxicity Criteria
CTU	Clinical Trials Unit
CTV	Clinical Tumour Volume
CXR	Chest X-ray
DDX	Doctors and Dentists Exemption
DNA	Deoxyribonucleic Acid
DPA	Data Protection Act
ERC	Endpoint Review Committee
ES	Efficacy Stage
ICH	International Conference on Harmonization
ECG	Electro cardiogram
FBC	Full Blood Count
FFS	Failure-Free Survival

Abbreviation	Expansion
GCP	Good Clinical Practice
GP	General Practitioner
GRO	General Register Office
HE	Health Economics
HES	Hospital Episode Statistics
hr	Hour
HR	Hazard Ratio
HRPC	Hormone Refractory Prostate Cancer
HT	Hormone Therapy
IDMC	Independent Data Monitoring Committee
IM	Intramuscular
IMRT	Intensity Modulated Radiation Therapy
ISRCTN	International Standard Randomised Controlled Trial Number
IU	International Units
IV	Intravenous
LD	Longest diameter
LFTs	Liver Function Tests
LHRH	Luteinising Hormone Releasing Hormone
LREC	Local Research Ethics Committee
MHRA	Medicine and Healthcare Products Regulatory Agency
m	Month
min	Minutes
MRC	Medical Research Council
MREC	Multi-Centre Research Ethics Committee
MRI	Magnetic resonance imaging
NCI	National Cancer Institute (USA)
NCRN	National Cancer Research Network
NHS	National Health Service
NSAID	Non-Steroidal Anti-inflammatory Drugs
ONS	Office for National Statistics
OS	Overall Survival
PI	Principal Investigator
PIS	Patient Information Sheet
po	per orum (orally)

Abbreviation	Expansion
PSA	Prostate Specific Antigen
pts	Patients
PTV	Planned Tumour Volume
QALY	Quality-adjusted Life Years
qds	quater die sumendus (4 times each day)
QL	Quality of Life
R&D	Research and Development
RECIST	Response Evaluation Criteria In Solid Tumours
SAE	Serious Adverse Event
SAR	Serious Adverse Reaction
sc	Sub-cutaneous (under skin)
SNP	Single Nucleotide Polymorphism
SSA	Site Specific Assessment
STAMPEDE	Systemic Therapy in Advancing and Metastatic Prostate Cancer: Evaluation of Drug Efficacy
SUSAR	Suspected Unexpected Serious Adverse Reactions
SWOG	South West Oncology Group
TMG	Trial Management Group
TURP	Trans-Urethral Resection of Prostate
TSC	Trial Steering Committee
ULN	Upper Limit of Normal
U+E	Urea and Electrolytes
WHO	World Health Organisation

1 SUMMARY

1.1 LAY SUMMARY

Prostate cancers depend upon the male hormone testosterone for their growth. Lowering testosterone levels (either by removing all or part of both testes, or by giving anti-hormone treatment) slows the growth of prostate cancers. This type of treatment is called hormone treatment and is often used when prostate cancers have spread outside the prostate gland. Although hormone treatment is usually successful at stopping the cancer growing for a period of time, the cancer will begin to grow again in most men.

There are increasing numbers of treatments available for advanced prostate cancer. These treatments are usually used in prostate cancer when hormone treatment is no longer effective and the cancer has started to grow again. The aim of this trial, which is called STAMPEDE, is to assess some of these treatments, given earlier in the course of the disease in combination with hormone treatment.

The treatments currently being assessed during the trial are:

1. Zoledronic acid: Prostate cancer cells can spread to bones and weaken them. Zoledronic acid is a drug that reduces bone destruction and hardens bones. This may make them more resistant to attack by cancer cells.

2. Docetaxel: A drug that stops cells replicating that is currently being used to treat a range of cancers including lung, breast and ovarian cancer as well as prostate cancer. Docetaxel prolongs survival in men with relapsed metastatic prostate cancer.

3. Celecoxib: An aspirin-like drug that is used to treat arthritis. It slows down the growth of cancer cells in the laboratory. We wished to see if it had the same effect on cancer cells in patients. Recruitment to new patients for the evaluation of this drug is finished as a planned interim analysis failed to demonstrate sufficient activity.

4. Abiraterone (included from protocol version 8.0)**:** An inhibitor of steroid hormone synthesis that blocks prostate cancer cells from generating their own male hormones. This is thought to be a major way in which prostate cancer cells resume growth following castration based therapies. The agent prolongs survival when given to men following failure of docetaxel chemotherapy.

5. Prostate radiotherapy (included from protocol version 9.0)**:** treatment with high-energy x-rays targeted to the prostate gland. This treatment is now mandatory for patients with cancer that is confined to the prostate gland as large trials have shown it improves survival times. We are not certain whether we should give radiotherapy to the prostate if the cancer has already spread.

STAMPEDE will look at the effect of combining one or two of the treatments described above with hormone treatment. A computer program will be used to allocate which treatment the patient receives, using a chance process. The trial will look at the effects of the combined treatments on quality of life and find out whether the new treatment combinations increase the time when the cancer is not growing and ultimately results in patients living longer. The study will also look at which treatment provides the greater value for money for the health service. More than 5,000 patients will join the trial with answers becoming available over 7 to 12 years.

1.2 ABSTRACT AND SUMMARY OF TRIAL DESIGN

STAMPEDE is a multi-centre, randomised controlled trial for patients with locally advanced or metastatic prostate cancer who are about to commence Androgen Deprivation Therapy (ADT). Patients can have either newly diagnosed disease, or have been previously treated with radical radiotherapy or surgery but now have a rising prostate specific antigen (PSA) (further details on eligibility see **Section 4**). The trial will assess the effects of adding different agents, both as single agents and in combinations, to androgen deprivation therapy. The investigational agents are (i) a bisphosphonate, zoledronic acid, (ii) a cytotoxic chemotherapeutic agent, docetaxel and (iii) a cyclooxygenase (Cox-2) inhibitor, celecoxib. (iv) a novel androgen deprivation therapy drug called abiraterone, a steroid synthesis inhibitor. Recruitment to the celecoxib arms (D and F) is now closed. An additional arm containing abiraterone was added in protocol version 8.0. A further comparison arm involving prostate radiotherapy for patients with metastatic disease is added in the current protocol version 9.0. The trial has multiple arms; the control arm of the trial is androgen deprivation therapy (ADT) only, achieved through the use of luteinising hormone releasing hormone (LHRH) analogues or LHRH antagonists, or bilateral orchidectomy according to local practice. The other trial arms are summarised in **Figure 1 to 5**.

Figure 1: Recruiting arms of the STAMPEDE trial to Apr-2011

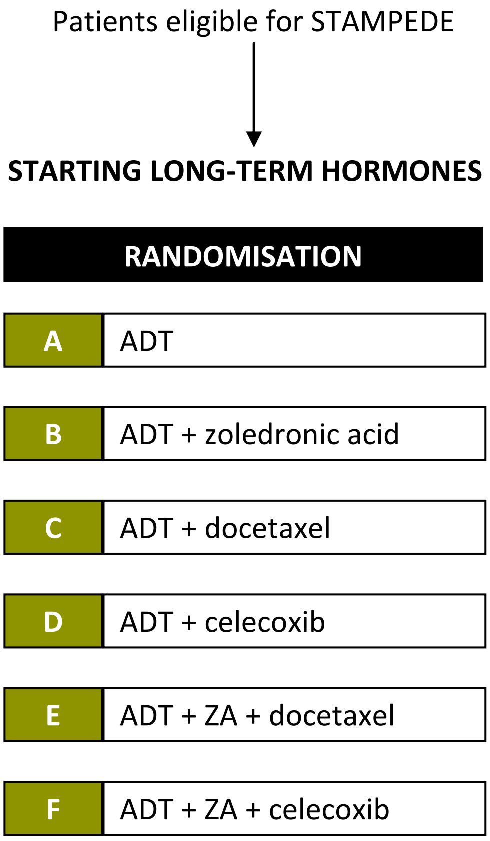

Figure 2: Recruiting arms of the STAMPEDE trial from Apr-2011 to Nov-2011 (v7.0)

Patients eligible for STAMPEDE

↓

STARTING LONG-TERM HORMONES

RANDOMISATION

**A**	ADT
**B**	ADT + zoledronic acid
**C**	ADT + docetaxel
**E**	ADT + ZA + docetaxel

Accrual stopped to celecoxib-containing arms, D and F, after their Activity Stage II analysis

Figure 3: Recruiting arms of the STAMPEDE trial from Nov-2011 to Jan-2013 (v9.0)

Patients eligible for STAMPEDE

↓

STARTING LONG-TERM HORMONES[1]

RANDOMISATION

**A**	ADT
**B**	ADT + zoledronic acid
**C**	ADT + docetaxel
**E**	ADT + ZA + docetaxel
**G**	ADT + abiraterone

[1] All suitable pts with newly diagnosed locally advanced disease should also have RT to the prostate

Accrual was initiated to the abiraterone arm, Arm G, in Nov-2011.

Figure 4: Recruiting arms of the STAMPEDE trial from protocol version 9.0 (Jan-2013 to Mar-2013)

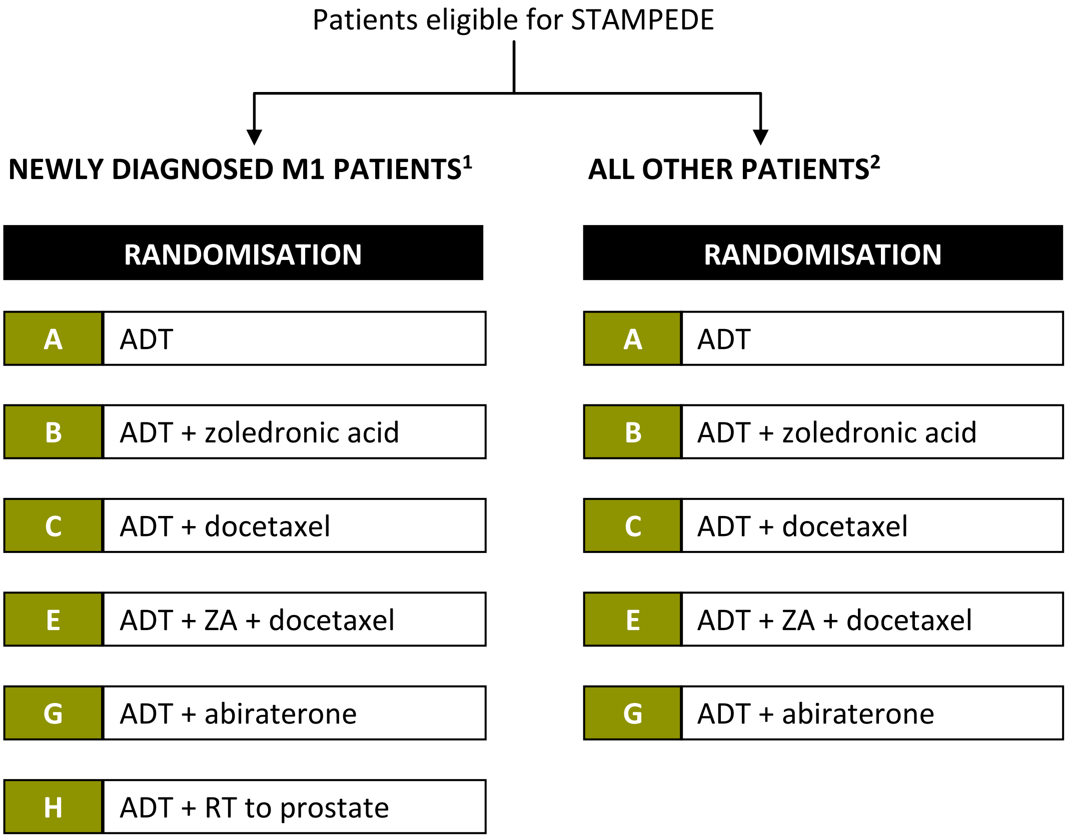

[1] Except pts with a contra-indication to RT
[2] All suitable pts with newly diagnosed locally advanced disease should also have RT to the prostate

Accrual was initiated to the radiotherapy-to-the-prostate for metastatic disease arm, Arm H, in Jan-2013.

Figure 5: Arms of the STAMPEDE Trial from protocol version 10.0 (after original research arms complete accrual)

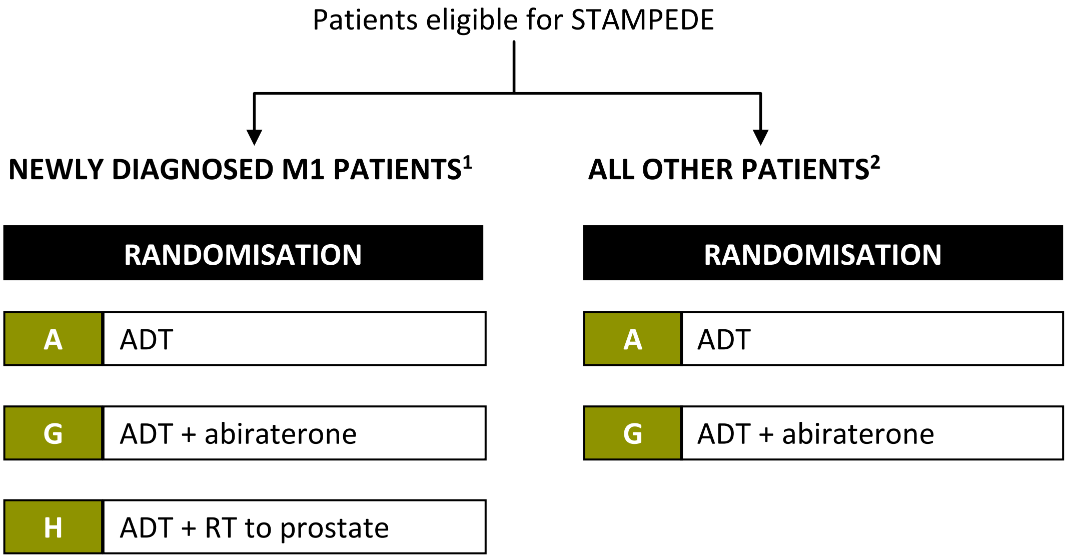

[1] Except pts with a contra-indication to RT
[2] All suitable pts with newly diagnosed locally advanced disease should also have RT to the prostate1

Accrual was completed to original research arms B, C and E in Mar-2013.

For each comparison of research arm against control, the trial will be conducted in five stages: a Pilot Phase, Activity Stages I to III and Efficacy Stage IV. The primary outcome measure of the Pilot Phase is the safety, with 30 patients recruited to each research arm. Research arms will only continue to recruitment in the next stage if they have been shown to be both safe and feasible, although patient data from all patients and all stages will be included in the final analyses. In Activity Stages I-III the primary outcome measure is failure-free survival (FFS). Further patients will be recruited until a certain number of FFS events have been observed in the control arm (see **Section 9** for further detail). Some evidence of activity will be required for a research arm to proceed to further recruitment in each stage and guidelines are in place. In Efficacy Stage IV, patients will be recruited when a certain number of primary outcome measure events have been reported. This is when around 403 deaths have been reported in the control arm for the original research comparisons and 267 (control arm deaths) for both the abiraterone comparison and the local-RT-for-M1-disease comparison. The exact number of patients and duration of the trial will depend on the observed accrual rate, observed event rate and the number of patients accruing at each stage.

Recruitment to arms D (ADT + celecoxib) and F (ADT + zoledronic acid + celecoxib) was stopped in Apr-2011 after the second planned activity analysis when the IDMC and TSC considered the lack-of-benefit guidelines.(1) Refer to **Section 9.3**. for further information regarding the guidelines for stopping accrual to research arms during the activity stages of the trial.

In version 8.0 of the protocol a new arm G (ADT + abiraterone) was added. Arm H (ADT+ prostate radiotherapy) will be added in protocol version 9.0. The trial stages remain as at trial inception but will be staggered with respect to the stages for the original arms A-F.

Patients will be assessed 6 weekly for the first 24 weeks after randomisation and then every 12 weeks up to 2 years, then 6-monthly until 5 years and annually, thereafter. The first 700 patients on

trial completed questionnaires aimed at assessing the effects of the investigational treatments on their quality of life (QL) and on their use of health care resources (Health Economics (HE) study). From protocol version 8.0, the QL and HE study has been re-opened to all new patients.

In addition, there are translational sub-studies. Patients willing to participate will be asked to donate a droplet of blood at randomisation which will be stored for either DNA and protein analysis in order to try to identify markers that are associated with response to therapy, side-effects or susceptibility to prostate cancer.

Patients will also be asked to give permission to use some of their stored material for further studies on the causes and nature of prostate cancer. In selected centres patients were asked to participate in a bone mineral density sub-study. This sub-study has now stopped recruitment. There are separate patient information sheets for the QL and HE study and the translational sub-studies (For further details of ancillary studies, see **Section 17**).

1.3 TRIAL DOCUMENTATION

Table 1 presents a summary of the required trial documentation for participating centres and **Table 2** presents a summary of the timings of the case report forms (CRFs) for your randomised patients.

Table 1: Summary of trial documentation required ahead of initial accreditation

TRIAL DOCUMENTATION	TIMING
R&D approval (including IRMER approval)	Before centre participation
Investigator Statement	Before centre participation
Signature list & delegation of responsibilities	Before centre participation
Trial personnel contact details	Before centre participation
PIS, GP & CF on local paper	Before centre participation
Signed Clinical Trial Agreement between Trust and Sponsor	Before centre participation

Table 2: Summary of trial documentation required ahead of re-accreditation (protocol v9.0 only)

TRIAL DOCUMENTATION	TIMING
R&D approval (including IRMER approval)	Before centre re-activation
PIS, GP & CF on local paper	Before centre re-activation
RTQA accreditation	Before centre re-activation

2 BACKGROUND

2.1 INTRODUCTION AND SETTING

Prostate cancer is a major health problem world-wide and accounts for nearly one fifth of all newly diagnosed male cancers. In the UK, approximately 35,000 men are diagnosed with prostate cancer each year and in 2008 almost 10,000 men died from the disease.(2)

2.1.1 LONG-TERM ANDROGEN DEPRIVATION THERAPY

The initial (first line) treatment for locally advanced or metastatic prostate cancer is androgen deprivation therapy (ADT) achieved either surgically with bilateral orchidectomy, or medically with LHRH agonists or antagonist or oral anti-androgens alone. (3) Oral anti-androgens were permitted in the trial but were used by very few patients and are no longer permitted for new patients within the trial from version 8.0.

ADT produces responses in up to 95% of patients but it is not curative and disease recurs in virtually all patients treated with ADT as sole therapy, with a median time to progression of 18-24 months. (3) Such disease is referred to as hormone or increasingly as castrate refractory prostate cancer (HRPC or CRPC).

2.1.2 ROLE OF RADIOTHERAPY FOR PATIENTS WITH M0 DISEASE

Two randomised trials, SPCG7 (4) and NCIC PR.3 / MRC PR07 (5-7) have tested the question of whether androgen deprivation therapy alone or combined with radiotherapy is the best treatment for high-risk patients with no evidence of spread outside the pelvis. Both trials demonstrate an improvement in overall and disease specific survival from the addition of radiotherapy to androgen deprivation therapy. The size of this overall survival benefit is substantial (hazard ratio 0.68 in SPCG7 and HR 0.77 in PR07). With substantial benefit demonstrated in two mature, large, well conducted randomised trials, we now recommend that radiotherapy be considered standard for patients with no nodal or metastatic spread. Patients in this category will now only be allowed to enter the trial if standard radiotherapy is planned, with the exception of those for whom radiotherapy is contra-indicated who should be discussed with the Trials Unit prior to inclusion. For patients with node positive, M0 disease there are no clear data on whether radiotherapy is or is not indicated. The NCIC PR.3 / MRC PR07 trial included patients with unknown nodal status who received whole pelvic radiotherapy. Given the large overall benefit observed in this trial, the STAMPEDE TMG recommends that pelvic nodal radiotherapy be considered for patients with node positive, non-metastatic disease at the discretion of the treating clinician.

2.2 RATIONALE

There are increasing numbers of treatments which are used post relapse of first-line androgen deprivation therapy in patients with CRPC, but little evidence as to which is associated with the best response or how they may be combined or sequenced or whether any of them might have a role in first-line treatment. Such treatments include further hormonal manipulations, bisphosphonates, (8), cytotoxic chemotherapy (9), new hormone therapies (10) and palliative radiotherapy. The traditional approach to the testing and introduction of new treatments for prostate cancer is to use them in patients with castrate refractory disease. An alternative approach is to investigate new drugs and new approaches to treatment, as first-line therapy in patients starting androgen deprivation

therapy. At this point patients should be fitter and better able to tolerate treatment than when they have CRPC and there is the possibility of having a larger and longer lasting effect.

2.3 DESIGN

STAMPEDE (also known as MRC PR08) is an innovative, multi-arm multi-stage, multi-centre, randomised controlled trial. It initially assessed the effects of a bisphosphonate (zoledronic acid), a cytotoxic chemotherapeutic agent (docetaxel) and a cyclooxygenase (Cox-2) inhibitor (celecoxib), as single agents or combinations, in patients commencing androgen deprivation therapy for advancing or metastatic prostate cancer. Each comparison is divided into five stages such that, for each investigational arm, safety and activity data are generated in the first four stages; an investigational arm will only proceed to the fifth and final stage of recruitment, where it will be assessed for its effect on overall survival, if it has been shown to be sufficiently safe and active. It is important to note, however, that patient data from all arms and all stages will be included in the final analyses of the primary outcome measure, even if the investigational arm did not proceed to the final stage.

Planned interim analysis failed to demonstrate sufficient activity for celecoxib (James, 2012 –ref to be added to list) and this agent has now been removed from the trial recruitment; patients remaining on celecoxib treatment reverted to standard care. Protocol version 8.0 added a new drug abiraterone to the study as an additional arm (see **Section 2.7**). Protocol version 9.0 adds a new comparison arm involving prostate radiotherapy for patients with metastatic disease (see **Section 2.8**). The current amendment (version 10.0) reflects the successful completion of recruitment to three docetaxel- and bisphosphonate-containing arms (Arms B, C and E) and removes references to these agents in the information sheets for new patients.

2.4 RESEARCH TREATMENT AND BIOSPHOSPHONATES

Note: recruitment stopped to the zoledronic acid and docetaxel-containing arms in Mar-2013 at the end of Activity Stage IV.

The bisphosphonates are a class of drug that act by reducing osteoclast formation, inhibiting osteoclast activity and inducing osteoclast apoptosis. They are effective at controlling hypercalcaemia and preventing skeletal complications associated with malignant disease. (11, 12) Zoledronic acid is a highly potent, third generation bisphosphonate; studies comparing the efficacy of zoledronic acid to other bisphosphonates suggest that zoledronic acid has a 40-850 fold higher potency than clodronate in preclinical models of bone resorption. (13). It has also been shown to be more effective than pamidronate (90mg) in controlling malignant hypercalcaemia. (14) In addition, zoledronic acid has also demonstrated direct anti-cancer activity, including inhibition of proliferation of breast cancer and prostate cancer cells in vitro. (15)

In randomised controlled trials of 1,648 patients, 4mg zoledronic acid was more effective than pamidronate in reducing the risk of skeletal complications in patients with bone metastases from breast cancer. (16, 17) Also, in metastatic prostate cancer, zoledronic acid has been shown to reduce the rate of skeletal-related events compared to placebo in a trial involving 429 men. (18) In April 2002, zoledronic acid received approval from the Committee for Proprietary Medicinal Products for the prevention of skeletal-related events (for example, fractures) in patients with any advanced malignancies involving bone.

The MRC PR05 prostate cancer trial showed that a first generation bisphosphonate (clodronate) commenced at the time of androgen deprivation therapy initiation, delayed time to progression in patients with bony metastatic disease and there was some evidence that it may also improve survival. (19) There is, therefore, a good rationale for investigating a more potent bisphosphonate in patients with prostate cancer who are about to commence ADT therapy.

2.5 RESEARCH TREATMENT: CHEMOTHERAPY

Note: recruitment stopped to the zoledronic acid and docetaxel-containing arms in Mar-2013 at the end of Activity Stage IV.

There is increasing evidence of the clinical efficacy of chemotherapy in prostate cancer. (9) Two randomised phase III trials in patients with metastatic hormone refractory prostate cancer (HRPC) using a docetaxel-containing regimen have been completed: the SWOG 9916 study (20) and the TAX-327 study. (21) Both studies show that the use of a docetaxel-based regimen improved survival for patients with metastatic HRPC and had significantly greater PSA response rates compared to the mitoxantrone plus prednisolone arm.

In the TAX-327 trial, (21) 1,006 patients with metastatic HRPC were randomized to receive either mitoxantrone 12 mg/m^2 with prednisone 10mg daily (Arm C) or docetaxel 75mg/m^2 3-weekly for 10 cycles with prednisone (Arm A) or docetaxel 30 mg/m^2/wk x 5 of 6 weeks x 5 cycles with prednisone (Arm B). Median overall survival was 16.5 months for patients treated with mitoxantrone versus 18.9 months for the 3-weekly docetaxel regimen (hazard ratio 0.76 (0.62-0.94)). There was also improvements for 3-weekly docetaxel in pain (22% vs 35%, p = 0.01) and PSA response (32% vs 45%, p=0.0005).

In June 2006 in the UK docetaxel was given NICE (National Institute for Health and Clinical Excellence) approval for use in hormone (now more commonly termed castrate) refractory prostate cancer patients.

2.6 RESEARCH TREATMENT: CYCLOOXYGENASE-2 INHIBITORS

Note: recruitment completed to both celecoxib-containing arms in Apr-2011 at the end of Activity Stage II of this comparison

Cyclooxygenase-2 (Cox-2) is an isoenzyme induced by a variety of mitogens, cytokines and growth factors that are associated with a range of process including inflammation, (22) and carcinogenesis.(23, 24) There is a growing body of evidence that inhibition of Cox-2 may play an important role in the prevention of cancer and the delay of progression in established cancer. A number of case-control studies have shown a reduction in risk of prostate cancer associated with the use of non-steroidal anti-inflammatory drugs (NSAID), which include inhibition of Cox-2 amongst their mode of action. (25) Pathological studies show Cox-2 is upregulated in carcinomas (26) and one study suggested that NSAID use may delay progression from subclinical to clinical prostate cancer. (27)

Celecoxib, a Cox-2 inhibitor, is better tolerated than other NSAIDs and there is evidence that it is active as a chemoprevention agent. (28) It also has important antineoplastic properties such as the ability to inhibit angiogenic factors and induce apoptosis in human cancer cells including prostate cancer. (29)

Evidence has suggested that an anti-cancer effect is only seen at higher doses of celecoxib than required for an anti-inflammatory effect. (30) Therefore, the dose of 800mg/day for STAMPEDE patients has been chosen. Although there is some high profile evidence of a small absolute increase in CVS toxicity risk associated with higher doses of celecoxib, (31) most current cancer trials are using a dose of 800mg/day as it is believed that a higher dose will result in a greater increase in cancer effect.

There is also some evidence of a schedule effect on CVS toxicity. It has been observed that CVS toxicity becomes evident after one year of taking celecoxib. (31) Therefore, a maximum duration of one year has been set for celecoxib use in this trial. Any potential risks of course have to be weighed against any potential benefits of celecoxib in the delay of progression in established prostate cancer.

Given case-control data suggesting effects on prostate cancer, pathological expression of Cox-2 in prostate cancer and in vitro data suggesting that inhibition of Cox-2 inhibits growth and invasiveness, further investigation in prostate cancer is warranted.

2.7 RESEARCH TREATMENT: STEROID SYNTHESIS INHIBITORS

Recent evidence suggests that an important mechanism for escape from tumour control by androgen ablation is the intracellular conversion of steroid precursors to androgenic steroids by prostate cancer cells. A key enzyme in this process is CYP17, which therefore represents a logical target for therapy in CRPC. (10) Abiraterone acetate is a selective inhibitor of CYP17 and is highly active in patients developing resistance to standard androgen ablation therapies. (32-34) Recruitment to a phase III study comparing abiraterone acetate to placebo in CRPC patients post-docetaxel, completed accrual in 2009 and reported initial results in 2010 with an improvement in overall survival of around 4 months and a hazard ratio of 0.65. (35) The drug has now received a marketing authorisation in the USA and in the EU from September 2011. A second trial in pre-chemotherapy CRPC patients completed recruitment April 2010; preliminary results are positive and are were published in 2012 (36) and the licence for abiraterone was extended to the pre-chemotherapy CRPC population in Europe in 2013. Side-effects with abiraterone acetate are modest with the main adverse effects being elevated transaminases (usually mild), hypokalaemia and hypertension due to secondary hyperaldosteronism (preventable by low doses of glucocorticoids) and fluid retention. In order to prevent secondary hyperaldosteronism, it is recommended that prednisolone (or prednisone) 10mg daily be administered in the CRPC setting. Within more recent studies in earlier stage patients, lower doses (typically 5mg of prednisone/prednisolone) are being used due to concerns about long-term exposure to glucocorticoid side effects. More recent evidence even suggests that for most patients, no glucocorticoids may be needed. (37) Within the STAMPEDE trial, we propose to use a prednisone/prednisolone dose of 5mg daily.

We hypothesise that the agent may be more active still when given up-front in combination with first-line androgen deprivation therapy by preventing or delaying the development of castrate refractory disease.

2.8 RESEARCH TREATMENT: RADIOTHERAPY TO THE PROSTATE FOR PATIENTS WITH NEWLY-DIAGNOSED METASTATIC DISEASE

Therapy directed against the primary tumour in the presence of metastatic disease has been evaluated rigorously in only one malignancy to date: renal cell carcinoma. Two cooperative groups ran randomised trials enrolling patients with previously untreated metastatic RCC whose primary tumours were amenable to surgical resection. Patients were randomized to receive the standard systemic therapy of the day, interferon-alpha, either alone or with radical nephrectomy. The combination of nephrectomy and interferon was shown to significantly improve median survival from 7 to 17 months in one trial(38) and from 8 to 11 months in the other.(39) The mechanism by which nephrectomy improves survival remains obscure. In preclinical models, the primary tumour has been found to secrete molecules that prime the microenvironment in which metastases can develop. An implication of this work is that therapy directed at the primary tumour, by abrogating this endocrine signalling, could retard the formation and the growth of distant metastases.

The results of two large-scale randomised trials of prostate radiotherapy are also provocative. The Scandinavian SPCG-7 trial and the MRC PR07 trial randomised men with locally advanced prostate cancer, who were at high risk of possessing occult metastatic disease, to either androgen deprivation therapy (ADT) alone or ADT plus prostate radiotherapy.(4, 40) The addition of radiotherapy dramatically improved 10-year outcomes: mortality from prostate cancer was halved. Interestingly, the benefit of radiotherapy started to emerge as early as three years from the time of randomisation. This seems improbably early if the benefit of local treatment is mediated via the prevention of subsequent disease dissemination. Rather, it is more consistent with the possibility

that local treatment has a beneficial impact on the rate of progression of existing micrometastatic disease.

We hypothesise that local therapy to the primary site may retard distant disease progression and prolong survival in patients with metastatic prostate cancer.

2.9 RESEARCH TREATMENT: COMBINATIONS

2.9.1 Bisphosphonate And Chemotherapy

Note: recruitment stopped to the zoledronic acid and docetaxel-containing arms in Mar-2013 at the end of Activity Stage IV.

Zoledronic acid and docetaxel have different mechanisms of action. In addition to its skeletal protection activity, zoledronic acid has shown direct activity against prostate cancer cells, both in vitro and in vivo. (15) There is also in vitro and in vivo evidence to suggest synergy between zoledronic acid and chemotherapy in breast cancer cells and anti-angiogenic effects in patients. (41, 42)

Toxicities of the two agents are complementary and administration in combination is expected to be feasible and safe. These aspects were evaluated in the initial Pilot Phase of the trial. Since both agents show considerable promise as single agents and there is in vitro evidence of synergy, we believe there is a strong rationale for evaluating these two agents in combination.

2.9.2 Bisphosphonate And Cyclooxygenase-2 Inhibitors

Note: recruitment stopped to both celecoxib-containing arms in Apr-2011 at the end of Activity Stage II.

An alternative approach to combination therapy is to target the principal site of relapse and a key mode of progression and this is the rationale for combining zoledronic acid with a Cox-2 inhibitor. Bisphosphonates have already been shown to delay bone disease progression in hormone refractory disease. (19) Cox-2 appears to play a crucial role in the molecular phenotype of advanced prostate cancer as outlined above, and this effect is likely to be apparent in both soft tissue and in bone. Toxicities of the two agents are likely to be complementary and there is no strong a priori reason to anticipate unacceptable toxicity. The Pilot Phase of the trial will evaluate tolerability and safety of the combination. Targeting both bone progression and the underlying molecular changes leading to progression can be expected to have synergistic benefits in terms of delaying development of hormone refractory disease.

Figure 6: Use of RT in STAMPEDE

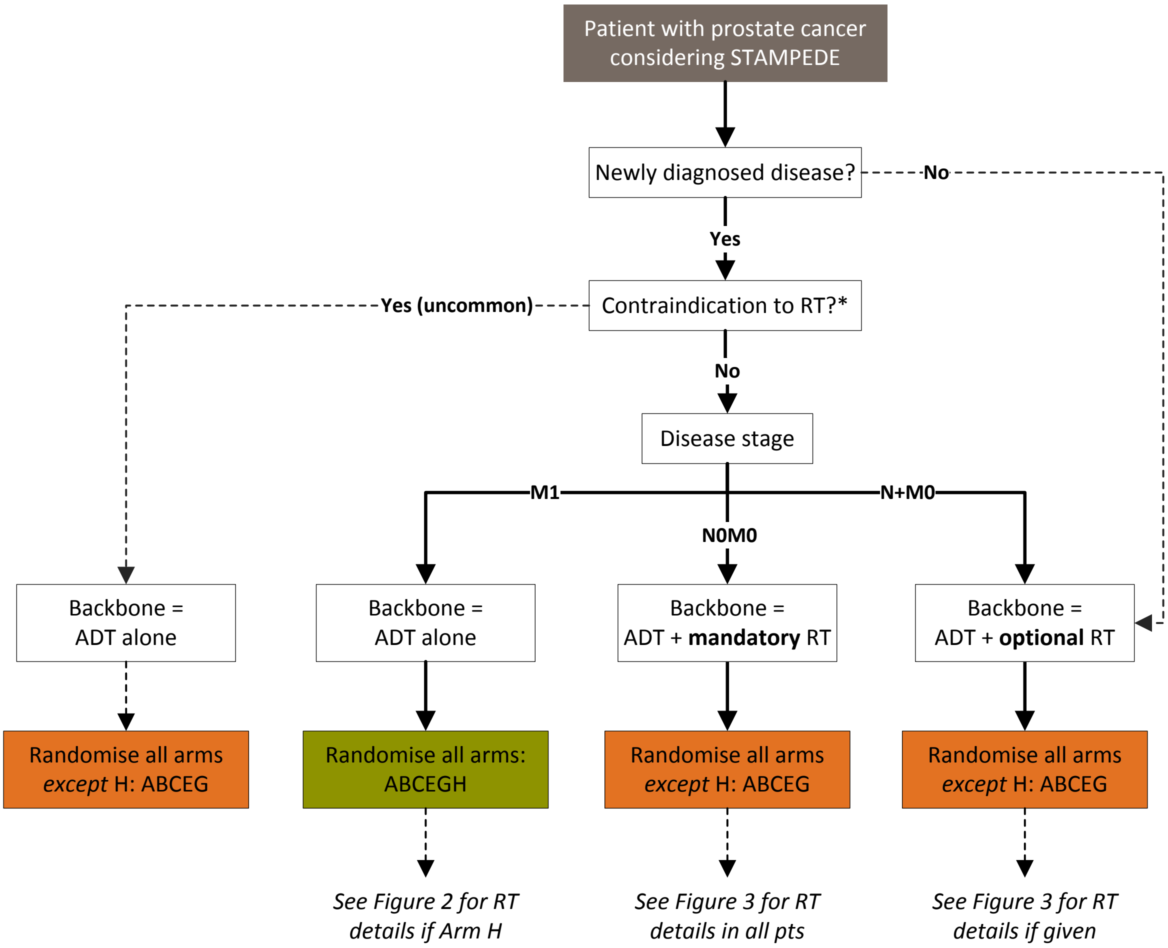

*It is expected that only around 1% of patients will have a contraindication to RT e.g. inflammatory bowel disease. These cases should be discussed with the trials unit prior to randomisation (see Section 2.7).

3 SELECTION OF INSTITUTIONS AND INVESTIGATORS

Centres who wish to participate in the STAMPEDE trial should be registered with the Medical Research Council Clinical Trials Unit (MRC CTU) for this purpose. Before any patients are randomised the MRC CTU must receive a completed and signed Investigator Statement. The STAMPEDE investigator statement is signed by the Principal Investigator for that institution (**Appendix M**). R&D approval for the site, along with a fully-signed model agreement are also required before recruitment can begin.

In addition and in compliance with the principles of GCP all institutions participating in the trial will complete a delegation log and forward this to the MRC CTU. Each person working on the STAMPEDE trial must sign off a section of this log indicating their responsibilities. The MRC CTU must be notified of any changes to trial personnel and/or their responsibilities. An up-to-date copy of this log must be stored in the Investigator Site file at the institution and also at the MRC CTU.

The Clinical Trial Authorisation (CTA) for the STAMPEDE trial requires that the Medicines and Healthcare Products Regulatory Agency (MHRA) be supplied with the names and addresses of all participating investigators/institutions. Trial staff at the MRC CTU will perform this task; hence, it is vital to receive full contact details for all investigators prior to their entering patients.

Finally, before a patient is entered into the trial written informed consent must be obtained. Approved patient information sheets and informed consent forms are supplied as templates.

Only a limited number of centres participated in the initial Pilot Phase of the original trial; this was to ensure that safety and feasibility data were collected expediently. Subsequent stages of the trial are open to any centre that wishes to participate and has fulfilled the requirements described above.

3.1 RADIOTHERAPY ACCREDITATION

The introduction of the RT comparison in v9.0 introduces the need for RTQA accreditation in sites giving radiotherapy. The details of RTQA accreditation is in **Appendix K**. However, centres that have been RTQA accredited for another multi-centre prostate radiotherapy trial in the UK (e.g. MRC RT01, RADICALS or CHHIP) will be automatically granted STAMPEDE RTQA accreditation.

4 SELECTION OF PATIENTS

4.1 PATIENT INCLUSION CRITERIA

Patients must fulfil both of the criteria in **Section 4.1.1** **or** one criterion in **Section 4.1.2** **or** at least one criteria in **Section 4.1.3**. Additionally, all patients must fulfil the criteria in **Section 4.1.4**.

4.1.1 High-Risk Newly Diagnosed Non-Metastatic Node-Negative Disease

Both:

- At least two of: Stage T3/4, PSA≥40ng/ml or Gleason sum score 8-10
- Intention to treat with radical radiotherapy (unless there is a contra-indication; exemption can sought in advance of consent, after discussion with MRC CTU)

OR

4.1.2 Newly Diagnosed Metastatic Or Node-Positive Disease

At least one of:

- Stage T_{any} N+ M0
- Stage T_{any} N_{any} M+

OR

4.1.3 Previously Treated With Radical Surgery And/Or Radiotherapy, Now Relapsing[1]

At least one of:

- PSA ≥4ng/ml and rising with doubling time less than 6 months
- PSA ≥20ng/ml
- N+
- M+

AND

4.1.4 For All Patients

I. Histologically confirmed prostate adenocarcinoma
II. Intention to treat with long-term androgen deprivation therapy
III. Fit for all protocol treatment[2] and follow-up, WHO performance status 0-2[3]
IV. Have completed the appropriate investigations prior to randomisation
V. Adequate haematological function: neutrophil count >$1.5x10^9$/l and platelets >$100x10^9$/l
VI. Estimated creatinine clearance >30ml/min
VII. Serum potassium ≥3.5mmol/L
VIII. Written informed consent
IX. Willing and expected to comply with follow-up schedule
X. Using effective contraceptive method if applicable

[1] Courses of hormone therapy for localised disease must have been completed at least 12 months previously and have been no longer than 12 months in duration. It can have been given as adjuvant or neoadjuvant therapy.
[2] Medical contraindications to the trial medications are given in **Appendix G**
[3] For WHO performance status definitions see **Appendix A**

4.2 PATIENT EXCLUSION CRITERIA[4]

Patients must not fulfil any of the criteria, below.

I. Prior systemic therapy for locally advanced or metastatic prostate cancer except as listed in **Section 4.1.3**.
II. Metastatic brain disease or leptomeningeal disease
III. Abnormal liver functions consisting of any of the following:
   - Serum bilirubin ≥1.5 x ULN (except for patients with Gilbert's disease, for whom the upper limit of serum bilirubin is 51.3µmol/l or 3mg/dl)
   - Aspartate aminotransferase (AST) or alanine aminotransferase (ALT) ≥2.5 x ULN
IV. Any other previous or current malignant disease which, in the judgement of the responsible physician, is likely to interfere with STAMPEDE treatment or assessment
V. Patients with active peptic ulceration, gastrointestinal bleeding, inflammatory bowel disease
VI. Symptomatic peripheral neuropathy grade ≥2 (NCI CTC)[5]
VII. Any surgery (e.g. TURP) performed within the past 4 weeks
VIII. Patients with significant cardiovascular disease such that, in the investigator's opinion, the patient is unfit for any of the study treatments. This might include:
   - Severe/unstable angina
   - Myocardial infarction less than 6 months prior to randomisation
   - Arterial thrombotic events less than 6 months prior to randomisation
   - Clinically significant cardiac failure requiring treatment (NYHA II-IV)6
   - Cerebrovascular disease (e.g. stroke or transient ischaemic episode) less than 2 years prior to randomisation
   - Patients with uncontrolled hypertension defined as systolic BP greater or equal than 160 mmHg or diastolic BP greater or equal than 95 mmHg
IX. Patients who have been scheduled to have major dental extractions within the next 2 years
X. Patients receiving treatment with drugs known to induce CYP3A4 (including phenytoin, carbamazepine, Phenobarbital)[7]
XI. Prior exposure to abiraterone
XII. Prior chemotherapy for prostate cancer
XIII. Prior therapy with zoledronic acid other than short-term treatment for hypercalcaemia
XIV. Prior exposure to policy of long-term hormone therapy before randomisation (unless as described in **Section 4.4.2**)

[4] The exclusion criteria for patients who have been on a Cox-2-inhibitor for 6+ months has been removed
[5] See **Appendix I** for common toxicity grading
[6] NYHA classifications can be found in **Appendix A**
[7] A full list is included in **Appendix G**

4.3 SELECTION CRITERIA FOR COMPARISON OF RESEARCH (M1) RT FOR METASTATIC DISEASE

All patients meeting criteria in Section 4.1 and 4.2 are eligible for the trial, but not all can be allocated to the research (M1) radiotherapy arm. The selection criteria for this "RT to the prostate" comparison are:

- Newly diagnosed prostate cancer
- Demonstrable M1 disease
- No contraindication to radiotherapy e.g. no previous pelvic radiotherapy,
- No previous radical prostatectomy

Patients meeting these criteria will have a chance to be allocated to Arms A and H.

4.4 SCREENING PROCEDURES

4.4.1 Investigations Prior To Randomisation

All patients should have the following examinations performed. The latest available scans should be used:

- CT or MRI of pelvis and abdomen
- Bone Scan (or equivalent e.g. whole body MRI)
- Chest X-ray (only if chest was not included in CT)
- ECG
- PSA Test

The following blood tests within 8 weeks (56 days) prior to randomisation:

- Testosterone (if available)
- Urea and Electrolytes
- Liver function tests
- Serum creatinine
- Serum corrected calcium
- Phosphates
- Magnesium
- Albumin
- Total cholesterol
- HDL cholesterol
- Systolic blood pressure
- Diastolic blood pressure
- Waist circumference measure

Patients who initially fail to meet the eligibility criteria can be re-screened at a later date.

Prior to randomisation:

- Check details of any prior treatments for prostate cancer
- Check any contraindications to radiotherapy

4.4.2 Androgen Deprivation Therapy Prior To Randomisation

It is preferable that patients are not started on hormones prior to randomisation. However, if androgen deprivation therapy has already started, the primary therapy should have not have started

more than 12 weeks before randomisation, and the baseline PSA measurement must be taken before this was initiated (please report the latest PSA measurement taken before the start of androgen deprivation therapy).

Short periods (not exceeding 2 weeks duration) of prior anti-androgens to cover tumour flare are allowed but will not be counted in the 12 week time period mentioned above; but a PSA measurement must be taken before this is initiated.

Note that long-term anti-androgen monotherapy is not permitted in the trial for newly recruited patients from version 8.0 (see **Section 6.1**); patients may change treatment to join the trial, provided that they have not had more than 12 weeks of androgen deprivation therapy prior to randomisation. Further details on hormone therapies allowed prior to randomisation are discussed in **Appendix L**.

Relapsing patients previously treated with radical surgery or radiotherapy must have completed a policy of hormone therapy at least 12 months previously and have been no longer than 12 months in duration, given as adjuvant or neo-adjuvant therapy.

Note that baseline testosterone measurements will not be required in patients who have already commenced hormone manipulation prior to randomisation.

4.4.3 Hypercalcaemia At Randomisation

For patients who are hypercalcaemic prior to randomisation and require treatment, it is recommended that they are treated with a bisphosphonate and that the treatment should be discontinued when they are stabilised.

4.4.4 NSAIDs And Cox-2 Inhibitors At Randomisation

Note: recruitment completed to both celecoxib-containing arms in Apr-2011 at the end of Activity Stage II

For patients who are currently on a Cox-2-inhibitor and who meet the inclusion criteria, please ensure that treatment is discontinued before randomisation. If the patient is allocated to an arm, which does not include celecoxib (arms A, B, C or E), it is advised that the Cox-2 be replaced with a suitable NSAID.

For patients who are taking an NSAID prior to randomisation and are allocated a celecoxib arm (Arm D or F), a clinical decision should be taken as to whether the patient should continue taking the NSAID alongside the celecoxib. This decision should take into account the risk of gastrointestinal problems, and consideration should be given to the co-administration of a proton pump inhibitor

4.4.5 Starting Trial Treatment

Trial treatment should be commenced as soon as possible after randomisation. Investigators should aim that this is at least within 4 weeks post randomisation and within 12 weeks of starting androgen Deprivation Therapy (see **Section 6**).

Radiotherapy for patients allocated to Arm H should be commenced within 4 weeks from randomisations and continued according to the predefined scheduled unless toxicity is reported. Any delays in starting research radiotherapy should be discussed with the STAMPEDE team and recorded as appropriate in the relevant CRF.

4.4.6 Concomitant Medications

All concomitant medications should be recorded including any vitamin and mineral supplements the patient is taking, regular consumption of NSAID and/or aspirin and use of other bisphosphonates (see **Section 4.3.1**). Of particular interest in this regards, are herbal preparations such as PC-SPES,

Prostasol, Saw Palmetto and St John's Wort. All concomitant medications should be continued throughout the trial unless the responsible clinician decides otherwise.

4.5 ADDITIONAL DETAILS FOR PATIENTS JOINING SUB-STUDIES

An additional droplet of blood must be taken if the patient has given their consent to participate in the DNA analysis sub-study.

The local pathologist will also be asked to give the remaining tumour sample for tissue micro array analysis to be carried out, if the patient has given consent for his remaining samples to be used for further analyses. Full details of all sub-studies and instructions relating to the handling of the blood sample are given in **Section 17** and **Appendix D**.

5 RANDOMISATION AND ENROLMENT

Patients will be allocated to any of the open research arms for which they are suitable. Patients with non-metastatic disease or who have had previous local therapy to the prostate or who have a contraindication to radiotherapy will not be allocated to Arm H (see **Section 4.3**).

To enter a patient the randomisation form should be completed carefully and the MRC CTU contacted by phone:

RANDOMISATIONS
To randomise, call MRC CTU, Monday to Friday 0900-1700
excluding public holidays or dates when notice has been given by the CTU.
Tel: +44 (0) 20 7670 4777

A trial number and treatment will be allocated and given over the phone or by return fax. In addition, a letter confirming these details will be sent. The trial number will be the primary way in which the patient will be identified and should be used in all correspondence.

5.1 CO-ENROLMENT GUIDELINES

Ideally, patients should not be participating in any other clinical trial of prostate cancer treatment when they enter STAMPEDE and should not enter any other trials until the patient has had a failure-free survival (FFS) event reported. After this point, the patient may be entered into further, second-line treatment studies. The primary outcome measure of STAMPEDE is overall survival. Participation in post-progression studies should be reported on the Co-enrolment CRF.

Data sharing agreements with “down-stream” trials are encouraged to improve data quality in both trials and to reduce costs to both organisations.

6 TREATMENT OF PATIENTS

6.1 TRIAL TREATMENT

Patients will be randomised to the control arm (Arm A) or one of the research arms. All patients will receive androgen deprivation therapy (ADT) to achieve castration levels of testosterone. The method of ADT is a local choice but must be specified for each patient prior to randomisation. The recommended methods of ADT are given in Section 6.1.1. All trial treatments should commence as soon as practically possible after randomisation. Patients having a bilateral orchidectomy should commence any additional treatment with 4 weeks of the operation unless there is a strong clinical reason not to do so. Note that from protocol version 8.0 onwards, bicalutamide monotherapy is no longer a permitted as a trial therapy for new patients (but patients may switch to a permitted therapy to join the trial – see Section 4.3.2).

6.1.1 Arm A: ADT Alone Or ADT + Standard-of-Care (M0) RT (Control Arm)

The standard of care for this patient group is **androgen deprivation therapy** (see Section 6.1.1.1). For some patient groups, this should now be supplemented with standard radiotherapy (see Section 6.1.1.2).

6.1.1.A Hormone Therapy

The recommended methods of ADT are bilateral orchidectomy, LHRH analogues and LHRH antagonists. Anti-androgens alone are not permissible as hormone therapy for patients participating in STAMPEDE, but their use is recommended in the short-term to prevent tumour “flare” which may occur after commencing LHRH analogues. Anti-androgen prophylaxis of tumour flare is not required when using LHRH antagonists. At the time of randomisation, centres will be asked to specify the method of ADT for each patient. Other methods of ADT should be discussed with the Chief Investigator or the Trial Surgeon. The planned duration of ADT should be at least 2 years.

Bilateral orchidectomy: Operations should be performed by appropriately trained surgeons. A total or subcapsular orchidectomy may be performed.

LHRH agonists: LHRH agonists/analogues used according to local practice. The prophylactic use of anti-androgens to prevent tumour “flare” is recommended.

LHRH antagonists: LHRH antagonists used according to local practice. The use of prophylactic use of anti-androgens to prevent tumour “flare” is not necessary.

6.1.1.B Standard-of-care (M0) RT

N0M0 patients: Investigators should give standard radiotherapy (RT) to patients with node negative, non-metastatic disease (N0M0), in accordance with the recent data from the PR07 and SPCG trials. If there is an intention to omit radiotherapy (e.g contraindications) in patients with N0M0 disease this must be discussed with the Trials Office before consent. See Section 6.6 for further details of radiotherapy administration.

N+M0 patients: the benefit of radiotherapy in this group is at present uncertain with no firm data to either support or refute its use. However, the PR07 trial included some node positive patients as cross sectional imaging was not a part of the baseline assessment in this trial, which did include whole pelvis radiotherapy. For patients with node positive, non-metastatic disease, radiotherapy is

therefore recommended in suitable cases. Investigators will be asked to state their intention with regard to planned radiotherapy in this group at randomisation. Intention to give radiotherapy (or not) for node positive patients must be stated at randomisation to ensure that there is no bias towards particular combinations of systemic therapy with radiotherapy.

Standard radiotherapy is not a core part of the trial, therefore we intend to collect minimal data about the radiotherapy administered. It is accepted that some patients will develop progressive disease before radiotherapy can be administered and if this occurs the reasons for non-delivery of treatment must be recorded on the radiotherapy form.

6.1.2 Arm B: ADT + Zoledronic Acid

Note: recruitment stopped to the zoledronic acid and docetaxel-containing arms in Mar-2013 at the end of Activity Stage IV.

Androgen deprivation therapy (+/- standard-of-care M0 RT) as described in Section 6.1.1.

Zoledronic Acid: 4mg 15min IV infusion every 3 weeks, for 6 treatments followed by zoledronic acid 4mg 15min IV infusion every 4 weeks up to a maximum of 2 years from the start of the treatment or until disease (including PSA) progression (see Section 7.2). Patients should also receive an oral supplement of 500mg calcium and 400IU vitamin D daily. These doses are available as a combination tablet. See Section 6.2.1 for further information.

6.1.3 Arm C: ADT + Docetaxel

Note: recruitment stopped to the zoledronic acid and docetaxel-containing arms in Mar-2013 at the end of Activity Stage IV.

Androgen deprivation therapy (+/-standard-of-care M0 RT) as described in Section 6.1.1.

Docetaxel: 75mg/m2 Day 1 as 1hr IV infusion, plus prednisolone 5mg bid daily for 21 days. The cycle should be repeated every 3 weeks for a maximum of 6 cycles. The recommended administration schedule, anti-emetic regimen and dose modifications for docetaxel are given in Appendix F. See Section 6.2.2 for further information.

6.1.4 Arm D: ADT + Celecoxib

Note: recruitment completed to both celecoxib-containing arms in Apr-2011 at the end of its Activity Stage II

Androgen deprivation therapy as described in Section 6.1.1.

Celecoxib 400mg bid until the sooner of 1 year or disease (including PSA) progression (see Section 7.2). See Section 6.2.3 for further information.

6.1.5 Arm E: ADT + Docetaxel + Zoledronic Acid

Note: recruitment stopped to the zoledronic acid and docetaxel-containing arms in Mar-2013 at the end of Activity Stage IV.

Androgen deprivation therapy (+/- standard-of-care M0 RT) as described in Section 6.1.1.

Docetaxel: 75mg/m2 Day 1 as 1hr IV infusion, plus prednisolone 5mg bid daily for 21 days. The cycle should be repeated every 3 weeks for a maximum of 6 cycles. The recommended administration schedule, anti-emetic regimen and dose modifications for docetaxel are given in Appendix F. See Section 6.2.2 for further information.

Zoledronic Acid: 4mg 15min IV infusion every 3 weeks, for 6 treatments followed by zoledronic acid 4mg 15min IV infusion every 4 weeks up to a maximum of 2 years from the start of the treatment or until disease (including PSA) progression (see Section 7.2). Patients should also receive an oral supplement of 500mg calcium and 400IU vitamin D daily. These doses are available as a combination tablet. See Section 6.2.1 for further information.

Co-administration of docetaxel and zoledronic acid: Docetaxel 75mg/m2 Day 1 as 1hr IV infusion, plus prednisolone 5mg bid daily followed by zoledronic acid 4mg 15min IV infusion. There is evidence to suggest that the co-administration of docetaxel and zoledronic acid is sequence dependent (39). Consequently, docetaxel should be administered before zoledronic acid

6.1.6 Arm F: ADT + Zoledronic Acid + Celecoxib

Note: recruitment completed to both celecoxib-containing arms in Apr-2011 at the end of Activity Stage II

Androgen deprivation therapy as described in Section 6.1.1.

Zoledronic Acid 4mg 15min IV infusion every 3 weeks, for 6 treatments followed by zoledronic acid 4mg 15min IV infusion every 4 weeks up to a maximum of 2 years from the start of the treatment or until disease (including PSA) progression (see Section 7.2). Patients should also receive an oral supplement of 500mg calcium and 400IU vitamin D daily (Calcichew). These doses are available as a combination tablet. See Section 6.2.1 for further information.

Celecoxib 400mg bid until the sooner of 1 year or disease (including PSA) progression (see Section 7.2). See Section 6.2.3 for further information.

6.1.7 Arm G: ADT + Abiraterone

Androgen deprivation therapy (+/- standard-of-care M0 RT) as described in **Section 6.1.1**.

Abiraterone will be administered as a single 1000mg daily oral dose (4 tablets to be taken together once a day) together with prednisolone or prednisone 5mg daily to prevent secondary ACTH excess.

In patients with M1 disease, treatment with abiraterone will continue from randomisation until clinical disease progression, consistent with the COU-AA-301 trial (35) i.e., abiraterone would be given for these patients until a composite of PSA progression (as defined in **Appendix J**), radiological progression (appearance of new lesions or progression of existing lesions) and clinical progression (defined as new cancer-related symptoms). It is accepted that these flexible criteria for stopping treatment with abiraterone are open to the investigator's interpretation and discretion. Patients might continue treatment beyond the first failure-free survival (FFS) event (see **Table 1** in **Section 9.2**); the first FFS event must be reported as per the other arms.

In patients with N0M0 disease or N+M0 disease undergoing radical radiotherapy, treatment would continue for 2 years or disease progression as defined for M1 patients, whichever is the sooner. ADT can be discontinued in this group at 2 years at the discretion of the local investigator (see **Section 6.1.1.A**).

For patients with N+M0 disease not planned for radical radiotherapy, treatment will continue as for patients with M1 disease until disease progression.

See **Section 6.2.4** for further information for all groups.

6.1.8 ARM H: ADT + PROSTATE RADIOTHERAPY IN M1 PATIENTS

Androgen deprivation therapy as described in **Section 6.1.1**.

Radiotherapy will commence as soon as practicable and within eight weeks after randomization. Treatment will be according to the guidelines in **Section 6.2.5**. Two radiotherapy dose-fractionation schedules are permitted:

- 36Gy in 6 fractions of 6Gy, administered weekly over 6 consecutive weeks
- 55Gy in 20 fractions of 2.75Gy, administered daily, five days per week, over 4 consecutive weeks

Details of the recommendations for outlining, CTV and PTV are in **Section 6.2.5**.

6.2 ADMINISTRATION AND DOSE MODIFICATIONS

6.2.1 ZOLEDRONIC ACID

Note: recruitment stopped to the zoledronic acid and docetaxel-containing arms in Mar-2013 at the end of Activity Stage IV.

Zoledronic acid will be administered by IV infusion in accordance with the instructions in the summary of product characteristics at a target dose of 4mg (adjusted for renal function, see below) every 3 weeks for the first 6 cycles and every 4 weeks, thereafter.

Serum Creatinine Measurements: Serum creatinine should be measured at baseline and within 48 hours prior to every administration of zoledronic acid. It is permissible to have serum creatinine levels measured on Fridays prior to the administration of zoledronic acid on the following Monday.

Serum Electrolytes and FBC: Serum electrolytes including calcium, phosphate and magnesium should also be measured prior to each infusion. FBC should be measured at least 3 monthly. Zoledronic acid should be discontinued if there is any evidence of hypersensitivity to the drug. In patients with mild to moderate renal impairment, lower doses of zoledronic acid are recommended according to standard dose reduction schedules for administration of this drug. In rare cases, zoledronic acid treatment has been associated with the development of osteonecrosis of the jaw, particularly following dental extractions. If a patient develops osteonecrosis of the jaw then the zoledronic acid should be immediately and permanently discontinued. For full details of zoledronic acid administration and does reductions see Appendix F. Contraindications, special precautions, interactions and side effects are listed in Appendix G.

6.2.2 DOCETAXEL

Note: recruitment stopped to the zoledronic acid and docetaxel-containing arms in Mar-2013 at the end of Activity Stage IV.

The use of docetaxel should be confined to units specialised in the administration of cytotoxic chemotherapy and it should only be administered under the supervision of a physician qualified in the use of anticancer chemotherapy.

Docetaxel will be administered by IV infusion in accordance with the instructions in the summary of product characteristics at a dose of 75mg/m2 (up to a maximum dose of 160mg) on Day 1 of the study treatment

period and then every 3 weeks thereafter for a maximum of 6 doses. Patients with a body surface area (BSA) greater than 2.13m2 should be dosed as though they have a BSA of 2.13m2. No ideal weight should be used for BSA calculations. Prednisolone or prednisone 5mg bid will be given until completion of chemotherapy. Additional dexamethasone should be given pre- and post-docetaxel infusion to suppress allergic reactions.

Please note that liver function test (LFTs) should be carried out within a week before the first cycle of docetaxel if an anti-androgen has been administered. This is due to an increased risk of neutropenia associated with docetaxel use following anti-androgen administration. Treatment should be delayed if LFTs are abnormal.

For full details of premedication schedule, recommended anti-emetic regimen and dose modifications for docetaxel see Appendix F. Contraindications, special precautions, interactions and side effects are listed in Appendix G.

Docetaxel in combination with prednisone or prednisolone is indicated for the treatment of patients with hormone refractory metastatic prostate cancer. (20, 21)

6.2.3 Celecoxib

Note: recruitment completed to both celecoxib-containing arms in Apr-2011 at the end of Activity Stage II. No new patients should be receiving this agent now within the trial.

Celecoxib should be administered in accordance with the instructions in the summary of product characteristics at a dose of 400mg bid orally. Rarely this drug is poorly tolerated and in this instance should be discontinued; particular care should be taken with patients with a history of gastrointestinal disease and patients with significant risk factors for cardiovascular events (see Appendix G). Patients with confirmed severe cardiovascular history should not be in STAMPEDE (see exclusion criteria, Section 4.2). Contraindications, special precautions, interactions and side effects are listed in Appendix G. Dose reductions are not anticipated.

6.2.4 Abiraterone

Abiraterone absorption is increased by food. The tablets should be taken at least 2 hours after food, swallowed whole with some water. No food should be eaten for 1 hour afterwards. Prednisolone (prednisone in Switzerland) should be taken as a single dose with food in the morning.

If clinical symptoms or signs suggestive of hepatotoxicity develop, serum transaminases, in particular serum alanine aminotransferase (ALT), should be measured immediately. If a rise in transaminases or bilirubin is confirmed, action should be taken as detailed in **Appendix G**.

6.2.4.A Steroid Dose Modifications

Prednisolone or prednisone will be started at 5mg once daily, to prevent secondary mineralocorticoid excess. Prednisone/prednisolone dose increase of up to 10mg/day is permitted to manage mineralocorticoid-related toxicities (e.g., hypokalaemia, hypertension) which are refractory to standard management. Patients experiencing serious Cushing symptoms (e.g., weight gain, muscle loss) can decrease or discontinue (temporarily or permanently) steroids at the investigator's discretion. It should be noted that weight gain and muscle loss are also associated with androgen deprivation therapy.

6.2.5 Research (M1) Prostate Radiotherapy

A treatment planning CT scan will be acquired with the patient supine, with empty rectum and comfortably full bladder.

Megavoltage equipment is required with effective photon energies ≥6MV. Minimum source-to-axis distance is 100cm. Field arrangement is at the clinician's discretion: acceptable treatment techniques (field arrangement) include a 3-field (anterior, right lateral, and left lateral), 4-field (anterior, posterior, right lateral, and left lateral), or 6-field (right and left anterior oblique, right and left posterior oblique, and right and left lateral) or equivalent coplanar technique with multi-leaf collimation for all fields to adequately protect normal structures.

The Clinical Target Volume (CTV) will consist of the prostate gland alone as visualized on the treatment-planning CT scan. The base of the seminal vesicles may also be included if they are macroscopically involved. Inclusion of pelvic lymph nodes in the CTV is not permitted. The Planning Target Volume will have a 0.8 cm margin posteriorly and 1.0 cm margin in all other directions around the CTV to account for prostate gland motion and uncertainty in daily treatment setup.

Critical normal tissues should be delineated on the treatment-planning CT scan by the treating clinician:

- Rectum – inferior limit: level of ischial tuberosities; superior limit: sigmoid flexure
- Bladder – entirety

Two radiotherapy dose-fractionation schedules are permitted. In either case, radiotherapy is prescribed such that at least 95% of the PTV receives the prescribed dose:

- 36Gy in 6 fractions of 6Gy, administered weekly over 6 consecutive weeks
- 55Gy in 20 fractions of 2.75Gy, administered daily, five days per week, over 4 consecutive weeks

Dose-volume objectives for each dose-fractionation schedule are shown in **Tables 3** and **4** below. Values have been calculated using the formula BED = D[1+d/(alpha-beta ratio)] assuming an alpha-beta ratio of 3 for rectum and bladder. These are provided for guidance only.

Table 3: Rectal dose volume objectives

55GY/20F	36GY/6F	MAX VOL (%)
52.5 Gy	33.3 Gy	50%
43.5 Gy	27.8 Gy	60%
26.1 Gy	16.7 Gy	80%

Table 4: Bladder dose-volume objectives

55GY/20F	36GY/6F	MAX VOL (%)
52.2	33.3	50%
43.5	27.8	25%

Portal imaging to verify accuracy of treatment delivery may be done according to the participating centre's local guidelines. Image-guidance technology (e.g., gold seed intraprostatic fiducial markers, cone-beam CT scanning) will be permitted according to clinician preference but is not required. Further illustration on the research radiotherapy arm schedule is shown in **Figure 7**.

Figure 7: Diagram for deciding approach to research (M1) RT

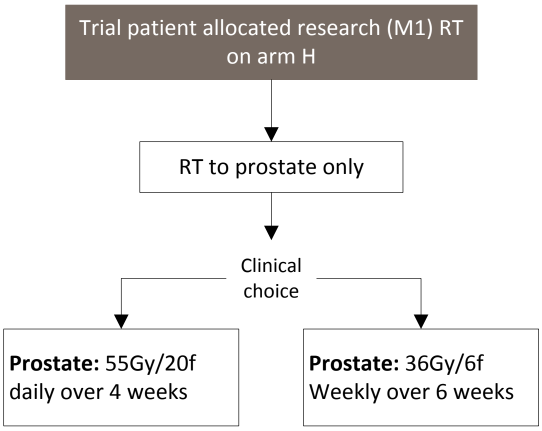

6.3 TRIAL PRODUCTS

Details of the procedures for obtaining the drugs within the trial, dispensing and disposal of unused drug are given in **Appendix E**.

Arrangements for free or discounted drugs are given in the Finance section (**Section 15**).

6.4 MEASURES OF COMPLIANCE/ADHERENCE

Date of treatment, dose, delays and reasons for delays or dose modifications of all study infusions (zoledronic acid and docetaxel) will be recorded. The estimated number of abiraterone tablets taken in a given time period will also be recorded as well as any dose reductions.

6.5 TREATMENT DATA COLLECTION

Data will be recorded on case report forms (CRFs); the top copy/original should be sent to the MRC CTU for data entry and a copy kept at the local centre. Up-to-date versions of all CRFs can be found on the trial website (http://www.stampedetrial.org/) and centres will be notified of any changes throughout the course of the trial The type of data to be recorded is detailed in the Assessments and Procedures section (**Section 7**).

6.6 ADMINISTRATION OF STANDARD RADIOTHERAPY[8] TO NON-METASTATIC PATIENTS

6.6.1 TREATMENT DETAILS

Standard radiotherapy will be given to appropriate patients in each of the trial arms, following a period of neo-adjuvant ADT therapy, as is generally standard in UK practice. For patients receiving

[8] **Note:** this text has been transferred into the protocol from the Appendices in version 8.0, and updated

docetaxel, this period needs to be a minimum of 6 months after randomisation to ensure that chemotherapy is completed and toxicity resolved before RT begins. To ensure consistency of timing of administration of standard radiotherapy in all arms, this same 6 months period is recommended for all patients. For patients with negative nodes on axial imaging, clinicians may choose between irradiating prostate and seminal vesicles alone or including the pelvic nodes in addition. Additional staging tests such as pelvic node sampling may be considered in making this decision. Conformal or intensity modulated radiotherapy should be used in all patients. Where patients have good clinical evidence that nodes are free of tumour or patients for whom nodal radiotherapy is contra-indicated (e.g. significant bowel disease), treatment may be given to the prostate gland and seminal vesicles only. The recommended dose is 74Gy in 37 fractions to the prostate and seminal vesicles or the equivalent using hypofractionated schedules. These recommendations are summarised **Figure 3**. Alternative dosing schedules are permitted but must be agreed with the STAMPEDE Trial Management Group.

6.6.1.A Standard-of-care RT Timing in M0 patients

Radiotherapy should be given around 6 to 9 months after randomisation in all trial arms and, if receiving docetaxel, the patient must have recovered from any docetaxel toxicity before RT can begin.

6.6.1.B Type Of standard-of-care RT in M0 patients

Conformal or intensity modulated radiotherapy.

6.6.1.C Standard Clinical Target Volume in M0 patients

- **CTV1:** Prostate plus seminal vesicles
- **CTV2:** Regional lymph nodes to include internal iliac and the inferior part of the common iliac nodes as used in EORTC trial 22961 (43)
- **PTV1:** CTV1 plus 10-15 mm according to local practice
- **PTV2:** CTV2 plus 10-20mm according to local practice

6.6.1.D Standard-of-care RT Dose in M0 patients

Prostate dose of 74Gy in 2Gy fractions or equivalent, with optional dose to the pelvic nodes of 46-50Gy in 2Gy fractions or equivalent using IMRT to deliver the treatment over 37 fractions, suggested dose is 55Gy in 37 fractions with IMRT. Higher doses may be considered if the department is experienced in using IMRT for nodal radiotherapy, particularly as data emerges from the PIVOTAL trial of nodal IMRT in high-risk node negative patients where a nodal dose of 60Gy in 37 fractions is being evaluated. Alternative schedules should be agreed with the STAMPEDE Trial Management Group.

Figure 8: Diagram for deciding recommended approach to standard-of-care (M0) RT in non-metastatic patients

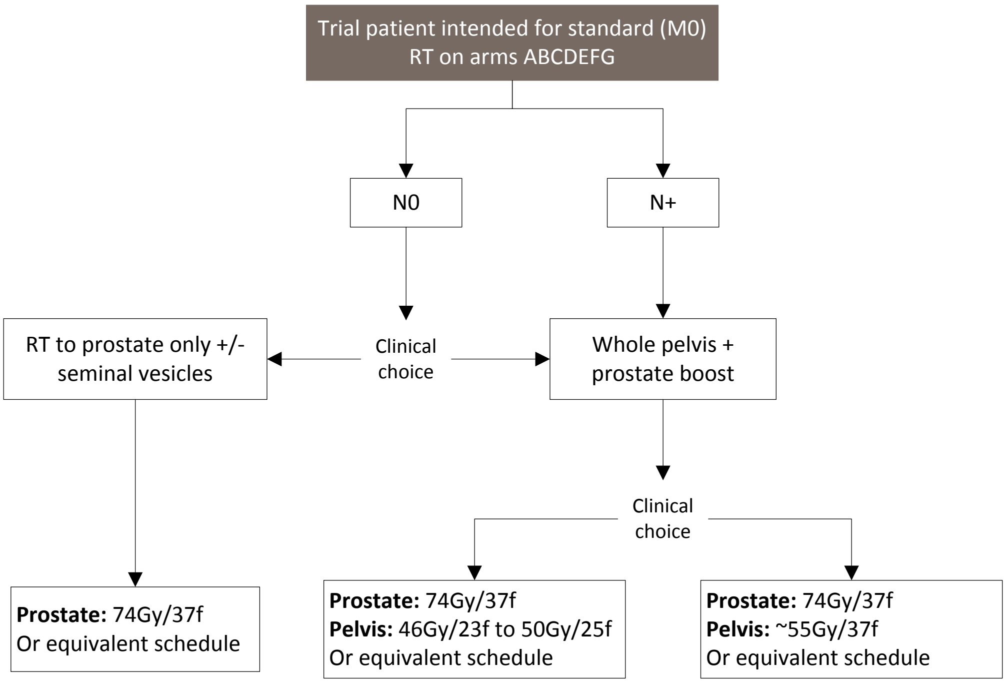

6.7 NON-TRIAL TREATMENT

6.7.1 MEDICATIONS PERMITTED

Any additional treatment that the responsible physician feels is appropriate is permitted.

6.7.2 DATA ON CONCOMITANT MEDICATION

All concomitant medication will be recorded on the baseline form prior to randomisation and on any subsequent Serious Adverse Event forms. This should include aspirin that may be taken on a regular basis for cardiovascular disease, the use of any Non-Steroidal Anti-inflammatory Drugs (NSAID) as well as any vitamin or mineral supplements the patient is taking.

7 ASSESSMENTS AND PROCEDURES

7.1 FLOW CHART/SCHEDULE FOR FOLLOW-UP

A detailed follow-up schedule is given in **Table 5**.

7.1.1 PSA MEASUREMENTS

All patients should have PSA measured pre-androgen deprivation therapy and at weeks 6, 12, 18 and 24 and every 12 weeks, thereafter, up to 2 years post randomisation. Following this, PSA should be measured every 6 months until 5 years and annually, thereafter. For patients who do not have a scheduled hospital visit, it would be acceptable for arrangements to be made for blood samples to be drawn in a GP's surgery.

7.1.2 ASSESSMENT OF TREATMENT FAILURE (DEFINITION OF PROGRESSION)

It is not proposed to routinely assess patients for response. However, in order that objective progression can be assessed, it is necessary to have imaging taken at time of best response as judged by the treating clinician. All patients should have baseline radiological examinations as detailed in **Section 4.3.1**. In addition it is recommended that all patients should have scans or X-rays repeated at 24 weeks (and whenever clinically appropriate) if they were abnormal at baseline, particularly if they have a low PSA value on entry in to the trial making biochemical assessment of treatment failure difficult. The following events would constitute a disease progression and should be reported on a progression form:

- Biochemical failure – must be reported alongside castrate levels of testosterone if the patient has received intermittent ADT (see **Appendix J**).
- Local progression
- Lymph node progression
- Progression in distant metastases
- Development of new metastases

Please note that skeletal-related events (SREs) may be indicative of disease progression but can have other causes such as osteoporotic fracture. All SREs should be investigated further to establish whether or not the patient has progressed, in which case a progression form should be completed.

7.1.3 ADDITIONAL SAFETY ASSESSMENT

Due to the risk of liver toxicity and secondary hyperaldosteronism with abiraterone, patients will require 2 weekly U+Es, LFTs and blood pressure measurement for the first 12 weeks. It is not proposed to collect the detail of these measurements unless results are abnormal; in this instance, they should be reported as AEs (on the next Follow-up CRFs) and as an SAEs (see **Section 11**) if appropriate.

Medical review and PSA measurements follow the pattern in the control arm: visits at weeks 6, 12, 18 and 24 and every 12 weeks, thereafter, up to 2 years post randomisation. Following this, PSA should be measured every 6 months until 5 years and annually, thereafter. For patients who do not have a scheduled hospital visit, it would be acceptable for arrangements to be made for blood samples to be drawn either in a GP's surgery or in the patient's home.

7.1.4 DATA COLLECTION AND NON-ADMINISTRATION OF STANDARD RADIOTHERAPY

There are CRFs to be completed for patients receiving primary radiotherapy whether this is standard radiotherapy for M0 patients on any arm or prostate radiotherapy for Arm H patients. All

radiotherapy and acute side effects details will be recorded on the Radiotherapy Form; any late side effects will be recorded on the Follow up form.

If it is decided not to give the planned radiotherapy (for example, due to early metastatic progression or patient refusal), this should be stated on the Standard Radiotherapy form together with the reason for non-administration of the treatment.

7.1.5 DATA COLLECTION PALLIATIVE RADIOTHERAPY

For patients who receive palliative radiotherapy as part of first line treatment, a Palliative Radiotherapy CRF should be completed. Details of salvage RT for relapse and palliative treatment will be requested and completed only on the Progression Form.

7.1.6 DATA COLLECTION RESEARCH (M1) RADIOTHERAPY

There are arm specific CRFs for patients randomised to arm H. Adverse events such as hip fractures, TURPs, skeletal-related events will be collected retrospectively via the Hospital Episode Statistics (HES) database.

7.1.7 FOLLOW-UP SCHEDULES

An individualised form with a follow-up schedule will be provided for each randomised patient. For patients who are receiving LHRH analogues, it is assumed that any additional treatment will commence within two weeks of randomisation. For patients who are due to have an orchidectomy it is recognised that surgery will have to be scheduled and the scheduling of any additional treatments may be affected by post-operative recovery. It is recommended that all patients who had abnormal radiological investigations at baseline or present with a low PSA on entry into the STAMPEDE trial should have them repeated 24 weeks after randomisation.

7.2 FOLLOW-UP

Every effort should be made to follow-up all patients who have been randomised. Patients should, if possible, remain under the care of an oncologist or urologist for the duration of the trial. If care of a patient is returned to the GP, it is the responsibility of the consultant who obtained the patient's consent to participate in the trial to ensure that the data collection forms are completed. If the patient moves from the local area, arrangements should be made for trial follow-up to be undertaken by their new local centre. Details of other participating centres can be obtained from the MRC CTU. The consent of patients should be obtained for their names to be flagged for survival information through national registries, for example NHS Information Centre/Office of National Statistics (ONS) in England/Wales and General Register Office in Scotland, Hospital Episode Statistics (HES). If the clinician moves, appropriate arrangements should be made to arrange for trial follow-up to continue at the centre.

Table 5: Summary of timing of case report forms

CASE REPORT FORMS	TIMING OF ASSESSMENT AND CRF
Bone Density Risk Factor	At randomisation
Randomisation	At randomisation
Baseline	At randomisation
Cardiovascular Assessment	At randomisation
Pathology	At randomisation. When pathology sample has been taken and sent to UCL laboratory.
Pre-18 Week Bisphosphonate	Treatment administered every 3. Form holds data for 2 cycles. Form to be sent after 2nd cycle given.
Post-18 Week Bisphosphonate Treatment	Treatment administered every 4. Form holds data for 3 cycles. Form to be sent after 3rd cycle given.
Docetaxel Treatment	Treatment administered every 3 weeks Form holds 2 cycles. Form to be sent after 2nd cycle given.
Standard-of-Care (M0) RT	When standard radiotherapy is completed or if planned RT is no longer to be given (all patients planned for RT in Arms A to G)
Research (M1) RT	When research radiotherapy (Arm H) is completed or at 3 months (Arm A)
Follow-Up	Every 6 weeks for 6 months, then every 12 weeks until 2 years. Every 6 months until 5 years and annually thereafter. (See **Table 6** for more information.)
Palliative Radiotherapy	If applicable, when the palliative radiotherapy course is completed.
End of Treatment	When each treatment is completed (either at end of scheduled treatment or at early cessation of treatment).
Progression & Additional Treatment	At the first occurrence of each type of progression and whenever a patient that has progressed receives additional treatment.
Serious Adverse Event	Following any Serious Adverse Event
Skeletal-related Event	Whenever a patient experiences a skeletal-related event
Death	At Death
Patient Transfer	When a patient is transferred to a different hospital for the administration of trial treatment and follow up

Note: Quality of Life Study is only for first 700 patients entered into the trial and those who were recruited after the implementation of version 8.0 of the protocol. MRC CTU will inform centres of which of their patients this applies to.

Table 6: Data required on follow-up forms

TIMING OF FOLLOW-UP	PSA	EVIDENCE OF PROGRESSION	ANDROGEN DEPRIVATION THERAPY	ABIRATERONE TREATMENT	UNSCHEDULED VISITS	TOXICITIES
Before progression	✓	✓	✓	✓	✓	✓
After Progression	-	✓	✓	✓	✓	✓

7.3 TRIAL CLOSURE

For the purpose of complying with UK Clinical Regulations introduced on May 2004, the trial will be considered 'closed' when the follow-up point for the primary analysis of the final comparison has been reached. However, further observational follow-up of all patients enrolled in the trial will continue until all randomised patients have died. This will initially be via the hospital, but in the longer term may employ national registers.

Table 7: Schedule for completion of treatment and outcome forms by arm.

Timing From Randomisation			Treatment Forms			Outcome forms	
Years	Months	Weeks	Zol. acid	Docetaxel	RT	Follow-up$^{\Psi}$	QL + HE$^{¥}$
6-Weekly							
-	-	6	B,E,F (†)	C,E (†)	-	All arms	All arms
-	-	12	B,E,F (†)	C,E (†)	M1: A,H	All arms	All arms
-	-	18	B,E,F (†)	C,E (†)	-	All arms	All arms
-	-	24	B,E,F (‡)	-	-	All arms	All arms
12-Weekly							
-	-	36	B,E,F (‡)	-	-	All arms	All arms
-	-	48	B,E,F (‡)	-	M0: A,B,C,E,G	All arms	All arms
-	-	60	B,E,F (‡)	-	-	All arms	All arms
-	-	72	B,E,F (‡)	-	-	All arms	All arms
-	-	84	B,E,F (‡)	-	-	All arms	All arms
-	-	86	B,E,F (‡)	-	-	All arms	All arms
6-Monthly							
2	24	104	B,E,F (‡)	-	-	All arms	All arms
	30	130	-	-	-	All arms	All arms
3	36	156	-	-	-	All arms	All arms
	42	182	-	-	-	All arms	All arms
4	48	208	-	-	-	All arms	All arms
	54	234	-	-	-	All arms	All arms
5	60	260	-	-	-	All arms	All arms
Annual							
6	-	-	-	-	-	All arms	All arms
7	-	-	-	-	-	All arms	All arms
Etc	-	-	-	-	-	All arms	All arms

Key:
A = ADT alone
B = ADT + zoledronic acid
C = ADT + docetaxel
D = ADT + celecoxib
E = ADT + zoledronic acid + docetaxel
F = ADT + zoledronic acid + celecoxib
G = ADT + abiraterone
H = ADT + M1 research RT to the prostate

Notes:
$^{\Psi}$ See Table 6 for information required at follow-up
† Form records data for two cycles
‡ Form records data for three cycles
¥ 1st 700 patients and those recruited from protocol version 8.0 onwards only

Note: recruitment completed to both celecoxib-containing arms in Apr-2011 at the end of Activity Stage II

Radiotherapy, Late RT Toxicity, Palliative Radiotherapy Progression, SAE, End of Treatment, Co-enrolment and Death forms to be completed as required.

8 STOPPING OF TREATMENT OR FOLLOW UP

Patients should be given every encouragement to adhere to protocol treatment and follow-up schedule, in order to reduce biases. However, a patient has the right to withdraw consent for participation in any aspect of this trial at any time.

8.1 STOPPING RESEARCH INTERVENTIONS

A patient may stop trial treatment for the following reasons:

- Progression whilst on therapy (trial treatment must be discontinued in this instance). For patients randomised to Arm G, please refer to **Section 6.1.7** for criteria to stop treatment
- Unacceptable toxicity
- Intercurrent illness which prevents further treatment
- Withdrawal of consent for treatment
- Any alteration in the patient's condition which justifies the discontinuation of treatment in the clinician's opinion
- Intention to commence a new anti-cancer treatment due to evidence of relapse.

The reason should be recorded on the treatment and/or follow-up forms as well as the End of Treatment form. In the case of abiraterone, the disease event for stopping abiraterone may be after the first reportable failure-free survival event (see **Section 6.1.7**). Unless a patient states otherwise, it should be assumed that consent is given to continue to record trial data.

8.2 PATIENT TRANSFERS

For patients moving from the area, every effort should be made for the patient to be followed-up at another participating trial centre and for this trial centre to take over responsibility for the patient. To document the transfer process the main contact person at both the current and receiving hospitals should complete and sign the Patient Transfer Confirmation form. A fully completed form must be returned to the CTU prior to the patient transfer and ideally any data queries for the patient should be completed prior to transfer.

On receipt of the completed transfer form a member of the STAMPEDE team will confirm the database has been updated and request confirmation of the name of the patient's new Clinician. Photocopies of the following documents may then be sent to the new hospital to complete the transfer and copies must be also retained at the original site for monitoring purposes:

- Consent form
- Completed CRFs
- Any documentation relating to the patient's participation in STAMPEDE (patient names must be removed from any documentation).

8.3 WITHDRAWAL FROM THE TRIAL COMPLETELY

If a patient explicitly withdraws consent to have any data recorded their decision must be respected and the MRC CTU must be informed in writing. All communication surrounding the withdrawal

should be noted in the patient's records and no further STAMPEDE CRFs should be completed for that patient.

Early stopping of follow-up should not be undertaken lightly and the site must consider the implications for the trial and the patient in reaching such a decision.

Patients can change their minds about withdrawal at any time and re-consent to participate in the trial. Follow-up data should be collected only from the point of when consent was re-instated.

9 STATISTICAL CONSIDERATIONS

9.1 METHOD OF RANDOMISATION

Patients will be randomised centrally using a computerised algorithm developed and maintained by the MRC CTU. Randomisation will be performed using the method of minimisation over a number of clinically important stratification factors with an additional random element. To decrease determinability, the factors are not listed here but can be found in the Statistical Design Document. From the outset, the trial had 1 control arm (A) and 5 research arms (B, C, D, E and F); from version 8.0, an additional research arm (G) was introduced.

As the control arm is the comparator arm for all the research arms, it is intended to recruit twice as many patients to the control arm as to each of the original research arms as this is an efficient design. Therefore, the initial randomisation ratio will be 2A:1B:1C:1D:1E:1F. From version 7.0, accrual to the celecoxib-containing arms was halted and the allocation ratio was 2A:1B:C1:D0:E1:F0.

The allocation weighting for the additional arm G (version 8.0 onwards) will be 2, meaning that as many patients will be randomised to arm G as the control arm A: the randomisation ratio will be 2:2 (equivalent to 1:1 control:abiraterone). This gave an overall allocation ratio of A2:B1:C1:D0:E1:F0:G2. When recruitment has been completed to the ongoing original research arms B, C and E (which will be around 2 years before completion of accrual to G), the allocation ratio will be A2:B0:C0:E0:D0:F0:G2 (or 2:2). This is more efficient for this comparison than the 2:1 allocation ratio employed for the original research arms because of the minimal co-recruitment period.

Version 9.0 introduced a RT comparison for men with metastatic disease which is irrelevant to a subset of men joining STAMPEDE. This can only be achieved by splitting the randomisation system so that patients with newly diagnosed M1 disease and no contraindication will be randomised A2:B1:C1:D0:E1:F0:G2:H2 and other men will be randomised A2:B1:C1:D0:E1:F0:G2:H0. Note that the allocation ratio for each pairwise comparison in unaffected, only the rate at which comparisons accrue.

Version 10.0 follows the successful completion of recruitment to arms B, C and E. Therefore, the allocation ratio will be A2:B0:C0:E0:D0:F0:G2 (or A2:G2) for M0 patients and A2:B0:C0:E0:D0:F0:G2:H2 for M1 radiotherapy arm patients (2A:2G:2H).The equal allocation ratio is suitable with fewer research arms open.

9.2 OUTCOME MEASURES

The overall, definitive primary outcome measure for the trial for each comparison is overall survival (all-cause mortality). The design of the trial is such that it is important to have additional intermediate outcome measures to assess each research arm as the trial progresses. These are listed in **Table 8**. The intermediate primary outcome measure is failure-free survival. The reasons for different emphases in each recruitment stage are explained in **Section 9.3**.

Table 8: Trial Outcome Measures by Comparison Stage

TRIALS STAGE	PRIMARY OUTCOME MEASURES	SECONDARY OUTCOME MEASURES
Pilot phase	Safety*	Feasibility
Activity Stage (AS) I-III	Failure-free survival (FFS)†	Overall survival (OS)<br>Toxicity<br>Skeletal-related events
Efficacy Stage (ES) IV	Overall survival	Quality of life<br>Cost effectiveness<br>Failure-free survival†<br>Toxicity<br>Skeletal-related events

*Based on toxicity
†Including biochemical failure (see **Appendix J**)

9.3 SAMPLE SIZE: PRINCIPLES AND ASSUMPTIONS

The overall design for this study is a multi-arm multi-stage, multi-centre randomised controlled trial. There are five stages for each research arm: a Pilot Phase, Activity Stages I-III and Efficacy Stage IV. Full details of the methodology underlying the trial design are given by Royston et al. (44, 45) The sample size calculations were performed using the `stage2` (version 1.2.0, March 2002) and `stagen` (version 1.1.1, 18 May 2004) programs, both implemented in Stata (Stata Corp, TX) and updated using the later `nstage` program (version 1.0.3, 13-jun-2007; version 2.1.0, 28-jun-2009). (46)

The trial is designed under the assumptions in **Table 9**, and additionally, we assume a slightly higher proportion of non-metastatic than metastatic patients such that the median FFS is two years and median OS four years.

Table 9: Hazard ratio assumptions under null and alternative hypotheses

SIZE OF HR	PILOT	AS I-III	ES IV
Under null hypothesis (H0)	n/a	HR(FFS) = 1.00	HR(OS) = 1.00
Under alternative hypothesis (H1)	n/a	HR(FFS) = 0.75	HR(OS) = 0.75

The HR of 0.75 for any research arm relative to control would translate into an absolute improvement in FFS of 10%, from approximately 50% to 60% at two years and OS of 10%, from approximately 50% to 60% at four years. A beneficial difference of this size would be clinically worthwhile and, indeed, experience tells us it may be unrealistic to expect a larger difference. Therefore, we have adequately powered the trial to detect a HR of 0.75 for overall survival. This

design gives 95% power at Activity Stages I-III and 90% power at Efficacy Stage IV for each comparison. Further details of the sample size calculations are summarised in **Sections 9.4** and **9.5** and detailed in a separate Statistical Design Document which is available on request.

Note that, from version 8.0, standard-of-care M0 RT was introduced to the majority of patients with N0 M0 disease. This is likely improve the outcomes for this group. Further agents are starting to be licensed for patients with castration-refractory disease which may also improve survival rates. Improved FFS rates would delay the intermediate analyses; improved survival rates would delay the definitive analyses. The Statistical Design Document includes models where median survival is estimated at 5 years rather than 4 years. The trial is powered to detect a difference in relative improvement and the analyses will be performed when a pre-planned number of events has been reported in the control arm, rather than after a certain number of patients have been recruited or a certain amount of time elapsed. **Sections 9.4** and **9.5** provide more detail.

9.4 SAMPLE SIZE ISSUES AND TRIAL STAGES: ORIGINAL RESEARCH ARMS (B-F)

9.4.1 PILOT PHASE: ORIGINAL RESEARCH ARMS (B-F)

It was anticipated that 210 patients will be recruited to the Pilot Phase from a limited number of centres over a one year period. Approximately 60 patients would be randomised to the control arm and 30 patients to each of the five research arms, each of which would be assessed for safety and feasibility. If recruitment proved unfeasible or any of the research arms proved unsafe or not feasible to administer (e.g., poorly tolerated or unexpected toxicity) recruitment to these arms would have been discontinued. There were already considerable safety data on the use of docetaxel and zoledronic acid in patients with malignancies including prostate cancer, and on the use of Cox-2 inhibitors (including celecoxib), although mainly from patients with musculoskeletal disorders. There were fewer data on the combination arms, but it was thought very unlikely that any of the research arms would be discontinued during the Pilot Phase. When 210 patients had been on the trial for a minimum of 18 weeks, the independent Data Monitoring Committee (IDMC) would review the data from the Pilot Phase. Recruitment continued to the trial during this period as equipoise remained. Recruitment continued beyond this point. Safety data continues to be assessed throughout the trial.

9.4.2 ACTIVITY STAGES I-III: ORIGINAL RESEARCH ARMS (B-F)

In the sample size calculations, we assumed that all research arms successfully pass through the Pilot Phase to Activity Stage I and that patients would be recruited at a rate of approximately 500 per year. This was faster than in the Pilot Phase because the trial would recruit from additional centres, both in the UK and internationally. The analysis of Activity Stages I, II and III were planned for when around 113, 216 and 334 failure-free survival events have been observed in the control arm, respectively.

The Activity Stage analyses comprise pairwise comparisons of FFS between the control arm and each of the 5 research arms (i=B, C, D, E, F). Let HR_i(true) represent the hazard ratio (HR) of the ith research arm to the control arm, and HRi(observed) the observed value. Discontinuation of accrual of further patients will be considered for the ith research regimen at each of Activity Stages I-III according to the guidelines in **Table 10**.

Table 10: Guidelines for stopping accrual to the ith original research arm

Activity Stage	Number of control arm events	Consider discontinuation if HR_i(observed) is...
I	~113	>1.00
II	~216	>0.92
III	~334	>0.89

9.4.3 Efficacy Stage IV: Original Research Arms (B-F)

The analysis of Efficacy Stage IV for the original research arms will be performed when around 403 deaths have been observed in the control arm. This would give 90% power to detect the targeted hazard ratio of 0.75 at one-sided significance level of 0.025. The actual length of this stage, balancing continued accrual with just follow-up, depended on the number of arms passing through to further recruitment from Activity Stages I-III and the observed accrual and event rates.

9.4.4 Sample Size For Original Research Arms (B-F)

Assuming an accrual rate of 500 patients/year, between 2800 and 3600 patients were planned to be entered into the original research comparisons of the trial over a period of 5½ and 7 years. The exact number of patients to be entered depends on the observed accrual rate and the observed event rate, which is, in itself, dependent on the mix of patients joining the trial from the broad spectrum of eligibility. The primary analysis on overall survival requires around 403 deaths to be observed on the control arm. Accrual continued until the main analysis can be foreseen so that the overall duration of the comparisons would be as short as possible (longer accrual facilitates this) and so that few, if any, patients remain on treatment when the main results are released. The statistical team have monitored and projected the timelines using the `artpep` command in Stata. Results should be due in 2015. Further information is available in the Statistical Master File.

9.5 SAMPLE SIZE ISSUES AND TRIAL STAGES: ADDITIONAL RESEARCH ARM G

9.5.1 Pilot Phase: Additional Research Arm G

A similar approach will be followed for the additional research arm G, as detailed for the original research arms in **Section 9.4.1**. The IDMC reviewed safety data, in the context of data from the control arm, when the first 30 patients allocated to arm G had been on trial for at least 18 weeks.

Furthermore, an additional review of safety was performed when 30 patients with newly diagnosed non-metastatic disease allocated to arm G had been on trial for at least 18 weeks.

Both of these milestones were successfully completed.

9.5.2 Activity Stages I-III: Additional Research Arm G

The same principles will be applied to the new comparison as to the previous comparisons. The notable difference will be in the accrual rate to this comparison which is anticipated to be higher. There are two reasons for this. First, STAMPEDE started to recruit slowly in only a limited number of pilot sites. As more sites have been activated, including internationally, accrual has increased. At the time of version 8.0 of the protocol, monthly accrual to the study was averaging around 60 patients/month (over 700 patients/year). Second, there is an equal allocation ratio for the abiraterone arm compared to the control arm. It is this different allocation ratio which means that the number of control arm events required to trigger the intermediate analyses is different for the

assessment of abiraterone to the assessment of the original research arms. This is shown in **Table 11**.

Table 11: Guidelines for stopping accrual to the additional research arm G

ACTIVITY STAGE	NUMBER OF CONTROL ARM EVENTS	CONSIDER DISCONTINUATION IF HR_G(OBSERVED) IS...
I	~75	>1.00
II	~142	>0.92
III	~221	>0.89

9.5.3 EFFICACY STAGE IV: ADDITIONAL RESEARCH ARM G

The analysis of Efficacy Stage IV for the additional comparison will be performed when around 267 deaths have been observed in the control arm. This would give 90% power to detect the targeted hazard ratio of 0.75 at one-sided significance level of 0.025.

9.5.4 SAMPLE SIZE FOR ADDITIONAL RESEARCH ARM G

Consideration will be given to ceasing further randomisations to arm G if it is not showing sufficient evidence of activity, just as for research arms B to F. Up to around 1500 patients will join the abiraterone comparison with half allocated to the research arm. Providing the accrual rate remains above 50 patients/months, accrual will be halted when 1500 patients have been recruited or after 3 years, whichever is the sooner. The total number of patients joining this comparison depends on the same issues as for the original comparisons (notably, observed accrual and event rates) but also the length of time that the original research arms co-recruit alongside the additional research arm. It is assumed that this will be for approximately 1 year. The sample size calculations and projected durations are fairly robust to changes in the co-recruitment with the original research arms and future co-recruitment of any further research arms which the Trial Management Group may introduce. This is detailed in the Statistical Design Document.

9.6 SAMPLE SIZE ISSUES AND TRIAL STAGES: ADDITIONAL RESEARCH ARM H

9.6.1 PILOT PHASE: ADDITIONAL RESEARCH ARM H

A similar approach will be followed for the additional research arm H as detailed for the original research arms in **Section 9.4.1**. The IDMC will review safety data, in the context of data from the control arm, when the first 30 patients allocated to arm H have been on trial for around 18 weeks.

9.6.2 ACTIVITY STAGES I-III: ADDITIONAL RESEARCH ARM H

The same principles will be applied to the new comparison as to the previous comparisons and an equal allocation ratio of control arm patients to patients allocated arm H will be employed, as for Arm G. The number of control arm events required to trigger the intermediate analyses will be the same as for the abiraterone comparison (see **Table 13**).

9.6.3 EFFICACY STAGE IV: ADDITIONAL RESEARCH ARM H

The analysis of Efficacy Stage IV for the additional comparison will be performed when around 267 deaths have been observed in the control arm. This would give 90% power to detect the targeted hazard ratio of 0.75 at one-sided significance level of 0.025.

9.6.4 SAMPLE SIZE FOR ADDITIONAL RESEARCH ARM H

Consideration will be given to ceasing further randomisations to arm H if it is not showing sufficient evidence of activity on the intermediate primary outcome measure (FFS), just as for the other research arms. This research comparison is relevant to around 60% of patients joining STAMPEDE. At the point of the scientific approval, accrual was averaging around 80 patients per month to the trial. If accrual to the trial was slower at 70 patients per month, then accrual to this comparison could be between 18 and 42 patients per month, depending on which other trial arms are open to recruitment at the time.

We are targeting a 25% relative improvement in overall survival following local radiotherapy to the prostate in this patient group. This is the same size of effect targeted with the other research arms in STAMPEDE. This relative improvement can be further justified in the light of MRC PR07 which demonstrated an improvement of this magnitude for adding radiotherapy to ADT in locally advanced disease, with a hazard ratio for overall survival of 0.77 (95% CI 0.61 to 0.98). In that trial, fewer than half of the deaths were from prostate cancer, whereas in newly diagnosed metastatic patients nearly all men will die of their disease. Therefore, it is relevant to note the relative benefit of radiotherapy in PR07 in terms of prostate cancer-specific survival, where the hazard ratio was 0.54 (95% CI 0.27 to 0.78). Long-term survival-based data, with a median follow-up of ~10 years, were presented orally at the American Society of Clinical Oncology 2012 which confirmed these findings.(7)

We anticipate that around 1250 patients are required over 4 years to observe 267 control arm deaths after 5.25 years. In addition to the factors listed in **Section 2.1.2**, this assumes that (i) recruitment is constantly 70 pts/m to the trial overall, (ii) the original research arms stop accrual within 6 months after activation of the RT arm, (iii) the abiraterone arm stops accrual around 24 months after activation of the RT arm, and (iv) a further new research arm with an equal allocation ratio is introduced 18 months after activation of the RT arm.

With variations on these factors, between 1000 and 1400 patients are required over 2.75 to 4.50 years to address survival within 4.50 to 6.50 years. These sample scenarios will be documented in the Trial Master File.

All patients joining the trial will be starting long-term ADT for the first time. The focus of this comparison will be on the newly diagnosed, metastatic patients (and no contraindications to RT), which is the largest subgroup of patients in the trial and the group of patients at highest risk. Patients with non-metastatic disease will be excluded from this particular comparison as there are already randomised data demonstrating the survival benefit from radiotherapy in patients with locally advanced disease. Radiotherapy is now mandatory in node negative patients; it is also recommended in the node-positive, non-metastatic (N+ M0) group.

For the control arm of the whole trial, we assumed that median failure-free survival is 18, 24 or 30 months and constructed sample size scenarios around each of these options; the event rate would depend on the patient mix. We now know that 60% of patients have M1 disease at trial entry and we have reported that FFS at 24 months is 51% across the whole of the control arm.(1)

For the sample size calculation for this new planned comparison, we have based our estimates on the subgroup of patients with newly diagnosed M1 disease in the control arm. Therefore, we estimate median FFS to be 1 year and estimate that median overall survival will be 3.5 years.

Figure 9: Progress Of STAMPEDE Through The Trial Stages

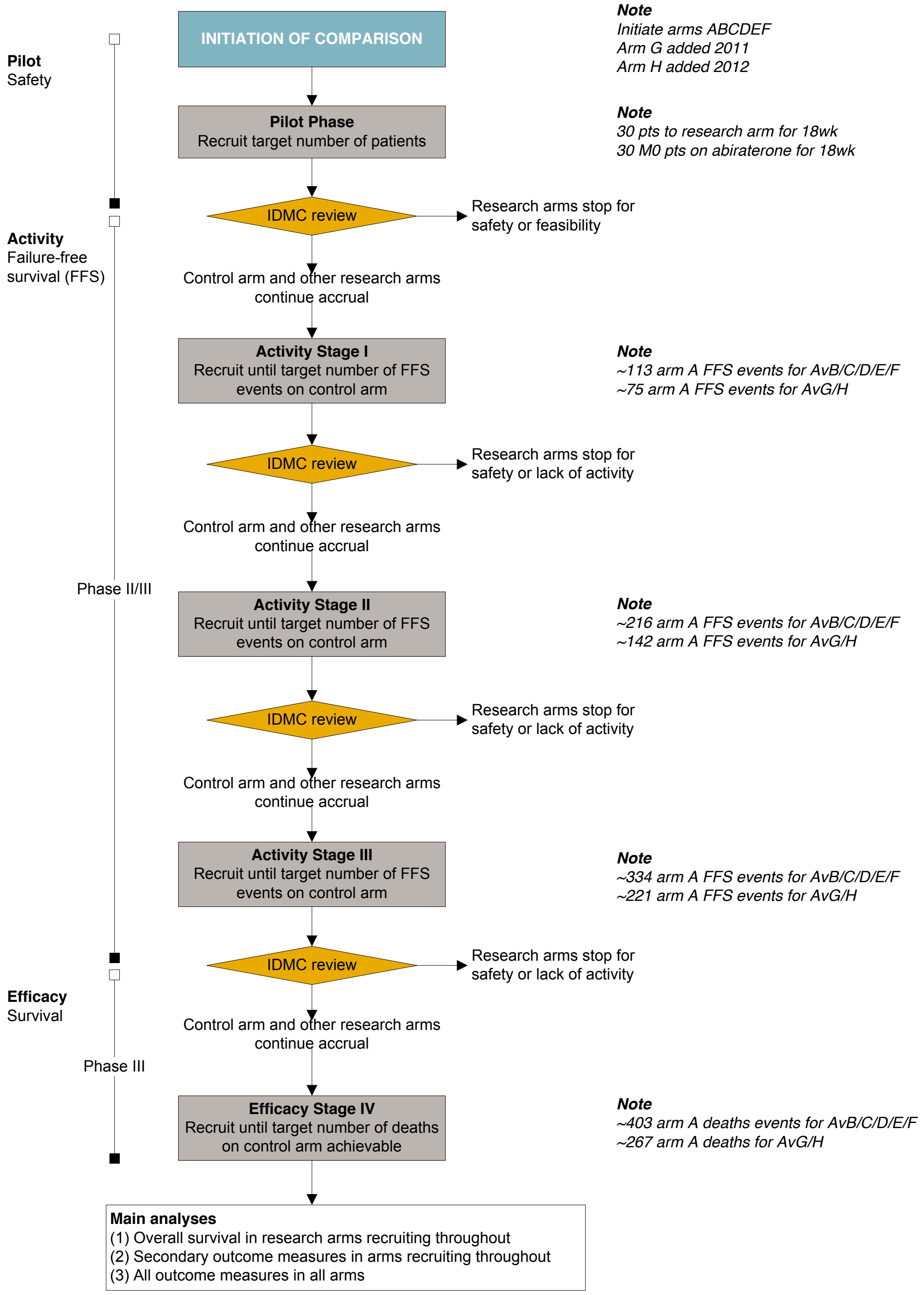

Key

FFS: Failure-free survival
HR: Hazard ratio
IDMC: Independent Data Monitoring Committee
Pts: Patients

Notes

Exact accrual depends on many factors including accrual rate, event rate and arms recruiting in each stage

9.7 FURTHER NOTES ON TRIAL DESIGN

9.7.1 Overall Sample Size

Given the adaptive nature of the study, there is no formal overall sample size target, but the numbers of patients required for each comparison are detailed in **Sections 9.4** and **9.5**. It is expected that more than 5000 patients will likely be recruited overall.

9.7.2 Factorial Design

We note here that we have not employed a factorial design in this trial because we anticipate the possibility of synergy between ADT, zoledronic acid and docetaxel and between ADT, zoledronic acid and celecoxib. It would not be possible to assess any such interactions reliably in a factorial trial (see the Statistical Design Document for further details).

9.8 INTERIM MONITORING AND ANALYSES

The accumulating data will be reviewed at regular intervals (approximately annually) by an Independent Data Monitoring Committee (IDMC), including pre-specified formal intermediate analyses of activity data (see also **Section 16**). These analyses will be performed by the trial team at the MRC CTU. Only patients randomised contemporaneously will be included in the comparison of each research arm against control i.e. patients allocated to the control arm prior to version 8.0 will not contribute to the comparison of abiraterone (Arm A vs Arm G).

The IDMC will be asked to give advice on whether the accumulating data from the trial with the guidelines for discontinuation of accrual for Activity Stages I-III, together with results from any other relevant trials, justifies continuing recruitment of further patients or further follow-up. A decision to discontinue recruitment, in all patients or in selected subgroups will be made only if the result is likely to convince a broad range of clinicians including those entering patients into the trial and the general clinical community. The intermediate stopping guidelines apply to the intermediate primary outcome measure. To stop accrual early for benefit in any comparison would require convincing data in terms of the definitive primary outcome measure, overall survival. For example, this could be $p<0.001$ as proposed by Haybittle-Peto.(47, 48) The use of such a guideline for stopping for benefit has a minimal impact on the operating characteristics.

If a decision is made to continue without change, the IDMC will advise on the frequency of future reviews of the data on the basis of accrual and event rates. The IDMC will make recommendations to the Trial Steering Committee (TSC, see **Section 16**) as to whether the trial should continue in its present form. While the trial is ongoing the accumulating data will generally remain confidential, unless the TSC and IDMC agree that the data should be made public.

9.9 OUTLINE ANALYSIS PLAN

Analyses will be performed on an intention-to-treat basis. The standard unadjusted log-rank approach will be applied to analyses of FFS and OS. The impact of potential confounders including the stratification factors used at randomisation will be considered in a Cox proportional hazard model. Flexible parametric models will be used to calculate the absolute differences between the arms to show treatment differences over time and to estimate restricted mean "survival" times (RMST). The estimated difference in restricted means survival time will be used preferentially to compare treatment arms if the proportional hazards assumptions required for hazard ratios cannot be supported. The χ^2 test or Mann-Whitney test will be implemented for categorical data

comparisons, including toxicity, as appropriate. The primary outcome measures (see **Section 9.2**) will be considered for all arms of the trial at each phase, but the main emphasis will be placed on the comparison of the research arms that have continued to recruit throughout the trial.

9.9.1 Pilot / Safety Phases

The Pilot Phase randomised patients between all the trial arms so that the results from these patients can be included in the main trial. Feasibility is considered in terms of the acceptability of the trial randomisation and reported toxicities and adherence to trial medication. Centres participating in the Pilot Phase for the original research arms were required to keep an anonymised log of all patients assessed for trial eligibility (see protocol version 2.0) so that the number of patients who did not participate in the study and the number of eligible patients who choose to not participate in the study could be summarised (reasons for non-participation were collected where the patients was willing). The anonymised logs will not be needed for new research arms like Arm G introduced in version 8.0 or Arm H introduced in version 9.0.

For the patients who are randomised, we shall describe the incidence of expected and unexpected severe toxicities and adverse events/reactions (see **Section 11**) to decide whether to continue with research arms beyond the Pilot Phase. As indicated above, we do not anticipate that recruitment to the research arms will be discontinued after the Pilot Phase, as there is considerable experience with zoledronic acid and docetaxel when combined with ADT, while Cox-2 inhibitors generally have a good toxicity profile. Although there are limited data on the combinations, we do not expect severe toxicity.

9.9.2 Activity And Efficacy Stages

The approach to analysis of these stages is summarised within the sample size calculations (see Section 9.4.3). Each research arm will be compared in a pairwise fashion against the control arm.

Full details are available in the Statistical Analysis Plan.

10 MONITORING AND QUALITY ASSURANCE

10.1 MONITORING AT MRC CTU

Data provided to the MRC CTU will be checked for missing or unusual values (range checks) and consistency over time. If missing or questionable data are identified, staff at the MRC CTU will request that the data be clarified. The exact procedures for data clarification and the amendment of CRFs will be described in the trial specific SOPs and instructions will be sent to all STAMPEDE institutions as soon as they have been approved to participate in the trial. The MRC CTU will also send reminders for any overdue data.

10.2 DIRECT ACCESS TO DATA

Collaborating institutions should be aware that direct access to patient data by MRC CTU staff may be required for trial-related monitoring or audit. Patient consent for this will be obtained as part of the general trial consent process.

10.3 VISITS TO INVESTIGATOR SITES

A selection of institutions will be visited at least once during the course of the STAMPEDE trial. The MRC CTU will give the responsible investigator adequate notice of the monitoring visit to allow adequate time, space and staff for these visits. The standard operating procedures (SOP) for monitoring are available from the MRC CTU.

After the monitoring visit the monitor will complete a site visit report. This report will be circulated to the TMG for comment. Once the TMG have reviewed the report and agreed on any recommendations the monitor will finalise the report and send a copy to the Principal Investigator (PI) at the site. A copy will also be sent to the CI for the trial and another copy will be kept in the MRC CTU STAMPEDE trial master file.

10.4 CONFIDENTIALITY

All information collected during the course of the research will be kept strictly confidential. In addition, all procedures for handling, processing, storage and destruction of data are compliant with the Data Protection Act 1998. No individual patients will be identified when the results of the trial are published.

Patients will be asked for permission for information about their health status to be obtained from the Office of National Statistics (ONS) or via the NHS Strategic Tracing Service or similar by the Medical Research Council, if necessary. In addition, patients will be asked for permission to inform their GP of their involvement in the STAMPEDE trial.

11 SAFETY REPORTING

ICH GCP requires that both investigators and sponsors follow specific procedures when reporting adverse events/reactions in clinical trials. These procedures are described in this section of the protocol. Further information on the expected toxicities for the trial interventions (docetaxel, zoledronic acid, abiraterone and radiotherapy) can be found in **Appendix G**.

11.1 DEFINITIONS

The safety reporting definitions from ICH GCP apply in this trial protocol. These definitions are given in **Table 12**.

Table 12: Event Terms and Definitions

TERM	DEFINITION
Adverse Event (**AE**)	Any untoward medical occurrence in a patient or clinical trial subject to whom a medicinal product has been administered including occurrences which are not necessarily caused by or related to that product.
Adverse Reaction (**AR**)	Any untoward and unintended response to an investigational medicinal product related to any dose administered.
Unexpected Adverse Reaction (**UAR**)	An adverse reaction, the nature or severity of which is not consistent with the information about the medicinal product in question set out in the summary of product characteristics (or Investigator brochure) for that product.
Serious Adverse Event (**SAE**) or Serious Adverse Reaction (**SAR**) or Suspected Unexpected Serious Adverse Reaction (**SUSAR**)	Respectively any adverse event, adverse reaction or unexpected adverse reaction that:<br>• results in death<br>• is life-threatening*<br>• requires hospitalisation or prolongation of existing hospitalisation**<br>• results in persistent or significant disability or incapacity<br>• consists of a congenital anomaly or birth defect<br>• Other important medical condition***

Clarifications and Exceptions

*The term 'life-threatening' in the definition of 'serious' refers to an event in which the patient was at risk of death at the time of the event; it does not refer to an event which hypothetically might have caused death if it were more severe.

**Hospitalisation is defined as an inpatient admission, regardless of length of stay, even if the hospitalisation is a precautionary measure for continued observation. Hospitalisations for a pre-existing condition (including elective procedures that have not worsened) do not constitute an SAE.

***Medical judgement should be exercised in deciding whether an AE/AR is serious in other situations. Important AE/ARs that are not immediately life-threatening or do not result in death or hospitalisation but may jeopardise the subject or may require intervention to prevent one of the other outcomes listed in the definition above, should also be considered serious.

Pregnancy occurring in a STAMPEDE patient's partner during the patient's participation in the trial, must be reported to the MRC CTU within the same timelines as an SAE and classified as an 'other important medical condition' on the SAE form. The outcome of a pregnancy should be followed up carefully and any abnormal outcome to the mother or child should be reported.

11.1.1 Trial-Specific Exemptions

Disease progression or death as a result of disease progression are not considered to be SAEs and should be reported on the STAMPEDE Progression Form or Death Form.

The following situations that fulfill the definition of an SAE are excluded from expedited notification on an SAE form and should be reported only on the STAMPEDE follow-up form:

- Elective hospitalisation and surgery for treatment of locally advanced or metastatic prostate cancer or its complications
- Elective hospitalisation to simplify treatment or procedures
- Elective hospitalisation for pre-existing conditions that have not been exacerbated by trial treatment

11.2 INSTITUTION/INVESTIGATOR RESPONSIBILITIES

All non-serious AEs/ARs, whether expected or not, should be recorded in the toxicity (symptoms) section of the Follow-up CRF and sent to the MRC CTU within one month of the form being due. SAEs/SARs should be notified to the MRC CTU as described below.

The severity (i.e. intensity) of all AEs/ARs (serious and non-serious) in this trial should be should be graded using Common Terminology Criteria for Adverse Events (CTCAE) v3.0 (ctep.cancer.gov/reporting/index.html). A flowchart is given in **Appendix I** to help explain the notification procedures. Any questions concerning this process should be directed to the MRC CTU in the first instance.

11.2.1 Investigator Assessment

11.2.1.A Seriousness

When an AE/AR occurs the investigator responsible for the care of the patient must first assess whether the event is serious using the definitions given in **Table 12**. If the event is serious and not exempt from expedited reporting, then an SAE form must be completed and the MRC CTU notified.

11.2.1.B Causality

The Investigator must assess the causality of all serious events/reactions in relation to the trial therapy using the definitions in **Table 13**. There are 5 categories: unrelated, unlikely, possible, probable and definitely related. If the causality assessment is unrelated or unlikely to be related the event is classified as a SAE. If the causality is assessed as either possible, probable or definitely related then the event is classified as a SAR.

Table 13: Assigning type of SAE through causality

RELATIONSHIP	DESCRIPTION	EVENT TYPE
Unrelated	There is no evidence of any causal relationship	Unrelated SAE
Unlikely	There is little evidence to suggest there is a causal relationship (e.g. the event did not occur within a reasonable time after administration of the trial medication). There is another reasonable explanation for the event (e.g. the patient's clinical condition, other concomitant treatment).	Unrelated SAE
Possible	There is some evidence to suggest a causal relationship (e.g. because the event occurs within a reasonable time after administration of the trial medication). However, the influence of other factors may have contributed to the event (e.g. the patient's clinical condition, other concomitant treatments).	SAR
Probable	There is evidence to suggest a causal relationship and the influence of other factors is unlikely.	SAR
Definitely	There is clear evidence to suggest a causal relationship and other possible contributing factors can be ruled out.	SAR

11.2.1.C Expectedness

If the event is a SAR the Investigator must assess the expectedness of the event. Please see Appendix G (Table G.2) for a list of expected toxicities associated with the drugs being used in this trial. If a SAR is assessed as being unexpected it becomes a SUSAR.

11.2.1.D Notification

Investigators must notify the MRC CTU of all SAEs occurring from the time of randomisation until 30 days after the last protocol treatment administration. SAEs occurring in patients randomised to Arm A must be reported until 2 years and 2 months or progression (whichever is sooner). SARs and SUSARs must be notified to the MRC CTU indefinitely (i.e. no matter when they occur after randomisation).

11.2.2 NOTIFICATION PROCEDURE

The SAE form must be completed by the Investigator (consultant named on the signature list and delegation of responsibilities log who is responsible for the patient's care), with due care being paid to the grading, causality and expectedness of the event as outlined above. In the absence of the responsible investigator the form should be completed and signed by a member of the site trial team. The responsible investigator should subsequently check the SAE form, make changes as appropriate, sign and then re-fax to the MRC CTU as soon as possible. The initial report shall be followed by detailed, written reports as appropriate.

Send the SAE form by fax to the MRC CTU. Fax Number: + 44 (0) 20 7670 4818. The STAMPEDE trial team will confirm receipt of the SAE report to the main point of contact via email

Follow-up: Patients must be followed-up until clinical recovery is complete and laboratory results have returned to normal or baseline, or until the event has stabilised. Follow-up should continue after completion of protocol treatment if necessary. Follow-up information should be noted on a

further SAE form by ticking the box marked 'follow-up' and faxing to the MRC CTU as information becomes available. Extra, annotated information and/or copies of test results may be provided separately. The patient must be identified by trial number, date of birth and initials only. The patient's name should not be used on any correspondence.

11.3 MRC CTU RESPONSIBILITIES

Medically qualified staff at the MRC CTU and/or the Chief Investigator (or a medically qualified delegate) will review all SAE reports received. The causality assessment given by the local Investigator at the hospital cannot be overruled and in the case of disagreement, both opinions will be provided in any subsequent reports.

The MRC CTU is undertaking the duties of trial sponsor and is responsible for the reporting of SUSARs and other SARs to the regulatory authorities (MHRA and competent authorities of other European member states and any other countries in which the trial is taking place) and the research ethics committees as appropriate.

The MRC CTU will also keep all investigators informed of any safety issues that arise during the course of the trial.

SAE REPORTING
Fax to 020 7670 4818 within 24 hours of becoming aware of the event

12 ETHICAL CONSIDERATIONS AND APPROVAL

12.1 ETHICAL CONSIDERATIONS

This is a randomised trial therefore neither the patients nor their physicians will be able to choose the patients' treatment. Treatment will be allocated randomly using a computer-based algorithm. This is to ensure that the groups of patients receiving each of the different treatments are similar.

Androgen deprivation therapy alone is the standard treatment for these forms of prostate cancer. Patients will be randomised to one or two of the newer treatments in combination with hormone treatment. The trial employs an unequal allocation ratio for efficiency, these are explained in detail in the patient information sheet.

The newer combined treatment options are being assessed in a detailed and systematic fashion in this trial. There is some evidence to suggest that the newer treatment options may have advantages over standard treatment (androgen deprivation therapy) alone with regards clinical outcome, but this is not confirmed and toxicity may be increased. This trial will follow a large group of men who have been randomly allocated to either the standard treatment (androgen deprivation therapy alone) or the newer combined treatment options in order to measure the benefits of the new treatments. The patients will also be followed-up for toxicity and safety issues, so that any benefits can be weighed against any negative aspects.

Patients participating in the trial will have some additional hospital visits and some extra blood samples taken compared to patients who are not participating in the trial, with the amount varying according to the allocated treatment. Sometimes the blood samples can be taken when the patient is attending hospital for treatment, anyway. On some of the trial arms, the patient may have to make additional visits to the hospital for the blood sample to be taken, although in some cases it may be possible for the blood sample to be taken in the GP's surgery. The additional visits and blood samples are to ensure that follow-up of patients is comparable in all the treatment groups. The blood samples will also be used for genetic and serum marker studies, where this information will be considered with clinical data. Blood samples will be link-anonymised. There will be no feedback to individual patients.

If new information emerges during the course of the trial which may affect the treatment or follow-up of patients who have joined the trial, information will be provided through by the trial team to all Principal Investigators. PIs have therefore the duty to inform patients in their care of any new information emerged using any appropriate channel (e.g. letter, communication at follow up clinic, etc).

12.2 ETHICAL APPROVAL

The protocol has a Favourable Opinion from an appropriate Research Ethics Committee, according to national guidelines. Additionally, each site must also obtain management permission for research (R&D approval) from the relevant host organisations before patients can be entered into the trial. The patient's informed consent to participate in the trial should be obtained after a full explanation has been given of the treatment options, including the conventional and generally accepted methods of treatment. Patient information sheets and patient consent forms are given in **Appendix B**.

The right of the patient to refuse to participate in the trial without giving reasons must be respected. After the patient has entered the trial, the clinician must remain free to give alternative treatment to that specified in the protocol, at any stage, if he feels it to be in the best interest of the patient. However, the reason for doing so should be recorded and the patient will remain within the trial for the purpose of follow-up and data analysis according to the treatment option to which he has been allocated. Similarly, the patient must remain free to withdraw at any time from the protocol treatment without giving reasons and without prejudicing his further treatment.

A statement of MRC policy on ethical considerations in clinical trials of cancer therapy, including the question of informed consent, is available from the MRC Head Office web site (http://www.mrc.ac.uk).

13 REGULATORY APPROVAL

This trial has been approved in the UK by the MHRA and will be conducted under a CTA (Ref: 00316/0026/001-0001) in the UK.

The trial has been approved in Switzerland by Swissmedic (Ref: 2009 DR 3235).

14 INDEMNITY

The MRC adheres to the principles of the Research Governance Framework for Health and Social Care (Department of Health, England). The MRC is not insured but it has indemnity arrangements in place such that public funding is provided to meet claims.

The likely scenarios in which the MRC might face claims for damages are set out below.

As Sponsor, the MRC accepts that it might face claims for damages where the MRC, or any of its employees, or any person formally acting with MRC's authority, have a negligent or have failed to adhere to the relevant guidelines/guidance, legislation or procedure on good practice in relation to medical research and that negligence or failure has caused or materially contributed to the personal injury suffered by the individual making the claim.

The MRC may consider an ex- gratia payment when a significant adverse reaction in the form of a personal injury has occurred which is likely to have been caused by, or materially contributed to, by participation to the research study.

Where studies are carried out in a hospital, the hospital continues to have a duty of care to a patient being treated within the hospital, whether or not the patient is participating in an MRC-supported study. MRC does not accept liability for any breach in the hospital's duty of care, or any negligence on the part of employees of hospitals. This applies whether the hospital is a NHS Trust or not.

The Swiss Group for Clinical Cancer Research (SAKK) have provided trial-specific insurance to provide indemnity for Swiss sites against claims relating to non-negligent harm.

15 FINANCE

STAMPEDE is funded by the Clinical Trials Advisory Awards Committee (CTAAC) on behalf of Cancer Research UK; it is also funded by the MRC through the MRC Clinical Trials Unit. The trial has National Cancer Research Network (NCRN) approval and, therefore, local NCRN funds may be available at each centre to support entry of patients into this trial.

Zoledronic acid is manufactured by Novartis. Novartis have agreed to provide an educational grant to support the conduct of this study. Novartis have also agreed to supply the study drug, zoledronic acid free of charge for patients participating in the study.

Docetaxel is manufactured by Sanofi-Aventis Pharma. They have agreed to supply the study drug, docetaxel at a discounted rate for patients that are participating in the trial and to provide an educational grant to support the conduct of the study. The Department of Health has agreed to provide a central subvention as follow: £1,787 per patient randomised to arms C and E of the trial and prescribed docetaxel. This amount is payable in respect of a hospital trust randomising more than 3 patients. For more details contact the STAMPEDE Trial Manager.

Celecoxib is manufactured by Pfizer. They agreed to supply free drug and to provide funds to distribute drug to participating sites.

Abiraterone is manufactured by Janssen Pharma PV (pharmaceutical companies of Johnson & Johnson). They have agreed to provide free drug and funds to distribute drug to participating sites and to help support the conduct and management of the trial.

16 TRIAL COMMITTEES

16.1 TRIAL MANAGEMENT GROUP (TMG)

A Trial Management Group (TMG) has been formed comprising the Chief Investigator, other co-investigators and members of the MRC CTU. The membership of the TMG may be expanded if other groups of trialists wish to participate. It will also be amended during the trial if other circumstances require e.g. retirement.

The TMG will be responsible for the day-to-day running and management of the trial and will meet by teleconference at least 3 monthly and in person as needed. The TMG members are detailed in **Appendix K**.

Further details of TMG functioning are provided in the TMG charter (available on request).

16.2 TRIAL STEERING COMMITTEE (TSC)

A Trial Steering Committee (TSC) has been formed to provide overall supervision for the trial and provide advice through its independent chair. The ultimate decision for the continuation of the trial lies with the TSC. The TSC will meet regularly.

Further details of TSC functioning are provided in the TSC charter (available on request)

16.3 INDEPENDENT DATA MONITORING COMMITTEE (IDMC)

An Independent Data Monitoring Committee (IDMC) has been formed. The IDMC will be the only group who sees the confidential, accumulating data to the trial. Reports to the IDMC will be produced by the MRC CTU. The IDMC will meet within 6 months of the trial opening with the frequency of meetings dictated by the IDMC. The IDMC will consider data in accordance with the analysis plan (see **Section 9.5**) and will be advisory to the TSC. The IDMC can recommend premature closure or reporting of the trial, or that recruitment to any research arm be discontinued.

From version 8.0 onwards, any recommendation from the IDMC to stop recruitment to one or more trial arms will be acted upon immediately, pending ratification from the TSC. As this period between meetings should be very short, sites would not be notified until after the TSC have made a decision. IDMC recommendations based on emerging safety issues would be discussed with sites promptly.

Further details of IDMC functioning and the procedures for interim analysis and monitoring are provided in the IDMC charter (available on request).

Figure 10: Diagram of relationships between trial committees

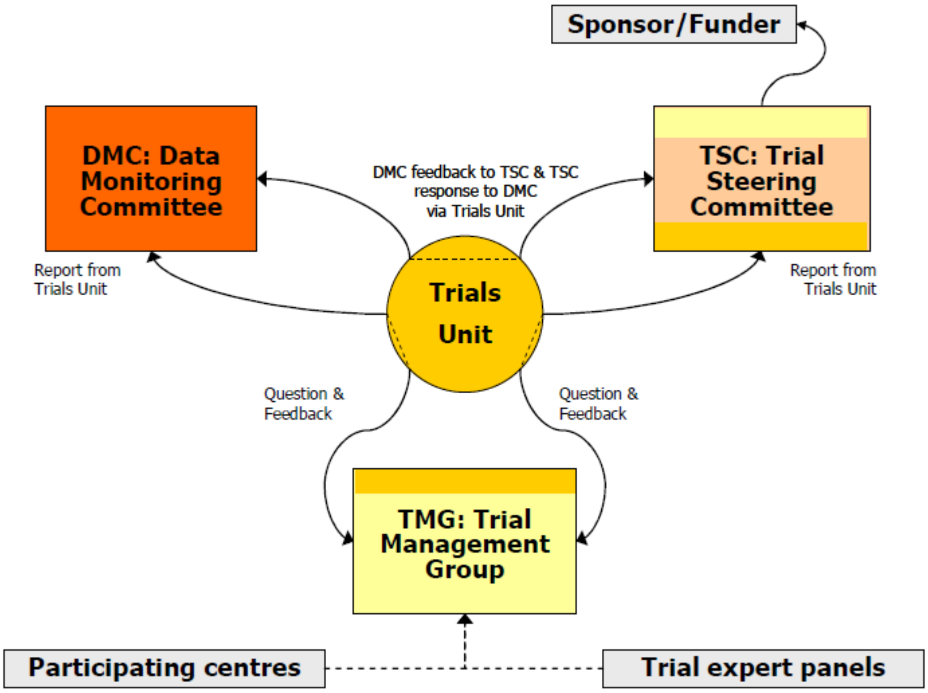

17 ANCILLARY STUDIES

17.1 QUALITY OF LIFE

A quality of life (QL) study is being performed to assess the impact of each treatment arm on the quality of patient's lives and participation in this study was limited to the first 700 patients recruited (this was reached in September 2008) patients. The QL study re-opened from the implementation of version 8.0 of the protocol. The EORTC QLQ-C30 with the prostate-specific module QLQ PR25 will be used. Key items for assessment are pain reduction for patients with metastatic disease and urinary symptoms for patients with locally advanced disease. In addition specific hypotheses will be generated for each of the research arms. The EuroQol (EQ-5D) (49) will be used in the study as a generic measure of health-related quality of life which can be linked to public preferences. These data will be used to calculate quality-adjusted life-years as part of the economic evaluation (see **Section 17.2**). Patients who were recruited into the QL study, should continue on the study throughout the trial. Questionnaires should be self-administered, although it is recommended that a key person (e.g. research nurse) at each centre be responsible for the data collection to optimise compliance and completeness of the data.

The QL and the HE questionnaires should be completed without conferring with friends or relatives and all questions should be answered even if the patient feels them to be irrelevant.

The responsible person should check each questionnaire for its completeness, ensuring that the correct date of completion and patient identifiers are present. The research nurse should approach patients at appropriate clinical visits to complete a questionnaire. If no clinical visit is scheduled for the patient (with a window of 4 weeks around the expected date) the nurse should organise the completion of the questionnaire, by post or by a visit to the patient at home (or in a hospice).

17.2 HEALTH ECONOMICS

A health economics (HE) sub-study will be performed. Core resource use information will be collected, using CRFs on days in hospital (by speciality) and outpatient visits. Data being collected on concomitant medication will also be used in the economic analysis. Information on patients' use of primary care and community-based services will be collected as additional questions in the QL questionnaire. Costs will be calculated on the basis of representative UK unit costs at the point of analysis. Health outcomes will be assessed in terms of quality-adjusted life years (QALYs). Quality adjustments will be based on patients' responses to the EQ-5D health status measure which will be administered at baseline and each point of follow-up as part of the QL questionnaire. A cost-effectiveness analysis will compare all regimens that continue to recruit into their Activity Stage IV.

17.3 TRANSLATIONAL SUB-STUDIES

17.3.1 DNA ANALYSIS

Blood samples from as many patients as possible will be collected for future research. With patient consent, an additional droplet of blood sample will be collected and stored for DNA and protein analysis in order to try to identify molecular features of clinical significance.

Blood samples should be sent directly to the central laboratory on the FTA elute cards provided. Patient information sheets and consent forms which highlight this research are given in **Appendix B**, while details of specimen collection, posting and contact details are given in **Appendix D**.

17.3.2 Tissue Microarray

Patient consent will be sought to utilise paraffin embedded tissue for the construction of tissue microarrays from needle cores. One needle biopsy will be selected for microarray and the remaining tissue will be returned to the originating histopathologist. Given the entry criteria for the trial, the majority of patients will have extensive disease in the diagnostic needle core biopsies, in contrast to men with localised, low grade disease. Consequently, removal of one core is unlikely to compromise any subsequent histopathological assessment. Details regarding transfer of samples will be issued at the time of construction of the micro array. Additional analyses e.g. DNA extraction may also be performed on the tissue arrays.

18 PUBLICATION

The results from different centres will be analysed together and published as soon as possible. Individual clinicians must not publish data concerning their patients that are directly relevant to questions posed by the study until the TMG has published its report. The TMG together with the STAMPEDE collaborators will form the basis of the writing committee and decide on the nature of publications. Any release, of efficacy or safety data, presentation or publication will be agreed with the TSC according to the terms of their charter.

All publications will acknowledge the participating centres and clinicians, and these will be detailed in an appendix to the main report. Papers will have named authors determined by the TMG according to the following principles:

- To be as inclusive as possible where this is practicable
- To ensure that there is justification for anyone to be named as an author
- Reasons for nomination for authorship may include: trial design; grant holding; day-to-day trial oversight (TMG membership); analysis; discussion and interpretation of data; representation for key groups; active participation at large recruiting sites. It should be accepted that the people qualifying for authorship will vary over time. In addition, key positions will vary depending on the nature of the publication: clinical lead for clinical papers, statistician lead for methodology papers, translational papers may be lead by authors not on the main TMG if appropriate (e.g., the bone sub-study). In the event of any dispute related to authorship or data release, the TSC will be responsible for making the executive decision.

In the manuscript, a full list of sites and the number of patients recruited will be provided. In the presentations, this list of sites will also be shown. The term “the STAMPEDE investigators” will clearly be stated and relevant names included in the presentation credits.

19 PROTOCOL AMENDMENTS

19.1 PROTOCOL

19.1.1 Amendments Made To Sections In Protocol Version 1.0 (May 2004)

Administrative changes such as typos, word change etc.

Name additions/changes to:

TMG members

TSC members

IDMC members

'General Information' Section – additional information re. Abridged version of protocol

Section 1.2 – Figure 1, Celecoxib duration amended

Section 1.3 – Figure 2, addition of cardiovascular assessment form, name and timings amended

Section 2.3 – Docetaxel information updated

Section 2.4 – Additional text re dose and duration justification for Celecoxib use.

Section 3 – Title change and content updated

Section 4.2 – New exclusion criteria added

Section 4.3.1 – New investigations added and additional text re testosterone measurements and additional text re. prior celecoxib treatment

Section 6.1.4 – Celecoxib duration amended

Section 6.1.5 – Additional text re. Co-administration of docetaxel and bisphosphonates

Section 6.1.6 – Celecoxib duration amended

Section 6.2.2 – additional docetaxel information

Section 6.2.3 – addition of CV event history

Section 11 – Safety reporting updated

Section 12.1 – Additional text re. the collection of blood for genetic and serum marker studies

Section 15 – Additional information re. Central Subvention for docetaxel arms

19.1.2 Amendments Made To Sections In Protocol Version 1.1 (May 2005)

Section 6.2 Administration and Dose Modifications, subsection 6.2.1 Zoledronic Acid

19.1.3 Amendments Made To Sections In Protocol Version 2.0 (Jun 2005)

General Information section – SAE reporting fax number and timeframe added.

Section 1.2 – Addition of anti-androgen use for M0 patients as a method of HT

Section 1.2 – Increase in amount of blood needed & addition tissue sample request.

Section 1.3 Trial Documentation updated to include new table detailing trial documentation ahead of accreditation, the inclusion of the radiotherapy forms and correct case report form timings

Section 2.1 – Addition of anti-androgen use for M0 patients as a method of HT

Section 4.1.3 – Inclusion criteria Vii "Normal testosterone prior to hormone treatment" removed.

Section 4.1.3 - φnote has been omitted and moved to section 4.2 (see number 8)

Section 4.2 – Exclusion criteria added to exclude patients with active peptic ulceration, gastrointestinal bleeding and inflammatory bowel disease.

Section 4.2 – Exclusion Criteria added to exclude patients with planned major dental work

Section 4.3.1 - All blood test timelines changed from 14 days to 28 days.

Section 4.3.1 – Hormone Therapy pre-randomisation deadline extended from 4 weeks to 12 weeks.

Section 4.3.1 – Additional information regarding the use of NSAIDs and cox-2-inhibitors before coming on to the STAMPEDE study and once commenced on study treatment

Section 4.3.2 – Updated to ask for all vitamins and minerals the patient is taking to be recorded.

Section 4.3.3 – Updated to include the extra blood required and the request for consent of patients' tissue samples.

Section 6.1.1 – Addition of anti-androgen use for M0 patients as a method of HT

Section 6.1.6 – Addition of the calcium & vitamin name "calcichew".

Section 6.6.2 – asking also to collect vitamins and minerals under concomitant medication.

Section 6.6.3 – New section to inform investigators that patient's, who they wish to give radiotherapy to, are also eligible for STAMPEDE

Section 6.6.4 – New section to detail what data is being collected on the radiotherapy given to patients.

Section 7.1; figure 4 – Addition of radiotherapy form and in note, addition of AA alone

Section 7.1.2 – omission of repeated scans and x-rays at 24 weeks, also omitted in note under figure 4.

Chapter 11 – Safety reporting section updated

Section 17.3 – Increase in amount of blood needed & additional tissue sample request.

19.1.4 Amendments Made To Section In Protocol Version 3.0 (Jul 2006)

Front Cover - NCRN logo added for accuracy

Front Cover - Clafication that protocol developed with NCRI rather than on behalf of

Front Cover - Clarification the it is a 6 arm trial

General Information section - MRC CTU staff section updated

Section 1.2 – Statistics section updated.

Section 1.2 - Additional research paragragh updated to refelct additional studies and for clarification of terms

Section 1.2 - Blood collection volume changed to reflect new technique used

Section 1.3 (figure 3) - Table showing case report form scheduyle updated to reflect clarification of follow-up schedule and addition of new CRF (End of Treatment)

Section 2.2 - AS changed to HT (clarification of terms)

Section 2.3 - Updated in information in reagrd to use of docetaxel added to reflect up to date practice

Section 2.5 - Sub-headings numbered for consistancy

Section 3.0 - Information in reagrd to the Pilot Phase now written in past tense as Pilot Phase has now been completed

Section 4.1.1 - Inclusion criteria extended so that patinets who fulfil 2 out of the three of the first inclusion criteria can be eligible.

Section 4.3.1 - Change in time scales by which baseline investigations need to be completed.

Section 4.3.1 - Clarification that chest X-ray is only required if chest is not included in the CT

Section 4.3.1 - Removal of 12 week timeline for basline PSA test ot be performed. (Stipulation that it must be performed before start of HT)

Section 4.3.2 – Information added in regard to time allowed from randomisation to start of treatment

Section 4.3.3 - Additional research paragragh updated to refelct additional studies and for clarification of terms

Section 4.3.3 - Blood collection volume changed to reflect new technique used

Sections 6.1.2-6.1.6 - Androgen Supression replaced with hormone therapy for consistemcy of terms

Section 6.2.2 - '(Taxotere)' Removed for consistancy

Section 6.2.2 _ information added in regard to the need to closely monitor liver function prior to docetaxel adminstration

Section 7.1 - Page number reference updated

Section 7.1.1 - PSA measurement timings updated to accurately reflect follow-up schedule

Section 7.3 (Table 4) - Table and key updated to accurately refelct follow-up schedule and to include information about new CRFs and removal of withdrawal CRF

Section 8 - Rewording for clarifcation of definition of trial withdrawal

Section 8.1 - Instruction that withdrawal from trial treatment should be recorded on End of Treatment Form rather than withdrawal form

Section 8.1 - Information updated to emphasise that trial treatment must be discontinued following a progression

Section 8.2- Information added in regard to patient transfers

Section 8.3 - Instruction that withdrawal from trial completely must be notified in writing to the MRC CTU rather than included on withdrawal form

Section 9 and Summary – Target event numbers updated to reflect the slightly revised numbers obtained by using –nstage- which is the new, recommended program for MAMS trials

Sections 11.1 and 11.2 - Form numbers removed to allow for future changes in numbering

Section 11.2 – Reference to toxicity grading website added

Section 11.2.1 - Reference to table in appendix G added

Section 12.2 - 'Suggested' removed from 'Suggested patient information sheets'

Section 13 - CTA reference added

Section 17.3 - Information added to reflect new blood collection method for DNA analysis and in regard to additional translational studies for which funding has recently been approved

19.1.5 Amendments Made To Protocol Version 4.0 (Dec 2007)

General Information Section - Randomisation and SAE reporting details sections clarified

Section 1.2 and throughout protocol - Efficacy Stages 1-111 renamed to Activity Stages 1-111 for accuracy and clarity

Section 1.2 - Follow schedule corrected

Section 4.1.2 - Inclusion criteria widened to include high risk relapsing patients, that would not have met the previous PSA based criteria

Section 4.1.3 - Note added to reference location of WHO performance status definitions

Section 4.2 - Notes added to reference locations of toxicity gradings and NYHA classifications

Section 4.3.1 - Timings of baseline scan information changed to accurately reflect most common current practice

Section 6.1.1 - Information about use of LHRH antagonists to ensure that the protocol accurately reflects current and future practice

Section 6.1.1 - Information about suggested duration of hormone therapy added to ensure that the protocol accurately reflects current practice

Section 6.2.2 - Additional information added about the timing of liver function tests prior to docetaxel administration added for clarity

Section 6.6.4 - Information on radiotherapy data collection added

Section 7.1.1 - Erroneous information about the timing of PSA measurements removed

Figure 3 - Moved to new section in protocol for clarity and extended to include current information on data collection

Figure 3b - Added to describe how extent of data collection during follow-up should change, post treatment and post progression

Figure 4 - Notes added to explain the changes in data collected at follow-up and to information that the quality of life study will be applicable to the first 700 patients randomised only

Figure 4 - Note added to include palliative radiotherapy CRF

Section 11.3 - SAE reporting information updated

Section 19 - Protocol amendments list updated

19.1.6 Amendments Made To Protocol Version 5.0 (Aug 2008)

1. General Information Section – Randomisation phone line number updated – non UK extension added

2. Section 3 – Information about QL study removed to reflect closure of QL study after first 700 patients

3. Section 4.2 – Exclusion criteria clarified to explain that only patients with severe poor cardiovascular history should be excluded

4. Section 4.3.1 – Information on co-administration of NSAIDS with celecoxib changed based on clinical advice.

5. Section 5 - Randomisation phone line number updated – non UK extension added

6. Section 6.2.1. – Information added to clarify that patients who develop an osteonecrosis of the jaw should stop zoledronic acid treatment

7. Section 6.2.3 – 'severe' text added to acurately reflect which patients should be excluded based on their cardiovascular history

8. Section 7.1.2 – Defination of disease progression extended for clarity

9. Figure 3 – Updated to include reference to newly created skeltal related event form

10. Figure 4 – Previous error in table amended to show that the 4th Zoledronic Acid form that is submitted contains information about 3 cycles rather than 2 as previously indicated

11. Table 4 – 'Other important medical condition' added to definition of serious in the SAE section, to accuratly reflect SAE form and current practice

12. Section 11.1 – Information added on reporting or pregnancies

13. Section 17 - Information about QL study removed to reflect closure of QL study after first 700 patients

19.1.7 Amendments Made To Protocol Version 6.0 (Jul 2009)

1. General Information Section – Trial Pharmacist removed and changes of:

Co-Investigator

Patient Representatives

Trial Manager

Data Manager

General Information Section - Coordinating Centre – address change

General Information Section – change of Sponsor address

Section 1.1 – ratio of patients randomised to the investigational arms updated

Section 1.2 – figure 1b added to clarify trial design from Apr-2011 onwards

Section 1.2 – paragraph added to explain trial changes after the second activity analysis

Section 1.2 – wording added to clarify that QL data only collected for first 700 patients randomised

Section 1.3 – SSA Favourable Opinion removed from list of trial documentation required ahead of site accreditation

Section 2.1 – Amount of men diagnosed with prostate cancer annually updated

Section 2.4 –note added to explain completion of recruitment to celecoxib- containing arms

Section 2.5.2 - note added to explain completion of recruitment to celecoxib- containing arms

Section 3 – SSA Favourable Opinion removed

Section 4.2 – Exclusion criterion xiii greyed out

Section 4.3.1 – paragraph removed regarding potential randomisation to celecoxib-containing arms

Section 5 – Randomisation instructions expanded to exclude public holidays or dates when notice has been given by the CTU

Section 6.1.4 – formatting changed to grey font to reflect recruitment completion for arm D

Section 6.1.6 - formatting changed to grey font to reflect recruitment completion for arm F

Section 6.2.3 – recruitment note added

Section 6.6.3 – radiotherapy statement changed to reflect data from recent trials

Section 7.1.2 – removal of reference to SRE- specific CRF

Section 7.3 – Figure 3 - Addition of Bone Density Risk Factor Form and BMD sub-study assessment forms to summary of timing table

Section 7.3 – Figure 4 – Weeks added to timings of assessments post 2 years

Section 7.3- Figure 4 – note added to explain recruitment completion for arms D and F

Section 12.1 – Wording changed to reflect change to randomisation allocation ratio

Section 12.1 – Addition of statement regarding new information emerging during the trial

Section 12.2 – Reference to SSA removed

Section 16.3 – Statement added regarding actioning IDMC recommendation ahead of TSC ratification

19.1.8 Amendments Made To Protocol Version 7.0 (Jul 2011)

1. General Information Section- SAE reporting fax number corrected

2. Section 11- SAE reporting fax number corrected

19.1.9 Amendments Made To Protocol Version 7.1 (Jul 2011)

Throughout protocol – numbering has been updated in some sections new accommodate new information that has been added.

General Information Section – contact details updated

General Information Section – Funding information updated to include involvement from additional company

General Information Section – Wording on compliance and regulations updated to reflect current MRC CTU standard wording

General Information Section – Abbreviations list updated

Section 1.1 – The number of investigational agents being studied updated from three to four

Section 1.1 – Information regarding celecoxib updated to reflect that recruitment to these arms was discontinued in Apr-2011

Section 1.1 – Information about new IMP, Abiraterone inserted

Section 1.1 – Sample size and trial duration information updated to reflect changes brought about by additional trial arm

Section 1.2 – Summary information updated to reflect the discontinuation of recruitment to celecoxib arms and the addition of abiraterone

Figures 1a, b and c - Updated to reflect the discontinuation of recruitment to celecoxib arms and the addition of abiraterone

Section 1.2 – Information on trial stages updated to reflect changes brought about by additional trial arm

Section 1.2 – Information updated regarding the re-opening of the quality of life sub-study from implementation of protocol version 8.0

Section 2.1 – Wording related to hormone therapy updated for clarity

Section 2.1 - Updated to reflect the discontinuation of recruitment to celecoxib arms and the addition of abiraterone

Section 2.2 – Updated references added

Section 2.3 – Updated references added

Section 2.5 – Section added to give background information on new IMP, abiraterone

Section 2.6.1 – Updated references added

Section 2.7 – Section added to give information regarding radiotherapy which is to be given as part of standard care following recently published trial data.

Section 3 – Wording updated regarding selection of investigators to reflect current MRC CTU practice

Section 4.1 – Inclusion criteria updated with new criterion regarding radiotherapy use

Section 4.1 - Inclusion criteria updated with new criterion regarding contraceptive use

Section 4.1 – Wording of inclusion and exclusion criteria updated for clarity

Section 4.1 – Exclusion criteria updated with new criterion regarding acceptable liver function for trial entry

Section 4.1 – Exclusion criteria updated with specifics related to blood pressure levels

Section 4.1 - Exclusion criteria updated with new criterion regarding concomitant medications

Section 4.1 - Exclusion criteria updated with new criterion regarding prior treatment with abiraterone

Section 4.1 - Exclusion criteria updated with new criterion regarding prior treatment with chemotherapy

Section 4.1 - Exclusion criteria updated with new criterion regarding prior treatment with zoledronic acid

Section 4.3 – Wording updated to reflect that patients who initially fail screening can be re-screened at a later date

Section 4.3.2 – Wording updated regarding prior anti-androgen and LHRH use updated for clarity

Section 5.1 – Co-enrolment guidelines information updated to describe newly created co-enrolment CRF

Section 6.1 – Trial treatment information updated to reflect the fact that anti-androgens alone will be no longer permitted as hormone therapy

Section 6.1.1 – Updated to describe patients for whom radiotherapy should be given as standard practice

Section 6.1.1 a and b - Sections added to give information regarding radiotherapy treatment

Section 6.1.1-6.1.6 – References to further sections updated

Section 6.1.7 – Section added to describe abiraterone treatment

Section 6.2.4 - Section added to describe abiraterone treatment

Section 6.6 - Section added to give information regarding radiotherapy treatment

Section 7.1.1 – Reference to blood being taken at patient's home removed as this does not occur in practice

Section 7.1.2 – Wording updated regarding the reporting of biochemical failures for clarity

Section 7.1.2 – Wording updated regarding skeletal-related events for clarity

Section 7.1.3 – Section added to describe additional assessments required related to abiraterone treatment

Section 7.1.4 – Section added to provide information on when treatment should commence

Figure 4 – Updated for clarity regarding return of BMD sub-study forms, the addition the co-enrolment CRF and the description of the re-opening of the QoL Sub-study.

Figure 5 – Updated with reference to abiraterone and co-enrolment form

Section 7.3 - Wording on trial closure updated to reflect current MRC CTU standard wording

Section 8.1 – Additional criteria for definition of progression added for clarity

Section 8.1 – Definition of progression for abiraterone patients added.

Section 9 – Statistical information updated to describe the addition of the new trial arm

Section 11 – Safety reporting wording updated for clarity

Section 11 – SAE reporting fax number updated

Section 12 – Ethical information updated to describe the unequal randomisation allocation ratio

Section 12 – Ethical information updated to describe that the visit schedule will vary according to trial arm

Section 12.2 – Wording updated to reflect international participation in the trial

Section 13 – Wording updated to reflect international participation in the trial

Section 14 – Wording updated to reflect international participation in the trial

Section 15 - Updated to reflect the discontinuation of recruitment to celecoxib arms and the addition of abiraterone

Section 16 – Reference to trial committee charters added for information

Section 17.1 – Information added to reflect re-opening of quality of life sub-study

Section 17.2 – Timing of health economics analysis updated to previous error

Section 18 – Information on publication policy expanded for clarity

Section 19 – Information regarding amendments to protocol appendices moved to the separate appendices document

Section 20 – References extensively updated

19.1.10 Amendments Made To Protocol Version 8.0 (Sep 2011)

Throughout protocol – numbering, sections headings, tables, figures and bibliographical references have been updated in some sections to accommodate new information that has been added

Throughout protocol – Androgen Deprivation Therapy has replaced Hormone Therapy as deemed more representative of the type of hormone therapy used in the study

General Information Section – New staff members of the MRC CTU and Co-Investigators added and contact details updated

General Information Section – Abbreviations list updated

Section 1.1 – Information regarding the new research radiotherapy treatment inserted

Section 1.1 – Information regarding docetaxel updated

Section 1.2 – Wording updated to reflect the addition of the new research comparison arm

Section 1.3 – Additional criteria for the re-accreditation of participating centres (for protocol version 9.0 only)

Section 2.1.1 – Wording updated to clarify the use of anti-androgen in trial patients

Section 2.1.2 – Information added to describe the rationale for the RT comparison arm

Section 2.8 – Information added to describe research RT treatment to prostate for patients with newly diagnosed metastatic disease

Section 3.1 – Information added to describe RT Quality Assurance procedures and centre accreditation

Section 4.1.1 to 4.1.3 – Wording updated to clarify inclusion criteria for all patients groups (newly diagnosed non-metastatic, metastatic and relapsing patients)

Section 4.2 – Clarification added on cardiovascular exclusion criteria

Section 4.2 – New exclusion criterion added concerning patients with prior exposure to hormone therapy

Section 4.2 – New exclusion criterion added to reflect the addition of the new RT comparison arm

Section 4.4.1 – Clarification added regarding pre-randomisation checks

Section 4.4.2 – Clarification added regarding permissible hormone therapy duration prior to randomisation

Section 4.4.5 – Information added regarding starting research radiotherapy treatment

Section 4.4.6 – Information updated on concomitant medications

Section 5 – Clarification regarding randomisation allocation added to reflect the addition of the new RT research arm

Section 6.1.8 – Information added to describe the administration of research radiotherapy

Section 6.2.1 – Clarification added regarding the measurement of serum creatinine levels prior to the administration of zoledronic acid

Section 6.2.3 – Clarification regarding the completion of recruitment to the celecoxib containing arms

Section 6.25 – Information added regarding the administration of research radiotherapy treatment

Section 6.6 – Clarification incorporated to describe the administration of standard-of-care radiotherapy

Section 7.1.4 – Information added regarding data collection and non administration of standard radiotherapy

Section 7.2 – Section updated to include new treatment specific CRFs and timing of CRFs

Section 8.1 – Clarification added for the criteria to stop treatment for patients randomised to arm G

Section 8.2 – Section expanded to include additional details on study patient transfer to different centres

Section 8.3 – Additional sentence inserted to reinforce the importance of compliance with follow up assessments

Section 9.1 – Additional paragraph inserted to clarify the method of randomisation and allocation distribution in the light of the introduction of the new RT arm

Section 9.4 – Wording updated to clarify the assessment of safety data

Section 9.5.4 – Wording updated concerning the end of randomisations to arm G

Section 9.6 to 9.6.4 – Section added describing sample size issues and trial stages for arm H

Section 9.8 – Clarification on intermediate stopping guidelines

Section 9.9 – Clarification on the outline analysis plan

Section 11 – Information on safety reporting updated to reflect the addition of the research RT comparison arm

Section 11 – Clarification added regarding arm A safety reporting timelines

Section 12.1 – Clarification added regarding the Principal Investigator’s responsibilities

Section 14 – Indemnity section updated to reflect current MRC policy

Section 16 – Clarification regarding TMG membership

Section 17.3 – Section on Bone Mineral Density sub-study removed

Section 19 – Information regarding amendments to protocol appendices moved to the separate appendices document

Section 20 – References updated

19.1.11 Amendments Made To Protocol Version 9.0 (Oct-2012)

Throughout protocol – numbering, sections headings, tables, figures and bibliographical references have been updated in some sections to accommodate the completion of recruitment to original research arms B, C and E.

Throughout protocol – Tenses have been changed to reflect activities that were in the future and which have now been passed.

Section 1 – Figure added and clarifications added to each figure

Section 2 – Previous reference 8 removed

Section 4 – Clarification of acceptable alternatives to bone scans

Section 17.3.2 – Clarification that DNA may be extracted

20 REFERENCES

1. James ND, Sydes MR, Mason MD, Clarke NW, Anderson J, Dearnaley DP, et al. Celecoxib plus hormone therapy versus hormone therapy alone for hormone-sensitive prostate cancer: first results from the STAMPEDE multiarm, multistage, randomised controlled trial. The lancet oncology. 2012;13(5):549-58. Epub 26-Mar-2012.

2. Cancer Research UK. CancerStats Key Facts: Prostate Cancer. Cancer Research UK. 2011.

3. Sharifi N, Gulley JL, Dahut WL, Sharifi N, Gulley JL, Dahut WL. An update on androgen deprivation therapy for prostate cancer. Endocrine-Related Cancer. 2010;17(4):R305-15.

4. Widmark A, Klepp O, Solberg A, Damber JE, Angelsen A, Fransson P, et al. Endocrine treatment, with or without radiotherapy, in locally advanced prostate cancer (SPCG-7/SFUO-3): an open randomised phase III trial. Lancet. 2009;373(9660):301-8. Epub 2008/12/19.

5. Warde PR, Mason MD, Sydes MR, Gospodarowicz MK, Swanson GP, Kirkbride P, et al. Intergroup randomized phase III study of androgen deprivation therapy (ADT) plus radiation therapy (RT) in locally advanced prostate cancer (CaP) (NCIC-CTG, SWOG, MRC-UK, INT: T94-0110; NCT00002633). J Clin Oncol. 2010;28(18s Supplement: Proceedings of ASCO 2010):Abstr CRA4504.

6. Warde P, Mason M, Ding K, Kirkbride P, Brundage M, Cowan R, et al. Survival Benefit with Combined Androgen Deprivation and Radiation Therapy in Locally Advanced Prostate Cancer – Results of a Phase III Trial. The Lancet. 2011 - in press.

7. Mason M, Sydes M, Parulekar W, Parmar M, Anderson J, Barber J, et al. Final analysis of intergroup randomized phase III study of androgen deprivation therapy (ADT) + radiation therapy (RT) in locally advanced prostate cancer (CaP) (NCIC-CTG, SWOG, MRC-UK, INT: T94-0110). National Cancer Research Institute (NCRI) Cancer Conference 2012. 2012;2012.

8. Saad F, Colombel M. Management of castration-resistant prostate cancer: bisphosphonates and emerging therapies. Expert Review of Anticancer Therapy. 2010;10(12):1991-2002.

9. Sartor O, Halstead M, Katz L, Sartor O, Halstead M, Katz L. Improving outcomes with recent advances in chemotherapy for castrate-resistant prostate cancer. Clinical Genitourinary Cancer. 2011;8(1):23-8.

10. Attard G, Richards J, de Bono JS, Attard G, Richards J, de Bono JS. New strategies in metastatic prostate cancer: targeting the androgen receptor signaling pathway. Clinical Cancer Research. 2011;17(7):1649-57.

11. Lipton A. Improving progression-free and overall survival in patients with cancer: a potential role for bisphosphonates. Expert Opinion on Pharmacotherapy. 2011;12(5):749-62.

12. Lipton A, Small E, Saad F, Gleason D, Gordon D, Smith M, et al. The new bisphosphonate, Zometa (zoledronic acid), decreases skeletal complications in both osteolytic and osteoblastic lesions: a comparison to pamidronate. [Review] [55 refs]. Cancer Investigation. 2002;20 Suppl 2:45-54.

13. Green JR, Muller K, Jaeggi KA. Preclinical pharmacology of CGP 42'446, a new, potent, heterocyclic bisphosphonate compound. Journal of Bone & Mineral Research. 1994;9(5):745-51.

14. Major P, Lortholary A, Hon J, Abdi E, Mills G, Menssen HD, et al. Zoledronic acid is superior to pamidronate in the treatment of hypercalcemia of malignancy: a pooled analysis of two randomized, controlled clinical trials. J Clin Oncol. 2001;19(2):558-67. Epub 2001/02/24.

15. Costa L, Harper P, Coleman RE, Lipton A, Costa L, Harper P, et al. Anticancer evidence for zoledronic acid across the cancer continuum. Critical Reviews in Oncology-Hematology. 2011;77 Suppl 1:S31-7.

16. Rosen LS, Gordon D, Kaminski M, Howell A, Belch A, Mackey J, et al. Long-term efficacy and safety of zoledronic acid compared with pamidronate disodium in the treatment of skeletal complications in patients with advanced multiple myeloma or breast carcinoma: a randomized, double-blind, multicenter, comparative trial. Cancer. 2003;98(8):1735-44. Epub 2003/10/10.

17. Rosen LS, Gordon DH, Dugan W, Jr., Major P, Eisenberg PD, Provencher L, et al. Zoledronic acid is superior to pamidronate for the treatment of bone metastases in breast carcinoma patients with at least one osteolytic lesion. Cancer. 2004;100(1):36-43. Epub 2003/12/24.

18. Saad F, Gleason D, Murray R, Tchekmedyian S, Venner P, Lacombe L, et al. A Randomized, Placebo-Controlled Trial of Zoledronic Acid in Patients With Hormone-Refractory Metastatic Prostate Carcinoma Journal of the National Cancer Institute. 2002;94(19).

19. Dearnaley DP, Sydes MR, Mason MD, Stott M, Powell CS, Robinson AC, et al. A double-blind, placebo-controlled, randomized trial of oral sodium clodronate for metastatic prostate cancer (MRC PR05 Trial). J NatlCancer Inst. 2003;95(17):1300-11.

20. Petrylak DP, Tangen CM, Hussain MH, Lara PN, Jr., Jones JA, Taplin ME, et al. Docetaxel and estramustine compared with mitoxantrone and prednisone for advanced refractory prostate cancer. N Engl J Med. 2004;351(15):1513-20. Epub 2004/10/08.

21. Tannock IF, de Wit R, Berry WR, Horti J, Pluzanska A, Chi KN, et al. Docetaxel plus prednisone or mitoxantrone plus prednisone for advanced prostate cancer. N Engl J Med. 2004;351(15):1502-12. Epub 2004/10/08.

22. Hawkey CJ. COX-2 inhibitors.[comment]. Lancet. 1999;353(9149):307-14.

23. Taketo MM. Cyclooxygenase-2 inhibitors in tumorigenesis (Part II). Journal of the National Cancer Institute. 1998;90(21):1609-20.

24. Taketo MM. Cyclooxygenase-2 inhibitors in tumorigenesis (Part I). Journal of the National Cancer Institute. 1998;90(20):1529-36.

25. Nelson JE, Harris RE. Inverse association of prostate cancer and non-steroidal anti-inflammatory drugs (NSAIDs): results of a case-control study. Oncology Reports. 2000;7(1):169-70.

26. Eberhart CE, Coffey RJ, Radhika A, Giardiello FM, Ferrenbach S, DuBois RN. Up-regulation of cyclooxygenase 2 gene expression in human colorectal adenomas and adenocarcinomas. Gastroenterology. 1994;107(4):1183-8.

27. Roberts RO, Jacobson DJ, Girman CJ, Rhodes T, Lieber MM, Jacobsen SJ. A population-based study of daily nonsteroidal anti-inflammatory drug use and prostate cancer. [see comments.]. Mayo Clinic Proceedings. 2002;77(3):219-25.

28. Basler JW, Piazza GA, Basler JW, Piazza GA. Nonsteroidal anti-inflammatory drugs and cyclooxygenase-2 selective inhibitors for prostate cancer chemoprevention. Journal of Urology. 2004;171(2 Pt 2):S59-62; discussion S-3.

29. Masferrer JL, Leahy KM, Koki AT, Zweifel BS, Settle SL, Woerner BM, et al. Antiangiogenic and antitumor activities of cyclooxygenase-2 inhibitors. Cancer Research. 2000;60(5):1306-11.

30. Steinbach G, Lynch PM, Phillips RK, Wallace MH, Hawk E, Gordon GB, et al. The effect of celecoxib, a cyclooxygenase-2 inhibitor, in familial adenomatous polyposis. New England Journal of Medicine. 2000;342(26):1946-52.

31. Solomon SD, McMurray JJ, Pfeffer MA, Wittes J, Fowler R, Finn P, et al. Cardiovascular risk associated with celecoxib in a clinical trial for colorectal adenoma prevention. New England Journal of Medicine. 2005;352(11):1071-80.

32. Danila DC, Morris MJ, de Bono JS, Ryan CJ, Denmeade SR, Smith MR, et al. Phase II multicenter study of abiraterone acetate plus prednisone therapy in patients with docetaxel-treated castration-resistant prostate cancer. J Clin Oncol. 2010;28(9):1496-501. Epub 2010/02/18.

33. Reid AH, Attard G, Danila DC, Oommen NB, Olmos D, Fong PC, et al. Significant and sustained antitumor activity in post-docetaxel, castration-resistant prostate cancer with the CYP17 inhibitor abiraterone acetate. J Clin Oncol. 2010;28(9):1489-95. Epub 2010/02/18.

34. Ryan CJ, Smith MR, Fong L, Rosenberg JE, Kantoff P, Raynaud F, et al. Phase I clinical trial of the CYP17 inhibitor abiraterone acetate demonstrating clinical activity in patients with castration-resistant prostate cancer who received prior ketoconazole therapy. Journal of Clinical Oncology. 2010;28(9):1481-8.

35. de Bono JS, Logothetis CJ, Molina A, Fizazi K, North S, Chu L, et al. Abiraterone and increased survival in metastatic prostate cancer. New England Journal of Medicine. 2011;364(21):1995-2005.

36. Ryan CJ, Smith MR, de Bono JS, Molina A, Logothetis C, de Souza PL, et al. Interim analysis (IA) results of COU-AA-302, a randomized, phase III study of abiraterone acetate (AA) in chemotherapy-naive patients (pts) with metastatic castration-resistant prostate cancer (mCRPC). J Clin Oncol. 2012;30(Suppl):Abstr LBA4518.

37. Attard G, Reid AH, de Bono JS, Attard G, Reid AHM, de Bono JS. Abiraterone acetate is well tolerated without concomitant use of corticosteroids. Journal of Clinical Oncology. 2010;28(29):e560-1; author reply e2.

38. Flanigan RC, Salmon SE, Blumenstein BA, Bearman SI, Roy V, McGrath PC, et al. Nephrectomy followed by interferon alfa-2b compared with interferon alfa-2b alone for metastatic renal-cell cancer. N Engl J Med. 2001;345(23):1655-9. Epub 2002/01/05.

39. Mickisch GH, Garin A, van Poppel H, de Prijck L, Sylvester R. Radical nephrectomy plus interferon-alfa-based immunotherapy compared with interferon alfa alone in metastatic renal-cell carcinoma: a randomised trial. Lancet. 2001;358(9286):966-70. Epub 2001/10/05.

40. Warde P, Mason M, Ding K, Kirkbride P, Brundage M, Cowan R, et al. Combined androgen deprivation therapy and radiation therapy for locally advanced prostate cancer: a randomised, phase 3 trial. The Lancet. 2011;378:2104-11. Epub 03-Nov-2011.

41. Neville-Webbe HL, Holen I, Coleman RE. The anti-tumour activity of bisphosphonates. Cancer Treatment Reviews. 2002;28(6):305-19.

42. Witters LM, Crispino J, Fraterrigo T, Green J, Lipton A. Effect of the combination of docetaxel, zoledronic acid, and a COX-2 inhibitor on the growth of human breast cancer cell lines. American journal of clinical oncology. 2003;26(4):S92-7. Epub 2003/08/07.

43. Kouloulias V, Giraud J-Y, Davis BJ, Dusserre A, Zurlo A, Bolla M. Quality assurance of the 22961 EORTC trial. A phase III study of the optimal combination of hormonal adjuvant treatment by LHRH analogue and radiation therapy for the management of locally advanced prostate cancer: the dummy run. 2004(0167-8140 (Print)).

44. Royston P, Parmar MKB, Qian W. Novel designs for multi-arm clinical trials with survival outcomes with an application in ovarian cancer. Statistics in Medicine. 2003;22(14):2239-56.

45. Royston P, Barthel FMS, Parmar MKB, Choodari-Oskooei B, Isham V. Designs for clinical trials with time-to-event outcomes based on stopping guidelines for lack of benefit. Trials. 2011;12(1):81.

46. Royston P. nstage: MAMS trial sample size calculator. MRC Clinical Trials Unit, London2009.

47. Haybittle JL. Repeated assessment of results in clinical trials of cancer treatment. The British journal of radiology. 1971;44(526):793-7. Epub 1971/10/01.

48. Peto R, Pike MC, Armitage P, Breslow NE, Cox DR, Howard SV, et al. Design and analysis of randomized clinical trials requiring prolonged observation of each patient. I. Introduction and design. British journal of cancer. 1976;34(6):585-612. Epub 1976/12/01.

49. Dolan P, Gudex C, Kind P, Williams A. Discussion Paper 138: A social tarif for EuroQol: results from a UK General Population Survey. . Centre for Health Economics. University of York, 1995.

STAMPEDE

Systemic Therapy in Advancing or Metastatic Prostate Cancer: Evaluation of Drug Efficacy

A multi-arm multi-stage randomised controlled trial

Version: 11
Date: 17-Sep-2013

MRC CTU ID:	PR08
ISRCTN #:	ISRCTN78818544
NCT #:	NCT00268476
EUDRACT #:	2004-000193-31
CTA #:	00316/0026/001-0001
MREC #:	04/MRE07/35

Authorised by:

Name:	PROFESSOR NICHOLAS D JAMES
Role:	CHIEF INVESTIGATOR
Name:	MATTHEW SYDES
Role:	TRIAL STATISTICIAN

GENERAL INFORMATION

This document was constructed using the MRC CTU Protocol Template Version 4.0. It describes the STAMPEDE trial, coordinated by the Medical Research Council (MRC) Clinical Trials Unit (CTU) at University College London (UCL), and provides information about procedures for entering patients into it. The protocol should not be used as an aide-memoire or guide for the treatment of other patients. Every care has been taken in drafting this protocol, but corrections or amendments may be necessary. These will be circulated to the registered investigators in the trial, but sites entering patients for the first time are advised to contact the Cancer Group, MRC CTU at UCL, London, UK, to confirm they have the most up-to-date version.

COMPLIANCE

The trial will be conducted in compliance with the approved protocol, the Declaration of Helsinki 1996, the principles of Good Clinical Practice (GCP), Commission Directive 2005/28/EC with the implementation in national legislation in the UK by Statutory Instrument 2004/1031 and subsequent amendments, the UK Data Protection Act (DPA number: Z5886415), and the National Health Service (NHS) Research Governance Framework for Health and Social Care (RGF). International sites will comply with the principles of GCP as laid down by the ICH topic E6 (Note for Guidance on GCP), Commission Directive 2005/28/EC (the European Directive 2001/20/EC [where applicable]) and applicable national regulations.

SPONSOR

Medical Research Council, 2nd Floor, David Phillips Building, Polaris House, North Star Avenue, Swindon, SN2 1FL, UK

FUNDING

Clinical Trials Advisory Awards Committee (on behalf of Cancer Research UK, Medical Research Council, and other charities) together with educational grants from Novartis, Sanofi-Aventis, Pfizer and Janssen Pharma NV.

AUTHORISATIONS AND APPROVALS

The following persons are authorised to sign the final protocol and protocol amendments for the sponsor: Professor Nicholas James (Chief Investigator) and Matthew Sydes (Trial Statistician).

TRIAL REGISTRATION

This trial has been registered with the ClinicalTrials.gov Clinical Trials Register, where it is identified as NCT00268476.

RANDOMISATIONS
To randomise, call MRC CTU, Monday to Friday 0900-1700
excluding public holidays or dates when notice has been given by the CTU.
Tel: +44 (0) 20 7670 4777

SAE REPORTING
Fax to 020 7670 4818 within 24 hours of becoming aware of the event

TRIAL ADMINISTRATION

COORDINATING SITE

MRC Clinical Trials Unit
Aviation House
Kingsway
London
WC2B 6NH
UK

Switchboard: 020 7670 4700
Fax: 020 7670 4818
Email: stampede@ctu.mrc.ac.uk

MRC CTU STAFF

Trial Manager:	Francesca Schiavone	Tel:	0207 670 4632
Trial Manager:	Alanna Brown	Tel:	0207 670 4882
Data Manager:	Dominic Hague	Tel:	0207 670 4947
Data Manager:	Katie Ward	Tel:	0207 670 4794
Data Manager:	Peter Vaughan	Tel:	0207 670 4655
Clinical Project Manager	Claire Amos	Tel:	0207 670 4771
Statistician:	Melissa Spears	Tel:	0207 670 4764
Senior Trial Statistician:	Matt Sydes	Tel:	0207 670 4798
Director of MRC CTU:	Max Parmar	Tel:	0207 670 4729
Trial Surgeon:	Alastair Ritchie	Tel:	Please contact STAMPEDE team

CHIEF INVESTIGATOR

Professor Nicholas James
School of Cancer Sciences
University of Birmingham
Edgbaston, Birmingham
B15 2TT
UK

Tel: 0121 414 4097/4474

Fax: 0121 414 3263

Email: n.d.James@bham.ac.uk
j.d.alonzo@bham.ac.uk(sec)

CO-INVESTIGATORS

Prof Noel Clarke, Urological Surgeon	Manchester, UK
Prof David Dearnaley, Oncologist	Sutton, UK
Prof Johann de Bono, Oncologist	Sutton, UK
Prof Malcolm Mason, Oncologist	Cardiff, UK
Dr Chris Parker, Oncologist	Sutton, UK
Dr Martin Russell, Oncologist	Glasgow, UK
Prof George Thalmann, Urological Surgeon	Bern, Switzerland
Daniel Aebersold, Oncologist	Bern, Switzerland

Patient Representative

Robin Millman, Patient

Health Economics

Prof Mark Sculpher	York, UK

Molecular Genetics

Prof John Masters, Pathologist	London, UK

For full details of all trial committees, please see **Appendix M**.

CONTENTS

GENERAL INFORMATION I

CONTENTS 4

ABBREVIATIONS 8

1 SUMMARY 11
1.1 LAY SUMMARY 11
1.2 ABSTRACT AND SUMMARY OF TRIAL DESIGN 12
1.3 TRIAL DOCUMENTATION 16

2 BACKGROUND 18
2.1 INTRODUCTION AND SETTING 18
2.1.1 Long-term Androgen Deprivation Therapy 18
2.1.2 Role of Radiotherapy for Patients With M0 Disease 18
2.2 RATIONALE 18
2.3 DESIGN 19
2.4 RESEARCH TREATMENT AND BIOSPHOSPHONATES 19
2.5 RESEARCH TREATMENT: CHEMOTHERAPY 20
2.6 RESEARCH TREATMENT: CYCLOOXYGENASE-2 INHIBITORS 20
2.7 RESEARCH TREATMENT: STEROID SYNTHESIS INHIBITORS 21
2.8 RESEARCH TREATMENT: RADIOTHERAPY TO THE PROSTATE FOR PATIENTS WITH NEWLY-DIAGNOSED METASTATIC DISEASE 21
2.9 RESEARCH TREATMENT: COMBINATIONS 22
2.9.1 Bisphosphonate And Chemotherapy 22
2.9.2 Bisphosphonate And Cyclooxygenase-2 Inhibitors 22

3 SELECTION OF INSTITUTIONS AND INVESTIGATORS 24
3.1 RADIOTHERAPY ACCREDITATION 24

4 SELECTION OF PATIENTS 25
4.1 PATIENT INCLUSION CRITERIA 25
4.1.1 High-Risk Newly Diagnosed Non-Metastatic Node-Negative Disease 25
4.1.2 Newly Diagnosed Metastatic Or Node-Positive Disease 25
4.1.3 Previously Treated With Radical Surgery And/Or Radiotherapy, Now Relapsing 25
4.1.4 For All Patients 25
4.2 PATIENT EXCLUSION CRITERIA 26
4.3 SELECTION CRITERIA FOR COMPARISON OF RESEARCH (M1) RT FOR METASTATIC DISEASE 27
4.4 SCREENING PROCEDURES 27
4.4.1 Investigations Prior To Randomisation 27
4.4.2 Androgen Deprivation Therapy Prior To Randomisation 28
4.4.3 Hypercalcaemia At Randomisation 28
4.4.4 NSAIDs And Cox-2 Inhibitors At Randomisation 28
4.4.5 Starting Trial Treatment 28
4.4.6 Concomitant Medications 29
4.5 ADDITIONAL DETAILS FOR PATIENTS JOINING SUB-STUDIES 29

5 RANDOMISATION AND ENROLMENT 30
5.1 CO-ENROLMENT GUIDELINES 30

6 TREATMENT OF PATIENTS 31
6.1 TRIAL TREATMENT 31
6.1.1 Arm A: ADT Alone Or ADT + Standard-of-Care (M0) RT (Control Arm) 31
6.1.2 Arm B: ADT + Zoledronic Acid 32
6.1.3 Arm C: ADT + Docetaxel 32
6.1.4 Arm D: ADT + Celecoxib 32
6.1.5 Arm E: ADT + Docetaxel + Zoledronic Acid 32
6.1.6 Arm F: ADT + Zoledronic Acid + Celecoxib 33
6.1.7 Arm G: ADT + Abiraterone 33
6.1.8 ARM H: ADT + Prostate Radiotherapy in M1 patients 34
6.2 ADMINISTRATION AND DOSE MODIFICATIONS 34
6.2.1 Zoledronic Acid 34
6.2.2 Docetaxel 35
6.2.3 Celecoxib 35
6.2.4 Abiraterone 35
6.2.5 Research (M1) Prostate Radiotherapy 37
6.3 TRIAL PRODUCTS 39
6.4 MEASURES OF COMPLIANCE/ADHERENCE 39
6.5 TREATMENT DATA COLLECTION 39
6.6 ADMINISTRATION OF STANDARD RADIOTHERAPY TO NON-METASTATIC PATIENTS 40
6.6.1 Treatment Details 40
6.7 NON-TRIAL TREATMENT 41
6.7.1 Medications Permitted 41
6.7.2 Data On Concomitant Medication 41

7 ASSESSMENTS AND PROCEDURES 42
7.1 FLOW CHART/SCHEDULE FOR FOLLOW-UP 42
7.1.1 Psa Measurements 42
7.1.2 Assessment Of Treatment Failure (Definition Of Progression) 42
7.1.3 Additional Safety Assessment 42
7.1.4 Data Collection and Non-Administration Of Standard Radiotherapy 43
7.1.5 Data Collection Palliative Radiotherapy 43
7.1.6 Data Collection Research (M1) Radiotherapy 43
7.1.7 Follow-Up Schedules 43
7.2 FOLLOW-UP 43
7.3 TRIAL CLOSURE 45

8 STOPPING OF TREATMENT OR FOLLOW UP 47
8.1 STOPPING RESEARCH INTERVENTIONS 47
8.2 PATIENT TRANSFERS 47
8.3 WITHDRAWAL FROM THE TRIAL COMPLETELY 48

9 STATISTICAL CONSIDERATIONS 49
9.1 METHOD OF RANDOMISATION 49
9.2 OUTCOME MEASURES 50
9.3 SAMPLE SIZE: PRINCIPLES AND ASSUMPTIONS 51

9.4 SAMPLE SIZE ISSUES AND TRIAL STAGES: ORIGINAL RESEARCH ARMS (B-F) 51
9.4.1 Pilot Phase: Original Research Arms (B-F) 51
9.4.2 Activity Stages I-Iii: Original Research Arms (B-F) 52
9.4.3 Efficacy Stage Iv: Original Research Arms (B-F) 52
9.4.4 Sample Size For Original Research Arms (B-F) 52
9.5 SAMPLE SIZE ISSUES AND TRIAL STAGES: ADDITIONAL RESEARCH ARM G 53
9.5.1 Pilot Phase: Additional Research Arm G 53
9.5.2 Activity Stages I-Iii: Additional Research Arm G 53
9.5.3 Efficacy Stage Iv: Additional Research Arm G 53
9.5.4 Sample Size For Additional Research Arm G 53
9.6 SAMPLE SIZE ISSUES AND TRIAL STAGES: ADDITIONAL RESEARCH ARM H 54
9.6.1 Pilot Phase: Additional Research Arm H 54
9.6.2 Activity Stages I-III: Additional Research Arm H 54
9.6.3 Efficacy Stage Iv: Additional Research Arm H 54
9.6.4 Sample Size For Additional Research Arm H 54
9.7 FURTHER NOTES ON TRIAL DESIGN 57
9.7.1 Overall Sample Size 57
9.7.2 Factorial Design 57
9.8 INTERIM MONITORING AND ANALYSES 57
9.9 OUTLINE ANALYSIS PLAN 57
9.9.1 Pilot / Safety Phases 58
9.9.2 Activity And Efficacy Stages 58

10 MONITORING AND QUALITY ASSURANCE 59
10.1 MONITORING AT MRC CTU 59
10.2 DIRECT ACCESS TO DATA 59
10.3 VISITS TO INVESTIGATOR SITES 59
10.4 CONFIDENTIALITY 59

11 SAFETY REPORTING 60
11.1 DEFINITIONS 60
11.1.1 Trial-Specific Exemptions 61
11.2 INSTITUTION/INVESTIGATOR RESPONSIBILITIES 61
11.2.1 Investigator Assessment 61
11.2.2 Notification Procedure 62
11.3 MRC CTU RESPONSIBILITIES 63

12 ETHICAL CONSIDERATIONS AND APPROVAL 64
12.1 ETHICAL CONSIDERATIONS 64
12.2 ETHICAL APPROVAL 64

13 REGULATORY APPROVAL 66

14 INDEMNITY 67

15 FINANCE 68

16 TRIAL COMMITTEES 69
16.1 TRIAL MANAGEMENT GROUP (TMG) 69

16.2 TRIAL STEERING COMMITTEE (TSC) 69
16.3 INDEPENDENT DATA MONITORING COMMITTEE (IDMC) 69

17 ANCILLARY STUDIES 71
17.1 QUALITY OF LIFE 71
17.2 HEALTH ECONOMICS 71
17.3 TRANSLATIONAL SUB-STUDIES 71
17.3.1 DNA Analysis 71
17.3.2 Tissue Microarray 72

18 PUBLICATION 73

19 PROTOCOL AMENDMENTS 74
19.1 PROTOCOL 74
19.1.1 Amendments Made To Sections In Protocol Version 1.0 (May 2004) 74
19.1.2 Amendments Made To Sections In Protocol Version 1.1 (May 2005) 74
19.1.3 Amendments Made To Sections In Protocol Version 2.0 (Jun 2005) 74
19.1.4 Amendments Made To Section In Protocol Version 3.0 (Jul 2006) 75
19.1.5 Amendments Made To Protocol Version 4.0 (Dec 2007) 76
19.1.6 Amendments Made To Protocol Version 5.0 (Aug 2008) 76
19.1.7 Amendments Made To Protocol Version 6.0 (Jul 2009) 77
19.1.8 Amendments Made To Protocol Version 7.0 (Jul 2011) 78
19.1.9 Amendments Made To Protocol Version 7.1 (Jul 2011) 78
19.1.10 Amendments Made To Protocol Version 8.0 (Sep 2011) 79

20 REFERENCES 82

ABBREVIATIONS

Abbreviation	Expansion
ACE	Angiotensin-Converting Enzyme
ACTH	Adrenocorticotropic hormone
ADT	Androgen deprivation therapy
AS	Activity Stage
bid	Twice a day (bis in die)
BP	Blood pressure
BSA	Body surface area
CERES	Consumers for Ethics in Research
CF	Consent Form
CI	Chief Investigator
CI	Confidence interval
COSTART	Coding Symbols for a Thesaurus of Adverse Reaction Terms
Cox 2	Cyclooxygenase 2
CRF	Case Report Form
CRUK	Cancer Research UK
CRPC	Castrate Refractory Prostate Cancer
CT	Computerised tomography
CTA	Clinical Trials Authorisation
CTAAC	Clinical Trials Advisory and Awards Committee
CTC	Common Toxicity Criteria
CTU	Clinical Trials Unit
CTV	Clinical Tumour Volume
CXR	Chest X-ray
DDX	Doctors and Dentists Exemption
DNA	Deoxyribonucleic Acid
DPA	Data Protection Act
ERC	Endpoint Review Committee
ES	Efficacy Stage
ICH	International Conference on Harmonization
ECG	Electro cardiogram
FBC	Full Blood Count

Abbreviation	Expansion
FFS	Failure-Free Survival
GCP	Good Clinical Practice
GP	General Practitioner
GRO	General Register Office
HE	Health Economics
HES	Hospital Episode Statistics
hr	Hour
HR	Hazard Ratio
HRPC	Hormone Refractory Prostate Cancer
HT	Hormone Therapy
IDMC	Independent Data Monitoring Committee
IM	Intramuscular
IMRT	Intensity Modulated Radiation Therapy
ISRCTN	International Standard Randomised Controlled Trial Number
IU	International Units
IV	Intravenous
LD	Longest diameter
LFTs	Liver Function Tests
LHRH	Luteinising Hormone Releasing Hormone
LREC	Local Research Ethics Committee
MHRA	Medicine and Healthcare Products Regulatory Agency
m	Month
min	Minutes
MRC	Medical Research Council
MREC	Multi-Centre Research Ethics Committee
MRI	Magnetic resonance imaging
NCI	National Cancer Institute (USA)
NCRN	National Cancer Research Network
NHS	National Health Service
NSAID	Non-Steroidal Anti-inflammatory Drugs
ONS	Office for National Statistics
OS	Overall Survival
PI	Principal Investigator

Abbreviation	Expansion
PIS	Patient Information Sheet
po	per orum (orally)
PSA	Prostate Specific Antigen
pts	Patients
PTV	Planned Tumour Volume
QALY	Quality-adjusted Life Years
qds	quater die sumendus (4 times each day)
QL	Quality of Life
R&D	Research and Development
RECIST	Response Evaluation Criteria In Solid Tumours
SAE	Serious Adverse Event
SAR	Serious Adverse Reaction
sc	Sub-cutaneous (under skin)
SNP	Single Nucleotide Polymorphism
SSA	Site Specific Assessment
STAMPEDE	Systemic Therapy in Advancing and Metastatic Prostate Cancer: Evaluation of Drug Efficacy
SUSAR	Suspected Unexpected Serious Adverse Reactions
SWOG	South West Oncology Group
TMG	Trial Management Group
TMT	Trial Management Team
TURP	Trans-Urethral Resection of Prostate
TSC	Trial Steering Committee
UCL	University College London
ULN	Upper Limit of Normal
U+E	Urea and Electrolytes
WHO	World Health Organisation

1 SUMMARY

1.1 LAY SUMMARY

Prostate cancers depend upon the male hormone testosterone for their growth. Lowering testosterone levels (either by removing all or part of both testes, or by giving anti-hormone treatment) slows the growth of prostate cancers. This type of treatment is called hormone treatment and is often used when prostate cancers have spread outside the prostate gland. Although hormone treatment is usually successful at stopping the cancer growing for a period of time, the cancer will begin to grow again in most men.

There are increasing numbers of treatments available for advanced prostate cancer. These treatments are usually used in prostate cancer when hormone treatment is no longer effective and the cancer has started to grow again. The aim of this trial, which is called STAMPEDE, is to assess some of these treatments, given earlier in the course of the disease in combination with hormone treatment.

The treatments that have, or are being, assessed during the trial are:

1. Zoledronic acid: Prostate cancer cells can spread to bones and weaken them. Zoledronic acid is a drug that reduces bone destruction and hardens bones. This may make them more resistant to attack by cancer cells.

2. Docetaxel: A drug that stops cells replicating that is currently being used to treat a range of cancers including lung, breast and ovarian cancer as well as prostate cancer. Docetaxel prolongs survival in men with relapsed metastatic prostate cancer.

3. Celecoxib: An aspirin-like drug that is used to treat arthritis. It slows down the growth of cancer cells in the laboratory. We wished to see if it had the same effect on cancer cells in patients. Recruitment to new patients for the evaluation of this drug is finished as a planned interim analysis failed to demonstrate sufficient activity.

4. Abiraterone (included from protocol version 8.0)**:** An inhibitor of steroid hormone synthesis that blocks prostate cancer cells from generating their own male hormones. This is thought to be a major way in which prostate cancer cells resume growth following castration based therapies. The agent prolongs survival when given to men following failure of docetaxel chemotherapy.

5. Prostate radiotherapy (included from protocol version 9.0)**:** treatment with high-energy x-rays targeted to the prostate gland. This treatment is now mandatory for patients with cancer that is confined to the prostate gland as large trials have shown it improves survival times. We are not certain whether we should give radiotherapy to the prostate if the cancer has already spread.

STAMPEDE will look at the effect of combining one or two of the treatments described above with hormone treatment. A computer program will be used to allocate which treatment the patient receives, using a chance process. The trial will look at the effects of the combined treatments on quality of life and find out whether the new treatment combinations increase the time when the cancer is not growing and ultimately results in patients living longer. The study will also look at which

treatment provides the greater value for money for the health service. More than 5,000 patients will join the trial with answers becoming available over 7 to 12 years.

1.2 ABSTRACT AND SUMMARY OF TRIAL DESIGN

STAMPEDE is a multi-centre, randomised controlled trial for patients with locally advanced or metastatic prostate cancer who are about to commence Androgen Deprivation Therapy (ADT). Patients can have either newly diagnosed disease, or have been previously treated with radical radiotherapy or surgery but now have a rising prostate specific antigen (PSA) (further details on eligibility see **Section 4**). The trial will assess the effects of adding different agents, both as single agents and in combinations, to androgen deprivation therapy. The investigational agents are (i) a bisphosphonate, zoledronic acid, (ii) a cytotoxic chemotherapeutic agent, docetaxel and (iii) a cyclooxygenase (Cox-2) inhibitor, celecoxib. (iv) a novel androgen deprivation therapy drug called abiraterone, a steroid synthesis inhibitor. Recruitment to the celecoxib arms (D and F) is now closed. An additional arm containing abiraterone was added in protocol version 8.0. A further comparison arm involving prostate radiotherapy for patients with metastatic disease was added in protocol version 9.0. The trial has multiple arms; the control arm of the trial is androgen deprivation therapy (ADT) only, achieved through the use of luteinising hormone releasing hormone (LHRH) analogues or LHRH antagonists, or bilateral orchidectomy according to local practice. The other trial arms are summarised in **Figure 1 to 5**.

Figure 1: Recruiting arms of the STAMPEDE trial to Apr-2011

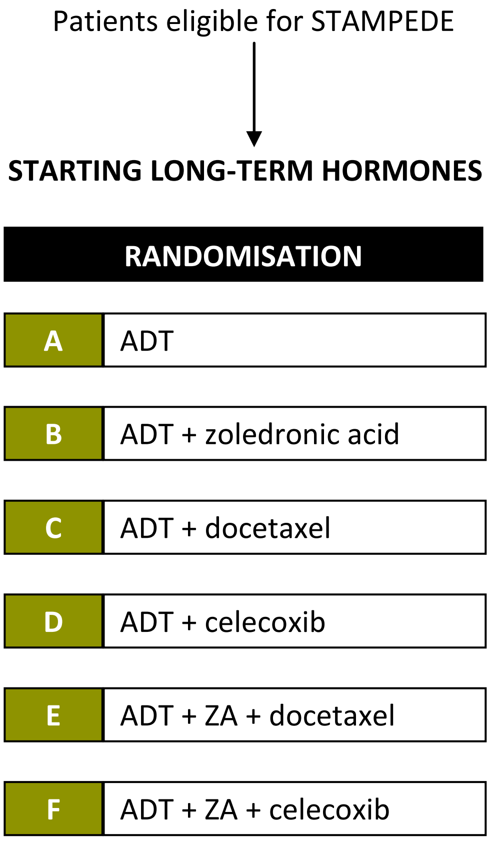

Figure 2: Recruiting arms of the STAMPEDE trial from Apr-2011 to Nov-2011 (v7.0)

Patients eligible for STAMPEDE

↓

STARTING LONG-TERM HORMONES

RANDOMISATION	
**A**	ADT
**B**	ADT + zoledronic acid
**C**	ADT + docetaxel
**E**	ADT + ZA + docetaxel

Accrual stopped to celecoxib-containing arms, D and F, after their Activity Stage II analysis

Figure 3: Recruiting arms of the STAMPEDE trial from Nov-2011 to Jan-2013 (v9.0)

Patients eligible for STAMPEDE

↓

STARTING LONG-TERM HORMONES[1]

RANDOMISATION	
**A**	ADT
**B**	ADT + zoledronic acid
**C**	ADT + docetaxel
**E**	ADT + ZA + docetaxel
**G**	ADT + abiraterone

[1] All suitable pts with newly diagnosed locally advanced disease should also have RT to the prostate

Accrual was initiated to the abiraterone arm, Arm G, in Nov-2011.

Figure 4: Recruiting arms of the STAMPEDE trial from protocol version 9.0 (Jan-2013 to Mar-2013)

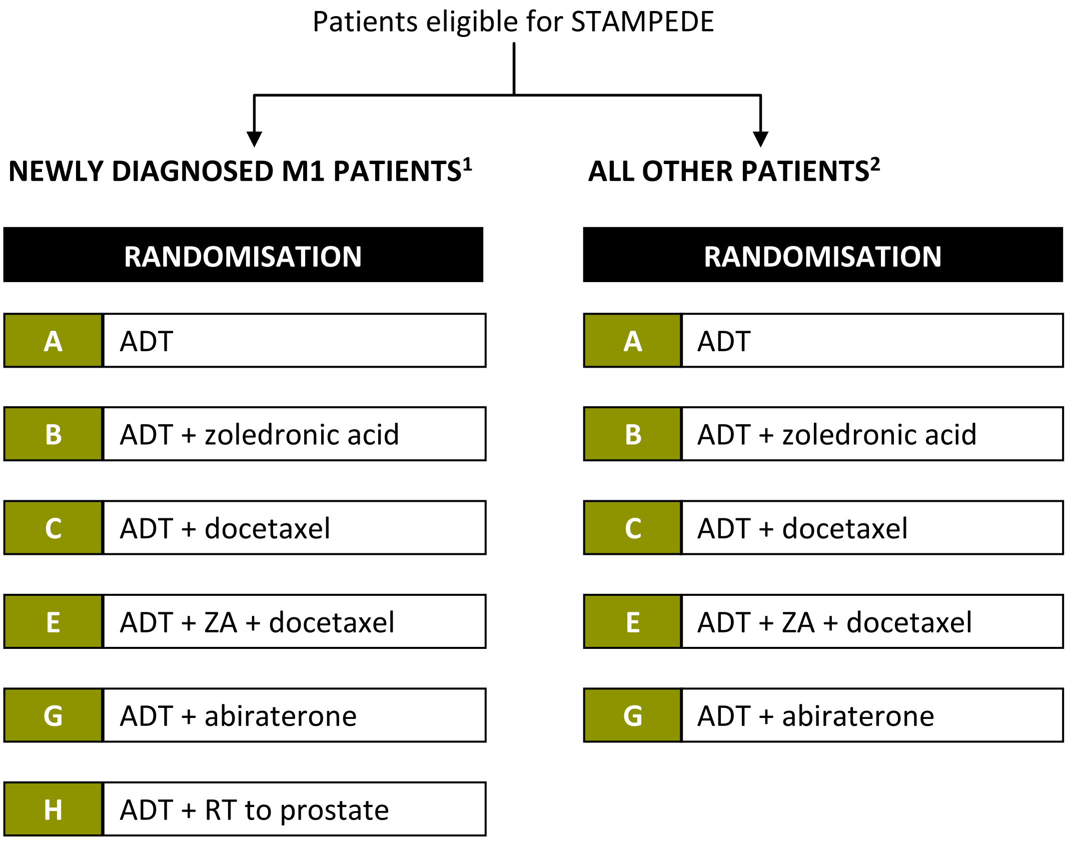

[1] Except pts with a contra-indication to RT
[2] All suitable pts with newly diagnosed locally advanced disease should also have RT to the prostate

Accrual was initiated to the radiotherapy-to-the-prostate for metastatic disease arm, Arm H, in Jan-2013.

Figure 5: Arms of the STAMPEDE Trial from protocol version 10.0 (after original research arms completed accrual)

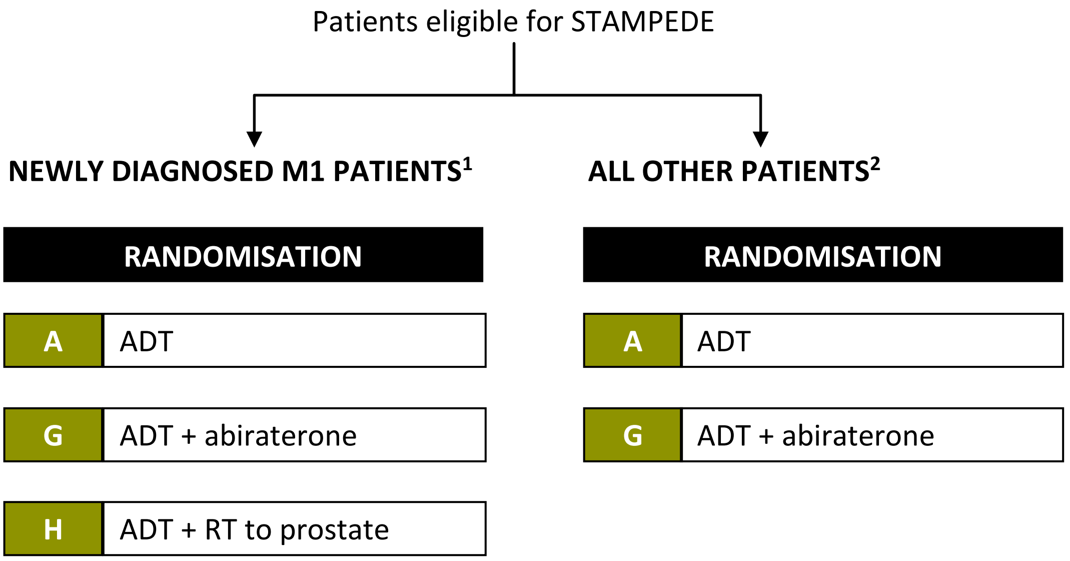

[1] Except pts with a contra-indication to RT
[2] All suitable pts with newly diagnosed locally advanced disease should also have RT to the prostate1

Accrual was completed to original research arms B, C and E in Mar-2013.

Figure 6: Arms of the STAMPEDE trial open to recruitment over time

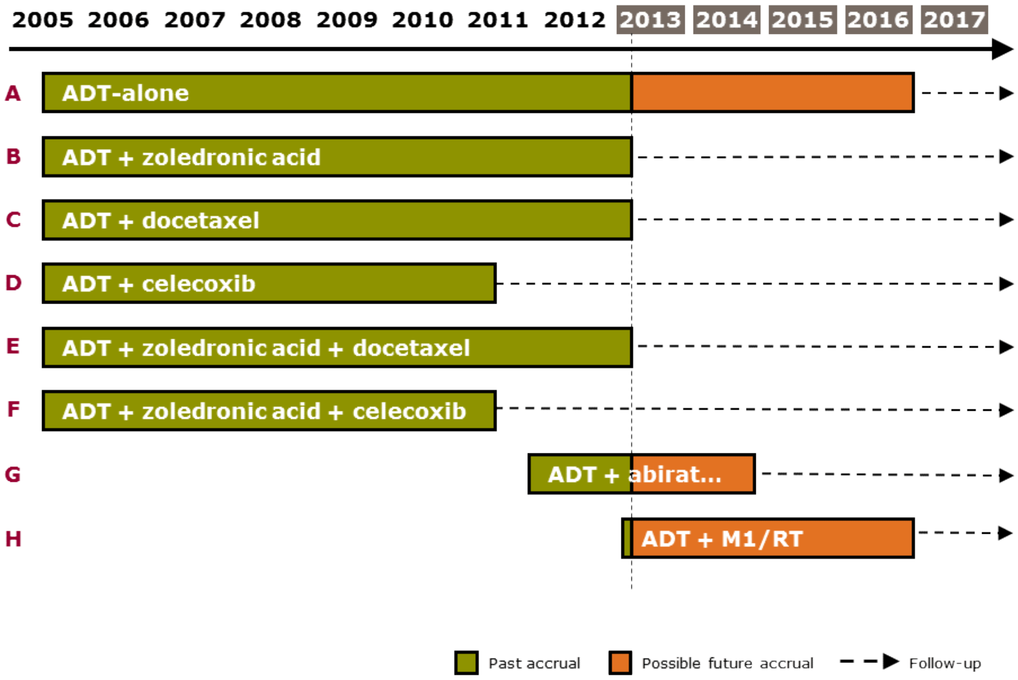

For each comparison of research arm against control, the trial will be conducted in five stages: a Pilot Phase, Activity Stages I to III and Efficacy Stage IV. The primary outcome measure of the Pilot Phase is the safety, with 30 patients recruited to each research arm. Research arms will only continue to recruitment in the next stage if they have been shown to be both safe and feasible, although patient

data from all patients and all stages will be included in the final analyses. In Activity Stages I-III the primary outcome measure is failure-free survival (FFS). Further patients will be recruited until a certain number of FFS events have been observed in the control arm (see **Section 9** for further detail). Some evidence of activity will be required for a research arm to proceed to further recruitment in each stage and guidelines are in place. In Efficacy Stage IV, patients will be recruited when a certain number of primary outcome measure events have been reported. This is when around 403 deaths have been reported in the control arm for the original research comparisons and 267 (control arm deaths) for both the abiraterone comparison and the local-RT-for-M1-disease comparison. The exact number of patients and duration of the trial will depend on the observed accrual rate, observed event rate and the number of patients accruing at each stage.

Recruitment to arms D (ADT + celecoxib) and F (ADT + zoledronic acid + celecoxib) was stopped in Apr-2011 after the second planned activity analysis when the IDMC and TSC considered the lack-of-benefit guidelines.(1) Refer to **Section 9.3**. for further information regarding the guidelines for stopping accrual to research arms during the activity stages of the trial.

In version 8.0 of the protocol a new arm G (ADT + abiraterone) was added. Arm H (ADT+ prostate radiotherapy) was added in protocol version 9.0. The trial stages remain as at trial inception but will be staggered in time compared to the stages for the original arms A-F.

Patients will be assessed 6 weekly for the first 24 weeks after randomisation and then every 12 weeks up to 2 years, then 6-monthly until 5 years and annually, thereafter. The first 700 patients on trial completed questionnaires aimed at assessing the effects of the investigational treatments on their quality of life (QL) and on their use of health care resources (Health Economics (HE) study). From protocol version 8.0, the QL and HE study has been re-opened to all new patients.

In addition, there are translational sub-studies. Patients willing to participate will be asked at randomisation to donate a droplet of blood, which will be stored for DNA and protein analysis in order to try to identify markers that are associated with response to therapy, side-effects or susceptibility to prostate cancer.

Patients will also be asked to give permission to use some of their stored material (blood or biopsy samples) for further studies on the causes and nature of prostate cancer. In selected centres patients were asked to participate in a bone mineral density sub-study. This sub-study has now stopped recruitment. There are separate patient information sheets for the QL and HE study and the translational sub-studies (For further details of ancillary studies, see **Section 17**).

1.3 TRIAL DOCUMENTATION

Table 1 presents a summary of the required trial documentation for participating centres and **Table 2** presents a summary of the timings of the case report forms (CRFs) for your randomised patients.

Table 1: Summary of trial documentation required ahead of initial accreditation

TRIAL DOCUMENTATION	TIMING
R&D approval (including IRMER approval)	Before centre participation
Investigator Statement	Before centre participation

Signature list & delegation of responsibilities	Before centre participation
Trial personnel contact details	Before centre participation
PIS, GP & CF on local paper	Before centre participation
Signed Clinical Trial Agreement between Trust and Sponsor	Before centre participation

Table 2: Summary of trial documentation required ahead of re-accreditation (protocol v9.0 only)

TRIAL DOCUMENTATION	TIMING
R&D approval (including IRMER approval)	Before centre re-activation
PIS, GP & CF on local paper	Before centre re-activation
RTQA accreditation	Before centre re-activation

2 BACKGROUND

2.1 INTRODUCTION AND SETTING

Prostate cancer is a major health problem world-wide and accounts for nearly one fifth of all newly diagnosed male cancers. In the UK, approximately 35,000 men are diagnosed with prostate cancer each year and in 2008 almost 10,000 men died from the disease.(2)

2.1.1 LONG-TERM ANDROGEN DEPRIVATION THERAPY

The initial (first line) treatment for locally advanced or metastatic prostate cancer is androgen deprivation therapy (ADT) achieved either surgically with bilateral orchidectomy, or medically with LHRH agonists or antagonist or oral anti-androgens alone. (3) Oral anti-androgens were permitted in the trial but were used by very few patients and are no longer permitted for new patients within the trial from version 8.0.

ADT produces responses in up to 95% of patients but it is not curative and disease recurs in virtually all patients treated with ADT as sole therapy, with a median time to progression of 18-24 months. (3) Such disease is referred to as hormone or increasingly as castrate refractory prostate cancer (HRPC or CRPC); this latter term is unpopular with patient groups due to its perceived pejorative overtones and hence terminology may yet change again in the future.

2.1.2 ROLE OF RADIOTHERAPY FOR PATIENTS WITH M0 DISEASE

Two randomised trials, SPCG7 (4) and NCIC PR.3 / MRC PR07 (5-7) have tested the question of whether androgen deprivation therapy alone or combined with radiotherapy is the best treatment for high-risk patients with no evidence of spread outside the pelvis. Both trials demonstrate an improvement in overall and disease specific survival from the addition of radiotherapy to androgen deprivation therapy. The size of this overall survival benefit is substantial (hazard ratio 0.68 in SPCG7 and HR 0.77 in PR07). With substantial benefit demonstrated in two mature, large, well conducted randomised trials, we now recommend that radiotherapy be considered standard for patients with no nodal or metastatic spread. Patients in this category will now only be allowed to enter the trial if standard radiotherapy is planned, with the exception of those for whom radiotherapy is contra-indicated who should be discussed with the Trials Unit prior to inclusion. For patients with node positive, M0 disease there are no clear data on whether radiotherapy is or is not indicated. The NCIC PR.3 / MRC PR07 trial included patients with unknown nodal status who received whole pelvic radiotherapy. Given the large overall benefit observed in this trial, the STAMPEDE TMG recommends that pelvic nodal radiotherapy be considered for patients with node positive, non-metastatic disease at the discretion of the treating clinician.

2.2 RATIONALE

There are increasing numbers of treatments which are used post relapse of first-line androgen deprivation therapy in patients with CRPC, but little evidence as to which is associated with the best response or how they may be combined or sequenced or whether any of them might have a role in first-line treatment. Such treatments include further hormonal manipulations, bisphosphonates, (8), cytotoxic chemotherapy (9), new hormone therapies (10) and palliative radiotherapy. The traditional approach to the testing and introduction of new treatments for prostate cancer is to use them in patients with castrate refractory disease. An alternative approach is to investigate new drugs and

new approaches to treatment, as first-line therapy in patients starting androgen deprivation therapy. At this point, patients should be fitter and better able to tolerate treatment than when they have CRPC and there is the possibility of having a larger and longer lasting effect.

2.3 DESIGN

STAMPEDE (also known as MRC PR08) is an innovative, multi-arm multi-stage, multi-centre, randomised controlled trial. It initially assessed the effects of a bisphosphonate (zoledronic acid), a cytotoxic chemotherapeutic agent (docetaxel) and a cyclooxygenase (Cox-2) inhibitor (celecoxib), as single agents or combinations, in patients commencing androgen deprivation therapy for advancing or metastatic prostate cancer. Each comparison is divided into five stages such that, for each investigational arm, safety and activity data are generated in the first four stages; an investigational arm will only proceed to the fifth and final stage of recruitment, where it will be assessed for its effect on overall survival, if it has been shown to be sufficiently safe and active. It is important to note, however, that patient data from all arms and all stages will be included in the final analyses of the primary outcome measure, even if the investigational arm did not proceed to the final stage.

Planned interim analysis failed to demonstrate sufficient activity for celecoxib (James, 2012 –ref to be added to list) and this agent has now been removed from the trial recruitment; patients remaining on celecoxib treatment reverted to standard care. Protocol version 8.0 added a new drug abiraterone to the study as an additional arm (see **Section 2.7**). Protocol version 9.0 added a new comparison arm involving prostate radiotherapy for patients with metastatic disease (see **Section 2.8**). Protocol version 10.0 reflected the successful completion of recruitment to three docetaxel- and bisphosphonate-containing arms (Arms B, C and E) and removes references to these agents in the information sheets for new patients. The current protocol (version 11) extends the recruitment target for the abiraterone research comparison (A vs G) from 1,500 to 1,800 patients.

2.4 RESEARCH TREATMENT AND BIOSPHOSPHONATES

Note: recruitment stopped to the zoledronic acid and docetaxel-containing arms in Mar-2013 at the end of Activity Stage IV.

The bisphosphonates are a class of drug that act by reducing osteoclast formation, inhibiting osteoclast activity and inducing osteoclast apoptosis. They are effective at controlling hypercalcaemia and preventing skeletal complications associated with malignant disease. (11, 12) Zoledronic acid is a highly potent, third generation bisphosphonate; studies comparing the efficacy of zoledronic acid to other bisphosphonates suggest that zoledronic acid has a 40-850 fold higher potency than clodronate in preclinical models of bone resorption. (13). It has also been shown to be more effective than pamidronate (90mg) in controlling malignant hypercalcaemia. (14) In addition, zoledronic acid has also demonstrated direct anti-cancer activity, including inhibition of proliferation of breast cancer and prostate cancer cells in vitro. (15)

In randomised controlled trials of 1,648 patients, 4mg zoledronic acid was more effective than pamidronate in reducing the risk of skeletal complications in patients with bone metastases from breast cancer. (16, 17) Also, in metastatic prostate cancer, zoledronic acid has been shown to reduce the rate of skeletal-related events compared to placebo in a trial involving 429 men. (18) In April 2002, zoledronic acid received approval from the Committee for Proprietary Medicinal Products for the prevention of skeletal-related events (for example, fractures) in patients with any advanced malignancies involving bone.

The MRC PR05 prostate cancer trial showed that a first generation bisphosphonate (clodronate) commenced at the time of androgen deprivation therapy initiation, delayed time to progression in patients with bony

metastatic disease and there was some evidence that it may also improve survival. (19) There is, therefore, a good rationale for investigating a more potent bisphosphonate in patients with prostate cancer who are about to commence ADT therapy.

2.5 RESEARCH TREATMENT: CHEMOTHERAPY

Note: recruitment stopped to the zoledronic acid and docetaxel-containing arms in Mar-2013 at the end of Activity Stage IV.

There is increasing evidence of the clinical efficacy of chemotherapy in prostate cancer. (9) Two randomised phase III trials in patients with metastatic hormone refractory prostate cancer (HRPC) using a docetaxel-containing regimen have been completed: the SWOG 9916 study (20) and the TAX-327 study. (21) Both studies show that the use of a docetaxel-based regimen improved survival for patients with metastatic HRPC and had significantly greater PSA response rates compared to the mitoxantrone plus prednisolone arm.

In the TAX-327 trial, (21) 1,006 patients with metastatic HRPC were randomized to receive either mitoxantrone 12 mg/m² with prednisone 10mg daily (Arm C) or docetaxel 75mg/m² 3-weekly for 10 cycles with prednisone (Arm A) or docetaxel 30 mg/m²/wk x 5 of 6 weeks x 5 cycles with prednisone (Arm B). Median overall survival was 16.5 months for patients treated with mitoxantrone versus 18.9 months for the 3-weekly docetaxel regimen (hazard ratio 0.76 (0.62-0.94)). There was also improvements for 3-weekly docetaxel in pain (22% vs 35%, p = 0.01) and PSA response (32% vs 45%, p=0.0005).

In June 2006 in the UK docetaxel was given NICE (National Institute for Health and Clinical Excellence) approval for use in hormone (now more commonly termed castrate) refractory prostate cancer patients.

2.6 RESEARCH TREATMENT: CYCLOOXYGENASE-2 INHIBITORS

Note: recruitment completed to both celecoxib-containing arms in Apr-2011 at the end of Activity Stage II of this comparison

Cyclooxygenase-2 (Cox-2) is an isoenzyme induced by a variety of mitogens, cytokines and growth factors that are associated with a range of process including inflammation, (22) and carcinogenesis.(23, 24) There is a growing body of evidence that inhibition of Cox-2 may play an important role in the prevention of cancer and the delay of progression in established cancer. A number of case-control studies have shown a reduction in risk of prostate cancer associated with the use of non-steroidal anti-inflammatory drugs (NSAID), which include inhibition of Cox-2 amongst their mode of action. (25) Pathological studies show Cox-2 is upregulated in carcinomas (26) and one study suggested that NSAID use may delay progression from subclinical to clinical prostate cancer. (27)

Celecoxib, a Cox-2 inhibitor, is better tolerated than other NSAIDs and there is evidence that it is active as a chemoprevention agent. (28) It also has important antineoplastic properties such as the ability to inhibit angiogenic factors and induce apoptosis in human cancer cells including prostate cancer. (29)

Evidence has suggested that an anti-cancer effect is only seen at higher doses of celecoxib than required for an anti-inflammatory effect. (30) Therefore, the dose of 800mg/day for STAMPEDE patients has been chosen. Although there is some high profile evidence of a small absolute increase in CVS toxicity risk associated with higher doses of celecoxib, (31) most current cancer trials are using a dose of 800mg/day as it is believed that a higher dose will result in a greater increase in cancer effect.

There is also some evidence of a schedule effect on CVS toxicity. It has been observed that CVS toxicity becomes evident after one year of taking celecoxib. (31) Therefore, a maximum duration of one year has been set for celecoxib use in this trial. Any potential risks of course have to be weighed against any potential benefits of celecoxib in the delay of progression in established prostate cancer.

Given case-control data suggesting effects on prostate cancer, pathological expression of Cox-2 in prostate cancer and in vitro data suggesting that inhibition of Cox-2 inhibits growth and invasiveness, further investigation in prostate cancer is warranted.

2.7 RESEARCH TREATMENT: CYP17A1 INHIBITORS

Recent evidence suggests that an important mechanism for escape from tumour control by androgen ablation is the intracellular conversion of steroid precursors to androgenic steroids by prostate cancer cells. A key enzyme in this process is CYP17, which therefore represents a logical target for therapy in CRPC. (10) Abiraterone acetate is a selective inhibitor of CYP17 and is highly active in patients developing resistance to standard androgen ablation therapies. (32-34) Recruitment to a phase III study comparing abiraterone acetate to placebo in CRPC patients post-docetaxel, completed accrual in 2009 and reported initial results in 2011 with an improvement in overall survival of around 4 months and a hazard ratio of 0.65. (35) The drug has now received a marketing authorisation in the USA and in the EU from September 2011. A second trial in pre-chemotherapy CRPC patients completed recruitment April 2010; preliminary results are positive and are were published in 2012 (36) and the licence for abiraterone was extended to the pre-chemotherapy CRPC population in Europe in 2012. Side-effects with abiraterone acetate are modest with the main adverse effects being elevated transaminases (usually mild), hypokalaemia and hypertension due to secondary hyperaldosteronism and fluid retention (preventable by low doses of glucocorticoids). In order to prevent secondary hyperaldosteronism, it is recommended that prednisolone (or prednisone) 10mg daily be administered in the CRPC setting. Within more recent studies in earlier stage patients, lower doses (typically 5mg of prednisone/prednisolone) are being used due to concerns about long-term exposure to glucocorticoid side effects. More recent evidence even suggests that for most patients, no glucocorticoids may be needed. (37) Within the STAMPEDE trial, we propose to use a prednisone/prednisolone dose of 5mg daily.

We hypothesise that the agent may be more active still when given up-front in combination with first-line androgen deprivation therapy by preventing or delaying the development of castrate refractory disease.

2.8 RESEARCH TREATMENT: RADIOTHERAPY TO THE PROSTATE FOR PATIENTS WITH NEWLY-DIAGNOSED METASTATIC DISEASE

Therapy directed against the primary tumour in the presence of metastatic disease has been evaluated rigorously in only one malignancy to date: renal cell carcinoma. Two cooperative groups ran randomised trials enrolling patients with previously untreated metastatic RCC whose primary tumours were amenable to surgical resection. Patients were randomized to receive the standard systemic therapy of the day, interferon-alpha, either alone or with radical nephrectomy. The combination of nephrectomy and interferon was shown to significantly improve median survival from 7 to 17 months in one trial (38) and from 8 to 11 months in the other.(39) The mechanism by which nephrectomy improves survival remains obscure. In preclinical models, the primary tumour has been found to secrete molecules that prime the microenvironment in which metastases can develop. An implication of this work is that therapy directed at the primary tumour, by abrogating this endocrine signalling, could retard the formation and the growth of distant metastases.

The results of two large-scale randomised trials of prostate radiotherapy are also provocative. The Scandinavian SPCG-7 trial and the MRC PR07 trial randomised men with locally advanced prostate cancer, who were at high risk of possessing occult metastatic disease, to either androgen deprivation therapy (ADT) alone or ADT plus prostate radiotherapy.(4, 40) The addition of radiotherapy dramatically improved 10-year outcomes: mortality from prostate cancer was halved. Interestingly, the benefit of radiotherapy started to emerge as early as three years from the time of randomisation. This seems improbably early if the benefit of local treatment is mediated via the prevention of subsequent disease dissemination. Rather, it is more consistent with the possibility that local treatment has a beneficial impact on the rate of progression of existing micrometastatic disease.

We hypothesise that local therapy to the primary site may retard distant disease progression and prolong survival in patients with metastatic prostate cancer.

2.9 RESEARCH TREATMENT: COMBINATIONS

2.9.1 Bisphosphonate And Chemotherapy

Note: recruitment stopped to the zoledronic acid and docetaxel-containing arms in Mar-2013 at the end of Activity Stage IV.

Zoledronic acid and docetaxel have different mechanisms of action. In addition to its skeletal protection activity, zoledronic acid has shown direct activity against prostate cancer cells, both in vitro and in vivo. (15) There is also in vitro and in vivo evidence to suggest synergy between zoledronic acid and chemotherapy in breast cancer cells and anti-angiogenic effects in patients. (41, 42)

Toxicities of the two agents are complementary and administration in combination is expected to be feasible and safe. These aspects were evaluated in the initial Pilot Phase of the trial. Since both agents show considerable promise as single agents and there is in vitro evidence of synergy, we believe there is a strong rationale for evaluating these two agents in combination.

2.9.2 Bisphosphonate And Cyclooxygenase-2 Inhibitors

Note: recruitment stopped to both celecoxib-containing arms in Apr-2011 at the end of Activity Stage II.

An alternative approach to combination therapy is to target the principal site of relapse and a key mode of progression and this is the rationale for combining zoledronic acid with a Cox-2 inhibitor. Bisphosphonates have already been shown to delay bone disease progression in hormone refractory disease. (19) Cox-2 appears to play a crucial role in the molecular phenotype of advanced prostate cancer as outlined above, and this effect is likely to be apparent in both soft tissue and in bone. Toxicities of the two agents are likely to be complementary and there is no strong a priori reason to anticipate unacceptable toxicity. The Pilot Phase of the trial will evaluate tolerability and safety of the combination. Targeting both bone progression and the underlying molecular changes leading to progression can be expected to have synergistic benefits in terms of delaying development of hormone refractory disease.

Figure 7: Use of RT in STAMPEDE

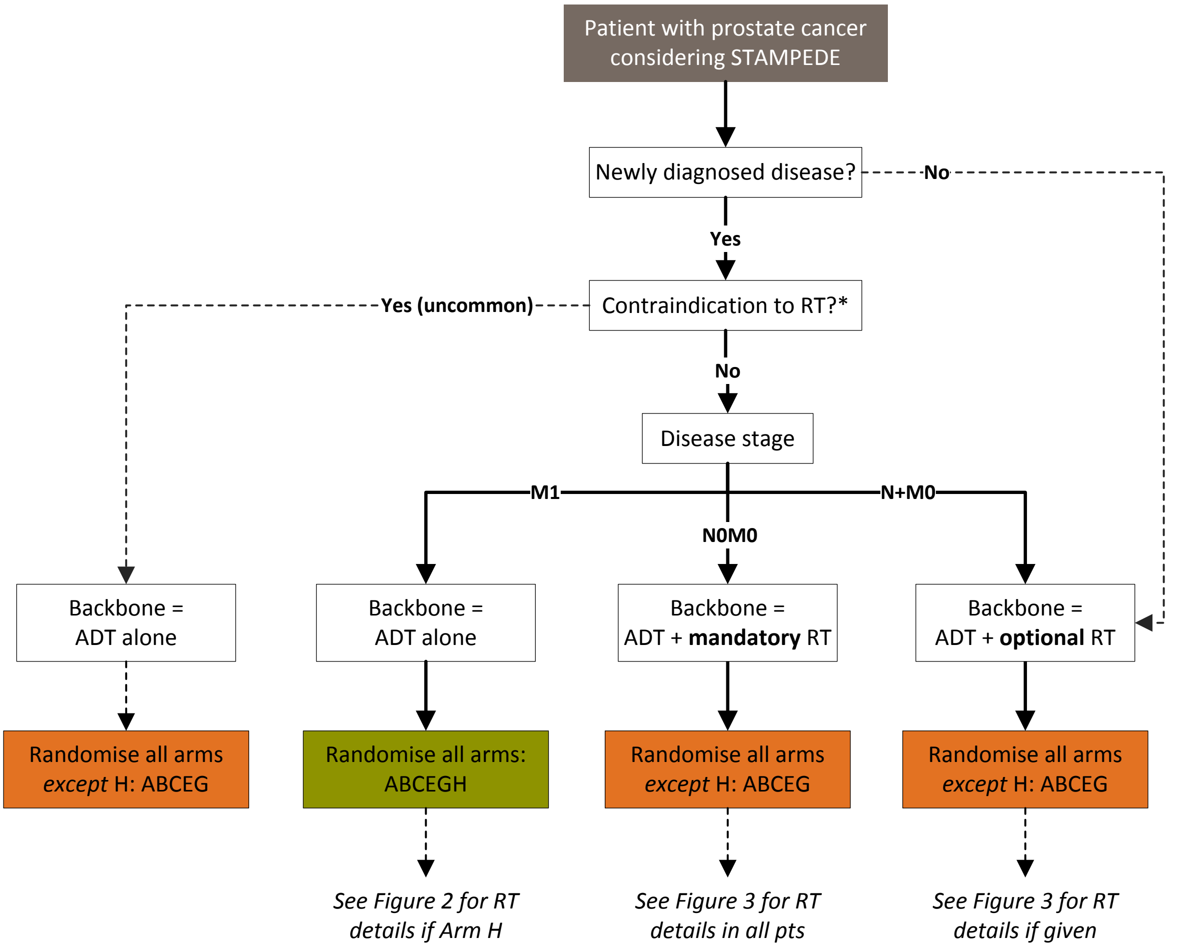

*It is expected that only around 1% of patients will have a contraindication to RT e.g. inflammatory bowel disease. These cases should be discussed with the trials unit prior to randomisation (see **Section** 4.3).

3 SELECTION OF INSTITUTIONS AND INVESTIGATORS

Centres who wish to participate in the STAMPEDE trial should be registered with the Medical Research Council Clinical Trials Unit at University College London (MRC CTU at UCL) for this purpose. Before any patients are randomised the MRC CTU must receive a completed and signed Investigator Statement. The STAMPEDE investigator statement is signed by the Principal Investigator for that institution (**Appendix M**). R&D approval for the site, along with a fully-signed model agreement are also required before recruitment can begin.

In addition and in compliance with the principles of GCP all institutions participating in the trial will complete a delegation log and forward this to the MRC CTU. Each person working on the STAMPEDE trial must sign off a section of this log indicating their responsibilities. The MRC CTU must be notified of any changes to trial personnel and/or their responsibilities. An up-to-date copy of this log must be stored in the Investigator Site file at the institution and also at the MRC CTU.

The Clinical Trial Authorisation (CTA) for the STAMPEDE trial requires that the Medicines and Healthcare Products Regulatory Agency (MHRA) be supplied with the names and addresses of all participating investigators/institutions. Trial staff at the MRC CTU will perform this task; hence, it is vital to receive full contact details for all investigators prior to their entering patients.

Finally, before a patient is entered into the trial written informed consent must be obtained. Approved patient information sheets and informed consent forms are supplied as templates.

Only a limited number of centres participated in the initial Pilot Phase of the original trial; this was to ensure that safety and feasibility data were collected expediently. Subsequent stages of the trial are open to any centre that wishes to participate and has fulfilled the requirements described above.

3.1 RADIOTHERAPY ACCREDITATION

The introduction of the RT comparison in v9.0 introduces the need for RTQA accreditation in sites giving radiotherapy. The details of RTQA accreditation is in **Appendix K**. However, centres that have been RTQA accredited for another multi-centre prostate radiotherapy trial in the UK (e.g. RADICALS or CHHIP) will be automatically granted STAMPEDE RTQA accreditation.

4 SELECTION OF PATIENTS

4.1 PATIENT INCLUSION CRITERIA

Patients must fulfil both of the criteria in **Section 4.1.1** **or** one criterion in **Section 4.1.2** **or** at least one criteria in **Section 4.1.3**. Additionally, all patients must fulfil the criteria in **Section 4.1.4**.

4.1.1 High-Risk Newly Diagnosed Non-Metastatic Node-Negative Disease

Both:

- At least two of: Stage T3/4, PSA≥40ng/ml or Gleason sum score 8-10
- Intention to treat with radical radiotherapy (unless there is a contra-indication; exemption can be sought in advance of consent, after discussion with MRC CTU)

OR

4.1.2 Newly Diagnosed Metastatic Or Node-Positive Disease

At least one of:

- Stage T_{any} N+ M0
- Stage T_{any} N_{any} M+

OR

4.1.3 Previously Treated With Radical Surgery And/Or Radiotherapy, Now Relapsing[1]

At least one of:

- PSA ≥4ng/ml and rising with doubling time less than 6 months
- PSA ≥20ng/ml
- N+
- M+

AND

4.1.4 For All Patients

I. Histologically confirmed prostate adenocarcinoma
II. Intention to treat with long-term androgen deprivation therapy
III. Fit for all protocol treatment[2] and follow-up, WHO performance status 0-2[3]
IV. Have completed the appropriate investigations prior to randomisation
V. Adequate haematological function: neutrophil count >1.5x10^9/l and platelets >100x10^9/l
VI. Estimated creatinine clearance >30ml/min
VII. Serum potassium ≥3.5mmol/L
VIII. Written informed consent
IX. Willing and expected to comply with follow-up schedule
X. Using effective contraceptive method if applicable

[1] Courses of hormone therapy for localised disease must have been completed at least 12 months previously and have been no longer than 12 months in duration. It can have been given as adjuvant or neoadjuvant therapy.
[2] Medical contraindications to the trial medications are given in **Appendix G**
[3] For WHO performance status definitions see **Appendix A**

4.2 PATIENT EXCLUSION CRITERIA[4]

Patients must not fulfil any of the criteria, below.

I. Prior systemic therapy for locally advanced or metastatic prostate cancer except as listed in **Section 4.1.3**.
II. Metastatic brain disease or leptomeningeal disease
III. Abnormal liver functions consisting of any of the following:
   - Serum bilirubin ≥1.5 x ULN (except for patients with Gilbert's disease, for whom the upper limit of serum bilirubin is 51.3µmol/l or 3mg/dl)
   - Aspartate aminotransferase (AST) or alanine aminotransferase (ALT) ≥2.5 x ULN
IV. Any other previous or current malignant disease which, in the judgement of the responsible physician, is likely to interfere with STAMPEDE treatment or assessment
V. Patients with contra-indications to Prednisolone, including active peptic ulceration or a history of gastrointestinal bleeding
VI. Patients with active inflammatory bowel disease
VII. Symptomatic peripheral neuropathy grade ≥2 (NCI CTC)[5]
VIII. Any surgery (e.g. TURP) performed within the past 4 weeks
IX. Patients with significant cardiovascular disease such that, in the investigator's opinion, the patient is unfit for any of the study treatments. This might include:
   - Severe/unstable angina
   - Myocardial infarction less than 6 months prior to randomisation
   - Arterial thrombotic events less than 6 months prior to randomisation
   - Clinically significant cardiac failure requiring treatment (NYHA II-IV)6
   - Cerebrovascular disease (e.g. stroke or transient ischaemic episode) less than 2 years prior to randomisation
   - Patients with uncontrolled hypertension defined as systolic BP greater or equal than 160 mmHg or diastolic BP greater or equal than 95 mmHg
X. Patients receiving treatment with drugs known to induce CYP3A4 (including phenytoin, carbamazepine, Phenobarbital)[7]
XI. Prior exposure to abiraterone
XII. Prior chemotherapy for prostate cancer
XIII. Prior therapy with zoledronic acid other than short-term treatment for hypercalcaemia or low bone density
XIV. Prior exposure to policy of long-term hormone therapy before randomisation (unless as described in **Section 4.4.2**)

[4] The exclusion criteria for patients who have been on a Cox-2-inhibitor for 6+ months has been removed
[5] See **Appendix I** for common toxicity grading
[6] NYHA classifications can be found in **Appendix A**
[7] A full list is included in **Appendix G**

4.3 SELECTION CRITERIA FOR COMPARISON OF RESEARCH (M1) RT FOR METASTATIC DISEASE

All patients meeting criteria in **Section 4.1** and **4.2** are eligible for the trial, but not all can be allocated to the research (M1) radiotherapy arm. The selection criteria for this "RT to the prostate" comparison are:

- Newly diagnosed prostate cancer
- Demonstrable M1 disease
- No contraindication to radiotherapy e.g. no previous pelvic radiotherapy and no history of inflammatory bowel disease
- No previous radical prostatectomy

Patients meeting these criteria will have a chance to be allocated to Arms A and H.

4.4 SCREENING PROCEDURES

4.4.1 INVESTIGATIONS PRIOR TO RANDOMISATION

All patients should have the following examinations performed. The latest available scans should be used:

- CT or MRI of pelvis and abdomen
- Bone Scan (or equivalent e.g. whole body MRI)
- Chest X-ray (only if chest was not included in CT)
- ECG
- PSA Test

The following blood tests within 8 weeks (56 days) prior to randomisation:

- Testosterone (if available)
- Urea and Electrolytes
- Liver function tests
- Serum creatinine
- Serum corrected calcium
- Phosphates
- Magnesium
- Albumin
- Total cholesterol
- HDL cholesterol
- Systolic blood pressure
- Diastolic blood pressure
- Waist circumference measure

Patients who initially fail to meet the eligibility criteria can be re-screened at a later date.

Prior to randomisation:

- Check details of any prior treatments for prostate cancer
- Check any contraindications to radiotherapy

4.4.2 Androgen Deprivation Therapy Prior To Randomisation

It is preferable that patients are not started on hormones prior to randomisation. However, if androgen deprivation therapy has already started, the primary therapy should have not have started more than 12 weeks before randomisation, and the baseline PSA measurement must be taken before this was initiated (please report the latest PSA measurement taken before the start of androgen deprivation therapy).

Short periods (not exceeding 2 weeks duration) of prior anti-androgens to cover tumour flare are allowed but will not be counted in the 12 week time period mentioned above; but a PSA measurement must be taken before this is initiated.

Note that long-term anti-androgen monotherapy is not permitted in the trial for newly recruited patients from version 8.0 (see **Section 6.1**); patients may change treatment to join the trial, provided that they have not had more than 12 weeks of androgen deprivation therapy prior to randomisation. Further details on hormone therapies allowed prior to randomisation are discussed in **Appendix L**.

Relapsing patients previously treated with radical surgery or radiotherapy must have completed a period of hormone therapy at least 12 months previously and have been no longer than 12 months in duration, given as adjuvant or neo-adjuvant therapy.

Note that baseline testosterone measurements will not be required in patients who have already commenced hormone manipulation prior to randomisation.

4.4.3 Hypercalcaemia At Randomisation

For patients who are hypercalcaemic prior to randomisation and require treatment, it is recommended that they are treated with a bisphosphonate and that the treatment should be discontinued when they are stabilised.

4.4.4 NSAIDs And Cox-2 Inhibitors At Randomisation

Note: recruitment completed to both celecoxib-containing arms in Apr-2011 at the end of Activity Stage II

For patients who are currently on a Cox-2-inhibitor and who meet the inclusion criteria, please ensure that treatment is discontinued before randomisation. If the patient is allocated to an arm, which does not include celecoxib (arms A, B, C or E), it is advised that the Cox-2 be replaced with a suitable NSAID.

For patients who are taking an NSAID prior to randomisation and are allocated a celecoxib arm (Arm D or F), a clinical decision should be taken as to whether the patient should continue taking the NSAID alongside the celecoxib. This decision should take into account the risk of gastrointestinal problems, and consideration should be given to the co-administration of a proton pump inhibitor

4.4.5 Starting Trial Treatment

Trial treatment should be commenced as soon as possible after randomisation. Investigators should aim that this is at least within 4 weeks post randomisation and within 12 weeks of starting androgen Deprivation Therapy (see **Section 6**).

Radiotherapy for patients allocated to Arm H should be commenced within 4 weeks from randomisations and continued according to the predefined scheduled unless toxicity is reported. Any delays in starting research radiotherapy should be discussed with the STAMPEDE team and recorded as appropriate in the relevant CRF.

4.4.6 Concomitant Medications

All concomitant medications should be recorded including any vitamin and mineral supplements the patient is taking, regular consumption of NSAID and/or aspirin and use of other bisphosphonates (see **Section 4.3.1**). Of particular interest in this are herbal preparations such as PC-SPES, Prostasol, Saw Palmetto and St John's Wort. All concomitant medications should be continued throughout the trial unless the responsible clinician decides otherwise.

4.5 ADDITIONAL DETAILS FOR PATIENTS JOINING SUB-STUDIES

An additional droplet of blood must be taken if the patient has given their consent to participate in the DNA analysis sub-study.

The local pathologist will also be asked to give the remaining tumour sample for tissue micro array analysis to be carried out, if the patient has given consent for his remaining samples to be used for further analyses. Full details of all sub-studies and instructions relating to the handling of the blood sample are given in **Section 17** and **Appendix D**.

5 RANDOMISATION AND ENROLMENT

Patients will be allocated to any of the open research arms for which they are suitable. Patients with non-metastatic disease or who have had previous local therapy to the prostate or who have a contraindication to radiotherapy will not be allocated to Arm H (see **Section 4.3**).

To enter a patient the randomisation form should be completed carefully and the MRC CTU contacted by phone:

RANDOMISATIONS
To randomise, call MRC CTU, Monday to Friday 0900-1700
excluding public holidays or dates when notice has been given by the CTU.
Tel: +44 (0) 20 7670 4777

A trial number and treatment will be allocated and given over the phone or by return fax. In addition, a letter confirming these details will be sent. The trial number will be the primary way in which the patient will be identified and should be used in all correspondence.

5.1 CO-ENROLMENT GUIDELINES

Ideally, patients should not be participating in any other clinical trial of prostate cancer treatment when they enter STAMPEDE and should not enter any other trials until the patient has had a failure-free survival (FFS) event reported. After this point, the patient may be entered into further, second-line treatment studies. The primary outcome measure of STAMPEDE is overall survival. Participation in post-progression studies should be reported on the Co-enrolment CRF.

Data sharing agreements with “down-stream” trials are encouraged to improve data quality in both trials and to reduce costs to both organisations.

6 TREATMENT OF PATIENTS

6.1 TRIAL TREATMENT

Patients will be randomised to the control arm (Arm A) or one of the research arms. All patients will receive androgen deprivation therapy (ADT) to achieve castration levels of testosterone. The method of ADT is a local choice but must be specified for each patient prior to randomisation. The recommended methods of ADT are given in **Section 6.1.1**. All trial treatments should commence as soon as practically possible after randomisation. Patients having a bilateral orchidectomy should commence any additional treatment within 12 weeks of the operation unless there is a strong clinical reason not to do so. Note that from protocol version 8.0 onwards, bicalutamide monotherapy is no longer a permitted as a trial therapy for new patients (but patients may switch to a permitted therapy to join the trial – see **Section 4.3.2**).

6.1.1 Arm A: ADT Alone Or ADT + Standard-of-Care (M0) RT (Control Arm)

The standard of care for this patient group is **androgen deprivation therapy** (see **Section 6.1.1.1**). For some patient groups, this should now be supplemented with standard radiotherapy (see **Section 6.1.1.2**).

6.1.1.A Hormone Therapy

The permitted methods of ADT are bilateral orchidectomy, LHRH analogues and LHRH antagonists. Anti-androgens alone are not permissible as hormone therapy for patients participating in STAMPEDE, but their use is recommended in the short-term to prevent tumour "flare" which may occur after commencing LHRH analogues. Anti-androgen prophylaxis of tumour flare is not required when using LHRH antagonists. At the time of randomisation, centres will be asked to specify the method of ADT for each patient. Other methods of ADT should be discussed with the Chief Investigator or the Trial Surgeon. The planned duration of ADT should be at least 2 years.

Bilateral orchidectomy: Operations should be performed by appropriately trained surgeons. A total or subcapsular orchidectomy may be performed.

LHRH agonists: LHRH agonists/analogues used according to local practice. The prophylactic use of anti-androgens to prevent tumour "flare" is recommended.

LHRH antagonists: LHRH antagonists used according to local practice. The use of prophylactic use of anti-androgens to prevent tumour "flare" is not necessary.

6.1.1.B Standard-of-care (M0) RT

N0M0 patients: Investigators should give standard radiotherapy (RT) to patients with node negative, non-metastatic disease (N0M0), in accordance with the data from the PR07 and SPCG trials. If there is an intention to omit radiotherapy (e.g contraindications) in patients with N0M0 disease this must be discussed with the Trials Office before consent. See **Section 6.6** for further details of radiotherapy administration.

N+M0 patients: the benefit of radiotherapy in this group is at present uncertain with no firm data to either support or refute its use. However, the PR07 trial included some node positive patients as cross sectional imaging was not a part of the baseline assessment in this trial, which did include

whole pelvis radiotherapy. For patients with node positive, non-metastatic disease, radiotherapy is therefore recommended in suitable cases. Investigators will be asked to state their intention with regards to planned radiotherapy in this group at randomisation. Intention to give radiotherapy (or not) for node positive patients must be stated at randomisation to ensure that there is no bias towards particular combinations of systemic therapy with radiotherapy.

Standard radiotherapy is not a core part of the trial, therefore we intend to collect minimal data about the radiotherapy administered. It is accepted that some patients will develop progressive disease before radiotherapy can be administered and if this occurs the reasons for non-delivery of treatment must be recorded on the radiotherapy form.

6.1.2 Arm B: ADT + Zoledronic Acid

Note: recruitment stopped to the zoledronic acid and docetaxel-containing arms in Mar-2013 at the end of Activity Stage IV.

Androgen deprivation therapy (+/- standard-of-care M0 RT) as described in Section 6.1.1.

Zoledronic Acid: 4mg 15min IV infusion every 3 weeks, for 6 treatments followed by zoledronic acid 4mg 15min IV infusion every 4 weeks up to a maximum of 2 years from the start of the treatment or until disease (including PSA) progression (see Section 7.2). Patients should also receive an oral supplement of 500mg calcium and 400IU vitamin D daily. These doses are available as a combination tablet. See Section 6.2.1 for further information.

6.1.3 Arm C: ADT + Docetaxel

Note: recruitment stopped to the zoledronic acid and docetaxel-containing arms in Mar-2013 at the end of Activity Stage IV.

Androgen deprivation therapy (+/-standard-of-care M0 RT) as described in Section 6.1.1.

Docetaxel: 75mg/m2 Day 1 as 1hr IV infusion, plus prednisolone 5mg bid daily for 21 days. The cycle should be repeated every 3 weeks for a maximum of 6 cycles. The recommended administration schedule, anti-emetic regimen and dose modifications for docetaxel are given in Appendix F. See Section 6.2.2 for further information.

6.1.4 Arm D: ADT + Celecoxib

Note: recruitment completed to both celecoxib-containing arms in Apr-2011 at the end of its Activity Stage II

Androgen deprivation therapy as described in Section 6.1.1.

Celecoxib 400mg bid until the sooner of 1 year or disease (including PSA) progression (see Section 7.2). See Section 6.2.3 for further information.

6.1.5 Arm E: ADT + Docetaxel + Zoledronic Acid

Note: recruitment stopped to the zoledronic acid and docetaxel-containing arms in Mar-2013 at the end of Activity Stage IV.

Androgen deprivation therapy (+/- standard-of-care M0 RT) as described in Section 6.1.1.

Docetaxel: 75mg/m2 Day 1 as 1hr IV infusion, plus prednisolone 5mg bid daily for 21 days. The cycle should be repeated every 3 weeks for a maximum of 6 cycles. The recommended administration schedule, anti-emetic regimen and dose modifications for docetaxel are given in Appendix F. See Section 6.2.2 for further information.

Zoledronic Acid: 4mg 15min IV infusion every 3 weeks, for 6 treatments followed by zoledronic acid 4mg 15min IV infusion every 4 weeks up to a maximum of 2 years from the start of the treatment or until disease (including PSA) progression (see Section 7.2). Patients should also receive an oral supplement of 500mg calcium and 400IU vitamin D daily. These doses are available as a combination tablet. See Section 6.2.1 for further information.

Co-administration of docetaxel and zoledronic acid: Docetaxel 75mg/m2 Day 1 as 1hr IV infusion, plus prednisolone 5mg bid daily followed by zoledronic acid 4mg 15min IV infusion. There is evidence to suggest that the co-administration of docetaxel and zoledronic acid is sequence dependent (39). Consequently, docetaxel should be administered before zoledronic acid

6.1.6 Arm F: ADT + Zoledronic Acid + Celecoxib

Note: recruitment completed to both celecoxib-containing arms in Apr-2011 at the end of Activity Stage II

Androgen deprivation therapy as described in Section 6.1.1.

Zoledronic Acid 4mg 15min IV infusion every 3 weeks, for 6 treatments followed by zoledronic acid 4mg 15min IV infusion every 4 weeks up to a maximum of 2 years from the start of the treatment or until disease (including PSA) progression (see Section 7.2). Patients should also receive an oral supplement of 500mg calcium and 400IU vitamin D daily (Calcichew). These doses are available as a combination tablet. See Section 6.2.1 for further information.

Celecoxib 400mg bid until the sooner of 1 year or disease (including PSA) progression (see Section 7.2). See Section 6.2.3 for further information.

6.1.7 Arm G: ADT + Abiraterone

Androgen deprivation therapy (+/- standard-of-care M0 RT) as described in **Section 6.1.1**.

Abiraterone will be administered as a single 1000mg daily oral dose (4 tablets to be taken together once a day) together with prednisolone or prednisone 5mg daily to prevent secondary mineralocorticoid excess. Abiraterone absorption is increased by food. The tablets should be taken at least 2 hours after food, swallowed whole with some water. No food should be eaten for 1 hour afterwards.

Prednisolone (prednisone in Switzerland) should be taken as a single dose with food in the morning.

In patients with M1 disease, treatment with abiraterone will continue from randomisation until clinical disease progression, consistent with the COU-AA-301 trial (35) i.e., abiraterone would be given for these patients until a composite of PSA progression (as defined in **Appendix J**), radiological progression (appearance of new lesions or progression of existing lesions) and clinical progression (defined as new cancer-related symptoms). It is accepted that these flexible criteria for stopping treatment with abiraterone are open to the investigator's interpretation and discretion. Patients

might continue treatment beyond the first failure-free survival (FFS) event (see **Table 1** in **Section 9.2**); the first FFS event must be reported as per the other arms.

In patients with N0M0 disease or N+M0 disease undergoing radical radiotherapy, treatment would continue for 2 years or disease progression as defined for M1 patients, whichever is the sooner. ADT can be discontinued in this group at 2 years at the discretion of the local investigator (see **Section 6.1.1.A**).

For patients with N+M0 disease not planned for radical radiotherapy, treatment will continue as for patients with M1 disease until disease progression.

See **Section 6.2.4** for further information for all groups.

6.1.8 ARM H: ADT + PROSTATE RADIOTHERAPY IN M1 PATIENTS

Androgen deprivation therapy as described in **Section 6.1.1**.

Radiotherapy will commence as soon as practicable and ideally within four weeks after randomization. Treatment will be according to the guidelines in **Section 6.2.5**. Two radiotherapy dose-fractionation schedules are permitted:

- 36Gy in 6 fractions of 6Gy, administered weekly over 6 consecutive weeks
- 55Gy in 20 fractions of 2.75Gy, administered daily, five days per week, over 4 consecutive weeks

Details of the recommendations for outlining, CTV and PTV are in **Section 6.2.5**.

6.2 ADMINISTRATION AND DOSE MODIFICATIONS

6.2.1 ZOLEDRONIC ACID

Note: recruitment stopped to the zoledronic acid and docetaxel-containing arms in Mar-2013 at the end of Activity Stage IV.

Zoledronic acid will be administered by IV infusion in accordance with the instructions in the summary of product characteristics at a target dose of 4mg (adjusted for renal function, see below) every 3 weeks for the first 6 cycles and every 4 weeks, thereafter.

Serum Creatinine Measurements: Serum creatinine should be measured at baseline and within 48 hours prior to every administration of zoledronic acid. It is permissible to have serum creatinine levels measured on Fridays prior to the administration of zoledronic acid on the following Monday.

Serum Electrolytes and FBC: Serum electrolytes including calcium, phosphate and magnesium should also be measured prior to each infusion. FBC should be measured at least 3 monthly. Zoledronic acid should be discontinued if there is any evidence of hypersensitivity to the drug. In patients with mild to moderate renal impairment, lower doses of zoledronic acid are recommended according to standard dose reduction schedules for administration of this drug. In rare cases, zoledronic acid treatment has been associated with the development of osteonecrosis of the jaw, particularly following dental extractions. If a patient develops osteonecrosis of the jaw then the zoledronic acid should be immediately and permanently discontinued. For full details of zoledronic acid administration and does reductions see Appendix F. Contraindications, special precautions, interactions and side effects are listed in Appendix G.

6.2.2 DOCETAXEL

Note: recruitment stopped to the zoledronic acid and docetaxel-containing arms in Mar-2013 at the end of Activity Stage IV.

The use of docetaxel should be confined to units specialised in the administration of cytotoxic chemotherapy and it should only be administered under the supervision of a physician qualified in the use of anticancer chemotherapy.

Docetaxel will be administered by IV infusion in accordance with the instructions in the summary of product characteristics at a dose of 75mg/m2 (up to a maximum dose of 160mg) on Day 1 of the study treatment period and then every 3 weeks thereafter for a maximum of 6 doses. Patients with a body surface area (BSA) greater than 2.13m2 should be dosed as though they have a BSA of 2.13m2. No ideal weight should be used for BSA calculations. Prednisolone or prednisone 5mg bid will be given until completion of chemotherapy. Additional dexamethasone should be given pre- and post-docetaxel infusion to suppress allergic reactions.

Please note that liver function test (LFTs) should be carried out within a week before the first cycle of docetaxel if an anti-androgen has been administered. This is due to an increased risk of neutropenia associated with docetaxel use following anti-androgen administration. Treatment should be delayed if LFTs are abnormal.

For full details of premedication schedule, recommended anti-emetic regimen and dose modifications for docetaxel see Appendix F. Contraindications, special precautions, interactions and side effects are listed in Appendix G.

Docetaxel in combination with prednisone or prednisolone is indicated for the treatment of patients with hormone refractory metastatic prostate cancer. (20, 21)

6.2.3 CELECOXIB

Note: recruitment completed to both celecoxib-containing arms in Apr-2011 at the end of Activity Stage II. No new patients should be receiving this agent now within the trial.

Celecoxib should be administered in accordance with the instructions in the summary of product characteristics at a dose of 400mg bid orally. Rarely this drug is poorly tolerated and in this instance should be discontinued; particular care should be taken with patients with a history of gastrointestinal disease and patients with significant risk factors for cardiovascular events (see Appendix G). Patients with confirmed severe cardiovascular history should not be in STAMPEDE (see exclusion criteria, Section 4.2). Contraindications, special precautions, interactions and side effects are listed in Appendix G. Dose reductions are not anticipated.

6.2.4 ABIRATERONE

Abiraterone absorption is increased by food. The tablets should be taken at least 2 hours after food, swallowed whole with some water. No food should be eaten for 1 hour afterwards. Prednisolone (prednisone in Switzerland) should be taken as a single dose with food in the morning.

If clinical symptoms or signs suggestive of hepatotoxicity develop, serum transaminases, in particular serum alanine aminotransferase (ALT), should be measured immediately. If a rise in transaminases or bilirubin is confirmed, action should be taken as detailed in **Appendix G**.

6.2.4.A Management of Specific Toxicities from Abiraterone

The safety monitoring and toxicity management plan described below takes into account AEs based on the reported clinical safety data of abiraterone.

Hypokalaemia:
At the initial observation of **Grade 1** hypokalaemia (serum potassium <3.5 mM or below lower limit of normal range, but ≥3.0 mM) and oral potassium supplement will be initiated. The dose of potassium supplement must be carefully titrated to maintain serum potassium at ≥3.5 mM but ≤5.0 mM. Any subject with low potassium while on study or a history of hypokalaemia from a pre-existing or concurrent medical condition will undergo weekly or more frequent laboratory electrolyte evaluation. The investigator should consider maintaining potassium level at ≥4.0 mM in these subjects.

If any subject experiences **Grade 3** hypokalaemia (serum potassium levels <3.0 mM–2.5 mM, NCI CTCAE v4.0) or life-threatening hypokalaemia with potassium levels <2.5 mM (NCI CTCAE v4.0 hypokalaemia grade 4), abiraterone will be discontinued and the subject will be hospitalized for intravenous potassium replacement and cardiac monitoring. After the return of serum potassium to normal, prednisone will be discontinued but the patient can be maintained on abiraterone.

Hypertension:
If **Grade 1-2:** Management per investigator with anti-hypertensive treatment.

If **Grade 3-4:** Withhold abiraterone. Adjust or add anti-hypertensive medications to mitigate the toxicity. When hypertension resolves to **Grade ≤1**, resume abiraterone at full dose with prednisone 5mg bid.

Fluid retention/oedema:
If **Grade 1-2:** Increase prednisone dose to 5mg bid.

If **Grade 3-4:** Withhold abiraterone. Consider addition of mineralocorticoid receptor antagonist eplerenone until resolution of symptoms. When fluid retention/oedema resolves to ≤Grade 1, resume abiraterone at full dose with prednisone 5mg bid.

Abnormal liver function tests:
If **Grade 1** increases in AST, ALT or bilirubin occur (eg, increase in AST or ALT from ULN to 2.5 x ULN; increase in total bilirubin from ULN to 1.5 x ULN): the frequency of liver function test monitoring should be increased, if the investigator judges that the laboratory abnormalities are potentially related to study medication. No dose reduction is required.

If **Grade 2** increases in AST, ALT or bilirubin occur (eg, increase in AST or ALT to >2.5-5 x ULN; increase in total bilirubin from >1.5-3 x ULN): the frequency of liver function test monitoring should be increased to ≥ _once a week, if the investigator judges that the laboratory abnormalities are potentially related to study medication. No dose reduction is required.

If **Grade 3** or higher increases in AST, ALT, or bilirubin occur (eg, increase in AST or ALT to >5 x ULN; increase in total bilirubin to >3 x ULN), withhold abiraterone and all other concomitant medications that are potentially hepatotoxic. Frequent laboratory evaluations (at least once

weekly) should be conducted until the liver function tests return to baseline value or grade 1. If study treatment resumption is considered for subjects who have experienced grade 3 increases in AST, ALT, or bilirubin, resume abiraterone with the first dose level reduction (3 tablets, 750 mg of study treatment) when grade 3 toxicities resolve to grade 1 or baseline.

If **Grade 4** increases in AST, ALT, or bilirubin occur (eg, increase in AST or ALT to >20 x ULN; increase in total bilirubin to >10 x ULN), subjects must discontinue abiraterone and enzalutamide immediately. They should be followed-up until resolution of abnormal liver function tests and then prednisone can be discontinued and the investigator can consider restarting abiraterone.

6.2.4.B Management of Specific Toxicities from Prednisolone/Prednisone

Prednisolone or prednisone will be started at 5mg once daily, to prevent secondary mineralocorticoid excess. Prednisone/prednisolone dose increase of up to 10mg/day is permitted to manage mineralocorticoid-related toxicities (e.g., hypokalaemia, hypertension) which are refractory to standard management. Patients experiencing serious Cushingoid symptoms (e.g., weight gain, muscle loss) can decrease or discontinue (temporarily or permanently) steroids at the investigator's discretion. It should be noted that weight gain and muscle loss are also associated with androgen deprivation therapy.

6.2.5 Research (M1) Prostate Radiotherapy

A treatment planning CT scan will be acquired with the patient supine, with empty rectum and comfortably full bladder.

Megavoltage equipment is required with effective photon energies ≥6MV. Minimum source-to-axis distance is 100cm. Field arrangement is at the clinician's discretion: acceptable treatment techniques (field arrangement) include a 3-field (anterior, right lateral, and left lateral), 4-field (anterior, posterior, right lateral, and left lateral), or 6-field (right and left anterior oblique, right and left posterior oblique, and right and left lateral) or equivalent coplanar technique with multi-leaf collimation for all fields to adequately protect normal structures.

The Clinical Target Volume (CTV) will consist of the prostate gland alone as visualized on the treatment-planning CT scan. The base of the seminal vesicles may also be included if they are macroscopically involved. Inclusion of pelvic lymph nodes in the CTV is not permitted. The Planning Target Volume will have a 0.8 cm margin posteriorly and 1.0 cm margin in all other directions around the CTV to account for prostate gland motion and uncertainty in daily treatment setup.

Critical normal tissues should be delineated on the treatment-planning CT scan by the treating clinician:

- Rectum – inferior limit: level of ischial tuberosities; superior limit: sigmoid flexure
- Bladder – entirety

Two radiotherapy dose-fractionation schedules are permitted. In either case, radiotherapy is prescribed such that the PTV receives at least 95% of the prescribed dose:

- 36Gy in 6 fractions of 6Gy, administered weekly over 6 consecutive weeks
- 55Gy in 20 fractions of 2.75Gy, administered daily, five days per week, over 4 consecutive weeks

Dose-volume objectives for each dose-fractionation schedule are shown in **Tables 3** and **4** below. Values have been calculated using the formula BED = D[1+d/(alpha-beta ratio)] assuming an alpha-beta ratio of 3 for rectum and bladder. These are provided for guidance only.

Table 3: Rectal dose volume objectives

55Gy/20F	36Gy/6F	Max Vol (%)
52.5 Gy	33.3 Gy	50%
43.5 Gy	27.8 Gy	60%
26.1 Gy	16.7 Gy	80%

Table 4: Bladder dose-volume objectives

55Gy/20F	36Gy/6F	Max Vol (%)
52.2	33.3	25%
43.5	27.8	50%

Portal imaging to verify accuracy of treatment delivery may be done according to the participating centre's local guidelines. Image-guidance technology (e.g., gold seed intraprostatic fiducial markers, cone-beam CT scanning) will be permitted according to clinician preference but is not required. Further illustration on the research radiotherapy arm schedule is shown in **Figure 7**.

Figure 8: Diagram for deciding approach to research (M1) RT to the prostate

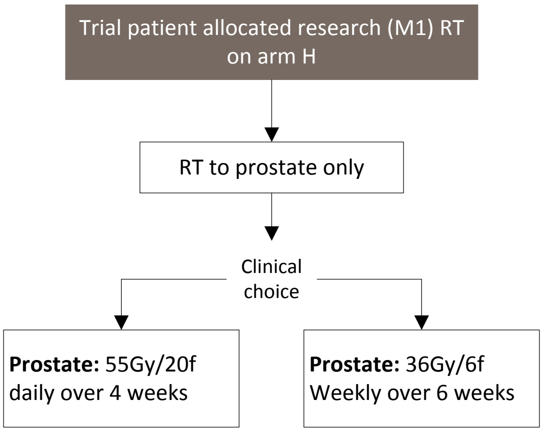

6.3 TRIAL PRODUCTS

Details of the procedures for obtaining the drugs within the trial, dispensing and disposal of unused drug are given in **Appendix E**.

Arrangements for free or discounted drugs are given in the Finance section (**Section 15**).

6.4 MEASURES OF COMPLIANCE/ADHERENCE

Date of treatment, dose, delays and reasons for delays or dose modifications of all study infusions (zoledronic acid and docetaxel) will be recorded. The estimated number of abiraterone tablets taken in a given time period will also be recorded as well as any dose reductions.

6.5 TREATMENT DATA COLLECTION

Data will be recorded on case report forms (CRFs); the top copy/original should be sent to the MRC CTU for data entry and a copy kept at the local centre. Up-to-date versions of all CRFs can be found on the trial website (http://www.stampedetrial.org/) and centres will be notified of any changes throughout the course of the trial. The type of data to be recorded is detailed in the Assessments and Procedures section (**Section 7**).

6.6 ADMINISTRATION OF STANDARD RADIOTHERAPY[8] TO NON-METASTATIC PATIENTS

6.6.1 Treatment Details

Standard radiotherapy will be given to appropriate patients in each of the trial arms, following a period of neo-adjuvant ADT therapy, as is generally standard in UK practice. For patients receiving docetaxel, this period needs to be a minimum of 6 months after randomisation to ensure that chemotherapy is completed and toxicity resolved before RT begins. To ensure consistency of timing of administration of standard radiotherapy in all arms, this same 6 months period is recommended for all patients. For patients with negative nodes on axial imaging, clinicians may choose between irradiating prostate and seminal vesicles alone or including the pelvic nodes in addition. Additional staging tests such as pelvic node sampling may be considered in making this decision. Conformal or intensity modulated radiotherapy should be used in all patients. Where patients have good clinical evidence that nodes are free of tumour or patients for whom nodal radiotherapy is contra-indicated (e.g. significant bowel disease), treatment may be given to the prostate gland and seminal vesicles only. The recommended dose is 74Gy in 37 fractions to the prostate and seminal vesicles or the equivalent using hypofractionated schedules. These recommendations are summarised **Table 3**. Alternative dosing schedules are permitted but must be agreed with the STAMPEDE Trial Management Group.

6.6.1.A Standard-of-care RT Timing in M0 patients

Radiotherapy should be given around 6 to 9 months after randomisation in all trial arms and, if receiving docetaxel, the patient must have recovered from any docetaxel toxicity before RT can begin.

6.6.1.B Type Of standard-of-care RT in M0 patients

Conformal or intensity modulated radiotherapy.

6.6.1.C Standard Clinical Target Volume in M0 patients

- **CTV1:** Prostate plus seminal vesicles
- **CTV2:** (Node positive patients) Regional lymph nodes to include internal iliac and the inferior part of the common iliac nodes as used in EORTC trial 22961 (43)
- **PTV1:** CTV1 plus 10-15 mm according to local practice
- **PTV2:** CTV2 plus 10-20mm according to local practice

6.6.1.D Standard-of-care RT Dose in M0 patients

Prostate dose of 74Gy in 2Gy fractions or equivalent, with optional dose to the pelvic nodes of 46-50Gy in 2Gy fractions or equivalent using IMRT to deliver the treatment over 37 fractions, suggested dose is 55Gy in 37 fractions with IMRT. Higher doses may be considered if the department is experienced in using IMRT for nodal radiotherapy, particularly as data emerges from the PIVOTAL trial of nodal IMRT in high-risk node negative patients where a nodal dose of 60Gy in 37 fractions is being evaluated. Alternative schedules should be agreed with the STAMPEDE Trial Management Group.

[8] **Note:** this text has been transferred into the protocol from the Appendices in version 8.0, and updated

Figure 9: Diagram for deciding recommended approach to standard-of-care (M0) RT in non-metastatic patients

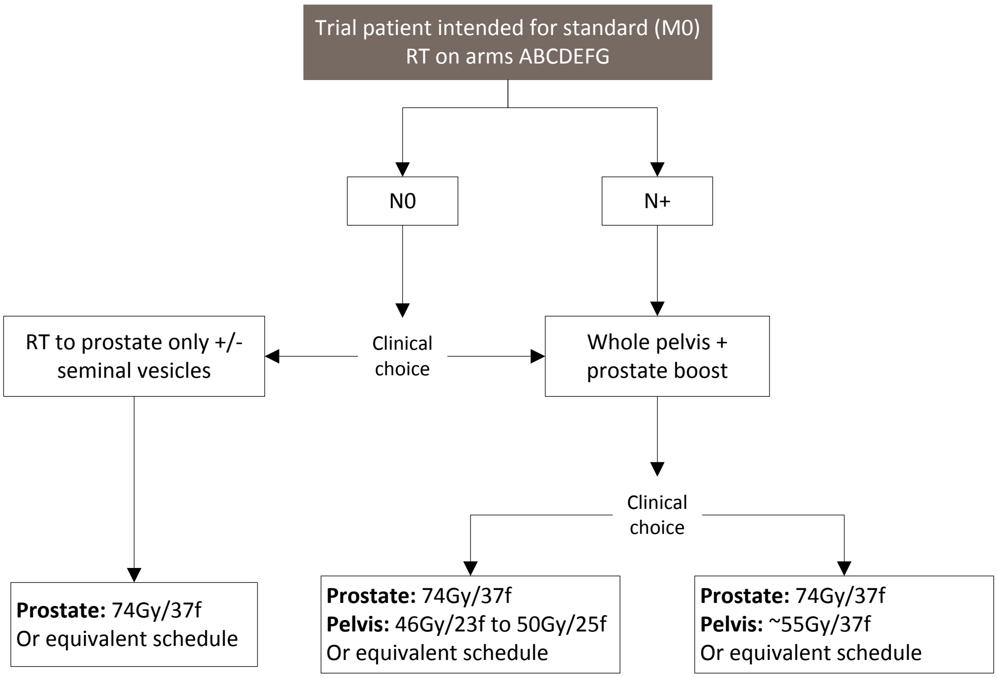

6.7 NON-TRIAL TREATMENT

6.7.1 MEDICATIONS PERMITTED

Any additional treatment that the responsible physician feels is appropriate is permitted.

6.7.2 DATA ON CONCOMITANT MEDICATION

All concomitant medication will be recorded on the baseline form prior to randomisation and on any subsequent Serious Adverse Event forms. This should include aspirin that may be taken on a regular basis for cardiovascular disease, the use of any Non-Steroidal Anti-inflammatory Drugs (NSAID) as well as any vitamin or mineral supplements the patient is taking.

7 ASSESSMENTS AND PROCEDURES

7.1 FLOW CHART/SCHEDULE FOR FOLLOW-UP

A detailed follow-up schedule is given in **Table 5**.

7.1.1 PSA MEASUREMENTS

All patients should have PSA measured pre-androgen deprivation therapy and at weeks 6, 12, 18 and 24 and every 12 weeks, thereafter, up to 2 years post randomisation. Following this, PSA should be measured every 6 months until 5 years and annually, thereafter. For patients who do not have a scheduled hospital visit, it would be acceptable for arrangements to be made for blood samples to be drawn in a GP's surgery.

7.1.2 ASSESSMENT OF TREATMENT FAILURE (DEFINITION OF PROGRESSION)

It is not proposed to routinely assess patients for response. However, in order that objective progression can be assessed, it is necessary to have imaging taken at time of best response as judged by the treating clinician. All patients should have baseline radiological examinations as detailed in **Section 4.3.1**. In addition it is recommended that all patients should have scans or X-rays repeated at 24 weeks (and whenever clinically appropriate) if they were abnormal at baseline, particularly if they have a low PSA value on entry in to the trial making biochemical assessment of treatment failure difficult. The following events would constitute a disease progression and should be reported on a progression form:

- Biochemical failure – must be reported alongside castrate levels of testosterone if the patient has received intermittent ADT (see **Appendix J**).
- Local progression
- Lymph node progression
- Progression in distant metastases
- Development of new metastases

Please note that skeletal-related events (SREs) may be indicative of disease progression but can have other causes such as osteoporotic fracture. All SREs should be investigated further to establish whether or not the patient has progressed, in which case a progression form should be completed.

7.1.3 ADDITIONAL SAFETY ASSESSMENT

Due to the risk of liver toxicity and secondary hyperaldosteronism with abiraterone, patients will require 2 weekly U+Es, LFTs and blood pressure measurement for the first 12 weeks. It is not proposed to collect the detail of these measurements unless results are abnormal; in this instance, they should be reported as AEs (on the next Follow-up CRFs) and as an SAEs (see **Section 11**) if appropriate.

Medical review and PSA measurements follow the pattern in the control arm: visits at weeks 6, 12, 18 and 24 and every 12 weeks, thereafter, up to 2 years post randomisation. Following this, PSA should be measured every 6 months until 5 years and annually, thereafter. For patients who do not have a scheduled hospital visit, it would be acceptable for arrangements to be made for blood samples to be drawn either in a GP's surgery or in the patient's home.

7.1.4 Data Collection and Non-Administration Of Standard Radiotherapy

There are CRFs to be completed for patients receiving primary radiotherapy whether this is standard radiotherapy for M0 patients on any arm or prostate radiotherapy for Arm H patients. All radiotherapy and acute side effects details will be recorded on the Radiotherapy Form; any late side effects will be recorded on the Follow up form.

If it is decided not to give the planned radiotherapy (for example, due to early metastatic progression or patient refusal), this should be stated on the Standard Radiotherapy form together with the reason for non-administration of the treatment.

7.1.5 Data Collection Palliative Radiotherapy

For patients who receive palliative radiotherapy as part of first line treatment, a Palliative Radiotherapy CRF should be completed. Details of salvage RT for relapse and palliative treatment will be requested and completed only on the Progression Form.

7.1.6 Data Collection Research (M1) Radiotherapy

There are arm specific CRFs for patients randomised to arm H. Adverse events such as hip fractures, TURPs, skeletal-related events will be collected retrospectively via the Hospital Episode Statistics (HES) database.

7.1.7 Follow-Up Schedules

An individualised form with a follow-up schedule will be provided for each randomised patient. For patients who are receiving LHRH analogues, it is assumed that any additional treatment will commence within two weeks of randomisation. For patients who are due to have an orchidectomy it is recognised that surgery will have to be scheduled and the scheduling of any additional treatments may be affected by post-operative recovery. It is recommended that all patients who had abnormal radiological investigations at baseline or present with a low PSA on entry into the STAMPEDE trial should have them repeated 24 weeks after randomisation.

7.2 FOLLOW-UP

Every effort should be made to follow-up all patients who have been randomised. Patients should, if possible, remain under the care of an oncologist or urologist for the duration of the trial. If care of a patient is returned to the GP, it is the responsibility of the consultant who obtained the patient's consent to participate in the trial to ensure that the data collection forms are completed. If the patient moves from the local area, arrangements should be made for trial follow-up to be undertaken by their new local centre. Details of other participating centres can be obtained from the MRC CTU. The consent of patients should be obtained for their names to be flagged for survival information through national registries, for example NHS Information Centre/Office of National Statistics (ONS) in England/Wales and General Register Office in Scotland, Hospital Episode Statistics (HES). If the clinician moves, appropriate arrangements should be made to arrange for trial follow-up to continue at the centre.

Table 5: Summary of timing of case report forms

CASE REPORT FORMS	TIMING OF ASSESSMENT AND CRF
Bone Density Risk Factor	At randomisation
Randomisation	At randomisation
Baseline	At randomisation
Cardiovascular Assessment	At randomisation
Pathology	At randomisation. When pathology sample has been taken and sent to UCL laboratory.
Pre-18 Week Bisphosphonate	Treatment administered every 3. Form holds data for 2 cycles. Form to be sent after 2nd cycle given.
Post-18 Week Bisphosphonate Treatment	Treatment administered every 4. Form holds data for 3 cycles. Form to be sent after 3rd cycle given.
Docetaxel Treatment	Treatment administered every 3 weeks Form holds 2 cycles. Form to be sent after 2nd cycle given.
RT detail	When standard radiotherapy is completed or if planned RT is no longer to be given (all patients planned for RT in Arms A to G) or when research radiotherapy (Arm H) is completed or at 3 months (Arm A)
RT Acute Toxicity	For all patients who receive primary RT.
Follow-Up	Every 6 weeks for 6 months, then every 12 weeks until 2 years. Every 6 months until 5 years and annually thereafter. (See **Table 6** for more information.)
Palliative Radiotherapy	If applicable, when the palliative radiotherapy course is completed.
End of Treatment	When each treatment is completed (either at end of scheduled treatment or at early cessation of treatment).
Progression & Additional Treatment	At the first occurrence of each type of progression and whenever a patient that has progressed receives additional treatment.
Serious Adverse Event	Following any Serious Adverse Event
Skeletal-related Event	Whenever a patient experiences a skeletal-related event
Death	At Death
Patient Transfer	When a patient is transferred to a different hospital for the administration of trial treatment and follow up

Note: Quality of Life Study is only for first 700 patients entered into the trial and those who were recruited after the implementation of version 8.0 of the protocol. MRC CTU will inform centres of which of their patients this applies to.

Table 6: Data required on follow-up forms

Timing of follow-up	PSA	Evidence of Progression	Androgen deprivation therapy	Abiraterone treatment	Unscheduled Visits	Toxicities
Before progression	✓	✓	✓	✓	✓	✓
After Progression	-	✓	✓	✓	✓	✓

7.3 TRIAL CLOSURE

For the purpose of complying with UK Clinical Regulations introduced on May 2004, the trial will be considered 'closed' when the follow-up point for the primary analysis of the final comparison has been reached. However, further observational follow-up of all patients enrolled in the trial will continue until all randomised patients have died. This will initially be via the hospital, but in the longer term may employ national registers.

Table 7: Schedule for completion of treatment and outcome forms by arm.

Timing From Randomisation			Treatment Forms			Outcome forms	
Years	Months	Weeks	Zol. Acid	Docetaxel	RT	Follow-up$^{\Psi}$	QL + HE$^{¥}$
6-Weekly							
-	-	6	B,E,F (†)	C,E (†)	-	All arms	All arms
-	-	12	B,E,F (†)	C,E (†)	M1: A,H	All arms	All arms
-	-	18	B,E,F (†)	C,E (†)	-	All arms	All arms
-	-	24	B,E,F (‡)	-	-	All arms	All arms
12-Weekly							
-	-	36	B,E,F (‡)	-	-	All arms	All arms
-	-	48	B,E,F (‡)	-	M0: A,B,C,E,G	All arms	All arms
-	-	60	B,E,F (‡)	-	-	All arms	All arms
-	-	72	B,E,F (‡)	-	-	All arms	All arms
-	-	84	B,E,F (‡)	-	-	All arms	All arms
-	-	86	B,E,F (‡)	-	-	All arms	All arms
6-Monthly							
2	24	104	B,E,F (‡)	-	-	All arms	All arms
	30	130	-	-	-	All arms	All arms
3	36	156	-	-	-	All arms	All arms
	42	182	-	-	-	All arms	All arms
4	48	208	-	-	-	All arms	All arms
	54	234	-	-	-	All arms	All arms
5	60	260	-	-	-	All arms	All arms
Annual							
6	-	-	-	-	-	All arms	All arms
7	-	-	-	-	-	All arms	All arms
Etc	-	-	-	-	-	All arms	All arms

Key:
A = ADT alone
B = ADT + zoledronic acid
C = ADT + docetaxel
D = ADT + celecoxib
E = ADT + zoledronic acid + docetaxel
F = ADT + zoledronic acid + celecoxib
G = ADT + abiraterone
H = ADT + M1 research RT to the prostate

Notes:
$^{\Psi}$ See Table 6 for information required at follow-up
† Form records data for two cycles
‡ Form records data for three cycles
¥ 1st 700 patients and those recruited from protocol version 8.0 onwards only

Note: recruitment completed to both celecoxib-containing arms in Apr-2011 at the end of Activity Stage II

Radiotherapy, Late RT Toxicity, Palliative Radiotherapy Progression, SAE, End of Treatment, Co-enrolment and Death forms to be completed as required.

8 STOPPING OF TREATMENT OR FOLLOW UP

Patients should be given every encouragement to adhere to protocol treatment and follow-up schedule, in order to reduce biases. However, a patient has the right to withdraw consent for participation in any aspect of this trial at any time.

8.1 STOPPING RESEARCH INTERVENTIONS

A patient may stop trial treatment for the following reasons:

- Progression whilst on therapy (trial treatment must be discontinued in this instance). For patients randomised to Arm G, please refer to **Section 6.1.7** for criteria to stop treatment
- Unacceptable toxicity
- Intercurrent illness which prevents further treatment
- Withdrawal of consent for treatment
- Any alteration in the patient's condition which justifies the discontinuation of treatment in the clinician's opinion
- Intention to commence a new anti-cancer treatment due to evidence of relapse.

The reason should be recorded on the treatment and/or follow-up forms as well as the End of Treatment form. In the case of abiraterone, the disease event for stopping abiraterone may be after the first reportable failure-free survival event (see **Section 6.1.7**). Unless a patient states otherwise, it should be assumed that consent is given to continue to record trial data.

8.2 PATIENT TRANSFERS

For patients moving from the area, every effort should be made for the patient to be followed-up at another participating trial centre and for this trial centre to take over responsibility for the patient. To document the transfer process the main contact person at both the current and receiving hospitals should complete and sign the Patient Transfer Confirmation form. A fully completed form must be returned to the CTU prior to the patient transfer and ideally any data queries for the patient should be completed prior to transfer.

On receipt of the completed transfer form a member of the STAMPEDE team will confirm the database has been updated and request confirmation of the name of the patient's new Clinician. Photocopies of the following documents may then be sent to the new hospital to complete the transfer and copies must be also retained at the original site for monitoring purposes:

- Consent form
- Completed CRFs
- Any documentation relating to the patient's participation in STAMPEDE (patient names must be removed from any documentation).

8.3 WITHDRAWAL FROM THE TRIAL COMPLETELY

If a patient explicitly withdraws consent to have any data recorded their decision must be respected and the MRC CTU must be informed in writing. All communication surrounding the withdrawal should be noted in the patient's records and no further STAMPEDE CRFs should be completed for that patient.

Early stopping of follow-up should not be undertaken lightly and the site must consider the implications for the trial and the patient in reaching such a decision.

Patients can change their minds about withdrawal at any time and re-consent to participate in the trial. Follow-up data should be collected only from the point of when consent was re-instated.

9 STATISTICAL CONSIDERATIONS

9.1 METHOD OF RANDOMISATION

Patients will be randomised centrally using a computerised algorithm developed and maintained by the MRC CTU. Randomisation will be performed using the method of minimisation over a number of clinically important stratification factors with an additional random element. To decrease determinability, the factors are not listed here but can be found in the Statistical Design Document.

Table 8 shows the allocation weighting for each arm by protocol version. The relative weighting within each pairwise comparison remains constant throughout.

9.1.1 TO VERSION 7

From the outset, the trial had 1 control arm (A) and 5 research arms (B, C, D, E and F).

As the control arm is the comparator arm for all the research arms, twice as many patients were recruited to the control arm as to each of the original research arms as this is an efficient design. Therefore, the initial randomisation ratio will be A2:B1:C1:D1:E1:F1. From version 7.0, accrual to the celecoxib-containing arms was halted and the allocation ratio was A2:B1:C1:D0:E1:F0.

9.1.2 VERSION 8

From version 8.0, an additional research arm (G) was introduced. The allocation weighting for the additional arm G is 2, meaning that as many patients are contemporaneously randomised to arm G as the control arm A: the randomisation ratio is 2:2 (equivalent to 1:1 control:abiraterone). This gave an overall allocation ratio of A2:B1:C1:D0:E1:F0:G2. When recruitment has been completed to the ongoing original research arms B, C and E (which will be around 2 years before completion of accrual to arm G), the allocation ratio will be A2:B0:C0:E0:D0:F0:G2 (or A2:G2). This is more efficient for this comparison than the 2:1 allocation ratio employed for the original research arms because of the minimal co-recruitment period.

Version 9.0 introduced a RT comparison for men with newly-diagnosed metastatic disease which is irrelevant to a subset of men joining STAMPEDE. This could only be achieved by splitting the randomisation system so that newly-diagnosed patients with M1 disease and no contraindication to RT are randomised A2:B1:C1:D0:E1:F0:G2:H2 and other men are randomised A2:B1:C1:D0:E1:F0:G2:H0. Note that the allocation ratio for each pairwise comparison in unaffected, only the rate at which comparisons accrue.

9.1.3 VERSION 10 AND 11

Version 10.0 followed the successful completion of recruitment to arms B, C and E. Therefore, the allocation ratio will be A2:B0:C0:E0:D0:F0:G2 (or A2:G2) for M0 patients and A2:B0:C0:E0:D0:F0:G2:H2 for M1 radiotherapy arm patients (2A:2G:2H).The equal allocation ratio is suitable with fewer research arms open.

Table 8: Allocation to each arm by protocol version

PROTOCOL	NEWLY-DIAGNOSED M1 PATIENTS								OTHER PATIENTS							
VERSION	A	B	C	D	E	F	G	H	A	B	C	D	E	F	G	H
V1	2	1	1	1	1	1	-	-	2	1	1	1	1	1	-	-
V2	2	1	1	1	1	1	-	-	2	1	1	1	1	1	-	-
V3	2	1	1	1	1	1	-	-	2	1	1	1	1	1	-	-
V4	2	1	1	1	1	1	-	-	2	1	1	1	1	1	-	-
V5	2	1	1	1	1	1	-	-	2	1	1	1	1	1	-	-
V6	2	1	1	1	1	1	-	-	2	1	1	1	1	1	-	-
V7	2	1	1	*0*	1	*0*	-	-	2	1	1	*0*	1	*0*	-	-
V8	2	1	1	*0*	1	*0*	2	-	2	1	1	*0*	1	*0*	2	-
V9	2	1	1	*0*	1	*0*	2	2	2	1	1	*0*	1	*0*	2	2
V10	2	*0*	*0*	*0*	*0*	*0*	2	2	2	*0*	*0*	*0*	*0*	*0*	2	2
V11	2	*0*	*0*	*0*	*0*	*0*	2	2	2	*0*	*0*	*0*	*0*	*0*	2	2

9.2 OUTCOME MEASURES

The overall, definitive primary outcome measure for the trial for each comparison is overall survival (all-cause mortality). The design of the trial is such that it is important to have additional intermediate outcome measures to assess each research arm as the trial progresses. These are listed in **Table 9**. The intermediate primary outcome measure is failure-free survival. The reasons for different emphases in each recruitment stage are explained in **Section 9.3**.

Table 9: Trial Outcome Measures by Comparison Stage

TRIALS STAGE	PRIMARY OUTCOME MEASURES	SECONDARY OUTCOME MEASURES
Pilot phase	Safety*	Feasibility
Activity Stage (AS) I-III	Failure-free survival (FFS)†	Overall survival (OS)<br>Toxicity<br>Skeletal-related events
Efficacy Stage (ES) IV	Overall survival	Quality of life<br>Cost effectiveness<br>Failure-free survival†<br>Toxicity<br>Skeletal-related events

*Based on toxicity
†Including biochemical failure (see **Appendix J**)

9.3 SAMPLE SIZE: PRINCIPLES AND ASSUMPTIONS

The overall design for this study is a multi-arm multi-stage, multi-centre randomised controlled trial. There are five stages for each research arm: a Pilot Phase, Activity Stages I-III and Efficacy Stage IV. Full details of the methodology underlying the trial design are given by Royston et al. (44, 45) The sample size calculations were performed using the `stage2` (version 1.2.0, March 2002) and `stagen` (version 1.1.1, 18 May 2004) programs, both implemented in Stata (Stata Corp, TX) and updated using the later `nstage` program (version 1.0.3, 13-jun-2007; version 2.1.0, 28-jun-2009). (46)

The trial was designed under the assumptions in **Table 10**, and additionally, we assume a slightly higher proportion of non-metastatic than metastatic patients joining the trial such that the median FFS is two years and median OS four years for the whole cohort.

Table 10: Hazard ratio assumptions under null and alternative hypotheses

Size of HR	Pilot	AS I-III	ES IV
Under null hypothesis (H0)	n/a	HR(FFS) = 1.00	HR(OS) = 1.00
Under alternative hypothesis (H1)	n/a	HR(FFS) = 0.75	HR(OS) = 0.75

The HR of 0.75 for any research arm relative to control would translate into an absolute improvement in FFS of 10%, from approximately 50% to 60% at two years and OS of 10%, from approximately 50% to 60% at four years. A beneficial difference of this size would be clinically worthwhile and, indeed, experience tells us it may be unrealistic to expect a larger difference. Therefore, we have adequately powered the trial to detect a HR of 0.75 for overall survival. This design gives 95% power at Activity Stages I-III and 90% power at Efficacy Stage IV for each comparison. Further details of the sample size calculations are summarised in **Sections 9.4** and **9.5** and detailed in a separate Statistical Design Document which is available on request.

Note that, from version 8.0, standard-of-care M0 RT was introduced to the majority of patients with N0 M0 disease. This is likely improve the outcomes for this group. Further agents are starting to be licensed for patients with castration-refractory disease which may also improve survival rates. Improved FFS rates would delay the intermediate analyses; improved survival rates would delay the definitive analyses. The Statistical Design Document includes models where median survival is estimated at 5 years rather than 4 years. The trial is powered to detect a difference in relative improvement and the analyses will be performed when a pre-planned number of events has been reported in the control arm, rather than after a certain number of patients have been recruited or a certain amount of time elapsed. **Sections 9.4** and **9.5** provide more detail.

9.4 SAMPLE SIZE ISSUES AND TRIAL STAGES: ORIGINAL RESEARCH ARMS (B-F)

9.4.1 Pilot Phase: Original Research Arms (B-F)

It was anticipated that 210 patients would be recruited to the Pilot Phase from a limited number of centres over a one year period. Approximately 60 patients would be randomised to the control arm and 30 patients to each of the five research arms, each of which were assessed for safety and feasibility. If recruitment proved unfeasible or any of the research arms proved unsafe or not feasible to administer (e.g., poorly tolerated or unexpected toxicity) recruitment to these arms

would have been discontinued. There were already considerable safety data on the use of docetaxel and zoledronic acid in patients with malignancies including prostate cancer, and on the use of Cox-2 inhibitors (including celecoxib), although mainly from patients with musculoskeletal disorders. There were fewer data on the combination arms, but it was thought very unlikely that any of the research arms would be discontinued during the Pilot Phase. When 210 patients had been on the trial for a minimum of 18 weeks, the Independent Data Monitoring Committee (IDMC) reviewed the data from the Pilot Phase and continued to the trial during this period as equipoise remained. Recruitment continued beyond this point. Safety data are assessed throughout the trial.

9.4.2 Activity Stages I-III: Original Research Arms (B-F)

In the sample size calculations, we assumed that all research arms successfully pass through the Pilot Phase to Activity Stage I and that patients would be recruited at a rate of approximately 500 per year. This was faster than in the Pilot Phase because the trial would recruit from additional centres, both in the UK and internationally. The analysis of Activity Stages I, II and III were planned for when around 113, 216 and 334 failure-free survival events had been observed in the control arm, respectively.

The Activity Stage analyses comprise pairwise comparisons of FFS between the control arm and each of the 5 research arms (i=B, C, D, E, F). Let HR_i(true) represent the hazard ratio (HR) of the ith research arm to the control arm, and HRi(observed) the observed value. Discontinuation of accrual of further patients was considered for the ith research regimen at each of Activity Stages I-III according to the guidelines in **Table 11**.

Table 11: Guidelines for stopping accrual to the ith original research arm

Activity Stage	Number of control arm events	Consider discontinuation if HR_i(observed) is…
I	~113	>1.00
II	~216	>0.92
III	~334	>0.89

9.4.3 Efficacy Stage IV: Original Research Arms (B-F)

The analysis of Efficacy Stage IV for the original research arms will be performed when around 403 deaths have been observed in the control arm. This would give 90% power to detect the targeted hazard ratio of 0.75 at one-sided significance level of 0.025. The actual length of this stage, balancing continued accrual with just follow-up, depended on the number of arms passing through to further recruitment from Activity Stages I-III and the observed accrual and event rates.

9.4.4 Sample Size For Original Research Arms (B-F)

Assuming an accrual rate of 500 patients/year, between 2800 and 3600 patients were planned to be entered into the original research comparisons of the trial over a period of 5½ and 7 years. The exact number of patients to be entered depends on the observed accrual rate and the observed event rate, which is, in itself, dependent on the mix of patients joining the trial from the broad spectrum of eligibility. The primary analysis on overall survival requires around 403 deaths to be observed on the control arm. Accrual continued until the main analysis can be foreseen so that the overall duration of the comparisons would be as short as possible (longer accrual facilitates this) and so that few, if any, patients remain on treatment when the main results are released. The statistical team have

monitored and projected the analysis timelines using the `artpep` command in Stata. Results should be due in 2015. Further information is available in the Statistical Master File.

9.5 SAMPLE SIZE ISSUES AND TRIAL STAGES: ADDITIONAL RESEARCH ARM G

9.5.1 Pilot Phase: Additional Research Arm G

A similar approach is being followed for the additional research arm G, as detailed for the original research arms in **Section 9.4.1**. The IDMC reviewed safety data, in the context of data from the control arm, when the first 30 patients allocated to arm G had been on trial for at least 18 weeks.

Furthermore, an additional review of safety was performed when 30 patients with newly diagnosed non-metastatic disease allocated to arm G had been on trial for at least 18 weeks.

Both of these milestones were successfully completed.

9.5.2 Activity Stages I-III: Additional Research Arm G

The same principles are applied to the new comparison as to the previous comparisons. The notable difference will be in the accrual rate to this comparison which is anticipated to be higher. There are two reasons for this. First, STAMPEDE started to recruit slowly in only a limited number of pilot sites. As more sites have been activated, including internationally, accrual has increased. At the time of version 8.0 of the protocol, monthly accrual to the study was averaging around 60 patients/month (over 700 patients/year). Second, there is an equal allocation ratio for the abiraterone arm compared to the control arm. It is this different allocation ratio which means that the number of control arm events required to trigger the intermediate analyses is different for the assessment of abiraterone to the assessment of the original research arms. This is shown in **Table 11**.

Table 12: Guidelines for stopping accrual to the additional research arm G

Activity Stage	Number of control arm events	Consider discontinuation if HR_G(observed) is...
I	~75	>1.00
II	~142	>0.92
III	~221	>0.89

9.5.3 Efficacy Stage IV: Additional Research Arm G

The analysis of Efficacy Stage IV for the additional comparison will be performed when around 267 deaths have been observed in the control arm. This would give 90% power to detect the targeted hazard ratio of 0.75 at a one-sided significance level of 0.025.

9.5.4 Sample Size For Additional Research Arm G

Up to around 1,800 patients will join the abiraterone comparison, with half allocated to the research arm. Consideration will be given to ceasing further randomisations to arm G if it is not showing sufficient evidence of activity at the interim analyses, just as was done for research arms B to F.

The original plan intended for accrual to be halted either when 1,500 patients had been recruited or after 3 years, whichever was the sooner, providing the accrual rate remained above 50 patients/months.

The total number of patients joining this comparison depends not just on the same issues as the original comparisons (notably, observed accrual and event rates), but also the length of time that the original research arms co-recruit alongside the additional research arm; it was originally assumed that this would be for approximately 1 year, but it was closer to 1.5 years. The sample size calculations and projected durations are fairly robust to changes in the length of co-recruitment with the original research arms and future co-recruitment with any further research arms which the Trial Management Group may introduce. Many scenarios are detailed in the Statistical Design Document.

In Sep-2013, the target sample size for the abiraterone comparison was increased from around 1,500 patients to around 1,800 patients, with the efficacy analysis still to be triggered by 267 control arm deaths. This increase in sample size was primarily because of an increase in the proportion of non-metastatic patients joining the comparison; this related to the activation of Arm H which only recruits patients with newly diagnosed metastatic disease and thereby reduces the numbers of metastatic patients randomised to the abiraterone comparison. Non-metastatic patients have a lower event rate than the metastatic patients and maintaining the same overall sample size would lead to a delay in time to the primary analysis. The increase in sample size was achievable because recruitment rates to the trial had been substantially higher than 50 patients/month for the preceeding 6 months.

9.6 SAMPLE SIZE ISSUES AND TRIAL STAGES: ADDITIONAL RESEARCH ARM H

9.6.1 Pilot Phase: Additional Research Arm H

A similar approach will be followed for the additional research arm H as detailed for the original research arms in **Section 9.4.1**. The IDMC will review safety data, in the context of data from the control arm, when the first 30 patients allocated to arm H have been on trial for around 18 weeks.

9.6.2 Activity Stages I-III: Additional Research Arm H

The same principles will be applied to the new comparison as to the previous comparisons and an equal allocation ratio of control arm patients to patients allocated arm H will be employed, as for Arm G. The number of control arm events required to trigger the intermediate analyses will be the same as for the abiraterone comparison (see **Table 13**).

9.6.3 Efficacy Stage Iv: Additional Research Arm H

The analysis of Efficacy Stage IV for the additional comparison will be performed when around 267 deaths have been observed in the control arm. This would give 90% power to detect the targeted hazard ratio of 0.75 at one-sided significance level of 0.025.

9.6.4 Sample Size For Additional Research Arm H

Consideration will be given to ceasing further randomisations to arm H if it is not showing sufficient evidence of activity on the intermediate primary outcome measure (FFS), just as for the other research arms. This research comparison is relevant to around 60% of patients joining STAMPEDE. At the point of the scientific approval, accrual was averaging around 80 patients per month to the trial. If accrual to the trial was slower at 70 patients per month, then accrual to this comparison could be between 18 and 42 patients per month, depending on which other trial arms are open to recruitment at the time.

We are targeting a 25% relative improvement in overall survival following local radiotherapy to the prostate in this patient group. This is the same size of effect targeted with the other research arms in STAMPEDE. This relative improvement can be further justified in the light of MRC PR07 which demonstrated an improvement of this magnitude for adding radiotherapy to ADT in locally advanced disease, with a hazard ratio for overall survival of 0.77 (95% CI 0.61 to 0.98). In that trial, fewer than half of the deaths were from prostate cancer, whereas in newly diagnosed metastatic patients nearly all men will die of their disease. Therefore, it is relevant to note the relative benefit of radiotherapy in PR07 in terms of prostate cancer-specific survival, where the hazard ratio was 0.54 (95% CI 0.27 to 0.78). Long-term survival-based data, with a median follow-up of ~10 years, were presented orally at the American Society of Clinical Oncology 2012 which confirmed these findings.(7)

We anticipate that around 1250 patients are required over 4 years to observe 267 control arm deaths after 5.25 years. In addition to the factors listed in **Section 2.1.2**, this assumes that (i) recruitment is constantly 70 pts/m to the trial overall, (ii) the original research arms stop accrual within 6 months after activation of the RT arm, (iii) the abiraterone arm stops accrual around 24 months after activation of the RT arm, and (iv) a further new research arm with an equal allocation ratio is introduced 18 months after activation of the RT arm.

With variations on these factors, between 1000 and 1400 patients are required over 2.75 to 4.50 years to address survival within 4.50 to 6.50 years. These sample scenarios will be documented in the Trial Master File.

All patients joining the trial will be starting long-term ADT for the first time. The focus of this comparison will be on the newly diagnosed, metastatic patients (and no contraindications to RT), which is the largest subgroup of patients in the trial and the group of patients at highest risk of death from prostate cancer. Patients with non-metastatic disease will be excluded from this particular comparison as there are already randomised data demonstrating the survival benefit from radiotherapy in patients with locally advanced disease. Radiotherapy is now mandatory in node negative patients; it is also recommended in the node-positive, non-metastatic (N+ M0) group.

For the control arm of the whole trial, we constructed sample size scenarios median failure-free survival being 18, 24 or 30 months and constructed sample size scenarios around each of these options; the event rate would depend on the patient mix. We now know that 60% of patients have M1 disease at trial entry and we have reported that FFS at 24 months is 51% across the whole of the control arm.(1)

For the sample size calculation for this new planned comparison, we have based our estimates on the subgroup of patients with newly diagnosed M1 disease in the control arm. Therefore, we estimate median FFS to be 1 year and estimate that median overall survival will be 3.5 years.

Figure 10: Progress Of STAMPEDE Through The Trial Stages For Research Arms B to H

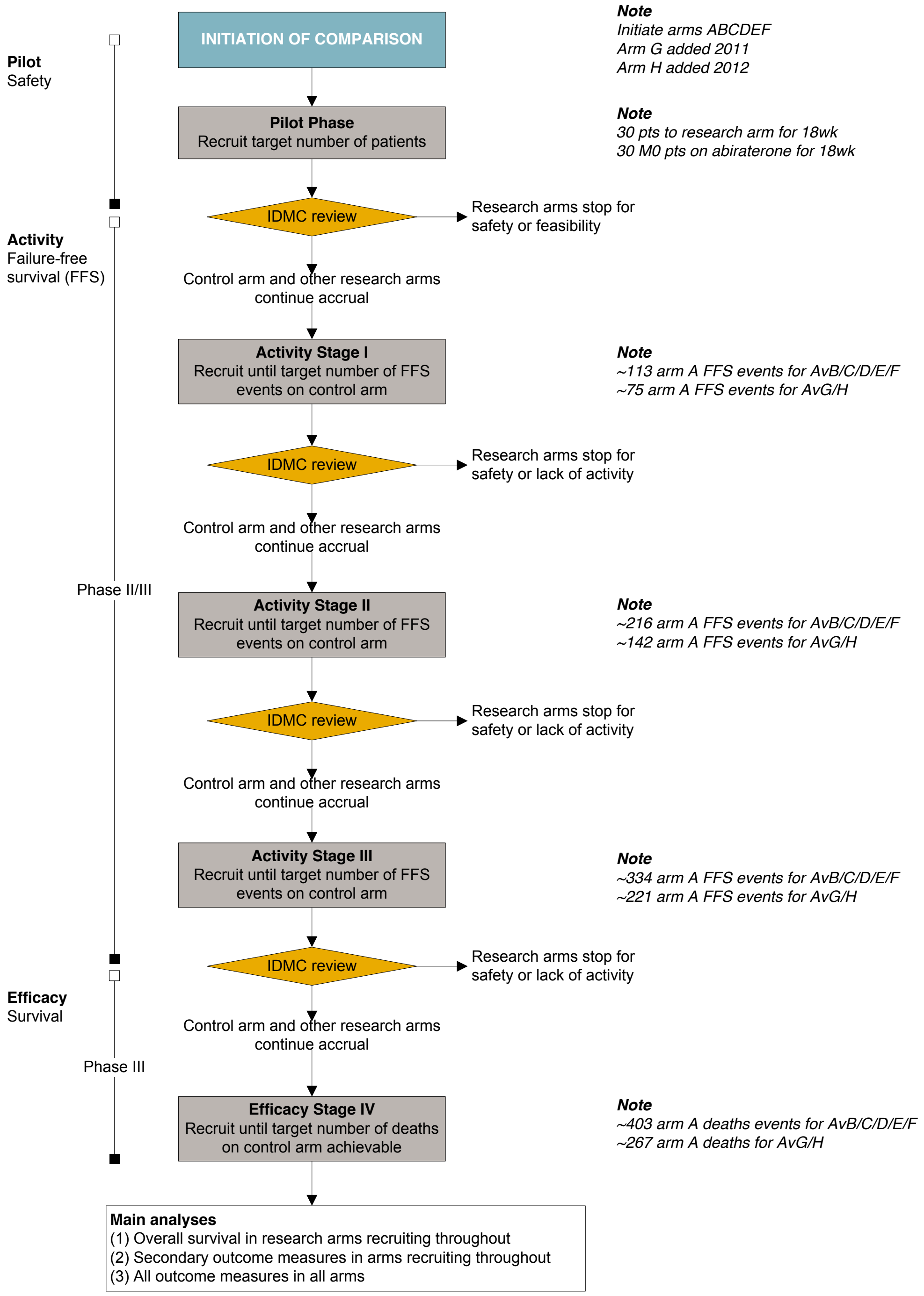

Key

FFS: Failure-free survival
HR: Hazard ratio
IDMC: Independent Data Monitoring Committee
Pts: Patients

Notes

Exact accrual depends on many factors including accrual rate, event rate and arms recruiting in each stage

9.7 FURTHER NOTES ON TRIAL DESIGN

9.7.1 Overall Sample Size

Given the adaptive nature of the study, there is no formal overall sample size target, but the numbers of patients required for each comparison are detailed in **Sections 9.4** and **9.5**. It is expected that more than 5000 patients will likely be recruited overall.

9.7.2 Factorial Design

We note here that we have not employed a factorial design in this trial because we anticipate the possibility of synergy between ADT, zoledronic acid and docetaxel and between ADT, zoledronic acid and celecoxib. It would not be possible to assess any such interactions reliably in a factorial trial (see the Statistical Design Document for further details).

9.8 INTERIM MONITORING AND ANALYSES

The accumulating data will be reviewed at regular intervals (approximately annually) by an Independent Data Monitoring Committee (IDMC), including pre-specified formal intermediate analyses of activity data (see also **Section 16**). These analyses will be performed by the trial team at the MRC CTU. Only patients randomised contemporaneously will be included in the comparison of each research arm against control e.g. patients allocated to the control arm prior to version 8.0 will not contribute to the comparison of abiraterone (Arm A vs Arm G).

The IDMC will be asked to give advice on whether the accumulating data from the trial with the guidelines for discontinuation of accrual for Activity Stages I-III, together with results from any other relevant trials, justifies continuing recruitment of further patients or further follow-up. A decision to discontinue recruitment, in all patients or in selected subgroups will be made only if the result is likely to convince a broad range of clinicians including those entering patients into the trial and the general clinical community. The intermediate stopping guidelines apply to the intermediate primary outcome measure. To stop accrual early for benefit in any comparison would require convincing data in terms of the definitive primary outcome measure, overall survival. For example, this could be $p<0.001$ as proposed by Haybittle-Peto.(47, 48) The use of such a guideline for stopping for benefit has a minimal impact on the operating characteristics.

If a decision is made to continue without change, the IDMC will advise on the frequency of future reviews of the data on the basis of accrual and event rates. The IDMC will make recommendations to the Trial Steering Committee (TSC, see **Section 16**) as to whether the trial should continue in its present form. While the trial is ongoing the accumulating data will generally remain confidential, unless the TSC and IDMC agree that the data should be made public.

9.9 OUTLINE ANALYSIS PLAN

Analyses will be performed on an intention-to-treat basis. The standard unadjusted log-rank approach will be applied to analyses of FFS and OS. The impact of potential confounders including the stratification factors used at randomisation will be considered in a Cox proportional hazard model. Flexible parametric models will be used to calculate the absolute differences between the arms to show treatment differences over time and to estimate restricted mean “survival” times (RMST). The estimated difference in restricted means survival time will be used preferentially to compare treatment arms if the proportional hazards assumptions required for hazard ratios cannot

be supported. The χ^2 test or Mann-Whitney test will be implemented for categorical data comparisons, including toxicity, as appropriate. The primary outcome measures (see **Section 9.2**) will be considered for all arms of the trial at each phase, but the main emphasis will be placed on the comparison of the research arms that have continued to recruit throughout the trial.

9.9.1 Pilot / Safety Phases

The Pilot Phase randomised patients between all the trial arms so that the results from these patients can be included in the main trial. Feasibility is considered in terms of the acceptability of the trial randomisation and reported toxicities and adherence to trial medication. Centres participating in the Pilot Phase for the original research arms were required to keep an anonymised log of all patients assessed for trial eligibility (see protocol version 2.0) so that the number of patients who did not participate in the study and the number of eligible patients who choose to not participate in the study could be summarised (reasons for non-participation were collected where the patients was willing). The anonymised logs will not be needed for new research arms like Arm G introduced in version 8.0 or Arm H introduced in version 9.0.

For the patients who are randomised, we shall describe the incidence of expected and unexpected severe toxicities and adverse events/reactions (see **Section 11**) to decide whether to continue with research arms beyond the Pilot Phase. As indicated above, we do not anticipate that recruitment to the research arms will be discontinued after the Pilot Phase, as there is considerable experience with zoledronic acid and docetaxel when combined with ADT, while Cox-2 inhibitors generally have a good toxicity profile. Although there are limited data on the combinations, we do not expect severe toxicity.

9.9.2 Activity And Efficacy Stages

The approach to analysis of these stages is summarised within the sample size calculations (see earlier subsections of Section **9.4.3**). Each research arm will be compared in a pairwise fashion against the control arm.

Full details are available in the Statistical Analysis Plan.

10 MONITORING AND QUALITY ASSURANCE

10.1 MONITORING AT MRC CTU

Data provided to the MRC CTU will be checked for missing or unusual values (range checks) and consistency over time. If missing or questionable data are identified, staff at the MRC CTU will request that the data be clarified. The exact procedures for data clarification and the amendment of CRFs will be described in the trial specific SOPs and instructions will be sent to all STAMPEDE institutions as soon as they have been approved to participate in the trial. The MRC CTU will also send reminders for any overdue data.

10.2 DIRECT ACCESS TO DATA

Collaborating institutions should be aware that direct access to patient data by MRC CTU staff may be required for trial-related monitoring or audit. Patient consent for this will be obtained as part of the general trial consent process.

10.3 VISITS TO INVESTIGATOR SITES

A selection of institutions will be visited at least once during the course of the STAMPEDE trial. The MRC CTU will give the responsible investigator adequate notice of the monitoring visit to allow adequate time, space and staff for these visits. The standard operating procedures (SOP) for monitoring are available from the MRC CTU.

After the monitoring visit the monitor will complete a site visit report. This report will be circulated to the TMG for comment. Once the TMT have reviewed the report and agreed on any recommendations the monitor will finalise the report and send a copy to the Principal Investigator (PI) at the site. A copy will also be sent to the CI and TMG for the trial and another copy will be kept in the MRC CTU STAMPEDE trial master file.

10.4 CONFIDENTIALITY

All information collected during the course of the research will be kept strictly confidential. In addition, all procedures for handling, processing, storage and destruction of data are compliant with the Data Protection Act 1998. No individual patients will be identified when the results of the trial are published.

Patients will be asked for permission for information about their health status to be obtained from the Office of National Statistics (ONS) or via the NHS Strategic Tracing Service or similar by the Medical Research Council, if necessary. In addition, patients will be asked for permission to inform their GP of their involvement in the STAMPEDE trial.

11 SAFETY REPORTING

ICH GCP requires that both investigators and sponsors follow specific procedures when reporting adverse events/reactions in clinical trials. These procedures are described in this section of the protocol. Further information on the expected toxicities for the trial interventions (docetaxel, zoledronic acid, abiraterone and radiotherapy) can be found in **Appendix G**.

11.1 DEFINITIONS

The safety reporting definitions from ICH GCP apply in this trial protocol. These definitions are given in **Table 12**.

Table 13: Event Terms and Definitions

TERM	DEFINITION
Adverse Event (**AE**)	Any untoward medical occurrence in a patient or clinical trial subject to whom a medicinal product has been administered including occurrences which are not necessarily caused by or related to that product.
Adverse Reaction (**AR**)	Any untoward and unintended response to an investigational medicinal product related to any dose administered.
Unexpected Adverse Reaction (**UAR**)	An adverse reaction, the nature or severity of which is not consistent with the information about the medicinal product in question set out in the summary of product characteristics (or Investigator brochure) for that product.
Serious Adverse Event (**SAE**) or Serious Adverse Reaction (**SAR**) or Suspected Unexpected Serious Adverse Reaction (**SUSAR**)	Respectively any adverse event, adverse reaction or unexpected adverse reaction that:<br>• results in death<br>• is life-threatening*<br>• requires hospitalisation or prolongation of existing hospitalisation**<br>• results in persistent or significant disability or incapacity<br>• consists of a congenital anomaly or birth defect<br>• Other important medical condition***

Clarifications and Exceptions

*The term 'life-threatening' in the definition of 'serious' refers to an event in which the patient was at risk of death at the time of the event; it does not refer to an event which hypothetically might have caused death if it were more severe.

**Hospitalisation is defined as an inpatient admission, regardless of length of stay, even if the hospitalisation is a precautionary measure for continued observation. Hospitalisations for a pre-existing condition (including elective procedures that have not worsened) do not constitute an SAE.

***Medical judgement should be exercised in deciding whether an AE/AR is serious in other situations. Important AE/ARs that are not immediately life-threatening or do not result in death or hospitalisation but may jeopardise the subject or

may require intervention to prevent one of the other outcomes listed in the definition above, should also be considered serious.

Pregnancy occurring in a STAMPEDE patient's partner during the patient's participation in the trial, must be reported to the MRC CTU within the same timelines as an SAE and classified as an 'other important medical condition' on the SAE form. The outcome of a pregnancy should be followed up carefully and any abnormal outcome to the mother or child should be reported.

11.1.1 Trial-Specific Exemptions

Disease progression or death as a result of disease progression are not considered to be SAEs and should be reported on the STAMPEDE Progression Form or Death Form.

The following situations that fulfill the definition of an SAE are excluded from expedited notification on an SAE form and should be reported only on the STAMPEDE follow-up form:

- Elective hospitalisation and surgery for treatment of locally advanced or metastatic prostate cancer or its complications
- Elective hospitalisation to simplify treatment or procedures
- Elective hospitalisation for pre-existing conditions that have not been exacerbated by trial treatment

11.2 INSTITUTION/INVESTIGATOR RESPONSIBILITIES

All non-serious AEs/ARs, whether expected or not, should be recorded in the toxicity (symptoms) section of the Follow-up CRF and sent to the MRC CTU within one month of the form being due. SAEs/SARs should be notified to the MRC CTU as described below.

The severity (i.e. intensity) of all AEs/ARs (serious and non-serious) in this trial should be should be graded using Common Terminology Criteria for Adverse Events (CTCAE) v3.0 (ctep.cancer.gov/reporting/index.html). A flowchart is given in **Appendix I** to help explain the notification procedures. Any questions concerning this process should be directed to the MRC CTU in the first instance.

11.2.1 Investigator Assessment

11.2.1.A Seriousness

When an AE/AR occurs the investigator responsible for the care of the patient must first assess whether the event is serious using the definitions given in **Table 12**. If the event is serious and not exempt from expedited reporting, then an SAE form must be completed and the MRC CTU notified.

11.2.1.B Causality

The Investigator must assess the causality of all serious events/reactions in relation to the trial therapy using the definitions in **Table 13**. There are 5 categories: unrelated, unlikely, possible, probable and definitely related. If the causality assessment is unrelated or unlikely to be related the event is classified as a SAE. If the causality is assessed as either possible, probable or definitely related then the event is classified as a SAR.

Table 14: Assigning type of SAE through causality

RELATIONSHIP	DESCRIPTION	EVENT TYPE
Unrelated	There is no evidence of any causal relationship	Unrelated SAE
Unlikely	There is little evidence to suggest there is a causal relationship (e.g. the event did not occur within a reasonable time after administration of the trial medication). There is another reasonable explanation for the event (e.g. the patient's clinical condition, other concomitant treatment).	Unrelated SAE
Possible	There is some evidence to suggest a causal relationship (e.g. because the event occurs within a reasonable time after administration of the trial medication). However, the influence of other factors may have contributed to the event (e.g. the patient's clinical condition, other concomitant treatments).	SAR
Probable	There is evidence to suggest a causal relationship and the influence of other factors is unlikely.	SAR
Definitely	There is clear evidence to suggest a causal relationship and other possible contributing factors can be ruled out.	SAR

11.2.1.C Expectedness

If the event is a SAR the Investigator must assess the expectedness of the event. Please see Appendix G (Table G.2) for a list of expected toxicities associated with the drugs being used in this trial. If a SAR is assessed as being unexpected it becomes a SUSAR.

11.2.1.D Notification

Investigators must notify the MRC CTU of all SAEs occurring from the time of randomisation until 30 days after the last protocol treatment administration. SAEs occurring in patients randomised to Arm A must be reported until 2 years and 2 months or progression (whichever is sooner). SARs and SUSARs must be notified to the MRC CTU indefinitely (i.e. no matter when they occur after randomisation).

11.2.2 NOTIFICATION PROCEDURE

The SAE form must be completed by the Investigator (consultant named on the signature list and delegation of responsibilities log who is responsible for the patient's care), with due care being paid to the grading, causality and expectedness of the event as outlined above. In the absence of the responsible investigator the form should be completed and signed by a member of the site trial team. The responsible investigator should subsequently check the SAE form, make changes as appropriate, sign and then re-fax to the MRC CTU as soon as possible. The initial report shall be followed by detailed, written reports as appropriate.

Send the SAE form by fax to the MRC CTU. Fax Number: + 44 (0) 20 7670 4818. The STAMPEDE trial team will confirm receipt of the SAE report to the main point of contact via email

Follow-up: Patients must be followed-up until clinical recovery is complete and laboratory results have returned to normal or baseline, or until the event has stabilised. Follow-up should continue after completion of protocol treatment if necessary. Follow-up information should be noted on a further SAE form by ticking the box marked ‘follow-up’ and faxing to the MRC CTU as information becomes available. Extra, annotated information and/or copies of test results may be provided separately. The patient must be identified by trial number, date of birth and initials only. The patient’s name should not be used on any correspondence.

11.3 MRC CTU RESPONSIBILITIES

Medically qualified staff at the MRC CTU and/or the Chief Investigator (or a medically qualified delegate) will review all SAE reports received. The causality assessment given by the local Investigator at the hospital cannot be overruled and in the case of disagreement, both opinions will be provided in any subsequent reports.

The MRC CTU is undertaking the duties of trial sponsor and is responsible for the reporting of SUSARs and other SARs to the regulatory authorities (MHRA and competent authorities of other European member states and any other countries in which the trial is taking place) and the research ethics committees as appropriate.

The MRC CTU will also keep all investigators informed of any safety issues that arise during the course of the trial.

SAE REPORTING

Fax to 020 7670 4818 within 24 hours of becoming aware of the event

12 ETHICAL CONSIDERATIONS AND APPROVAL

12.1 ETHICAL CONSIDERATIONS

This is a randomised trial therefore neither the patients nor their physicians will be able to choose the patients' treatment. Treatment will be allocated randomly using a computer-based algorithm. This is to ensure that the groups of patients receiving each of the different treatments are similar.

Androgen deprivation therapy alone is the standard treatment for these forms of prostate cancer. Patients will be randomised to one or two of the newer treatments in combination with hormone treatment. The trial employs an unequal allocation ratio for efficiency, these are explained in detail in the patient information sheet.

The newer combined treatment options are being assessed in a detailed and systematic fashion in this trial. There is some evidence to suggest that the newer treatment options may have advantages over standard treatment (androgen deprivation therapy) alone with regards clinical outcome, but this is not confirmed and toxicity may be increased. This trial will follow a large group of men who have been randomly allocated to either the standard treatment (androgen deprivation therapy alone) or the newer combined treatment options in order to measure the benefits of the new treatments. The patients will also be followed-up for toxicity and safety issues, so that any benefits can be weighed against any negative aspects.

Patients participating in the trial will have some additional hospital visits and some extra blood samples taken compared to patients who are not participating in the trial, with the amount varying according to the allocated treatment. Sometimes the blood samples can be taken when the patient is attending hospital for treatment, anyway. On some of the trial arms, the patient may have to make additional visits to the hospital for the blood sample to be taken, although in some cases it may be possible for the blood sample to be taken in the GP's surgery. The additional visits and blood samples are to ensure that follow-up of patients is comparable in all the treatment groups. The blood samples will also be used for genetic and serum marker studies, where this information will be considered with clinical data. Blood samples will be link-anonymised. There will be no feedback to individual patients.

If new information emerges during the course of the trial which may affect the treatment or follow-up of patients who have joined the trial, information will be provided through by the trial team to all Principal Investigators. PIs have therefore the duty to inform patients in their care of any new information emerged using any appropriate channel (e.g. letter, communication at follow up clinic, etc).

12.2 ETHICAL APPROVAL

The protocol has a Favourable Opinion from an appropriate Research Ethics Committee, according to national guidelines. Additionally, each site must also obtain management permission for research (R&D approval) from the relevant host organisations before patients can be entered into the trial. The patient's informed consent to participate in the trial should be obtained after a full explanation has been given of the treatment options, including the conventional and generally accepted methods of treatment. Patient information sheets and patient consent forms are given in **Appendix B**.

The right of the patient to refuse to participate in the trial without giving reasons must be respected. After the patient has entered the trial, the clinician must remain free to give alternative treatment to that specified in the protocol, at any stage, if he feels it to be in the best interest of the patient. However, the reason for doing so should be recorded and the patient will remain within the trial for the purpose of follow-up and data analysis according to the treatment option to which he has been allocated. Similarly, the patient must remain free to withdraw at any time from the protocol treatment without giving reasons and without prejudicing his further treatment.

A statement of MRC policy on ethical considerations in clinical trials of cancer therapy, including the question of informed consent, is available from the MRC Head Office web site (http://www.mrc.ac.uk).

13 REGULATORY APPROVAL

This trial has been approved in the UK by the MHRA and will be conducted under a CTA (Ref: 00316/0026/001-0001) in the UK.

The trial has been approved in Switzerland by Swissmedic (Ref: 2009 DR 3235).

14 INDEMNITY

University College London holds insurance against claims from participants for injury caused by their participation in this clinical trial. Participants may be able to claim compensation if they can prove that UCL has been negligent. However, as this clinical trial is being carried out in a hospital, the hospital continues to have a duty of care to the participant of the clinical trial. University College London does not accept liability for any breach in the hospital's duty of care, or any negligence on the part of hospital employees. This applies whether the hospital is an NHS Trust or otherwise.

Participants may also be able to claim compensation for injury caused by participation in this clinical trial without the need to prove negligence on the part of University College London or another party. Participants who sustain injury and wish to make a claim for compensation should do so in writing in the first instance to the Chief Investigator, who will pass the claim to the managing organisation's Insurers, via the managing organisation's office.
Hospitals selected to participate in this clinical trial must provide clinical negligence insurance cover for harm caused by their employees and a copy of the relevant insurance policy or summary can be provided on request.

15 FINANCE

STAMPEDE is funded by the Clinical Trials Advisory Awards Committee (CTAAC) on behalf of Cancer Research UK; it is also funded by the MRC through the MRC Clinical Trials Unit. The trial has National Cancer Research Network (NCRN) approval and, therefore, local NCRN funds may be available at each centre to support entry of patients into this trial.

Zoledronic acid is manufactured by Novartis. Novartis have agreed to provide an educational grant to support the conduct of this study. Novartis have also agreed to supply the study drug, zoledronic acid free of charge for patients participating in the study.

Docetaxel is manufactured by Sanofi-Aventis Pharma. They have agreed to supply the study drug, docetaxel at a discounted rate for patients that are participating in the trial and to provide an educational grant to support the conduct of the study. The Department of Health has agreed to provide a central subvention as follow: £1,787 per patient randomised to arms C and E of the trial and prescribed docetaxel. This amount is payable in respect of a hospital trust randomising more than 3 patients. For more details contact the STAMPEDE Trial Manager.

Celecoxib is manufactured by Pfizer. They agreed to supply free drug and to provide funds to distribute drug to participating sites.

Abiraterone is manufactured by Janssen Pharma PV (pharmaceutical companies of Johnson & Johnson). They have agreed to provide free drug and funds to distribute drug to participating sites and to help support the conduct and management of the trial.

16 TRIAL COMMITTEES

16.1 TRIAL MANAGEMENT GROUP (TMG)

A Trial Management Group (TMG) has been formed comprising the Chief Investigator, other co-investigators and members of the MRC CTU. The membership of the TMG may be expanded if other groups of trialists wish to participate. It will also be amended during the trial if other circumstances require e.g. retirement.

The TMG will be responsible for the day-to-day running and management of the trial and will meet by teleconference at least 3 monthly and in person as needed. The TMG members are detailed in **Appendix K**.

Further details of TMG functioning are provided in the TMG charter (available on request).

16.2 TRIAL STEERING COMMITTEE (TSC)

A Trial Steering Committee (TSC) has been formed to provide overall supervision for the trial and provide advice through its independent chair. The ultimate decision for the continuation of the trial lies with the TSC. The TSC will meet regularly.

Further details of TSC functioning are provided in the TSC charter (available on request)

16.3 INDEPENDENT DATA MONITORING COMMITTEE (IDMC)

An Independent Data Monitoring Committee (IDMC) has been formed. The IDMC will be the only group who sees the confidential, accumulating data to the trial. Reports to the IDMC will be produced by the MRC CTU. The IDMC will meet within 6 months of the trial opening with the frequency of meetings dictated by the IDMC. The IDMC will consider data in accordance with the analysis plan (see **Section 9.5**) and will be advisory to the TSC. The IDMC can recommend premature closure or reporting of the trial, or that recruitment to any research arm be discontinued.

From version 8.0 onwards, any recommendation from the IDMC to stop recruitment to one or more trial arms will be acted upon immediately, pending ratification from the TSC. As this period between meetings should be very short, sites would not be notified until after the TSC have made a decision. IDMC recommendations based on emerging safety issues would be discussed with sites promptly.

Further details of IDMC functioning and the procedures for interim analysis and monitoring are provided in the IDMC charter (available on request).

Figure 11: Diagram of relationships between trial committees

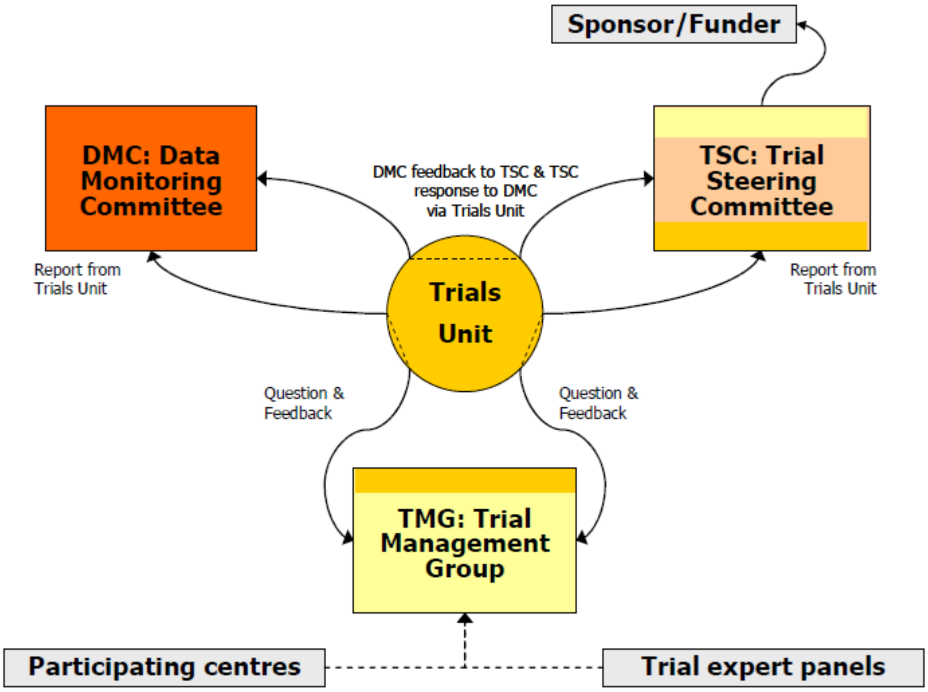

17 ANCILLARY STUDIES

17.1 QUALITY OF LIFE

A quality of life (QL) study is being performed to assess the impact of each treatment arm on the quality of patient's lives and participation in this study was limited to the first 700 patients recruited (this was reached in September 2008) patients. The QL study re-opened from the implementation of version 8.0 of the protocol. The EORTC QLQ-C30 with the prostate-specific module QLQ PR25 will be used. Key items for assessment are pain reduction for patients with metastatic disease and urinary symptoms for patients with locally advanced disease. In addition specific hypotheses will be generated for each of the research arms. The EuroQol (EQ-5D) (49) will be used in the study as a generic measure of health-related quality of life which can be linked to public preferences. These data will be used to calculate quality-adjusted life-years as part of the economic evaluation (see **Section 17.2**). Patients who were recruited into the QL study, should continue on the study throughout the trial. Questionnaires should be self-administered, although it is recommended that a key person (e.g. research nurse) at each centre be responsible for the data collection to optimise compliance and completeness of the data.

The QL and the HE questionnaires should be completed without conferring with friends or relatives and all questions should be answered even if the patient feels them to be irrelevant.

The responsible person should check each questionnaire for its completeness, ensuring that the correct date of completion and patient identifiers are present. The research nurse should approach patients at appropriate clinical visits to complete a questionnaire. If no clinical visit is scheduled for the patient (with a window of 4 weeks around the expected date) the nurse should organise the completion of the questionnaire, by post or by a visit to the patient at home (or in a hospice).

17.2 HEALTH ECONOMICS

A health economics (HE) sub-study will be performed. Core resource use information will be collected, using CRFs on days in hospital (by speciality) and outpatient visits. Data being collected on concomitant medication will also be used in the economic analysis. Information on patients' use of primary care and community-based services will be collected as additional questions in the QL questionnaire. Costs will be calculated on the basis of representative UK unit costs at the point of analysis. Health outcomes will be assessed in terms of quality-adjusted life years (QALYs). Quality adjustments will be based on patients' responses to the EQ-5D health status measure which will be administered at baseline and each point of follow-up as part of the QL questionnaire. A cost-effectiveness analysis will compare all regimens that continue to recruit into their Activity Stage IV.

17.3 TRANSLATIONAL SUB-STUDIES

17.3.1 DNA Analysis

Blood samples from as many patients as possible will be collected for future research. With patient consent, an additional droplet of blood sample will be collected and stored for DNA and protein analysis in order to try to identify molecular features of clinical significance.

Blood samples should be sent directly to the central laboratory on the FTA elute cards provided. Patient information sheets and consent forms which highlight this research are given in **Appendix B**, while details of specimen collection, posting and contact details are given in **Appendix D**.

17.3.2 Tissue Microarray

Patient consent will be sought to utilise paraffin embedded tissue for the construction of tissue microarrays from needle cores. One needle biopsy will be selected for microarray and the remaining tissue will be returned to the originating histopathologist. Given the entry criteria for the trial, the majority of patients will have extensive disease in the diagnostic needle core biopsies, in contrast to men with localised, low grade disease. Consequently, removal of one core is unlikely to compromise any subsequent histopathological assessment. Details regarding transfer of samples will be issued at the time of construction of the micro array. Additional analyses e.g. DNA extraction may also be performed on the tissue arrays.

18 PUBLICATION

The results from different centres will be analysed together and published as soon as possible. Individual clinicians must not publish data concerning their patients that are directly relevant to questions posed by the study until the TMG has published its report. The TMG together with the STAMPEDE collaborators will form the basis of the writing committee and decide on the nature of publications. Any release, of efficacy or safety data, presentation or publication will be agreed with the TSC according to the terms of their charter.

All publications will acknowledge the participating centres and clinicians, and these will be detailed in an appendix to the main report. Papers will have named authors determined by the TMG according to the following principles:

- To be as inclusive as possible where this is practicable
- To ensure that there is justification for anyone to be named as an author
- Reasons for nomination for authorship may include: trial design; grant holding; day-to-day trial oversight (TMG membership); analysis; discussion and interpretation of data; representation for key groups; active participation at large recruiting sites. It should be accepted that the people qualifying for authorship will vary over time. In addition, key positions will vary depending on the nature of the publication: clinical lead for clinical papers, statistician lead for methodology papers, translational papers may be lead by authors not on the main TMG if appropriate (e.g., the bone sub-study). In the event of any dispute related to authorship or data release, the TSC will be responsible for making the executive decision.

In the manuscript, a full list of sites and the number of patients recruited will be provided. In the presentations, this list of sites will also be shown. The term “the STAMPEDE investigators” will clearly be stated and relevant names included in the presentation credits.

A detailed Publication Plan is documented elsewhere.

19 PROTOCOL AMENDMENTS

19.1 PROTOCOL

19.1.1 Amendments Made To Sections In Protocol Version 1.0 (May 2004)

Administrative changes such as typos, word change etc.

Name additions/changes to:

TMG members

TSC members

IDMC members

'General Information' Section – additional information re. Abridged version of protocol

Section 1.2 – Figure 1, Celecoxib duration amended

Section 1.3 – Figure 2, addition of cardiovascular assessment form, name and timings amended

Section 2.3 – Docetaxel information updated

Section 2.4 – Additional text re dose and duration justification for Celecoxib use.

Section 3 – Title change and content updated

Section 4.2 – New exclusion criteria added

Section 4.3.1 – New investigations added and additional text re testosterone measurements and additional text re. prior celecoxib treatment

Section 6.1.4 – Celecoxib duration amended

Section 6.1.5 – Additional text re. Co-administration of docetaxel and bisphosphonates

Section 6.1.6 – Celecoxib duration amended

Section 6.2.2 – additional docetaxel information

Section 6.2.3 – addition of CV event history

Section 11 – Safety reporting updated

Section 12.1 – Additional text re. the collection of blood for genetic and serum marker studies

Section 15 – Additional information re. Central Subvention for docetaxel arms

19.1.2 Amendments Made To Sections In Protocol Version 1.1 (May 2005)

Section 6.2 Administration and Dose Modifications, subsection 6.2.1 Zoledronic Acid

19.1.3 Amendments Made To Sections In Protocol Version 2.0 (Jun 2005)

General Information section – SAE reporting fax number and timeframe added.

Section 1.2 – Addition of anti-androgen use for M0 patients as a method of HT

Section 1.2 – Increase in amount of blood needed & addition tissue sample request.

Section 1.3 Trial Documentation updated to include new table detailing trial documentation ahead of accreditation, the inclusion of the radiotherapy forms and correct case report form timings

Section 2.1 – Addition of anti-androgen use for M0 patients as a method of HT

Section 4.1.3 – Inclusion criteria Vii "Normal testosterone prior to hormone treatment" removed.

Section 4.1.3 - φnote has been omitted and moved to section 4.2 (see number 8)

Section 4.2 – Exclusion criteria added to exclude patients with active peptic ulceration, gastrointestinal bleeding and inflammatory bowel disease.

Section 4.2 – Exclusion Criteria added to exclude patients with planned major dental work

Section 4.3.1 - All blood test timelines changed from 14 days to 28 days.

Section 4.3.1 – Hormone Therapy pre-randomisation deadline extended from 4 weeks to 12 weeks.

Section 4.3.1 – Additional information regarding the use of NSAIDs and cox-2-inhibitors before coming on to the STAMPEDE study and once commenced on study treatment

Section 4.3.2 – Updated to ask for all vitamins and minerals the patient is taking to be recorded.

Section 4.3.3 – Updated to include the extra blood required and the request for consent of patients' tissue samples.

Section 6.1.1 – Addition of anti-androgen use for M0 patients as a method of HT

Section 6.1.6 – Addition of the calcium & vitamin name "calcichew".

Section 6.6.2 – asking also to collect vitamins and minerals under concomitant medication.

Section 6.6.3 – New section to inform investigators that patient's, who they wish to give radiotherapy to, are also eligible for STAMPEDE

Section 6.6.4 – New section to detail what data is being collected on the radiotherapy given to patients.

Section 7.1; figure 4 – Addition of radiotherapy form and in note, addition of AA alone

Section 7.1.2 – omission of repeated scans and x-rays at 24 weeks, also omitted in note under figure 4.

Chapter 11 – Safety reporting section updated

Section 17.3 – Increase in amount of blood needed & additional tissue sample request.

19.1.4 Amendments Made To Section In Protocol Version 3.0 (Jul 2006)

Front Cover - NCRN logo added for accuracy

Front Cover - Clafication that protocol developed with NCRI rather than on behalf of

Front Cover - Clarification the it is a 6 arm trial

General Information section - MRC CTU staff section updated

Section 1.2 – Statistics section updated.

Section 1.2 - Additional research paragragh updated to refelct additional studies and for clarification of terms

Section 1.2 - Blood collection volume changed to reflect new technique used

Section 1.3 (figure 3) - Table showing case report form scheduyle updated to reflect clarification of follow-up schedule and addition of new CRF (End of Treatment)

Section 2.2 - AS changed to HT (clarification of terms)

Section 2.3 - Updated in information in reagrd to use of docetaxel added to reflect up to date practice

Section 2.5 - Sub-headings numbered for consistancy

Section 3.0 - Information in reagrd to the Pilot Phase now written in past tense as Pilot Phase has now been completed

Section 4.1.1 - Inclusion criteria extended so that patinets who fulfil 2 out of the three of the first inclusion criteria can be eligible.

Section 4.3.1 - Change in time scales by which baseline investigations need to be completed.

Section 4.3.1 - Clarification that chest X-ray is only required if chest is not included in the CT

Section 4.3.1 - Removal of 12 week timeline for basline PSA test ot be performed. (Stipulation that it must be performed before start of HT)

Section 4.3.2 – Information added in regard to time allowed from randomisation to start of treatment

Section 4.3.3 - Additional research paragragh updated to refelct additional studies and for clarification of terms

Section 4.3.3 - Blood collection volume changed to reflect new technique used

Sections 6.1.2-6.1.6 - Androgen Supression replaced with hormone therapy for consistemcy of terms

Section 6.2.2 - '(Taxotere)' Removed for consistancy

Section 6.2.2 _ information added in regard to the need to closely monitor liver function prior to docetaxel adminstration

Section 7.1 - Page number reference updated

Section 7.1.1 - PSA measurement timings updated to accurately reflect follow-up schedule

Section 7.3 (Table 4) - Table and key updated to accurately refelct follow-up schedule and to include information about new CRFs and removal of withdrawal CRF

Section 8 - Rewording for clarifcation of definition of trial withdrawal

Section 8.1 - Instruction that withdrawal from trial treatment should be recorded on End of Treatment Form rather than withdrawal form

Section 8.1 - Information updated to emphasise that trial treatment must be discontinued following a progression

Section 8.2- Information added in regard to patient transfers

Section 8.3 - Instruction that withdrawal from trial completely must be notified in writing to the MRC CTU rather than included on withdrawal form

Section 9 and Summary – Target event numbers updated to reflect the slightly revised numbers obtained by using –nstage- which is the new, recommended program for MAMS trials

Sections 11.1 and 11.2 - Form numbers removed to allow for future changes in numbering

Section 11.2 – Reference to toxicity grading website added

Section 11.2.1 - Reference to table in appendix G added

Section 12.2 - 'Suggested' removed from 'Suggested patient information sheets'

Section 13 - CTA reference added

Section 17.3 - Information added to reflect new blood collection method for DNA analysis and in regard to additional translational studies for which funding has recently been approved

19.1.5 Amendments Made To Protocol Version 4.0 (Dec 2007)

General Information Section - Randomisation and SAE reporting details sections clarified

Section 1.2 and throughout protocol - Efficacy Stages 1-111 renamed to Activity Stages 1-111 for accuracy and clarity

Section 1.2 - Follow schedule corrected

Section 4.1.2 - Inclusion criteria widened to include high risk relapsing patients, that would not have met the previous PSA based criteria

Section 4.1.3 - Note added to reference location of WHO performance status definitions

Section 4.2 - Notes added to reference locations of toxicity gradings and NYHA classifications

Section 4.3.1 - Timings of baseline scan information changed to accurately reflect most common current practice

Section 6.1.1 - Information about use of LHRH antagonists to ensure that the protocol accurately reflects current and future practice

Section 6.1.1 - Information about suggested duration of hormone therapy added to ensure that the protocol accurately reflects current practice

Section 6.2.2 - Additional information added about the timing of liver function tests prior to docetaxel administration added for clarity

Section 6.6.4 - Information on radiotherapy data collection added

Section 7.1.1 - Erroneous information about the timing of PSA measurements removed

Figure 3 - Moved to new section in protocol for clarity and extended to include current information on data collection

Figure 3b - Added to describe how extent of data collection during follow-up should change, post treatment and post progression

Figure 4 - Notes added to explain the changes in data collected at follow-up and to information that the quality of life study will be applicable to the first 700 patients randomised only

Figure 4 - Note added to include palliative radiotherapy CRF

Section 11.3 - SAE reporting information updated

Section 19 - Protocol amendments list updated

19.1.6 Amendments Made To Protocol Version 5.0 (Aug 2008)

1. General Information Section – Randomisation phone line number updated – non UK extension added
2. Section 3 – Information about QL study removed to reflect closure of QL study after first 700 patients
3. Section 4.2 – Exclusion criteria clarified to explain that only patients with severe poor cardiovascular history should be excluded
4. Section 4.3.1 – Information on co-administration of NSAIDS with celecoxib changed based on clinical advice.

5. Section 5 - Randomisation phone line number updated – non UK extension added

6. Section 6.2.1. – Information added to clarify that patients who develop an osteonecrosis of the jaw should stop zoledronic acid treatment

7. Section 6.2.3 – 'severe' text added to acurately reflect which patients should be excluded based on their cardiovascular history

8. Section 7.1.2 – Defination of disease progression extended for clarity

9. Figure 3 – Updated to include reference to newly created skeltal related event form

10. Figure 4 – Previous error in table amended to show that the 4th Zoledronic Acid form that is submitted contains information about 3 cycles rather than 2 as previously indicated

11. Table 4 – 'Other important medical condition' added to definition of serious in the SAE section, to accuratly reflect SAE form and current practice

12. Section 11.1 – Information added on reporting or pregnancies

13. Section 17 - Information about QL study removed to reflect closure of QL study after first 700 patients

19.1.7 Amendments Made To Protocol Version 6.0 (Jul 2009)

1. General Information Section – Trial Pharmacist removed and changes of:

Co-Investigator

Patient Representatives

Trial Manager

Data Manager

General Information Section - Coordinating Centre – address change

General Information Section – change of Sponsor address

Section 1.1 – ratio of patients randomised to the investigational arms updated

Section 1.2 – figure 1b added to clarify trial design from Apr-2011 onwards

Section 1.2 – paragraph added to explain trial changes after the second activity analysis

Section 1.2 – wording added to clarify that QL data only collected for first 700 patients randomised

Section 1.3 – SSA Favourable Opinion removed from list of trial documentation required ahead of site accreditation

Section 2.1 – Amount of men diagnosed with prostate cancer annually updated

Section 2.4 –note added to explain completion of recruitment to celecoxib- containing arms

Section 2.5.2 - note added to explain completion of recruitment to celecoxib- containing arms

Section 3 – SSA Favourable Opinion removed

Section 4.2 – Exclusion criterion xiii greyed out

Section 4.3.1 – paragraph removed regarding potential randomisation to celecoxib-containing arms

Section 5 – Randomisation instructions expanded to exclude public holidays or dates when notice has been given by the CTU

Section 6.1.4 – formatting changed to grey font to reflect recruitment completion for arm D

Section 6.1.6 - formatting changed to grey font to reflect recruitment completion for arm F

Section 6.2.3 – recruitment note added

Section 6.6.3 – radiotherapy statement changed to reflect data from recent trials

Section 7.1.2 – removal of reference to SRE- specific CRF

Section 7.3 – Figure 3 - Addition of Bone Density Risk Factor Form and BMD sub-study assessment forms to summary of timing table

Section 7.3 – Figure 4 – Weeks added to timings of assessments post 2 years

Section 7.3- Figure 4 – note added to explain recruitment completion for arms D and F

Section 12.1 – Wording changed to reflect change to randomisation allocation ratio

Section 12.1 – Addition of statement regarding new information emerging during the trial

Section 12.2 – Reference to SSA removed

Section 16.3 – Statement added regarding actioning IDMC recommendation ahead of TSC ratification

19.1.8 Amendments Made To Protocol Version 7.0 (Jul 2011)

1. General Information Section- SAE reporting fax number corrected

2. Section 11- SAE reporting fax number corrected

19.1.9 Amendments Made To Protocol Version 7.1 (Jul 2011)

Throughout protocol – numbering has been updated in some sections new accommodate new information that has been added.

General Information Section – contact details updated

General Information Section – Funding information updated to include involvement from additional company

General Information Section – Wording on compliance and regulations updated to reflect current MRC CTU standard wording

General Information Section – Abbreviations list updated

Section 1.1 – The number of investigational agents being studied updated from three to four

Section 1.1 – Information regarding celecoxib updated to reflect that recruitment to these arms was discontinued in Apr-2011

Section 1.1 – Information about new IMP, Abiraterone inserted

Section 1.1 – Sample size and trial duration information updated to reflect changes brought about by additional trial arm

Section 1.2 – Summary information updated to reflect the discontinuation of recruitment to celecoxib arms and the addition of abiraterone

Figures 1a, b and c - Updated to reflect the discontinuation of recruitment to celecoxib arms and the addition of abiraterone

Section 1.2 – Information on trial stages updated to reflect changes brought about by additional trial arm

Section 1.2 – Information updated regarding the re-opening of the quality of life sub-study from implementation of protocol version 8.0

Section 2.1 – Wording related to hormone therapy updated for clarity

Section 2.1 - Updated to reflect the discontinuation of recruitment to celecoxib arms and the addition of abiraterone

Section 2.2 – Updated references added

Section 2.3 – Updated references added

Section 2.5 – Section added to give background information on new IMP, abiraterone

Section 2.6.1 – Updated references added

Section 2.7 – Section added to give information regarding radiotherapy which is to be given as part of standard care following recently published trial data.

Section 3 – Wording updated regarding selection of investigators to reflect current MRC CTU practice

Section 4.1 – Inclusion criteria updated with new criterion regarding radiotherapy use

Section 4.1 - Inclusion criteria updated with new criterion regarding contraceptive use

Section 4.1 – Wording of inclusion and exclusion criteria updated for clarity

Section 4.1 – Exclusion criteria updated with new criterion regarding acceptable liver function for trial entry

Section 4.1 – Exclusion criteria updated with specifics related to blood pressure levels

Section 4.1 - Exclusion criteria updated with new criterion regarding concomitant medications

Section 4.1 - Exclusion criteria updated with new criterion regarding prior treatment with abiraterone

Section 4.1 - Exclusion criteria updated with new criterion regarding prior treatment with chemotherapy

Section 4.1 - Exclusion criteria updated with new criterion regarding prior treatment with zoledronic acid

Section 4.3 – Wording updated to reflect that patients who initially fail screening can be re-screened at a later date

Section 4.3.2 – Wording updated regarding prior anti-androgen and LHRH use updated for clarity

Section 5.1 – Co-enrolment guidelines information updated to describe newly created co-enrolment CRF

Section 6.1 – Trial treatment information updated to reflect the fact that anti-androgens alone will be no longer permitted as hormone therapy

Section 6.1.1 – Updated to describe patients for whom radiotherapy should be given as standard practice

Section 6.1.1 a and b - Sections added to give information regarding radiotherapy treatment

Section 6.1.1-6.1.6 – References to further sections updated

Section 6.1.7 – Section added to describe abiraterone treatment

Section 6.2.4 - Section added to describe abiraterone treatment

Section 6.6 - Section added to give information regarding radiotherapy treatment

Section 7.1.1 – Reference to blood being taken at patient's home removed as this does not occur in practice

Section 7.1.2 – Wording updated regarding the reporting of biochemical failures for clarity

Section 7.1.2 – Wording updated regarding skeletal-related events for clarity

Section 7.1.3 – Section added to describe additional assessments required related to abiraterone treatment

Section 7.1.4 – Section added to provide information on when treatment should commence

Figure 4 – Updated for clarity regarding return of BMD sub-study forms, the addition the co-enrolment CRF and the description of the re-opening of the QoL Sub-study.

Figure 5 – Updated with reference to abiraterone and co-enrolment form

Section 7.3 - Wording on trial closure updated to reflect current MRC CTU standard wording

Section 8.1 – Additional criteria for definition of progression added for clarity

Section 8.1 – Definition of progression for abiraterone patients added.

Section 9 – Statistical information updated to describe the addition of the new trial arm

Section 11 – Safety reporting wording updated for clarity

Section 11 – SAE reporting fax number updated

Section 12 – Ethical information updated to describe the unequal randomisation allocation ratio

Section 12 – Ethical information updated to describe that the visit schedule will vary according to trial arm

Section 12.2 – Wording updated to reflect international participation in the trial

Section 13 – Wording updated to reflect international participation in the trial

Section 14 – Wording updated to reflect international participation in the trial

Section 15 - Updated to reflect the discontinuation of recruitment to celecoxib arms and the addition of abiraterone

Section 16 – Reference to trial committee charters added for information

Section 17.1 – Information added to reflect re-opening of quality of life sub-study

Section 17.2 – Timing of health economics analysis updated to previous error

Section 18 – Information on publication policy expanded for clarity

Section 19 – Information regarding amendments to protocol appendices moved to the separate appendices document

Section 20 – References extensively updated

19.1.10 Amendments Made To Protocol Version 8.0 (Sep 2011)

Throughout protocol – numbering, sections headings, tables, figures and bibliographical references have been updated in some sections to accommodate new information that has been added

Throughout protocol – Androgen Deprivation Therapy has replaced Hormone Therapy as deemed more representative of the type of hormone therapy used in the study

General Information Section – New staff members of the MRC CTU and Co-Investigators added and contact details updated

General Information Section – Abbreviations list updated

Section 1.1 – Information regarding the new research radiotherapy treatment inserted

Section 1.1 – Information regarding docetaxel updated

Section 1.2 – Wording updated to reflect the addition of the new research comparison arm

Section 1.3 – Additional criteria for the re-accreditation of participating centres (for protocol version 9.0 only)

Section 2.1.1 – Wording updated to clarify the use of anti-androgen in trial patients

Section 2.1.2 – Information added to describe the rationale for the RT comparison arm

Section 2.8 – Information added to describe research RT treatment to prostate for patients with newly diagnosed metastatic disease

Section 3.1 – Information added to describe RT Quality Assurance procedures and centre accreditation

Section 4.1.1 to 4.1.3 – Wording updated to clarify inclusion criteria for all patients groups (newly diagnosed non-metastatic, metastatic and relapsing patients)

Section 4.2 – Clarification added on cardiovascular exclusion criteria

Section 4.2 – New exclusion criterion added concerning patients with prior exposure to hormone therapy

Section 4.2 – New exclusion criterion added to reflect the addition of the new RT comparison arm

Section 4.4.1 – Clarification added regarding pre-randomisation checks

Section 4.4.2 – Clarification added regarding permissible hormone therapy duration prior to randomisation

Section 4.4.5 – Information added regarding starting research radiotherapy treatment

Section 4.4.6 – Information updated on concomitant medications

Section 5 – Clarification regarding randomisation allocation added to reflect the addition of the new RT research arm

Section 6.1.8 – Information added to describe the administration of research radiotherapy

Section 6.2.1 – Clarification added regarding the measurement of serum creatinine levels prior to the administration of zoledronic acid

Section 6.2.3 – Clarification regarding the completion of recruitment to the celecoxib containing arms

Section 6.25 – Information added regarding the administration of research radiotherapy treatment

Section 6.6 – Clarification incorporated to describe the administration of standard-of-care radiotherapy

Section 7.1.4 – Information added regarding data collection and non administration of standard radiotherapy

Section 7.2 – Section updated to include new treatment specific CRFs and timing of CRFs

Section 8.1 – Clarification added for the criteria to stop treatment for patients randomised to arm G

Section 8.2 – Section expanded to include additional details on study patient transfer to different centres

Section 8.3 – Additional sentence inserted to reinforce the importance of compliance with follow up assessments

Section 9.1 – Additional paragraph inserted to clarify the method of randomisation and allocation distribution in the light of the introduction of the new RT arm

Section 9.4 – Wording updated to clarify the assessment of safety data

Section 9.5.4 – Wording updated concerning the end of randomisations to arm G

Section 9.6 to 9.6.4 – Section added describing sample size issues and trial stages for arm H

Section 9.8 – Clarification on intermediate stopping guidelines

Section 9.9 – Clarification on the outline analysis plan

Section 11 – Information on safety reporting updated to reflect the addition of the research RT comparison arm

Section 11 – Clarification added regarding arm A safety reporting timelines

Section 12.1 – Clarification added regarding the Principal Investigator's responsibilities

Section 14 – Indemnity section updated to reflect current MRC policy

Section 16 – Clarification regarding TMG membership

Section 17.3 – Section on Bone Mineral Density sub-study removed

Section 19 – Information regarding amendments to protocol appendices moved to the separate appendices document

Section 20 – References updated

19.1.11 Amendments Made To Protocol Version 9.0 (Oct-2012)

Throughout protocol – numbering, sections headings, tables, figures and bibliographical references have been updated in some sections to accommodate the completion of recruitment to original research arms B, C and E.

Throughout protocol – Tenses have been changed to reflect activities that were in the future and which have now been passed.

Section 1 – Figure added and clarifications added to each figure

Section 2 – Previous reference 8 removed

Section 4 – Clarification of acceptable alternatives to bone scans

Section 6.2.5 – Correction of an error defining the PTV: the wording has been reordered

Table 4 – Dose-volume objectives corrected: order swapped

Table 5- Correction CRFs names

Section 17.3.2 – Clarification that DNA may be extracted

19.1.12 Amendments Made To Protocol Version 10.0 (Apr-2013)

Throughout protocol – numbering, sections headings, tables, figures and bibliographical references have been updated in some sections

Throughout protocol – typos have been corrected

Section 4 –Clarification of exclusion criteria V (now V and VI)

Section 6 – Timing of orchidectomy prior to randomisation extended to 12 weeks

Section 6 – Clarification of hypokalaemia, blood pressure and fluid retention management

Section 9 – Statistical considerations amended in light of the recruitment extension for the abiraterone comparison

Section 14 - Section updated to reflect the changes in the structure of the MRC CTU (now MRC CTU at UCL) and indemnity arrangements

20 REFERENCES

1. James ND, Sydes MR, Mason MD, Clarke NW, Anderson J, Dearnaley DP, et al. Celecoxib plus hormone therapy versus hormone therapy alone for hormone-sensitive prostate cancer: first results from the STAMPEDE multiarm, multistage, randomised controlled trial. The lancet oncology. 2012;13(5):549-58. Epub 26-Mar-2012.

2. Cancer Research UK. CancerStats Key Facts: Prostate Cancer. Cancer Research UK. 2011.

3. Sharifi N, Gulley JL, Dahut WL, Sharifi N, Gulley JL, Dahut WL. An update on androgen deprivation therapy for prostate cancer. Endocrine-Related Cancer. 2010;17(4):R305-15.

4. Widmark A, Klepp O, Solberg A, Damber JE, Angelsen A, Fransson P, et al. Endocrine treatment, with or without radiotherapy, in locally advanced prostate cancer (SPCG-7/SFUO-3): an open randomised phase III trial. Lancet. 2009;373(9660):301-8. Epub 2008/12/19.

5. Warde PR, Mason MD, Sydes MR, Gospodarowicz MK, Swanson GP, Kirkbride P, et al. Intergroup randomized phase III study of androgen deprivation therapy (ADT) plus radiation therapy (RT) in locally advanced prostate cancer (CaP) (NCIC-CTG, SWOG, MRC-UK, INT: T94-0110; NCT00002633). J Clin Oncol. 2010;28(18s Supplement: Proceedings of ASCO 2010):Abstr CRA4504.

6. Warde P, Mason M, Ding K, Kirkbride P, Brundage M, Cowan R, et al. Survival Benefit with Combined Androgen Deprivation and Radiation Therapy in Locally Advanced Prostate Cancer – Results of a Phase III Trial. The Lancet. 2011 - in press.

7. Mason M, Sydes M, Parulekar W, Parmar M, Anderson J, Barber J, et al. Final analysis of intergroup randomized phase III study of androgen deprivation therapy (ADT) + radiation therapy (RT) in locally advanced prostate cancer (CaP) (NCIC-CTG, SWOG, MRC-UK, INT: T94-0110). National Cancer Research Institute (NCRI) Cancer Conference 2012. 2012;2012.

8. Saad F, Colombel M. Management of castration-resistant prostate cancer: bisphosphonates and emerging therapies. Expert Review of Anticancer Therapy. 2010;10(12):1991-2002.

9. Sartor O, Halstead M, Katz L, Sartor O, Halstead M, Katz L. Improving outcomes with recent advances in chemotherapy for castrate-resistant prostate cancer. Clinical Genitourinary Cancer. 2011;8(1):23-8.

10. Attard G, Richards J, de Bono JS, Attard G, Richards J, de Bono JS. New strategies in metastatic prostate cancer: targeting the androgen receptor signaling pathway. Clinical Cancer Research. 2011;17(7):1649-57.

11. Lipton A. Improving progression-free and overall survival in patients with cancer: a potential role for bisphosphonates. Expert Opinion on Pharmacotherapy. 2011;12(5):749-62.

12. Lipton A, Small E, Saad F, Gleason D, Gordon D, Smith M, et al. The new bisphosphonate, Zometa (zoledronic acid), decreases skeletal complications in both osteolytic and osteoblastic lesions: a comparison to pamidronate. [Review] [55 refs]. Cancer Investigation. 2002;20 Suppl 2:45-54.

13. Green JR, Muller K, Jaeggi KA. Preclinical pharmacology of CGP 42'446, a new, potent, heterocyclic bisphosphonate compound. Journal of Bone & Mineral Research. 1994;9(5):745-51.

14. Major P, Lortholary A, Hon J, Abdi E, Mills G, Menssen HD, et al. Zoledronic acid is superior to pamidronate in the treatment of hypercalcemia of malignancy: a pooled analysis of two randomized, controlled clinical trials. J Clin Oncol. 2001;19(2):558-67. Epub 2001/02/24.

15. Costa L, Harper P, Coleman RE, Lipton A, Costa L, Harper P, et al. Anticancer evidence for zoledronic acid across the cancer continuum. Critical Reviews in Oncology-Hematology. 2011;77 Suppl 1:S31-7.

16. Rosen LS, Gordon D, Kaminski M, Howell A, Belch A, Mackey J, et al. Long-term efficacy and safety of zoledronic acid compared with pamidronate disodium in the treatment of skeletal complications in patients with advanced multiple myeloma or breast carcinoma: a randomized, double-blind, multicenter, comparative trial. Cancer. 2003;98(8):1735-44. Epub 2003/10/10.

17. Rosen LS, Gordon DH, Dugan W, Jr., Major P, Eisenberg PD, Provencher L, et al. Zoledronic acid is superior to pamidronate for the treatment of bone metastases in breast carcinoma patients with at least one osteolytic lesion. Cancer. 2004;100(1):36-43. Epub 2003/12/24.

18. Saad F, Gleason D, Murray R, Tchekmedyian S, Venner P, Lacombe L, et al. A Randomized, Placebo-Controlled Trial of Zoledronic Acid in Patients With Hormone-Refractory Metastatic Prostate Carcinoma Journal of the National Cancer Institute. 2002;94(19).

19. Dearnaley DP, Sydes MR, Mason MD, Stott M, Powell CS, Robinson AC, et al. A double-blind, placebo-controlled, randomized trial of oral sodium clodronate for metastatic prostate cancer (MRC PR05 Trial). J NatlCancer Inst. 2003;95(17):1300-11.

20. Petrylak DP, Tangen CM, Hussain MH, Lara PN, Jr., Jones JA, Taplin ME, et al. Docetaxel and estramustine compared with mitoxantrone and prednisone for advanced refractory prostate cancer. N Engl J Med. 2004;351(15):1513-20. Epub 2004/10/08.

21. Tannock IF, de Wit R, Berry WR, Horti J, Pluzanska A, Chi KN, et al. Docetaxel plus prednisone or mitoxantrone plus prednisone for advanced prostate cancer. N Engl J Med. 2004;351(15):1502-12. Epub 2004/10/08.

22. Hawkey CJ. COX-2 inhibitors.[comment]. Lancet. 1999;353(9149):307-14.

23. Taketo MM. Cyclooxygenase-2 inhibitors in tumorigenesis (Part II). Journal of the National Cancer Institute. 1998;90(21):1609-20.

24. Taketo MM. Cyclooxygenase-2 inhibitors in tumorigenesis (Part I). Journal of the National Cancer Institute. 1998;90(20):1529-36.

25. Nelson JE, Harris RE. Inverse association of prostate cancer and non-steroidal anti-inflammatory drugs (NSAIDs): results of a case-control study. Oncology Reports. 2000;7(1):169-70.

26. Eberhart CE, Coffey RJ, Radhika A, Giardiello FM, Ferrenbach S, DuBois RN. Up-regulation of cyclooxygenase 2 gene expression in human colorectal adenomas and adenocarcinomas. Gastroenterology. 1994;107(4):1183-8.

27. Roberts RO, Jacobson DJ, Girman CJ, Rhodes T, Lieber MM, Jacobsen SJ. A population-based study of daily nonsteroidal anti-inflammatory drug use and prostate cancer. [see comments.]. Mayo Clinic Proceedings. 2002;77(3):219-25.

28. Basler JW, Piazza GA, Basler JW, Piazza GA. Nonsteroidal anti-inflammatory drugs and cyclooxygenase-2 selective inhibitors for prostate cancer chemoprevention. Journal of Urology. 2004;171(2 Pt 2):S59-62; discussion S-3.

29. Masferrer JL, Leahy KM, Koki AT, Zweifel BS, Settle SL, Woerner BM, et al. Antiangiogenic and antitumor activities of cyclooxygenase-2 inhibitors. Cancer Research. 2000;60(5):1306-11.

30. Steinbach G, Lynch PM, Phillips RK, Wallace MH, Hawk E, Gordon GB, et al. The effect of celecoxib, a cyclooxygenase-2 inhibitor, in familial adenomatous polyposis. New England Journal of Medicine. 2000;342(26):1946-52.

31. Solomon SD, McMurray JJ, Pfeffer MA, Wittes J, Fowler R, Finn P, et al. Cardiovascular risk associated with celecoxib in a clinical trial for colorectal adenoma prevention. New England Journal of Medicine. 2005;352(11):1071-80.

32. Danila DC, Morris MJ, de Bono JS, Ryan CJ, Denmeade SR, Smith MR, et al. Phase II multicenter study of abiraterone acetate plus prednisone therapy in patients with docetaxel-treated castration-resistant prostate cancer. J Clin Oncol. 2010;28(9):1496-501. Epub 2010/02/18.

33. Reid AH, Attard G, Danila DC, Oommen NB, Olmos D, Fong PC, et al. Significant and sustained antitumor activity in post-docetaxel, castration-resistant prostate cancer with the CYP17 inhibitor abiraterone acetate. J Clin Oncol. 2010;28(9):1489-95. Epub 2010/02/18.

34. Ryan CJ, Smith MR, Fong L, Rosenberg JE, Kantoff P, Raynaud F, et al. Phase I clinical trial of the CYP17 inhibitor abiraterone acetate demonstrating clinical activity in patients with castration-resistant prostate cancer who received prior ketoconazole therapy. Journal of Clinical Oncology. 2010;28(9):1481-8.

35. de Bono JS, Logothetis CJ, Molina A, Fizazi K, North S, Chu L, et al. Abiraterone and increased survival in metastatic prostate cancer. New England Journal of Medicine. 2011;364(21):1995-2005.

36. Ryan CJ, Smith MR, de Bono JS, Molina A, Logothetis CJ, de Souza P, et al. Abiraterone in metastatic prostate cancer without previous chemotherapy. N Engl J Med. 2013;368(2):138-48. Epub 2012/12/12.

37. Attard G, Reid AHM, de Bono JS. Abiraterone acetate is well tolerated without concomitant use of corticosteroids. Journal of Clinical Oncology. 2010;28(29):e560-1; author reply e2.

38. Flanigan RC, Salmon SE, Blumenstein BA, Bearman SI, Roy V, McGrath PC, et al. Nephrectomy followed by interferon alfa-2b compared with interferon alfa-2b alone for metastatic renal-cell cancer. N Engl J Med. 2001;345(23):1655-9. Epub 2002/01/05.

39. Mickisch GH, Garin A, van Poppel H, de Prijck L, Sylvester R. Radical nephrectomy plus interferon-alfa-based immunotherapy compared with interferon alfa alone in metastatic renal-cell carcinoma: a randomised trial. Lancet. 2001;358(9286):966-70. Epub 2001/10/05.

40. Warde P, Mason M, Ding K, Kirkbride P, Brundage M, Cowan R, et al. Combined androgen deprivation therapy and radiation therapy for locally advanced prostate cancer: a randomised, phase 3 trial. The Lancet. 2011;378:2104-11. Epub 03-Nov-2011.

41. Neville-Webbe HL, Holen I, Coleman RE. The anti-tumour activity of bisphosphonates. Cancer Treatment Reviews. 2002;28(6):305-19.

42. Witters LM, Crispino J, Fraterrigo T, Green J, Lipton A. Effect of the combination of docetaxel, zoledronic acid, and a COX-2 inhibitor on the growth of human breast cancer cell lines. American journal of clinical oncology. 2003;26(4):S92-7. Epub 2003/08/07.

43. Kouloulias V, Giraud J-Y, Davis BJ, Dusserre A, Zurlo A, Bolla M. Quality assurance of the 22961 EORTC trial. A phase III study of the optimal combination of hormonal adjuvant treatment by LHRH analogue and radiation therapy for the management of locally advanced prostate cancer: the dummy run. 2004(0167-8140 (Print)).

44. Royston P, Parmar MKB, Qian W. Novel designs for multi-arm clinical trials with survival outcomes with an application in ovarian cancer. Statistics in Medicine. 2003;22(14):2239-56.

45. Royston P, Barthel FMS, Parmar MKB, Choodari-Oskooei B, Isham V. Designs for clinical trials with time-to-event outcomes based on stopping guidelines for lack of benefit. Trials. 2011;12(1):81.

46. Royston P. nstage: MAMS trial sample size calculator. MRC Clinical Trials Unit, London2009.

47. Haybittle JL. Repeated assessment of results in clinical trials of cancer treatment. The British journal of radiology. 1971;44(526):793-7. Epub 1971/10/01.

48. Peto R, Pike MC, Armitage P, Breslow NE, Cox DR, Howard SV, et al. Design and analysis of randomized clinical trials requiring prolonged observation of each patient. I. Introduction and design. British journal of cancer. 1976;34(6):585-612. Epub 1976/12/01.

49. Dolan P, Gudex C, Kind P, Williams A. Discussion Paper 138: A social tarif for EuroQol: results from a UK General Population Survey. . Centre for Health Economics. University of York, 1995.

STAMPEDE

Systemic Therapy in Advancing or Metastatic Prostate Cancer: Evaluation of Drug Efficacy

A multi-arm multi-stage randomised controlled trial

**Version:**	**12.0**
**Date:**	**13-Jan-2014**

MRC CTU ID:	PR08
ISRCTN #:	ISRCTN78818544
NCT #:	NCT00268476
EUDRACT #:	2004-000193-31
CTA #:	00316/0026/001-0001
MREC #:	04/MRE07/35

Authorised by:

Name:	PROFESSOR NICHOLAS D JAMES
Role:	CHIEF INVESTIGATOR

Name:	MATTHEW SYDES
Role:	TRIAL STATISTICIAN

GENERAL INFORMATION

This document was constructed using the MRC CTU Protocol Template Version 4.0. It describes the STAMPEDE trial, coordinated by the Medical Research Council (MRC) Clinical Trials Unit (CTU) at University College London (UCL), and provides information about procedures for entering patients into it. The protocol should not be used as an aide-memoire or guide for the treatment of other patients. Every care has been taken in drafting this protocol, but corrections or amendments may be necessary. These will be circulated to the registered investigators in the trial, but sites entering patients for the first time are advised to contact the Cancer And Other Non-Infectious Diseases Group, MRC CTU at UCL, London, UK, to confirm they have the most up-to-date version.

COMPLIANCE

The trial will be conducted in compliance with the approved protocol, the Declaration of Helsinki 1996, the principles of Good Clinical Practice (GCP), Commission Directive 2005/28/EC with the implementation in national legislation in the UK by Statutory Instrument 2004/1031 and subsequent amendments, the UK Data Protection Act (DPA number: Z5886415), and the National Health Service (NHS) Research Governance Framework for Health and Social Care (RGF). International sites will comply with the principles of GCP as laid down by the ICH topic E6 (Note for Guidance on GCP), Commission Directive 2005/28/EC (the European Directive 2001/20/EC [where applicable]) and applicable national regulations.

SPONSOR

Medical Research Council, 2nd Floor, David Phillips Building, Polaris House, North Star Avenue, Swindon, SN2 1FL, UK

FUNDING

Clinical Trials Advisory Awards Committee (on behalf of Cancer Research UK, Medical Research Council, and other charities) together with educational grants from Novartis, Sanofi-Aventis, Pfizer ,Janssen Pharma NV, Astellas.

AUTHORISATIONS AND APPROVALS

The following persons are authorised to sign the final protocol and protocol amendments for the sponsor: Professor Nicholas James (Chief Investigator) and Matthew Sydes (Trial Statistician).

TRIAL REGISTRATION

This trial has been registered with the ClinicalTrials.gov Clinical Trials Register, where it is identified as NCT00268476.

RANDOMISATIONS
To randomise, call MRC CTU at UCL, Monday to Friday 0900-1700
excluding public holidays or dates when notice has been given by the CTU.
Tel: +44 (0) 20 7670 4777

SAE REPORTING
Fax to 020 7670 4818 within 24 hours of becoming aware of the event

TRIAL ADMINISTRATION

COORDINATING SITE

MRC Clinical Trials Unit at UCL
Aviation House
Kingsway
London
WC2B 6NH
UK

Switchboard: 020 7670 4700
Fax: 020 7670 4818
Email: mrcctu.stampede@ucl.ac.uk

MRC CTU STAFF

Trial Manager:	Francesca Schiavone	Tel:	0207 670 4632
Trial Manager:	Alanna Brown	Tel:	0207 670 4882
Data Manager:	Dominic Hague	Tel:	0207 670 4947
Data Manager:	Katie Ward	Tel:	0207 670 4794
Data Manager:	Peter Vaughan	Tel:	0207 670 4655
Clinical Project Manager	Claire Amos	Tel:	0207 670 4771
Statistician:	Melissa Spears	Tel:	0207 670 4764
Senior Trial Statistician:	Matt Sydes	Tel:	0207 670 4798
Director of MRC CTU:	Max Parmar	Tel:	0207 670 4729
Trial Surgeon:	Alastair Ritchie	Tel:	Please contact STAMPEDE team

Chief Investigator

Professor Nicholas James
Warwick Medical School
University of Warwick
Coventry
CV4 7AL
UK

Tel: 024 7657 4365 (Secretary)
Fax:
Email: n.d.james@warwick.ac.uk

Co-Investigators

Prof Noel Clarke<br>Urologist	Manchester, UK	Vice-Chair
Prof Johann De Bono<br>Oncologist	London, UK	
Prof Malcolm Mason<br>Oncologist	Cardiff, UK	Vice-Chair
Dr Chris Parker<br>Oncologist	London, UK	Lead: M1/RT comparison
Dr Gerhardt Attard<br>Oncologist	London, UK	Lead: Enzalutamide + Abiraterone comparison
Dr Daniel Aebersold<br>Oncologist	Bern, Switzerland	
Dr Martin Russell	Glasgow, UK	
Prof George Thalmann<br>Urologist	Bern, Switzerland	

Patient Representative
Robin Millman
Patient

David Matheson
Patient

Health Economics
Prof Mark Sculpher — York, UK

Molecular Genetics
Prof John Masters, Pathologist — London, UK

For full details of all trial committees, please see **Appendix M**.

CONTENTS

GENERAL INFORMATION I

CONTENTS 4

ABBREVIATIONS 8

1 SUMMARY 11
1.1 LAY SUMMARY 11
1.2 ABSTRACT AND SUMMARY OF TRIAL DESIGN 12
1.3 TRIAL DOCUMENTATION 18

2 BACKGROUND 19
2.1 INTRODUCTION AND SETTING 19
2.1.1 Long-term Androgen Deprivation Therapy 19
2.1.2 Role of Radiotherapy for Patients With M0 Disease 19
2.2 RATIONALE 19
2.3 DESIGN 20
2.4 RESEARCH TREATMENT AND BISPHOSPHONATES 20
2.5 RESEARCH TREATMENT: CHEMOTHERAPY 21
2.6 RESEARCH TREATMENT: CYCLOOXYGENASE-2 INHIBITORS 21
2.7 RESEARCH TREATMENT: STEROID SYNTHESIS INHIBITORS 22
2.8 RESEARCH TREATMENT: RADIOTHERAPY TO THE PROSTATE FOR PATIENTS WITH NEWLY-DIAGNOSED METASTATIC DISEASE 22
2.9 RESEARCH TREATMENT: COMBINATIONS OF ORIGINAL RESEARCH ARMS 23
2.9.1 Bisphosphonate And Chemotherapy 23
2.9.2 Bisphosphonate And Cyclooxygenase-2 Inhibitors 24
2.10 COMBINATION OF STEROID SYNTHESIS INHIBITORS AND ANDROGEN RECEPTOR SIGNALLING INHIBITOR 25
2.10.1 Supplementing Abiraterone And Prednisolone With Enzalutamide 25
2.10.2 Supplementing Enzalutamide With Abiraterone And Prednisolone 25
2.10.3 Summary Of Rationale For This Combination 25

3 SELECTION OF INSTITUTIONS AND INVESTIGATORS 27
3.1 RADIOTHERAPY ACCREDITATION 27

4 SELECTION OF PATIENTS 28
4.1 PATIENT INCLUSION CRITERIA 28
4.1.1 High-Risk Newly-Diagnosed Non-Metastatic Node-Negative Disease 28
4.1.2 Newly-Diagnosed Metastatic Or Node-Positive Disease 28
4.1.3 Previously Treated With Radical Surgery And/Or Radiotherapy, Now Relapsing 28
4.1.4 For All Patients 28
4.2 PATIENT EXCLUSION CRITERIA 29
4.3 SELECTION CRITERIA FOR COMPARISON OF RESEARCH (M1) RT FOR METASTATIC DISEASE 30
4.4 SCREENING PROCEDURES 30
4.4.1 Investigations Prior To Randomisation 30
4.4.2 Androgen Deprivation Therapy Prior To Randomisation 31
4.4.3 Hypercalcaemia At Randomisation 31

4.4.4 NSAIDs And Cox-2 Inhibitors At Randomisation 31
4.4.5 Starting Trial Treatment 31
4.4.6 Concomitant Medications 32
4.5 ADDITIONAL DETAILS FOR PATIENTS JOINING SUB-STUDIES 32

5 RANDOMISATION AND ENROLMENT 33
5.1 CO-ENROLMENT GUIDELINES 33

6 TREATMENT OF PATIENTS 34
6.1 TRIAL TREATMENT 34
6.2 ARM A: ADT ALONE OR ADT + STANDARD-OF-CARE (M0) RT (CONTROL ARM) 34
6.2.1 Hormone Therapy 34
6.2.2 Standard-of-care (M0) RT 34
6.3 ARM B: ADT + ZOLEDRONIC ACID 35
6.4 ARM C: ADT + DOCETAXEL 35
6.5 ARM D: ADT + CELECOXIB 35
6.6 ARM E: ADT + DOCETAXEL + ZOLEDRONIC ACID 36
6.7 ARM F: ADT + ZOLEDRONIC ACID + CELECOXIB 36
6.8 ARM G: ADT + ABIRATERONE 36
6.9 ARM H: ADT + PROSTATE RADIOTHERAPY IN M1 PATIENTS 37
6.10 ARM J: ADT + ABIRATERONE + PREDNISOLONE + ENZALUTAMIDE ADMINISTRATION 38
6.11 ADMINISTRATION AND DOSE MODIFICATIONS 38
6.11.1 Zoledronic Acid 38
6.11.2 Docetaxel 39
6.11.3 Celecoxib 39
6.11.4 Abiraterone or Enzalutamide + Abiraterone 40
6.11.5 Research (M1) Prostate Radiotherapy 42
6.12 TRIAL PRODUCTS 44
6.13 MEASURES OF COMPLIANCE/ADHERENCE 44
6.14 TREATMENT DATA COLLECTION 44
6.15 ADMINISTRATION OF STANDARD RADIOTHERAPY TO NON-METASTATIC PATIENTS 44
6.15.1 Treatment Details 44
6.16 NON-TRIAL TREATMENT 46
6.16.1 Medications Permitted 46
6.16.2 Data On Concomitant Medication 46

7 ASSESSMENTS AND PROCEDURES 47
7.1 SCHEDULE FOR ASSESSMENTS 47
7.1.1 PSA Measurements 47
7.1.2 Assessment Of Treatment Failure (Definition Of Progression) 47
7.1.3 Additional Safety Assessment 47
7.1.4 Data Collection and Non-Administration Of Standard Radiotherapy 48
7.1.5 Data Collection Palliative Radiotherapy 48
7.1.6 Data Collection Research (M1) Radiotherapy 48
7.1.7 Follow-Up Schedules 48
7.2 FOLLOW-UP 48
7.3 TRIAL CLOSURE 52

8 STOPPING OF TREATMENT OR FOLLOW UP 53
8.1 STOPPING RESEARCH INTERVENTIONS 53

8.2 PATIENT TRANSFERS 53
8.3 WITHDRAWAL FROM THE TRIAL COMPLETELY 53

9 STATISTICAL CONSIDERATIONS 55
9.1 METHOD OF RANDOMISATION 55
9.1.1 To Version 7 55
9.1.2 Version 8 55
9.1.3 Version 10 and 11 55
9.1.4 Version 12 55
9.2 OUTCOME MEASURES 56
9.3 SAMPLE SIZE: PRINCIPLES AND ASSUMPTIONS 57
9.4 SAMPLE SIZE ISSUES AND TRIAL STAGES: ORIGINAL RESEARCH ARMS (B-F) 58
9.4.1 Pilot Phase: Original Research Arms (B-F) 58
9.4.2 Activity Stages I-III: Original Research Arms (B-F) 58
9.4.3 Efficacy Stage IV: Original Research Arms (B-F) 59
9.4.4 Sample Size For Original Research Arms (B-F) 59
9.5 SAMPLE SIZE ISSUES AND TRIAL STAGES: ADDITIONAL RESEARCH ARM G 59
9.5.1 Pilot Phase: Additional Research Arm G 59
9.5.2 Activity Stages I-III: Additional Research Arm G 59
9.5.3 Efficacy Stage IV: Additional Research Arm G 60
9.5.4 Sample Size For Additional Research Arm G 60
9.6 SAMPLE SIZE ISSUES AND TRIAL STAGES: ADDITIONAL RESEARCH ARM H 61
9.6.1 Pilot Phase: Additional Research Arm H 61
9.6.2 Activity Stages I-III: Additional Research Arm H 61
9.6.3 Efficacy Stage IV: Additional Research Arm H 61
9.6.4 Sample Size For Additional Research Arm H 61
9.7 SAMPLE SIZE ISSUES AND TRIAL STAGES: ADDITIONAL RESEARCH ARM J 62
9.7.1 Pilot Phase: Additional Research Arm J 62
9.7.2 Activity Stages I-II: Additional Research Arm J 62
9.7.3 Efficacy Stage III: Additional Research Arm J 62
9.7.4 Sample Size For Additional Research Arm J 63
9.7.5 Further Sample Size Issues For Additional Research Arm J 63
9.8 FURTHER NOTES ON TRIAL DESIGN 64
9.8.1 Overall Sample Size 64
9.8.2 Factorial Design 64
9.9 INTERIM MONITORING AND ANALYSES 65
9.10 OUTLINE ANALYSIS PLAN 65
9.10.1 Pilot / Safety Phases 65
9.10.2 Activity And Efficacy Stages 66

10 MONITORING AND QUALITY ASSURANCE 67
10.1 MONITORING AT MRC CTU 67
10.2 DIRECT ACCESS TO DATA 67
10.3 VISITS TO INVESTIGATOR SITES 67
10.4 CONFIDENTIALITY 67

11 SAFETY REPORTING 68
11.1 DEFINITIONS 68
11.1.1 Trial-Specific Exemptions 69
11.2 INSTITUTION/INVESTIGATOR RESPONSIBILITIES 69

11.2.1 Investigator Assessment 69
11.2.2 Notification Procedure 70
11.3 MRC CTU RESPONSIBILITIES 71

12 ETHICAL CONSIDERATIONS AND APPROVAL 72
12.1 ETHICAL CONSIDERATIONS 72
12.2 ETHICAL APPROVAL 72

13 REGULATORY APPROVAL 74

14 INDEMNITY 75

15 FINANCE 76

16 TRIAL COMMITTEES 77
16.1 TRIAL MANAGEMENT GROUP (TMG) 77
16.2 TRIAL STEERING COMMITTEE (TSC) 77
16.3 INDEPENDENT DATA MONITORING COMMITTEE (IDMC) 77

17 ANCILLARY STUDIES 79
17.1 QUALITY OF LIFE 79
17.2 HEALTH ECONOMICS 79
17.3 TRANSLATIONAL SUB-STUDIES 79
17.3.1 DNA Analysis 79
17.3.2 Tissue Microarray 80

18 PUBLICATION 81

19 PROTOCOL AMENDMENTS 82
19.1 PROTOCOL 82
19.1.1 Amendments Made To Sections In Protocol Version 1.0 (May 2004) 82
19.1.2 Amendments Made To Sections In Protocol Version 1.1 (May 2005) 82
19.1.3 Amendments Made To Sections In Protocol Version 2.0 (Jun 2005) 82
19.1.4 Amendments Made To Section In Protocol Version 3.0 (Jul 2006) 83
19.1.5 Amendments Made To Protocol Version 4.0 (Dec 2007) 84
19.1.6 Amendments Made To Protocol Version 5.0 (Aug 2008) 84
19.1.7 Amendments Made To Protocol Version 6.0 (Jul 2009) 85
19.1.8 Amendments Made To Protocol Version 7.0 (Jul 2011) 86
19.1.9 Amendments Made To Protocol Version 7.1 (Jul 2011) 86
19.1.10 Amendments Made To Protocol Version 8.0 (Sep 2011) 87
19.1.11 Amendments Made To Protocol Version 9.0 (Oct-2012) 88
19.1.12 Amendments Made To Protocol Version 10.0 (Apr-2013) 89
19.1.13 Amendments Made To Protocol Version 11.0 (Sep-2013) 89

20 REFERENCES 90

ABBREVIATIONS

Abbreviation	Expansion
ACE	Angiotensin-Converting Enzyme
ACTH	Adrenocorticotropic hormone
ADT	Androgen deprivation therapy
AR	Androgen receptor
AS	Activity Stage
bid	Twice a day (bis in die)
BP	Blood pressure
BSA	Body surface area
CERES	Consumers for Ethics in Research
CF	Consent Form
CI	Chief Investigator
CI	Confidence interval
COSTART	Coding Symbols for a Thesaurus of Adverse Reaction Terms
Cox 2	Cyclooxygenase 2
CRF	Case Report Form
CRUK	Cancer Research UK
CRPC	Castrate Refractory Prostate Cancer
CT	Computerised tomography
CTA	Clinical Trials Authorisation
CTAAC	Clinical Trials Advisory and Awards Committee
CTC	Common Toxicity Criteria
CTU	Clinical Trials Unit
CTV	Clinical Tumour Volume
CXR	Chest X-ray
DDX	Doctors and Dentists Exemption
DHT	Dihydrotestosterone
DNA	Deoxyribonucleic Acid
DPA	Data Protection Act
ERC	Endpoint Review Committee
ES	Efficacy Stage
ICH	International Conference on Harmonization

Abbreviation	Expansion
ECG	Electro cardiogram
FBC	Full Blood Count
FFS	Failure-Free Survival
GCP	Good Clinical Practice
GP	General Practitioner
GRO	General Register Office
HE	Health Economics
HES	Hospital Episode Statistics
hr	Hour
HR	Hazard Ratio
HRPC	Hormone Refractory Prostate Cancer
HT	Hormone Therapy
IDMC	Independent Data Monitoring Committee
IM	Intramuscular
IMRT	Intensity Modulated Radiation Therapy
ISRCTN	International Standard Randomised Controlled Trial Number
IU	International Units
IV	Intravenous
LD	Longest diameter
LFTs	Liver Function Tests
LHRH	Luteinising Hormone Releasing Hormone
LREC	Local Research Ethics Committee
m	Month
MHRA	Medicine and Healthcare Products Regulatory Agency
min	Minutes
MRC	Medical Research Council
MREC	Multi-Centre Research Ethics Committee
MRI	Magnetic resonance imaging
M0	Non-metastatic
M1	Metastatic
NCI	National Cancer Institute (USA)
NCRN	National Cancer Research Network
NHS	National Health Service

Abbreviation	Expansion
NSAID	Non-Steroidal Anti-inflammatory Drugs
ONS	Office for National Statistics
OS	Overall Survival
PI	Principal Investigator
PIS	Patient Information Sheet
po	per orum (orally)
PSA	Prostate Specific Antigen
pts	Patients
PTV	Planned Tumour Volume
QALY	Quality-adjusted Life Years
qds	quater die sumendus (4 times each day)
QL	Quality of Life
R&D	Research and Development
RECIST	Response Evaluation Criteria In Solid Tumours
SAE	Serious Adverse Event
SAR	Serious Adverse Reaction
sc	Sub-cutaneous (under skin)
SNP	Single Nucleotide Polymorphism
SSA	Site Specific Assessment
STAMPEDE	Systemic Therapy in Advancing and Metastatic Prostate Cancer: Evaluation of Drug Efficacy
SUSAR	Suspected Unexpected Serious Adverse Reactions
SWOG	South West Oncology Group
TMG	Trial Management Group
TMT	Trial Management Team
TURP	Trans-Urethral Resection of Prostate
TSC	Trial Steering Committee
UCL	University College London
ULN	Upper Limit of Normal
U+E	Urea and Electrolytes
WHO	World Health Organisation

1 SUMMARY

1.1 LAY SUMMARY

Prostate cancers depend upon the male hormone testosterone for their growth. Lowering testosterone levels (either by removing all or part of both testes, or by giving anti-hormone treatment) slows the growth of prostate cancers. This type of treatment is called hormone treatment and is often used when prostate cancers have spread outside the prostate gland. Although hormone treatment is usually successful at stopping the cancer growing for a period of time, the cancer will begin to grow again in most men.

There are increasing numbers of treatments available for advanced prostate cancer. These treatments are usually used in prostate cancer when hormone treatment is no longer effective and the cancer has started to grow again. The aim of this trial, which is called STAMPEDE, is to assess some of these treatments, given earlier in the course of the disease in combination with hormone treatment.

The treatments that have been, or are being, assessed during the trial are:

1. Zoledronic acid: Prostate cancer cells can spread to bones and weaken them. Zoledronic acid is a drug that reduces bone destruction and hardens bones. This may make them more resistant to attack by cancer cells.

2. Docetaxel: A drug that stops cells replicating that is currently being used to treat a range of cancers including lung, breast and ovarian cancer as well as prostate cancer. Docetaxel prolongs survival in men with relapsed metastatic prostate cancer.

3. Celecoxib: An aspirin-like drug that is used to treat arthritis. It slows down the growth of cancer cells in the laboratory. We wished to see if it had the same effect on cancer cells in patients. Recruitment to new patients for the evaluation of this drug is finished as a planned interim analysis failed to demonstrate sufficient activity.

4. Abiraterone (included from protocol version 8.0)**:** An inhibitor of steroid hormone synthesis that blocks prostate cancer cells from generating their own male hormones. This is thought to be a major way in which prostate cancer cells resume growth following anti-hormonal therapies. The agent prolongs survival when given to men following failure of docetaxel chemotherapy.

5. Prostate radiotherapy (included from protocol version 9.0)**:** treatment with high-energy x-rays targeted to the prostate gland. This treatment is now mandatory for patients with cancer that is confined to the prostate gland as large trials have shown it improves survival times. We are not certain whether we should give radiotherapy to the prostate if the cancer has already spread.

6. Enzalutamide (included from protocol version 12.0)**:** This is a blocker of androgen receptors. These stimulate the cancer when hormone therapies have failed. Enzalutamide may be mutually complementary to abiraterone in terms of blocking mechanisms of resistance. The agent prolongs survival when given to men following failure of docetaxel chemotherapy.

STAMPEDE will look at the effect of combining one or two of the treatments described above with hormone treatment. A computer program will be used to allocate which treatment the patient receives, using a chance process. The trial will look at the effects of the combined treatments on quality of life and find out whether the new treatment combinations increase the time when the cancer is not growing and ultimately results in patients living longer. The study will also look at which treatment provides the greater value for money for the health service. More than 7,000 patients will join the trial with answers becoming available over 7 to 12 years.

1.2 ABSTRACT AND SUMMARY OF TRIAL DESIGN

STAMPEDE is a multi-centre, randomised controlled trial for patients with locally advanced or metastatic prostate cancer who are about to commence Androgen Deprivation Therapy (ADT). Patients can have either newly diagnosed disease, or have been previously treated with radical radiotherapy or surgery but now have signed of progression such as a rising prostate specific antigen (PSA) (further details on eligibility see **Section 4**). The trial will assess the effects of adding different agents, both as single agents and in combinations, to androgen deprivation therapy. The investigational agents are (i) a bisphosphonate, zoledronic acid, (ii) a cytotoxic chemotherapeutic agent, docetaxel and (iii) a cyclooxygenase (Cox-2) inhibitor, celecoxib (iv) a novel androgen deprivation therapy drug called abiraterone, a steroid synthesis inhibitor and a androgen receptor signalling inhibitor (v) enzalutamide. Recruitment to the celecoxib arms (D and F) is now closed. An additional arm containing abiraterone was added in protocol version 8.0. A further comparison arm involving prostate radiotherapy for patients with metastatic disease was added in protocol version 9.0. The trial has multiple arms; the control arm of the trial is androgen deprivation therapy (ADT) only, achieved through the use of luteinising hormone releasing hormone (LHRH) analogues or LHRH antagonists, or bilateral orchidectomy according to local practice. The other trial arms are summarised in **Figures 1 to 7**.

Figure 1: Recruiting arms of the STAMPEDE trial to Apr-2011

Patients eligible for STAMPEDE

↓

STARTING LONG-TERM HORMONES

RANDOMISATION

**A**	ADT
**B**	ADT + zoledronic acid
**C**	ADT + docetaxel
**D**	ADT + celecoxib
**E**	ADT + ZA + docetaxel
**F**	ADT + ZA + celecoxib

Figure 2: Recruiting arms of the STAMPEDE trial from Apr-2011 to Nov-2011 (v7.0)

Patients eligible for STAMPEDE

↓

STARTING LONG-TERM HORMONES

RANDOMISATION

**A**	ADT
**B**	ADT + zoledronic acid
**C**	ADT + docetaxel
**E**	ADT + ZA + docetaxel

Accrual stopped to celecoxib-containing Arms, D and F, after their Activity Stage II analysis

Figure 3: Recruiting arms of the STAMPEDE trial from Nov-2011 to Jan-2013 (v9.0)

Patients eligible for STAMPEDE

↓

STARTING LONG-TERM HORMONES[1]

RANDOMISATION

**A**	ADT
**B**	ADT + zoledronic acid
**C**	ADT + docetaxel
**E**	ADT + ZA + docetaxel
**G**	ADT + abiraterone

[1] All suitable pts with newly diagnosed locally advanced disease should also have RT to the prostate

Accrual was initiated to the abiraterone arm, Arm G, in Nov-2011.

Figure 4: Recruiting arms of the STAMPEDE trial from protocol version 9.0 (Jan-2013 to Mar-2013)

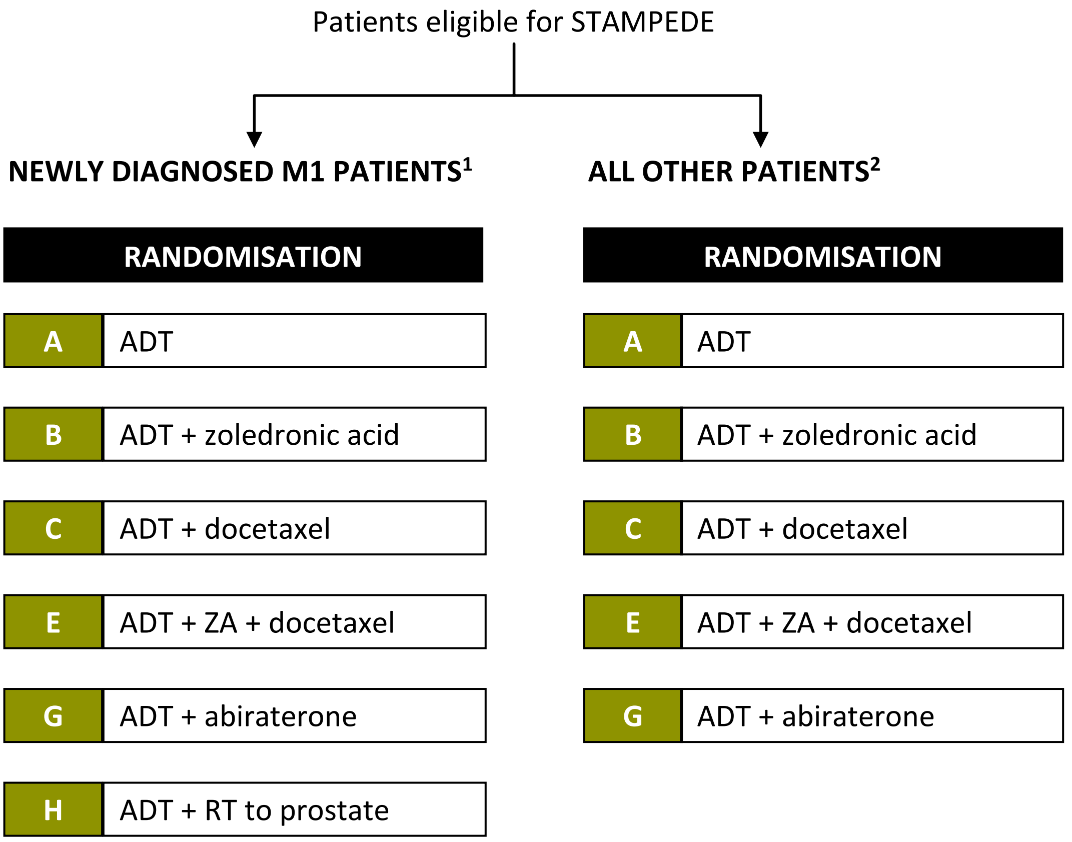

[1] Except pts with a contra-indication to RT
[2] All suitable pts with newly diagnosed locally advanced disease should also have RT to the prostate

Accrual was initiated to the radiotherapy-to-the-prostate for metastatic disease arm, Arm H, in Jan-2013.

Figure 5: Arms of the STAMPEDE Trial from protocol version 10.0 (after original research arms completed accrual in March 2013)

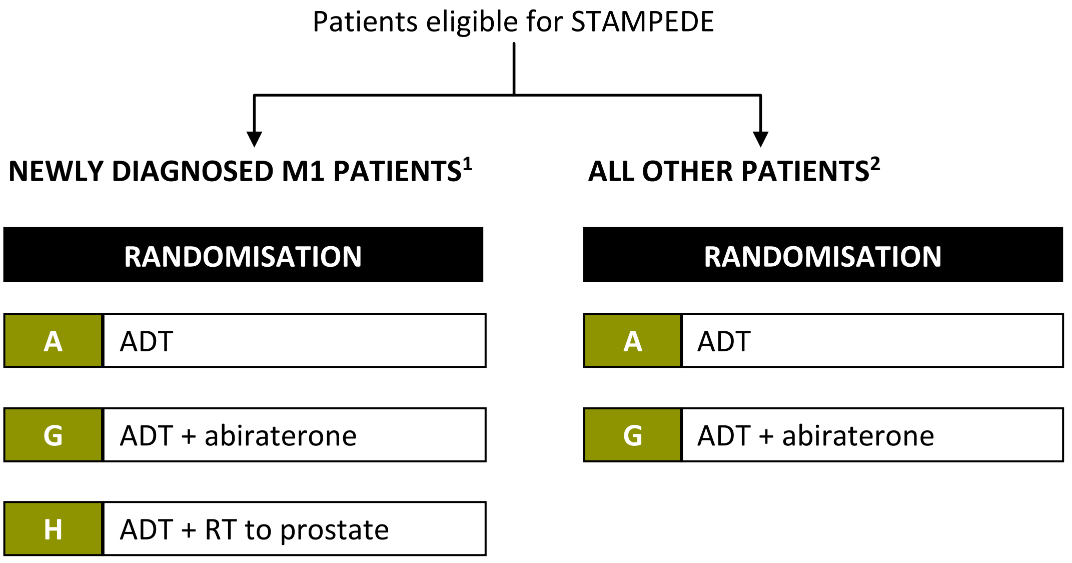

[1] Except pts with a contra-indication to RT
[2] All suitable pts with newly diagnosed locally advanced disease should also have RT to the prostate

Figure 6: Arms of the STAMPEDE Trial from protocol version 12.0 (end of recruitment to Arm G and introduction of enzalutamide + abiraterone comparison)

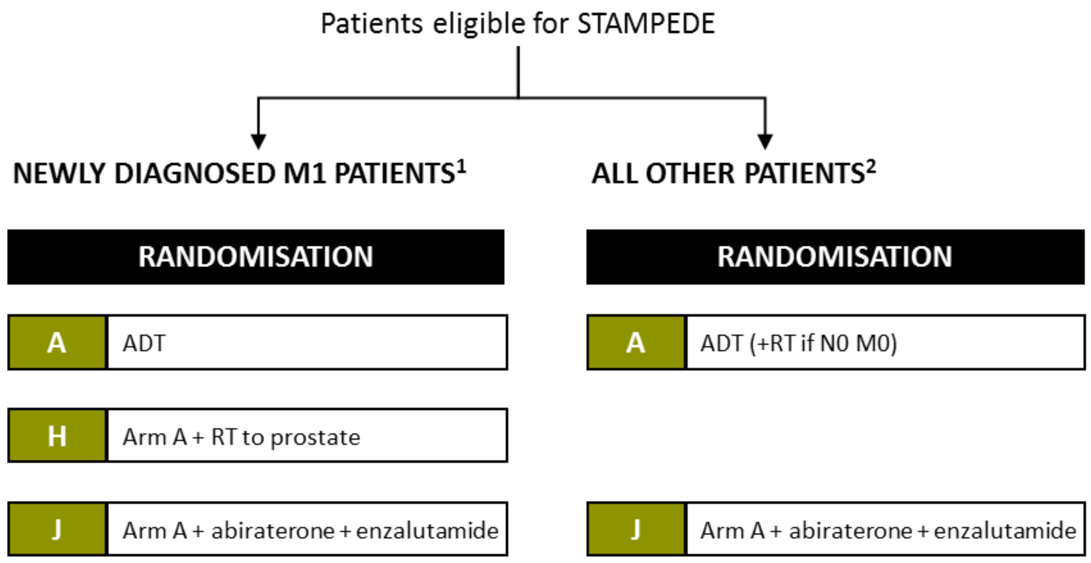

[1] Except pts with a contra-indication to RT
[2] All suitable pts with newly diagnosed locally advanced disease should also have RT to the prostate1

Figure 7: Arms of the STAMPEDE trial open to recruitment over time

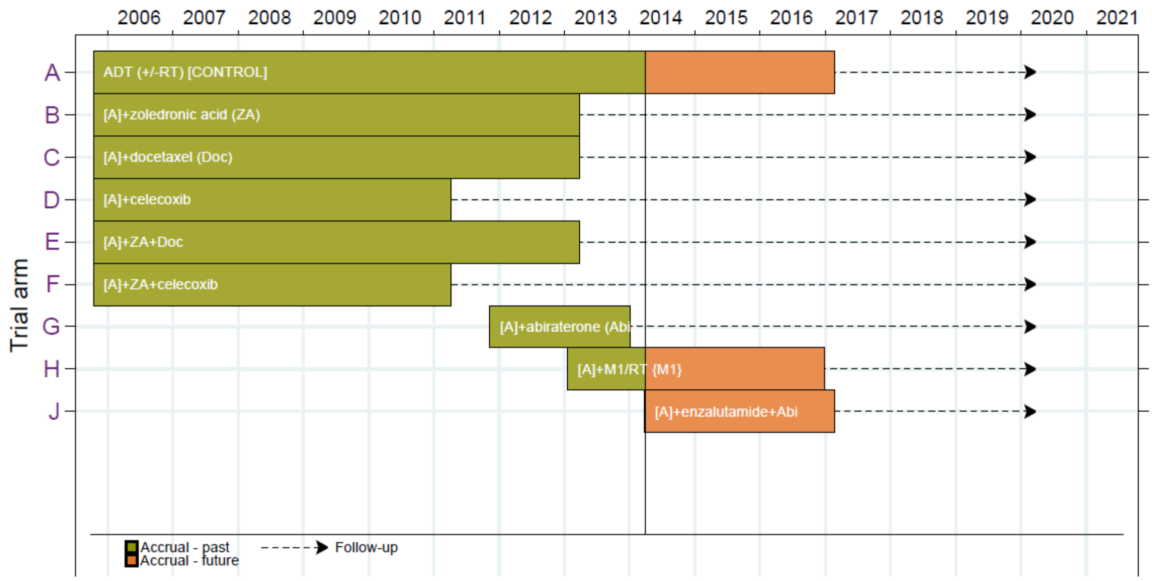

For each comparison of research arm against control, the trial will be conducted in a number of stages: a Pilot/Safety Phase, Activity Stages and a final Efficacy Stage. The primary outcome measure of the Pilot/Safety Phase is the safety, with 30 patients recruited to each research arm. Research arms will only continue to recruitment in the next stage if they have been shown to be both safe and feasible, although patient data from all patients and all stages will be included in the final analyses. In the Activity Stages until the primary outcome measure is failure-free survival (FFS). Further patients will be recruited until a certain number of FFS events have been observed in the control arm (see **Section 9** for further detail). Some evidence of activity will be required for a research arm to proceed to further recruitment in each stage and guidelines are in place. In the Efficacy Stage, patients will be recruited when a certain number of primary outcome measure events are foreseeable in the control arm. This is when around 403 deaths have been reported in the control arm for the "original research comparisons" and around 267 in the control arm for the "abiraterone comparison", the "M1/RT comparison" and the "enzalutamide+abiraterone comparison". The exact number of patients and duration of the trial will depend on the observed accrual rate, observed event rate and the number of other arms open to recruitment contemporaneously.

Recruitment to Arms D (ADT + celecoxib) and F (ADT + zoledronic acid + celecoxib) was stopped in Apr-2011 after the second planned activity analysis when the IDMC and TSC considered the lack-of-benefit guidelines.(1) Refer to **Section 9.3** for further information regarding the guidelines for stopping accrual to research arms during the activity stages of the trial.

In version 8.0 of the protocol a new arm G (ADT + abiraterone) was added. Arm H (ADT+ prostate radiotherapy) was added in protocol version 9.0. The trial stages remain as at trial inception but will be staggered in time compared to the stages for the original arms A-F. Protocol version 10.0 was approved following the completion of recruitment to the remaining original trial arms (B, C and E) and was a "housekeeping" change to remove references to the completed arms from the information sheets. Protocol version 11.0 was approved following the extension of the recruitment target sample for the "abiraterone comparison" from 1,500 to 1,800 patients. Protocol version 12.0 adds a new combination therapy arm containing abiraterone with enzalutamide.

Patients will be assessed 6 weekly for the first 24 weeks after randomisation and then every 12 weeks up to 2 years, then 6-monthly until 5 years and annually, thereafter. The first 700 patients on trial completed questionnaires aimed at assessing the effects of the investigational treatments on their quality of life (QL) and on their use of health care resources (Health Economics (HE) study). From protocol version 8.0, the QL and HE study has been re-opened to all new patients.

In addition, there are translational sub-studies. Patients willing to participate will be asked at randomisation to donate a droplet of blood, which will be stored for DNA and protein analysis in order to try to identify markers that are associated with response to therapy, side-effects or susceptibility to prostate cancer.

Patients will also be asked to give permission to use some of their stored material (blood or biopsy samples) for further studies on the causes and nature of prostate cancer. In selected centres patients were asked to participate in a bone mineral density sub-study. This sub-study has now stopped recruitment. There are separate patient information sheets for the QL and HE study and the translational sub-studies (For further details of ancillary studies, see **Section 17**).

1.3 TRIAL DOCUMENTATION

Table 1 presents a summary of the required trial documentation for participating centres

Table 1: Trial documentation required for participating centres

TRIAL DOCUMENTATION	TIMING
R&D approval (including IRMER approval)	Before centre participation
Investigator Statement	Before centre participation
Signature list & delegation of responsibilities	Before centre participation
Trial personnel contact details	Before centre participation
PIS, GP & CF on local paper	Before centre participation
Signed Clinical Trial Agreement between Trust and Sponsor	Before centre participation
RTQA accreditation	Before centre participation
Clinical Trial Agreement (or Variation if applicable)	Before centre participation

2 BACKGROUND

2.1 INTRODUCTION AND SETTING

Prostate cancer is a major health problem world-wide and accounts for nearly one fifth of all newly diagnosed male cancers. In the UK, approximately 35,000 men are diagnosed with prostate cancer each year and in 2008 almost 10,000 men died from the disease.(2)

2.1.1 LONG-TERM ANDROGEN DEPRIVATION THERAPY

The initial (first line) treatment for locally advanced or metastatic prostate cancer is androgen deprivation therapy (ADT) achieved either surgically with bilateral orchidectomy, or medically with LHRH agonists or antagonists or oral anti-androgens alone. (3) Oral anti-androgens were permitted in the trial but were used by very few patients and are no longer permitted for new patients within the trial from version 8.0.

ADT produces responses in up to 95% of patients but it is not curative and disease recurs in virtually all patients treated with ADT as sole therapy, with a median time to progression of 18-24 months. (3) Such disease is referred to as hormone-refractory or, increasingly, as castrate resistant prostate cancer (HRPC or CRPC); this latter term is unpopular with patient groups due to its perceived pejorative overtones related to castration and hence terminology may yet change again in the future.

2.1.2 ROLE OF RADIOTHERAPY FOR PATIENTS WITH M0 DISEASE

Two randomised trials, SPCG7 (4) and NCIC PR.3 / MRC PR07 (5-7) have tested the question of whether androgen deprivation therapy alone or combined with radiotherapy is the best treatment for high-risk patients with no evidence of spread outside the pelvis. Both trials demonstrate an improvement in overall and disease specific survival from the addition of radiotherapy to androgen deprivation therapy. The size of this overall survival benefit is substantial (hazard ratio 0.68 in SPCG7 and HR 0.77 in PR07). With substantial benefit demonstrated in two mature, large, well conducted randomised trials, we now recommend that radiotherapy be considered standard for patients with no nodal or metastatic spread. Patients in this category will now only be allowed to enter the trial if standard radiotherapy is planned, with the exception of those for whom radiotherapy is contra-indicated. Such patients should be discussed with the Trials Unit prior to inclusion. For patients with node positive, M0 disease there are no clear data on whether radiotherapy is or is not indicated. The NCIC PR.3 / MRC PR07 trial included patients with unknown nodal status who received whole pelvic radiotherapy. Given the large overall benefit observed in this trial, the STAMPEDE TMG recommends that pelvic nodal radiotherapy be considered for patients with node positive, non-metastatic disease at the discretion of the treating clinician.

2.2 RATIONALE

There are increasing numbers of treatments which are used post relapse of first-line androgen deprivation therapy in patients with CRPC, but little evidence as to which is associated with the best response or how they may be combined or sequenced or whether any of them might have a role in first-line treatment. Such treatments include further hormonal manipulations, bisphosphonates, (8), cytotoxic chemotherapy (9), new hormone therapies (10) and palliative radiotherapy. The traditional approach to the testing and introduction of new treatments for prostate cancer is to use them in

patients with castrate resistant disease. An alternative approach is to investigate new drugs and new approaches to treatment, as first-line therapy in patients starting androgen deprivation therapy. At this point, patients should be fitter and better able to tolerate treatment than when they have CRPC and there is the possibility of having a larger and longer lasting effect.

2.3 DESIGN

STAMPEDE (also known as MRC PR08) is an innovative, multi-arm multi-stage, multi-centre, randomised controlled trial. It initially assessed the effects of a bisphosphonate (zoledronic acid), a cytotoxic chemotherapeutic agent (docetaxel) and a cyclooxygenase (Cox-2) inhibitor (celecoxib), as single agents or combinations, in patients commencing androgen deprivation therapy for advancing or metastatic prostate cancer. Each comparison is divided into five stages such that, for each investigational arm, safety and activity data are generated in the first four stages; an investigational arm will only proceed to the fifth and final stage of recruitment, where it will be assessed for its effect on overall survival, if it has been shown to be sufficiently safe and active. It is important to note, however, that patient data from all arms and all stages will be included in the final analyses of the primary outcome measure, even if the investigational arm did not proceed to the final stage.

Planned interim analysis failed to demonstrate sufficient activity for celecoxib and this agent has now been removed from the trial recruitment; patients remaining on celecoxib treatment reverted to standard care. Protocol version 8.0 added a new drug abiraterone to the study as an additional arm (see Section 2.7). Protocol version 9.0 added a new comparison arm involving prostate radiotherapy for patients with metastatic disease (see Section 2.8). Protocol version 10.0 reflected the successful completion of recruitment to three docetaxel- and bisphosphonate-containing arms (Arms B, C and E) and removed references to these agents in the information sheets for new patients. Protocol version 11 extended the recruitment target for the abiraterone research comparison (A vs G) from 1,500 to 1,800 patients.   Current protocol 12.0 adds a new comparison involving the combination of abiraterone and enzalutamide.

2.4 RESEARCH TREATMENT AND BISPHOSPHONATES

Note: recruitment stopped to the zoledronic acid and docetaxel-containing arms in Mar-2013 at the end of Activity Stage IV.

The bisphosphonates are a class of drug that act by reducing osteoclast formation, inhibiting osteoclast activity and inducing osteoclast apoptosis. They are effective at controlling hypercalcaemia and preventing skeletal complications associated with malignant disease. (11, 12) Zoledronic acid is a highly potent, third generation bisphosphonate; studies comparing the efficacy of zoledronic acid to other bisphosphonates suggest that zoledronic acid has a 40-850 fold higher potency than clodronate in preclinical models of bone resorption. (13). It has also been shown to be more effective than pamidronate (90mg) in controlling malignant hypercalcaemia. (14) In addition, zoledronic acid has also demonstrated direct anti-cancer activity, including inhibition of proliferation of breast cancer and prostate cancer cells in vitro. (15)

In randomised controlled trials of 1,648 patients, 4mg zoledronic acid was more effective than pamidronate in reducing the risk of skeletal complications in patients with bone metastases from breast cancer. (16, 17) Also, in metastatic prostate cancer, zoledronic acid has been shown to reduce the rate of skeletal-related events compared to placebo in a trial involving 429 men. (18) In April 2002, zoledronic acid received approval from the Committee for Proprietary Medicinal Products for the prevention of skeletal-related events (for example, fractures) in patients with any advanced malignancies involving bone.

The MRC PR05 prostate cancer trial showed that a first generation bisphosphonate (clodronate) commenced at the time of androgen deprivation therapy initiation, delayed time to progression in patients with bony metastatic disease and there was some evidence that it may also improve survival. (19) There is, therefore, a good rationale for investigating a more potent bisphosphonate in patients with prostate cancer who are about to commence ADT therapy.

2.5 RESEARCH TREATMENT: CHEMOTHERAPY

Note: recruitment stopped to the zoledronic acid and docetaxel-containing arms in Mar-2013 at the end of Activity Stage IV.

There is increasing evidence of the clinical efficacy of chemotherapy in prostate cancer. (9) Two randomised phase III trials in patients with metastatic hormone refractory prostate cancer (HRPC) using a docetaxel-containing regimen have been completed: the SWOG 9916 study (20) and the TAX-327 study. (21) Both studies show that the use of a docetaxel-based regimen improved survival for patients with metastatic HRPC and had significantly greater PSA response rates compared to the mitoxantrone plus prednisolone arm.

In the TAX-327 trial, (21) 1,006 patients with metastatic HRPC were randomized to receive either mitoxantrone 12 mg/m² with prednisone 10mg daily (Arm C) or docetaxel 75mg/m² 3-weekly for 10 cycles with prednisone (Arm A) or docetaxel 30 mg/m²/wk x 5 of 6 weeks x 5 cycles with prednisone (Arm B). Median overall survival was 16.5 months for patients treated with mitoxantrone versus 18.9 months for the 3-weekly docetaxel regimen (hazard ratio 0.76 (0.62-0.94)). There was also improvements for 3-weekly docetaxel in pain (22% vs 35%, p = 0.01) and PSA response (32% vs 45%, p=0.0005).

In June 2006 in the UK docetaxel was given NICE (National Institute for Health and Clinical Excellence) approval for use in hormone (now more commonly termed castrate) refractory prostate cancer patients.

2.6 RESEARCH TREATMENT: CYCLOOXYGENASE-2 INHIBITORS

Note: recruitment completed to both celecoxib-containing arms in Apr-2011 at the end of Activity Stage II of this comparison

Cyclooxygenase-2 (Cox-2) is an isoenzyme induced by a variety of mitogens, cytokines and growth factors that are associated with a range of process including inflammation, (22) and carcinogenesis.(23, 24) There is a growing body of evidence that inhibition of Cox-2 may play an important role in the prevention of cancer and the delay of progression in established cancer. A number of case-control studies have shown a reduction in risk of prostate cancer associated with the use of non-steroidal anti-inflammatory drugs (NSAID), which include inhibition of Cox-2 amongst their mode of action. (25) Pathological studies show Cox-2 is upregulated in carcinomas (26) and one study suggested that NSAID use may delay progression from subclinical to clinical prostate cancer. (27)

Celecoxib, a Cox-2 inhibitor, is better tolerated than other NSAIDs and there is evidence that it is active as a chemoprevention agent. (28) It also has important antineoplastic properties such as the ability to inhibit angiogenic factors and induce apoptosis in human cancer cells including prostate cancer. (29)

Evidence has suggested that an anti-cancer effect is only seen at higher doses of celecoxib than **is** required for an anti-inflammatory effect. (30) Therefore, the dose of 800mg/day for STAMPEDE patients has been chosen. Although there is some high profile evidence of a small absolute increase in CVS toxicity risk associated with higher doses of celecoxib, (31) most current cancer trials are using a dose of 800mg/day as it is believed that a higher dose will result in a greater increase in cancer effect.

There is also some evidence of a schedule effect on CVS toxicity. It has been observed that CVS toxicity becomes evident after one year of taking celecoxib. (31) Therefore, a maximum duration of one year has been

set for celecoxib use in this trial. Any potential risks of course have to be weighed against any potential benefits of celecoxib in the delay of progression in established prostate cancer.

Given case-control data suggesting effects on prostate cancer, pathological expression of Cox-2 in prostate cancer and in vitro data suggesting that inhibition of Cox-2 inhibits growth and invasiveness, further investigation in prostate cancer is warranted.

2.7 RESEARCH TREATMENT: STEROID SYNTHESIS INHIBITORS

Recent evidence suggests that an important mechanism for escape from tumour control by androgen ablation is the intracellular conversion of steroid precursors to androgenic steroids by prostate cancer cells. A key enzyme in this process is CYP17, which therefore represents a logical target for therapy in CRPC. (10) Abiraterone acetate is a selective inhibitor of CYP17 and is highly active in patients developing resistance to standard androgen ablation therapies. (32-34) Recruitment to a phase III study comparing abiraterone acetate to placebo in CRPC patients post-docetaxel, completed accrual in 2009 and reported initial results in 2011 with an improvement in overall survival of around 4 months and a hazard ratio of 0.65. (35) The drug has now received a marketing authorisation in the USA and in the EU from September 2011. A second trial in pre-chemotherapy CRPC patients completed recruitment April 2010; preliminary results are positive and were published in 2012 (36) and the licence for abiraterone was extended to the pre-chemotherapy CRPC population in Europe in 2012. Side-effects with abiraterone acetate are modest with the main adverse effects being elevated transaminases (usually mild), hypokalaemia and hypertension due to secondary hyperaldosteronism and fluid retention (preventable by low doses of glucocorticoids). In order to prevent secondary hyperaldosteronism, it is recommended that prednisolone (or prednisone) 10mg daily be administered in the CRPC setting. Within more recent studies in earlier stage patients, lower doses (typically 5mg of prednisone/prednisolone) are being used due to concerns about long-term exposure to glucocorticoid side effects. More recent evidence even suggests that for most patients, no glucocorticoids may be needed. (37) Within the STAMPEDE trial, we suggest prednisolone/ prednisone dose of 5mg daily.

We hypothesise that the agent may be more active still when given up-front in combination with first-line androgen deprivation therapy by preventing or delaying the development of castrate refractory disease.

2.8 RESEARCH TREATMENT: RADIOTHERAPY TO THE PROSTATE FOR PATIENTS WITH NEWLY-DIAGNOSED METASTATIC DISEASE

Therapy directed against the primary tumour in the presence of metastatic disease has been evaluated rigorously in only one malignancy to date: renal cell carcinoma. Two cooperative groups ran randomised trials enrolling patients with previously untreated metastatic RCC whose primary tumours were amenable to surgical resection. Patients were randomized to receive the standard systemic therapy of the day, interferon-alpha, either alone or with radical nephrectomy. The combination of nephrectomy and interferon was shown to significantly improve median survival from 7 to 17 months in one trial (38) and from 8 to 11 months in the other.(39) The mechanism by which nephrectomy improves survival remains obscure. In preclinical models, the primary tumour has been found to secrete molecules that prime the microenvironment in which metastases can develop. An implication of this work is that therapy directed at the primary tumour, by abrogating this endocrine signalling, could retard the formation and the growth of distant metastases.

The results of two large-scale randomised trials of prostate radiotherapy are also provocative. The Scandinavian SPCG-7 trial and the MRC PR07 trial randomised men with locally advanced prostate cancer, who were at high risk of possessing occult metastatic disease, to either androgen deprivation therapy (ADT) alone or ADT plus prostate radiotherapy.(4, 40) The addition of radiotherapy

dramatically improved 10-year outcomes: mortality from prostate cancer was halved. Interestingly, the benefit of radiotherapy started to emerge as early as three years from the time of randomisation. This seems improbably early if the benefit of local treatment is mediated via the prevention of subsequent disease dissemination. Rather, it is more consistent with the possibility that local treatment has a beneficial impact on the rate of progression of existing micrometastatic disease.

We hypothesise that local therapy to the primary site may retard distant disease progression and prolong survival in patients with metastatic prostate cancer.

Figure 8: Use of RT in STAMPEDE

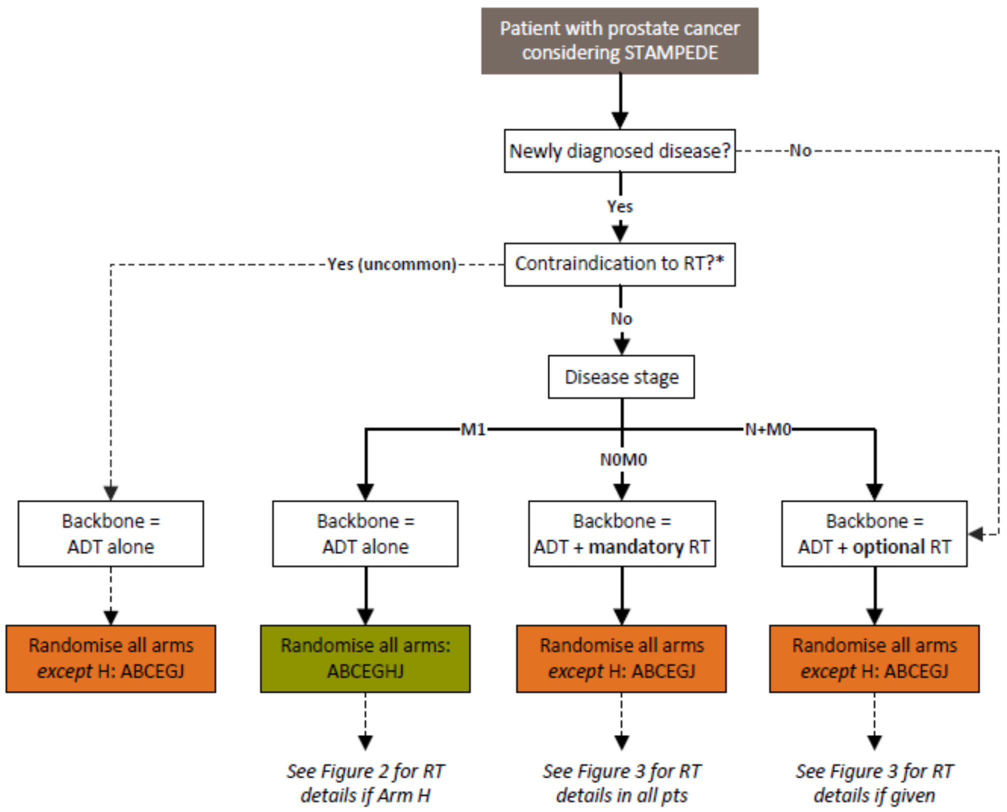

*It is expected that only around 1% of patients will have a contraindication to RT e.g. inflammatory bowel disease. These cases should be discussed with the trials unit prior to randomisation (see **Section 4.3**).

2.9 RESEARCH TREATMENT: COMBINATIONS OF ORIGINAL RESEARCH ARMS

2.9.1 BISPHOSPHONATE AND CHEMOTHERAPY

Note: recruitment stopped to the zoledronic acid and docetaxel-containing arms in Mar-2013 at the end of Activity Stage IV.

Zoledronic acid and docetaxel have different mechanisms of action. In addition to its skeletal protection activity, zoledronic acid has shown direct activity against prostate cancer cells, both in vitro and in vivo. (15)

There is also in vitro and in vivo evidence to suggest synergy between zoledronic acid and chemotherapy in breast cancer cells and anti-angiogenic effects in patients. (41, 42)

Toxicities of the two agents are complementary and administration in combination is expected to be feasible and safe. These aspects were evaluated in the initial Pilot Phase of the trial. Since both agents show considerable promise as single agents and there is in vitro evidence of synergy, we believe there is a strong rationale for evaluating these two agents in combination.

2.9.2 BISPHOSPHONATE AND CYCLOOXYGENASE-2 INHIBITORS

Note: recruitment stopped to both celecoxib-containing arms in Apr-2011 at the end of Activity Stage II.

An alternative approach to combination therapy is to target the principal site of relapse and a key mode of progression and this is the rationale for combining zoledronic acid with a Cox-2 inhibitor. Bisphosphonates have already been shown to delay bone disease progression in hormone refractory disease. (19) Cox-2 appears to play a crucial role in the molecular phenotype of advanced prostate cancer as outlined above, and this effect is likely to be apparent in both soft tissue and in bone. Toxicities of the two agents are likely to be complementary and there is no strong a priori reason to anticipate unacceptable toxicity. The Pilot Phase of the trial will evaluate tolerability and safety of the combination. Targeting both bone progression and the underlying molecular changes leading to progression can be expected to have synergistic benefits in terms of delaying development of hormone refractory disease.

2.10 COMBINATION OF STEROID SYNTHESIS INHIBITORS AND ANDROGEN RECEPTOR SIGNALLING INHIBITOR

The majority of patients with advanced prostate cancer who have disease progression on abiraterone or enzalutamide taken as single agents, have a rise in PSA, suggesting reactivation of androgen receptor (AR), or other steroid signalling pathways resulting in increased PSA transcription, is the pathway to the development of resistance.(43)

The question under investigation is: can progression be delayed (and survival extended) by using a combination of abiraterone and enzalutamide?

2.10.1 Supplementing Abiraterone And Prednisolone With Enzalutamide

Several studies have shown that the AR can become promiscuously activated by very low levels of androgens or other steroid metabolites and drugs that bind the AR.(44-47) It is known that very low levels of androgens can persist in patients treated with abiraterone acetate.(48) Drugs that bind the AR, may include co-administered glucocorticoids. Furthermore, AR mutations of the sort previously described in castration-resistant prostate cancer (CRPC), can be activated by cortisol and other glucocorticoids at levels much lower than those reported in patients treated with abiraterone and prednisolone at a dose of 5mg bid.(47, 49) Moreover, abiraterone binds the AR and although weak antagonism of wild-type and most previously described AR mutations are observed,(49) a similar mechanism to that described with classical anti-androgens, such as bicalutamide, could lead to change-of-function AR mutations associated with AR activation following abiraterone binding. Therefore, concomitant treatment with an androgen receptor signalling inhibitor could prevent “promiscuous” AR activation in patients treated with abiraterone. Enzalutamide is a androgen receptor signalling inhibitor and has gained recent approval for use on its own in the treatment of advanced CRPC,(50) and there is evidence of activity for hormone-naïve prostate cancer.(51)

2.10.2 Supplementing Enzalutamide With Abiraterone And Prednisolone

Enzalutamide in combination with ADT is effective and well tolerated in CRPC.(50) However, recent studies have suggested that intratumoral testosterone levels increase in patients treated with enzalutamide.(52) The implications of this finding are that the increase in intratumoral testosterone could be associated with up-regulation of enzymes involved in steroid biosynthesis.(53) Although enzalutamide has a high affinity for the AR, this is several-fold lower than both the natural ligands testosterone and DHT,(54) which means that enzalutamide would be out-competed at the AR ligand-binding domain if and when androgen levels rise. In vitro, a ten-fold rise in intra-cellular androgen was sufficient to prevent inhibition of AR by 30uM of enzalutamide;(49) these levels are representative of the plasma levels of enzalutamide active metabolites, which can be achieved with enzalutamide 160mg po daily.(55)

A strategy for preventing the rise in intra-cellular androgens in patients treated with enzalutamide would be inhibition of CYP17A1. Abiraterone is currently the only CYP17A1 inhibitor with proven efficacy. It therefore seems logical to use the combination of enzalutamide and abiraterone to both block a rise of intra-cellular androgens and prevent promiscuous activation of the AR.

2.10.3 Summary Of Rationale For This Combination

To date, investigation has focussed on patients with CRPC but there is a strong rationale for the combination of enzalutamide and abiraterone in the hormone treatment-naïve setting in which STAMPEDE is focused.

STAMPEDE already has an abiraterone plus conventional ADT arm but we will not assess the combination of conventional ADT plus enzalutamide; other trials by industry and other cooperative groups will address that question. The inclusion of an arm with ADT and enzalutamide in STAMPEDE was therefore considered to be a duplication of effort and was not supported by the Trial Management Group.

The combination of enzalutamide and abiraterone is a novel approach and offers considerable promise in delaying progression – it therefore represents an attractive addition to the comparisons under investigation in STAMPEDE, and one that is unlikely to be replicated in other planned trials of this size.

3 SELECTION OF INSTITUTIONS AND INVESTIGATORS

Centres who wish to participate in the STAMPEDE trial should be registered with the Medical Research Council Clinical Trials Unit at University College London (MRC CTU at UCL) for this purpose. Before any patients are randomised the MRC CTU must receive a completed and signed Investigator Statement. The STAMPEDE investigator statement is signed by the Principal Investigator for that institution (Appendix M). R&D approval for the site, along with a fully-signed model agreement, are also required before recruitment can begin.

In addition and in compliance with the principles of GCP all institutions participating in the trial will complete a delegation log and forward this to the MRC CTU. Each person working on the STAMPEDE trial must sign off a section of this log indicating their responsibilities. The MRC CTU must be notified of any changes to trial personnel and/or their responsibilities. An up-to-date copy of this log must be stored in the Investigator Site file at the institution and also at the MRC CTU.

The Clinical Trial Authorisation (CTA) for the STAMPEDE trial requires that the Medicines and Healthcare Products Regulatory Agency (MHRA) be supplied with the names and addresses of all participating investigators/institutions. Trial staff at the MRC CTU will perform this task; hence, it is vital to receive full contact details for all investigators prior to their entering patients.

Finally, before a patient is entered into the trial written informed consent must be obtained. Approved patient information sheets and informed consent forms are supplied as templates.

Only a limited number of centres participated in the initial Pilot Phase of the original trial; this was to ensure that safety and feasibility data were collected expediently. Subsequent stages of the trial are open to any centre that wishes to participate and has fulfilled the requirements described above.

3.1 RADIOTHERAPY ACCREDITATION

The introduction of the RT comparison in v9.0 introduced the need for RTQA accreditation in sites giving radiotherapy. The details of RTQA accreditation is in Appendix K. However, centres that have been RTQA accredited for another multi-centre prostate radiotherapy trial in the UK (e.g. RADICALS or CHHIP) will be automatically granted STAMPEDE RTQA accreditation.

4 SELECTION OF PATIENTS

4.1 PATIENT INCLUSION CRITERIA

Patients must fulfil both of the criteria in **Section 4.1.1** **or** one criterion in **Section 4.1.2** **or** at least one criterion in **Section 4.1.3**. Additionally, all patients must fulfil the criteria in **Section 4.1.4**.

4.1.1 High-Risk Newly-Diagnosed Non-Metastatic Node-Negative Disease

Both:

- At least two of: Stage T3/4, PSA≥40ng/ml or Gleason sum score 8-10
- Intention to treat with radical radiotherapy (unless there is a contra-indication; exemption can be sought in advance of consent, after discussion with MRC CTU)

OR

4.1.2 Newly-Diagnosed Metastatic Or Node-Positive Disease

At least one of:

- Stage T_{any} N+ M0
- Stage T_{any} N_{any} M+

OR

4.1.3 Previously Treated With Radical Surgery And/Or Radiotherapy, Now Relapsing[1]

At least one of:

- PSA ≥4ng/ml and rising with doubling time less than 6 months
- PSA ≥20ng/ml
- N+
- M+

AND

4.1.4 For All Patients

I. Histologically confirmed prostate adenocarcinoma
II. Intention to treat with long-term androgen deprivation therapy
III. Fit for all protocol treatment[2] and follow-up, WHO performance status 0-2[3]
IV. Have completed the appropriate investigations prior to randomisation
V. Adequate haematological function: neutrophil count >$1.5x10^9$/l and platelets >$100x10^9$/l
VI. Estimated creatinine clearance >30ml/min
VII. Serum potassium ≥3.5mmol/L
VIII. Written informed consent
IX. Willing and expected to comply with follow-up schedule
X. Using effective contraceptive method if applicable

[1] Courses of hormone therapy for localised disease must have been completed at least 12 months previously and have been no longer than 12 months in duration. It can have been given as adjuvant or neoadjuvant therapy.
[2] Medical contraindications to the trial medications are given in **Appendix G**
[3] For WHO performance status definitions see **Appendix A**

4.2 PATIENT EXCLUSION CRITERIA[4]

Patients must not fulfil any of the criteria, below.

I. Prior systemic therapy for locally advanced or metastatic prostate cancer except as listed in **Section 4.1.3**
II. Metastatic brain disease or leptomeningeal disease
III. Abnormal liver functions consisting of any of the following:
   - Serum bilirubin ≥1.5 x ULN (except for patients with Gilbert's disease, for whom the upper limit of serum bilirubin is 51.3µmol/l or 3mg/dl)
   - Aspartate aminotransferase (AST) or alanine aminotransferase (ALT) ≥2.5 x ULN
IV. Any other previous or current malignant disease which, in the judgement of the responsible physician, is likely to interfere with STAMPEDE treatment or assessment
V. Patients with contra-indications to prednisolone, including active peptic ulceration or a history of gastrointestinal bleeding
VI. Patients with active inflammatory bowel disease
VII. Symptomatic peripheral neuropathy grade ≥2 (NCI CTC)[5]
VIII. Any surgery (e.g. TURP) performed within the past 4 weeks
IX. Patients with significant cardiovascular disease such that, in the investigator's opinion, the patient is unfit for any of the study treatments. This might include:
   - Severe/unstable angina
   - Myocardial infarction less than 6 months prior to randomisation
   - Arterial thrombotic events less than 6 months prior to randomisation
   - Clinically significant cardiac failure requiring treatment (NYHA II-IV)[6]
   - Cerebrovascular disease (e.g. stroke or transient ischaemic episode) less than 2 years prior to randomisation
   - Patients with uncontrolled hypertension defined as systolic BP greater or equal than 160 mmHg or diastolic BP greater or equal than 95 mmHg
X. Patients receiving treatment with drugs known to induce CYP3A4 (including phenytoin, carbamazepine, Phenobarbital)[7]
XI. Prior exposure to abiraterone
XII. Prior exposure to enzalutamide
XIII. Prior chemotherapy for prostate cancer
XIV. Prior therapy with zoledronic acid or other bisphosphonates other than treatment for hypercalcaemia or low bone density
XV. Prior exposure to policy of long-term hormone therapy before randomisation (unless as described in **Section 4.4.2**)
XVI. History of seizure including any febrile seizure, loss of consciousness, or transient ischaemic attack within 12 months of randomisation or any condition that may pre-dispose to seizure (e.g., prior stroke, brain arteriovenous malformation, head trauma with loss of consciousness requiring hospitalization)
XVII. Unexplained history of loss of consciousness within 12 months of randomisation
XVIII. Operation of heavy machinery during treatment

[4] The exclusion criteria for patients who have been on a Cox-2-inhibitor for 6+ months has been removed
[5] See **Appendix I** for common toxicity grading
[6] NYHA classifications can be found in **Appendix A**
[7] A full list is included in **Appendix G**

4.3 SELECTION CRITERIA FOR COMPARISON OF RESEARCH (M1) RT FOR METASTATIC DISEASE

All patients meeting criteria in **Section 4.1** and **4.2** are eligible for the trial, but not all can be allocated to the research (M1) radiotherapy arm. The selection criteria for this “RT to the prostate” comparison are:

- Newly-diagnosed prostate cancer
- Demonstrable M1 disease
- No contraindication to radiotherapy e.g. no previous pelvic radiotherapy and no history of inflammatory bowel disease
- No previous radical prostatectomy

Any patients meeting these criteria will have a chance to be allocated to Arm H.

4.4 SCREENING PROCEDURES

4.4.1 INVESTIGATIONS PRIOR TO RANDOMISATION

All patients should have the following examinations performed. The latest available scans should be used:

- CT or MRI of pelvis and abdomen
- Bone Scan (or equivalent e.g. whole body MRI)
- Chest X-ray (only if chest was not included in CT)
- ECG
- PSA Test

The following blood tests within 8 weeks (56 days) prior to randomisation:

- Testosterone (if available)
- Urea and Electrolytes
- Liver function tests
- Serum creatinine
- Serum corrected calcium
- Phosphates
- Magnesium
- Albumin
- Total cholesterol
- HDL cholesterol
- Systolic blood pressure
- Diastolic blood pressure
- Waist circumference measure

Patients who initially fail to meet the eligibility criteria can be re-screened at a later date.

Prior to randomisation:

- Check details of any prior treatments for prostate cancer
- Check any contraindications to radiotherapy

4.4.2 Androgen Deprivation Therapy Prior To Randomisation

It is preferable that patients are not started on hormones prior to randomisation. However, if androgen deprivation therapy has already started, the primary therapy should have not have started more than 12 weeks before randomisation, and the baseline PSA measurement must be taken before this was initiated (please report the latest PSA measurement taken before the start of androgen deprivation therapy).

Short periods of prior anti-androgens to cover tumour flare are allowed but will not be counted in the 12 week time period mentioned above; but a PSA measurement must be taken before this is initiated.

Note that long-term anti-androgen monotherapy is not permitted in the trial for newly recruited patients from version 8.0 (see **Section 6.1**); patients may change treatment to join the trial, provided that they have not had more than 12 weeks of androgen deprivation therapy prior to randomisation. Further details on hormone therapies allowed prior to randomisation are discussed in Appendix L.

Any relapsing patients treated with adjuvant or neo-adjuvant hormone therapy alongside their radical surgery or radiotherapy must have completed that period of hormone therapy at least 12 months before joining STAMPEDE and it must have been no longer than 12 months in duration .

Note that baseline testosterone measurements will not be required in patients who have already commenced hormone manipulation prior to randomisation.

4.4.3 Hypercalcaemia At Randomisation

For patients who are hypercalcaemic prior to randomisation and require treatment, it is recommended that they are treated with a bisphosphonate and that the treatment should be discontinued when they are stabilised.

4.4.4 NSAIDs And Cox-2 Inhibitors At Randomisation

Note: recruitment completed to both celecoxib-containing arms in Apr-2011 at the end of Activity Stage II

For patients who are currently on a Cox-2-inhibitor and who meet the inclusion criteria, please ensure that treatment is discontinued before randomisation. If the patient is allocated to an arm, which does not include celecoxib (arms A, B, C or E), it is advised that the Cox-2 be replaced with a suitable NSAID.

For patients who are taking an NSAID prior to randomisation and are allocated a celecoxib arm (Arm D or F), a clinical decision should be taken as to whether the patient should continue taking the NSAID alongside the celecoxib. This decision should take into account the risk of gastrointestinal problems, and consideration should be given to the co-administration of a proton pump inhibitor

4.4.5 Starting Trial Treatment

Trial treatment should be commenced as soon as possible after randomisation. Investigators should aim that this is at least within 4 weeks post randomisation and within 12 weeks of starting androgen Deprivation Therapy (see Section 6).

Radiotherapy for patients allocated to Arm H should be commenced within 4 weeks from randomisations and continued according to the predefined scheduled unless toxicity is reported. Any delays in starting research radiotherapy should be discussed with the STAMPEDE team and recorded as appropriate in the relevant CRF.

4.4.6 CONCOMITANT MEDICATIONS

All concomitant medications should be recorded including any vitamin and mineral supplements the patient is taking, regular consumption of NSAID and/or aspirin and use of other bisphosphonates (see **Section 4.3.1**). Of particular interest in this are herbal preparations such as PC-SPES, Prostasol, Saw Palmetto and St John's Wort. All concomitant medications should be continued throughout the trial unless the responsible clinician decides otherwise.

4.5 ADDITIONAL DETAILS FOR PATIENTS JOINING SUB-STUDIES

An additional droplet of blood must be taken if the patient has given their consent to participate in the DNA analysis sub-study.

The local pathologist will also be asked to give the tumour sample remaining after primary interrogation for tissue micro array analysis to be carried out, if the patient has given consent for his remaining samples to be used for further analyses. Full details of all sub-studies and instructions relating to the handling of the blood sample are given in **Section 17** and **Appendix D**.

5 RANDOMISATION AND ENROLMENT

Patients will be allocated to any of the open research arms for which they are suitable. Patients with non-metastatic disease or who have had previous local therapy to the prostate or who have a contraindication to radiotherapy will not be allocated to Arm H (see **Section 4.3**).

To enter a patient the randomisation form should be completed carefully and the MRC CTU contacted by phone:

RANDOMISATIONS
To randomise, call MRC CTU at UCL, Monday to Friday 0900-1700
excluding public holidays or dates when notice has been given by the CTU.
Tel: +44 (0) 20 7670 4777

A trial number and treatment will be allocated and given over the phone or by return fax. In addition, a letter confirming these details will be sent. The trial number will be the primary way in which the patient will be identified and should be used in all correspondence.

5.1 CO-ENROLMENT GUIDELINES

Ideally, patients should not be participating in any other clinical trial of prostate cancer treatment when they enter STAMPEDE and should not enter any other trials until the patient has had a failure-free survival (FFS) event reported. After this point, the patient may be entered into further, second-line treatment studies. The primary outcome measure of STAMPEDE is overall survival. Participation in post-progression studies should be reported on the Co-enrolment CRF.

Data sharing agreements with “down-stream” trials are encouraged to improve data quality in both trials and to reduce costs to both organisations.

6 TREATMENT OF PATIENTS

6.1 TRIAL TREATMENT

Patients will be randomised to the control arm (Arm A) or one of the research arms. All patients will receive androgen deprivation therapy (ADT) to achieve castration levels of testosterone. The method of ADT is a local choice but must be specified for each patient prior to randomisation. The recommended methods of ADT are given in **Section 6.1.1**. All trial treatments should commence as soon as practically possible after randomisation. Patients having a bilateral orchidectomy should commence any additional treatment within 12 weeks of the operation unless there is a strong clinical reason not to do so. Note that from protocol version 8.0 onwards, bicalutamide monotherapy is no longer permitted as a trial therapy for new patients (but patients may switch to a permitted therapy to join the trial – see **Section 4.3.2**).

6.2 ARM A: ADT ALONE OR ADT + STANDARD-OF-CARE (M0) RT (CONTROL ARM)

The standard of care for this patient group is **androgen deprivation therapy** (see **Section 6.2.1**). For some patient groups, this should now be supplemented with standard radiotherapy (see **Section 6.2.2**).

6.2.1 HORMONE THERAPY

The permitted methods of ADT are bilateral orchidectomy, LHRH analogues and LHRH antagonists. Anti-androgens alone are not permissible as hormone therapy for patients participating in STAMPEDE, but their use is recommended in the short-term to prevent tumour “flare” which may occur after commencing LHRH analogues. Anti-androgen prophylaxis of tumour flare is not required when using LHRH antagonists. At the time of randomisation, centres will be asked to specify the method of ADT for each patient. Other methods of ADT should be discussed with the Chief Investigator or the Trial Surgeon. The planned duration of ADT should be at least 2 years.

Bilateral orchidectomy: Operations should be performed by appropriately trained surgeons. A total or subcapsular orchidectomy may be performed.

LHRH agonists: LHRH agonists/analogues used according to local practice. The prophylactic use of anti-androgens to prevent tumour “flare” is recommended.

LHRH antagonists: LHRH antagonists used according to local practice. The use of prophylactic use of anti-androgens to prevent tumour “flare” is not necessary.

6.2.2 STANDARD-OF-CARE (M0) RT

N0M0 patients: Investigators should give standard radiotherapy (RT) to patients with node negative, non-metastatic disease (N0M0), in accordance with the data from the PR07 and SPCG trials. If there is an intention to omit radiotherapy (e.g contraindications) in patients with N0M0 disease this must be discussed with the Trials Office before consent. See **Section 6.6** for further details of radiotherapy administration.

N+M0 patients: the benefit of radiotherapy in this group is at present uncertain with no firm data to either support or refute its use. However, the PR07 trial included some node positive patients as

cross sectional imaging was not a part of the baseline assessment in this trial, which did include whole pelvis radiotherapy. For patients with node positive, non-metastatic disease, radiotherapy is therefore recommended in suitable cases. Investigators will be asked to state their intention with regards to planned radiotherapy in this group at randomisation. Intention to give radiotherapy (or not) for node positive patients must be stated at randomisation to ensure that there is no bias towards particular combinations of systemic therapy with radiotherapy.

Standard radiotherapy is not a core part of the trial, therefore we intend to collect minimal data about the radiotherapy administered. It is accepted that some patients will develop progressive disease before radiotherapy can be administered and if this occurs the reasons for non-delivery of treatment must be recorded on the radiotherapy form.

6.3 ARM B: ADT + ZOLEDRONIC ACID

Note: recruitment stopped to the zoledronic acid and docetaxel-containing arms in Mar-2013 at the end of Activity Stage IV.

Androgen deprivation therapy (+/- standard-of-care M0 RT) as described in **Section 6.2.1**.

Zoledronic Acid: 4mg 15min IV infusion every 3 weeks, for 6 treatments followed by zoledronic acid 4mg 15min IV infusion every 4 weeks up to a maximum of 2 years from the start of the treatment or until disease (including PSA) progression (see **Section 7.2**). Patients should also receive an oral supplement of 500mg calcium and 400IU vitamin D daily. These doses are available as a combination tablet. See **Section 6.6** for further information.

6.4 ARM C: ADT + DOCETAXEL

Note: recruitment stopped to the zoledronic acid and docetaxel-containing arms in Mar-2013 at the end of Activity Stage IV.

Androgen deprivation therapy (+/-standard-of-care M0 RT) as described in Section 6.2.1.

Docetaxel: 75mg/m2 Day 1 as 1hr IV infusion, plus prednisolone 5mg bid daily for 21 days. The cycle should be repeated every 3 weeks for a maximum of 6 cycles. The recommended administration schedule, anti-emetic regimen and dose modifications for docetaxel are given in Appendix F. See Section 6.2.2 for further information.

6.5 ARM D: ADT + CELECOXIB

Note: recruitment completed to both celecoxib-containing arms in Apr-2011 at the end of its Activity Stage II

Androgen deprivation therapy as described in Section 6.2.1.

Celecoxib 400mg bid until the sooner of 1 year or disease (including PSA) progression (see Section 7.2). See Section 6.2.3 for further information.

6.6 ARM E: ADT + DOCETAXEL + ZOLEDRONIC ACID

Note: recruitment stopped to the zoledronic acid and docetaxel-containing arms in Mar-2013 at the end of Activity Stage IV.

Androgen deprivation therapy (+/- standard-of-care M0 RT) as described in **Section 6.2.1**.

Docetaxel: 75mg/m2 Day 1 as 1hr IV infusion, plus prednisolone 5mg bid daily for 21 days. The cycle should be repeated every 3 weeks for a maximum of 6 cycles. The recommended administration schedule, anti-emetic regimen and dose modifications for docetaxel are given in Appendix F. See Section 6.4 for further information.

Zoledronic Acid: 4mg 15min IV infusion every 3 weeks, for 6 treatments followed by zoledronic acid 4mg 15min IV infusion every 4 weeks up to a maximum of 2 years from the start of the treatment or until disease (including PSA) progression (see **Section 7.2**). Patients should also receive an oral supplement of 500mg calcium and 400IU vitamin D daily. These doses are available as a combination tablet. See **Section 6.3** for further information.

Co-administration of docetaxel and zoledronic acid: Docetaxel 75mg/m2 Day 1 as 1hr IV infusion, plus prednisolone 5mg bid daily followed by zoledronic acid 4mg 15min IV infusion. There is evidence to suggest that the co-administration of docetaxel and zoledronic acid is sequence dependent.(42) Consequently, docetaxel should be administered before zoledronic acid

6.7 ARM F: ADT + ZOLEDRONIC ACID + CELECOXIB

Note: recruitment completed to both celecoxib-containing arms in Apr-2011 at the end of Activity Stage II

Androgen deprivation therapy as described in **Section 6.2.1**.

Zoledronic Acid 4mg 15min IV infusion every 3 weeks, for 6 treatments followed by zoledronic acid 4mg 15min IV infusion every 4 weeks up to a maximum of 2 years from the start of the treatment or until disease (including PSA) progression (see Section 7.2). Patients should also receive an oral supplement of 500mg calcium and 400IU vitamin D daily (Calcichew). These doses are available as a combination tablet. See **Section 6.3** for further information.

Celecoxib 400mg bid until the sooner of 1 year or disease (including PSA) progression (see Section 7.2). See Section 6.5 for further information.

6.8 ARM G: ADT + ABIRATERONE

Note: recruitment to the "abiraterone comparison" completed in January 2014. Please note that some patients will continue treatment until progression or up to a maximum of 2 years. Please see sections below for more information

Androgen deprivation therapy (+/- standard-of-care M0 RT) as described in **Section 6.2.1.**

Abiraterone will be administered as a single 1000mg daily oral dose (4 tablets to be taken together once a day) together with prednisolone or prednisone 5mg daily to prevent secondary mineralocorticoid excess. Abiraterone absorption is increased by food. The tablets should be taken at least 2 hours after food, swallowed whole with some water. No food should be eaten for 1 hour afterwards.

Prednisolone (prednisone in Switzerland) should be taken as a single dose with food in the morning.

In patients with M1 disease, treatment with abiraterone will continue from randomisation until clinical disease progression, consistent with the COU-AA-301 trial (35) i.e., abiraterone would be given for these patients until a composite of PSA progression (as defined in Appendix J), radiological progression (appearance of new lesions or progression of existing lesions) and clinical progression (defined as new cancer-related symptoms). It is accepted that these flexible criteria for stopping treatment with abiraterone are open to the investigator's interpretation and discretion. Patients might continue treatment beyond the first failure-free survival (FFS) event (see Table 1 in **Section 9.2**); the first FFS event must be reported as per the other arms.

In patients with N0M0 disease or N+M0 disease undergoing radical radiotherapy, treatment would continue for 2 years or disease progression as defined for M1 patients, whichever is the sooner. ADT can be discontinued in this group at 2 years at the discretion of the local investigator (see **Section 6.2.1**).

For patients with N+M0 disease not planned for radical radiotherapy, treatment will continue as for patients with M1 disease until disease progression. Trial treatment must stop if other systemic treatments are initiated (such as anti-androgen therapy for biochemical failure).

If a patient allocated to Arm G develops only biochemical failure, the responsible clinician might switch from abiraterone + prednisolone 5mg od to abiraterone and dexamethasone 0.5mg od. Trial treatment must stop if other systemic treatments are initiated at any time for disease progression control (including the addition or swapping of anti-androgens, chemotherapy etc).

See **Section 6.2.4 and 6.2.6** for further information for all groups.

6.9 ARM H: ADT + PROSTATE RADIOTHERAPY IN M1 PATIENTS

Androgen deprivation therapy as described in **Section 6.2.1**.

Radiotherapy will commence as soon as practicable and ideally within four weeks after randomization. Treatment will be according to the guidelines in **Section 6.11.5**. Two radiotherapy dose-fractionation schedules are permitted:
- 36Gy in 6 fractions of 6Gy, administered weekly over 6 consecutive weeks
- 55Gy in 20 fractions of 2.75Gy, administered daily, five days per week, over 4 consecutive weeks

Details of the recommendations for outlining, CTV and PTV are in **Section 6.11.5**.

6.10 ARM J: ADT + ABIRATERONE + PREDNISOLONE + ENZALUTAMIDE ADMINISTRATION

Androgen deprivation therapy (+/- standard-of-care M0 RT) as described in **Section 6.2.1**.

Abiraterone as described in **Section 6.8**.

Prednisolone as described in **Section 6.8**.

Enzalutamide will be administered as a 160mg oral dose (four capsules), taken together at the same time every day, with or without food.

In patients with M1 disease, treatment with both abiraterone and enzalutamide will continue from randomisation until clinical disease progression, consistent with the approach taken for abiraterone (see **Section 6.8**) i.e., abiraterone and enzalutamide would be given for these patients until a composite of PSA progression (as defined in **Appendix J**), radiological progression (appearance of new lesions or progression of existing lesions) and clinical progression (defined as new cancer-related symptoms). It is accepted that these flexible criteria for stopping treatment with abiraterone and enzalutamide are open to the investigator's interpretation and discretion. Patients may continue treatment beyond the first failure-free survival (FFS) event (see Table 1 in Section 9.2); the first FFS event must be reported as per the other arms.

In patients with N0M0 disease or N+M0 disease undergoing radical radiotherapy, treatment would continue for 2 years or disease progression as defined for M1 patients, whichever is the sooner. ADT can be discontinued in this group at 2 years at the discretion of the local investigator (see **Section 6.2.1**).

For patients with N+M0 disease not planned for radical radiotherapy, treatment will continue as for patients with M1 disease until disease progression.

Trial treatment must stop if other systemic treatments are initiated at any time for disease progression or control (including the addition or swapping of anti-androgens, chemotherapy etc).

See **Section 6.2.4** and **Section 6.2.6** for further information for all groups.

6.11 ADMINISTRATION AND DOSE MODIFICATIONS

6.11.1 ZOLEDRONIC ACID

Note: recruitment stopped to the zoledronic acid and docetaxel-containing arms in Mar-2013 at the end of Activity Stage IV.

Zoledronic acid will be administered by IV infusion in accordance with the instructions in the summary of product characteristics at a target dose of 4mg (adjusted for renal function, see below) every 3 weeks for the first 6 cycles and every 4 weeks, thereafter.

Serum Creatinine Measurements: Serum creatinine should be measured at baseline and within 48 hours prior to every administration of zoledronic acid. It is permissible to have serum creatinine levels measured on Fridays prior to the administration of zoledronic acid on the following Monday.

Serum Electrolytes and FBC: Serum electrolytes including calcium, phosphate and magnesium should also be measured prior to each infusion. FBC should be measured at least 3 monthly. Zoledronic acid should be discontinued if there is any evidence of hypersensitivity to the drug. In patients with mild to moderate renal impairment, lower doses of zoledronic acid are recommended according to standard dose reduction schedules for administration of this drug. In rare cases, zoledronic acid treatment has been associated with the development of osteonecrosis of the jaw, particularly following dental extractions. If a patient develops osteonecrosis of the jaw then the zoledronic acid should be immediately and permanently discontinued. For full details of zoledronic acid administration and does reductions see **Appendix F**. Contraindications, special precautions, interactions and side effects are listed in **Appendix G**.

6.11.2 Docetaxel

Note: recruitment stopped to the zoledronic acid and docetaxel-containing arms in Mar-2013 at the end of Activity Stage IV.

The use of docetaxel should be confined to units specialised in the administration of cytotoxic chemotherapy and it should only be administered under the supervision of a physician qualified in the use of anticancer chemotherapy.

Docetaxel will be administered by IV infusion in accordance with the instructions in the summary of product characteristics at a dose of 75mg/m2 (up to a maximum dose of 160mg) on Day 1 of the study treatment period and then every 3 weeks thereafter for a maximum of 6 doses. Patients with a body surface area (BSA) greater than 2.13m^2 should be dosed as though they have a BSA of 2.13m^2. No ideal weight should be used for BSA calculations. Prednisolone or prednisone 5mg bid will be given until completion of chemotherapy. Additional dexamethasone should be given pre- and post-docetaxel infusion to suppress allergic reactions.

Please note that liver function test (LFTs) should be carried out within a week before the first cycle of docetaxel if an anti-androgen has been administered. This is due to an increased risk of neutropenia associated with docetaxel use following anti-androgen administration. Treatment should be delayed if LFTs are abnormal.

For full details of premedication schedule, recommended anti-emetic regimen and dose modifications for docetaxel (see Appendix F). Contraindications, special precautions, interactions and side effects are listed in Appendix G.

Docetaxel in combination with prednisone or prednisolone is indicated for the treatment of patients with hormone refractory metastatic prostate cancer.(20, 21)

6.11.3 Celecoxib

Note: recruitment completed to both celecoxib-containing arms in Apr-2011 at the end of Activity Stage II. No new patients should be receiving this agent now within the trial.

Celecoxib should be administered in accordance with the instructions in the summary of product characteristics at a dose of 400mg bid orally. Rarely this drug is poorly tolerated and in this instance should be discontinued; particular care should be taken with patients with a history of gastrointestinal disease and patients with significant risk factors for cardiovascular events (see Appendix G). Patients with confirmed severe cardiovascular history should not be in STAMPEDE (see exclusion criteria, Section 4.2). Contraindications, special precautions, interactions and side effects are listed in Appendix G. Dose reductions are not anticipated.

6.11.4 ABIRATERONE OR ENZALUTAMIDE + ABIRATERONE

Abiraterone absorption is increased by food. The tablets should be taken at least 2 hours after food, swallowed whole with some water. No food should be eaten for 1 hour afterwards. Prednisolone (prednisone in Switzerland) should be taken as a single dose with food in the morning.

Enzalutamide can be taken with or without food.

If clinical symptoms or signs suggestive of hepatotoxicity develop, serum transaminases, in particular serum alanine aminotransferase (ALT) should be measured immediately. If a rise in transaminases or bilirubin is confirmed, action should be taken as detailed in **Appendix G**.

6.11.4.A Management of Specific Toxicities from Abiraterone

The safety monitoring and toxicity management plan described below takes into account AEs based on the reported clinical safety data of abiraterone.

Hypokalemia:
At the initial observation of **Grade 1** hypokalemia (serum potassium <3.5mM or below lower limit of normal range, but ≥3.0 mM), oral potassium supplement will be initiated. The dose of potassium supplement must be carefully titrated to maintain serum potassium at ≥3.5mM but ≤5.0mM. Any subject with low potassium while on study or a history of hypokalemia from a pre-existing or concurrent medical condition will undergo weekly or more frequent laboratory electrolyte evaluation. The investigator should consider maintaining potassium level at ≥4.0mM in these subjects.

If any subject experiences **Grade 3** hypokalemia (serum potassium levels <3.0mM–2.5mM, NCI CTCAE v4.0) or life-threatening hypokalemia with potassium levels <2.5mM (NCI CTCAE v4.0 hypokalemia grade 4), abiraterone will be discontinued and the subject will be hospitalized for intravenous potassium replacement and cardiac monitoring. After the return of serum potassium to normal, prednisolone will be discontinued but the patient can be maintained on enzalutamide.

Hypertension:
If **Grade 1-2:** Management per investigator with anti-hypertensive treatment.

If **Grade 3-4:** Withhold abiraterone. Adjust or add anti-hypertensive medications to mitigate the toxicity. When hypertension resolves to **Grade ≤1**, resume abiraterone at full dose with prednisolone 5mg bid. Enzalutamide can be continued.

Fluid retention/oedema:
If **Grade 1-2:** Increase prednisolone dose to 5mg bid.

If **Grade 3-4:** Withhold abiraterone. Consider addition of mineralocorticoid receptor antagonist eplerenone until resolution of symptoms. When fluid retention/oedema resolves to ≤Grade 1, resume abiraterone at full dose with prednisone 5mg bid. Enzalutamide can be continued. Abiraterone may be re-started when symptoms return to baseline or are equivalent to grade 1; if oedema does not resolve, abiraterone should not be re-started.

Abnormal liver function tests:
If **Grade 1** increases in AST, ALT or bilirubin occur (eg, increase in AST or ALT from ULN to 2.5 x ULN; increase in total bilirubin from ULN to 1.5 x ULN): the frequency of liver function test monitoring should be increased, if the investigator judges that the laboratory abnormalities are potentially related to study medication. No dose reduction is required.

If **Grade 2** increases in AST, ALT or bilirubin occur (eg, increase in AST or ALT to >2.5-5 x ULN; increase in total bilirubin from >1.5-3 x ULN): the frequency of liver function test monitoring should be increased to ≥once a week, if the investigator judges that the laboratory abnormalities are potentially related to study medication. No dose reduction is required.

If **Grade 3** or higher increases in AST, ALT, or bilirubin occur (eg, increase in AST or ALT to >5 x ULN; increase in total bilirubin to >3 x ULN), withhold abiraterone and all other concomitant medications that are potentially hepatotoxic. Frequent laboratory evaluations (at least once weekly) should be conducted until the liver function tests return to baseline value or grade 1. If study treatment resumption is considered for subjects who have experienced grade 3 increases in AST, ALT, or bilirubin, resume abiraterone with the first dose level reduction (3 tablets, 750 mg of study treatment) when grade 3 toxicities resolve to grade 1 or baseline.

If **Grade 4** increases in AST, ALT, or bilirubin occur (eg, increase in AST or ALT to >20 x ULN; increase in total bilirubin to >10 x ULN), subjects must discontinue abiraterone and enzalutamide immediately. They should be followed-up until resolution of abnormal liver function tests and then prednisone can be discontinued and the investigator can consider restarting enzalutamide.

6.11.4.B Management of Specific Toxicities from Prednisolone
Prednisolone or prednisone will be started at 5mg once daily, to prevent secondary mineralocorticoid excess. Prednisolone/prednisone dose increase of up to 10mg/day is permitted to manage mineralocorticoid-related toxicities (e.g., hypokalaemia, hypertension) which are refractory to standard management. Patients experiencing serious Cushing symptoms (e.g., weight gain, muscle loss) can decrease or discontinue (temporarily or permanently) steroids at the investigator's discretion. It should be noted that weight gain and muscle loss are also associated with androgen deprivation therapy.

6.11.4.C Management of Specific Toxicities from Enzalutamide
If any subject suffers a seizure whilst on treatment, enzalutamide should be discontinued. Abiraterone and prednisolone can be continued if the subject is not suffering from any abiraterone-specific toxicities.

Fatigue:
If **Grade 1-2:** No change in treatment.

If **Grade 3-4:** Withhold abiraterone and enzalutamide. Restarting of all treatments with a dose reduction of enzalutamide to 80mg/day can be considered when fatigue resolves.

6.11.4.D Management of Specific Toxicities from Combination of Enzalutamide + Abiraterone
To date no specific toxicities from the combination of abiraterone and enzalutamide have been described (N = 57 patients with mCRPC exposed for a median of 5.5 months).(56)

6.11.5 Research (M1) Prostate Radiotherapy

A treatment planning CT scan will be acquired with the patient supine, with empty rectum and comfortably full bladder.

Megavoltage equipment is required with effective photon energies ≥6MV. Minimum source-to-axis distance is 100cm. Field arrangement is at the clinician's discretion: acceptable treatment techniques (field arrangement) include a 3-field (anterior, right lateral, and left lateral), 4-field (anterior, posterior, right lateral, and left lateral), or 6-field (right and left anterior oblique, right and left posterior oblique, and right and left lateral) or equivalent coplanar technique with multi-leaf collimation for all fields to adequately protect normal structures.

The Clinical Target Volume (CTV) will consist of the prostate gland alone as visualized on the treatment-planning CT scan. The base of the seminal vesicles may also be included if they are macroscopically involved. Inclusion of pelvic lymph nodes in the CTV is not permitted. The Planning Target Volume will have a 0.8 cm margin posteriorly and 1.0 cm margin in all other directions around the CTV to account for prostate gland motion and uncertainty in daily treatment setup.

Critical normal tissues should be delineated on the treatment-planning CT scan by the treating clinician:

- Rectum – inferior limit: level of ischial tuberosities; superior limit: sigmoid flexure
- Bladder – entirety

Two radiotherapy dose-fractionation schedules are permitted. In either case, radiotherapy is prescribed such that the PTV receives at least 95% of the prescribed dose:

- 36Gy in 6 fractions of 6Gy, administered weekly over 6 consecutive weeks
- 55Gy in 20 fractions of 2.75Gy, administered daily, five days per week, over 4 consecutive weeks

Dose-volume objectives for each dose-fractionation schedule are shown in **Tables 2** and **3** below. Values have been calculated using the formula BED = D[1+d/(alpha-beta ratio)] assuming an alpha-beta ratio of 3 for rectum and bladder. These are provided for guidance only.

Portal imaging to verify accuracy of treatment delivery may be done according to the participating centre's local guidelines. Image-guidance technology (e.g., gold seed intraprostatic fiducial markers, cone-beam CT scanning) will be permitted according to clinician preference but is not required. Further illustration on the research radiotherapy arm schedule is shown in **Figure 9**.

Table 2: Rectal dose volume objectives

55Gy/20F	36Gy/6F	Max Vol (%)
52.5 Gy	33.3 Gy	50%
43.5 Gy	27.8 Gy	60%
26.1 Gy	16.7 Gy	80%

Table 3: Bladder dose-volume objectives

55Gy/20F	36Gy/6F	Max Vol (%)
52.2	33.3	25%
43.5	27.8	50%

Figure 9: Diagram for deciding approach to research (M1) RT to the prostate

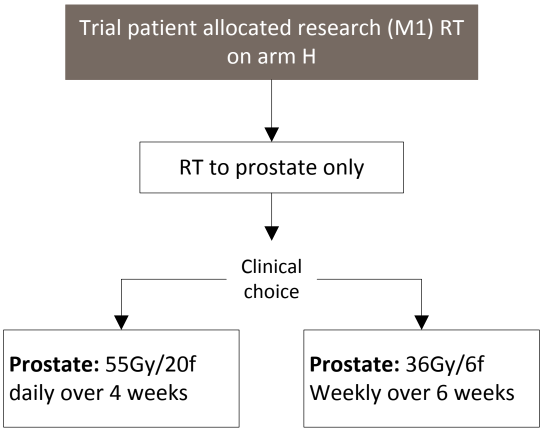

6.12 TRIAL PRODUCTS

Details of the procedures for obtaining the drugs within the trial, dispensing and disposal of unused drug are given in **Appendix E**.

Arrangements for free or discounted drugs are given in the Finance section (**Section 15**).

6.13 MEASURES OF COMPLIANCE/ADHERENCE

Date of treatment, dose, delays and reasons for delays or dose modifications of all study infusions (zoledronic acid and docetaxel) will be recorded. The estimated number of abiraterone tablets and enzalutamide capsules taken in a given time period will also be recorded as well as any dose reductions.

6.14 TREATMENT DATA COLLECTION

Data will be recorded on case report forms (CRFs); the top copy/original should be sent to the MRC CTU for data entry and a copy kept at the local centre. Up-to-date versions of all CRFs can be found on the trial website (http://www.stampedetrial.org/) and centres will be notified of any changes throughout the course of the trial. The type of data to be recorded is detailed in the Assessments and Procedures section (**Section 7**).

6.15 ADMINISTRATION OF STANDARD RADIOTHERAPY[8] TO NON-METASTATIC PATIENTS

6.15.1 TREATMENT DETAILS

Standard radiotherapy will be given to appropriate patients in each of the trial arms, following a period of neo-adjuvant ADT therapy, as is generally standard in UK practice. For patients receiving docetaxel, this period needs to be a minimum of 6 months after randomisation to ensure that

[8] **Note:** this text has been transferred into the protocol from the Appendices in version 8.0, and updated

chemotherapy is completed and toxicity resolved before RT begins. To ensure consistency of timing of administration of standard radiotherapy in all arms, this same 6 months period is recommended for all patients. For patients with negative nodes on axial imaging, clinicians may choose between irradiating prostate and seminal vesicles alone or including the pelvic nodes in addition. Additional staging tests such as pelvic node sampling may be considered in making this decision. Conformal or intensity modulated radiotherapy should be used in all patients. Where patients have good clinical evidence that nodes are free of tumour or patients for whom nodal radiotherapy is contra-indicated (e.g. significant bowel disease), treatment may be given to the prostate gland and seminal vesicles only. The recommended dose is 74Gy in 37 fractions to the prostate and seminal vesicles or the equivalent using hypofractionated schedules. These recommendations are summarised in **Figure 10**. Alternative dosing schedules are permitted but must be agreed with the STAMPEDE Trial Management Group.

6.15.1.A Standard-of-care RT Timing in M0 patients

Radiotherapy should be given around 6 to 9 months after randomisation in all trial arms and, if receiving docetaxel, the patient must have recovered from any docetaxel toxicity before RT can begin.

6.15.1.B Type Of standard-of-care RT in M0 patients

Conformal or intensity modulated radiotherapy.

6.15.1.C Standard Clinical Target Volume in M0 patients

- **CTV1:** Prostate plus seminal vesicles
- **CTV2:** (Node positive patients) Regional lymph nodes to include internal iliac and the inferior part of the common iliac nodes as used in EORTC trial 22961 (57)
- **PTV1:** CTV1 plus 10-15 mm according to local practice
- **PTV2:** CTV2 plus 10-20mm according to local practice

6.15.1.D Standard-of-care RT Dose in M0 patients

Prostate dose of 74Gy in 2Gy fractions or equivalent, with optional dose to the pelvic nodes of 46-50Gy in 2Gy fractions or equivalent using IMRT to deliver the treatment over 37 fractions, suggested dose is 55Gy in 37 fractions with IMRT. Higher doses may be considered if the department is experienced in using IMRT for nodal radiotherapy, particularly as data emerges from the PIVOTAL trial of nodal IMRT in high-risk node negative patients where a nodal dose of 60Gy in 37 fractions is being evaluated. Alternative schedules should be agreed with the STAMPEDE Trial Management Group.

Figure 10: Diagram for deciding recommended approach to standard-of-care (M0) RT in non-metastatic patients

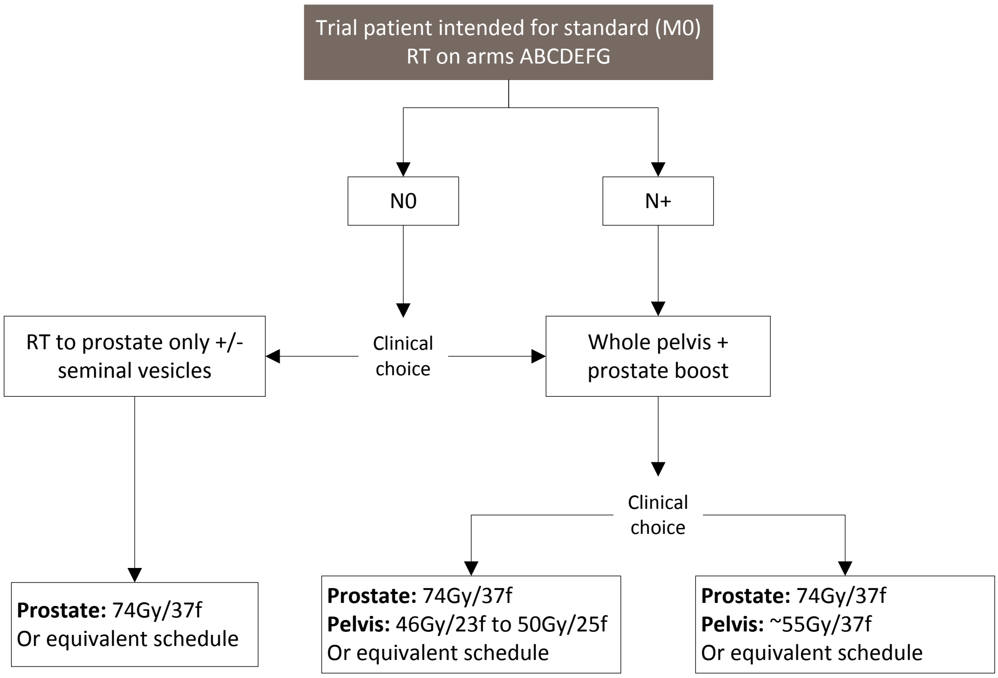

6.16 NON-TRIAL TREATMENT

6.16.1 MEDICATIONS PERMITTED

Any additional treatment that the responsible physician feels is appropriate is permitted.

6.16.2 DATA ON CONCOMITANT MEDICATION

All concomitant medication will be recorded on the baseline form prior to randomisation and on any subsequent Serious Adverse Event forms. This should include aspirin that may be taken on a regular basis for cardiovascular disease, the use of any Non-Steroidal Anti-inflammatory Drugs (NSAID) as well as any vitamin or mineral supplements the patient is taking.

7 ASSESSMENTS AND PROCEDURES

7.1 SCHEDULE FOR ASSESSMENTS

A detailed follow-up schedule is given in **Table 4, 5 and 6**.

7.1.1 PSA Measurements

All patients should have PSA measured pre-androgen deprivation therapy and at weeks 6, 12, 18 and 24 and every 12 weeks, thereafter, up to 2 years post randomisation. Following this, PSA should be measured every 6 months until 5 years and annually, thereafter. For patients who do not have a scheduled hospital visit, it would be acceptable for arrangements to be made for blood samples to be drawn in a GP's surgery.

7.1.2 Assessment Of Treatment Failure (Definition Of Progression)

It is not proposed to routinely assess patients for response. However, in order that objective progression can be assessed, it is necessary to have imaging taken at time of best response as judged by the treating clinician. All patients should have baseline radiological examinations as detailed in **Section 4.3.1**. In addition it is recommended that all patients should have scans or X-rays repeated at 24 weeks (and whenever clinically appropriate) if they were abnormal at baseline, particularly if they have a low PSA value on entry in to the trial making biochemical assessment of treatment failure difficult. The following events would constitute a disease progression and should be reported on a progression form:

- Biochemical failure – must be reported alongside castrate levels of testosterone if the patient has received intermittent ADT (see **Appendix J**).
- Local progression
- Lymph node progression
- Progression in distant metastases
- Development of new metastases

Please note that skeletal-related events (SREs) may be indicative of disease progression but can have other causes such as osteoporotic fracture. All SREs should be investigated further to establish whether or not the patient has progressed, in which case a progression form should be completed.

7.1.3 Additional Safety Assessment

Due to the risk of liver toxicity and secondary hyperaldosteronism with abiraterone, patients will require 2 weekly U+Es, LFTs and blood pressure measurement for the first 12 weeks. It is not proposed to collect the detail of these measurements unless results are abnormal; in this instance, they should be reported as AEs (on the next Follow-up CRFs) and as SAEs (see **Section 11**) if appropriate.

Medical review and PSA measurements follow the pattern in the control arm: visits at weeks 6, 12, 18 and 24 and every 12 weeks, thereafter, up to 2 years post randomisation. Following this, PSA should be measured every 6 months until 5 years and annually, thereafter. For patients who do not have a scheduled hospital visit, it would be acceptable for arrangements to be made for blood samples to be drawn either in a GP's surgery or in the patient's home.

7.1.4 Data Collection and Non-Administration Of Standard Radiotherapy

There are CRFs to be completed for patients receiving primary radiotherapy whether this is standard radiotherapy for M0 patients on any arm or prostate radiotherapy for Arm H patients. All radiotherapy and acute side effects details will be recorded on the Radiotherapy Form; any late side effects will be recorded on the Follow up form.

If it is decided not to give the planned radiotherapy (for example, due to early metastatic progression or patient refusal), this should be stated on the Standard Radiotherapy form together with the reason for non-administration of the treatment.

7.1.5 Data Collection Palliative Radiotherapy

For patients who receive palliative radiotherapy as part of first line treatment, a Palliative Radiotherapy CRF should be completed. Details of salvage RT for relapse and palliative treatment will be requested and completed only on the Progression Form.

7.1.6 Data Collection Research (M1) Radiotherapy

There are arm specific CRFs for patients randomised to arm H. Adverse events such as hip fractures, TURPs, skeletal-related events will be collected retrospectively via the Hospital Episode Statistics (HES) database.

7.1.7 Follow-Up Schedules

An individualised form with a follow-up schedule will be provided for each randomised patient. For patients who are receiving LHRH analogues, it is assumed that any additional treatment will commence within two weeks of randomisation. For patients who are due to have an orchidectomy it is recognised that surgery will have to be scheduled and the scheduling of any additional treatments may be affected by post-operative recovery. It is recommended that all patients who had abnormal radiological investigations at baseline or present with a low PSA on entry into the STAMPEDE trial should have them repeated 24 weeks after randomisation.

7.2 FOLLOW-UP

Every effort should be made to follow-up all patients who have been randomised. Patients should, if possible, remain under the care of an oncologist or urologist for the duration of the trial. If care of a patient is returned to the GP, it is the responsibility of the consultant who obtained the patient's consent to participate in the trial to ensure that the data collection forms are completed. If the patient moves from the local area, arrangements should be made for trial follow-up to be undertaken by their new local centre. Details of other participating centres can be obtained from the MRC CTU. The consent of patients should be obtained for their names to be flagged for survival information through national registries, for example NHS Information Centre/Office of National Statistics (ONS) in England/Wales and General Register Office in Scotland, Hospital Episode Statistics (HES). If the clinician moves, appropriate arrangements should be made to arrange for trial follow-up to continue at the centre.

Table 4: Summary of timing of case report forms

CASE REPORT FORMS	TIMING OF ASSESSMENT AND CRF
**Baseline**	
Bone Density Risk Factor	At randomisation
Randomisation	At randomisation
Baseline	At randomisation
Cardiovascular Assessment	At randomisation
Pathology	At randomisation. When pathology sample has been taken and sent to UCL laboratory.
**Treatment**	
Pre-18 Week Bisphosphonate	Treatment administered every 3. Form holds data for 2 cycles. Form to be sent after 2nd cycle given.
Post-18 Week Bisphosphonate Treatment	Treatment administered every 4. Form holds data for 3 cycles. Form to be sent after 3rd cycle given.
Docetaxel Treatment	Treatment administered every 3 weeks Form holds 2 cycles. Form to be sent after 2nd cycle given.
Abiraterone and Enzalutamide Treatment	Treatments administered daily; form to be sent at each follow up visit
RT detail	• When standard-of-care radiotherapy is completed or if planned RT is no longer to be given<br>• Arm H when research RT completed<br>• Arm A (M1) at 3 months
RT Acute Toxicity	For all patients who receive primary RT.
**Assessments**	
Follow-Up	Every 6 weeks for 6 months, then every 12 weeks until 2 years. Every 6 months until 5 years and annually thereafter. (See **Table 7** for more information.)
Palliative Radiotherapy	If applicable, when the palliative radiotherapy course is completed.
End of Treatment	When each treatment is completed (either at end of scheduled treatment or at early cessation of treatment).
Progression & Additional Treatment	At the first occurrence of each type of progression and whenever a patient that has progressed receives additional treatment.
Serious Adverse Event	Following any Serious Adverse Event
Skeletal-related Event	Whenever a patient experiences a skeletal-related event
Death	At Death
**Administration**	
Patient Transfer	When a patient is transferred to a different hospital for the administration of trial treatment and follow up
Co-enrolment	When a patient is co-enrolled in any other clinical trial. Please see **Section 5.1** for more information

Table 5: Data required on follow-up forms

TIMING OF FOLLOW-UP	PSA	EVIDENCE OF PROGRESSION	ANDROGEN DEPRIVATION THERAPY	TREATMENT	UNSCHEDULED VISITS	TOXICITIES
Before progression	✓	✓	✓	✓	✓	✓
After Progression	-	✓	✓	✓	✓	✓

Table 6: Schedule for completion of treatment and outcome forms by arm.

Timing From Randomisation			Treatment Forms			Outcome forms	
Years	Months	Weeks	Zol. acid	Abi and/or enza	RT	Follow-up^Ψ	QL + HE^¥
**6-Weekly**							
-	-	6	B,E,F (†)	G, J	-	All arms	All arms
-	-	12	B,E,F (†)	G, J	M1: A,H	All arms	All arms
-	-	18	B,E,F (†)	G, J	-	All arms	All arms
-	-	24	B,E,F (‡)	G, J	-	All arms	All arms
**12-Weekly**							
-	-	36	B,E,F (‡)	G, J	-	All arms	All arms
-	-	48	B,E,F (‡)	G, J	M0: A,B,C,E,G, J	All arms	All arms
-	-	60	B,E,F (‡)	G, J	-	All arms	All arms
-	-	72	B,E,F (‡)	G, J	-	All arms	All arms
-	-	84	B,E,F (‡)	G, J	-	All arms	All arms
-	-	86	B,E,F (‡)	G, J	-	All arms	All arms
**6-Monthly**							
2	24	104	B,E,F (‡)	G, J	-	All arms	All arms
	30	130	-	G, J	-	All arms	All arms
3	36	156	-	G, J	-	All arms	All arms
	42	182	-	G, J	-	All arms	All arms
4	48	208	-	G, J	-	All arms	All arms
	54	234	-	G, J	-	All arms	All arms
5	60	260	-	G, J	-	All arms	All arms
**Annual**							
6	-	-	-	G, J	-	All arms	All arms
7	-	-	-	G, J	-	All arms	All arms
Etc	-	-	-	G, J	-	All arms	All arms

Key:

A = ADT alone
B = ADT + zoledronic acid
C = ADT + docetaxel
D = ADT + celecoxib
E = ADT + zoledronic acid + docetaxel
F = ADT + zoledronic acid + celecoxib
G = ADT + abiraterone
H = ADT + M1 research RT to the prostate
J = ADT + enzalutamide + abiraterone

Notes:

Ψ See Table 6 for information required at follow-up
† Form records data for two cycles
‡ Form records data for three cycles
¥ 1st 700 patients and those recruited from protocol version 8.0 onwards only

Note: Radiotherapy, Late RT Toxicity, Palliative Radiotherapy Progression, SAE, End of Treatment, Co-enrolment and Death forms to be completed as required.

Note: Docetaxel forms are no longer shown on the table as all patients will have completed treatment with docetaxel

Note: recruitment completed to Arms D and F in April 2011; Arms B, C and E in March 2013; Arm G in January 2014

Note: Quality of Life Study is only for first 700 patients entered into the trial and those who were recruited after the implementation of version 8.0 of the protocol. MRC CTU will inform centres of which of their patients this applies to.

7.3 TRIAL CLOSURE

For the purpose of complying with UK Clinical Regulations introduced on May 2004, the trial will be considered 'closed' when the follow-up point for the primary analysis of the final comparison has been reached. However, further observational follow-up of all patients enrolled in the trial will continue until all randomised patients have died. This will initially be via the hospital, but in the longer term may employ national registers.

8 STOPPING OF TREATMENT OR FOLLOW UP

Patients should be given every encouragement to adhere to protocol treatment and follow-up schedule, in order to reduce bias. However, a patient has the right to withdraw consent for participation in any aspect of this trial at any time.

8.1 STOPPING RESEARCH INTERVENTIONS

A patient may stop trial treatment for the following reasons:

- Progression whilst on therapy (trial treatment must be discontinued in this instance). For patients randomised to Arm G, please refer to **Section 6.8** for criteria to stop treatment
- Unacceptable toxicity
- Intercurrent illness which prevents further treatment
- Withdrawal of consent for treatment
- Any alteration in the patient's condition which justifies the discontinuation of treatment in the clinician's opinion
- Intention to commence a new anti-cancer treatment due to evidence of relapse.

The reason should be recorded on the treatment and/or follow-up forms as well as the End of Treatment form. In the case of abiraterone, the disease event for stopping abiraterone may be after the first reportable failure-free survival event (see **Section 6.8**). Unless a patient states otherwise, it should be assumed that consent is given to continue to record trial data.

8.2 PATIENT TRANSFERS

For patients moving from the area, every effort should be made for the patient to be followed-up at another participating trial centre and for this trial centre to take over responsibility for the patient. To document the transfer process the main contact person at both the current and receiving hospitals should complete and sign the Patient Transfer Confirmation form. A fully completed form must be returned to the CTU prior to the patient transfer and ideally any data queries for the patient should be completed prior to transfer.

On receipt of the completed transfer form a member of the STAMPEDE team will confirm the database has been updated and request confirmation of the name of the patient's new Clinician. Photocopies of the following documents may then be sent to the new hospital to complete the transfer and copies must be also retained at the original site for monitoring purposes:

- Consent form
- Completed CRFs
- Any documentation relating to the patient's participation in STAMPEDE (patient names must be removed from any documentation).

8.3 WITHDRAWAL FROM THE TRIAL COMPLETELY

If a patient explicitly withdraws consent to have any data recorded their decision must be respected and the MRC CTU must be informed in writing. All communication surrounding the withdrawal

should be noted in the patient's records and no further STAMPEDE CRFs should be completed for that patient.

Early stopping of follow-up should not be undertaken lightly and the site must consider the implications for the trial and the patient in reaching such a decision.

Patients can change their minds about withdrawal at any time and re-consent to participate in the trial. Follow-up data should be collected only from the point of when consent was re-instated.

9 STATISTICAL CONSIDERATIONS

9.1 METHOD OF RANDOMISATION

Patients will be randomised centrally using a computerised algorithm developed and maintained by the MRC CTU. Randomisation will be performed using the method of minimisation over a number of clinically important stratification factors with an additional random element. To decrease determinability, the factors are not listed here but can be found in the Statistical Design Document.

Table 8 shows the allocation weighting for each arm by protocol version. The relative weighting within each pairwise comparison remains constant throughout.

9.1.1 To Version 7

From the outset, the trial had 1 control arm (A) and 5 research arms (B, C, D, E and F).

As the control arm is the comparator arm for all the research arms, twice as many patients were recruited to the control arm as to each of the original research arms as this is an efficient design. Therefore, the initial randomisation ratio will be A2:B1:C1:D1:E1:F1. From version 7.0, accrual to the celecoxib-containing arms was halted and the allocation ratio was A2:B1:C1:D0:E1:F0.

9.1.2 Version 8

From version 8.0, an additional research arm (G) was introduced. The allocation weighting for the additional Arm G is 2, meaning that as many patients are contemporaneously randomised to Arm G as the control Arm A: the randomisation ratio is 2:2 (equivalent to 1:1 control:abiraterone). This gave an overall allocation ratio of A2:B1:C1:D0:E1:F0:G2. When recruitment has been completed to the ongoing original research Arms B, C and E (which will be around 2 years before completion of accrual to arm G), the allocation ratio will be A2:B0:C0:E0:D0:F0:G2 (or A2:G2). This is more efficient for this comparison than the 2:1 allocation ratio employed for the original research arms because of the minimal co-recruitment period.

Version 9.0 introduced a RT comparison for men with newly-diagnosed metastatic disease which is irrelevant to a subset of men joining STAMPEDE. This could only be achieved by splitting the randomisation system so that newly-diagnosed patients with M1 disease and no contraindication to RT are randomised A2:B1:C1:D0:E1:F0:G2:H2 and other men are randomised A2:B1:C1:D0:E1:F0:G2:H0. Note that the allocation ratio for each pairwise comparison in unaffected, only the rate at which comparisons accrue.

9.1.3 Version 10 and 11

Version 10.0 followed the successful completion of recruitment to Arms B, C and E. Therefore, the allocation ratio will be A2:B0:C0:E0:D0:F0:G2 (or A2:G2) for M0 patients and A2:B0:C0:E0:D0:F0:G2:H2 for M1 radiotherapy arm patients (2A:2G:2H).The equal allocation ratio is suitable with fewer research arms open.

9.1.4 Version 12

Version 12.0 introduces a further allocation, Arm J: HT + abiraterone + enzalutamide. This allocation will be available to all patients. Accounting for Arm H still recruiting, this can only be achieved by keeping the randomisation system split so that newly-diagnosed patients with M1 disease and no contraindication to RT will be randomised A2:B0:C0:D0:E0:F0:G0:H2:J2 and other men will be

randomised A2:B0:C0:D0:E0:F0:G0:H0:J2. This can be simplified to equal allocation in these groups to A: H:J and A:J.

Table 7: Allocation to each arm by protocol version

PROTOCOL	NEWLY-DIAGNOSED M1 PATIENTS									OTHER PATIENTS								
VERSION	A	B	C	D	E	F	G	H	J	A	B	C	D	E	F	G	H	J
V1	2	1	1	1	1	1	-	-	-	2	1	1	1	1	1	-	-	-
V2	2	1	1	1	1	1	-	-	-	2	1	1	1	1	1	-	-	-
V3	2	1	1	1	1	1	-	-	-	2	1	1	1	1	1	-	-	-
V4	2	1	1	1	1	1	-	-	-	2	1	1	1	1	1	-	-	-
V5	2	1	1	1	1	1	-	-	-	2	1	1	1	1	1	-	-	-
V6	2	1	1	1	1	1	-	-	-	2	1	1	1	1	1	-	-	-
V7	2	1	1	0	1	0	-	-	-	2	1	1	0	1	0	-	-	-
V8	2	1	1	0	1	0	2	-	-	2	1	1	0	1	0	2	-	-
V9	2	1	1	0	1	0	2	2	-	2	1	1	0	1	0	2	2	-
V10	2	0	0	0	0	0	2	2	-	2	0	0	0	0	0	2	2	-
V11	2	0	0	0	0	0	2	2	-	2	0	0	0	0	0	2	2	-
V12	2	0	0	0	0	0	0	2	2	2	0	0	0	0	0	0	0	2

9.2 OUTCOME MEASURES

The overall, definitive primary outcome measure for the trial for each comparison is overall survival (all-cause mortality). The design of the trial is such that it is important to have additional intermediate outcome measures to assess each research arm as the trial progresses. These are listed in **Table 9**. The intermediate primary outcome measure is failure-free survival. The reasons for different emphases in each recruitment stage are explained in **Section 9.3**.

Table 8: Trial Outcome Measures by Comparison Stage

TRIALS STAGE	PRIMARY OUTCOME MEASURES	SECONDARY OUTCOME MEASURES
Pilot phase	Safety*	Feasibility
Activity Stage (AS) I-III	Failure-free survival (FFS)†	Overall survival (OS)<br><br>Toxicity<br>Skeletal-related events
Efficacy Stage (ES) IV	Overall survival	Quality of life<br><br>Cost effectiveness<br>Failure-free survival†<br>Toxicity<br>Skeletal-related events

*Based on toxicity
†Including biochemical failure (see **Appendix J**)

9.3 SAMPLE SIZE: PRINCIPLES AND ASSUMPTIONS

The overall design for this study is a multi-arm multi-stage, multi-centre randomised controlled trial. There are a number of stages for each research arm: a Pilot Phase, Activity Stages and a final Efficacy Stage. Full details of the methodology underlying the trial design are given by Royston et al. (58, 59) The sample size calculations were performed using the `stage2` (version 1.2.0, March 2002) and `stagen` (version 1.1.1, 18 May 2004) programs, both implemented in Stata (Stata Corp, TX) and updated using the later `nstage` program (version 1.0.3, 13-jun-2007; version 2.1.0, 28-jun-2009). (60)

The trial was designed under the assumptions in **Table 10**, and additionally, we assume a slightly higher proportion of non-metastatic than metastatic patients joining the trial such that the median FFS is two years and median OS four years for the whole cohort.

Table 9: Hazard ratio assumptions under null and alternative hypotheses

SIZE OF HR	PILOT	AS I-III	ES IV
Under null hypothesis (H0)	n/a	HR(FFS) = 1.00	HR(OS) = 1.00
Under alternative hypothesis (H1)	n/a	HR(FFS) = 0.75	HR(OS) = 0.75

The HR of 0.75 for any research arm relative to control would translate into an absolute improvement in FFS of 10%, from approximately 50% to 60% at two years and OS of 10%, from approximately 50% to 60% at four years. A beneficial difference of this size would be clinically worthwhile and, indeed, experience tells us it may be unrealistic to expect a larger difference. Therefore, we have adequately powered the trial to detect a HR of 0.75 for overall survival. This design gives 95% power at Activity Stages I-III and 90% power at Efficacy Stage IV for each comparison. Further details of the sample size calculations are summarised in **Sections 9.4** and **9.5** and detailed in a separate Statistical Design Documents which are available on request.

Note that, from version 8.0, standard-of-care M0 RT was introduced to the majority of patients with N0 M0 disease. This is likely to improve the outcomes for this group. Further agents are starting to be licensed for patients with castration-refractory disease which may also improve survival rates. Improved FFS rates would delay the intermediate analyses; improved survival rates would delay the definitive analyses. The Statistical Design Document includes models where median survival is estimated at 5, 6 and 7 years rather than just 3and 4 years. The trial is powered to detect a difference in relative improvement and the analyses will be performed when a pre-planned number of events has been reported in the control arm, rather than after a certain number of patients have been recruited or a certain amount of time elapsed. **Sections 9.4** and **9.5** provide more detail, including some variations on these assumptions.

Throughout recruitment to version 12.0, at least, the proportion of metastatic men joining the trial has been fairly constant, at around 60%. From version 9.0, we introduced an allocation, Arm H, only for men with M1 disease. This means that further comparisons for the whole patient group will have proportionately fewer metastatic patients and, therefore, fewer events at any given moment in time. This will affect contemporaneously-recruiting comparisons, such as the "enzalutamide + abiraterone comparison" introduced in the current version 12.0. Median survival may therefore be higher in that comparison, at around 7 years.

9.4 SAMPLE SIZE ISSUES AND TRIAL STAGES: ORIGINAL RESEARCH ARMS (B-F)

9.4.1 Pilot Phase: Original Research Arms (B-F)

It was anticipated that 210 patients would be recruited to the Pilot Phase from a limited number of centres over a one year period. Approximately 60 patients would be randomised to the control arm and 30 patients to each of the five research arms, each of which were assessed for safety and feasibility. If recruitment proved unfeasible or any of the research arms proved unsafe or not feasible to administer (e.g., poorly tolerated or unexpected toxicity) recruitment to these arms would have been discontinued. There were already considerable safety data on the use of docetaxel and zoledronic acid in patients with malignancies including prostate cancer, and on the use of Cox-2 inhibitors (including celecoxib), although mainly from patients with musculoskeletal disorders. There were fewer data on the combination arms, but it was thought very unlikely that any of the research arms would be discontinued during the Pilot Phase. When 210 patients had been on the trial for a minimum of 18 weeks, the Independent Data Monitoring Committee (IDMC) reviewed the data from the Pilot Phase and continued to the trial during this period as equipoise remained. Recruitment continued beyond this point. Safety data are assessed throughout the trial.

9.4.2 Activity Stages I-III: Original Research Arms (B-F)

In the sample size calculations, we assumed that all research arms successfully pass through the Pilot Phase to Activity Stage I and that patients would be recruited at a rate of approximately 500 per year. This was faster than in the Pilot Phase because the trial would recruit from additional centres, both in the UK and internationally. The analysis of Activity Stages I, II and III were planned for when around 113, 216 and 334 failure-free survival events had been observed in the control arm, respectively.

The Activity Stage analyses comprise pairwise comparisons of FFS between the control arm and each of the 5 research arms (i=B, C, D, E, F). Let HR_i(true) represent the hazard ratio (HR) of the ith research arm to the control arm, and HRi(observed) the observed value. Discontinuation of accrual

of further patients was considered for the ith research regimen at each of Activity Stages I-III according to the guidelines in **Table 11**.

Table 10: Guidelines for stopping accrual to the ith original research arm

Activity Stage	Number of control arm events	Consider discontinuation if HR_i(observed) is…
I	~113	>1.00
II	~216	>0.92
III	~334	>0.89

9.4.3 Efficacy Stage IV: Original Research Arms (B-F)

The analysis of Efficacy Stage IV for the original research arms will be performed when around 403 deaths have been observed in the control arm. This would give 90% power to detect the targeted hazard ratio of 0.75 at one-sided significance level of 0.025. The actual length of this stage, balancing continued accrual with just follow-up, depended on the number of arms passing through to further recruitment from Activity Stages I-III and the observed accrual and event rates.

9.4.4 Sample Size For Original Research Arms (B-F)

Assuming an accrual rate of 500 patients/year, between 2800 and 3600 patients were planned to be entered into the original research comparisons of the trial over a period of 5½ and 7 years. The exact number of patients to be entered depends on the observed accrual rate and the observed event rate, which is, in itself, dependent on the mix of patients joining the trial from the broad spectrum of eligibility. The primary analysis on overall survival requires around 403 deaths to be observed on the control arm. Accrual continued until the main analysis can be foreseen so that the overall duration of the comparisons would be as short as possible (longer accrual facilitates this) and so that few, if any, patients remain on treatment when the main results are released. The statistical team have monitored and projected the analysis timelines using the `artpep` command in Stata. Results should be due in 2015. Further information is available in the Statistical Master File.

9.5 SAMPLE SIZE ISSUES AND TRIAL STAGES: ADDITIONAL RESEARCH ARM G

9.5.1 Pilot Phase: Additional Research Arm G

A similar approach is being followed for the additional research Arm G, as detailed for the original research arms in **Section 9.4.1**. The IDMC reviewed safety data, in the context of data from the control arm, when the first 30 patients allocated to Arm G had been on trial for at least 18 weeks.

Furthermore, an additional review of safety was performed when 30 patients with newly-diagnosed non-metastatic disease allocated to Arm G had been on trial for at least 18 weeks.

Both of these milestones were successfully completed.

9.5.2 Activity Stages I-III: Additional Research Arm G

The same principles are applied to the new comparison as to the previous comparisons. The notable difference will be in the accrual rate to this comparison which is anticipated to be higher. There are two reasons for this. First, STAMPEDE started to recruit slowly in only a limited number of pilot sites. As more sites have been activated, including internationally, accrual has increased. At the time of

version 8.0 of the protocol, monthly accrual to the study was averaging around 60 patients/month (over 700 patients/year). Second, there is an equal allocation ratio for the abiraterone arm compared to the control arm. It is this different allocation ratio which means that the number of control arm events required to trigger the intermediate analyses is different for the assessment of abiraterone to the assessment of the original research arms. This is shown in **Table 11**.

Table 11: Guidelines for stopping accrual to the additional research Arm G

ACTIVITY STAGE	NUMBER OF CONTROL ARM EVENTS	CONSIDER DISCONTINUATION IF HR_G(OBSERVED) IS…
I	~75	>1.00
II	~142	>0.92
III	~221	>0.89

9.5.3 EFFICACY STAGE IV: ADDITIONAL RESEARCH ARM G

The analysis of Efficacy Stage IV for the additional comparison will be performed when around 267 deaths have been observed in the control arm. This would give 90% power to detect the targeted hazard ratio of 0.75 at a one-sided significance level of 0.025.

9.5.4 SAMPLE SIZE FOR ADDITIONAL RESEARCH ARM G

Up to around 1,800 patients will join the abiraterone comparison, with half allocated to the research arm. Consideration will be given to ceasing further randomisations to Arm G if it is not showing sufficient evidence of activity at the interim analyses, just as was done for research Arms B to F.

The original plan intended for accrual to be halted either when 1,500 patients had been recruited or after 3 years, whichever was the sooner, providing the accrual rate remained above 50 patients/months.

The total number of patients joining this comparison depends not just on the same issues as the original comparisons (notably, observed accrual and event rates), but also the length of time that the original research arms co-recruit alongside the additional research arm; it was originally assumed that this would be for approximately 1 year, but it was closer to 1.5 years. The sample size calculations and projected durations are fairly robust to changes in the length of co-recruitment with the original research arms and future co-recruitment with any further research arms which the Trial Management Group may introduce. Many scenarios are detailed in the Statistical Design Document.

In Sep-2013, the target sample size for the abiraterone comparison was increased from around 1,500 patients to around 1,800 patients, with the efficacy analysis still to be triggered by 267 control arm deaths. This increase in sample size was primarily because of an increase in the proportion of non-metastatic patients joining the comparison; this related to the activation of Arm H which only recruits patients with newly-diagnosed metastatic disease and thereby reduces the numbers of metastatic patients randomised to the abiraterone comparison. Non-metastatic patients have a lower event rate than the metastatic patients and maintaining the same overall sample size would lead to a delay in time to the primary analysis. The increase in sample size was achievable because recruitment rates to the trial had been substantially higher than 50 patients/month for the preceding 6 months.

9.6 SAMPLE SIZE ISSUES AND TRIAL STAGES: ADDITIONAL RESEARCH ARM H

9.6.1 Pilot Phase: Additional Research Arm H

A similar approach will be followed for the additional research Arm H as detailed for the original research arms in **Section 9.4.1**. The IDMC will review safety data, in the context of data from the control arm, when the first 30 patients allocated to arm H have been on trial for around six months.

9.6.2 Activity Stages I-III: Additional Research Arm H

The same principles will be applied to the new comparison as to the previous comparisons and an equal allocation ratio of control arm patients to patients allocated to Arm H will be employed, as for Arm G. The number of control arm events required to trigger the intermediate analyses will be the same as for the abiraterone comparison (see **Table 13**).

9.6.3 Efficacy Stage IV: Additional Research Arm H

The analysis of Efficacy Stage IV for the additional comparison will be performed when around 267 deaths have been observed in the control arm. This would give 90% power to detect the targeted hazard ratio of 0.75 at one-sided significance level of 0.025.

9.6.4 Sample Size For Additional Research Arm H

Consideration will be given to ceasing further randomisations to Arm H if it is not showing sufficient evidence of activity on the intermediate primary outcome measure (FFS), just as for the other research arms. This research comparison is relevant to around 60% of patients joining STAMPEDE. At the point of the scientific approval, accrual was averaging around 80 patients per month to the trial. If accrual to the trial was slower at 70 patients per month, then accrual to this comparison could be between 18 and 42 patients per month, depending on which other trial arms are open to recruitment at the time.

We are targeting a 25% relative improvement in overall survival following local radiotherapy to the prostate in this patient group. This is the same size of effect targeted with the other research arms in STAMPEDE. This relative improvement can be further justified in the light of MRC PR07 which demonstrated an improvement of this magnitude for adding radiotherapy to ADT in locally advanced disease, with a hazard ratio for overall survival of 0.77 (95% CI 0.61 to 0.98). In that trial, fewer than half of the deaths were from prostate cancer, whereas in newly-diagnosed metastatic patients nearly all men will die of their disease. Therefore, it is relevant to note the relative benefit of radiotherapy in PR07 in terms of prostate cancer-specific survival, where the hazard ratio was 0.54 (95% CI 0.27 to 0.78). Long-term survival-based data, with a median follow-up of ~10 years, were presented orally at the American Society of Clinical Oncology 2012 which confirmed these findings.(7)

We anticipate that around 1250 patients are required over 4 years to observe 267 control arm deaths after 5.25 years. In addition to the factors listed in **Section 2.1.2**, this assumes that (i) recruitment is constantly 70 pts/m to the trial overall, (ii) the original research arms stop accrual within 6 months after activation of the RT arm, (iii) the abiraterone arm stops accrual around 24 months after activation of the RT arm, and (iv) a further new research arm with an equal allocation ratio is introduced 18 months after activation of the RT arm.

With variations on these factors, between 1000 and 1400 patients are required over 2.75 to 4.50 years to address survival within 4.50 to 6.50 years. These sample scenarios will be documented in the Trial Master File.

All patients joining the trial will be starting long-term ADT for the first time. The focus of this comparison will be on the newly-diagnosed, metastatic patients (and no contraindications to RT), which is the largest subgroup of patients in the trial and the group of patients at highest risk of death from prostate cancer. Patients with non-metastatic disease will be excluded from this particular comparison as there are already randomised data demonstrating the survival benefit from radiotherapy in patients with locally advanced disease. Radiotherapy is now mandatory in node negative patients; it is also recommended in the node-positive, non-metastatic (N+ M0) group.

For the control arm of the whole trial, we constructed sample size scenarios median failure-free survival being 18, 24 or 30 months and constructed sample size scenarios around each of these options; the event rate would depend on the patient mix. We now know that around 60% of patients have M1 disease at trial entry and we have reported that FFS at 24 months is 51% across the whole of the control arm.(1)

For the sample size calculation for this new planned comparison, we have based our estimates on the subgroup of patients with newly-diagnosed M1 disease in the control arm. Therefore, we estimate median FFS to be 1 year and estimate that median overall survival will be 3.5 years.

9.7 SAMPLE SIZE ISSUES AND TRIAL STAGES: ADDITIONAL RESEARCH ARM J

9.7.1 Pilot Phase: Additional Research Arm J

A similar approach will be followed for the additional research Arm J as detailed for the original research arms in **Section 9.4.1**. The IDMC will first review safety data for this combination when the first 50 patients allocated to Arm J have been on trial around 6 weeks (i.e. to the first follow-up visit).

The IDMC will review safety data again when 50 patients are 6 months out from randomisation. Additional safety reviews will be performed if the IDMC raises any concerns over safety and routinely reviewed at regular intervals.

Direct comparison will be available with contemporaneously randomised patients on Arm A (hormones alone). Contextual data will be provided from Arm G (hormones plus abiraterone. Indicative safety data may also be available on the combination from other studies in CRPC.

9.7.2 Activity Stages I-II: Additional Research Arm J

The principles of intermediate analyses will be applied to this new comparison, but some of the details will be different. Owing to the expected accrual rate (>100 pts/m) and the expected slower event rate, only two activity stages are planned before accrual is completed. These are set out in **Table 11**.

9.7.3 Efficacy Stage III: Additional Research Arm J

The analysis of Efficacy Stage III for the additional comparison will be performed when around 267 deaths have been observed in the control arm. This would give 90% power to detect the targeted hazard ratio of 0.75 at a one-sided significance level of 0.025.

Table 12: Guidelines for stopping accrual to the additional research Arm J

Stage	Sig Level	Power	Targeted HR	Number of control arm events	Consider discontinuation if HR_J(observed) is…
I – Activity	0.40	95%	0.70	~66	>0.957
II – Activity	0.12	95%	0.70	~139	>0.869
III – Efficacy	0.025	90%	0.75	~267	>0.845

9.7.4 Sample Size For Additional Research Arm J

Consideration will be given to ceasing further randomisations to Arm J if it is not showing sufficient evidence of activity on the intermediate primary outcome measure (FFS), just as for the other research arms.

The patient mix for this comparison is likely to represent a more favourable prognosis in average than in the original research trial's other arms, due to concurrent recruitment of M1 but not M0 patients, to Arm H.

We anticipate that up to about 1800 patients are required within 3.5 years to observe ~267 control arm deaths within 6 years. This time will be dependent on the observed overall survival. The default scenario assumes that (i) recruitment is constantly 70pts/m to the trial overall, (ii) the M1/RT arm accrues throughout and (iii) a further new research arm with an equal allocation ratio is introduced 18 months after activation of Arm J.The stopping date for Arm G is no longer an assumption.

With variations on these factors (documented in a Statistical Design Document), 1800 patients are required over 2.5 to 3.5 years to address survival within 6 years. If accrual rates to the trial are at 150pts/m (as observed during Summer 2013), accrual of 1,800 patients to the comparison could be achieved within 2 years. These sample scenarios will be documented in the Trial Master File.

9.7.5 Further Sample Size Issues For Additional Research Arm J

Careful consideration will be given to the emerging data from the abiraterone comparison (Arms G vs A) and whether this arm continues to recruit throughout. It is anticipated that recruitment to this Arm J comparison will be completed *before* survival data emerge from the abiraterone comparison.

Indirect comparisons to understand the contribution from each agent may be possible if this research arm is demonstrably superior to the standard-of-care. These plans will be developed and documented elsewhere, but a higher number of patients will help with the power to the indirect comparison.

Figure 11: Schema of progress of STAMPEDE through the trial

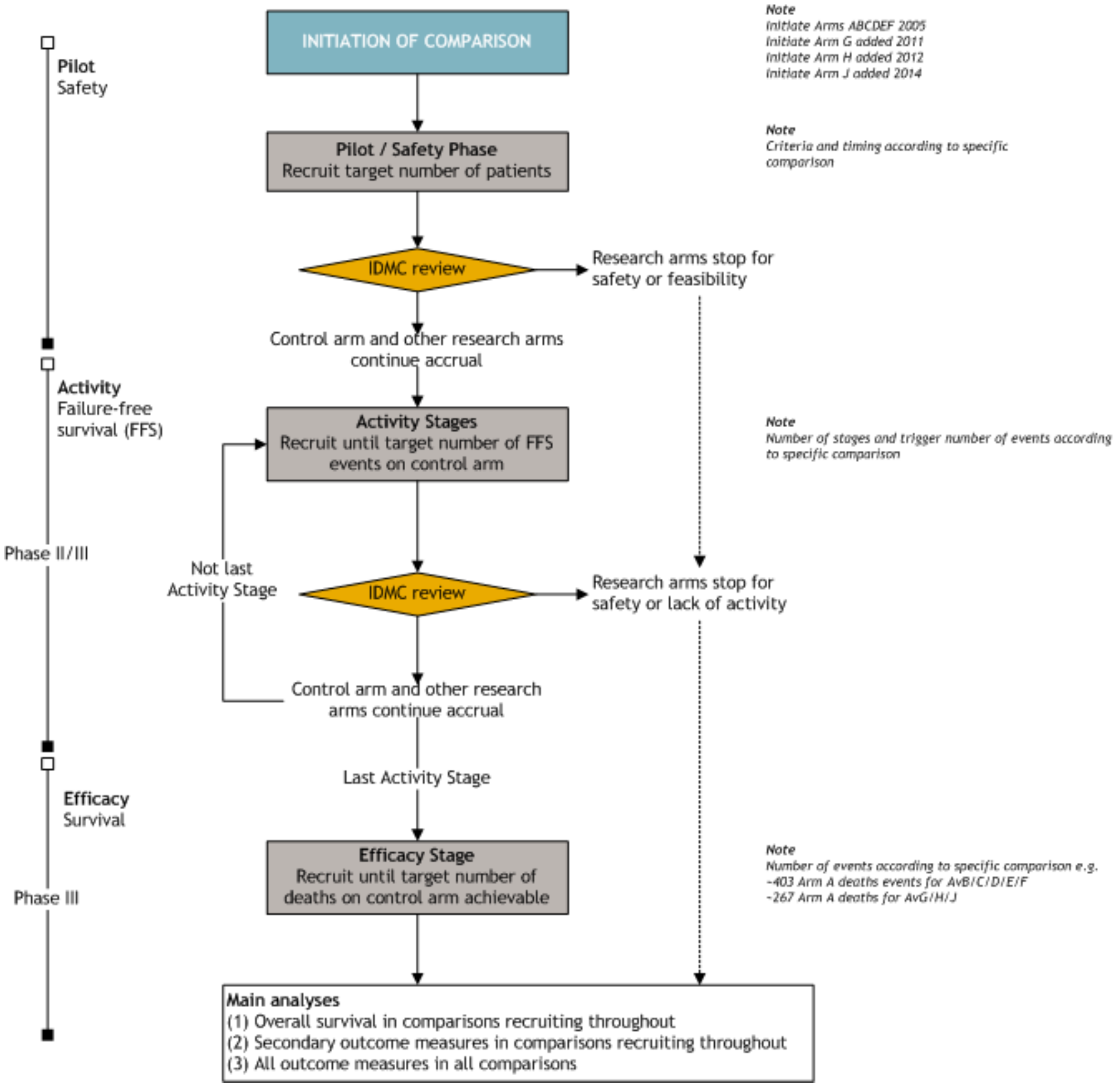

Key
FFS: Failure-free survival
HR: Hazard ratio
IDMC: Independent Data Monitoring Committee
Pts: Patients

Notes
Exact accrual depends on many factors including accrual rate, event rate and arms recruiting in each stage

9.8 FURTHER NOTES ON TRIAL DESIGN

9.8.1 OVERALL SAMPLE SIZE

Given the adaptive nature of the study, there is no formal overall sample size target, but the numbers of patients required for each comparison are detailed in **Sections 9.4** to **9.7**. It is expected that more than 7,000 patients will likely be recruited overall.

9.8.2 FACTORIAL DESIGN

We note here that we have not employed a factorial design in this trial because we anticipate the possibility of synergy between ADT, zoledronic acid and docetaxel and between ADT, zoledronic acid

and celecoxib. It would not be possible to assess any such interactions reliably in a factorial trial (see the Statistical Design Document for further details).

9.9 INTERIM MONITORING AND ANALYSES

The accumulating data will be reviewed at regular intervals (approximately annually) by an Independent Data Monitoring Committee (IDMC), including pre-specified formal intermediate analyses of activity data (see also **Section 16**). These analyses will be performed by the trial team at the MRC CTU. Only patients randomised contemporaneously will be included in the comparison of each research arm against control e.g. patients allocated to the control arm prior to version 12.0 will not contribute to the "enzalutamide + abiraterone comparison" (Arm A vs Arm J).

The IDMC will be asked to give advice on whether the accumulating data from the trial with the guidelines for discontinuation of accrual for the relevant Activity Stages, together with results from any other relevant trials, justifies continuing recruitment of further patients or further follow-up. A decision to discontinue recruitment, in all patients or in selected subgroups will be made only if the result is likely to convince a broad range of clinicians including those entering patients into the trial and the general clinical community. The intermediate stopping guidelines apply to the intermediate primary outcome measure. To stop accrual early for benefit in any comparison would require convincing data in terms of the definitive primary outcome measure, overall survival. For example, this could be p<0.001 as proposed by Haybittle-Peto.(61, 62) The use of such a guideline for stopping for benefit has a minimal impact on the operating characteristics.

If a decision is made to continue without change, the IDMC will advise on the frequency of future reviews of the data on the basis of accrual and event rates. The IDMC will make recommendations to the Trial Steering Committee (TSC, see **Section 16**) as to whether the trial should continue in its present form. While the trial is ongoing the accumulating data will generally remain confidential, unless the TSC and IDMC agree that the data should be made public.

9.10 OUTLINE ANALYSIS PLAN

Analyses will be performed on an intention-to-treat basis. The standard unadjusted log-rank approach will be applied to analyses of FFS and OS. The impact of potential confounders including the stratification factors used at randomisation will be considered in a Cox proportional hazard model. Flexible parametric models will be used to calculate the absolute differences between the arms to show treatment differences over time and to estimate restricted mean “survival” times (RMST). The estimated difference in restricted means survival time will be used preferentially to compare treatment arms if the proportional hazards assumptions required for hazard ratios cannot be supported. The χ^2 test or Mann-Whitney test will be implemented for categorical data comparisons, including toxicity, as appropriate. The primary outcome measures (see **Section 9.2**) will be considered for all arms of the trial at each phase, but the main emphasis will be placed on the comparison of the research arms that have continued to recruit throughout the trial.

9.10.1 PILOT / SAFETY PHASES

The Pilot Phase randomised patients between all the trial arms so that the results from these patients can be included in the main trial. Feasibility is considered in terms of the acceptability of the trial randomisation and reported toxicities and adherence to trial medication. Centres participating in the Pilot Phase for the original research arms were required to keep an anonymised log of all

patients assessed for trial eligibility (see protocol version 2.0) so that the number of patients who did not participate in the study and the number of eligible patients who choose to not participate in the study could be summarised (reasons for non-participation were collected where the patients was willing). The anonymised logs will not be needed for new research arms after v 8.0.

For the patients who are randomised, we shall describe the incidence of expected and unexpected severe toxicities and adverse events/reactions (see **Section 11**) to decide whether to continue with research arms beyond the Pilot Phase. As indicated above, we do not anticipate that recruitment to the research arms will be discontinued after the Pilot Phase, as there is considerable experience with zoledronic acid and docetaxel when combined with ADT, while Cox-2 inhibitors generally have a good toxicity profile. Although there are limited data on the combinations, we do not expect severe toxicity.

9.10.2 Activity And Efficacy Stages

The approach to analysis of these stages is summarised within the sample size calculations (see earlier subsections of Se**ction 9.4.3**). Each research arm will be compared in a pairwise fashion against the control arm.

Full details are available in the Statistical Analysis Plan.

10 MONITORING AND QUALITY ASSURANCE

10.1 MONITORING AT MRC CTU

Data provided to the MRC CTU will be checked for missing or unusual values (range checks) and consistency over time. If missing or questionable data are identified, staff at the MRC CTU will request that the data be clarified. The exact procedures for data clarification and the amendment of CRFs will be described in the trial specific SOPs and instructions will be sent to all STAMPEDE institutions as soon as they have been approved to participate in the trial. The MRC CTU will also send reminders for any overdue data.

10.2 DIRECT ACCESS TO DATA

Collaborating institutions should be aware that direct access to patient data by MRC CTU staff may be required for trial-related monitoring or audit. Patient consent for this will be obtained as part of the general trial consent process.

10.3 VISITS TO INVESTIGATOR SITES

A selection of institutions will be visited at least once during the course of the STAMPEDE trial. The MRC CTU will give the responsible investigator adequate notice of the monitoring visit to allow adequate time, space and staff for these visits. The standard operating procedures (SOP) for monitoring are available from the MRC CTU.

After the monitoring visit the monitor will complete a site visit report. This report will be circulated to the TMG for comment. Once the TMT have reviewed the report and agreed on any recommendations the monitor will finalise the report and send a copy to the Principal Investigator (PI) at the site. A copy will also be sent to the CI and TMG for the trial and another copy will be kept in the MRC CTU STAMPEDE trial master file.

10.4 CONFIDENTIALITY

All information collected during the course of the research will be kept strictly confidential. In addition, all procedures for handling, processing, storage and destruction of data are compliant with the Data Protection Act 1998. No individual patients will be identified when the results of the trial are published.

Patients will be asked for permission for information about their health status to be obtained from the Office of National Statistics (ONS) or via the NHS Strategic Tracing Service or similar by the Medical Research Council, if necessary. In addition, patients will be asked for permission to inform their GP of their involvement in the STAMPEDE trial.

11 SAFETY REPORTING

ICH GCP requires that both investigators and sponsors follow specific procedures when reporting adverse events/reactions in clinical trials. These procedures are described in this section of the protocol. Further information on the expected toxicities for the trial interventions (docetaxel, zoledronic acid, abiraterone and radiotherapy) can be found in **Appendix G**.

11.1 DEFINITIONS

The safety reporting definitions from ICH GCP apply in this trial protocol. These definitions are given in **Table 14**.

Table 13: Event Terms and Definitions

TERM	DEFINITION
Adverse Event (**AE**)	Any untoward medical occurrence in a patient or clinical trial subject to whom a medicinal product has been administered including occurrences which are not necessarily caused by or related to that product.
Adverse Reaction (**AR**)	Any untoward and unintended response to an investigational medicinal product related to any dose administered.
Unexpected Adverse Reaction (**UAR**)	An adverse reaction, the nature or severity of which is not consistent with the information about the medicinal product in question set out in the summary of product characteristics (or Investigator brochure) for that product.
Serious Adverse Event (**SAE**) or Serious Adverse Reaction (**SAR**) or Suspected Unexpected Serious Adverse Reaction (**SUSAR**)	Respectively any adverse event, adverse reaction or unexpected adverse reaction that:<br>• results in death<br>• is life-threatening*<br>• requires hospitalisation or prolongation of existing hospitalisation**<br>• results in persistent or significant disability or incapacity<br>• consists of a congenital anomaly or birth defect<br>• Other important medical condition***

Clarifications and Exceptions

*The term 'life-threatening' in the definition of 'serious' refers to an event in which the patient was at risk of death at the time of the event; it does not refer to an event which hypothetically might have caused death if it were more severe.

**Hospitalisation is defined as an inpatient admission, regardless of length of stay, even if the hospitalisation is a precautionary measure for continued observation. Hospitalisations for a pre-existing condition (including elective procedures that have not worsened) do not constitute an SAE.

***Medical judgement should be exercised in deciding whether an AE/AR is serious in other situations. Important AE/ARs that are not immediately life-threatening or do not result in death or hospitalisation but may jeopardise the subject or

may require intervention to prevent one of the other outcomes listed in the definition above, should also be considered serious.

Pregnancy occurring in a STAMPEDE patient's partner during the patient's participation in the trial, must be reported to the MRC CTU within the same timelines as an SAE and classified as an 'other important medical condition' on the SAE form. The outcome of a pregnancy should be followed up carefully and any abnormal outcome to the mother or child should be reported.

11.1.1 Trial-Specific Exemptions

Disease progression or death as a result of disease progression are not considered to be SAEs and should be reported on the STAMPEDE Progression Form or Death Form.

The following situations that fulfil the definition of an SAE are excluded from expedited notification on an SAE form and should be reported only on the STAMPEDE follow-up form:

- Elective hospitalisation and surgery for treatment of locally advanced or metastatic prostate cancer or its complications
- Elective hospitalisation to simplify treatment or procedures
- Elective hospitalisation for pre-existing conditions that have not been exacerbated by trial treatment

11.2 INSTITUTION/INVESTIGATOR RESPONSIBILITIES

All non-serious AEs/ARs, whether expected or not, should be recorded in the toxicity (symptoms) section of the Follow-up CRF and sent to the MRC CTU within one month of the form being due. SAEs/SARs should be notified to the MRC CTU as described below.

The severity (i.e. intensity) of all AEs/ARs (serious and non-serious) in this trial should be should be graded using Common Terminology Criteria for Adverse Events (CTCAE) v3.0 (ctep.cancer.gov/reporting/index.html). A flowchart is given in **Appendix I** to help explain the notification procedures. Any questions concerning this process should be directed to the MRC CTU in the first instance.

11.2.1 Investigator Assessment

11.2.1.A Seriousness

When an AE/AR occurs the investigator responsible for the care of the patient must first assess whether the event is serious using the definitions given in **Table 14**. If the event is serious and not exempt from expedited reporting, then an SAE form must be completed and the MRC CTU notified.

11.2.1.B Causality

The Investigator must assess the causality of all serious events/reactions in relation to the trial therapy using the definitions in **Table 15**. There are 5 categories: unrelated, unlikely, possible, probable and definitely related. If the causality assessment is unrelated or unlikely to be related the event is classified as a SAE. If the causality is assessed as either possible, probable or definitely related then the event is classified as a SAR.

Table 14: Assigning type of SAE through causality

RELATIONSHIP	DESCRIPTION	EVENT TYPE
Unrelated	There is no evidence of any causal relationship	Unrelated SAE
Unlikely	There is little evidence to suggest there is a causal relationship (e.g. the event did not occur within a reasonable time after administration of the trial medication). There is another reasonable explanation for the event (e.g. the patient's clinical condition, other concomitant treatment).	Unrelated SAE
Possible	There is some evidence to suggest a causal relationship (e.g. because the event occurs within a reasonable time after administration of the trial medication). However, the influence of other factors may have contributed to the event (e.g. the patient's clinical condition, other concomitant treatments).	SAR
Probable	There is evidence to suggest a causal relationship and the influence of other factors is unlikely.	SAR
Definitely	There is clear evidence to suggest a causal relationship and other possible contributing factors can be ruled out.	SAR

11.2.1.C Expectedness

If the event is a SAR the Investigator must assess the expectedness of the event. Please see Appendix G (Table G.2) for a list of expected toxicities associated with the drugs being used in this trial. If a SAR is assessed as being unexpected it becomes a SUSAR.

11.2.1.D Notification

Investigators must notify the MRC CTU of all SAEs occurring from the time of randomisation until 30 days after the last protocol treatment administration. Similarly, SAEs occurring in patients randomised to Arm A must be reported until 2 months after last injection or progression (whichever is sooner). SARs and SUSARs must be notified to the MRC CTU indefinitely (i.e. no matter when they occur after randomisation).

11.2.2 NOTIFICATION PROCEDURE

The SAE form must be completed by the Investigator (consultant named on the signature list and delegation of responsibilities log who is responsible for the patient's care), with due care being paid to the grading, causality and expectedness of the event as outlined above. In the absence of the responsible investigator the form should be completed and signed by a member of the site trial team. The responsible investigator should subsequently check the SAE form, make changes as appropriate, sign and then re-fax to the MRC CTU as soon as possible. The initial report shall be followed by detailed, written reports as appropriate.

Send the SAE form by fax to the MRC CTU. Fax Number: + 44 (0) 20 7670 4818. The STAMPEDE trial team will confirm receipt of the SAE report to the main point of contact via email

Follow-up: Patients must be followed-up until clinical recovery is complete and laboratory results have returned to normal or baseline, or until the event has stabilised. Follow-up should continue after completion of protocol treatment if necessary. Follow-up information should be noted on a

further SAE form by ticking the box marked ‘follow-up’ and faxing to the MRC CTU as information becomes available. Extra, annotated information and/or copies of test results may be provided separately. The patient must be identified by trial number, date of birth and initials only. The patient’s name should not be used on any correspondence.

11.3 MRC CTU RESPONSIBILITIES

Medically qualified staff at the MRC CTU and/or the Chief Investigator (or a medically qualified delegate) will review all SAE reports received. The causality assessment given by the local Investigator at the hospital cannot be overruled and in the case of disagreement, both opinions will be provided in any subsequent reports.

The MRC CTU is undertaking the duties of trial sponsor and is responsible for the reporting of SUSARs and other SARs to the regulatory authorities (MHRA and competent authorities of other European member states and any other countries in which the trial is taking place) and the research ethics committees as appropriate.

The MRC CTU will also keep all investigators informed of any safety issues that arise during the course of the trial.

SAE REPORTING
Fax to 020 7670 4818 within 24 hours of becoming aware of the event

12 ETHICAL CONSIDERATIONS AND APPROVAL

12.1 ETHICAL CONSIDERATIONS

This is a randomised trial therefore neither the patients nor their physicians will be able to choose the patients' treatment. Treatment will be allocated randomly using a computer-based algorithm. This is to ensure that the groups of patients receiving each of the different treatments are similar.

Androgen deprivation therapy alone is the standard treatment for these forms of prostate cancer. Patients will be randomised to one or two of the newer treatments in combination with hormone treatment. The trial has employed an unequal allocation ratio for some comparison to maximise efficiency; this was explained in detail in the patient information sheet.

The newer combined treatment options are being assessed in a detailed and systematic fashion in this trial. There is some evidence to suggest that the newer treatment options may have advantages over standard treatment (androgen deprivation therapy) alone with regards clinical outcome, but this is not confirmed and toxicity may be increased. This trial will follow a large group of men who have been randomly allocated to either the standard treatment (androgen deprivation therapy alone) or the newer combined treatment options in order to measure the benefits of the new treatments. The patients will also be followed-up for toxicity and safety issues, so that any benefits can be weighed against any negative aspects.

Patients participating in the trial will have some additional hospital visits and some extra blood samples taken compared to patients who are not participating in the trial, with the amount varying according to the allocated treatment. Sometimes the blood samples can be taken when the patient is attending hospital for treatment, anyway. On some of the trial arms, the patient may have to make additional visits to the hospital for the blood sample to be taken, although in some cases it may be possible for the blood sample to be taken in the GP's surgery. The additional visits and blood samples are to ensure that follow-up of patients is comparable in all the treatment groups. The blood samples will also be used for genetic and serum marker studies, where this information will be considered with clinical data. Blood samples will be link-anonymised. There will be no feedback to individual patients.

If new information emerges during the course of the trial which may affect the treatment or follow-up of patients who have joined the trial, information will be provided through by the trial team to all Principal Investigators. PIs have therefore the duty to inform patients in their care of any new information emerged using any appropriate channel (e.g. letter, communication at follow up clinic, etc).

12.2 ETHICAL APPROVAL

The protocol has a Favourable Opinion from an appropriate Research Ethics Committee, according to national guidelines. Additionally, each site must also obtain management permission for research (R&D approval) from the relevant host organisations before patients can be entered into the trial. The patient's informed consent to participate in the trial should be obtained after a full explanation has been given of the treatment options, including the conventional and generally accepted methods of treatment. Patient information sheets and patient consent forms are given in **Appendix B**.

The right of the patient to refuse to participate in the trial without giving reasons must be respected. After the patient has entered the trial, the clinician must remain free to give alternative treatment to that specified in the protocol, at any stage, if he feels it to be in the best interest of the patient. However, the reason for doing so should be recorded and the patient will remain within the trial for the purpose of follow-up and data analysis according to the treatment option to which he has been allocated. Similarly, the patient must remain free to withdraw at any time from the protocol treatment without giving reasons and without prejudicing his further treatment.

A statement of MRC policy on ethical considerations in clinical trials of cancer therapy, including the question of informed consent, is available from the MRC Head Office web site (http://www.mrc.ac.uk).

13 REGULATORY APPROVAL

This trial has been approved in the UK by the MHRA and will be conducted under a CTA (Ref: 00316/0026/001-0001) in the UK.

The trial has been approved in Switzerland by Swissmedic (Ref: 2009 DR 3235).

14 INDEMNITY

University College London holds insurance against claims from participants for injury caused by their participation in this clinical trial. Participants may be able to claim compensation if they can prove that UCL has been negligent. However, as this clinical trial is being carried out in a hospital, the hospital continues to have a duty of care to the participant of the clinical trial. University College London does not accept liability for any breach in the hospital's duty of care, or any negligence on the part of hospital employees. This applies whether the hospital is an NHS Trust or otherwise.

Participants may also be able to claim compensation for injury caused by participation in this clinical trial without the need to prove negligence on the part of University College London or another party. Participants who sustain injury and wish to make a claim for compensation should do so in writing in the first instance to the Chief Investigator, who will pass the claim to the managing organisation's Insurers, via the managing organisation's office.
Hospitals selected to participate in this clinical trial must provide clinical negligence insurance cover for harm caused by their employees and a copy of the relevant insurance policy or summary can be provided on request.

15 FINANCE

STAMPEDE is funded by the Clinical Trials Advisory Awards Committee (CTAAC) on behalf of Cancer Research UK; it is also funded by the MRC through the MRC Clinical Trials Unit. The trial has National Cancer Research Network (NCRN) approval and, therefore, local NCRN funds may be available at each centre to support entry of patients into this trial.

Zoledronic acid is manufactured by Novartis. Novartis have agreed to provide an educational grant to support the conduct of this study. Novartis have also agreed to supply the study drug, zoledronic acid free of charge for patients participating in the study.

Docetaxel is manufactured by Sanofi-Aventis Pharma. They have agreed to supply the study drug, docetaxel at a discounted rate for patients that are participating in the trial and to provide an educational grant to support the conduct of the study. The Department of Health has agreed to provide a central subvention as follow: £1,787 per patient randomised to Arms C and E of the trial and prescribed docetaxel. This amount is payable in respect of a hospital trust randomising more than 3 patients. For more details contact the STAMPEDE Trial Manager.

Celecoxib is manufactured by Pfizer. They agreed to supply free drug and to provide funds to distribute drug to participating sites.

Abiraterone is manufactured by Janssen Pharma PV (pharmaceutical companies of Johnson & Johnson). They have agreed to provide free drug and funds to distribute drug to participating sites and to help support the conduct and management of the trial.

Enzalutamide is manufactured by Astellas Pharma. They have agreed to provide free drug and funds to distribute drug to participating sites and to help support the conduct and management of the trial.

16 TRIAL COMMITTEES

16.1 TRIAL MANAGEMENT GROUP (TMG)

A Trial Management Group (TMG) has been formed comprising the Chief Investigator, other co-investigators and members of the MRC CTU. The membership of the TMG may be expanded if other groups of trialists wish to participate. It will also be amended during the trial if other circumstances require e.g. retirement.

The TMG will be responsible for the day-to-day running and management of the trial and will meet by teleconference at least 3 monthly and in person as needed. The TMG members are detailed in **Appendix K**.

Further details of TMG functioning are provided in the TMG charter (available on request).

16.2 TRIAL STEERING COMMITTEE (TSC)

A Trial Steering Committee (TSC) has been formed to provide overall supervision for the trial and provide advice through its independent chair. The ultimate decision for the continuation of the trial lies with the TSC. The TSC will meet regularly.

Further details of TSC functioning are provided in the TSC charter (available on request).

16.3 INDEPENDENT DATA MONITORING COMMITTEE (IDMC)

An Independent Data Monitoring Committee (IDMC) has been formed. The IDMC will be the only group who sees the confidential, accumulating data to the trial. Reports to the IDMC will be produced by the MRC CTU. The IDMC will meet within 6 months of the trial opening with the frequency of meetings dictated by the IDMC. The IDMC will consider data in accordance with the analysis plan (see **Section 9.5**) and will be advisory to the TSC. The IDMC can recommend premature closure or reporting of the trial, or that recruitment to any research arm be discontinued.

From version 8.0 onwards, any recommendation from the IDMC to stop recruitment to one or more trial arms will be acted upon immediately, pending ratification from the TSC. As this period between meetings should be very short, sites would not be notified until after the TSC have made a decision. IDMC recommendations based on emerging safety issues would be discussed with sites promptly.

Further details of IDMC functioning and the procedures for interim analysis and monitoring are provided in the IDMC charter (available on request).

Figure 12: Diagram of relationships between trial committees

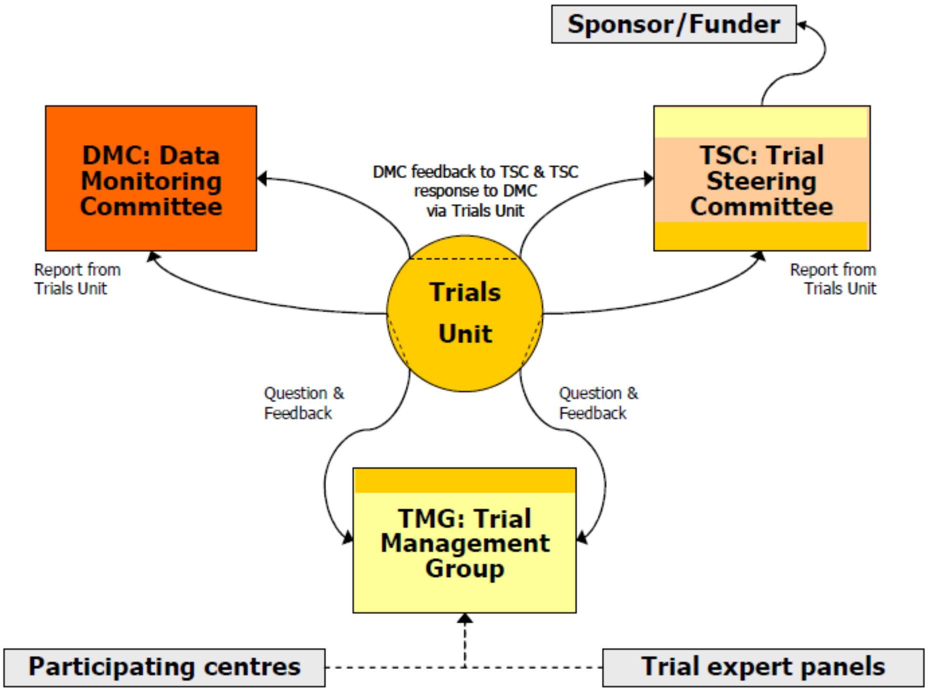

17 ANCILLARY STUDIES

17.1 QUALITY OF LIFE

A quality of life (QL) study is being performed to assess the impact of each treatment arm on the quality of patient's lives and participation in this study was limited to the first 700 patients recruited (this was reached in September 2008) patients. The QL study re-opened from the implementation of version 8.0 of the protocol. The EORTC QLQ-C30 with the prostate-specific module QLQ PR25 will be used. Key items for assessment are pain reduction for patients with metastatic disease and urinary symptoms for patients with locally advanced disease. In addition specific hypotheses will be generated for each of the research arms. The EuroQol (EQ-5D) (63) will be used in the study as a generic measure of health-related quality of life which can be linked to public preferences. These data will be used to calculate quality-adjusted life-years as part of the economic evaluation (see **Section 17.2**). Patients who were recruited into the QL study, should continue on the study throughout the trial. Questionnaires should be self-administered, although it is recommended that a key person (e.g. research nurse) at each centre be responsible for the data collection to optimise compliance and completeness of the data.

The QL and the HE questionnaires should be completed without conferring with friends or relatives and all questions should be answered even if the patient feels them to be irrelevant.

The responsible person should check each questionnaire for its completeness, ensuring that the correct date of completion and patient identifiers are present. The research nurse should approach patients at appropriate clinical visits to complete a questionnaire. If no clinical visit is scheduled for the patient (with a window of 4 weeks around the expected date) the nurse should organise the completion of the questionnaire, by post or by a visit to the patient at home (or in a hospice).

17.2 HEALTH ECONOMICS

A health economics (HE) sub-study will be performed. Core resource use information will be collected, using CRFs on days in hospital (by speciality) and outpatient visits. Data being collected on concomitant medication will also be used in the economic analysis. Information on patients' use of primary care and community-based services will be collected as additional questions in the QL questionnaire. Costs will be calculated on the basis of representative UK unit costs at the point of analysis. Health outcomes will be assessed in terms of quality-adjusted life years (QALYs). Quality adjustments will be based on patients' responses to the EQ-5D health status measure which will be administered at baseline and each point of follow-up as part of the QL questionnaire. A cost-effectiveness analysis will compare all regimens that continue to recruit into their Activity Stage IV.

17.3 TRANSLATIONAL SUB-STUDIES

17.3.1 DNA ANALYSIS

Blood samples from as many patients as possible will be collected for future research. With patient consent, an additional droplet of blood sample will be collected and stored for DNA and protein analysis in order to try to identify molecular features of clinical significance.

Blood samples should be sent directly to the central laboratory on the FTA elute cards provided. Patient information sheets and consent forms which highlight this research are given in **Appendix B**, while details of specimen collection, posting and contact details are given in **Appendix D**.

17.3.2 TISSUE MICROARRAY

Patient consent will be sought to utilise paraffin embedded tissue for the construction of tissue microarrays from needle cores. One needle biopsy will be selected for microarray and the remaining tissue will be returned to the originating histopathology lab. Given the entry criteria for the trial, the majority of patients will have extensive disease in the diagnostic needle core biopsies, in contrast to men with localised, low grade disease. Consequently, removal of one core is unlikely to compromise any subsequent histopathological assessment. Details regarding transfer of samples will be issued at the time of construction of the micro array. Additional analyses e.g. DNA extraction may also be performed on the tissue arrays.

18 PUBLICATION

The results from different centres will be analysed together and published as soon as possible. Individual clinicians must not publish data concerning their patients that are directly relevant to questions posed by the study until the TMG has published its report. The TMG together with the STAMPEDE collaborators will form the basis of the writing committee and decide on the nature of publications. Any release, of efficacy or safety data, presentation or publication will be agreed with the TSC according to the terms of their charter.

All publications will acknowledge the participating centres and clinicians, and these will be detailed in an appendix to the main report. Papers will have named authors determined by the TMG according to the following principles:

- To be as inclusive as possible where this is practicable
- To ensure that there is justification for anyone to be named as an author
- Reasons for nomination for authorship may include: trial design; grant holding; day-to-day trial oversight (TMG membership); analysis; discussion and interpretation of data; representation for key groups; active participation at large recruiting sites. It should be accepted that the people qualifying for authorship will vary over time. In addition, key positions will vary depending on the nature of the publication: clinical lead for clinical papers, statistician lead for methodology papers, translational papers may be led by authors not on the main TMG if appropriate (e.g., the bone sub-study). In the event of any dispute related to authorship or data release, the TSC will be responsible for making the executive decision.

In the manuscript, a full list of sites and the number of patients recruited will be provided. In the presentations, this list of sites will also be shown. The term “the STAMPEDE investigators” will clearly be stated and relevant names included in the presentation credits.

A detailed Publication Plan is documented elsewhere.

19 PROTOCOL AMENDMENTS

19.1 PROTOCOL

19.1.1 Amendments Made To Sections In Protocol Version 1.0 (May 2004)

Administrative changes such as typos, word change etc.

Name additions/changes to:

TMG members

TSC members

IDMC members

'General Information' Section – additional information re. Abridged version of protocol

Section 1.2 – Figure 1, Celecoxib duration amended

Section 1.3 – Figure 2, addition of cardiovascular assessment form, name and timings amended

Section 2.3 – Docetaxel information updated

Section 2.4 – Additional text re dose and duration justification for Celecoxib use.

Section 3 – Title change and content updated

Section 4.2 – New exclusion criteria added

Section 4.3.1 – New investigations added and additional text re testosterone measurements and additional text re. prior celecoxib treatment

Section 6.1.4 – Celecoxib duration amended

Section 6.1.5 – Additional text re. Co-administration of docetaxel and bisphosphonates

Section 6.1.6 – Celecoxib duration amended

Section 6.2.2 – additional docetaxel information

Section 6.2.3 – addition of CV event history

Section 11 – Safety reporting updated

Section 12.1 – Additional text re. the collection of blood for genetic and serum marker studies

Section 15 – Additional information re. Central Subvention for docetaxel arms

19.1.2 Amendments Made To Sections In Protocol Version 1.1 (May 2005)

Section 6.2 Administration and Dose Modifications, subsection 6.2.1 Zoledronic Acid

19.1.3 Amendments Made To Sections In Protocol Version 2.0 (Jun 2005)

General Information section – SAE reporting fax number and timeframe added.

Section 1.2 – Addition of anti-androgen use for M0 patients as a method of HT

Section 1.2 – Increase in amount of blood needed & addition tissue sample request.

Section 1.3 Trial Documentation updated to include new table detailing trial documentation ahead of accreditation, the inclusion of the radiotherapy forms and correct case report form timings

Section 2.1 – Addition of anti-androgen use for M0 patients as a method of HT

Section 4.1.3 – Inclusion criteria Vii "Normal testosterone prior to hormone treatment" removed.

Section 4.1.3 - φnote has been omitted and moved to section 4.2 (see number 8)

Section 4.2 – Exclusion criteria added to exclude patients with active peptic ulceration, gastrointestinal bleeding and inflammatory bowel disease.

Section 4.2 – Exclusion Criteria added to exclude patients with planned major dental work

Section 4.3.1 - All blood test timelines changed from 14 days to 28 days.

Section 4.3.1 – Hormone Therapy pre-randomisation deadline extended from 4 weeks to 12 weeks.

Section 4.3.1 – Additional information regarding the use of NSAIDs and cox-2-inhibitors before coming on to the STAMPEDE study and once commenced on study treatment

Section 4.3.2 – Updated to ask for all vitamins and minerals the patient is taking to be recorded.

Section 4.3.3 – Updated to include the extra blood required and the request for consent of patients' tissue samples.

Section 6.1.1 – Addition of anti-androgen use for M0 patients as a method of HT

Section 6.1.6 – Addition of the calcium & vitamin name "calcichew".

Section 6.6.2 – asking also to collect vitamins and minerals under concomitant medication.

Section 6.6.3 – New section to inform investigators that patient's, who they wish to give radiotherapy to, are also eligible for STAMPEDE

Section 6.6.4 – New section to detail what data is being collected on the radiotherapy given to patients.

Section 7.1; figure 4 – Addition of radiotherapy form and in note, addition of AA alone

Section 7.1.2 – omission of repeated scans and x-rays at 24 weeks, also omitted in note under figure 4.

Chapter 11 – Safety reporting section updated

Section 17.3 – Increase in amount of blood needed & additional tissue sample request.

19.1.4 Amendments Made To Section In Protocol Version 3.0 (Jul 2006)

Front Cover - NCRN logo added for accuracy

Front Cover - Clarification that protocol developed with NCRI rather than on behalf of

Front Cover - Clarification that it is a 6 arm trial

General Information section - MRC CTU staff section updated

Section 1.2 – Statistics section updated.

Section 1.2 - Additional research paragraph updated to reflect additional studies and for clarification of terms

Section 1.2 - Blood collection volume changed to reflect new technique used

Section 1.3 (figure 3) - Table showing case report form schedule updated to reflect clarification of follow-up schedule and addition of new CRF (End of Treatment)

Section 2.2 - AS changed to HT (clarification of terms)

Section 2.3 - Updated in information in regard to use of docetaxel added to reflect up to date practice

Section 2.5 - Sub-headings numbered for consistency

Section 3.0 - Information in regard to the Pilot Phase now written in past tense as Pilot Phase has now been completed

Section 4.1.1 - Inclusion criteria extended so that patients who fulfil 2 out of the three of the first inclusion criteria can be eligible.

Section 4.3.1 - Change in time scales by which baseline investigations need to be completed.

Section 4.3.1 - Clarification that chest X-ray is only required if chest is not included in the CT

Section 4.3.1 - Removal of 12 week timeline for baseline PSA test to be performed. (Stipulation that it must be performed before start of HT)

Section 4.3.2 – Information added in regard to time allowed from randomisation to start of treatment

Section 4.3.3 - Additional research paragraph updated to reflect additional studies and for clarification of terms

Section 4.3.3 - Blood collection volume changed to reflect new technique used

Sections 6.1.2-6.1.6 - Androgen Suppression replaced with hormone therapy for consistency of terms

Section 6.2.2 - '(Taxotere)' Removed for consistency

Section 6.2.2 _ information added in regard to the need to closely monitor liver function prior to docetaxel administration

Section 7.1 - Page number reference updated

Section 7.1.1 - PSA measurement timings updated to accurately reflect follow-up schedule

Section 7.3 (Table 4) - Table and key updated to accurately reflect follow-up schedule and to include information about new CRFs and removal of withdrawal CRF

Section 8 - Rewording for clarification of definition of trial withdrawal

Section 8.1 - Instruction that withdrawal from trial treatment should be recorded on End of Treatment Form rather than withdrawal form

Section 8.1 - Information updated to emphasise that trial treatment must be discontinued following a progression

Section 8.2- Information added in regard to patient transfers

Section 8.3 - Instruction that withdrawal from trial completely must be notified in writing to the MRC CTU rather than included on withdrawal form

Section 9 and Summary – Target event numbers updated to reflect the slightly revised numbers obtained by using –nstage- which is the new, recommended program for MAMS trials

Sections 11.1 and 11.2 - Form numbers removed to allow for future changes in numbering

Section 11.2 – Reference to toxicity grading website added

Section 11.2.1 - Reference to table in appendix G added

Section 12.2 - 'Suggested' removed from 'Suggested patient information sheets'

Section 13 - CTA reference added

Section 17.3 - Information added to reflect new blood collection method for DNA analysis and in regard to additional translational studies for which funding has recently been approved

19.1.5 Amendments Made To Protocol Version 4.0 (Dec 2007)

General Information Section - Randomisation and SAE reporting details sections clarified

Section 1.2 and throughout protocol - Efficacy Stages 1-111 renamed to Activity Stages 1-111 for accuracy and clarity

Section 1.2 - Follow schedule corrected

Section 4.1.2 - Inclusion criteria widened to include high risk relapsing patients, that would not have met the previous PSA based criteria

Section 4.1.3 - Note added to reference location of WHO performance status definitions

Section 4.2 - Notes added to reference locations of toxicity gradings and NYHA classifications

Section 4.3.1 - Timings of baseline scan information changed to accurately reflect most common current practice

Section 6.1.1 - Information about use of LHRH antagonists to ensure that the protocol accurately reflects current and future practice

Section 6.1.1 - Information about suggested duration of hormone therapy added to ensure that the protocol accurately reflects current practice

Section 6.2.2 - Additional information added about the timing of liver function tests prior to docetaxel administration added for clarity

Section 6.6.4 - Information on radiotherapy data collection added

Section 7.1.1 - Erroneous information about the timing of PSA measurements removed

Figure 3 - Moved to new section in protocol for clarity and extended to include current information on data collection

Figure 3b - Added to describe how extent of data collection during follow-up should change, post treatment and post progression

Figure 4 - Notes added to explain the changes in data collected at follow-up and to information that the quality of life study will be applicable to the first 700 patients randomised only

Figure 4 - Note added to include palliative radiotherapy CRF

Section 11.3 - SAE reporting information updated

Section 19 - Protocol amendments list updated

19.1.6 Amendments Made To Protocol Version 5.0 (Aug 2008)

1. General Information Section – Randomisation phone line number updated – non UK extension added
2. Section 3 – Information about QL study removed to reflect closure of QL study after first 700 patients
3. Section 4.2 – Exclusion criteria clarified to explain that only patients with severe poor cardiovascular history should be excluded
4. Section 4.3.1 – Information on co-administration of NSAIDS with celecoxib changed based on clinical advice.

5. Section 5 - Randomisation phone line number updated – non UK extension added

6. Section 6.2.1. – Information added to clarify that patients who develop an osteonecrosis of the jaw should stop zoledronic acid treatment

7. Section 6.2.3 – 'severe' text added to accurately reflect which patients should be excluded based on their cardiovascular history

8. Section 7.1.2 – Definition of disease progression extended for clarity

9. Figure 3 – Updated to include reference to newly created skeletal related event form

10. Figure 4 – Previous error in table amended to show that the 4th Zoledronic Acid form that is submitted contains information about 3 cycles rather than 2 as previously indicated

11. Table 4 – 'Other important medical condition' added to definition of serious in the SAE section, to accurately reflect SAE form and current practice

12. Section 11.1 – Information added on reporting or pregnancies

13. Section 17 - Information about QL study removed to reflect closure of QL study after first 700 patients

19.1.7 Amendments Made To Protocol Version 6.0 (Jul 2009)

1. General Information Section – Trial Pharmacist removed and changes of:

Co-Investigator

Patient Representatives

Trial Manager

Data Manager

General Information Section - Coordinating Centre – address change

General Information Section – change of Sponsor address

Section 1.1 – ratio of patients randomised to the investigational arms updated

Section 1.2 – figure 1b added to clarify trial design from Apr-2011 onwards

Section 1.2 – paragraph added to explain trial changes after the second activity analysis

Section 1.2 – wording added to clarify that QL data only collected for first 700 patients randomised

Section 1.3 – SSA Favourable Opinion removed from list of trial documentation required ahead of site accreditation

Section 2.1 – Amount of men diagnosed with prostate cancer annually updated

Section 2.4 –note added to explain completion of recruitment to celecoxib- containing arms

Section 2.5.2 - note added to explain completion of recruitment to celecoxib- containing arms

Section 3 – SSA Favourable Opinion removed

Section 4.2 – Exclusion criterion xiii greyed out

Section 4.3.1 – paragraph removed regarding potential randomisation to celecoxib-containing arms

Section 5 – Randomisation instructions expanded to exclude public holidays or dates when notice has been given by the CTU

Section 6.1.4 – formatting changed to grey font to reflect recruitment completion for arm D

Section 6.1.6 - formatting changed to grey font to reflect recruitment completion for arm F

Section 6.2.3 – recruitment note added

Section 6.6.3 – radiotherapy statement changed to reflect data from recent trials

Section 7.1.2 – removal of reference to SRE- specific CRF

Section 7.3 – Figure 3 - Addition of Bone Density Risk Factor Form and BMD sub-study assessment forms to summary of timing table

Section 7.3 – Figure 4 – Weeks added to timings of assessments post 2 years

Section 7.3- Figure 4 – note added to explain recruitment completion for arms D and F

Section 12.1 – Wording changed to reflect change to randomisation allocation ratio

Section 12.1 – Addition of statement regarding new information emerging during the trial

Section 12.2 – Reference to SSA removed

Section 16.3 – Statement added regarding actioning IDMC recommendation ahead of TSC ratification

19.1.8 Amendments Made To Protocol Version 7.0 (Jul 2011)

1. General Information Section- SAE reporting fax number corrected

2. Section 11- SAE reporting fax number corrected

19.1.9 Amendments Made To Protocol Version 7.1 (Jul 2011)

Throughout protocol – numbering has been updated in some sections new accommodate new information that has been added.

General Information Section – contact details updated

General Information Section – Funding information updated to include involvement from additional company

General Information Section – Wording on compliance and regulations updated to reflect current MRC CTU standard wording

General Information Section – Abbreviations list updated

Section 1.1 – The number of investigational agents being studied updated from three to four

Section 1.1 – Information regarding celecoxib updated to reflect that recruitment to these arms was discontinued in Apr-2011

Section 1.1 – Information about new IMP, Abiraterone inserted

Section 1.1 – Sample size and trial duration information updated to reflect changes brought about by additional trial arm

Section 1.2 – Summary information updated to reflect the discontinuation of recruitment to celecoxib arms and the addition of abiraterone

Figures 1a, b and c - Updated to reflect the discontinuation of recruitment to celecoxib arms and the addition of abiraterone

Section 1.2 – Information on trial stages updated to reflect changes brought about by additional trial arm

Section 1.2 – Information updated regarding the re-opening of the quality of life sub-study from implementation of protocol version 8.0

Section 2.1 – Wording related to hormone therapy updated for clarity

Section 2.1 - Updated to reflect the discontinuation of recruitment to celecoxib arms and the addition of abiraterone

Section 2.2 – Updated references added

Section 2.3 – Updated references added

Section 2.5 – Section added to give background information on new IMP, abiraterone

Section 2.6.1 – Updated references added

Section 2.7 – Section added to give information regarding radiotherapy which is to be given as part of standard care following recently published trial data.

Section 3 – Wording updated regarding selection of investigators to reflect current MRC CTU practice

Section 4.1 – Inclusion criteria updated with new criterion regarding radiotherapy use

Section 4.1 - Inclusion criteria updated with new criterion regarding contraceptive use

Section 4.1 – Wording of inclusion and exclusion criteria updated for clarity

Section 4.1 – Exclusion criteria updated with new criterion regarding acceptable liver function for trial entry

Section 4.1 – Exclusion criteria updated with specifics related to blood pressure levels

Section 4.1 - Exclusion criteria updated with new criterion regarding concomitant medications

Section 4.1 - Exclusion criteria updated with new criterion regarding prior treatment with abiraterone

Section 4.1 - Exclusion criteria updated with new criterion regarding prior treatment with chemotherapy

Section 4.1 - Exclusion criteria updated with new criterion regarding prior treatment with zoledronic acid

Section 4.3 – Wording updated to reflect that patients who initially fail screening can be re-screened at a later date

Section 4.3.2 – Wording updated regarding prior anti-androgen and LHRH use updated for clarity

Section 5.1 – Co-enrolment guidelines information updated to describe newly created co-enrolment CRF

Section 6.1 – Trial treatment information updated to reflect the fact that anti-androgens alone will be no longer permitted as hormone therapy

Section 6.1.1 – Updated to describe patients for whom radiotherapy should be given as standard practice

Section 6.1.1 a and b - Sections added to give information regarding radiotherapy treatment

Section 6.1.1-6.1.6 – References to further sections updated

Section 6.1.7 – Section added to describe abiraterone treatment

Section 6.2.4 - Section added to describe abiraterone treatment

Section 6.6 - Section added to give information regarding radiotherapy treatment

Section 7.1.1 – Reference to blood being taken at patient's home removed as this does not occur in practice

Section 7.1.2 – Wording updated regarding the reporting of biochemical failures for clarity

Section 7.1.2 – Wording updated regarding skeletal-related events for clarity

Section 7.1.3 – Section added to describe additional assessments required related to abiraterone treatment

Section 7.1.4 – Section added to provide information on when treatment should commence

Figure 4 – Updated for clarity regarding return of BMD sub-study forms, the addition the co-enrolment CRF and the description of the re-opening of the QoL Sub-study.

Figure 5 – Updated with reference to abiraterone and co-enrolment form

Section 7.3 - Wording on trial closure updated to reflect current MRC CTU standard wording

Section 8.1 – Additional criteria for definition of progression added for clarity

Section 8.1 – Definition of progression for abiraterone patients added.

Section 9 – Statistical information updated to describe the addition of the new trial arm

Section 11 – Safety reporting wording updated for clarity

Section 11 – SAE reporting fax number updated

Section 12 – Ethical information updated to describe the unequal randomisation allocation ratio

Section 12 – Ethical information updated to describe that the visit schedule will vary according to trial arm

Section 12.2 – Wording updated to reflect international participation in the trial

Section 13 – Wording updated to reflect international participation in the trial

Section 14 – Wording updated to reflect international participation in the trial

Section 15 - Updated to reflect the discontinuation of recruitment to celecoxib arms and the addition of abiraterone

Section 16 – Reference to trial committee charters added for information

Section 17.1 – Information added to reflect re-opening of quality of life sub-study

Section 17.2 – Timing of health economics analysis updated to previous error

Section 18 – Information on publication policy expanded for clarity

Section 19 – Information regarding amendments to protocol appendices moved to the separate appendices document

Section 20 – References extensively updated

19.1.10 Amendments Made To Protocol Version 8.0 (Sep 2011)

Throughout protocol – numbering, sections headings, tables, figures and bibliographical references have been updated in some sections to accommodate new information that has been added

Throughout protocol – Androgen Deprivation Therapy has replaced Hormone Therapy as deemed more representative of the type of hormone therapy used in the study

General Information Section – New staff members of the MRC CTU and Co-Investigators added and contact details updated

General Information Section – Abbreviations list updated

Section 1.1 – Information regarding the new research radiotherapy treatment inserted

Section 1.1 – Information regarding docetaxel updated

Section 1.2 – Wording updated to reflect the addition of the new research comparison arm

Section 1.3 – Additional criteria for the re-accreditation of participating centres (for protocol version 9.0 only)

Section 2.1.1 – Wording updated to clarify the use of anti-androgen in trial patients

Section 2.1.2 – Information added to describe the rationale for the RT comparison arm

Section 2.8 – Information added to describe research RT treatment to prostate for patients with newly diagnosed metastatic disease

Section 3.1 – Information added to describe RT Quality Assurance procedures and centre accreditation

Section 4.1.1 to 4.1.3 – Wording updated to clarify inclusion criteria for all patients groups (newly diagnosed non-metastatic, metastatic and relapsing patients)

Section 4.2 – Clarification added on cardiovascular exclusion criteria

Section 4.2 – New exclusion criterion added concerning patients with prior exposure to hormone therapy

Section 4.2 – New exclusion criterion added to reflect the addition of the new RT comparison arm

Section 4.4.1 – Clarification added regarding pre-randomisation checks

Section 4.4.2 – Clarification added regarding permissible hormone therapy duration prior to randomisation

Section 4.4.5 – Information added regarding starting research radiotherapy treatment

Section 4.4.6 – Information updated on concomitant medications

Section 5 – Clarification regarding randomisation allocation added to reflect the addition of the new RT research arm

Section 6.1.8 – Information added to describe the administration of research radiotherapy

Section 6.2.1 – Clarification added regarding the measurement of serum creatinine levels prior to the administration of zoledronic acid

Section 6.2.3 – Clarification regarding the completion of recruitment to the celecoxib containing arms

Section 6.25 – Information added regarding the administration of research radiotherapy treatment

Section 6.6 – Clarification incorporated to describe the administration of standard-of-care radiotherapy

Section 7.1.4 – Information added regarding data collection and non-administration of standard radiotherapy

Section 7.2 – Section updated to include new treatment specific CRFs and timing of CRFs

Section 8.1 – Clarification added for the criteria to stop treatment for patients randomised to arm G

Section 8.2 – Section expanded to include additional details on study patient transfer to different centres

Section 8.3 – Additional sentence inserted to reinforce the importance of compliance with follow up assessments

Section 9.1 – Additional paragraph inserted to clarify the method of randomisation and allocation distribution in the light of the introduction of the new RT arm

Section 9.4 – Wording updated to clarify the assessment of safety data

Section 9.5.4 – Wording updated concerning the end of randomisations to arm G

Section 9.6 to 9.6.4 – Section added describing sample size issues and trial stages for arm H

Section 9.8 – Clarification on intermediate stopping guidelines

Section 9.9 – Clarification on the outline analysis plan

Section 11 – Information on safety reporting updated to reflect the addition of the research RT comparison arm

Section 11 – Clarification added regarding arm A safety reporting timelines

Section 12.1 – Clarification added regarding the Principal Investigator’s responsibilities

Section 14 – Indemnity section updated to reflect current MRC policy

Section 16 – Clarification regarding TMG membership

Section 17.3 – Section on Bone Mineral Density sub-study removed

Section 19 – Information regarding amendments to protocol appendices moved to the separate appendices document

Section 20 – References updated

19.1.11 Amendments Made To Protocol Version 9.0 (Oct-2012)

Throughout protocol – numbering, sections headings, tables, figures and bibliographical references have been updated in some sections to accommodate the completion of recruitment to original research arms B, C and E.

Throughout protocol – Tenses have been changed to reflect activities that were in the future and which have now been passed.

Section 1 – Figure added and clarifications added to each figure

Section 2 – Previous reference 8 removed

Section 4 – Clarification of acceptable alternatives to bone scans

Section 6.2.5 – Correction of an error defining the PTV: the wording has been reordered

Table 4 – Dose-volume objectives corrected: order swapped

Table 5- Correction CRFs names

Section 17.3.2 – Clarification that DNA may be extracted

19.1.12 Amendments Made To Protocol Version 10.0 (Apr-2013)

Throughout protocol – numbering, sections headings, tables, figures and bibliographical references have been updated in some sections

Throughout protocol – typos have been corrected

Section 4 –Clarification of exclusion criteria V (now V and VI)

Section 6 – Timing of orchidectomy prior to randomisation extended to 12 weeks

Section 6 – Clarification of hypokalaemia, blood pressure and fluid retention management

Section 9 – Statistical considerations amended in light of the recruitment extension for the abiraterone comparison

Section 14 - Section updated to reflect the changes in the structure of the MRC CTU (now MRC CTU at UCL) and indemnity arrangements

19.1.13 Amendments Made To Protocol Version 11.0 (Sep-2013)

Throughout protocol – numbering, sections headings, tables, figures and bibliographical references have been updated in some sections

Throughout protocol – typos have been corrected

Co-investigators list updated to reflect the addition of the “enzalutamide + abiraterone comparison” lead

Section 1.2 – Enzalutamide added as trial treatment

Section 1.2 – Protocol version 12.0 added to the list of amendments

Section 2.10 – Rationale for the combination of enzalutamide and abiraterone

Section 4.2 – Eligibility criteria amended to reflect the addition of enzalutamide + abiraterone arm

Section 4.4.2 – Wording clarified

Section 6.8 – Clarification regarding end of trial treatment after starting trial therapy

Section 6.10 – Section added to describe enzalutamide and abiraterone treatment for the new research arm (Arm J)

Section 6.11.4.A – Section added to describe the management of toxicities from trial abiraterone

Section 6.11.4.B - Section added to describe the management of toxicities from trial enzalutamide

Section 9.1.4 – Section added to describe the statistical considerations concerning the introduction of Arm J

Section 9.3 – Principles and assumption for the introduction of Arm J added

Section 9.7 and sub-sections – Sample size issues and trial stages for Arm J

Section 9.9 – Details on interim monitoring and analyses for Arm J added

Section 11.2.1.D – Wording clarified regarding safety reporting requirements for control arm

Section 12.1 – Wording clarified

Section 15 – Details on funding for the “enzalutamide + abiraterone comparison” added

Section 19 - Amendments made to protocol updated

Reference list updated

20 REFERENCES

1. James ND, Sydes MR, Mason MD, Clarke NW, Anderson J, Dearnaley DP, et al. Celecoxib plus hormone therapy versus hormone therapy alone for hormone-sensitive prostate cancer: first results from the STAMPEDE multiarm, multistage, randomised controlled trial. The lancet oncology. 2012;13(5):549-58. Epub 26-Mar-2012.
2. Cancer Research UK. CancerStats Key Facts: Prostate Cancer. Cancer Research UK. 2011.
3. Sharifi N, Gulley JL, Dahut WL, Sharifi N, Gulley JL, Dahut WL. An update on androgen deprivation therapy for prostate cancer. Endocrine-Related Cancer. 2010;17(4):R305-15.
4. Widmark A, Klepp O, Solberg A, Damber JE, Angelsen A, Fransson P, et al. Endocrine treatment, with or without radiotherapy, in locally advanced prostate cancer (SPCG-7/SFUO-3): an open randomised phase III trial. Lancet. 2009;373(9660):301-8. Epub 2008/12/19.
5. Warde PR, Mason MD, Sydes MR, Gospodarowicz MK, Swanson GP, Kirkbride P, et al. Intergroup randomized phase III study of androgen deprivation therapy (ADT) plus radiation therapy (RT) in locally advanced prostate cancer (CaP) (NCIC-CTG, SWOG, MRC-UK, INT: T94-0110; NCT00002633). J Clin Oncol. 2010;28(18s Supplement: Proceedings of ASCO 2010):Abstr CRA4504.
6. Warde P, Mason M, Ding K, Kirkbride P, Brundage M, Cowan R, et al. Survival Benefit with Combined Androgen Deprivation and Radiation Therapy in Locally Advanced Prostate Cancer – Results of a Phase III Trial. The Lancet. 2011 - in press.
7. Mason M, Sydes M, Parulekar W, Parmar M, Anderson J, Barber J, et al. Final analysis of intergroup randomized phase III study of androgen deprivation therapy (ADT) + radiation therapy (RT) in locally advanced prostate cancer (CaP) (NCIC-CTG, SWOG, MRC-UK, INT: T94-0110). National Cancer Research Institute (NCRI) Cancer Conference 2012. 2012;2012.
8. Saad F, Colombel M. Management of castration-resistant prostate cancer: bisphosphonates and emerging therapies. Expert Review of Anticancer Therapy. 2010;10(12):1991-2002.
9. Sartor O, Halstead M, Katz L, Sartor O, Halstead M, Katz L. Improving outcomes with recent advances in chemotherapy for castrate-resistant prostate cancer. Clinical Genitourinary Cancer. 2011;8(1):23-8.
10. Attard G, Richards J, de Bono JS, Attard G, Richards J, de Bono JS. New strategies in metastatic prostate cancer: targeting the androgen receptor signaling pathway. Clinical Cancer Research. 2011;17(7):1649-57.
11. Lipton A. Improving progression-free and overall survival in patients with cancer: a potential role for bisphosphonates. Expert Opinion on Pharmacotherapy. 2011;12(5):749-62.
12. Lipton A, Small E, Saad F, Gleason D, Gordon D, Smith M, et al. The new bisphosphonate, Zometa (zoledronic acid), decreases skeletal complications in both osteolytic and osteoblastic lesions: a comparison to pamidronate. [Review] [55 refs]. Cancer Investigation. 2002;20 Suppl 2:45-54.
13. Green JR, Muller K, Jaeggi KA. Preclinical pharmacology of CGP 42'446, a new, potent, heterocyclic bisphosphonate compound. Journal of Bone & Mineral Research. 1994;9(5):745-51.
14. Major P, Lortholary A, Hon J, Abdi E, Mills G, Menssen HD, et al. Zoledronic acid is superior to pamidronate in the treatment of hypercalcemia of malignancy: a pooled analysis of two randomized, controlled clinical trials. J Clin Oncol. 2001;19(2):558-67. Epub 2001/02/24.
15. Costa L, Harper P, Coleman RE, Lipton A, Costa L, Harper P, et al. Anticancer evidence for zoledronic acid across the cancer continuum. Critical Reviews in Oncology-Hematology. 2011;77 Suppl 1:S31-7.
16. Rosen LS, Gordon D, Kaminski M, Howell A, Belch A, Mackey J, et al. Long-term efficacy and safety of zoledronic acid compared with pamidronate disodium in the treatment of skeletal complications in patients with advanced multiple myeloma or breast carcinoma: a randomized, double-blind, multicenter, comparative trial. Cancer. 2003;98(8):1735-44. Epub 2003/10/10.

17. Rosen LS, Gordon DH, Dugan W, Jr., Major P, Eisenberg PD, Provencher L, et al. Zoledronic acid is superior to pamidronate for the treatment of bone metastases in breast carcinoma patients with at least one osteolytic lesion. Cancer. 2004;100(1):36-43. Epub 2003/12/24.
18. Saad F, Gleason D, Murray R, Tchekmedyian S, Venner P, Lacombe L, et al. A Randomized, Placebo-Controlled Trial of Zoledronic Acid in Patients With Hormone-Refractory Metastatic Prostate Carcinoma Journal of the National Cancer Institute. 2002;94(19).
19. Dearnaley DP, Sydes MR, Mason MD, Stott M, Powell CS, Robinson AC, et al. A double-blind, placebo-controlled, randomized trial of oral sodium clodronate for metastatic prostate cancer (MRC PR05 Trial). J NatlCancer Inst. 2003;95(17):1300-11.
20. Petrylak DP, Tangen CM, Hussain MH, Lara PN, Jr., Jones JA, Taplin ME, et al. Docetaxel and estramustine compared with mitoxantrone and prednisone for advanced refractory prostate cancer. N Engl J Med. 2004;351(15):1513-20. Epub 2004/10/08.
21. Tannock IF, de Wit R, Berry WR, Horti J, Pluzanska A, Chi KN, et al. Docetaxel plus prednisone or mitoxantrone plus prednisone for advanced prostate cancer. N Engl J Med. 2004;351(15):1502-12. Epub 2004/10/08.
22. Hawkey CJ. COX-2 inhibitors.[comment]. Lancet. 1999;353(9149):307-14.
23. Taketo MM. Cyclooxygenase-2 inhibitors in tumorigenesis (Part II). Journal of the National Cancer Institute. 1998;90(21):1609-20.
24. Taketo MM. Cyclooxygenase-2 inhibitors in tumorigenesis (Part I). Journal of the National Cancer Institute. 1998;90(20):1529-36.
25. Nelson JE, Harris RE. Inverse association of prostate cancer and non-steroidal anti-inflammatory drugs (NSAIDs): results of a case-control study. Oncology Reports. 2000;7(1):169-70.
26. Eberhart CE, Coffey RJ, Radhika A, Giardiello FM, Ferrenbach S, DuBois RN. Up-regulation of cyclooxygenase 2 gene expression in human colorectal adenomas and adenocarcinomas. Gastroenterology. 1994;107(4):1183-8.
27. Roberts RO, Jacobson DJ, Girman CJ, Rhodes T, Lieber MM, Jacobsen SJ. A population-based study of daily nonsteroidal anti-inflammatory drug use and prostate cancer. [see comments.]. Mayo Clinic Proceedings. 2002;77(3):219-25.
28. Basler JW, Piazza GA, Basler JW, Piazza GA. Nonsteroidal anti-inflammatory drugs and cyclooxygenase-2 selective inhibitors for prostate cancer chemoprevention. Journal of Urology. 2004;171(2 Pt 2):S59-62; discussion S-3.
29. Masferrer JL, Leahy KM, Koki AT, Zweifel BS, Settle SL, Woerner BM, et al. Antiangiogenic and antitumor activities of cyclooxygenase-2 inhibitors. Cancer Research. 2000;60(5):1306-11.
30. Steinbach G, Lynch PM, Phillips RK, Wallace MH, Hawk E, Gordon GB, et al. The effect of celecoxib, a cyclooxygenase-2 inhibitor, in familial adenomatous polyposis. New England Journal of Medicine. 2000;342(26):1946-52.
31. Solomon SD, McMurray JJ, Pfeffer MA, Wittes J, Fowler R, Finn P, et al. Cardiovascular risk associated with celecoxib in a clinical trial for colorectal adenoma prevention. New England Journal of Medicine. 2005;352(11):1071-80.
32. Danila DC, Morris MJ, de Bono JS, Ryan CJ, Denmeade SR, Smith MR, et al. Phase II multicenter study of abiraterone acetate plus prednisone therapy in patients with docetaxel-treated castration-resistant prostate cancer. J Clin Oncol. 2010;28(9):1496-501. Epub 2010/02/18.
33. Reid AH, Attard G, Danila DC, Oommen NB, Olmos D, Fong PC, et al. Significant and sustained antitumor activity in post-docetaxel, castration-resistant prostate cancer with the CYP17 inhibitor abiraterone acetate. J Clin Oncol. 2010;28(9):1489-95. Epub 2010/02/18.
34. Ryan CJ, Smith MR, Fong L, Rosenberg JE, Kantoff P, Raynaud F, et al. Phase I clinical trial of the CYP17 inhibitor abiraterone acetate demonstrating clinical activity in patients with castration-resistant prostate cancer who received prior ketoconazole therapy. Journal of Clinical Oncology. 2010;28(9):1481-8.

35. de Bono JS, Logothetis CJ, Molina A, Fizazi K, North S, Chu L, et al. Abiraterone and increased survival in metastatic prostate cancer. New England Journal of Medicine. 2011;364(21):1995-2005.
36. Ryan CJ, Smith MR, de Bono JS, Molina A, Logothetis CJ, de Souza P, et al. Abiraterone in metastatic prostate cancer without previous chemotherapy. N Engl J Med. 2013;368(2):138-48. Epub 2012/12/12.
37. Attard G, Reid AHM, de Bono JS. Abiraterone acetate is well tolerated without concomitant use of corticosteroids. Journal of Clinical Oncology. 2010;28(29):e560-1; author reply e2.
38. Flanigan RC, Salmon SE, Blumenstein BA, Bearman SI, Roy V, McGrath PC, et al. Nephrectomy followed by interferon alfa-2b compared with interferon alfa-2b alone for metastatic renal-cell cancer. N Engl J Med. 2001;345(23):1655-9. Epub 2002/01/05.
39. Mickisch GH, Garin A, van Poppel H, de Prijck L, Sylvester R. Radical nephrectomy plus interferon-alfa-based immunotherapy compared with interferon alfa alone in metastatic renal-cell carcinoma: a randomised trial. Lancet. 2001;358(9286):966-70. Epub 2001/10/05.
40. Warde P, Mason M, Ding K, Kirkbride P, Brundage M, Cowan R, et al. Combined androgen deprivation therapy and radiation therapy for locally advanced prostate cancer: a randomised, phase 3 trial. The Lancet. 2011;378:2104-11. Epub 03-Nov-2011.
41. Neville-Webbe HL, Holen I, Coleman RE. The anti-tumour activity of bisphosphonates. Cancer Treatment Reviews. 2002;28(6):305-19.
42. Witters LM, Crispino J, Fraterrigo T, Green J, Lipton A. Effect of the combination of docetaxel, zoledronic acid, and a COX-2 inhibitor on the growth of human breast cancer cell lines. American journal of clinical oncology. 2003;26(4):S92-7. Epub 2003/08/07.
43. Attard G, Cooper CS, de Bono JS. Steroid hormone receptors in prostate cancer: a hard habit to break? Cancer cell. 2009;16(6):458-62. Epub 2009/12/08.
44. Chen G, Wang X, Zhang S, Lu Y, Sun Y, Zhang J, et al. Androgen receptor mutants detected in recurrent prostate cancer exhibit diverse functional characteristics. The Prostate. 2005;63(4):395-406. Epub 2004/12/24.
45. Taplin ME, Bubley GJ, Ko YJ, Small EJ, Upton M, Rajeshkumar B, et al. Selection for androgen receptor mutations in prostate cancers treated with androgen antagonist. Cancer Res. 1999;59(11):2511-5. Epub 1999/06/11.
46. Veldscholte J, Ris-Stalpers C, Kuiper GG, Jenster G, Berrevoets C, Claassen E, et al. A mutation in the ligand binding domain of the androgen receptor of human LNCaP cells affects steroid binding characteristics and response to anti-androgens. Biochemical and biophysical research communications. 1990;173(2):534-40. Epub 1990/12/14.
47. Zhao XY, Malloy PJ, Krishnan AV, Swami S, Navone NM, Peehl DM, et al. Glucocorticoids can promote androgen-independent growth of prostate cancer cells through a mutated androgen receptor. Nature medicine. 2000;6(6):703-6. Epub 2000/06/03.
48. Attard G, Reid AH, Auchus RJ, Hughes BA, Cassidy AM, Thompson E, et al. Clinical and biochemical consequences of CYP17A1 inhibition with abiraterone given with and without exogenous glucocorticoids in castrate men with advanced prostate cancer. The Journal of clinical endocrinology and metabolism. 2012;97(2):507-16. Epub 2011/12/16.
49. Richards J, Lim AC, Hay CW, Taylor AE, Wingate A, Nowakowska K, et al. Interactions of abiraterone, eplerenone, and prednisolone with wild-type and mutant androgen receptor: a rationale for increasing abiraterone exposure or combining with MDV3100. Cancer Res. 2012;72(9):2176-82. Epub 2012/03/14.
50. Scher HI, Fizazi K, Saad F, Taplin ME, Sternberg CN, Miller K, et al. Increased survival with enzalutamide in prostate cancer after chemotherapy. N Engl J Med. 2012;367(13):1187-97. Epub 2012/08/17.
51. Tombal B, Borre M, Rathenborg P, Werbrouck P, Heidenreich A, Iversen P, et al. Enzalutamide monotherapy: Phase II study results in patients with hormone-naive prostate cancer. Journal of Clinical Oncology. 2013;31(Supplement 6):Abstract 18.

52. Efstathiou E, Titus MA, Tsavachidou A, Hoang A, Karlou M, Wen S, et al. MDV3100 effects on androgen receptor (AR) signaling and bone marrow testosterone concentration modulation: A preliminary report. . 2011 ASCO Annual Meeting, 2011 J Clin Oncol 2011.
53. Locke JA, Guns ES, Lubik AA, Adomat HH, Hendy SC, Wood CA, et al. Androgen levels increase by intratumoral de novo steroidogenesis during progression of castration-resistant prostate cancer. Cancer Res. 2008;68(15):6407-15. Epub 2008/08/05.
54. Tran C, Ouk S, Clegg NJ, Chen Y, Watson PA, Arora V, et al. Development of a second-generation antiandrogen for treatment of advanced prostate cancer. Science. 2009;324(5928):787-90. Epub 2009/04/11.
55. Scher HI, Beer TM, Higano CS, Anand A, Taplin ME, Efstathiou E, et al. Antitumour activity of MDV3100 in castration-resistant prostate cancer: a phase 1-2 study. Lancet. 2010;375(9724):1437-46. Epub 2010/04/20.
56. Efstathiou E, Titus M, Wen A, Miguel AS, Hoang A, De A, et al. The effects of enzalutamide (ENZA) in combination with abiraterone acetate (AA) in patients with bone metastatic castration resistant prostate cancer (mCRPC). Eur J Cancer. 2013;49(ECCO 2013 Supplement):Abstract 2854.
57. Kouloulias V, Giraud J-Y, Davis BJ, Dusserre A, Zurlo A, Bolla M. Quality assurance of the 22961 EORTC trial. A phase III study of the optimal combination of hormonal adjuvant treatment by LHRH analogue and radiation therapy for the management of locally advanced prostate cancer: the dummy run. 2004(0167-8140 (Print)).
58. Royston P, Parmar MKB, Qian W. Novel designs for multi-arm clinical trials with survival outcomes with an application in ovarian cancer. Statistics in Medicine. 2003;22(14):2239-56.
59. Royston P, Barthel FMS, Parmar MKB, Choodari-Oskooei B, Isham V. Designs for clinical trials with time-to-event outcomes based on stopping guidelines for lack of benefit. Trials. 2011;12(1):81.
60. Royston P. nstage: MAMS trial sample size calculator. MRC Clinical Trials Unit, London2009.
61. Haybittle JL. Repeated assessment of results in clinical trials of cancer treatment. The British journal of radiology. 1971;44(526):793-7. Epub 1971/10/01.
62. Peto R, Pike MC, Armitage P, Breslow NE, Cox DR, Howard SV, et al. Design and analysis of randomized clinical trials requiring prolonged observation of each patient. I. Introduction and design. British journal of cancer. 1976;34(6):585-612. Epub 1976/12/01.
63. Dolan P, Gudex C, Kind P, Williams A. Discussion Paper 138: A social tarif for EuroQol: results from a UK General Population Survey. . Centre for Health Economics. University of York, 1995.

STAMPEDE

Systemic Therapy in Advancing or Metastatic Prostate Cancer: Evaluation of Drug Efficacy

A multi-arm multi-stage randomised controlled trial

**Version:**	**13.0**
**Date:**	**14-Feb-2015**

MRC CTU ID:	PR08
ISRCTN #:	ISRCTN78818544
NCT #:	NCT00268476
EUDRACT #:	2004-000193-31
CTA #:	00316/0026/001-0001
MREC #:	04/MRE07/35

Authorised by:

Name:	PROFESSOR NICHOLAS D JAMES
Role:	CHIEF INVESTIGATOR

Name:	MATTHEW SYDES
Role:	TRIAL STATISTICIAN

GENERAL INFORMATION

This document was constructed using the MRC CTU Protocol Template Version 4.0. It describes the STAMPEDE trial, coordinated by the Medical Research Council (MRC) Clinical Trials Unit (CTU) at University College London (UCL), and provides information about procedures for entering patients into it. The protocol should not be used as an aide-memoire or guide for the treatment of other patients. Every care has been taken in drafting this protocol, but corrections or amendments may be necessary. These will be circulated to the registered investigators in the trial, but sites entering patients for the first time are advised to contact the Cancer And Other Non-Infectious Diseases Group, MRC CTU at UCL, London, UK, to confirm they have the most up-to-date version.

COMPLIANCE

The trial will be conducted in compliance with the approved protocol, the Declaration of Helsinki 1996, the principles of Good Clinical Practice (GCP), Commission Directive 2005/28/EC with the implementation in national legislation in the UK by Statutory Instrument 2004/1031 and subsequent amendments, the UK Data Protection Act (DPA number: Z5886415), and the National Health Service (NHS) Research Governance Framework for Health and Social Care (RGF). International sites will comply with the principles of GCP as laid down by the ICH topic E6 (Note for Guidance on GCP), Commission Directive 2005/28/EC (the European Directive 2001/20/EC [where applicable]) and applicable national regulations.

SPONSOR

Medical Research Council, 2nd Floor, David Phillips Building, Polaris House, North Star Avenue, Swindon, SN2 1FL, UK

FUNDING

Clinical Trials Advisory Awards Committee (on behalf of Cancer Research UK, Medical Research Council, and other charities) together with educational grants from Novartis, Sanofi-Aventis, Pfizer ,Janssen Pharma NV, Astellas.

AUTHORISATIONS AND APPROVALS

The following persons are authorised to sign the final protocol and protocol amendments for the sponsor: Professor Nicholas James (Chief Investigator) and Matthew Sydes (Trial Statistician).

TRIAL REGISTRATION

This trial has been registered with the ClinicalTrials.gov Clinical Trials Register, where it is identified as NCT00268476.

RANDOMISATIONS
To randomise, call MRC CTU at UCL, Monday to Friday 0900-1700
excluding public holidays or dates when notice has been given by the CTU.
Tel: +44 (0) 20 7670 4777

SAE REPORTING
Fax to 020 7670 4818 within 24 hours of becoming aware of the event

TRIAL ADMINISTRATION

COORDINATING SITE

MRC Clinical Trials Unit at UCL	Switchboard:	020 7670 4700
Aviation House	Fax:	020 7670 4818
Kingsway	Email:	mrcctu.stampede@ucl.ac.uk
London		
WC2B 6NH		
UK		

MRC CTU STAFF

Trial Manager:	Francesca Schiavone	Tel:	0207 670 4632
Trial Manager:	Alanna Brown	Tel:	0207 670 4882
Trial Manager:	Orla Prendiville	Tel:	0207 670 4840
Data Manager:	Carly Au	Tel:	0207 670 4947
Data Manager:	Zohrah Khan	Tel:	0207 670 4794
Data Manager:	Peter Vaughan	Tel:	0207 670 4655
Clinical Project Manager	Claire Amos	Tel:	0207 670 4771
Statistician:	Melissa Spears	Tel:	0207 670 4764
Senior Trial Statistician:	Matt Sydes	Tel:	0207 670 4798
Director of MRC CTU:	Max Parmar	Tel:	0207 670 4729
Trial Surgeon:	Alastair Ritchie	Tel:	Please contact STAMPEDE team

CHIEF INVESTIGATOR

Professor Nicholas James
Warwick Medical School
University of Warwick
Coventry
CV4 7AL
UK

Tel: 024 7657 4365 (Secretary)
Fax:
Email: n.d.james@warwick.ac.uk

CO-INVESTIGATORS

Prof Noel Clarke<br>Urologist	Manchester, UK	Vice-Chair
Prof Johann De Bono<br>Oncologist	London, UK	
Prof David Dearnaley	London, UK	
Prof Malcolm Mason<br>Oncologist	Cardiff, UK	Vice-Chair
Dr Chris Parker<br>Oncologist	London, UK	Lead: "M1|RT comparison"
Dr Gerhardt Attard<br>Oncologist	London, UK	Lead:"Enzalutamide+Abiraterone comparison"
Dr Daniel Aebersold<br>Oncologist	Bern, Switzerland	
Dr Martin Russell	Glasgow, UK	
Prof George Thalmann<br>Urologist	Bern, Switzerland	

Patient Representatives
Robin Millman
Patient

David Matheson
Patient

Health Economics
Prof Mark Sculpher — York, UK

Molecular Genetics
To be nominated

For full details of all trial committees, please see **Appendix M**.

CONTENTS

GENERAL INFORMATION I

CONTENTS 4

ABBREVIATIONS 8

1 SUMMARY 11
1.1 LAY SUMMARY 11
1.2 ABSTRACT AND SUMMARY OF TRIAL DESIGN 12
1.3 TRIAL DOCUMENTATION 18

2 BACKGROUND 19
2.1 INTRODUCTION AND SETTING 19
2.1.1 Long-term Androgen Deprivation Therapy 19
2.1.2 Role of Radiotherapy for Patients With M0 Disease 19
2.2 RATIONALE 19
2.3 DESIGN 20
2.4 RESEARCH TREATMENT AND BISPHOSPHONATES 20
2.5 RESEARCH TREATMENT: CHEMOTHERAPY 21
2.6 RESEARCH TREATMENT: CYCLOOXYGENASE-2 INHIBITORS 21
2.7 RESEARCH TREATMENT: STEROID SYNTHESIS INHIBITORS 22
2.8 RESEARCH TREATMENT: RADIOTHERAPY TO THE PROSTATE FOR PATIENTS WITH NEWLY-DIAGNOSED METASTATIC DISEASE 22
2.9 RESEARCH TREATMENT: COMBINATIONS OF ORIGINAL RESEARCH ARMS 24
2.9.1 Bisphosphonate And Chemotherapy 24
2.9.2 Bisphosphonate And Cyclooxygenase-2 Inhibitors 24
2.10 COMBINATION OF STEROID SYNTHESIS INHIBITORS AND ANDROGEN RECEPTOR SIGNALLING INHIBITOR 25
2.10.1 Supplementing Abiraterone And Prednisolone With Enzalutamide 25
2.10.2 Supplementing Enzalutamide With Abiraterone And Prednisolone 25
2.10.3 Summary Of Rationale For This Combination 25

3 SELECTION OF INSTITUTIONS AND INVESTIGATORS 27
3.1 RADIOTHERAPY ACCREDITATION 27

4 SELECTION OF PATIENTS 28
4.1 PATIENT INCLUSION CRITERIA 28
4.1.1 High-Risk Newly-Diagnosed Non-Metastatic Node-Negative Disease 28
4.1.2 Newly-Diagnosed Metastatic Or Node-Positive Disease 28
4.1.3 Previously Treated With Radical Surgery And/Or Radiotherapy, Now Relapsing 28
4.1.4 For All Patients 28
4.2 PATIENT EXCLUSION CRITERIA 29
4.3 SELECTION CRITERIA FOR COMPARISON OF RESEARCH (M1) RT FOR METASTATIC DISEASE 30
4.4 SCREENING PROCEDURES 30
4.4.1 Investigations Prior To Randomisation 30
4.4.2 Androgen Deprivation Therapy Prior To Randomisation 31
4.4.3 Hypercalcaemia At Randomisation 31

4.4.4 NSAIDs And Cox-2 Inhibitors At Randomisation 31
4.4.5 Starting Trial Treatment 31
4.4.6 Concomitant Medications 32
4.5 ADDITIONAL DETAILS FOR PATIENTS JOINING SUB-STUDIES 32

5 RANDOMISATION AND ENROLMENT 33
5.1 CO-ENROLMENT GUIDELINES 33

6 TREATMENT OF PATIENTS 34
6.1 TRIAL TREATMENT 34
6.2 ARM A: ADT ALONE OR ADT + STANDARD-OF-CARE (M0) RT (CONTROL ARM) 34
6.2.1 Hormone Therapy 34
6.2.2 Standard-of-care (M0) RT 34
6.3 ARM B: ADT + ZOLEDRONIC ACID 35
6.4 ARM C: ADT + DOCETAXEL 35
6.5 ARM D: ADT + CELECOXIB 35
6.6 ARM E: ADT + DOCETAXEL + ZOLEDRONIC ACID 36
6.7 ARM F: ADT + ZOLEDRONIC ACID + CELECOXIB 36
6.8 ARM G: ADT + ABIRATERONE 36
6.9 ARM H: ADT + PROSTATE RADIOTHERAPY IN M1 PATIENTS 37
6.10 ARM J: ADT + ABIRATERONE + PREDNISOLONE + ENZALUTAMIDE ADMINISTRATION 38
6.11 ADMINISTRATION AND DOSE MODIFICATIONS 38
6.11.1 Zoledronic Acid 38
6.11.2 Docetaxel 39
6.11.3 Celecoxib 39
6.11.4 Abiraterone or Enzalutamide + Abiraterone 40
6.11.5 Research (M1) Prostate Radiotherapy 42
6.12 TRIAL PRODUCTS 44
6.13 MEASURES OF COMPLIANCE/ADHERENCE 44
6.14 TREATMENT DATA COLLECTION 44
6.15 ADMINISTRATION OF STANDARD RADIOTHERAPY TO NON-METASTATIC PATIENTS 44
6.15.1 Treatment Details 44
6.16 NON-TRIAL TREATMENT 46
6.16.1 Medications Permitted 46
6.16.2 Data On Concomitant Medication 46

7 ASSESSMENTS AND PROCEDURES 47
7.1 SCHEDULE FOR ASSESSMENTS 47
7.1.1 PSA Measurements 47
7.1.2 Assessment Of Treatment Failure (Definition Of Progression) 47
7.1.3 Additional Safety Assessment 47
7.1.4 Data Collection and Non-Administration Of Standard Radiotherapy 48
7.1.5 Data Collection Palliative Radiotherapy 48
7.1.6 Data Collection Research (M1) Radiotherapy 48
7.1.7 Follow-Up Schedules 48
7.2 FOLLOW-UP 48
7.3 TRIAL CLOSURE 52

8 STOPPING OF TREATMENT OR FOLLOW UP 53
8.1 STOPPING RESEARCH INTERVENTIONS 53

8.2 PATIENT TRANSFERS 53
8.3 EARLY CESSATION OF TRIAL PARTICIPATION 54

9 STATISTICAL CONSIDERATIONS 55
9.1 METHOD OF RANDOMISATION 55
9.1.1 To Version 7 55
9.1.2 Version 8 55
9.1.3 Version 10 and 11 55
9.1.4 Version 12 55
9.2 OUTCOME MEASURES 56
9.3 SAMPLE SIZE: PRINCIPLES AND ASSUMPTIONS 57
9.4 SAMPLE SIZE ISSUES AND TRIAL STAGES: ORIGINAL RESEARCH ARMS (B-F) 58
9.4.1 Pilot Phase: Original Research Arms (B-F) 58
9.4.2 Activity Stages I-III: Original Research Arms (B-F) 58
9.4.3 Efficacy Stage IV: Original Research Arms (B-F) 59
9.4.4 Sample Size For Original Research Arms (B-F) 59
9.5 SAMPLE SIZE ISSUES AND TRIAL STAGES: ADDITIONAL RESEARCH ARM G 59
9.5.1 Pilot Phase: Additional Research Arm G 59
9.5.2 Activity Stages I-III: Additional Research Arm G 59
9.5.3 Efficacy Stage IV: Additional Research Arm G 60
9.5.4 Sample Size For Additional Research Arm G 60
9.6 SAMPLE SIZE ISSUES AND TRIAL STAGES: ADDITIONAL RESEARCH ARM H 61
9.6.1 Pilot Phase: Additional Research Arm H 61
9.6.2 Activity Stages I-III: Additional Research Arm H 61
9.6.3 Efficacy Stage IV: Additional Research Arm H 61
9.6.4 Sample Size For Additional Research Arm H 61
9.7 SAMPLE SIZE ISSUES AND TRIAL STAGES: ADDITIONAL RESEARCH ARM J 63
9.7.1 Pilot Phase: Additional Research Arm J 63
9.7.2 Activity Stages I-II: Additional Research Arm J 63
9.7.3 Efficacy Stage III: Additional Research Arm J 63
9.7.4 Sample Size For Additional Research Arm J 63
9.7.5 Further Sample Size Issues For Additional Research Arm J 64
9.8 FURTHER NOTES ON TRIAL DESIGN 65
9.8.1 Overall Sample Size 65
9.8.2 Factorial Design 65
9.9 INTERIM MONITORING AND ANALYSES 66
9.10 OUTLINE ANALYSIS PLAN 66
9.10.1 Pilot / Safety Phases 66
9.10.2 Activity And Efficacy Stages 67

10 MONITORING AND QUALITY ASSURANCE 68
10.1 MONITORING AT MRC CTU 68
10.2 DIRECT ACCESS TO DATA 68
10.3 VISITS TO INVESTIGATOR SITES 68
10.4 CONFIDENTIALITY 68

11 SAFETY REPORTING 69
11.1 DEFINITIONS 69
11.1.1 Trial-Specific Exemptions 70
11.2 INSTITUTION/INVESTIGATOR RESPONSIBILITIES 70

11.2.1 Investigator Assessment 70
11.2.2 Notification Procedure 71
11.3 MRC CTU RESPONSIBILITIES 72

12 ETHICAL CONSIDERATIONS AND APPROVAL 73
12.1 ETHICAL CONSIDERATIONS 73
12.2 ETHICAL APPROVAL 73

13 REGULATORY APPROVAL 75

14 INDEMNITY 76

15 FINANCE 77

16 TRIAL COMMITTEES 78
16.1 TRIAL MANAGEMENT GROUP (TMG) 78
16.2 TRIAL STEERING COMMITTEE (TSC) 78
16.3 INDEPENDENT DATA MONITORING COMMITTEE (IDMC) 78

17 ANCILLARY STUDIES 80
17.1 QUALITY OF LIFE 80
17.2 HEALTH ECONOMICS 80
17.3 TRANSLATIONAL SUB-STUDIES 80
17.3.1 DNA Analysis 80
17.3.2 Tissue Microarray 81

18 PUBLICATION 82

19 PROTOCOL AMENDMENTS 83
19.1 PROTOCOL 83
19.1.1 Amendments Made To Sections In Protocol Version 1.0 (May 2004) 83
19.1.2 Amendments Made To Sections In Protocol Version 1.1 (May 2005) 83
19.1.3 Amendments Made To Sections In Protocol Version 2.0 (Jun 2005) 83
19.1.4 Amendments Made To Section In Protocol Version 3.0 (Jul 2006) 84
19.1.5 Amendments Made To Protocol Version 4.0 (Dec 2007) 85
19.1.6 Amendments Made To Protocol Version 5.0 (Aug 2008) 85
19.1.7 Amendments Made To Protocol Version 6.0 (Jul 2009) 86
19.1.8 Amendments Made To Protocol Version 7.0 (Jul 2011) 87
19.1.9 Amendments Made To Protocol Version 7.1 (Jul 2011) 87
19.1.10 Amendments Made To Protocol Version 8.0 (Sep 2011) 88
19.1.11 Amendments Made To Protocol Version 9.0 (Oct-2012) 89
19.1.12 Amendments Made To Protocol Version 10.0 (Apr-2013) 90
19.1.13 Amendments Made To Protocol Version 11.0 (Sep-2013) 90
19.1.14 Amendments Made To Protocol Version 12.0 (Jan-2014) 91

20 REFERENCES 92

ABBREVIATIONS

Abbreviation	Expansion
ACE	Angiotensin-Converting Enzyme
ACTH	Adrenocorticotropic hormone
ADT	Androgen deprivation therapy
AR	Androgen receptor
AS	Activity Stage
bid	Twice a day (bis in die)
BP	Blood pressure
BSA	Body surface area
CERES	Consumers for Ethics in Research
CF	Consent Form
CI	Chief Investigator
CI	Confidence interval
COSTART	Coding Symbols for a Thesaurus of Adverse Reaction Terms
Cox 2	Cyclooxygenase 2
CRF	Case Report Form
CRUK	Cancer Research UK
CRPC	Castrate Refractory Prostate Cancer
CT	Computerised tomography
CTA	Clinical Trials Authorisation
CTAAC	Clinical Trials Advisory and Awards Committee
CTC	Common Toxicity Criteria
CTU	Clinical Trials Unit
CTV	Clinical Tumour Volume
CXR	Chest X-ray
DDX	Doctors and Dentists Exemption
DHT	Dihydrotestosterone
DNA	Deoxyribonucleic Acid
DPA	Data Protection Act
ERC	Endpoint Review Committee
ES	Efficacy Stage
ICH	International Conference on Harmonization

Abbreviation	Expansion
ECG	Electro cardiogram
FBC	Full Blood Count
FFS	Failure-Free Survival
GCP	Good Clinical Practice
GP	General Practitioner
GRO	General Register Office
HE	Health Economics
HES	Hospital Episode Statistics
hr	Hour
HR	Hazard Ratio
HRPC	Hormone Refractory Prostate Cancer
HT	Hormone Therapy
IDMC	Independent Data Monitoring Committee
IM	Intramuscular
IMRT	Intensity Modulated Radiation Therapy
ISRCTN	International Standard Randomised Controlled Trial Number
IU	International Units
IV	Intravenous
LD	Longest diameter
LFTs	Liver Function Tests
LHRH	Luteinising Hormone Releasing Hormone
LREC	Local Research Ethics Committee
m	Month
MHRA	Medicine and Healthcare Products Regulatory Agency
min	Minutes
MRC	Medical Research Council
MREC	Multi-Centre Research Ethics Committee
MRI	Magnetic resonance imaging
M0	Non-metastatic
M1	Metastatic
NCI	National Cancer Institute (USA)
NCRN	National Cancer Research Network
NHS	National Health Service

Abbreviation	Expansion
NSAID	Non-Steroidal Anti-inflammatory Drugs
ONS	Office for National Statistics
OS	Overall Survival
PI	Principal Investigator
PIS	Patient Information Sheet
po	per orum (orally)
PSA	Prostate Specific Antigen
pts	Patients
PTV	Planned Tumour Volume
QALY	Quality-adjusted Life Years
qds	quater die sumendus (4 times each day)
QL	Quality of Life
R&D	Research and Development
RECIST	Response Evaluation Criteria In Solid Tumours
SAE	Serious Adverse Event
SAR	Serious Adverse Reaction
sc	Sub-cutaneous (under skin)
SNP	Single Nucleotide Polymorphism
SSA	Site Specific Assessment
STAMPEDE	Systemic Therapy in Advancing and Metastatic Prostate Cancer: Evaluation of Drug Efficacy
SUSAR	Suspected Unexpected Serious Adverse Reactions
SWOG	South West Oncology Group
TMG	Trial Management Group
TMT	Trial Management Team
TURP	Trans-Urethral Resection of Prostate
TSC	Trial Steering Committee
UCL	University College London
ULN	Upper Limit of Normal
U+E	Urea and Electrolytes
WHO	World Health Organisation

1 SUMMARY

1.1 LAY SUMMARY

Prostate cancers depend upon the male hormone testosterone for their growth. Lowering testosterone levels (either by removing all or part of both testes, or by giving anti-hormone treatment) slows the growth of prostate cancers. This type of treatment is called hormone treatment and is often used when prostate cancers have spread outside the prostate gland. Although hormone treatment is usually successful at stopping the cancer growing for a period of time, the cancer will begin to grow again in most men.

There are increasing numbers of treatments available for advanced prostate cancer. These treatments are usually used in prostate cancer when hormone treatment is no longer effective and the cancer has started to grow again. The aim of this trial, which is called STAMPEDE, is to assess some of these treatments, given earlier in the course of the disease in combination with hormone treatment.

The treatments that have been, or are being, assessed during the trial are:

1. Zoledronic acid: Prostate cancer cells can spread to bones and weaken them. Zoledronic acid is a drug that reduces bone destruction and hardens bones. This may make them more resistant to attack by cancer cells.

2. Docetaxel: A drug that stops cells replicating that is currently being used to treat a range of cancers including lung, breast and ovarian cancer as well as prostate cancer. Docetaxel prolongs survival in men with relapsed metastatic prostate cancer.

3. Celecoxib: An aspirin-like drug that is used to treat arthritis. It slows down the growth of cancer cells in the laboratory. We wished to see if it had the same effect on cancer cells in patients. Recruitment to new patients for the evaluation of this drug is finished as a planned interim analysis failed to demonstrate sufficient activity.

4. Abiraterone (included from protocol version 8.0)**:** An inhibitor of steroid hormone synthesis that blocks prostate cancer cells from generating their own male hormones. This is thought to be a major way in which prostate cancer cells resume growth following anti-hormonal therapies. The agent prolongs survival when given to men following failure of docetaxel chemotherapy.

5. Prostate radiotherapy (included from protocol version 9.0)**:** treatment with high-energy x-rays targeted to the prostate gland. This treatment is now mandatory for patients with cancer that is confined to the prostate gland as large trials have shown it improves survival times. We are not certain whether we should give radiotherapy to the prostate if the cancer has already spread.

6. Enzalutamide (included from protocol version 12.0)**:** This is a blocker of androgen receptors. These stimulate the cancer when hormone therapies have failed. Enzalutamide may be mutually complementary to abiraterone in terms of blocking mechanisms of resistance. The agent prolongs survival when given to men following failure of docetaxel chemotherapy.

STAMPEDE will look at the effect of combining one or two of the treatments described above with hormone treatment. A computer program will be used to allocate which treatment the patient receives, using a chance process. The trial will look at the effects of the combined treatments on quality of life and find out whether the new treatment combinations increase the time when the cancer is not growing and ultimately results in patients living longer. The study will also look at which treatment provides the greater value for money for the health service. More than 7,000 patients will join the trial with answers becoming available over 7 to 12 years.

1.2 ABSTRACT AND SUMMARY OF TRIAL DESIGN

STAMPEDE is a multi-centre, randomised controlled trial for patients with locally advanced or metastatic prostate cancer who are about to commence Androgen Deprivation Therapy (ADT). Patients can have either newly diagnosed disease, or have been previously treated with radical radiotherapy or surgery but now have signs of progression such as a rising prostate specific antigen (PSA) (further details on eligibility see **Section 4**). The trial will assess the effects of adding different agents, both as single agents and in combinations, to androgen deprivation therapy. The investigational agents are (i) a bisphosphonate, zoledronic acid, (ii) a cytotoxic chemotherapeutic agent, docetaxel and (iii) a cyclooxygenase (Cox-2) inhibitor, celecoxib (iv) a novel androgen deprivation therapy drug called abiraterone, a steroid synthesis inhibitor and an androgen receptor signalling inhibitor (v) enzalutamide. Recruitment to the celecoxib arms (D and F) is now closed. An additional arm containing abiraterone was added in protocol version 8.0. A further comparison arm involving prostate radiotherapy for patients with metastatic disease was added in protocol version 9.0, with the addition of an arm considering the combination of enzalutamide and abiraterone added in protocol version 12.0. The trial has multiple arms; the control arm of the trial is androgen deprivation therapy (ADT) only, achieved through the use of luteinising hormone releasing hormone (LHRH) analogues or LHRH antagonists, or bilateral orchidectomy according to local practice. The other trial arms are summarised in **Figures 1 to 7**.

Figure 1: Recruiting arms of the STAMPEDE trial to Apr-2011

Patients eligible for STAMPEDE

↓

STARTING LONG-TERM HORMONES

RANDOMISATION

**A**	ADT
**B**	ADT + zoledronic acid
**C**	ADT + docetaxel
**D**	ADT + celecoxib
**E**	ADT + ZA + docetaxel
**F**	ADT + ZA + celecoxib

Figure 2: Recruiting arms of the STAMPEDE trial from Apr-2011 to Nov-2011 (v7.0)

Patients eligible for STAMPEDE

↓

STARTING LONG-TERM HORMONES

RANDOMISATION

**A**	ADT
**B**	ADT + zoledronic acid
**C**	ADT + docetaxel
**E**	ADT + ZA + docetaxel

Accrual stopped to celecoxib-containing Arms, D and F, after their Activity Stage II analysis

Figure 3: Recruiting arms of the STAMPEDE trial from Nov-2011 to Jan-2013 (v9.0)

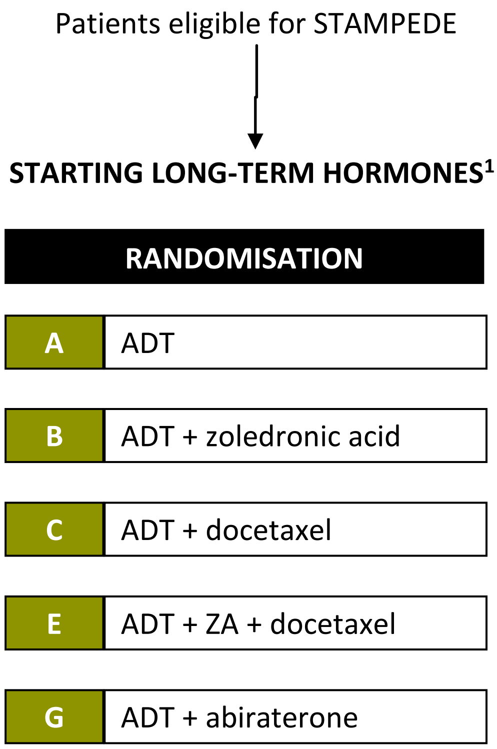

[1] All suitable pts with newly diagnosed locally advanced disease should also have RT to the prostate

Accrual was initiated to the abiraterone arm, Arm G, in Nov-2011.

Figure 4: Recruiting arms of the STAMPEDE trial from protocol version 9.0 (Jan-2013 to Mar-2013)

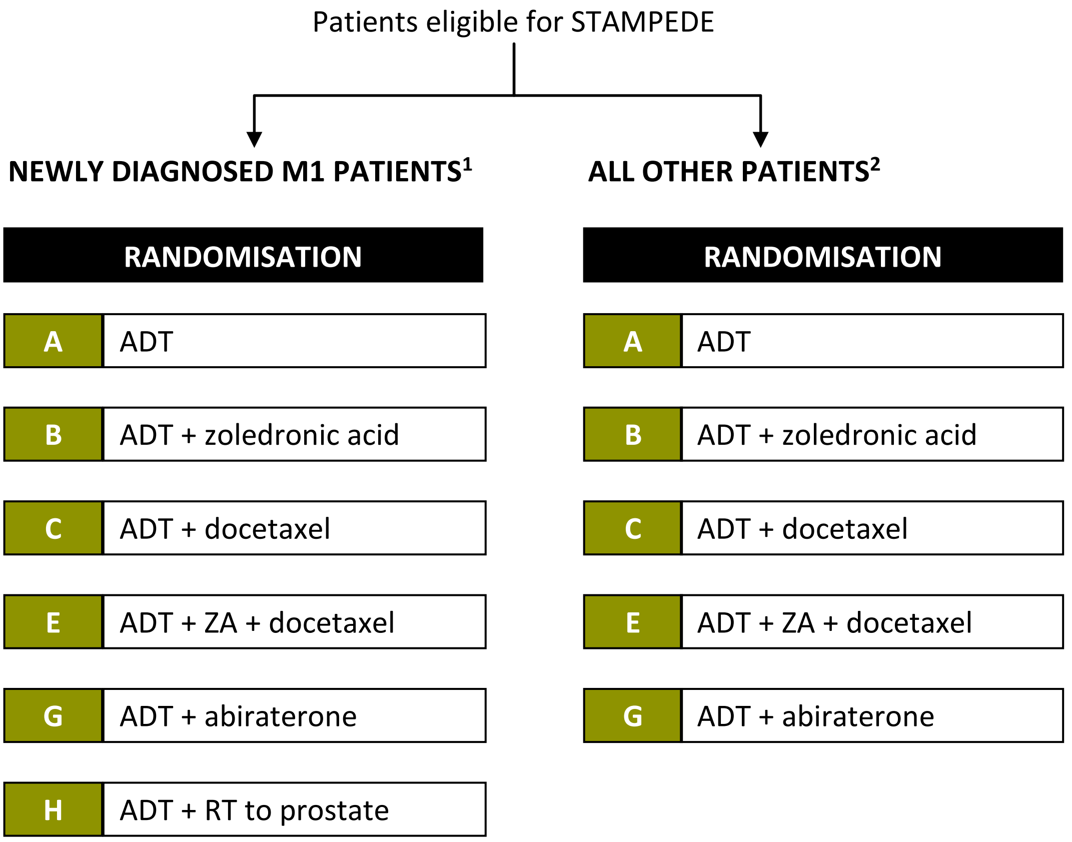

[1] Except pts with a contra-indication to RT
[2] All suitable pts with newly diagnosed locally advanced disease should also have RT to the prostate

Accrual was initiated to the radiotherapy-to-the-prostate for metastatic disease arm, Arm H, in Jan-2013.

Figure 5: **Arms of the STAMPEDE Trial from protocol version 10.0 (after original research arms completed accrual in March 2013)**

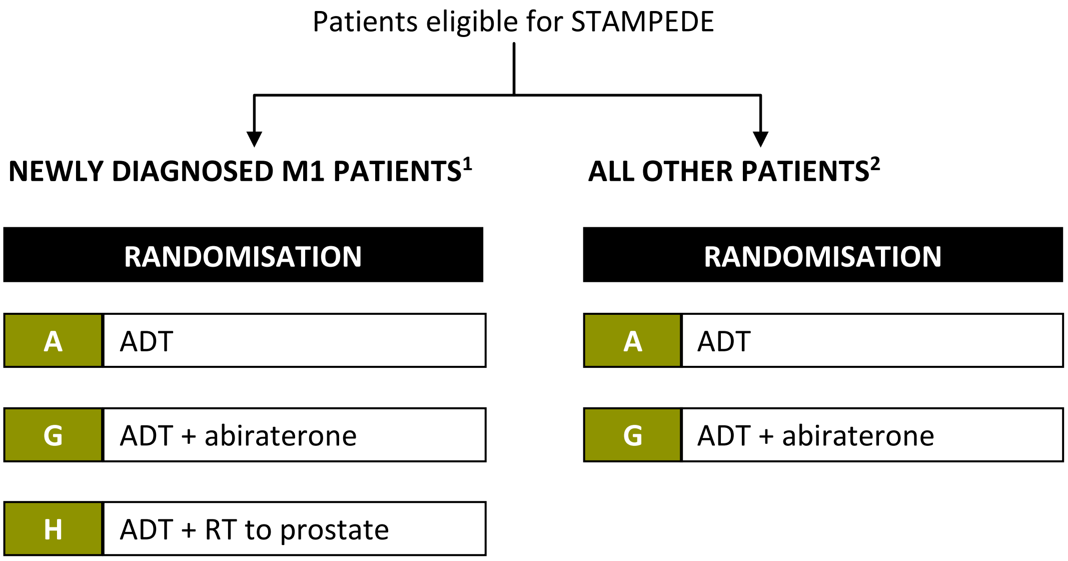

[1] Except pts with a contra-indication to RT
[2] All suitable pts with newly diagnosed locally advanced disease should also have RT to the prostate

Figure 6: Arms of the STAMPEDE Trial from protocol version 12.0 (end of recruitment to Arm G and introduction of enzalutamide + abiraterone comparison)

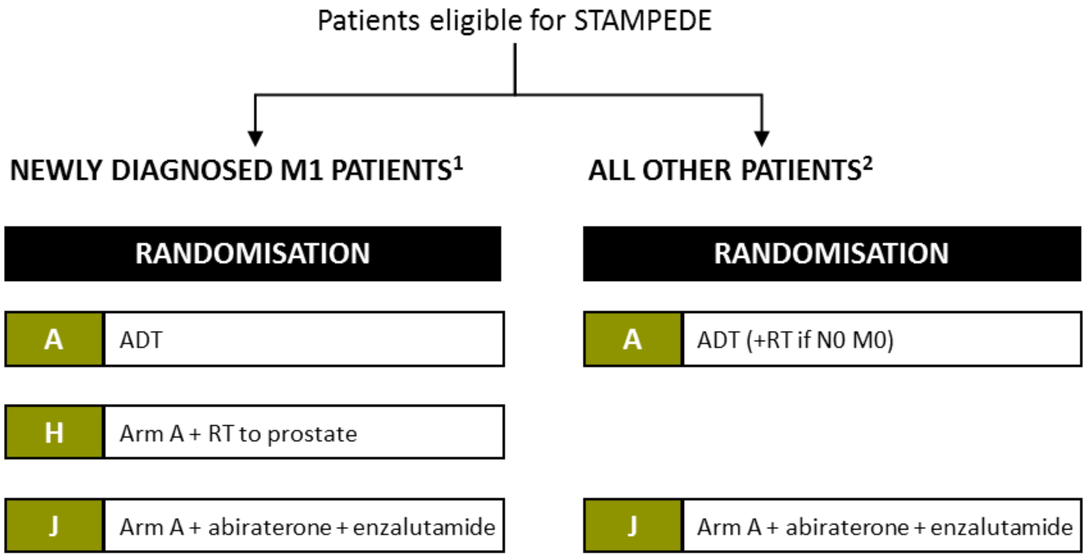

[1] Except pts with a contra-indication to RT
[2] All suitable pts with newly diagnosed locally advanced disease should also have RT to the prostate

Figure 7: Arms of the STAMPEDE trial open to recruitment over time

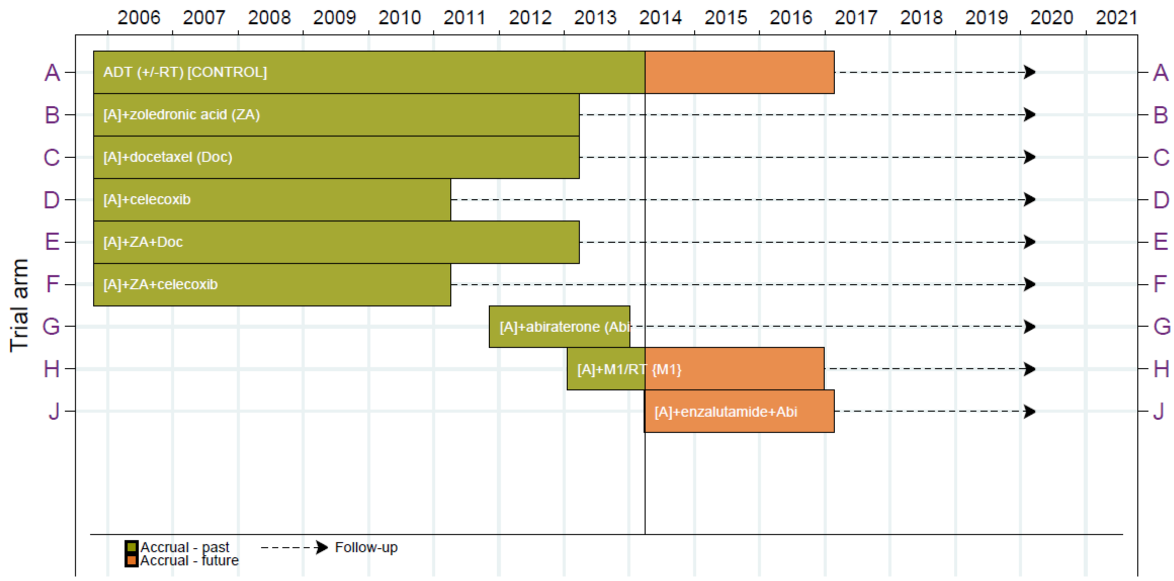

For each comparison of research arm against control, the trial will be conducted in a number of stages: a Pilot/Safety Phase, Activity Stages and a final Efficacy Stage. The primary outcome measure of the Pilot/Safety Phase is the safety, with 30-50 patients recruited to each research arm. Research arms will only continue to recruitment in the next stage if they have been shown to be both safe and feasible, although patient data from all patients and all stages will be included in the final analyses. In the Activity Stages the primary outcome measure is failure-free survival (FFS). Further patients will be recruited until a certain number of FFS events have been observed in the control arm (see **Section 9** for further detail). Some evidence of activity will be required for a research arm to continue recruitment in each stage and guidelines are in place. The Efficacy Stage will take place when a certain number of primary outcome measure events are observed in the control arm patients for that relevant comparison. This is when around 403 deaths have been reported in the control arm for the “original research comparisons” (involving docetaxel and zoledronic acid) and around 267 in the control arm for the “abiraterone comparison”, the “M1|RT comparison” and the “enzalutamide+abiraterone comparison”. The exact number of patients and duration of the trial will depend on the observed accrual rate, observed event rate and the number of other research arms open to recruitment contemporaneously.

Recruitment to Arms D (ADT + celecoxib) and F (ADT + zoledronic acid + celecoxib) was stopped in Apr-2011 after the second planned activity analysis when the IDMC and TSC considered the lack-of-benefit guidelines.(1) Refer to **Section 9.3** for further information regarding the guidelines for stopping accrual to research arms during the activity stages of the trial.

In version 8.0 of the protocol a new arm G (ADT + abiraterone) was added. Arm H (ADT+ prostate radiotherapy) was added in protocol version 9.0. The trial stages remain similar to at trial inception but will be staggered in time compared to the stages for the original arms A-F. Protocol version 10.0 was approved following the completion of recruitment to the remaining original trial arms (B, C and E) and was a "housekeeping" change to remove references to the completed arms from the information sheets. Protocol version 11.0 was approved following the extension of the recruitment target sample for the “abiraterone comparison” from 1,500 to around 1,800 patients. Protocol

version 12.0 added a new combination therapy arm containing abiraterone with enzalutamide; for this comparison we envisage only two pre-planned interim analyses.

Patients will be assessed 6 weekly for the first 24 weeks after randomisation and then every 12 weeks up to 2 years, then 6-monthly until 5 years and annually, thereafter. The first 700 patients on trial completed questionnaires aimed at assessing the effects of the investigational treatments on their quality of life (QL) and on their use of health care resources (Health Economics (HE) study). From protocol version 8.0, the QL and HE study has been re-opened to all new patients.

In addition, there are translational sub-studies. Patients willing to participate will be asked at randomisation to donate a droplet of blood, which will be stored for DNA and protein analysis in order to try to identify markers that are associated with response to therapy, side-effects or susceptibility to prostate cancer.

Patients will also be asked to give permission to use some of their stored material (blood or biopsy samples) for further studies on the causes and nature of prostate cancer. In selected centres patients were asked to participate in a bone mineral density sub-study. This sub-study has now stopped recruitment. There are separate patient information sheets for the QL and HE study and the translational sub-studies (For further details of ancillary studies, see **Section 17**).

1.3 TRIAL DOCUMENTATION

Table 1 presents a summary of the required trial documentation for participating centres

Table 1: Trial documentation required for participating centres

TRIAL DOCUMENTATION	TIMING
R&D approval (including IRMER approval)	Before centre participation
Investigator Statement	Before centre participation
Signature list & delegation of responsibilities	Before centre participation
Trial personnel contact details	Before centre participation
PIS, GP & CF on local paper	Before centre participation
Signed Clinical Trial Agreement between Trust and Sponsor	Before centre participation
RTQA accreditation	Before centre participation
Clinical Trial Agreement (or Variation if applicable)	Before centre participation

2 BACKGROUND

2.1 INTRODUCTION AND SETTING

Prostate cancer is a major health problem world-wide and accounts for nearly one fifth of all newly diagnosed male cancers. In the UK, approximately 35,000 men are diagnosed with prostate cancer each year and in 2008 almost 10,000 men died from the disease.(2)

2.1.1 LONG-TERM ANDROGEN DEPRIVATION THERAPY

The initial (first line) treatment for locally advanced or metastatic prostate cancer is androgen deprivation therapy (ADT) achieved either surgically with bilateral orchidectomy, or medically with LHRH agonists or antagonists or oral anti-androgens alone. (3) Oral anti-androgens were permitted in the trial but were used by very few patients and are no longer permitted for new patients within the trial from version 8.0.

ADT produces responses in up to 95% of patients but it is not curative and disease recurs in virtually all patients treated with ADT as sole therapy, with a median time to progression of 18-24 months. (3) Such disease is referred to as hormone-refractory or, increasingly, as castrate resistant prostate cancer (HRPC or CRPC); this latter term is unpopular with patient groups due to its perceived pejorative overtones related to castration and hence terminology may yet change again in the future.

2.1.2 ROLE OF RADIOTHERAPY FOR PATIENTS WITH M0 DISEASE

Two randomised trials, SPCG7 (4) and NCIC PR.3 / MRC PR07 (5-7) have tested the question of whether androgen deprivation therapy alone or combined with radiotherapy is the best treatment for high-risk patients with no evidence of spread outside the pelvis. Both trials demonstrated an improvement in overall and disease specific survival from the addition of radiotherapy to androgen deprivation therapy. The size of this overall survival benefit is substantial (hazard ratio 0.68 in SPCG7 and HR 0.77 in PR07). With substantial benefit demonstrated in two mature, large, well conducted randomised trials, we now recommend that radiotherapy be considered standard for patients with no nodal or metastatic spread. Patients in this category will now only be allowed to enter the trial if standard radiotherapy is planned, with the exception of those for whom radiotherapy is contra-indicated. Such patients should be discussed with the Trials Unit prior to inclusion. For patients with node positive, M0 disease there are no clear data on whether radiotherapy is or is not indicated. The NCIC PR.3 / MRC PR07 trial included patients with unknown nodal status who received whole pelvic radiotherapy. Given the large overall benefit observed in this trial, the STAMPEDE TMG recommends that pelvic nodal radiotherapy be considered for patients with node positive, non-metastatic disease at the discretion of the treating clinician.

2.2 RATIONALE

There are increasing numbers of treatments which are used post relapse of first-line androgen deprivation therapy in patients with CRPC, but little evidence as to which is associated with the best response or how they may be combined or sequenced or whether any of them might have a role in first-line treatment. Such treatments include further hormonal manipulations, bisphosphonates, (8), cytotoxic chemotherapy (9), new hormone therapies (10) and palliative radiotherapy. The traditional approach to the testing and introduction of new treatments for prostate cancer is to use them in

patients with castrate resistant disease. An alternative approach is to investigate new drugs and new approaches to treatment, as first-line therapy in patients starting androgen deprivation therapy. At this point, patients should be fitter and better able to tolerate treatment than when they have CRPC and there is the possibility of having a larger and longer lasting effect.

2.3 DESIGN

STAMPEDE (also known as MRC PR08) is an innovative, multi-arm multi-stage, multi-centre, randomised controlled trial. It initially assessed the effects of a bisphosphonate (zoledronic acid), a cytotoxic chemotherapeutic agent (docetaxel) and a cyclooxygenase (Cox-2) inhibitor (celecoxib), as single agents or combinations, in patients commencing androgen deprivation therapy for advancing or metastatic prostate cancer. Each comparison is divided into five stages such that, for each investigational arm, safety and activity data are generated in the first four stages; an investigational arm will only proceed to the fifth and final stage of recruitment, where it will be assessed for its effect on overall survival, if it has been shown to be sufficiently safe and active at all prior activity stages. It is important to note, however, that patient data from all arms and all stages will be included in the final analyses of the primary outcome measure, even if the investigational arm did not proceed to the final stage.

Planned interim analysis failed to demonstrate sufficient activity for celecoxib and this agent has now been removed from the trial recruitment; patients remaining on celecoxib treatment reverted to standard care. Protocol version 8.0 added a new drug abiraterone to the study as an additional arm (see Section 2.7). Protocol version 9.0 added a new comparison arm involving prostate radiotherapy for patients with metastatic disease (see Section 2.8). Protocol version 10.0 reflected the successful completion of recruitment to three docetaxel- and bisphosphonate-containing arms (Arms B, C and E) and removed references to these agents in the information sheets for new patients. Protocol version 11.0 extended the recruitment target for the abiraterone research comparison (A vs G) from 1,500 to around 1,800 patients. Protocol version 12.0 added a new comparison involving the combination of abiraterone and enzalutamide. Protocol version 13.0 extended the recruitment target for the M1|RT comparison from 1,250 to around 1,800 patients.

2.4 RESEARCH TREATMENT AND BISPHOSPHONATES

Note: recruitment stopped to the zoledronic acid and docetaxel-containing arms in Mar-2013 at the end of Activity Stage IV.

The bisphosphonates are a class of drug that act by reducing osteoclast formation, inhibiting osteoclast activity and inducing osteoclast apoptosis. They are effective at controlling hypercalcaemia and preventing skeletal complications associated with malignant disease. (11, 12) Zoledronic acid is a highly potent, third generation bisphosphonate; studies comparing the efficacy of zoledronic acid to other bisphosphonates suggest that zoledronic acid has a 40-850 fold higher potency than clodronate in preclinical models of bone resorption. (13). It has also been shown to be more effective than pamidronate (90mg) in controlling malignant hypercalcaemia. (14) In addition, zoledronic acid has also demonstrated direct anti-cancer activity, including inhibition of proliferation of breast cancer and prostate cancer cells in vitro. (15)

In randomised controlled trials of 1,648 patients, 4mg zoledronic acid was more effective than pamidronate in reducing the risk of skeletal complications in patients with bone metastases from breast cancer. (16, 17) Also, in metastatic prostate cancer, zoledronic acid has been shown to reduce the rate of skeletal-related events compared to placebo in a trial involving 429 men. (18) In April 2002, zoledronic acid received approval from

the Committee for Proprietary Medicinal Products for the prevention of skeletal-related events (for example, fractures) in patients with any advanced malignancies involving bone.

The MRC PR05 prostate cancer trial showed that a first generation bisphosphonate (clodronate) commenced at the time of androgen deprivation therapy initiation, delayed time to progression in patients with bony metastatic disease and there was some evidence that it may also improve survival. (19) There is, therefore, a good rationale for investigating a more potent bisphosphonate in patients with prostate cancer who are about to commence ADT therapy.

2.5 RESEARCH TREATMENT: CHEMOTHERAPY

Note: recruitment stopped to the zoledronic acid and docetaxel-containing arms in Mar-2013 at the end of Activity Stage IV.

There is increasing evidence of the clinical efficacy of chemotherapy in prostate cancer. (9) Two randomised phase III trials in patients with metastatic hormone refractory prostate cancer (HRPC) using a docetaxel-containing regimen have been completed: the SWOG 9916 study (20) and the TAX-327 study. (21) Both studies show that the use of a docetaxel-based regimen improved survival for patients with metastatic HRPC and had significantly greater PSA response rates compared to the mitoxantrone plus prednisolone arm.

In the TAX-327 trial, (21) 1,006 patients with metastatic HRPC were randomized to receive either mitoxantrone 12 mg/m² with prednisone 10mg daily (Arm C) or docetaxel 75mg/m² 3-weekly for 10 cycles with prednisone (Arm A) or docetaxel 30 mg/m²/wk x 5 of 6 weeks x 5 cycles with prednisone (Arm B). Median overall survival was 16.5 months for patients treated with mitoxantrone versus 18.9 months for the 3-weekly docetaxel regimen (hazard ratio 0.76 (0.62-0.94)). There was also improvements for 3-weekly docetaxel in pain (22% vs 35%, p = 0.01) and PSA response (32% vs 45%, p=0.0005).

In June 2006 in the UK docetaxel was given NICE (National Institute for Health and Clinical Excellence) approval for use in hormone (now more commonly termed castrate) refractory prostate cancer patients.

2.6 RESEARCH TREATMENT: CYCLOOXYGENASE-2 INHIBITORS

Note: recruitment completed to both celecoxib-containing arms in Apr-2011 at the end of Activity Stage II of this comparison

Cyclooxygenase-2 (Cox-2) is an isoenzyme induced by a variety of mitogens, cytokines and growth factors that are associated with a range of process including inflammation, (22) and carcinogenesis.(23, 24) There is a growing body of evidence that inhibition of Cox-2 may play an important role in the prevention of cancer and the delay of progression in established cancer. A number of case-control studies have shown a reduction in risk of prostate cancer associated with the use of non-steroidal anti-inflammatory drugs (NSAID), which include inhibition of Cox-2 amongst their mode of action. (25) Pathological studies show Cox-2 is upregulated in carcinomas (26) and one study suggested that NSAID use may delay progression from subclinical to clinical prostate cancer. (27)

Celecoxib, a Cox-2 inhibitor, is better tolerated than other NSAIDs and there is evidence that it is active as a chemoprevention agent. (28) It also has important antineoplastic properties such as the ability to inhibit angiogenic factors and induce apoptosis in human cancer cells including prostate cancer. (29)

Evidence has suggested that an anti-cancer effect is only seen at higher doses of celecoxib than **is** required for an anti-inflammatory effect. (30) Therefore, the dose of 800mg/day for STAMPEDE patients has been chosen. Although there is some high profile evidence of a small absolute increase in CVS toxicity risk associated with higher doses of celecoxib, (31) most current cancer trials are using a dose of 800mg/day as it is believed that a higher dose will result in a greater increase in cancer effect.

There is also some evidence of a schedule effect on CVS toxicity. It has been observed that CVS toxicity becomes evident after one year of taking celecoxib. (31) Therefore, a maximum duration of one year has been set for celecoxib use in this trial. Any potential risks of course have to be weighed against any potential benefits of celecoxib in the delay of progression in established prostate cancer.

Given case-control data suggesting effects on prostate cancer, pathological expression of Cox-2 in prostate cancer and in vitro data suggesting that inhibition of Cox-2 inhibits growth and invasiveness, further investigation in prostate cancer is warranted.

2.7 RESEARCH TREATMENT: STEROID SYNTHESIS INHIBITORS

Recent evidence suggests that an important mechanism for escape from tumour control by androgen ablation is the intracellular conversion of steroid precursors to androgenic steroids by prostate cancer cells. A key enzyme in this process is CYP17, which therefore represents a logical target for therapy in CRPC. (10) Abiraterone acetate is a selective inhibitor of CYP17 and is highly active in patients developing resistance to standard androgen ablation therapies. (32-34) Recruitment to a phase III study comparing abiraterone acetate to placebo in CRPC patients post-docetaxel, completed accrual in 2009 and reported initial results in 2011 with an improvement in overall survival of around 4 months and a hazard ratio of 0.65. (35) The drug has now received a marketing authorisation in the USA and in the EU from September 2011. A second trial in pre-chemotherapy CRPC patients completed recruitment April 2010; preliminary results are positive and were published in 2012 (36) and the licence for abiraterone was extended to the pre-chemotherapy CRPC population in Europe in 2012. Side-effects with abiraterone acetate are modest with the main adverse effects being elevated transaminases (usually mild), hypokalaemia and hypertension due to secondary hyperaldosteronism and fluid retention (preventable by low doses of glucocorticoids). In order to prevent secondary hyperaldosteronism, it is recommended that prednisolone (or prednisone) 10mg daily be administered in the CRPC setting. Within more recent studies in earlier stage patients, lower doses (typically 5mg of prednisone/prednisolone) are being used due to concerns about long-term exposure to glucocorticoid side effects. More recent evidence even suggests that for most patients, no glucocorticoids may be needed. (37) Within the STAMPEDE trial, we suggest prednisolone/ prednisone dose of 5mg daily.

We hypothesise that the agent may be more active still when given up-front in combination with first-line androgen deprivation therapy by preventing or delaying the development of castrate refractory disease.

2.8 RESEARCH TREATMENT: RADIOTHERAPY TO THE PROSTATE FOR PATIENTS WITH NEWLY-DIAGNOSED METASTATIC DISEASE

Therapy directed against the primary tumour in the presence of metastatic disease has been evaluated rigorously in only one malignancy to date: renal cell carcinoma. Two cooperative groups ran randomised trials enrolling patients with previously untreated metastatic RCC whose primary tumours were amenable to surgical resection. Patients were randomised to receive the standard systemic therapy of the day, interferon-alpha, either alone or with radical nephrectomy. The combination of nephrectomy and interferon was shown to significantly improve median survival from 7 to 17 months in one trial (38) and from 8 to 11 months in the other.(39) The mechanism by which nephrectomy improves survival remains obscure. In preclinical models, the primary tumour has been found to secrete molecules that prime the microenvironment in which metastases can develop. An implication of this work is that therapy directed at the primary tumour, by abrogating this endocrine signalling, could retard the formation and the growth of distant metastases.

The results of two large-scale randomised trials of prostate radiotherapy are also provocative. The Scandinavian SPCG-7 trial and the MRC PR07 trial randomised men with locally advanced prostate cancer, who were at high risk of possessing occult metastatic disease, to either androgen deprivation therapy (ADT) alone or ADT plus prostate radiotherapy.(4, 40) The addition of radiotherapy dramatically improved 10-year outcomes: mortality from prostate cancer was halved. Interestingly, the benefit of radiotherapy started to emerge as early as three years from the time of randomisation. This seems improbably early if the benefit of local treatment is mediated via the prevention of subsequent disease dissemination. Rather, it is more consistent with the possibility that local treatment has a beneficial impact on the rate of progression of existing micrometastatic disease.

We hypothesise that local therapy to the primary site may retard distant disease progression and prolong survival in patients with metastatic prostate cancer.

Figure 8: Use of RT in STAMPEDE

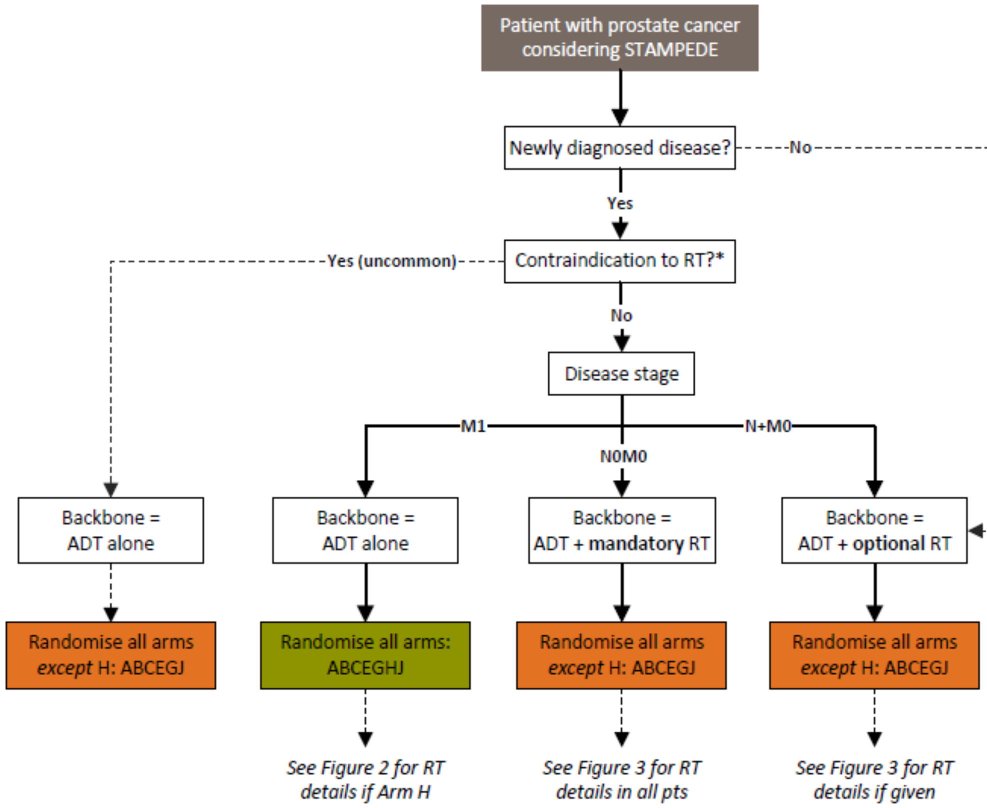

*It is expected that only around 1% of patients will have a contraindication to RT e.g. inflammatory bowel disease. These cases should be discussed with the trials unit prior to randomisation (see **Section 4.3**).

2.9 RESEARCH TREATMENT: COMBINATIONS OF ORIGINAL RESEARCH ARMS

2.9.1 BISPHOSPHONATE AND CHEMOTHERAPY

Note: recruitment stopped to the zoledronic acid and docetaxel-containing arms in Mar-2013 at the end of Activity Stage IV.

Zoledronic acid and docetaxel have different mechanisms of action. In addition to its skeletal protection activity, zoledronic acid has shown direct activity against prostate cancer cells, both in vitro and in vivo. (15) There is also in vitro and in vivo evidence to suggest synergy between zoledronic acid and chemotherapy in breast cancer cells and anti-angiogenic effects in patients. (41, 42)

Toxicities of the two agents are complementary and administration in combination is expected to be feasible and safe. These aspects were evaluated in the initial Pilot Phase of the trial. Since both agents show considerable promise as single agents and there is in vitro evidence of synergy, we believe there is a strong rationale for evaluating these two agents in combination.

2.9.2 BISPHOSPHONATE AND CYCLOOXYGENASE-2 INHIBITORS

Note: recruitment stopped to both celecoxib-containing arms in Apr-2011 at the end of Activity Stage II.

An alternative approach to combination therapy is to target the principal site of relapse and a key mode of progression and this is the rationale for combining zoledronic acid with a Cox-2 inhibitor. Bisphosphonates have already been shown to delay bone disease progression in hormone refractory disease. (19) Cox-2 appears to play a crucial role in the molecular phenotype of advanced prostate cancer as outlined above, and this effect is likely to be apparent in both soft tissue and in bone. Toxicities of the two agents are likely to be complementary and there is no strong a priori reason to anticipate unacceptable toxicity. The Pilot Phase of the trial will evaluate tolerability and safety of the combination. Targeting both bone progression and the underlying molecular changes leading to progression can be expected to have synergistic benefits in terms of delaying development of hormone refractory disease.

2.10 COMBINATION OF STEROID SYNTHESIS INHIBITORS AND ANDROGEN RECEPTOR SIGNALLING INHIBITOR

The majority of patients with advanced prostate cancer who have disease progression on abiraterone or enzalutamide taken as single agents, have a rise in PSA, suggesting reactivation of androgen receptor (AR), or other steroid signalling pathways resulting in increased PSA transcription, is the pathway to the development of resistance.(43)

The question under investigation is: can progression be delayed (and survival extended) by using a combination of abiraterone and enzalutamide?

2.10.1 Supplementing Abiraterone And Prednisolone With Enzalutamide

Several studies have shown that the AR can become promiscuously activated by very low levels of androgens or other steroid metabolites and drugs that bind the AR.(44-47) It is known that very low levels of androgens can persist in patients treated with abiraterone acetate.(48) Drugs that bind the AR, may include co-administered glucocorticoids. Furthermore, AR mutations of the sort previously described in castration-resistant prostate cancer (CRPC), can be activated by cortisol and other glucocorticoids at levels much lower than those reported in patients treated with abiraterone and prednisolone at a dose of 5mg bid.(47, 49) Moreover, abiraterone binds the AR and, although weak antagonism of wild-type and most previously described AR mutations are observed,(49) a similar mechanism to that described with classical anti-androgens, such as bicalutamide, could lead to change-of-function AR mutations associated with AR activation following abiraterone binding. Therefore, concomitant treatment with an androgen receptor signalling inhibitor could prevent “promiscuous” AR activation in patients treated with abiraterone. Enzalutamide is a androgen receptor signalling inhibitor and has gained recent approval for use on its own in the treatment of advanced CRPC,(50) and there is evidence of activity for hormone-naïve prostate cancer.(51)

2.10.2 Supplementing Enzalutamide With Abiraterone And Prednisolone

Enzalutamide in combination with ADT is effective and well tolerated in CRPC.(50) However, recent studies have suggested that intratumoral testosterone levels increase in patients treated with enzalutamide.(52) The implications of this finding are that the increase in intratumoral testosterone could be associated with up-regulation of enzymes involved in steroid biosynthesis.(53) Although enzalutamide has a high affinity for the AR, this is several-fold lower than both the natural ligands testosterone and DHT,(54) which means that enzalutamide would be out-competed at the AR ligand-binding domain if and when androgen levels rise. In vitro, a ten-fold rise in intra-cellular androgen was sufficient to prevent inhibition of AR by 30uM of enzalutamide;(49) these levels are representative of the plasma levels of enzalutamide active metabolites, which can be achieved with enzalutamide 160mg po daily.(55)

A strategy for preventing the rise in intra-cellular androgens in patients treated with enzalutamide would be inhibition of CYP17A1. Abiraterone is currently the only CYP17A1 inhibitor with proven efficacy. It therefore seems logical to use the combination of enzalutamide and abiraterone to both block a rise of intra-cellular androgens and prevent promiscuous activation of the AR.

2.10.3 Summary Of Rationale For This Combination

To date, investigation has focussed on patients with CRPC but there is a strong rationale for the combination of enzalutamide and abiraterone in the hormone treatment-naïve setting in which STAMPEDE is focused.

STAMPEDE already has an abiraterone plus conventional ADT arm but we will not assess the combination of conventional ADT plus enzalutamide; other trials by industry and other cooperative groups will address that question. The inclusion of an arm with ADT and enzalutamide in STAMPEDE was therefore considered to be a duplication of effort and was not supported by the Trial Management Group.

The combination of enzalutamide and abiraterone is a novel approach and offers considerable promise in delaying progression – it therefore represents an attractive addition to the comparisons under investigation in STAMPEDE, and one that is unlikely to be replicated in other planned trials of this size.

3 SELECTION OF INSTITUTIONS AND INVESTIGATORS

Centres who wish to participate in the STAMPEDE trial should be registered with the Medical Research Council Clinical Trials Unit at University College London (MRC CTU at UCL) for this purpose. Before any patients are randomised the MRC CTU must receive a completed and signed Investigator Statement. The STAMPEDE investigator statement is signed by the Principal Investigator for that institution (**Appendix M**). R&D approval for the site, along with a fully-signed model agreement, are also required before recruitment can begin.

In addition, and in compliance with the principles of GCP, all institutions participating in the trial will complete a delegation log and forward this to the MRC CTU. Each person working on the STAMPEDE trial must sign off a section of this log indicating their responsibilities. The MRC CTU must be notified of any changes to trial personnel and/or their responsibilities. An up-to-date copy of this log must be stored in the Investigator Site file at the institution and also at the MRC CTU.

The Clinical Trial Authorisation (CTA) for the STAMPEDE trial requires that the Medicines and Healthcare Products Regulatory Agency (MHRA) be supplied with the names and addresses of all participating investigators/institutions. Trial staff at the MRC CTU will perform this task; hence, it is vital to receive full contact details for all investigators prior to their entering patients.

Finally, before a patient is entered into the trial written informed consent must be obtained. Approved patient information sheets and informed consent forms are supplied as templates.

Only a limited number of centres participated in the initial Pilot Phase of the original trial; this was to ensure that safety and feasibility data were collected expediently. Subsequent stages of the trial are open to any centre that wishes to participate and has fulfilled the requirements described above.

3.1 RADIOTHERAPY ACCREDITATION

The introduction of the RT comparison in Protocol 9.0 introduced the need for RTQA accreditation in sites giving radiotherapy. The detail of RTQA accreditation is in **Appendix K**. However, centres that have been RTQA accredited for another multi-centre prostate radiotherapy trial in the UK (e.g. RADICALS or CHHIP) will be automatically granted STAMPEDE RTQA accreditation.

4 SELECTION OF PATIENTS

4.1 PATIENT INCLUSION CRITERIA

Patients must fulfil both of the criteria in **Section 4.1.1** **or** one criterion in **Section 4.1.2** **or** at least one criterion in **Section 4.1.3**. Additionally, all patients must fulfil the criteria in **Section 4.1.4**.

4.1.1 HIGH-RISK NEWLY-DIAGNOSED NON-METASTATIC NODE-NEGATIVE DISEASE

Both:

- At least two of: Stage T3/4, PSA≥40ng/ml or Gleason sum score 8-10
- Intention to treat with radical radiotherapy (unless there is a contra-indication; exemption can be sought in advance of consent, after discussion with MRC CTU)

OR

4.1.2 NEWLY-DIAGNOSED METASTATIC OR NODE-POSITIVE DISEASE

At least one of:

- Stage T_{any} N+ M0
- Stage T_{any} N_{any} M+

OR

4.1.3 PREVIOUSLY TREATED WITH RADICAL SURGERY AND/OR RADIOTHERAPY, NOW RELAPSING[1]

At least one of:

- PSA ≥4ng/ml and rising with doubling time less than 6 months
- PSA ≥20ng/ml
- N+
- M+

AND

4.1.4 FOR ALL PATIENTS

I. Histologically confirmed prostate adenocarcinoma
II. Intention to treat with long-term androgen deprivation therapy
III. Fit for all protocol treatment[2] and follow-up, WHO performance status 0-2[3]
IV. Have completed the appropriate investigations prior to randomisation
V. Adequate haematological function: neutrophil count >1.5x10^9/l and platelets >100x10^9/l
VI. Estimated creatinine clearance >30ml/min
VII. Serum potassium ≥3.5mmol/L
VIII. Written informed consent
IX. Willing and expected to comply with follow-up schedule
X. Using effective contraceptive method if applicable

---

[1] Courses of hormone therapy for localised disease must have been completed at least 12 months previously and have been no longer than 12 months in duration. It can have been given as adjuvant or neoadjuvant therapy.
[2] Medical contraindications to the trial medications are given in **Appendix G**
[3] For WHO performance status definitions see **Appendix A**

4.2 PATIENT EXCLUSION CRITERIA[4]

Patients must not fulfil any of the criteria, below.

I. Prior systemic therapy for locally advanced or metastatic prostate cancer except as listed in **Section 4.1.3**
II. Metastatic brain disease or leptomeningeal disease
III. Abnormal liver functions consisting of any of the following:
  - Serum bilirubin ≥1.5 x ULN (except for patients with Gilbert's disease, for whom the upper limit of serum bilirubin is 51.3µmol/l or 3mg/dl)
  - Aspartate aminotransferase (AST) or alanine aminotransferase (ALT) ≥2.5 x ULN
IV. Any other previous or current malignant disease which, in the judgement of the responsible physician, is likely to interfere with STAMPEDE treatment or assessment
V. Patients with contra-indications to prednisolone, including active peptic ulceration or a history of gastrointestinal bleeding
VI. Patients with active inflammatory bowel disease
VII. Symptomatic peripheral neuropathy grade ≥2 (NCI CTC)[5]
VIII. Any surgery (e.g. TURP) performed within the past 4 weeks
IX. Patients with significant cardiovascular disease such that, in the investigator's opinion, the patient is unfit for any of the study treatments. This might include:
  - Severe/unstable angina
  - Myocardial infarction less than 6 months prior to randomisation
  - Arterial thrombotic events less than 6 months prior to randomisation
  - Clinically significant cardiac failure requiring treatment (NYHA II-IV)[6]
  - Cerebrovascular disease (e.g. stroke or transient ischaemic episode) less than 2 years prior to randomisation
  - Patients with uncontrolled hypertension defined as systolic BP greater or equal than 160 mmHg or diastolic BP greater or equal than 95 mmHg
X. Patients receiving treatment with drugs known to induce CYP3A4 (including phenytoin, carbamazepine, Phenobarbital)[7]
XI. Prior exposure to abiraterone
XII. Prior exposure to enzalutamide
XIII. Prior chemotherapy for prostate cancer
XIV. Prior therapy with zoledronic acid or other bisphosphonates other than treatment for hypercalcaemia or low bone density
XV. Prior exposure to policy of long-term hormone therapy before randomisation (unless as described in **Section 4.4.2**)
XVI. History of seizure including any febrile seizure, loss of consciousness, or transient ischaemic attack within 12 months of randomisation or any condition that may pre-dispose to seizure (e.g., prior stroke, brain arteriovenous malformation, head trauma with loss of consciousness requiring hospitalization)
XVII. Unexplained history of loss of consciousness within 12 months of randomisation
XVIII. Operation of heavy machinery during treatment

---

[4] The exclusion criteria for patients who have been on a Cox-2-inhibitor for 6+ months has been removed
[5] See **Appendix I** for common toxicity grading
[6] NYHA classifications can be found in **Appendix A**
[7] A full list is included in **Appendix G**

4.3 SELECTION CRITERIA FOR COMPARISON OF RESEARCH (M1) RT FOR METASTATIC DISEASE

All patients meeting criteria in **Section 4.1** and **4.2** are eligible for the trial, but not all can be allocated to the research (M1) radiotherapy arm. The selection criteria for this “RT to the prostate” comparison are:

- Newly-diagnosed prostate cancer
- Demonstrable M1 disease
- No contraindication to radiotherapy e.g. no previous pelvic radiotherapy and no history of inflammatory bowel disease
- No previous radical prostatectomy

Any patients meeting these criteria will have a chance to be allocated to Arm H. For those rare cases where radical RT is planned for a newly-diagnosed M1 patient, the TMT and TMG will need to review and approve the inclusion of the patient for randomisation only between Arm A and J.

4.4 SCREENING PROCEDURES

4.4.1 INVESTIGATIONS PRIOR TO RANDOMISATION

All patients should have the following examinations performed. The latest available scans should be used:

- CT or MRI of pelvis and abdomen
- Bone Scan (or equivalent e.g. whole body MRI)
- Chest X-ray (only if chest was not included in CT)
- ECG
- PSA Test

The following blood tests within 8 weeks (56 days) prior to randomisation:

- Testosterone (if available)
- Urea and Electrolytes
- Liver function tests
- Serum creatinine
- Serum corrected calcium
- Phosphates
- Magnesium
- Albumin
- Total cholesterol
- HDL cholesterol
- Systolic blood pressure
- Diastolic blood pressure
- Waist circumference measure

Patients who initially fail to meet the eligibility criteria can be re-screened at a later date.

Prior to randomisation:

- Check details of any prior treatments for prostate cancer
- Check any contraindications to radiotherapy

4.4.2 Androgen Deprivation Therapy Prior To Randomisation

It is preferable that patients are not started on hormones prior to randomisation. However, if androgen deprivation therapy has already started, the primary therapy should have not have started more than 12 weeks before randomisation, and the baseline PSA measurement must be taken before this was initiated (please report the latest PSA measurement taken before the start of androgen deprivation therapy).

Short periods of prior anti-androgens to cover tumour flare are allowed but will not be counted in the 12 week time period mentioned above; but a PSA measurement must be taken before this is initiated. The start date of anti-androgens cannot be more than 14 weeks before randomisation and patients will not be eligible if anti-androgen therapy has exceeded 14 weeks.

Note that long-term anti-androgen monotherapy is not permitted in the trial for newly recruited patients from protocol version 8.0 (see **Section 6.1**); patients may change treatment to join the trial, provided that they have not had more than 12 weeks of androgen deprivation therapy prior to randomisation. Further details on hormone therapies allowed prior to randomisation are discussed in **Appendix L**.

Any relapsing patients treated with adjuvant or neo-adjuvant hormone therapy alongside their radical surgery or radiotherapy must have completed that period of hormone therapy at least 12 months before joining STAMPEDE and it must have been no longer than 12 months in duration.

Note that baseline testosterone measurements will not be required in patients who have already commenced hormone manipulation prior to randomisation.

4.4.3 Hypercalcaemia At Randomisation

For patients who are hypercalcaemic prior to randomisation and require treatment, it is recommended that they are treated with a bisphosphonate and that the treatment should be discontinued when they are stabilised.

4.4.4 NSAIDs And Cox-2 Inhibitors At Randomisation

Note: recruitment completed to both celecoxib-containing arms in Apr-2011 at the end of Activity Stage II

For patients who are currently on a Cox-2-inhibitor and who meet the inclusion criteria, please ensure that treatment is discontinued before randomisation. If the patient is allocated to an arm, which does not include celecoxib (arms A, B, C or E), it is advised that the Cox-2 be replaced with a suitable NSAID.

For patients who are taking an NSAID prior to randomisation and are allocated a celecoxib arm (Arm D or F), a clinical decision should be taken as to whether the patient should continue taking the NSAID alongside the celecoxib. This decision should take into account the risk of gastrointestinal problems, and consideration should be given to the co-administration of a proton pump inhibitor

4.4.5 Starting Trial Treatment

Trial treatment should be commenced as soon as possible after randomisation. Investigators should aim that this is at least within 4 weeks post randomisation and within 12 weeks of starting androgen Deprivation Therapy (see Section 6).

Radiotherapy for patients allocated to Arm H should be commenced within 4 weeks from randomisations and continued according to the predefined scheduled unless toxicity is reported. Any

delays in starting research radiotherapy should be discussed with the STAMPEDE team and recorded as appropriate in the relevant CRF.

4.4.6 Concomitant Medications

All concomitant medications should be recorded including any vitamin and mineral supplements the patient is taking, regular consumption of NSAID and/or aspirin and use of other bisphosphonates (see **Section 4.2**). Of particular interest in this are herbal preparations such as PC-SPES, Prostasol, Saw Palmetto and St John's Wort. All concomitant medications should be continued throughout the trial unless the responsible clinician decides otherwise.

4.5 ADDITIONAL DETAILS FOR PATIENTS JOINING SUB-STUDIES

If the patient has given their consent to participate in the DNA analysis sub-study, a saliva sample will be collected. This replaces the droplet of blood collected in previous versions of the protocol.

The local pathologist will also be asked to give the tumour sample remaining after primary interrogation for tissue micro array analysis to be carried out, if the patient has given consent for his remaining samples to be used for further analyses. Full details of all sub-studies and instructions relating to the handling of the blood sample are given in **Section 17** and **Appendix D**.

5 RANDOMISATION AND ENROLMENT

Patients will be allocated to any of the open research arms for which they are suitable. Patients with non-metastatic disease or who have had previous local therapy to the prostate or who have a contraindication to radiotherapy will not be allocated to Arm H (see **Section 4.3**).

To enter a patient the randomisation form should be completed carefully and the MRC CTU contacted by phone:

RANDOMISATIONS
To randomise, call MRC CTU at UCL, Monday to Friday 0900-1700
excluding public holidays or dates when notice has been given by the CTU.
Tel: +44 (0) 20 7670 4777

A trial number and treatment will be allocated and given over the phone or by return fax. In addition, a letter confirming these details will be sent. The trial number will be the primary way in which the patient will be identified and should be used in all correspondence.

5.1 CO-ENROLMENT GUIDELINES

Ideally, patients should not be participating in any other clinical trial of prostate cancer treatment when they enter STAMPEDE and should not enter any other trials until the patient has had a failure-free survival (FFS) event reported. After this point, the patient may be entered into further, second-line treatment studies. The primary outcome measure of STAMPEDE is overall survival. Participation in post-progression studies should be reported on the Co-enrolment CRF.

Data sharing agreements with “down-stream” trials are encouraged to improve data quality in both trials and to reduce costs to both organisations.

6 TREATMENT OF PATIENTS

6.1 TRIAL TREATMENT

Patients will be randomised to the control arm (Arm A) or one of the research arms. All patients will receive androgen deprivation therapy (ADT) to achieve castration levels of testosterone. The method of ADT is a local choice but must be specified for each patient prior to randomisation. The recommended methods of ADT are given in **Section 6.1.1**. All trial treatments should commence as soon as practically possible after randomisation. Patients having a bilateral orchidectomy should commence any additional treatment within 12 weeks of the operation unless there is a strong clinical reason not to do so. Note that from protocol version 8.0 onwards, bicalutamide monotherapy is no longer permitted as a trial therapy for new patients (but patients may switch to a permitted therapy to join the trial – see **Section 4.3.2**).

6.2 ARM A: ADT ALONE OR ADT + STANDARD-OF-CARE (M0) RT (CONTROL ARM)

The standard of care for this patient group is **androgen deprivation therapy** (see **Section 6.2.1**). For some patient groups, this should now be supplemented with standard radiotherapy (see **Section 6.2.2**).

6.2.1 HORMONE THERAPY

The permitted methods of ADT are bilateral orchidectomy, LHRH analogues and LHRH antagonists. Anti-androgens alone are not permissible as hormone therapy for patients participating in STAMPEDE, but their use is recommended in the short-term to prevent tumour "flare" which may occur after commencing LHRH analogues. Anti-androgen prophylaxis of tumour flare is not required when using LHRH antagonists. At the time of randomisation, centres will be asked to specify the method of ADT for each patient. Other methods of ADT should be discussed with the Chief Investigator or the Trial Surgeon. The planned duration of ADT should be at least 2 years.

Bilateral orchidectomy: Operations should be performed by appropriately trained surgeons. A total or subcapsular orchidectomy may be performed.

LHRH agonists: LHRH agonists/analogues used according to local practice. The prophylactic use of anti-androgens to prevent tumour "flare" is recommended.

LHRH antagonists: LHRH antagonists used according to local practice. The use of prophylactic use of anti-androgens to prevent tumour "flare" is not necessary.

6.2.2 STANDARD-OF-CARE (M0) RT

N0M0 patients: Investigators should give standard radiotherapy (RT) to patients with node negative, non-metastatic disease (N0M0), in accordance with the data from the PR07 and SPCG trials. If there is an intention to omit radiotherapy (e.g contraindications) in patients with N0M0 disease this must be discussed with the Trials Office before consent. See **Section 6.6** for further details of radiotherapy administration.

N+M0 patients: the benefit of radiotherapy in this group is at present uncertain with no firm data to either support or refute its use. However, the PR07 trial included some node positive patients as

cross sectional imaging was not a part of the baseline assessment in this trial, which did include whole pelvis radiotherapy. For patients with node positive, non-metastatic disease, radiotherapy is therefore recommended in suitable cases. Investigators will be asked to state their intention with regards to planned radiotherapy in this group at randomisation. Intention to give radiotherapy (or not) for node positive patients must be stated at randomisation to ensure that there is no bias towards particular combinations of systemic therapy with radiotherapy.

Standard radiotherapy is not a core part of the trial, therefore we intend to collect minimal data about the radiotherapy administered. It is accepted that some patients will develop progressive disease before radiotherapy can be administered and if this occurs the reasons for non-delivery of treatment must be recorded on the radiotherapy form.

6.3 ARM B: ADT + ZOLEDRONIC ACID

Note: recruitment stopped to the zoledronic acid and docetaxel-containing arms in Mar-2013 at the end of Activity Stage IV.

Androgen deprivation therapy (+/- standard-of-care M0 RT) as described in **Section 6.2.1**.

Zoledronic Acid: 4mg 15min IV infusion every 3 weeks, for 6 treatments followed by zoledronic acid 4mg 15min IV infusion every 4 weeks up to a maximum of 2 years from the start of the treatment or until disease (including PSA) progression (see **Section 7.2**). Patients should also receive an oral supplement of 500mg calcium and 400IU vitamin D daily. These doses are available as a combination tablet. See **Section 6.6** for further information.

6.4 ARM C: ADT + DOCETAXEL

Note: recruitment stopped to the zoledronic acid and docetaxel-containing arms in Mar-2013 at the end of Activity Stage IV.

Androgen deprivation therapy (+/-standard-of-care M0 RT) as described in Section 6.2.1.

Docetaxel: 75mg/m2 Day 1 as 1hr IV infusion, plus prednisolone 5mg bid daily for 21 days. The cycle should be repeated every 3 weeks for a maximum of 6 cycles. The recommended administration schedule, anti-emetic regimen and dose modifications for docetaxel are given in Appendix F. See Section 6.2.2 for further information.

6.5 ARM D: ADT + CELECOXIB

Note: recruitment completed to both celecoxib-containing arms in Apr-2011 at the end of its Activity Stage II

Androgen deprivation therapy as described in Section 6.2.1.

Celecoxib 400mg bid until the sooner of 1 year or disease (including PSA) progression (see Section 7.2). See Section 6.2.3 for further information.

6.6 ARM E: ADT + DOCETAXEL + ZOLEDRONIC ACID

Note: recruitment stopped to the zoledronic acid and docetaxel-containing arms in Mar-2013 at the end of Activity Stage IV.

Androgen deprivation therapy (+/- standard-of-care M0 RT) as described in **Section 6.2.1**.

Docetaxel: 75mg/m2 Day 1 as 1hr IV infusion, plus prednisolone 5mg bid daily for 21 days. The cycle should be repeated every 3 weeks for a maximum of 6 cycles. The recommended administration schedule, anti-emetic regimen and dose modifications for docetaxel are given in Appendix F. See Section 6.4 for further information.

Zoledronic Acid: 4mg 15min IV infusion every 3 weeks, for 6 treatments followed by zoledronic acid 4mg 15min IV infusion every 4 weeks up to a maximum of 2 years from the start of the treatment or until disease (including PSA) progression (see **Section 7.2**). Patients should also receive an oral supplement of 500mg calcium and 400IU vitamin D daily. These doses are available as a combination tablet. See **Section 6.3** for further information.

Co-administration of docetaxel and zoledronic acid: Docetaxel 75mg/m2 Day 1 as 1hr IV infusion, plus prednisolone 5mg bid daily followed by zoledronic acid 4mg 15min IV infusion. There is evidence to suggest that the co-administration of docetaxel and zoledronic acid is sequence dependent.(42) Consequently, docetaxel should be administered before zoledronic acid

6.7 ARM F: ADT + ZOLEDRONIC ACID + CELECOXIB

Note: recruitment completed to both celecoxib-containing arms in Apr-2011 at the end of Activity Stage II

Androgen deprivation therapy as described in **Section 6.2.1**.

Zoledronic Acid 4mg 15min IV infusion every 3 weeks, for 6 treatments followed by zoledronic acid 4mg 15min IV infusion every 4 weeks up to a maximum of 2 years from the start of the treatment or until disease (including PSA) progression (see Section 7.2). Patients should also receive an oral supplement of 500mg calcium and 400IU vitamin D daily (Calcichew). These doses are available as a combination tablet. See **Section 6.3** for further information.

Celecoxib 400mg bid until the sooner of 1 year or disease (including PSA) progression (see Section 7.2). See Section 6.5 for further information.

6.8 ARM G: ADT + ABIRATERONE

Note: recruitment to the "abiraterone comparison" completed in January 2014. Please note that some patients will continue treatment until progression or up to a maximum of 2 years. Please see sections below for more information

Androgen deprivation therapy (+/- standard-of-care M0 RT) as described in **Section 6.2.1.**

Abiraterone will be administered as a single 1000mg daily oral dose (4 tablets to be taken together once a day) together with prednisolone or prednisone 5mg daily to prevent secondary mineralocorticoid excess. Abiraterone absorption is increased by food. The tablets should be taken at least 2 hours after food, swallowed whole with some water. No food should be eaten for 1 hour afterwards.

Prednisolone (prednisone in Switzerland) should be taken as a single dose with food in the morning.

In patients with M1 disease, treatment with abiraterone will continue from randomisation until clinical disease progression, consistent with the COU-AA-301 trial (35) i.e., abiraterone would be given for these patients until a composite of PSA progression (as defined in **Appendix J**), radiological progression (appearance of new lesions or progression of existing lesions) and clinical progression (defined as new cancer-related symptoms). It is accepted that these flexible criteria for stopping treatment with abiraterone are open to the investigator's interpretation and discretion. Patients might continue treatment beyond the first failure-free survival (FFS) event (see Table 1 in **Section 9.2**); the first FFS event must be reported as per the other arms.

In patients with N0M0 disease or N+M0 disease undergoing radical radiotherapy, treatment would continue for 2 years or disease progression as defined for M1 patients, whichever is the sooner. ADT can be discontinued in this group at 2 years at the discretion of the local investigator (see **Section 6.2.1**).

For patients with N+M0 disease not planned for radical radiotherapy, treatment will continue as for patients with M1 disease until disease progression.

If a patient allocated to Arm G develops only biochemical failure, the responsible clinician might switch from abiraterone + prednisolone 5mg od to abiraterone and dexamethasone 0.5mg od.

Trial treatment must stop if other systemic treatments are initiated at any time for disease progression control (including the addition or swapping of anti-androgens, chemotherapy etc).

See **Section 6.2.4 and 6.2.6** for further information for all groups.

6.9 ARM H: ADT + PROSTATE RADIOTHERAPY IN M1 PATIENTS

Androgen deprivation therapy as described in **Section 6.2.1**.

Radiotherapy will commence as soon as practicable and ideally within four weeks after randomisation. Treatment will be according to the guidelines in **Section 6.11.5**. Two radiotherapy dose-fractionation schedules are permitted:
- 36Gy in 6 fractions of 6Gy, administered weekly over 6 consecutive weeks
- 55Gy in 20 fractions of 2.75Gy, administered daily, five days per week, over 4 consecutive weeks

Details of the recommendations for outlining, CTV and PTV are in **Section 6.11.5**.

6.10 ARM J: ADT + ABIRATERONE + PREDNISOLONE + ENZALUTAMIDE ADMINISTRATION

Androgen deprivation therapy (+/- standard-of-care M0 RT) as described in **Section 6.2.1**.

Abiraterone as described in **Section 6.8**.

Prednisolone as described in **Section 6.8**.

Enzalutamide will be administered as a 160mg oral dose (four capsules), taken together at the same time every day, with or without food.

In patients with M1 disease, treatment with both abiraterone and enzalutamide will continue from randomisation until clinical disease progression, consistent with the approach taken for abiraterone (see **Section 6.8**) i.e., abiraterone and enzalutamide would be given for these patients until a composite of PSA progression (as defined in **Appendix J**), radiological progression (appearance of new lesions or progression of existing lesions) and clinical progression (defined as new cancer-related symptoms). It is accepted that these flexible criteria for stopping treatment with abiraterone and enzalutamide are open to the investigator's interpretation and discretion. Patients may continue treatment beyond the first failure-free survival (FFS) event (see Table 1 in Section 9.2); the first FFS event must be reported as per the other arms.

In patients with N0M0 disease or N+M0 disease undergoing radical radiotherapy, treatment would continue for 2 years or disease progression as defined for M1 patients, whichever is the sooner. ADT can be discontinued in this group at 2 years at the discretion of the local investigator (see **Section 6.2.1**).

For patients with N+M0 disease not planned for radical radiotherapy, treatment will continue as for patients with M1 disease until disease progression.

If a patient allocated to Arm J develops only biochemical failure, the responsible clinician might switch from abiraterone + prednisolone 5mg od to abiraterone and dexamethasone 0.5mg od.

Trial treatment must stop if other systemic treatments are initiated at any time for disease progression control (including the addition or swapping of anti-androgens, chemotherapy etc).

See **Section 6.2.4** and **Section 6.2.6** for further information for all groups.

6.11 ADMINISTRATION AND DOSE MODIFICATIONS

6.11.1 ZOLEDRONIC ACID

Note: recruitment stopped to the zoledronic acid and docetaxel-containing arms in Mar-2013 at the end of Activity Stage IV.

Zoledronic acid will be administered by IV infusion in accordance with the instructions in the summary of product characteristics at a target dose of 4mg (adjusted for renal function, see below) every 3 weeks for the first 6 cycles and every 4 weeks, thereafter.

Serum Creatinine Measurements: Serum creatinine should be measured at baseline and within 48 hours prior to every administration of zoledronic acid. It is permissible to have serum creatinine levels measured on Fridays prior to the administration of zoledronic acid on the following Monday.

Serum Electrolytes and FBC: Serum electrolytes including calcium, phosphate and magnesium should also be measured prior to each infusion. FBC should be measured at least 3 monthly. Zoledronic acid should be discontinued if there is any evidence of hypersensitivity to the drug. In patients with mild to moderate renal impairment, lower doses of zoledronic acid are recommended according to standard dose reduction schedules for administration of this drug. In rare cases, zoledronic acid treatment has been associated with the development of osteonecrosis of the jaw, particularly following dental extractions. If a patient develops osteonecrosis of the jaw then the zoledronic acid should be immediately and permanently discontinued. For full details of zoledronic acid administration and does reductions see **Appendix F**. Contraindications, special precautions, interactions and side effects are listed in **Appendix G**.

6.11.2 Docetaxel

Note: recruitment stopped to the zoledronic acid and docetaxel-containing arms in Mar-2013 at the end of Activity Stage IV.

The use of docetaxel should be confined to units specialised in the administration of cytotoxic chemotherapy and it should only be administered under the supervision of a physician qualified in the use of anticancer chemotherapy.

Docetaxel will be administered by IV infusion in accordance with the instructions in the summary of product characteristics at a dose of 75mg/m2 (up to a maximum dose of 160mg) on Day 1 of the study treatment period and then every 3 weeks thereafter for a maximum of 6 doses. Patients with a body surface area (BSA) greater than 2.13m^2 should be dosed as though they have a BSA of 2.13m^2. No ideal weight should be used for BSA calculations. Prednisolone or prednisone 5mg bid will be given until completion of chemotherapy. Additional dexamethasone should be given pre- and post-docetaxel infusion to suppress allergic reactions.

Please note that liver function test (LFTs) should be carried out within a week before the first cycle of docetaxel if an anti-androgen has been administered. This is due to an increased risk of neutropenia associated with docetaxel use following anti-androgen administration. Treatment should be delayed if LFTs are abnormal.

For full details of premedication schedule, recommended anti-emetic regimen and dose modifications for docetaxel (see Appendix F). Contraindications, special precautions, interactions and side effects are listed in Appendix G.

Docetaxel in combination with prednisone or prednisolone is indicated for the treatment of patients with hormone refractory metastatic prostate cancer.(20, 21)

6.11.3 Celecoxib

Note: recruitment completed to both celecoxib-containing arms in Apr-2011 at the end of Activity Stage II. No new patients should be receiving this agent now within the trial.

Celecoxib should be administered in accordance with the instructions in the summary of product characteristics at a dose of 400mg bid orally. Rarely this drug is poorly tolerated and in this instance should be discontinued; particular care should be taken with patients with a history of gastrointestinal disease and patients with significant risk factors for cardiovascular events (see Appendix G). Patients with confirmed severe cardiovascular history should not be in STAMPEDE (see exclusion criteria, Section 4.2). Contraindications, special precautions, interactions and side effects are listed in Appendix G. Dose reductions are not anticipated.

6.11.4 ABIRATERONE OR ENZALUTAMIDE + ABIRATERONE

Abiraterone absorption is increased by food. The tablets should be taken at least 2 hours after food, swallowed whole with some water. No food should be eaten for 1 hour afterwards. Prednisolone (prednisone in Switzerland) should be taken as a single dose with food in the morning.

Enzalutamide can be taken with or without food.

If clinical symptoms or signs suggestive of hepatotoxicity develop, serum transaminases, in particular serum alanine aminotransferase (ALT) should be measured immediately. If a rise in transaminases or bilirubin is confirmed, action should be taken as detailed in **Appendix G**.

6.11.4.A Management of Specific Toxicities from Abiraterone

The safety monitoring and toxicity management plan described below takes into account AEs based on the reported clinical safety data of abiraterone.

Hypokalemia:
At the initial observation of **Grade 1** hypokalemia (serum potassium <3.5mM or below lower limit of normal range, but ≥3.0 mM), oral potassium supplement will be initiated. The dose of potassium supplement must be carefully titrated to maintain serum potassium at ≥3.5mM but ≤5.0mM. Any subject with low potassium while on study or a history of hypokalemia from a pre-existing or concurrent medical condition will undergo weekly or more frequent laboratory electrolyte evaluation. The investigator should consider maintaining potassium level at ≥4.0mM in these subjects.

If any subject experiences **Grade 3** hypokalemia (serum potassium levels <3.0mM–2.5mM, NCI CTCAE v3.0) or life-threatening hypokalemia with potassium levels <2.5mM (NCI CTCAE v3.0 hypokalemia grade 4), abiraterone will be discontinued and the subject will be hospitalized for intravenous potassium replacement and cardiac monitoring. After the return of serum potassium to normal, prednisolone will be discontinued but the patient can be maintained on enzalutamide.

Hypertension:
If **Grade 1-2:** Management per investigator with anti-hypertensive treatment.

If **Grade 3-4:** Withhold abiraterone. Adjust or add anti-hypertensive medications to mitigate the toxicity. When hypertension resolves to **Grade ≤1**, resume abiraterone at full dose with prednisolone 5mg bid. Enzalutamide can be continued.

Fluid retention/oedema:
If **Grade 1-2:** Increase prednisolone dose to 5mg bid.

If **Grade 3-4:** Withhold abiraterone. Consider addition of mineralocorticoid receptor antagonist eplerenone until resolution of symptoms. When fluid retention/oedema resolves to ≤Grade 1, resume abiraterone at full dose with prednisone 5mg bid. Enzalutamide can be continued. Abiraterone may be re-started when symptoms return to baseline or are equivalent to grade 1; if oedema does not resolve, abiraterone should not be re-started.

Abnormal liver function tests:
If **Grade 1** increases in AST, ALT or bilirubin occur (eg, increase in AST or ALT from ULN to 2.5 x ULN; increase in total bilirubin from ULN to 1.5 x ULN): the frequency of liver function test monitoring should be increased, if the investigator judges that the laboratory abnormalities are potentially related to study medication. No dose reduction is required.

If **Grade 2** increases in AST, ALT or bilirubin occur (eg, increase in AST or ALT to >2.5-5 x ULN; increase in total bilirubin from >1.5-3 x ULN): the frequency of liver function test monitoring should be increased to ≥once a week, if the investigator judges that the laboratory abnormalities are potentially related to study medication. No dose reduction is required.

If **Grade 3** or higher increases in AST, ALT, or bilirubin occur (eg, increase in AST or ALT to >5 x ULN; increase in total bilirubin to >3 x ULN), withhold abiraterone and all other concomitant medications that are potentially hepatotoxic. Frequent laboratory evaluations (at least once weekly) should be conducted until the liver function tests return to baseline value or grade 1. If study treatment resumption is considered for subjects who have experienced grade 3 increases in AST, ALT, or bilirubin, resume abiraterone with the first dose level reduction (3 tablets, 750 mg of study treatment) when grade 3 toxicities resolve to grade 1 or baseline.

If **Grade 4** increases in AST, ALT, or bilirubin occur (eg, increase in AST or ALT to >20 x ULN; increase in total bilirubin to >10 x ULN), subjects must discontinue abiraterone and enzalutamide immediately. They should be followed-up until resolution of abnormal liver function tests and then prednisone can be discontinued and the investigator can consider restarting enzalutamide.

6.11.4.B Management of Specific Toxicities from Prednisolone
Prednisolone or prednisone will be started at 5mg once daily, to prevent secondary mineralocorticoid excess. Prednisolone/prednisone dose increase of up to 10mg/day is permitted to manage mineralocorticoid-related toxicities (e.g., hypokalaemia, hypertension) which are refractory to standard management. Patients experiencing serious Cushing symptoms (e.g., weight gain, muscle loss) can decrease or discontinue (temporarily or permanently) steroids at the investigator's discretion. It should be noted that weight gain and muscle loss are also associated with androgen deprivation therapy.

6.11.4.C Management of Specific Toxicities from Enzalutamide
If any subject suffers a seizure whilst on treatment, enzalutamide should be discontinued. Abiraterone and prednisolone can be continued if the subject is not suffering from any abiraterone-specific toxicities.

Fatigue:
If **Grade 1-2:** No change in treatment.

If **Grade 3-4:** Withhold abiraterone and enzalutamide. Restarting of all treatments with a dose reduction of enzalutamide to 80mg/day can be considered when fatigue resolves.

Patients who experience a grade 3 or higher toxicity that is attributed to enzalutamide and cannot be ameliorated by the use of adequate medical intervention may interrupt treatment with enzalutamide for 1 week or until the toxicity grade improves to grade 2 or lower severity. Subsequently, study drug dosing may be re-started at the original dose (160 mg/day) or at a reduced dose (120mg/day or 80mg/day) in consultation with the study team.

6.11.4.D Management of Specific Toxicities from Combination of Enzalutamide + Abiraterone

To date no specific toxicities from the combination of abiraterone and enzalutamide have been described (N = 57 patients with mCRPC exposed for a median of 5.5 months).(56)

6.11.5 Research (M1) Prostate Radiotherapy

A treatment planning CT scan will be acquired with the patient supine, with empty rectum and comfortably full bladder.

Megavoltage equipment is required with effective photon energies ≥6MV. Minimum source-to-axis distance is 100cm. Field arrangement is at the clinician's discretion: acceptable treatment techniques (field arrangement) include a 3-field (anterior, right lateral, and left lateral), 4-field (anterior, posterior, right lateral, and left lateral), or 6-field (right and left anterior oblique, right and left posterior oblique, and right and left lateral) or equivalent coplanar technique with multi-leaf collimation for all fields to adequately protect normal structures.

The Clinical Target Volume (CTV) will consist of the prostate gland alone as visualized on the treatment-planning CT scan. The base of the seminal vesicles may also be included if they are macroscopically involved. Inclusion of pelvic lymph nodes in the CTV is not permitted. The Planning Target Volume will have a 0.8 cm margin posteriorly and 1.0 cm margin in all other directions around the CTV to account for prostate gland motion and uncertainty in daily treatment setup.

Critical normal tissues should be delineated on the treatment-planning CT scan by the treating clinician:

- Rectum – inferior limit: level of ischial tuberosities; superior limit: sigmoid flexure
- Bladder – entirety

Two radiotherapy dose-fractionation schedules are permitted. In either case, radiotherapy is prescribed such that the PTV receives at least 95% of the prescribed dose:

- 36Gy in 6 fractions of 6Gy, administered weekly over 6 consecutive weeks
- 55Gy in 20 fractions of 2.75Gy, administered daily, five days per week, over 4 consecutive weeks

Dose-volume objectives for each dose-fractionation schedule are shown in **Tables 2** and **3** below. Values have been calculated using the formula BED = D[1+d/(alpha-beta ratio)] assuming an alpha-beta ratio of 3 for rectum and bladder. These are provided for guidance only.

Portal imaging to verify accuracy of treatment delivery may be done according to the participating centre's local guidelines. Image-guidance technology (e.g., gold seed intraprostatic fiducial markers, cone-beam CT scanning) will be permitted according to clinician preference but is not required. Further illustration on the research radiotherapy arm schedule is shown in **Figure 9**.

Table 2: Rectal dose volume objectives

55Gy/20F	36Gy/6F	Max Vol (%)
52.5 Gy	33.3 Gy	50%
43.5 Gy	27.8 Gy	60%
26.1 Gy	16.7 Gy	80%

Table 3: Bladder dose-volume objectives

55Gy/20F	36Gy/6F	Max Vol (%)
52.2	33.3	25%
43.5	27.8	50%

Figure 9: Diagram for deciding approach to research (M1) RT to the prostate

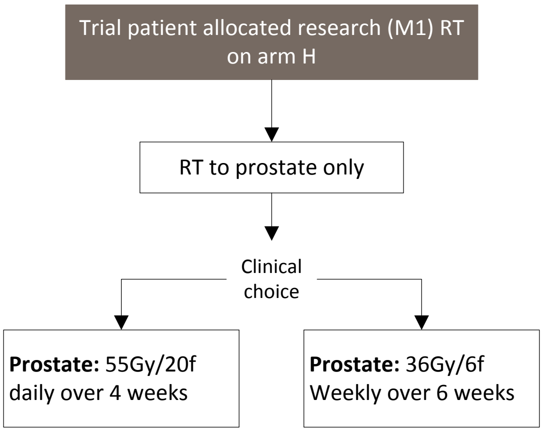

6.12 TRIAL PRODUCTS

Details of the procedures for obtaining the drugs within the trial, dispensing and disposal of unused drug are given in **Appendix E**.

Arrangements for free or discounted drugs are given in the Finance section (**Section 15**).

6.13 MEASURES OF COMPLIANCE/ADHERENCE

Date of treatment, dose, delays and reasons for delays or dose modifications of all study infusions (zoledronic acid and docetaxel) will be recorded. The estimated number of abiraterone tablets and enzalutamide capsules taken in a given time period will also be recorded as well as any dose reductions.

6.14 TREATMENT DATA COLLECTION

Data will be recorded on case report forms (CRFs); the top copy/original should be sent to the MRC CTU for data entry and a copy kept at the local centre. Up-to-date versions of all CRFs can be found on the trial website (http://www.stampedetrial.org/) and centres will be notified of any changes throughout the course of the trial. The type of data to be recorded is detailed in the Assessments and Procedures section (**Section 7**).

6.15 ADMINISTRATION OF STANDARD RADIOTHERAPY[8] TO NON-METASTATIC PATIENTS

6.15.1 TREATMENT DETAILS

Standard radiotherapy will be given to appropriate patients in each of the trial arms, following a period of neo-adjuvant ADT therapy, as is generally standard in UK practice. For patients receiving docetaxel, this period needs to be a minimum of 6 months after randomisation to ensure that

[8] **Note:** this text has been transferred into the protocol from the Appendices in version 8.0, and updated

chemotherapy is completed and toxicity resolved before RT begins. To ensure consistency of timing of administration of standard radiotherapy in all arms, this same 6 months period is recommended for all patients. For patients with negative nodes on axial imaging, clinicians may choose between irradiating prostate and seminal vesicles alone or including the pelvic nodes in addition. Additional staging tests such as pelvic node sampling may be considered in making this decision. Conformal or intensity modulated radiotherapy should be used in all patients. Where patients have good clinical evidence that nodes are free of tumour or patients for whom nodal radiotherapy is contra-indicated (e.g. significant bowel disease), treatment may be given to the prostate gland and seminal vesicles only. The recommended dose is 74Gy in 37 fractions to the prostate and seminal vesicles or the equivalent using hypofractionated schedules. These recommendations are summarised in **Figure 10**. Alternative dosing schedules are permitted but must be agreed with the STAMPEDE Trial Management Group.

6.15.1.A Standard-of-care RT Timing in M0 patients

Radiotherapy should be given around 6 to 9 months after randomisation in all trial arms and, if receiving docetaxel, the patient must have recovered from any docetaxel toxicity before RT can begin.

6.15.1.B Type Of standard-of-care RT in M0 patients

Conformal or intensity modulated radiotherapy.

6.15.1.C Standard Clinical Target Volume in M0 patients

- **CTV1:** Prostate plus seminal vesicles
- **CTV2:** (Node positive patients) Regional lymph nodes to include internal iliac and the inferior part of the common iliac nodes as used in EORTC trial 22961 (57)
- **PTV1:** CTV1 plus 10-15 mm according to local practice
- **PTV2:** CTV2 plus 10-20mm according to local practice

6.15.1.D Standard-of-care RT Dose in M0 patients

Prostate dose of 74Gy in 2Gy fractions or equivalent, with optional dose to the pelvic nodes of 46-50Gy in 2Gy fractions or equivalent using IMRT to deliver the treatment over 37 fractions, suggested dose is 55Gy in 37 fractions with IMRT. Higher doses may be considered if the department is experienced in using IMRT for nodal radiotherapy, particularly as data emerges from the PIVOTAL trial of nodal IMRT in high-risk node negative patients where a nodal dose of 60Gy in 37 fractions is being evaluated. Alternative schedules should be agreed with the STAMPEDE Trial Management Group.

Figure 10: Diagram for deciding recommended approach to standard-of-care (M0) RT in non-metastatic patients

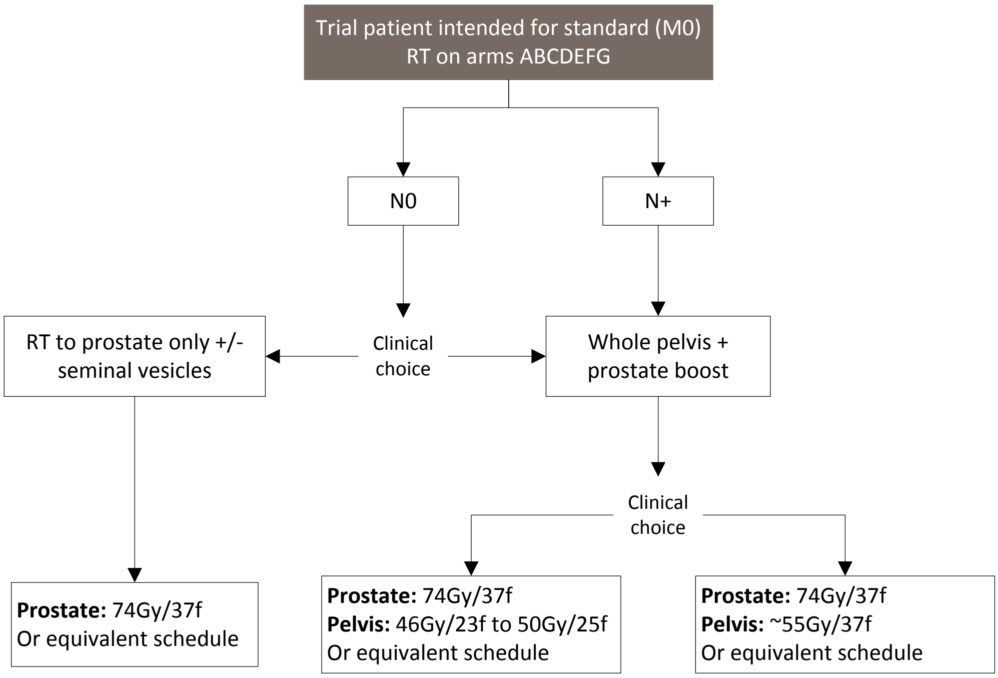

6.16 NON-TRIAL TREATMENT

6.16.1 MEDICATIONS PERMITTED

Any additional treatment that the responsible physician feels is appropriate is permitted.

6.16.2 DATA ON CONCOMITANT MEDICATION

All concomitant medication will be recorded on the baseline form prior to randomisation and on any subsequent Serious Adverse Event forms. This should include aspirin that may be taken on a regular basis for cardiovascular disease, the use of any Non-Steroidal Anti-inflammatory Drugs (NSAID) as well as any vitamin or mineral supplements the patient is taking.

7 ASSESSMENTS AND PROCEDURES

7.1 SCHEDULE FOR ASSESSMENTS

A detailed follow-up schedule is given in **Table 4, 5 and 6**.

7.1.1 PSA Measurements

All patients should have PSA measured pre-androgen deprivation therapy and at weeks 6, 12, 18 and 24 and every 12 weeks, thereafter, up to 2 years post randomisation. Following this, PSA should be measured every 6 months until 5 years and annually, thereafter. For patients who do not have a scheduled hospital visit, it would be acceptable for arrangements to be made for blood samples to be drawn in a GP's surgery.

7.1.2 Assessment Of Treatment Failure (Definition Of Progression)

It is not proposed to routinely assess patients for response. However, in order that objective progression can be assessed, it is necessary to have imaging taken at time of best response as judged by the treating clinician. All patients should have baseline radiological examinations as detailed in **Section 4.4.1**. In addition it is recommended that all patients should have scans or X-rays repeated at 24 weeks (and whenever clinically appropriate) if they were abnormal at baseline, particularly if they have a low PSA value on entry in to the trial making biochemical assessment of treatment failure difficult. The following events would constitute a disease progression and should be reported on a progression form:

- Biochemical failure – must be reported alongside castrate levels of testosterone if the patient has received intermittent ADT (see **Appendix J**).
- Local progression
- Lymph node progression
- Progression in distant metastases
- Development of new metastases

Please note that skeletal-related events (SREs) may be indicative of disease progression but can have other causes such as osteoporotic fracture. All SREs should be investigated further to establish whether or not the patient has progressed, in which case a progression form should be completed.

7.1.3 Additional Safety Assessment

Due to the risk of liver toxicity and secondary hyperaldosteronism with abiraterone, patients will require 2 weekly U+Es, LFTs and blood pressure measurement for the first 12 weeks. It is not proposed to collect the detail of these measurements unless results are abnormal; in this instance, they should be reported as AEs (on the next Follow-up CRFs) and as SAEs (see **Section 11**) if appropriate.

Medical review and PSA measurements follow the pattern in the control arm: visits at weeks 6, 12, 18 and 24 and every 12 weeks, thereafter, up to 2 years post randomisation. Following this, PSA should be measured every 6 months until 5 years and annually, thereafter. For patients who do not have a scheduled hospital visit, it would be acceptable for arrangements to be made for blood samples to be drawn either in a GP's surgery or in the patient's home.

7.1.4 Data Collection and Non-Administration Of Standard Radiotherapy

There are CRFs to be completed for patients receiving primary radiotherapy whether this is standard radiotherapy for M0 patients on any arm or prostate radiotherapy for Arm H patients. All radiotherapy and acute side effects details will be recorded on the Radiotherapy Form; any late side effects will be recorded on the Follow up form.

If it is decided not to give the planned radiotherapy (for example, due to early metastatic progression or patient refusal), this should be stated on the Standard Radiotherapy form together with the reason for non-administration of the treatment.

7.1.5 Data Collection Palliative Radiotherapy

For patients who receive palliative radiotherapy as part of first line treatment, a Palliative Radiotherapy CRF should be completed. Details of salvage RT for relapse and palliative treatment will be requested and completed only on the Progression Form.

7.1.6 Data Collection Research (M1) Radiotherapy

There are arm specific CRFs for patients randomised to arm H. Adverse events such as hip fractures, TURPs, skeletal-related events will be collected retrospectively via the Hospital Episode Statistics (HES) database.

7.1.7 Follow-Up Schedules

An individualised form with a follow-up schedule will be provided for each randomised patient. For patients who are receiving LHRH analogues, it is assumed that any additional treatment will commence within two weeks of randomisation. For patients who are due to have an orchidectomy it is recognised that surgery will have to be scheduled and the scheduling of any additional treatments may be affected by post-operative recovery. It is recommended that all patients who had abnormal radiological investigations at baseline or present with a low PSA on entry into the STAMPEDE trial should have them repeated 24 weeks after randomisation.

7.2 FOLLOW-UP

Every effort should be made to follow-up all patients who have been randomised. Patients should, if possible, remain under the care of an oncologist or urologist for the duration of the trial. If care of a patient is returned to the GP, it is the responsibility of the consultant who obtained the patient’s consent to participate in the trial to ensure that the data collection forms are completed. If the patient moves from the local area, arrangements should be made for trial follow-up to be undertaken by their new local centre. Details of other participating centres can be obtained from the MRC CTU. The consent of patients should be obtained for their names to be flagged for survival information through national registries, for example NHS Information Centre/Office of National Statistics (ONS) in England/Wales and General Register Office in Scotland, Hospital Episode Statistics (HES). If the clinician moves, appropriate arrangements should be made to arrange for trial follow-up to continue at the centre.

Table 4: Summary of timing of case report forms

CASE REPORT FORMS	TIMING OF ASSESSMENT AND CRF
**Baseline**	
Bone Density Risk Factor	At randomisation
Randomisation	At randomisation
Baseline	At randomisation
Cardiovascular Assessment	At randomisation
Pathology	At randomisation. When pathology sample has been taken and sent to UCL laboratory.
**Treatment**	
Pre-18 Week Bisphosphonate	Treatment administered every 3 weeks. Form holds data for 2 cycles. Form to be sent after 2nd cycle given.
Post-18 Week Bisphosphonate Treatment	Treatment administered every 4 weeks. Form holds data for 3 cycles. Form to be sent after 3rd cycle given.
Docetaxel Treatment	Treatment administered every 3 weeks Form holds 2 cycles. Form to be sent after 2nd cycle given.
Hormone Therapy	Form to be sent with corresponding follow-up form if there is a change in hormone therapy to report
Abiraterone and Enzalutamide Treatment	Treatments administered daily; form to be sent at week 6 and with corresponding follow-up form if there is a change in treatment to report
RT detail	• When standard-of-care radiotherapy is completed or if planned RT is no longer to be given<br>• Arm H when research RT completed<br>• Arm A (M1) at 3 months<br>All patients: once primary RT is complete (Arm H or standard-of-care)<br>For patients who did not receive primary RT (since Protocol 9.0 regardless of being planned): complete 10 months after randomisation to confirm RT was not given
RT Acute Toxicity	For all patients who receive primary RT.
**Assessments**	
Follow-Up	Every 6 weeks for 6 months, then every 12 weeks until 2 years, then every 6 months until 5 years and annually thereafter. (See **Table 7** for more information.)
Palliative Radiotherapy	If applicable, when a palliative radiotherapy course is completed.
End of Treatment	When each treatment is completed (either at end of scheduled treatment or at early cessation of treatment).

CASE REPORT FORMS	TIMING OF ASSESSMENT AND CRF
Progression & Additional Treatment	At the first occurrence of each type of progression, including skeletal-related events and whenever a patient that has progressed receives additional treatment.
Additional Treatment Update	Whenever a patient that has previously progressed received additional treatment but has not experienced a new type of progression
Serious Adverse Event	Following any Serious Adverse Event
Death	At Death
**Administration**	
Patient Transfer	When a patient is transferred to a different hospital for the administration of trial treatment and follow up
Co-enrolment	When a patient is co-enrolled in any other clinical trial. Please see **Section 5.1** for more information

Table 5: Data required on follow-up forms

TIMING OF FOLLOW-UP	PSA	EVIDENCE OF PROGRESSION	ANDROGEN DEPRIVATION THERAPY	TREATMENT	UNSCHEDULED VISITS	TOXICITIES
Follow-up Form	✓	✓	✓	✓	✓	✓
Follow-up Form (Post-Progression)	✓	✓	✓	x	✓	X*

* Toxicity information will be collected for Arm G and J patients if progression has occurred but trial treatment continues

Table 6: Schedule for completion of treatment and outcome forms by arm.

Timing From Randomisation			Treatment Forms		Outcome forms	
Years	Months	Weeks	Abi and/or enza	RT	Follow-up Ψ	QL + HE¥
**6-Weekly**						
-	-	6	G, J	-	All arms	All arms
-	-	12	G, J	M1: A,H	All arms	All arms
-	-	18	G, J	-	All arms	All arms
-	6	24	G, J	-	All arms	All arms
**12-Weekly**						
-	9	36	G, J	-	All arms	All arms
1	12	48	G, J	M0: A,B,C,E,G, J	All arms	All arms
-	15	60	G, J	-	All arms	All arms
-	18	72	G, J	-	All arms	All arms
-	21	84	G, J	-	All arms	All arms
-	-	96	G, J	-	All arms	All arms
**6-Monthly**						
2	24	104	G, J	-	All arms	All arms
	30	130	G, J	-	All arms	All arms
3	36	156	G, J	-	All arms	All arms
	42	182	G, J	-	All arms	All arms
4	48	208	G, J	-	All arms	All arms
	54	234	G, J	-	All arms	All arms
5	60	260	G, J	-	All arms	All arms
**Annual**						
6	72	-	G, J	-	All arms	All arms
7	84	-	G, J	-	All arms	All arms
Etc	-	-	G, J	-	All arms	All arms

Key:
A = ADT alone
B = ADT + zoledronic acid
C = ADT + docetaxel
D = ADT + celecoxib
E = ADT + zoledronic acid + docetaxel
F = ADT + zoledronic acid + celecoxib
G = ADT + abiraterone
H = ADT + M1 research RT to the prostate
J = ADT + enzalutamide + abiraterone

Notes:

Ψ See Table 6 for information required at follow-up
† Form records data for two cycles
‡ Form records data for three cycles
¥ 1st 700 patients and those recruited from protocol version 8.0 onwards only

Note: Radiotherapy Detail & Acute Toxicity, Late RT Toxicity, HT and Abiraterone and Enzalutamide Treatment, Palliative Radiotherapy Progression, SAE, End of Treatment, Co-enrolment and Death forms to be completed as required.

Note: Docetaxel forms are no longer shown on the table as all patients will have completed treatment with docetaxel

Note: Recruitment completed to Arms D and F in April 2011; Arms B, C and E in March 2013; Arm G in January 2014

Note: Quality of Life Study is only for first 700 patients entered into the trial and those who were recruited after the implementation of version 8.0 of the protocol. MRC CTU will inform centres of which of their patients this applies to.

7.3 TRIAL CLOSURE

For the purpose of complying with UK Clinical Regulations introduced on May 2004, the trial will be considered ‘closed’ when the follow-up point for the primary analysis of the final comparison has been reached. However, further observational follow-up of all patients enrolled in the trial will continue until all randomised patients have died. This will initially be via the hospital, but in the longer term may employ national registers.

8 STOPPING OF TREATMENT OR FOLLOW UP

Patients should be given every encouragement to adhere to protocol treatment and follow-up schedule, in order to reduce bias. However, a patient has the right to withdraw consent for participation in any aspect of this trial at any time.

8.1 STOPPING RESEARCH INTERVENTIONS

A patient may stop trial treatment for the following reasons:

- Progression whilst on therapy (trial treatment must be discontinued in this instance). For patients randomised to Arm G, please refer to **Section 6.8** for criteria to stop treatment
- Unacceptable toxicity
- Intercurrent illness which prevents further treatment
- Withdrawal of consent for treatment
- Any alteration in the patient's condition which justifies the discontinuation of treatment in the clinician's opinion
- Intention to commence a new anti-cancer treatment due to evidence of relapse.

The reason should be recorded on the treatment and/or follow-up forms as well as the End of Treatment form. In the case of abiraterone, the disease event for stopping abiraterone may be after the first reportable failure-free survival event (see **Section 6.8**). Unless a patient states otherwise, it should be assumed that consent is given to continue to record trial data.

8.2 PATIENT TRANSFERS

For patients moving from the area, every effort should be made for the patient to be followed-up at another participating trial centre and for this trial centre to take over responsibility for the patient. To document the transfer process the main contact person at both the current and receiving hospitals should complete and sign the Patient Transfer Confirmation form. A fully completed form must be returned to the CTU prior to the patient transfer and ideally any data queries for the patient should be completed prior to transfer.

On receipt of the completed transfer form a member of the STAMPEDE team will confirm the database has been updated and request confirmation of the name of the patient's new Clinician. Photocopies of the following documents may then be sent to the new hospital to complete the transfer and copies must be also retained at the original site for monitoring purposes:

- Consent form
- Completed CRFs
- Any documentation relating to the patient's participation in STAMPEDE (patient names must be removed from any documentation).

8.3 EARLY CESSATION OF TRIAL PARTICIPATION

If a patient explicitly withdraws consent to have any further data recorded their decision must be respected and the MRC CTU must be informed in writing. All communication surrounding the early cessation of trial participation should be noted in the patient's records.

In the majority of cases, patients give permission for their data and information on their health to continue to be collected via clinical notes and national registries. Any information on the follow-up status, however minimal, would be helpful.

Early stopping of follow-up should not be undertaken lightly and the site must consider the implications for the trial and the patient in reaching such a decision. Without long-term data, the efficacy of trial treatments would be less reliable and could lead to inconclusive results. The early stopping of trial treatment should not lead to the early cessation of trial participation and in such cases follow-up assessments should be continued as per trial protocol.

Patients can change their minds about withdrawal at any time and re-consent to participate in the trial. Follow-up data should be collected only from the point of when consent was re-instated.

9 STATISTICAL CONSIDERATIONS

9.1 METHOD OF RANDOMISATION

Patients will be randomised centrally using a computerised algorithm developed and maintained by the MRC CTU. Randomisation will be performed using the method of minimisation over a number of clinically important stratification factors with an additional random element. To decrease determinability, the factors are not listed here but can be found in the Statistical Design Document.

Table 8 shows the allocation weighting for each arm by protocol version. The relative weighting within each pairwise comparison remains constant throughout.

9.1.1 To Version 7

From the outset, the trial had 1 control arm (A) and 5 research arms (B, C, D, E and F).

As the control arm is the comparator arm for all the research arms, twice as many patients were recruited to the control arm as to each of the original research arms as this is an efficient design. Therefore, the initial randomisation ratio will be A2:B1:C1:D1:E1:F1. From version 7.0, accrual to the celecoxib-containing arms was halted and the allocation ratio was A2:B1:C1:D0:E1:F0.

9.1.2 Version 8

From version 8.0, an additional research arm (G) was introduced. The allocation weighting for the additional Arm G is 2, meaning that as many patients are contemporaneously randomised to Arm G as the control Arm A: the randomisation ratio is 2:2 (equivalent to 1:1, control:abiraterone). This gave an overall allocation ratio of A2:B1:C1:D0:E1:F0:G2. When recruitment has been completed to the ongoing original research Arms B, C and E (which will be around 2 years before completion of accrual to arm G), the allocation ratio will be A2:B0:C0:E0:D0:F0:G2 (or A2:G2). This is more efficient for this comparison than the 2:1 allocation ratio employed for the original research arms because of the minimal co-recruitment period.

Version 9.0 introduced a RT comparison for men with newly-diagnosed metastatic disease which is irrelevant to a subset of men joining STAMPEDE. This could only be achieved by splitting the randomisation system so that newly-diagnosed patients with M1 disease, no planned RT and no contraindication to RT are randomised A2:B1:C1:D0:E1:F0:G2:H2 and other men are randomised A2:B1:C1:D0:E1:F0:G2:H0. Note that the allocation ratio for each pairwise comparison in unaffected, only the rate at which comparisons accrue.

9.1.3 Version 10 and 11

Version 10.0 followed the successful completion of recruitment to Arms B, C and E. Therefore, the allocation ratio will be A2:B0:C0:E0:D0:F0:G2 (or A2:G2) for M0 patients and A2:B0:C0:E0:D0:F0:G2:H2 for M1 radiotherapy arm patients (A2:G2:H2).The equal allocation ratio is suitable with fewer research arms open.

9.1.4 Version 12

Version 12.0 introduces a further allocation, Arm J: HT + abiraterone + enzalutamide. This allocation will be available to all patients. Accounting for Arm H still recruiting, this can only be achieved by keeping the randomisation system split so that newly-diagnosed patients with M1 disease and no contraindication to RT will be randomised A2:B0:C0:D0:E0:F0:G0:H2:J2 and other men will be

randomised A2:B0:C0:D0:E0:F0:G0:H0:J2. This can be simplified to equal allocation in these groups to A:H:J and A:J respectively.

Table 7: Allocation to each arm by protocol version

PROTOCOL	NEWLY-DIAGNOSED M1 PATIENTS									OTHER PATIENTS								
VERSION	A	B	C	D	E	F	G	H	J	A	B	C	D	E	F	G	H	J
V1	2	1	1	1	1	1	-	-	-	2	1	1	1	1	1	-	-	-
V2	2	1	1	1	1	1	-	-	-	2	1	1	1	1	1	-	-	-
V3	2	1	1	1	1	1	-	-	-	2	1	1	1	1	1	-	-	-
V4	2	1	1	1	1	1	-	-	-	2	1	1	1	1	1	-	-	-
V5	2	1	1	1	1	1	-	-	-	2	1	1	1	1	1	-	-	-
V6	2	1	1	1	1	1	-	-	-	2	1	1	1	1	1	-	-	-
V7	2	1	1	*0*	1	*0*	-	-	-	2	1	1	*0*	1	*0*	-	-	-
V8	2	1	1	*0*	1	*0*	2	-	-	2	1	1	*0*	1	*0*	2	-	-
V9	2	1	1	*0*	1	*0*	2	2	-	2	1	1	*0*	1	*0*	2	0	-
V10	2	*0*	*0*	*0*	*0*	*0*	2	2	-	2	*0*	*0*	*0*	*0*	*0*	2	0	-
V11	2	*0*	*0*	*0*	*0*	*0*	2	2	-	2	*0*	*0*	*0*	*0*	*0*	2	0	-
V12	2	*0*	*0*	*0*	*0*	*0*	*0*	2	2	2	*0*	*0*	*0*	*0*	*0*	*0*	0	2

9.2 OUTCOME MEASURES

The overall, definitive primary outcome measure for the trial for each comparison is overall survival (all-cause mortality). The design of the trial is such that it is important to have additional intermediate outcome measures to assess activity in each research arm as the trial progresses. These are listed in **Table 9**. The intermediate primary outcome measure is failure-free survival. The reasons for different emphases in each recruitment stage are explained in **Section 9.3**.

Table 8: Trial Outcome Measures by Comparison Stage

TRIALS STAGE	PRIMARY OUTCOME MEASURES	SECONDARY OUTCOME MEASURES
Pilot phase	Safety*	Feasibility
Activity Stage (AS) I-III	Failure-free survival (FFS)†	Overall survival (OS)<br><br>Toxicity<br>Skeletal-related events
Efficacy Stage (ES) IV	Overall survival	Quality of life<br><br>Cost effectiveness<br>Failure-free survival†<br>Toxicity<br>Skeletal-related events

*Based on toxicity
†Including biochemical failure (see **Appendix J**)

9.3 SAMPLE SIZE: PRINCIPLES AND ASSUMPTIONS

The overall design for this study is a multi-arm multi-stage, multi-centre randomised controlled trial. There are a number of stages for each research arm: a Pilot Phase, Activity Stages and a final Efficacy Stage. Full details of the methodology underlying the trial design are given by Royston et al. (58, 59) The sample size calculations were performed using the `stage2` (version 1.2.0, March 2002) and `stagen` (version 1.1.1, 18 May 2004) programs, both implemented in Stata (Stata Corp, TX) and updated using the later `nstage` program (version 1.0.3, 13-jun-2007; version 2.1.0, 28-jun-2009). (60)

The trial was designed under the assumptions in **Table 9**, and additionally, we assume a slightly higher proportion of non-metastatic than metastatic patients joining the trial such that the median FFS is two years and median OS four years for the whole cohort.

Table 9: Hazard ratio assumptions under null and alternative hypotheses

SIZE OF HR	PILOT	AS I-III	ES IV
Under null hypothesis (H0)	n/a	HR(FFS) = 1.00	HR(OS) = 1.00
Under alternative hypothesis (H1)	n/a	HR(FFS) = 0.75	HR(OS) = 0.75

The HR of 0.75 for any research arm relative to control would translate into an absolute improvement in FFS of 10%, from approximately 50% to 60% at two years and OS of 10%, from approximately 50% to 60% at four years. A beneficial difference of this size would be clinically worthwhile and, indeed, experience tells us it may be unrealistic to expect a larger difference. Therefore, we have adequately powered the trial to detect a HR of 0.75 for overall survival. This design gives 95% power at Activity Stages I-III and 90% power at Efficacy Stage IV for each

comparison. Further details of the sample size calculations are summarised in Sections 9.4 and 9.5 and detailed in a separate Statistical Design Documents which are available on request.

Note that, from protocol version 8.0, standard-of-care M0 RT was introduced to the majority of patients with N0 M0 disease. This is likely to improve the outcomes for this group. Further agents are starting to be licensed for patients with castration-refractory disease which may also improve survival rates. Improved FFS rates would delay the intermediate analyses; improved survival rates would delay the definitive analyses. The Statistical Design Document includes models where median survival is estimated at 5, 6 and 7 years rather than just 3and 4 years. The trial is powered to detect a difference in relative improvement and the analyses will be performed when a pre-planned number of events has been reported in the control arm, rather than after a certain number of patients have been recruited or a certain amount of time elapsed. Sections 9.4 and 9.5 provide more detail, including some variations on these assumptions.

Throughout recruitment to protocol version 12.0, at least, the proportion of metastatic men joining the trial has been fairly constant, at around 60%. From protocol version 9.0, we introduced an allocation, Arm H, only for men with (newly diagnosed) M1 disease. This means that further comparisons for the whole patient group will have proportionately fewer metastatic patients and, therefore, fewer events at any given moment in time. This will affect contemporaneously-recruiting comparisons, such as the “enzalutamide + abiraterone comparison” introduced in protocol version 12.0. Median survival may therefore be higher in that comparison, at around 7 years.

9.4 SAMPLE SIZE ISSUES AND TRIAL STAGES: ORIGINAL RESEARCH ARMS (B-F)

9.4.1 PILOT PHASE: ORIGINAL RESEARCH ARMS (B-F)

It was anticipated that 210 patients would be recruited to the Pilot Phase from a limited number of centres over a one year period. Approximately 60 patients would be randomised to the control arm and 30 patients to each of the five research arms, each of which were assessed for safety and feasibility. If recruitment proved unfeasible or any of the research arms proved unsafe or not feasible to administer (e.g., poorly tolerated or unexpected toxicity) recruitment to these arms would have been discontinued. There were already considerable safety data on the use of docetaxel and zoledronic acid in patients with malignancies including prostate cancer, and on the use of Cox-2 inhibitors (including celecoxib), although mainly from patients with musculoskeletal disorders. There were fewer data on the combination arms, but it was thought very unlikely that any of the research arms would be discontinued during the Pilot Phase. When 210 patients had been on the trial for a minimum of 18 weeks, the Independent Data Monitoring Committee (IDMC) reviewed the data from the Pilot Phase and continued to the trial during this period as equipoise remained. Recruitment continued beyond this point. Safety data are assessed throughout the trial.

9.4.2 ACTIVITY STAGES I-III: ORIGINAL RESEARCH ARMS (B-F)

In the sample size calculations, we assumed that all research arms successfully pass through the Pilot Phase to Activity Stage I and that patients would be recruited at a rate of approximately 500 per year. This was faster than in the Pilot Phase because the trial would recruit from additional centres, both in the UK and internationally. The analysis of Activity Stages I, II and III were planned for when around 113, 216 and 334 failure-free survival events had been observed in the control arm, respectively.

The Activity Stage analyses comprise pairwise comparisons of FFS between the control arm and each of the 5 research arms (i=B, C, D, E, F). Let HR_i(true) represent the hazard ratio (HR) of the ith

research arm to the control arm, and HRi(observed) the observed value. Discontinuation of accrual of further patients was considered for the ith research regimen at each of Activity Stages I-III according to the guidelines in **Table 10**.

Table 10: Guidelines for stopping accrual to the ith original research arm

Activity Stage	Number of control arm events	Consider discontinuation if HR_i(observed) is…
I	~113	>1.00
II	~216	>0.92
III	~334	>0.89

9.4.3 Efficacy Stage IV: Original Research Arms (B-F)

The analysis of Efficacy Stage IV for the original research arms will be performed when around 403 deaths have been observed in the control arm. This would give 90% power to detect the targeted hazard ratio of 0.75 at one-sided significance level of 0.025. The actual length of this stage, balancing continued accrual with just follow-up, depended on the number of arms passing through to further recruitment from Activity Stages I-III and the observed accrual and event rates.

9.4.4 Sample Size For Original Research Arms (B-F)

Assuming an accrual rate of 500 patients/year, between 2800 and 3600 patients were planned to be entered into the original research comparisons of the trial over a period of 5½ and 7 years. The exact number of patients to be entered depends on the observed accrual rate and the observed event rate, which is, in itself, dependent on the mix of patients joining the trial from the broad spectrum of eligibility. The primary analysis on overall survival requires around 403 deaths to be observed on the control arm. Accrual continued until the main analysis can be foreseen so that the overall duration of the comparisons would be as short as possible (longer accrual facilitates this) and so that few, if any, patients remain on treatment when the main results are released. The statistical team have monitored and projected the analysis timelines using the `artpep` command in Stata. Results should be due in 2015. Further information is available in the Statistical Master File.

9.5 SAMPLE SIZE ISSUES AND TRIAL STAGES: ADDITIONAL RESEARCH ARM G

9.5.1 Pilot Phase: Additional Research Arm G

A similar approach is being followed for the additional research Arm G, as detailed for the original research arms in **Section 9.4.1**. The IDMC reviewed safety data, in the context of data from the control arm, when the first 30 patients allocated to Arm G had been on trial for at least 18 weeks.

Furthermore, an additional review of safety was performed when 30 patients with newly-diagnosed non-metastatic disease allocated to Arm G had been on trial for at least 18 weeks.

Both of these milestones were successfully completed.

9.5.2 Activity Stages I-III: Additional Research Arm G

The same principles are applied to the new comparison as to the previous comparisons. The notable difference will be in the accrual rate to this comparison which is anticipated to be higher. There are two reasons for this. First, STAMPEDE started to recruit slowly in only a limited number of pilot sites.

As more sites have been activated, including internationally, accrual has increased. At the time of version 8.0 of the protocol, monthly accrual to the study was averaging around 60 patients/month (over 700 patients/year). Second, there is an equal allocation ratio for the abiraterone arm compared to the control arm. It is this different allocation ratio which means that the number of control arm events required to trigger the intermediate analyses is different for the assessment of abiraterone to the assessment of the original research arms. This is shown in **Table 11**.

Table 11: Guidelines for stopping accrual to the additional research Arm G

ACTIVITY STAGE	NUMBER OF CONTROL ARM EVENTS	CONSIDER DISCONTINUATION IF HR_G(OBSERVED) IS...
I	~75	>1.00
II	~142	>0.92
III	~221	>0.89

9.5.3 EFFICACY STAGE IV: ADDITIONAL RESEARCH ARM G

The analysis of Efficacy Stage IV for the additional comparison will be performed when around 267 deaths have been observed in the control arm. This would give 90% power to detect the targeted hazard ratio of 0.75 at a one-sided significance level of 0.025.

9.5.4 SAMPLE SIZE FOR ADDITIONAL RESEARCH ARM G

Up to around 1,800 patients will join the abiraterone comparison, with half allocated to the research arm. Consideration will be given to ceasing further randomisations to Arm G if it is not showing sufficient evidence of activity at the interim analyses, just as was done for research Arms B to F.

The original plan intended for accrual to be halted either when 1,500 patients had been recruited or after 3 years, whichever was the sooner, providing the accrual rate remained above 50 patients/months.

The total number of patients joining this comparison depends not just on the same issues as the original comparisons (notably, observed accrual and event rates), but also the length of time that the original research arms co-recruit alongside the additional research arm; it was originally assumed that this would be for approximately 1 year, but it was closer to 1.5 years. The sample size calculations and projected durations are fairly robust to changes in the length of co-recruitment with the original research arms and future co-recruitment with any further research arms which the Trial Management Group may introduce. Many scenarios are detailed in the Statistical Design Document.

In Sep-2013, the target sample size for the abiraterone comparison was increased from around 1,500 patients to around 1,800 patients, with the efficacy analysis still to be triggered by around 267 control arm deaths. This increase in sample size was primarily because of an increase in the proportion of non-metastatic patients joining the comparison; this related to the activation of Arm H which only recruits patients with newly-diagnosed metastatic disease and thereby reduces the numbers of metastatic patients randomised to the abiraterone comparison. Non-metastatic patients have a lower event rate than the metastatic patients and maintaining the same overall sample size would lead to a delay in time to the primary analysis. The increase in sample size was achievable because recruitment rates to the trial had been substantially higher than 50 patients/month for the preceding 6 months.

9.6 SAMPLE SIZE ISSUES AND TRIAL STAGES: ADDITIONAL RESEARCH ARM H

9.6.1 Pilot Phase: Additional Research Arm H

A similar approach will be followed for the additional research Arm H as detailed for the original research arms in **Section 9.4.1**. The IDMC will review safety data, in the context of data from the control arm, when the first 30 patients allocated to arm H have been on trial for around six months.

9.6.2 Activity Stages I-III: Additional Research Arm H

The same principles will be applied to the new comparison as to the previous comparisons and an equal allocation ratio of control arm patients to patients allocated to Arm H will be employed, as for Arm G. The number of control arm events required to trigger the intermediate analyses will be the same as for the abiraterone comparison (see **Table 13**).

9.6.3 Efficacy Stage IV: Additional Research Arm H

The analysis of Efficacy Stage IV for the additional comparison will be performed when around 267 deaths have been observed in the control arm. This would give 90% power to detect the targeted hazard ratio of 0.75 at one-sided significance level of 0.025.

9.6.4 Sample Size For Additional Research Arm H

Consideration will be given to ceasing further randomisations to Arm H if it is not showing sufficient evidence of activity on the intermediate primary outcome measure (FFS), just as for the other research arms. This research comparison is relevant to around 60% of patients joining STAMPEDE. At the point of the scientific approval, accrual was averaging around 80 patients per month to the trial. If accrual to the trial was slower at 70 patients per month, then accrual to this comparison could be between 18 and 42 patients per month, depending on which other trial arms are open to recruitment at the time.

We are targeting a 25% relative improvement in overall survival following local radiotherapy to the prostate in this patient group. This is the same size of effect targeted with the other research arms in STAMPEDE. This relative improvement can be further justified in the light of MRC PR07 which demonstrated an improvement of this magnitude for adding radiotherapy to ADT in locally advanced disease, with a hazard ratio for overall survival of 0.77 (95% CI 0.61 to 0.98). In that trial, fewer than half of the deaths were from prostate cancer, whereas in newly-diagnosed metastatic patients nearly all men will die of their disease. Therefore, it is relevant to note the relative benefit of radiotherapy in PR07 in terms of prostate cancer-specific survival, where the hazard ratio was 0.54 (95% CI 0.27 to 0.78). Long-term survival-based data, with a median follow-up of ~10 years, were presented orally at the American Society of Clinical Oncology 2012 which confirmed these findings.(7)

We anticipated that around 1250 patients were required over 4 years to observe 267 control arm deaths after 5.25 years. In addition to the factors listed in **Section 2.1.2**, this assumed that (i) recruitment was constantly 70 pts/m to the trial overall; (ii) the original research arms stopped accrual within 6 months after activation of the RT arm; (iii) the abiraterone arm stops accrual around 24 months after activation of the RT arm; and (iv) a further new research arm with an equal allocation ratio was introduced 18 months after activation of the RT arm.

In protocol version 13.0, we reflect on these four points: (i) recruitment to the trial has been faster; (ii) the original research arms completed accrual 2 months after activation of the RT arm; (iii) the abiraterone arm stopped accrual 12 months after activation of the RT arm; and (iv) Arm J was activated 18 months after activation of the RT arm, Arm H.

Of patients joining STAMPEDE during this time, 60% have been eligible for the "M1|RT comparison". Prior to randomisation, a RT schedule must be nominated: Weekly or Daily (see Section 6.9). We have observed that around half of patients in the comparison are nominated for RT with the Daily schedule and half for the Weekly schedule, primarily chosen by trial site with patient groups nominated for each schedule observed to be comparable at baseline. There will likely be interest to know the effect of each RT schedule when the main results are reported. This will be explored by "within schedule" comparisons of patients randomised to research vs control (arms H vs A) within each nominated RT schedule.

Therefore, in protocol version 13.0, the target sample size is increased from 1,250 patients up to around 1,800 patients, resulting in an approximate increase in the split by planned RT schedule from 625 to 900 in each "within schedule" analysis. A FFS analysis "within schedule" will be carried out at the time of the "main analysis"; predicted to have ~300 control arm FFS events by schedule (FFS "within schedule" analysis parameters: target HR=0.75, power 90%, 1-sided α=0.015). For either of the RT schedules showing evidence of an effect on FFS, a comparative "within schedule" analysis will be carried out on survival when ~199 control arm deaths are observed in that schedule comparison. This is a closed test with OS only formally compared within schedule if there is an advantage in FFS for that RT schedule at the main analysis. Thus, extending recruitment enables a secondary analysis of the impact of RT on survival by planned "RT schedule" to happen within around 18 months from the first main analysis.

All sample scenarios are documented in the Trial Master File.

All patients joining the trial will be starting long-term ADT for the first time. The focus of this comparison will be on the newly-diagnosed, metastatic patients (and no contraindications to RT), which is the largest subgroup of patients in the trial and the group of patients at highest risk of death from prostate cancer. Patients with non-metastatic disease will be excluded from this particular comparison as there are already randomised data demonstrating the survival benefit from radiotherapy in patients with locally advanced disease. Radiotherapy is now mandatory in node negative patients; it is also recommended in the node-positive, non-metastatic (N+ M0) group. Relapsing patients are also excluded from this comparison.

For the control arm of the whole trial, we constructed sample size scenarios median failure-free survival being 18, 24 or 30 months and constructed sample size scenarios around each of these options; the event rate would depend on the patient mix. We now know that around 60% of patients have M1 disease at trial entry and we have reported that FFS at 24 months is 51% across the whole of the control arm.(1)

For the sample size calculation for this new planned comparison, we have based our estimates on the subgroup of patients with newly-diagnosed M1 disease in the control arm. Therefore, we estimate median FFS to be 1 year and estimate that median overall survival will be 3.5 years.

9.7 SAMPLE SIZE ISSUES AND TRIAL STAGES: ADDITIONAL RESEARCH ARM J

9.7.1 Pilot Phase: Additional Research Arm J

A similar approach will be followed for the additional research Arm J as detailed for the original research arms in **Section 9.4.1**. The IDMC will first review safety data for this combination when the first 50 patients allocated to Arm J have been on trial around 6 weeks (i.e. to the first follow-up visit).

The IDMC will review safety data again when 50 patients are 6 months out from randomisation. Additional safety reviews will be performed if the IDMC raises any concerns over safety and routinely reviewed at regular intervals.

Direct comparison will be available with contemporaneously randomised patients on Arm A (hormones alone). Contextual data will be provided from Arm G (hormones plus abiraterone). Indicative safety data may also be available on the combination from other studies in CRPC.

9.7.2 Activity Stages I-II: Additional Research Arm J

The principles of intermediate analyses will be applied to this new comparison, but some of the details will be different. Owing to the expected accrual rate (>100 pts/m) and the expected slower event rate, only two activity stages are planned before accrual is completed. These are set out in **Table 12**.

Table 12: Guidelines for stopping accrual to the additional research Arm J

Activity Stage	Sig Level	Power	Targeted HR	Number of control arm events	Consider discontinuation if HR_J(observed) is...
I	0.40	95%	0.70	~66	>0.957
II	0.12	95%	0.70	~139	>0.869

9.7.3 Efficacy Stage III: Additional Research Arm J

The analysis of the final Efficacy Stage for this comparison will be performed when around 267 deaths have been observed in the control arm. This would give 90% power to detect the targeted hazard ratio of 0.75 at a one-sided significance level of 0.025.

9.7.4 Sample Size For Additional Research Arm J

Consideration will be given to ceasing further randomisations to Arm J if it is not showing sufficient evidence of activity on the intermediate primary outcome measure (FFS), just as for the other research arms.

The patient mix for this comparison is likely to represent a more favourable prognosis on average than in the original research trial's other arms, due to concurrent recruitment of M1 but not M0 patients, to Arm H.

We anticipate that around 1800 patients are required within 3.5 years to observe ~267 control arm deaths within 6 years. This time will be dependent on the observed overall survival. The default scenario assumes that (i) recruitment is constantly 70pts/m to the trial overall, (ii) the M1|RT arm accrues throughout and (iii) a further new research arm with an equal allocation ratio is introduced 18 months after activation of Arm J. The stopping date for Arm G is no longer an assumption.

Variations on these factors are documented in a Statistical Design Document. If accrual rates to the trial are at 150pts/m (as observed during Summer 2013), accrual of around 1,800 patients to the comparison could be achieved within 2 years. These sample scenarios will also be documented in the Trial Master File.

9.7.5 FURTHER SAMPLE SIZE ISSUES FOR ADDITIONAL RESEARCH ARM J

Careful consideration will be given to the emerging data from the abiraterone comparison (Arm A vs Arm G) and whether this arm continues to recruit throughout. It is anticipated that recruitment to this Arm J comparison will be completed *before* survival data emerge from the abiraterone comparison.

Indirect comparisons to understand the contribution from each agent may be possible if this research arm is demonstrably superior to the standard-of-care. These plans will be developed and documented elsewhere, but a higher number of patients will help with the power to the indirect comparison.

Figure 11: Schema of progress of STAMPEDE through the trial

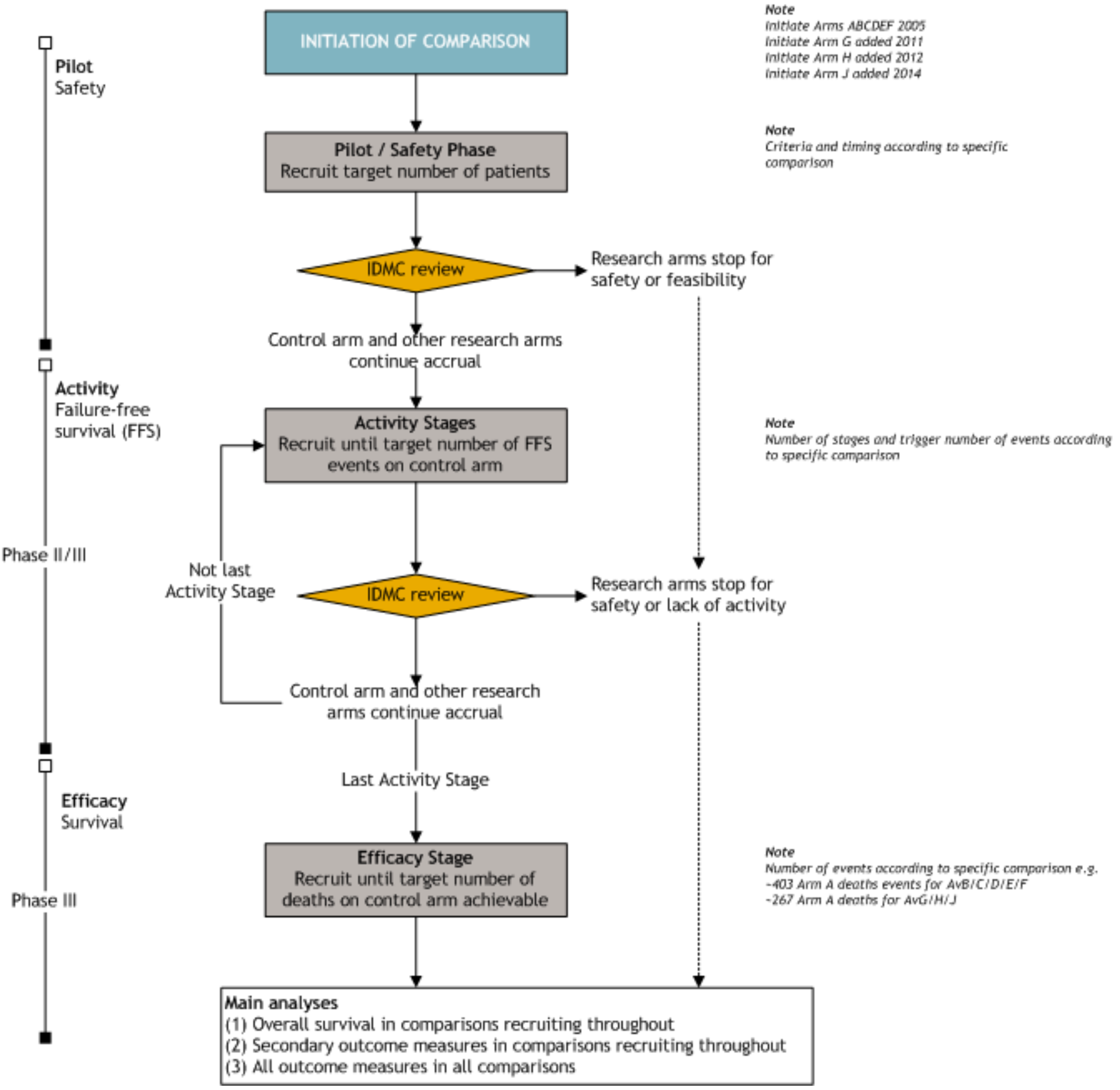

Key

FFS: Failure-free survival
HR: Hazard ratio
IDMC: Independent Data Monitoring Committee
Pts: Patients

Notes

Exact accrual depends on many factors including accrual rate, event rate and arms recruiting in each stage

9.8 FURTHER NOTES ON TRIAL DESIGN

9.8.1 OVERALL SAMPLE SIZE

Given the adaptive nature of the study, there is no formal overall sample size target, but the numbers of patients required for each comparison are detailed in **Sections 9.4** to **9.7**. It is expected that more than 7,000 patients will likely be recruited overall.

9.8.2 FACTORIAL DESIGN

We note here that we have not employed a factorial design in this trial because we anticipate the possibility of synergy between ADT, zoledronic acid and docetaxel and between ADT, zoledronic acid

and celecoxib. It would not be possible to assess any such interactions reliably in a factorial trial (see the Statistical Design Document for further details).

9.9 INTERIM MONITORING AND ANALYSES

The accumulating data will be reviewed at regular intervals (approximately annually) by an Independent Data Monitoring Committee (IDMC), including pre-specified formal intermediate analyses of activity data (see also **Section 16**). These analyses will be performed by the trial team at the MRC CTU. Only patients randomised contemporaneously will be included in the comparison of each research arm against control e.g. patients allocated to the control arm prior to protocol version 12.0 will not contribute to the "enzalutamide + abiraterone comparison" (Arm A vs Arm J).

The IDMC will be asked to give advice on whether the accumulating data from the trial with the guidelines for discontinuation of accrual for the relevant Activity Stages, together with results from any other relevant trials, justifies continuing recruitment of further patients or further follow-up. A decision to discontinue recruitment, in all patients or in selected subgroups will be made only if the result is likely to convince a broad range of clinicians including those entering patients into the trial and the general clinical community. The intermediate stopping guidelines apply to the intermediate primary outcome measure. To stop accrual early for benefit in any comparison would require convincing data in terms of the definitive primary outcome measure, overall survival. For example, this could be $p<0.001$ as proposed by Haybittle-Peto.(61, 62) The use of such a guideline for stopping for benefit has a minimal impact on the operating characteristics.

If a decision is made to continue without change, the IDMC will advise on the frequency of future reviews of the data on the basis of accrual and event rates. The IDMC will make recommendations to the Trial Steering Committee (TSC, see **Section 16**) as to whether the trial should continue in its present form. While the trial is ongoing the accumulating data will generally remain confidential, unless the TSC and IDMC agree that the data should be made public.

9.10 OUTLINE ANALYSIS PLAN

Analyses will be performed on an intention-to-treat basis. The standard unadjusted log-rank approach will be applied to analyses of FFS and OS. The impact of potential confounders including the stratification factors used at randomisation will be considered in a Cox proportional hazard model. Flexible parametric models will be used to calculate the absolute differences between the arms to show treatment differences over time and to estimate restricted mean "survival" times (RMST). The estimated difference in restricted means survival time will be used preferentially to compare treatment arms if the proportional hazards assumptions required for hazard ratios cannot be supported. The χ^2 test or Mann-Whitney test will be implemented for categorical data comparisons, including toxicity, as appropriate. The primary outcome measures (see **Section 9.2**) will be considered for all arms of the trial at each phase, but the main emphasis will be placed on the comparison of the research arms that have continued to recruit throughout the trial.

9.10.1 PILOT / SAFETY PHASES

The Pilot Phase randomised patients between all the trial arms so that the results from these patients can be included in the main trial. Feasibility is considered in terms of the acceptability of the trial randomisation and reported toxicities and adherence to trial medication. Centres participating in the Pilot Phase for the original research arms were required to keep an anonymised log of all

patients assessed for trial eligibility (see protocol version 2.0) so that the number of patients who did not participate in the study and the number of eligible patients who choose to not participate in the study could be summarised (reasons for non-participation were collected where the patients was willing). The anonymised logs will not be needed for new research arms after Protocol version 8.0.

For the patients who are randomised, we shall describe the incidence of expected and unexpected severe toxicities and adverse events/reactions (see **Section 11**) to decide whether to continue with research arms beyond the Pilot Phase. As indicated above, we do not anticipate that recruitment to the research arms will be discontinued after the Pilot Phase, as there is considerable experience with zoledronic acid and docetaxel when combined with ADT, while Cox-2 inhibitors generally have a good toxicity profile. Although there are limited data on the combinations, we do not expect severe toxicity.

9.10.2 Activity And Efficacy Stages

The approach to analysis of these stages is summarised within the sample size calculations (see earlier subsections of **Section 9.4.3**). Each research arm will be compared in a pairwise fashion against the control arm.

Full details are available in the Statistical Analysis Plan.

10 MONITORING AND QUALITY ASSURANCE

10.1 MONITORING AT MRC CTU

Data provided to the MRC CTU will be checked for missing or unusual values (range checks) and consistency over time. If missing or questionable data are identified, staff at the MRC CTU will request that the data be clarified. The exact procedures for data clarification and the amendment of CRFs will be described in the trial specific SOPs and instructions will be sent to all STAMPEDE institutions as soon as they have been approved to participate in the trial. The MRC CTU will also send reminders for any overdue data.

10.2 DIRECT ACCESS TO DATA

Collaborating institutions should be aware that direct access to patient data by MRC CTU staff may be required for trial-related monitoring or audit. Patient consent for this will be obtained as part of the general trial consent process.

10.3 VISITS TO INVESTIGATOR SITES

A selection of institutions will be visited at least once during the course of the STAMPEDE trial. The MRC CTU will give the responsible investigator adequate notice of the monitoring visit to allow adequate time, space and staff for these visits. The standard operating procedures (SOP) for monitoring are available from the MRC CTU.

After the monitoring visit the monitor will complete a site visit report. This report will be circulated to the TMG for comment. Once the TMT have reviewed the report and agreed on any recommendations the monitor will finalise the report and send a copy to the Principal Investigator (PI) at the site. A copy will also be sent to the CI and TMG for the trial and another copy will be kept in the MRC CTU STAMPEDE trial master file.

10.4 CONFIDENTIALITY

All information collected during the course of the research will be kept strictly confidential. In addition, all procedures for handling, processing, storage and destruction of data are compliant with the Data Protection Act 1998. No individual patients will be identified when the results of the trial are published.

Patients will be asked for permission for information about their health status to be obtained from the Office of National Statistics (ONS) or via the NHS Strategic Tracing Service or similar by the Medical Research Council, if necessary. In addition, patients will be asked for permission to inform their GP of their involvement in the STAMPEDE trial.

11 SAFETY REPORTING

ICH GCP requires that both investigators and sponsors follow specific procedures when reporting adverse events/reactions in clinical trials. These procedures are described in this section of the protocol. Further information on the expected toxicities for the trial interventions (docetaxel, zoledronic acid, abiraterone and radiotherapy) can be found in **Appendix G**.

11.1 DEFINITIONS

The safety reporting definitions from ICH GCP apply in this trial protocol. These definitions are given in **Table 13**.

Table 13: Event Terms and Definitions

TERM	DEFINITION
Adverse Event (**AE**)	Any untoward medical occurrence in a patient or clinical trial subject to whom a medicinal product has been administered including occurrences which are not necessarily caused by or related to that product.
Adverse Reaction (**AR**)	Any untoward and unintended response to an investigational medicinal product related to any dose administered.
Unexpected Adverse Reaction (**UAR**)	An adverse reaction, the nature or severity of which is not consistent with the information about the medicinal product in question set out in the summary of product characteristics (or Investigator brochure) for that product.
Serious Adverse Event (**SAE**) or Serious Adverse Reaction (**SAR**) or Suspected Unexpected Serious Adverse Reaction (**SUSAR**)	Respectively any adverse event, adverse reaction or unexpected adverse reaction that:<br>• results in death<br>• is life-threatening*<br>• requires hospitalisation or prolongation of existing hospitalisation**<br>• results in persistent or significant disability or incapacity<br>• consists of a congenital anomaly or birth defect<br>• Other important medical condition***

Clarifications and Exceptions

*The term 'life-threatening' in the definition of 'serious' refers to an event in which the patient was at risk of death at the time of the event; it does not refer to an event which hypothetically might have caused death if it were more severe.

**Hospitalisation is defined as an inpatient admission, regardless of length of stay, even if the hospitalisation is a precautionary measure for continued observation. Hospitalisations for a pre-existing condition (including elective procedures that have not worsened) do not constitute an SAE.

***Medical judgement should be exercised in deciding whether an AE/AR is serious in other situations. Important AE/ARs that are not immediately life-threatening or do not result in death or hospitalisation but may jeopardise the subject or

may require intervention to prevent one of the other outcomes listed in the definition above, should also be considered serious.

Pregnancy occurring in a STAMPEDE patient's partner during the patient's participation in the trial, must be reported to the MRC CTU within the same timelines as an SAE and classified as an 'other important medical condition' on the SAE form. The outcome of a pregnancy should be followed up carefully and any abnormal outcome to the mother or child should be reported.

Patients who develop any new primary carcinomas should have the event reported on a SAE CRF as "other important medical condition".

11.1.1 Trial-Specific Exemptions

Disease progression or death as a result of disease progression are not considered to be SAEs and should be reported on the STAMPEDE Progression Form or Death Form only.

The following situations that fulfil the definition of an SAE are excluded from expedited notification on an SAE form and should be reported only on the STAMPEDE follow-up form:

- Elective hospitalisation and surgery for treatment of locally advanced or metastatic prostate cancer or its complications
- Elective hospitalisation to simplify treatment or procedures
- Elective hospitalisation for pre-existing conditions that have not been exacerbated by trial treatment

11.2 INSTITUTION/INVESTIGATOR RESPONSIBILITIES

All non-serious AEs/ARs, whether expected or not, should be recorded in the toxicity (symptoms) section of the Follow-up CRF and sent to the MRC CTU within one month of the form being due. SAEs/SARs should be notified to the MRC CTU as described below.

The severity (i.e. intensity) of all AEs/ARs (serious and non-serious) in this trial should be should be graded using Common Terminology Criteria for Adverse Events (CTCAE) v3.0 (ctep.cancer.gov/reporting/index.html). A flowchart is given in **Appendix I** to help explain the notification procedures. Any questions concerning this process should be directed to the MRC CTU in the first instance.

11.2.1 Investigator Assessment

11.2.1.A Seriousness

When an AE/AR occurs the investigator responsible for the care of the patient must first assess whether the event is serious using the definitions given in **Table 13**. If the event is serious and not exempt from expedited reporting, then an SAE form must be completed and the MRC CTU notified.

11.2.1.B Causality

The Investigator must assess the causality of all serious events/reactions in relation to the trial therapy using the definitions in **Table 14**. There are 5 categories: unrelated, unlikely, possible, probable and definitely related. If the causality assessment is unrelated or unlikely to be related the event is classified as a SAE. If the causality is assessed as either possible, probable or definitely related then the event is classified as a SAR.

Table 14: Assigning type of SAE through causality

RELATIONSHIP	DESCRIPTION	EVENT TYPE
Unrelated	There is no evidence of any causal relationship	Unrelated SAE
Unlikely	There is little evidence to suggest there is a causal relationship (e.g. the event did not occur within a reasonable time after administration of the trial medication). There is another reasonable explanation for the event (e.g. the patient's clinical condition, other concomitant treatment).	Unrelated SAE
Possible	There is some evidence to suggest a causal relationship (e.g. because the event occurs within a reasonable time after administration of the trial medication). However, the influence of other factors may have contributed to the event (e.g. the patient's clinical condition, other concomitant treatments).	SAR
Probable	There is evidence to suggest a causal relationship and the influence of other factors is unlikely.	SAR
Definitely	There is clear evidence to suggest a causal relationship and other possible contributing factors can be ruled out.	SAR

11.2.1.C Expectedness

If the event is a SAR the Investigator must assess the expectedness of the event. Please see Appendix G (Table G.2) for a list of expected toxicities associated with the drugs being used in this trial. If a SAR is assessed as being unexpected it becomes a SUSAR.

11.2.1.D Notification

Investigators must notify the MRC CTU of all SAEs occurring from the time of randomisation until 30 days after the last protocol treatment administration for any research arm in the trial. Similarly, SAEs occurring in patients randomised to Arm A must be reported until 30 days after last injection or progression (whichever is sooner). SARs and SUSARs must be notified to the MRC CTU indefinitely for all research arms (i.e. no matter when they occur after randomisation).

11.2.2 NOTIFICATION PROCEDURE

The SAE form must be completed by the Investigator (consultant named on the signature list and delegation of responsibilities log who is responsible for the patient's care), with due care being paid to the grading, causality and expectedness of the event as outlined above. In the absence of the responsible investigator the form should be completed and signed by a member of the site trial team. The responsible investigator should subsequently check the SAE form, make changes as appropriate, sign and then re-fax to the MRC CTU as soon as possible. The initial report shall be followed by detailed, written reports as appropriate.

Send the SAE form by fax to the MRC CTU. Fax Number: + 44 (0) 20 7670 4818. The STAMPEDE trial team will confirm receipt of the SAE report to the main point of contact via email. Contact the STAMPEDE trial team If receipt is not received within 24 hours.

Follow-up: Patients must be followed-up until clinical recovery is complete and laboratory results have returned to normal or baseline, or until the event has stabilised. Follow-up should continue after completion of protocol treatment if necessary. Follow-up information can be updated on the originial SAE form by ticking the box marked 'follow-up' and faxing to the MRC CTU as information becomes available. Extra, annotated information and/or copies of test results may be provided separately. The patient must be identified by trial number, date of birth and initials only. The patient's name should not be used on any correspondence.

11.3 MRC CTU RESPONSIBILITIES

Medically qualified staff at the MRC CTU and/or the Chief Investigator (or a medically qualified delegate) will review all SAE reports received. The causality assessment given by the local Investigator at the hospital cannot be overruled and in the case of disagreement, both opinions will be provided in any subsequent reports.

The MRC CTU is undertaking the duties of trial sponsor and is responsible for the reporting of SUSARs and other SARs to the regulatory authorities (MHRA and competent authorities of other European member states and any other countries in which the trial is taking place) and the research ethics committees as appropriate.

The MRC CTU will also keep all investigators informed of any safety issues that arise during the course of the trial.

SAE REPORTING
Fax to 020 7670 4818 within 24 hours of becoming aware of the event

12 ETHICAL CONSIDERATIONS AND APPROVAL

12.1 ETHICAL CONSIDERATIONS

This is a randomised trial therefore neither the patients nor their physicians will be able to choose the patients' treatment. Treatment will be allocated randomly using a computer-based algorithm. This is to ensure that the groups of patients receiving each of the different treatments are similar.

Androgen deprivation therapy alone is the standard treatment for these forms of prostate cancer. Patients may be randomised to one or two of the newer treatments in combination with hormone treatment. The trial has employed an unequal allocation ratio for some comparisons to maximise efficiency; this was explained in detail in the patient information sheet.

The newer combined treatment options are being assessed in a detailed and systematic fashion in this trial. There is some evidence to suggest that the newer treatment options may have advantages over standard treatment (androgen deprivation therapy) alone with regards clinical outcome, but this is not confirmed and toxicity may be increased. This trial will follow a large group of men who have been randomly allocated to either the standard treatment (androgen deprivation therapy alone) or the newer combined treatment options in order to measure the benefits of the new treatments. The patients will also be followed-up for toxicity and safety issues, so that any benefits can be weighed against any negative aspects.

Patients participating in the trial will have some additional hospital visits and some extra blood samples taken compared to patients who are not participating in the trial, with the amount varying according to the allocated treatment. Sometimes the blood samples can be taken when the patient is attending hospital for treatment, anyway. On some of the trial arms, the patient may have to make additional visits to the hospital for the blood sample to be taken, although in some cases it may be possible for the blood sample to be taken in the GP's surgery. The additional visits and blood samples are to ensure that follow-up of patients is comparable in all the treatment groups. The blood samples will also be used for genetic and serum marker studies, where this information will be considered with clinical data. Blood samples will be link-anonymised. There will be no feedback to individual patients.

If new information emerges during the course of the trial which may affect the treatment or follow-up of patients who have joined the trial, information will be provided through by the trial team to all Principal Investigators. PIs have therefore the duty to inform patients in their care of any new information emerged using any appropriate channel (e.g. letter, communication at follow up clinic, etc).

12.2 ETHICAL APPROVAL

The protocol has a Favourable Opinion from an appropriate Research Ethics Committee, according to national guidelines. Additionally, each site must also obtain management permission for research (R&D approval) from the relevant host organisations before patients can be entered into the trial. The patient's informed consent to participate in the trial should be obtained after a full explanation has been given of the treatment options, including the conventional and generally accepted methods of treatment. Patient information sheets and patient consent forms are given in **Appendix B**.

The right of the patient to refuse to participate in the trial without giving reasons must be respected. After the patient has entered the trial, the clinician must remain free to give alternative treatment to that specified in the protocol, at any stage, if he feels it to be in the best interest of the patient. However, the reason for doing so should be recorded and the patient will remain within the trial for the purpose of follow-up and data analysis according to the treatment option to which he has been allocated. Similarly, the patient must remain free to withdraw at any time from the protocol treatment without giving reasons and without prejudicing his further treatment.

A statement of MRC policy on ethical considerations in clinical trials of cancer therapy, including the question of informed consent, is available from the MRC Head Office web site (http://www.mrc.ac.uk).

13 REGULATORY APPROVAL

This trial has been approved in the UK by the MHRA and will be conducted under a CTA (Ref: 00316/0026/001-0001) in the UK.

The trial has been approved in Switzerland by Swissmedic (Ref: 2009 DR 3235).

14 INDEMNITY

University College London holds insurance against claims from participants for injury caused by their participation in this clinical trial. Participants may be able to claim compensation if they can prove that UCL has been negligent. However, as this clinical trial is being carried out in a hospital, the hospital continues to have a duty of care to the participant of the clinical trial. University College London does not accept liability for any breach in the hospital’s duty of care, or any negligence on the part of hospital employees. This applies whether the hospital is an NHS Trust or otherwise.

Participants may also be able to claim compensation for injury caused by participation in this clinical trial without the need to prove negligence on the part of University College London or another party. Participants who sustain injury and wish to make a claim for compensation should do so in writing in the first instance to the Chief Investigator, who will pass the claim to the managing organisation’s Insurers, via the managing organisation’s office.

Hospitals selected to participate in this clinical trial must provide clinical negligence insurance cover for harm caused by their employees and a copy of the relevant insurance policy or summary can be provided on request.

15 FINANCE

STAMPEDE is funded by the Clinical Trials Advisory Awards Committee (CTAAC) on behalf of Cancer Research UK; it is also funded by the MRC through the MRC Clinical Trials Unit. The trial has National Cancer Research Network (NCRN) approval and, therefore, local NCRN funds may be available at each centre to support entry of patients into this trial.

Zoledronic acid is manufactured by Novartis. Novartis have agreed to provide an educational grant to support the conduct of this study. Novartis have also agreed to supply the study drug, zoledronic acid free of charge for patients participating in the study.

Docetaxel is manufactured by Sanofi-Aventis Pharma. They have agreed to supply the study drug, docetaxel at a discounted rate for patients that are participating in the trial and to provide an educational grant to support the conduct of the study. The Department of Health has agreed to provide a central subvention as follow: £1,787 per patient randomised to Arms C and E of the trial and prescribed docetaxel. This amount is payable in respect of a hospital trust randomising more than 3 patients. For more details contact the STAMPEDE Trial Manager.

Celecoxib is manufactured by Pfizer. They agreed to supply free drug and to provide funds to distribute drug to participating sites.

Abiraterone is manufactured by Janssen Pharma PV (pharmaceutical companies of Johnson & Johnson). They have agreed to provide free drug and funds to distribute drug to participating sites and to help support the conduct and management of the trial.

Enzalutamide is manufactured by Astellas Pharma. They have agreed to provide free drug and funds to distribute drug to participating sites and to help support the conduct and management of the trial.

16 TRIAL COMMITTEES

16.1 TRIAL MANAGEMENT GROUP (TMG)

A Trial Management Group (TMG) has been formed comprising the Chief Investigator, other co-investigators and members of the MRC CTU. The membership of the TMG may be expanded if other groups of trialists wish to participate. It will also be amended during the trial if other circumstances require e.g. retirement.

The TMG will be responsible for the day-to-day running and management of the trial and will meet by teleconference at least 3 monthly and in person as needed. The TMG members are detailed in **Appendix K**.

Further details of TMG functioning are provided in the TMG charter (available on request).

16.2 TRIAL STEERING COMMITTEE (TSC)

A Trial Steering Committee (TSC) has been formed to provide overall supervision for the trial and provide advice through its independent chair. The ultimate decision for the continuation of the trial lies with the TSC. The TSC will meet regularly.

Further details of TSC functioning are provided in the TSC charter (available on request).

16.3 INDEPENDENT DATA MONITORING COMMITTEE (IDMC)

An Independent Data Monitoring Committee (IDMC) has been formed. The IDMC will be the only group who sees the confidential, accumulating data to the trial. Reports to the IDMC will be produced by the MRC CTU. The IDMC will meet within 6 months of the trial opening with the frequency of meetings dictated by the IDMC. The IDMC will consider data in accordance with the analysis plan (see **Section 9.5**) and will be advisory to the TSC. The IDMC can recommend premature closure or reporting of the trial, or that recruitment to any research arm be discontinued.

From protocol version 8.0 onwards, any recommendation from the IDMC to stop recruitment to one or more trial arms will be acted upon immediately, pending ratification from the TSC. As this period between meetings should be very short, sites would not be notified until after the TSC have made a decision. IDMC recommendations based on emerging safety issues would be discussed with sites promptly.

Further details of IDMC functioning and the procedures for interim analysis and monitoring are provided in the IDMC charter (available on request).

Figure 12: Diagram of relationships between trial committees

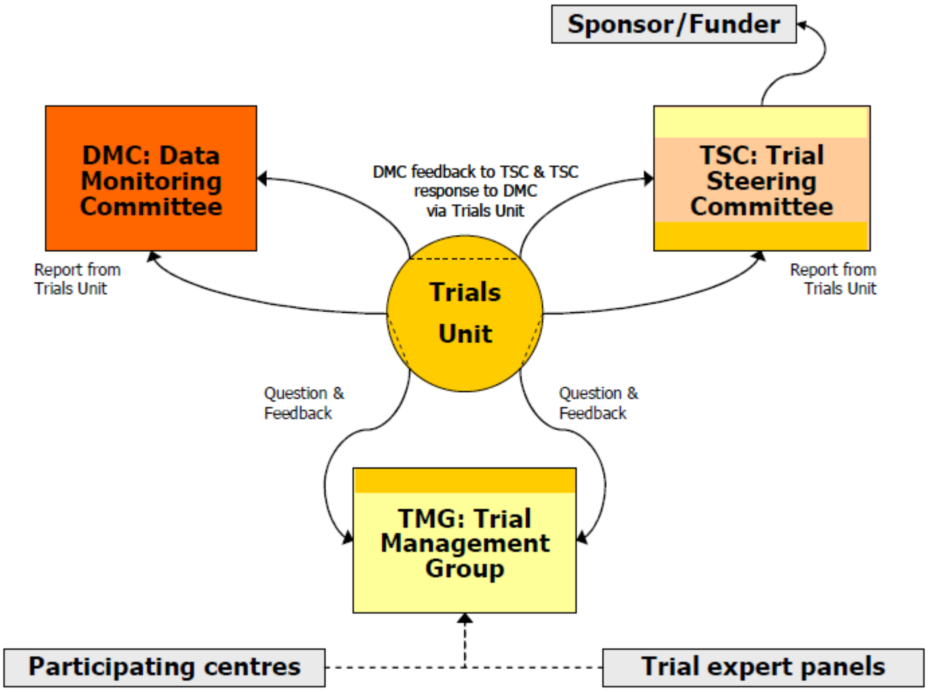

17 ANCILLARY STUDIES

17.1 QUALITY OF LIFE

A quality of life (QL) study is being performed to assess the impact of each treatment arm on the quality of patient's lives and participation in this study was limited to the first 700 patients recruited (this was reached in September 2008) patients. The QL study re-opened from the implementation of version 8.0 of the protocol. The EORTC QLQ-C30 with the prostate-specific module QLQ PR25 will be used. Key items for assessment are pain reduction for patients with metastatic disease and urinary symptoms for patients with locally advanced disease. In addition specific hypotheses will be generated for each of the research arms. The EuroQol (EQ-5D) (63) will be used in the study as a generic measure of health-related quality of life which can be linked to public preferences. These data will be used to calculate quality-adjusted life-years as part of the economic evaluation (see **Section 17.2**). Patients who were recruited into the QL study, should continue on the study throughout the trial. Questionnaires should be self-administered, although it is recommended that a key person (e.g. research nurse) at each centre be responsible for the data collection to optimise compliance and completeness of the data.

The QL and the HE questionnaires should be completed without conferring with friends or relatives and all questions should be answered even if the patient feels them to be irrelevant.

The responsible person should check each questionnaire for its completeness, ensuring that the correct date of completion and patient identifiers are present. The research nurse should approach patients at appropriate clinical visits to complete a questionnaire. If no clinical visit is scheduled for the patient (with a window of 4 weeks around the expected date) the nurse should organise the completion of the questionnaire, by post or by a visit to the patient at home (or in a hospice).

17.2 HEALTH ECONOMICS

A health economics (HE) sub-study will be performed. Core resource use information will be collected, using CRFs on days in hospital (by speciality) and outpatient visits. Data being collected on concomitant medication will also be used in the economic analysis. Information on patients' use of primary care and community-based services will be collected as additional questions in the QL questionnaire. Costs will be calculated on the basis of representative UK unit costs at the point of analysis. Health outcomes will be assessed in terms of quality-adjusted life years (QALYs). Quality adjustments will be based on patients' responses to the EQ-5D health status measure which will be administered at baseline and each point of follow-up as part of the QL questionnaire. A cost-effectiveness analysis will compare all regimens that continue to recruit into their Activity Stage IV.

17.3 TRANSLATIONAL SUB-STUDIES

17.3.1 DNA Analysis

Blood samples from as many patients as possible have been collected for future translational research. With patient consent, an additional droplet of blood sample has been collected using FTA Elute cards and stored for DNA and protein analysis in order to try to identify molecular features of clinical significance.

FTA Elute cards supplies have not been available since Dec-2013 and the STAMPEDE TMG has pursued an alternative method for genomic DNA collection using the Oragene® DNA kits for saliva sampling.

Oragene kits are widely used for collection of DNA from patients participating in clinical trials and they have been demonstrated to be a suitable alternative to DNA collection from whole blood providing a non-invasive, painless method of high quality sample collection.

A subset of patients may be asked if they would like to donate a blood sample for additional genetic research analysis.

Details of specimen collection, posting and contact details are given in **Appendix D**.

17.3.2 TISSUE MICROARRAY

Patient consent will be sought to utilise paraffin embedded tissue for the construction of tissue microarrays from needle cores. One needle biopsy will be selected for microarray and the remaining tissue will be returned to the originating histopathology lab. Given the entry criteria for the trial, the majority of patients will have extensive disease in the diagnostic needle core biopsies, in contrast to men with localised, low grade disease. Consequently, removal of one core is unlikely to compromise any subsequent histopathological assessment. Details regarding transfer of samples will be issued at the time of construction of the micro array. Additional analyses e.g. DNA extraction may also be performed on the tissue arrays.

18 PUBLICATION

The results from different centres will be analysed together and published as soon as possible. Individual clinicians must not publish data concerning their patients that are directly relevant to questions posed by the study until the TMG has published its report. The TMG together with the STAMPEDE collaborators will form the basis of the writing committee and decide on the nature of publications. Any release, of efficacy or safety data, presentation or publication will be agreed with the TSC according to the terms of their charter.

All publications will acknowledge the participating centres and clinicians, and these will be detailed in an appendix to the main report. Papers will have named authors determined by the TMG according to the following principles:

- To be as inclusive as possible where this is practicable
- To ensure that there is justification for anyone to be named as an author
- Reasons for nomination for authorship may include: trial design; grant holding; day-to-day trial oversight (TMG membership); analysis; discussion and interpretation of data; representation for key groups; active participation at large recruiting sites. It should be accepted that the people qualifying for authorship will vary over time. In addition, key positions will vary depending on the nature of the publication: clinical lead for clinical papers, statistician lead for methodology papers, translational papers may be led by authors not on the main TMG if appropriate (e.g., the bone sub-study). In the event of any dispute related to authorship or data release, the TSC will be responsible for making the executive decision.

In the manuscript, a full list of sites and the number of patients recruited will be provided. In the presentations, this list of sites will also be shown. The term “the STAMPEDE investigators” will clearly be stated and relevant names included in the presentation credits.

A detailed Publication Plan is documented elsewhere.

19 PROTOCOL AMENDMENTS

19.1 PROTOCOL

19.1.1 Amendments Made To Sections In Protocol Version 1.0 (May 2004)

Administrative changes such as typos, word change etc.

Name additions/changes to:

TMG members

TSC members

IDMC members

'General Information' Section – additional information re. Abridged version of protocol

Section 1.2 – Figure 1, Celecoxib duration amended

Section 1.3 – Figure 2, addition of cardiovascular assessment form, name and timings amended

Section 2.3 – Docetaxel information updated

Section 2.4 – Additional text re dose and duration justification for Celecoxib use.

Section 3 – Title change and content updated

Section 4.2 – New exclusion criteria added

Section 4.3.1 – New investigations added and additional text re testosterone measurements and additional text re. prior celecoxib treatment

Section 6.1.4 – Celecoxib duration amended

Section 6.1.5 – Additional text re. Co-administration of docetaxel and bisphosphonates

Section 6.1.6 – Celecoxib duration amended

Section 6.2.2 – additional docetaxel information

Section 6.2.3 – addition of CV event history

Section 11 – Safety reporting updated

Section 12.1 – Additional text re. the collection of blood for genetic and serum marker studies

Section 15 – Additional information re. Central Subvention for docetaxel arms

19.1.2 Amendments Made To Sections In Protocol Version 1.1 (May 2005)

Section 6.2 Administration and Dose Modifications, subsection 6.2.1 Zoledronic Acid

19.1.3 Amendments Made To Sections In Protocol Version 2.0 (Jun 2005)

General Information section – SAE reporting fax number and timeframe added.

Section 1.2 – Addition of anti-androgen use for M0 patients as a method of HT

Section 1.2 – Increase in amount of blood needed & addition tissue sample request.

Section 1.3 Trial Documentation updated to include new table detailing trial documentation ahead of accreditation, the inclusion of the radiotherapy forms and correct case report form timings

Section 2.1 – Addition of anti-androgen use for M0 patients as a method of HT

Section 4.1.3 – Inclusion criteria Vii "Normal testosterone prior to hormone treatment" removed.

Section 4.1.3 - φnote has been omitted and moved to section 4.2 (see number 8)

Section 4.2 – Exclusion criteria added to exclude patients with active peptic ulceration, gastrointestinal bleeding and inflammatory bowel disease.

Section 4.2 – Exclusion Criteria added to exclude patients with planned major dental work

Section 4.3.1 - All blood test timelines changed from 14 days to 28 days.

Section 4.3.1 – Hormone Therapy pre-randomisation deadline extended from 4 weeks to 12 weeks.

Section 4.3.1 – Additional information regarding the use of NSAIDs and cox-2-inhibitors before coming on to the STAMPEDE study and once commenced on study treatment

Section 4.3.2 – Updated to ask for all vitamins and minerals the patient is taking to be recorded.

Section 4.3.3 – Updated to include the extra blood required and the request for consent of patients' tissue samples.

Section 6.1.1 – Addition of anti-androgen use for M0 patients as a method of HT

Section 6.1.6 – Addition of the calcium & vitamin name "calcichew".

Section 6.6.2 – asking also to collect vitamins and minerals under concomitant medication.

Section 6.6.3 – New section to inform investigators that patient's, who they wish to give radiotherapy to, are also eligible for STAMPEDE

Section 6.6.4 – New section to detail what data is being collected on the radiotherapy given to patients.

Section 7.1; figure 4 – Addition of radiotherapy form and in note, addition of AA alone

Section 7.1.2 – omission of repeated scans and x-rays at 24 weeks, also omitted in note under figure 4.

Chapter 11 – Safety reporting section updated

Section 17.3 – Increase in amount of blood needed & additional tissue sample request.

19.1.4 Amendments Made To Section In Protocol Version 3.0 (Jul 2006)

Front Cover - NCRN logo added for accuracy

Front Cover - Clarification that protocol developed with NCRI rather than on behalf of

Front Cover - Clarification that it is a 6 arm trial

General Information section - MRC CTU staff section updated

Section 1.2 – Statistics section updated.

Section 1.2 - Additional research paragraph updated to reflect additional studies and for clarification of terms

Section 1.2 - Blood collection volume changed to reflect new technique used

Section 1.3 (figure 3) - Table showing case report form schedule updated to reflect clarification of follow-up schedule and addition of new CRF (End of Treatment)

Section 2.2 - AS changed to HT (clarification of terms)

Section 2.3 - Updated in information in regard to use of docetaxel added to reflect up to date practice

Section 2.5 - Sub-headings numbered for consistency

Section 3.0 - Information in regard to the Pilot Phase now written in past tense as Pilot Phase has now been completed

Section 4.1.1 - Inclusion criteria extended so that patients who fulfil 2 out of the three of the first inclusion criteria can be eligible.

Section 4.3.1 - Change in time scales by which baseline investigations need to be completed.

Section 4.3.1 - Clarification that chest X-ray is only required if chest is not included in the CT

Section 4.3.1 - Removal of 12 week timeline for baseline PSA test to be performed. (Stipulation that it must be performed before start of HT)

Section 4.3.2 – Information added in regard to time allowed from randomisation to start of treatment

Section 4.3.3 - Additional research paragraph updated to reflect additional studies and for clarification of terms

Section 4.3.3 - Blood collection volume changed to reflect new technique used

Sections 6.1.2-6.1.6 - Androgen Suppression replaced with hormone therapy for consistency of terms

Section 6.2.2 - '(Taxotere)' Removed for consistency

Section 6.2.2 _ information added in regard to the need to closely monitor liver function prior to docetaxel administration

Section 7.1 - Page number reference updated

Section 7.1.1 - PSA measurement timings updated to accurately reflect follow-up schedule

Section 7.3 (Table 4) - Table and key updated to accurately reflect follow-up schedule and to include information about new CRFs and removal of withdrawal CRF

Section 8 - Rewording for clarification of definition of trial withdrawal

Section 8.1 - Instruction that withdrawal from trial treatment should be recorded on End of Treatment Form rather than withdrawal form

Section 8.1 - Information updated to emphasise that trial treatment must be discontinued following a progression

Section 8.2- Information added in regard to patient transfers

Section 8.3 - Instruction that withdrawal from trial completely must be notified in writing to the MRC CTU rather than included on withdrawal form

Section 9 and Summary – Target event numbers updated to reflect the slightly revised numbers obtained by using –nstage- which is the new, recommended program for MAMS trials

Sections 11.1 and 11.2 - Form numbers removed to allow for future changes in numbering

Section 11.2 – Reference to toxicity grading website added

Section 11.2.1 - Reference to table in appendix G added

Section 12.2 - 'Suggested' removed from 'Suggested patient information sheets'

Section 13 - CTA reference added

Section 17.3 - Information added to reflect new blood collection method for DNA analysis and in regard to additional translational studies for which funding has recently been approved

19.1.5 Amendments Made To Protocol Version 4.0 (Dec 2007)

General Information Section - Randomisation and SAE reporting details sections clarified

Section 1.2 and throughout protocol - Efficacy Stages 1-111 renamed to Activity Stages 1-111 for accuracy and clarity

Section 1.2 - Follow schedule corrected

Section 4.1.2 - Inclusion criteria widened to include high risk relapsing patients, that would not have met the previous PSA based criteria

Section 4.1.3 - Note added to reference location of WHO performance status definitions

Section 4.2 - Notes added to reference locations of toxicity gradings and NYHA classifications

Section 4.3.1 - Timings of baseline scan information changed to accurately reflect most common current practice

Section 6.1.1 - Information about use of LHRH antagonists to ensure that the protocol accurately reflects current and future practice

Section 6.1.1 - Information about suggested duration of hormone therapy added to ensure that the protocol accurately reflects current practice

Section 6.2.2 - Additional information added about the timing of liver function tests prior to docetaxel administration added for clarity

Section 6.6.4 - Information on radiotherapy data collection added

Section 7.1.1 - Erroneous information about the timing of PSA measurements removed

Figure 3 - Moved to new section in protocol for clarity and extended to include current information on data collection

Figure 3b - Added to describe how extent of data collection during follow-up should change, post treatment and post progression

Figure 4 - Notes added to explain the changes in data collected at follow-up and to information that the quality of life study will be applicable to the first 700 patients randomised only

Figure 4 - Note added to include palliative radiotherapy CRF

Section 11.3 - SAE reporting information updated

Section 19 - Protocol amendments list updated

19.1.6 Amendments Made To Protocol Version 5.0 (Aug 2008)

1. General Information Section – Randomisation phone line number updated – non UK extension added

2. Section 3 – Information about QL study removed to reflect closure of QL study after first 700 patients

3. Section 4.2 – Exclusion criteria clarified to explain that only patients with severe poor cardiovascular history should be excluded

4. Section 4.3.1 – Information on co-administration of NSAIDS with celecoxib changed based on clinical advice.

5. Section 5 - Randomisation phone line number updated – non UK extension added

6. Section 6.2.1. – Information added to clarify that patients who develop an osteonecrosis of the jaw should stop zoledronic acid treatment

7. Section 6.2.3 – 'severe' text added to accurately reflect which patients should be excluded based on their cardiovascular history

8. Section 7.1.2 – Definition of disease progression extended for clarity

9. Figure 3 – Updated to include reference to newly created skeletal related event form

10. Figure 4 – Previous error in table amended to show that the 4th Zoledronic Acid form that is submitted contains information about 3 cycles rather than 2 as previously indicated

11. Table 4 – 'Other important medical condition' added to definition of serious in the SAE section, to accurately reflect SAE form and current practice

12. Section 11.1 – Information added on reporting or pregnancies

13. Section 17 - Information about QL study removed to reflect closure of QL study after first 700 patients

19.1.7 Amendments Made To Protocol Version 6.0 (Jul 2009)

1. General Information Section – Trial Pharmacist removed and changes of:

Co-Investigator

Patient Representatives

Trial Manager

Data Manager

General Information Section - Coordinating Centre – address change

General Information Section – change of Sponsor address

Section 1.1 – ratio of patients randomised to the investigational arms updated

Section 1.2 – figure 1b added to clarify trial design from Apr-2011 onwards

Section 1.2 – paragraph added to explain trial changes after the second activity analysis

Section 1.2 – wording added to clarify that QL data only collected for first 700 patients randomised

Section 1.3 – SSA Favourable Opinion removed from list of trial documentation required ahead of site accreditation

Section 2.1 – Amount of men diagnosed with prostate cancer annually updated

Section 2.4 –note added to explain completion of recruitment to celecoxib- containing arms

Section 2.5.2 - note added to explain completion of recruitment to celecoxib- containing arms

Section 3 – SSA Favourable Opinion removed

Section 4.2 – Exclusion criterion xiii greyed out

Section 4.3.1 – paragraph removed regarding potential randomisation to celecoxib-containing arms

Section 5 – Randomisation instructions expanded to exclude public holidays or dates when notice has been given by the CTU

Section 6.1.4 – formatting changed to grey font to reflect recruitment completion for arm D

Section 6.1.6 - formatting changed to grey font to reflect recruitment completion for arm F

Section 6.2.3 – recruitment note added

Section 6.6.3 – radiotherapy statement changed to reflect data from recent trials

Section 7.1.2 – removal of reference to SRE- specific CRF

Section 7.3 – Figure 3 - Addition of Bone Density Risk Factor Form and BMD sub-study assessment forms to summary of timing table

Section 7.3 – Figure 4 – Weeks added to timings of assessments post 2 years

Section 7.3- Figure 4 – note added to explain recruitment completion for arms D and F

Section 12.1 – Wording changed to reflect change to randomisation allocation ratio

Section 12.1 – Addition of statement regarding new information emerging during the trial

Section 12.2 – Reference to SSA removed

Section 16.3 – Statement added regarding actioning IDMC recommendation ahead of TSC ratification

19.1.8 Amendments Made To Protocol Version 7.0 (Jul 2011)

1. General Information Section- SAE reporting fax number corrected

2. Section 11- SAE reporting fax number corrected

19.1.9 Amendments Made To Protocol Version 7.1 (Jul 2011)

Throughout protocol – numbering has been updated in some sections new accommodate new information that has been added.

General Information Section – contact details updated

General Information Section – Funding information updated to include involvement from additional company

General Information Section – Wording on compliance and regulations updated to reflect current MRC CTU standard wording

General Information Section – Abbreviations list updated

Section 1.1 – The number of investigational agents being studied updated from three to four

Section 1.1 – Information regarding celecoxib updated to reflect that recruitment to these arms was discontinued in Apr-2011

Section 1.1 – Information about new IMP, Abiraterone inserted

Section 1.1 – Sample size and trial duration information updated to reflect changes brought about by additional trial arm

Section 1.2 – Summary information updated to reflect the discontinuation of recruitment to celecoxib arms and the addition of abiraterone

Figures 1a, b and c - Updated to reflect the discontinuation of recruitment to celecoxib arms and the addition of abiraterone

Section 1.2 – Information on trial stages updated to reflect changes brought about by additional trial arm

Section 1.2 – Information updated regarding the re-opening of the quality of life sub-study from implementation of protocol version 8.0

Section 2.1 – Wording related to hormone therapy updated for clarity

Section 2.1 - Updated to reflect the discontinuation of recruitment to celecoxib arms and the addition of abiraterone

Section 2.2 – Updated references added

Section 2.3 – Updated references added

Section 2.5 – Section added to give background information on new IMP, abiraterone

Section 2.6.1 – Updated references added

Section 2.7 – Section added to give information regarding radiotherapy which is to be given as part of standard care following recently published trial data.

Section 3 – Wording updated regarding selection of investigators to reflect current MRC CTU practice

Section 4.1 – Inclusion criteria updated with new criterion regarding radiotherapy use

Section 4.1 - Inclusion criteria updated with new criterion regarding contraceptive use

Section 4.1 – Wording of inclusion and exclusion criteria updated for clarity

Section 4.1 – Exclusion criteria updated with new criterion regarding acceptable liver function for trial entry

Section 4.1 – Exclusion criteria updated with specifics related to blood pressure levels

Section 4.1 - Exclusion criteria updated with new criterion regarding concomitant medications

Section 4.1 - Exclusion criteria updated with new criterion regarding prior treatment with abiraterone

Section 4.1 - Exclusion criteria updated with new criterion regarding prior treatment with chemotherapy

Section 4.1 - Exclusion criteria updated with new criterion regarding prior treatment with zoledronic acid

Section 4.3 – Wording updated to reflect that patients who initially fail screening can be re-screened at a later date

Section 4.3.2 – Wording updated regarding prior anti-androgen and LHRH use updated for clarity

Section 5.1 – Co-enrolment guidelines information updated to describe newly created co-enrolment CRF

Section 6.1 – Trial treatment information updated to reflect the fact that anti-androgens alone will be no longer permitted as hormone therapy

Section 6.1.1 – Updated to describe patients for whom radiotherapy should be given as standard practice

Section 6.1.1 a and b - Sections added to give information regarding radiotherapy treatment

Section 6.1.1-6.1.6 – References to further sections updated

Section 6.1.7 – Section added to describe abiraterone treatment

Section 6.2.4 - Section added to describe abiraterone treatment

Section 6.6 - Section added to give information regarding radiotherapy treatment

Section 7.1.1 – Reference to blood being taken at patient's home removed as this does not occur in practice

Section 7.1.2 – Wording updated regarding the reporting of biochemical failures for clarity

Section 7.1.2 – Wording updated regarding skeletal-related events for clarity

Section 7.1.3 – Section added to describe additional assessments required related to abiraterone treatment

Section 7.1.4 – Section added to provide information on when treatment should commence

Figure 4 – Updated for clarity regarding return of BMD sub-study forms, the addition the co-enrolment CRF and the description of the re-opening of the QoL Sub-study.

Figure 5 – Updated with reference to abiraterone and co-enrolment form

Section 7.3 - Wording on trial closure updated to reflect current MRC CTU standard wording

Section 8.1 – Additional criteria for definition of progression added for clarity

Section 8.1 – Definition of progression for abiraterone patients added.

Section 9 – Statistical information updated to describe the addition of the new trial arm

Section 11 – Safety reporting wording updated for clarity

Section 11 – SAE reporting fax number updated

Section 12 – Ethical information updated to describe the unequal randomisation allocation ratio

Section 12 – Ethical information updated to describe that the visit schedule will vary according to trial arm

Section 12.2 – Wording updated to reflect international participation in the trial

Section 13 – Wording updated to reflect international participation in the trial

Section 14 – Wording updated to reflect international participation in the trial

Section 15 - Updated to reflect the discontinuation of recruitment to celecoxib arms and the addition of abiraterone

Section 16 – Reference to trial committee charters added for information

Section 17.1 – Information added to reflect re-opening of quality of life sub-study

Section 17.2 – Timing of health economics analysis updated to previous error

Section 18 – Information on publication policy expanded for clarity

Section 19 – Information regarding amendments to protocol appendices moved to the separate appendices document

Section 20 – References extensively updated

19.1.10 Amendments Made To Protocol Version 8.0 (Sep 2011)

Throughout protocol – numbering, sections headings, tables, figures and bibliographical references have been updated in some sections to accommodate new information that has been added

Throughout protocol – Androgen Deprivation Therapy has replaced Hormone Therapy as deemed more representative of the type of hormone therapy used in the study

General Information Section – New staff members of the MRC CTU and Co-Investigators added and contact details updated

General Information Section – Abbreviations list updated

Section 1.1 – Information regarding the new research radiotherapy treatment inserted

Section 1.1 – Information regarding docetaxel updated

Section 1.2 – Wording updated to reflect the addition of the new research comparison arm

Section 1.3 – Additional criteria for the re-accreditation of participating centres (for protocol version 9.0 only)

Section 2.1.1 – Wording updated to clarify the use of anti-androgen in trial patients

Section 2.1.2 – Information added to describe the rationale for the RT comparison arm

Section 2.8 – Information added to describe research RT treatment to prostate for patients with newly diagnosed metastatic disease

Section 3.1 – Information added to describe RT Quality Assurance procedures and centre accreditation

Section 4.1.1 to 4.1.3 – Wording updated to clarify inclusion criteria for all patients groups (newly diagnosed non-metastatic, metastatic and relapsing patients)

Section 4.2 – Clarification added on cardiovascular exclusion criteria

Section 4.2 – New exclusion criterion added concerning patients with prior exposure to hormone therapy

Section 4.2 – New exclusion criterion added to reflect the addition of the new RT comparison arm

Section 4.4.1 – Clarification added regarding pre-randomisation checks

Section 4.4.2 – Clarification added regarding permissible hormone therapy duration prior to randomisation

Section 4.4.5 – Information added regarding starting research radiotherapy treatment

Section 4.4.6 – Information updated on concomitant medications

Section 5 – Clarification regarding randomisation allocation added to reflect the addition of the new RT research arm

Section 6.1.8 – Information added to describe the administration of research radiotherapy

Section 6.2.1 – Clarification added regarding the measurement of serum creatinine levels prior to the administration of zoledronic acid

Section 6.2.3 – Clarification regarding the completion of recruitment to the celecoxib containing arms

Section 6.25 – Information added regarding the administration of research radiotherapy treatment

Section 6.6 – Clarification incorporated to describe the administration of standard-of-care radiotherapy

Section 7.1.4 – Information added regarding data collection and non-administration of standard radiotherapy

Section 7.2 – Section updated to include new treatment specific CRFs and timing of CRFs

Section 8.1 – Clarification added for the criteria to stop treatment for patients randomised to arm G

Section 8.2 – Section expanded to include additional details on study patient transfer to different centres

Section 8.3 – Additional sentence inserted to reinforce the importance of compliance with follow up assessments

Section 9.1 – Additional paragraph inserted to clarify the method of randomisation and allocation distribution in the light of the introduction of the new RT arm

Section 9.4 – Wording updated to clarify the assessment of safety data

Section 9.5.4 – Wording updated concerning the end of randomisations to arm G

Section 9.6 to 9.6.4 – Section added describing sample size issues and trial stages for arm H

Section 9.8 – Clarification on intermediate stopping guidelines

Section 9.9 – Clarification on the outline analysis plan

Section 11 – Information on safety reporting updated to reflect the addition of the research RT comparison arm

Section 11 – Clarification added regarding arm A safety reporting timelines

Section 12.1 – Clarification added regarding the Principal Investigator’s responsibilities

Section 14 – Indemnity section updated to reflect current MRC policy

Section 16 – Clarification regarding TMG membership

Section 17.3 – Section on Bone Mineral Density sub-study removed

Section 19 – Information regarding amendments to protocol appendices moved to the separate appendices document

Section 20 – References updated

19.1.11 Amendments Made To Protocol Version 9.0 (Oct-2012)

Throughout protocol – numbering, sections headings, tables, figures and bibliographical references have been updated in some sections to accommodate the completion of recruitment to original research arms B, C and E.

Throughout protocol – Tenses have been changed to reflect activities that were in the future and which have now been passed.

Section 1 – Figure added and clarifications added to each figure

Section 2 – Previous reference 8 removed

Section 4 – Clarification of acceptable alternatives to bone scans

Section 6.2.5 – Correction of an error defining the PTV: the wording has been reordered

Table 4 – Dose-volume objectives corrected: order swapped

Table 5- Correction CRFs names

Section 17.3.2 – Clarification that DNA may be extracted

19.1.12 Amendments Made To Protocol Version 10.0 (Apr-2013)

Throughout protocol – numbering, sections headings, tables, figures and bibliographical references have been updated in some sections

Throughout protocol – typos have been corrected

Section 4 –Clarification of exclusion criteria V (now V and VI)

Section 6 – Timing of orchidectomy prior to randomisation extended to 12 weeks

Section 6 – Clarification of hypokalaemia, blood pressure and fluid retention management

Section 9 – Statistical considerations amended in light of the recruitment extension for the abiraterone comparison

Section 14 - Section updated to reflect the changes in the structure of the MRC CTU (now MRC CTU at UCL) and indemnity arrangements

19.1.13 Amendments Made To Protocol Version 11.0 (Sep-2013)

Throughout protocol – numbering, sections headings, tables, figures and bibliographical references have been updated in some sections

Throughout protocol – typos have been corrected

Co-investigators list updated to reflect the addition of the “enzalutamide + abiraterone comparison” lead

Section 1.2 – Enzalutamide added as trial treatment

Section 1.2 – Protocol version 12.0 added to the list of amendments

Section 2.10 – Rationale for the combination of enzalutamide and abiraterone

Section 4.2 – Eligibility criteria amended to reflect the addition of enzalutamide + abiraterone arm

Section 4.4.2 – Wording clarified

Section 6.8 – Clarification regarding end of trial treatment after starting trial therapy

Section 6.10 – Section added to describe enzalutamide and abiraterone treatment for the new research arm (Arm J)

Section 6.11.4.A – Section added to describe the management of toxicities from trial abiraterone

Section 6.11.4.B - Section added to describe the management of toxicities from trial enzalutamide

Section 9.1.4 – Section added to describe the statistical considerations concerning the introduction of Arm J

Section 9.3 – Principles and assumption for the introduction of Arm J added

Section 9.7 and sub-sections – Sample size issues and trial stages for Arm J

Section 9.9 – Details on interim monitoring and analyses for Arm J added

Section 11.2.1.D – Wording clarified regarding safety reporting requirements for control arm

Section 12.1 – Wording clarified

Section 15 – Details on funding for the “enzalutamide + abiraterone comparison” added

Section 19 - Amendments made to protocol updated

Reference list updated

19.1.14 Amendments Made To Protocol Version 12.0 (Jan-2014)

Throughout protocol – typos have been corrected

Section 4.4.2. Wording clarified

Section 4.3. Wording clarified for eligibility to M1|RT comparison

Section 6.10. Addition of use of dexamethasone post-biochemical progression for Arm J patients

Section 6.11.4.A. Correction of CTCAE version

Section 6.11.4.C. Clarification on enzalutamide dose modification to be in line with current SmPC

Section 9.6. Sample size increase for M1|RT comparison

Section 11. Correction of safety reporting timelines for Arm A patients

Section 17. Addition of saliva samples collection for DNA analysis

Table 4, 5 and 6. Clarification on Case Report Forms and Follow-up schedule

20 REFERENCES

1. James ND, Sydes MR, Mason MD, Clarke NW, Anderson J, Dearnaley DP, et al. Celecoxib plus hormone therapy versus hormone therapy alone for hormone-sensitive prostate cancer: first results from the STAMPEDE multiarm, multistage, randomised controlled trial. The lancet oncology. 2012;13(5):549-58. Epub 26-Mar-2012.
2. Cancer Research UK. CancerStats Key Facts: Prostate Cancer. Cancer Research UK. 2011.
3. Sharifi N, Gulley JL, Dahut WL, Sharifi N, Gulley JL, Dahut WL. An update on androgen deprivation therapy for prostate cancer. Endocrine-Related Cancer. 2010;17(4):R305-15.
4. Widmark A, Klepp O, Solberg A, Damber JE, Angelsen A, Fransson P, et al. Endocrine treatment, with or without radiotherapy, in locally advanced prostate cancer (SPCG-7/SFUO-3): an open randomised phase III trial. Lancet. 2009;373(9660):301-8. Epub 2008/12/19.
5. Warde PR, Mason MD, Sydes MR, Gospodarowicz MK, Swanson GP, Kirkbride P, et al. Intergroup randomized phase III study of androgen deprivation therapy (ADT) plus radiation therapy (RT) in locally advanced prostate cancer (CaP) (NCIC-CTG, SWOG, MRC-UK, INT: T94-0110; NCT00002633). J Clin Oncol. 2010;28(18s Supplement: Proceedings of ASCO 2010):Abstr CRA4504.
6. Warde P, Mason M, Ding K, Kirkbride P, Brundage M, Cowan R, et al. Survival Benefit with Combined Androgen Deprivation and Radiation Therapy in Locally Advanced Prostate Cancer – Results of a Phase III Trial. The Lancet. 2011 - in press.
7. Mason M, Sydes M, Parulekar W, Parmar M, Anderson J, Barber J, et al. Final analysis of intergroup randomized phase III study of androgen deprivation therapy (ADT) + radiation therapy (RT) in locally advanced prostate cancer (CaP) (NCIC-CTG, SWOG, MRC-UK, INT: T94-0110). National Cancer Research Institute (NCRI) Cancer Conference 2012. 2012;2012.
8. Saad F, Colombel M. Management of castration-resistant prostate cancer: bisphosphonates and emerging therapies. Expert Review of Anticancer Therapy. 2010;10(12):1991-2002.
9. Sartor O, Halstead M, Katz L, Sartor O, Halstead M, Katz L. Improving outcomes with recent advances in chemotherapy for castrate-resistant prostate cancer. Clinical Genitourinary Cancer. 2011;8(1):23-8.
10. Attard G, Richards J, de Bono JS, Attard G, Richards J, de Bono JS. New strategies in metastatic prostate cancer: targeting the androgen receptor signaling pathway. Clinical Cancer Research. 2011;17(7):1649-57.
11. Lipton A. Improving progression-free and overall survival in patients with cancer: a potential role for bisphosphonates. Expert Opinion on Pharmacotherapy. 2011;12(5):749-62.
12. Lipton A, Small E, Saad F, Gleason D, Gordon D, Smith M, et al. The new bisphosphonate, Zometa (zoledronic acid), decreases skeletal complications in both osteolytic and osteoblastic lesions: a comparison to pamidronate. [Review] [55 refs]. Cancer Investigation. 2002;20 Suppl 2:45-54.
13. Green JR, Muller K, Jaeggi KA. Preclinical pharmacology of CGP 42'446, a new, potent, heterocyclic bisphosphonate compound. Journal of Bone & Mineral Research. 1994;9(5):745-51.
14. Major P, Lortholary A, Hon J, Abdi E, Mills G, Menssen HD, et al. Zoledronic acid is superior to pamidronate in the treatment of hypercalcemia of malignancy: a pooled analysis of two randomized, controlled clinical trials. J Clin Oncol. 2001;19(2):558-67. Epub 2001/02/24.
15. Costa L, Harper P, Coleman RE, Lipton A, Costa L, Harper P, et al. Anticancer evidence for zoledronic acid across the cancer continuum. Critical Reviews in Oncology-Hematology. 2011;77 Suppl 1:S31-7.
16. Rosen LS, Gordon D, Kaminski M, Howell A, Belch A, Mackey J, et al. Long-term efficacy and safety of zoledronic acid compared with pamidronate disodium in the treatment of skeletal complications in patients with advanced multiple myeloma or breast carcinoma: a randomized, double-blind, multicenter, comparative trial. Cancer. 2003;98(8):1735-44. Epub 2003/10/10.

17. Rosen LS, Gordon DH, Dugan W, Jr., Major P, Eisenberg PD, Provencher L, et al. Zoledronic acid is superior to pamidronate for the treatment of bone metastases in breast carcinoma patients with at least one osteolytic lesion. Cancer. 2004;100(1):36-43. Epub 2003/12/24.
18. Saad F, Gleason D, Murray R, Tchekmedyian S, Venner P, Lacombe L, et al. A Randomized, Placebo-Controlled Trial of Zoledronic Acid in Patients With Hormone-Refractory Metastatic Prostate Carcinoma Journal of the National Cancer Institute. 2002;94(19).
19. Dearnaley DP, Sydes MR, Mason MD, Stott M, Powell CS, Robinson AC, et al. A double-blind, placebo-controlled, randomized trial of oral sodium clodronate for metastatic prostate cancer (MRC PR05 Trial). J NatlCancer Inst. 2003;95(17):1300-11.
20. Petrylak DP, Tangen CM, Hussain MH, Lara PN, Jr., Jones JA, Taplin ME, et al. Docetaxel and estramustine compared with mitoxantrone and prednisone for advanced refractory prostate cancer. N Engl J Med. 2004;351(15):1513-20. Epub 2004/10/08.
21. Tannock IF, de Wit R, Berry WR, Horti J, Pluzanska A, Chi KN, et al. Docetaxel plus prednisone or mitoxantrone plus prednisone for advanced prostate cancer. N Engl J Med. 2004;351(15):1502-12. Epub 2004/10/08.
22. Hawkey CJ. COX-2 inhibitors.[comment]. Lancet. 1999;353(9149):307-14.
23. Taketo MM. Cyclooxygenase-2 inhibitors in tumorigenesis (Part II). Journal of the National Cancer Institute. 1998;90(21):1609-20.
24. Taketo MM. Cyclooxygenase-2 inhibitors in tumorigenesis (Part I). Journal of the National Cancer Institute. 1998;90(20):1529-36.
25. Nelson JE, Harris RE. Inverse association of prostate cancer and non-steroidal anti-inflammatory drugs (NSAIDs): results of a case-control study. Oncology Reports. 2000;7(1):169-70.
26. Eberhart CE, Coffey RJ, Radhika A, Giardiello FM, Ferrenbach S, DuBois RN. Up-regulation of cyclooxygenase 2 gene expression in human colorectal adenomas and adenocarcinomas. Gastroenterology. 1994;107(4):1183-8.
27. Roberts RO, Jacobson DJ, Girman CJ, Rhodes T, Lieber MM, Jacobsen SJ. A population-based study of daily nonsteroidal anti-inflammatory drug use and prostate cancer. [see comments.]. Mayo Clinic Proceedings. 2002;77(3):219-25.
28. Basler JW, Piazza GA, Basler JW, Piazza GA. Nonsteroidal anti-inflammatory drugs and cyclooxygenase-2 selective inhibitors for prostate cancer chemoprevention. Journal of Urology. 2004;171(2 Pt 2):S59-62; discussion S-3.
29. Masferrer JL, Leahy KM, Koki AT, Zweifel BS, Settle SL, Woerner BM, et al. Antiangiogenic and antitumor activities of cyclooxygenase-2 inhibitors. Cancer Research. 2000;60(5):1306-11.
30. Steinbach G, Lynch PM, Phillips RK, Wallace MH, Hawk E, Gordon GB, et al. The effect of celecoxib, a cyclooxygenase-2 inhibitor, in familial adenomatous polyposis. New England Journal of Medicine. 2000;342(26):1946-52.
31. Solomon SD, McMurray JJ, Pfeffer MA, Wittes J, Fowler R, Finn P, et al. Cardiovascular risk associated with celecoxib in a clinical trial for colorectal adenoma prevention. New England Journal of Medicine. 2005;352(11):1071-80.
32. Danila DC, Morris MJ, de Bono JS, Ryan CJ, Denmeade SR, Smith MR, et al. Phase II multicenter study of abiraterone acetate plus prednisone therapy in patients with docetaxel-treated castration-resistant prostate cancer. J Clin Oncol. 2010;28(9):1496-501. Epub 2010/02/18.
33. Reid AH, Attard G, Danila DC, Oommen NB, Olmos D, Fong PC, et al. Significant and sustained antitumor activity in post-docetaxel, castration-resistant prostate cancer with the CYP17 inhibitor abiraterone acetate. J Clin Oncol. 2010;28(9):1489-95. Epub 2010/02/18.
34. Ryan CJ, Smith MR, Fong L, Rosenberg JE, Kantoff P, Raynaud F, et al. Phase I clinical trial of the CYP17 inhibitor abiraterone acetate demonstrating clinical activity in patients with castration-resistant prostate cancer who received prior ketoconazole therapy. Journal of Clinical Oncology. 2010;28(9):1481-8.

35. de Bono JS, Logothetis CJ, Molina A, Fizazi K, North S, Chu L, et al. Abiraterone and increased survival in metastatic prostate cancer. New England Journal of Medicine. 2011;364(21):1995-2005.
36. Ryan CJ, Smith MR, de Bono JS, Molina A, Logothetis CJ, de Souza P, et al. Abiraterone in metastatic prostate cancer without previous chemotherapy. N Engl J Med. 2013;368(2):138-48. Epub 2012/12/12.
37. Attard G, Reid AHM, de Bono JS. Abiraterone acetate is well tolerated without concomitant use of corticosteroids. Journal of Clinical Oncology. 2010;28(29):e560-1; author reply e2.
38. Flanigan RC, Salmon SE, Blumenstein BA, Bearman SI, Roy V, McGrath PC, et al. Nephrectomy followed by interferon alfa-2b compared with interferon alfa-2b alone for metastatic renal-cell cancer. N Engl J Med. 2001;345(23):1655-9. Epub 2002/01/05.
39. Mickisch GH, Garin A, van Poppel H, de Prijck L, Sylvester R. Radical nephrectomy plus interferon-alfa-based immunotherapy compared with interferon alfa alone in metastatic renal-cell carcinoma: a randomised trial. Lancet. 2001;358(9286):966-70. Epub 2001/10/05.
40. Warde P, Mason M, Ding K, Kirkbride P, Brundage M, Cowan R, et al. Combined androgen deprivation therapy and radiation therapy for locally advanced prostate cancer: a randomised, phase 3 trial. The Lancet. 2011;378:2104-11. Epub 03-Nov-2011.
41. Neville-Webbe HL, Holen I, Coleman RE. The anti-tumour activity of bisphosphonates. Cancer Treatment Reviews. 2002;28(6):305-19.
42. Witters LM, Crispino J, Fraterrigo T, Green J, Lipton A. Effect of the combination of docetaxel, zoledronic acid, and a COX-2 inhibitor on the growth of human breast cancer cell lines. American journal of clinical oncology. 2003;26(4):S92-7. Epub 2003/08/07.
43. Attard G, Cooper CS, de Bono JS. Steroid hormone receptors in prostate cancer: a hard habit to break? Cancer cell. 2009;16(6):458-62. Epub 2009/12/08.
44. Chen G, Wang X, Zhang S, Lu Y, Sun Y, Zhang J, et al. Androgen receptor mutants detected in recurrent prostate cancer exhibit diverse functional characteristics. The Prostate. 2005;63(4):395-406. Epub 2004/12/24.
45. Taplin ME, Bubley GJ, Ko YJ, Small EJ, Upton M, Rajeshkumar B, et al. Selection for androgen receptor mutations in prostate cancers treated with androgen antagonist. Cancer Res. 1999;59(11):2511-5. Epub 1999/06/11.
46. Veldscholte J, Ris-Stalpers C, Kuiper GG, Jenster G, Berrevoets C, Claassen E, et al. A mutation in the ligand binding domain of the androgen receptor of human LNCaP cells affects steroid binding characteristics and response to anti-androgens. Biochemical and biophysical research communications. 1990;173(2):534-40. Epub 1990/12/14.
47. Zhao XY, Malloy PJ, Krishnan AV, Swami S, Navone NM, Peehl DM, et al. Glucocorticoids can promote androgen-independent growth of prostate cancer cells through a mutated androgen receptor. Nature medicine. 2000;6(6):703-6. Epub 2000/06/03.
48. Attard G, Reid AH, Auchus RJ, Hughes BA, Cassidy AM, Thompson E, et al. Clinical and biochemical consequences of CYP17A1 inhibition with abiraterone given with and without exogenous glucocorticoids in castrate men with advanced prostate cancer. The Journal of clinical endocrinology and metabolism. 2012;97(2):507-16. Epub 2011/12/16.
49. Richards J, Lim AC, Hay CW, Taylor AE, Wingate A, Nowakowska K, et al. Interactions of abiraterone, eplerenone, and prednisolone with wild-type and mutant androgen receptor: a rationale for increasing abiraterone exposure or combining with MDV3100. Cancer Res. 2012;72(9):2176-82. Epub 2012/03/14.
50. Scher HI, Fizazi K, Saad F, Taplin ME, Sternberg CN, Miller K, et al. Increased survival with enzalutamide in prostate cancer after chemotherapy. N Engl J Med. 2012;367(13):1187-97. Epub 2012/08/17.
51. Tombal B, Borre M, Rathenborg P, Werbrouck P, Heidenreich A, Iversen P, et al. Enzalutamide monotherapy: Phase II study results in patients with hormone-naive prostate cancer. Journal of Clinical Oncology. 2013;31(Supplement 6):Abstract 18.

52. Efstathiou E, Titus MA, Tsavachidou A, Hoang A, Karlou M, Wen S, et al. MDV3100 effects on androgen receptor (AR) signaling and bone marrow testosterone concentration modulation: A preliminary report. . 2011 ASCO Annual Meeting, 2011 J Clin Oncol 2011.
53. Locke JA, Guns ES, Lubik AA, Adomat HH, Hendy SC, Wood CA, et al. Androgen levels increase by intratumoral de novo steroidogenesis during progression of castration-resistant prostate cancer. Cancer Res. 2008;68(15):6407-15. Epub 2008/08/05.
54. Tran C, Ouk S, Clegg NJ, Chen Y, Watson PA, Arora V, et al. Development of a second-generation antiandrogen for treatment of advanced prostate cancer. Science. 2009;324(5928):787-90. Epub 2009/04/11.
55. Scher HI, Beer TM, Higano CS, Anand A, Taplin ME, Efstathiou E, et al. Antitumour activity of MDV3100 in castration-resistant prostate cancer: a phase 1-2 study. Lancet. 2010;375(9724):1437-46. Epub 2010/04/20.
56. Efstathiou E, Titus M, Wen A, Miguel AS, Hoang A, De A, et al. The effects of enzalutamide (ENZA) in combination with abiraterone acetate (AA) in patients with bone metastatic castration resistant prostate cancer (mCRPC). Eur J Cancer. 2013;49(ECCO 2013 Supplement):Abstract 2854.
57. Kouloulias V, Giraud J-Y, Davis BJ, Dusserre A, Zurlo A, Bolla M. Quality assurance of the 22961 EORTC trial. A phase III study of the optimal combination of hormonal adjuvant treatment by LHRH analogue and radiation therapy for the management of locally advanced prostate cancer: the dummy run. 2004(0167-8140 (Print)).
58. Royston P, Parmar MKB, Qian W. Novel designs for multi-arm clinical trials with survival outcomes with an application in ovarian cancer. Statistics in Medicine. 2003;22(14):2239-56.
59. Royston P, Barthel FMS, Parmar MKB, Choodari-Oskooei B, Isham V. Designs for clinical trials with time-to-event outcomes based on stopping guidelines for lack of benefit. Trials. 2011;12(1):81.
60. Royston P. nstage: MAMS trial sample size calculator. MRC Clinical Trials Unit, London2009.
61. Haybittle JL. Repeated assessment of results in clinical trials of cancer treatment. The British journal of radiology. 1971;44(526):793-7. Epub 1971/10/01.
62. Peto R, Pike MC, Armitage P, Breslow NE, Cox DR, Howard SV, et al. Design and analysis of randomized clinical trials requiring prolonged observation of each patient. I. Introduction and design. British journal of cancer. 1976;34(6):585-612. Epub 1976/12/01.
63. Dolan P, Gudex C, Kind P, Williams A. Discussion Paper 138: A social tarif for EuroQol: results from a UK General Population Survey. . Centre for Health Economics. University of York, 1995.

STAMPEDE

Systemic Therapy in Advancing or Metastatic Prostate Cancer: Evaluation of Drug Efficacy

A multi-arm multi-stage randomised controlled trial

**Version:**	**14.0**
**Date:**	**29-Oct-2015**

MRC CTU ID:	PR08
ISRCTN #:	ISRCTN78818544
NCT #:	NCT00268476
EUDRACT #:	2004-000193-31
CTA #:	00316/0026/001-0001
MREC #:	04/MRE07/35

Authorised by:

Name:	PROFESSOR NICHOLAS D JAMES
Role:	CHIEF INVESTIGATOR
Signature:	

Name:	MATTHEW SYDES
Role:	TRIAL STATISTICIAN

GENERAL INFORMATION

This document was constructed using the MRC CTU Protocol Template Version 4.0. It describes the STAMPEDE trial, coordinated by the Medical Research Council (MRC) Clinical Trials Unit (CTU) at University College London (UCL), and provides information about procedures for entering patients into it. The protocol should not be used as an aide-memoire or guide for the treatment of other patients. Every care has been taken in drafting this protocol, but corrections or amendments may be necessary. These will be circulated to the registered investigators in the trial, but sites entering patients for the first time are advised to contact the Cancer And Other Non-Infectious Diseases Group, MRC CTU at UCL, London, UK, to confirm they have the most up-to-date version.

COMPLIANCE

The trial will be conducted in compliance with the approved protocol, the Declaration of Helsinki 1996, the principles of Good Clinical Practice (GCP), Commission Directive 2005/28/EC with the implementation in national legislation in the UK by Statutory Instrument 2004/1031 and subsequent amendments, the UK Data Protection Act (DPA number: Z5886415), and the National Health Service (NHS) Research Governance Framework for Health and Social Care (RGF). International sites will comply with the principles of GCP as laid down by the ICH topic E6 (Note for Guidance on GCP), Commission Directive 2005/28/EC (the European Directive 2001/20/EC [where applicable]) and applicable national regulations.

SPONSOR

Medical Research Council, 2nd Floor, David Phillips Building, Polaris House, North Star Avenue, Swindon, SN2 1FL, UK

FUNDING

Clinical Trials Advisory Awards Committee (on behalf of Cancer Research UK, Medical Research Council, and other charities) together with educational grants from Novartis, Sanofi-Aventis, Pfizer, Janssen Pharma NV, Astellas.

AUTHORISATIONS AND APPROVALS

The following persons are authorised to sign the final protocol and protocol amendments for the sponsor: Professor Nicholas James (Chief Investigator) and Matthew Sydes (Trial Statistician).

TRIAL REGISTRATION

This trial has been registered with the ClinicalTrials.gov Clinical Trials Register, where it is identified as NCT00268476.

RANDOMISATIONS
To randomise, call MRC CTU at UCL, Monday to Friday 0900-1700
excluding public holidays or dates when notice has been given by the Unit.
Tel: +44 (0) 20 7670 4777

SAE REPORTING
Fax to 020 7670 4818 within 24 hours of becoming aware of the event

TRIAL ADMINISTRATION

COORDINATING SITE

MRC Clinical Trials Unit at UCL	Switchboard:	020 7670 4700
Aviation House	Fax:	020 7670 4818
125 Kingsway London	Email:	mrcctu.stampede@ucl.ac.uk
WC2B 6NH		
UK		

MRC CTU STAFF

Trial Manager:	Francesca Schiavone	Tel:	0207 670 4632
Trial Manager:	Orla Prendiville	Tel:	0207 670 4840
Data Manager:	Carly Au	Tel:	0207 670 4947
Data Manager:	Zohrah Khan	Tel:	0207 670 4794
Data Manager:	Peter Vaughan	Tel:	0207 670 4655
Data Manager:	Nargis Begum	Tel:	0207 670 4693
Data Manager:	Jenna Grabey	Tel:	0207 670 4626
Clinical Project Manager:	Claire Amos	Tel:	0207 670 4771
Statistician:	Melissa Spears	Tel:	0207 670 4764
Senior Trial Statistician:	Matt Sydes	Tel:	0207 670 4798
Director of MRC CTU:	Max Parmar	Tel:	0207 670 4729
Trial Surgeon:	Alastair Ritchie	Tel:	Please contact STAMPEDE team
Clinical Research Fellow	Clare Gilson	Tel:	Please contact STAMPEDE team

Chief Investigator

Prof Nicholas James
Warwick Medical School
University of Warwick
Coventry, CV4 7AL
UK

Tel: 024 7657 4365 (Secretary)
Email: n.d.james@warwick.ac.uk

Co-investigators

Prof Noel Clarke<br>Urologist	Manchester, UK	Vice-Chair
Prof Malcolm Mason<br>Oncologist	Cardiff, UK	Vice-Chair
Dr Gerhardt Attard<br>Oncologist	London, UK	Lead:"Enzalutamide+Abiraterone comparison"
Mr William Cross<br>Urologist	Leeds, UK	
Prof David Dearnaley<br>Oncologist	London, UK	
Dr Rob Jones<br>Oncologist	Glasgow, UK	
Dr Chris Parker<br>Oncologist	London, UK	Lead: "M1|RT comparison"
Dr Martin Russell<br>Oncologist	Glasgow, UK	
Prof George Thalmann<br>Urologist	Bern, Switzerland	

Patient Representatives

Robin Millman
Patient

David Matheson
Patient

Health Economics

Prof Mark Sculpher<br>Health Economics Advisor	York, UK

Biological Research Group

Malcolm Mason	Chair

For full details of all trial committees, please see **Appendix M**.

CONTENTS

GENERAL INFORMATION I

CONTENTS 4

ABBREVIATIONS 9

1 SUMMARY 12
1.1 LAY SUMMARY 12
1.2 ABSTRACT AND SUMMARY OF TRIAL DESIGN 13
1.3 TRIAL DOCUMENTATION 16

2 BACKGROUND 17
2.1 INTRODUCTION AND SETTING 17
2.1.1 Long-term Androgen Deprivation Therapy 17
2.1.2 Role of Radiotherapy for Men With M0 Disease 17
2.2 RATIONALE 18
2.3 DESIGN 18
2.4 RESEARCH TREATMENT: BISPHOSPHONATES 19
2.5 RESEARCH TREATMENT: CHEMOTHERAPY 19
2.6 RESEARCH TREATMENT: CYCLOOXYGENASE-2 INHIBITORS 19
2.7 RESEARCH TREATMENT: STEROID SYNTHESIS INHIBITORS 19
2.8 RESEARCH TREATMENT: RADIOTHERAPY TO THE PROSTATE FOR PATIENTS WITH NEWLY-DIAGNOSED METASTATIC DISEASE 20
2.9 RESEARCH TREATMENT: COMBINATIONS OF ORIGINAL RESEARCH ARMS 21
2.9.1 Bisphosphonate And Chemotherapy 21
2.9.2 Bisphosphonate And Cyclooxygenase-2 Inhibitors 21
2.10 COMBINATION OF STEROID SYNTHESIS INHIBITORS AND ANDROGEN RECEPTOR SIGNALLING INHIBITOR 22
2.10.1 Supplementing Abiraterone And Prednisolone With Enzalutamide 22
2.10.2 Supplementing Enzalutamide With Abiraterone And Prednisolone 22
2.10.3 Summary Of Rationale For This Combination 23

3 SELECTION OF INSTITUTIONS AND INVESTIGATORS 24
3.1 RADIOTHERAPY ACCREDITATION 24

4 SELECTION OF PATIENTS 25
4.1 PATIENT INCLUSION CRITERIA 25
4.1.1 High-Risk Newly-Diagnosed Non-Metastatic Node-Negative Disease 25
4.1.2 Newly-Diagnosed Metastatic Or Node-Positive Disease 25
4.1.3 Previously Treated With Radical Surgery And/Or Radiotherapy, Now Relapsing 25
4.1.4 For All Patients 25
4.2 PATIENT EXCLUSION CRITERIA 26
4.3 SELECTION CRITERIA FOR COMPARISON OF RESEARCH RT FOR METASTATIC DISEASE (M1|RT) 27
4.4 SCREENING PROCEDURES 27
4.4.1 Investigations Prior To Randomisation 27
4.4.2 Androgen Deprivation Therapy Prior To Randomisation 28
4.4.3 Choice About Standard-Of-Care Docetaxel 28

4.4.4 Hypercalcaemia At Randomisation 28
4.4.5 NSAIDs And Cox-2 Inhibitors At Randomisation 28
4.4.6 Starting Trial Treatment 28
4.4.7 Concomitant Medications 29
4.5 ADDITIONAL DETAILS FOR PATIENTS JOINING SUB-STUDIES 29

5 RANDOMISATION AND ENROLMENT 30
5.1 CO-ENROLMENT GUIDELINES 30

6 TREATMENT OF PATIENTS 31
6.1 TRIAL TREATMENT 31
6.2 ARM A: ADT ALONE OR ADT + STANDARD-OF-CARE (M0) RT OR ADT +/- DOCETAXEL +/- (M0) RT ... 31
6.2.1 Hormone Therapy 31
6.2.2 Standard-of-care (M0) RT 31
6.3 ARM B: ADT + ZOLEDRONIC ACID 32
6.4 ARM C: ADT + DOCETAXEL 33
6.5 ARM D: ADT + CELECOXIB 33
6.6 ARM E: ADT + DOCETAXEL + ZOLEDRONIC ACID 33
6.7 ARM F: ADT + ZOLEDRONIC ACID + CELECOXIB 33
6.8 ARM G: ADT + ABIRATERONE 33
6.9 ARM H: ADT + PROSTATE RADIOTHERAPY IN M1 PATIENTS 34
6.9.1 Research (M1) Prostate Radiotherapy Treatment Administration 34
6.10 ARM J: ADT + ABIRATERONE + PREDNISOLONE + ENZALUTAMIDE ADMINISTRATION 36
6.11 ADMINISTRATION, DOSE MODIFICATIONS AND MANAGEMENT OF TOXICITIES 37
6.11.1 Zoledronic Acid 37
6.11.2 Docetaxel 37
6.11.3 Celecoxib 37
6.11.4 Abiraterone or Enzalutamide + Abiraterone 37
6.12 CONCOMITANT MEDICATIONS AND DRUG INTERACTION 43
6.12.1 Abiraterone: Interaction with other medicinal products and other forms of interaction 43
6.12.2 Enzalutamide: Interaction with other medicinal products and other forms of interaction 45
6.13 TRIAL PRODUCTS 47
6.14 MEASURES OF COMPLIANCE/ADHERENCE 47
6.15 TREATMENT DATA COLLECTION 47
6.16 ADMINISTRATION OF STANDARD RADIOTHERAPY TO NON-METASTATIC PATIENTS 47
6.16.1 Treatment Details 47
6.17 NON-TRIAL TREATMENT 48
6.17.1 Medications Permitted 48
6.17.2 Data On Concomitant Medication 48

7 ASSESSMENTS AND PROCEDURES 49
7.1 SCHEDULE FOR ASSESSMENTS 49
7.1.1 PSA Measurements 49
7.1.2 Assessment Of Treatment Failure (Definition Of Progression) 49
7.1.3 Additional Safety Assessment 49
7.1.4 Data collection for standard docetaxel 50
7.1.5. Data Collection and Non-Administration Of Standard Radiotherapy 50
7.1.6. Data Collection Palliative Radiotherapy 50
7.1.7 Data Collection Research (M1) Radiotherapy 50
7.1.8. Follow-Up Schedules 50

7.2 FOLLOW-UP **50**
7.3 TRIAL CLOSURE **51**

8 STOPPING OF TREATMENT OR FOLLOW -UP **55**
8.1 STOPPING RESEARCH INTERVENTIONS **55**
8.2 PATIENT TRANSFERS **55**
8.3 EARLY CESSATION OF TRIAL PARTICIPATION **56**

9 STATISTICAL CONSIDERATIONS **57**
9.1 METHOD OF RANDOMISATION **57**
9.1.1 Version 7 57
9.1.2 Version 8 57
9.1.3 Version 9 57
9.1.4 Version 10 and 11 57
9.1.5 Version 12 58
9.2 OUTCOME MEASURES **58**
9.3 SAMPLE SIZE: PRINCIPLES AND ASSUMPTIONS **59**
9.4 SAMPLE SIZE ISSUES AND TRIAL STAGES: ORIGINAL RESEARCH ARMS (B-F) **60**
9.4.1 Pilot Phase: Original Research Arms (B-F) 60
9.4.2 Activity Stages I-III: Original Research Arms (B-F) 60
9.4.3 Efficacy Stage IV: Original Research Arms (B-F) 61
9.4.4 Sample Size For Original Research Arms (B-F) 61
9.5 SAMPLE SIZE ISSUES AND TRIAL STAGES: ADDITIONAL RESEARCH ARM G **61**
9.5.1 Pilot Phase: Additional Research Arm G 61
9.5.2 Activity Stages I-III: Additional Research Arm G 61
9.5.3 Efficacy Stage IV: Additional Research Arm G 62
9.5.4 Sample Size For Additional Research Arm G 62
9.6 SAMPLE SIZE ISSUES AND TRIAL STAGES: ADDITIONAL RESEARCH ARM H **63**
9.6.1 Pilot Phase: Additional Research Arm H 63
9.6.2 Activity Stages I-III: Additional Research Arm H 63
9.6.3 Efficacy Stage IV: Additional Research Arm H 63
9.6.4 Sample Size For Additional Research Arm H 63
9.7 SAMPLE SIZE ISSUES AND TRIAL STAGES: ADDITIONAL RESEARCH ARM J **65**
9.7.1 Pilot Phase: Additional Research Arm J 65
9.7.2 Activity Stages I-II: Additional Research Arm J 65
9.7.3 Efficacy Stage III: Additional Research Arm J 65
9.7.4 Sample Size For Additional Research Arm J 65
9.7.5 Further Sample Size Issues For Additional Research Arm J 66
9.8 FURTHER NOTES ON TRIAL DESIGN **67**
9.8.1 Overall Sample Size 67
9.8.2 Factorial Design 67
9.9 INTERIM MONITORING AND ANALYSES **68**
9.10 OUTLINE ANALYSIS PLAN **68**
9.10.1 Pilot / Safety Phases 68
9.10.2 Activity And Efficacy Stages 69

10 MONITORING AND QUALITY ASSURANCE **70**
10.1 MONITORING AT MRC CTU **70**
10.2 DIRECT ACCESS TO DATA **70**
10.3 VISITS TO INVESTIGATOR SITES **70**

10.4 CONFIDENTIALITY **70**

11 SAFETY REPORTING **71**
11.1 DEFINITIONS **71**
11.1.1 Trial-Specific Exemptions 72
11.2 INSTITUTION/INVESTIGATOR RESPONSIBILITIES **72**
11.2.1 Investigator Assessment 72
11.2.2 Notification Procedure 73
11.3 MRC CTU RESPONSIBILITIES **74**

12 ETHICAL CONSIDERATIONS AND APPROVAL **75**
12.1 ETHICAL CONSIDERATIONS **75**
12.2 ETHICAL APPROVAL **75**

13 REGULATORY APPROVAL **77**

14 INDEMNITY **78**

15 FINANCE **79**

16 TRIAL COMMITTEES **80**
16.1 TRIAL MANAGEMENT GROUP (TMG) **80**
16.2 TRIAL STEERING COMMITTEE (TSC) **80**
16.3 INDEPENDENT DATA MONITORING COMMITTEE (IDMC) **80**

17 ANCILLARY STUDIES **82**
17.1 QUALITY OF LIFE **82**
17.2 HEALTH ECONOMICS **82**
17.3 TRANSLATIONAL SUB-STUDIES **82**
17.3.1 DNA Analysis 82
17.3.2 Tissue Microarray 83

18 PUBLICATION **84**

19 PROTOCOL AMENDMENTS **85**
19.1 PROTOCOL **85**
19.1.1 Amendments Made To Sections In Protocol Version 1.0 (May 2004) 85
19.1.2 Amendments Made To Sections In Protocol Version 1.1 (May 2005) 85
19.1.3 Amendments Made To Sections In Protocol Version 2.0 (Jun 2005) 85
19.1.4 Amendments Made To Section In Protocol Version 3.0 (Jul 2006) 86
19.1.5 Amendments Made To Protocol Version 4.0 (Dec 2007) 87
19.1.6 Amendments Made To Protocol Version 5.0 (Aug 2008) 87
19.1.7 Amendments Made To Protocol Version 6.0 (Jul 2009) 88
19.1.8 Amendments Made To Protocol Version 7.0 (Jul 2011) 89
19.1.9 Amendments Made To Protocol Version 7.1 (Jul 2011) 89
19.1.10 Amendments Made To Protocol Version 8.0 (Sep 2011) 90
19.1.11 Amendments Made To Protocol Version 9.0 (Oct-2012) 91
19.1.12 Amendments Made To Protocol Version 10.0 (Apr-2013) 92
19.1.13 Amendments Made To Protocol Version 11.0 (Sep-2013) 92

19.1.14 Amendments Made To Protocol Version 12.0 (Jan-2014) 93
19.1.15 Amendments Made To Protocol Version 13.0 (FEB-2014) 93

20 REFERENCES 94

ABBREVIATIONS

Abbreviation	Expansion
ACE	Angiotensin-Converting Enzyme
ACTH	Adrenocorticotropic hormone
ADT	Androgen deprivation therapy
AR	Androgen receptor
AS	Activity Stage
bid	Twice a day (bis in die)
BP	Blood pressure
BRG	Biological Research Group
BSA	Body surface area
CERES	Consumers for Ethics in Research
CF	Consent Form
CI	Chief Investigator
CI	Confidence interval
COSTART	Coding Symbols for a Thesaurus of Adverse Reaction Terms
Cox 2	Cyclooxygenase 2
CRF	Case Report Form
CRUK	Cancer Research UK
CRPC	Castrate Refractory Prostate Cancer
CT	Computerised tomography
CTA	Clinical Trials Authorisation
CTAAC	Clinical Trials Advisory and Awards Committee
CTC	Common Toxicity Criteria
CTU	Clinical Trials Unit
CTV	Clinical Tumour Volume
CXR	Chest X-ray
DDX	Doctors and Dentists Exemption
DHT	Dihydrotestosterone
DNA	Deoxyribonucleic Acid
DPA	Data Protection Act
ERC	Endpoint Review Committee
ES	Efficacy Stage

Abbreviation	Expansion
ICH	International Conference on Harmonization
ECG	Electro cardiogram
FBC	Full Blood Count
FFS	Failure-Free Survival
GCP	Good Clinical Practice
GP	General Practitioner
GRO	General Register Office
HE	Health Economics
HES	Hospital Episode Statistics
hr	Hour
HR	Hazard Ratio
HRPC	Hormone Refractory Prostate Cancer
HT	Hormone Therapy
IDMC	Independent Data Monitoring Committee
IM	Intramuscular
IMRT	Intensity Modulated Radiation Therapy
ISRCTN	International Standard Randomised Controlled Trial Number
IU	International Units
IV	Intravenous
LD	Longest diameter
LFTs	Liver Function Tests
LHRH	Luteinising Hormone Releasing Hormone
LREC	Local Research Ethics Committee
m	Month
MHRA	Medicine and Healthcare Products Regulatory Agency
min	Minutes
MRC	Medical Research Council
MREC	Multi-Centre Research Ethics Committee
MRI	Magnetic resonance imaging
M0	Non-metastatic
M1	Metastatic
NCI	National Cancer Institute (USA)
NCRN	National Cancer Research Network

Abbreviation	Expansion
NHS	National Health Service
NSAID	Non-Steroidal Anti-inflammatory Drugs
ONS	Office for National Statistics
OS	Overall Survival
PI	Principal Investigator
PIS	Patient Information Sheet
po	per orum (orally)
PSA	Prostate Specific Antigen
pts	Patients
PTV	Planned Tumour Volume
QALY	Quality-adjusted Life Years
qds	quater die sumendus (4 times each day)
QL	Quality of Life
R&D	Research and Development
RECIST	Response Evaluation Criteria In Solid Tumours
SAE	Serious Adverse Event
SAR	Serious Adverse Reaction
sc	Sub-cutaneous (under skin)
SNP	Single Nucleotide Polymorphism
SOC	Standard-of-Care
SSA	Site Specific Assessment
STAMPEDE	Systemic Therapy in Advancing and Metastatic Prostate Cancer: Evaluation of Drug Efficacy
SUSAR	Suspected Unexpected Serious Adverse Reactions
SWOG	South West Oncology Group
TMG	Trial Management Group
TMT	Trial Management Team
TURP	Trans-Urethral Resection of Prostate
TSC	Trial Steering Committee
UCL	University College London
ULN	Upper Limit of Normal
U+E	Urea and Electrolytes
WHO	World Health Organisation

1 SUMMARY

1.1 LAY SUMMARY

Prostate cancers depend upon the male hormone testosterone for their growth. Lowering testosterone levels (either by removing all or part of both testes, or by giving anti-hormone treatment) slows the growth of prostate cancers. This type of treatment is called hormone treatment or androgen deprivation therapy (ADT) and is often used when prostate cancers have spread outside the prostate gland. Although hormone treatment is usually successful at stopping the cancer growing for a period of time, the cancer will begin to grow again in most men.

There are increasing numbers of treatments available for advanced prostate cancer. These treatments are usually used in prostate cancer when hormone treatment is no longer effective and the cancer has started to grow again. The aim of this trial, which is called STAMPEDE, is to assess some of these treatments, given earlier in the course of the disease in combination with the current standard-of-care.

The treatments that have been, or are being, assessed during the trial are:

1. Zoledronic acid: Prostate cancer cells can spread to bones and weaken them. Zoledronic acid is a drug that reduces bone destruction and hardens bones. This may make them more resistant to attack by cancer cells. Recruitment to this treatment has been completed and the results show that the addition of zoledronic acid does not prolong survival.

2. Docetaxel: A drug that stops cells replicating that is currently being used to treat a range of cancers including lung, breast and ovarian cancer as well as prostate cancer. Docetaxel prolongs survival in men with relapsed metastatic prostate cancer. Recruitment to this treatment has been completed and the results show that the addition of docetaxel to hormone treatment does improve survival in men with metastatic disease and delays the time to progression for men with locally advanced and metastatic disease. Docetaxel may now be given as part of standard treatment to all men entering STAMPEDE (from protocol version 14.0).

3. Celecoxib: An aspirin-like drug that is used to treat arthritis. It slows down the growth of cancer cells in the laboratory. We wished to see if it had the same effect on cancer cells in patients. Recruitment to new patients for the evaluation of this drug is finished as a planned intermediate analysis failed to demonstrate sufficient effect of this drug.

4. Abiraterone (included from protocol version 8.0)**:** An inhibitor of steroid hormone synthesis that blocks prostate cancer cells from generating their own male hormones. This is thought to be a major way in which prostate cancer cells resume growth following anti-hormonal therapies. Abiraterone has been shown to prolonging surival in men with advanced disease when given before and after chemotherapy.

5. Prostate radiotherapy (included from protocol version 9.0)**:** treatment with high-energy x-rays targeted to the prostate gland. This treatment is now mandatory for patients with cancer that is confined to the prostate gland as large trials have shown it improves survival times. We are not certain whether we should give radiotherapy to the prostate if the cancer has already spread and so we are investigating this in STAMPEDE.

6. Enzalutamide (included from protocol version 12.0)**:** This is a blocker of androgen receptors. These stimulate the cancer when hormone therapies have failed. Enzalutamide may be mutually complementary to abiraterone in terms of blocking mechanisms of resistance. The agent prolongs survival when given to men following failure of docetaxel chemotherapy.

STAMPEDE will look at the effect of combining one or two of the treatments described above with hormone treatment. A computer program will be used to allocate which treatment the patient receives, using a chance process. The trial will look at the effects of the combined treatments on quality of life and find out whether the new treatment combinations increase the time when the cancer is not growing and ultimately results in patients living longer. The study will also look at which treatment provides the greater value for money for the health service. More than 8,000 patients will join the trial with answers becoming available throughout the trial.

1.2 ABSTRACT AND SUMMARY OF TRIAL DESIGN

STAMPEDE is a multi-centre, randomised controlled trial for patients with locally advanced or metastatic prostate cancer who are commencing long-term Androgen Deprivation Therapy (ADT). Patients can have either newly diagnosed disease, or have been previously treated with radical radiotherapy or surgery but now have signs of progression such as a rising prostate specific antigen (PSA) (further details on eligibility see **Section 4**). The trial will assess the effects of adding different agents, both as single agents and in combinations, to the standard-of-care. The investigational agents are (i) a bisphosphonate, zoledronic acid, (ii) a cytotoxic chemotherapeutic agent, docetaxel and (iii) a cyclooxygenase (Cox-2) inhibitor, celecoxib (iv), abiraterone, a steroid synthesis inhibitor and an androgen receptor signalling inhibitor (v) enzalutamide. Recruitment to the celecoxib arms (D and F) is now closed. An additional arm containing abiraterone was added in protocol version 8.0 which has now completed recruitment. A further comparison arm involving prostate radiotherapy for patients with newly-diagnosed metastatic disease was added in protocol version 9.0, with the addition of an arm considering the combination of enzalutamide and abiraterone added in protocol version 12.0. The trial has multiple arms; the control arm of the trial receives standard therapy alone. When the trial started standard treatment was androgen deprivation therapy (ADT) only, achieved through the use of luteinising hormone releasing hormone (LHRH) analogues e.g. zoledex or LHRH antagonists, or bilateral orchidectomy according to local practice. Since primary results from the trial "original comparisons" have emerged showing a benefit in overall survival for patients receiving docetaxel in addition to ADT, the standard treatment has changed accordingly. Standard treatment may now include docetaxel chemotherapy for all men entering STAMPEDE. Radiotherapy is also mandated for men with node negative non-metastatic disease. The current trial design is shown in **Figure 1;** previous trial designs can be viewed in Protocol version 13.0.

Figure 1: Arms of the STAMPEDE trial from protocol 14.0 (amending the standard of care to permit docetaxel)

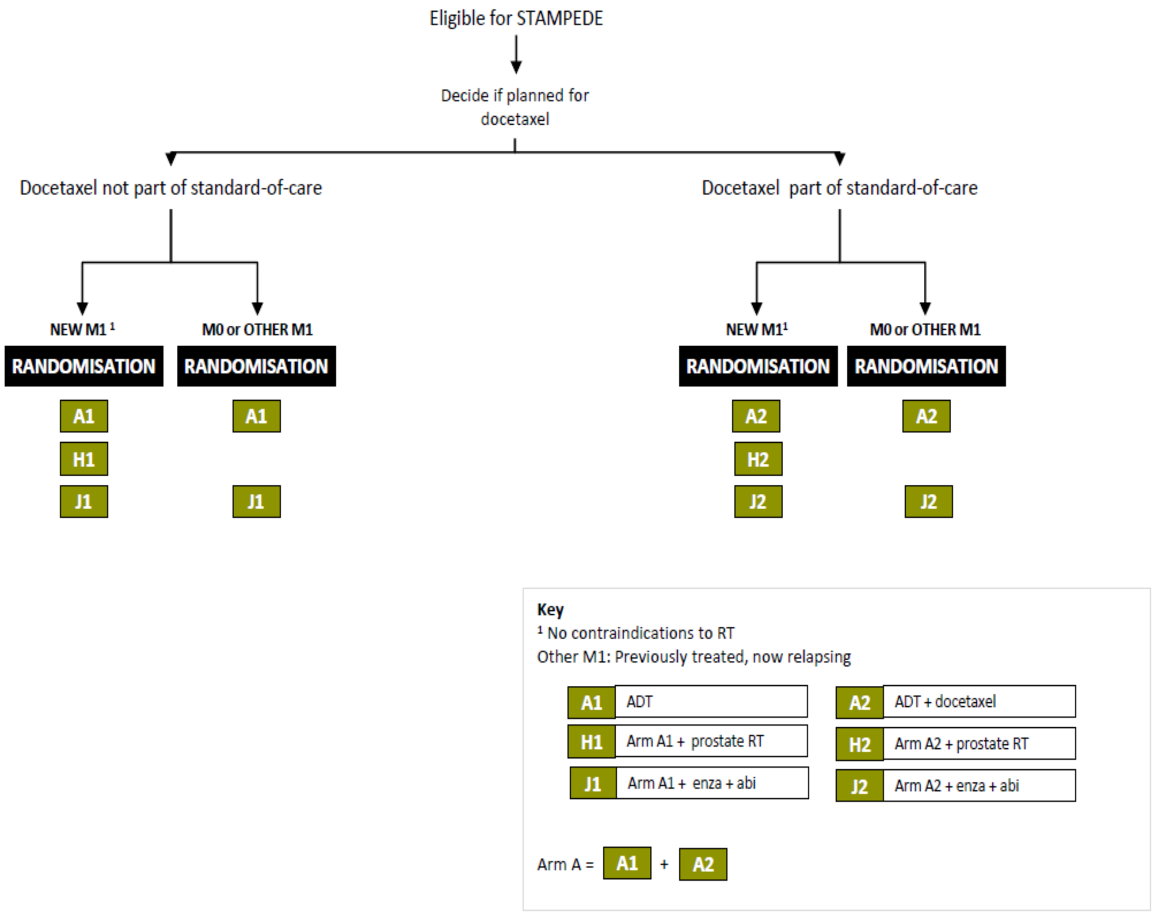

Figure 2: Arms of the STAMPEDE trial open to recruitment over time

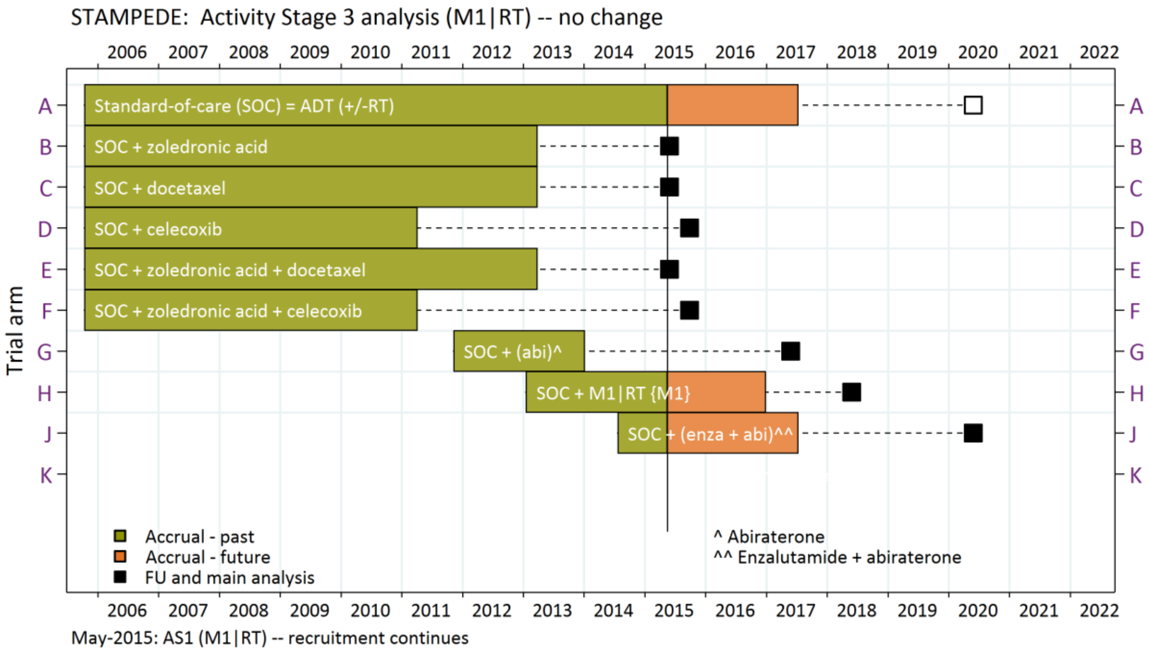

For each comparison of research arm against control, the trial will be conducted in a number of stages: a Pilot/Safety Phase, Activity Stages and a final Efficacy Stage. The primary outcome measure of the Pilot/Safety Phase is safety, with 30-50 patients recruited to each research arm. Research arms will only continue to recruitment in the next stage if they have been shown to be both safe and feasible, although patient data from all patients and all stages will be included in the final analyses. In the Activity Stages the primary outcome measure is failure-free survival (FFS). Each Activity Stage is triggered when a pre-specified number of FFS events have been observed in the control arm of the relevant comparison (see **Section 9** for further detail). Recruitment to Arms D (ADT + celecoxib) and F (ADT + zoledronic acid + celecoxib) was stopped in Apr-2011 after the second planned activity analysis when the IDMC and TSC considered the lack-of-benefit guidelines.(1) (See to **Section 9.4** for further information regarding the guidelines for stopping accrual to research arms during the activity stages of the trial).

Some evidence of activity will be required for a research arm to continue past each stage and guidelines are in place for this assessment of activity. The Efficacy Stage will take place when a pre-specified number of deaths are observed amongst the control arm patients for that relevant comparison. This was when around 403 deaths had been reported in the control arm for the “original comparisons” (involving docetaxel and zoledronic acid) and will be when around 267 deaths are reported in the control arm for the “abiraterone comparison”, the “M1|RT comparison” and the “enzalutamide+abiraterone comparison”. The exact number of patients randomised to, and duration of, the trial will depend on the observed accrual rate, observed event rate and the number of other research arms open to recruitment.

In version 8.0 of the protocol a new arm G (ADT + abiraterone) was added. Arm H (ADT+ prostate radiotherapy) was added in protocol version 9.0. The trial stages remain similar to those at trial inception but will be staggered in time compared to the stages for the original arms A-F. Protocol version 10.0 was approved following the completion of recruitment to the remaining original trial arms (B, C and E) and was a "housekeeping" change to remove references to the completed arms

from the information sheets. Protocol version 11.0 was approved following the extension of the recruitment target sample for the “abiraterone comparison” from 1,500 to around 1,800 patients. Protocol version 12.0 added a new combination therapy arm containing abiraterone with enzalutamide; for this comparison we envisage only two pre-planned interim analyses. Protocol 13.0 was approved following the extension of the recruitment target sample for the "M1|RT comparison", from 1,250 to around 1,800 patients, and the introduction of saliva sample collection for DNA analysis. This current protocol amendment (Protocol 14.0) updates the standard-of-care to permit docetaxel in response to the results of the primary analysis of the "original comparisons".

Patients will be assessed 6 weekly for the first 24 weeks after randomisation and then every 12 weeks up to 2 years, 6-monthly until 5 years and annually, thereafter. The first 700 patients on trial completed questionnaires aimed at assessing the effects of the investigational treatments on their quality of life (QL) and their use of health care resources (Health Economics (HE) study). From protocol version 8.0, the QL and HE study has been re-opened to all new patients.

In addition, there are translational sub-studies. Patients willing to participate will be asked at randomisation to donate a saliva sample (previously a droplet of blood), which will be stored for DNA and protein analysis in order to try to identify markers that are associated with response to therapy, side-effects or susceptibility to prostate cancer.

Patients will also be asked to give permission to use some of their stored material (blood or biopsy samples) for further studies on the causes and nature of prostate cancer. In selected centres patients were previously asked to participate in a bone mineral density sub-study (sub-study now closed). There are separate patient information sheets for the QL and HE study and the translational sub-studies (For further details of ancillary studies, see Section 17).

1.3 TRIAL DOCUMENTATION

Table 1 presents a summary of the required trial documentation for participating centres

Table 1: Trial documentation required for participating centres

TRIAL DOCUMENTATION	TIMING
R&D approval (including IRMER approval)	Before centre participation
Investigator Statement	Before centre participation
Signature list & delegation of responsibilities	Before centre participation
Trial personnel contact details	Before centre participation
PIS, GP & CF on local paper	Before centre participation
Signed Clinical Trial Agreement between Trust and Sponsor (or Variation if applicable)	Before centre participation
RTQA accreditation	Before centre participation

2 BACKGROUND

2.1 INTRODUCTION AND SETTING

Prostate cancer is a major health problem world-wide and accounts for nearly one fifth of all newly diagnosed male cancers. In the UK, approximately 41,000 men are diagnosed with prostate cancer each year and in 2012 over 10,000 men died from the disease.(2)

2.1.1 LONG-TERM ANDROGEN DEPRIVATION THERAPY

The initial (first line) treatment for locally advanced or metastatic prostate cancer is androgen deprivation therapy (ADT) achieved either surgically with bilateral orchidectomy, or medically with LHRH agonists or antagonists (3) Oral anti-androgens are no longer permitted for new patients within the trial from version 8.0.

ADT produces responses in up to 95% of patients but it is not curative and disease recurs in virtually all patients treated with ADT as sole therapy, with a median time to progression of 18-24 months.(3) Data from STAMPEDE has shown progression to be just 12 months in men with newly-diagnosed disease. Such progressive disease is referred to as castrate resistant prostate cancer (CRPC); although this term is unpopular with patient groups due to its perceived pejorative overtones related to castration and hence terminology may yet change again in the future.

2.1.2 ROLE OF RADIOTHERAPY FOR MEN WITH M0 DISEASE

Two randomised trials, SPCG7 (4) and NCIC PR.3 / MRC PR07 (5-7) have tested the question of whether ADT alone combined with radiotherapy is the best treatment for high-risk patients with no evidence of spread outside the pelvis. Both trials demonstrated an improvement in overall and disease specific survival from the addition of radiotherapy to ADT. The size of this overall survival benefit is substantial (hazard ratio 0.68 in SPCG7 and HR 0.77 in PR07). With substantial benefit demonstrated in two mature, large, well conducted randomised trials, we now mandate that radiotherapy be standard for patients with no nodal or metastatic spread. Patients in this category will now only be allowed to enter the trial if standard radiotherapy is planned, with the exception of those for whom radiotherapy is contra-indicated. Such patients should be discussed with the Trials Unit prior to inclusion. For patients with node positive, M0 disease there are no clear data on whether radiotherapy is indicated or not. The NCIC PR.3 / MRC PR07 trial included patients with unknown nodal status who received whole pelvic radiotherapy. Given the large overall benefit observed in this trial, the STAMPEDE TMG recommends that pelvic nodal radiotherapy be considered for patients with node positive, non-metastatic disease at the discretion of the treating clinician [James et all (in press); JAMA Oncology].

2.1.3 ROLE OF DOCETAXEL FOR MEN WITH M0 OR M1 DISEASE

The primary analysis of the "original comparisons" has shown docetaxel to significantly prolong survival (HR 0.78; 95% CI 0.66-0.93). This is in support of the results of the CHAARTED study which showed docetaxel improved survival in men with metastatic disease.(8) There was no evidence of heterogeneity in the treatment effect across patient groups and median survival was improved by 10 months, from 71 to 81 months. In a well powered and pre-planned sub-group analysis of men with metastatic disease at diagnosis the treatment effect was most apparent with the median survival benefit of 15 months. As a result the STAMPEDE TMG recommends that docetaxel should be strongly considered in all men with metastatic disease at presentation who are commencing ADT for the first time and are fit enough to receive chemotherapy.

Survival data for men without metastases at diagnosis is less mature but a statistically significant improvement in failure free survival is seen, therefore, docetaxel may also be considered for men with high-risk non-metastatic disease.

Therefore, docetaxel is now permitted as part of the standard-of-care for all men entering STAMPEDE at the discretion of the treating clinican and patient.

2.2 RATIONALE

There are increasing numbers of treatments which are used post relapse of first-line ADT in patients with CRPC, but there is little evidence as to which is associated with the best response, how they may be combined or sequenced or whether any of them might have a role as first-line treatment. Such treatments include further hormonal manipulations, bisphosphonates, (9), cytotoxic chemotherapy (10), new hormone therapies (11) and palliative radiotherapy. The traditional approach to the testing and introduction of new treatments for prostate cancer is to use them in patients with castrate resistant disease. An alternative approach is to investigate new drugs and new approaches to treatment, as first-line therapy in patients starting ADT. At this point, patients should be fitter and better able to tolerate treatment than when they have CRPC, and there is the possibility of having a larger and longer lasting effect.

2.3 DESIGN

STAMPEDE (also known as MRC PR08) is an innovative, multi-arm multi-stage, multi-centre, randomised controlled trial. It initially assessed the effects of a bisphosphonate (zoledronic acid), a cytotoxic chemotherapeutic agent (docetaxel) and a cyclooxygenase (Cox-2) inhibitor (celecoxib), as single agents or combinations, in patients commencing long-term ADT for advancing or metastatic prostate cancer. For these questions, each comparison was divided into five stages such that, for each investigational arm, safety and activity data were generated in the first four stages; an investigational arm could only proceed to the fifth and final stage of recruitment, where it would be assessed for effect on overall survival, if shown to be sufficiently safe and active at all prior activity stages. It is important to note, however, that patient data from all arms and all stages are included in the final analyses of the primary outcome measure, even if the investigational arm did not proceed to the final stage.

A second planned interim analysis failed to demonstrate sufficient activity for celecoxib and this agent was removed from trial recruitment in April 2011; patients remaining on celecoxib treatment reverted to standard care. Protocol version 8.0 added a new drug abiraterone to the study as an additional arm (see **Section 2.7**). Protocol version 9.0 added a new comparison arm involving prostate radiotherapy for patients with newly-diagnosed metastatic disease (see **Section 2.8**). Protocol version 10.0 reflected the successful completion of recruitment to three docetaxel- and bisphosphonate-containing arms (Arms B, C and E) and removed references to these agents in the information sheets for new patients. Protocol version 11.0 extended the recruitment target for the "abiraterone comparison" (A vs G) from 1,500 to around 1,800 patients. Protocol version 12.0 added a new comparison involving the combination of abiraterone and enzalutamide. Protocol version 13.0 extended the recruitment target for the M1|RT comparison from 1,250 to around 1,800 patients. Protocol version 14.0 incorporates the permitted use of docetaxel in the standard-of-care.

2.4 RESEARCH TREATMENT: BISPHOSPHONATES

Note: recruitment stopped to the zoledronic acid and docetaxel-containing arms in Mar-2013 as recruitment target sample was reached.
Treatment has been completed in all patients and the results reported. See Protocol version 13.0 or older for details on the rationale.

2.5 RESEARCH TREATMENT: CHEMOTHERAPY

Note: recruitment stopped to the zoledronic acid and docetaxel-containing arms in Mar-2013 as recruitment target sample was reached.
Treatment has been completed in all patients and the results reported. See Protocol version 13.0 or older for details on the rationale.

2.6 RESEARCH TREATMENT: CYCLOOXYGENASE-2 INHIBITORS

Note: recruitment completed to both celecoxib-containing arms in Apr-2011 at the end of Activity Stage II of this comparison.
Treatment has been ceased in all patients and the results to be reported in 2016. See Protocol version 13.0 or older for details on the rationale.

2.7 RESEARCH TREATMENT: STEROID SYNTHESIS INHIBITORS

Recent evidence suggests that an important mechanism for escape from tumour control by androgen ablation is the intracellular conversion of steroid precursors to androgenic steroids by prostate cancer cells. A key enzyme in this process is CYP17, which therefore represents a logical target for therapy in CRPC. (11). Abiraterone acetate (3β-acetoxy-17-(3-pyridyl)androsta-5,16-diene, code CB7630; JNJ-212082) is rapidly converted in vivo to abiraterone (JNJ-589485; formerly code named CB7598). It is a selective, irreversible inhibitor of 17α-hydroxylase/C17,20-lyase (cytochrome P450c17 [CYP17]), an enzyme that is critical in the production of androgens in the testes, adrenal glands and prostate tumor tissue. Inhibition of CYP17 inhibits the conversion of pregnenolone or progesterone into dehydroepiandrosterone (DHEA) or androstenedione, respectively, each of which is a precursor of testosterone. The pharmacodynamic effect is a more effective androgen depletion than can be induced by surgical castration, or medically by gonadotropin releasing (GnRH) hormone analogues used as first line hormone therapy in prostate cancer.

Approximately 2,280 prostate cancer patients participated in the two Phase 3 RCTs (COU-AA-301 and COU-AA-302), with approximately 1,335 patients receiving abiraterone acetate at 1000mg daily dose continuously, in these studies. These studies have demonstrated abiraterone to prolong survival when given post-docetaxel (HR 0.65) and pre-docetaxel (HR 0.82). As a result it is now approved use in the USA and Europe in CRPC.(12, 13)

Side-effects with abiraterone acetate are modest with the main adverse effects being elevated transaminases (usually mild), hypokalaemia and hypertension due to secondary hyperaldosteronism and fluid retention (preventable by low doses of glucocorticoids). In order to prevent secondary hyperaldosteronism, it is recommended that prednisolone (or prednisone) 10mg daily be administered in the CRPC setting. Within more recent studies in earlier stage patients, lower doses (typically 5mg of prednisone/prednisolone) are being used due to concerns about side effects of long-term exposure to glucocorticoid. More recent evidence even suggests that for most patients,

no glucocorticoids may be needed. (14) Within the STAMPEDE trial, we suggest prednisolone/ prednisone dose of 5mg daily.

We hypothesise that the agent may be more active still when given up-front in combination with first-line ADT by preventing or delaying the development of castrate refractory disease.

2.8 RESEARCH TREATMENT: RADIOTHERAPY TO THE PROSTATE FOR PATIENTS WITH NEWLY-DIAGNOSED METASTATIC DISEASE

Therapy directed against the primary tumour in the presence of metastatic disease has been evaluated rigorously in only one malignancy to date: renal cell carcinoma. Two cooperative groups ran randomised trials enrolling patients with previously untreated metastatic RCC whose primary tumours were amenable to surgical resection. Patients were randomised to receive the standard systemic therapy of the day, interferon-alpha, either alone or with radical nephrectomy. The combination of nephrectomy and interferon was shown to significantly improve median survival from 7 to 17 months in one trial (15) and from 8 to 11 months in the other.(16) The mechanism by which nephrectomy improves survival remains obscure. In preclinical models, the primary tumour has been found to secrete molecules that prime the microenvironment in which metastases can develop. An implication of this work is that therapy directed at the primary tumour, by abrogating this endocrine signalling, could retard the formation and the growth of distant metastases.

The results of two large-scale randomised trials of prostate radiotherapy are also provocative. The Scandinavian SPCG-7 trial and the MRC PR07 trial randomised men with locally advanced prostate cancer, who were at high risk of possessing occult metastatic disease, to either ADT alone or ADT plus prostate radiotherapy.(4, 17) The addition of radiotherapy dramatically improved 10-year outcomes: mortality from prostate cancer was halved. Interestingly, the benefit of radiotherapy started to emerge as early as three years from the time of randomisation. This seems improbably early if the benefit of local treatment is mediated via the prevention of subsequent disease dissemination. Rather, it is more consistent with the possibility that local treatment has a beneficial impact on the rate of progression of existing micrometastatic disease.

We hypothesise that local therapy to the primary site may retard distant disease progression and prolong survival in patients with newly-diagnosed metastatic prostate cancer.

Figure 3: Use of RT in STAMPEDE

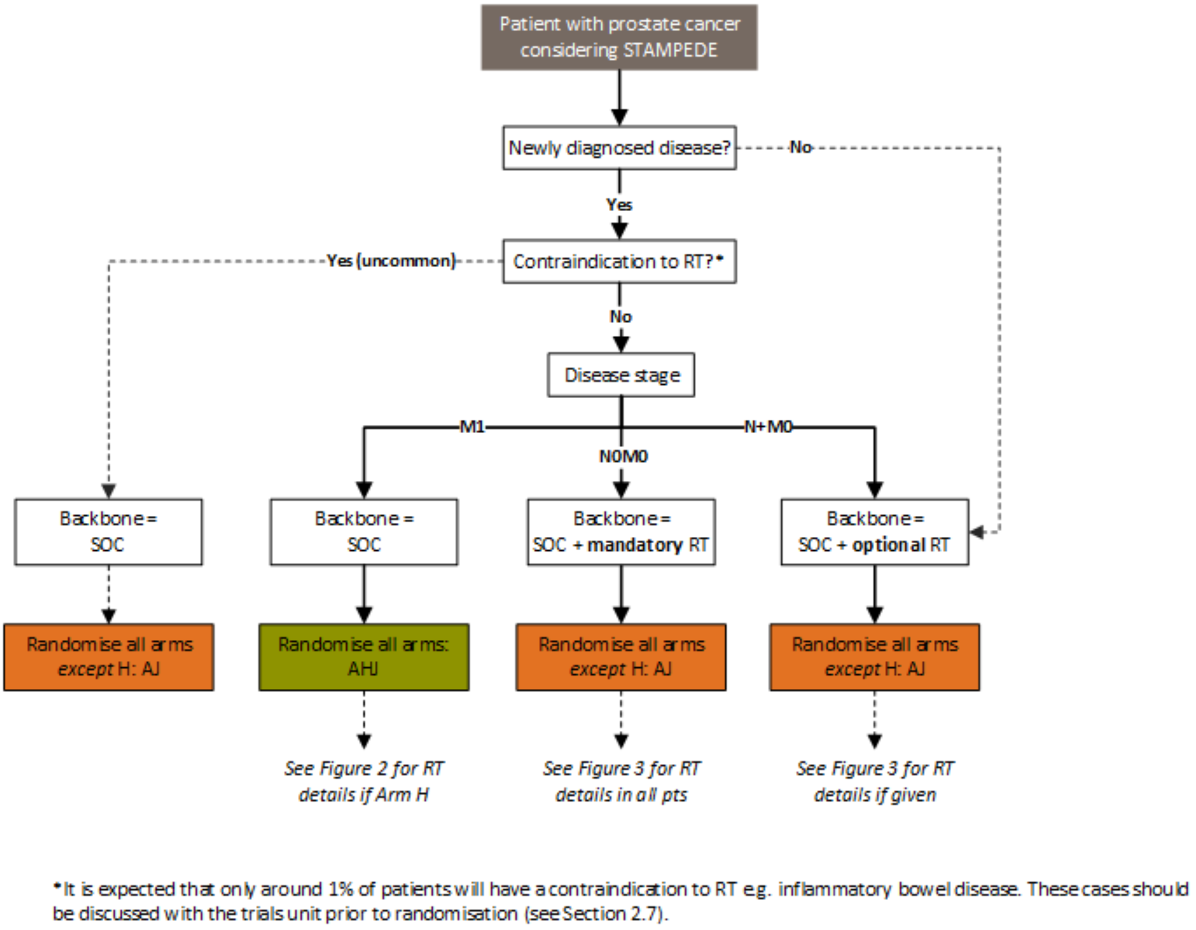

*It is expected that only around 1% of patients will have a contraindication to RT e.g. inflammatory bowel disease. These cases should be discussed with the trials unit prior to randomisation (see **Section 4.3**).

2.9 RESEARCH TREATMENT: COMBINATIONS OF ORIGINAL RESEARCH ARMS

2.9.1 BISPHOSPHONATE AND CHEMOTHERAPY

Note: recruitment stopped to the zoledronic acid and docetaxel-containing arms in Mar-2013 at the end of Activity Stage IV. Treatment has been completed in all patients and the results reported. See Protocol version 13.0 or older for details on the rationale.

2.9.2 BISPHOSPHONATE AND CYCLOOXYGENASE-2 INHIBITORS

Note: recruitment stopped to both celecoxib-containing arms in Apr-2011 at the end of Activity Stage II.

2.10 COMBINATION OF STEROID SYNTHESIS INHIBITORS AND ANDROGEN RECEPTOR SIGNALLING INHIBITOR

The majority of patients with advanced prostate cancer who have disease progression on abiraterone or enzalutamide taken as single agents, have a rise in PSA, suggesting reactivation of androgen receptor (AR), or other steroid signalling pathways resulting in increased PSA transcription, is the pathway to the development of resistance.(18)

The primary pharmacodynamic effect of enzalutamide is inhibition of androgen binding to the AR, AR nuclear translocation in the presence of androgen and AR:chromatin association. In multiple prostate cancer cell lines that specifically model CRPC (LNCaP/AR, VCaP, W741C LNCaP), the consequences of enzalutamide treatment include inhibition of AR-induced gene transcription, reduced cell proliferation, increased cell death by apoptosis and tumor regression.

In a mouse xenograft model of CRPC using prostate cancer cells that overexpress the AR (LNCaP/AR), enzalutamide inhibits tumor growth and reduces tumor size. A major human metabolite of enzalutamide, N-desmethyl enzalutamide, demonstrates key primary pharmacodynamics of similar potency to the parent molecule, while the carboxylic acid derivative metabolite has no known pharmacodynamic effect.

The question under investigation is: can progression be delayed (and survival extended) by using a combination of abiraterone and enzalutamide?

2.10.1 Supplementing Abiraterone And Prednisolone With Enzalutamide

Several studies have shown that the AR can become promiscuously activated by very low levels of androgens or other steroid metabolites and drugs that bind the AR.(19-22) It is known that very low levels of androgens can persist in patients treated with abiraterone acetate.(23) Drugs that bind the AR, may include co-administered glucocorticoids. Furthermore, AR mutations of the sort previously described in CRPC, can be activated by cortisol and other glucocorticoids at levels much lower than those reported in patients treated with abiraterone and prednisolone at a dose of 5mg bid.(22, 24) Moreover, abiraterone binds the AR and, although weak antagonism of wild-type and most previously described AR mutations are observed,(24) a similar mechanism to that described with classical anti-androgens, such as bicalutamide, could lead to change-of-function AR mutations associated with AR activation following abiraterone binding. Therefore, concomitant treatment with an androgen receptor signalling inhibitor could prevent “promiscuous” AR activation in patients treated with abiraterone. Enzalutamide is a androgen receptor signalling inhibitor and has gained recent approval for use on its own in the treatment of advanced CRPC,(25) and there is evidence of activity for hormone-naïve prostate cancer.(26)

2.10.2 Supplementing Enzalutamide With Abiraterone And Prednisolone

Enzalutamide in combination with ADT is both effective and well tolerated in CRPC.(25) However, recent studies have suggested that intra-tumoral testosterone levels increase in patients treated with enzalutamide.(27) The implications of this finding are that the increase in intra-tumoral testosterone could be associated with up-regulation of enzymes involved in steroid biosynthesis.(28) Although enzalutamide has a high affinity for the AR, this is several-fold lower than both the natural ligands testosterone and DHT,(29) which means that enzalutamide would be out-competed at the AR ligand-binding domain if and when androgen levels rise. In vitro, a ten-fold rise in intra-cellular androgen was sufficient to prevent inhibition of AR by 30uM of enzalutamide;(24) these levels are

representative of the plasma levels of enzalutamide active metabolites, which can be achieved with enzalutamide 160mg po daily.(30)

A strategy for preventing the rise in intra-cellular androgens in patients treated with enzalutamide would be inhibition of CYP17A1. Abiraterone is currently the only CYP17A1 inhibitor with proven efficacy. It therefore seems logical to use the combination of enzalutamide and abiraterone to both block a rise of intra-cellular androgens and prevent promiscuous activation of the AR.

2.10.3 Summary Of Rationale For This Combination

To date, investigation has focussed on patients with CRPC but there is a strong rationale for the combination of enzalutamide and abiraterone in the hormone treatment-naïve setting in which STAMPEDE is focused.

STAMPEDE is already evaluating abiraterone plus conventional ADT but we will not assess the combination of conventional ADT plus enzalutamide; other trials by industry and other cooperative groups will address that question. The inclusion of an arm with ADT and enzalutamide in STAMPEDE was therefore considered to be a duplication of effort and was not supported by the Trial Management Group.

The combination of enzalutamide and abiraterone is a novel approach and offers considerable promise in delaying progression – it therefore represents an attractive addition to the comparisons under investigation in STAMPEDE, and one that is unlikely to be replicated in other planned trials of this size.

3 SELECTION OF INSTITUTIONS AND INVESTIGATORS

Centres who wish to participate in the STAMPEDE trial should be registered with the Medical Research Council Clinical Trials Unit at University College London (MRC CTU at UCL) for this purpose. Before any patients are randomised the MRC CTU must receive a completed and signed Investigator Statement. The STAMPEDE investigator statement is signed by the Principal Investigator for that institution (download from www.stampedetrial.org). R&D approval for the site, along with a fully-signed model agreement, are also required before recruitment can begin.

In addition, and in compliance with the principles of GCP, all institutions participating in the trial will complete a delegation log and forward this to the MRC CTU. Each person working on the STAMPEDE trial must sign off a section of this log indicating their responsibilities. The MRC CTU must be notified of any changes to trial personnel and/or their responsibilities. An up-to-date copy of this log must be stored in the Investigator Site file at the institution and also at the MRC CTU.

The Clinical Trial Authorisation (CTA) for the STAMPEDE trial requires that the Medicines and Healthcare Products Regulatory Agency (MHRA) be supplied with the names and addresses of all participating investigators/institutions. Trial staff at the MRC CTU will perform this task; hence, it is vital to receive full contact details for all investigators prior to their entering patients.

Finally, before a patient is entered into the trial written informed consent must be obtained. Approved patient information sheets and informed consent forms are supplied as templates.

Only a limited number of centres participated in the initial Pilot Phase of the original trial; this was to ensure that safety and feasibility data were collected expediently. Subsequent stages of the trial are open to any centre that wishes to participate and has fulfilled the requirements described above.

3.1 RADIOTHERAPY ACCREDITATION

The introduction of the "M1|RT comparison" in Protocol 9.0 introduced the need for RTQA accreditation in sites giving radiotherapy. The detail of RTQA accreditation is in **Appendix K**. However, centres that have been RTQA accredited for another multi-centre prostate radiotherapy trial in the UK (e.g. RADICALS or CHHIP) will be automatically granted STAMPEDE RTQA accreditation.

4 SELECTION OF PATIENTS

4.1 PATIENT INCLUSION CRITERIA

Patients must fulfil both of the criteria in **Section 4.1.1** **or** one criterion in **Section 4.1.2** **or** at least one criterion in **Section 4.1.3**. Additionally, all patients must fulfil the criteria in **Section 4.1.4**.

4.1.1 High-Risk Newly-Diagnosed Non-Metastatic Node-Negative Disease

Both:

- At least two of: T category T3/4, PSA≥40ng/ml or Gleason sum score 8-10
- Intention to treat with radical radiotherapy (unless there is a contra-indication; exemption can be sought in advance of consent, after discussion with MRC CTU)

OR

4.1.2 Newly-Diagnosed Metastatic Or Node-Positive Disease

At least one of:

- Stage T_{any} N+ M0
- Stage T_{any} N_{any} M+

OR

4.1.3 Previously Treated With Radical Surgery And/Or Radiotherapy, Now Relapsing[1]

At least one of:

- PSA ≥4ng/ml and rising with doubling time less than 6 months
- PSA ≥20ng/ml
- N+
- M+

AND

4.1.4 For All Patients

I. Histologically confirmed prostate adenocarcinoma
II. Intention to treat with long-term androgen deprivation therapy
III. Treating clinician and patient must have decided if docetaxel is to be part of the standard-of-care prior to randomisation
IV. Fit for all protocol treatment[2] and follow-up, WHO performance status 0-2[3]
V. Have completed the appropriate investigations prior to randomisation
VI. Adequate haematological function: neutrophil count >1.5x10^9/l and platelets >100x10^9/l
VII. Estimated creatinine clearance >30ml/min
VIII. Serum potassium ≥3.5mmol/L
IX. Written informed consent
X. Willing and expected to comply with follow-up schedule
XI. Using effective contraceptive method if applicable

[1] Courses of hormone therapy for localised disease must have been completed at least 12 months previously and have been no longer than 12 months in duration. It can have been given as adjuvant or neoadjuvant therapy.
[2] Medical contraindications to the trial medications are given in **Section 6.11.4 and 4.3**
[3] For WHO performance status definitions see **Appendix A**

4.2 PATIENT EXCLUSION CRITERIA[4]

Patients must not fulfil any of the criteria, below.

I. Prior systemic therapy for locally advanced or metastatic prostate cancer except as listed in **Section 4.1.3**
II. Metastatic brain disease or leptomeningeal disease
III. Abnormal liver functions consisting of any of the following:
   - Serum bilirubin ≥1.5 x ULN (except for patients with Gilbert's disease, for whom the upper limit of serum bilirubin is 51.3µmol/l or 3mg/dl)
   - Aspartate aminotransferase (AST) or alanine aminotransferase (ALT) ≥2.5 x ULN
IV. Any other previous or current malignant disease which, in the judgement of the responsible clinician, is likely to interfere with STAMPEDE treatment or assessment
V. Patients with contra-indications to prednisolone, including active peptic ulceration or a history of gastrointestinal bleeding
VI. Patients with active inflammatory bowel disease
VII. Symptomatic peripheral neuropathy grade ≥2 (NCI CTC)[5]
VIII. Any surgery (e.g. TURP) performed within the past 4 weeks
IX. Patients with significant cardiovascular disease such that, in the investigator's opinion, the patient is unfit for any of the study treatments. This might include:
   - Severe/unstable angina
   - Myocardial infarction less than 6 months prior to randomisation
   - Arterial thrombotic events less than 6 months prior to randomisation
   - Clinically significant cardiac failure requiring treatment (NYHA II-IV)[6]
   - Cerebrovascular disease (e.g. stroke or transient ischaemic episode) less than 2 years prior to randomisation
   - Patients with uncontrolled hypertension defined as systolic BP greater or equal than 160 mmHg or diastolic BP greater or equal than 95 mmHg
X. Patients receiving treatment with drugs known to interact with CYP3A4 and CYP2C9 (please see **Table 11 and Table 12** for more details on drug interaction)
XI. Prior exposure to abiraterone
XII. Prior exposure to enzalutamide
XIII. Prior chemotherapy for prostate cancer (excluding patients receiving docetaxel as part of the new SOC)
XIV. Prior therapy with zoledronic acid or other bisphosphonates other than treatment for hypercalcaemia or low bone density
XV. Prior exposure to long-term hormone therapy before randomisation (unless as described in **Section 4.4.2**)
XVI. History of seizure including any febrile seizure, brain injury with loss of consciousness, or transient ischaemic attack within the 12 months prior to randomisation or any history of prior conditions that may pre-dispose to seizure (e.g., prior stroke, brain arteriovenous malformation)
XVII. Unexplained history of loss of consciousness within 12 months of randomisation
XVIII. Operation of heavy machinery during treatment

[4] The exclusion criteria for patients who have been on a Cox-2-inhibitor for 6+ months has been removed
[5] See **Appendix I** for common toxicity grading
[6] NYHA classifications can be found in **Appendix A**

4.3 SELECTION CRITERIA FOR COMPARISON OF RESEARCH RT FOR METASTATIC DISEASE (M1|RT)

All patients meeting criteria in **Section 4.1** and **4.2** are eligible for the trial, but not all can be allocated to the research (M1) radiotherapy arm. The selection criteria for this "RT to the prostate" comparison are:

- Newly-diagnosed prostate cancer
- Demonstrable M1 disease
- No contraindication to radiotherapy e.g. no previous pelvic radiotherapy and no history of inflammatory bowel disease
- No previous radical prostatectomy

Any patients meeting these criteria will have a chance to be allocated to Arm H. For those rare cases where radical RT is planned for a newly-diagnosed M1 patient, the TMT and TMG will need to review and approve the inclusion of the patient for randomisation only between Arm A and J.

4.4 SCREENING PROCEDURES

4.4.1 INVESTIGATIONS PRIOR TO RANDOMISATION

All patients should have the following examinations performed. The latest available scans should be used:

- CT or MRI of pelvis and abdomen
- Bone Scan (or equivalent e.g. whole body MRI)
- Chest X-ray (only if chest was not included in CT)
- ECG
- PSA Test

The following blood tests within 8 weeks (56 days) prior to randomisation:

- Testosterone (if available)
- Urea and Electrolytes
- Liver function tests
- Serum creatinine
- Serum corrected calcium
- Phosphates
- Magnesium
- Albumin
- Total cholesterol
- HDL cholesterol
- Systolic blood pressure
- Diastolic blood pressure
- Waist circumference measure

Patients who initially fail to meet the eligibility criteria can be re-screened at a later date.

Prior to randomisation:

- Check details of any prior treatments for prostate cancer
- Check any contraindications to radiotherapy

4.4.2 ANDROGEN DEPRIVATION THERAPY PRIOR TO RANDOMISATION

If ADT has already started prior to randomisation, the primary therapy should have not have started more than 12 weeks before randomisation. If a short course of anti-androgens (e.g. bicalutamide) was used to prevent tumour flare, this will not be counted in the 12 week period, but the PSA level must have been taken before this was started. The start date of anti-androgens cannot be more than 14 weeks before randomisation and patients will not be eligible if time since starting anti-androgen monotherapy has exceeded 14 weeks.

Note that long-term anti-androgen monotherapy is not permitted in the trial for newly recruited patients from protocol version 8.0; patients may change treatment to join the trial, provided that they have not had more than 12 weeks of ADT prior to randomisation. Further details on hormone therapies allowed prior to randomisation are discussed in **Appendix L**.

Any relapsing patients treated with adjuvant or neo-adjuvant hormone therapy alongside their radical surgery or radiotherapy must have completed that period of hormone therapy at least 12 months before joining STAMPEDE and it must have been no longer than 12 months in duration.

Note that baseline testosterone measurements will not be required in patients who have already commenced hormone manipulation prior to randomisation.

4.4.3 CHOICE ABOUT STANDARD-OF-CARE DOCETAXEL

The treating clinician and patient must have decided, prior to randomisation, whether docetaxel is to be given as part of standard-of-care. Docetaxel treatment must start within 12 weeks after starting ADT, preferably within 8 weeks. Patients can have already started docetaxel treatment when randomised providing this is within 12 weeks after starting ADT.

4.4.4 HYPERCALCAEMIA AT RANDOMISATION

For patients who are hypercalcaemic prior to randomisation and require treatment, it is recommended that they are treated with a bisphosphonate and that the treatment should be discontinued when they are stabilised.

4.4.5 NSAIDS AND COX-2 INHIBITORS AT RANDOMISATION

Note: recruitment completed to both celecoxib-containing arms in Apr-2011 at the end of Activity Stage II

4.4.6 STARTING TRIAL TREATMENT

Patients not receiving docetaxel as part of the standard-of-care should start allocated trial treatment as soon as possible after randomisation. Investigators should aim that this is at least within 4 weeks post-randomisation and within 12 weeks of starting ADT (see **Section 6**).

Radiotherapy for patients allocated to Arm H should be commenced within 4 weeks from randomisation and continued according to the predefined scheduled unless toxicity is reported. Any delays in starting research radiotherapy should be discussed with the STAMPEDE team and recorded as appropriate in the relevant CRF.

Docetaxel-treated patients should aim to start allocated trial treatment within 3-4 weeks after the last administration of docetaxel, preferably within a maximum of 6 weeks. Should any docetaxel-related toxicities occur during treatment these need to have recovered to grade 1 before the allocated research treatment begins. Please discuss with the trial team if any docetaxel-related

toxicities of greater severity than grade 1 persist (this excludes alopecia, nail changes and neuropathy, which do not need to have resolved prior to starting research treatment).

Investigators should discuss with the trial team any patient who may not start their allocated trial treatment or where a delay of 6 or more weeks, between last administration and starting research treatment is expected.

4.4.7 Concomitant Medications

Before randomising all patients, please check **Table 11 and 12** for drug interactions.

All concomitant medications should be recorded including any vitamin and mineral supplements the patient is taking, regular consumption of NSAID and/or aspirin and use of other bisphosphonates (see **Section 4.2**). Of particular interest in this are herbal preparations such as PC-SPES, Prostasol, Saw Palmetto and St John's Wort.

All concomitant medications should be continued throughout the trial unless the responsible clinician decides otherwise. If patients continue to require medication for the management of docetaxel-related toxicities, please discuss this with the trial team. Please see **Section 6** for more information on concomitant medications and their use with abiraterone and enzalutamide.

4.5 ADDITIONAL DETAILS FOR PATIENTS JOINING SUB-STUDIES

If the patient has given their consent to participate in the DNA analysis sub-study, a saliva sample will be collected. This replaces the droplet of blood collected in previous versions of the protocol. An additional element of DNA analysis will involve the collection of plasma samples for patients in the "enzalutamide + abiraterone comparison".

The local pathologist will also be asked to make the tissue block from the tumour sample remaining after primary interrogation available for tissue micro array analysis to be carried out, if the patient has given consent for his remaining samples to be used for further analyses. Full details of all sub-studies and instructions relating to the handling of the saliva and blood sample are given in **Section 17** and **Appendix D**.

5 RANDOMISATION AND ENROLMENT

Patients will be allocated to any of the open research arms for which they are suitable. Patients with non-metastatic disease or who have had previous local therapy to the prostate or who have a contraindication to radiotherapy will not be allocated to Arm H (see **Section 4.3**).

To enter a patient the randomisation form should be completed carefully and the MRC CTU contacted by phone:

RANDOMISATIONS
To randomise, call MRC CTU at UCL, Monday to Friday 0900-1700
excluding public holidays or dates when notice has been given by the CTU.
Tel: +44 (0) 20 7670 4777

A trial number and treatment will be allocated and given over the phone or by return fax. In addition, a letter confirming these details will be sent. The trial number will be the primary way in which the patient will be identified and should be used in all correspondence.

5.1 CO-ENROLMENT GUIDELINES

Ideally, patients should not be participating in any other clinical trial of prostate cancer treatment when they enter STAMPEDE and should not enter any other trials until a failure-free survival (FFS) event has been experienced and reported. After this point, the patient may be entered into further, second-line treatment studies. The primary outcome measure of STAMPEDE is overall survival. Participation in post-progression studies must be reported on the Co-enrolment CRF.

Data sharing agreements with “down-stream” trials are encouraged to improve data quality in both trials and to reduce costs to both organisations.

6 TREATMENT OF PATIENTS

6.1 TRIAL TREATMENT

Patients will be randomised to the control arm (Arm A) or one of the research arms. All patients will receive androgen deprivation therapy (ADT) to achieve castration levels of testosterone. The method of ADT is a local choice but must be specified for each patient prior to randomisation. The recommended methods of ADT are given in Section 6.2.1. Note that from protocol version 8.0 onwards, bicalutamide monotherapy is no longer permitted as a trial therapy for new patients but patients may switch to a permitted therapy to join the trial.

6.1.1 Required timelines when starting trial treatment

Allocated treatment should start promptly after randomisation. In patients having docetaxel as part of standard-of-care, this will be within 3-4 weeks after the last docetaxel cycle, preferably within a maximum of 6 weeks. In patients not having docetaxel trial treatment should start as soon as possible after randomisation.

6.2 ARM A: ADT ALONE OR ADT + STANDARD-OF-CARE (M0) RT OR ADT +/- DOCETAXEL +/- (M0) RT

The standard-of-care for this patient group is **androgen deprivation therapy** (see Section 6.2.1). For some patient groups, this should now be supplemented with standard radiotherapy (see Section 6.2.2). From Protocol 14.0 onwards the standard-of-care includes permitted use of docetaxel for patients joining STAMPEDE (see Section 6.2.3)

6.2.1 Hormone Therapy

The permitted methods of ADT are bilateral orchidectomy, LHRH analogues and LHRH antagonists. Patients having a bilateral orchidectomy are required to adher to the same timelines as specified in Section 4.4.2. unless there is a strong clinical reason not to do so. Other methods of ADT should be discussed with the Chief Investigator or the Trial Surgeon. The planned duration of ADT should be at least 2 years.

Bilateral orchidectomy: Operations should be performed by appropriately trained surgeons. A total or sub-capsular orchidectomy may be performed.

LHRH agonists: LHRH agonists/analogues used according to local practice. The prophylactic use of anti-androgens to prevent tumour “flare” is recommended.

LHRH antagonists: LHRH antagonists used according to local practice. The use of prophylactic use of anti-androgens to prevent tumour “flare” is not necessary.

6.2.2 Standard-of-care (M0) RT

N0M0 patients: Investigators should give standard radiotherapy (RT) to patients with node negative, non-metastatic disease (N0M0), in accordance with data from the PR07 and SPCG trials. If there is an intention to omit radiotherapy (e.g. contraindications) in patients with N0M0 disease this must be discussed with the Trials Office before consent. See Section 6.16 for further details of radiotherapy administration.

N+M0 patients: the benefit of radiotherapy in this group is at present uncertain with no firm data to either support or refute its use. However, the PR07 trial included some node positive patients as cross sectional imaging was not a part of the baseline assessment in this trial, which did include whole pelvis radiotherapy. For patients with node positive, non-metastatic disease, radiotherapy is therefore recommended in suitable cases [James et all (in press); JAMA Oncology].

Investigators will be asked to state their intention with regards to planned radiotherapy in this group at randomisation. Intention to give radiotherapy (or not) for node positive patients must be stated at randomisation to ensure that there is no bias towards particular combinations of systemic therapy with radiotherapy.

Standard-of-care radiotherapy is not a core part of the trial, therefore we intend to collect minimal data about the radiotherapy administered. It is accepted that some patients will develop progressive disease before radiotherapy can be administered and if this occurs the reasons for non-delivery of treatment must be recorded on the radiotherapy detail form.

6.2.3 STANDARD-OF-CARE DOCETAXEL

Investigators are strongly encouraged to consider giving docetaxel as part of the standard-of-care for patients with newly-diagnosed metastatic disease based on the survival benefit demonstrated by both STAMPEDE in the primary analysis of the "original comparisons" and CHAARTED.(8) Investigators may also consider giving docetaxel to patients with high-risk locally advanced disease given both the significant improvement in failure-free-survival and consistency of effect for prostate cancer-specific survival shown by STAMPEDE.

The treating clinician and patient must have decided if docetaxel is to be given prior to randomisation and treatment may have started when the patient is randomised. As with standard radiotherapy, minimum data collection will be required however the start and end dates of treatment are needed to ensure the appropriate timelines are met (see **Section 6.1.1**). A confirmation whether the treatment with docetaxel has started or not as planned will also need to be sent to the trial team.

Docetaxel is given according to local protocols as a standard non-trial treatment. The regime used previously within STAMPEDE was 75mg/m2 Day 1 as 1hr IV infusion, plus prednisolone 5mg bid daily for 21 days repeated every 3 weeks for a maximum of 6 cycles.

The STAMPEDE TMG would suggest prednisolone could be omitted and data on the use of co-prescribed steroid will be collected on the relevant CRF (please see **Table 13** for more details)

6.3 ARM B: ADT + ZOLEDRONIC ACID

Note: recruitment stopped to the zoledronic acid and docetaxel-containing arms in Mar-2013 as recruitment target sample was reached. Please see Protocol version 13.0 for information on the administration of this trial drug

6.4 ARM C: ADT + DOCETAXEL

Note: recruitment stopped to the zoledronic acid and docetaxel-containing arms in Mar-2013 as recruitment target sample was reached. Please see Protocol version 13.0 for information on the administration of this trial drug

6.5 ARM D: ADT + CELECOXIB

Note: recruitment completed to both celecoxib-containing arms in Apr-2011 at the end of its Activity Stage II. Please see Protocol version 13.0 for information on the administration of this trial drug

6.6 ARM E: ADT + DOCETAXEL + ZOLEDRONIC ACID

Note: recruitment stopped to the zoledronic acid and docetaxel-containing arms in Mar-2013 as recruitment target sample was reached. Please see Protocol version 13.0 for information on the administration of this trial drug

6.7 ARM F: ADT + ZOLEDRONIC ACID + CELECOXIB

Note: recruitment completed to both celecoxib-containing arms in Apr-2011 at the end of its Activity Stage II. Please see Protocol version 13.0 for information on the administration of this trial drug

6.8 ARM G: ADT + ABIRATERONE

Note: recruitment to the "abiraterone comparison" completed in Jan-2014. Please note that some patients will continue treatment until all types of disease progression or up to a maximum of 2 years. Please see sections below for more information

Androgen deprivation therapy (+/- standard-of-care M0 RT) as described in **Section 6.2.**

Abiraterone will be administered as a single 1000mg daily oral dose (4 tablets to be taken together once a day) together with prednisolone or prednisone 5mg daily to prevent secondary mineralocorticoid excess. Abiraterone absorption is increased by food. The tablets should be taken at least 2 hours after food, swallowed whole with some water. No food should be eaten for 1 hour afterwards.

Prednisolone (prednisone in Switzerland) should be taken as a single dose with food in the morning.

In patients with M1 disease, treatment with abiraterone will continue from randomisation until all types of progression have occurred, consistent with the COU-AA-301 trial (31) i.e., abiraterone would be given for these patients until a composite assessment based on:

- PSA progression (as defined in **Appendix J**)
- Radiological progression (appearance of new lesions or progression of existing lesions) **and**
- Clinical progression (defined as new cancer-related symptoms)

It is accepted that these flexible criteria for stopping treatment with abiraterone are open to the investigator's interpretation and discretion. Patients might continue treatment beyond the first

failure-free survival (FFS) event; the first FFS event must be reported as per the other arms; all types of progression (PSA, radiological and clinical) need to be reported once.

In patients with N0M0 disease or N+M0 disease undergoing radical radiotherapy, treatment would continue for 2 years or all types of disease progression as defined for M1 patients, whichever is the sooner. ADT can be discontinued in this group at 2 years at the discretion of the local investigator (see **Section 6.2.1**).

For patients with N+M0 disease not planned for radical radiotherapy, treatment will continue as for patients with M1 disease until all types of disease progression.

If a patient allocated to Arm G develops only biochemical failure, the responsible clinician might switch from abiraterone + prednisolone 5mg od to abiraterone and dexamethasone 0.5mg od.

Trial treatment must stop if other systemic treatments are initiated at any time for disease progression control (including chemotherapy, radium 223 etc). Anti-androgens (i.e. bicalutamide) should not be given in combination with abiraterone due to the risk of toxicity.

See **Section 7.1.2** for further information on the trial definition of progression.

6.9 ARM H: ADT + PROSTATE RADIOTHERAPY IN M1 PATIENTS

Standard-of-care: androgen deprivation therapy +/- docetaxel (as described in **Sections 6.2.1** and **6.2.3**)

Radiotherapy will commence as soon as practicable. For non-docetaxel treated patients, this should ideally be within 4 weeks after randomisation. Docetaxel-treated patients should aim to start trial treatment within 3-4 weeks after the last administration of docetaxel, preferably within 6 weeks (see **Section 6.9.1**)

Treatment will be according to the guidelines in **Section 6.9.1**. Two radiotherapy dose-fractionation schedules are permitted:
- 36Gy in 6 fractions of 6Gy, administered weekly over 6 consecutive weeks
- 55Gy in 20 fractions of 2.75Gy, administered daily, five days per week, over 4 consecutive weeks

Details of the recommendations for outlining, CTV and PTV are in **Section 6.9.1**.

6.9.1 RESEARCH (M1) PROSTATE RADIOTHERAPY TREATMENT ADMINISTRATION

A treatment planning CT scan will be acquired with the patient supine, with empty rectum and comfortably full bladder.

Megavoltage equipment is required with effective photon energies ≥6MV. Minimum source-to-axis distance is 100cm. Field arrangement is at the clinician's discretion: acceptable treatment techniques (field arrangement) include a 3-field (anterior, right lateral, and left lateral), 4-field (anterior, posterior, right lateral, and left lateral), or 6-field (right and left anterior oblique, right and left posterior oblique, and right and left lateral) or equivalent coplanar technique with multi-leaf collimation for all fields to adequately protect normal structures.

The Clinical Target Volume (CTV) will consist of the prostate gland alone as visualized on the treatment-planning CT scan. The base of the seminal vesicles may also be included if they are macroscopically involved. Inclusion of pelvic lymph nodes in the CTV is not permitted. The Planning Target Volume will have a 0.8 cm margin posteriorly and 1.0 cm margin in all other directions around the CTV to account for prostate gland motion and uncertainty in daily treatment setup.

Critical normal tissues should be delineated on the treatment-planning CT scan by the treating clinician:

- Rectum – inferior limit: level of ischial tuberosities; superior limit: sigmoid flexure
- Bladder – entirety

Two radiotherapy dose-fractionation schedules are permitted. In either case, radiotherapy is prescribed such that the PTV receives at least 95% of the prescribed dose:

- 36Gy in 6 fractions of 6Gy, administered weekly over 6 consecutive weeks
- 55Gy in 20 fractions of 2.75Gy, administered daily, five days per week, over 4 consecutive weeks

Dose-volume objectives for each dose-fractionation schedule are shown in **Tables 2** and **3** below. Values have been calculated using the formula BED = D[1+d/(alpha-beta ratio)] assuming an alpha-beta ratio of 3 for rectum and bladder. These are provided for guidance only.

Portal imaging to verify accuracy of treatment delivery may be done according to the participating centre's local guidelines. Image-guidance technology (e.g., gold seed intraprostatic fiducial markers, cone-beam CT scanning) will be permitted according to clinician preference but is not required. Further illustration on the research radiotherapy arm schedule is shown in **Figure 4**.

Table 2: Rectal dose volume objectives

55Gy/20F	36Gy/6F	Max Vol (%)
52.5 Gy	33.3 Gy	50%
43.5 Gy	27.8 Gy	60%
26.1 Gy	16.7 Gy	80%

Table 3: Bladder dose-volume objectives

55Gy/20F	36Gy/6F	Max Vol (%)
52.2	33.3	25%
43.5	27.8	50%

Figure 4: Diagram for deciding approach to research (M1) RT to the prostate

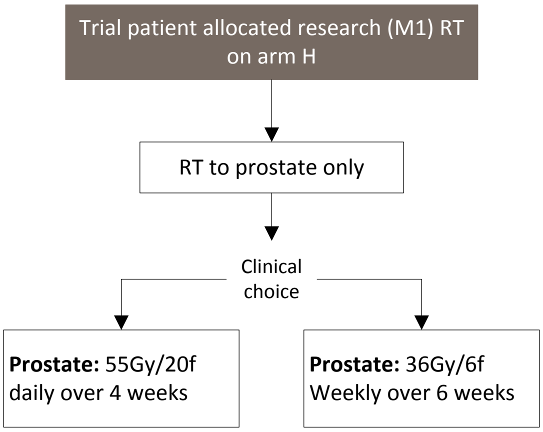

6.10 ARM J: ADT + ABIRATERONE + PREDNISOLONE + ENZALUTAMIDE ADMINISTRATION

Standard-of-care: androgen deprivation therapy +/- docetaxel and +/- M0 RT (as described in Section 6.2)

Abiraterone as described in Section 6.8.

Prednisolone as described in Section 6.8.

Enzalutamide will be administered as a 160mg oral dose (four capsules), taken together at the same time every day, with or without food.

Trial treatment must stop if other systemic treatments are initiated at any time for disease progression control (including chemotherapy, radium 223 etc). Anti-androgens (i.e. bicalutamide) should not be given in combination with enzalutamide due to the risk of toxicity.

In patients with M1 disease, treatment with both abiraterone and enzalutamide will continue until all types of progression have occurred, consistent with the approach taken for abiraterone (see Section 6.8) i.e., abiraterone and enzalutamide will be given until a composite of:
- PSA progression (as defined in Appendix J)
- Radiological progression (appearance of new lesions or progression of existing lesions) **and**
- Clinical progression (defined as new cancer-related symptoms).

It is accepted that these flexible criteria for stopping treatment with abiraterone and enzalutamide are open to the investigator's interpretation and discretion. Patients may continue treatment beyond the first failure-free survival (FFS) event ; the first FFS event must be reported as per the other arms.

In patients with N0M0 disease or N+M0 disease undergoing radical radiotherapy, treatment would continue for 2 years or all types of disease progression as defined for M1 patients, whichever is the sooner. ADT can be discontinued in this group at 2 years at the discretion of the local investigator (see Section 6.2.1).

For patients with N+M0 disease not planned for radical radiotherapy, treatment will continue as for patients with M1 disease until all types of disease progression.

If a patient has had PSA progression before commencing abiraterone and enzalutamide (i.e. on or shortly after completing docetaxel), they should still start trial treatment and should continue until radiological and/or clinical progression occurs. In the rare instances of a patient commencing abiraterone and enzalutamide having had biochemical **and** radiological progression, they should continue trial treatment whilst there is perceived evidence of benefit as judged by the local investigator. If a patient develops PSA progression only whilst on abiraterone and enzalutamide, the local investigator might consider switching from from abiraterone + prednisolone 5mg od to abiraterone and dexamethasone 0.5mg od.

See **Section 7.1.2** for further information on the definition of progression.

6.11 ADMINISTRATION, DOSE MODIFICATIONS AND MANAGEMENT OF TOXICITIES

6.11.1 Zoledronic Acid

Note: recruitment stopped to the zoledronic acid and docetaxel-containing arms in Mar-2013 as recruitment target sample was reached.
Please see Protocol version 13.0 for information on the administration of this trial drug

6.11.2 Docetaxel

Note: recruitment stopped to the zoledronic acid and docetaxel-containing arms in Mar-2013 as recruitment target sample was reached.
Please see Protocol version 13.0 for information on the administration of this trial drug

6.11.3 Celecoxib

Note: recruitment completed to both celecoxib-containing arms in Apr-2011 at the end of Activity Stage II. No new patients should be receiving this agent as first-line within the trial.
Please see Protocol version 13.0 for information on the administration of this trial drug

6.11.4 Abiraterone or Enzalutamide + Abiraterone

Abiraterone absorption is increased by food. The tablets should be taken at least 2 hours after food, swallowed whole with some water. No food should be eaten for 1 hour afterwards. Prednisolone (prednisone in Switzerland) should be taken as a single dose with food in the morning.

Enzalutamide can be taken with or without food.

6.11.4.A Abiraterone Contraindications

- Unusual or allergic reaction to past abiraterone acetate treatment
- Uncontrolled hypertension
- Uncontrolled heart failure
- Abnormal liver function or active or chronic liver disease

6.11.4.B Abiraterone Special Warnings and Precautions For Use

Timing of administration compared with meals
Administration of abiraterone acetate with food significantly increased the absorption of abiraterone acetate. Administration of 1000mg dose of abiraterone acetate tablets in fed conditions increased systemic exposure to abiraterone compared with the fasted state. Abiraterone mean Cmax and AUC values increased approximately 7- and 5-fold, respectively, when administered immediately after a low-fat meal. Abiraterone mean Cmax and AUC values increased approximately 17- and 10-fold, respectively, when administered immediately after a high-fat meal. It is, therefore, recommended that abiraterone acetate is taken on an empty stomach.

Cardiovascular history
Abiraterone acetate should be used with caution in patients with a history of cardiovascular disease. The safety of abiraterone acetate in patients with left ventricular ejection fraction <50% or New York Heart Association (NYHA) Class III or IV heart failure has not been established. Before treatment with abiraterone acetate, hypertension must be controlled and hypokalemia must be corrected.

Caution is required in treating patients whose underlying medical conditions might be compromised by increases in blood pressure, hypokalemia, or fluid retention, e.g. those with heart failure, recent myocardial infarction, or ventricular arrhythmia.

Blood pressure management
Blood pressure should be monitored every 2 weeks until week 12 and at every follow-up visit after week 12 whilst the patients remain on treatment. For the management of abiraterone induced hypertension see **Table 5.**

Hepatic Impairment
The pharmacokinetics of abiraterone was examined in patients with pre-existing mild or moderate hepatic impairment (Child-Pugh class A and B, respectively) and in healthy control patients. Systemic exposure to abiraterone acetate after a single oral 1000mg dose increased by approximately 11% and by 260% in patients with mild and moderate pre-existing hepatic impairment, respectively. The mean half-life of abiraterone was prolonged to approximately 17.7 hours in patients with mild hepatic impairment and to approximately 18.6 hours in patients with moderate hepatic impairment. No dosage adjustment was necessary for patients with pre-existing mild hepatic impairment. Abiraterone acetate should not be used in patients with pre-existing moderate or severe hepatic impairment.

Hepatotoxicity
Marked increases in liver enzymes leading to drug discontinuation or dosage modification occurred in controlled clinical studies. Serum transaminase and bilirubin levels should be measured prior to starting treatment with abiraterone acetate, every 2 weeks for the first 3 months of treatment, and monthly thereafter.

If clinical symptoms or signs suggestive of hepatotoxicity develop, serum transaminases, in particular serum alanine aminotransferase (ALT), should be measured immediately. See **Table 4** for the management of abiraterone induced hepatotoxicty.

Renal Impairment
The pharmacokinetics of abiraterone was compared in patients with end-stage renal disease on a stable hemodialysis schedule versus matched control patients with normal renal function. Systemic

exposure to abiraterone after a single oral 1000mg dose did not increase in patients with end-stage renal disease on dialysis.

6.11.4.C Abiraterone Undesirable Effects

The safety profile of abiraterone acetate across studies in CRPC was distinct from the safety profile typically associated with myelosuppressive cytotoxic agents. The most common adverse drug reactions observed in the integrated safety data for those patients who received 1000mg abiraterone acetate plus prednisone or prednisolone in clinical studies (n=1,070) were fatigue, arthralgia, peripheral oedema, back pain, bone pain, nausea, constipation, hypokalemia and anaemia.

The adverse events graded as 3 or 4 and which occurred in more than 5% of patients were fatigue, peripheral oedema, anaemia and back pain see **Appendix G**.

6.11.4.D Abiraterone Overdose

There have been no reports of overdose of abiraterone acetate during clinical studies. There is no specific antidote to abiraterone acetate. In the event of an overdose, administration of abiraterone acetate should be stopped and general supportive measures undertaken, including monitoring for cardiac arrhythmias. Liver function should also be assessed.

6.11.4.E Enzalutamide Contraindications

The full induction potential of enzalutamide may not occur until approximately 1 month after the start of treatment, when steady-state plasma concentrations of enzalutamide are reached, although some induction effects may be apparent earlier. Monitoring should continue for at least the first month of treatment and dose adjustments considered. Given the long half-life of enzalutamide (5.8 days), effects on enzymes may persist for one month or longer after stopping enzalutamide. A gradual dose reduction of the concomitant medicinal product may be necessary when stopping enzalutamide treatment.

6.11.4.F Enzalutamide Special Warnings and Precautions For Use

History of seizures

Caution should be used in administering enzalutamide to patients with a history of seizures or other predisposing factors including, but not limited to, underlying brain injury, stroke, primary brain tumors or brain metastases or alcoholism. In addition, the risk of seizure may be increased in patients receiving concomitant medications that may lower the seizure threshold. Enzalutamide should be permanently discontinued in patients who have a seizure while on treatment.

Hepatic impairment

A hepatic impairment study showed that the composite AUC of enzalutamide plus N-desmethyl enzalutamide after administration of a single dose of enzalutamide was similar in patients with baseline mild, moderate or severe hepatic impairment (Child-Pugh Class A, B or C respectively) relative to patients with normal hepatic function, and no starting dose adjustment is needed.

Renal impairment

Based on population pharmacokinetics modeling the factors of age, weight and renal function (Creatinine clearance ≥ 30 mL/min) do not have clinically meaningful effects on enzalutamide exposures; therefore, no dose adjustments are needed.. Clinical data are insufficient to assess the

potential effect of severe renal impairment (Creatinine clearance < 30 mL/min) and end-stage renal disease on enzalutamide pharmacokinetics.

6.11.4.G Enzalutamide Overdose

There is no antidote for enzalutamide. In the setting of an overdose, stop treatment with enzalutamide and initiate general supportive measures taking into consideration the t1/2 of 5.8 days. Patients may be at increased risk of seizures following an overdose.

6.11.4.H Management of specific toxicities due to abiraterone and enzalutamide

The safety monitoring and toxicity management plan described below takes into account AEs based on the reported clinical safety data of abiraterone and enzalutamide given separately. There are limited reported data on the safety and toxicity of the combination of enzalutamide and abiraterone however the recommendations summarised here have been updated in light of the experience gained in STAMPEDE as recommended by the STAMPEDE TMG.

Seizures

If any patient suffers a seizure whilst on treatment, enzalutamide should be discontinued immediately. Abiraterone and prednisolone can be continued providing there are no abiraterone-specific toxicities.

Table 4: Management of Abnormal Liver Function Tests (LFTs)

TOXICITY EVENT	ACTION
**Grade 1** increases in AST, ALT or bilirubin<br>(e.g. increase in AST or ALT from ULN to 2.5X ULN; increase in total bilirubin from ULN to 1.5X ULN)	The frequency of LFT monitoring should be increased to at least weekly, if the investigator judges that the laboratory abnormalities are potentially related to study medication. No dose reduction is required.
**Grade 2** increases in AST, ALT or bilirubin<br>(e.g. increase in AST or ALT to >2.5-5X ULN; increase in total bilirubin from >1.5-3X ULN)	Withhold abiraterone, enzalutamide and all other concomitant medications that are potentially hepatotoxic. The frequency of LFT monitoring should be increased to at least weekly until the liver function tests return to baseline value or grade 1 when all trial medication can be re-started. No dose reduction is required after one episode providing this resolved within 4 weeks but should be considered if Grade 2 derangements recurs.
**Grade 3** increases in AST, ALT or bilirubin<br>(e.g. increase in AST or ALT to >5X ULN; increase in total bilirubin to >3X ULN),	Withhold abiraterone and enzalutamide and all other concomitant medications that are potentially hepatotoxic. At least weekly monitoring is required until the LFTs return to baseline value or grade 1. Enzaluatmide can be re-started with no dose reduction. See below for abiraterone re-challange.
**Grade 4** increases in AST, ALT or bilirubin<br>(e.g. increase in AST or ALT to >20x ULN; increase in total bilirubin to >10x ULN)	Patients must discontinue abiraterone and enzalutamide immediately. At least weekly monitoring is required until the LFTs return to baseline value or grade 1 and then prednisone can be discontinued and the investigator can consider restarting enzalutamide. Abiraterone should not be re-introduced.
**RE-CHALLENGE AFTER GRADE 3 TOXICITY**	**ACTION**
If study treatment resumption is considered for patients who have experienced Grade 3 increases in AST, ALT, or bilirubin	Resume study treatment with abiraterone dose reduction to 750mg when grade 3 toxicities resolve to grade 1 or baseline.
If Grade 3 or higher increases in AST, ALT or bilirubin recur after the first dose reduction	Hold study medication and all other concomitant medications that are potentially hepatotoxic. At least weekly LFT monitoring is required, starting immediately regardless of study schedule and continued until a return to baseline values or Grade 1.
If study treatment resumption is considered for patients who have experienced Grade 3 increases in AST, ALT, or bilirubin with the first dose reduction	Resume study treatment with abiraterone dose reduction to 500mg when AST, ALT or bilirubin returns to baseline value or Grade 1.

An opinion from a hepatologist should be considered if there are any concerns or liver function derangement shows no improvement within 2 weeks of discontinuation of abiraterone.

Table 5: Management of hypertension

TOXICITY EVENT	ACTION
Grade 1-2	Management as per investigator with anti-hypertensive treatment and increase frequency of blood pressure monitoring to at least weekly. Follow local guidance for selection of anti-hypertensives but avoid thiazide diuretics to minimise risk of serum potassium derrangment. Calcium channel antagonists of beta blockers are often preferred.<br><br>As with other symptoms of minerlocorticoid excess, consider increasing prednisolone dose to 5mg BID.
Grade 3-4	Withhold abiraterone and enzalutamide. Adjust or add anti-hypertensive medications to mitigate the toxicity. When hypertension resolves to Grade ≤1, resume both enzalutamide and abiraterone at full dose with prednisolone 5mg bid.

An opinion from a cardiologist should be considered if blood pressure control is not achieved within 4 weeks.

Table 6: Management of hypokalaemia

TOXICITY EVENT	ACTION
Grade 1*<br>(LLN- 3.0nM)	Supplement with oral potassium and monitor closely and increase prednisolone dose to 5mg BID.<br>Exclude and manage other causes of hypokalemia.
Grade 3<br>(<3.0mM–2.5mM)<br><br>or life-threatening<br>Grade 4 (<2.5mM)	Abiraterone will be permenently discontinued and the patients will be hospitalized for intravenous potassium replacement and cardiac monitoring. After the return of serum potassium to normal, prednisolone will be discontinued. The patient can continue on enzaluatamide alone. If hypokalaemia persists consider a dose reduction of enzalutamide to 120mg once a day.

*No Grade 2 definition in CTCAE v3.0

Table 7: Management of fluid retention/oedema

TOXICITY EVENT	ACTION
Grade 1-2	Increase prednisolone dose to 5mg bid.
Grade 3-4	Withhold abiraterone. Consider addition of mineralocorticoid receptor antagonist eplerenone until resolution of symptoms. Enzalutamide can be continued. When fluid retention/oedema returns to baseline or resolves to ≤Grade 1, resume abiraterone at full dose with prednisone 5mg bid, if symptoms do not resolve abiraterone should not be re-started and enzalutamide should be dose reduced to 120 mg per day.

Table 8: Management of diarrhoea

Toxicity event	Action
Grade 1-2	Symptomatic management.
Grade 3-4	Withhold abiraterone. If no improvement reduce dose of enzalutamide to 120 mg per day. Once resolved to Grade 1, recommence abiraterone at 750 mg per day.

Table 9: Management of arthralgia & muscle Pain

Toxicity event	Action
Grade 1-2	Symptomatic management.
Grade 3-4	Reduce dose of enzalutamide to 120 mg per day

Table 10: Management of fatigue

Toxicity event	Action
Grade 1-2	No change in treatment
Grade 3-4	Patients who experience a grade 3 or higher toxicity that is attributed to enzalutamide and cannot be ameliorated by the use of adequate medical intervention may interrupt treatment with enzalutamide for 1 week or until the toxicity grade improves to grade 2 or lower severity. Subsequently, study drug dosing may be re-started at the original dose (160 mg/day) or at a reduced dose (120mg/day or 80mg/day) in consultation with the study team.

6.11.4.I Management of Specific Toxicities from Prednisolone

Prednisolone/prednisone will be started at 5mg once daily, to prevent secondary mineralocorticoid excess.

Prednisolone/prednisone dose increase of up to 10mg/day is permitted to manage mineralocorticoid-related toxicities (e.g., hypokalaemia, hypertension) which are refractory to standard management.

Patients experiencing serious symptoms of cushings syndrome (e.g., weight gain, muscle loss) can decrease or discontinue (temporarily or permanently) steroids at the investigator’s discretion. It should be noted that weight gain and muscle loss are also associated with ADT.

6.12 CONCOMITANT MEDICATIONS AND DRUG INTERACTION

6.12.1 Abiraterone: Interaction with other medicinal products and other forms of interaction

Details on drug interactions are described in **Appendix G.** The table below provides a summary on the main interactions.

Anti-androgens

Abiraterone is steroid synthesis inhibitor and should not be given together with any other anti-androgens given the risk of toxicity. Cyproterone acetate should be discontinued 10 days and

finasteride stopped 48 hours before commencing enzalutamide. Concommitent use of dutasteride, bicalutamide and flutamide are all contraindicated.

Table 11: Drugs which may interact with Abiraterone

DRUGS WHICH MAY INCREASE ABIRATERONE LEVELS			
**Substrate**	**Clinical Use**	**Drug**	**Recommendation**
CYP3A4 inhibitors	Macrolide antibiotics	Clarithromycin	Avoid or hold abiraterone if short term use unavoidable given increased risk of abiraterone toxicity
	Anti-fungals	Ketoconazole<br>Itraconazole<br>Voriconazole	Avoid or hold abiraterone if short term use unavoidable given increased risk of abiraterone toxicity
**DRUGS WHICH MAY REDUCE ABIRATERONE LEVELS**			
**Substrate**	**Clinical Use**	**Drug**	**Recommendation**
CYP3A4	Anti-epileptics*	Phenytoin<br>Carbamazepine<br>Phenobarbital<br>Primadone	Contraindicated
	Anti-depressants	St Johns Wart	Contraindicated
	Anti-TB	Rifampicin<br>Rifabutin	Contraindicated
	Anti-retroviral	Atazanavir<br>Saquinavir<br>Ritonavir<br>Indinavir<br>Nelfanavir	Contraindicated
**DRUGS WHICH MAY ACCUMULATE WHEN GIVEN WITH ABIRATERONE**			
**Substrate**	**Clinical Use**	**Drug**	**Recommendation**
CYP2D6	Cardiac	Metoprolol<br>Propranolol<br>Propafenone<br>Flecanide	Monitoring required as drug levels may increase with abiraterone use
	Anti-depressants	Desipramine<br>Venlafaxine<br>Citalopram	Monitoring required as drug levels may increase with abiraterone use
	Anti-psychotics	Haloperidol<br>Risperidone	Monitoring required as drug levels may increase with abiraterone use
	Analgesia	Tramadol<br>Codeine<br>Oxycodone	Monitoring required as drug levels may increase with abiraterone use
	Alpha blockers	Tamsulosin	Monitoring required as drug levels may increase with abiraterone use
	Anti-diabetic	Repaglinide	Monitoring required as drug levels may increase with abiraterone use

*Please note that any hisotry of epilepsy is an exclusion criteria.

6.12.2 ENZALUTAMIDE: INTERACTION WITH OTHER MEDICINAL PRODUCTS AND OTHER FORMS OF INTERACTION

Details on drug interactions are described in **Appendix G.** The table below provides a summary on the main interactions

Anti-androgens

Enzalutamide is potent androgen receptor antagonist and should not be given together with any

other anti-androgens given the risk of toxicity. Cyproterone acetate should be discontinued 10 days and finasteride stopped 48 hours before commencing enzalutamide. Concommitent use of dutasteride, bicalutamide and flutamide are all contraindicated.

Table 12: Drugs which may interact with Enzalutamide

**DRUGS WHICH MAY INCREASE ENZALUTAMIDE LEVELS**			
**Substrate**	**Clinical Use**	**Drug**	**Recommendation**
CYP2C8 inhibitors	Lipid-lowering	Gemfibrozil	Avoid, if no alternatives, reduce enzalutamide dose to 80mg
**DRUGS WHICH MAY DECREASE ENZALUTAMIDE LEVELS**			
**Substrate**	**Clinical Use**	**Drug**	**Recommendation**
CYP2C8 inducers	Anti-TB	Rifampicin<br>Rifabutin	Avoid and switch to an alternative if possible
CYP3A4 inducers	Anti-epileptics	Phenytoin<br>Carbamazepine<br>Phenobarbital	Contraindicated
	Anti-depressant	St Johns Wart	Contraindicated
	Anti-retrovirals		Contraindicated
**ENZALUTAMIDE MAY REDUCE DRUG LEVELS**			
**Substrate**	**Clinical Use**	**Drug**	**Recommendation**
CYP2C19	Gastric protection	Omeprazole	Omeprazole AUC reduced by 70% Consider increasing dose of omeprazole for same therapeutic effect
CYP3A4	Analgesia	Fentanyl*<br>Alfentanil*<br>Tramadol	Monitor closely and consider alteratives
	Immunosuppressants	Sirolimus*<br>Tacrolimus*<br>Cyclosporine*	Monitor closely
	Anti-migraine	Ergotamine	Monitor closely
	Cardiac	Nifedipine<br>Ivabradine	Monitor closely, consider alternatives
CYP2C9	Anti-epileptics	Phenytoin*	Contraindicated
	Anti-coagulants	Warfarin*	Warfarin AUC reduced by 56% Consider switching to low molecular heparin, increase INR monitoring if this is not possible
**DRUGS WHICH MAY ACCUMULATE WHEN GIVEN WITH ENZALUTAMIDE**			
**Substrate**	**Clinical Use**	**Drug**	**Recommendation**
p-gp		Colchicine*<br>Dabigatran*<br>Digoxin*	Monitor closely

*narrow therapeutic index

6.13 TRIAL PRODUCTS

Details of the procedures for obtaining the drugs within the trial, dispensing and disposal of unused drug are given in **Appendix E**. Arrangements for free or discounted drugs are given in the Finance section (**Section 15**).

6.14 MEASURES OF COMPLIANCE/ADHERENCE

Date of treatment, dose, delays and reasons for delays or dose modifications of all trial treatments will be recorded. The estimated number of abiraterone tablets and enzalutamide capsules taken in a given time period will also be recorded as well as any dose reductions.

6.15 TREATMENT DATA COLLECTION

Data will be recorded on case report forms (CRFs); the top copy/original should be sent to the MRC CTU for data entry and a copy kept at the local centre. Up-to-date versions of all CRFs can be found on the trial website (http://www.stampedetrial.org/) and centres will be notified of any changes throughout the course of the trial. The type of data to be recorded is detailed in the Assessments and Procedures section (**Section 7**).

6.16 ADMINISTRATION OF STANDARD RADIOTHERAPY TO NON-METASTATIC PATIENTS

6.16.1 Treatment Details

Standard radiotherapy will be given to appropriate patients in each of the trial arms, following a period of neo-adjuvant ADT therapy, as is generally standard in UK practice. For patients with negative nodes on axial imaging, clinicians may choose between irradiating prostate and seminal vesicles alone or including the pelvic nodes in addition. Additional staging tests such as pelvic node sampling may be considered in making this decision. Conformal or intensity modulated radiotherapy should be used in all patients. Where patients have good clinical evidence that nodes are free of tumour or patients for whom nodal radiotherapy is contra-indicated (e.g. significant bowel disease), treatment may be given to the prostate gland and seminal vesicles only. The recommended dose is 74Gy in 37 fractions to the prostate and seminal vesicles or the equivalent using hypo-fractionated schedules. These recommendations are summarised in **Figure 5**. Alternative dosing schedules are permitted but must be agreed with the STAMPEDE Trial Management Group.

6.16.1.A Standard-of-care RT Timing in M0 patients

Radiotherapy should be given around 6 to 9 months after randomisation in all trial arms and, if receiving docetaxel as part the standard-of-care (permitted from Protocol 14.0), the patient must have sufficiently recovered from any docetaxel toxicity before RT can begin.

6.16.1.B Type Of standard-of-care RT in M0 patients

Conformal or intensity modulated radiotherapy.

6.16.1.C Standard Clinical Target Volume in M0 patients

- **CTV1:** Prostate plus seminal vesicles
- **CTV2:** (Node positive patients) Regional lymph nodes to include internal iliac and the inferior part of the common iliac nodes as used in EORTC trial 22961 (32)
- **PTV1:** CTV1 plus 10-15 mm according to local practice

- **PTV2:** CTV2 plus 10-20mm according to local practice

6.16.1.D Standard-of-care RT Dose in M0 patients

Prostate dose of 74Gy in 2Gy fractions or equivalent, with optional dose to the pelvic nodes of 55-60Gy in 2Gy fractions or equivalent using IMRT to deliver the treatment over 37 fractions, suggested dose is 55Gy in 37 fractions with IMRT in line with CHHIP trial. Higher doses may be considered if the department is experienced in using IMRT for nodal radiotherapy, particularly as data emerges from the PIVOTAL trial of nodal IMRT in high-risk node negative patients where a nodal dose of 60Gy in 37 fractions is being evaluated. Alternative schedules should be agreed with the STAMPEDE Trial Management Group.

Figure 5: Diagram for deciding recommended approach to standard-of-care (M0) RT in non-metastatic patients

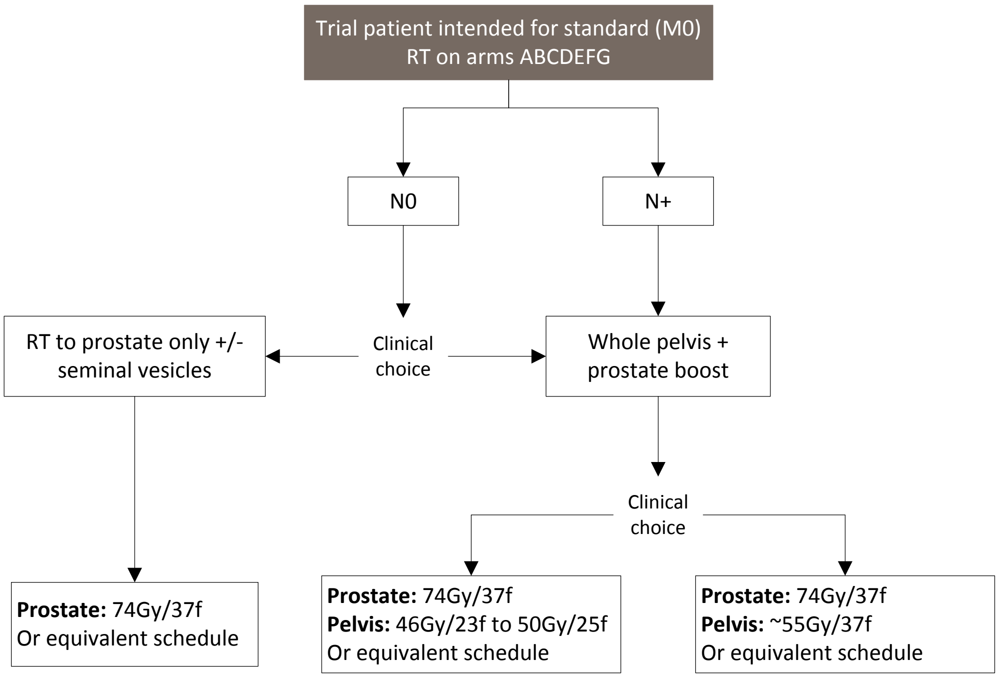

6.17 NON-TRIAL TREATMENT

6.17.1 Medications Permitted

Guidance on concomitant medications and drug interaction is detailed in **Section 6.12**.

6.17.2 Data On Concomitant Medication

All concomitant medication will be recorded on the baseline form prior to randomisation and on any subsequent Serious Adverse Event forms. This should include aspirin that may be taken on a regular basis for cardiovascular disease, the use of any Non-Steroidal Anti-inflammatory Drugs (NSAID) as well as any vitamin or mineral supplements the patient is taking.

Please see **Section 6.12** for further details on drug interactions for abiraterone and enzalutamide.

7 ASSESSMENTS AND PROCEDURES

7.1 SCHEDULE FOR ASSESSMENTS

A detailed follow-up schedule is given in **Table 13, 14 and 15**.

7.1.1 PSA Measurements

All patients should have PSA measured pre-androgen deprivation therapy and at weeks 6, 12, 18 and 24 and every 12 weeks, thereafter, up to 2 years post randomisation. Following this, PSA should be measured every 6 months until 5 years and annually, thereafter. For patients who do not have a scheduled hospital visit, it would be acceptable for arrangements to be made for blood samples to be drawn in a GP's surgery.

7.1.2 Assessment Of Treatment Failure (Definition Of Progression)

It is not proposed to routinely assess patients for response. However, in order that objective progression can be assessed, it is necessary to have imaging taken at time of best response as judged by the treating clinician. All patients should have baseline radiological examinations as detailed in **Section 4.4.1**. In addition it is recommended that all patients should have scans or X-rays repeated at 24 weeks (and whenever clinically appropriate) if they were abnormal at baseline, particularly if they have a low PSA value on entry in to the trial making biochemical assessment of treatment failure difficult. The following events should be reported on a progression form & additional treatment form:

- Biochemical failure – must be reported alongside castrate levels of testosterone if the patient has received intermittent ADT (see **Appendix J**).
- Local progression
- Lymph node progression
- Progression in distant metastases
- Development of new metastases
- Skeletal-related events

Please note that skeletal-related events (SREs) may be indicative of disease progression but can have other causes such as osteoporotic fracture. All SREs should be investigated further to establish whether or not the patient has progressed, in which case a progression form should be completed. All SREs are reported on the Progression and Additional Treatment CRF to confirm whether this represents disease progression.

7.1.3 Additional Safety Assessment

Due to the risk of liver toxicity and secondary hyperaldosteronism with abiraterone, patients will require 2 weekly U+Es, LFTs and blood pressure measurement for the first 12 weeks. It is not necessary to report these unless abnormal; in this instance, they should be reported as AEs (on the next Follow-up CRFs) and as SAEs (see **Section 11**) if appropriate.

Medical review and PSA measurements follow the same pattern in the control arm: visits at weeks 6, 12, 18 and 24 and every 12 weeks, thereafter, up to 2 years post randomisation. Following this, PSA should be measured every 6 months until 5 years and annually, thereafter. For patients who do not have a scheduled hospital visit, it would be acceptable for arrangements to be made for blood samples to be drawn either in a GP's surgery or in the patient's home.

7.1.4 DATA COLLECTION FOR STANDARD DOCETAXEL

The decision to use docetaxel as part of the standard-of-care must be made before randomisation and should be recorded on the randomisation CRF. The date of the first cycle should be recorded at the time of randomisation; this can be a planned date when randomisation occurs prior to docetaxel commencing but must be within 12 weeks of starting ADT (see **Section 4.4.3**). All further details should be recorded on the standard-of-care docetaxel CRF.

If a patient does not receive the planned docetaxel this must also be recorded on the standard docetaxel CRF together with the reason why.

7.1.5. DATA COLLECTION AND NON-ADMINISTRATION OF STANDARD RADIOTHERAPY

There are CRFs to be completed for patients receiving primary radiotherapy whether this is standard radiotherapy for M0 patients on any arm or prostate radiotherapy for Arm H patients. All radiotherapy and acute side effects details will be recorded on the Radiotherapy Detail and Acute Toxicity Forms; any late side effects will be recorded on the follow up form.

If it is decided not to give the planned radiotherapy (for example, due to early metastatic progression or patient refusal), this should be stated on the Radiotherapy Detail form together with the reason for non-administration of the treatment.

7.1.6. DATA COLLECTION PALLIATIVE RADIOTHERAPY

For patients who receive palliative radiotherapy as part of first line treatment, a Palliative Radiotherapy CRF should be completed.

The progression and additional treatment CRF is used to record details of any further palliative radiotherapy given at progression. This includes palliative RT for SREs e.g. bone pain and spinal cord compression, as well as salvage RT to the prostate. For SRE's an additional assessment is required to determine if this represents progression.

7.1.7 DATA COLLECTION RESEARCH (M1) RADIOTHERAPY

All RT data should be reported on the RT Detail CRF; acute toxicity data should be reported once the primary course of RT has been completed. Adverse events such, TURPs, SREs should be reported on the FU form.

7.1.8. FOLLOW-UP SCHEDULES

An individualised form with a follow-up schedule will be provided for each randomised patient. For patients who are receiving LHRH analogues, it is assumed that any additional treatment will commence within two weeks of randomisation. For patients who are due to have an orchidectomy it is recognised that surgery will have to be scheduled and the scheduling of any additional treatments may be affected by post-operative recovery. It is recommended that all patients who had abnormal radiological investigations at baseline or present with a low PSA on entry into the STAMPEDE trial should have radiological investigations repeated 24 weeks after randomisation.

7.2 FOLLOW-UP

Every effort should be made to follow-up all patients who have been randomised. Patients should, if possible, remain under the care of an oncologist or urologist for the duration of the trial. If care of a patient is returned to the GP, it is the responsibility of the consultant who obtained the patient’s

consent to participate in the trial to ensure that all relevant data collection forms are completed. If the patient moves from the local area, arrangements should be made for trial follow-up to be undertaken by their new local centre. Details of other participating centres can be obtained from the MRC CTU. The consent of patients should be obtained for their names to be flagged for survival information through national registries, for example NHS Information Centre/Office of National Statistics (ONS) in England/Wales and General Register Office in Scotland, Hospital Episode Statistics (HES). If the clinician moves, appropriate arrangements should be made to arrange for trial follow-up to continue at the centre.

7.3 TRIAL CLOSURE

For the purpose of complying with UK Clinical Regulations introduced on May 2004, the trial will be considered 'closed' when the follow-up point for the primary analysis of the final comparison has been reached. However, further observational follow-up of all patients enrolled in the trial will continue until all randomised patients have died. This will initially be via the hospital, but in the longer term may employ national registers.

Table 13: Summary of timing of case report forms

Case Report Forms	Timing Of Assessment and CRF
**Baseline**	
Bone Density Risk Factor	At randomisation
Randomisation	At randomisation
Baseline	At randomisation
Cardiovascular Assessment	At randomisation
Pathology	At randomisation. When pathology sample has been taken and sent to Sponsor's designated laboratory.
**Treatment**	
Standard-of-care docetaxel	Complete for all patients 20 weeks after randomisation
Hormone Therapy	Form to be sent with corresponding follow-up form if there is a change in hormone therapy to report
Abiraterone and Enzalutamide Treatment	Treatments administered daily; form to be sent at week 6 and with corresponding follow-up form if there is a change in treatment to report
RT detail	• When standard-of-care radiotherapy is completed or if planned RT is no longer to be given<br>• Arm H when research RT completed<br>• Arm A (M1) at 3 months<br>Once primary RT is completed for Arm H patients or those receiving RT as standard-of-care<br>For patients who did not receive primary RT (since Protocol 9.0 regardless of being planned) this should be completed 10 months after randomisation or 3 months for newly-diagnosed M1 patients to confirm RT was not given
RT Acute Toxicity	For all patients who receive primary RT.
**Assessments**	
Follow-Up	Every 6 weeks for 6 months, then every 12 weeks until 2 years, then every 6 months until 5 years and annually thereafter. (See **Table 7** for more information.)
Palliative Radiotherapy	If applicable, when a palliative radiotherapy course is completed.
End of Treatment	When each treatment is completed (either at end of scheduled treatment or at early cessation of treatment).
Progression & Additional Treatment	At the first occurrence of each type of progression, including skeletal-related events and whenever a patient that has progressed receives additional treatment for progression. All SREs requiring additional treatment should be recorded on this form and an assessment made as to whether this constitutes progression.
Additional Treatment Update	Whenever a patient who has previously progressed received additional treatment but has not experienced a new type of progression
Serious Adverse Event	Following any Serious Adverse Event
Death	At Death
**Administration**	
Patient Transfer	When a patient is transferred to a different hospital for the administration of trial treatment and follow up
Co-enrolment	When a patient is co-enrolled in any other clinical trial. Please see **Section 5.1** for more information

Table 14: Data required on follow-up forms

Timing of follow-up	PSA	Evidence of Progression	Androgen deprivation therapy	treatment	Unscheduled Visits	Toxicities
Follow-up Form	✓	✓	✓	✓	✓	✓
Follow-up Form (Post-Progression)	✓	✓	✓	x	✓	X*

* Toxicity information will be collected for Arm G and J patients if progression has occurred but trial treatment continues

Table 15: Schedule for completion of treatment and outcome forms by arm.

Timing From Randomisation			Treatment Forms		Outcome forms	
Years	Months	Weeks	Abi and/or Enza	RT	Follow-up[Ψ]	QL + HE[¥]
**6-Weekly**						
-	-	6	G, J	-	All arms	All arms
-	-	12	G, J	M1: A,H	All arms	All arms
-	-	18	G, J	-	All arms	All arms
-	6	24	G, J	-	All arms	All arms
**12-Weekly**						
-	9	36	G, J	-	All arms	All arms
1	12	48	G, J	M0: A,B,C,E,G, J	All arms	All arms
-	15	60	G, J	-	All arms	All arms
-	18	72	G, J	-	All arms	All arms
-	21	84	G, J	-	All arms	All arms
-	-	96	G, J	-	All arms	All arms
**6-Monthly**						
2	24	104	G, J	-	All arms	All arms
	30	130	G, J	-	All arms	All arms
3	36	156	G, J	-	All arms	All arms
	42	182	G, J	-	All arms	All arms
4	48	208	G, J	-	All arms	All arms
	54	234	G, J	-	All arms	All arms
5	60	260	G, J	-	All arms	All arms
**Annual**						
6	72	-	G, J	-	All arms	All arms
7	84	-	G, J	-	All arms	All arms
Etc	-	-	G, J	-	All arms	All arms

Key:

A = SOC
B = SOC + zoledronic acid
C = SOC + docetaxel
D = SOC + celecoxib
E = SOC + zoledronic acid + docetaxel
F = SOC + zoledronic acid + celecoxib
G = SOC + abiraterone
H = SOC + M1 research RT to the prostate
J = SOC + enzalutamide + abiraterone

Notes:

Ψ See Table 6 for information required at follow-up
† Form records data for two cycles
‡ Form records data for three cycles
¥ 1st 700 patients and those recruited from protocol version 8.0 onwards only

Note: Radiotherapy Detail & Acute Toxicity, Late RT Toxicity, HT and Abiraterone & Enzalutamide Treatment, Palliative Radiotherapy Progression, SAE, End of Treatment, Co-enrolment and Death forms to be completed as required.

Note: Docetaxel forms are no longer shown on the table as all patients will have completed treatment with docetaxel

Note: Recruitment was stopped to Arms D and F in April 2011 and completed to Arms B, C and E in March 2013; Arm G in January 2014

8 STOPPING OF TREATMENT OR FOLLOW -UP

Patients should be given every encouragement to adhere to their allocated protocol treatment and follow-up schedule, in order to reduce bias. However, a patient has the right to withdraw consent for participation in any aspect of this trial at any time.

8.1 STOPPING RESEARCH INTERVENTIONS

A patient may stop trial treatment for the following reasons:

- Progression whilst on therapy (trial treatment must be discontinued in this instance). For patients randomised to Arm G or J, please refer to **Section 6.8** for criteria to stop treatment
- Unacceptable toxicity
- Intercurrent illness which prevents further treatment
- Withdrawal of consent for treatment
- Any alteration in the patient's condition which justifies the discontinuation of treatment in the clinician's opinion
- Intention to commence a new anti-cancer treatment due to evidence of relapse.

The reason should be recorded on the relevant treatment and the End of Treatment form. In the case of abiraterone or enzalutamide and abiraterone, the disease event for stopping treatment may be after the first reportable failure-free survival event (see **Section 6.8**). In the event of PSA progression whilst on standard docetaxel, allocated trial treatment may be still initiated and continued until evidence of radiological and/or clinical progression. If a patient commences trial treatment having already progressed (biochemical and/or radiological) this should be recorded on a progression form but trial treatment should start and continue whilst there is perceived evidence of benefit as judged by the local investigator.

Unless a patient states otherwise, consent is assumed for continued recording trial data.

8.2 PATIENT TRANSFERS

For patients moving from the area, every effort should be made for the patient to be followed-up at another participating trial centre and for this trial centre to take over responsibility for the patient. To document the transfer process the main contact person at both the current and receiving hospitals should complete and sign the Patient Transfer Confirmation form. A fully completed form must be returned to the CTU prior to the patient transfer and ideally any outstanding data queries for the patient should be completed prior to transfer.

On receipt of the completed transfer form a member of the STAMPEDE team will confirm the database has been updated and request confirmation of the name of the patient's new Clinician. Photocopies of the following documents may then be sent to the new hospital to complete the transfer and copies must be also retained at the original site for monitoring purposes:

- Consent form
- Completed CRFs

- Any documentation relating to the patient's participation in STAMPEDE (patient names must be removed from any documentation).

8.3 EARLY CESSATION OF TRIAL PARTICIPATION

If a patient explicitly withdraws consent to have any further trial data recorded their decision must be respected and the MRC CTU must be informed in writing. All communication surrounding the early cessation of trial participation should be noted in the patient's records. Please note data prior to this decision will still be required.

In the majority of cases, patients give permission for their data and information on their health to continue to be collected via clinical notes and national registries. Any information on the follow-up status, however minimal, would be helpful.

Early stopping of follow-up should not be undertaken lightly and the site must consider the implications for the trial and the patient in reaching such a decision. Without long-term data, the efficacy of trial treatments would be less reliable and could lead to inconclusive results. The early stopping of trial treatment should not lead to the early cessation of trial participation and in such cases follow-up assessments should be continued as per trial protocol.

Patients can change their minds about withdrawal at any time and re-consent to participate in the trial. Follow-up data should be collected only from the point of when consent was re-instated.

9 STATISTICAL CONSIDERATIONS

9.1 METHOD OF RANDOMISATION

Patients will be randomised centrally using a computerised algorithm developed and maintained by the MRC CTU. Randomisation will be performed using the method of minimisation over a number of clinically important stratification factors with an additional random element. To decrease determinability, the factors are not listed here but can be found in the Statistical Design Document.

Table 8 shows the allocation weighting for each arm by protocol version. The relative weighting within each pairwise comparison remains constant throughout.

9.1.1 VERSION 7

From the outset, the trial had 1 control arm (A) and 5 research arms (B, C, D, E and F).

As the control arm is the comparator arm for all the research arms, twice as many patients were recruited to the control arm as to each of the original research arms as this is an efficient design where there are multiple comparisons to be made. Therefore, the initial randomisation ratio will be 2A:1B:1C:1D:1E:1F. From version 7.0, accrual to the celecoxib-containing arms was halted and the allocation ratio was 2A:1B:1C:0D:1E:0F.

9.1.2 VERSION 8

From version 8.0, an additional research arm (G) was introduced. The allocation weighting for the additional Arm G is 2, meaning that as many patients are contemporaneously randomised to Arm G as the control Arm A: the randomisation ratio is 2:2 (equivalent to 1:1, control:abiraterone). This gave an overall allocation ratio of 2A:1B:1C:0D:1E:0F:2G. When recruitment has been completed to the ongoing original research Arms B, C and E (which will be around 2 years before completion of accrual to arm G), the allocation ratio will be 2A:0B:0C:0D:0E:0F:2G (or 2A:2G). This is more efficient for this comparison than the 2:1 allocation ratio employed for the original research arms because of the minimal co-recruitment period.

9.1.3 VERSION 9

Version 9.0 introduced a "M1|RT comparison" for men with newly-diagnosed metastatic disease which is irrelevant to a subset of men joining STAMPEDE. This could only be achieved by splitting the randomisation system so that newly-diagnosed patients with M1 disease, no planned RT and no contraindication to RT are randomised 2A:1B:1C:0D:1E:0F:2G:2H and other men are randomised 2A:1B:1C:0D:1E:0F:2G:0H. Note that the allocation ratio for each pairwise comparison in unaffected, only the rate at which comparisons accrue.

9.1.4 VERSION 10 AND 11

Version 10.0 followed the successful completion of recruitment to Arms B, C and E. Therefore, the allocation ratio will be 2A:0B:0C:0D:0E:0F:2G (or 2A:2G) for M0 patients and A2:B0:C0:E0:D0:F0:G2:H2 for M1 radiotherapy arm patients (2A:2G:2H).The equal allocation ratio is suitable with fewer research arms open.

Version 11.0 then followed the successful completion of recruitment to Arm G, therefore for a short period of time STAMPEDE was only open to recruitment of newly-diagnosed M1 patients eligible for the "M1|RT comparison". Therefore the allocation ratio was A:H.

9.1.5 VERSION 12

Version 12.0 introduces a further allocation, Arm J: HT + abiraterone + enzalutamide. This allocation will be available to all patients. Accounting for Arm H still recruiting, this can only be achieved by keeping the randomisation system split so that newly-diagnosed patients with M1 disease and no contraindication to RT will be randomised 2A:0B:0C:0D:0E:0F:0G:2H:2J and other men will be randomised 2A:0B:0C:0D:0E:0F:0G:0H:2J. This can be simplified to equal allocation in these groups to A:H:J and A:J respectively.

Table 16: Allocation to each arm by protocol version

PROTOCOL VERSION	NEWLY-DIAGNOSED M1 PATIENTS									OTHER PATIENTS								
	A	B	C	D	E	F	G	H	J	A	B	C	D	E	F	G	H	J
V1	2	1	1	1	1	1	-	-	-	2	1	1	1	1	1	-	-	-
V2	2	1	1	1	1	1	-	-	-	2	1	1	1	1	1	-	-	-
V3	2	1	1	1	1	1	-	-	-	2	1	1	1	1	1	-	-	-
V4	2	1	1	1	1	1	-	-	-	2	1	1	1	1	1	-	-	-
V5	2	1	1	1	1	1	-	-	-	2	1	1	1	1	1	-	-	-
V6	2	1	1	1	1	1	-	-	-	2	1	1	1	1	1	-	-	-
V7	2	1	1	*0*	1	*0*	-	-	-	2	1	1	*0*	1	*0*	-	-	-
V8	2	1	1	*0*	1	*0*	2	-	-	2	1	1	*0*	1	*0*	2	-	-
V9	2	1	1	*0*	1	*0*	2	2	-	2	1	1	*0*	1	*0*	2	0	-
V10	2	*0*	*0*	*0*	*0*	*0*	2	2	-	2	*0*	*0*	*0*	*0*	*0*	2	0	-
V11	2	*0*	*0*	*0*	*0*	*0*	*0*	2	-	2	*0*	*0*	*0*	*0*	*0*	*0*	0	-
V12	2	*0*	*0*	*0*	*0*	*0*	*0*	2	2	2	*0*	*0*	*0*	*0*	*0*	*0*	0	2
V13	2	*0*	*0*	*0*	*0*	*0*	*0*	2	2	2	*0*	*0*	*0*	*0*	*0*	*0*	0	2
V14	2	*0*	*0*	*0*	*0*	*0*	*0*	2	2	2	*0*	*0*	*0*	*0*	*0*	*0*	0	2

9.2 OUTCOME MEASURES

The overall, definitive primary outcome measure for the trial for each comparison is overall survival (all-cause mortality). The design of the trial is such that it is important to have additional intermediate outcome measures to assess activity in each research arm as the trial progresses. These are listed in **Table 17**. The intermediate primary outcome measure is failure-free survival. The reasons for different emphases in each recruitment stage are explained in **Section 9.3**.

Table 17: Trial Outcome Measures by Comparison Stage

TRIALS STAGE	PRIMARY OUTCOME MEASURES	SECONDARY OUTCOME MEASURES
Pilot phase	Safety*	Feasibility
Activity Stage (AS) I-III	Failure-free survival (FFS)†	Overall survival (OS)<br><br>Toxicity<br>Skeletal-related events
Efficacy Stage (ES) IV	Overall survival	Quality of life<br><br>Cost effectiveness<br>Failure-free survival†<br>Toxicity<br>Skeletal-related events

*Based on toxicity
†Including biochemical failure (see **Appendix J**)

9.3 SAMPLE SIZE: PRINCIPLES AND ASSUMPTIONS

The overall design for this study is a multi-arm multi-stage, multi-centre randomised controlled trial. There are a number of stages for each research arm: a Pilot Phase, several Activity Stages and a final Efficacy Stage. Full details of the methodology underlying the trial design are given by Royston et al. (33, 34) The sample size calculations were performed using the `stage2` (version 1.2.0, March 2002) and `stagen` (version 1.1.1, 18 May 2004) programs, both implemented in Stata (Stata Corp, TX) and updated using the later `nstage` program (version 1.0.3, 13-jun-2007; version 2.1.0, 28-jun-2009). (35)

The trial was designed under the assumptions in **Table 18**, and additionally, we assume a slightly higher proportion of non-metastatic than metastatic patients joining the trial such that median FFS is two years and median OS four years for the whole cohort.

Table 18: Hazard ratio assumptions under null and alternative hypotheses

SIZE OF HR	PILOT	AS I-III	ES IV
Under null hypothesis (H0)	n/a	HR(FFS) = 1.00	HR(OS) = 1.00
Under alternative hypothesis (H1)	n/a	HR(FFS) = 0.75	HR(OS) = 0.75

The HR of 0.75 for any research arm relative to control would translate into an absolute improvement in FFS of 10%, from approximately 50% to 60% at two years and OS of 10%, from approximately 50% to 60% at four years. A beneficial difference of this size would be clinically worthwhile and, indeed, experience tells us it may be unrealistic to expect a larger difference. Therefore, we have adequately powered the trial to detect a HR of 0.75 for overall survival. This design gives 95% power at Activity Stages I-III and 90% power at Efficacy Stage IV for each comparison. Further details of the sample size calculations are summarised in **Sections 9.4** and **9.5** and detailed in a separate Statistical Design Documents which are available on request.

Note that, from protocol version 8.0, standard-of-care RT was introduced to the majority of patients with N0 M0 disease. This is likely to improve the outcomes for this group. Further agents are starting to be licensed for patients with castrate-refractory disease which may also improve survival rates. Improved FFS rates would delay the intermediate analyses; improved survival rates would delay the definitive analyses. The Statistical Design Document includes models where median survival is estimated at 5, 6 and 7 years rather than just 3and 4 years. The trial is powered to detect a difference in relative improvement and the analyses will be performed when a pre-planned number of events has been reported in the control arm, rather than after a certain number of patients have been recruited or a certain amount of time elapsed. **Sections 9.4** and **9.5** provide more detail, including some variations on these assumptions.

Throughout recruitment to protocol version 12.0, at least, the proportion of metastatic men joining the trial has been fairly constant, at around 60%. From protocol version 9.0, we introduced an allocation, Arm H, only for men with (newly diagnosed) M1 disease. This means that further comparisons for the whole patient group will have proportionately fewer metastatic patients and, therefore, fewer events at any given moment in time. This will affect contemporaneously-recruiting comparisons, such as the "enzalutamide + abiraterone comparison" introduced in protocol version 12.0. Median survival may therefore be higher in that comparison, at around 7 years.

9.4 SAMPLE SIZE ISSUES AND TRIAL STAGES: ORIGINAL RESEARCH ARMS (B-F)

9.4.1 PILOT PHASE: ORIGINAL RESEARCH ARMS (B-F)

It was anticipated that 210 patients would be recruited to the Pilot Phase from a limited number of centres over a one year period. Approximately 60 patients would be randomised to the control arm and 30 patients to each of the five research arms, each of which were assessed for safety and feasibility. If recruitment proved unfeasible or any of the research arms proved unsafe or not feasible to administer (e.g., poorly tolerated or unexpected toxicity) recruitment to these arms would have been discontinued. There were already considerable safety data on the use of docetaxel and zoledronic acid in patients with malignancies including prostate cancer, and on the use of Cox-2 inhibitors (including celecoxib), although mainly from patients with musculoskeletal disorders. There were fewer data on the combination arms, but it was thought very unlikely that any of the research arms would be discontinued during the Pilot Phase. When 210 patients had been on the trial for a minimum of 18 weeks, the Independent Data Monitoring Committee (IDMC) reviewed the data from the Pilot Phase and continued to the trial during this period as equipoise remained. Recruitment continued beyond this point. Safety data are assessed throughout the trial.

9.4.2 ACTIVITY STAGES I-III: ORIGINAL RESEARCH ARMS (B-F)

In the sample size calculations, we assumed that all research arms successfully pass through the Pilot Phase to Activity Stage I and that patients would be recruited at a rate of approximately 500 per year. This was faster than in the Pilot Phase because the trial would recruit from additional centres, both in the UK and internationally. The analysis of Activity Stages I, II and III were planned for when around 113, 216 and 334 failure-free survival events had been observed in the control arm, respectively.

The Activity Stage analyses comprise pairwise comparisons of FFS between the control arm and each of the 5 research arms (i=B, C, D, E, F). Let HR_i(true) represent the hazard ratio (HR) of the ith research arm to the control arm, and HRi(observed) the observed value. Discontinuation of accrual of further patients was considered for the ith research regimen at each of Activity Stages I-III according to the guidelines in **Table 19**.

Table 19: Guidelines for stopping accrual to the original research arm

Activity Stage	Number of control arm events	Consider discontinuation if HR_I(observed) is…
I	~113	>1.00
II	~216	>0.92
III	~334	>0.89

9.4.3 Efficacy Stage IV: Original Research Arms (B-F)

The analysis of Efficacy Stage IV for the original research arms was planned for when around 403 deaths have been observed in the control arm. This gave 90% power to detect the targeted hazard ratio of 0.75 at one-sided significance level of 0.025. The actual timing of this analysis, balancing continued accrual with just follow-up, depended on the number of arms passing through to further recruitment from Activity Stages I-III and the observed accrual and event rates.

9.4.4 Sample Size For Original Research Arms (B-F)

Assuming an accrual rate of 500 patients/year, between 2,800 and 3,600 patients were planned to be entered into the original research comparisons of the trial over a period of between 5½ and 7 years. The exact number of patients entered depended on the observed accrual rate and the observed event rate, which was, in itself, dependent on the mix of patients joining the trial from the broad spectrum of eligibility. The primary analysis on overall survival required around 403 deaths to be observed on the control arm. Accrual continued until the main analysis could be foreseen so that the overall duration of the comparisons would be as short as possible (longer accrual facilitates this) and so that few, if any, patients remained on treatment when the main results are released. The statistical team have monitored and projected the analysis timelines using the `artpep` command in Stata. Results were presented in May-2015. Further information is available in the Statistical Master File.

9.5 SAMPLE SIZE ISSUES AND TRIAL STAGES: ADDITIONAL RESEARCH ARM G

9.5.1 Pilot Phase: Additional Research Arm G

A similar approach is being followed for the additional research Arm G, as detailed for the original research arms in Section 9.4.1. The IDMC reviewed safety data, in the context of data from the control arm, when the first 30 patients allocated to Arm G had been on trial for at least 18 weeks.

Furthermore, an additional review of safety was performed when 30 patients with newly-diagnosed non-metastatic disease allocated to Arm G had been on trial for at least 18 weeks. Both of these milestones were successfully completed.

9.5.2 Activity Stages I-III: Additional Research Arm G

The same principles are applied to the new comparison as to the previous comparisons. The notable difference will be in the accrual rate to this comparison which is anticipated to be higher. There are two reasons for this. First, STAMPEDE first started recruitment slowly in only a limited number of pilot sites. As more sites have been activated, including internationally, accrual has increased. At the time of version 8.0 of the protocol, monthly accrual to the study was averaging around 60 patients/month (over 700 patients/year). Second, there is an equal allocation ratio for the abiraterone arm compared to the control arm. It is this different allocation ratio which means that

the number of control arm events required to trigger the intermediate analyses is lower for the assessment of abiraterone to the assessment of the original research arms. This is shown in Table 20.

Table 20: Guidelines for stopping accrual to additional research Arms G and H

Activity Stage	Number of control arm events	Consider discontinuation if HR_G(observed) is...
I	~75	>1.00
II	~142	>0.92
III	~221	>0.89

9.5.3 Efficacy Stage IV: Additional Research Arm G

The analysis of Efficacy Stage IV for the additional comparison will be performed when around 267 deaths have been observed in the control arm. This will give 90% power to detect the targeted hazard ratio of 0.75 at a one-sided significance level of 0.025.

9.5.4 Sample Size For Additional Research Arm G

Up to around 1,800 patients will join the abiraterone comparison, with half allocated to the research arm. Consideration was given to ceasing further randomisations to Arm G if it was not showing sufficient evidence of activity at the interim analyses, just as was done for research Arms B to F.

The original plan intended for accrual to be halted either when 1,500 patients had been recruited or after 3 years, whichever was the sooner, providing the accrual rate remained above 50 patients/months.

The total number of patients joining this comparison depended not just on the same issues as the "original comparisons" (notably, observed accrual and event rates), but also the length of time that the original research arms co-recruited alongside this additional research arm; it was originally assumed that this would be for approximately 1 year, but it was closer to 1.5 years. The sample size calculations and projected durations are fairly robust to changes in the length of co-recruitment with the original research arms and future co-recruitment with any further research arms which the Trial Management Group may introduce. Many scenarios are detailed in the Statistical Design Document.

In Sep-2013, the target sample size for the "abiraterone comparison" was increased from around 1,500 patients to around 1,800 patients, with note that the efficacy analysis remains unchanged and is still to be triggered by around 267 control arm deaths. This increase in sample size was primarily because of an increase in the proportion of non-metastatic patients joining the comparison; this related to the activation of Arm H which only recruits patients with newly-diagnosed metastatic disease and thereby reduces the numbers of metastatic patients randomised to the "abiraterone comparison". Non-metastatic patients have a lower event rate than the metastatic patients and maintaining the same overall sample size would lead to a delay in time to the primary analysis. The increase in sample size was achievable because recruitment rates to the trial had been substantially higher than 50 patients/month for the preceding 6 months.

9.6 SAMPLE SIZE ISSUES AND TRIAL STAGES: ADDITIONAL RESEARCH ARM H

9.6.1 Pilot Phase: Additional Research Arm H

A similar approach will be followed for the additional research Arm H as detailed for the original research arms in **Section 9.4.1**. The IDMC reviewed safety data, in the context of data from the control arm, when the first 30 patients allocated to arm H had been on trial for around six months.

9.6.2 Activity Stages I-III: Additional Research Arm H

The same principles will be applied to the new comparison as to previous comparisons and an equal allocation ratio of control arm patients to patients allocated to Arm H will be employed, as for Arm G. The number of control arm events required to trigger the intermediate analyses will be the same as for the abiraterone comparison (see **Table 20**).

9.6.3 Efficacy Stage IV: Additional Research Arm H

The analysis of Efficacy Stage IV for the additional comparison will be performed when around 267 deaths have been observed in the control arm. This will give 90% power to detect the targeted hazard ratio of 0.75 at one-sided significance level of 0.025.

9.6.4 Sample Size For Additional Research Arm H

Consideration was given to ceasing further randomisations to Arm H if it was not showing sufficient evidence of activity on the intermediate primary outcome measure (FFS), just as for the other research arms. This research comparison is relevant to around 60% of patients joining STAMPEDE. At the point of the scientific approval, accrual was averaging around 80 patients per month to the trial. If accrual to the trial was slower at 70 patients per month, then accrual to this comparison could be between 18 and 42 patients per month, depending on which other trial arms are open to recruitment at the time.

We are targeting a 25% relative improvement in overall survival following local radiotherapy to the prostate in this patient group. This is the same size of effect targeted with the other research arms in STAMPEDE. This relative improvement can be further justified in the light of MRC PR07 which demonstrated an improvement of this magnitude for adding radiotherapy to ADT in locally advanced disease, with a hazard ratio for overall survival of 0.77 (95% CI 0.61 to 0.98). In that trial, fewer than half of the deaths were from prostate cancer, whereas in newly-diagnosed metastatic patients nearly all men will die of their disease. Therefore, it is relevant to note the relative benefit of radiotherapy in PR07 in terms of prostate cancer-specific survival, where the hazard ratio was 0.54 (95% CI 0.27 to 0.78). Long-term survival-based data, with a median follow-up of ~10 years, were presented orally at the American Society of Clinical Oncology 2012 which confirmed these findings.(7)

We anticipated that around 1250 patients were required over 4 years to observe 267 control arm deaths after 5.25 years. This assumed that (i) recruitment was constantly 70 pts/m to the trial overall; (ii) the original research arms stopped accrual within 6 months after activation of the RT arm; (iii) the abiraterone arm stops accrual around 24 months after activation of the RT arm; and (iv) a further new research arm with an equal allocation ratio was introduced 18 months after activation of the RT arm.

In protocol version 13.0, we reflect on these four points: (i) recruitment to the trial has been faster; (ii) the original research arms completed accrual 2 months after activation of the RT arm; (iii) the

abiraterone arm stopped accrual 12 months after activation of the RT arm; and (iv) Arm J was activated 18 months after activation of the RT arm, Arm H.

Of patients joining STAMPEDE during this time, 60% have been eligible for the "M1|RT comparison". Prior to randomisation, a RT schedule must be nominated: Weekly or Daily (see **Section 6.9**). We have observed that around half of patients in the comparison are nominated for RT with the Daily schedule and half for the Weekly schedule, primarily chosen by trial site with patient groups nominated for each schedule observed to be comparable at baseline. There will likely be interest to know the effect of each RT schedule when the main results are reported. This will be explored by "within schedule" comparisons of patients randomised to research vs control (arms H vs A) within each nominated RT schedule.

Therefore, in protocol version 13.0, the target sample size was increased from 1,250 patients up to around 1,800 patients, resulting in an approximate increase in the split by planned RT schedule from 625 to 900 in each "within schedule" analysis. A FFS analysis "within schedule" will be carried out at the time of the "main analysis"; predicted to have ~300 control arm FFS events by schedule (FFS "within schedule" analysis parameters: target HR=0.75, power 90%, 1-sided α=0.015). For either of the RT schedules showing evidence of an effect on FFS, a comparative "within schedule" analysis will be carried out on survival when ~199 control arm deaths are observed in that schedule comparison. This is a closed test with OS only formally compared within schedule if there is an advantage in FFS for that RT schedule at the main analysis. Thus, extending recruitment enables a secondary analysis of the impact of RT on survival by planned "RT schedule" to happen within around 18 months from the first main analysis.

All sample scenarios are documented in the Trial Master File.

All patients joining the trial will be starting long-term ADT for the first time. The focus of this comparison will be on the newly-diagnosed, metastatic patients (with no contraindications to RT), which is the largest subgroup of patients in the trial and the group of patients at highest risk of death from prostate cancer. Patients with non-metastatic disease will be excluded from this particular comparison as there are already randomised data demonstrating the survival benefit from radiotherapy in patients with locally advanced disease. Radiotherapy is now mandatory in node negative patients; it is also recommended in the node-positive, non-metastatic (N+ M0) group. Relapsing patients are also excluded from this comparison.

For the control arm of the whole trial, we constructed sample size scenarios median failure-free survival being 18, 24 or 30 months and constructed sample size scenarios around each of these options; the event rate would depend on the patient mix. We now know that around 60% of patients have M1 disease at trial entry and we have reported that FFS at 24 months is 51% across the whole of the control arm.(1)

For the sample size calculation for this new planned comparison, we have based our estimates on the subgroup of patients with newly-diagnosed M1 disease in the control arm. Therefore, we estimate median FFS to be 1 year and estimate that median overall survival will be around 3.5 years.

9.7 SAMPLE SIZE ISSUES AND TRIAL STAGES: ADDITIONAL RESEARCH ARM J

9.7.1 Pilot Phase: Additional Research Arm J

A similar approach will be followed for the additional research Arm J as detailed for the original research arms in **Section 9.4.1**. The IDMC first reviewed safety data for this combination when the first 50 patients allocated to Arm J had been on trial around 6 weeks (i.e. to the first follow-up visit).

The IDMC reviewed safety data again when 50 patients were 6 months out from randomisation. Additional safety reviews will be performed if the IDMC raises any concerns over safety and routinely reviewed at regular intervals.

Direct comparison will be available with contemporaneously randomised patients on Arm A (hormones alone). Contextual data will be provided from Arm G (hormones plus abiraterone). Indicative safety data may also be available on the combination from other studies in CRPC.

9.7.2 Activity Stages I-II: Additional Research Arm J

The principles of intermediate analyses will be applied to this new comparison, but some of the details will be different. Owing to the expected accrual rate (>100 pts/m) and the expected slower event rate, only two activity stages are planned before accrual is completed. These are set out in **Table 21**.

Table 21: Guidelines for stopping accrual to the additional research Arm J

Activity	Sig Level	Power	Targeted	Number of control	Consider discontinuation
I	0.40	95%	0.70	~66	>0.957
II	0.12	95%	0.70	~139	>0.869

9.7.3 Efficacy Stage III: Additional Research Arm J

The analysis of the final Efficacy Stage for this comparison will be performed when around 267 deaths have been observed in the control arm. This would give 90% power to detect the targeted hazard ratio of 0.75 at a one-sided significance level of 0.025.

9.7.4 Sample Size For Additional Research Arm J

Consideration will be given to ceasing further randomisations to Arm J if it is not showing sufficient evidence of activity on the intermediate primary outcome measure (FFS), just as for the other research arms.

The patient mix for this comparison is likely to represent a more favourable prognosis on average than in the original research trial's other arms, due to concurrent recruitment of M1 but not M0 patients, to Arm H.

We anticipate that around 1800 patients are required within 3.5 years to observe ~267 control arm deaths within 6 years. This time will be dependent on the observed overall survival. The default scenario assumes that (i) recruitment is constantly 70pts/m to the trial overall, (ii) the M1|RT arm accrues throughout and (iii) a further new research arm with an equal allocation ratio is introduced 18 months after activation of Arm J. The stopping date for Arm G is no longer an assumption.

Variations on these factors are documented in a Statistical Design Document. If accrual rates to the trial are at 150pts/m (as observed during Summer 2013), accrual of around 1,800 patients to the comparison could be achieved within 2 years. These sample scenarios will also be documented in the Trial Master File.

Updating the standard-of-care to include docetaxel has minimal impact on the projected time to maturity of the “enzalutamide + abiraterone comparison”. Within 3 months after this protocol version is activated, the TMG will review the mix of patients that do and do not include docetaxel as part of their standard-of-care. The TMG will consider whether changes are provoked, based on these administrative, patient characteristic data, with reference, if necessary, to the published events rates of similar patient groups from the “original comparisons”.

9.7.5 Further Sample Size Issues For Additional Research Arm J

Careful consideration will be given to the emerging data from the "abiraterone comparison" (Arm A vs Arm G) and whether this arm continues to recruit throughout. It is anticipated that recruitment to this Arm J comparison will be completed *before* survival data emerge from the "abiraterone comparison".

Indirect comparisons to understand the contribution from each agent may be possible if this research arm is demonstrably superior to the standard-of-care. These plans will be developed and documented elsewhere, but a higher number of patients will help with the power to the indirect comparison.

Figure 6: Schema of progress of STAMPEDE through the trial

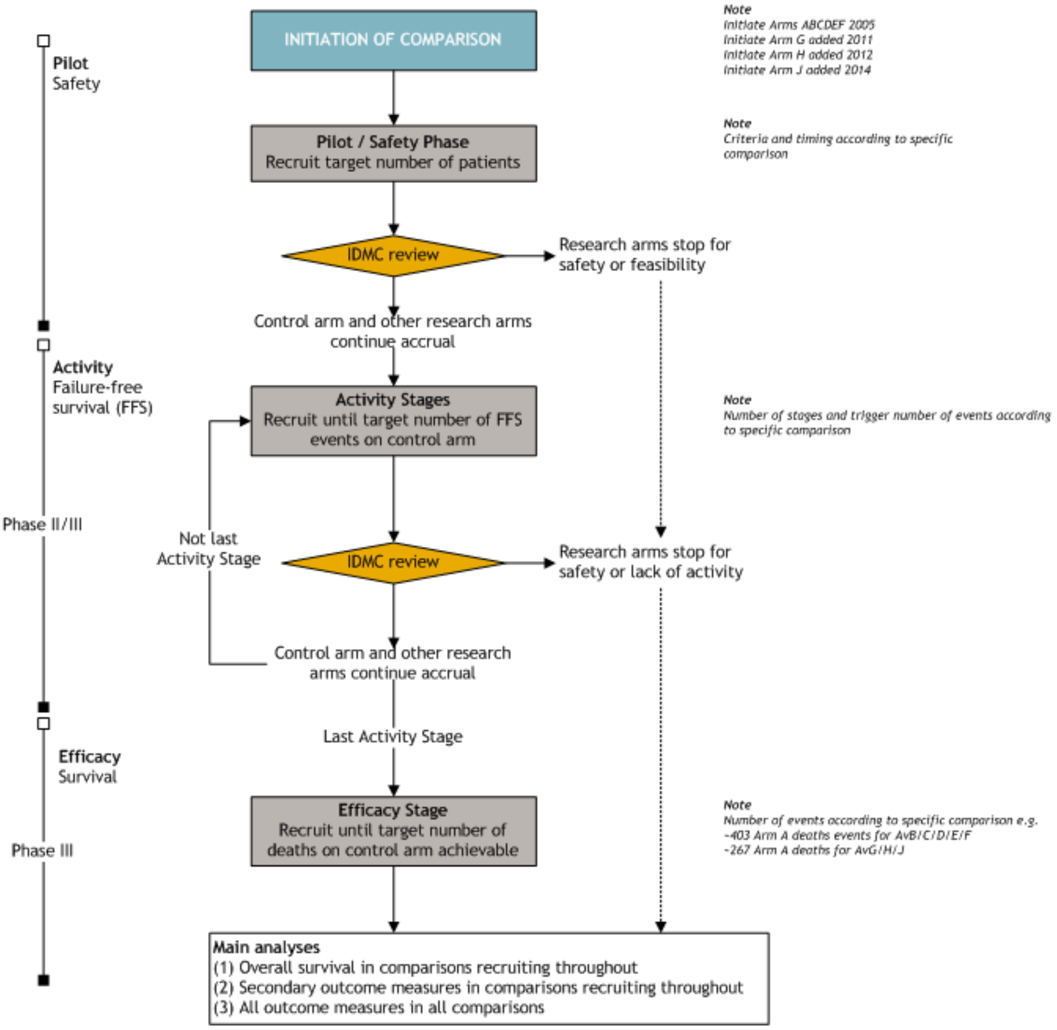

Key
FFS: Failure-free survival
HR: Hazard ratio
IDMC: Independent Data Monitoring Committee
Pts: Patients

Notes
Exact accrual depends on many factors including accrual rate, event rate and arms recruiting in each stage

9.8 FURTHER NOTES ON TRIAL DESIGN

9.8.1 Overall Sample Size

Given the adaptive nature of the study, there is no formal overall sample size target, but the numbers of patients required for each comparison are detailed in Sections 9.4 to 9.7. To date, more than 7,000 patients have been recruited overall.

9.8.2 Factorial Design

We note here that we have not employed a factorial design in this trial because we anticipate the possibility of synergy between ADT, zoledronic acid and docetaxel and between ADT, zoledronic acid and celecoxib.

It would not be possible to assess any such interactions reliably in a factorial trial (see the Statistical Design Document for further details).

9.9 INTERIM MONITORING AND ANALYSES

The accumulating data will be reviewed at regular intervals (approximately annually) by an Independent Data Monitoring Committee (IDMC), including pre-specified formal intermediate analyses of activity data (see also **Section 16**). These analyses will be performed by the trial team at the MRC CTU. Only patients randomised contemporaneously, and eligible for that comparison, will be included in the comparison of each research arm against control e.g. patients allocated to the control arm prior to protocol version 12.0 will not contribute to the "enzalutamide + abiraterone comparison" (Arm A vs Arm J).

The IDMC will be asked to give advice on whether the accumulating data from the trial justifies continuing recruitment of further patients or further follow-up; guidelines for discontinuation of accrual for the relevant Activity Stages, together with results from any other relevant trials will aid them in this. A decision to discontinue recruitment, in all patients or in selected subgroups will be made only if the result is likely to convince a broad range of clinicians including those entering patients into the trial and the general clinical community. The intermediate stopping guidelines apply to the intermediate primary outcome measure. To stop accrual early for benefit in any comparison would require convincing data in terms of the definitive primary outcome measure, overall survival. For example, this could be $p<0.001$ as proposed by Haybittle-Peto.(36, 37) The use of such a guideline for stopping for benefit has a minimal impact on the operating characteristics.

If a decision is made to continue without change, the IDMC will advise on the frequency of future reviews of the data on the basis of accrual and event rates. The IDMC will make recommendations to the Trial Steering Committee (TSC, see **Section 16**) as to whether the trial should continue in its present form. While the trial is ongoing the accumulating data will generally remain confidential, unless the TSC and IDMC agree that the data should be made public.

9.10 OUTLINE ANALYSIS PLAN

Analyses will be performed on an intention-to-treat basis. The standard unadjusted log-rank approach will be applied to analyses of FFS and OS. The impact of potential confounders including the stratification factors used at randomisation will be considered in a Cox proportional hazard model. Flexible parametric models will be used to calculate the absolute differences between the arms to show treatment differences over time and to estimate restricted mean “survival” times (RMST). The estimated difference in restricted means survival time (RMST) will be used preferentially to compare treatment arms if the proportional hazards assumptions required for hazard ratios cannot be supported. The χ^2 test or Mann-Whitney test will be implemented for categorical data comparisons, including toxicity, as appropriate. The primary outcome measures (see **Section 9.2**) will be considered for all arms of the trial at each phase, but the main emphasis will be placed on the comparison of the research arms that have continued to recruit throughout the trial.

9.10.1 PILOT / SAFETY PHASES

The Pilot Phase randomised patients between all the trial arms so that the results from these patients can be included in the main trial. Feasibility is considered in terms of acceptability of the trial randomisation and reported toxicities and adherence to trial medication. Centres participating

in the Pilot Phase for the original research arms were required to keep an anonymised log of all patients assessed for trial eligibility (see Protocol version 2.0) so that the number of patients who did not participate in the study and the number of eligible patients who chose to not participate in the study could be summarised (reasons for non-participation were collected where the patients was willing). The anonymised logs will not be needed for new research arms after Protocol version 8.0.

For the patients who are randomised, we shall describe the incidence of expected and unexpected severe toxicities and adverse events/reactions (see **Section 11**) to decide whether to continue with research arms beyond the Pilot Phase.

9.10.2 Activity And Efficacy Stages

The approach to analysis of these stages is summarised within the sample size calculations (see earlier subsections of **Section 9.4.3**). Each research arm will be compared in a pairwise fashion against the contemperaneously recruited control arm.

Full details are available in the Statistical Analysis Plan. See **Figure 6** for an overview of the schema of progress.

10 MONITORING AND QUALITY ASSURANCE

10.1 MONITORING AT MRC CTU

Data provided to the MRC CTU will be checked for missing or unusual values (range checks) and consistency over time. If missing or questionable data are identified, staff at the MRC CTU will request that the data be clarified. The exact procedures for data clarification and the amendment of CRFs will be described in the trial specific SOPs and instructions will be sent to all STAMPEDE institutions as soon as they have been approved to participate in the trial. The MRC CTU will also send reminders for any overdue data.

10.2 DIRECT ACCESS TO DATA

Collaborating institutions should be aware that direct access to patient data by MRC CTU staff may be required for trial-related monitoring or audit. Patient consent for this will be obtained as part of the general trial consent process.

10.3 VISITS TO INVESTIGATOR SITES

A selection of institutions will be visited at least once during the course of the STAMPEDE trial. The MRC CTU will give the responsible investigator adequate notice of the monitoring visit to allow adequate time, space and staff for these visits. The standard operating procedures (SOP) for monitoring are available from the MRC CTU.

After the monitoring visit the monitor will complete a site visit report. This report may be circulated to the TMG for comment. Once the TMT have reviewed the report and agreed on any recommendations the monitor will finalise the report and send a copy to the Principal Investigator (PI) at the site. A copy copy will be kept in the MRC CTU STAMPEDE trial master file.

10.4 CONFIDENTIALITY

All information collected during the course of the research will be kept strictly confidential. In addition, all procedures for handling, processing, storage and destruction of data are compliant with the Data Protection Act 1998. No individual patients will be identified when results from the trial are published.

Patients will be asked for permission for information about their health status to be obtained from the Office of National Statistics (ONS) or via the NHS Strategic Tracing Service or similar by the Medical Research Council, if necessary. In addition, patients will be asked for permission to inform their GP of their involvement in the STAMPEDE trial.

11 SAFETY REPORTING

ICH GCP requires that both investigators and sponsors follow specific procedures when reporting adverse events/reactions in clinical trials. These procedures are described in this section of the protocol. Further information on the expected toxicities for the trial interventions (docetaxel, zoledronic acid, abiraterone and radiotherapy) can be found in **Appendix G**.

11.1 DEFINITIONS

The safety reporting definitions from ICH GCP apply in this trial protocol. These definitions are given in **Table 22**.

Table 22: Event Terms and Definitions

TERM	DEFINITION
Adverse Event (**AE**)	Any untoward medical occurrence in a patient or clinical trial patient to whom a medicinal product has been administered including occurrences which are not necessarily caused by or related to that product.
Adverse Reaction (**AR**)	Any untoward and unintended response to an investigational medicinal product related to any dose administered.
Unexpected Adverse Reaction (**UAR**)	An adverse reaction, the nature or severity of which is not consistent with the information about the medicinal product in question set out in the summary of product characteristics (or Investigator brochure) for that product.
Serious Adverse Event (**SAE**) or Serious Adverse Reaction (**SAR**) or Suspected Unexpected Serious Adverse Reaction (**SUSAR**)	Respectively any adverse event, adverse reaction or unexpected adverse reaction that:<br>• results in death<br>• is life-threatening*<br>• requires hospitalisation or prolongation of existing hospitalisation**<br>• results in persistent or significant disability or incapacity<br>• consists of a congenital anomaly or birth defect<br>• Other important medical condition***

Clarifications and Exceptions

*The term 'life-threatening' in the definition of 'serious' refers to an event in which the patient was at risk of death at the time of the event; it does not refer to an event which hypothetically might have caused death if it were more severe.

**Hospitalisation is defined as an inpatient admission, regardless of length of stay, even if the hospitalisation is a precautionary measure for continued observation. Hospitalisations for a pre-existing condition (including elective procedures that have not worsened) do not constitute an SAE.

***Medical judgement should be exercised in deciding whether an AE/AR is serious in other situations. Important AE/ARs that are not immediately life-threatening or do not result in death or hospitalisation but may jeopardise the patient or

may require intervention to prevent one of the other outcomes listed in the definition above, should also be considered serious.

Pregnancy occurring in a STAMPEDE patient's partner during the patient's participation in the trial, must be reported to the MRC CTU within the same timelines as an SAE and classified as an 'other important medical condition' on the SAE form. The outcome of a pregnancy should be followed up carefully and any abnormal outcome to the mother or child should be reported.

Patients who develop any new primary carcinomas should have the event reported on a SAE CRF as "other important medical condition".

11.1.1 Trial-Specific Exemptions

Disease progression or death as a result of disease progression are not considered to be SAEs and should be reported on the STAMPEDE Progression Form or Death Form only.

The following situations that fulfil the definition of an SAE are excluded from expedited notification on an SAE form and should be reported only on the STAMPEDE follow-up form:

- Elective hospitalisation and surgery for treatment of locally advanced or metastatic prostate cancer or its complications
- Elective hospitalisation to simplify treatment or procedures
- Elective hospitalisation for pre-existing conditions that have not been exacerbated by trial treatment

11.2 INSTITUTION/INVESTIGATOR RESPONSIBILITIES

All non-serious AEs/ARs, whether expected or not, should be recorded in the toxicity (symptoms) section of the Follow-up CRF and sent to the MRC CTU within one month of the form being due. SAEs/SARs should be notified to the MRC CTU as described below.

The severity (i.e. intensity) of all AEs/ARs (serious and non-serious) in this trial should be should be graded using Common Terminology Criteria for Adverse Events (CTCAE) v3.0 (ctep.cancer.gov/reporting/index.html). Any questions concerning this process should be directed to the MRC CTU in the first instance.

11.2.1 Investigator Assessment

11.2.1.A Seriousness

When an AE/AR occurs the investigator responsible for the care of the patient must first assess whether the event is serious using the definitions given in **Table 22**. If the event is serious and not exempt from expedited reporting, then an SAE form must be completed and the MRC CTU notified.

11.2.1.B Causality

The Investigator must assess the causality of all serious events/reactions in relation to the trial therapy using the definitions in **Table 23**. There are 5 categories: unrelated, unlikely, possible, probable and definitely related. If the causality assessment is unrelated or unlikely to be related the event is classified as a SAE. If the causality is assessed as either possible, probable or definitely related then the event is classified as a SAR.

Table 23: Assigning type of SAE through causality

RELATIONSHIP	DESCRIPTION	EVENT TYPE
Unrelated	There is no evidence of any causal relationship	Unrelated SAE
Unlikely	There is little evidence to suggest there is a causal relationship (e.g. the event did not occur within a reasonable time after administration of the trial medication). There is another reasonable explanation for the event (e.g. the patient's clinical condition, other concomitant treatment).	Unrelated SAE
Possible	There is some evidence to suggest a causal relationship (e.g. because the event occurs within a reasonable time after administration of the trial medication). However, the influence of other factors may have contributed to the event (e.g. the patient's clinical condition, other concomitant treatments).	SAR
Probable	There is evidence to suggest a causal relationship and the influence of other factors is unlikely.	SAR
Definitely	There is clear evidence to suggest a causal relationship and other possible contributing factors can be ruled out.	SAR

11.2.1.C Expectedness

If the event is a SAR the Investigator must assess the expectedness of the event. Please see **Table 6** for a list of expected toxicities associated with the drugs being used in this trial. If a SAR is assessed as being unexpected it becomes a SUSAR.

11.2.1.D Notification

Investigators must notify the MRC CTU of all SAEs occurring from the time of randomisation until 30 days after the last protocol treatment administration for any research arm in the trial, including standard-of-care treatments, HT and docetaxel. Similarly, SAEs occurring in patients randomised to Arm A must be reported until 30 days after last injection or progression (whichever is sooner). SARs and SUSARs must be notified to the MRC CTU indefinitely for all research arms (i.e. no matter when they occur after randomisation).

11.2.2 NOTIFICATION PROCEDURE

The SAE form must be completed by the Investigator (consultant named on the signature list and delegation of responsibilities log who is responsible for the patient's care), with due care being paid to the grading, causality and expectedness of the event as outlined above. In the absence of the responsible investigator the form should be completed and signed by a member of the site trial team. The responsible investigator should subsequently check the SAE form, make changes as appropriate, sign and then re-fax to the MRC CTU as soon as possible. The initial report shall be followed by detailed, written reports as appropriate.

Send the SAE form by fax to the MRC CTU. Fax Number: + 44 (0) 20 7670 4818. The STAMPEDE trial team will confirm receipt of the SAE report to the main point of contact via email. Contact the STAMPEDE trial team If receipt is not received within 24 hours.

Follow-up: Patients must be followed-up until clinical recovery is complete and laboratory results have returned to normal or baseline, or until the event has stabilised. Follow-up should continue after completion of protocol treatment if necessary. Follow-up information can be updated on the originial SAE form by ticking the box marked 'follow-up' and faxing to the MRC CTU as information becomes available. Extra, annotated information and/or copies of test results may be provided separately. The patient must be identified by trial number, date of birth and initials only. The patient's name should not be used on any correspondence.

11.3 MRC CTU RESPONSIBILITIES

Medically qualified staff at the MRC CTU and/or the Chief Investigator (or a medically qualified delegate) will review all SAE reports received. The causality assessment given by the local Investigator at the hospital cannot be overruled and in the case of disagreement, both opinions will be provided in any subsequent reports.

The MRC CTU is undertaking the duties of trial sponsor and is responsible for the reporting of SUSARs and other SARs to the regulatory authorities (MHRA and competent authorities of other European member states and any other countries in which the trial is taking place) and the research ethics committees as appropriate.

The MRC CTU will also keep all investigators informed of any safety issues that arise during the course of the trial.

SAE REPORTING
Fax to 020 7670 4818 within 24 hours of becoming aware of the event

12 ETHICAL CONSIDERATIONS AND APPROVAL

12.1 ETHICAL CONSIDERATIONS

This is a randomised trial therefore neither the patients nor their physicians will be able to choose the patients' treatment. Treatment will be allocated randomly using a computer-based algorithm. This is to ensure that the groups of patients receiving each of the different treatments are as similar as possible.

All patients will receive standard treatment which will include ADT and may include radiotherapy and/or docetaxel. Patients may be randomised to one (or two; dependent on allocated arm) of the newer treatments in combination with hormone treatment. The trial has employed an unequal allocation ratio for some comparisons to maximise efficiency; this was explained in detail in the patient information sheet.

The newer combined treatment options are being assessed in a detailed and systematic fashion in this trial. There is some evidence to suggest that the newer treatment options may have advantages over standard treatment alone with regards to clinical outcome, but this is not confirmed and toxicity may be increased. This trial will follow a large group of men who have been randomly allocated to either the standard treatment (androgen deprivation therapy alone) or the newer combined treatment options in order to measure the benefits of the new treatments. The patients will also be followed-up for toxicity and safety issues, so that any benefits can be weighed against any negative aspects.

Patients participating in the trial will have some additional hospital visits and some extra blood samples taken compared to patients who are not participating in the trial, with the amount varying according to the allocated treatment. Sometimes the blood samples can be taken when the patient is attending hospital for treatment, anyway. On some of the trial arms, the patient may have to make additional visits to the hospital for the blood sample to be taken, although in some cases it may be possible for the blood sample to be taken in the GP's surgery. The additional visits and blood samples are to ensure that follow-up of patients is comparable in all the treatment groups. The blood samples will also be used for genetic and serum marker studies, where this information will be considered with clinical data. Blood samples will be link-anonymised. There will be no feedback to individual patients.

If new information emerges during the course of the trial which may affect the treatment or follow-up of patients who have joined the trial, information will be provided through by the trial team to all Principal Investigators. PIs therefore have the duty to inform the patients in their care of any new information emerging using any appropriate channel (e.g. letter, communication at follow up clinic, etc).

12.2 ETHICAL APPROVAL

The protocol has a Favourable Opinion from an appropriate Research Ethics Committee, according to national guidelines. Additionally, each site must also obtain management permission for research (R&D approval) from the relevant host organisations before patients can be entered into the trial. The patient's informed consent to participate in the trial should be obtained after a full explanation has been given of the treatment options, including the conventional and generally accepted

methods of treatment. Patient information sheets and patient consent forms are given in **Appendix B**.

The right of the patient to refuse to participate in the trial without giving reasons must be respected. After the patient has entered the trial, the clinician must remain free to give alternative treatment to that specified in the protocol, at any stage, if he feels it to be in the best interest of the patient. However, the reason for doing so should be recorded and the patient will remain within the trial for the purpose of follow-up and data analysis according to the treatment option to which he has been allocated. Similarly, the patient must remain free to withdraw at any time from the protocol treatment without giving reasons and without prejudicing his further treatment.

A statement of MRC policy on ethical considerations in clinical trials of cancer therapy, including the question of informed consent, is available from the MRC Head Office web site (http://www.mrc.ac.uk).

13 REGULATORY APPROVAL

This trial has been approved in the UK by the MHRA and will be conducted under a CTA (Ref: 00316/0026/001-0001) in the UK.

The trial has been approved in Switzerland by Swissmedic (Ref: 2009 DR 3235).

14 INDEMNITY

University College London holds insurance against claims from participants for injury caused by their participation in this clinical trial. Participants may be able to claim compensation if they can prove that UCL has been negligent. However, as this clinical trial is being carried out in a hospital, the hospital continues to have a duty of care to the participant of the clinical trial. University College London does not accept liability for any breach in the hospital's duty of care, or any negligence on the part of hospital employees. This applies whether the hospital is an NHS Trust or otherwise.

Participants may also be able to claim compensation for injury caused by participation in this clinical trial without the need to prove negligence on the part of University College London or another party. Participants who sustain injury and wish to make a claim for compensation should do so in writing in the first instance to the Chief Investigator, who will pass the claim to the managing organisation's Insurers, via the managing organisation's office.

Hospitals selected to participate in this clinical trial must provide clinical negligence insurance cover for harm caused by their employees and a copy of the relevant insurance policy or summary can be provided on request.

15 FINANCE

STAMPEDE is funded by the Clinical Trials Advisory Awards Committee (CTAAC) on behalf of Cancer Research UK; it is also funded by the MRC through the MRC Clinical Trials Unit. The trial has National Institute for Health Research Clinical Research Network (NIHR CRN) approval and, therefore, local NCRN funds may be available at each centre to support entry of patients into this trial.

M1|RT will be administered as per trial protocol using NHS RT equipment following successful RTQA by trial team.

Abiraterone is manufactured by Janssen Pharma PV (pharmaceutical companies of Johnson & Johnson). They have agreed to provide free drug and funds to distribute drug to participating sites and to help support the conduct and management of the trial.

Enzalutamide is manufactured by Astellas Pharma. They have agreed to provide free drug and funds to distribute drug to participating sites and to help support the conduct and management of the trial.

16 TRIAL COMMITTEES

16.1 TRIAL MANAGEMENT GROUP (TMG)

A Trial Management Group (TMG) has been formed comprising the Chief Investigator, other co-investigators and members of the MRC CTU. The membership of the TMG may be expanded if other groups of trialists wish to participate. It will also be amended during the trial if other circumstances require e.g. retirement.

The TMG will be responsible for the day-to-day running and management of the trial. They will meet by teleconference at least 3-monthly and in person as needed. The TMG members are detailed in **Appendix M**.

Further details of TMG functioning are provided in the TMG charter (available on request).

16.2 TRIAL STEERING COMMITTEE (TSC)

A Trial Steering Committee (TSC) has been formed to provide overall supervision for the trial and provide advice through its independent chair. The ultimate decision for the continuation of the trial lies with the TSC. The TSC will meet regularly.

Further details of TSC functioning are provided in the TSC charter (available on request).

16.3 INDEPENDENT DATA MONITORING COMMITTEE (IDMC)

An Independent Data Monitoring Committee (IDMC) has been formed. The IDMC will be the only group who sees the confidential, accumulating data to the trial. Reports to the IDMC will be produced by the MRC CTU. The IDMC will meet within 6 months of the trial opening with the frequency of meetings dictated by the IDMC. The IDMC will consider data in accordance with the analysis plan (see **Section 9**) and will be advisory to the TSC. The IDMC can recommend premature closure or reporting of the trial, or that recruitment to any research arm is discontinued.

From protocol version 8.0 onwards, any recommendation from the IDMC to stop recruitment to one or more trial arms will be acted upon immediately, pending ratification from the TSC. As this period between meetings should be very short, sites would not be notified until after the TSC have made a decision. IDMC recommendations based on emerging safety issues will be discussed with sites promptly.

Further details of IDMC functioning and the procedures for interim analysis and monitoring are provided in the IDMC charter (available on request).

Figure 7: Diagram of relationships between trial committees

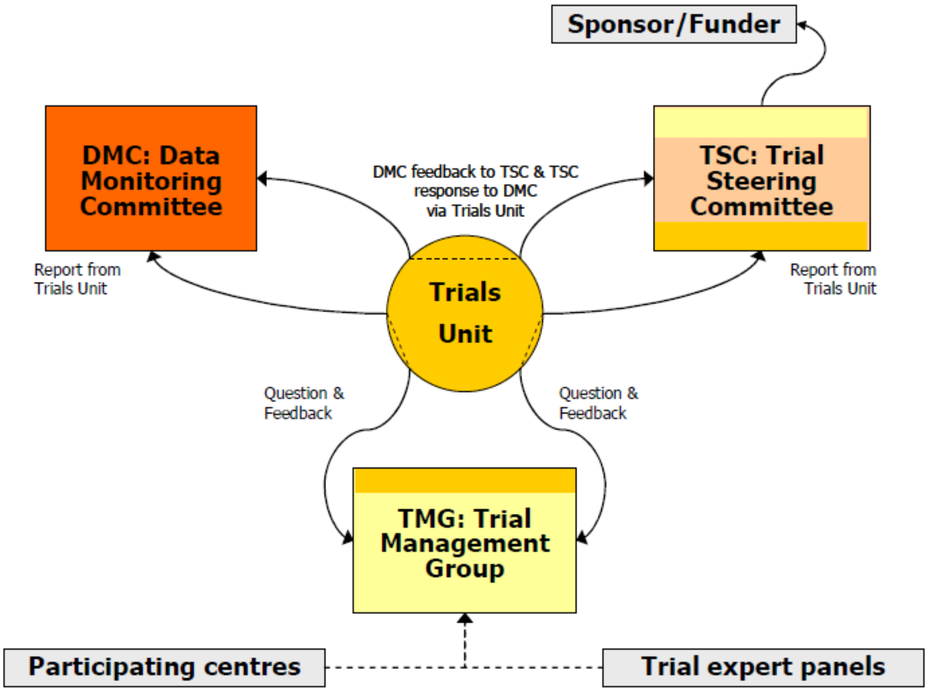

17 ANCILLARY STUDIES

17.1 QUALITY OF LIFE

A quality of life (QL) study is being performed to assess the impact of each treatment arm on the quality of patient's lives. Initial participation in this study was limited to the first 700 patients recruited (this was reached in Sep-2008) patients. The QL study re-opened from the implementation of version 8.0 of the protocol. The EORTC QLQ-C30 with the prostate-specific module QLQ PR25 will be used. Key items for assessment are pain reduction for patients with metastatic disease and urinary symptoms for patients with locally advanced disease. In addition specific hypotheses will be generated for each of the research arms. The EuroQol (EQ-5D) (38) will be used in the study as a generic measure of health-related quality of life which can be linked to public preferences. These data will be used to calculate quality-adjusted life-years as part of the economic evaluation (see Section 17.2). Patients recruited into the QL study, should continue on the study throughout the trial. Questionnaires should be self-administered, although it is recommended that a key person (e.g. research nurse) at each centre be responsible for the data collection to optimise compliance and completeness of the data.

The QL and the HE questionnaires should be completed without conferring with friends or relatives and all questions should be answered even if the patient feels them to be irrelevant.

The responsible person should check each questionnaire for its completeness, ensuring that the correct date of completion and patient identifiers are present. The research nurse should approach patients at appropriate clinical visits to complete a questionnaire. If no clinical visit is scheduled for the patient (with a window of 4 weeks around the expected date) the nurse should organise the completion of the questionnaire, by post or by a visit to the patient at home (or in a hospice).

17.2 HEALTH ECONOMICS

A health economics (HE) sub-study will be performed. Core resource use information will be collected, using CRFs on days in hospital (by speciality) and outpatient visits. Data collected on concomitant medication will also be used in the economic analysis. Information on patients' use of primary care and community-based services will be collected as additional questions in the QL questionnaire. Costs will be calculated on the basis of representative UK unit costs at the point of analysis. Health outcomes will be assessed in terms of quality-adjusted life years (QALYs). Quality adjustments will be based on patients' responses to the EQ-5D health status measure which will be administered at baseline and each point of follow-up as part of the QL questionnaire. A cost-effectiveness analysis will compare all regimens that continue to recruit into their final Efficacy Stage IV.

17.3 TRANSLATIONAL SUB-STUDIES

17.3.1 DNA Analysis

Blood samples from as many patients as possible have been collected for future translational research. With patient consent, an additional droplet of blood sample has been collected using FTA

Elute cards and stored for DNA and protein analysis in order to try to identify molecular features of clinical significance.

FTA Elute cards supplies have not been available since Dec-2013 and the STAMPEDE TMG has pursued an alternative method for genomic DNA collection using the Oragene® DNA kits for saliva sampling.

Oragene kits are widely used for collection of DNA from patients participating in clinical trials and they have been demonstrated to be a suitable alternative to DNA collection from whole blood providing a non-invasive, painless method of high quality sample collection.

A subset of patients may be asked if they would like to donate a blood sample for additional genetic research analysis.

Details of specimen collection, posting and contact details are given in **Appendix D**.

17.3.2 Tissue Microarray

Patient consent will be sought to utilise paraffin embedded tissue for the construction of tissue microarrays from needle cores. One needle biopsy will be selected for microarray and the remaining tissue will be returned to the originating histopathology lab. Given the entry criteria for the trial, the majority of patients will have extensive disease in the diagnostic needle core biopsies, in contrast to men with localised, low grade disease. Consequently, removal of one core is unlikely to compromise any subsequent histopathological assessment. Details regarding transfer of samples will be issued at the time of construction of the micro array. Additional analyses e.g. DNA extraction may also be performed on the tissue arrays.

18 PUBLICATION

The results from different centres will be analysed together and published as soon as possible. Individual clinicians must not publish data concerning their patients that are directly relevant to questions posed by the study until the TMG has published its report. The TMG together with the STAMPEDE collaborators will form the basis of the writing committee and decide on the nature of publications. Any release, of efficacy or safety data, presentation or publication will be agreed with the TSC according to the terms of their charter.

All publications will acknowledge the participating centres and clinicians, and these will be detailed in an appendix to the main report. Papers will have named authors determined by the TMG according to the following principles:

- To be as inclusive as possible where this is practicable
- To ensure that there is justification for anyone to be named as an author
- Reasons for nomination for authorship may include: trial design; grant holding; day-to-day trial oversight (TMG membership); analysis; discussion and interpretation of data; representation for key groups; active participation at large recruiting sites. It should be accepted that the people qualifying for authorship will vary over time. In addition, key positions will vary depending on the nature of the publication: clinical lead for clinical papers, statistician lead for methodology papers, translational papers may be led by authors not on the main TMG if appropriate (e.g., the bone sub-study). In the event of any dispute related to authorship or data release, the TSC will be responsible for making the executive decision.

In the manuscript, a full list of sites and the number of patients recruited will be provided. In the presentations, this list of sites will also be shown. The term “the STAMPEDE investigators” will clearly be stated and relevant names included in the presentation credits.

A detailed Publication Plan is documented elsewhere.

19 PROTOCOL AMENDMENTS

19.1 PROTOCOL

19.1.1 AMENDMENTS MADE TO SECTIONS IN PROTOCOL VERSION 1.0 (MAY 2004)

Administrative changes such as typos, word change etc.

Name additions/changes to:

TMG members

TSC members

IDMC members

'General Information' Section – additional information re. Abridged version of protocol

Section 1.2 – Figure 1, Celecoxib duration amended

Section 1.3 – Figure 2, addition of cardiovascular assessment form, name and timings amended

Section 2.3 – Docetaxel information updated

Section 2.4 – Additional text re dose and duration justification for Celecoxib use.

Section 3 – Title change and content updated

Section 4.2 – New exclusion criteria added

Section 4.3.1 – New investigations added and additional text re testosterone measurements and additional text re. prior celecoxib treatment

Section 6.1.4 – Celecoxib duration amended

Section 6.1.5 – Additional text re. Co-administration of docetaxel and bisphosphonates

Section 6.1.6 – Celecoxib duration amended

Section 6.2.2 – additional docetaxel information

Section 6.2.3 – addition of CV event history

Section 11 – Safety reporting updated

Section 12.1 – Additional text re. the collection of blood for genetic and serum marker studies

Section 15 – Additional information re. Central Subvention for docetaxel arms

19.1.2 AMENDMENTS MADE TO SECTIONS IN PROTOCOL VERSION 1.1 (MAY 2005)

Section 6.2 Administration and Dose Modifications, subsection 6.2.1 Zoledronic Acid

19.1.3 AMENDMENTS MADE TO SECTIONS IN PROTOCOL VERSION 2.0 (JUN 2005)

General Information section – SAE reporting fax number and timeframe added.

Section 1.2 – Addition of anti-androgen use for M0 patients as a method of HT

Section 1.2 – Increase in amount of blood needed & addition tissue sample request.

Section 1.3 Trial Documentation updated to include new table detailing trial documentation ahead of accreditation, the inclusion of the radiotherapy forms and correct case report form timings

Section 2.1 – Addition of anti-androgen use for M0 patients as a method of HT

Section 4.1.3 – Inclusion criteria Vii "Normal testosterone prior to hormone treatment" removed.

Section 4.1.3 - φnote has been omitted and moved to section 4.2 (see number 8)

Section 4.2 – Exclusion criteria added to exclude patients with active peptic ulceration, gastrointestinal bleeding and inflammatory bowel disease.

Section 4.2 – Exclusion Criteria added to exclude patients with planned major dental work

Section 4.3.1 - All blood test timelines changed from 14 days to 28 days.

Section 4.3.1 – Hormone Therapy pre-randomisation deadline extended from 4 weeks to 12 weeks.

Section 4.3.1 – Additional information regarding the use of NSAIDs and cox-2-inhibitors before coming on to the STAMPEDE study and once commenced on study treatment

Section 4.3.2 – Updated to ask for all vitamins and minerals the patient is taking to be recorded.

Section 4.3.3 – Updated to include the extra blood required and the request for consent of patients' tissue samples.

Section 6.1.1 – Addition of anti-androgen use for M0 patients as a method of HT

Section 6.1.6 – Addition of the calcium & vitamin name "calcichew".

Section 6.6.2 – asking also to collect vitamins and minerals under concomitant medication.

Section 6.6.3 – New section to inform investigators that patient's, who they wish to give radiotherapy to, are also eligible for STAMPEDE

Section 6.6.4 – New section to detail what data is being collected on the radiotherapy given to patients.

Section 7.1; figure 4 – Addition of radiotherapy form and in note, addition of AA alone

Section 7.1.2 – omission of repeated scans and x-rays at 24 weeks, also omitted in note under figure 4.

Chapter 11 – Safety reporting section updated

Section 17.3 – Increase in amount of blood needed & additional tissue sample request.

19.1.4 Amendments Made To Section In Protocol Version 3.0 (Jul 2006)

Front Cover - NCRN logo added for accuracy

Front Cover - Clarification that protocol developed with NCRI rather than on behalf of

Front Cover - Clarification that it is a 6 arm trial

General Information section - MRC CTU staff section updated

Section 1.2 – Statistics section updated.

Section 1.2 - Additional research paragraph updated to reflect additional studies and for clarification of terms

Section 1.2 - Blood collection volume changed to reflect new technique used

Section 1.3 (figure 3) - Table showing case report form schedule updated to reflect clarification of follow-up schedule and addition of new CRF (End of Treatment)

Section 2.2 - AS changed to HT (clarification of terms)

Section 2.3 - Updated in information in regard to use of docetaxel added to reflect up to date practice

Section 2.5 - Sub-headings numbered for consistency

Section 3.0 - Information in regard to the Pilot Phase now written in past tense as Pilot Phase has now been completed

Section 4.1.1 - Inclusion criteria extended so that patients who fulfil 2 out of the three of the first inclusion criteria can be eligible.

Section 4.3.1 - Change in time scales by which baseline investigations need to be completed.

Section 4.3.1 - Clarification that chest X-ray is only required if chest is not included in the CT

Section 4.3.1 - Removal of 12 week timeline for baseline PSA test to be performed. (Stipulation that it must be performed before start of HT)

Section 4.3.2 – Information added in regard to time allowed from randomisation to start of treatment

Section 4.3.3 - Additional research paragraph updated to reflect additional studies and for clarification of terms

Section 4.3.3 - Blood collection volume changed to reflect new technique used

Sections 6.1.2-6.1.6 - Androgen Suppression replaced with hormone therapy for consistency of terms

Section 6.2.2 - '(Taxotere)' Removed for consistency

Section 6.2.2 _ information added in regard to the need to closely monitor liver function prior to docetaxel administration

Section 7.1 - Page number reference updated

Section 7.1.1 - PSA measurement timings updated to accurately reflect follow-up schedule

Section 7.3 (Table 4) - Table and key updated to accurately reflect follow-up schedule and to include information about new CRFs and removal of withdrawal CRF

Section 8 - Rewording for clarification of definition of trial withdrawal

Section 8.1 - Instruction that withdrawal from trial treatment should be recorded on End of Treatment Form rather than withdrawal form

Section 8.1 - Information updated to emphasise that trial treatment must be discontinued following a progression

Section 8.2- Information added in regard to patient transfers

Section 8.3 - Instruction that withdrawal from trial completely must be notified in writing to the MRC CTU rather than included on withdrawal form

Section 9 and Summary – Target event numbers updated to reflect the slightly revised numbers obtained by using –nstage- which is the new, recommended program for MAMS trials

Sections 11.1 and 11.2 - Form numbers removed to allow for future changes in numbering

Section 11.2 – Reference to toxicity grading website added

Section 11.2.1 - Reference to table in appendix G added

Section 12.2 - 'Suggested' removed from 'Suggested patient information sheets'

Section 13 - CTA reference added

Section 17.3 - Information added to reflect new blood collection method for DNA analysis and in regard to additional translational studies for which funding has recently been approved

19.1.5 Amendments Made To Protocol Version 4.0 (Dec 2007)

General Information Section - Randomisation and SAE reporting details sections clarified

Section 1.2 and throughout protocol - Efficacy Stages 1-111 renamed to Activity Stages 1-111 for accuracy and clarity

Section 1.2 - Follow schedule corrected

Section 4.1.2 - Inclusion criteria widened to include high risk relapsing patients, that would not have met the previous PSA based criteria

Section 4.1.3 - Note added to reference location of WHO performance status definitions

Section 4.2 - Notes added to reference locations of toxicity gradings and NYHA classifications

Section 4.3.1 - Timings of baseline scan information changed to accurately reflect most common current practice

Section 6.1.1 - Information about use of LHRH antagonists to ensure that the protocol accurately reflects current and future practice

Section 6.1.1 - Information about suggested duration of hormone therapy added to ensure that the protocol accurately reflects current practice

Section 6.2.2 - Additional information added about the timing of liver function tests prior to docetaxel administration added for clarity

Section 6.6.4 - Information on radiotherapy data collection added

Section 7.1.1 - Erroneous information about the timing of PSA measurements removed

Figure 3 - Moved to new section in protocol for clarity and extended to include current information on data collection

Figure 3b - Added to describe how extent of data collection during follow-up should change, post treatment and post progression

Figure 4 - Notes added to explain the changes in data collected at follow-up and to information that the quality of life study will be applicable to the first 700 patients randomised only

Figure 4 - Note added to include palliative radiotherapy CRF

Section 11.3 - SAE reporting information updated

Section 19 - Protocol amendments list updated

19.1.6 Amendments Made To Protocol Version 5.0 (Aug 2008)

1. General Information Section – Randomisation phone line number updated – non UK extension added
2. Section 3 – Information about QL study removed to reflect closure of QL study after first 700 patients
3. Section 4.2 – Exclusion criteria clarified to explain that only patients with severe poor cardiovascular history should be excluded
4. Section 4.3.1 – Information on co-administration of NSAIDS with celecoxib changed based on clinical advice.

5. Section 5 - Randomisation phone line number updated – non UK extension added

6. Section 6.2.1. – Information added to clarify that patients who develop an osteonecrosis of the jaw should stop zoledronic acid treatment

7. Section 6.2.3 – 'severe' text added to accurately reflect which patients should be excluded based on their cardiovascular history

8. Section 7.1.2 – Definition of disease progression extended for clarity

9. Figure 3 – Updated to include reference to newly created skeletal related event form

10. Figure 4 – Previous error in table amended to show that the 4th Zoledronic Acid form that is submitted contains information about 3 cycles rather than 2 as previously indicated

11. Table 4 – 'Other important medical condition' added to definition of serious in the SAE section, to accurately reflect SAE form and current practice

12. Section 11.1 – Information added on reporting or pregnancies

13. Section 17 - Information about QL study removed to reflect closure of QL study after first 700 patients

19.1.7 Amendments Made To Protocol Version 6.0 (Jul 2009)

1. General Information Section – Trial Pharmacist removed and changes of:

Co-Investigator

Patient Representatives

Trial Manager

Data Manager

General Information Section - Coordinating Centre – address change

General Information Section – change of Sponsor address

Section 1.1 – ratio of patients randomised to the investigational arms updated

Section 1.2 – figure 1b added to clarify trial design from Apr-2011 onwards

Section 1.2 – paragraph added to explain trial changes after the second activity analysis

Section 1.2 – wording added to clarify that QL data only collected for first 700 patients randomised

Section 1.3 – SSA Favourable Opinion removed from list of trial documentation required ahead of site accreditation

Section 2.1 – Amount of men diagnosed with prostate cancer annually updated

Section 2.4 –note added to explain completion of recruitment to celecoxib- containing arms

Section 2.5.2 - note added to explain completion of recruitment to celecoxib- containing arms

Section 3 – SSA Favourable Opinion removed

Section 4.2 – Exclusion criterion xiii greyed out

Section 4.3.1 – paragraph removed regarding potential randomisation to celecoxib-containing arms

Section 5 – Randomisation instructions expanded to exclude public holidays or dates when notice has been given by the CTU

Section 6.1.4 – formatting changed to grey font to reflect recruitment completion for arm D

Section 6.1.6 - formatting changed to grey font to reflect recruitment completion for arm F

Section 6.2.3 – recruitment note added

Section 6.6.3 – radiotherapy statement changed to reflect data from recent trials

Section 7.1.2 – removal of reference to SRE- specific CRF

Section 7.3 – Figure 3 - Addition of Bone Density Risk Factor Form and BMD sub-study assessment forms to summary of timing table

Section 7.3 – Figure 4 – Weeks added to timings of assessments post 2 years

Section 7.3- Figure 4 – note added to explain recruitment completion for arms D and F

Section 12.1 – Wording changed to reflect change to randomisation allocation ratio

Section 12.1 – Addition of statement regarding new information emerging during the trial

Section 12.2 – Reference to SSA removed

Section 16.3 – Statement added regarding actioning IDMC recommendation ahead of TSC ratification

19.1.8 Amendments Made To Protocol Version 7.0 (Jul 2011)

1. General Information Section- SAE reporting fax number corrected

2. Section 11- SAE reporting fax number corrected

19.1.9 Amendments Made To Protocol Version 7.1 (Jul 2011)

Throughout protocol – numbering has been updated in some sections new accommodate new information that has been added.

General Information Section – contact details updated

General Information Section – Funding information updated to include involvement from additional company

General Information Section – Wording on compliance and regulations updated to reflect current MRC CTU standard wording

General Information Section – Abbreviations list updated

Section 1.1 – The number of investigational agents being studied updated from three to four

Section 1.1 – Information regarding celecoxib updated to reflect that recruitment to these arms was discontinued in Apr-2011

Section 1.1 – Information about new IMP, Abiraterone inserted

Section 1.1 – Sample size and trial duration information updated to reflect changes brought about by additional trial arm

Section 1.2 – Summary information updated to reflect the discontinuation of recruitment to celecoxib arms and the addition of abiraterone

Figures 1a, b and c - Updated to reflect the discontinuation of recruitment to celecoxib arms and the addition of abiraterone

Section 1.2 – Information on trial stages updated to reflect changes brought about by additional trial arm

Section 1.2 – Information updated regarding the re-opening of the quality of life sub-study from implementation of protocol version 8.0

Section 2.1 – Wording related to hormone therapy updated for clarity

Section 2.1 - Updated to reflect the discontinuation of recruitment to celecoxib arms and the addition of abiraterone

Section 2.2 – Updated references added

Section 2.3 – Updated references added

Section 2.5 – Section added to give background information on new IMP, abiraterone

Section 2.6.1 – Updated references added

Section 2.7 – Section added to give information regarding radiotherapy which is to be given as part of standard care following recently published trial data.

Section 3 – Wording updated regarding selection of investigators to reflect current MRC CTU practice

Section 4.1 – Inclusion criteria updated with new criterion regarding radiotherapy use

Section 4.1 - Inclusion criteria updated with new criterion regarding contraceptive use

Section 4.1 – Wording of inclusion and exclusion criteria updated for clarity

Section 4.1 – Exclusion criteria updated with new criterion regarding acceptable liver function for trial entry

Section 4.1 – Exclusion criteria updated with specifics related to blood pressure levels

Section 4.1 - Exclusion criteria updated with new criterion regarding concomitant medications

Section 4.1 - Exclusion criteria updated with new criterion regarding prior treatment with abiraterone

Section 4.1 - Exclusion criteria updated with new criterion regarding prior treatment with chemotherapy

Section 4.1 - Exclusion criteria updated with new criterion regarding prior treatment with zoledronic acid

Section 4.3 – Wording updated to reflect that patients who initially fail screening can be re-screened at a later date

Section 4.3.2 – Wording updated regarding prior anti-androgen and LHRH use updated for clarity

Section 5.1 – Co-enrolment guidelines information updated to describe newly created co-enrolment CRF

Section 6.1 – Trial treatment information updated to reflect the fact that anti-androgens alone will be no longer permitted as hormone therapy

Section 6.1.1 – Updated to describe patients for whom radiotherapy should be given as standard practice

Section 6.1.1 a and b - Sections added to give information regarding radiotherapy treatment

Section 6.1.1-6.1.6 – References to further sections updated

Section 6.1.7 – Section added to describe abiraterone treatment

Section 6.2.4 - Section added to describe abiraterone treatment

Section 6.6 - Section added to give information regarding radiotherapy treatment

Section 7.1.1 – Reference to blood being taken at patient's home removed as this does not occur in practice

Section 7.1.2 – Wording updated regarding the reporting of biochemical failures for clarity

Section 7.1.2 – Wording updated regarding skeletal-related events for clarity

Section 7.1.3 – Section added to describe additional assessments required related to abiraterone treatment

Section 7.1.4 – Section added to provide information on when treatment should commence

Figure 4 – Updated for clarity regarding return of BMD sub-study forms, the addition the co-enrolment CRF and the description of the re-opening of the QoL Sub-study.

Figure 5 – Updated with reference to abiraterone and co-enrolment form

Section 7.3 - Wording on trial closure updated to reflect current MRC CTU standard wording

Section 8.1 – Additional criteria for definition of progression added for clarity

Section 8.1 – Definition of progression for abiraterone patients added.

Section 9 – Statistical information updated to describe the addition of the new trial arm

Section 11 – Safety reporting wording updated for clarity

Section 11 – SAE reporting fax number updated

Section 12 – Ethical information updated to describe the unequal randomisation allocation ratio

Section 12 – Ethical information updated to describe that the visit schedule will vary according to trial arm

Section 12.2 – Wording updated to reflect international participation in the trial

Section 13 – Wording updated to reflect international participation in the trial

Section 14 – Wording updated to reflect international participation in the trial

Section 15 - Updated to reflect the discontinuation of recruitment to celecoxib arms and the addition of abiraterone

Section 16 – Reference to trial committee charters added for information

Section 17.1 – Information added to reflect re-opening of quality of life sub-study

Section 17.2 – Timing of health economics analysis updated to previous error

Section 18 – Information on publication policy expanded for clarity

Section 19 – Information regarding amendments to protocol appendices moved to the separate appendices document

Section 20 – References extensively updated

19.1.10 Amendments Made To Protocol Version 8.0 (Sep 2011)

Throughout protocol – numbering, sections headings, tables, figures and bibliographical references have been updated in some sections to accommodate new information that has been added

Throughout protocol – Androgen Deprivation Therapy has replaced Hormone Therapy as deemed more representative of the type of hormone therapy used in the study

General Information Section – New staff members of the MRC CTU and Co-Investigators added and contact details updated

General Information Section – Abbreviations list updated

Section 1.1 – Information regarding the new research radiotherapy treatment inserted

Section 1.1 – Information regarding docetaxel updated

Section 1.2 – Wording updated to reflect the addition of the new research comparison arm

Section 1.3 – Additional criteria for the re-accreditation of participating centres (for protocol version 9.0 only)

Section 2.1.1 – Wording updated to clarify the use of anti-androgen in trial patients

Section 2.1.2 – Information added to describe the rationale for the RT comparison arm

Section 2.8 – Information added to describe research RT treatment to prostate for patients with newly diagnosed metastatic disease

Section 3.1 – Information added to describe RT Quality Assurance procedures and centre accreditation

Section 4.1.1 to 4.1.3 – Wording updated to clarify inclusion criteria for all patients groups (newly diagnosed non-metastatic, metastatic and relapsing patients)

Section 4.2 – Clarification added on cardiovascular exclusion criteria

Section 4.2 – New exclusion criterion added concerning patients with prior exposure to hormone therapy

Section 4.2 – New exclusion criterion added to reflect the addition of the new RT comparison arm

Section 4.4.1 – Clarification added regarding pre-randomisation checks

Section 4.4.2 – Clarification added regarding permissible hormone therapy duration prior to randomisation

Section 4.4.5 – Information added regarding starting research radiotherapy treatment

Section 4.4.6 – Information updated on concomitant medications

Section 5 – Clarification regarding randomisation allocation added to reflect the addition of the new RT research arm

Section 6.1.8 – Information added to describe the administration of research radiotherapy

Section 6.2.1 – Clarification added regarding the measurement of serum creatinine levels prior to the administration of zoledronic acid

Section 6.2.3 – Clarification regarding the completion of recruitment to the celecoxib containing arms

Section 6.25 – Information added regarding the administration of research radiotherapy treatment

Section 6.6 – Clarification incorporated to describe the administration of standard-of-care radiotherapy

Section 7.1.4 – Information added regarding data collection and non-administration of standard radiotherapy

Section 7.2 – Section updated to include new treatment specific CRFs and timing of CRFs

Section 8.1 – Clarification added for the criteria to stop treatment for patients randomised to arm G

Section 8.2 – Section expanded to include additional details on study patient transfer to different centres

Section 8.3 – Additional sentence inserted to reinforce the importance of compliance with follow up assessments

Section 9.1 – Additional paragraph inserted to clarify the method of randomisation and allocation distribution in the light of the introduction of the new RT arm

Section 9.4 – Wording updated to clarify the assessment of safety data

Section 9.5.4 – Wording updated concerning the end of randomisations to arm G

Section 9.6 to 9.6.4 – Section added describing sample size issues and trial stages for arm H

Section 9.8 – Clarification on intermediate stopping guidelines

Section 9.9 – Clarification on the outline analysis plan

Section 11 – Information on safety reporting updated to reflect the addition of the research RT comparison arm

Section 11 – Clarification added regarding arm A safety reporting timelines

Section 12.1 – Clarification added regarding the Principal Investigator's responsibilities

Section 14 – Indemnity section updated to reflect current MRC policy

Section 16 – Clarification regarding TMG membership

Section 17.3 – Section on Bone Mineral Density sub-study removed

Section 19 – Information regarding amendments to protocol appendices moved to the separate appendices document

Section 20 – References updated

19.1.11 Amendments Made To Protocol Version 9.0 (Oct-2012)

Throughout protocol – numbering, sections headings, tables, figures and bibliographical references have been updated in some sections to accommodate the completion of recruitment to original research arms B, C and E.

Throughout protocol – Tenses have been changed to reflect activities that were in the future and which have now been passed.

Section 1 – Figure added and clarifications added to each figure

Section 2 – Previous reference 8 removed

Section 4 – Clarification of acceptable alternatives to bone scans

Section 6.2.5 – Correction of an error defining the PTV: the wording has been reordered

Table 4 – Dose-volume objectives corrected: order swapped

Table 5- Correction CRFs names

Section 17.3.2 – Clarification that DNA may be extracted

19.1.12 Amendments Made To Protocol Version 10.0 (Apr-2013)

Throughout protocol – numbering, sections headings, tables, figures and bibliographical references have been updated in some sections

Throughout protocol – typos have been corrected

Section 4 –Clarification of exclusion criteria V (now V and VI)

Section 6 – Timing of orchidectomy prior to randomisation extended to 12 weeks

Section 6 – Clarification of hypokalaemia, blood pressure and fluid retention management

Section 9 – Statistical considerations amended in light of the recruitment extension for the abiraterone comparison

Section 14 - Section updated to reflect the changes in the structure of the MRC CTU (now MRC CTU at UCL) and indemnity arrangements

19.1.13 Amendments Made To Protocol Version 11.0 (Sep-2013)

Throughout protocol – numbering, sections headings, tables, figures and bibliographical references have been updated in some sections

Throughout protocol – typos have been corrected

Co-investigators list updated to reflect the addition of the “enzalutamide + abiraterone comparison” lead

Section 1.2 – Enzalutamide added as trial treatment

Section 1.2 – Protocol version 12.0 added to the list of amendments

Section 2.10 – Rationale for the combination of enzalutamide and abiraterone

Section 4.2 – Eligibility criteria amended to reflect the addition of enzalutamide + abiraterone arm

Section 4.4.2 – Wording clarified

Section 6.8 – Clarification regarding end of trial treatment after starting trial therapy

Section 6.10 – Section added to describe enzalutamide and abiraterone treatment for the new research arm (Arm J)

Section 6.11.4.A – Section added to describe the management of toxicities from trial abiraterone

Section 6.11.4.B - Section added to describe the management of toxicities from trial enzalutamide

Section 9.1.4 – Section added to describe the statistical considerations concerning the introduction of Arm J

Section 9.3 – Principles and assumption for the introduction of Arm J added

Section 9.7 and sub-sections – Sample size issues and trial stages for Arm J

Section 9.9 – Details on interim monitoring and analyses for Arm J added

Section 11.2.1.D – Wording clarified regarding safety reporting requirements for control arm

Section 12.1 – Wording clarified

Section 15 – Details on funding for the “enzalutamide + abiraterone comparison” added

Section 19 - Amendments made to protocol updated

Reference list updated

19.1.14 Amendments Made To Protocol Version 12.0 (Jan-2014)

Throughout protocol – typos have been corrected

Section 4.4.2. Wording clarified

Section 4.3. Wording clarified for eligibility to M1|RT comparison

Section 6.10. Addition of use of dexamethasone post-biochemical progression for Arm J patients

Section 6.11.4.A. Correction of CTCAE version

Section 6.11.4.C. Clarification on enzalutamide dose modification to be in line with current SmPC

Section 9.6. Sample size increase for M1|RT comparison

Section 11. Correction of safety reporting timelines for Arm A patients

Section 17. Addition of saliva samples collection for DNA analysis

Table 4, 5 and 6. Clarification on Case Report Forms and Follow-up schedule

19.1.15 Amendments Made To Protocol Version 13.0 (FEB-2014)

Throughout protocol – typos have been corrected

Throughout protocol – clarification on the new definition of standard-of-care

Table of contents updated to reflect any changes to the protocol

Section 1.1. Wording added throughout section to include reference to survival results from “original comparisons”

Section 2.1.1. Section improved to include reference to survival results from “original research comparisons”

Section 2.1.2. Section improved to include reference to survival results from “original research comparisons”.

Section 2.1.3. Additional section added to describe the role of docetaxel for men with M0 or M1 disease

Section 2.9. Clarification on treatment completion and primary results for “original research comparisons”

Section 4.2. Clarification of Exclusion criteria XIII and XVI

Section 4.4.2. Clarification on HT prior to randomisation

Section4.4.3. New section to clarify standard-of-care docetaxel treatment prior to randomisation

Section 4.4.7. Clarification on concomitant medication and contra-indicated concomitant medications

Section 4.5. Clarification provided on tissue block collection

Section 6. Inclusion of docetaxel into the standard-of-care

Section 6.2.3 New section to describe standard-of-care docetaxel administration

Section 6.11. Improvement throughout sections and sub-sections for abiraterone and enzalutamide-related toxicity management

Section 6.12. Section improved throughout to incorporate clearer details on concomitant medications and drug-to-drug interactions

Section 7.1.4. New section to describe data collection for standard-of-care docetaxel

Section 9.7.4. Clarification provided about implications for “enzalutamide+ abiraterone comparison” following change of standard-of-care treatment

Section 11.2.1.D Clarification on SAE notification timelines to reflect change in standard-of-care treatments (addition of docetaxel)

Figure 1. Figure updated to reflect change in standard-of-care

Figure 2. Figure updated to reflect trial history and recruitment over time

Figure 3. Figure updated to reflect changes in standard-of-care and recruiting arms

Table 1. Table updated to remove repetition

Table 13. Table updated to include new CRF to report standard-of-care docetaxel treatment

Table 15. Table updated to include only active trial treatments

20 REFERENCES

1. James ND, Sydes MR, Mason MD, Clarke NW, Anderson J, Dearnaley DP, et al. Celecoxib plus hormone therapy versus hormone therapy alone for hormone-sensitive prostate cancer: first results from the STAMPEDE multiarm, multistage, randomised controlled trial. The lancet oncology. 2012 May-2012;13(5):549-58. PubMed PMID: 22452894. Pubmed Central PMCID: 22452894. Epub 26-Mar-2012.

2. Cancer Research UK. CancerStats Key Facts: Prostate Cancer. Cancer Research UK. 2011.

3. Sharifi N, Gulley JL, Dahut WL, Sharifi N, Gulley JL, Dahut WL. An update on androgen deprivation therapy for prostate cancer. Endocrine-Related Cancer. 2010 2010;17(4):R305-15. PubMed PMID: 20861285.

4. Widmark A, Klepp O, Solberg A, Damber JE, Angelsen A, Fransson P, et al. Endocrine treatment, with or without radiotherapy, in locally advanced prostate cancer (SPCG-7/SFUO-3): an open randomised phase III trial. Lancet. 2009 Jan 24;373(9660):301-8. PubMed PMID: 19091394. Epub 2008/12/19. eng.

5. Warde PR, Mason MD, Sydes MR, Gospodarowicz MK, Swanson GP, Kirkbride P, et al. Intergroup randomized phase III study of androgen deprivation therapy (ADT) plus radiation therapy (RT) in locally advanced prostate cancer (CaP) (NCIC-CTG, SWOG, MRC-UK, INT: T94-0110; NCT00002633). J Clin Oncol. 2010;28(18s Supplement: Proceedings of ASCO 2010):Abstr CRA4504.

6. Warde P, Mason M, Ding K, Kirkbride P, Brundage M, Cowan R, et al. Survival Benefit with Combined Androgen Deprivation and Radiation Therapy in Locally Advanced Prostate Cancer – Results of a Phase III Trial. The Lancet. 2011 - in press.

7. Mason M, Sydes M, Parulekar W, Parmar M, Anderson J, Barber J, et al. Final analysis of intergroup randomized phase III study of androgen deprivation therapy (ADT) + radiation therapy (RT) in locally advanced prostate cancer (CaP) (NCIC-CTG, SWOG, MRC-UK, INT: T94-0110). National Cancer Research Institute (NCRI) Cancer Conference 2012. 2012;2012.

8. Sweeney CJ, Chen YH, Carducci M, Liu G, Jarrard DF, Eisenberger M, et al. Chemohormonal Therapy in Metastatic Hormone-Sensitive Prostate Cancer. The New England journal of medicine. 2015 Aug 20;373(8):737-46. PubMed PMID: 26244877. Pubmed Central PMCID: 4562797.

9. Saad F, Colombel M. Management of castration-resistant prostate cancer: bisphosphonates and emerging therapies. Expert Review of Anticancer Therapy. 2010 Dec;10(12):1991-2002. PubMed PMID: 21110764.

10. Sartor O, Halstead M, Katz L, Sartor O, Halstead M, Katz L. Improving outcomes with recent advances in chemotherapy for castrate-resistant prostate cancer. Clinical Genitourinary Cancer. 2011 Dec 1;8(1):23-8. PubMed PMID: 21208852.

11. Attard G, Richards J, de Bono JS, Attard G, Richards J, de Bono JS. New strategies in metastatic prostate cancer: targeting the androgen receptor signaling pathway. Clinical Cancer Research. 2011 Apr 1;17(7):1649-57. PubMed PMID: 21372223.

12. Fizazi K, Scher HI, Molina A, Logothetis CJ, Chi KN, Jones RJ, et al. Abiraterone acetate for treatment of metastatic castration-resistant prostate cancer: final overall survival analysis of the COU-AA-301 randomised, double-blind, placebo-controlled phase 3 study. The Lancet Oncology. 2012 Oct;13(10):983-92. PubMed PMID: 22995653.

13. Rathkopf DE, Smith MR, de Bono JS, Logothetis CJ, Shore ND, de Souza P, et al. Updated interim efficacy analysis and long-term safety of abiraterone acetate in metastatic castration-resistant prostate cancer patients without prior chemotherapy (COU-AA-302). European urology. 2014 Nov;66(5):815-25. PubMed PMID: 24647231. Pubmed Central PMCID: 4418928.

14. Attard G, Reid AHM, de Bono JS. Abiraterone acetate is well tolerated without concomitant use of corticosteroids. Journal of Clinical Oncology. 2010 Oct 10;28(29):e560-1; author reply e2. PubMed PMID: 20805462. Pubmed Central PMCID: 20805462

15. Flanigan RC, Salmon SE, Blumenstein BA, Bearman SI, Roy V, McGrath PC, et al. Nephrectomy followed by interferon alfa-2b compared with interferon alfa-2b alone for metastatic renal-cell cancer. N Engl J Med. 2001 Dec 6;345(23):1655-9. PubMed PMID: 11759643. Epub 2002/01/05. eng.

16. Mickisch GH, Garin A, van Poppel H, de Prijck L, Sylvester R. Radical nephrectomy plus interferon-alfa-based immunotherapy compared with interferon alfa alone in metastatic renal-cell carcinoma: a randomised trial. Lancet. 2001 Sep 22;358(9286):966-70. PubMed PMID: 11583750. Epub 2001/10/05. eng.

17. Warde P, Mason M, Ding K, Kirkbride P, Brundage M, Cowan R, et al. Combined androgen deprivation therapy and radiation therapy for locally advanced prostate cancer: a randomised, phase 3 trial. The Lancet. 2011;378:2104-11. PubMed PMID: 22056152. Epub 03-Nov-2011.

18. Attard G, Cooper CS, de Bono JS. Steroid hormone receptors in prostate cancer: a hard habit to break? Cancer cell. 2009 Dec 8;16(6):458-62. PubMed PMID: 19962664. Epub 2009/12/08. eng.

19. Chen G, Wang X, Zhang S, Lu Y, Sun Y, Zhang J, et al. Androgen receptor mutants detected in recurrent prostate cancer exhibit diverse functional characteristics. The Prostate. 2005 Jun 1;63(4):395-406. PubMed PMID: 15617028. Epub 2004/12/24. eng.

20. Taplin ME, Bubley GJ, Ko YJ, Small EJ, Upton M, Rajeshkumar B, et al. Selection for androgen receptor mutations in prostate cancers treated with androgen antagonist. Cancer Res. 1999 Jun 1;59(11):2511-5. PubMed PMID: 10363963. Epub 1999/06/11. eng.

21. Veldscholte J, Ris-Stalpers C, Kuiper GG, Jenster G, Berrevoets C, Claassen E, et al. A mutation in the ligand binding domain of the androgen receptor of human LNCaP cells affects steroid binding characteristics and response to anti-androgens. Biochemical and biophysical research communications. 1990 Dec 14;173(2):534-40. PubMed PMID: 2260966. Epub 1990/12/14. eng.

22. Zhao XY, Malloy PJ, Krishnan AV, Swami S, Navone NM, Peehl DM, et al. Glucocorticoids can promote androgen-independent growth of prostate cancer cells through a mutated androgen receptor. Nature medicine. 2000 Jun;6(6):703-6. PubMed PMID: 10835690. Epub 2000/06/03. eng.

23. Attard G, Reid AH, Auchus RJ, Hughes BA, Cassidy AM, Thompson E, et al. Clinical and biochemical consequences of CYP17A1 inhibition with abiraterone given with and without exogenous glucocorticoids in castrate men with advanced prostate cancer. The Journal of clinical endocrinology and metabolism. 2012 Feb;97(2):507-16. PubMed PMID: 22170708. Epub 2011/12/16. eng.

24. Richards J, Lim AC, Hay CW, Taylor AE, Wingate A, Nowakowska K, et al. Interactions of abiraterone, eplerenone, and prednisolone with wild-type and mutant androgen receptor: a rationale for increasing abiraterone exposure or combining with MDV3100. Cancer Res. 2012 May 1;72(9):2176-82. PubMed PMID: 22411952. Epub 2012/03/14. eng.

25. Scher HI, Fizazi K, Saad F, Taplin ME, Sternberg CN, Miller K, et al. Increased survival with enzalutamide in prostate cancer after chemotherapy. The New England journal of medicine. 2012 Sep 27;367(13):1187-97. PubMed PMID: 22894553.

26. Tombal B, Borre M, Rathenborg P, Werbrouck P, Heidenreich A, Iversen P, et al. Enzalutamide monotherapy: Phase II study results in patients with hormone-naive prostate cancer. Journal of Clinical Oncology. 2013;31(Supplement 6):Abstract 18.

27. Efstathiou E, Titus MA, Tsavachidou A, Hoang A, Karlou M, Wen S, et al. MDV3100 effects on androgen receptor (AR) signaling and bone marrow testosterone concentration modulation: A preliminary report. . 2011 ASCO Annual Meeting, 2011 J Clin Oncol 2011.

28. Locke JA, Guns ES, Lubik AA, Adomat HH, Hendy SC, Wood CA, et al. Androgen levels increase by intratumoral de novo steroidogenesis during progression of castration-resistant prostate cancer. Cancer Res. 2008 Aug 1;68(15):6407-15. PubMed PMID: 18676866. Epub 2008/08/05. eng.

29. Tran C, Ouk S, Clegg NJ, Chen Y, Watson PA, Arora V, et al. Development of a second-generation antiandrogen for treatment of advanced prostate cancer. Science. 2009 May 8;324(5928):787-90. PubMed PMID: 19359544. Pubmed Central PMCID: 2981508. Epub 2009/04/11. eng.

30. Scher HI, Beer TM, Higano CS, Anand A, Taplin ME, Efstathiou E, et al. Antitumour activity of MDV3100 in castration-resistant prostate cancer: a phase 1-2 study. Lancet. 2010 Apr 24;375(9724):1437-46. PubMed PMID: 20398925. Pubmed Central PMCID: 2948179. Epub 2010/04/20. eng.

31. de Bono JS, Logothetis CJ, Molina A, Fizazi K, North S, Chu L, et al. Abiraterone and increased survival in metastatic prostate cancer. New England Journal of Medicine. 2011 May 26;364(21):1995-2005. PubMed PMID: 21612468.

32. Kouloulias V, Giraud J-Y, Davis BJ, Dusserre A, Zurlo A, Bolla M. Quality assurance of the 22961 EORTC trial. A phase III study of the optimal combination of hormonal adjuvant treatment by LHRH analogue and radiation therapy for the management of locally advanced prostate cancer: the dummy run. 2004 20041006 DCOM- 20050225(0167-8140 (Print)). eng.

33. Royston P, Parmar MKB, Qian W. Novel designs for multi-arm clinical trials with survival outcomes with an application in ovarian cancer. Statistics in Medicine. 2003;22(14):2239-56.

34. Royston P, Barthel FMS, Parmar MKB, Choodari-Oskooei B, Isham V. Designs for clinical trials with time-to-event outcomes based on stopping guidelines for lack of benefit. Trials. 2011;12(1):81.

35. Royston P. nstage: MAMS trial sample size calculator. MRC Clinical Trials Unit, London2009.

36. Haybittle JL. Repeated assessment of results in clinical trials of cancer treatment. The British journal of radiology. 1971 Oct;44(526):793-7. PubMed PMID: 4940475. Epub 1971/10/01. eng.

37. Peto R, Pike MC, Armitage P, Breslow NE, Cox DR, Howard SV, et al. Design and analysis of randomized clinical trials requiring prolonged observation of each patient. I. Introduction and design. British journal of cancer. 1976 Dec;34(6):585-612. PubMed PMID: 795448. Pubmed Central PMCID: 2025229. Epub 1976/12/01. eng.

38. Dolan P, Gudex C, Kind P, Williams A. Discussion Paper 138: A social tarif for EuroQol: results from a UK General Population Survey. . Centre for Health Economics. University of York, 1995.

STAMPEDE

Systemic Therapy in Advancing or Metastatic Prostate Cancer: Evaluation of Drug Efficacy

A multi-arm multi-stage randomised controlled trial

**Version:**	**15.0**
**Date:**	**29 -Mar-2016**

MRC CTU ID:	PR08
ISRCTN #:	ISRCTN78818544
NCT #:	NCT00268476
EUDRACT #:	2004-000193-31
CTA #:	00316/0026/001-0001
MREC #:	04/MRE07/35

Authorised by:

Name:	PROFESSOR NICHOLAS D JAMES
Role:	CHIEF INVESTIGATOR
Signature:	

Name:	MATTHEW SYDES
Role:	TRIAL STATISTICIAN

GENERAL INFORMATION

This document was constructed using the MRC CTU Protocol Template Version 4.0. It describes the STAMPEDE trial, coordinated by the Medical Research Council (MRC) Clinical Trials Unit (CTU) at University College London (UCL), and provides information about procedures for entering patients into it. The protocol should not be used as an aide-memoire or guide for the treatment of other patients. Every care has been taken in drafting this protocol, but corrections or amendments may be necessary. These will be circulated to the registered investigators in the trial, but sites entering patients for the first time are advised to contact the Cancer And Other Non-Infectious Diseases Group, MRC CTU at UCL, London, UK, to confirm they have the most up-to-date version.

COMPLIANCE

The trial will be conducted in compliance with the approved protocol, the Declaration of Helsinki 1996, the principles of Good Clinical Practice (GCP), Commission Directive 2005/28/EC with the implementation in national legislation in the UK by Statutory Instrument 2004/1031 and subsequent amendments, the UK Data Protection Act (DPA number: Z5886415), and the National Health Service (NHS) Research Governance Framework for Health and Social Care (RGF). International sites will comply with the principles of GCP as laid down by the ICH topic E6 (Note for Guidance on GCP), Commission Directive 2005/28/EC (the European Directive 2001/20/EC [where applicable]) and applicable national regulations.

SPONSOR

Medical Research Council, 2nd Floor, David Phillips Building, Polaris House, North Star Avenue, Swindon, SN2 1FL, UK

FUNDING

Clinical Trials Advisory Awards Committee (on behalf of Cancer Research UK, Medical Research Council, and other charities) together with educational grants from Novartis, Sanofi-Aventis, Pfizer, Janssen Pharma NV, Astellas.

AUTHORISATIONS AND APPROVALS

The following persons are authorised to sign the final protocol and protocol amendments for the sponsor: Professor Nicholas James (Chief Investigator) and Matthew Sydes (Trial Statistician).

TRIAL REGISTRATION

This trial has been registered with the ClinicalTrials.gov Clinical Trials Register, where it is identified as NCT00268476.

RANDOMISATIONS
To randomise, call MRC CTU at UCL, Monday to Friday 0900-1700
excluding public holidays or dates when notice has been given by the Unit.
Tel: +44 (0) 20 7670 4777

SAE REPORTING
Fax to 020 7670 4818 within 24 hours of becoming aware of the event

TRIAL ADMINISTRATION

COORDINATING SITE

MRC Clinical Trials Unit at UCL	Switchboard:	020 7670 4700
Aviation House	Fax:	020 7670 4818
125 Kingsway London	Email:	mrcctu.stampede@ucl.ac.uk
WC2B 6NH		
UK		

MRC CTU AT UCL STAFF

Trial Manager:	Francesca Schiavone	Tel:	0207 670 4632
Trial Manager:	Orla Prendiville	Tel:	0207 670 4840
Trial Manager	Claire Murphy	Tel:	0207 670 4620
Trial Manager	Katie Ward	Tel:	0207 670 4916
Data Manager:	Carly Au	Tel:	0207 670 4794
Data Manager:	Zohrah Khan	Tel:	0207 670 4947
Data Manager:	Peter Vaughan	Tel:	0207 670 4655
Data Manager:	Nargis Begum	Tel:	0207 670 4693
Data Manager:	Jenna Grabey	Tel:	0207 670 4626
Clinical Project Manager:	Claire Amos	Tel:	0207 670 4771
Statistician:	Melissa Spears	Tel:	0207 670 4764
Senior Trial Statistician:	Matthew Sydes	Tel:	0207 670 4798
Director of MRC CTU at UCL:	Max Parmar	Tel:	Please contact STAMPEDE team
Trial Surgeon:	Alastair Ritchie	Tel:	Please contact STAMPEDE team
Clinical Research Fellow	Clare Gilson	Tel:	Please contact STAMPEDE team

CHIEF INVESTIGATOR

Prof Nicholas James
Warwick Medical School
University of Warwick
Coventry, CV4 7AL
UK
Tel: 024 7657 4365 (Secretary)
Email: n.d.james@warwick.ac.uk

CO-INVESTIGATORS

Prof Noel Clarke<br>Urologist	Manchester, UK	Vice-Chair<br>Co-lead of "Metformin comparison"
Prof Malcolm Mason<br>Oncologist	Cardiff, UK	Vice-Chair
Dr Gerhardt Attard<br>Oncologist	London, UK	Lead:"Enzalutamide+Abiraterone comparison"
Mr William Cross<br>Urologist	Leeds, UK	
Prof David Dearnaley<br>Oncologist	London, UK	
Prof Rob Jones<br>Oncologist	Glasgow, UK	
Dr Chris Parker<br>Oncologist	London, UK	Lead: "M1|RT comparison"
Dr Martin Russell<br>Oncologist	Glasgow, UK	
Prof George Thalmann<br>Urologist	Bern, Switzerland	
Prof Silke Gillessen<br>Oncologist	St Gallen, Switzerland	Co-lead of "Metformin comparison"
Dr Amanda Adler<br>Diabetologist	Cambridge, UK	Diabetologist "Metformin comparison"

Patient Representatives

Robin Millman

David Matheson

Health Economics

Prof Mark Sculpher<br>Health Economics Advisor	York, UK

SAAK Coordinating Centre

Estelle Cassoly	Bern, Switzerland

Trial Expert Groups

Biological Research Group	Prof Malcolm Mason (Chair)
Metabolic Translational Group	Prof Noel Clarke (Chair)

CONTENTS

GENERAL INFORMATION 2

CONTENTS 5

ABBREVIATIONS 10

1 SUMMARY 13
1.1 LAY SUMMARY 13
1.2 ABSTRACT AND SUMMARY OF TRIAL DESIGN 14
1.3 TRIAL DOCUMENTATION 17

2 BACKGROUND 18
2.1 INTRODUCTION AND SETTING 18
2.1.1 Long-term Androgen Deprivation Therapy 18
2.1.2 Role of Radiotherapy for Men With M0 Disease 18
2.2 RATIONALE 19
2.3 DESIGN 19
2.4 RESEARCH TREATMENT: BISPHOSPHONATES 20
2.5 RESEARCH TREATMENT: CHEMOTHERAPY 20
2.6 RESEARCH TREATMENT: CYCLOOXYGENASE-2 INHIBITORS 20
2.7 RESEARCH TREATMENT: STEROID SYNTHESIS INHIBITORS 20
2.8 RESEARCH TREATMENT: RADIOTHERAPY TO THE PROSTATE FOR PATIENTS WITH NEWLY-DIAGNOSED METASTATIC DISEASE 21
2.9 RESEARCH TREATMENT: COMBINATIONS OF ORIGINAL RESEARCH ARMS 22
2.9.1 Bisphosphonate And Chemotherapy 22
2.9.2 Bisphosphonate And Cyclooxygenase-2 Inhibitors 22
2.10 COMBINATION OF STEROID SYNTHESIS INHIBITORS AND ANDROGEN RECEPTOR SIGNALLING INHIBITOR ... 22
2.10.1 Supplementing Abiraterone And Prednisolone With Enzalutamide 23
2.10.2 Supplementing Enzalutamide With Abiraterone And Prednisolone 23
2.10.3 Summary Of Rationale For This Combination 23
2.11 RATIONALE FOR THE "METFORMIN COMPARISON" 24

3 SELECTION OF INSTITUTIONS AND INVESTIGATORS 26
3.1 RADIOTHERAPY ACCREDITATION 26

4 SELECTION OF PATIENTS 27
4.1 GENERAL INCLUSION CRITERIA 27
4.1.1 High-Risk Newly-Diagnosed Non-Metastatic Node-Negative Disease 27
4.1.2 Newly-Diagnosed Metastatic Or Node-Positive Disease 27
4.1.3 Previously Radically Treated, now relapsing (prior Radical Surgery And/Or Radiotherapy) ... 27
4.1.4 For All Patients 27
4.2 GENERAL EXCLUSION CRITERIA 28
4.3 SELECTION CRITERIA FOR COMPARISON OF RESEARCH RT FOR METASTATIC DISEASE (M1|RT) 28
4.4 SELECTION CRITERIA FOR METFORMIN COMPARISON 29
4.5 SCREENING PROCEDURES 29
4.5.1 Investigations Prior To Randomisation 29
4.5.2 Additional baseline Investigations 30

4.5.3 Androgen Deprivation Therapy Prior To Randomisation 30
4.5.4 Choice About Standard-Of-Care Docetaxel 31
4.5.5 Starting Trial Treatment 31
4.5.6 Concomitant Medications 31
4.6 ADDITIONAL DETAILS FOR PATIENTS JOINING SUB-STUDIES 32

5 RANDOMISATION AND ENROLMENT 33
5.1 CO-ENROLMENT GUIDELINES 33

6 TREATMENT OF PATIENTS 34
6.1 TRIAL TREATMENT 34
6.2 ARM A: STANDARD-OF-CARE 34
6.2.1 Hormone Therapy 34
6.2.2 Standard-of-care (M0) RT 34
6.3 ARM B: ADT + ZOLEDRONIC ACID 35
6.4 ARM C: ADT + DOCETAXEL 36
6.5 ARM D: ADT + CELECOXIB 36
6.6 ARM E: ADT + DOCETAXEL + ZOLEDRONIC ACID 36
6.7 ARM F: ADT + ZOLEDRONIC ACID + CELECOXIB 36
6.8 ARM G: SOC + ABIRATERONE 36
6.9 ARM H: SOC + PROSTATE RADIOTHERAPY IN M1 PATIENTS 37
6.9.1 Research (M1) Prostate Radiotherapy Treatment Administration 37
6.10 ARM J: SOC + ENZALUTAMIDE + ABIRATERONE + PREDNISOLONE 39
6.11 ARM K: SOC + METFORMIN 40
6.12 ADMINISTRATION, DOSE MODIFICATIONS AND MANAGEMENT OF TOXICITIES 41
6.12.1 Zoledronic Acid or Docetaxel (arms B, C and E) 41
6.12.2 Celecoxib (arms D and F) 41
6.12.3 Abiraterone or Enzalutamide + Abiraterone (arms G and J) 41
6.12.4 Metformin 47
6.13 CONCOMITANT MEDICATIONS AND DRUG INTERACTION 50
6.13.1 Abiraterone: Interaction with other medicinal products and other forms of interaction 50
6.13.2 Enzalutamide: Interaction with other medicinal products and other forms of interaction 51
6.13.3 Metformin: Interaction with other medicinal products and other forms of interaction 54
6.14 TRIAL PRODUCTS 54
6.15 MEASURES OF COMPLIANCE/ADHERENCE 54
6.16 TREATMENT DATA COLLECTION 54
6.17 ADMINISTRATION OF STANDARD RADIOTHERAPY TO NON-METASTATIC PATIENTS 55
6.17.1 Treatment Details 55
6.18 NON-TRIAL TREATMENT 56
6.18.1 Medications Permitted 56
6.18.2 Data On Concomitant Medication 56

7 ASSESSMENTS AND PROCEDURES 57
7.1 SCHEDULE FOR ASSESSMENTS 57
7.1.1 PSA Measurements 57
7.1.2 Assessment Of Treatment Failure (Definition Of Progression) 57
7.1.2.A Additional Metabolic and Cardiovascular Outcomes 58
7.1.3 Additional Safety Assessment 59
7.1.4 Data collection for standard docetaxel 60
7.1.5. Data Collection and Non-Administration Of Standard Radiotherapy 60
7.1.6. Data Collection Palliative Radiotherapy 60

7.1.7 Data Collection Research (M1) Radiotherapy 61
7.1.8. Follow-Up Schedules 61
7.2 FOLLOW-UP 61
7.2.1 Follow-Up Telephone Consultations 61
7.3 TRIAL CLOSURE 62

8 STOPPING OF TREATMENT OR FOLLOW-UP 65
8.1 STOPPING RESEARCH INTERVENTIONS 65
8.2 PATIENT TRANSFERS 65
8.3 EARLY CESSATION OF TRIAL PARTICIPATION 66

9 STATISTICAL CONSIDERATIONS 67
9.1 METHOD OF RANDOMISATION 67
9.2 OUTCOME MEASURES 67
9.3 SAMPLE SIZE: PRINCIPLES 68
9.4 SAMPLE SIZE ISSUES AND TRIAL STAGES: RESEARCH ARMS B-F 69
9.5 SAMPLE SIZE ISSUES AND TRIAL STAGES: ADDITIONAL RESEARCH ARM G 69
9.5.1 Pilot Phase: Additional Research Arm G 69
9.5.2 Activity Stages I-III: Additional Research Arm G 69
9.5.3 Efficacy Stage IV: Additional Research Arm G 69
9.5.4 Sample Size For Additional Research Arm G 70
9.6 SAMPLE SIZE ISSUES AND TRIAL STAGES: ADDITIONAL RESEARCH ARM H 70
9.6.1 Pilot Phase: Additional Research Arm H 70
9.6.2 Activity Stages I-III: Additional Research Arm H 70
9.6.3 Efficacy Stage IV: Additional Research Arm H 70
9.6.4 Sample Size For Additional Research Arm H 71
9.7 SAMPLE SIZE ISSUES AND TRIAL STAGES: ADDITIONAL RESEARCH ARM J 72
9.7.1 Pilot Phase: Additional Research Arm J 72
9.7.2 Activity Stages I-II: Additional Research Arm J 72
9.7.3 Efficacy Stage III: Additional Research Arm J 73
9.7.4 Sample Size For Additional Research Arm J 73
9.7.5 Further Sample Size Issues For Additional Research Arm J 73
9.8 SAMPLE SIZE ISSUES AND TRIAL STAGES: ADDITIONAL RESEARCH ARM K 74
9.8.1 Implementation: Additional Research Arm K 74
9.8.2 Outcome Measures: Additional Research Arm K 74
9.8.3 Pilot Phase: Additional Research Arm K 75
9.8.4 Activity Stage I: Additional Research Arm K 75
9.8.5 Efficacy Stage II: Additional Research Arm K 76
9.8.6 Sample Size For Additional Research Arm K 76
9.8.7 Further Sample Size Issues For Additional Research Arm K 76
9.9 FURTHER NOTES ON TRIAL DESIGN 78
9.9.1 Overall Sample Size 78
9.9.2 Factorial Design 78
9.10 INTERIM MONITORING AND ANALYSES 78
9.11 OUTLINE ANALYSIS PLAN 78
9.11.1 Pilot / Safety Phases 79
9.11.2 Activity And Efficacy Stages 79

10 MONITORING AND QUALITY ASSURANCE 80
10.1 MONITORING AT MRC CTU 80
10.2 DIRECT ACCESS TO DATA 80

10.3 VISITS TO INVESTIGATOR SITES 80
10.4 CONFIDENTIALITY 80

11 SAFETY REPORTING 81
11.1 DEFINITIONS 81
11.1.1 Trial-Specific Exemptions 82
11.2 INSTITUTION/INVESTIGATOR RESPONSIBILITIES 82
11.2.1 Investigator Assessment 82
11.2.2 Notification Procedure 84
11.3 MRC CTU RESPONSIBILITIES 84

12 ETHICAL CONSIDERATIONS AND APPROVAL 86
12.1 ETHICAL CONSIDERATIONS 86
12.2 ETHICAL APPROVAL 87

13 REGULATORY APPROVAL 88

14 INDEMNITY 89

15 FINANCE 90

16 TRIAL COMMITTEES 91
16.1 TRIAL MANAGEMENT GROUP (TMG) 91
16.2 TRIAL STEERING COMMITTEE (TSC) 91
16.3 INDEPENDENT DATA MONITORING COMMITTEE (IDMC) 91
16.4 TRIAL EXPERT PANELS 91

17 ANCILLARY STUDIES 93
17.1 QUALITY OF LIFE 93
17.2 HEALTH ECONOMICS 93
17.3 TRANSLATIONAL SUB-STUDIES 93
17.3.1 DNA Analysis 93
17.3.2 Analysis of tumour Tissue 94

18 PUBLICATIONS 95

19 PROTOCOL AMENDMENTS 96
19.1 PROTOCOL 96
19.1.1 Amendments Made To Sections In Protocol Version 1.0 (May 2004) 96
19.1.2 Amendments Made To Sections In Protocol Version 1.1 (May 2005) 96
19.1.3 Amendments Made To Sections In Protocol Version 2.0 (Jun 2005) 96
19.1.4 Amendments Made To Section In Protocol Version 3.0 (Jul 2006) 97
19.1.5 Amendments Made To Protocol Version 4.0 (Dec 2007) 98
19.1.6 Amendments Made To Protocol Version 5.0 (Aug 2008) 98
19.1.7 Amendments Made To Protocol Version 6.0 (Jul 2009) 99
19.1.8 Amendments Made To Protocol Version 7.0 (Jul 2011) 99
19.1.9 Amendments Made To Protocol Version 7.1 (Jul 2011) 100
19.1.10 Amendments Made To Protocol Version 8.0 (Sep 2011) 101
19.1.11 Amendments Made To Protocol Version 9.0 (Oct-2012) 102
19.1.12 Amendments Made To Protocol Version 10.0 (Apr-2013) 103

19.1.13 Amendments Made To Protocol Version 11.0 (Sep-2013) 103
19.1.14 Amendments Made To Protocol Version 12.0 (Jan-2014) 103
19.1.15 Amendments Made To Protocol Version 13.0 (FEB-2014) 104
19.1.16 Amendments Made To Protocol Version 14.0 (Oct-2015) 104

20 REFERENCES 106

ABBREVIATIONS

Abbreviation	Expansion
ACE	Angiotensin-Converting Enzyme
ACTH	Adrenocorticotropic hormone
ADT	Androgen deprivation therapy
AR	Androgen receptor
AS	Activity Stage
bid	Twice a day (bis in die)
BP	Blood pressure
BRG	Biological Research Group
BSA	Body surface area
CF	Consent Form
CI	Chief Investigator
CI	Confidence interval
Cox-2	Cyclooxygenase 2
CRF	Case Report Form
CRUK	Cancer Research UK
CRPC	Castrate Refractory Prostate Cancer
CT	Computerised tomography
CTA	Clinical Trials Authorisation
CTAAC	Clinical Trials Advisory and Awards Committee
CTC	Common Toxicity Criteria
CTU	Clinical Trials Unit
CTV	Clinical Tumour Volume
CXR	Chest X-ray
DDX	Doctors and Dentists Exemption
DHT	Dihydrotestosterone
DNA	Deoxyribonucleic Acid
DPA	Data Protection Act
ERC	Endpoint Review Committee
ES	Efficacy Stage
ICH	International Conference on Harmonization
ECG	Electro cardiogram
FBC	Full Blood Count

Abbreviation	Expansion
FFS	Failure-Free Survival
GCP	Good Clinical Practice
GP	General Practitioner
GRO	General Register Office
HbA1c	Glycated haemaglobin
Hb	Haemoglobin
HE	Health Economics
HES	Hospital Episode Statistics
Hr	Hour
HR	Hazard Ratio
HRPC	Hormone Refractory Prostate Cancer
HSCIC	Health & Social Care Information Centre
HT	Hormone Therapy
IDMC	Independent Data Monitoring Committee
IM	Intramuscular
IMRT	Intensity Modulated Radiation Therapy
ISRCTN	International Standard Randomised Controlled Trial Number
IU	International Units
IV	Intravenous
LFTs	Liver Function Tests
LHRH	Luteinising Hormone Releasing Hormone
LREC	Local Research Ethics Committee
m	Month
MHRA	Medicine and Healthcare Products Regulatory Agency
min	Minutes
MRC	Medical Research Council
MREC	Multi-Centre Research Ethics Committee
MRI	Magnetic resonance imaging
M0	Non-metastatic
M1	Metastatic
NCI	National Cancer Institute (USA)
NCRN	National Cancer Research Network
NHS	National Health Service
NSAID	Non-Steroidal Anti-inflammatory Drugs

Abbreviation	Expansion
ONS	Office for National Statistics
OS	Overall Survival
PI	Principal Investigator
PIS	Patient Information Sheet
po	per orum (orally)
PSA	Prostate Specific Antigen
pts	Patients
PTV	Planned Tumour Volume
QALY	Quality-adjusted Life Years
qds	quater die sumendus (4 times each day)
QL	Quality of Life
R&D	Research and Development
RECIST	Response Evaluation Criteria In Solid Tumours
SAE	Serious Adverse Event
SAR	Serious Adverse Reaction
sc	Sub-cutaneous (under skin)
SNP	Single Nucleotide Polymorphism
SOC	Standard-of-Care
SSA	Site Specific Assessment
STAMPEDE	Systemic Therapy in Advancing and Metastatic Prostate Cancer: Evaluation of Drug Efficacy
SUSAR	Suspected Unexpected Serious Adverse Reactions
SWOG	South West Oncology Group
TMG	Trial Management Group
TMT	Trial Management Team
TURP	Trans-Urethral Resection of Prostate
TSC	Trial Steering Committee
UCL	University College London
ULN	Upper Limit of Normal
U+E	Urea and Electrolytes
WHO	World Health Organisation

1 SUMMARY

1.1 LAY SUMMARY

Prostate cancers depend upon the male hormone testosterone for their growth. Lowering testosterone levels (either by removing all or part of both testes, or by giving anti-hormone treatment) slows the growth of prostate cancers. This type of treatment is called hormone treatment or androgen deprivation therapy (ADT) and is often used when prostate cancers have spread outside the prostate gland. Although hormone treatment is usually successful at stopping the cancer growing for a period of time, the cancer will begin to grow again in most men.

There are increasing numbers of treatments available for advanced prostate cancer. These treatments are usually used in prostate cancer when hormone treatment is no longer effective and the cancer has started to grow again. The aim of this trial, which is called STAMPEDE, is to assess some of these treatments, given earlier in the course of the disease in combination with the current standard-of-care.

The treatments that have been, or are being, assessed during the trial are:

1. Zoledronic acid (now closed to recruitment)**:** Prostate cancer cells can spread to bones and weaken them. Zoledronic acid is a drug that reduces bone destruction and hardens bones cells. Recruitment to this treatment has been completed and the results show that the addition of zoledronic acid does not prolong survival.

2. Docetaxel (now closed to recruitment):**:** A drug that stops cells replicating that is currently being used to treat a range of cancers including lung, breast and ovarian cancer as well as prostate cancer. Docetaxel prolongs survival in men with relapsed metastatic prostate cancer. Recruitment to this treatment has been completed and the results show that the addition of docetaxel to hormone treatment does improve survival in men with metastatic disease and delays the time to progression for men with locally advanced and metastatic disease. Docetaxel may now be given as part of standard treatment to all men entering STAMPEDE (from Protocol version 14.0).

3. Celecoxib (now closed to recruitment):**:** An aspirin-like drug that is used to treat arthritis. It slows down the growth of cancer cells in the laboratory. We wished to see if it had the same effect on cancer cells in patients. Recruitment stopped early as a planned intermediate analysis failed to demonstrate sufficient effect of this drug.

4. Abiraterone (in single use included from Protocol version 8.0; in combination with enzalutamide included from Protocol 12.0)**:** An inhibitor of steroid hormone synthesis that blocks prostate cancer cells from generating their own male hormones. This is thought to be a major way in which prostate cancer cells resume growth following anti-hormonal therapies. Abiraterone has been shown to prolonging surival in men with advanced disease when given before and after chemotherapy.

5. Prostate radiotherapy (included from Protocol version 9.0)**:** treatment with high-energy x-rays targeted to the prostate gland. This treatment is now mandatory for patients with cancer that is confined to the prostate gland as large trials have shown it improves survival times. We are not certain whether we should give radiotherapy to the prostate if the cancer has already spread and so we are investigating this in STAMPEDE. Recruitment to this treatment group is ongoing.

6. Enzalutamide (in combination with abiraterone included from Protocol version 12.0)**:** This is a blocker of androgen receptors. These stimulate the cancer when hormone therapies have failed. Enzalutamide may be mutually complementary to abiraterone in terms of blocking mechanisms of resistance. The agent prolongs survival when given to men following failure of docetaxel chemotherapy. Recruitment to this treatment group is now completed.

7. **Metformin** (included from Protocol version 15.0): This anti-diabetic medication is proposed to have both anti-cancer effects and may help prevent the adverse metabolic effects of long term ADT. STAMPEDE will investigate whether adding metformin to the current standard-of-care for non-diabetic men can improve all-cause survival.

STAMPEDE will look at the effect of combining one or two of the treatments described above with hormone treatment. A computer program will be used to allocate which treatment each participant receives, using a chance process. The trial will look at the effects of the combined treatments on quality of life and find out whether the new treatment combinations control prostate cancer growth and enable men to live longer. The study will also look at which treatment provides the greater value for money for the health service. More than 9,000 men will join the trial with answers becoming available throughout the trial.

1.2 ABSTRACT AND SUMMARY OF TRIAL DESIGN

STAMPEDE is a multi-centre, randomised controlled trial for patients with locally advanced or metastatic prostate cancer who are commencing long-term Androgen Deprivation Therapy (ADT). Participants can have either newly diagnosed disease, or have been previously treated with radical radiotherapy or surgery but now have signs of progression such as a rising prostate specific antigen (PSA) (further details on eligibility see **Section 4**). The trial will assess the effects of adding different agents, both as single agents and in combinations, to the standard-of-care.

When the trial opened in 2005 there were five "original comparisons" which included the following investigational agents (i) a bisphosphonate, zoledronic acid, (ii) a cytotoxic chemotherapeutic agent, docetaxel and (iii) a cyclooxygenase (Cox-2) inhibitor, celecoxib. The results of these comparisons have now been presented.

Since then, the trial has been amended to include additional research arms in order to evaluate(iv) abiraterone, a steroid synthesis inhibitor and (v) enzalutamide, an inhibitor of androgen receptor signalling. A further research arm involving prostate radiotherapy for patients with newly-diagnosed metastatic disease was added (Protocol version 9.0).

From Protocol version 15.0, (vi) metformin, an anti-diabetic medication will be evaluated (Arm K) and recruitment to the "enzalutamide and abiraterone comparison" (Arm J) has now closed. The current arms open to recruitment are therefore Arm H (Prostate RT for M1 patients) and Arm K ("metformin comparison").

The trial has multiple arms; the control arm of the trial receives standard therapy alone. When the trial started standard treatment was androgen deprivation therapy (ADT) only, achieved through the use of luteinising hormone releasing hormone (LHRH) analogues or antagonist or bilateral orchidectomy according to local practice. Since primary results from the trial "original comparisons" have emerged showing a benefit in overall survival for patients receiving docetaxel in addition to ADT, the standard treatment has changed accordingly. Standard treatment may now include

docetaxel chemotherapy for all men entering STAMPEDE. Radiotherapy is also mandated for men with node negative non-metastatic disease. The current trial design is shown in **Figure 1;** previous trial designs can be viewed in Protocol version 13.0.

Figure 1: Arms of the STAMPEDE trial from Protocol version 15.0

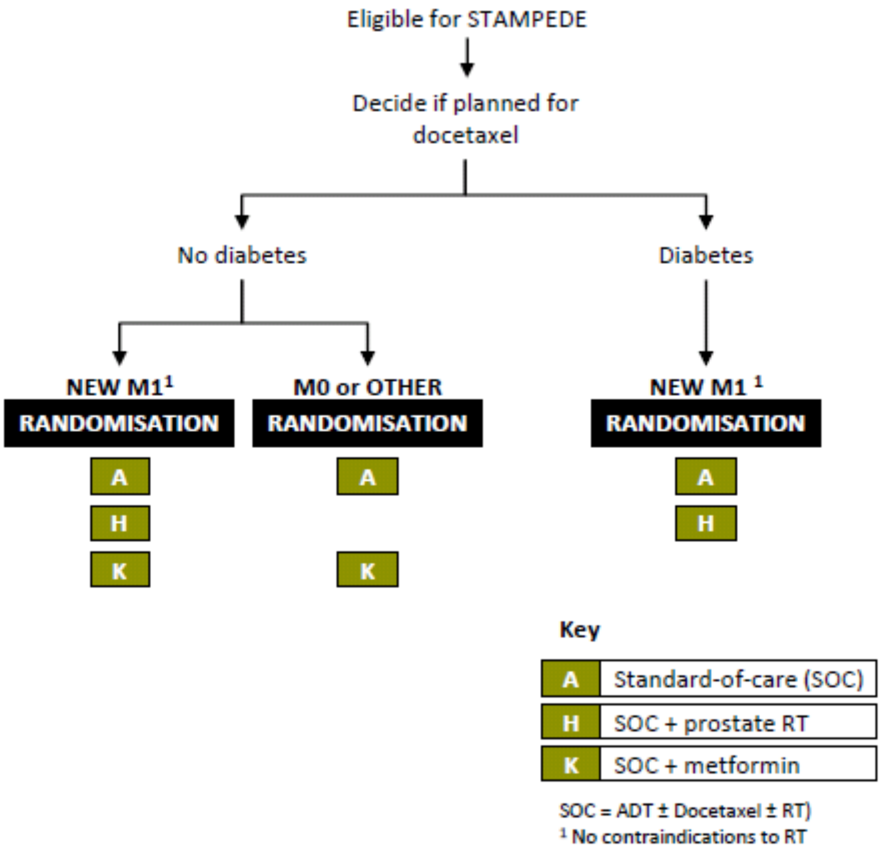

Figure 2: Arms of the STAMPEDE trial open to recruitment over time

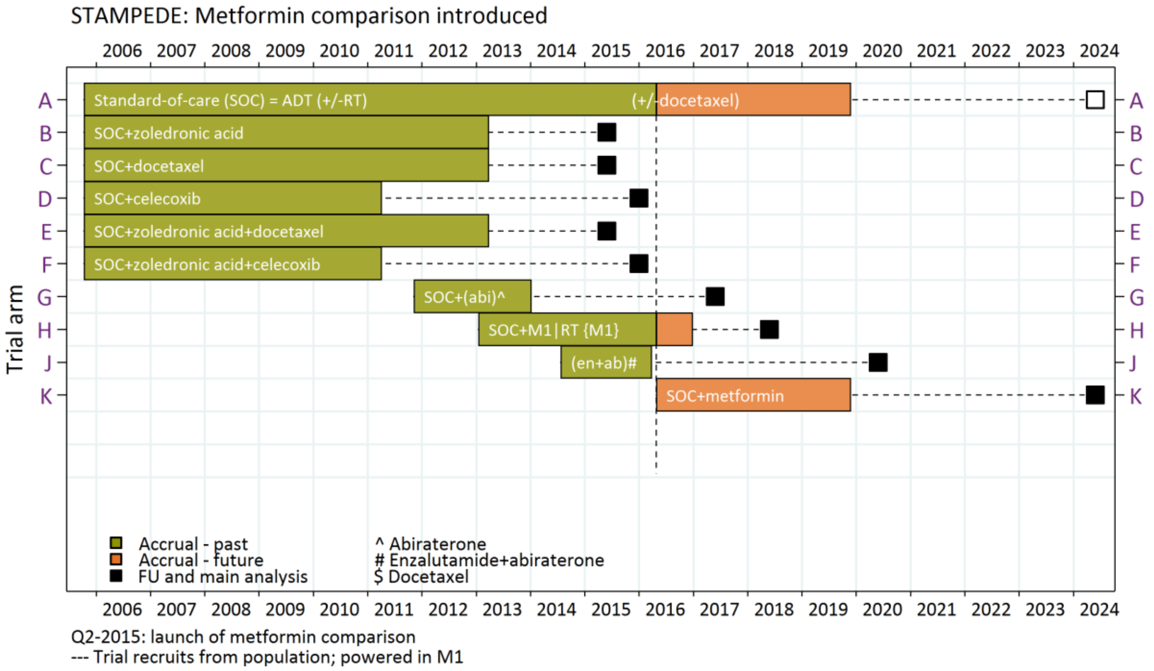

For each comparison of research arm against control, the trial will be conducted in a number of stages: a Pilot/Safety Phase, Activity Stages and a final Efficacy Stage. The primary outcome measure of the Pilot/Safety Phase is safety, with 30-50 patients recruited to each research arm. Research arms will only continue to recruitment in the next stage if they have been shown to be both safe and feasible, although patient data from all patients and all stages will be included in the final analyses. In the Activity Stages the primary outcome measure is failure-free survival (FFS). Each Activity Stage is triggered when a pre-specified number of FFS events have been observed in the control arm of the relevant comparison (see **Section 9** for further detail). Recruitment to Arms D (ADT + celecoxib) and F (ADT + zoledronic acid + celecoxib) was stopped in Apr-2011 after the second planned activity analysis when the IDMC and TSC considered the lack-of-benefit guidelines.(1) (See to **Section 9** for further information regarding the guidelines for stopping accrual to research arms during the activity stages of the trial).

Some evidence of activity will be required for a research arm to continue past each stage and guidelines are in place for this assessment of activity. The Efficacy Stage will take place when a pre-specified number of deaths are observed amongst the control arm patients for that relevant comparison. This was when around 403 deaths had been reported in the control arm for the "original comparisons" (involving docetaxel and zoledronic acid) and will be when around 267 deaths are reported in the control arm for the "abiraterone comparison", the "M1|RT comparison" and the "enzalutamide+abiraterone comparison". The exact number of patients randomised to, and duration of, the trial will depend on the observed accrual rate, observed event rate and the number of other research arms open to recruitment.

In Protocol version 8.0 a new Arm G (ADT + abiraterone) was added. Arm H (ADT+ prostate radiotherapy) was added in Protocol version 9.0. The trial stages remain similar to those at trial inception but will be staggered in time compared to the stages for the original Arms A-F. Protocol version 10.0 was approved following the completion of recruitment to the remaining original trial arms (B, C and E) and was a "housekeeping" change to remove references to the completed arms from the information sheets. Protocol version 11.0 was approved following the extension of the

recruitment target sample for the “abiraterone comparison” from 1,500 to around 1,800 patients. Protocol version 12.0 added a new combination therapy arm containing abiraterone with enzalutamide; for this comparison we envisage only two pre-planned interim analyses. Protocol version 13.0 was approved following the extension of the recruitment target sample for the "M1|RT comparison", from 1,250 to around 1,800 patients, and the introduction of saliva sample collection for DNA analysis. In response to the results of the primary analysis of the "original comparisons" the standard-of-care was updated to permit docetaxel in Protocol version 14. Protocol version 15.0 includes the addition of the metformin comparison as a new research arm, open to all non-diabetic men and reflects the completion of accrual to the “enzalutamide + abiraterone comparison”.

Patients will be assessed 6 weekly for the first 24 weeks after randomisation and then every 12 weeks up to 2 years, 6-monthly until 5 years and annually, thereafter. Quality of Life (QL) and use of health care resources (Health Economics) data is being collected in all patients until first Failure Free Survival event is reached or trial treatment completed. Please see **Section 17** for more details.

In addition, there are translational sub-studies. Patients willing to participate will be asked at randomisation to donate a saliva sample (previously a droplet of blood), which will be stored for DNA and protein analysis to try to identify markers that are associated with response to therapy, side-effects or susceptibility to prostate cancer.

Patients will also be asked for permission to use some of their stored material (e.g. tissue samples obtained at prostate biopsy or surgery) for further studies aiming to understand the causes and nature of prostate cancer and to identify biomarkers of treatment response. For further details of ancillary studies, see **Section 17**.

1.3 TRIAL DOCUMENTATION

Table 1 presents a summary of the required trial documentation for participating centres

Table 1: Trial documentation required for participating centres

TRIAL DOCUMENTATION	TIMING
R&D approval (including IRMER approval)	Before centre participation
Investigator Statement	Before centre participation
Signature list & delegation of responsibilities	Before centre participation
Trial personnel contact details	Before centre participation
PIS, GP & CF on local paper	Before centre participation
Signed Clinical Trial Agreement between Trust and Sponsor (or Variation if applicable)	Before centre participation
RTQA accreditation	Before centre participation
Site initiation training	Before centre participation
Pharmacy Pack acknowledgment	Before centre participation

2 BACKGROUND

2.1 INTRODUCTION AND SETTING

Prostate cancer is a major health problem world-wide and accounts for nearly one fifth of all newly diagnosed male cancers. In the UK, approximately 41,000 men are diagnosed with prostate cancer each year and in 2012 over 10,000 men died from the disease.(2)

2.1.1 LONG-TERM ANDROGEN DEPRIVATION THERAPY

The initial (first line) treatment for locally advanced or metastatic prostate cancer is androgen deprivation therapy (ADT) achieved either surgically with bilateral orchidectomy, or medically with LHRH agonists or antagonists (3). Oral anti-androgens are no longer permitted for new patients within the trial from Protocol version 8.0.

ADT produces responses in up to 95% of patients but it is not curative and disease recurs in virtually all patients treated with ADT as sole therapy, with a median time to progression of 18-24 months (3). Data from the control arm in STAMPEDE has shown that for men with newly-diagnosed metastatic disease time to progression is just 11 months. Such progressive disease is referred to as castrate resistant prostate cancer (CRPC); although this term is unpopular with patient groups due to its perceived pejorative overtones related to castration and hence terminology may yet change again in the future.

2.1.2 ROLE OF RADIOTHERAPY FOR MEN WITH M0 DISEASE

Two randomised trials, SPCG7 (4) and NCIC PR.3 / MRC PR07 (5-7) have tested the question of whether ADT alone combined with radiotherapy is the best treatment for high-risk patients with no evidence of spread outside the pelvis. Both trials demonstrated an improvement in overall and disease specific survival from the addition of radiotherapy to ADT. The size of this overall survival benefit is substantial (hazard ratio 0.68 in SPCG7 and HR 0.77 in PR07). With substantial benefit demonstrated in two mature, large, well conducted randomised trials, we now mandate that radiotherapy be standard for patients with no nodal or metastatic spread. Patients in this category will now only be allowed to enter the trial if standard radiotherapy is planned, with the exception of those for whom radiotherapy is contra-indicated. Such patients should be discussed with the Trials Unit prior to inclusion. For patients with node positive, M0 disease there are no clear data on whether radiotherapy is indicated or not. The NCIC PR.3 / MRC PR07 trial included patients with unknown nodal status who received whole pelvic radiotherapy (8). Given the large overall benefit observed in this trial, the STAMPEDE TMG recommends that pelvic nodal radiotherapy be considered for patients with node positive, non-metastatic disease at the discretion of the treating clinician (9).

2.1.3 ROLE OF DOCETAXEL FOR MEN WITH M0 OR M1 DISEASE

The primary analysis of the "original comparisons" has shown docetaxel to significantly prolong survival (HR 0.78; 95% CI 0.66-0.93). This is in support of the results of the CHAARTED study which showed docetaxel improved survival in men with metastatic disease.(10) There was no evidence of heterogeneity in the treatment effect across patient groups and median survival was improved by 10 months, from 71 to 81 months. In a well powered and pre-planned sub-group analysis of men with metastatic disease at diagnosis the treatment effect was most apparent with the median survival benefit of 15 months. As a result the STAMPEDE TMG recommends that docetaxel should be strongly considered in all men with metastatic disease at presentation who are commencing ADT for the first time and are fit enough to receive chemotherapy.

Survival data for men without metastases at diagnosis is less mature but a statistically significant improvement in failure free survival is seen, therefore, docetaxel may also be considered for men with high-risk non-metastatic disease.

Therefore, docetaxel is now permitted as part of the standard-of-care for all men entering STAMPEDE at the discretion of the treating clinican and patient.

2.2 RATIONALE

There are increasing numbers of treatments which are used post relapse of first-line ADT in patients with CRPC, but there has been limited evidence as to which is associated with the best response, how they may be combined or sequenced or whether any of them might have a role as first-line treatment. An alternative approach is to investigate new drugs and new approaches to treatment, as first-line therapy in patients starting ADT. At this point, patients should be fitter and better able to tolerate treatment than when they have CRPC, and there is the possibility of having a larger and longer lasting effect. The pre-clinical and epidemiological evidence of the anti-prostate cancer effect of metformin, together with the general good tolerability of this drug underpins the rationale for evaluating metformin within the trial as a re-purposed treatment for prostate cancer. In addition metformin may counteract the metabolic effects of long-term ADT that are increasingly well recognised.

2.3 DESIGN

STAMPEDE (also known as MRC PR08) is an innovative, multi-arm multi-stage, multi-centre, randomised controlled trial. It initially assessed the effects of a bisphosphonate (zoledronic acid), a cytotoxic chemotherapeutic agent (docetaxel) and a cyclooxygenase (Cox-2) inhibitor (celecoxib), as single agents or combinations, in patients commencing long-term ADT for advancing or metastatic prostate cancer. For these questions, each comparison was divided into five stages such that, for each investigational arm, safety and activity data were generated in the first four stages; an investigational arm could only proceed to the fifth and final stage of recruitment, where it would be assessed for effect on overall survival, if shown to be sufficiently safe and active at all prior activity stages. It is important to note, however, that patient data from all arms and all stages are included in the final analyses of the primary outcome measure, even if the investigational arm did not proceed to the final stage.

A second planned interim analysis failed to demonstrate sufficient activity for celecoxib and this agent was removed from trial recruitment in April 2011; patients remaining on celecoxib treatment reverted to standard care. Protocol version 8.0 added a new drug abiraterone to the study as an additional arm (see **Section 2.7**). Protocol version 9.0 added a new comparison arm involving prostate radiotherapy for patients with newly-diagnosed metastatic disease (see **Section 2.8**). Protocol version 10.0 reflected the successful completion of recruitment to three docetaxel- and bisphosphonate-containing arms (Arms B, C and E) and removed references to these agents in the information sheets for new patients. Protocol version 11.0 extended the recruitment target for the "abiraterone comparison" (A vs G) from 1,500 to around 1,800 patients. Protocol version 12.0 added a new comparison involving the combination of abiraterone and enzalutamide. Protocol version 13.0 extended the recruitment target for the M1|RT comparison from 1,250 to around 1,800 patients. Protocol version 14.0 incorporates the permitted use of docetaxel in the standard-of-care. Protocol 15.0 sees the addition of new research (Arm K) assessing the combination of the current SOC and metformin. Protocol version 15.0 also marks the closure of Arm J (target sample size reached).

2.4 RESEARCH TREATMENT: BISPHOSPHONATES

Note: recruitment stopped to the zoledronic acid and docetaxel-containing arms in Mar-2013 as recruitment target sample was reached.
Treatment has been completed in all patients and the results reported. See Protocol version 13.0 or older for details on the rationale.

2.5 RESEARCH TREATMENT: CHEMOTHERAPY

Note: recruitment stopped to the zoledronic acid and docetaxel-containing arms in Mar-2013 as recruitment target sample was reached.
Treatment has been completed in all patients and the results reported. See Protocol version 13.0 or older for details on the rationale.

2.6 RESEARCH TREATMENT: CYCLOOXYGENASE-2 INHIBITORS

Note: recruitment completed to both celecoxib-containing arms in Apr-2011 at the end of Activity Stage II of this comparison.
Treatment has been ceased in all patients and the results to be reported in 2016. See Protocol version 13.0 or older for details on the rationale.

2.7 RESEARCH TREATMENT: STEROID SYNTHESIS INHIBITORS

Recent evidence suggests that an important mechanism for escape from tumour control by androgen ablation is the intracellular conversion of steroid precursors to androgenic steroids by prostate cancer cells. A key enzyme in this process is CYP17, which therefore represents a logical target for therapy in CRPC. (11). Abiraterone acetate (3β-acetoxy-17-(3-pyridyl)androsta-5,16-diene, code CB7630; JNJ-212082) is rapidly converted in vivo to abiraterone (JNJ-589485; formerly code named CB7598). It is a selective, irreversible inhibitor of 17α-hydroxylase/C17,20-lyase (cytochrome P450c17 [CYP17]), an enzyme that is critical in the production of androgens in the testes, adrenal glands and prostate tumor tissue. Inhibition of CYP17 inhibits the conversion of pregnenolone or progesterone into dehydroepiandrosterone (DHEA) or androstenedione, respectively, each of which is a precursor of testosterone. The pharmacodynamic effect is a more effective androgen depletion than can be induced by surgical castration, or medically by gonadotropin releasing (GnRH) hormone analogues used as first line hormone therapy in prostate cancer.

Approximately 2,280 prostate cancer patients participated in the two Phase 3 RCTs (COU-AA-301 and COU-AA-302), with approximately 1,335 patients receiving abiraterone acetate at 1000mg daily dose continuously, in these studies. These studies have demonstrated abiraterone to prolong survival when given post-docetaxel (HR 0.65) and pre-docetaxel (HR 0.82). As a result it is now approved use in the USA and Europe in CRPC.(12, 13)

Side-effects with abiraterone acetate are modest with the main adverse effects being elevated transaminases (usually mild), hypokalaemia and hypertension due to secondary hyperaldosteronism and fluid retention (preventable by low doses of glucocorticoids). In order to prevent secondary hyperaldosteronism, it is recommended that prednisolone (or prednisone) 10mg daily be administered in the CRPC setting. Within more recent studies in earlier stage patients, lower doses (typically 5mg of prednisone/prednisolone) are being used due to concerns about side effects of long-term exposure to glucocorticoid. Within the STAMPEDE trial, we suggest prednisolone/ prednisone dose of 5mg OD which may be increased to 5mg BID at the investigator's discretion if there are any concerns about monitoring or risks for the patient with 5mg OD.

We hypothesise that the agent may be more active still when given up-front in combination with first-line ADT by preventing or delaying the development of castrate refractory disease.

2.8 RESEARCH TREATMENT: RADIOTHERAPY TO THE PROSTATE FOR PATIENTS WITH NEWLY-DIAGNOSED METASTATIC DISEASE

Therapy directed against the primary tumour in the presence of metastatic disease has been evaluated rigorously in only one malignancy to date: renal cell carcinoma. Two cooperative groups ran randomised trials enrolling patients with previously untreated metastatic RCC whose primary tumours were amenable to surgical resection. Patients were randomised to receive the standard systemic therapy of the day, interferon-alpha, either alone or with radical nephrectomy. The combination of nephrectomy and interferon was shown to significantly improve median survival from 7 to 17 months in one trial (14) and from 8 to 11 months in the other (15). The mechanism by which nephrectomy improves survival remains obscure. In preclinical models, the primary tumour has been found to secrete molecules that prime the microenvironment in which metastases can develop. An implication of this work is that therapy directed at the primary tumour, by abrogating this endocrine signalling, could retard the formation and the growth of distant metastases.

The results of two large-scale randomised trials of prostate radiotherapy are also provocative. The Scandinavian SPCG-7 trial and the MRC PR07 trial randomised men with locally advanced prostate cancer, who were at high risk of possessing occult metastatic disease, to either ADT alone or ADT plus prostate radiotherapy (4, 8). The addition of radiotherapy dramatically improved 10-year outcomes: mortality from prostate cancer was halved. Interestingly, the benefit of radiotherapy started to emerge as early as three years from the time of randomisation. This seems improbably early if the benefit of local treatment is mediated via the prevention of subsequent disease dissemination. Rather, it is more consistent with the possibility that local treatment has a beneficial impact on the rate of progression of existing micrometastatic disease.

We hypothesise that local therapy to the primary site may retard distant disease progression and prolong survival in patients with newly-diagnosed metastatic prostate cancer.

Figure 3: Use of RT in STAMPEDE

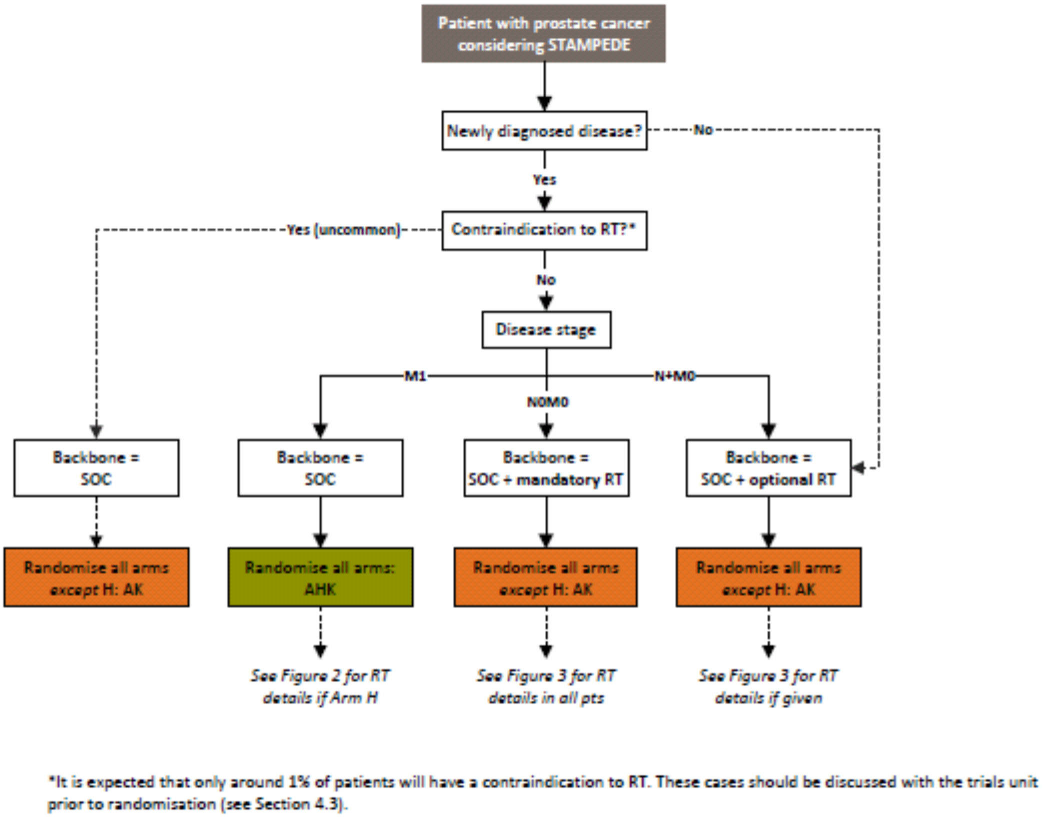

2.9 RESEARCH TREATMENT: COMBINATIONS OF ORIGINAL RESEARCH ARMS

2.9.1 Bisphosphonate And Chemotherapy

Note: recruitment stopped to the zoledronic acid and docetaxel-containing arms in Mar-2013 at the end of Activity Stage IV. Treatment has been completed in all patients and the results reported. See Protocol version 13.0 or older for details on the rationale.

2.9.2 Bisphosphonate And Cyclooxygenase-2 Inhibitors

Note: recruitment stopped to both celecoxib-containing arms in Apr-2011 at the end of Activity Stage II.

2.10 COMBINATION OF STEROID SYNTHESIS INHIBITORS AND ANDROGEN RECEPTOR SIGNALLING INHIBITOR

The majority of patients with advanced prostate cancer who have disease progression on abiraterone or enzalutamide taken as single agents, have a rise in PSA, suggesting reactivation of androgen receptor (AR), or other steroid signalling pathways resulting in increased PSA transcription, is the pathway to the development of resistance.(16)

The primary pharmacodynamic effect of enzalutamide is inhibition of androgen binding to the AR, AR nuclear translocation in the presence of androgen and AR:chromatin association. In multiple prostate cancer cell lines that specifically model CRPC (LNCaP/AR, VCaP, W741C LNCaP), the consequences of enzalutamide treatment include inhibition of AR-induced gene transcription, reduced cell proliferation, increased cell death by apoptosis and tumor regression.

In a mouse xenograft model of CRPC using prostate cancer cells that overexpress the AR (LNCaP/AR), enzalutamide inhibits tumor growth and reduces tumor size. A major human metabolite of enzalutamide, N-desmethyl enzalutamide, demonstrates key primary pharmacodynamics of similar potency to the parent molecule, while the carboxylic acid derivative metabolite has no known pharmacodynamic effect.

The question under investigation is: can progression be delayed (and survival extended) by using a combination of abiraterone and enzalutamide?

2.10.1 Supplementing Abiraterone And Prednisolone With Enzalutamide

Several studies have shown that the AR can become promiscuously activated by very low levels of androgens or other steroid metabolites and drugs that bind the AR (17-20). It is known that very low levels of androgens can persist in patients treated with abiraterone acetate (21). Drugs that bind the AR, may include co-administered glucocorticoids. Furthermore, AR mutations of the sort previously described in CRPC, can be activated by cortisol and other glucocorticoids at levels much lower than those reported in patients treated with abiraterone and prednisolone at a dose of 5mg bid (20, 22). Moreover, abiraterone binds the AR and, although weak antagonism of wild-type and most previously described AR mutations are observed (22), a similar mechanism to that described with classical anti-androgens, such as bicalutamide, could lead to change-of-function AR mutations associated with AR activation following abiraterone binding. Therefore, concomitant treatment with an androgen receptor signalling inhibitor could prevent “promiscuous” AR activation in patients treated with abiraterone. Enzalutamide is a androgen receptor signalling inhibitor is approved for use on its own in the treatment of advanced CRPC (23), and there is evidence of activity for hormone-naïve prostate cancer (24).

2.10.2 Supplementing Enzalutamide With Abiraterone And Prednisolone

Enzalutamide in combination with ADT is both effective and well tolerated in CRPC.(23) However, recent studies have suggested that intra-tumoral testosterone levels increase in patients treated with enzalutamide (25). The implications of this finding are that the increase in intra-tumoral testosterone could be associated with up-regulation of enzymes involved in steroid biosynthesis.(26) Although enzalutamide has a high affinity for the AR, this is several-fold lower than both the natural ligands testosterone and DHT (27), which means that enzalutamide would be out-competed at the AR ligand-binding domain if and when androgen levels rise. In vitro, a ten-fold rise in intra-cellular androgen was sufficient to prevent inhibition of AR by 30uM of enzalutamide;(22) these levels are representative of the plasma levels of enzalutamide active metabolites, which can be achieved with enzalutamide 160mg po daily (28).

A strategy for preventing the rise in intra-cellular androgens in patients treated with enzalutamide would be inhibition of CYP17A1. Abiraterone is currently the only CYP17A1 inhibitor with proven efficacy. It therefore seems logical to use the combination of enzalutamide and abiraterone to both block a rise of intra-cellular androgens and prevent promiscuous activation of the AR.

2.10.3 Summary Of Rationale For This Combination

To date, investigation has focussed on patients with CRPC but there is a strong rationale for the combination of enzalutamide and abiraterone in the hormone treatment-naïve setting in which STAMPEDE is focused.

STAMPEDE is already evaluating abiraterone plus conventional ADT but we will not assess the combination of conventional ADT plus enzalutamide; other trials by industry and other cooperative groups will address that question. The inclusion of an arm with ADT and enzalutamide in STAMPEDE was therefore considered to be a duplication of effort and was not supported by the Trial Management Group.

The combination of enzalutamide and abiraterone is a novel approach and offers considerable promise in delaying progression – it therefore represents an attractive addition to the comparisons under investigation in STAMPEDE, and one that is unlikely to be replicated in other planned trials of this size.

2.11 RATIONALE FOR THE "METFORMIN COMPARISON"

All men joining STAMPEDE are planned for long-term ADT, a treatment associated with an increased risk of insulin resistance, hyperglycaemia, dyslipidaemia and obesity. Over 50% of men receiving long-term ADT will develop Metabolic Syndrome (29) resulting in increased cardiovascular morbidity and mortality. Obesity and high bind insulin C-peptide levels, indicating insulin resistance are independent predictors of increased prostate cancer-specific mortality and the presence of metabolic syndrome and diabetes in men treated with ADT is associated with shorter survival.

Metformin, which in non-diabetic individuals has been shown to lower the incidence of diabetes, counteracts some of these side-effects of ADT, including insulin insensitivity, hyperinsulinaemia and diabetes. It also reduces the levels of cholesterol, LDLs and triglycerides by inhibiting the fatty acid synthesis via activation of Adenosine Monophosphate Activated Kinase (AMPK) and decreases the platelet aggregation factor 1, platelet aggregation, vascular adhesion molecules, CRP and leptin (30-33). Through mitigation of the cardiovascular and metabolic consequences of ADT, metformin is proposed to reduce treatment associated morbidity and improve all-cause mortality.

In addition, recent data has emerged consolidating the knowledge that cancer progression is linked integrally with metabolic modulators and that modification of this process by metformin has an important effect on cancer progression and survival. Pre-clinical data has shown that metformin is an important stimulator of AMPK which acts as the cellular "masterswitch" for energy regulation. AMPK acts to inhibit the effects of elevated insulin levels which promote metastasis, tumour growth and treatment resistance. Insulin increases mRNA and protein expression of steroidogenic enzymes leading to the up-regulation of intracellular testosterone levels, secreted androgens, thereby activating the AR (34). Metformin also influences the PI3K-AKT pathway and has an anti-proliferative effect via inhibitor of mTOR as well as targeting cancer stem cells. In vitro, metformin has been shown to inhibit androgen-induced IGF-IR up-regulation through disruption of androgen signalling (35).

Evidence in support of this includes a systematic review and meta-analysis of 13,008 men with type 2 diabetes mellitus (T2DM) and concurrent cancer which has shown improved survival in men treated with metformin compared with other anti-diabetic agents. In a systematic review of observational data from over 1 million men, there was a significant association seen between metformin and decreased risk of death from any cancer. Another systematic review found that the use of metformin in diabetic patients was associated with a significantly lower risk of cancer incidence and cancer mortality (36). In a large retrospective cohort study of 3837 diabetic men with prostate cancer, metformin was associated with a decreased risk of prostate cancer specific mortality (HR=0.76 [0.64-0.89]) and death (HR=0.76 [0.70-0.82]. In a prospective non-randomised phase II study in non-diabetic CRPC patients, 36% of patients receiving metformin were progression-free at 3 months and >50% had a prolongation of their PSA doubling time (37). In summary,

meformin is proposed to mitigate many of the adverse side effects of long-term ADT as well as having multiple potential anti-cancer effects and therefore STAMPEDE will evaluate re-purposing this treatment as a novel therapeutic approach in the management of high risk or metastatic prostate cancer.

3 SELECTION OF INSTITUTIONS AND INVESTIGATORS

Centres who wish to participate in the STAMPEDE trial should be registered with the Medical Research Council Clinical Trials Unit at University College London (MRC CTU at UCL) for this purpose. Before any patients are randomised the MRC CTU must receive a completed and signed Investigator Statement. The STAMPEDE investigator statement is signed by the Principal Investigator for that institution (download from www.stampedetrial.org). R&D approval for the site, along with a fully-signed model agreement, is also required before recruitment can begin.

In addition, and in compliance with the principles of GCP, all institutions participating in the trial will complete a delegation log and forward this to the MRC CTU. Each person working on the STAMPEDE trial must sign off a section of this log indicating their responsibilities. The MRC CTU must be notified of any changes to trial personnel and/or their responsibilities. An up-to-date copy of this log must be stored in the Investigator Site file at the institution and also at the MRC CTU.

The Clinical Trial Authorisation (CTA) for the STAMPEDE trial requires that the Medicines and Healthcare Products Regulatory Agency (MHRA) be supplied with the names and addresses of all participating investigators/institutions. Trial staff at the MRC CTU will perform this task; hence, it is vital to receive full contact details for all investigators prior to their entering patients.

Finally, before a patient is entered into the trial written informed consent must be obtained. Approved patient information sheets and informed consent forms are supplied as templates.

Only a limited number of centres participated in the initial Pilot Phase of the original trial; this was to ensure that safety and feasibility data were collected expediently. Subsequent stages of the trial are open to any centre that wishes to participate and has fulfilled the requirements described above.

3.1 RADIOTHERAPY ACCREDITATION

The introduction of the "M1|RT comparison" in Protocol version 9.0 introduced the need for RTQA accreditation in sites giving radiotherapy. The detail of RTQA accreditation is in **Appendix K**. However, centres that have been RTQA accredited for another multi-centre prostate radiotherapy trial in the UK (e.g. RADICALS or CHHIP) will be automatically granted STAMPEDE RTQA accreditation.

4 SELECTION OF PATIENTS

4.1 GENERAL INCLUSION CRITERIA

Participants must fulfil both of the criteria in **Section 4.1.1** **or** one criterion in **Section 4.1.2** **or** at least one criterion in **Section 4.1.3**. Additionally, all patients must fulfil the criteria in **Section 4.1.4**.

4.1.1 High-Risk Newly-Diagnosed Non-Metastatic Node-Negative Disease

Both:

- At least two of: T category T3/4, PSA≥40ng/ml or Gleason sum score 8-10
- Intention to treat with radical radiotherapy (unless there is a contra-indication; exemption can be sought in advance of consent, after discussion with MRC CTU)

OR

4.1.2 Newly-Diagnosed Metastatic Or Node-Positive Disease

At least one of:

- Stage T_{any} N+ M0
- Stage T_{any} N_{any} M+

OR

4.1.3 Previously Radically Treated, now relapsing (prior Radical Surgery And/Or Radiotherapy)

At least one of:

- PSA ≥4ng/ml and rising with doubling time less than 6 months
- PSA ≥20ng/ml
- N+
- M+

AND

4.1.4 For All Patients

I. Histologically confirmed prostate adenocarcinoma
II. Intention to treat with long-term androgen deprivation therapy
III. Treating clinician and patient should have decided if docetaxel is to be part of the standard-of-care prior to randomisation
IV. Fit for all protocol treatment[1] and follow-up, WHO performance status 0-2[2]
V. Have completed the appropriate investigations prior to randomisation
VI. Adequate haematological function: neutrophil count >1.5x10^9/l and platelets >100x10^9/l
VII. Estimated creatinine clearance >30ml/min
VIII. Serum potassium ≥3.5mmol/L
IX. Written informed consent
X. Willing and expected to comply with follow-up schedule
XI. Using effective contraceptive method if applicable

[1] Medical contraindications to the trial medications are given in **Section 6**
[2] For WHO performance status definitions see **Appendix A**

4.2 GENERAL EXCLUSION CRITERIA

Patients must not fulfil any of the criteria, below.

I. Prior systemic therapy for locally advanced or metastatic prostate cancer except as listed in **Section 4.1.3**
II. Metastatic brain disease or leptomeningeal disease
III. Abnormal liver functions consisting of any of the following:
   - Serum bilirubin ≥1.5 x ULN (except for patients with Gilbert's disease, for whom the upper limit of serum bilirubin is 51.3µmol/l or 3mg/dl)
   - Aspartate aminotransferase (AST) or alanine aminotransferase (ALT) ≥2.5 x ULN
IV. Any other previous or current malignant disease which, in the judgement of the responsible clinician, is likely to interfere with STAMPEDE treatment or assessment
V. Symptomatic peripheral neuropathy grade ≥2 (NCI CTC)[3]
VI. Any surgery (e.g. TURP) performed within the past 4 weeks
VII. Patients with significant cardiovascular disease such that, in the investigator's opinion, the patient is unfit for any of the study treatments. This might include:
   - Severe/unstable angina
   - Myocardial infarction less than 6 months prior to randomisation
   - Arterial thrombotic events less than 6 months prior to randomisation
   - Clinically significant cardiac failure requiring treatment (NYHA II-IV)[4]
   - Cerebrovascular disease (e.g. stroke or transient ischaemic episode) less than 6 months prior to randomisation
   - Patients with uncontrolled hypertension defined as systolic BP greater or equal than 160 mmHg or diastolic BP greater or equal than 95 mmHg
VIII. Prior chemotherapy for prostate cancer (excluding patients receiving docetaxel as part of the new SOC)
IX. Prior exposure to long-term hormone therapy before randomisation (unless as described in **Section 4.5.3**)
X. Prior exposure to systemic treatment for prostate cancer (excluding hormone therapy) e.g. abiraterone and enzalutamide.

4.3 SELECTION CRITERIA FOR COMPARISON OF RESEARCH RT FOR METASTATIC DISEASE (M1|RT)

All patients meeting criteria in **Section 4.1** and **4.2** are eligible for the trial, but not all can be allocated to the research (M1) radiotherapy arm. The selection criteria for the "M1|RT comparison" are:

- Newly-diagnosed prostate cancer
- Demonstrable M1 disease
- No contraindication to radiotherapy e.g. no previous pelvic radiotherapy and no history of inflammatory bowel disease
- No previous radical prostatectomy

Any patients meeting these criteria will have a chance to be allocated to Arm H. For those rare cases where radical RT is planned for a newly-diagnosed M1 patient, the TMT and TMG will need to review and approve the inclusion of the patient for randomisation only between Arm A and K.

[3] See **Appendix I** for common toxicity grading
[4] NYHA classifications can be found in **Appendix A**

4.4 SELECTION CRITERIA FOR METFORMIN COMPARISON

All patients meeting criteria in **Section 4.1** and **4.2** are eligible for the trial, but patients with known diabetes mellitus are not eligible for randomisation to the "metformin comparison".

All non-diabetic patients require an HbA1c to be performed ideally within 8 weeks prior to randomisation to confirm their non-diabetic status.

In summary, additional inclusion criteria for the "metformin comparison" are:

- HbA1c <48 mmol/mol (equivalent to <6.5%)
- Estimated creatinine clearance ≥60mls/min
- No history of metabolic acidosis or pre-disposing conditions
- Not current or previous treatment with metformin
- No contra-indications to metformin

If following screening the patient is found to have diabetes mellulitis (i.e. HbA1c is 6.5% or higher) the patient is only eligible for randomisation (between Arms A and H only) if they meet all of the selection criteria for the M1|RT comparison (see **Section 4.2**). M0 or previously treated patients with known diabetes mellitus, or who are found to be diabetic on screening, are currently ineligible for randomisation and will therefore be screen failures.

All patients with abnormal baseline HbA1c should be informed and investigated futher according to local practice e.g. referred to their GP for further management.

If the patients is currently receiving short-term corticosteroids, ideally repeated screening should be performed once corticosteroid treatment has stopped providing this remains within 12 weeks of starting ADT (see **Section 4.5.3**).

4.5 SCREENING PROCEDURES

4.5.1 Investigations Prior To Randomisation

All patients should have the following examinations performed within 8 weeks to confirm eligibility prior to randomisation.

The following standard imaging is required and the latest available scans should be used:

- CT or MRI of pelvis and abdomen
- Bone Scan (or equivalent e.g. whole body MRI, choline-PET-CT, PSMA-CT-PET)
- Chest X-ray (only if chest was not included in CT or MRI which would be preferable)

Any additional imaging such as CT-PET scanning can be performed according to local practice but, for the purposes of the trial, the recorded stage should be the CT stage only; additional information on the CT-PET stage will also be collected.

The following bloods and additional measurements are required prior to randomisation:

- ECG
- Blood pressure
- PSA Test
- HbA1c
- Full blood count

- Urea and Electrolytes
- Liver function tests
- Serum creatinine
- Systolic and diastolic blood pressure

Patients who initially fail to meet the eligibility criteria can be re-screened at a later date. Of note, for patients receiving standard-of-care docetaxel at the point of screening, it is acceptable to use a full blood count measurement prior to chemotherapy to confirm eligibility.

Prior to randomisation:
- Check details of any prior treatments for prostate cancer
- Check any contraindications to radiotherapy or metformin
- Check concomitant medications

4.5.2 Additional baseline Investigations

The following blood tests are required at baseline (within 4 weeks before or after to randomisation):
- Testosterone (pre-ADT, if available)
- Serum corrected calcium
- Phosphate
- Magnesium
- Albumin
- Fasting glucose (mandatory)
- Fasting triglycerides (mandatory)
- Lipid profile (non-fasting; total cholesterol, LDL and HDL) (mandatory)

The following additional procedures are required and mandatory at baseline
- Waist circumference measurement
- Weight and height

A trial screening log will be available to all centres; copies are not required by the STAMPEDE Trial Team unless requested.

4.5.3 Androgen Deprivation Therapy Prior To Randomisation

If ADT has already started prior to randomisation, the first LHRHa must have been given within 12 weeks of randomisation. Additionally, if anti-androgens are being used, these must have started within 14 weeks of randomisation. A PSA level must have been taken prior to treatment starting.

Note that long-term anti-androgen monotherapy is not permitted in the trial for newly recruited patients from Protocol version 8.0; patients may change treatment to join the trial, provided that they have not had more than 12 weeks of ADT prior to randomisation. Further details on hormone therapies allowed prior to randomisation are discussed in **Appendix L**.

Any relapsing patients treated with adjuvant or neo-adjuvant hormone therapy alongside their radical surgery or radiotherapy must have completed that period of hormone therapy at least 12 months before joining STAMPEDE and it must have been no longer than 12 months in duration.

Note that baseline testosterone measurements will not be required in patients who have already commenced hormone manipulation prior to randomisation.

4.5.4 Choice About Standard-Of-Care Docetaxel

The treating clinician and patient must have decided, prior to randomisation, whether docetaxel is to be given as part of standard-of-care. Docetaxel treatment must start within 12 weeks after starting ADT, preferably within 8 weeks. Patients can have already started docetaxel treatment when randomised providing this is within 12 weeks after starting ADT.

4.5.5 Starting Trial Treatment

For patients allocated to Arm H: For patients not receiving SOC docetaxel, RT should not be commenced until SOC docetaxel has been completed; this should start within 3-4 weeks from after the last administration of docetaxel and continued according to the predefined scheduled unless toxicity is reported. Should any docetaxel-related toxicities occur during treatment these need to have recovered to grade 1 before the allocated research treatment begins. Please discuss with the trial team if any docetaxel-related toxicities of greater severity than grade 1 persist (this excludes alopecia, nail changes and neuropathy, which do not need to have resolved prior to starting research treatment).

Investigators should discuss with the trial team any patient who may not start their allocated trial treatment or where a delay of 6 or more weeks, between last administration and starting research treatment is expected. Any delays in starting research radiotherapy should be discussed with the STAMPEDE team and recorded as appropriate in the relevant CRF.

For patients allocated to Arm K: For all patient allocated to metformin, treatment should start as soon as possible after randomisation. Metformin can be given in combination with SOC docetaxel. Investigators should aim that this is at least within 4 weeks post-randomisation and within 12 weeks of starting ADT (see **Section 6**).

4.5.6 Concomitant Medications

All concomitant medications should be recorded including any vitamin and mineral supplements the patient is taking, regular consumption of NSAID and/or aspirin and use of other bisphosphonates (see **Section 4.2**). Of particular interest are herbal preparations such as PC-SPES, Prostasol, Saw Palmetto and St John's Wort.

Caution should be exercised when starting any concomitant medications that may result in a worsening of renal function e.g. initiating anti-hypertensive therapies such as ACE inhibitors, diuretics such as frusemide, or starting a non-steroidal anti-inflammatory drug (NSAID). Please refer to **Table 15** for more information on drugs which may require additional monitoring of renal functions.

All concomitant medications should be continued throughout the trial unless the responsible clinician decides otherwise. If patients continue to require medication for the management of docetaxel-related toxicities, please discuss this with the trial team. Please see **Section 6** for more information on concomitant medications and their use with abiraterone and enzalutamide.

From Protocol version 15.0 onwards the trial will require information regarding long-term (>6 months) use of the following concommitant medications of classes of interest.

- Statins
- Metformin
- Aspirin
- Bisphosphonates or Denosumab/calcium and vitamin D

- Opiate pain killers
- ACE inhibitors or angiotension II antagonists
- Vitamin B12

This information will be collected at each follow up (see **Table 16**).

4.6 ADDITIONAL DETAILS FOR PATIENTS JOINING SUB-STUDIES

If the patient has given their consent to participate in the DNA analysis sub-study, a saliva sample will be collected. This replaces the droplet of blood collected in previous versions of the protocol. An additional element of DNA analysis will involve the collection of plasma samples for patients in the "enzalutamide + abiraterone comparison".

Patients have been asked to consent for the use of remaining tissue samples obtained at prostate biopsy or following surgery for use in additional research. Randomising sites will be asked to assist in the retrieval of tissue samples stored as FFPE blocks from pathology stores or referring hospitals when these are required for additional translational sub-studies. Full details of all sub-studies and instructions relating to the handling of the saliva and blood sample are given in **Section 17** and **Appendix D**.

5 RANDOMISATION AND ENROLMENT

Patients will be allocated to any of the open research arms for which they are suitable. Patients with non-metastatic disease or who have had previous local therapy to the prostate or who have a contraindication to radiotherapy will not be allocated to Arm H (see **Section 4.3**). Patients with a known diagnosis of diabetes will not be allocated to Arm K (see **Section 4.4)**

To enter a patient the randomisation form should be completed carefully and the MRC CTU contacted by phone:

RANDOMISATIONS
To randomise, call MRC CTU at UCL, Monday to Friday 0900-1700
excluding public holidays or dates when notice has been given by the CTU.
Tel: +44 (0) 20 7670 4777

A trial number and treatment will be allocated and given over the phone or by return fax. In addition, a letter confirming these details will be sent. The trial number will be the primary way in which the patient will be identified and should be used in all correspondence.

5.1 CO-ENROLMENT GUIDELINES

Ideally, patients should not be participating in any other clinical trial of prostate cancer treatment when they enter STAMPEDE and should not enter any other trials until a failure-free survival (FFS) event has been experienced and reported. After this point, the patient may be entered into further, second-line treatment studies. The primary outcome measure of STAMPEDE is overall survival. Participation in post-progression studies must be reported on the Co-enrolment CRF.

Data sharing agreements with “down-stream” trials are encouraged to improve data quality in both trials and to reduce costs to both organisations.

6 TREATMENT OF PATIENTS

6.1 TRIAL TREATMENT

Patients will be randomised to the control arm (Arm A) or one of the research arms. All patients will receive androgen deprivation therapy (ADT) to achieve castration levels of testosterone. The method of ADT is a local choice but must be specified for each patient prior to randomisation. The recommended methods of ADT are given in **Section 6.2.1**. Note that from Protocol version 8.0 onwards, bicalutamide monotherapy is no longer permitted as a trial therapy for new patients but patients may switch to a permitted therapy to join the trial. Please see **Appendix L** for more information on ADT timing before randomisation.

6.1.1 Required timelines when starting trial treatment

Allocated treatment should start promptly after randomisation. Please refer to **Section 4.5.3, 4.5.4, 4.5.5** for more information on starting of trial treatment.

6.2 ARM A: STANDARD-OF-CARE

The standard-of-care for this patient group is **androgen deprivation therapy** (see **Section 6.2.1**). For some patient groups, this should now be supplemented with standard radiotherapy (see **Section 6.2.2**). From Protocol version 14.0 onwards the standard-of-care includes permitted use of docetaxel for all suitable patients (see **Section 6.2.3**).

In summary, SOC treatments may include:

- ADT alone
- ADT + Radiotherapy
- ADT ± Radiotherapy ± Docetaxel

6.2.1 Hormone Therapy

The permitted methods of ADT are bilateral orchidectomy, LHRH analogues and LHRH antagonists. Patients having a bilateral orchidectomy are required to adher to the same timelines as specified in **Section 4.4.2.** unless there is a strong clinical reason not to do so. Other methods of ADT should be discussed with the Chief Investigator or the Trial Surgeon. The planned duration of ADT should be at least 2 years.

Bilateral orchidectomy: Operations should be performed by appropriately trained surgeons. A total or sub-capsular orchidectomy may be performed.

LHRH agonists: LHRH agonists/analogues used according to local practice. The prophylactic use of anti-androgens to prevent tumour “flare” is recommended.

LHRH antagonists: LHRH antagonists used according to local practice. The use of prophylactic use of anti-androgens to prevent tumour “flare” is not necessary.

6.2.2 Standard-of-care (M0) RT

N0M0 patients: Investigators should give standard radiotherapy (RT) to patients with node negative, non-metastatic disease (N0M0), in accordance with data from the PR07 and SPCG trials. If there is an intention to omit radiotherapy (e.g. contraindications) in patients with N0M0 disease this must be

discussed with the Trials Office before consent. See **Section 6.17** for further details of radiotherapy administration.

N+M0 patients: the benefit of radiotherapy in this group is at present uncertain with no firm data to either support or refute its use. However, the PR07 trial included some node positive patients as cross sectional imaging was not a part of the baseline assessment in this trial, which did include whole pelvis radiotherapy (8). For patients with node positive, non-metastatic disease, radiotherapy is therefore recommended in suitable cases (9).

Investigators will be asked to state their intention with regards to planned radiotherapy in this group at randomisation. Intention to give radiotherapy (or not) for node positive patients must be stated at randomisation to ensure that there is no bias towards particular combinations of systemic therapy with radiotherapy.

Standard-of-care radiotherapy is not a core part of the trial, therefore we intend to collect minimal data about the radiotherapy administered. It is accepted that some patients will develop progressive disease before radiotherapy can be administered and if this occurs the reasons for non-delivery of treatment must be recorded on the radiotherapy detail form.

Any patient who has had a previous, definite diagnosis of inflammatory bowel disease is at increased risk of disease re-activation following radiotherapy, and the risks of this must be balanced against the potential benefits of radiotherapy on an individual basis.

6.2.3 STANDARD-OF-CARE DOCETAXEL

Investigators are strongly encouraged to consider giving docetaxel as part of the standard-of-care for patients with newly-diagnosed metastatic disease based on the survival benefit demonstrated by both STAMPEDE in the primary analysis of the "original comparisons" and CHAARTED (10) (38, 39). Investigators may also consider giving docetaxel to patients with high-risk locally advanced disease given both the significant improvement in failure-free-survival and consistency of effect for prostate cancer-specific survival shown by STAMPEDE.

The treating clinician and patient must have decided if docetaxel is to be given prior to randomisation and treatment may have started when the patient is randomised. As with standard radiotherapy, minimum data collection will be required however the start and end dates of treatment are needed to ensure the appropriate timelines are met (see **Section 4.5.4**). A confirmation whether the treatment with docetaxel has started or not as planned will also need to be sent to the trial team.

Docetaxel is given according to local protocols as a standard non-trial treatment. The regime used previously within STAMPEDE was 75mg/m2 Day 1 as 1hr IV infusion, plus prednisolone 5mg bid daily for 21 days repeated every 3 weeks for a maximum of 6 cycles.

The STAMPEDE TMG would suggest prednisolone could be omitted and data on the use of co-prescribed steroid will be collected on the relevant CRF (please see **Table 16** for more details)

6.3 ARM B: ADT + ZOLEDRONIC ACID

Note: recruitment stopped to the zoledronic acid and docetaxel-containing arms in Mar-2013 as recruitment target sample was reached. Please see Protocol version 13.0 for information on the administration of this trial drug. Trial treatment has now been completed for all patients.

6.4 ARM C: ADT + DOCETAXEL

Note: recruitment stopped to the zoledronic acid and docetaxel-containing arms in Mar-2013 as recruitment target sample was reached. Please see Protocol version 13.0 for information on the administration of this trial drug. Trial treatment has now been completed for all patients.

6.5 ARM D: ADT + CELECOXIB

Note: recruitment completed to both celecoxib-containing arms in Apr-2011 at the end of its Activity Stage II. Please see Protocol version 13.0 for information on the administration of this trial drug.

6.6 ARM E: ADT + DOCETAXEL + ZOLEDRONIC ACID

Note: recruitment stopped to the zoledronic acid and docetaxel-containing arms in Mar-2013 as recruitment target sample was reached. Please see Protocol version 13.0 for information on the administration of this trial drug. Trial reatment has now been completed for all patients.

6.7 ARM F: ADT + ZOLEDRONIC ACID + CELECOXIB

Note: recruitment completed to both celecoxib-containing arms in Apr-2011 at the end of its Activity Stage II. Please see Protocol version 13.0 for information on the administration of this trial drug

6.8 ARM G: SOC + ABIRATERONE

Note: recruitment to the "abiraterone comparison" completed in Jan-2014. Please note that some patients will continue treatment until all types of disease progression or up to a maximum of 2 years. All N0M0 patients have now reached the maximum duration on trial treatment. Please see sections below for more information.

Standard-of-care: As described in **Section 6.2.**

Abiraterone will be administered as a single 1000mg daily oral dose (4 tablets to be taken together once a day) together with prednisolone or prednisone 5mg daily to prevent secondary mineralocorticoid excess. Abiraterone absorption is increased by food. The tablets should be taken at least 2 hours after food, swallowed whole with some water. No food should be eaten for 1 hour afterwards.

Prednisolone (prednisone in Switzerland) should be taken as a single dose with food in the morning.

In patients with M1 disease, treatment with abiraterone will continue from randomisation until all types of progression have occurred, consistent with the COU-AA-301 trial (40) i.e., abiraterone would be given for these patients until a composite assessment based on:

- PSA progression (as defined in **Appendix J**)
- Radiological progression (appearance of new lesions or progression of existing lesions) **and**
- Clinical progression (defined as new cancer-related symptoms)

It is accepted that these flexible criteria for stopping treatment with abiraterone are open to the investigator's interpretation and discretion. Patients might continue treatment beyond the first failure-free survival (FFS) event; the first FFS event must be reported as per the other arms; all types of progression (PSA, radiological and clinical) need to be reported once.

In patients with N0M0 disease or N+M0 disease undergoing radical radiotherapy, treatment would continue for 2 years or all types of disease progression as defined for M1 patients, whichever is the sooner. ADT can be discontinued in this group at 2 years at the discretion of the local investigator (see **Section 6.2**).

For patients with N+M0 disease not planned for radical radiotherapy, treatment will continue as for patients with M1 disease until all types of disease progression.

If a patient allocated to Arm G develops only biochemical failure, the responsible clinician might switch from abiraterone + prednisolone 5mg od to abiraterone and dexamethasone 0.5mg od.

Trial treatment must stop if other systemic treatments are initiated at any time for disease progression control (including chemotherapy, radium 223 etc). Anti-androgens (i.e. bicalutamide) should not be given in combination with abiraterone due to the risk of toxicity.

See **Section 7.1.2** for further information on the trial definition of progression.

6.9 ARM H: SOC + PROSTATE RADIOTHERAPY IN M1 PATIENTS

Standard-of-care: androgen deprivation therapy +/- docetaxel (as described in **Sections 6.2.1** and **6.2.3**).

Radiotherapy will commence as soon as practicable. For non-docetaxel treated patients, this should ideally be within 4 weeks after randomisation. Docetaxel-treated patients should aim to start trial treatment within 3-4 weeks after the last administration of docetaxel, preferably within 6 weeks (see **Section 6.9.1**).

Treatment will be according to the guidelines in **Section 6.9.1**. Two radiotherapy dose-fractionation schedules are permitted:

- 36Gy in 6 fractions of 6Gy, administered weekly over 6 consecutive weeks
- 55Gy in 20 fractions of 2.75Gy, administered daily, five days per week, over 4 consecutive weeks

Details of the recommendations for outlining, CTV and PTV are in **Section 6.9.1**.

6.9.1 RESEARCH (M1) PROSTATE RADIOTHERAPY TREATMENT ADMINISTRATION

A treatment planning CT scan will be acquired with the patient supine, with empty rectum and comfortably full bladder.

Megavoltage equipment is required with effective photon energies ≥6MV. Minimum source-to-axis distance is 100cm. Field arrangement is at the clinician's discretion: acceptable treatment techniques (field arrangement) include a 3-field (anterior, right lateral, and left lateral), 4-field (anterior, posterior, right lateral, and left lateral), or 6-field (right and left anterior oblique, right and left posterior oblique, and right and left lateral) or equivalent coplanar technique with multi-leaf collimation for all fields to adequately protect normal structures.

The Clinical Target Volume (CTV) will consist of the prostate gland alone as visualized on the treatment-planning CT scan. The base of the seminal vesicles may also be included if they are macroscopically involved. Inclusion of pelvic lymph nodes in the CTV is not permitted. The Planning Target Volume will have a 0.8 cm margin posteriorly and 1.0 cm margin in all other directions around the CTV to account for prostate gland motion and uncertainty in daily treatment setup.

Critical normal tissues should be delineated on the treatment-planning CT scan by the treating clinician:

- Rectum – inferior limit: level of ischial tuberosities; superior limit: sigmoid flexure
- Bladder – entirety

Two radiotherapy dose-fractionation schedules are permitted. In either case, radiotherapy is prescribed such that the PTV receives at least 95% of the prescribed dose:

- 36Gy in 6 fractions of 6Gy, administered weekly over 6 consecutive weeks
- 55Gy in 20 fractions of 2.75Gy, administered daily, five days per week, over 4 consecutive weeks

Dose-volume objectives for each dose-fractionation schedule are shown in **Tables 2** and **3** below. Values have been calculated using the formula BED = D[1+d/(alpha-beta ratio)] assuming an alpha-beta ratio of 3 for rectum and bladder. These are provided for guidance only.

Portal imaging to verify accuracy of treatment delivery may be done according to the participating centre's local guidelines. Image-guidance technology (e.g., gold seed intraprostatic fiducial markers, cone-beam CT scanning) will be permitted according to clinician preference but is not required. Further illustration on the research radiotherapy arm schedule is shown in **Figure 4**.

Table 2: Rectal dose volume objectives

55GY/20F	36GY/6F	MAX VOL (%)
52.5 Gy	33.3 Gy	50%
43.5 Gy	27.8 Gy	60%
26.1 Gy	16.7 Gy	80%

Table 3: Bladder dose-volume objectives

55GY/20F	36GY/6F	MAX VOL (%)
52.2	33.3	25%
43.5	27.8	50%

Figure 4: Diagram for deciding approach to research (M1) RT to the prostate

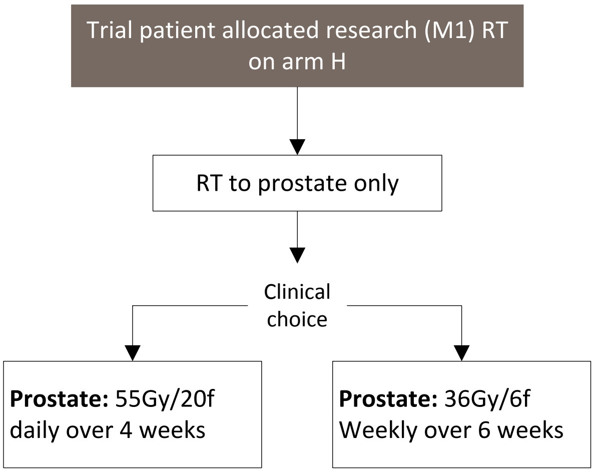

6.10 ARM J: SOC + ENZALUTAMIDE + ABIRATERONE + PREDNISOLONE

Note: recruitment to the "enzalutamide + abiraterone comparison" completed in March-2016. Please note that some patients will continue treatment until all types of disease progression or up to a maximum of 2 years. Please see sections below for more information.

Standard-of-care: androgen deprivation therapy +/- docetaxel and +/- M0 RT (as described in Section 6.2).

Abiraterone as described in Section 6.8.

Prednisolone as described in Section 6.8.

Enzalutamide will be administered as a 160mg oral dose (four capsules), taken together at the same time every day, with or without food.

Trial treatment must stop if other systemic treatments are initiated at any time for disease progression control (including chemotherapy, radium 223 etc). Anti-androgens (i.e. bicalutamide) should not be given in combination with enzalutamide due to the risk of toxicity.

In patients with M1 disease, treatment with both abiraterone and enzalutamide will continue until all types of progression have occurred, consistent with the approach taken for abiraterone (see Section 6.8) i.e., abiraterone and enzalutamide will be given until a composite of:

- PSA progression (as defined in Appendix J)
- Radiological progression (appearance of new lesions or progression of existing lesions) **and**
- Clinical progression (defined as new cancer-related symptoms).

It is accepted that these flexible criteria for stopping treatment with abiraterone and enzalutamide are open to the investigator's interpretation and discretion. Patients may continue treatment beyond the first failure-free survival (FFS) event; the first FFS event must be reported as per the other arms.

In patients with N0M0 disease or N+M0 disease undergoing radical radiotherapy, treatment would continue for 2 years or all types of disease progression as defined for M1 patients, whichever is the

sooner. ADT can be discontinued in this group at 2 years at the discretion of the local investigator (see **Section 6.2.1**).

For patients with N+M0 disease not planned for radical radiotherapy, treatment will continue as for patients with M1 disease until all types of disease progression.

If a patient has had PSA progression before commencing abiraterone and enzalutamide (i.e. on or shortly after completing docetaxel), they should still start trial treatment and should continue until radiological and/or clinical progression occurs. In the rare instances of a patient commencing abiraterone and enzalutamide having had biochemical **and** radiological progression, they should continue trial treatment whilst there is perceived evidence of benefit as judged by the local investigator. If a patient develops PSA progression only whilst on abiraterone and enzalutamide, the local investigator might consider switching from from abiraterone + prednisolone 5mg od to abiraterone and dexamethasone 0.5mg od.

See **Section 7.1.2** for further information on the definition of progression.

6.11 ARM K: SOC + METFORMIN

Standard-of-care: ADT ± RT (M0) ± docetaxel (as described in **Section 6.2**)

Metformin will be given as a daily dose in addition to standard-of-care treatment. The starting daily dose is 850mg once-daily. If tolerated, this should be increased to the target dose of 850mg twice-daily after 1 month.

Metformin will be given as a daily dose in addition to standard-of-care treatment. The starting daily dose is 850mg once-daily. If tolerated, this should be increased to the target dose of 850mg twice-daily after 1 month.

In the case of **M0 patients**, if ADT is stopped after a minimum of 2 years, metformin should continue for a minimum of 3 years following randomisation and for a further 12 months after the administration of the last LHRH (which ever is longer). This is to allow for the delay in testosterone levels returning to normal following stopping ADT. In the event that ADT is re-started whilst patients remain on metformin (i.e. within 12 months of the last administration of LHRH) then metformin should continue whilst on ADT. If metformin is stopped 12 months after the last administration of LHRH it should not be re-started in the event of relapse.

For **M1 patients** metformin should continue whilst on ADT. Treatment should continue post-progression providing it is judged to be in the patients best interest. Metformin can be given together with any additional treatments started for progression excluding other IMPs i.e. investigators may choose to stop metformin treatment post progression in order to enable patients to participate in another clinical trial evaluating treatments for CRPC.

If metformin is paused for more than 3 months or >50% of doses are missed please discuss with the trial team as treatment is likely to need to be stopped.

In the event of stopping research treatment, unless a patient states otherwise, consent is assumed for continued recording trial data.

6.12 ADMINISTRATION, DOSE MODIFICATIONS AND MANAGEMENT OF TOXICITIES

6.12.1 Zoledronic Acid or Docetaxel (arms B, C and E)

Note: recruitment stopped to the zoledronic acid and the docetaxel-containing arms in Mar-2013 as recruitment target sample was reached. Primary survival analysis was presented at ASCO in Jun-2015.
Please see Protocol version 13.0 for information on how these drugs were previously administered in the trial.

6.12.2 Celecoxib (arms D and F)

Note: recruitment completed to both celecoxib-containing arms in Apr-2011 at the end of Activity Stage II. No new patients should be receiving this agent as first-line within the trial. Primary survival analysis was presented at GU ASCO in Jan-2016.
Please see Protocol version 13.0 for information on how this drug was previously administered in the trial.

6.12.3 Abiraterone or Enzalutamide + Abiraterone (arms G and J)

Abiraterone absorption is increased by food. The tablets should be taken at least 2 hours after food, swallowed whole with some water. No food should be eaten for 1 hour afterwards. Prednisolone (prednisone in Switzerland) should be taken as a single dose with food in the morning.

Enzalutamide can be taken with or without food.

6.12.3.A Abiraterone Contraindications

- Unusual or allergic reaction to past abiraterone acetate treatment
- Uncontrolled hypertension
- Uncontrolled heart failure
- Abnormal liver function or active or chronic liver disease

6.12.3.B Abiraterone Special Warnings and Precautions For Use

Timing of administration compared with meals
Administration of abiraterone acetate with food significantly increased the absorption of abiraterone acetate, it is therefore recommended that abiraterone acetate is taken on an empty stomach.

Cardiovascular history
Abiraterone acetate should be used with caution in patients with a history of cardiovascular disease. The safety of abiraterone acetate in patients with left ventricular ejection fraction <50% or New York Heart Association (NYHA) Class III or IV heart failure has not been established. Before treatment with abiraterone acetate, hypertension must be controlled and hypokalemia must be corrected.

Caution is required in treating patients whose underlying medical conditions might be compromised by increases in blood pressure, hypokalemia, or fluid retention, e.g. those with heart failure, recent myocardial infarction, or ventricular arrhythmia.

Blood pressure management
Blood pressure should be monitored **every 2 weeks until week 12** and **at every follow-up visit after** week 12 whilst the patients remain on treatment. For the management of abiraterone induced hypertension see **Table 5.**

Hepatic Impairment

No dosage adjustment was necessary for patients with pre-existing mild hepatic impairment. Abiraterone acetate should not be used in patients with pre-existing moderate or severe hepatic impairment.

Hepatotoxicity
Marked increases in liver enzymes leading to drug discontinuation or dosage modification occurred in controlled clinical studies. Serum transaminase and bilirubin levels should be measured prior to starting treatment with abiraterone acetate**, every 2 weeks for the first 3 months of treatment, and monthly thereafter.**

If clinical symptoms or signs suggestive of hepatotoxicity develop, serum transaminases, in particular serum alanine aminotransferase (ALT), should be measured immediately. See **Table 4** for the management of abiraterone induced hepatotoxicty.

Renal Impairment
No dose adjustment are required in renal impairment; however caution is advised if patients develop severe renal impairment as there is limited clinical data in this population. Systemic exposure to abiraterone after a single oral 1000mg dose did not increase in patients with end-stage renal disease on dialysis.

6.12.3.C Abiraterone Undesirable Effects

The most common adverse drug reactions observed in the integrated safety data for those patients who received 1000mg abiraterone acetate plus prednisone or prednisolone in clinical studies (n=1,070) were fatigue, arthralgia, peripheral oedema, back pain, bone pain, nausea, constipation, hypokalemia and anaemia.

The adverse events graded as 3 or 4 and which occurred in more than 5% of patients were fatigue, peripheral oedema, anaemia and back pain see **Appendix G**.

6.12.3.D Abiraterone Overdose

There have been no reports of overdose of abiraterone acetate during clinical studies. There is no specific antidote to abiraterone acetate. In the event of an overdose, administration of abiraterone acetate should be stopped and general supportive measures undertaken, including monitoring for cardiac arrhythmias. Liver function should also be assessed.

6.12.3.E Enzalutamide Contraindications

The full induction potential of enzalutamide may not occur until approximately 1 month after the start of treatment, when steady-state plasma concentrations of enzalutamide are reached, although some induction effects may be apparent earlier. Monitoring should continue for at least the first month of treatment and dose adjustments considered. Given the long half-life of enzalutamide (5.8 days), effects on enzymes may persist for one month or longer after stopping enzalutamide. A gradual dose reduction of the concomitant medicinal product may be necessary when stopping enzalutamide treatment.

6.12.3.F Enzalutamide Special Warnings and Precautions For Use

History of seizures

Caution should be used in administering enzalutamide to patients with a history of seizures or other predisposing factors including, but not limited to, underlying brain injury, stroke, primary brain tumors or brain metastases or alcoholism. In addition, the risk of seizure may be increased in patients receiving concomitant medications that may lower the seizure threshold. Enzalutamide should be permanently discontinued in patients who have a seizure while on treatment.

Hepatic impairment

A hepatic impairment study showed that the composite AUC of enzalutamide plus N-desmethyl enzalutamide after administration of a single dose of enzalutamide was similar in patients with baseline mild, moderate or severe hepatic impairment (Child-Pugh Class A, B or C respectively) relative to patients with normal hepatic function, and no starting dose adjustment is needed.

Renal impairment

No dose adjustment are required in renal impairment; however caution is advised if patients develop severe renal impairment as there is limited clinical data in this population.

6.12.3.G Enzalutamide Overdose

There is no antidote for enzalutamide. In the setting of an overdose, stop treatment with enzalutamide and initiate general supportive measures taking into consideration the $t^{1/2}$ of 5.8 days. Patients may be at increased risk of seizures following an overdose.

6.12.3.H Management of specific toxicities due to abiraterone and enzalutamide

The safety monitoring and toxicity management plan described below takes into account AEs based on the reported clinical safety data of abiraterone and enzalutamide given separately. There are limited reported data on the safety and toxicity of the combination of enzalutamide and abiraterone however the recommendations summarised here have been updated in light of the experience gained in STAMPEDE as recommended by the STAMPEDE TMG.

Seizures

If any patient suffers a seizure whilst on treatment, enzalutamide should be discontinued immediately. Abiraterone and prednisolone can be continued providing there are no abiraterone-specific toxicities.

Table 4: Management of Abnormal Liver Function Tests (LFTs) associated with abiraterone (given alone or in combination with enzalutamide)

TOXICITY EVENT	ACTION
**Grade 1** increases in AST, ALT or bilirubin<br>(e.g. increase in AST or ALT from ULN to 2.5X ULN; increase in total bilirubin from ULN to 1.5X ULN)	The frequency of LFT monitoring should be increased to at least weekly, if the investigator judges that the laboratory abnormalities are potentially related to study medication.<br><br>No dose reduction is required.<br><br>Providing LFTs are stable for 4 weeks, resume monthly checks
**Grade 2** increases in AST, ALT or bilirubin<br>(e.g. increase in AST or ALT to >2.5-5X ULN; increase in total bilirubin from >1.5-3X ULN)	Withhold abiraterone, enzalutamide and all other concomitant medications that are potentially hepatotoxic.<br><br>The frequency of LFT monitoring should be increased to at least weekly until the liver function tests return to baseline value or grade 1 when all trial medication can be re-started.<br><br>No dose reduction is required after one episode providing this resolved within 4 weeks but should be considered if Grade 2 derangements recurs.
**Grade 3** increases in AST, ALT or bilirubin<br>(e.g. increase in AST or ALT to >5X ULN; increase in total bilirubin to >3X ULN),	Withhold abiraterone and enzalutamide and all other concomitant medications that are potentially hepatotoxic.<br><br>At least weekly monitoring is required until the LFTs return to baseline value or grade 1.<br><br>Enzaluatmide can be re-started with no dose reduction. See below for abiraterone re-challange.
**Grade 4** increases in AST, ALT or bilirubin<br>(e.g. increase in AST or ALT to >20x ULN; increase in total bilirubin to >10x ULN)	Patients must discontinue abiraterone and enzalutamide immediately.<br><br>At least weekly monitoring is required until the LFTs return to baseline value or grade 1 and then prednisone can be discontinued and the investigator can consider restarting enzalutamide.<br><br>Abiraterone should not be re-introduced.
**RE-CHALLENGE**	**ACTION**
Recurrent G2 derangement	Reduce to 750mg once LFTs return to G1
If study treatment resumption is considered for patients who have experienced Grade 3 increases in AST,	Resume study treatment with abiraterone dose reduction to 750mg when grade 3 toxicities resolve to grade 1 or baseline.

TOXICITY EVENT	ACTION
ALT, or bilirubin	
If Grade 3 or higher increases in AST, ALT or bilirubin recur after the first dose reduction	Hold study medication and all other concomitant medications that are potentially hepatotoxic. At least weekly LFT monitoring is required, starting immediately regardless of study schedule and continued until a return to baseline values or Grade 1.
If study treatment resumption is considered for patients who have experienced Grade 3 increases in AST, ALT, or bilirubin with the first dose reduction	Resume study treatment with abiraterone dose reduction to 500mg when AST, ALT or bilirubin returns to baseline value or Grade 1.

An opinion from a hepatologist should be considered if there are any concerns or liver function derangement shows no improvement within 2 weeks of discontinuation of abiraterone.

Table 5: Management of hypertension associated with abiraterone (given alone or in combination with enzalutamide)

TOXICITY EVENT	ACTION
Grade 1-2	Management as per investigator with anti-hypertensive treatment and increase frequency of blood pressure monitoring to at least weekly. Follow local guidance for selection of anti-hypertensives but avoid thiazide diuretics to minimise risk of serum potassium derrangment. Calcium channel antagonists or beta blockers are often preferred.<br><br>As with other symptoms of minerlocorticoid excess, consider increasing prednisolone dose to 5mg BID.
Grade 3-4	Withhold abiraterone and enzalutamide. Adjust or add anti-hypertensive medications to mitigate the toxicity. When hypertension resolves to Grade ≤1, resume both enzalutamide and abiraterone at full dose with prednisolone 5mg bid.

An opinion from a cardiologist should be considered if blood pressure control is not achieved within 4 weeks.

Table 6: Management of hypokalaemia associated with abiraterone (given alone or in combination with enzalutamide)

TOXICITY EVENT	ACTION
Grade 1<br>(LLN- 3.0mmol/L)	Supplement with oral potassium and monitor closely and increase prednisolone dose to 5mg BID.<br>Exclude and manage other causes of hypokalemia.
Grade 2<br>(<LLN – 3.0mmol/L and symptomatic)	Pause abiraterone<br>Supplement with oral potassium and monitor closely and increase prednisolone dose to 5mg BID.<br>Exclude and manage other causes of hypokalemia<br>Re-start abiraterone with close monitoring, discontinue if recurs
Grade 3	Abiraterone will be permenently discontinued and the patients will be

TOXICITY EVENT	ACTION
(<3.0–2.5mmol/L)<br><br>or life-threatening Grade 4 (<2.5mM)	hospitalized for intravenous potassium replacement and cardiac monitoring. After the return of serum potassium to normal, prednisolone will be discontinued. The patient can continue on enzaluatamide alone. If hypokalaemia persists consider a dose reduction of enzalutamide to 120mg once a day.

Table 7: Management of fluid retention/oedema associated with abiraterone (given alone or in combination with enzalutamide)

TOXICITY EVENT	ACTION
Grade 1-2	Increase prednisolone dose to 5mg bid.
Grade 3-4	Withhold abiraterone.<br><br>Consider addition of mineralocorticoid receptor antagonist eplerenone until resolution of symptoms. Enzalutamide can be continued. When fluid retention/oedema returns to baseline or resolves to ≤Grade 1, resume abiraterone at full dose with prednisone 5mg bid, if symptoms do not resolve abiraterone should not be re-started and enzalutamide should be dose reduced to 120 mg per day.

Table 8: Management of diarrhoea (associated with abiraterone or enzalutamide)

TOXICITY EVENT	ACTION
Grade 1-2	Symptomatic management.
Grade 3-4	Withhold abiraterone.<br>If no improvement reduce dose of enzalutamide to 120 mg per day. Once resolved to Grade 1, recommence abiraterone at 750 mg per day.

Table 9: Management of arthralgia & muscle pain

TOXICITY EVENT	ACTION
Grade 1-2	Symptomatic management
Grade 3-4	Reduce dose of enzalutamide to 120 mg per day

Table 10: Management of fatigue (associated to enzalutamide)

TOXICITY EVENT	ACTION
Grade 1-2	Consider a dose reduction to 120mg/day
Grade 3-4	Pause enzalutamide for 1 week or until the toxicity grade improves to grade 2 or lower severity. Re-started at a reduced dose (120mg/day or 80mg/day) in consultation with the study team.

6.12.3.I Management of Specific Toxicities from Prednisolone

Prednisolone/prednisone will be started at 5mg once daily, to prevent secondary mineralocorticoid excess.

Prednisolone/prednisone dose increase of up to 5mg BID is permitted to manage mineralocorticoid-related toxicities (e.g., hypokalaemia, hypertension) which are refractory to standard management.

Patients experiencing serious symptoms of cushings syndrome (e.g., weight gain, muscle loss) can decrease or discontinue (temporarily or permanently) steroids at the investigator's discretion but should be closely monitored for symptoms of secondary mineralocorticoid excess. It should be noted that weight gain and muscle loss are also associated with ADT.

6.12.4 Metformin

The starting dose for metformin is 850mg once daily. If tolerated this should be increased to the target dose of 850mg twice daily after 1 month. Metformin should be taken around the same time each day and treatment tolerance is best if taken with or after food. For twice daily dosing, the minimum time between doses should be 8 hours, doses should not be taken closer together if forgotten or missed.

6.12.4.A Metformin Contraindications

Metformin should be permanently stopped if the eGFR falls to ≤60mls/min/1.73m^2.

6.12.4.B Metformin Special Warnings and Precautions For Use

Renal impairment

Metformin is not nephrotoxic, but is exclusively excreted by the kidneys. Therefore treatment should only be started in participants with stable renal function (eGFR >60min/ml/1.73m^2). Renal function should be monitored at least every 6 months in participants with stable renal function and at least two to four times a year in participants with serum creatinine levels at the upper limit of normal and >75 years. Additional monitoring is required in any patient at risk of deteriorating renal function (see **Table 11**). If the eGFR is between 45-60 min/ml/1.73m^2 a dose reduction to 500mg BID is required in accordance with SmPC of product being used.

Lactic acidosis is a very rare (3/100,000 patient years), but serious metabolic consequence. Reported cases have occurred primarily in diabetic patients with significant renal impairment. The risk factors for lactic acidosis are: renal impairment, prolonged fasting or malnutrition, excessive alcohol intake, hepatic insufficiency or any condition associated with hypoxia e.g. cardiac or respiratory failure or circulatory shock due to any cause.

See **Table 11** for situations when metformin treatment should be paused due to the risk of deterioration in renal function.

Table 11: Situations when metformin treatment should be paused

	RISK FACTOR
Iodinated contrast agents	Pause metformin 24 hours prior to receiving contrast and re-start 48 hours post administration.
Anaesthesia (peridural; spinal or general)	Pause metformin 48 hours prior to procedure and re-start no earlier than 48 hours following procedure, providing oral intake re-established and renal function is stable and at baseline.
Surgery	Pause metformin 48 hours prior to procedure and re-start no earlier than 48 hours following procedure, providing oral intake re-established and renal function is stable and at baseline.
Dehydration e.g. nausea, vomiting or diarrhoea	Pause metformin and re-start only when oral intake is re-established and renal function is stable and at baseline.
Obstructive uropathy e.g. urinary retention or ureteric obstruction	Pause metformin and re-start only when renal function confirmed to be stable and at baseline.

Treatment breaks
It is anticipated that metformin treatment will be paused for approximately 72 hours around the time of contrast enhanced CT scans (see **Table 11**) and may need to be paused during episodes of inter-current illness. If metformin is paused for 6 days or less this information does not need to be recorded and no additional action is needed. Treatment pauses of 7 days or more should be recorded by updating the metformin treatment log.

If metformin treatment is paused for more than 2 weeks, investigators may consider re-starting at 850mg once daily for the first 4 weeks before escalating to full dose providing tolerance is acceptable. It is suggested that, providing patients have a sufficient supply of labelled IMP metformin tablets, a telephone consultation may be sufficient to assess tolerance and advice regarding dose modification in order to limit hospital visits.

If treatment is paused for more than 3 months or >50% of doses are missed for any reason the trial team should be informed as metformin may need to be discontinued.

6.12.4.C Management of Specific Toxicities from Metformin

Gastrointestinal disturbance
Gastrointestinal disturbances are very common with metformin and include nausea, vomiting, diarrhoea, abdominal pain and loss of appetite. These are most common when first starting treatment (>1/10).

Table 12: Management of metformin related gastrointestinal toxicity

TOXICITY EVENT	ACTION
Grade 1	Ensure metformin is taken with or after food.<br><br>Consider switching to 750 mg sustained release preparation if available.<br><br>If unavailable consider a 1 week treatment pause and re-start at 850mg once daily and attempt an escalation after 1 month.<br><br>If necessary, remain at 850mg OD.<br><br>If unable to tolerate 850mg OD or sustained release preparations are not available consider dose reduction to 500mg OD.
Grade 2 or higher	Reduce to 850mg OD and if symptoms improve to grade 1 or better, re-attempt dose escalation after 1 week.<br><br>If symptoms recur at grade 2 or higher, pause treatment for 2 weeks.<br><br>Re-start at 850mg sustained release or if not available 500mg OD.<br><br>Re-attempt a dose escalation 2 months later.<br><br>Continue at the maximum tolerated dose providing symptoms ≤ grade 1.

Other possible metformin related toxicites are taste disturbance and B12 deficiency resulting in megalobastic anaemia and skin reactions (see **Appendix Table 7**). Any patient who experiences anaemia whilst taking metformin should have haematinics including vitamin B12 measured and replaced if deficient.

Lactic acidosis

This is a very rare but serious toxicity which has been observed in diabetic patients taking metformin. It is unclear whether this is due to the underlying diabetes or metformin. This is supported by a meta-analysis demonstrating similar rates of lactic acidosis in diabetics taking metformin compared with diabetics not taking metformin (41). This evidence suggests this side effect may be a complication of diabetes and may not be associated with metformin treatment. The risk of lactic acidosis should be considered in the event of non-specific symptoms such as muscle cramps, abdominal pain and/or severe weakness or lethargy. Any patient with a suspected metabolic acidosis requires immediate discontinuation of metformin and evaluation. Lactic acidosis is characterised by metabolic acidosis (decreased blood pH, high lactate above 5mmol/L and an increased anion gap and lactate/pyruvate ratio). The most effective way to remove lactate and metformin is haemodialysis.

Metformin overdose

Hypoglycaemia has not been reported with metformin doses up to 85g although lactic acidosis has rarely occurred in such circumstances. Patients should be urgently assessed in the event of an overdose and hospital admission considered. The management of metformin overdoses should be as per standard clinical care by the local team. The most effective way to remove lactate and metformin is haemodialysis.

6.13 CONCOMITANT MEDICATIONS AND DRUG INTERACTION

6.13.1 ABIRATERONE: INTERACTION WITH OTHER MEDICINAL PRODUCTS AND OTHER FORMS OF INTERACTION

Details on drug interactions are described in **Appendix G.** The table below provides a summary on the main interactions.

Drugs which may interact with Abiraterone
Note: Anti-androgens. Abiraterone is steroid synthesis inhibitor and should not be given together with any other anti-androgens given the risk of toxicity. Cyproterone acetate should be discontinued 10 days and finasteride stopped 48 hours before commencing abiraterone. Concomitant use of dutasteride, bicalutamide and flutamide are all contraindicated.

Table 13: Drugs which may interact with Abiraterone

DRUGS WHICH MAY INCREASE ABIRATERONE LEVELS			
**Substrate**	**Clinical Use**	**Drug**	**Recommendation**
CYP3A4 inhibitors	Macrolide antibiotics	Clarithromycin	Avoid or hold abiraterone if short term use unavoidable given increased risk of abiraterone toxicity
	Anti-fungals	Ketoconazole<br>Itraconazole<br>Voriconazole	Avoid or hold abiraterone if short term use unavoidable given increased risk of abiraterone toxicity
**DRUGS WHICH MAY REDUCE ABIRATERONE LEVELS**			
**Substrate**	**Clinical Use**	**Drug**	**Recommendation**
CYP3A4	Anti-epileptics*	Phenytoin<br>Carbamazepine<br>Phenobarbital<br>Primadone	Contraindicated
	Anti-depressants	St Johns Wart	Contraindicated
	Anti-TB	Rifampicin<br>Rifabutin	Contraindicated
	Anti-retroviral	Atazanavir<br>Saquinavir<br>Ritonavir<br>Indinavir<br>Nelfanavir	Contraindicated. Seek specialist advice and discuss with trial team
**DRUGS WHICH MAY ACCUMULATE WHEN GIVEN WITH ABIRATERONE**			
**Substrate**	**Clinical Use**	**Drug**	**Recommendation**
CYP2D6	Cardiac	Metoprolol<br>Propranolol<br>Propafenone<br>Flecanide	Monitoring required as drug levels may increase with abiraterone use
	Anti-depressants	Desipramine<br>Venlafaxine<br>Citalopram	Monitoring required as drug levels may increase with abiraterone use
	Anti-psychotics	Haloperidol<br>Risperidone	Monitoring required as drug levels may increase with abiraterone use
	Analgesia	Tramadol<br>Codeine<br>Oxycodone	Monitoring required as drug levels may increase with abiraterone use
	Alpha blockers	Tamsulosin	Monitoring required as drug levels may increase with abiraterone use
	Anti-diabetic	Repaglinide	Monitoring required as drug levels may increase with abiraterone use

*Please note that any hisotry of epilepsy is an exclusion criteria.

6.13.2 ENZALUTAMIDE: INTERACTION WITH OTHER MEDICINAL PRODUCTS AND OTHER FORMS OF INTERACTION

Details on drug interactions are described in **Appendix G.** The table below provides a summary on the main interactions

Anti-androgens

Enzalutamide is potent androgen receptor antagonist and should not be given together with any

other anti-androgens given the risk of toxicity. Cyproterone acetate should be discontinued 10 days and finasteride stopped 48 hours before commencing enzalutamide. Concommitent use of dutasteride, bicalutamide and flutamide are all contraindicated.

Table 14: Drugs which may interact with Enzalutamide

DRUGS WHICH MAY INCREASE ENZALUTAMIDE LEVELS			
**Substrate**	**Clinical Use**	**Drug**	**Recommendation**
CYP2C8 inhibitors	Lipid-lowering	Gemfibrozil	Avoid, if no alternatives, reduce enzalutamide dose to 80mg
**DRUGS WHICH MAY DECREASE ENZALUTAMIDE LEVELS**			
**Substrate**	**Clinical Use**	**Drug**	**Recommendation**
CYP2C8 inducers	Anti-TB	Rifampicin<br>Rifabutin	Avoid and switch to an alternative if possible
CYP3A4 inducers	Anti-epileptics	Phenytoin<br>Carbamazepine<br>Phenobarbital	Contraindicated
	Anti-depressant	St Johns Wart	Contraindicated
	Anti-retrovirals	Atazanavir<br>Saquinavir<br>Ritonavir<br>Indinavir<br>Nelfanavir	Contraindicated. Seek specialist advice and discuss with trial team
**ENZALUTAMIDE MAY REDUCE DRUG LEVELS**			
**Substrate**	**Clinical Use**	**Drug**	**Recommendation**
CYP2C19	Gastric protection	Omeprazole	Omeprazole AUC reduced by 70%<br>Consider increasing dose of omeprazole for same therapeutic effect
CYP3A4	Analgesia	Fentanyl*<br>Alfentanil*<br>Tramadol	Monitor closely and consider alteratives
	Immunosuppressants	Sirolimus*<br>Tacrolimus*<br>Cyclosporine*	Monitor closely
	Anti-migraine	Ergotamine	Monitor closely
	Cardiac	Nifedipine<br>Ivabradine	Monitor closely, consider alternatives as clinical effect may be reduced
CYP2C9	Anti-epileptics	Phenytoin*	Contraindicated
	Anti-coagulants	Warfarin*	Warfarin AUC reduced by 56%<br>Consider switching to low molecular heparin, increase INR monitoring if this is not possible
**DRUGS WHICH MAY ACCUMULATE WHEN GIVEN WITH ENZALUTAMIDE**			
**Substrate**	**Clinical Use**	**Drug**	**Recommendation**
p-gp		Colchicine*<br>Dabigatran*<br>Digoxin*	Monitor closely

*narrow therapeutic index

6.13.3 Metformin: Interaction with other medicinal products and other forms of interaction

Metformin does not interact with any of the other treatments for prostate cancer and can be continued during all further treatments started on progression.

Caution is needed however when initiating potential nephrotoxic drugs as metformin is renal excreted therefore may accumulate if renal function deteriorates.

As metformin is being given as an IMP in the context of a clinical trial, continued use will not be permitted if patients participate in other interventional clinical trials for prostate cancer (i.e. CRPC setting). Investigators should use their discretion and discuss discontinuing metformin with the trial team if it is felt to be in the patient's best interest.

Table 15: Drugs which require additional monitoring of renal function

Clinical use	Drug	Recommentation
Anti-hypertensives and other cardiac disease	ACE inhibitors/angiotension II receptor blockers e.g. ramipril, lisinopril, Irbesartan	Monitor renal function until confirmed to be stable and providing eGFR remains >60mls/min/m^2 . Repeat test if necessary
	Diuretics e.g Frusemide, budesonide	
Antibiotics	Aminoglycoside antibiotics e.g Gentamycin or amikacin	Hold metformin during treatment and re-start providing renal function confirmed to be stable and eGFR remains >60mls/min/m^2
Analgesia	NSAIDS e.g. Ibuprofen, diclofenac, naproxen	Avoid if possible If no alternative increase renal monitoring to until confirmed to be stable and providing eGFR remains >60mls/min/m^2

6.14 TRIAL PRODUCTS

Details of the procedures for obtaining the drugs within the trial, dispensing and disposal of unused drug are given in **Appendix E**. Arrangements for free or discounted drugs are given in the Finance section (**Section 15**).

6.15 MEASURES OF COMPLIANCE/ADHERENCE

Date of treatment, dose, delays and reasons for delays or dose modifications of all trial treatments will be recorded. The estimated number of abiraterone tablets and enzalutamide capsules taken in a given time period will also be recorded as well as any dose reductions.

6.16 TREATMENT DATA COLLECTION

Data will be recorded on case report forms (CRFs); the top copy/original should be sent to the MRC CTU for data entry and a copy kept at the local centre. Up-to-date versions of all CRFs can be found on the trial website (http://www.stampedetrial.org/) and centres will be notified of any changes throughout the course of the trial. The type of data to be recorded is detailed in the Assessments and Procedures section (**Section 7**).

6.17 ADMINISTRATION OF STANDARD RADIOTHERAPY TO NON-METASTATIC PATIENTS

6.17.1 Treatment Details

Standard radiotherapy will be given to appropriate patients in each of the trial arms, following a period of neo-adjuvant ADT therapy, as is generally standard in UK practice. For patients with negative nodes on axial imaging, clinicians may choose between irradiating prostate and seminal vesicles alone or including the pelvic nodes in addition. Additional staging tests such as pelvic node sampling may be considered in making this decision. Conformal or intensity modulated radiotherapy should be used in all patients. Where patients have good clinical evidence that nodes are free of tumour or patients for whom nodal radiotherapy is contra-indicated (e.g. significant bowel disease), treatment may be given to the prostate gland and seminal vesicles only. The recommended dose is 74Gy in 37 fractions to the prostate and seminal vesicles or the equivalent using hypo-fractionated schedules. These recommendations are summarised in **Figure 5**. Alternative dosing schedules are permitted but must be agreed with the STAMPEDE Trial Management Group.

6.17.1.A Standard-of-care RT Timing in M0 patients

If receiving docetaxel as part the standard-of-care (permitted from Protocol version 14.0), the patient must have sufficiently recovered from any docetaxel toxicity before RT can begin.

In all other patients not receiving SOC docetaxel, SOC RT may be started sooner (2-6 months post-randomisation) in line the data from the MRC PR07 trial (8).

6.17.1.B Type Of standard-of-care RT in M0 patients

Conformal or intensity modulated radiotherapy. EBRT also permitted

6.17.1.C Standard Clinical Target Volume in M0 patients

- **CTV1:** Prostate plus seminal vesicles
- **CTV2:** (Node positive patients) Regional lymph nodes to include internal iliac and the inferior part of the common iliac nodes as used in EORTC trial 22961 (42)
- **PTV1:** CTV1 plus 10-15 mm according to local practice
- **PTV2:** CTV2 plus 10-20mm according to local practice

6.17.1.D Standard-of-care RT Dose in M0 patients

Prostate dose of 74Gy in 2Gy fractions or equivalent, with optional dose to the pelvic nodes of 55-60Gy in 2Gy fractions or equivalent dose using IMRT is recommended. Recent evidence from the CHHIP trial has shown a dose of 60Gy in 20 fractions to be non-inferior and therefore it is now also permitted. Higher nodal doses may be considered if the department is experienced in using IMRT for nodal radiotherapy, particularly as data emerges from the PIVOTAL trial of nodal IMRT in high-risk node negative patients where a nodal dose of 60Gy in 37 fractions is being evaluated. Alternative schedules should be agreed with the STAMPEDE Trial Management Group.

Figure 5: Diagram for deciding recommended approach to standard-of-care (M0) RT in non-metastatic patients

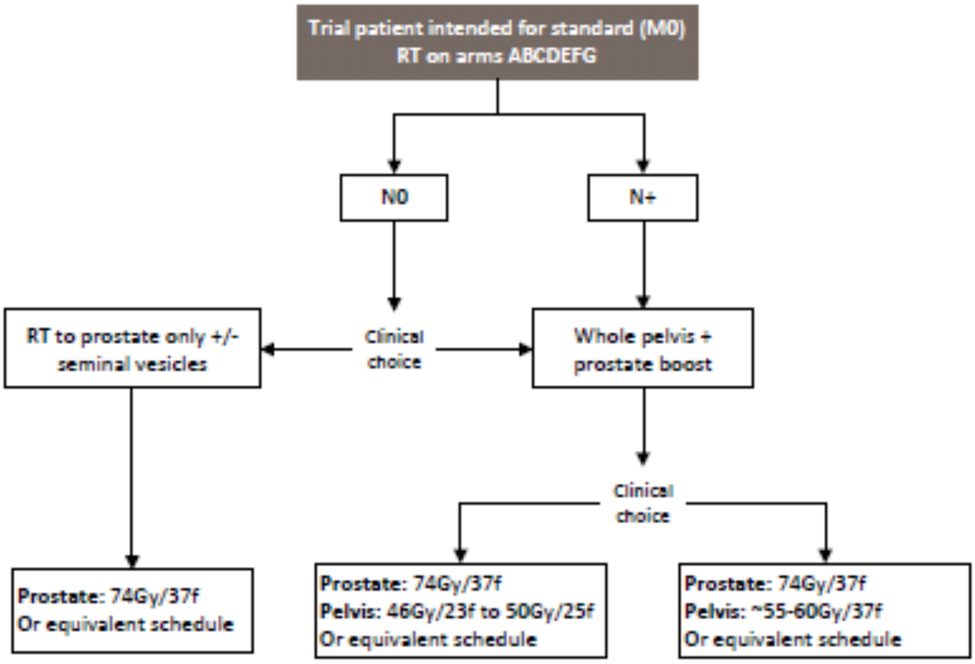

6.18 NON-TRIAL TREATMENT

6.18.1 MEDICATIONS PERMITTED

Guidance on concomitant medications and drug interaction is detailed in **Section 6.13**.

6.18.2 DATA ON CONCOMITANT MEDICATION

All concomitant medication will be recorded on the baseline form prior to randomisation and on any subsequent Serious Adverse Event forms. From Protocol version 15.0 onwards, details of long-term use of concomitant medications of classes of interest will be recorded on the follow up forms.

Please see **Section 6.13** for further details on drug interactions for abiraterone and enzalutamide.

7 ASSESSMENTS AND PROCEDURES

7.1 SCHEDULE FOR ASSESSMENTS

A detailed follow-up schedule is given in **Table 16, 17, 18 and 19**.

7.1.1 PSA Measurements

All patients should have PSA measured prior to starting Androgen Deprivation Therapy and at weeks 6, 12, 18 and 24 and every 12 weeks, thereafter, up to 2 years post randomisation. Following this, PSA should be measured every 6 months until 5 years and annually, thereafter. For patients who do not have a scheduled hospital visit, it would be acceptable for arrangements to be made for blood samples to be drawn at their GP surgery.

7.1.2 Assessment Of Treatment Failure (Definition Of Progression)

It is not proposed to routinely assess patients for response. However, in order that objective progression can be assessed, it is necessary to have imaging taken at time of best response as judged by the treating clinician. All patients should have baseline radiological examinations as detailed in **Section 4.4.1**. In addition, it is recommended all patients should have scans or X-rays repeated at 24 weeks (and whenever clinically appropriate) if they were abnormal at baseline, particularly if they have a low PSA value on entry in to the trial making biochemical assessment of treatment failure difficult.

The following outcomes should be reported on a Progression form & Additional Treatment CRF:

- Biochemical failure – should be reported alongside castrate levels of testosterone (see **Appendix J**).
- Local progression
- Lymph node progression
- Progression or development of new distant metastases, defined as lymph nodes outside the pelvis, bone or organ involvement
- Skeletal-related events (see below)

The first instance of each type of progression event should be reported. For all outcomes excluding biochemical progression, information is required on how this has been detected e.g. clinical based on symptoms or objectively based on imaging. The date of progression is defined as the earliest date of detection e.g. if clinical progression is detected and subsequently confirmed imaging please give the date of clinical progression.

Skeletal-related events (SREs) are defined as:

- Pathological Fracture
- Spinal cord compression
- Requirement for RT to bone (e.g. for pain or impending fracture)
- Requirement for surgery (e.g. for prevention or management of fracture)

SREs are a secondary outcome measure and a disease event of interest. SREs may represent disease progression but can also occur due to treatment related effects e.g. osteoporotic fracture due to treatment related bone-mineral density loss. From Protocol version 15.0 information regarding SREs will be collected at each follow up visit. All SREs should be investigated further to establish whether or not the patient has progressed and, if confirmed as progression, a Progression and Additional

Treatment CRF should be completed to record this and give details of any treatment received (e.g. palliative RT or surgery)

The summary of timing of Case Report Forms can be viewed in **Table 17**.

7.1.2.A ADDITIONAL METABOLIC AND CARDIOVASCULAR OUTCOMES

Metformin is hypothesised to mitigate the metabolic and cardiovascular effects of long-term ADT. Additional metabolic and cardiovascular outcomes are now to be collected from Protocol version 15.0 onwards to explore this effect.

The summary of timing of Case Report Forms can be viewed in **Table 16 and 17**.

Table 16: Collection of additional metabolic and cardiovascular outcomes

OUTCOME OF INTEREST	TIMING OF ASSESSMENT	CRF
**Eligibility screening**		
HbA1c	Prior to randomisation	Randomisation
**Baseline**		
Lipid profile (cholesterol, HDL, LDL)	Randomisation	Baseline
Fasting glucose	Randomisation	Baseline
Fasting triglyceride	Randomisation	Baseline
Weight and BMI	Randomisation	Baseline
Waist circumference	Randomisation	Baseline
**Follow-up**		
HbA1c	6 months<br>12 months<br>24 months	FU
Fasting glucose	6 months<br>12 months<br>24 months	FU
Fasting triglyceride	6 months<br>12 months<br>24 months	FU
Lipid profile (cholesterol, HDL, LDL)	Annual	FU
**Metabolic and cardiovascular events**		
New diagnosis of diabetes	As and when metabolic and cardiovascular event occurs	FU
Cardiac event: myocardial infarction or revascularization (e.g PCI or CABG)	As and when metabolic and cardiovascular event occurs	FU
Stroke or transient ischaemic event	As and when metabolic and cardiovascular event occurs	FU

7.1.3 ADDITIONAL SAFETY ASSESSMENT

Due to the risk of liver toxicity and secondary hyperaldosteronism with abiraterone, patients will require **2 weekly U+Es, LFTs** for the first **12 weeks and monthly BP checks**. It is not necessary to report these unless abnormal; in this instance, they should be reported as AEs (on the next Follow-up CRFs) and as SAEs (see Section 11) if appropriate. After 12 weeks, **monthly liver function tests** will be required on all patients receiving treatment on trial abiraterone.

Medical review and PSA measurements are repeated for all patients across all research arms (including the control arm) and follow the trial FU schedule (every 6 weeks for 6 months, every 12 weeks up to 2 years, six-monthly up to 5 years and annually thereafter). For patients who do not have a scheduled hospital visit, it would be acceptable for arrangements to be made for blood samples to be drawn either in a GP's surgery or in the patient's home.

Patients with normal and stable renal function receiving **metformin** require monitoring of **renal function (U&Es) every 6 months** whilst on treatment. More frequent monitoring is required in patients with declining renal function, or when initiating new potentially nephrotoxic medications or at times of intercurrent illness (see **Section 6.12.4**). Changes in renal function (eGFR, graded according to CTCAEv.4) are recorded on the follow up CRF. It is acceptable for bloods sampling to be arranged via the GP at the patient's home or local hospital.

The summary of timing of Case Report Forms can be viewed in **Table 17**.

7.1.4 DATA COLLECTION FOR STANDARD DOCETAXEL

The decision to use docetaxel as part of the standard-of-care must be made before randomisation and should be recorded on the randomisation CRF. The date of the first cycle should be recorded at the time of randomisation; this can be a planned date when randomisation occurs prior to docetaxel commencing but must be within 12 weeks of starting ADT (see **Section 6.2.3**). All further details should be recorded on the standard-of-care docetaxel CRF upon completion of the final cycle.

If a patient does not receive the planned docetaxel this must also be recorded on the standard docetaxel CRF, together with the reason why.

The summary of timing of Case Report Forms can be viewed in **Table 17**.

7.1.5. DATA COLLECTION AND NON-ADMINISTRATION OF STANDARD RADIOTHERAPY

There are CRFs to be completed for patients receiving primary radiotherapy whether this is standard-of-care radiotherapy for M0 patients (on any research arm) or research RT to the prostate for Arm H patients.

All radiotherapy and acute side effects details will be recorded on the Radiotherapy Detail and Acute Toxicity CRFs upon completion of the RT schedule; any late side effects will be recorded on the follow up form.

If RT is not given, this should be stated on the Radiotherapy Detail CRF together with the reason for non-administration of the treatment in those instances where RT was planned and not given (for example, due to early metastatic progression or patient refusal).

The summary of timing of Case Report Forms can be viewed in **Table 17**.

7.1.6. DATA COLLECTION PALLIATIVE RADIOTHERAPY

Details of palliative radiotherapy given for progressive disease should be recorded on the progression and additional treatment CRF. This includes palliative RT for SREs e.g. bone pain and spinal cord compression, as well as salvage RT to the prostate.

7.1.7 Data Collection Research (M1) Radiotherapy

All radiotherapy and acute side effects details will be recorded on the Radiotherapy Detail and Acute Toxicity CRFs upon completion of the RT schedule; any late side effects will be recorded on the follow up form.

If RT is not given as planned (for example, due to early metastatic progression or patient refusal), this should be stated on the Radiotherapy Detail CRF together with the reason for non-administration of the treatment.

The summary of timing of Case Report Forms can be viewed in **Table 17**.

7.1.8. Follow-Up Schedules

An individualised form with a follow-up schedule will be provided for each randomised patient. For patients who are receiving LHRH analogues, it is assumed that any research treatment will commence within four weeks of randomisation. For patients who are due to have an orchidectomy, it is recognised that surgery will have to be scheduled and the scheduling of any additional treatments may be affected by post-operative recovery. For patients who are receiving SOC docetaxel, it is assumed that any research treatment will commence within three to four weeks from completion of the final chemotherapy cycle.

7.2 FOLLOW-UP

Every effort should be made to follow-up all patients who have been randomised. Patients should, if possible, remain under the care of an oncologist or urologist for the duration of the trial. If care of a patient is returned to the GP, it is the responsibility of the responsible clinician who obtained the patient's consent to participate in the trial to ensure that all relevant data collection forms are completed. Nurse-led follow-up is permitted and conducted in line with local practice and procedures.

If the patient moves from the local area, arrangements should be made for trial follow-up to be undertaken by their new local centre. Details of other participating centres can be obtained from the STAMPEDE Trial Team. Details on patient transfer procedures are detailed in **Section 8.2.** If the responsible clinician moves, appropriate arrangements should be made to arrange for trial follow-up to continue at the centre.

All efforts should be made to preserve the initial patient's consent for long-term survival information to be flagged through national registries, for example the Health and Social Care Information Centre, Office of National Statistics (ONS) in England/Wales and General Register Office in Scotland, Hospital Episode Statistics (HES).

Please see **Section 8** for more information on early stopping of follow-up.

7.2.1 Follow-Up Telephone Consultations

In certain circumstances it may be appropriate to replace hospital visits with telephone consultations providing that it is still possible to collect all the necessary follow-up information. Situations where this may be considered include at the point where patients would normally be discharged from oncology or urology services. In these instances, it is acceptable to alternate appointments with telephone consultations providing the required blood results are available to the research team. All

necessary information required to complete the follow up CRF is still required. All details on the telephone consultation must be recorded in the patients' notes as per in person assessments

Other circumstances where is may be appropriate to use telephone consultations is when assessing metformin tolerance and advising regarding dose modification. This is relevant when commencing treatment for the first time or after a treatment break of 2 weeks of more when a dose escalation is advised.

7.3 TRIAL CLOSURE

For the purpose of complying with UK Clinical Regulations introduced on May 2004, the trial will be considered 'closed' when the follow-up point for the primary analysis of the final comparison has been reached. However, further observational follow-up of all patients enrolled in the trial will continue until all randomised patients have died. This will initially be via the hospital, but in the longer term may employ national registers.

Table 17: Summary of timing of case report forms

CASE REPORT FORMS	TIMING OF ASSESSMENT AND CRF
**Baseline**	
Randomisation	At randomisation
Baseline	At randomisation
Cardiovascular Assessment	At randomisation
Bone Density Risk Factor	At randomisation
Pathology	At randomisation. When pathology sample has been taken and sent to Sponsor's designated laboratory.
**Treatment**	
Standard-of-care 1st line docetaxel	Complete for all patients 20 weeks after randomisation
SOC Hormone Therapy	Form to be sent with corresponding follow-up form if there is a change in hormone therapy to report
Abiraterone and Enzalutamide Treatment	To be completed when treatment first started and updated when reported dose changes, treatment pauses and re-starting.
Metformin Treatment	To be completed when treatment first started and updated when reported dose changes, treatment pauses and re-starting.
RT detail	• When standard-of-care radiotherapy is completed or if planned RT is no longer to be given<br>• Arm H when research RT completed<br>• Arm A (M1) at 3 months<br>Once primary RT is completed for Arm H patients or those receiving RT as standard-of-care<br>For patients who did not receive primary RT (since Protocol version 9.0 regardless of being planned) this should be completed 10 months after randomisation or 3 months for newly-diagnosed M1 patients to confirm RT was not given
RT Acute Toxicity	For all patients who receive primary RT.
**Assessments**	
Follow-Up	Every 6 weeks for 6 months, then every 12 weeks until 2 years, then every 6 months until 5 years and annually thereafter. (See Table 7 for more information.)
End of Treatment	When each treatment is completed (either at end of scheduled treatment or at early cessation of treatment).
Progression & Additional Treatment	At the first occurrence of each progression event, including skeletal-related events
Additional Treatment Update	Whenever a patient who has previously progressed received additional treatment but has not experienced a new type of progression
Serious Adverse Event	Following any Serious Adverse Event
Death	At Death
**Administration**	
Patient Transfer	When a patient is transferred to a different hospital for the administration of trial treatment and follow up
Co-enrolment	When a patient is co-enrolled in any other clinical trial. Please see Section 5.1 for more information

Table 18: Schedule for completion of treatment and outcome forms by arm.

Timing From Randomisation			Treatment Log (if required)	RT	Outcome Forms	
Years	Months	Weeks			Follow-up$^{\Psi}$	QL + HE$^{¥}$
**6-Weekly**						
-	-	6	G, J, K	-	All arms	All arms
-	-	12	G, J, K	-	All arms	All arms
-	-	18	G, J, K	-	All arms	All arms
-	6	24	G, J, K	Arm H	All arms	All arms
**12-Weekly**						
-	9	36	G, J, K	-	All arms	All arms
1	12	48	G, J, K	All arms	All arms	All arms
-	15	60	G, J, K	-	All arms	All arms
-	18	72	G, J, K	-	All arms	All arms
-	21	84	G, J, K	-	All arms	All arms
-	-	96	G, J, K	-	All arms	All arms
**6-Monthly**						
2	24	104	G, J, K	-	All arms	All arms
	30	130	G, J, K	-	All arms	All arms
3	36	156	G, J, K	-	All arms	All arms
	42	182	G, J, K	-	All arms	All arms
4	48	208	G, J, K	-	All arms	All arms
	54	234	G, J, K	-	All arms	All arms
5	60	260	G, J, K	-	All arms	All arms
**Annual**						
6	72	-	G, J, K	-	All arms	All arms
7	84	-	G, J, K	-	All arms	All arms
Etc	-	-	G, J, K	-	All arms	All arms

Key:

A = SOC
B = SOC + zoledronic acid
C = SOC + docetaxel
D = SOC + celecoxib
E = SOC + zoledronic acid + docetaxel
F = SOC + zoledronic acid + celecoxib
G = SOC + abiraterone
H = SOC + M1 research RT to the prostate
J = SOC + enzalutamide + abiraterone
K = SOC + metformin

Notes:

Ψ See Table 16 for information required at follow-up
† Form records data for two cycles
‡ Form records data for three cycles
¥ 1st 700 patients and those recruited from Protocol version 8.0 onwards only

8 STOPPING OF TREATMENT OR FOLLOW-UP

Patients should be given every encouragement to adhere to their allocated protocol treatment and follow-up schedule, in order to reduce bias. However, a patient has the right to withdraw consent for participation in any aspect of this trial at any time.

8.1 STOPPING RESEARCH INTERVENTIONS

A patient may stop **any trial treatment** for the following reasons:

- Unacceptable toxicity
- Intercurrent illness which prevents further treatment
- Withdrawal of consent for treatment
- Any alteration in the patient's condition which justifies the discontinuation of treatment in the clinician's opinion

For patients randomised to **Arm G or J, trial treatment** should also be discontinued for the following reasons:

- Disease progression whilst on therapy (please refer to **Section 6.8**)
- Intention to commence a new anti-cancer treatment due to evidence of relapse

As detailed in **Section 6.8**, the disease event for stopping treatment may be after the first reportable failure-free survival event. The reason should be recorded on the relevant treatment and the End of Treatment form.

For patients randomised to **Arm K,** treatment duration is detailed in **Section 6.11**.

Reasons for early stopping of metformin can be:

- Decline in renal function (eGFR <60ml/min/1.73m^2)
- Decline in performance status (WHO PS >2)
- Unacceptable toxicity
- Patient refusal
- Intercurrent illness preventing continued metformin treatment
- Investigator decision e.g. administration of IMP within a CTIMP in CRPC setting

If metformin is paused for more than 3 months or >50% of doses are missed please discuss with the trial team as treatment is likely to need to be stopped.

In the event of stopping research treatment, unless a patient states otherwise, consent is assumed for continued recording trial data.

8.2 PATIENT TRANSFERS

For patients moving from the area, every effort should be made for the patient to be followed-up at another participating trial centre and for this trial centre to take over responsibility for the patient. To document the transfer process the main contact person at both the current and receiving hospitals should complete and sign the Patient Transfer Confirmation form.

A fully completed form must be returned to the CTU prior to the patient transfer and any outstanding data queries for the patient should be completed prior to transfer.

On receipt of the completed transfer form a member of the STAMPEDE team will confirm the database has been updated and request confirmation of the name of the patient's new clinician. Photocopies of the following documents may then be sent to the new hospital to complete the transfer and copies must be also retained at the original site for monitoring purposes:

- Consent form
- Completed CRFs
- Any documentation relating to the patient's participation in STAMPEDE (patient names must be removed from any documentation).

8.3 EARLY CESSATION OF TRIAL PARTICIPATION

If a patient explicitly withdraws consent to have any further trial data recorded their decision must be respected and the CTU must be informed in writing. All communication surrounding the early cessation of trial participation should be noted in the patient's records. Please note data prior to this decision will still be required.

In the majority of cases, patients give permission for their data and information on their health to continue to be collected via clinical notes and national registries. Any information on the follow-up status, however minimal, would be helpful. Investigators are encouraged to faciliate ongoing collection of follow up data for example, through considering telephone consultations (see **Section 7.2.1**).

Early stopping of follow-up should not be undertaken lightly and the site must consider the implications for the trial and the patient in reaching such a decision. Without long-term data, the efficacy of trial treatments would be less reliable and could lead to inconclusive results. The early stopping of trial treatment should not lead to the early cessation of trial participation and in such cases follow-up assessments should be continued as per trial protocol.

Patients can change their minds about withdrawal at any time and re-consent to participate in the trial. Follow-up data should be collected only from the point of when consent was re-instated.

9 STATISTICAL CONSIDERATIONS

9.1 METHOD OF RANDOMISATION

Patients will be randomised centrally using a computerised algorithm developed and maintained by the CTU. Randomisation will be performed using the method of minimisation over a number of clinically important stratification factors with an additional random element. To decrease determinability, the factors are not listed here but can be found in the Statistical Design Document.

Protocol version 15.0 introduces a new allocation, Arm K: SOC + metformin, and marks the closure of Arm J: SOC + enzalutamide + abiraterone with accrual completed on 31-Mar-2016. Allocation to Arm K is only available to non-diabetic patients with no contraindication to metformin.

Therefore there are three possible groups at present:

- Newly-diagnosed, non-diabetic patients with M1 disease and no contraindication to RT or metformin will be randomised A:H:K
- Newly-diagnosed patients with M1 disease and no contraindication to RT but with diabetes or with a contra-indication to metformin will be randomised A:H
- Non-diabetic patients with no contra-indication to metformin and with either newly-diagnosed M1 disease but RT contraindicated or without newly-diagnosed M1 disease will be randomised A:K.

Note that the following groups of patients are currently not eligible for randomisation:

- Patients with either diabetes or with a contra-indication to metformin and without newly-diagnosed M1 disease
- Patients with diabetes and with newly-diagnosed M1 disease but who have a contraindication to RT

See Appendix Q Table 10 for the allocation weighting of each arm by previous protocol version; this also shows allocation weighting for previously closed research arms.

9.2 OUTCOME MEASURES

The overall, definitive primary outcome measure for the trial for each comparison is overall survival (all-cause mortality).

The design of the trial is such that it is important to have additional intermediate outcome measures to assess activity in each research arm as the trial progresses. For comparisons involving research arms B to J the intermediate primary outcome measure is failure-free survival; this and other outcome measures are listed in Table 20. For the “metformin comparison” the intermediate primary outcome measure is survival; see Table 23 for full details of all outcome measures for that comparison.

The reasons for different emphases in each recruitment stage are explained in Section 9.3.

Table 19: Trial Outcome Measures by Comparison Stage (Arms B-J)

COMPARISON STAGE	PRIMARY OUTCOME MEASURE	SECONDARY OUTCOME MEASURES
Pilot phase	Safety*	Feasibility
Activity Stages (AS)	Failure-free survival (FFS)†	Overall survival (OS)<br>Toxicity<br>Skeletal-related events
Efficacy Stage (ES)	Overall survival	Quality of life<br>Cost effectiveness<br>Failure-free survival†<br>Toxicity<br>Skeletal-related events

*Based on toxicity
†Including biochemical failure (see Appendix J)

9.3 SAMPLE SIZE: PRINCIPLES

The design is a multi-arm multi-stage, multi-centre, platform, randomised controlled trial. There are a number of stages for each research arm: a Pilot/Feasibility/Safety Phase, Activity Stages and a final Efficacy Stage. Full details of the methodology underlying the trial design are given by Royston et al. (43, 44) The original sample size calculations were performed using the stage2 (version 1.2.0, March 2002) and stagen (version 1.1.1, 18 May 2004) programs, both implemented in Stata (Stata Corp, TX) and updated using the later nstage program (version 1.0.3, 13-jun-2007; version 2.1.0, 28-jun-2009; version 3.0.1, 29-Sep-2014). (45)

We have adequately powered each comparison to detect an appropriate improvement in overall survival, with high power at each of the planned interim Activity Stages and at the final Efficacy Stage. For example, in a cohort with 2 years median FFS and 4 years median survival a target HR of 0.75 for research arm relative to control would translate into an absolute improvement in FFS of 10%, from approximately 50% to 60% at two years and OS of 10%, from approximately 50% to 60% at four years. As each comparison is powered to detect a difference in relative improvement, the analyses will be performed when the pre-planned number of events has been reported in the control arm, rather than after a certain number of patients have been recruited or a certain amount of time elapsed. Further details of the sample size calculations and varying assumptions for each comparison are summarised in the relevant **Sections 9.5-8** and detailed in a separate Statistical Design Document which are available on request.

As with all trials, changes in both the standard-of-care and second line therapies over time are possible which improve outcomes and thus will affect the observed control arm event rates and associated reporting timelines. In particular, from Protocol version 8.0, standard-of-care RT was mandated for all patients with N0 M0 disease and no RT contraindication (this is likely to improve outcomes for this subgroup) and docetaxel from Protocol version 14.0. Further agents are starting to be licensed for patients with castrate-refractory disease which may also improve survival rates. Improved FFS rates would delay the intermediate analyses, for comparisons where FFS is the intermediate primary outcome measure; whilst improved survival rates would delay the definitive analyses. For each comparison event rates are estimated based on data which are publicly available

at the time of design. The Statistical Design Document includes models where median survival is varied around such estimated rates.

9.4 SAMPLE SIZE ISSUES AND TRIAL STAGES: RESEARCH ARMS B-F

For details on those five research arms opened at trial inception in 2005 please see Protocol version 13.0

9.5 SAMPLE SIZE ISSUES AND TRIAL STAGES: ADDITIONAL RESEARCH ARM G

This is the “abiraterone comparison” and includes patients allocated to research Arm G and patients contemporaneously allocated to the control Arm A.

9.5.1 PILOT PHASE: ADDITIONAL RESEARCH ARM G

A similar approach was followed for the additional research Arm G as for the original research arms. The IDMC reviewed safety data, in the context of data from the control arm, when the first 30 patients allocated to Arm G had been on trial for at least 18 weeks.

Furthermore, an additional review of safety was performed when 30 patients with newly-diagnosed non-metastatic disease, allocated to Arm G, had been on trial for at least 18 weeks. Both of these milestones were successfully completed.

9.5.2 ACTIVITY STAGES I-III: ADDITIONAL RESEARCH ARM G

The same principles were applied to this new comparison as to the original research comparisons. The notable difference was in the accrual rate to this comparison which was anticipated to be higher. There were two reasons for this. First, STAMPEDE initially started recruitment slowly in only a limited number of pilot sites. As more sites have been activated, including internationally, accrual has increased. At the time of adding Arm G (protocol version 8.0), monthly accrual to the trial was averaging around 60 patients/month (over 700 patients/year). Second, there was an equal allocation ratio for the abiraterone arm compared to the control arm. It was this different allocation ratio which meant that the number of control arm events required to trigger the intermediate analyses was lower for the assessment of abiraterone than the assessment of the original research arms. This is shown in the table below:

Table 20: Guidelines for stopping accrual to additional research Arms G and H

ACTIVITY STAGE	SIG LEVEL	POWER	TARGETED HR	NUMBER OF CONTROL ARM EVENTS	CONSIDER DISCONTINUATION IF HR(OBSERVED) IS...
I	0.50	95%	0.75	~75	>1.00
II	0.25	95%	0.75	~142	>0.92
III	0.10	95%	0.75	~221	>0.89

9.5.3 EFFICACY STAGE IV: ADDITIONAL RESEARCH ARM G

The analysis of Efficacy Stage IV for this comparison will be performed when around 267 deaths have been observed in the control arm. This will give 90% power to detect the targeted hazard ratio of 0.75 at a one-sided significance level of 0.025.

9.5.4 Sample Size For Additional Research Arm G

Up to around 1,800 patients were targeted join the abiraterone comparison, with half allocated to the research arm G; the observed allocation was 1,917. Consideration was given to ceasing further randomisations to Arm G if it was not showing sufficient evidence of activity at the interim analyses, just as was done for research Arms B to F.

The original plan intended for accrual to be halted either when 1,500 patients had been recruited or after 3 years, whichever was the sooner, providing the accrual rate remained above 50 patients/months.

The total number of patients joining this comparison depended not just on the same issues as the "original comparisons" (notably, observed accrual and event rates), but also the length of time that the original research arms co-recruited alongside this additional research arm; it was originally assumed that this would be for approximately 1 year, but it was closer to 1.5 years. The sample size calculations and projected durations are fairly robust to changes in the length of co-recruitment with the original research arms and future co-recruitment with any further research arms which the Trial Management Group may introduce. Many scenarios are detailed in the Statistical Design Document.

In Sep-2013, the target sample size for the "abiraterone comparison" was increased from around 1,500 patients to around 1,800 patients, with note that the efficacy analysis remains unchanged and is still to be triggered by around 267 control arm deaths. This increase in sample size was primarily because of an increase in the proportion of non-metastatic patients joining the comparison; this related to the activation of Arm H which only recruits patients with newly-diagnosed metastatic disease and thereby reduces the numbers of metastatic patients randomised to the "abiraterone comparison". Non-metastatic patients have a lower event rate than the metastatic patients and maintaining the same overall sample size would lead to a delay in time to the primary analysis. The increase in sample size was achievable because recruitment rates to the trial had been substantially higher than the anticipated 50 patients/month for the 6 months preceding the increase.

9.6 SAMPLE SIZE ISSUES AND TRIAL STAGES: ADDITIONAL RESEARCH ARM H

This is the “M1|RT comparison” and includes patients allocated to research Arm H and newly-diagnosed M1 patients with no contraindication to RT allocated to the control Arm A whilst Arm H is open to recruitment. Suitability for allocation to the comparison is assessed before randomisation to ensure comparability with contemporaneous control arm patients.

9.6.1 Pilot Phase: Additional Research Arm H

The IDMC reviewed safety data, in the context of data from the control arm, when the first 30 patients allocated to Arm H had been on trial for around six months.

9.6.2 Activity Stages I-III: Additional Research Arm H

The same principles were applied to this new comparison as to previous comparisons and an equal allocation ratio of control arm patients to patients allocated to Arm H was employed; as for Arm G. The number of control arm events required to trigger the intermediate analyses were the same as for the “abiraterone comparison” (see **Table 21**).

9.6.3 Efficacy Stage IV: Additional Research Arm H

The analysis of Efficacy Stage IV for this comparison will be performed when around 267 deaths have been observed in the relevant control arm patients. This will give 90% power to detect the targeted hazard ratio of 0.75 at one-sided significance level of 0.025.

9.6.4 Sample Size For Additional Research Arm H

Consideration was given to ceasing further randomisations to Arm H if it was not showing sufficient evidence of activity on the intermediate primary outcome measure (FFS), just as for the other research arms. This research comparison is relevant to around 60% of patients joining STAMPEDE. At the point of the scientific approval, accrual was averaging around 80 patients per month to the trial. If accrual to the trial was slower at 70 patients per month, then accrual to this comparison could be between 18 and 42 patients per month, depending on which other trial arms are open to recruitment at the time.

We are targeting a 25% relative improvement in overall survival following local radiotherapy to the prostate in this patient group. This is the same size of effect targeted with the other research arms in STAMPEDE. This relative improvement can be further justified in the light of MRC PR07 which demonstrated an improvement of this magnitude for adding radiotherapy to ADT in locally advanced disease, with a hazard ratio for overall survival of 0.77 (95% CI 0.61 to 0.98). In that trial, fewer than half of the deaths were from prostate cancer, whereas in newly-diagnosed metastatic patients nearly all men will die of their disease. Therefore, it is relevant to note the relative benefit of radiotherapy in PR07 in terms of prostate cancer-specific survival, where the hazard ratio was 0.54 (95% CI 0.27 to 0.78). Long-term survival-based data, with a median follow-up of ~10 years, were presented orally at the American Society of Clinical Oncology 2012 which confirmed these findings.(7)

We anticipated that around 1250 patients were required over 4 years to observe 267 control arm deaths after 5.25 years. This assumed that (i) recruitment was constantly 70 pts/m to the trial overall; (ii) the original research arms stopped accrual within 6 months after activation of the RT arm; (iii) the abiraterone arm stops accrual around 24 months after activation of the RT arm; and (iv) a further new research arm with an equal allocation ratio was introduced 18 months after activation of the RT arm. In protocol version 13.0, we reflected on these four points: (i) recruitment to the trial has been faster; (ii) the original research arms completed accrual 2 months after activation of the RT arm; (iii) the abiraterone arm stopped accrual 12 months after activation of the RT arm; and (iv) Arm J was activated 18 months after activation of the RT arm, Arm H.

Of patients joining STAMPEDE during this time, 60% have been eligible for the “M1|RT comparison”. Prior to randomisation, a RT schedule must be nominated: Weekly or Daily (see **Section 6.9**). We have observed that around half of patients in the comparison are nominated for RT with the Daily schedule and half for the Weekly schedule, primarily chosen by trial site with patient groups nominated for each schedule observed to be comparable at baseline. There will likely be interest to know the effect of each RT schedule when the main results are reported. This will be explored by “within schedule” comparisons of patients randomised to research vs control (arms H vs A) within each nominated RT schedule.

Therefore, in protocol version 13.0, the target sample size was increased from 1,250 patients up to around 1,800 patients, resulting in an approximate increase in the split by planned RT schedule from 625 to 900 in each “within schedule” analysis. A FFS analysis “within schedule” will be carried out at the time of the “main analysis”; predicted to have ~300 control arm FFS events by schedule (FFS “within schedule” analysis parameters: target HR=0.75, power 90%, 1-sided α=0.015). For either of the RT schedules showing evidence of an effect on FFS, a comparative “within schedule” analysis will be carried out on survival when ~199 control arm deaths are observed in that schedule comparison. This is a closed test with OS only formally compared within schedule if there is an advantage in FFS for that RT schedule at the main analysis. Thus, extending recruitment enables a secondary analysis

of the impact of RT on survival by planned "RT schedule" to happen within around 18 months from the first main analysis.

All sample scenarios are documented in the Trial Master File.

All patients joining the trial will be starting long-term ADT for the first time. The focus of this comparison will be on the newly-diagnosed, metastatic patients (with no contraindications to RT), which is the largest subgroup of patients in the trial and the group of patients at highest risk of death from prostate cancer. Patients with non-metastatic disease will be excluded from this particular comparison as there are already randomised data demonstrating the survival benefit from radiotherapy in patients with locally advanced disease. Radiotherapy is now mandatory in node negative patients; it is also recommended in the node-positive, non-metastatic (N+ M0) group. Relapsing patients are also excluded from this comparison.

For the control arm of the whole trial, we constructed sample size scenarios median failure-free survival being 18, 24 or 30 months and constructed sample size scenarios around each of these options; the event rate would depend on the patient mix. We now know that around 60% of patients have M1 disease at trial entry and we have reported that FFS at 24 months is 51% across the whole of the control arm.(1)

For the updated sample size calculation for this comparison, we based our estimates on the subgroup of patients with newly-diagnosed M1 disease in the control arm. Therefore, we estimate median FFS to be 1 year and estimate that median overall survival will be around 3.5 years.

9.7 SAMPLE SIZE ISSUES AND TRIAL STAGES: ADDITIONAL RESEARCH ARM J

This is the "enzalutamide + abiraterone comparison" and included patients allocated to research Arm J and patients contemporaneously allocated to the control Arm A.

9.7.1 Pilot Phase: Additional Research Arm J

The IDMC first reviewed safety data for this combination when the first 50 patients allocated to Arm J had been on trial around 6 weeks (i.e. to the first follow-up visit).

Furthermore, an additional review of safety was performed when these 50 Arm J patients had been on trial for around 6 months. Safety is routinely reviewed at regular intervals and additional safety reviews will be performed if the IDMC raises any concerns.

Direct comparison will be available with contemporaneously randomised patients on Arm A (hormones alone). Contextual data will be provided from Arm G (hormones plus abiraterone). Indicative safety data may also be available on the combination from other studies in CRPC.

9.7.2 Activity Stages I-II: Additional Research Arm J

The principles of intermediate analyses will be applied to this new comparison as to previous comparisons, but some of the details will be different, and an equal allocation ratio of control arm patients to patients allocated to Arm J was employed; as for Arms G and H. Owing to the expected accrual rate to the trial (>100 pts/m) and the expected slower event rate, only two activity stages are planned before accrual is completed. These are set out in **Table 22**.

Table 21: Guidelines for stopping accrual to the additional research Arm J

Activity Stage	Sig Level	Power	Targeted HR	Number of control arm events	Consider discontinuation if HRJ(observed) is…
I	0.40	95%	0.70	~66	>0.957
II	0.12	95%	0.70	~139	>0.869

9.7.3 Efficacy Stage III: Additional Research Arm J

The analysis of the final Efficacy Stage for this comparison will be performed when around 267 deaths have been observed in the control arm. This would give 90% power to detect the targeted hazard ratio of 0.75 at a one-sided significance level of 0.025.

9.7.4 Sample Size For Additional Research Arm J

Consideration was given to ceasing further randomisations to Arm J if it was not showing sufficient evidence of activity on the intermediate primary outcome measure (FFS), just as for the other research arms.

The patient mix for this comparison is likely to represent a more favourable prognosis on average than in the original research trial's other arms, due to concurrent recruitment of M1 but not M0 patients, to Arm H.

We anticipate that around 1,800 patients are required within 3.5 years to observe ~267 control arm deaths within 6 years. This time will be dependent on the observed overall survival. The default scenario assumes that (i) recruitment is constantly 70pts/m to the trial overall, (ii) the M1|RT arm H accrues throughout and (iii) a further new research arm with an equal allocation ratio is introduced 18 months after activation of Arm J. The stopping date for Arm G is no longer an assumption.

Variations on these factors are documented in a Statistical Design Document. If accrual rates to the trial are at 150pts/m (as observed during Summer 2013), accrual of around 1,800 patients to the comparison could be achieved within 2 years. These sample scenarios will also be documented in the Trial Master File.

Updating the standard-of-care to include docetaxel has minimal impact on the projected time to maturity of the "enzalutamide + abiraterone comparison".

9.7.5 Further Sample Size Issues For Additional Research Arm J

Careful consideration will be given to the implications of any emerging data from the "abiraterone comparison". This would have minimal to no effect on the "enzalutamide + abiraterone comparison" because the recruitment target will be reached before any data are available from the "abiraterone comparison".

Indirect comparisons to understand the contribution from each agent may be possible if this research arm is demonstrably superior to the standard-of-care. These plans will be developed and documented elsewhere, but a higher number of patients will help with the power to the indirect comparison.

9.8 SAMPLE SIZE ISSUES AND TRIAL STAGES: ADDITIONAL RESEARCH ARM K

This is the “metformin comparison” and includes patients allocated to research Arm K and the equivalent non-diabetic patients with no contraindication to metformin contemporaneously allocated to the control Arm A whilst Arm K is open to recruitment. Suitability for allocation to the comparison is assessed before randomisation to ensure comparability with contemporaneous control arm patients

9.8.1 Implementation: Additional Research Arm K

The implementation of the MAMS principles are different in this comparison for the following reasons:

- Although all non-diabetic patients will be eligible for allocation to the “metformin comparison”, the timing of the analyses will be driven only by the M1 patients.
- Failure-free survival will not be used as the intermediate primary outcome measure; overall survival will be used as both the intermediate and definitive primary outcome measure. This is because we are not convinced that any comment on metformin’s usefulness should be determined from an ability to act on a PSA-driven outcome measure. Furthermore, treatment with metformin is intended to continue throughout long-term hormone therapy which may include going well beyond an FFS event, particularly in M1 patients.
- The target HR is 0.80 for overall survival (a 20% relative improvement). This is a smaller relative improvement in survival than targeted for previous comparisons because of metformin’s known low toxicity profile, the low cost of the drug and the potential positive effects on metabolic parameters and morbidity; a smaller impact may still have clinical benefit.

9.8.2 Outcome Measures: Additional Research Arm K

Table 22 lists the outcome measures for this comparison and can be compared with the outcome measures for the other comparisons in **Table 19**.

Table 22: Trial outcome measures by stage for the "metformin comparison"

COMPARISON STAGE	PRIMARY OUTCOME MEASURES	SECONDARY OUTCOME MEASURES
Pilot phase	Safety*	Feasibility<br>Metabolic effects including:<br>:: Changes in BMI<br>:: Changes in haemoglobin A1c (HbA1c)<br>:: Changes in waist circumference<br>:: New diagnosis of diabetes mellitus<br>:: Cardiovascular event: major adverse cardiac events‡
Activity Stage (AS) I	Overall survival	Metabolic effects<br>Toxicity<br>Symptomatic skeletal events<br>Failure-free survival† (FFS)
Efficacy Stage (ES) II	Overall survival	Metabolic effects<br>Toxicity<br>Symptomatic skeletal events (SSE)<br>Failure-free survival† (FFS)<br>Quality of life<br>Cost effectiveness<br>Correlative outcomes₳

*Based on toxicity
‡MACE; nonfatal MI, nonfatal stroke, and death from cardiovascular causes
†Including biochemical failure (see Appendix J)
₳Plasma lipid and fasting triglyceride levels, fasting plasma glucose
Sarcopenia and/or radiological progression free survival (rPFS)
Plasma insulin
AMP Kinase
Note: All arms are unblinded so primary outcome measures for this comparison are objectively measured with caution to be taken around interpretation of more subjective secondary outcome measures such as symptomatic skeletal events

9.8.3 Pilot Phase: Additional Research Arm K

The IDMC will review safety data for this comparisons when the first 50 patients allocated to Arm K have been on trial around 12 months. Furthermore, analyses will be conducted on metabolic parameters (see **Table 23**). If there is harm observed in metabolic effects, or any serious concerns regarding the toxicity profile, recruitment would be stopped; there are no formal criteria to guide this.

Safety is routinely reviewed at regular intervals and additional safety reviews will be performed if the IDMC raises any concerns.

9.8.4 Activity Stage I: Additional Research Arm K

The principles of intermediate analyses will be applied to this new comparison as to previous comparisons, but some of the details will be different, and an equal allocation ratio of control arm patients to patients allocated to Arm K is employed; as for Arms G, H and J. Owing to the expected accrual rate to the trial overall (>100 pts/m) and the interim primary outcome being overall survival, only one intermediate activity stage is planned before accrual is completed; this is set out in the **Table 24**.

Although analyses are triggered by events in M1 patients, they will include all patients in the "metformin comparison"; this will have high power. A separate subgroup analysis in M1 patients (conventionally-powered) and M0 patients (limited power) will then look at consistency of effect; there will be few deaths in M0 patients at this time. The IDMC recommendation will be based on the totality of the available data, including safety, metabolic and compliance data.

Table 23: Guidelines for stopping accrual to the additional research Arm K

Activity Stage	Sig Level	Power	Targeted HR	Number of control arm events	Consider discontinuation if HR_K (observed) is...
I	0.40	90%	0.80	~104 M1 deaths	>0.965

9.8.5 Efficacy Stage II: Additional Research Arm K

The analysis of the final Efficacy Stage for this comparison will be performed when around 374 deaths have been observed for M1 patients randomised contemperaneously to the control arm. This would give 85% power to detect the targeted hazard ratio of 0.80 at a one-sided significance level of 0.025.

As with the intermediate activity, this analysis will include all patients in the comparison, with a separate subgroup analysis in M1 and M0 patients looking at consistency of effect. At this time point we predict <60 control arm M0 deaths will be observed. Further subgroup analyses, defined by the stratification factors, are planned to check for consistency of effect at intermediate and final analyses.

9.8.6 Sample Size For Additional Research Arm K

Consideration will be given to ceasing further randomisations to Arm K if it is not showing sufficient evidence of improvement on overall survival at the intermediate analysis.

We anticipate that around 1,800 patients, including around 1,100 M1 patients, are required over 3 years to observe ~374 control arm M1 deaths over around 8 years. This number and time will be dependent on the observed overall survival. The default scenario assumes (i) recruitment is constantly 100pts/m to the trial overall, (ii) co-recruitment throughout of the equivalent of one other research arm, and (iii) the majority of metastatic patients will also have docetaxel but non-metastatic patients will not.

Variations on these factors are documented in a Statistical Design Document. If accrual rates to the trial are at 150pts/m (as observed during summer 2013), accrual of around 1,800 patients to the comparison could be achieved within 2 years. These sample scenarios will also be documented in the Trial Master File.

Updating the standard-of-care to permit first-line use of docetaxel was assumed within the sample size scenarios and is reflected in the projected time to maturity of the "metformin comparison".

9.8.7 Further Sample Size Issues For Additional Research Arm K

Careful consideration will be given to the emerging data from the "abiraterone comparison" when this reports in 2017.

Analyses for the “metformin comparison” will be timed from randomisation. The point of randomisation compared to the start of hormone therapy may differ, depending on the planned use of docetaxel. This practical information will be reviewed by the TMG and IDMC.

- **Schema of progress of STAMPEDE through the trial**

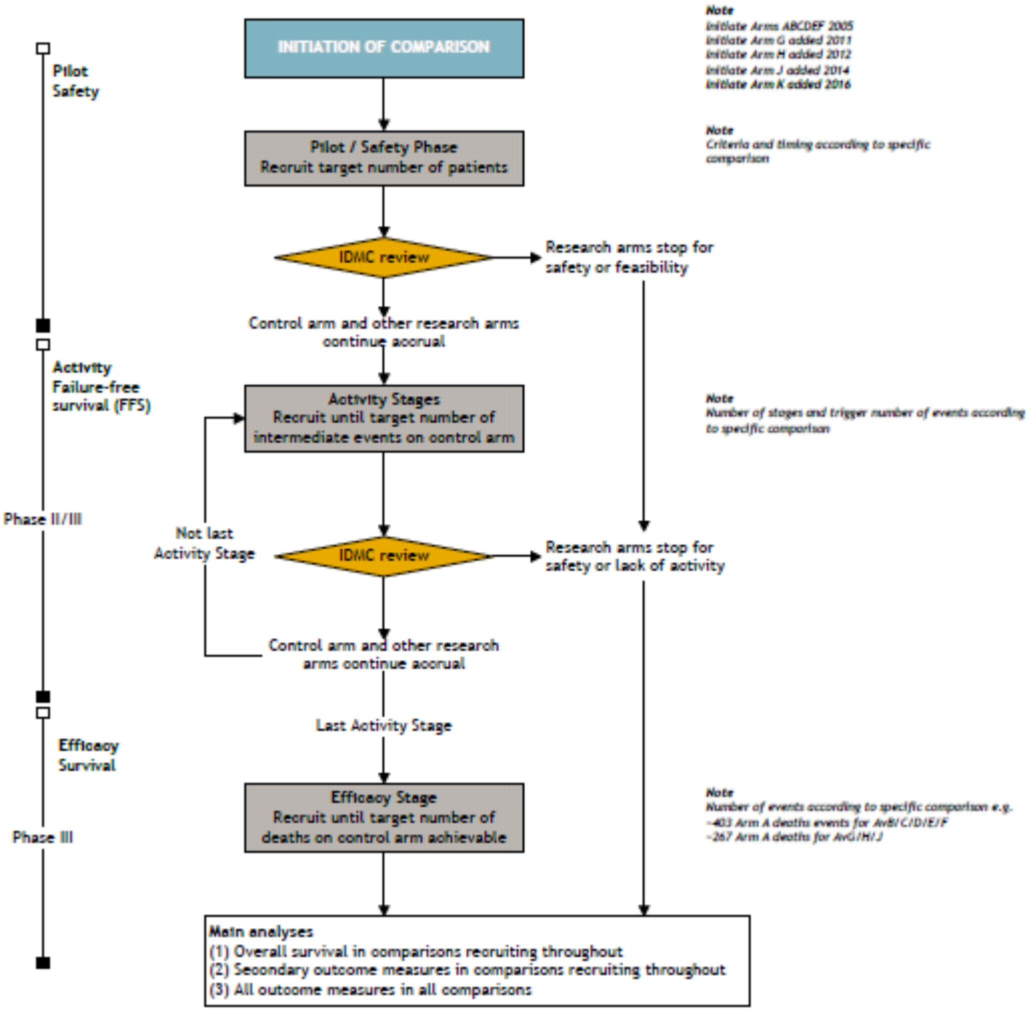

Key
FFS: Failure-free survival
HR: Hazard ratio
IDMC: Independent Data Monitoring Committee
Pts: Patients

Notes
Exact accrual depends on many factors including accrual rate, event rate and arms recruiting in each stage

9.9 FURTHER NOTES ON TRIAL DESIGN

9.9.1 Overall Sample Size

Given the adaptive nature of the study, there is no formal overall sample size target, but the numbers of patients required for each comparison are detailed in **Sections 9.5-8**. To date, more than 8,000 patients have been recruited overall.

9.9.2 Factorial Design

We note here that we have not employed a factorial design in the original design of this trial because we anticipate the possibility of synergy between SOC, zoledronic acid and docetaxel and between SOC, zoledronic acid and celecoxib.

It would not be possible to assess any such interactions reliably in a factorial trial (see the Statistical Design Document for further details).

9.10 INTERIM MONITORING AND ANALYSES

The accumulating data will be reviewed at regular intervals (approximately annually) by an Independent Data Monitoring Committee (IDMC), including pre-specified formal intermediate analyses of activity data (see also **Section 16**). These analyses will be performed by the trial team at the CTU. Only patients randomised contemporaneously, and eligible for that comparison, will be included in the comparison of each research arm against control e.g. patients allocated to the control arm prior to Protocol version 12.0 will not contribute to the "enzalutamide + abiraterone comparison" (Arm A vs Arm J).

The IDMC will be asked to give advice on whether the accumulating data from the trial justifies continuing recruitment of further patients or further follow-up; guidelines for discontinuation of accrual for the relevant Activity Stages, together with results from any other relevant trials will aid them in this. A decision to discontinue recruitment, either in all patients or in selected subgroups, will be made only if the result is likely to convince a broad range of clinicians including those entering patients into the trial and the general clinical community. The intermediate stopping guidelines apply to the intermediate primary outcome measure. To stop accrual early for benefit in any comparison would require convincing data in terms of the definitive primary outcome measure, overall survival. For example, this could be $p<0.001$ as proposed by Haybittle-Peto.(46, 47) The use of such a guideline for stopping for benefit has a minimal impact on the operating characteristics.

If a decision is made to continue without change, the IDMC will advise on the frequency of future reviews of the data on the basis of accrual and event rates. The IDMC will make recommendations to the Trial Steering Committee (TSC, see **Section 16**) as to whether the trial should continue in its present form. While the trial is ongoing the accumulating data will generally remain confidential, unless the TSC and IDMC agree that the data should be made public.

9.11 OUTLINE ANALYSIS PLAN

Analyses will be performed on an intention-to-treat basis. The standard unadjusted log-rank approach will be applied to analyses of FFS and OS. The impact of potential confounders including the stratification factors used at randomisation will be considered in a Cox proportional hazard model. Flexible parametric models will be used to calculate the absolute differences between the arms to show treatment differences over time and to estimate restricted mean “survival” times (RMST). The estimated difference in restricted means survival time (RMST) will be used preferentially to compare treatment arms if the proportional hazards assumptions required for

hazard ratios cannot be supported. The $\chi2$ test or Mann-Whitney test will be implemented for categorical data comparisons, including toxicity, as appropriate. Where relevant the primary outcome measures (see **Section 9.2**) will be considered for all arms of the trial at each phase, but the main emphasis will be placed on the comparison of the research arms that have continued to recruit throughout the trial.

9.11.1 Pilot / Safety Phases

The Pilot Phase randomised patients between all the trial arms so that the results from these patients can be included in the main trial. Feasibility is considered in terms of acceptability of the trial randomisation and reported toxicities and adherence to trial medication. Centres participating in the Pilot Phase for the original research arms were required to keep an anonymised log of all patients assessed for trial eligibility (see Protocol version 2.0) so that the number of patients who did not participate in the study and the number of eligible patients who chose to not participate in the study could be summarised (reasons for non-participation were collected where the patients was willing). The anonymised logs are no longer needed for new research arms (since Protocol version 8.0).

For the patients who are randomised, we shall describe the incidence of expected and unexpected severe toxicities and adverse events/reactions (see **Section 11**) to decide whether to continue with research arms beyond the Pilot Phase.

9.11.2 Activity And Efficacy Stages

The approach to analysis of these stages is summarised within the sample size calculations (see earlier subsections of **Section 9.4.3**). Each research arm will be compared in a pairwise fashion against the contemperaneously recruited control arm.

Full details are available in the Statistical Analysis Plan. See **Figure 6** for an overview of the schema of progress.

10 MONITORING AND QUALITY ASSURANCE

10.1 MONITORING AT MRC CTU

Data provided to the MRC CTU will be checked for missing or unusual values (range checks) and consistency over time. If missing or questionable data are identified, staff at the MRC CTU will request that the data be clarified. The exact procedures for data clarification and the amendment of CRFs will be described in the trial Data Management Plan and instructions will be sent to all STAMPEDE institutions as soon as they have been approved to participate in the trial. The MRC CTU will also send reminders for any overdue data.

Anonymised copies of the initial patient's consent form and any subsequent re-consent should be sent to the STAMPEDE team at the MRC CTU.

10.2 DIRECT ACCESS TO DATA

Collaborating institutions should be aware that direct access to patient data by MRC CTU staff may be required for trial-related monitoring or audit. Patient consent for this will be obtained as part of the general trial consent process.

10.3 VISITS TO INVESTIGATOR SITES

A selection of institutions will be visited at least once during the course of the STAMPEDE trial. The MRC CTU will give the responsible investigator adequate notice of the monitoring visit to allow adequate time, space and staff for these visits. The standard operating procedures (SOP) for monitoring are available from the MRC CTU.

After the monitoring visit the monitor will complete a site visit report. This report may be circulated to the TMT for comment. Once the TMT have reviewed the report and agreed on any recommendations the monitor will finalise the report and send a copy to the Principal Investigator (PI) at the site. A copy will be kept in the MRC CTU STAMPEDE Trial Master File.

10.4 CONFIDENTIALITY

All information collected during the course of the research will be kept strictly confidential. In addition, all procedures for handling, processing, storage and destruction of data are compliant with the Data Protection Act 1998. No individual patients will be identified when results from the trial are published.

Patients will be asked for permission for information about their health status to be obtained from the Office of National Statistics (ONS) or via the HSCIC or similar by the Medical Research Council, if necessary. In addition, patients will be asked for permission to inform their GP of their involvement in the STAMPEDE trial.

11 SAFETY REPORTING

ICH GCP requires that both investigators and sponsors follow specific procedures when reporting adverse events/reactions in clinical trials. These procedures are described in this section of the protocol. Further information on the expected toxicities for the trial interventions (docetaxel, zoledronic acid, abiraterone, radiotherapy, enzalutamide and metformin) can be found in **Appendix G**.

11.1 DEFINITIONS

The safety reporting definitions from ICH GCP apply in this trial protocol. These definitions are given in **Table 26**.

Table 24: Event Terms and Definitions

TERM	DEFINITION
Adverse Event (**AE**)	Any untoward medical occurrence in a patient or clinical trial patient to whom a medicinal product has been administered including occurrences which are not necessarily caused by or related to that product.
Adverse Reaction (**AR**)	Any untoward and unintended response to an investigational medicinal product related to any dose administered.
Unexpected Adverse Reaction (**UAR**)	An adverse reaction, the nature or severity of which is not consistent with the information about the medicinal product in question set out in the summary of product characteristics (or Investigator brochure) for that product.
Serious Adverse Event (**SAE**) or Serious Adverse Reaction (**SAR**) or Suspected Unexpected Serious Adverse Reaction (**SUSAR**)	Respectively any adverse event, adverse reaction or unexpected adverse reaction that:<br>• results in death<br>• is life-threatening*<br>• requires hospitalisation or prolongation of existing hospitalisation**<br>• results in persistent or significant disability or incapacity<br>• consists of a congenital anomaly or birth defect<br>• Other important medical condition***

Clarifications and Exceptions

*The term 'life-threatening' in the definition of 'serious' refers to an event in which the patient was at risk of death at the time of the event; it does not refer to an event which hypothetically might have caused death if it were more severe.

**Hospitalisation is defined as an inpatient admission, regardless of length of stay, even if the hospitalisation is a precautionary measure for continued observation. Hospitalisations for a pre-existing condition (including elective procedures that have not worsened) do not constitute an SAE.

***Medical judgement should be exercised in deciding whether an AE/AR is serious in other situations. Important AE/ARs that are not immediately life-threatening or do not result in death or hospitalisation but may jeopardise the patient or may require intervention to prevent one of the other outcomes listed in the definition above, should also be considered serious.

Pregnancy occurring in a STAMPEDE patient's partner during the patient's participation in the trial, must be reported to the MRC CTU within the same timelines as an SAE and classified as an 'other important medical condition' on the SAE form. The outcome of a pregnancy should be followed up carefully and any abnormal outcome to the mother or child should be reported.

Patients who develop any new primary carcinomas should have the event reported on a SAE CRF as "other important medical condition".

11.1.1 TRIAL-SPECIFIC EXEMPTIONS

Disease progression or death as a result of disease progression are not considered to be SAEs and should be reported on the STAMPEDE Progression Form or Death Form only.

The following situations that fulfil the definition of an SAE are excluded from expedited notification on an SAE form and should be reported only on the STAMPEDE follow-up form:

- Elective hospitalisation and surgery for treatment of locally advanced or metastatic prostate cancer or its complications
- Elective hospitalisation to simplify treatment or procedures
- Elective hospitalisation for pre-existing conditions that have not been exacerbated by trial treatment

Furthermore, given the solid accumulated evidence on its safety profile (38, 48), hospitalisation or prolongation of hospitalisation for the following SOC docetaxel-related events is exempted from reporting:

- Febrile neutropenia
- Thrombocytopenia

However, it is expected that investigators continue to report the abovementioned expected reactions via the MHRA Yellow Card Scheme (https://yellowcard.mhra.gov.uk/).

The exemption does **not** apply for events resulting in death; these should be reported as per procedures detailed in **Section 11.2.1.D.**

11.2 INSTITUTION/INVESTIGATOR RESPONSIBILITIES

All non-serious AEs/ARs, whether expected or not, should be recorded in the toxicity (symptoms) section of the Follow-up CRF and sent to the MRC CTU within one month of the form being due. SAEs/SARs should be notified to the MRC CTU as described below.

The severity (i.e. intensity) of all AEs/ARs (serious and non-serious) in this trial should be should be graded using Common Terminology Criteria for Adverse Events (CTCAE) v3.0 (ctep.cancer.gov/reporting/index.html). Any questions concerning this process should be directed to the MRC CTU in the first instance.

11.2.1 INVESTIGATOR ASSESSMENT

11.2.1.A Seriousness

When an AE/AR occurs the investigator responsible for the care of the patient must first assess whether the event is serious using the definitions given in **Table 25**. If the event is serious and not exempt from expedited reporting, then an SAE form must be completed and the MRC CTU notified.

11.2.1.B Causality

The Investigator must assess the causality of all serious events/reactions in relation to the trial therapy using the definitions in Table 25. There are 5 categories: unrelated, unlikely, possible, probable and definitely related. If the causality assessment is unrelated or unlikely to be related the event is classified as a SAE. If the causality is assessed as either possible, probable or definitely related then the event is classified as a SAR.

Table 25: Assigning type of SAE through causality

RELATIONSHIP	DESCRIPTION	EVENT TYPE
Unrelated	There is no evidence of any causal relationship	Unrelated SAE
Unlikely	There is little evidence to suggest there is a causal relationship (e.g. the event did not occur within a reasonable time after administration of the trial medication). There is another reasonable explanation for the event (e.g. the patient's clinical condition, other concomitant treatment).	Unrelated SAE
Possible	There is some evidence to suggest a causal relationship (e.g. because the event occurs within a reasonable time after administration of the trial medication). However, the influence of other factors may have contributed to the event (e.g. the patient's clinical condition, other concomitant treatments).	SAR
Probable	There is evidence to suggest a causal relationship and the influence of other factors is unlikely.	SAR
Definitely	There is clear evidence to suggest a causal relationship and other possible contributing factors can be ruled out.	SAR

11.2.1.C Expectedness

If the event is a SAR the Investigator must assess the expectedness of the event. Please see **Appendix Table 7** for a list of expected toxicities associated with the drugs being used in this trial. If a SAR is assessed as being unexpected it becomes a SUSAR.

11.2.1.D Notification

Investigators must notify the MRC CTU of all SAEs occurring from the time of randomisation until 30 days after the last protocol treatment administration for any research arm in the trial, including standard-of-care treatments, HT and docetaxel. Similarly, SAEs occurring in patients randomised to Arm A must be reported until 30 days after last injection or progression (whichever is sooner). SARs and SUSARs must be notified to the MRC CTU indefinitely for all arms (i.e. no matter when they occur after randomisation). See Table 26 for a summary of the SAE reporting timelines by treatment

SAE REPORTING

Fax to 020 7670 4818 within 24 hours of becoming aware of the event

Table 26: SAE reporting timelines by treatment

TREATMENT	SAE	SAR	SUSAR
ADT	Up to 30 days after last injection or progression (whichever is sooner	Indefinitely	Indefinitely
Docetaxel (Research)	Up to 30 days after last treatment	Indefinitely	Indefinitely
Docetaxel (SOC)	Up to 30 days after last treatment	Indefinitely	Indefinitely
Zoledronic Acid	Up to 30 days after last treatment	Indefinitely	Indefinitely
Celecoxib	Up to 30 days after last treatment	Indefinitely	Indefinitely
Research RT (Arm H)	Up to 30 days after last treatment	Indefinitely	Indefinitely
Abiraterone	Up to 30 days after last treatment	Indefinitely	Indefinitely
Enzalutamide	Up to 30 days after last treatment	Indefinitely	Indefinitely
Metformin	Up to 30 days after last treatment	Indefinitely	Indefinitely

11.2.2 NOTIFICATION PROCEDURE

The SAE form must be completed by the Investigator (consultant named on the signature list and delegation of responsibilities log who is responsible for the patient's care), with due care being paid to the grading, causality and expectedness of the event as outlined above. In the absence of the responsible investigator the form should be completed and signed by a member of the site trial team. The responsible investigator should subsequently check the SAE form, make changes as appropriate, sign and then re-fax to the MRC CTU as soon as possible. The initial report shall be followed by detailed, written reports as appropriate.

Send the SAE form by fax to the MRC CTU. Fax Number: + 44 (0) 20 7670 4818. The STAMPEDE trial team will confirm receipt of the SAE report to the main point of contact via email. Contact the STAMPEDE trial team If receipt is not received within 24 hours.

Follow-up: Patients must be followed-up until clinical recovery is complete and laboratory results have returned to normal or baseline, or until the event has stabilised. Follow-up should continue after completion of protocol treatment if necessary. Follow-up information can be updated on the originial SAE form by ticking the box marked 'follow-up' and faxing to the MRC CTU as information becomes available. Extra, annotated information and/or copies of test results may be provided separately. The patient must be identified by trial number, date of birth and initials only. The patient's name should not be used on any correspondence.

11.3 MRC CTU RESPONSIBILITIES

Medically qualified staff at the MRC CTU and/or the Chief Investigator (or a medically qualified delegate) will review all SAE reports received. The causality assessment given by the local Investigator at the hospital cannot be overruled and in the case of disagreement, both opinions will be provided in any subsequent reports.

The MRC CTU is undertaking the duties of trial sponsor and is responsible for the reporting of SUSARs and other SARs to the regulatory authorities (MHRA and competent authorities of other European member states and any other countries in which the trial is taking place) and the research ethics committees as appropriate.

The MRC CTU will also keep all investigators informed of any safety issues that arise during the course of the trial.

12 ETHICAL CONSIDERATIONS AND APPROVAL

12.1 ETHICAL CONSIDERATIONS

This is a randomised trial therefore neither the patients nor their physicians will be able to choose the patients' treatment. Treatment will be allocated randomly using a computer-based algorithm. This is to ensure that the groups of patients receiving each of the different treatments are as similar as possible.

All patients will receive standard treatment which will include ADT and may include radiotherapy and/or docetaxel. Patients may be randomised to one (or two; dependent on allocated arm) of the newer treatments in combination with standard-of-care treatment. The trial has employed an unequal allocation ratio for some comparisons to maximise efficiency; this was explained in detail in the patient information sheet.

The newer combined treatment options are being assessed in a detailed and systematic fashion in this trial. There is some evidence to suggest that the newer treatment options may have advantages over standard treatment alone with regards to clinical outcome, but this is not confirmed and toxicity may be increased. This trial will follow a large group of men who have been randomly allocated to either the standard treatment (androgen deprivation therapy alone) or the newer combined treatment options in order to measure the benefits of the new treatments. The patients will also be followed-up for toxicity and safety issues, so that any benefits can be weighed against any negative aspects.

Patients participating in the trial will have some additional hospital visits and some extra blood samples taken compared to patients who are not participating in the trial, with the amount varying according to the allocated treatment. Sometimes the blood samples can be taken when the patient is attending hospital for treatment, anyway. On some of the trial arms, the patient may have to make additional visits to the hospital for the blood sample to be taken, although in some cases it may be possible for the blood sample to be taken in the GP's surgery. The additional visits and blood samples are to ensure that follow-up of patients is comparable in all the treatment groups. The blood samples will also be used for genetic and serum marker studies, where this information will be considered with clinical data. Blood samples will be link-anonymised. There will be no feedback to individual patients.

If new information emerges during the course of the trial which may affect the treatment or follow-up of patients who have joined the trial, information will be provided through by the trial team to all Principal Investigators. PIs therefore have the duty to inform the patients in their care of any new information emerging using any appropriate channel (e.g. letter, communication at follow up clinic, etc).

The introduction of the "metformin comparison" means that all patients will be screened for diabetes prior to trial entry. This is to enable the effect of metformin to be studied in non-diabetic patients. All patients in whom screening bloods are abnormal will be referred for confirmatory tests and further management according to local guidelines e.g. via their GP. Screening is expected to lead to a small proportion of potential trial participants receiving a new diagnosis of diabetes but will ensure appropriate management of both conditions.

12.2 ETHICAL APPROVAL

The protocol has a Favourable Opinion from an appropriate Research Ethics Committee, according to national guidelines. Additionally, each site must also obtain management permission for research (R&D approval) from the relevant host organisations before patients can be entered into the trial. The patient's informed consent to participate in the trial should be obtained after a full explanation has been given of the treatment options, including the conventional and generally accepted methods of treatment. Patient information sheets and patient consent forms are available on the STAMPEDE website (www.stampedetrial.org).

The right of the patient to refuse to participate in the trial without giving reasons must be respected. After the patient has entered the trial, the clinician must remain free to give alternative treatment to that specified in the protocol, at any stage, if he feels it to be in the best interest of the patient. However, the reason for doing so should be recorded and the patient will remain within the trial for the purpose of follow-up and data analysis according to the treatment option to which he has been allocated. Similarly, the patient must remain free to withdraw at any time from the protocol treatment without giving reasons and without prejudicing his further treatment.

A statement of MRC policy on ethical considerations in clinical trials of cancer therapy, including the question of informed consent, is available from the MRC Head Office web site (http://www.mrc.ac.uk).

13 REGULATORY APPROVAL

This trial has been approved in the UK by the MHRA and will be conducted under a CTA (Ref: 00316/0026/001-0001) in the UK.

The trial has been approved in Switzerland by Swissmedic (Ref: 2009 DR 3235).

14 INDEMNITY

University College London holds insurance against claims from participants for injury caused by their participation in this clinical trial. Participants may be able to claim compensation if they can prove that UCL has been negligent. However, as this clinical trial is being carried out in a hospital, the hospital continues to have a duty of care to the participant of the clinical trial. University College London does not accept liability for any breach in the hospital's duty of care, or any negligence on the part of hospital employees. This applies whether the hospital is an NHS Trust or otherwise.

Participants may also be able to claim compensation for injury caused by participation in this clinical trial without the need to prove negligence on the part of University College London or another party. Participants who sustain injury and wish to make a claim for compensation should do so in writing in the first instance to the Chief Investigator, who will pass the claim to the managing organisation's Insurers, via the managing organisation's office.

Hospitals selected to participate in this clinical trial must provide clinical negligence insurance cover for harm caused by their employees and a copy of the relevant insurance policy or summary can be provided on request.

15 FINANCE

STAMPEDE is funded by the Clinical Trials Advisory Awards Committee (CTAAC) on behalf of Cancer Research UK; it is also funded by the MRC through the MRC Clinical Trials Unit. The trial has National Institute for Health Research Clinical Research Network (NIHR CRN) approval and, therefore, local NCRN funds may be available at each centre to support entry of patients into this trial.

Funding arrangements for research arms now closed to recruitment can be found in **Protocol version 13.0**

ADT will be administered as per routine clinical care using local NHS supplies.

M1|RT will be administered as per trial protocol using NHS RT equipment following successful RTQA by trial team.

Abiraterone is manufactured by Janssen Pharma PV (pharmaceutical companies of Johnson & Johnson). They have agreed to provide free drug and funds to distribute drug to participating sites and to help support the conduct and management of the trial.

Enzalutamide is manufactured by Astellas Pharma. They have agreed to provide free drug and funds to distribute drug to participating sites and to help support the conduct and management of the trial.

Metformin will be administered using local NHS supplies

16 TRIAL COMMITTEES

16.1 TRIAL MANAGEMENT GROUP (TMG)

A Trial Management Group (TMG) has been formed comprising the Chief Investigator, other co-investigators and members of the MRC CTU. The membership of the TMG may be expanded if other groups of trialists wish to participate. It will also be amended during the trial if other circumstances require e.g. retirement.

The TMG will be responsible for the day-to-day running and management of the trial. They will meet by teleconference at least 3-monthly and in person as needed. The TMG members are detailed in **Appendix M**.

Further details of TMG functioning are provided in the TMG charter (available on request).

16.2 TRIAL STEERING COMMITTEE (TSC)

A Trial Steering Committee (TSC) has been formed to provide overall supervision for the trial and provide advice through its independent chair. The ultimate decision for the continuation of the trial lies with the TSC. The TSC will meet regularly.

Further details of TSC functioning are provided in the TSC charter (available on request).

16.3 INDEPENDENT DATA MONITORING COMMITTEE (IDMC)

An Independent Data Monitoring Committee (IDMC) has been formed. The IDMC will be the only group who sees the confidential, accumulating data to the trial. Reports to the IDMC will be produced by the MRC CTU. The IDMC will meet within 6 months of the trial opening with the frequency of meetings dictated by the IDMC. The IDMC will consider data in accordance with the analysis plan (see **Section 9**) and will be advisory to the TSC. The IDMC can recommend premature closure or reporting of the trial, or that recruitment to any research arm is discontinued.

From Protocol version 8.0 onwards, any recommendation from the IDMC to stop recruitment to one or more trial arms will be acted upon immediately, pending ratification from the TSC. As this period between meetings should be very short, sites would not be notified until after the TSC have made a decision. IDMC recommendations based on emerging safety issues will be discussed with sites promptly.

Further details of IDMC functioning and the procedures for interim analysis and monitoring are provided in the IDMC charter (available on request).

16.4 TRIAL EXPERT PANELS

The trial has two established translational expert groups chaired byTMG members the Biological Research Group (BRG) and the Metabolic Translational Group (MTG). Both groups input and provide expert oversight of relevant translational aspects of the trial and associated sub-studies.

Figure 6: Diagram of relationships between trial committees

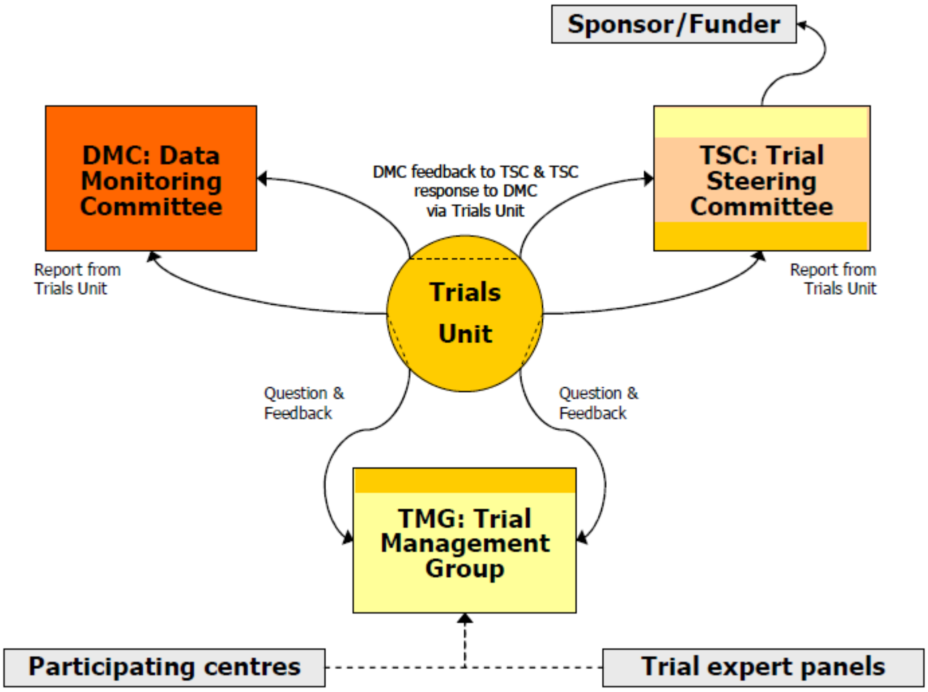

17 ANCILLARY STUDIES

17.1 QUALITY OF LIFE

A quality of life (QL) study is being performed to assess the impact of each treatment arm on the quality of patient's lives. Initial participation in this study was limited to the first 700 patients recruited (this was reached in Sep-2008) patients. The QL study re-opened from the implementation of version 8.0 of the protocol. The EORTC QLQ-C30 with the prostate-specific module QLQ PR25 will be used. Key items for assessment are pain reduction for patients with metastatic disease and urinary symptoms for patients with locally advanced disease. In addition specific hypotheses will be generated for each of the research arms. The EuroQol (EQ-5D) (49) will be used in the study as a generic measure of health-related quality of life which can be linked to public preferences. These data will be used to calculate quality-adjusted life-years as part of the economic evaluation (see **Section 17.2**). Patients recruited into the QL study, should continue on the study throughout the trial. Questionnaires should be self-administered, although it is recommended that a key person (e.g. research nurse) at each centre be responsible for the data collection to optimise compliance and completeness of the data.

The QL and the HE questionnaires should be completed without conferring with friends or relatives and all questions should be answered even if the patient feels them to be irrelevant.

The responsible person should check each questionnaire for its completeness, ensuring that the correct date of completion and patient identifiers are present. The research nurse should approach patients at appropriate clinical visits to complete a questionnaire. If no clinical visit is scheduled for the patient (with a window of 4 weeks around the expected date) the nurse should organise the completion of the questionnaire, by post or by a visit to the patient at home (or in a hospice).

17.2 HEALTH ECONOMICS

A health economics (HE) sub-study will be performed. Core resource use information will be collected, using CRFs on days in hospital (by speciality) and outpatient visits. Data collected on concomitant medication will also be used in the economic analysis. Information on patients' use of primary care and community-based services will be collected as additional questions in the QL questionnaire. Costs will be calculated on the basis of representative UK unit costs at the point of analysis. Health outcomes will be assessed in terms of quality-adjusted life years (QALYs). Quality adjustments will be based on patients' responses to the EQ-5D health status measure which will be administered at baseline and each point of follow-up as part of the QL questionnaire. A cost-effectiveness analysis will compare all regimens that continue to recruit into their final Efficacy Stage IV.

17.3 TRANSLATIONAL SUB-STUDIES

17.3.1 DNA ANALYSIS

Blood samples from as many patients as possible have been collected for future translational research. With patient consent, an additional droplet of blood sample has been collected using FTA Elute cards and stored for DNA and protein analysis in order to try to identify molecular features of clinical significance.

FTA Elute cards supplies have not been available since Dec-2013 and the STAMPEDE TMG has pursued an alternative method for genomic DNA collection using the Oragene® DNA kits for saliva sampling.

Oragene kits are widely used for collection of DNA from patients participating in clinical trials and they have been demonstrated to be a suitable alternative to DNA collection from whole blood providing a non-invasive, painless method of high quality sample collection.

A subset of patients may be asked if they would like to donate a blood sample for additional genetic research analysis.

Details of specimen collection, posting and contact details are given in **Appendix D**.

17.3.2 Analysis of tumour Tissue

All patients joining the trial have been asked to provide their consent for the use of remaining biological tissue obtained through prostate core biopsy or following prostate surgery (e.g. TURP). These samples are stored as FFPE samples at the site at which the procedure what performed or in storage respository. As the clinical outcome data is known analysis of archival tumour tissue is planned in order to address several translational research aims including the development of predictive biomarkers for the treatment evaluated within the trial. The trials's translational committee, the biological research group will act as an review and access committee for such trial sub-studies and where these are proposed by external collaborators this will require additional ethical approval. Randomising sites will be asked to facilitate and assist in sample retrieval and transfer to trial designated laboratories in support of this. Providing sufficient material remains following analysis, samples will be available for return to sites on request.

18 PUBLICATIONS

The results from different centres will be analysed together and published as soon as possible. Individual clinicians must not publish data concerning their patients that are directly relevant to questions posed by the study until the TMG has published its report. The TMG together with the STAMPEDE collaborators will form the basis of the writing committee and decide on the nature of publications. Any release, of efficacy or safety data, presentation or publication will be agreed with the TSC according to the terms of their charter.

All publications will acknowledge the participating centres and clinicians, and these will be detailed in an appendix to the main report. Papers will have named authors determined by the TMG according to the following principles:

- To be as inclusive as possible where this is practicable
- To ensure that there is justification for anyone to be named as an author
- Reasons for nomination for authorship may include: trial design; grant holding; day-to-day trial oversight (TMG membership); analysis; discussion and interpretation of data; representation for key groups; active participation at large recruiting sites. It should be accepted that the people qualifying for authorship will vary over time. In addition, key positions will vary depending on the nature of the publication: clinical lead for clinical papers, statistician lead for methodology papers, translational papers may be led by authors not on the main TMG if appropriate (e.g., the bone sub-study). In the event of any dispute related to authorship or data release, the TSC will be responsible for making the executive decision.

In the manuscript, a full list of sites and the number of patients recruited will be provided. In the presentations, this list of sites will also be shown. The term “the STAMPEDE investigators” will clearly be stated and relevant names included in the presentation credits.

A detailed Publication Plan is documented separately.

19 PROTOCOL AMENDMENTS

19.1 PROTOCOL

19.1.1 AMENDMENTS MADE TO SECTIONS IN PROTOCOL VERSION 1.0 (MAY 2004)

Administrative changes such as typos, word change etc.

Name additions/changes to:

TMG members

TSC members

IDMC members

'General Information' Section – additional information re. Abridged version of protocol

Section 1.2 – Figure 1, Celecoxib duration amended

Section 1.3 – Figure 2, addition of cardiovascular assessment form, name and timings amended

Section 2.3 – Docetaxel information updated

Section 2.4 – Additional text re dose and duration justification for Celecoxib use.

Section 3 – Title change and content updated

Section 4.2 – New exclusion criteria added

Section 4.3.1 – New investigations added and additional text re testosterone measurements and additional text re. prior celecoxib treatment

Section 6.1.4 – Celecoxib duration amended

Section 6.1.5 – Additional text re. Co-administration of docetaxel and bisphosphonates

Section 6.1.6 – Celecoxib duration amended

Section 6.2.2 – additional docetaxel information

Section 6.2.3 – addition of CV event history

Section 11 – Safety reporting updated

Section 12.1 – Additional text re. the collection of blood for genetic and serum marker studies

Section 15 – Additional information re. Central Subvention for docetaxel arms

19.1.2 AMENDMENTS MADE TO SECTIONS IN PROTOCOL VERSION 1.1 (MAY 2005)

Section 6.2 Administration and Dose Modifications, subsection 6.2.1 Zoledronic Acid

19.1.3 AMENDMENTS MADE TO SECTIONS IN PROTOCOL VERSION 2.0 (JUN 2005)

General Information section – SAE reporting fax number and timeframe added.

Section 1.2 – Addition of anti-androgen use for M0 patients as a method of HT

Section 1.2 – Increase in amount of blood needed & addition tissue sample request.

Section 1.3 Trial Documentation updated to include new table detailing trial documentation ahead of accreditation, the inclusion of the radiotherapy forms and correct case report form timings

Section 2.1 – Addition of anti-androgen use for M0 patients as a method of HT

Section 4.1.3 – Inclusion criteria Vii "Normal testosterone prior to hormone treatment" removed.

Section 4.1.3 - φnote has been omitted and moved to section 4.2 (see number 8)

Section 4.2 – Exclusion criteria added to exclude patients with active peptic ulceration, gastrointestinal bleeding and inflammatory bowel disease.

Section 4.2 – Exclusion Criteria added to exclude patients with planned major dental work

Section 4.3.1 - All blood test timelines changed from 14 days to 28 days.

Section 4.3.1 – Hormone Therapy pre-randomisation deadline extended from 4 weeks to 12 weeks.

Section 4.3.1 – Additional information regarding the use of NSAIDs and cox-2-inhibitors before coming on to the STAMPEDE study and once commenced on study treatment

Section 4.3.2 – Updated to ask for all vitamins and minerals the patient is taking to be recorded.

Section 4.3.3 – Updated to include the extra blood required and the request for consent of patients' tissue samples.

Section 6.1.1 – Addition of anti-androgen use for M0 patients as a method of HT

Section 6.1.6 – Addition of the calcium & vitamin name "calcichew".

Section 6.6.2 – asking also to collect vitamins and minerals under concomitant medication.

Section 6.6.3 – New section to inform investigators that patient's, who they wish to give radiotherapy to, are also eligible for STAMPEDE

Section 6.6.4 – New section to detail what data is being collected on the radiotherapy given to patients.

Section 7.1; figure 4 – Addition of radiotherapy form and in note, addition of AA alone

Section 7.1.2 – omission of repeated scans and x-rays at 24 weeks, also omitted in note under figure 4.

Chapter 11 – Safety reporting section updated

Section 17.3 – Increase in amount of blood needed & additional tissue sample request.

19.1.4 Amendments Made To Section In Protocol Version 3.0 (Jul 2006)

Front Cover - NCRN logo added for accuracy

Front Cover - Clarification that protocol developed with NCRI rather than on behalf of

Front Cover - Clarification that it is a 6 arm trial

General Information section - MRC CTU staff section updated

Section 1.2 – Statistics section updated.

Section 1.2 - Additional research paragraph updated to reflect additional studies and for clarification of terms

Section 1.2 - Blood collection volume changed to reflect new technique used

Section 1.3 (figure 3) - Table showing case report form schedule updated to reflect clarification of follow-up schedule and addition of new CRF (End of Treatment)

Section 2.2 - AS changed to HT (clarification of terms)

Section 2.3 - Updated in information in regard to use of docetaxel added to reflect up to date practice

Section 2.5 - Sub-headings numbered for consistency

Section 3.0 - Information in regard to the Pilot Phase now written in past tense as Pilot Phase has now been completed

Section 4.1.1 - Inclusion criteria extended so that patients who fulfil 2 out of the three of the first inclusion criteria can be eligible.

Section 4.3.1 - Change in time scales by which baseline investigations need to be completed.

Section 4.3.1 - Clarification that chest X-ray is only required if chest is not included in the CT

Section 4.3.1 - Removal of 12 week timeline for baseline PSA test to be performed. (Stipulation that it must be performed before start of HT)

Section 4.3.2 – Information added in regard to time allowed from randomisation to start of treatment

Section 4.3.3 - Additional research paragraph updated to reflect additional studies and for clarification of terms

Section 4.3.3 - Blood collection volume changed to reflect new technique used

Sections 6.1.2-6.1.6 - Androgen Suppression replaced with hormone therapy for consistency of terms

Section 6.2.2 - '(Taxotere)' Removed for consistency

Section 6.2.2 _ information added in regard to the need to closely monitor liver function prior to docetaxel administration

Section 7.1 - Page number reference updated

Section 7.1.1 - PSA measurement timings updated to accurately reflect follow-up schedule

Section 7.3 (Table 4) - Table and key updated to accurately reflect follow-up schedule and to include information about new CRFs and removal of withdrawal CRF

Section 8 - Rewording for clarification of definition of trial withdrawal

Section 8.1 - Instruction that withdrawal from trial treatment should be recorded on End of Treatment Form rather than withdrawal form

Section 8.1 - Information updated to emphasise that trial treatment must be discontinued following a progression

Section 8.2- Information added in regard to patient transfers

Section 8.3 - Instruction that withdrawal from trial completely must be notified in writing to the MRC CTU rather than included on withdrawal form

Section 9 and Summary – Target event numbers updated to reflect the slightly revised numbers obtained by using –nstage- which is the new, recommended program for MAMS trials

Sections 11.1 and 11.2 - Form numbers removed to allow for future changes in numbering

Section 11.2 – Reference to toxicity grading website added

Section 11.2.1 - Reference to table in appendix G added

Section 12.2 - 'Suggested' removed from 'Suggested patient information sheets'

Section 13 - CTA reference added

Section 17.3 - Information added to reflect new blood collection method for DNA analysis and in regard to additional translational studies for which funding has recently been approved

19.1.5 Amendments Made To Protocol Version 4.0 (Dec 2007)

General Information Section - Randomisation and SAE reporting details sections clarified

Section 1.2 and throughout protocol - Efficacy Stages 1-111 renamed to Activity Stages 1-111 for accuracy and clarity

Section 1.2 - Follow schedule corrected

Section 4.1.2 - Inclusion criteria widened to include high risk relapsing patients, that would not have met the previous PSA based criteria

Section 4.1.3 - Note added to reference location of WHO performance status definitions

Section 4.2 - Notes added to reference locations of toxicity gradings and NYHA classifications

Section 4.3.1 - Timings of baseline scan information changed to accurately reflect most common current practice

Section 6.1.1 - Information about use of LHRH antagonists to ensure that the protocol accurately reflects current and future practice

Section 6.1.1 - Information about suggested duration of hormone therapy added to ensure that the protocol accurately reflects current practice

Section 6.2.2 - Additional information added about the timing of liver function tests prior to docetaxel administration added for clarity

Section 6.6.4 - Information on radiotherapy data collection added

Section 7.1.1 - Erroneous information about the timing of PSA measurements removed

Figure 3 - Moved to new section in protocol for clarity and extended to include current information on data collection

Figure 3b - Added to describe how extent of data collection during follow-up should change, post treatment and post progression

Figure 4 - Notes added to explain the changes in data collected at follow-up and to information that the quality of life study will be applicable to the first 700 patients randomised only

Figure 4 - Note added to include palliative radiotherapy CRF

Section 11.3 - SAE reporting information updated

Section 19 - Protocol amendments list updated

19.1.6 Amendments Made To Protocol Version 5.0 (Aug 2008)

1. General Information Section – Randomisation phone line number updated – non UK extension added

2. Section 3 – Information about QL study removed to reflect closure of QL study after first 700 patients

3. Section 4.2 – Exclusion criteria clarified to explain that only patients with severe poor cardiovascular history should be excluded

4. Section 4.3.1 – Information on co-administration of NSAIDS with celecoxib changed based on clinical advice.

5. Section 5 - Randomisation phone line number updated – non UK extension added

6. Section 6.2.1. – Information added to clarify that patients who develop an osteonecrosis of the jaw should stop zoledronic acid treatment

7. Section 6.2.3 – 'severe' text added to accurately reflect which patients should be excluded based on their cardiovascular history

8. Section 7.1.2 – Definition of disease progression extended for clarity

9. Figure 3 – Updated to include reference to newly created skeletal related event form

10. Figure 4 – Previous error in table amended to show that the 4th Zoledronic Acid form that is submitted contains information about 3 cycles rather than 2 as previously indicated

11. Table 4 – 'Other important medical condition' added to definition of serious in the SAE section, to accurately reflect SAE form and current practice

12. Section 11.1 – Information added on reporting or pregnancies

13. Section 17 - Information about QL study removed to reflect closure of QL study after first 700 patients

19.1.7 Amendments Made To Protocol Version 6.0 (Jul 2009)

1. General Information Section – Trial Pharmacist removed and changes of:

Co-Investigator

Patient Representatives

Trial Manager

Data Manager

General Information Section - Coordinating Centre – address change

General Information Section – change of Sponsor address

Section 1.1 – ratio of patients randomised to the investigational arms updated

Section 1.2 – figure 1b added to clarify trial design from Apr-2011 onwards

Section 1.2 – paragraph added to explain trial changes after the second activity analysis

Section 1.2 – wording added to clarify that QL data only collected for first 700 patients randomised

Section 1.3 – SSA Favourable Opinion removed from list of trial documentation required ahead of site accreditation

Section 2.1 – Amount of men diagnosed with prostate cancer annually updated

Section 2.4 –note added to explain completion of recruitment to celecoxib- containing arms

Section 2.5.2 - note added to explain completion of recruitment to celecoxib- containing arms

Section 3 – SSA Favourable Opinion removed

Section 4.2 – Exclusion criterion xiii greyed out

Section 4.3.1 – paragraph removed regarding potential randomisation to celecoxib-containing arms

Section 5 – Randomisation instructions expanded to exclude public holidays or dates when notice has been given by the CTU

Section 6.1.4 – formatting changed to grey font to reflect recruitment completion for arm D

Section 6.1.6 - formatting changed to grey font to reflect recruitment completion for arm F

Section 6.2.3 – recruitment note added

Section 6.6.3 – radiotherapy statement changed to reflect data from recent trials

Section 7.1.2 – removal of reference to SRE- specific CRF

Section 7.3 – Figure 3 - Addition of Bone Density Risk Factor Form and BMD sub-study assessment forms to summary of timing table

Section 7.3 – Figure 4 – Weeks added to timings of assessments post 2 years

Section 7.3- Figure 4 – note added to explain recruitment completion for arms D and F

Section 12.1 – Wording changed to reflect change to randomisation allocation ratio

Section 12.1 – Addition of statement regarding new information emerging during the trial

Section 12.2 – Reference to SSA removed

Section 16.3 – Statement added regarding actioning IDMC recommendation ahead of TSC ratification

19.1.8 Amendments Made To Protocol Version 7.0 (Jul 2011)

1. General Information Section- SAE reporting fax number corrected

2. Section 11- SAE reporting fax number corrected

19.1.9 AMENDMENTS MADE TO PROTOCOL VERSION 7.1 (JUL 2011)

Throughout protocol – numbering has been updated in some sections new accommodate new information that has been added.

General Information Section – contact details updated

General Information Section – Funding information updated to include involvement from additional company

General Information Section – Wording on compliance and regulations updated to reflect current MRC CTU standard wording

General Information Section – Abbreviations list updated

Section 1.1 – The number of investigational agents being studied updated from three to four

Section 1.1 – Information regarding celecoxib updated to reflect that recruitment to these arms was discontinued in Apr-2011

Section 1.1 – Information about new IMP, Abiraterone inserted

Section 1.1 – Sample size and trial duration information updated to reflect changes brought about by additional trial arm

Section 1.2 – Summary information updated to reflect the discontinuation of recruitment to celecoxib arms and the addition of abiraterone

Figures 1a, b and c - Updated to reflect the discontinuation of recruitment to celecoxib arms and the addition of abiraterone

Section 1.2 – Information on trial stages updated to reflect changes brought about by additional trial arm

Section 1.2 – Information updated regarding the re-opening of the quality of life sub-study from implementation of protocol version 8.0

Section 2.1 – Wording related to hormone therapy updated for clarity

Section 2.1 - Updated to reflect the discontinuation of recruitment to celecoxib arms and the addition of abiraterone

Section 2.2 – Updated references added

Section 2.3 – Updated references added

Section 2.5 – Section added to give background information on new IMP, abiraterone

Section 2.6.1 – Updated references added

Section 2.7 – Section added to give information regarding radiotherapy which is to be given as part of standard care following recently published trial data.

Section 3 – Wording updated regarding selection of investigators to reflect current MRC CTU practice

Section 4.1 – Inclusion criteria updated with new criterion regarding radiotherapy use

Section 4.1 - Inclusion criteria updated with new criterion regarding contraceptive use

Section 4.1 – Wording of inclusion and exclusion criteria updated for clarity

Section 4.1 – Exclusion criteria updated with new criterion regarding acceptable liver function for trial entry

Section 4.1 – Exclusion criteria updated with specifics related to blood pressure levels

Section 4.1 - Exclusion criteria updated with new criterion regarding concomitant medications

Section 4.1 - Exclusion criteria updated with new criterion regarding prior treatment with abiraterone

Section 4.1 - Exclusion criteria updated with new criterion regarding prior treatment with chemotherapy

Section 4.1 - Exclusion criteria updated with new criterion regarding prior treatment with zoledronic acid

Section 4.3 – Wording updated to reflect that patients who initially fail screening can be re-screened at a later date

Section 4.3.2 – Wording updated regarding prior anti-androgen and LHRH use updated for clarity

Section 5.1 – Co-enrolment guidelines information updated to describe newly created co-enrolment CRF

Section 6.1 – Trial treatment information updated to reflect the fact that anti-androgens alone will be no longer permitted as hormone therapy

Section 6.1.1 – Updated to describe patients for whom radiotherapy should be given as standard practice

Section 6.1.1 a and b - Sections added to give information regarding radiotherapy treatment

Section 6.1.1-6.1.6 – References to further sections updated

Section 6.1.7 – Section added to describe abiraterone treatment

Section 6.2.4 - Section added to describe abiraterone treatment

Section 6.6 - Section added to give information regarding radiotherapy treatment

Section 7.1.1 – Reference to blood being taken at patient's home removed as this does not occur in practice

Section 7.1.2 – Wording updated regarding the reporting of biochemical failures for clarity

Section 7.1.2 – Wording updated regarding skeletal-related events for clarity

Section 7.1.3 – Section added to describe additional assessments required related to abiraterone treatment

Section 7.1.4 – Section added to provide information on when treatment should commence

Figure 4 – Updated for clarity regarding return of BMD sub-study forms, the addition the co-enrolment CRF and the description of the re-opening of the QoL Sub-study.

Figure 5 – Updated with reference to abiraterone and co-enrolment form

Section 7.3 - Wording on trial closure updated to reflect current MRC CTU standard wording

Section 8.1 – Additional criteria for definition of progression added for clarity

Section 8.1 – Definition of progression for abiraterone patients added.

Section 9 – Statistical information updated to describe the addition of the new trial arm

Section 11 – Safety reporting wording updated for clarity

Section 11 – SAE reporting fax number updated

Section 12 – Ethical information updated to describe the unequal randomisation allocation ratio

Section 12 – Ethical information updated to describe that the visit schedule will vary according to trial arm

Section 12.2 – Wording updated to reflect international participation in the trial

Section 13 – Wording updated to reflect international participation in the trial

Section 14 – Wording updated to reflect international participation in the trial

Section 15 - Updated to reflect the discontinuation of recruitment to celecoxib arms and the addition of abiraterone

Section 16 – Reference to trial committee charters added for information

Section 17.1 – Information added to reflect re-opening of quality of life sub-study

Section 17.2 – Timing of health economics analysis updated to previous error

Section 18 – Information on publication policy expanded for clarity

Section 19 – Information regarding amendments to protocol appendices moved to the separate appendices document

Section 20 – References extensively updated

19.1.10 Amendments Made To Protocol Version 8.0 (Sep 2011)

Throughout protocol – numbering, sections headings, tables, figures and bibliographical references have been updated in some sections to accommodate new information that has been added

Throughout protocol – Androgen Deprivation Therapy has replaced Hormone Therapy as deemed more representative of the type of hormone therapy used in the study

General Information Section – New staff members of the MRC CTU and Co-Investigators added and contact details updated

General Information Section – Abbreviations list updated

Section 1.1 – Information regarding the new research radiotherapy treatment inserted

Section 1.1 – Information regarding docetaxel updated

Section 1.2 – Wording updated to reflect the addition of the new research comparison arm

Section 1.3 – Additional criteria for the re-accreditation of participating centres (for protocol version 9.0 only)

Section 2.1.1 – Wording updated to clarify the use of anti-androgen in trial patients

Section 2.1.2 – Information added to describe the rationale for the RT comparison arm

Section 2.8 – Information added to describe research RT treatment to prostate for patients with newly diagnosed metastatic disease

Section 3.1 – Information added to describe RT Quality Assurance procedures and centre accreditation

Section 4.1.1 to 4.1.3 – Wording updated to clarify inclusion criteria for all patients groups (newly diagnosed non-metastatic, metastatic and relapsing patients)

Section 4.2 – Clarification added on cardiovascular exclusion criteria

Section 4.2 – New exclusion criterion added concerning patients with prior exposure to hormone therapy

Section 4.2 – New exclusion criterion added to reflect the addition of the new RT comparison arm

Section 4.4.1 – Clarification added regarding pre-randomisation checks

Section 4.4.2 – Clarification added regarding permissible hormone therapy duration prior to randomisation

Section 4.4.5 – Information added regarding starting research radiotherapy treatment

Section 4.4.6 – Information updated on concomitant medications

Section 5 – Clarification regarding randomisation allocation added to reflect the addition of the new RT research arm

Section 6.1.8 – Information added to describe the administration of research radiotherapy

Section 6.2.1 – Clarification added regarding the measurement of serum creatinine levels prior to the administration of zoledronic acid

Section 6.2.3 – Clarification regarding the completion of recruitment to the celecoxib containing arms

Section 6.25 – Information added regarding the administration of research radiotherapy treatment

Section 6.6 – Clarification incorporated to describe the administration of standard-of-care radiotherapy

Section 7.1.4 – Information added regarding data collection and non-administration of standard radiotherapy

Section 7.2 – Section updated to include new treatment specific CRFs and timing of CRFs

Section 8.1 – Clarification added for the criteria to stop treatment for patients randomised to arm G

Section 8.2 – Section expanded to include additional details on study patient transfer to different centres

Section 8.3 – Additional sentence inserted to reinforce the importance of compliance with follow up assessments

Section 9.1 – Additional paragraph inserted to clarify the method of randomisation and allocation distribution in the light of the introduction of the new RT arm

Section 9.4 – Wording updated to clarify the assessment of safety data

Section 9.5.4 – Wording updated concerning the end of randomisations to arm G

Section 9.6 to 9.6.4 – Section added describing sample size issues and trial stages for arm H

Section 9.8 – Clarification on intermediate stopping guidelines

Section 9.9 – Clarification on the outline analysis plan

Section 11 – Information on safety reporting updated to reflect the addition of the research RT comparison arm

Section 11 – Clarification added regarding arm A safety reporting timelines

Section 12.1 – Clarification added regarding the Principal Investigator's responsibilities

Section 14 – Indemnity section updated to reflect current MRC policy

Section 16 – Clarification regarding TMG membership

Section 17.3 – Section on Bone Mineral Density sub-study removed

Section 19 – Information regarding amendments to protocol appendices moved to the separate appendices document

Section 20 – References updated

19.1.11 Amendments Made To Protocol Version 9.0 (Oct-2012)

Throughout protocol – numbering, sections headings, tables, figures and bibliographical references have been updated in some sections to accommodate the completion of recruitment to original research arms B, C and E.

Throughout protocol – Tenses have been changed to reflect activities that were in the future and which have now been passed.

Section 1 – Figure added and clarifications added to each figure

Section 2 – Previous reference 8 removed

Section 4 – Clarification of acceptable alternatives to bone scans

Section 6.2.5 – Correction of an error defining the PTV: the wording has been reordered

Table 4 – Dose-volume objectives corrected: order swapped

Table 5- Correction CRFs names

Section 17.3.2 – Clarification that DNA may be extracted

19.1.12 Amendments Made To Protocol Version 10.0 (Apr-2013)

Throughout protocol – numbering, sections headings, tables, figures and bibliographical references have been updated in some sections

Throughout protocol – typos have been corrected

Section 4 –Clarification of exclusion criteria V (now V and VI)

Section 6 – Timing of orchidectomy prior to randomisation extended to 12 weeks

Section 6 – Clarification of hypokalaemia, blood pressure and fluid retention management

Section 9 – Statistical considerations amended in light of the recruitment extension for the abiraterone comparison

Section 14 - Section updated to reflect the changes in the structure of the MRC CTU (now MRC CTU at UCL) and indemnity arrangements

19.1.13 Amendments Made To Protocol Version 11.0 (Sep-2013)

Throughout protocol – numbering, sections headings, tables, figures and bibliographical references have been updated in some sections

Throughout protocol – typos have been corrected

Co-investigators list updated to reflect the addition of the “enzalutamide + abiraterone comparison” lead

Section 1.2 – Enzalutamide added as trial treatment

Section 1.2 – Protocol version 12.0 added to the list of amendments

Section 2.10 – Rationale for the combination of enzalutamide and abiraterone

Section 4.2 – Eligibility criteria amended to reflect the addition of enzalutamide + abiraterone arm

Section 4.4.2 – Wording clarified

Section 6.8 – Clarification regarding end of trial treatment after starting trial therapy

Section 6.10 – Section added to describe enzalutamide and abiraterone treatment for the new research arm (Arm J)

Section 6.11.4.A – Section added to describe the management of toxicities from trial abiraterone

Section 6.11.4.B - Section added to describe the management of toxicities from trial enzalutamide

Section 9.1.4 – Section added to describe the statistical considerations concerning the introduction of Arm J

Section 9.3 – Principles and assumption for the introduction of Arm J added

Section 9.7 and sub-sections – Sample size issues and trial stages for Arm J

Section 9.9 – Details on interim monitoring and analyses for Arm J added

Section 11.2.1.D – Wording clarified regarding safety reporting requirements for control arm

Section 12.1 – Wording clarified

Section 15 – Details on funding for the “enzalutamide + abiraterone comparison” added

Section 19 - Amendments made to protocol updated

Reference list updated

19.1.14 Amendments Made To Protocol Version 12.0 (Jan-2014)

Throughout protocol – typos have been corrected

Section 4.4.2. Wording clarified

Section 4.3. Wording clarified for eligibility to M1|RT comparison

Section 6.10. Addition of use of dexamethasone post-biochemical progression for Arm J patients

Section 6.11.4.A. Correction of CTCAE version

Section 6.11.4.C. Clarification on enzalutamide dose modification to be in line with current SmPC

Section 9.6. Sample size increase for M1|RT comparison

Section 11. Correction of safety reporting timelines for Arm A patients

Section 17. Addition of saliva samples collection for DNA analysis

Table 4, 5 and 6. Clarification on Case Report Forms and Follow-up schedule

19.1.15 Amendments Made To Protocol Version 13.0 (Feb-2014)

Throughout protocol – typos have been corrected

Throughout protocol – clarification on the new definition of standard-of-care

Table of contents updated to reflect any changes to the protocol

Section 1.1. Wording added throughout section to include reference to survival results from "original comparisons"

Section 2.1.1. Section improved to include reference to survival results from "original research comparisons"

Section 2.1.2. Section improved to include reference to survival results from "original research comparisons".

Section 2.1.3. Additional section added to describe the role of docetaxel for men with M0 or M1 disease

Section 2.9. Clarification on treatment completion and primary results for "original research comparisons"

Section 4.2. Clarification of Exclusion criteria XIII and XVI

Section 4.4.2. Clarification on HT prior to randomisation

Section4.4.3. New section to clarify standard-of-care docetaxel treatment prior to randomisation

Section 4.4.7. Clarification on concomitant medication and contra-indicated concomitant medications

Section 4.5. Clarification provided on tissue block collection

Section 6. Inclusion of docetaxel into the standard-of-care

Section 6.2.3 New section to describe standard-of-care docetaxel administration

Section 6.11. Improvement throughout sections and sub-sections for abiraterone and enzalutamide-related toxicity management

Section 6.12. Section improved throughout to incorporate clearer details on concomitant medications and drug-to-drug interactions

Section 7.1.4. New section to describe data collection for standard-of-care docetaxel

Section 9.7.4. Clarification provided about implications for "enzalutamide+ abiraterone comparison" following change of standard-of-care treatment

Section 11.2.1.D Clarification on SAE notification timelines to reflect change in standard-of-care treatments (addition of docetaxel)

Figure 1. Figure updated to reflect change in standard-of-care

Figure 2. Figure updated to reflect trial history and recruitment over time

Figure 3. Figure updated to reflect changes in standard-of-care and recruiting arms

Table 1. Table updated to remove repetition

Table 13. Table updated to include new CRF to report standard-of-care docetaxel treatment

Table 15. Table updated to include only active trial treatments

19.1.16 Amendments Made To Protocol Version 14.0 (Oct-2015)

Throughout protocol – typos have been corrected

Throughout protocol – clarification on the new definition of standard-of-care

Table of contents updated to reflect any changes to the protocol

Section 1. Wording added throughout section to include reference "metformin comparison"

Section 2. Section updated to include reference "metformin comparison"

Section 4.2. Exclusion criteria review to reflect Arm J closure and instruction of "metformin comparison"

Section 4.3. Clarification of comparison specific eligibility (M1|RT and metformin)

Section 4.5.7. Clarification on concomitant medication and contra-indicated concomitant medications

Section 6. Treatment sections improved throughout

Section 6.11. Section updated to include details on metformin treatment

Section 6.12. Amendment throughout sections and sub-sections for metformin treatment

Section 6.13. Amendment throughout sections and sub-sections for metformin treatment

Section 6.13. Improvement throughout sections and sub-sections for abiraterone and enzalutamide treatment

Section 7.0. Amendment throughout sections and sub-sections to include assessment and procedures specific to “metformin comparison”

Section 9.0. Section updated and streamlined to capture statistical considerations on each comparison

Section 9.0. Details on “metformin comparison” added

Section 11. Safety processes updated and clarified

Section 16.0 Membership to oversight groups updated

Section 11.2.1.D Clarification on SAE notification timelines to reflect change in standard-of-care treatments (addition of docetaxel)

20 REFERENCES

1. James ND, Sydes MR, Mason MD, Clarke NW, Anderson J, Dearnaley DP, et al. Celecoxib plus hormone therapy versus hormone therapy alone for hormone-sensitive prostate cancer: first results from the STAMPEDE multiarm, multistage, randomised controlled trial. The lancet oncology. 2012 May-2012;13(5):549-58. PubMed PMID: 22452894. Pubmed Central PMCID: 22452894. Epub 26-Mar-2012.
2. Cancer Research UK. CancerStats Key Facts: Prostate Cancer. Cancer Research UK. 2011.
3. Sharifi N, Gulley JL, Dahut WL, Sharifi N, Gulley JL, Dahut WL. An update on androgen deprivation therapy for prostate cancer. Endocrine-Related Cancer. 2010 2010;17(4):R305-15. PubMed PMID: 20861285.
4. Widmark A, Klepp O, Solberg A, Damber JE, Angelsen A, Fransson P, et al. Endocrine treatment, with or without radiotherapy, in locally advanced prostate cancer (SPCG-7/SFUO-3): an open randomised phase III trial. Lancet. 2009 Jan 24;373(9660):301-8. PubMed PMID: 19091394. Epub 2008/12/19. eng.
5. Warde PR, Mason MD, Sydes MR, Gospodarowicz MK, Swanson GP, Kirkbride P, et al. Intergroup randomized phase III study of androgen deprivation therapy (ADT) plus radiation therapy (RT) in locally advanced prostate cancer (CaP) (NCIC-CTG, SWOG, MRC-UK, INT: T94-0110; NCT00002633). J Clin Oncol. 2010;28(18s Supplement: Proceedings of ASCO 2010):Abstr CRA4504.
6. Warde P, Mason M, Ding K, Kirkbride P, Brundage M, Cowan R, et al. Survival Benefit with Combined Androgen Deprivation and Radiation Therapy in Locally Advanced Prostate Cancer – Results of a Phase III Trial. The Lancet. 2011 - in press.
7. Mason M, Sydes M, Parulekar W, Parmar M, Anderson J, Barber J, et al. Final analysis of intergroup randomized phase III study of androgen deprivation therapy (ADT) + radiation therapy (RT) in locally advanced prostate cancer (CaP) (NCIC-CTG, SWOG, MRC-UK, INT: T94-0110). National Cancer Research Institute (NCRI) Cancer Conference 2012. 2012;2012.
8. Warde P, Mason M, Ding K, Kirkbride P, Brundage M, Cowan R, et al. Combined androgen deprivation therapy and radiation therapy for locally advanced prostate cancer: a randomised, phase 3 trial. The Lancet. 2011;378:2104-11. PubMed PMID: 22056152. Epub 03-Nov-2011.
9. Nicholas D James MRS, Noel W Clarke, David P Dearnaley, Malcolm D Mason, Christopher C Parker, Alastair W S Ritchie, J. Martin Russell, Francesca Schiavone, Gerhardt Attard, Johann S de Bono, Alison Birtle,Daniel S Engeler,Tony Elliott, David Matheson, Joe O'Sullivan, Delia Pudney, Narayanan Srihari, Jan Wallace, Jim Barber, Isabel Syndikus, Mahesh K B Parmar, Matthew R Sydes. Failure-free survival and the impact of radiotherapy in patients with newly diagnosed non metastatic prostate cancer: Data from patients in the control arm of the STAMPEDE trial (MRC PR08, CRUK/06/019). JAMA Oncology. 2015.
10. Sweeney CJ, Chen YH, Carducci M, Liu G, Jarrard DF, Eisenberger M, et al. Chemohormonal Therapy in Metastatic Hormone-Sensitive Prostate Cancer. The New England journal of medicine. 2015 Aug 20;373(8):737-46. PubMed PMID: 26244877. Pubmed Central PMCID: 4562797.
11. Attard G, Richards J, de Bono JS, Attard G, Richards J, de Bono JS. New strategies in metastatic prostate cancer: targeting the androgen receptor signaling pathway. Clinical Cancer Research. 2011 Apr 1;17(7):1649-57. PubMed PMID: 21372223.
12. Fizazi K, Scher HI, Molina A, Logothetis CJ, Chi KN, Jones RJ, et al. Abiraterone acetate for treatment of metastatic castration-resistant prostate cancer: final overall survival analysis of the COU-AA-301 randomised, double-blind, placebo-controlled phase 3 study. The Lancet Oncology. 2012 Oct;13(10):983-92. PubMed PMID: 22995653.
13. Rathkopf DE, Smith MR, de Bono JS, Logothetis CJ, Shore ND, de Souza P, et al. Updated interim efficacy analysis and long-term safety of abiraterone acetate in metastatic castration-resistant prostate cancer patients without prior chemotherapy (COU-AA-302). European urology. 2014 Nov;66(5):815-25. PubMed PMID: 24647231. Pubmed Central PMCID: 4418928.

14. Flanigan RC, Salmon SE, Blumenstein BA, Bearman SI, Roy V, McGrath PC, et al. Nephrectomy followed by interferon alfa-2b compared with interferon alfa-2b alone for metastatic renal-cell cancer. N Engl J Med. 2001 Dec 6;345(23):1655-9. PubMed PMID: 11759643. Epub 2002/01/05. eng.
15. Mickisch GH, Garin A, van Poppel H, de Prijck L, Sylvester R. Radical nephrectomy plus interferon-alfa-based immunotherapy compared with interferon alfa alone in metastatic renal-cell carcinoma: a randomised trial. Lancet. 2001 Sep 22;358(9286):966-70. PubMed PMID: 11583750. Epub 2001/10/05. eng.
16. Attard G, Cooper CS, de Bono JS. Steroid hormone receptors in prostate cancer: a hard habit to break? Cancer cell. 2009 Dec 8;16(6):458-62. PubMed PMID: 19962664. Epub 2009/12/08. eng.
17. Chen G, Wang X, Zhang S, Lu Y, Sun Y, Zhang J, et al. Androgen receptor mutants detected in recurrent prostate cancer exhibit diverse functional characteristics. The Prostate. 2005 Jun 1;63(4):395-406. PubMed PMID: 15617028. Epub 2004/12/24. eng.
18. Taplin ME, Bubley GJ, Ko YJ, Small EJ, Upton M, Rajeshkumar B, et al. Selection for androgen receptor mutations in prostate cancers treated with androgen antagonist. Cancer Res. 1999 Jun 1;59(11):2511-5. PubMed PMID: 10363963. Epub 1999/06/11. eng.
19. Veldscholte J, Ris-Stalpers C, Kuiper GG, Jenster G, Berrevoets C, Claassen E, et al. A mutation in the ligand binding domain of the androgen receptor of human LNCaP cells affects steroid binding characteristics and response to anti-androgens. Biochemical and biophysical research communications. 1990 Dec 14;173(2):534-40. PubMed PMID: 2260966. Epub 1990/12/14. eng.
20. Zhao XY, Malloy PJ, Krishnan AV, Swami S, Navone NM, Peehl DM, et al. Glucocorticoids can promote androgen-independent growth of prostate cancer cells through a mutated androgen receptor. Nature medicine. 2000 Jun;6(6):703-6. PubMed PMID: 10835690. Epub 2000/06/03. eng.
21. Attard G, Reid AH, Auchus RJ, Hughes BA, Cassidy AM, Thompson E, et al. Clinical and biochemical consequences of CYP17A1 inhibition with abiraterone given with and without exogenous glucocorticoids in castrate men with advanced prostate cancer. The Journal of clinical endocrinology and metabolism. 2012 Feb;97(2):507-16. PubMed PMID: 22170708. Epub 2011/12/16. eng.
22. Richards J, Lim AC, Hay CW, Taylor AE, Wingate A, Nowakowska K, et al. Interactions of abiraterone, eplerenone, and prednisolone with wild-type and mutant androgen receptor: a rationale for increasing abiraterone exposure or combining with MDV3100. Cancer Res. 2012 May 1;72(9):2176-82. PubMed PMID: 22411952. Epub 2012/03/14. eng.
23. Scher HI, Fizazi K, Saad F, Taplin ME, Sternberg CN, Miller K, et al. Increased survival with enzalutamide in prostate cancer after chemotherapy. The New England journal of medicine. 2012 Sep 27;367(13):1187-97. PubMed PMID: 22894553.
24. Tombal B, Borre M, Rathenborg P, Werbrouck P, Heidenreich A, Iversen P, et al. Enzalutamide monotherapy: Phase II study results in patients with hormone-naive prostate cancer. Journal of Clinical Oncology. 2013;31(Supplement 6):Abstract 18.
25. Efstathiou E, Titus MA, Tsavachidou A, Hoang A, Karlou M, Wen S, et al. MDV3100 effects on androgen receptor (AR) signaling and bone marrow testosterone concentration modulation: A preliminary report. . 2011 ASCO Annual Meeting, 2011 J Clin Oncol 2011.
26. Locke JA, Guns ES, Lubik AA, Adomat HH, Hendy SC, Wood CA, et al. Androgen levels increase by intratumoral de novo steroidogenesis during progression of castration-resistant prostate cancer. Cancer Res. 2008 Aug 1;68(15):6407-15. PubMed PMID: 18676866. Epub 2008/08/05. eng.
27. Tran C, Ouk S, Clegg NJ, Chen Y, Watson PA, Arora V, et al. Development of a second-generation antiandrogen for treatment of advanced prostate cancer. Science. 2009 May 8;324(5928):787-90. PubMed PMID: 19359544. Pubmed Central PMCID: 2981508. Epub 2009/04/11. eng.
28. Scher HI, Beer TM, Higano CS, Anand A, Taplin ME, Efstathiou E, et al. Antitumour activity of MDV3100 in castration-resistant prostate cancer: a phase 1-2 study. Lancet. 2010 Apr 24;375(9724):1437-46. PubMed PMID: 20398925. Pubmed Central PMCID: 2948179. Epub 2010/04/20. eng.

29. Smith MR, Finkelstein JS, McGovern FJ, Zietman AL, Fallon MA, Schoenfeld DA, et al. Changes in body composition during androgen deprivation therapy for prostate cancer. The Journal of clinical endocrinology and metabolism. 2002 Feb;87(2):599-603. PubMed PMID: 11836291. Epub 2002/02/12. eng.
30. Eriksson A, Attvall S, Bonnier M, Eriksson JW, Rosander B, Karlsson FA. Short-term effects of metformin in type 2 diabetes. Diabetes, obesity & metabolism. 2007 Jul;9(4):483-9. PubMed PMID: 17587390. Epub 2007/06/26. eng.
31. Bailey CJ. Treating insulin resistance in type 2 diabetes with metformin and thiazolidinediones. Diabetes, obesity & metabolism. 2005 Nov;7(6):675-91. PubMed PMID: 16219011. Epub 2005/10/13. eng.
32. Ohira M, Miyashita Y, Ebisuno M, Saiki A, Endo K, Koide N, et al. Effect of metformin on serum lipoprotein lipase mass levels and LDL particle size in type 2 diabetes mellitus patients. Diabetes research and clinical practice. 2007 Oct;78(1):34-41. PubMed PMID: 17374417. Epub 2007/03/22. eng.
33. Wulffele MG, Kooy A, de Zeeuw D, Stehouwer CD, Gansevoort RT. The effect of metformin on blood pressure, plasma cholesterol and triglycerides in type 2 diabetes mellitus: a systematic review. Journal of internal medicine. 2004 Jul;256(1):1-14. PubMed PMID: 15189360. Epub 2004/06/11. eng.
34. Lubik AA, Gunter JH, Hendy SC, Locke JA, Adomat HH, Thompson V, et al. Insulin increases de novo steroidogenesis in prostate cancer cells. Cancer Res. 2011 Sep 1;71(17):5754-64. PubMed PMID: 21747118. Epub 2011/07/13. eng.
35. Venkateswaran V, Haddad AQ, Fleshner NE, Fan R, Sugar LM, Nam R, et al. Association of diet-induced hyperinsulinemia with accelerated growth of prostate cancer (LNCaP) xenografts. J Natl Cancer Inst. 2007 Dec 5;99(23):1793-800. PubMed PMID: 18042933. Epub 2007/11/29. eng.
36. Noto H, Goto A, Tsujimoto T, Noda M. Cancer risk in diabetic patients treated with metformin: a systematic review and meta-analysis. PloS one. 2012;7(3):e33411. PubMed PMID: 22448244. Pubmed Central PMCID: 3308971. Epub 2012/03/27. eng.
37. Rothermundt C, Hayoz S, Templeton AJ, Winterhalder R, Strebel RT, Bartschi D, et al. Metformin in chemotherapy-naive castration-resistant prostate cancer: a multicenter phase 2 trial (SAKK 08/09). European urology. 2014 Sep;66(3):468-74. PubMed PMID: 24412228. Epub 2014/01/15. eng.
38. James ND, Sydes MR, Clarke NW, Mason MD, Dearnaley DP, Spears MR, et al. Addition of docetaxel, zoledronic acid, or both to first-line long-term hormone therapy in prostate cancer (STAMPEDE): survival results from an adaptive, multiarm, multistage, platform randomised controlled trial. Lancet. 2015 Dec 21. PubMed PMID: 26719232. Epub 2016/01/01. Eng.
39. Vale CL, Burdett S, Rydzewska LH, Albiges L, Clarke NW, Fisher D, et al. Addition of docetaxel or bisphosphonates to standard of care in men with localised or metastatic, hormone-sensitive prostate cancer: a systematic review and meta-analyses of aggregate data. The lancet oncology. 2016 Feb;17(2):243-56. PubMed PMID: 26718929. Pubmed Central PMCID: 4737894. Epub 2016/01/01. eng.
40. de Bono JS, Logothetis CJ, Molina A, Fizazi K, North S, Chu L, et al. Abiraterone and increased survival in metastatic prostate cancer. New England Journal of Medicine. 2011 May 26;364(21):1995-2005. PubMed PMID: 21612468.
41. Salpeter SR, Greyber E, Pasternak GA, Salpeter EE. Risk of fatal and nonfatal lactic acidosis with metformin use in type 2 diabetes mellitus: systematic review and meta-analysis. Archives of internal medicine. 2003 Nov 24;163(21):2594-602. PubMed PMID: 14638559. Epub 2003/11/26. eng.
42. Kouloulias V, Giraud J-Y, Davis BJ, Dusserre A, Zurlo A, Bolla M. Quality assurance of the 22961 EORTC trial. A phase III study of the optimal combination of hormonal adjuvant treatment by LHRH analogue and radiation therapy for the management of locally advanced prostate cancer: the dummy run. 2004 20041006 DCOM- 20050225(0167-8140 (Print)). eng.
43. Royston P, Parmar MKB, Qian W. Novel designs for multi-arm clinical trials with survival outcomes with an application in ovarian cancer. Statistics in Medicine. 2003;22(14):2239-56.

44. Royston P, Barthel FMS, Parmar MKB, Choodari-Oskooei B, Isham V. Designs for clinical trials with time-to-event outcomes based on stopping guidelines for lack of benefit. Trials. 2011;12(1):81.
45. Royston P. nstage: MAMS trial sample size calculator. MRC Clinical Trials Unit, London2009.
46. Haybittle JL. Repeated assessment of results in clinical trials of cancer treatment. The British journal of radiology. 1971 Oct;44(526):793-7. PubMed PMID: 4940475. Epub 1971/10/01. eng.
47. Peto R, Pike MC, Armitage P, Breslow NE, Cox DR, Howard SV, et al. Design and analysis of randomized clinical trials requiring prolonged observation of each patient. I. Introduction and design. British journal of cancer. 1976 Dec;34(6):585-612. PubMed PMID: 795448. Pubmed Central PMCID: 2025229. Epub 1976/12/01. eng.
48. Schweizer MT, Huang P, Kattan MW, Kibel AS, de Wit R, Sternberg CN, et al. Adjuvant leuprolide with or without docetaxel in patients with high-risk prostate cancer after radical prostatectomy (TAX-3501): important lessons for future trials. Cancer. 2013 Oct 15;119(20):3610-8. PubMed PMID: 23943299. Pubmed Central PMCID: 4124610. Epub 2013/08/15. eng.
49. Dolan P, Gudex C, Kind P, Williams A. Discussion Paper 138: A social tarif for EuroQol: results from a UK General Population Survey. . Centre for Health Economics. University of York, 1995.

STAMPEDE

Systemic Therapy in Advancing or Metastatic Prostate Cancer: Evaluation of Drug Efficacy

A multi-arm multi-stage randomised controlled trial

**Version:**	**16.0**
**Date:**	**02-Mar-2017**

MRC CTU AT UCL ID:	PR08
ISRCTN #:	ISRCTN78818544
NCT #:	NCT00268476
EUDRACT #:	2004-000193-31
CTA #:	00316/0026/001-0001
MREC #:	04/MRE07/35

Authorised by:

Name:	PROFESSOR NICHOLAS D JAMES
Role:	CHIEF INVESTIGATOR
Signature:	

Name:	MATTHEW SYDES
Role:	TRIAL STATISTICIAN

GENERAL INFORMATION

This document was constructed using the MRC CTU at UCL Protocol Template Version 4.0. It describes the STAMPEDE trial, coordinated by the Medical Research Council (MRC) Clinical Trials Unit (CTU) at University College London (UCL), and provides information about procedures for entering patients into it. The protocol should not be used as an aide-memoire or guide for the treatment of other patients. Every care has been taken in drafting this protocol, but corrections or amendments may be necessary. These will be circulated to the registered investigators in the trial, but sites entering patients for the first time are advised to contact the Cancer And Other Non-Infectious Diseases Group, MRC CTU at UCL, London, UK, to confirm they have the most up-to-date version.

COMPLIANCE

The trial will be conducted in compliance with the approved protocol, the Declaration of Helsinki 1996, the principles of Good Clinical Practice (GCP), Commission Directive 2005/28/EC with the implementation in national legislation in the UK by Statutory Instrument 2004/1031 and subsequent amendments, the UK Data Protection Act (DPA number: Z5886415), and the National Health Service (NHS) Research Governance Framework for Health and Social Care (RGF). International sites will comply with the principles of GCP as laid down by the ICH topic E6 (Note for Guidance on GCP), Commission Directive 2005/28/EC (the European Directive 2001/20/EC [where applicable]) and applicable national regulations.

SPONSOR

Medical Research Council, 2nd Floor, David Phillips Building, Polaris House, North Star Avenue, Swindon, SN2 1FL, UK.

On 01-Aug-2013, the MRC CTU became part of University College London (UCL). The MRC maintains sponsorship for the trial however UCL is the legal entity responsible for the running of the trial. This responsibility is delegated to the coordinating trial unit, the MRC CTU at UCL.

FUNDING

Cancer Research UK's Clinical Research Committee (formerly the Clinical Trials Advisory Awards Committee), Medical Research Council, and educational grants from Novartis, Sanofi-Aventis, Pfizer, Janssen Pharma NV, Astellas, Clovis Oncology.

AUTHORISATIONS AND APPROVALS

The following persons are authorised to sign the final protocol and protocol amendments for the sponsor: Professor Nicholas James (Chief Investigator) and Matthew Sydes (Trial Statistician).

TRIAL REGISTRATION

This trial has been registered with the ClinicalTrials.gov Clinical Trials Register, where it is identified as NCT00268476.

REGISTRATION AND RANDOMISATION
Call MRC CTU at UCL, Monday to Friday 0900-1700
Excluding public holidays or dates when notice has been given by the Unit.
Tel: +44 (0) 20 7670 4777

SAE REPORTING
Fax to 020 7670 4818 within 24 hours of becoming aware of the event
Or send via **encrypted** email to mrcctu.stampede@ucl.ac.uk

TRIAL ADMINISTRATION

COORDINATING SITE

MRC Clinical Trials Unit at UCL
Aviation House
125 Kingsway
London WC2B 6NH
UK

Switchboard: 020 7670 4700
Fax: 020 7670 4818
Email: mrcctu.stampede@ucl.ac.uk
Website: http://www.stampedetrial.org/

MRC CTU AT UCL STAFF

Trial Manager:	Mazna Anjum	Tel:	0207 670 4772
Trial Manager:	Anna Herasimtschuk	Tel:	0207 670 4882
Trial Manager:	Claire Murphy	Tel:	0207 670 4620
Trial Manager	Orla Prendiville	Tel:	0207 670 4840
Trial Manager	Katie Ward	Tel:	0207 670 4916
Data Manager:	Carly Au	Tel:	0207 670 4794
Data Manager:	Lina Bergstrom	Tel:	0207 670 4693
Data Manager:	Danielle Johnson	Tel:	0207 670 4604
Data Manager:	Saba Khan	Tel:	020 670 4865
Data Manager:	Tasmin Philips	Tel:	0207 670 4947
Data Manager:	Peter Vaughan	Tel:	0207 670 4655
Clinical Project Manager:	Claire Amos	Tel:	0207 670 4771
Clinical Project Manager:	Nafisah Atako	Tel:	0207 670 4896
Statistician:	Chris Brawley	Tel:	0207 670 4677
Statistician:	Melissa Spears	Tel:	0207 670 4764
Senior Trial Statistician:	Matthew Sydes	Tel:	0207 670 4798
Transdermal Oestradiol Lead:	Duncan Gilbert	Tel:	Please contact STAMPEDE team
Clinical Research Fellow	Clare Gilson	Tel:	Please contact STAMPEDE team
Director, MRC CTU at UCL:	Max Parmar	Tel:	Please contact STAMPEDE team

CHIEF INVESTIGATOR

Prof Nicholas James

Clinical Oncology, The Medical School
University of Birmingham
Birmingham, B15 2TJ
UK

Tel: 0121 371 3615 (Secretary)

Email: n.d.james@bham.ac.uk

CO-INVESTIGATORS AND TMG MEMBERS

Prof Paul Abel<br>Imperial College	London, UK	Co-CCI "Transdermal oestradiol comparison"
Dr Gerhardt Attard<br>Oncologist	London, UK	CCI "Enzalutamide+Abiraterone comparison"
Dr Simon Chowdhury<br>Oncologist	London, UK	
Prof Noel Clarke<br>Urologist	Manchester, UK	TMG Vice-Chair<br>Co-CCI "Metformin comparison"
Mr William Cross<br>Urologist	Leeds, UK	
Prof David Dearnaley<br>Oncologist	London, UK	
Prof Silke Gillessen<br>Oncologist	St Gallen, Switzerland	Co-CCI "Metformin comparison"
Prof Rob Jones<br>Oncologist	Glasgow, UK	
Dr Zafar Malik<br>Oncologist	Liverpool, UK	
Prof Malcolm Mason<br>Oncologist	Cardiff, UK	TMG Vice-Chair
Prof Ruth Langley<br>Oncologist	London & Brighton, UK	Co-CCI "Transdermal oestradiol comparison"
Dr Chris Parker<br>Oncologist	London, UK	CCI "M1 RT comparison"
Mr Alastair Ritchie<br>Surgeon	Edinburgh, UK	
Dr Martin Russell<br>Oncologist	Glasgow, UK	
Prof George Thalmann<br>Urologist	Bern, Switzerland	
Robin Millman<br>Patient representative	UK	
David Matheson,<br>Patient representative	UK	
Prof Mark Sculpher<br>Health Economics Advisor	York, UK	
Dr Amanda Adler<br>Diabetologist	Cambridge, UK	Diabetologist "Metformin comparison"

SAAK Coordinating Centre

Corinne Schär	Bern, Switzerland

Trial Expert Groups

Biological Research Group	Prof Malcolm Mason (Chair)
	Prof David Waugh (Co-Chair)
Metabolic Translational Group	Prof Noel Clarke (Chair)
Stratified Science Panel	Prof David Waugh (Chair)
	Dr Gert Attard (Co-Chair)

SUMMARY OF TRIAL

SUMMARY INFORMATION TYPE	SUMMARY DETAILS
Acronym	STAMPEDE
Long Title of Trial	Systemic Therapy in Advancing or Metastatic Prostate Cancer: Evaluation of Drug Efficacy: A multi-arm multi-stage randomised controlled trial
Version	16.0
Date	02-Mar-2017
MRC CTU at UCL ID	PR08
NCT #	NCT00268476
EudraCT #	2004-000193-31
Study Design	Multi-arm multi-stage platform randomised controlled trial
Type of Participants to be Studied	Men starting long-term hormone therapy for metastatic or high-risk non-metastatic prostate cancer
Setting	Tertiary care
Interventions to be Compared	Various - see comparison-specific tables
Study Hypothesis	Various - see comparison-specific tables
Definitive Primary Outcome Measure	Overall survival (unless stated)
Intermediate Primary Outcome Measure	Failure-free survival (unless stated)
Secondary Outcome Measure(s)	Toxicity<br>Symptomatic skeletal events<br>Quality-of-life<br>Cost-effectiveness
Randomisation	Minimisation using a random element across a number of stratification factors
Number of Participants	See comparison-specific tables
Duration	See comparison-specific tables
Sponsor	Medical Research Council
Funders	Cancer Research UK<br>Medical Research Council<br>Astellas<br>Clovis Oncology<br>Janssen<br>Novartis<br>Pfizer<br>Sanofi-Aventis

SUMMARY INFORMATION TYPE	SUMMARY DETAILS
**“Original comparisons”**	
Type of Participants to be Studied	Men starting long-term hormone therapy for metastatic or high-risk non-metastatic prostate cancer
Control Arm	Arm A: Standard-of-care (SOC)
Interventions to be Compared	Arm B: SOC + zoledronic acid<br>Arm C: SOC + docetaxel<br>Arm D: SOC + celecoxib<br>Arm E: SOC + zoledronic acid + docetaxel<br>Arm F: SOC + zoledronic acid + celecoxib
Allocation ratio	2 control arm : 1 research arm [2A:1B:1C:1D:1E:1F]
Study Hypothesis	Research interventions will improve survival over SOC
Definitive Primary Outcome Measure	Overall survival
Intermediate Primary Outcome Measure	Failure-free survival
Number of Participants	Sufficient for 400 control arm definitive primary outcome measure events (in practice, >3000 patients)
Duration	10 years
Status	Primary results published (1)
**“Abiraterone comparison”**	
Type of Participants to be Studied	Men starting long-term hormone therapy for metastatic or high-risk non-metastatic prostate cancer
Control arm	Arm A: Standard-of-care (SOC)
Intervention to be Compared	Arm G: SOC + abiraterone
Allocation ratio	1 control arm : 1 research arm [1A:1G]
Study Hypothesis	Addition of abiraterone to SOC will improve survival over SOC alone
Definitive Primary Outcome Measure	Overall survival
Intermediate Primary Outcome Measure	Failure-free survival
Number of Participants	Around 1,800 patients for 267 control arm definitive primary outcome measure events
Duration	6 to 8 years
Status	In follow-up
**“M1I RT comparison”**	
Type of Participants to be Studied	Men starting long-term hormone therapy for newly-diagnosed metastatic prostate cancer with no contraindication to prostate radiotherapy
Control arm	Arm A: Standard-of-care (SOC)
Intervention to be Compared	Arm H: SOC + radiotherapy to the prostate (RT)
Allocation ratio	1 control arm : 1 research arm [1A:1H]

SUMMARY INFORMATION TYPE	SUMMARY DETAILS
Study Hypothesis	Addition of RT to SOC will improve survival over SOC alone
Definitive Primary Outcome Measure	Overall survival
Intermediate Primary Outcome Measure	Failure-free survival
Number of Participants	Around 1,800 patients for 267 control arm definitive primary outcome measure events
Duration	6 to 8 years
Status	In follow-up
**"Enzalutamide + abiraterone comparison"**	
Type of Participants	Men starting long-term hormone therapy for metastatic or high-risk non-metastatic prostate cancer
Control Arm	Arm A: Standard-of-care (SOC)
Interventions to be Compared	Arm J: SOC + enzalutamide + abiraterone
Allocation ratio	1 control arm : 1 research arm [1A:1J]
Study Hypothesis	Addition of enzalutamide, in combination with abiraterone, to SOC will improve survival over SOC alone
Definitive Primary Outcome Measure	Overall survival
Intermediate Primary Outcome Measure	Failure-free survival
Number of Participants	Around 1,800 patients for 267 control arm definitive primary outcome measure events
Duration	6 to 8 years
Status	In follow-up
**"Metformin comparison"**	
Type of Participants to be Studied	Non-diabetic men, with no contraindication to metformin, starting long-term hormone therapy for metastatic or high-risk non-metastatic prostate cancer
Control arm	Arm A: Standard-of-care (SOC)
Intervention to be Compared	Arm K: SOC + metformin
Allocation ratio	1 control arm : 1 research arm [1A:1K]
Study Hypothesis	Addition of metformin to SOC will improve survival over SOC alone
Definitive Primary Outcome Measure	Overall survival
Intermediate Primary Outcome Measure	Overall survival
Number of Participants	Around 1,800 patients, including around 1,100 M1 (metastatic) patients, for 374 control arm definitive primary outcome measure events among M1 patients

SUMMARY INFORMATION TYPE	SUMMARY DETAILS
Duration	10 years
Status	Recruiting
**“Transdermal oestradiol comparison”**	
Type of Participants to be Studied	Men starting long-term hormone therapy for metastatic or high-risk non-metastatic prostate cancer, having had no more than one 4-week (or one-month) LHRH injection & 8 weeks of AAs
Control arm	Arm A: Standard-of-care (SOC)
Intervention to be Compared	Arm L: Transdermal oestradiol ± RT ± docetaxel
Allocation ratio	1 control arm : 1 research arm [1A:1L]
Study Hypothesis	Transdermal oestradiol will be non-inferior to standard hormone therapy, while having fewer side-effects and improved quality-of-life
Definitive Primary Outcome Measures	Co-primary endpoints of Progression-free survival & Overall survival
Intermediate Primary Outcome Measure	Progression-free survival
Number of Participants	Around 500 to include within a meta-analysis with the PATCH trial, which will include around 2,000 patients overall
Duration	4 to 6 years
Status	Recruiting

Figure 1: Arms of the STAMPEDE trial from Protocol version 16.0

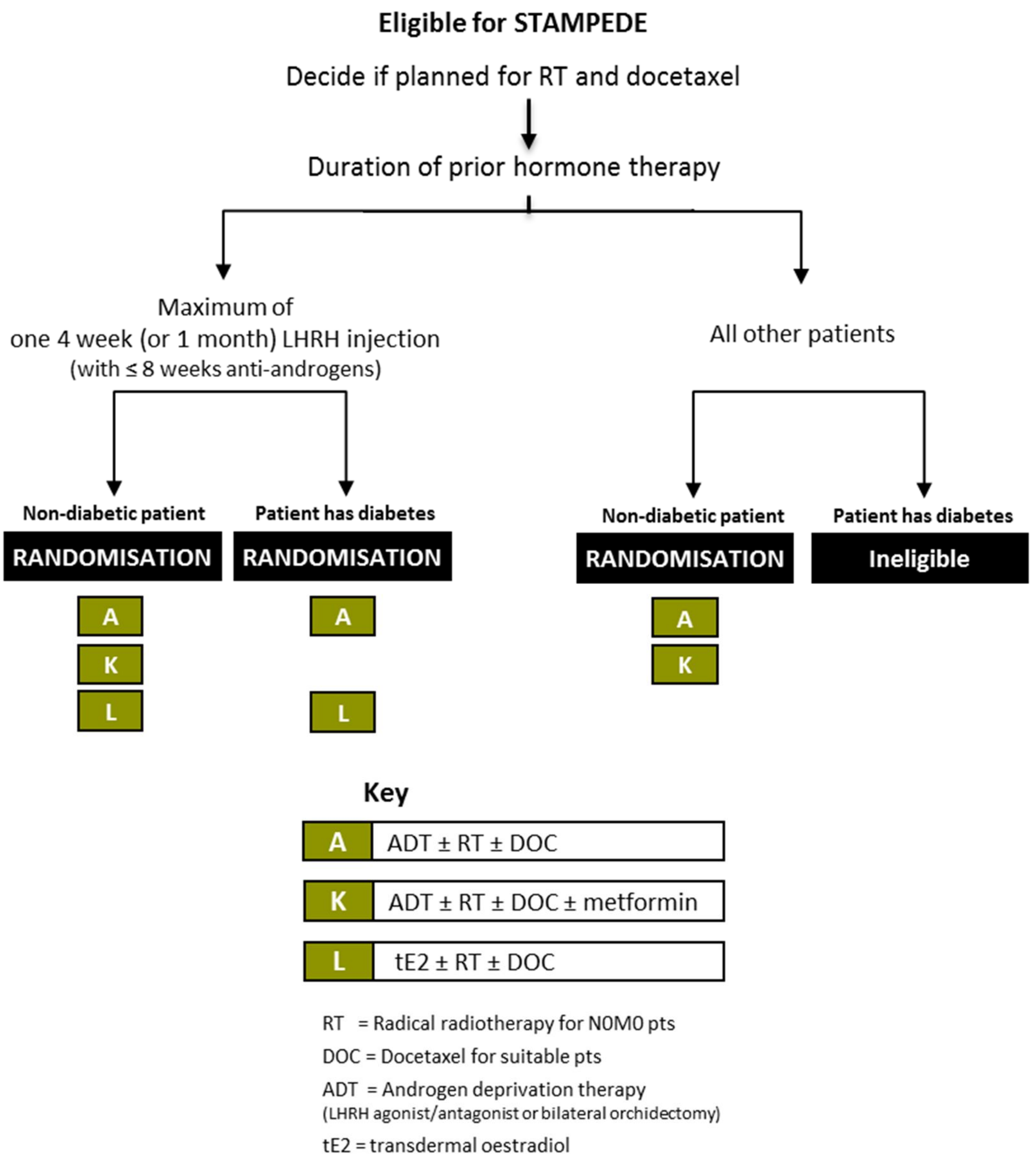

Figure 2: Arms of the STAMPEDE trial open to recruitment over time

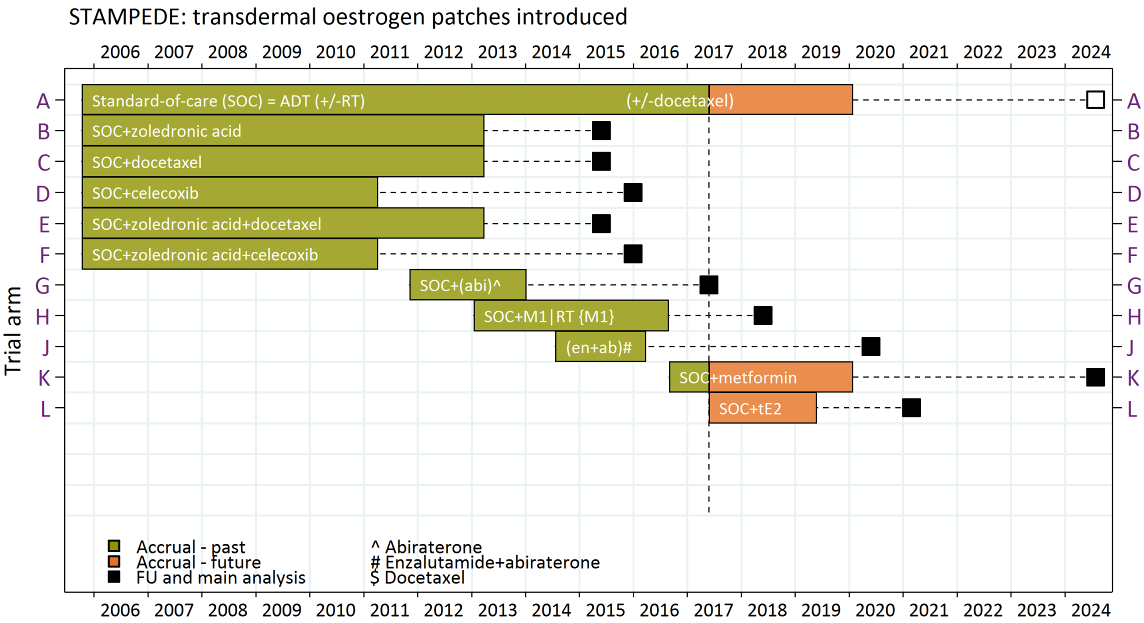

Note: dotted line represents activation of this protocol version

CONTENTS

GENERAL INFORMATION 2

SUMMARY OF TRIAL 7

CONTENTS 13

ABBREVIATIONS & GLOSSARY 18

1 LAY SUMMARY 22

2 BACKGROUND 25
2.1 INTRODUCTION AND SETTING 25
2.1.1 Long-term Androgen Deprivation Therapy 25
2.1.2 Role Of Radiotherapy For Men With M0 Disease 25
2.1.3 Role Of Docetaxel For Men With M0 Or M1 Disease 26
2.2 RATIONALE 26
2.3 DESIGN 26
2.4 PREVIOUSLY REPORTED RESEARCH TREATMENTS 27
2.5 RATIONALE FOR RESEARCH TREATMENTS UNDER EVALUATION 27
2.5.1 Steroid Synthesis Inhibitors 27
2.5.2 Radiotherapy To The Prostate For Patients With Newly-Diagnosed Metastatic Disease 28
2.5.3 Combination Of Steroid Synthesis Inhibitors And Androgen Receptor Signalling Inhibitor 28
2.5.4 Metformin 29
2.5.5 Transdermal Oestradiol 30

3 SELECTION OF INSTITUTIONS AND INVESTIGATORS 33
3.1 COMPARISON-SPECIFIC SITE ACCREDITATION 33
3.1.1 "Transdermal Oestradiol Comparison" 33
3.2 FUTURE PLANNED BIOMARKER-SELECTED COMPARISONS 33
3.3 REQUIRED TRIAL DOCUMENTATION 34

4 SELECTION OF PATIENTS 35
4.1 GENERAL INCLUSION CRITERIA 35
4.1.1 High-Risk Newly-Diagnosed Non-Metastatic Node-Negative Disease 35
4.1.2 Newly-Diagnosed Metastatic Or Node-Positive Disease 35
4.1.3 Previously Radically Treated, Now Relapsing (Prior Radical Surgery And/Or Radiotherapy) 35
4.1.4 For All Patients 35
4.2 GENERAL EXCLUSION CRITERIA 36
4.3 BIOMARKER-SCREENING PILOT 37
4.3.1 Selection Criteria For Patient Registration 37
4.4 COMPARISON-SPECIFIC SELECTION CRITERIA 37
4.4.1 For Randomisation To The "Metformin Comparison" 37
4.4.2 For Randomisation To The "Transdermal Oestradiol Comparison" 38
4.5 SCREENING PROCEDURES 39
4.5.1 Investigation Prior to Registration (for patients participating in biomarker-screening pilot) 39
4.5.2 Investigations Prior To Randomisation 39
4.5.3 Additional Baseline Investigations 40

4.5.4 Androgen Deprivation Therapy Prior To Randomisation 40
4.5.5 Standard-Of-Care (SOC) Radiotherapy 41
4.5.6 Standard-Of-Care (SOC) Docetaxel 42
4.5.7 Starting Trial Treatment 42
4.5.8 Concomitant Medications 42
4.6 ADDITIONAL DETAILS FOR PATIENTS JOINING SUB-STUDIES 43
4.6.1 Germline DNA Analysis (Saliva Samples) 43
4.6.2 Circulating Tumour-DNA Analysis (Sequential Blood Samples) 43
4.6.3 Tissue Sample Analysis (FFPE Blocks) 44
4.6.4 Biomarker-screening pilot 44
4.6.5 Informed Consent For Genetic Screening 45

5 REGISTRATION AND RANDOMISATION 46
5.1 TRIAL ENROLMENT: DEFINITIONS AND PROCESS 46
5.1.1 Registration 46
5.1.2 Randomisation 46
5.2 CO-ENROLMENT GUIDELINES 47

6 TREATMENT OF PATIENTS 48
6.1 STANDARD-OF-CARE (SOC) 48
6.1.1 Hormone Therapy 48
6.1.2 Standard-Of-Care (M0) RT 49
6.1.3 Standard-Of-Care Docetaxel 49
6.2 RESEARCH TREATMENTS 50
6.2.1 Required Timelines When Starting Research Treatment 50
6.2.2 Research Abiraterone + Prednisolone (arm G) 50
6.2.3 Abiraterone + Prednisolone: Administration, Dose Modification And Management Of Toxicities 51
6.2.4 Research Enzalutamide + Abiraterone + Prednisolone (Arm J) 55
6.2.5 Enzalutamide: Administration, Dose Modification And Management Of Toxicities 56
6.2.6 Research Metformin (Arm K) 57
6.2.7 Metformin: Administration, Dose Modifications And Management Of Toxicities 58
6.2.8 Research Transdermal Oestradiol (Arm L) 61
6.2.9 Transdermal Oestradiol: Administration, Dose Modifications And Management Of Toxicities 62
6.3 CONCOMITANT MEDICATIONS AND DRUG INTERACTION 62
6.3.1 Abiraterone: Interaction With Medicinal Products And Other Forms Of Interaction 62
6.3.2 Enzalutamide: Interaction With Medicinal Products And Other Forms Of Interaction 62
6.3.3 Metformin: Interaction With Medicinal Products And Other Forms Of Interaction 65
6.3.4 Transdermal Oestradiol: Drug Interactions 65
6.4 TRIAL PRODUCTS 66
6.5 MEASURES OF COMPLIANCE/ADHERENCE 66
6.6 TREATMENT DATA COLLECTION 66
6.7 ADMINISTRATION OF STANDARD RADIOTHERAPY TO M0 PATIENTS 66
6.7.1 Treatment Details 66

7 ASSESSMENTS AND PROCEDURES 67
7.1 SCHEDULE FOR ASSESSMENTS 67
7.1.1 Follow-Up Schedules 67
7.1.2 PSA, Testosterone And Oestradiol Measurements 67
7.1.3 Assessment Of Treatment Failure (Definition Of Progression) 69
7.1.4 Additional Metabolic And Cardiovascular Outcomes 70

7.1.5 Additional Safety Assessment 73
7.2 DATA COLLECTION PROCEDURES 73
7.2.1 Data Collection For SOC Hormone Therapy 73
7.2.2 Data Collection For Standard Docetaxel 74
7.2.3 Data Collection And Non-Administration Of Standard Radiotherapy 74
7.2.4 Data Collection For Palliative Radiotherapy 74
7.2.5 Data Collection for Research (M1) Radiotherapy 74
7.2.6 Data Collection for Additional Treatments Given for Disease Progression 75
7.3 FOLLOW-UP PROCEDURE 75
7.3.1 Follow-Up Telephone Consultations 75
7.4 TRIAL CLOSURE 76

8 STOPPING OF TREATMENT OR FOLLOW-UP 80
8.1 STOPPING RESEARCH INTERVENTIONS 80
8.1.1 Stopping Trial Treatment: Abiraterone, Enzalutamide + Abiraterone 80
8.1.2 Stopping Trial Treatment: Metformin 80
8.1.3 Stopping Trial Treatment: Transdermal Oestradiol 80
8.2 PATIENT TRANSFERS 81
8.3 EARLY CESSATION OF TRIAL PARTICIPATION 81

9 STATISTICAL CONSIDERATIONS 83
9.1 METHOD OF RANDOMISATION 83
9.2 OUTCOME MEASURES 83
9.3 SAMPLE SIZE: PRINCIPLES 84
9.4 SAMPLE SIZE ISSUES AND TRIAL STAGES: ADDITIONAL RESEARCH ARM G 86
9.4.1 Pilot Phase: Additional Research Arm G 86
9.4.2 Activity Stages I-III: Additional Research Arm G 86
9.4.3 Efficacy Stage IV: Additional Research Arm G 86
9.4.4 Sample Size For Additional Research Arm G 86
9.5 SAMPLE SIZE ISSUES AND TRIAL STAGES: ADDITIONAL RESEARCH ARM H 87
9.5.1 Pilot Phase: Additional Research Arm H 87
9.5.2 Activity Stages I-III: Additional Research Arm H 87
9.5.3 Efficacy Stage IV: Additional Research Arm H 87
9.5.4 Sample Size For Additional Research Arm H 87
9.6 SAMPLE SIZE ISSUES AND TRIAL STAGES: ADDITIONAL RESEARCH ARM J 89
9.6.1 Pilot Phase: Additional Research Arm J 89
9.6.2 Activity Stages I-II: Additional Research Arm J 89
9.6.3 Efficacy Stage III: Additional Research Arm J 90
9.6.4 Sample Size For Additional Research Arm J 90
9.6.5 Further Sample Size Issues For Additional Research Arm J 90
9.7 SAMPLE SIZE ISSUES AND TRIAL STAGES: ADDITIONAL RESEARCH ARM K 91
9.7.1 Implementation: Additional Research Arm K 91
9.7.2 Outcome Measures: Additional Research Arm K 91
9.7.3 Pilot Phase: Additional Research Arm K 92
9.7.4 Activity Stage I: Additional Research Arm K 92
9.7.5 Efficacy Stage II: Additional Research Arm K 93
9.7.6 Sample Size For Additional Research Arm K 93
9.7.7 Further Sample Size Issues For Additional Research Arm K 93
9.8 SAMPLE SIZE ISSUES AND TRIAL STAGES: ADDITIONAL RESEARCH ARM L 94
9.8.1 Implementation And Outcome Measures: Additional Research Arm L 94
9.8.2 Additional Use of Outcome Data from the "transdermal oestradiol comparison" 95

9.8.3 Definition of PFS and Use As Co-primary OM: Additional Research Arm L 96
9.9 FURTHER NOTES ON TRIAL DESIGN 96
9.9.1 Overall Sample Size 96
9.9.2 Factorial Design 96
9.10 INTERIM MONITORING AND ANALYSES 96
9.11 OUTLINE ANALYSIS PLAN 97
9.11.1 Pilot / Safety Phases 97
9.11.2 Activity And Efficacy Stages 98

10 MONITORING AND QUALITY ASSURANCE 99
10.1 MONITORING AT CTU 99
10.2 DIRECT ACCESS TO DATA 99
10.3 VISITS TO INVESTIGATOR SITES 99
10.4 CONFIDENTIALITY 99

11 SAFETY REPORTING 100
11.1 SAFETY REPORTING DEFINITIONS 100
11.1.1 Trial-Specific Exemptions 101
11.2 INSTITUTION/INVESTIGATOR RESPONSIBILITIES 101
11.2.1 Investigator Assessment 102
11.2.2 Notification Procedure 103
11.3 CTU RESPONSIBILITIES 104

12 ETHICAL CONSIDERATIONS AND APPROVAL 105
12.1 ETHICAL CONSIDERATIONS 105
12.2 ETHICAL APPROVAL 107

13 REGULATORY APPROVAL 108

14 INDEMNITY 109

15 FINANCE 110

16 TRIAL COMMITTEES 111
16.1 TRIAL MANAGEMENT GROUP (TMG) 111
16.2 TRIAL STEERING COMMITTEE (TSC) 111
16.3 INDEPENDENT DATA MONITORING COMMITTEE (IDMC) 111
16.4 TRIAL EXPERT PANELS 112

17 ANCILLARY STUDIES 113
17.1 QUALITY-OF-LIFE 113
17.2 HEALTH ECONOMICS 113
17.3 TRANSLATIONAL SUB-STUDIES 113

18 PUBLICATIONS 115

19 PROTOCOL AMENDMENTS 116
19.1 PROTOCOL 116
19.1.1 Amendments Made To Sections In Protocol Version 1.0 (May-2004) 116
19.1.2 Amendments Made To Sections In Protocol Version 1.1 (May-2005) 116

19.1.3 Amendments Made To Sections In Protocol Version 2.0 (Jun-2005) 116
19.1.4 Amendments Made To Section In Protocol Version 3.0 (Jul-2006) 117
19.1.5 Amendments Made To Protocol Version 4.0 (Dec-2007) 118
19.1.6 Amendments Made To Protocol Version 5.0 (Aug-2008) 118
19.1.7 Amendments Made To Protocol Version 6.0 (Jul-2009) 119
19.1.8 Amendments Made To Protocol Version 7.0 (Jul--2011) 119
19.1.9 Amendments Made To Protocol Version 7.1 (Jul-2011) 120
19.1.10 Amendments Made To Protocol Version 8.0 (Sep-2011).. 121
19.1.11 Amendments Made To Protocol Version 9.0 (Oct-2012).. 122
19.1.12 Amendments Made To Protocol Version 10.0 (Apr-2013).. 123
19.1.13 Amendments Made To Protocol Version 11.0 (Sep-2013).. 123
19.1.14 Amendments Made To Protocol Version 12.0 (Jan-2014) 123
19.1.15 Amendments Made To Protocol Version 13.0 (Feb-2015).. 124
19.1.16 Amendments Made To Protocol Version 14.0 (Oct-2015).. 124
19.1.17 Amendments Made To Protocol Version 15.0 (Mar-2016)... 125

20 REFERENCES 126

ABBREVIATIONS & GLOSSARY

ABBREVIATION	EXPANSION
AA	Anti-androgen
AAH	Amalgamated Anthracite Holdings
ACE	Angiotensin-Converting Enzyme
ACTH	Adrenocorticotropic hormone
ADT	Androgen deprivation therapy
AR	Androgen receptor
AS	Activity Stage
BID	Twice a day (bis in die)
BP	Blood pressure
BRG	Biological Research Group
BSA	Body surface area
CCI	Comparison Chief Investigator
CF	Consent Form
CI	Chief Investigator
CI	Confidence interval
Co-CCI	Comparison Co-Chief Investigator
Cox-2	Cyclooxygenase 2
CRF	Case Report Form
CRN	Clinical Research Network
CRUK	Cancer Research UK
CRPC	Castration Resistant Prostate Cancer
CT	Computerised tomography
CTA	Clinical Trials Authorisation
CTAAC	Clinical Trials Advisory and Awards Committee
CTC	Common Toxicity Criteria
CTU	Clinical Trials Unit
CTV	Clinical Tumour Volume
CXR	Chest X-ray
DAB	Dual Androgen Blockade
DHT	Dihydrotestosterone
DNA	Deoxyribonucleic Acid
DPA	Data Protection Act

ABBREVIATION	EXPANSION
ES	Efficacy Stage
ICH	International Conference on Harmonization
ECG	Electro cardiogram
FBC	Full Blood Count
FFS	Failure-Free Survival
GCP	Good Clinical Practice
GP	General Practitioner
HbA1c	Glycated haemaglobin
Hb	Haemoglobin
HE	Health Economics
HES	Hospital Episode Statistics
Hr	Hour
HR	Hazard Ratio
HSCIC	Health & Social Care Information Centre
HT	Hormone Therapy
IDMC	Independent Data Monitoring Committee
IM	Intramuscular
IMRT	Intensity Modulated Radiation Therapy
ISRCTN	International Standard Randomised Controlled Trial Number
IU	International Units
IV	Intravenous
LFTs	Liver Function Tests
LHRH	Luteinising Hormone Releasing Hormone
LREC	Local Research Ethics Committee
m	Month
mcg	Microgram
MHRA	Medicine and Healthcare Products Regulatory Agency
min	Minutes
MRC	Medical Research Council
MREC	Multi-Centre Research Ethics Committee
MRI	Magnetic resonance imaging
M0	Non-metastatic
M1	Metastatic
NCI	National Cancer Institute (USA)

ABBREVIATION	EXPANSION
NHS	National Health Service
N0	Node-negative
N+	Node-positive
NSAID	Non-Steroidal Anti-inflammatory Drugs
OD	Once per day (omne in die)
ONS	Office for National Statistics
OS	Overall Survival
PFS	Progression-free survival
PI	Principal Investigator
PIS	Patient Information Sheet
po	Orally (per orum)
PSA	Prostate Specific Antigen
pts	Patients
PTV	Planned Tumour Volume
QALY	Quality-adjusted Life Years
qds	Four times each day (quater die sumendus)
QL	Quality-of-life
R&D	Research and Development
RECIST	Response Evaluation Criteria In Solid Tumours
SAE	Serious Adverse Event
SAR	Serious Adverse Reaction
sc	Under skin (sub-cutaneous)
SOC	Standard-of-Care
SSA	Site Specific Assessment
STAMPEDE	Systemic Therapy in Advancing and Metastatic Prostate Cancer: Evaluation of Drug Efficacy
SUSAR	Suspected Unexpected Serious Adverse Reactions
SWOG	South West Oncology Group
tE2	Transdermal Oestradiol
TMG	Trial Management Group
TMT	Trial Management Team
TURP	Trans-Urethral Resection of Prostate
TSC	Trial Steering Committee
UCL	University College London

ABBREVIATION	EXPANSION
ULN	Upper Limit of Normal
U+E	Urea and Electrolytes
WHO	World Health Organisation

TERM	DEFINITION
ADT	Androgen deprivation therapy given in the form of LHRH agonists/antagonists (abbreviated to LHRH) or alternatively, transdermal oestradiol
Hormone Therapy	Refers to all forms of hormone therapy given in the first line setting and includes LHRH, anti-androgens and transdermal oestradiol.<br><br>This term does not include novel AR-targeted agents such as abiraterone or enzalutamide.
PSA nadir	For trial purposes, this refers to the lowest PSA value detected between randomisation and week 24 on trial. This is used to derive the PSA progression value.

1 LAY SUMMARY

Prostate cancers depend upon the male hormone testosterone for their growth. Lowering testosterone levels (either by removing all or part of both testes, or by giving anti-hormone treatment) slows the growth of prostate cancers. This type of treatment is called hormone treatment or androgen deprivation therapy (ADT) and is often used when prostate cancers have spread outside the prostate gland. Although hormone treatment is usually successful at stopping the cancer growing for a period of time, the cancer will begin to grow again in most men. In addition, standard hormone treatment with injections (LHRH) can cause a range of side-effects which may become serious and affect quality-of-life, particularly since some men could remain on treatment for a decade or longer.

The overall aim of this trial, which is called STAMPEDE, is to assess novel approaches for the treatment of men with prostate cancer who are starting long-term ADT for the first time. Since opening to accrual in Oct-2005, the trial has tested many ways of treating prostate cancer and some results are now already known. More than 10,000 men will join the trial with answers becoming available throughout the trial. The trial will also look at the effects each treatment has on quality-of-life, and which treatment provides the greater value for money for the health service.

New patients joining the trial from Protocol version 16.0 onwards may be eligible to join one of two treatment comparisons, metformin (treatment group K; the "metformin comparison") and transdermal oestradiol (treatment group L; the "transdermal oestradiol comparison"). A computer program will be used to allocate which treatment each participant receives, using a chance process.

Table 1: Summary of treatment groups currently open to recruitment (Protocol version 16.0)

TREATMENT BEING TESTED	TREATMENT GROUP	SUMMARY	FROM PROTOCOL VERSION
Metformin	Arm K	This anti-diabetic medication is proposed to have both anti-cancer effects and may help prevent the adverse metabolic effects of long-term ADT. STAMPEDE will investigate whether adding metformin to the current standard-of-care for non-diabetic men can improve all-cause survival.	15.0
Transdermal oestradiol	Arm L	This is a form of hormone treatment which can suppress testosterone as effectively as standard ADT and has been shown to avoid some of the side-effects. For example, treatment with transdermal oestradiol does not appear to cause the bone to thin, a common problem with standard forms of ADT which might lead to the bones becoming fragile (osteoporosis) and more likely to break. It may also help to avoid the adverse metabolic effects and fatigue and therefore improve overall quality of life compared with standard forms of ADT. STAMPEDE will investigate whether transdermal oestradiol can treat the cancer as well as current standard forms of ADT.	16.0

Further results are expected in the next few years from other treatments tested in STAMPEDE, which have completed recruitment. These include treatments currently used in different settings, including abiraterone and enzalutamide, both currently used when hormone treatment is no longer

effective and the cancer has started to grow again, termed castrate resistant prostate cancer (CRPC). Prostate radiotherapy, which is a treatment used in localised prostate cancer, has also been tested as an additional treatment for men with cancer that has spread to other parts of the body (metastatic prostate cancer). The results relating to these questions are expected in the next few years.

Table 2: Summary of treatment groups closed to recruitment; results awaited

TREATMENT BEING TESTED	TREATMENT GROUP	SUMMARY	FROM PROTOCOL VERSION
Abiraterone	Arm G	This is a novel hormone treatment which works by inhibiting steroid hormone synthesis so blocks prostate cancer cells from generating their own male hormones. This is thought to be a major way in which prostate cancer cells resume growth following anti-hormonal therapies. Abiraterone has been shown to prolong survival in men with advanced disease, when standard hormone treatments have stopped working. STAMPEDE is investigating whether abiraterone is beneficial when given earlier, when first starting long-term standard hormone therapy.	8.0
Prostate radiotherapy	Arm H	This is treatment with high-energy x-rays targeted to the prostate gland. This treatment is now mandatory within STAMPEDE for patients with cancer that is confined to the prostate gland as large trials have shown it improves life expectancy. We are not certain whether we should give radiotherapy to the prostate if the cancer has already spread and so we are investigating this in STAMPEDE.	9.0
Enzalutamide (given with abiraterone)	Arm J	Enzalutamide is another novel hormone treatment, similar to abiraterone, which is also used in advanced prostate cancer, when standard hormone therapy has stopped working. Enzalutamide works by blocking androgen receptors and this may complement abiraterone. STAMPEDE is testing whether this treatment combination is a more effective way of controlling prostate cancer growth for longer and improving life expectancy.	12.0

In the past STAMPEDE also tested whether adding docetaxel chemotherapy, zoledronic acid, celecoxib, alone or in combination, was benefical in controlling prostate cancer growth and improving life expectancy. Recruitment has now been completed to all of these original treatment groups and the results have been presented. For further information relevant to these treatment groups, refer to the STAMPEDE website where you can see earlier versions of the protocol and find summaries of the results and links to the scientific publications.

Table 3: Summary of treatment (groups) closed to recruitment; results reported

TREATMENT TESTED	TREATMENT GROUP	SUMMARY OF RATIONALE AND RESULTS	PROTOCOL VERSION ADDED
Zoledronic acid	Arm B	Prostate cancer cells can spread to bones and weaken them. Zoledronic acid is a drug that reduces bone destruction and hardens bones cells.<br>The results of STAMPEDE show that the addition of zoledronic acid does not prolong life expectancy. These results were compared with data from other similar trials that have tested this treatment, these data also support the findings of STAMPEDE.	1.0
Docetaxel	Arm C	Docetaxel is a type of chemotherapy which stops cells replicating. It has been used to treat advanced prostate cancer for some time, and is used in the treatment of lung, breast and ovarian cancer.<br>The results of STAMPEDE show that the addition of docetaxel to hormone treatment does improve survival, most markedly in men with metastatic disease, and delays time to progression for men with locally-advanced and metastatic disease.<br>The results of STAMPEDE were combined with other similar trials testing docetaxel and the results of the meta-analysis support this effect.<br>Docetaxel may now be given as part of standard treatment to all suitable men entering STAMPEDE (from Protocol version 14.0).	1.0
Celecoxib	Arm D	Celecoxib is an aspirin-like drug that is used to treat arthritis. It slows down the growth of cancer cells in the laboratory. STAMPEDE tested whether the addition of celecoxib could delay the growth of prostate cancer cells. Recruitment stopped early as a planned intermediate analysis failed to demonstrate sufficient effect of this drug. The final results were presented at GU ASCO 2016 and show that alone, celecoxib does not improve life expectancy.	1.0

Note that the combination of docetaxel and zoledronic acid was assessed in Arm E and, whilst beneficial overall, did not provide additional benefit over docetaxel. The combination of celecoxib and zoledronic acid was assessed in Arm F but did not show an overall benefit.

2 BACKGROUND

2.1 INTRODUCTION AND SETTING

Prostate cancer is a major health problem world-wide and accounts for nearly one fifth of all newly-diagnosed male cancers. In the UK, approximately 46,700 men were diagnosed with prostate cancer in 2014 and over 11,000 men died from the disease(3).

2.1.1 Long-term Androgen Deprivation Therapy

The initial (first-line) treatment for locally-advanced or metastatic prostate cancer is based on androgen deprivation therapy (ADT) achieved either surgically with bilateral orchidectomy, or medically with LHRH agonists or antagonists (4). Long-term use of oral anti-androgens is permitted only when given with LHRH agonists, to achieve dual androgen blockade (previously termed maximum androgen blockade - MAB).

ADT produces responses in up to 95% of patients but it is not curative and disease recurs in virtually all patients treated with ADT as sole therapy, with a median time to progression of 18-24 months (4). Data from the control arm in STAMPEDE has shown that for men with newly-diagnosed metastatic disease, treated with ADT alone, the time to progression is just 11 months. Such progressive disease is referred to as castrate resistant prostate cancer (CRPC).

Another important issue with ADT is the numerous associated side-effects, particularly with prolonged use. Since patients continue on LHRH after disease progression (with additional agents added), many men remain on treatment for a decade or longer, particularly as life expectancy for men with prostate cancer should continue to improve as the number of effective treatments increases. The adverse effects of ADT using LHRH analogues include osteoporosis (leading to an increased risk of fracture), adverse metabolic effects, cognitive decline, sexual dysfunction, hot flushes, physical deterioration and fatigue.

2.1.2 Role Of Radiotherapy For Men With M0 Disease

Two randomised trials, SPCG7 (5) and NCIC PR3 / MRC PR07 (6-8) have tested the question of whether ADT alone combined with radiotherapy is the best treatment for patients with high-risk localised prostate cancer (N0M0). Both trials demonstrated an improvement in overall and disease specific survival from the addition of radiotherapy to ADT. The size of this overall survival benefit is substantial (hazard ratio 0.68 in SPCG7 and 0.77 in PR07). As these two mature, large, well-conducted randomised trials have demonstrated benefit, we now mandate that radiotherapy be standard for patients with N0M0 disease (i.e. no nodal or metastatic spread). Patients with node-negative M0 prostate cancer will only be allowed to enter the trial if standard radiotherapy is planned. Any patients with N0M0 disease for whom radiotherapy is contra-indicated should be discussed with the STAMPEDE team prior to inclusion. For patients with node-positive, M0 disease there are no randomised data on whether radiotherapy is indicated or not. However the NCIC PR3 / MRC PR07 trial included patients with unknown nodal status who received whole pelvic radiotherapy (9) and demonstrated a large overall benefit. Additionally, non-randomised data from the STAMPEDE control arm suggests that the benefit observed in patients with N0M0 disease can be extended to those with pelvic nodal involvement. Therefore the STAMPEDE TMG recommends that pelvic nodal radiotherapy be considered for patients with node-positive, M0 disease at the discretion of the treating clinician (10).

2.1.3 Role Of Docetaxel For Men With M0 Or M1 Disease

The primary analysis of the "original comparisons" has shown docetaxel to significantly prolong survival (HR 0.78; 95% CI 0.66-0.93)(1). This is in support of the results of the CHAARTED trial which showed docetaxel improved survival in men with metastatic disease(11, 12). There was no evidence of heterogeneity in STAMPEDE in the treatment effect across any patient subgroups and median survival was improved by 10 months, from 71 to 81 months. In a well powered and pre-planned sub-group analysis of men with metastatic disease at randomisation the treatment effect was most apparent with a median survival benefit of 15 months. As a result the STAMPEDE TMG recommends that docetaxel should be strongly considered in all men with metastatic disease at presentation who are commencing ADT for the first time and are fit enough to receive chemotherapy.

Survival data for men without metastases at diagnosis is less mature but a statistically significant improvement in failure-free survival is seen, therefore, docetaxel may also be considered for men with high-risk non-metastatic disease who are commencing ADT for the first time and are fit enough to receive chemotherapy. Therefore, docetaxel is now permitted as part of the standard-of-care for all men entering STAMPEDE at the discretion of the treating clinician and patient.

2.2 RATIONALE

There are increasing numbers of treatments which are used post-relapse of first-line ADT in patients with CRPC, but there has been limited evidence as to which is associated with the best response, how they may be combined or sequenced or whether any of them might have a role as first-line treatment. An alternative approach is to investigate the addition of new drugs as part of first-line therapy in patients starting ADT. At this point, patients should be fitter and better able to tolerate treatment than when they have CRPC, and there is the possibility of having a larger and longer-lasting effect.

The increasing and widespread use of ADT in prostate cancer management has led to growing awareness of the adverse effects of LHRH. An alternative approach for improving long-term outcomes in patients is therefore to mitigate some of these side-effects. Many of these side-effects can affect quality-of-life as well as result in significant morbidities and potentially life-threatening consequences, particularly with prolonged treatment and in patients with exisiting co-morbidities.

For these reasons, metformin is being evaluated within the trial as a re-purposed treatment for prostate cancer, because of its potential anti-cancer effects (based on pre-clinical and epidemiological evidence) and the expectation that it may counteract the metabolic effects of long-term ADT. Similarly, transdermal oestradiol, another novel re-purposed treatment approach, is being evaluated as an alternative form of ADT which may be as effective or more effective than LHRH in treating prostate cancer but with fewer side-effects.

2.3 DESIGN

STAMPEDE (also known as MRC PR08) is an innovative, multi-arm multi-stage, multi-centre, randomised controlled trial. It initially assessed the effects of a bisphosphonate (zoledronic acid), a cytotoxic chemotherapeutic agent (docetaxel) and a cyclooxygenase (Cox-2) inhibitor (celecoxib), as single agents or combinations, in patients commencing long-term ADT for locally advancing or metastatic prostate cancer. For these questions, each comparison was divided into five stages such that, for each investigational arm, safety and activity data were generated in the first four stages; an

investigational arm could only proceed to the fifth and final stage of recruitment, where it would be assessed for effect on overall survival, if shown to be sufficiently safe and active at all prior activity stages. Patient data from all arms and all stages are, however, included in the final analyses of the primary outcome measure, even if the investigational arm did not proceed to the final stage. Of note, a second, pre-planned interim analysis failed to demonstrate sufficient activity for celecoxib and this agent was removed from trial recruitment in Apr-2011; patients remaining on celecoxib treatment reverted to standard care. Results for all of these "original comparisons" have now been reported(13, 14).

Since the start of the trial, a number of new research arms have been added to STAMPEDE over time to evaluate: abiraterone, a steroid synthesis inhibitor; prostate radiotherapy for patients with newly-diagnosed metastatic disease; enzalutamide, an inhibitor of androgen receptor signalling, given with abiraterone; and metformin, an anti-diabetic medication. In Protocol version 16.0, a new research arm is added for transdermal oestradiol, to be given as an alternative form of ADT.

2.4 PREVIOUSLY REPORTED RESEARCH TREATMENTS

Data have been reported on zoledronic acid, docetaxel, celecoxib and the combination of zoledronic acid with docetaxel or with celecoxib. As such the rationale for these treatments, along with their design and details of treatment administration are no longer covered within the Protocol.

2.5 RATIONALE FOR RESEARCH TREATMENTS UNDER EVALUATION

2.5.1 Steroid Synthesis Inhibitors

Note: recruitment to both the abiraterone containing comparisons has now been completed as the required target accrual was reached.

Recent evidence suggests that an important mechanism for escape from tumour control by androgen ablation is the intracellular conversion of steroid precursors to androgenic steroids by prostate cancer cells. A key enzyme in this process is CYP17, which therefore represents a logical target for therapy in CRPC(15). Abiraterone acetate (3β-acetoxy-17-(3-pyridyl)androsta-5,16-diene, code CB7630; JNJ-212082) is rapidly converted in vivo to abiraterone (JNJ-589485; formerly code named CB7598). It is a selective, irreversible inhibitor of 17α-hydroxylase/C17,20-lyase (cytochrome P450c17 [CYP17]), an enzyme that is critical in the production of androgens in the testes, adrenal glands and prostate tumour tissue. Inhibition of CYP17 inhibits the conversion of pregnenolone or progesterone into dehydroepiandrosterone (DHEA) or androstenedione, respectively, each of which is a precursor of testosterone. The pharmacodynamic effect is a more effective androgen depletion than can be induced by surgical castration, or medically by gonadotropin releasing (GnRH) hormone analogues used as first-line hormone therapy in prostate cancer.

Approximately 2,280 prostate cancer patients participated in the two Phase 3 RCTs (COU-AA-301 and COU-AA-302), with approximately 1,335 patients receiving abiraterone acetate at 1000mg daily dose continuously, in these studies. These studies have demonstrated abiraterone to prolong survival when given post-docetaxel (HR 0.65) and pre-docetaxel (HR 0.82). As a result it is now approved use in the USA and Europe in CRPC (16, 17).

Side-effects with abiraterone acetate are modest with the main adverse effects being elevated transaminases (usually mild), hypokalaemia and hypertension due to secondary hyperaldosteronism

and fluid retention (preventable by low doses of glucocorticoids). In order to prevent secondary hyperaldosteronism, it is recommended that prednisolone (or prednisone) 10mg daily be administered in the CRPC setting. Within more recent studies in earlier stage patients, lower doses (typically 5mg of prednisone/prednisolone) are being used due to concerns about side effects of long-term exposure to glucocorticoid. Within the STAMPEDE trial, we suggest prednisolone/ prednisone dose of 5mg OD, which may be increased to 5mg BID at the investigator's discretion if there are any concerns about monitoring or risks for the patient with 5mg OD.

We hypothesise that abiraterone may be more active still, when given up-front in combination with first-line ADT, by preventing or delaying the development of castrate refractory disease.

2.5.2 Radiotherapy To The Prostate For Patients With Newly-Diagnosed Metastatic Disease

Note: recruitment completed to the radiotherapy arm in Sep-2016 as the revised recruitment target sample was reached. Treatment has been completed in all patients and the results will be reported when the data has matured. See Protocol version 15.0 or older for details on the rationale.
(18)(19)(5, 9)

2.5.3 Combination Of Steroid Synthesis Inhibitors And Androgen Receptor Signalling Inhibitor

The most common form of disease progression for men on single-agent abiraterone or enzalutamide is a rise in PSA. This would suggest that the mechanism driving resistance is increased PSA transcription resulting from reactivation of the androgen receptor (AR), or another steroid signalling pathway(20).

The primary pharmacodynamic effect of enzalutamide is inhibition of androgen binding to the AR, AR nuclear translocation in the presence of androgen and AR chromatin association. In multiple prostate cancer cell lines that specifically model CRPC (LNCaP/AR, VCaP, W741C LNCaP), the consequences of enzalutamide treatment include inhibition of AR-induced gene transcription, reduced cell proliferation, increased cell death by apoptosis and tumour regression.

In a mouse xenograft model of CRPC using prostate cancer cells that overexpress the AR (LNCaP/AR), enzalutamide inhibits tumour growth and reduces tumour size. A major human metabolite of enzalutamide, N-desmethyl enzalutamide, demonstrates key primary pharmacodynamics of similar potency to the parent molecule, while the carboxylic acid derivative metabolite has no known pharmacodynamic effect.

The question under investigation is: can progression be delayed (and survival extended) by using a combination of abiraterone and enzalutamide given up-front in combination with first-line ADT?

2.5.3.A Supplementing Abiraterone And Prednisolone With Enzalutamide

Several studies have shown that the AR can become promiscuously activated by very low levels of androgens or other steroid metabolites and drugs that bind the AR (21-24). It is known that very low levels of androgens can persist in patients treated with abiraterone acetate (25). Drugs that bind the AR, may include co-administered glucocorticoids. Furthermore, AR mutations of the sort previously described in CRPC, can be activated by cortisol and other glucocorticoids at levels much lower than those reported in patients treated with abiraterone and prednisolone at a dose of 5mg bid (24, 26). Moreover, abiraterone binds the AR and, although weak antagonism of wild-type and most previously described AR mutations are observed (26), a similar mechanism to that described with

classical anti-androgens, such as bicalutamide, could lead to change-of-function AR mutations associated with AR activation following abiraterone binding. Therefore, concomitant treatment with an androgen receptor signalling inhibitor could prevent "promiscuous" AR activation in patients treated with abiraterone. Enzalutamide is an androgen receptor signalling inhibitor and is approved for use on its own in the treatment of advanced CRPC (27), and there is evidence of activity for hormone-naïve prostate cancer (28).

2.5.3.B Supplementing Enzalutamide With Abiraterone And Prednisolone

Enzalutamide in combination with ADT is both effective and well tolerated in CRPC(27). However, recent studies have suggested that intra-tumoral testosterone levels increase in patients treated with enzalutamide (29). The implications of this finding are that the increase in intra-tumoral testosterone could be associated with up-regulation of enzymes involved in steroid biosynthesis(30). Although enzalutamide has a high affinity for the AR, this is several-fold lower than both the natural ligands testosterone and DHT (31), which means that enzalutamide would be out-competed at the AR ligand-binding domain if and when androgen levels rise. In vitro, a ten-fold rise in intra-cellular androgen was sufficient to prevent inhibition of AR by 30uM of enzalutamide(26); these levels are representative of the plasma levels of enzalutamide active metabolites, which can be achieved with enzalutamide 160mg po daily (32).

A strategy for preventing the rise in intra-cellular androgens in patients treated with enzalutamide would be inhibition of CYP17A1. Abiraterone is currently the only CYP17A1 inhibitor with proven efficacy. It therefore seems logical to use the combination of enzalutamide and abiraterone to both block a rise of intra-cellular androgens and prevent promiscuous activation of the AR.

2.5.3.C Summary Of Rationale For This Combination

To date, investigation has focussed on patients with CRPC but there is a strong rationale for the combination of enzalutamide and abiraterone in the hormone treatment-naïve setting in which STAMPEDE is focused.

STAMPEDE is already evaluating abiraterone plus conventional ADT but we will not assess the combination of conventional ADT plus enzalutamide; other trials by industry and other cooperative groups will address that question. The inclusion of an arm with ADT and enzalutamide in STAMPEDE was therefore considered to be a duplication of effort and was not supported by the Trial Management Group.

The combination of enzalutamide and abiraterone is a novel approach and offers considerable promise in delaying progression – it therefore represents an attractive addition to the comparisons under investigation in STAMPEDE, and one that is unlikely to be replicated in other planned trials of this size.

2.5.4 Metformin

All men joining STAMPEDE are planned for long-term ADT, a treatment associated with an increased risk of insulin resistance, hyperglycaemia, dyslipidaemia and obesity. Over 50% of men receiving long-term ADT will develop Metabolic Syndrome (33) resulting in increased cardiovascular morbidity and mortality. Obesity and high bind insulin C-peptide levels, indicating insulin resistance are independent predictors of increased prostate cancer-specific mortality and the presence of metabolic syndrome and diabetes in men treated with ADT is associated with shorter survival.

Metformin, which in non-diabetic individuals has been shown to lower the incidence of diabetes, counteracts some of these side-effects of ADT, including insulin insensitivity, hyperinsulinaemia and diabetes. It also reduces the levels of cholesterol, LDLs and triglycerides by inhibiting the fatty acid synthesis via activation of Adenosine Monophosphate Activated Kinase (AMPK) and decreases the platelet aggregation factor 1, platelet aggregation, vascular adhesion molecules, CRP and leptin (34-37). Through mitigation of the cardiovascular and metabolic consequences of ADT, metformin is proposed to reduce treatment-associated morbidity and improve all-cause mortality.

In addition, recent data has emerged consolidating the knowledge that cancer progression is linked integrally with metabolic modulators and that modification of this process by metformin has an important effect on cancer progression and survival. Pre-clinical data has shown that metformin is an important stimulator of AMPK which acts as the cellular “masterswitch” for energy regulation. AMPK acts to inhibit the effects of elevated insulin levels which promote metastasis, tumour growth and treatment resistance. Insulin increases mRNA and protein expression of steroidogenic enzymes leading to the up-regulation of intracellular testosterone levels, secreted androgens, thereby activating the AR (38). Metformin also influences the PI3K-AKT pathway and has an anti-proliferative effect via inhibitor of mTOR as well as targeting cancer stem cells. In vitro, metformin has been shown to inhibit androgen-induced IGF-IR up-regulation through disruption of androgen signalling (39).

Evidence in support of this includes a systematic review and meta-analysis of 13,008 men with type 2 diabetes mellitus (T2DM) and concurrent cancer which has shown improved survival in men treated with metformin compared with other anti-diabetic agents. In a systematic review of observational data from over 1 million men, there was a significant association seen between metformin and decreased risk of death from any cancer. Another systematic review found that the use of metformin in diabetic patients was associated with a significantly lower risk of cancer incidence and cancer mortality (40). In a large retrospective cohort study of 3837 diabetic men with prostate cancer, metformin was associated with a decreased risk of prostate cancer specific mortality (HR=0.76 [0.64-0.89]) and death (HR=0.76 [0.70-0.82]). In a prospective non-randomised phase II study in non-diabetic CRPC patients, 36% of patients receiving metformin were progression-free at 3 months and >50% had a prolongation of their PSA doubling time (41).

In summary, meformin is proposed to mitigate many of the adverse side effects of long-term ADT as well as having multiple potential anti-cancer effects and therefore STAMPEDE will evaluate re-purposing this treatment as a novel therapeutic approach in the management of high risk locally-advanced or metastatic prostate cancer.

2.5.5 Transdermal Oestradiol

2.5.5.A Background & Rationale

ADT with LHRH analogue injections suppresses testosterone to castrate levels, but also depletes oestradiol, since around 80% of oestradiol in men is derived by aromatisation from testosterone. Thus men who are treated with LHRH will have toxicities caused by low levels of both testosterone and oestrogen. The LHRH-associated toxicities which are due to low testosterone include loss of libido, erectile dysfunction and decrease in muscle mass. Other toxicities associated with LHRH such as osteoporosis, increased fracture risk, hot flushes, memory loss, dyslipidemia and increased body fat deposition are thought to be due to oestradiol deficiency. In particular, the adverse effect of LHRH on bone health has been well documented. Oestradiol deficiency prolongs the life-span of bone-resorptive osteoclasts, with the resulting imbalance between osteoclasts and bone-forming

osteoblasts increasing the rate of bone thinning. This may lead to osteoporosis and increased risk of fracture, with the rate of fracture increasing with duration of LHRH.

Transdermal oestradiol is a potential alternative to LHRH that may avoid some treatment-related side-effects, therefore improving quality-of-life, which would be advantageous if shown to be equally effective at prolonging survival. Exogenous administration of oestradiol suppresses androgen production through a negative feedback loop involving the hypothalamic-pituitary axis, whilst avoiding the fall in oestradiol associated with castrate levels of testosterone(42). This, in turn, mitigates the toxicities of LHRH associated with oestradiol deficiency. Oral oestrogen was previously used for ADT before the development of LHRH, but discontinued as first-line treatment due to increased thromboembolic toxicity, attributable to first-pass hepatic metabolism (43).

Parenteral administration (e.g. intravenous, intramuscular or transdermal oestradiol) avoids first-pass hepatic metabolism, mitigating the cardiovascular risk, as supported by results so far from the ongoing PATCH (Prostate Adenocarcinoma TransCutaneous Hormones [MRC PR09; ISRCTN70406718]) trial and previous studies evaluating parenteral oestradiol in the form of intramuscular polyestradiol phosphate (42, 44).

To date, there are a number of encouraging results from the PATCH trial demonstrating the safety and early activity of transdermal oestradiol compared to LHRH agonists in men with advanced hormone-naïve prostate cancer (see **Appendix I** for further details). In particular, similar rates of cardiovascular events have been observed in the transdermal oestradiol and LHRH arms, as well as equivalent rates of testosterone suppression (based on around 900 patients enrolled up to Oct-2015) (42). Transdermal oestradiol has been shown to avoid the loss in bone mineral density associated with LHRH, and results in improved metabolic profiles and quality-of-life compared to LHRH(45). Furthermore, a pre-planned, confidential, interim analysis of the PATCH trial in Jun-2013 based on progression-free survival (PFS) led to the trial being extended to phase III; that analysis included n=638 participants with 206 PFS events, reviewed against a pre-specified non-inferiority margin hazard ratio of 1.25 and 1-sided alpha 0.25. The phase III evaluation of clinical efficacy for transdermal oestradiol will be based on progression-free and overall survival as co-primary outcome measures.

Demonstrating that transdermal oestradiol is an equally effective approach to ADT would provide a globally important alternative (to LHRH), with the potential to reduce treatment-associated morbidity and improve quality-of-life. In addition, there is a possibility that transdermal oestradiol may improve overall survival compared to standard hormone therapy. First, transdermal oestradiol may reduce treatment-associated morbidity and could potentially benefit overall survival. Second, up to 30% of men with castrate-resistant prostate cancer respond to oral oestrogen as post-relapse therapy, suggesting oestradiol may potentially have additional direct anti-tumour effects(46).

2.5.5.B Meta-Analysis With PATCH Trial

To further assess the clinical efficacy of transdermal oestradiol, the relevant data from the "transdermal oestradiol comparison" within STAMPEDE will be combined with data from patients recruited into PATCH i.e. the "transdermal oestradiol comparison" within STAMPEDE is not sufficiently powered to form a stand-alone analysis. The evaluation of transdermal oestradiol will be based on a non-inferiority approach (in contrast to the other comparisons within STAMPEDE which are superiority questions), to test the hypothesis that transdermal oestradiol is at least as effective as standard hormone therapy, but with fewer side-effects.

Recruitment of patients to the "transdermal oestradiol comparison" through STAMPEDE enables the transdermal oestradiol research question to be answered more quickly than via PATCH alone. It also

reduces the number of patients allocated standard treatment alone in both trials, thereby increasing the proportion of patients receiving a novel treatment approach and improving trial efficiency.

As of Feb-2017, nearly 1,200 patients have been recruited directly to the PATCH trial (also coordinated by MRC CTU at UCL) for the phase III evaluation of clinical efficacy of transdermal oestradiol. The overall recruitment target for the transdermal oestradiol evaluation is approximately 2,000 patients (including initially around 500 to be recruited through STAMPEDE).

3 SELECTION OF INSTITUTIONS AND INVESTIGATORS

Centres who wish to participate in the STAMPEDE trial should be registered with the MRC CTU at UCL for this purpose. Before any patients are registered or randomised, the CTU must receive a completed and signed Investigator Statement. The STAMPEDE Investigator Statement is signed by the Principal Investigator for that institution (download from http://www.stampedetrial.org/). The return of the Investigator Statement will be taken as confirmation of agreement to adhere to the trial protocol. In addition, a fully-signed model agreement is also required before recruitment can begin.

In compliance with the principles of GCP, all institutions participating in the trial will complete a delegation log and forward this to the CTU. Each person working on the STAMPEDE trial must sign off a section of this log indicating their responsibilities. CTU must be notified of any changes to trial personnel and/or their responsibilities. An up-to-date copy of this log must be stored in the Investigator Site file at the institution and also at CTU.

The Clinical Trial Authorisation (CTA) for the STAMPEDE trial requires that the Medicines and Healthcare Products Regulatory Agency (MHRA) be supplied with the names and addresses of all participating investigators/institutions. Trial staff at the CTU will perform this task; hence, it is vital to receive full contact details for all investigators prior to their entering patients.

Finally, before a patient is entered into the trial, and any trial-related procedures are conducted, written informed consent must be obtained. Approved patient information sheets and informed consent forms are supplied as templates.

Only a limited number of centres participated in the initial Pilot Phase of the original trial; this was to ensure that safety and feasibility data were collected expediently. Subsequent stages of the trial are open to any centre that wishes to participate and has fulfilled the requirements described above. In addition for some comparisons, there will be additional criteria required prior to accreditation, see **Sections 3.1** and **3.2**.

Following substantial amendments and future comparisons opening, sites will be notified of relevant documents and training required and if and when they are able to participate. Further accreditation packs may be circulated as a result to update trial documentation.

3.1 COMPARISON-SPECIFIC SITE ACCREDITATION

3.1.1 "Transdermal Oestradiol Comparison"

Only UK centres participating in STAMPEDE will be accredited for the "transdermal oestradiol comparison", since treatment with transdermal oestradiol is administered using Progynova TS 100mcg/24 hours transcutaneous oestradiol patches (see **Section 6.2.8**), which are currently unavailable in Switzerland.

3.2 FUTURE PLANNED BIOMARKER-SELECTED COMPARISONS

Initially, only UK centres participating in STAMPEDE will be accredited for the biomarker-selected comparisons, which are in development and will be incorporated in the next version of the protocol.

Sites wishing to gain accreditation for this future comparison should participate in the biomarker-screening pilot, being conducted within Protocol version 16.0.

Full details of the accreditation procedure for the future biomarker-selected comparisons will be available in the next version of the protocol. Centres participating in the biomarker-screening pilot will need to complete a feasibility assessment and will be provided with additional training prior to activation of the pilot. An additional agreement signed by the Head of Histopathology Service with contact details of a designated secretarial or technical person at each site will be required from each site participating in biomarker testing.

Please refer to the **Sample collection and handling manual** for details of the pilot feasibility study.

3.3 REQUIRED TRIAL DOCUMENTATION

Table 4 presents a summary of the required trial documentation for participating centres. Templates are provided on the STAMPEDE website www.stampedetrial.org.

Table 4: Trial documentation required for participating centres

TRIAL DOCUMENTATION	TIMING
R&D approval (or local equivalent; including IRMER approval)	Before centre participation
Investigator Statement	Before centre participation
Signature list & delegation of responsibilities	Before centre participation
Trial personnel contact details	Before centre participation
PIS, GP & CF on local paper	Before centre participation
Signed Clinical Trial Agreement between Trust and Sponsor (or Variation if applicable)	Before centre participation
Site initiation training	Before centre participation
Pharmacy Pack acknowledgment	Before centre participation
Pathology Statement (for sites participating in biomarker-screening)	Before centre participation

4 SELECTION OF PATIENTS

4.1 GENERAL INCLUSION CRITERIA

Participants must fulfil both of the criteria in Section 4.1.1 **or at least** one criterion in Section 4.1.2 **or** at least one criterion in Section 4.1.3. Additionally, all patients must fulfil the criteria in Section 4.1.4.

4.1.1 High-Risk Newly-Diagnosed Non-Metastatic Node-Negative Disease

Both:

- At least two of: T category T3/4, PSA≥40ng/ml or Gleason sum score 8-10
- Intention to treat with radical radiotherapy (unless there is a contra-indication; exemption can be sought in advance of consent, after discussion with CTU)

OR

4.1.2 Newly-Diagnosed Metastatic Or Node-Positive Disease

At least one of:

- Stage T_{any} N+ M0
- Stage T_{any} N_{any} M+

OR

4.1.3 Previously Radically Treated, Now Relapsing (Prior Radical Surgery And/Or Radiotherapy)

At least one of:

- PSA ≥4ng/ml and rising with doubling time less than 6 months
- PSA ≥20ng/ml
- N+
- M+

AND

4.1.4 For All Patients

I. Histologically confirmed prostate adenocarcinoma
II. Intention to treat with long-term androgen deprivation therapy
III. Treating clinician and patient should have decided if docetaxel is to be part of the standard-of-care prior to randomisation
IV. Fit for all protocol treatment[1] and follow-up, WHO performance status 0-2[2]
V. Have completed the appropriate investigations prior to randomisation
VI. Adequate haematological function: neutrophil count >1.5x10^9/l and platelets >100x10^9/l
VII. Adequate renal function, defined as GFR>30ml/min/1.73m^2
VIII. Serum potassium ≥3.5mmol/L
IX. Written informed consent
X. Willing and expected to comply with follow-up schedule
XI. Using effective contraceptive method if applicable

[1] Medical contraindications to the trial medications are given in Section 6
[2] For WHO performance status definitions see Appendix A

4.2 GENERAL EXCLUSION CRITERIA

Patients must not fulfil any of the criteria, below.

I. Prior systemic therapy for locally-advanced or metastatic prostate cancer except as listed in **Section 4.1.3**
II. Metastatic brain disease or leptomeningeal disease
III. Abnormal liver functions consisting of any of the following:
   - Serum bilirubin ≥1.5 x ULN (except for patients with Gilbert's disease, for whom the upper limit of serum bilirubin is 51.3μmol/l or 3mg/dl)
   - Aspartate aminotransferase (AST) or alanine aminotransferase (ALT) ≥2.5 x ULN
IV. Any other previous or current malignant disease which, in the judgement of the responsible clinician, is likely to interfere with STAMPEDE treatment or assessment
V. Any surgery (e.g. TURP) performed within the past 4 weeks
VI. Patients with significant cardiovascular disease such that, in the investigator's opinion, the patient is unfit for any of the study treatments. This might include:
   - Severe/unstable angina
   - Myocardial infarction less than 6 months prior to randomisation
   - Arterial thrombotic events less than 6 months prior to randomisation
   - Clinically significant cardiac failure requiring treatment (NYHA II-IV)[3]
   - Cerebrovascular disease (e.g. stroke or transient ischaemic episode) less than 6 months prior to randomisation
   - Patients with uncontrolled hypertension defined as systolic BP greater or equal than 160mmHg or diastolic BP greater or equal than 95mmHg[5]
VII. Prior chemotherapy for prostate cancer (excluding patients receiving docetaxel as part of the new SOC)
VIII. Prior exposure to long-term hormone therapy before randomisation (unless as described in **Section 4.5.4**)
IX. Prior exposure to systemic treatment for prostate cancer (excluding hormone therapy) e.g. abiraterone and enzalutamide.

[3] NYHA classifications can be found in **Appendix A**
5 Based on representative values, as judged by the investigator

4.3 BIOMARKER-SCREENING PILOT

4.3.1 Selection Criteria For Patient Registration

In preparation for the introduction of biomarker-selected comparisons, a Biomarker-Screening Pilot is being undertaken within Protocol version 16.0. This will be undertaken in a limited number of sites. Participation in the pilot will help to facilitate site accreditation for recruitment to the future biomarker-selected comparisons see Section 3.2.

All patients who fulfil the criteria below should be **registered without delay** in order to proceed to biomarker-screening.

- Registered at a site participating in biomarker-screening
- Confirmed metastatic disease (M1)
- Recent (obtained within 6 months prior to registration) FFPE tumour sample available for prompt transfer (within 1 week after registration)
- Written informed consent provided for the STAMPEDE trial and for biomarker-screening
- If hormone therapy has started, the maximum prior exposure allowed is 7 weeks of anti-androgens and if LHRH have been started, the maximum prior exposure is 5 weeks to registration. Ideally registration will occur as soon as possible and within 4 weeks of starting anti-androgens and within 2 weeks of starting LHRH.

Once eligibility is confirmed complete the registration CRF and contact the CTU to proceed to trial registration, see Section 5.1.

At the point of registration three samples are required to complete the biomarker-screening.
- Expedited retrieval of FFPE tumour block (to be transferred within a **maximum of 1 week**)
- Baseline blood sample collected using cell free DNA StreckTM tubes
- Saliva sample

For further details please refer to the **Sample collection and handling manual**.

Registration should occur **before** the patient is randomised. In the pilot, results of biomarker-screening are **not** required prior to randomisation as recruitment has not yet been activated for a biomarker-selected comparison; the patient can be randomised straight after being registered. For further details on results and feedback to patients please see Section 4.6.5.

Patients who participate in the pilot will continue to be allocated to any of the current open arms for which they are eligible (arm A, L and K).When recruitment is activated to biomarker-selected comparisons, the results of biomarker-screening will determine eligibility and will be required prior to randomisation. The screening pilot will inform the randomisation process for future biomarker-selected comparisons.

4.4 COMPARISON-SPECIFIC SELECTION CRITERIA

4.4.1 For Randomisation To The "Metformin Comparison"

Patients with known diabetes mellitus are not eligible for randomisation to the "metformin comparison".

All non-diabetic patients require an HbA1c to be performed prior to randomisation (ideal timeline: within 8 weeks prior to randomisation), to confirm their non-diabetic status.

In addition, an assessment of renal function is required to determine glomerular filtration rate (GFR). The method used to determine glomerular filtration rate may vary according local practice. Equations that either estimate glomerular filtration rate (eGFR) or creatinine clearance (CrCl) may be used and the same threshold value applies.

In summary, additional inclusion criteria specifically for the "metformin comparison" are:

- HbA1c <48mmol/mol (equivalent to <6.5%)*
- Adequate renal function, defined as GFR ≥45ml/min/1.73m^2
- No history of lactic acidosis or pre-disposing conditions
- Not current or previous treatment with metformin
- No contra-indications to metformin

* Except Switzerland, please refer to SAKK appendix for local guidance

Note that if the patient is known to be diabetic or the patient is found to have diabetes mellitus (i.e. HbA1c is 6.5% or higher) following screening, the patient is only eligible for randomisation if they meet all of the selection criteria for the “transdermal oestradiol comparison” (randomisation between Arms A and L only; see **Section 4.4.2**).

All patients with abnormal baseline HbA1c (i.e. 6.0% or higher) should be informed and referred to their GP for further management.

Where possible, the screening bloods, including HbA1c, should be performed prior to commencing SOC docetaxel. This is to reduce the likelihood of corticosteroid-related hyperglycaemia impacting on eligibility for the "metformin comparison".

4.4.2 For Randomisation To The “Transdermal Oestradiol Comparison”

Patients who have any of the following are not eligible for the “transdermal oestradiol comparison”:

- >8 weeks of anti-androgen use
- >1 dose of monthly or 4 weekly LHRH agonist/antagonist
- Prior LHRH agonist injection with a stated duration of effect greater than 1 month
- >12 weeks since first dose of any hormone therapy
- Bilateral orchidectomy
- Cyproterone acetate started prior to randomisation
- Known porphyria
- Any history of deep vein thrombosis or pulmonary embolism confirmed radiologically
- Known thrombophilic disorder (e.g. Protein C, prostein S, antithrombin deficiency)

Note that patients unsuitable for the “transdermal oestradiol comparison” will only be eligible for randomisation if they meet all of the selection criteria for the “metformin comparison” and therefore may be allocated to control (arm A) or metformin (arm K) only (see **Section 4.4.1**).

Patients presenting with relapsed disease who fulfil criteria in **Section 4.1.3** are also eligible for the “transdermal oestradiol comparison” providing the neo-adjuvant or adjuvant hormone therapy previously received adheres to criteria outlined in **Section 4.5.4.**

4.5 SCREENING PROCEDURES

Table 5: Summary of initial required screening and baseline investigations

TIMEPOINT	PATIENTS PARTICIPATING IN BIOMARKER-SCREENING PILOT
PRE-REGISTRATION	✓ Radiological confirmation of metastatic disease<br>✓ FBC, U&Es, LFTs, Creatinine or estimated GFR<br>✓ Retrieval of recent FFPE tumour block (within 6 months of registration)
POST REGISTRATION	✓ Blood collection (cell free DNA Streck™ tubes)<br>✓ Saliva sample
TIMEPOINT	ALL PATIENTS
PRIOR TO RANDOMISATION	✓ Bloods: FBC, U&Es, LFTs, Creatinine, HbA1c, PSA (including pre-treatment PSA within 6 months of randomisation)<br>✓ Cardiac: ECG, BP<br>✓ Imaging: Bone scan, CT or MRI pelvis and abdomen, CXR if chest not included in CT
BASELINE<br><br>(+/-4 WEEKS FROM DATE OF RANDOMISATION)	✓ Bloods: PO_4, Mg^{2+}, Albumin, cCa^{2+}, testosterone pre-ADT if available, PSA<br>✓ Fasting bloods: Glucose*, triglycerides*<br>✓ Fasting or non-fasting bloods: Lipid profile*<br>✓ Waist circumference<br>✓ Weight and height<br>✓ Blood collection (cell free DNA Streck™ tubes)<br>✓ Saliva sample

Key: FBC= Full blood count, LFT= liver function test, U&E= Urea and electrolytes, GFR=glomerular filtration rate, BP= blood pressure, PO_4=phosphate, cCa^{2+}=corrected calcium.
* Not required for patients allocated to research Arm L: transdermal oestradiol.

4.5.1 Investigation Prior to Registration (for patients participating in biomarker-screening pilot)

Sufficient screening investigations must have been completed to ensure that patient fulfil all of the selection criteria for the biomarker-screening pilot, see Section 4.3.1 prior to registration. In addition, confirmation of adequate organ function should be obtained through baseline bloods which at a minimum include FBC, U&Es and LFTs. Metastatic disease should be confirmed radiologically but all the required screening scans may not have been completed. All remaining screening investigations e.g. additional blood and imaging, may be completed following registration prior to randomisation.

4.5.2 Investigations Prior To Randomisation

All patients should have the following examinations performed to confirm eligibility prior to randomisation.

The following standard imaging is required and the latest available scans that reflect the patient's current disease status should be used:

- CT or MRI of pelvis and abdomen
- Bone Scan (or equivalent e.g. whole body MRI, choline-PET-CT, PSMA-CT-PET)

- Chest X-ray (only if chest was not included in CT or MRI which would be preferable)

Any additional imaging such as CT-PET scanning can be performed according to local practice but, for the purposes of the trial, the recorded stage should be the CT stage only; additional information on the CT-PET stage will also be collected.

The following bloods and additional measurements are required prior to randomisation:
- ECG
- PSA Test within 6 months (please supply the last pre-hormone therapy value)
- HbA1c
- Full blood count
- Urea and Electrolytes
- Liver function tests
- Serum creatinine
- Systolic and diastolic blood pressure

Patients who initially fail to meet the trial eligibility criteria can be re-screened at a later date. Of note, for patients receiving standard-of-care docetaxel at the point of screening, it is acceptable to use a full blood count measurement prior to chemotherapy to confirm eligibility.

Prior to randomisation:
- Check details of any prior treatments for prostate cancer
- Check any contraindications to radiotherapy or research treatment
- Check concomitant medications

4.5.3 Additional Baseline Investigations

The following blood tests are required at baseline (within 4 weeks before or after randomisation):
- Testosterone (pre-ADT, if available)
- Serum corrected calcium
- Phosphate
- Magnesium
- Albumin
- Fasting glucose (mandatory)
- Fasting triglycerides (mandatory)
- Lipid profile (fasting or non-fasting; total cholesterol, LDL and HDL) (mandatory)

The following additional procedures are required and **mandatory** at baseline:
- Waist circumference measurement
- Weight and height

A trial screening log will be *available* to all centres; copies are *not required* at this time (unless a specific issue is identified at a given site by the STAMPEDE Trial Team).

4.5.4 Androgen Deprivation Therapy Prior To Randomisation

From Protocol version 16.0, patients can potentially be randomised to the “transdermal oestradiol comparison” and it would be preferable for these patients to have had as little exposure to ADT as possible.

Within the separate PATCH trial, patients are randomised within 8 weeks of starting anti-androgens and cannot have received an LHRH injection. This approach is also favoured in STAMPEDE, but

patients who have received a single 4-week (or 1-month) LHRH injection remain eligible for randomisation to the "transdermal oestradiol comparison", as shown in Figure 3.

For all other comparisons, if ADT has already started prior to randomisation, the first LHRH injection must have been given within 12 weeks prior to randomisation. Additionally, if anti-androgens are being used, these must have started within 14 weeks prior to randomisation. A PSA level must have been taken prior to starting long-term hormone treatment. Note that baseline testosterone measurements will not be required in patients who have already commenced hormone manipulation prior to randomisation.

Figure 3 illustrates the maximum duration of ADT allowed pre-randomisation.

Note that anti-androgen monotherapy is not permitted as a form of long-term hormone therapy. It is accepted that sites participating in the Biomarker-Screening Pilot are unlikely to meet the "preferred timeline for randomisation".

Figure 3: Maximum duration of hormone therapy allowed prior to randomisation and the latest point of randomisation

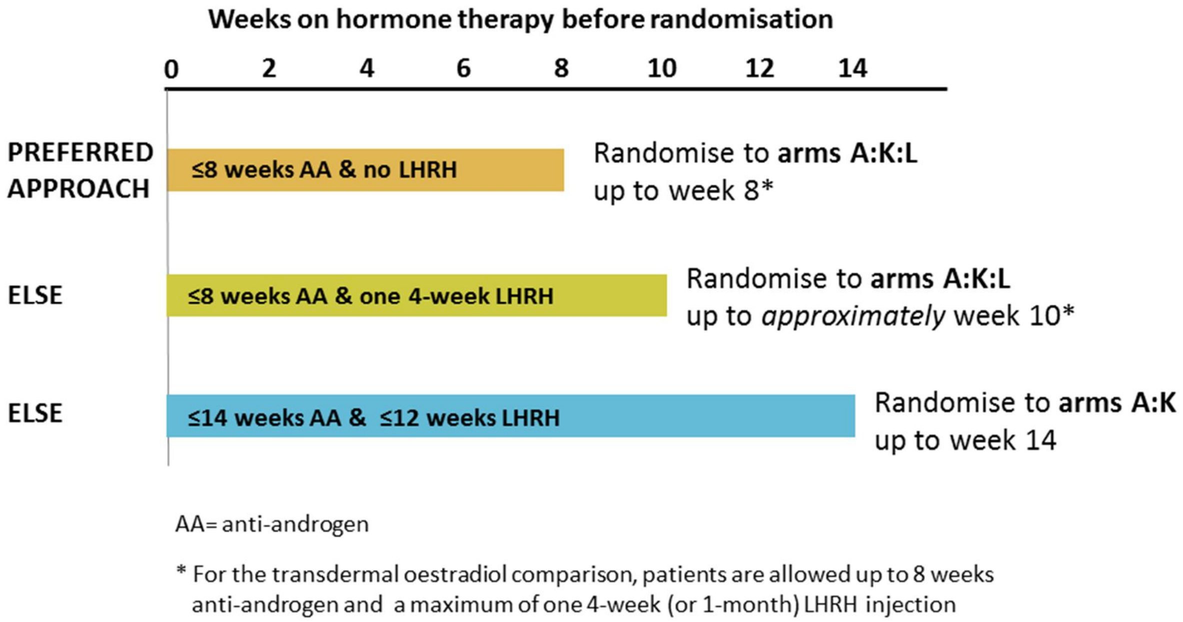

Permitted prior hormone therapy for now-relapsing disease: Any patients now presenting with relapsed disease, previously treated with adjuvant or neo-adjuvant hormone therapy alongside their radical surgery or radiotherapy, must have completed that period of hormone therapy **at least 12 months** before joining STAMPEDE and it must have been **no longer than 12 months in duration**.

4.5.5 Standard-Of-Care (SOC) Radiotherapy

The treating clinician and patient must have decided, prior to randomisation, whether radiotherapy is to be given as part of standard-of-care (SOC).

4.5.6 Standard-Of-Care (SOC) Docetaxel

The treating clinician and patient must have decided, prior to randomisation, whether docetaxel is to be given as part of standard-of-care (SOC). SOC docetaxel treatment should start within 14 weeks after starting hormone therapy (ideal timeline: within 8-10 weeks). Patients can have already started docetaxel treatment when randomised providing this is within 14 weeks after starting hormone therapy.

For those planned for SOC docetaxel and subsequently randomised to receive transdermal oestradiol (Arm L), it is recommended that docetaxel treatment commences *after* patients have been establised on transdermal oestradiol for around 4 weeks, when most patients are likely to have completed the induction period (see **Section 6.2.8**).

4.5.7 Starting Trial Treatment

4.5.7.A Metformin

For all patients allocated to metformin, treatment should start as soon as possible after randomisation. Investigators should aim that this is at least within 4 weeks post-randomisation and within 12 weeks of starting hormone therapy (see **Section 6.2.7**). Metformin can be given in combination with SOC docetaxel.

4.5.7.B Transdermal Oestradiol

For all patients allocated to transdermal oestradiol, treatment should start as soon as possible after randomisation (and ideally within 1 week after randomisation). It is not necessary to wait for completion of the 4-week (or 1-month) duration of the LHRH injection, if this was previously given prior to randomisation. For those prescribed bicalutamide or flutamide prior to randomisation, this treatment should be discontinued before treatment with transdermal oestradiol can commence (no washout period is needed).

4.5.8 Concomitant Medications

From Protocol version 15.0 onwards the trial requires the reporting to CTU of information regarding planned or actual long-term (>6 months) use of the following concomitant medications of classes of interest.

- Statins
- Metformin
- Aspirin
- Bisphosphonates or denosumab
- Opiate pain killers
- ACE inhibitors or angiotension II antagonists

This information is of interest both in terms of baseline use and ongoing use through the trial; as such it should be recorded on the Randomisation CRF and will be collected at each follow-up assessment (see **Table 18**).

Caution should be exercised when starting any concomitant medications that may result in a worsening of renal function e.g. initiating anti-hypertensive therapies such as ACE inhibitors, diuretics such as frusemide, or starting a non-steroidal anti-inflammatory drug (NSAID). Please refer to **Table 17** for more information on drugs which may require additional monitoring of renal functions.

All concomitant medications should be continued throughout the trial unless the responsible clinician decides otherwise. If patients continue to require medication for the management of docetaxel-related toxicities, please discuss this with the trial team. See **Section 6.3** for more information on concomitant medications and their use with abiraterone and enzalutamide.

4.6 ADDITIONAL DETAILS FOR PATIENTS JOINING SUB-STUDIES

All patients joining STAMPEDE are asked for additional, optional consent to provide samples for three ongoing sub-studies. Since Mar-2016, all consent for additional research is provided on a separate consent form.

For details regarding sample collection, please refer to the **Sample collection and handling manual**.

4.6.1 Germline DNA Analysis (Saliva Samples)

In collaboration with Professor Ros Eeles, Institute of Cancer Research, London, DNA is being extracted from saliva samples provided by consenting participants enrolled in STAMPEDE.
The aims of this sub-study are to examine the germline (inherited) genetic changes present in men with high-risk localised or metastatic prostate cancer. The aim is to determine the prevalance of germline genetic aberrations present pre-diagnosis and to correlate prostate cancer risk SNP genetic profiles, identified in Genome-wide Association Studies (GWAS) and other sequence variants from next generation sequencing (NGS), with duration of response to ADT and the experimental treatments tested in STAMPEDE.

All patients joining the trial are asked to consent to provide a saliva sample from which germline DNA can be extracted. This has been the case since Protocol version 15.0 and replaces the blood spot collection method used in previous versions of the protocol. Preliminary data has shown saliva to be a feasible method to collect sufficient DNA to conduct analyses of germline (inherited) genetic changes.

All patients who consent to part A (donation of saliva) on the Additional Research Consent Form version 1.0 onwards are eligible for this sub-study. Saliva samples should be provided after randomisation and all consenting patients from all arms can participate. See the **Sample collection and handling manual** for further details.

Patients who previously joined the trial prior to Protocol version 13.0 and who consented to provide a blood spot (Consent Form version 4.0 part K) can also be retrospectively approached to provide a saliva sample. These should be collected from patients randomised to the trial from Nov-2011 onwards (when recruitment to the abiraterone comparison was activated) who have received the REC approved letter explaining the need for additional saliva sample collection as the DNA extraction using the blood spot method did not work as well as anticipated.

4.6.2 Circulating Tumour-DNA Analysis (Sequential Blood Samples)

This sub-study is being conducted in collaboration with Dr Gerhardt Attard, Institute of Cancer Research, London. The aims of this analysis include to identify molecular subgroups with differential treatment effects and, through sequential sampling, identify molecular changes associated with disease progression to explore resistance mechanisms and early detection of treatment failure.

From Protocol version 14.0 (activated from Jan-2014 onwards), sequential blood samples were collected from patients within the “enzaluatmide and abiraterone” comparison, i.e. allocated to arm A or J between 29-Jul-2014 and 31-Mar-2016. From Protocol version 16 onwards, **all patients** joining

the trial will be asked to donate sequential blood samples from which genetic material shed by the tumour cells can be extracted, enabling tumour DNA analysis.

The sampling schedule is different for M0 and M1 patients and is detailed in the **Sample collection and handling manual**. Sequential samples are required in order to detect genetic changes within tumours over time. The most important sampling timepoint is at progression, as it is hoped this can inform the potential mechanisms of treatment resistance. From Protocol version 16.0 the sampling schedule has been updated and now includes a baseline sample, obtained as soon as possible after consent is provided. The aim of this additional sampling point is to explore the genetic changes initially present i.e. at the point at which treatment is first started.

From Protocol version 16.0 onwards, all patients allocated to all arms should be asked if they are willing to provide additional consent to participate in this sub-study which is recorded on part B of the Additional Research Consent Form.

4.6.3 Tissue Sample Analysis (FFPE Blocks)

As the clinical outcome data matures for several of the treatments comparisons evaluated within STAMPEDE, correlative analysis of the archival formalin-fixed paraffin-embedded (FFPE) tumour tissue will be undertaken, aiming to identify predictive and prognostic biomarkers. Targeted next-generation sequencing (tNGS) of FFPE tumour samples from selected, consenting STAMPEDE patients will be performed in order to explore the prevalance of genomic abberations and examine the predictive and prognostic effect of molecular sub-groups. FFPE blocks are currently being collected at selected STAMPEDE sites to support different projects.

All patients joining the trial have been asked to consent for the use of remaining tissue samples e.g. those obtained at prostate biopsy or following surgery, for use in additional research. These samples are usually stored as FFPE tissue blocks at the hospital where the procedure was performed. Randomising sites will be asked to assist in the retrieval of tissue samples stored in pathology stores or referring hospitals when these are required for additional translational sub-studies.

All patients who consent to part C on the additional research consent form are eligible for ongoing sub-studies involving FFPE tumour block analysis. For patients who previously joined the trial, consent for use of remaining samples was provided in part L of the informed consent form. Research teams at randomising sites will be required to provide an anonymised copy of the consent form required to request samples from pathology departments and facilitate the transfer of samples to the trial designated laboratories. Prior to FFPE sample transfer, a material transfer agreement is required to be in place between the sponsor and the site. Further details on the sample processing and transfer can be found in the **Sample collection and handling manual.**

4.6.4 Biomarker-screening pilot

Within Protocol version 16.0, a Biomarker-Screening Pilot will be activated in selected sites. Patients participating in biomarker-screening will be required to register prior to randomisation, see **Section 4.3** for details on the eligibility criteria for registration and see **Section 5** for details on registration and randomisation.

At the point of registration three samples are required to complete the biomarker-screening.

- Expedited retrieval of FFPE tumour block
- Baseline blood sample collected using cell free DNA Streck™ tubes
- Saliva sample

Please refer to the **Sample collection and handling manual** for details.

The aim of this pilot is to assess the feasibility of rapid pre-randomisation biomarker-screening which will be required for planned future biomarker-selected comparisons. The first biomarker-selected comparison has successsfully received independent peer-review through CRUK and is in the late stages of development.

When recruitment is activated to biomarker-selected comparisons, the results of biomarker-screening will be required prior to randomisation and will determine eligibility. The screening pilot will activate before randomisation to biomarker-selected comparisons and inform this process. Therefore patients who participate in the pilot will continue to be allocated to any of the current open arms for which they are eligible (arm A, L and K).

4.6.5 Informed Consent For Genetic Screening

For patients joining the trial from Protocol version 16.0 onwards, the consent processes has been updated. Trial participants will now be asked to provide explict informed consent if they wish to receive feedback of any results that arise from research analyses of genetic material extracted from any of the biological samples collected as part of the trial e.g. saliva, FFPE tumour blocks or circulating tumour DNA extracted from blood.

Only results which are of established clinical relevance and for which testing would be available under standard NHS genetic testing guidelines will be fed back e.g. pathogenic BRCA1/2 mutations. Any genetic analysis undertaken as part of additional research associated with STAMPEDE does not replace clinically indicated investigations as only a proportion of STAMPEDE patient will undergo prospective testing and therefore it cannot be guaranteed that results will be fed back in a timely fashion.

STAMPEDE investigators are recommended to refer all patients in whom a clinically relevant genetic result is detected during research analyses to a clinical geneticist. This is to facilitate access to genetic couselling and the required confirmatory testing, this is also necessary in order to offer appropriate advice to biological relatives in the event of detecting a germline (inherited) genetic abnormality . The list of clinically relevant gene mutations to be fed back will be based on current clinical guidelines. The STAMPEDE Biological Research Group will review this periodically to ensure it remains current and oversee this process.

In the information provided to STAMPEDE patients who joined the trial prior to Protocol version 16.0, it was stated that any subsequent genetic results would not be linked to them or their families and therefore results will not be provided in this instance. Going forward, the changes incorporated in Protocol version 16.0 will enable patients who may undergo analyses on genetic material extracted from FFPE tissue, saliva or circulating tumour DNA (extracted from blood) to opt to receive clinically relevant information.

5 REGISTRATION AND RANDOMISATION

5.1 TRIAL ENROLMENT: DEFINITIONS AND PROCESS

5.1.1 Registration

Currently, registration is only required for patients participating in the Biomarker-Screening Pilot. This is initially being activated in a proportion of STAMPEDE sites.

For sites participating in the biomarker-screening pilot, all patients who are eligible to participate in the Biomarker-Screening Pilot must be registered prior to randomisation. Once registered to participate in the pilot, patients can currently be randomised without waiting for the results of the biomarker-screening providing all the required screening information is known.

See **Section 4.3.1** for details on selection criteria, please confirm all criteria are met and complete the Registration CRF prior to contacting the STAMPEDE trial team at the MRC CTU.

All participants in the biomarker-screening pilot will be allocated a registration number which relates specifically to the biomarker-screening process. The registration number will be used to identify the patient until the point of randomisation when this will be linked and replaced by the trial number.

Once recruitment is activated to biomarker-selected comparisons biomarker-screening will be implemented. Registration will be then required for all patients participating in biomarker-screening and patients will only be eligible for randomisation once the results of biomarker-screening are known.

5.1.2 Randomisation

All other patients **not** participating in Biomarker-Screening may proceed immediately to randomisation. Eligibility will be confirmed during the randomisation process and patients will be allocated to any of the open research arms for which they are suitable (see **Section 4.4**).

To enter a patient into STAMPEDE (either to register or randomise); the relevant forms should be completed carefully, and the CTU contacted by phone:

REGISTRATION & RANDOMISATIONS
Call the MRC CTU at UCL, Monday to Friday 0900-1700
Excluding public holidays or dates when notice has been given by the CTU.
Tel: +44 (0) 20 7670 4777

A trial number and treatment will be allocated and given over the phone or by return fax. In addition, a letter confirming these details will be sent. The trial number will be the primary way in which the patient will be identified and should be used in all correspondence. Centres should send a letter to the patient's GP to inform them of their trial participation and treatment allocation. The GP letter is supplied as a template and can be downloaded from the trial website www.stampedetrial.org.

5.2 CO-ENROLMENT GUIDELINES

Ideally, patients should not be participating in any other clinical trial of prostate cancer treatment when they enter STAMPEDE and should not enter any other trials until a failure-free survival (FFS) event has been experienced and reported. After this point, the patient may be entered into further, second-line treatment studies. The primary outcome measure of STAMPEDE is overall survival. Participation in post-progression studies must be reported on the Co-enrolment CRF; details of any interventional treatments received for progression in such studies should be reported on the Additional Treatment Log.

Data sharing agreements with "down-stream" trials are encouraged to improve data quality in both trials and to reduce costs to both organisations.

6 TREATMENT OF PATIENTS

6.1 STANDARD-OF-CARE (SOC)

The standard-of-care for this patient group is **androgen deprivation therapy** as per local practice (see Section 6.1.1). For some patient groups, this should now be supplemented with standard radiotherapy (see Section 6.1.2). From Protocol version 14.0 onwards the standard-of-care includes permitted use of docetaxel for all suitable patients (see Section 6.1.3).

In summary, SOC treatment is defined as being **one** of the following combinations:

- ADT alone
- ADT + Radiotherapy
- ADT + Docetaxel
- ADT + Radiotherapy + Docetaxel

6.1.1 Hormone Therapy

Patients will be randomised either to the control arm (Arm A) or to one of the actively recruiting research arms for which the patient is eligible.

With the exception of those allocated to transdermal oestradiol (Arm L), all patients will receive ADT as per local practice to achieve castrate levels of testosterone. Please see Section 4.5.4 for more information on ADT timing before randomisation.

Patients allocated to Arm L will go on to receive transdermal oestradiol in place of standard ADT methods.

The method of planned or current long-term standard-of-care ADT must be specified for each patient prior to randomisation.

The permitted methods of ADT are:

6.1.1.A Bilateral Orchidectomy

Operations should be performed by appropriately trained surgeons. A total or sub-capsular orchidectomy may be performed. Patients having a bilateral orchidectomy are required to adhere to the same timelines as specified in Section 4.5.4 unless there is a strong clinical reason not to do so.

6.1.1.B LHRH Agonists

LHRH agonists used according to local practice. The prophylactic use of anti-androgens to prevent tumour “flare” is recommended.

6.1.1.C LHRH Antagonists

LHRH antagonists used according to local practice. The use of prophylactic use of anti-androgens to prevent tumour “flare” is not necessary.

6.1.1.D Dual Androgen Blockade

Long-term use of anti-androgens alongside LHRH agonists, according to local practice. Note this was previously referred to as maximum androgen blockade.

6.1.1.E Others

Other methods of ADT should be discussed with the STAMPEDE trial team. The planned duration of ADT should be at least 2 years.

6.1.2 Standard-Of-Care (M0) RT

6.1.2.A N0M0 Patients

Investigators should give standard radiotherapy (RT) to patients with node negative, non-metastatic disease (N0M0), in accordance with data from the PR07 and SPCG trials. If there is an intention to omit radiotherapy (e.g. RT is contraindicated for the patient) in patients with N0M0 disease this must be discussed with the STAMPEDE trial team before randomisation to confirm eligibility. See Section 6.7 for further details of radiotherapy administration.

6.1.2.B N+M0 Patients

The benefit of radiotherapy in this group is at present uncertain with no firm data to either support or refute its use. However, the PR07 trial included some node-positive patients as cross sectional imaging was not a part of the baseline assessment in this trial, which did include whole pelvis radiotherapy (9). For patients with node-positive, non-metastatic disease, radiotherapy is therefore recommended in suitable cases (10).

6.1.2.C Planned use of SOC RT

Investigators will be asked to state their intention with regards to planned radiotherapy in this group at randomisation. Intention to give radiotherapy (or not) for **all** patients must be stated at randomisation to ensure that there is no bias towards particular combinations of systemic therapy with radiotherapy.

Standard-of-care radiotherapy is not a core part of the trial, therefore we intend to collect minimal data about the radiotherapy administered. It is accepted that some patients will develop progressive disease before radiotherapy can be administered and if this occurs the reasons for non-delivery of treatment must be recorded on the Radiotherapy Detail CRF.

Suitability for radiotherapy is assessed by the treating clinicians. Any patient who has had a previous, definite diagnosis of inflammatory bowel disease is at increased risk of disease re-activation following radiotherapy, and the risks of this must be balanced against the potential benefits of radiotherapy on an individual basis.

6.1.3 Standard-Of-Care Docetaxel

Investigators are strongly encouraged to consider giving docetaxel as part of the standard-of-care for patients with newly-diagnosed metastatic disease, based on the survival benefit demonstrated by both STAMPEDE in the primary analysis of the "original comparisons" and CHAARTED (11) (12, 13). Investigators may also consider giving docetaxel to patients with high-risk locally-advanced disease, given both the significant improvement in failure-free-survival and consistency of effect for prostate cancer-specific survival shown by STAMPEDE.

The treating clinician and patient must have decided, prior to randomisation, if docetaxel is to be given. Chemotherapy treatment may have started when the patient is randomised. For patients allocated to receive transdermal oestradiol (Arm L) who have not already started docetaxel prior to randomisation, it is recommended that docetaxel commences around 4 weeks after starting research treatment (see Section 6.2.8). As with standard radiotherapy, minimum data collection will be required, however the start and end dates of docetaxel treatment are needed to ensure the appropriate timelines are met (see Section 4.5.6). A SOC Docetaxel Treatment CRF should be

completed for all patients confirming whether docetaxel was given or not, regardless of being planned.

Docetaxel is given according to local protocols as a standard non-trial treatment. The regime used previously within STAMPEDE was 75mg/m^2 Day 1 as 1hr IV infusion, plus prednisolone 5mg BID for 21 days repeated every 3 weeks for a maximum of 6 cycles. The STAMPEDE TMG would suggest prednisolone could be omitted and data on the use of co-prescribed steroid will be collected on the SOC Docetaxel Treatment CRF (please see Table 20 for more details).

6.2 RESEARCH TREATMENTS

6.2.1 Required Timelines When Starting Research Treatment

Allocated treatment should start promptly after randomisation. Please refer to Section 4.5.7 for more information on starting of research treatment.

6.2.2 Research Abiraterone + Prednisolone (arm G)

Note: recruitment has now closed to all research comparisons containing abiraterone; that is Arm G (SOC + abiraterone) and Arm J (SOC + enzalutamide + abiraterone).

Please note that for some patients treatment with abiraterone may continue until all categories of disease progression or up to a maximum duration of 2 years.

Arm G (SOC + abiraterone) patients who have now reached their maximum duration of 2 years on trial treatment include:

- All N0M0 patients
- All N+M0 patients receiving radical radiotherapy

Arm J (SOC + enzalutamide + abiraterone) patients who have now reached their maximum duration of 2 years on trial treatment include:

- N0M0 patients starting treatment over 2 years ago
- N+M0 patients receiving radical radiotherapy and starting treatment over 2 years ago

All such patients should have reported permanent stopping of research abiraterone on an End of Research Treatment CRF.

Please see sections below for more information.

Abiraterone will be administered as a single 1000mg daily oral dose (4 tablets to be taken together once a day) together with prednisolone or prednisone 5mg daily to prevent secondary mineralocorticoid excess. Abiraterone absorption is increased by food. The tablets should be taken at least 2 hours after food, swallowed whole with some water. No food should be eaten for 1 hour afterwards.

Prednisolone (prednisone in Switzerland) should be taken as a single dose with food in the morning.

Trial treatment must stop if other systemic treatments are initiated at any time for disease progression control (including chemotherapy, radium-223 etc).

Anti-androgens (e.g.. bicalutamide) should not be given in combination with abiraterone due to the risk of toxicity; as such patients on, or planned for MAB, at randomisation should not continue with their anti-androgen use if allocated to receive abiraterone, additionally anti-androgens started whilst on abiraterone treatment should trigger abiraterone to be stopped.
In patients with **M1 disease**, treatment with abiraterone will continue from randomisation until all categories of disease progression have occurred, consistent with the COU-AA-301 and COU-AA-302 trials (47, 48) i.e., abiraterone would be given for these patients until a composite of:

- PSA progression (as defined in **Section 7.1.3.A**)
- Radiological progression (appearance of new lesions or progression of existing lesions) **and**
- Clinical progression (defined as new cancer-related symptoms)

It is accepted that these flexible criteria for stopping treatment with abiraterone are open to the investigator's interpretation and discretion. Patients might continue treatment beyond the first failure-free survival (FFS) event; the first FFS event must be reported as per the other arms; all categories of disease progression (PSA, radiological and clinical) need to be reported once.

In patients **with N0M0 disease or N+M0 disease undergoing radical radiotherapy**, treatment would continue until the earliest of 2 years or all categories of disease progression as defined for M1 patients. ADT can be discontinued in this group at 2 years at the discretion of the local investigator (see **Section 6.1.1**).

For patients with **N+M0 disease not planned for radical radiotherapy**, or who do not receive planned prostate RT, treatment will continue as for patients with M1 disease until all categories of disease progression.

If a patient allocated to receive abiraterone develops only biochemical failure, the responsible clinician might switch from abiraterone + prednisolone 5mg od to abiraterone and dexamethasone 0.5mg od.

See **Section 7.1.3** for further information on the trial definition of progression.

6.2.3 Abiraterone + Prednisolone: Administration, Dose Modification And Management Of Toxicities

Abiraterone absorption is increased by food. The tablets should be taken at least 2 hours after food, swallowed whole with some water. No food should be eaten for 1 hour afterwards.

Prednisolone (prednisone in Switzerland) should be taken as a single dose with food in the morning.

6.2.3.A Abiraterone Contraindications

- Unusual or allergic reaction to past abiraterone acetate treatment
- Uncontrolled hypertension
- Uncontrolled heart failure
- Abnormal liver function or active or chronic liver disease

6.2.3.B Abiraterone Special Warnings And Precautions For Use

:: Timing of administration compared with meals
Administration of abiraterone acetate with food significantly increased the absorption of abiraterone acetate, it is therefore recommended that abiraterone acetate is taken on an empty stomach.

:: Cardiovascular history
Abiraterone acetate should be used with caution in patients with a history of cardiovascular disease. The safety of abiraterone acetate in patients with left ventricular ejection fraction <50% or New York Heart Association (NYHA) Class III or IV heart failure has not been established. Before treatment with abiraterone acetate, hypertension must be controlled and hypokalemia must be corrected.

Caution is required in treating patients whose underlying medical conditions might be compromised by increases in blood pressure, hypokalemia, or fluid retention, e.g. those with heart failure, recent myocardial infarction, or ventricular arrhythmia.

:: Blood pressure management
Blood pressure should be monitored **every 2 weeks until week 12** and **monthly after week 12** whilst the patients remain on treatment. For the management of abiraterone induced hypertension see **Table 6.**

:: Hepatic Impairment
No dosage adjustment was necessary for patients with pre-existing mild hepatic impairment. Abiraterone acetate should not be used in patients with pre-existing moderate or severe hepatic impairment.

:: Hepatotoxicity
Marked increases in liver enzymes leading to drug discontinuation or dosage modification occurred in controlled clinical studies. Serum transaminase and bilirubin levels should be measured prior to starting treatment with abiraterone acetate**, every 2 weeks for the first 3 months of treatment,** and **monthly thereafter.**

If clinical symptoms or signs suggestive of hepatotoxicity develop, serum transaminases, in particular serum alanine aminotransferase (ALT), should be measured immediately. See **Table 8** for the management of abiraterone induced hepatotoxicty.

:: Renal Impairment
No dose adjustment are required in renal impairement; however caution is advised if patients develop severe renal impairment as there is limited clinical data in this population. Systemic exposure to abiraterone after a single oral 1000mg dose did not increase in patients with end-stage renal disease on dialysis.

6.2.3.C Abiraterone Undesirable Effects

The most common adverse drug reactions observed in the integrated safety data for those patients who received 1000mg abiraterone acetate plus prednisone or prednisolone in clinical studies (n=1,070) were fatigue, arthralgia, peripheral oedema, back pain, bone pain, nausea, constipation, hypokalemia and anaemia.

The adverse events graded as 3 or 4 and which occurred in more than 5% of patients were fatigue, peripheral oedema, anaemia and back pain see **Appendix C.**

6.2.3.D Abiraterone Overdose

There have been no reports of overdose of abiraterone acetate during clinical studies. There is no specific antidote to abiraterone acetate. In the event of an overdose, administration of abiraterone acetate should be stopped and general supportive measures undertaken, including monitoring for cardiac arrhythmias. Liver function should also be assessed.

6.2.3.E Management Of Specific Toxicities From Prednisolone

Prednisolone/prednisone will be started at 5mg once daily, to prevent secondary mineralocorticoid excess.

Prednisolone/prednisone dose increase of up to 5mg BID is permitted to manage mineralocorticoid-related toxicities (e.g., hypokalaemia, hypertension) which are refractory to standard management.

Patients experiencing serious symptoms of cushings syndrome (e.g., weight gain, muscle loss) can decrease or discontinue (temporarily or permanently) steroids at the investigator's discretion but should be closely monitored for symptoms of secondary mineralocorticoid excess. It should be noted that weight gain and muscle loss are also associated with ADT.

Table 6: Management of hypertension associated with abiraterone (given alone or in combination with enzalutamide)

TOXICITY EVENT	ACTION
Grade 1-2	Management as per investigator with anti-hypertensive treatment and increase frequency of blood pressure monitoring to at least weekly. Follow local guidance for selection of anti-hypertensives but avoid thiazide diuretics to minimise risk of serum potassium derrangment. Calcium channel antagonists or beta blockers are often preferred.<br>As with other symptoms of minerlocorticoid excess, consider increasing prednisolone dose to 5mg BID.
Grade 3-4	Withhold abiraterone and enzalutamide. Adjust or add anti-hypertensive medications to mitigate the toxicity. When hypertension resolves to Grade ≤1, resume both enzalutamide and abiraterone at full dose with prednisolone 5mg bid.

A cardiologist's opinion should be considered if blood pressure control is not achieved within 4 weeks.

Table 7: Management of hypokalaemia associated with abiraterone (given alone or in combination with enzalutamide)

TOXICITY EVENT	ACTION
Grade 1<br>(LLN- 3.0mmol/L)	Supplement with oral potassium and monitor closely and increase prednisolone dose to 5mg BID.<br>Exclude and manage other causes of hypokalemia.
Grade 2<br>(<LLN – 3.0mmol/L and symptomatic)	Pause abiraterone.<br>Supplement with oral potassium and monitor closely and increase prednisolone dose to 5mg BID.<br>Exclude and manage other causes of hypokalemia.<br>Re-start abiraterone with close monitoring, discontinue if recurs
Grade 3<br>(<3.0–2.5mmol/L)<br><br>or life-threatening Grade 4 (<2.5mM)	Abiraterone will be permenently discontinued and the patients will be hospitalized for intravenous potassium replacement and cardiac monitoring. After the return of serum potassium to normal, prednisolone will be discontinued. The patient can continue on enzaluatamide alone. If hypokalaemia persists consider a dose reduction of enzalutamide to 120mg once a day.

Table 8: Management of Abnormal Liver Function Tests (LFTs) associated with abiraterone (given alone or in combination with enzalutamide)

TOXICITY EVENT	ACTION
**Grade 1** increases in AST, ALT or bilirubin (e.g. increase in AST or ALT from ULN to 2.5X ULN; increase in total bilirubin from ULN to 1.5X ULN)	The frequency of LFT monitoring should be increased to at least weekly, if the investigator judges that the laboratory abnormalities are potentially related to study medication.<br>No dose reduction is required.<br>Providing LFTs are stable for 4 weeks, resume monthly checks
**Grade 2** increases in AST, ALT or bilirubin (e.g. increase in AST or ALT to >2.5-5X ULN; increase in total bilirubin from >1.5-3X ULN)	Withhold abiraterone, enzalutamide and all other concomitant medications that are potentially hepatotoxic.<br>The frequency of LFT monitoring should be increased to at least weekly until the liver function tests return to baseline value or grade 1 when all trial medication can be re-started.<br>No dose reduction is required after one episode providing this resolved within 4 weeks but should be considered if Grade 2 derangements recurs.
**Grade 3** increases in AST, ALT or bilirubin (e.g. increase in AST or ALT to >5X ULN; increase in total bilirubin to >3X ULN),	Withhold abiraterone and enzalutamide and all other concomitant medications that are potentially hepatotoxic.<br>At least weekly monitoring is required until the LFTs return to baseline value or grade 1.<br>Enzaluatmide can be re-started with no dose reduction. See below for abiraterone re-challange.
**Grade 4** increases in AST, ALT or bilirubin (e.g. increase in AST or ALT to >20x ULN; increase in total bilirubin to >10x ULN)	Patients must discontinue abiraterone and enzalutamide immediately.<br>At least weekly monitoring is required until the LFTs return to baseline value or grade 1 and then prednisone can be discontinued and the investigator can consider restarting enzalutamide.<br>Abiraterone should not be re-introduced.
**RE-CHALLENGE**	**ACTION**
Recurrent G2 derangement	Reduce to 750mg once LFTs return to G1
If study treatment resumption is considered for patients who have experienced Grade 3 increases in AST, ALT, or bilirubin	Resume study treatment with abiraterone dose reduction to 750mg when grade 3 toxicities resolve to grade 1 or baseline.
If Grade 3 or higher increases in AST, ALT or bilirubin recur after the first dose reduction	Hold study medication and all other concomitant medications that are potentially hepatotoxic. At least weekly LFT monitoring is required, starting immediately regardless of study schedule and continued until a return to baseline values or Grade 1.
If study treatment resumption is considered for patients who have experienced Grade 3 increases in AST, ALT, or bilirubin with the first dose reduction	Resume study treatment with abiraterone dose reduction to 500mg when AST, ALT or bilirubin returns to baseline value or Grade 1.

An opinion from a hepatologist should be considered if there are any concerns or liver function derangement shows no improvement within 2 weeks of discontinuation of abiraterone.

Table 9: Management of fluid retention/oedema associated with abiraterone (given alone or in combination with enzalutamide)

TOXICITY EVENT	ACTION
Grade 1-2	Increase prednisolone dose to 5mg bid.
Grade 3-4	Withhold abiraterone.<br>Consider addition of mineralocorticoid receptor antagonist eplerenone until resolution of symptoms. Enzalutamide can be continued. When fluid retention/oedema returns to baseline or resolves to ≤Grade 1, resume abiraterone at full dose with prednisone 5mg bid, if symptoms do not resolve abiraterone should not be re-started and enzalutamide should be dose reduced to 120 mg per day.

Table 10: Management of diarrhoea (associated with abiraterone or enzalutamide)

TOXICITY EVENT	ACTION
Grade 1-2	Symptomatic management.
Grade 3-4	Withhold abiraterone.<br>If no improvement reduce dose of enzalutamide to 120 mg per day. Once resolved to Grade 1, recommence abiraterone at 750 mg per day.

6.2.4 Research Enzalutamide + Abiraterone + Prednisolone (Arm J)

Note: recruitment has now closed to Arm J (SOC + enzalutamide + abiraterone).

Please note that for some patients treatment with enzalutamide + abiraterone may continue until all categories of disease progression or up to a maximum duration of 2 years.

Arm J (SOC + enzalutamide + abiraterone) patients who have now reached their maximum duration of 2 years on trial treatment include:

- N0M0 patients starting treatment over 2 years ago
- N+M0 patients receiving radical radiotherapy and starting treatment over 2 years ago

Please see sections below for more information.

Abiraterone as described in Section 6.2.2.

Prednisolone/Prednisone as described in Section 6.2.2.

Enzalutamide will be administered as a 160mg oral dose (four capsules), taken together at the same time every day, with or without food.

Trial treatment must stop if other systemic treatments are initiated at any time for disease progression control (including chemotherapy, radium-223 etc).

Anti-androgens (i.e. bicalutamide) should not be given in combination with enzalutamide (as with abiraterone) due to the risk of toxicity; as such patients on, or planned for MAB, at randomisation should not continue with their anti-androgen use if allocated to receive enzalutamide + abiraterone, additionally anti-androgens started whilst on enzalutamide (+abiraterone) treatment should trigger enzalutamide (+abiraterone) to be stopped.

In patients with **M1 disease**, treatment with both abiraterone and enzalutamide will continue until all categories of progression have occurred, consistent with the approach taken for abiraterone (see Section 6.2.2) i.e., abiraterone and enzalutamide will be given until a composite of:

- PSA progression (as defined in Section 7.1.3.A)
- Radiological progression (appearance of new lesions or progression of existing lesions) **and**
- Clinical progression (defined as new cancer-related symptoms).

It is accepted that these flexible criteria for stopping treatment with abiraterone and enzalutamide are open to the investigator's interpretation and discretion. Patients may continue treatment beyond the first failure-free survival (FFS) event; the first FFS event must be reported as per the other arms.

In patients with **N0M0 disease or N+M0 disease undergoing radical radiotherapy**, treatment would continue for 2 years or all categories of disease progression as defined for M1 patients, whichever is the sooner. ADT can be discontinued in this group at 2 years at the discretion of the local investigator (see Section 6.1.1).

For patients with **N+M0 disease not planned for radical radiotherapy**, or who do not receive planned prostate RT, treatment will continue as for patients with M1 disease until all categories of disease progression.

If a patient develops PSA progression only whilst on abiraterone and enzalutamide, the local investigator might consider switching from from abiraterone + prednisolone 5mg od to abiraterone and dexamethasone 0.5mg OD.

See Section 7.1.3 for further information on the definition of progression.

6.2.5 Enzalutamide: Administration, Dose Modification And Management Of Toxicities

Enzalutamide can be taken with or without food.

6.2.5.A Enzalutamide Contraindications

The full induction potential of enzalutamide may not occur until approximately 1 month after the start of treatment, when steady-state plasma concentrations of enzalutamide are reached, although some induction effects may be apparent earlier. Monitoring should continue for at least the first month of treatment and dose adjustments considered. Given the long half-life of enzalutamide (5.8 days), effects on enzymes may persist for one month or longer after stopping enzalutamide. A gradual dose reduction of the concomitant medicinal product may be necessary when stopping enzalutamide treatment.

6.2.5.B Enzalutamide Special Warnings And Precautions For Use

:: History of seizures

Caution should be used in administering enzalutamide to patients with a history of seizures or other predisposing factors including, but not limited to, underlying brain injury, stroke, primary brain

tumours or brain metastases or alcoholism. In addition, the risk of seizure may be increased in patients receiving concomitant medications that may lower the seizure threshold. Enzalutamide should be permanently discontinued in patients who have a seizure while on treatment.

:: Hepatic impairment
A hepatic impairment study showed that the composite AUC of enzalutamide plus N-desmethyl enzalutamide after administration of a single dose of enzalutamide was similar in patients with baseline mild, moderate or severe hepatic impairment (Child-Pugh Class A, B or C respectively) relative to patients with normal hepatic function, and no starting dose adjustment is needed.

:: Renal impairment
No dose adjustment are required in renal impairment; however caution is advised if patients develop severe renal impairment as there is limited clinical data in this population.

6.2.5.C Enzalutamide Overdose
There is no antidote for enzalutamide. In the setting of an overdose, stop treatment with enzalutamide and initiate general supportive measures taking into consideration the $t^{1/2}$ of 5.8 days. Patients may be at increased risk of seizures following an overdose.

6.2.5.D Management Of Specific Toxicities Due To Abiraterone And Enzalutamide
The safety monitoring and toxicity management plan described below takes into account AEs based on the reported clinical safety data of abiraterone and enzalutamide given separately. There are limited reported data on the safety and toxicity of the combination of enzalutamide and abiraterone however the recommendations summarised here have been updated in light of the experience gained in STAMPEDE as recommended by the STAMPEDE TMG.

:: Seizures
If any patient suffers a seizure whilst on treatment, enzalutamide should be discontinued immediately. Abiraterone and prednisolone can be continued providing there are no abiraterone-specific toxicities.

Table 11: Management of arthralgia & muscle pain (associated with enzalutamide)

TOXICITY EVENT	ACTION
Grade 1-2	Symptomatic management
Grade 3-4	Reduce dose of enzalutamide to 120 mg per day

Table 12: Management of fatigue (associated to enzalutamide)

TOXICITY EVENT	ACTION
Grade 1-2	Consider a dose reduction to 120mg/day
Grade 3-4	Pause enzalutamide for 1 week or until the toxicity grade improves to grade 2 or lower severity. Re-started at a reduced dose (120mg/day or 80mg/day) in consultation with the study team.

6.2.6 Research Metformin (Arm K)
Metformin will be given as a daily dose in addition to standard-of-care treatment. The dose is 850mg OD. If tolerated, this should be increased to the target dose of 850mg BID after 4-6 weeks i.e. at the first follow-up visit.

In the case of **M0 patients**, if ADT is stopped after a minimum of 2 years, metformin should continue for a minimum of 3 years following randomisation and for a further 12 months after the administration of the last LHRH (whichever is longer). This is to allow for the delay in testosterone levels returning to normal following stopping ADT. If ADT is not stopped, then metformin should continue as it the case for M1 patients. In the event that ADT is stopped and then re-started for relapsed disease, if ADT is restarted whilst patients remain on metformin (i.e. within 12 months of the last administration of LHRH) then metformin should continue whilst on ADT. If metformin is stopped 12 months after the last administration of LHRH it should not be re-started in the event of relapse.

For **M1 patients** metformin should continue whilst on ADT. Treatment should continue post-progression providing it is judged to be in the patients best interest. Metformin can be given together with any additional treatments started for progression, excluding other IMPs i.e. investigators may choose to stop metformin treatment post-progression in order to enable patients to participate in another clinical trial evaluating treatments for CRPC.

In the event of stopping research treatment, unless a patient states otherwise, consent is assumed for continued recording trial data.

6.2.7 Metformin: Administration, Dose Modifications And Management Of Toxicities

The starting dose for metformin is 850mg once daily. If tolerated this should be increased to the target dose of 850mg twice daily after 4-6 weeks. Metformin should be taken around the same time each day and treatment tolerance is best if taken with or after food. For twice daily dosing, the minimum time between doses should be 8 hours, doses should not be taken closer together if forgotten or missed. If metformin is well tolerated and it is desirable to make a dose modification outside of the trial follow-up schedule, it is acceptable to conduct a telephone consultation. If metformin 850mg OD is not well tolerated, consider switching to 750mg SR OD or alternatively, reduce to 500mg OD.

6.2.7.A Metformin Special Warnings And Precautions For Use

:: Renal impairment
Metformin is not nephrotoxic, but is exclusively excreted by the kidneys. Therefore treatment should only be started in participants with stable renal function. From protocol version 16.0 the renal threshold has been revised in light of updated FDA guidance and published prescribing recommendations. Metformin should be only started when the GFR ≥45min/ml/1.73m^2. Renal function should be monitored **at least every 6 months** in participants with stable renal function. Additional monitoring is required in any patient at risk of deteriorating renal function (see Table 11). In line with published prescribing recommendations, if the GFR falls to between 30-45 min/ml/1.73m^2 a **dose reduction** is required to ensure the maximum 24hr dose is 1000mg or less and monitoring of renal function is required at least 3 monthly (49). Metformin should be **permanently stopped** if the GFR falls to ≤30ml/min/1.73m^2.

See Table 13 for situations when metformin treatment should be paused due to the risk of deterioration in renal function.

Table 13: Situations when metformin treatment should be paused

SITUATIONS	RISK FACTOR
Iodinated contrast agents	If the GFR<60 ml/min/1.73m^2 metformin should be paused for 24 hours prior to receiving contrast and re-started 48 hours post-administration.
Anaesthesia (peridural; spinal or general)	Pause metformin 48 hours prior to procedure and<br>re-start no earlier than 48 hours following procedure, providing oral intake re-established and renal function is stable and at baseline.
Surgery	Pause metformin 48 hours prior to procedure and<br>re-start no earlier than 48 hours following procedure, providing oral intake re-established and renal function is stable and at baseline.
Dehydration<br>e.g. nausea, vomiting or diarrhoea	Pause metformin and re-start only when oral intake is re-established and renal function is stable and at baseline.
Obstructive uropathy<br>e.g. urinary retention or ureteric obstruction	Pause metformin and re-start only when renal function confirmed to be stable and at baseline.

:: Treatment breaks
It is anticipated that metformin treatment will be paused for approximately 72 hours around the time of contrast enhanced CT scans (see Table 13) and may need to be paused during episodes of inter-current illness. If metformin is paused for 6 days or less this information does not need to be recorded and no additional action is needed. Treatment pauses of 7 days or more should be recorded by updating the Metformin Treatment Log.

If metformin treatment is paused for more than 2 weeks, investigators may consider re-starting at 850mg once daily for the first 4 weeks before escalating to full dose providing tolerance is acceptable. It is suggested that, providing patients have a sufficient supply of labelled IMP metformin tablets, a telephone consultation may be sufficient to assess tolerance and advice regarding dose modification in order to limit hospital visits.

If treatment is paused for more than 3 months or >50% of doses are missed for any reason the trial team should be informed as metformin may need to be discontinued.

6.2.7.B Management Of Specific Toxicities From Metformin

:: Gastrointestinal disturbance
Gastrointestinal disturbances are very common with metformin and include nausea, vomiting, diarrhoea, abdominal pain and loss of appetite. These are most common when first starting treatment (occur in >1/10 individuals).

If toxicities occur we recommend a dose reduction and/or a switch to a sustained release (SR) preparation if available (see Table 14).

Table 14: Management of metformin related gastrointestinal toxicity

TOXICITY EVENT	ACTION
Grade 1	• Ensure metformin is taken with or after food.<br>• Consider switching to 750 mg BID SR preparation if available.<br>Or, if unavailable consider:<br>• 1 week treatment pause, re-start at reduced dose 850mg once daily. Attempt an escalation after 1 month but if necessary, remain at 850mg OD.<br>And, if unable to tolerate 850mg OD or sustained release preparations are not available<br>• Consider dose reduction to 500mg OD (SR or IR if not available)
Grade 2<br>or higher	Reduce to 500mg OD SR (or IR if not available); re-attempt dose escalation after minimum of 1 week if symptoms improve, aiming to continue at the maximum tolerated dose<br>If grade 2 toxicity persists:<br>• Pause treatment for 2 weeks, and re-start at 850mg sustained release or if not available 500mg OD.<br>• And re-attempt a dose escalation 2 months later.<br>• And continue at the maximum tolerated dose providing symptoms ≤ grade 1.

If toxicities occur whilst on the initial starting dose of 850mg OD then the following dose modifications should be made:

- Switch to 750mg SR OD
- Alternatively, reduce to 500mg OD

If toxicities persist, consider a 1 week treatment pause, before re-starting at one of the dose-modified regimes and attempt an dose escalation after a minimum of 1 month.

Other possible metformin related toxicites included taste disturbance, skin reactions and B12 deficiency resulting in megaloblastic anaemia (see **Appendix C, Table 6**). Any patient who experiences anaemia whilst taking metformin should have haematinics including vitamin B12 measured and replaced if deficient.

:: Lactic acidosis
This is a very rare (3/100,000 patient years), but serious metabolic consequence. Reported cases have occurred primarily in diabetic patients with significant renal impairment who are also dehydrated. It is unclear whether this is due to the underlying diabetes or metformin. This is supported by a meta-analysis demonstrating similar rates of lactic acidosis in people with diabetes taking metformin compared with diabetic patients not taking metformin (50). This evidence suggests this side effect may be a complication of diabetes and may not be associated with metformin treatment. The risk factors for lactic acidosis are: renal impairment, prolonged fasting or malnutrition, excessive alcohol intake, hepatic insufficiency or any condition associated with hypoxia e.g. cardiac or respiratory failure or circulatory shock due to any cause.

The risk of lactic acidosis should be considered in the event of non-specific symptoms such as muscle cramps, abdominal pain and/or severe weakness or lethargy. Any patient with a suspected metabolic acidosis requires immediate discontinuation of metformin and evaluation. Lactic acidosis is characterised by metabolic acidosis (decreased blood pH, high lactate above 5mmol/L and an increased anion gap and lactate/pyruvate ratio). The most effective way to remove lactate and metformin is haemodialysis.

:: Metformin overdose
Hypoglycaemia has not been reported with metformin doses of up to 85g although lactic acidosis has occurred in such circumstances. Patients should be urgently assessed in the event of an overdose and hospital admission considered. The management of metformin overdoses should be as per standard clinical care by the local team. The most effective way to remove lactate and metformin is haemodialysis.

6.2.8 Research Transdermal Oestradiol (Arm L)

Patients randomised to receive transdermal oestradiol may also receive SOC radiotherapy (Section 6.1.2) and SOC docetaxel (Section 6.1.3) as clinically appropriate, as has been done in the PATCH trial. It is recommended that patients commence SOC docetaxel (where planned) after they have been on transdermal oestradiol for around 4 weeks when most will have completed the induction period; radiotherapy, if used, would follow later.

Transdermal oestradiol is delivered as Progynova TS 100mcg/24 hours transcutaneous oestradiol patches according to the following dose regimen which has been shown within the PATCH trial to be sufficient for achieving castrate levels of testosterone.

6.2.8.A Induction Regimen

Four Progynova TS 100 transdermal oestradiol patches to be changed twice weekly (e.g. Monday and Thursday) for four weeks. A confirmatory testosterone and oestradiol sample should be taken at 4 weeks with the sample drawn the **day before** the patches are changed.

6.2.8.B Maintenance Regimen

If the patient has achieved a testosterone value of ≤1.7nmol/L at 4 weeks then treatment is changed to a **maintenance regimen** of **three** patches changed twice weekly. The oestradiol level should also be monitored at the 4 week time point, with castrate levels of testosterone typically achieved with a plasma oestradiol level ≥500pmol/L.

If a patient's testosterone is >1.7nmol/L at four weeks then they should remain on the induction regimen for another 4 week period, with monitoring of testosterone and oestradiol samples taken at around the week 8 time point, the day before patches are changed. Once the patient achieves a castrate level of testosterone≤1.7nmol/L, they can be reduced to the maintenance regimen.

6.2.8.C Monitoring Hormone Levels

Oestradiol and testosterone levels should continue to be monitored throughout follow-up, while the patient remains on transdermal oestradiol treatment, to assess for evidence of compliance and to also ensure the patient is on the appropriate dose. See Section 7.1.2 and Table 18 for when these values are required, noting also that the samples can be taken at the same time as scheduled PSA measurements.
A repeat blood test should be carried out within 4 weeks if, at any time, the patient's oestradiol level is found to be <300pmol/L or >2000pmol/L, with particular attention paid to the day that the patches are changed compared to when the blood sample is drawn (should be the day before changing patches). If the patient continues to have out of range oestradiol levels, and/or persistent testosterone >1.7nmol/L, then a member of the CTU team should be contacted for advice.

If the maintenance patch dose is changed at any time (for example, reducing from 3 to 2 patches changed twice weekly), then additional oestradiol and testosterone tests are required around 4 weeks after dose modification.

6.2.9 Transdermal Oestradiol: Administration, Dose Modifications And Management Of Toxicities

Consecutive patches should be applied to different sites. It is recommended that patches are placed on dry, intact and hairless skin and on areas where little wrinkling occurs, at the following sites only:

- Shoulder girdle
- Back
- Upper arms
- Buttocks

Patches should not be placed on or near the breast area, or on areas of the body where there are large amounts of subcutaneous fat, particularly around the abdomen, as this could affect absorption. Please note that these recommendations are mainly based on studies in women using the patches.

To apply the patch, remove the protective liner and press on to the skin immediately, holding for at least 30 seconds to ensure proper adhesion. If necessary, tape can be used to fix the patch in place. If applied correctly, the patient can bath or shower as normal, however the patches might come off in very hot water or in a sauna.

Dermatitis can be a common side-effect of using the patches, especially in the induction period, which can usually be controlled by alternating the site of patch application. Patients should be advised that if patches become dislodged they should not put on extra patches, but apply their next set of patches when they are next due to be applied.

We expect patients to remain on the prescribed dose, and any potential dose modifications other than those indicated in Section 6.2.8 should be discussed with the CTU team.

If a patient has a cardiovascular event (see Section 7.1.4.B), discontinuation of treatment with transdermal oestradiol may be considered at the discretion of the treating clinician.

6.3 CONCOMITANT MEDICATIONS AND DRUG INTERACTION

6.3.1 Abiraterone: Interaction With Medicinal Products And Other Forms Of Interaction

Details on drug interactions are described in Appendix C. The table below provides a summary on the main interactions.

:: Anti-androgens.
Abiraterone is steroid synthesis inhibitor and should not be given together with any other anti-androgens given the risk of toxicity. Cyproterone acetate should be discontinued 10 days and finasteride stopped 48 hours before commencing abiraterone. Concomitant use of dutasteride, bicalutamide and flutamide are all contraindicated.

6.3.2 Enzalutamide: Interaction With Medicinal Products And Other Forms Of Interaction

Details on drug interactions are described in Appendix C. The table below provides a summary on the main interactions.

:: Anti-androgens
Enzalutamide is potent androgen receptor antagonist and should not be given together with any other anti-androgens given the risk of toxicity. Cyproterone acetate should be discontinued 10 days

and finasteride stopped 48 hours before commencing enzalutamide. Concommitent use of dutasteride, bicalutamide and flutamide are all contraindicated.

Table 15: Drugs which may interact with Abiraterone

DRUGS WHICH MAY INCREASE ABIRATERONE LEVELS			
**Substrate**	**Clinical Use**	**Drug**	**Recommendation**
CYP3A4 inhibitors	Macrolide antibiotics	Clarithromycin	Avoid or hold abiraterone if short term use unavoidable given increased risk of abiraterone toxicity
	Anti-fungals	Ketoconazole<br>Itraconazole<br>Voriconazole	Avoid or hold abiraterone if short term use unavoidable given increased risk of abiraterone toxicity
**DRUGS WHICH MAY REDUCE ABIRATERONE LEVELS**			
**Substrate**	**Clinical Use**	**Drug**	**Recommendation**
CYP3A4	Anti-epileptics*	Phenytoin<br>Carbamazepine<br>Phenobarbital<br>Primadone	Contraindicated
	Anti-depressants	St Johns Wart	Contraindicated
	Anti-TB	Rifampicin<br>Rifabutin	Contraindicated
	Anti-retroviral	Atazanavir<br>Saquinavir<br>Ritonavir<br>Indinavir<br>Nelfanavir	Contraindicated. Seek specialist advice and discuss with trial team
**DRUGS WHICH MAY ACCUMULATE WHEN GIVEN WITH ABIRATERONE**			
**Substrate**	**Clinical Use**	**Drug**	**Recommendation**
CYP2D6	Cardiac	Metoprolol<br>Propranolol<br>Propafenone<br>Flecanide	Monitoring required as drug levels may increase with abiraterone use
	Anti-depressants	Desipramine<br>Venlafaxine<br>Citalopram	Monitoring required as drug levels may increase with abiraterone use
	Anti-psychotics	Haloperidol<br>Risperidone	Monitoring required as drug levels may increase with abiraterone use
	Analgesia	Tramadol<br>Codeine<br>Oxycodone	Monitoring required as drug levels may increase with abiraterone use
	Alpha blockers	Tamsulosin	Monitoring required as drug levels may increase with abiraterone use
	Anti-diabetic	Repaglinide	Monitoring required as drug levels may increase with abiraterone use

Table 16: Drugs which may interact with Enzalutamide

DRUGS WHICH MAY INCREASE ENZALUTAMIDE LEVELS			
**Substrate**	**Clinical Use**	**Drug**	**Recommendation**
CYP2C8 inhibitors	Lipid-lowering	Gemfibrozil	Avoid, if no alternatives, reduce enzalutamide dose to 80mg
**DRUGS WHICH MAY DECREASE ENZALUTAMIDE LEVELS**			
**Substrate**	**Clinical Use**	**Drug**	**Recommendation**
CYP2C8 inducers	Anti-TB	Rifampicin<br>Rifabutin	Avoid and switch to an alternative if possible
CYP3A4 inducers	Anti-epileptics	Phenytoin<br>Carbamazepine<br>Phenobarbital	Contraindicated
	Anti-depressant	St Johns Wart	Contraindicated
	Anti-retrovirals	Atazanavir<br>Saquinavir<br>Ritonavir<br>Indinavir<br>Nelfanavir	Contraindicated. Seek specialist advice and discuss with trial team
**ENZALUTAMIDE MAY REDUCE DRUG LEVELS**			
**Substrate**	**Clinical Use**	**Drug**	**Recommendation**
CYP2C19	Gastric protection	Omeprazole	Omeprazole AUC reduced by 70% Consider increasing dose of omeprazole for same therapeutic effect
CYP3A4	Analgesia	Fentanyl*<br>Alfentanil*<br>Tramadol	Monitor closely and consider alteratives
	Immunosuppressants	Sirolimus*<br>Tacrolimus*<br>Cyclosporine*	Monitor closely
	Anti-migraine	Ergotamine	Monitor closely
	Cardiac	Nifedipine<br>Ivabradine	Monitor closely, consider alternatives as clinical effect may be reduced
CYP2C9	Anti-epileptics	Phenytoin*	Contraindicated
	Anti-coagulants	Warfarin*	Warfarin AUC reduced by 56% Consider switching to low molecular heparin, increase INR monitoring if this is not possible
**DRUGS WHICH MAY ACCUMULATE WHEN GIVEN WITH ENZALUTAMIDE**			
**Substrate**	**Clinical Use**	**Drug**	**Recommendation**
p-gp		Colchicine*<br>Dabigatran*<br>Digoxin*	Monitor closely

* narrow therapeutic index

6.3.3 Metformin: Interaction With Medicinal Products And Other Forms Of Interaction

Metformin does not interact with any of the other treatments for prostate cancer and can be continued during all further treatments started on progression.

Caution is needed however when initiating potential nephrotoxic drugs as metformin is renal excreted therefore may accumulate if renal function deteriorates.

As metformin is being given as an IMP in the context of a clinical trial, continued use will not be permitted if patients participate in other interventional clinical trials for prostate cancer (i.e. CRPC setting). Investigators should use their discretion and discuss discontinuing metformin with the trial team if it is felt to be in the patient's best interest.

Table 17: Drugs which require additional monitoring of renal function

Clinical use	Drug	Recommendation
Anti-hypertensives and other cardiac disease	ACE inhibitors/angiotension II receptor blockers e.g. ramipril, lisinopril, Irbesartan	Monitor renal function until confirmed to be stable and providing GFR remains >45ml/min/m^2. Repeat test if necessary
	Diuretics e.g Frusemide, budesonide	
Antibiotics	Aminoglycoside antibiotics e.g Gentamicin or amikacin	Hold metformin during treatment and re-start providing renal function confirmed to be stable and GFR remains >45ml/min/m^2
Analgesia	NSAIDS e.g. Ibuprofen, diclofenac, naproxen	Avoid if possible If no alternative increase renal monitoring to until confirmed to be stable and providing GFR remains >45ml/min/m^2

If the renal function declines to GFR<45ml/min/m^2 a dose reduction is required and the frequency of monitoring of renal function must increase. See Section 6.2.7.A.

6.3.4 Transdermal Oestradiol: Drug Interactions

The metabolism of oestrogens may be increased by concomitant use of substances known to induce drug metabolising enzymes, specifically cytochrome P450 enzymes, such as anticonvulsants (e.g. phenobarbital, phenytoin, carbamezapine) and anti-infectives (e.g. rifampicin, rifabutin, nevirapine, efavirenz). Ritonavir and nelfinavir, although known as strong inhibitors, by contrast exhibit inducing properties when used concomitantly with steroid hormones. Herbal preparations containing St. John's wort (Hypericum Perforatum) may induce the metabolism of oestrogens.

With transdermal administration, the first-pass effect in the liver is avoided and, thus, transdermally applied oestrogens might be less affected than oral hormones by enzyme inducers. Oestradiol levels are already monitored as part of trial follow-up while patients are on transdermal oestradiol. As a precaution, we recommend monitoring the drug levels of the above concomitant medications among patients receiving transdermal oestradiol.

6.4 TRIAL PRODUCTS

Details of the procedures for obtaining the drugs within the trial, dispensing and disposal of unused drug are given in Appendix B. Arrangements for free or discounted drugs are given in the Finance section (Section 15).

6.5 MEASURES OF COMPLIANCE/ADHERENCE

Date of treatment, dose, delays and reasons for delays or dose modifications of all trial treatments will be recorded. The estimated number of abiraterone tablets and enzalutamide capsules taken in a given time period will also be recorded as well as any dose reductions.

Oestradiol levels will be collected for patients in the transdermal oestradiol arm and used to assess compliance to treatment (see Section 6.2.8).

6.6 TREATMENT DATA COLLECTION

Data will be recorded on case report forms (CRFs); the top copy/original should be sent to CTU for data entry and a copy kept at the local centre. Up-to-date versions of all CRFs can be found on the trial website (http://www.stampedetrial.org/) and centres will be notified of any changes throughout the course of the trial. The type of data to be recorded is detailed in the Assessments and Procedures section (Section 7).

6.7 ADMINISTRATION OF STANDARD RADIOTHERAPY TO M0 PATIENTS

6.7.1 Treatment Details

Standard radiotherapy will be given to appropriate patients in each of the trial arms, following a period of neo-adjuvant ADT therapy, as is generally standard in UK practice. For patients with negative nodes on axial imaging, clinicians may choose between irradiating prostate and seminal vesicles alone or including the pelvic nodes in addition. Additional staging tests such as pelvic node sampling may be considered in making this decision. Conformal or intensity modulated radiotherapy should be used in all patients. Where patients have good clinical evidence that nodes are free of tumour or patients for whom nodal radiotherapy is contra-indicated (e.g. significant bowel disease), treatment may be given to the prostate gland and seminal vesicles only. The recommended dose is 74Gy in 37 fractions to the prostate and seminal vesicles or the equivalent using hypo-fractionated schedule, 60Gy in 20 fractions. Alternative dosing schedules are permitted but must be agreed with the STAMPEDE Trial Management Group.

6.7.1.A Standard-Of-Care RT Timing In M0 patients

If receiving docetaxel as part the standard-of-care (permitted from Protocol version 14.0), the patient must have sufficiently recovered from any docetaxel toxicity before RT can begin. In all other patients not receiving SOC docetaxel, SOC RT may be started sooner (2-6 months post-randomisation) in line the data from the MRC PR07 trial (9).

7 ASSESSMENTS AND PROCEDURES

7.1 SCHEDULE FOR ASSESSMENTS

7.1.1 Follow-Up Schedules

An individualised form with a follow-up schedule will be provided for each randomised patient. A detailed follow-up schedule is given in **Table 18, 19 and 20**.

For patients who are receiving ADT given as LHRH agonists or antagonists it is assumed that their allocated research treatment will commence within four weeks of randomisation.

For patients randomised to transdermal oestradiol, any treatment started with LHRH agonists, LHRH antagonists or anti-androgens should discontinue and treatment with transdermal oestradiol should commence as soon as practically possible after randomisation (ideal timeline: within one week of randomisation).

For patients who are due to have an orchidectomy, it is recognised that surgery will have to be scheduled and the scheduling of any additional treatments may be affected by post-operative recovery. (Note that this is the least commonly chosen method of hormone therapy in the trial).

Note that for the transdermal oestradiol arm, the first follow-up visit post-randomisation can be scheduled at 4 instead of 6 weeks to coincide with the 4-week hormone tests (see **Section 6.2.8A**).

See **Table 18** for a summary of required investigations at each follow-up visit.

7.1.2 PSA, Testosterone And Oestradiol Measurements

All patients should have PSA measured prior to starting ADT and at every subsequent trial follow-up visit. For patients who do not have a scheduled hospital visit, it would be acceptable for arrangements to be made for blood samples to be drawn at their GP surgery.

For arm L patients, oestradiol and testosterone levels should continue to be monitored while the patient remains on transdermal oestradiol treatment; see **Table 18** for when these measurements should be obtained. These samples could be taken at the same time as the PSA tests, unless additional tests are required as detailed in **Sections 6.2.8.B** and **6.2.8.C**. It is also preferable for samples to be taken the day before the oestradiol patches are changed, to allow consistent measurements of testosterone and oestradiol with respect to the pharmacokinetic profile of the patches.

Table 18: Summary of Required Follow-up Measurements and Investigations

	VISIT	ALL PATIENTS	ARM A	ARM G OR ARM J[c]	ARM K	ARM L
TIME FROM RANDOMISATION	4-6 wks	PSA<br>Weight & WC		LFTs<br>BP		Testosterone & Oestradiol Levels[e, f]
	12 wks	PSA<br>Weight & WC		LFTs<br>BP		Testosterone & Oestradiol Levels[e]
	18 wks	PSA<br>Weight & WC		LFTs<br>BP		
	24 wks	PSA<br>Weight & WC	HbA1c[b]<br>Fasting glucose[b]<br>Fasting lipid profile[b]	LFTs<br>BP	HbA1c<br>Fasting glucose<br>Fasting lipids<br>Renal function[d]	Testosterone & Oestradiol Levels[e]
	36 wks	PSA<br>Weight & WC		LFTs<br>BP		
	48 wks	PSA<br>x2 Streck™ tubes[a]<br>Weight & WC	HbA1c[b]<br>Fasting glucose[b]<br>Fasting lipid profile[b]	LFTs<br>BP	HbA1c<br>Fasting glucose<br>Fasting lipids<br>Renal function[d]	Testosterone & Oestradiol Levels[e]
	60 wks	PSA<br>Weight & WC		LFTs<br>BP		
	72 wks	PSA<br>x2 Streck™ tubes[a]<br>Weight & WC		LFTs<br>BP	Renal function[d]	Testosterone & Oestradiol Levels[e]
	84 wks	PSA<br>x2 Streck™ tubes[a]<br>Weight & WC		LFTs<br>BP	Renal function[d]	
	96 wks	PSA<br>Weight & WC		LFTs<br>BP	Renal function[d]	Testosterone & Oestradiol Levels[e]
	104 wks	PSA<br>Weight & WC	HbA1c[b]<br>Fasting glucose[b]<br>Fasting lipid profile[b]	LFTs<br>BP	HbA1c<br>Fasting glucose<br>Fasting lipids	Testosterone & Oestradiol Levels[e]
	All further FU visits	PSA<br>Weight & WC		LFTs<br>BP	Renal function[d]	Testosterone & Oestradiol Levels[e]

Key: LFT= liver function test, BP= blood pressure, WC=waist circumference

[a] M1 patients pre-progression participating in sequential blood sampling sub-study, see **Sample collection and handling manual** for further details.

[b] Patients without diabetes at baseline randomised to protocol version 16.0 i.e. eligible for metformin comparison, activated from Sept-2016 onwards.

[c] Additional safety monitoring is required for all patients receiving research abiraterone: LFTs and BP should be checked 2 weekly until 12 weeks and then monthly.

[d] Renal function must be monitored regularly whilst receiving research metformin. See **Section 6.2.7.A** for further details.

[e] Hormone tests are required whilst the patient is still receiving research transdermal oestradiol. Note that additional tests may be necessary as detailed in **Section 6.2.8.B** and **Section 6.2.8.C**.

[f] First hormone tests for patients receiving research transdermal oestradiol should be at 4 weeks, see **Section 6.2.8.A**.

7.1.3 Assessment Of Treatment Failure (Definition Of Progression)

It is not proposed to routinely assess patients for response. However, in order that objective progression can be assessed, it is necessary to have imaging taken at time of best response as judged by the treating clinician.

All patients should have baseline radiological examinations as detailed in Section 4.5.2. In addition, it is recommended all patients should have scans or X-rays repeated at 24 weeks (and whenever clinically appropriate) if they were abnormal at baseline, particularly if they have a low PSA value on entry in to the trial making biochemical assessment of treatment failure difficult.

The following outcomes should be reported on the Progression log:

- Biochemical failure
- Local progression
- Lymph node progression
- Progression or development of new distant metastases, defined as lymph nodes outside the pelvis, bone or organ involvement
- Skeletal-related events confirmed as progression (see below)

7.1.3.A Biochemical Failure

For the purposes of the STAMPEDE trial, a unique threshold PSA value for biochemical failure is calculated, referred to as the **PSA progression value**.

This value is derived for each patient based on their **PSA nadir,** defined as the lowest PSA value reported between randomisation and 24 weeks on trial.

The exact method for deriving the progression value for a patient depends on the value of their PSA nadir, and how this compares to their pre-treatment PSA value (i.e. the extent of the fall in PSA from the starting point).

The PSA progression value is calculated in one of three ways:

A. If the lowest recorded PSA value in the 24 weeks following randomisation is more than 4ng/ml and more than 50% of the pre-treatment PSA level then the patient fulfils the criteria for immediate treatment failure.

B. For patients whose PSA nadir in the 24 weeks following randomisation is less than or equal to 50% of the pre-treatment PSA level but remains above 4ng/ml, biochemical failure will be defined as a rise of 50% above the nadir level.

C. For patient whose PSA nadir is less than or equal to 4ng/ml, biochemical failure is defined as at least a 50% rise above the nadir value that is also above 4ng/ml.

Confirming biochemical failure: the timing of assessments needs to be considered because spurious rises in PSA can occur e.g. following procedures involving the urinary tract. For this reason, any isolated rise in PSA should be confirmed before reporting biochemical failure.

In the case that the raised PSA value reaches the progression value, a confirmatory PSA test should be performed between one week and 3 months later. Biochemical failure is confirmed if the second

value is around the same level or higher i.e. the trend is confirmed. The date of PSA progression should be provided as the date of the **first** raised PSA that fulfilled the trial definition of progression. Only the first instance of biochemical failure needs to be reported.

A confirmatory PSA is not required if there are other signs of progression e.g. progression of cancer related symptoms (clinical progression) or new radiological progression.

Second line treatment commenced specifically for biochemical failure should not start until the trial definition for biochemical failure has been met. However, if second line treatment does start before the trial definition is met then report the closest PSA value prior to the treatment start date as the progression value. This is not required if second line treatment is being started for other signs of progression e.g. clinical or radiological.

Testosterone levels: are only required when reporting biochemical progression whilst receiving hormone treatment to confirm the diagnosis of castrate resistant prostate cancer. Testosterone levels are not required when reporting biochemical progression in patients not receiving hormone therapy e.g. patients who presented with non-metastatic disease have relapsed following completion of treatment.

See Appendix E for further details on the trial definition of biochemical failure.

7.1.3.B Local, Lymph Node And Metastatic Failure

For each of local, lymph node and distant metastases progression, **both** the following should be reported:

1. Date of first clinical/symptomatic progression
2. Date of first objective/radiological progression

7.1.3.C Skeletal-related Events

Skeletal-related events (SREs) are defined as:

- Pathological Fracture
- Spinal cord compression
- Requirement for RT to bone (e.g. for pain or impending fracture)
- Requirement for surgery (e.g. for prevention or management of fracture)

SREs are a secondary outcome measure and a disease event of interest. SREs may represent disease progression but can also occur due to treatment-related effects e.g. osteoporotic fracture due to treatment-related bone-mineral density loss. From Protocol version 15.0 information regarding SREs will be collected at each follow-up visit. All SREs should be investigated further to establish whether or not the patient has progressed and, if confirmed as progression, a Progression Log should be completed to record this along with an Additional Treatment Log to give details of any treatment received (e.g. palliative RT or surgery)

The summary of timing of Case Report Forms can be viewed in Table 20.

7.1.4 Additional Metabolic And Cardiovascular Outcomes

A number of metabolic and cardiovascular (CVS) outcomes are being assessed in the “metformin comparison” and “transdermal oestradiol comparison” as outlined below.
The summary of timing of Case Report Forms can be viewed in Table 20.

7.1.4.A Metabolic And Cardiovascular Outcomes: Metformin Comparison

Metformin is hypothesised to mitigate the metabolic and cardiovascular effects of long-term ADT. Additional metabolic and cardiovascular outcomes are now to be collected from Protocol version 15.0 onwards to explore this effect.

7.1.4.B Cardiovascular Outcomes: Transdermal Oestradiol Comparison

Cardiovascular morbidity and mortality was the primary outcome measure for the first stage in the PATCH trial (completed in 2010), which showed similar rates of CVS events in patients receiving transdermal oestradiol compared to those receiving LHRH injections(42). These results have been confirmed by longer-term data within the trial (see **Appendix I**). Continued monitoring of CVS outcomes will be undertaken by the PATCH IDMC for both the PATCH trial, as well as for the patients in STAMPEDE allocated to transdermal oestradiol together with their contemporaneous controls.

While Arm L patients are undergoing treatment with transdermal oestradiol, the majority of these CVS events will fall under the definitions of Serious Adverse Events (see **Section 11**). Once a patient has a cardiovascular event, the discontinuation of treatment with transdermal oestradiol may be considered at the discretion of the treating clinician and the patient switched to standard of care hormone therapy.

An increased risk of venous thromboembolism has been observed when docetaxel is used in combination with certain agents for the treatment of prostate cancer. As yet, there are limited safety data available on docetaxel use in combination with transdermal oestradiol from the PATCH trial. Therefore, the rate of CVS events will be closely monitored among patients within Arm L who are receiving docetaxel as part of their first-line treatment. For more details see **Appendix I**.

Table 19: Collection of additional metabolic and cardiovascular outcomes

OUTCOME OF INTEREST	TIMING OF ASSESSMENT	CRF	ARMS
**Eligibility screening**			
HbA1c	Prior to randomisation	Randomisation	A, K
**Baseline**			
Lipid profile (cholesterol, HDL, LDL)	Randomisation	Baseline	A, K
Fasting glucose	Randomisation	Baseline	A, K
Fasting triglyceride	Randomisation	Baseline	A, K
Weight and BMI	Randomisation	Baseline	A, K
Waist circumference	Randomisation	Baseline	A, K
**Follow-Up**			
HbA1c	24 weeks<br>48 weeks<br>96 weeks	FU	A, K
Fasting glucose	24 weeks<br>48 weeks<br>96 weeks	FU	A, K
Fasting triglyceride	24 weeks<br>48 weeks<br>96 weeks	FU	A, K
Lipid profile (cholesterol, HDL, LDL)	Annually	FU	A, K
**Metabolic and cardiovascular events**			
New diagnosis of diabetes	As and when metabolic and cardiovascular event occurs	FU	A, K
Cardiac event: myocardial infarction or revascularization<br>(e.g PCI or CABG)	As and when metabolic and cardiovascular event occurs	FU	A, K, L
Stroke or transient ischaemic event	As and when metabolic and cardiovascular event occurs	FU	A, K, L
Venous Thromboembolism (Deep Vein Thrombosis / Pulmonary Embolism)	As and when metabolic and cardiovascular event occurs	FU	A, K, L
Congestive Cardiac Failure	As and when metabolic and cardiovascular event occurs	FU	A, K, L

FU = follow-up

7.1.5 Additional Safety Assessment

Medical review and PSA measurements are repeated for all patients across all research arms (including the control arm) and follow the trial FU schedule. Patients have FU assessments every 6 weeks for 6 months (apart from the transdermal oestradiol arm where the first assessment is at 4 weeks instead of 6 to coincide with the hormone tests), every 12 weeks up to 2 years, six-monthly up to 5 years and annually thereafter). For patients who do not have a scheduled hospital visit, it would be acceptable for arrangements to be made for blood samples to be drawn either in a GP's surgery or in the patient's home.

Additional safety assessments for specific arms are outlined below. The summary of the timing of Case Report Forms can be viewed in Table 20.

7.1.5.A Additional Safety Assessment: Abiraterone

Due to the risk of liver toxicity and secondary hyperaldosteronism with abiraterone, patients will require 2 weekly U+Es, LFTs for the first 12 weeks and monthly BP checks. It is not necessary to report these unless abnormal; in this instance, they should be reported as AEs (on the next Follow-Up CRFs) and as SAEs (see Section 11) if appropriate. After 12 weeks, monthly liver function tests will be required on all patients receiving treatment on trial abiraterone.

7.1.5.B Additional Safety Assessment: Metformin

Patients with normal and stable renal function receiving **metformin** require monitoring of **renal function (U&Es) every 6 months** whilst on treatment. More frequent monitoring is required in patients with declining renal function, or when initiating new potentially nephrotoxic medications or at times of intercurrent illness (see Section 6.2.7.A). Changes in renal function (eGFR, graded according to CTCAEv.4) are recorded on the Follow-Up CRF. It is acceptable for bloods sampling to be arranged via the GP at the patient's home or local hospital.

7.1.5.C Additional Safety Assessment: Transdermal Oestradiol

Hormone levels are monitored while patients are on transdermal oestradiol, and if oestradiol levels are found to be >2000pmol/L with confirmed repeat test, please contact CTU for advice (see Section 6.2.8.C).

7.2 DATA COLLECTION PROCEDURES

Treatment-related data are collected on Treatment Specific Forms or Logs. It is important that any treatment given for progressive disease is recorded on the Additional Treatment Log. This should be updated with any subsequent changes e.g. treatment for CRPC. The summary of timing of Case Report Forms can be viewed in Table 20.

7.2.1 Data Collection For SOC Hormone Therapy

Information relating to SOC hormone therapy is recorded on the SOC Hormone Therapy Log unless it is a treatment change for disease progression. The SOC Hormone Therapy Log should be updated with any changes in long-term hormone therapy e.g. if anti-androgens are being added to LHRH for dual androgen blockade in the **absence of progression**. If however, anti-androgens are being added as an additional treatment for progressive disease, then this should be recorded on the Additional Treatment Log. SOC hormone therapy only refers to LHRH or anti-androgens; if second-generation AR-targeted treatments such as abiraterone or enzalutamide are used as second-line treatments for progression this should only be recorded on the Additional Treatment Log. Corresponding details of the progression event should be reported on the Progression Log.

If a patient allocated to receive transdermal oestradiol switches to receiving SOC Hormone Therapy i.e. LHRH in the **absence of progression**, then this information should be recorded on the SOC Hormone Therapy Log. However, any changes in hormone therapy initiated to treat disease progression should be recorded on the Additional Treatment Log e.g. switching from transdermal oestradiol to LHRH due to progressive disease.

7.2.2 Data Collection For Standard Docetaxel

The decision to use docetaxel as part of the standard-of-care must be made before randomisation and should be recorded on the Randomisation CRF. The date of the first cycle should be recorded at the time of randomisation; this can be a planned date when randomisation occurs prior to docetaxel commencing but must be within 12 weeks of starting ADT (see Section 6.2.7). All further details should be recorded on the SOC Docetaxel Treatment CRF upon completion of the final cycle.

If a patient does not receive the planned docetaxel this must also be recorded on the SOC Docetaxel Treatment CRF, together with the reason why.

7.2.3 Data Collection And Non-Administration Of Standard Radiotherapy

There are CRFs to be completed for ALL patients regardless of being planned for, or subsequently receiving, primary radiotherapy. Where radiotherapy is not received a reason should be provided on the Radiotherapy Detail CRF whether this is standard-of-care radiotherapy for patients (on any research arm) or research RT to the prostate for Arm H patients.

All radiotherapy and acute side effects details should be recorded on the Radiotherapy Detail and Radiotherapy Acute Toxicity CRFs upon completion of the RT schedule; any RT late side effects should be recorded on the Follow-Up CRF under the section for RTOG Toxicities.

If RT is not given, this should be stated on the Radiotherapy Detail CRF together with the reason for non-administration of the treatment in those instances where RT was planned and not given (for example, due to early metastatic progression or patient refusal).

7.2.4 Data Collection For Palliative Radiotherapy

Details of any radiotherapy given for progressive disease should be recorded on the Additional Treatment Log.

This includes palliative RT for SREs e.g. bone pain and spinal cord compression (note that these should also be reported as SREs on the Follow-Up CRF and only reported on the Progression Log and Additional Treatment Log if confirmed as progression), as well as salvage RT to the prostate.

7.2.5 Data Collection for Research (M1) Radiotherapy

Arm H only: all radiotherapy and acute side-effects details will be recorded on the Radiotherapy Detail and Radiotherapy Acute Toxicity CRFs upon completion of the RT schedule; any RT late side effects will be recorded on the Follow-Up CRF under the section for RTOG Toxicities.

In those cases where RT is not given (for example, due to early metastatic progression or patient refusal), this should be stated on the Radiotherapy Detail CRF together with the reason for non-administration of the treatment.

7.2.6 Data Collection for Additional Treatments Given for Disease Progression

All treatments given for disease progression are recorded on the Additional Treatment Log. This should be updated with all subsequent changes to treatment. Only treatments for progressive disease need to be recorded; details of supportive treatments such as pain killers or bone-strengthening agents e.g. zoledronic acid, given to relieve symptoms, does not need to be provided.

In some scenarios, SOC hormone therapies such as LHRH or anti-androgens may be given as a treatment for progressive disease. For example, LHRH may be re-started on relapse for patients with M0 disease who discontinued hormone therapy and commenced surveillance. In addition, patients allocated to trandermal oestradiol may switch to LHRH on progression. Historically, some patients progressing on LHRH will have commenced anti-androgens (dual androgen blockade) as a treatment for progression. In all cases, if treatment is being started for disease progression, treatment data are collected on the Additional Treatment Log and the details of the progression event recorded on the Progression Log.

Please note that any change in ADT which are solely a change in the patient's long-term hormone therapy, and not for disease progression, should be reported on the SOC Hormone Therapy Log only and not on the Additional Treatment Log.

7.3 FOLLOW-UP PROCEDURE

Every effort should be made to follow-up all patients who have been randomised. Patients should, if possible, remain under the care of an oncologist or urologist for the duration of the trial. If care of a patient is returned to the GP, it is the responsibility of the responsible clinician who obtained the patient's consent to participate in the trial to ensure that all relevant data collection forms are completed. Nurse-led follow-up is permitted and should be conducted in line with local practice and procedures.

If the patient moves away from the local area, arrangements should be made for trial follow-up to be undertaken by their new local centre. Details of other participating centres can be obtained from the STAMPEDE Trial Team. Information on patient transfer procedures is detailed in **Section 8.2.** If the responsible clinician moves, appropriate arrangements should be made to arrange for trial follow-up to continue at the centre.

All efforts should be made to preserve the initial patient's consent for long-term survival information to be flagged through national registries, for example NHS Digital (previously the Health and Social Care Information Centre); Office of National Statistics (ONS) in England/Wales; General Register Office in Scotland; Hospital Episode Statistics (HES) or Public Health England.

Please see **Section 8** for more information on early stopping of follow-up.

7.3.1 Follow-Up Telephone Consultations

In certain circumstances it may be appropriate to replace hospital visits with telephone consultations providing that it is still possible to collect all the necessary follow-up information. Situations where this may be considered include at the point where patients would normally be discharged from oncology or urology services. In these instances, it is acceptable to alternate appointments with telephone consultations providing the required blood results are available to the research team. All necessary information required to complete the Follow-Up CRF is still required. All details on the telephone consultation must be recorded in the patients' notes as per in person assessments.

Other circumstances where it may be appropriate to use telephone consultations is when assessing treatment tolerance and advising regarding dose modifications, providing that all the required safety monitoring has been adhered to. For example, when re-commencing metformin after a treatment break it may be appropriate to confirm tolerance and advise regarding dose escalation over the phone.

7.4 TRIAL CLOSURE

For the purpose of complying with UK Clinical Regulations introduced in May-2004, the trial will be considered 'closed' when the follow-up point for the primary analysis of the final comparison has been reached. However, further observational follow-up of all patients enrolled in the trial will continue until all randomised patients have died. This will initially be via the hospital, but in the longer term may employ national registers.

Table 20: Summary of timing of case report forms

CASE REPORT FORMS	TIMING OF ASSESSMENT AND CRF
**Registration***	**At registration**
**Biomarker-screening request form**	At registration. For patients participating in the Biomarker-Screening Pilot, when FFPE tumour sample has been retrieved and is ready to be sent to the Sponsor's designated laboratory.
**Baseline**	
Randomisation	At randomisation
Baseline	At randomisation
Cardiovascular Assessment	At randomisation
Bone Density Risk Factor	At randomisation
Pathology	At randomisation. When pathology sample has been taken and sent to Sponsor's designated laboratory.
**Treatment**	
SOC Docetaxel Treatment	To be completed for all patients 20 weeks after randomisation.
SOC Hormone Therapy Log	To be completed every time there is a change in SOC hormone therapy to report (including when Arm L patients switch to SOC HT pre-progression). To be sent in with the corresponding Follow-Up CRF.
Abiraterone and Enzalutamide Treatment Log	To be completed when treatment is first started and subsequently every time there is a dose change, treatment pause and re-start.<br>To be sent in with the corresponding Follow-Up CRF.
Metformin Treatment	To be completed when treatment is first started and subsequently every time there is a dose change, treatment pause and re-start.<br>To be sent in with the corresponding Follow-Up CRF.
Transdermal Oestradiol Treatment Log**	To be completed when treatment is first started and subsequently when reporting change in dose or type of patch.
RT Detail	To be completed for all patients:<br>Upon completion of SOC RT<br>If planned RT is no longer to be given (at 10 months after randomisation)<br>Arm H patients when research RT completed<br>Arm A patients with newly-diagnosed M1 disease at 3 months to confirm RT was not given
RT Acute Toxicity	For all patients who receive primary RT.
**Assessments**	
Follow-Up	To be completed every 6 weeks for 6 months, then every 12 weeks until 2 years, then every 6 months until 5 years and annually thereafter*. (See Table 19 for more information).
Transdermal Oestradiol Treatment Hormone Results Log	To be complete whenever there are testosterone and oestradiol test results while arm L patients on transdermal oestradiol.

CASE REPORT FORMS	TIMING OF ASSESSMENT AND CRF
End of Research Treatment	To be completed when (each) allocated research treatment is permanently stopped or in the event that allocated research treatment is never started (in each case a reason for stopping/never starting should be provided).
Progression Log	To be completed at the occurrence of each progression event (PSA, local, nodal, distant metastases) and for each method of detection (clinical/symptomatic and objective/radiological).<br>Skeletal-related events confirmed as progression should also be reported here.
Additional Treatment Log	To be completed each time a patient who has progressed starts or completes any additional treatment for progression.
Serious Adverse Event	To be completed following any Serious Adverse Event
Death	At Death
**Administration**	
Patient Transfer Confirmation Form	To be completed when a patient is transferred to a different hospital for the administration of trial treatment and follow-up
Co-enrolment	To be completed when a patient is co-enrolled in any other clinical trial. Please see **Section 5.2** for more information

* For centres participating in the biomarker-screening only

** For the transdermal oestradiol arm, the 6-week follow-up form can be completed at the same time as the 4-week visit for the hormone tests (see Section 6.2.8.A)

Table 21: Schedule for completion of treatment and outcome forms by arm.

Timing From Randomisation			Treatment Log[$]	RT	Outcome forms	
Years	Months	Weeks	(if required)		Follow-Up[Ψ]	QL + HE[¥]
**6-Weekly**						
-	-	6*	G, J, K, L	-	All arms	A, G, H, J, K, L
-	-	12	G, J, K, L	-	All arms	A, G, H, J, K, L
-	-	18	G, J, K, L	-	All arms	A, G, H, J, K, L
-	6	24	G, J, K, L	Arm H	All arms	A, G, H, J, K, L
**12-Weekly**						
-	9	36	G, J, K, L	-	All arms	A, G, H, J, K, L
1	12	48	G, J, K, L	All arms	All arms	A, G, H, J, K, L
-	15	60	G, J, K, L	-	All arms	A, G, H, J, K, L
-	18	72	G, J, K, L	-	All arms	A, G, H, J, K, L
-	21	84	G, J, K, L	-	All arms	A, G, H, J, K, L
-	-	96	G, J, K, L	-	All arms	A, G, H, J, K, L
**6-Monthly**						
2	24	104	G, J, K, L	-	All arms	A, G, H, J, K, L
	30	130	G, J, K, L	-	All arms	A, G, H, J, K, L
3	36	156	G, J, K, L	-	All arms	A, G, H, J, K, L
	42	182	G, J, K, L	-	All arms	A, G, H, J, K, L
4	48	208	G, J, K, L	-	All arms	A, G, H, J, K, L
	54	234	G, J, K, L	-	All arms	A, G, H, J, K, L
5	60	260	G, J, K, L	-	All arms	A, G, H, J, K, L
**Annual**						
6	72	-	G, J, K, L	-	All arms	A, G, H, J, K, L
7	84	-	G, J, K, L	-	All arms	A, G, H, J, K, L
Etc.	-	-	G, J, K, L	-	All arms	A, G, H, J, K, L

Key:

H = SOC + RT
G = SOC + abiraterone
J = SOC + enzalutamide + abiraterone
K = SOC + metformin
L = Transdermal oestradiol ± RT ± docetaxel

Notes:

Ψ See Table 19 for information required at follow-up
¥ 1st 700 pts and from Protocol version 8.0 onwards only
* For the transdermal oestradiol arm, the 6-week follow-up form can be completed at the same time as the 4-week visit for the hormone tests (see Section 6.2.8.A)
$ For patients in Arm L on transdermal oestradiol, the hormone tests results are to be reported on the Transdermal Oestradiol Treatment Hormone Results Log

8 STOPPING OF TREATMENT OR FOLLOW-UP

Patients should be given every encouragement to adhere to their allocated protocol treatment and follow-up schedule, in order to reduce bias. However, a patient has the right to withdraw consent for participation in any aspect of this trial at any time.

8.1 STOPPING RESEARCH INTERVENTIONS

A patient may stop **any trial treatment** for the following reasons:

- Unacceptable toxicity
- Intercurrent illness which prevents further treatment
- Patient refusal
- Any alteration in the patient's condition which justifies the discontinuation of treatment in the clinician's opinion

8.1.1 Stopping Trial Treatment: Abiraterone, Enzalutamide + Abiraterone

For **patients randomised to Arm G or J**, trial treatment should also be discontinued for the following reasons:

- Disease progression whilst on therapy (please refer to Section 7.1.3)
- Intention to commence a new anti-cancer treatment due to evidence of relapse

As detailed in Section 7.1.3, the disease event for stopping treatment may be after the first reportable Failure-Free Survival event.

In all cases reason for permanent stopping of research treatment should be recorded on the End of Research Treatment CRF.

8.1.2 Stopping Trial Treatment: Metformin

For **patients randomised to Arm K**, treatment duration is detailed in Section 6.2.6.

Reasons for early stopping of metformin can be:

- Decline in renal function (metformin must be stopped if GFR<30ml/min/1.73m^2, see Section 6.2.7.A
- Decline in performance status (WHO PS>2)
- Unacceptable toxicity
- Patient refusal
- Intercurrent illness preventing continued metformin treatment
- Investigator decision e.g. administration of IMP within a CTIMP in CRPC setting

If metformin is paused for more than 3 months or >50% of doses are missed please discuss with the trial team as treatment is likely to need to be stopped.

8.1.3 Stopping Trial Treatment: Transdermal Oestradiol

For **patients randomised to Arm L**, treatment with transdermal oestradiol may be discontinued for the following main reasons:

- Unacceptable toxicity
- Patient refusal

- Intercurrent illness
- Investigator decision
- Cardiovascular event (see **Section 7.1.4B**)

In addition, upon evidence of disease progression and at the investigator's discretion, a switch to LHRH analogues is appropriate to facilitate the addition of further therapies where concurrent treatment with transdermal oestradiol is untested. For patients who are on the 3 patch maintenance dose and have castrate levels of testosterone, there is currently no evidence that increasing the number of patches further once the patient has progressed would be beneficial and is therefore not recommended.

In the event of stopping research treatment, unless a patient states otherwise, consent is assumed for continued recording of trial data.

8.2 PATIENT TRANSFERS

For patients moving away from the area and planning to transfer care, every effort should be made for the patient to be followed-up at another participating trial centre and for this trial centre to take over responsibility for the patient' ongoing participating in the trial.

To document the transfer process the main contact person at both the current and receiving hospitals should complete and sign the Patient Transfer Confirmation form.

A fully completed form must be returned to CTU prior to the patient transfer and any outstanding data queries for the patient should be completed prior to transfer.

On receipt of the completed transfer form a member of the STAMPEDE team will confirm the database has been updated and request confirmation of the name of the patient's new clinician. Photocopies of the following documents may then be sent to the new hospital to complete the transfer and copies must be also retained at the original site for monitoring purposes:

- Consent form
- Completed CRFs
- Any documentation relating to the patient's participation in STAMPEDE (patient names must be removed from any documentation).

8.3 EARLY CESSATION OF TRIAL PARTICIPATION

If a patient explicitly withdraws consent to have any further trial data recorded their decision must be respected and CTU must be informed in writing. All communication surrounding the early cessation of trial participation should be noted in the patient's records. Please note data for the patient prior to this decision will still be required.

In the majority of cases, patients continue to give permission for their data and information on their health to continue to be collected via clinical notes and national registries. Any information on the follow-up status, however minimal, would be helpful. Investigators are encouraged to faciliate ongoing collection of follow-up data for example, through considering telephone consultations (see **Section 7.3.1**).

Early cessation of trial participation should not be undertaken lightly and the site must consider the implications for the trial and the patient in reaching such a decision. Without long-term data, the efficacy of trial treatments would be less reliable and could lead to inconclusive results. The early stopping of trial treatment should not lead to the early cessation of trial participation and in such cases follow-up assessments should be continued as per trial protocol.

Patients can change their minds about withdrawal at any time and re-consent to participate in the trial. Follow-Up data should be collected only from the point of when consent was re-instated.

9 STATISTICAL CONSIDERATIONS

9.1 METHOD OF RANDOMISATION

Patients will be randomised centrally using a computerised algorithm developed and maintained by CTU. Randomisation will be performed using the method of minimisation over a number of clinically important stratification factors with an additional random element. To decrease determinability, the factors are not listed here but can be found in the Statistical Analysis Plan.

Protocol version 16.0 introduces a new allocation, Arm L: transdermal oestradiol. Allocation to Arm K remains available only to non-diabetic patients with no contraindication to metformin.

See Appendix H for the allocation weighting of each arm by previous protocol version; this also shows allocation weighting for previously closed research arms.

9.2 OUTCOME MEASURES

The overall, definitive primary outcome measure for each comparison in the trial is overall survival (all-cause mortality), unless otherwise stated. The design of the trial is such that it is important to have additional intermediate primary outcome measures to assess activity in each research arm as the trial progresses.

For comparisons involving research arms B to J the intermediate primary outcome measure is failure-free survival; this and other outcome measures are listed in Table 22.

Table 22: Trial Outcome Measures by Comparison Stage (Arms B-J)

COMPARISON STAGE	PRIMARY OUTCOME MEASURE	SECONDARY OUTCOME MEASURES
Pilot phase	Safety*	Feasibility
Activity Stages (AS)	Failure-free survival (FFS)†	Overall survival (OS)<br>Toxicity<br>Symptomatic skeletal events (SSE)
Efficacy Stage (ES)	Overall survival	Quality-of-life<br>Cost effectiveness<br>Failure-free survival†<br>Toxicity<br>Symptomatic skeletal events (SSE)

* Based on toxicity
†Including biochemical failure (see Section 7.1.3 and Appendix E)

For the "metformin comparison" the intermediate and definitive primary outcome measure are the same, being overall survival; see Table 25 for full details of all outcome measures for that comparison.

For the "transdermal oestradiol comparison", overall survival and progression-free survival are the definitive co-primary outcome measures, and the intermediate primary outcome measure is

progression-free survival (PFS); see **Table 27.** The rationale for choosing progression-free survival rather than failure-free survival as the outcome measure for this comparison is outlined in **Section 9.8.2**.

The reasons for different emphases in each recruitment stage are explained in **Section 9.3**.

9.3 SAMPLE SIZE: PRINCIPLES

The design is a multi-arm multi-stage, multi-centre, platform, randomised controlled trial. There are a number of stages for each research arm: a Pilot/Feasibility/Safety Phase, Activity Stages and a final Efficacy Stage. Full details of the methodology underlying the trial design are given by Royston et al. (52, 53) The original sample size calculations were performed using the stage2 (version 1.2.0, Mar-2002) and stagen (version 1.1.1, May-2004) programs, both implemented in Stata (Stata Corp, TX) and updated using the later nstage program (version 1.0.3, Jun-2007; version 2.1.0, Jun-2009; version 3.0.1, Sep-2014). (54)

For each of the comparsions, other than transdermal oestradiol, we have adequately powered each comparison to detect an appropriate improvement in overall survival at the final Efficacy Stage, with high power at each of the planned interim Activity Stages on the intermediate primary outcome. For example, in a cohort with 2 years median FFS and 4 years median survival a target HR of 0.75 for research arm relative to control would translate into an absolute improvement in FFS of 10%, from approximately 50% to 60% at two years and OS of 10%, from approximately 50% to 60% at four years.

The “transdermal oestradiol comparison” is powered only for contributing to a meta-analysis of patients from the STAMPEDE “transdermal oestradiol comparison” and the PATCH trial. It will assess non-inferiority of transdermal oestradiol in terms of overall and progression-free survival which are co-primary outcome measures.

As each comparison is powered to detect a difference in relative improvement, the analyses will be performed when the pre-planned number of events has been reported in the control arm, rather than after a certain number of patients have been recruited to the comparison or a certain amount of time has elapsed. Further details of the sample size calculations and varying assumptions for each research comparison are summarised in the relevant **Sections 9.4-9.8** and detailed in a separate Statistical Design Document which are available on request.

As with all trials, changes in both the standard-of-care and second-line therapies over time are possible which improve outcomes and thus will affect the observed control arm event rates and associated reporting timelines. In particular, from Protocol version 8.0, standard-of-care RT was mandated for all patients with N0 M0 disease and no RT contraindication (this is likely to improve outcomes for this subgroup) and docetaxel permitted from Protocol version 14.0. Further agents are starting to be licensed for patients with castrate-refractory disease which may also improve survival rates. Improved FFS rates would delay the intermediate analyses, for comparisons where FFS is the intermediate primary outcome measure; whilst improved survival rates would delay the definitive analyses. Similarly, improved PFS rates could delay both the time of intermediate and definitive analysis for the "transdermal oestradiol comparison". For each comparison event rates are estimated based on data which are publicly available at the time of design. The Statistical Design Document for arms A-K includes models where median survival is varied around such estimated rates.

Figure 4: Schema of progress of STAMPEDE through the trial*

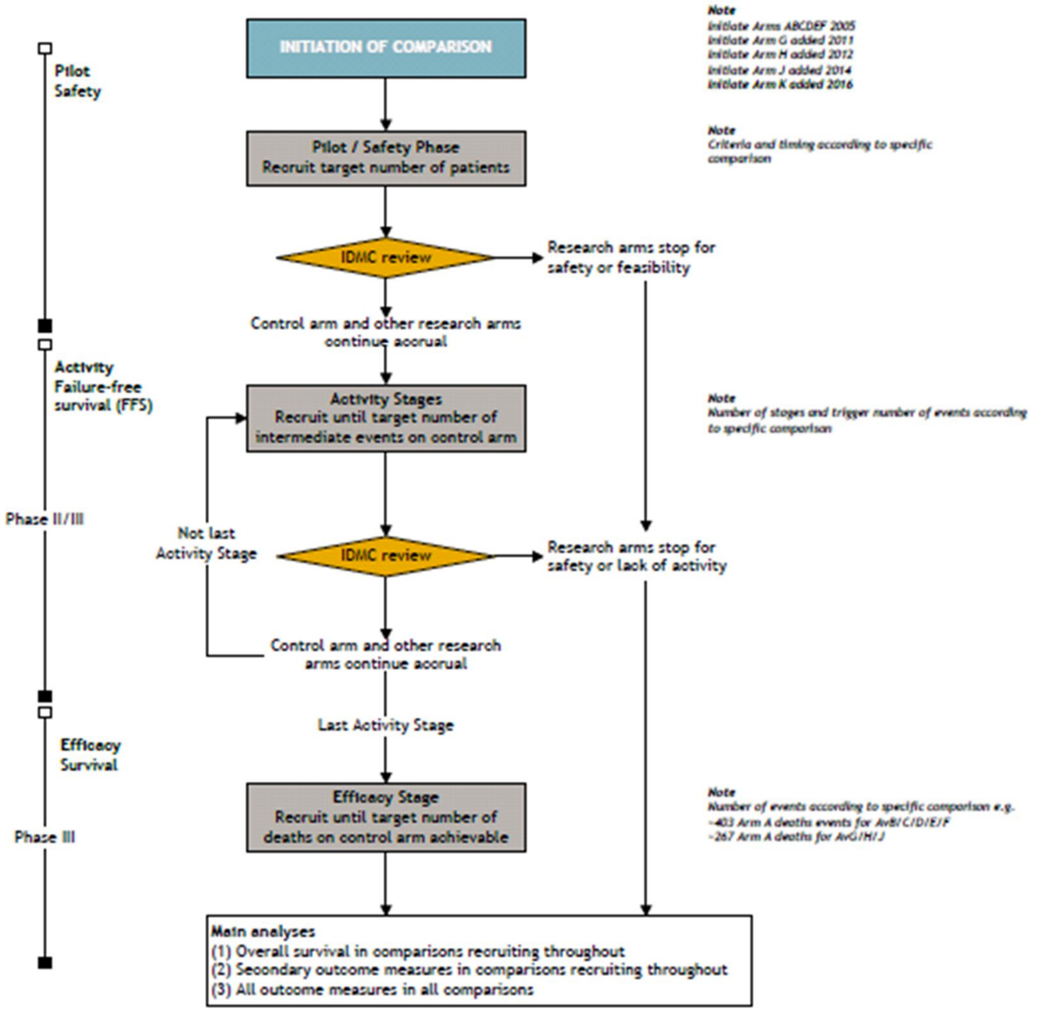

Key
FFS: Failure-free survival
HR: Hazard ratio
IDMC: Independent Data Monitoring Committee
Pts: Patients
* Except for the "transdermal oestradiol comparison"

Notes
Exact accrual depends on many factors including accrual rate, event rate and arms recruiting in each stage

9.4 SAMPLE SIZE ISSUES AND TRIAL STAGES: ADDITIONAL RESEARCH ARM G

This is the "abiraterone comparison" and includes patients allocated to research Arm G (SOC + abiraterone) and patients contemporaneously allocated to the control Arm A (SOC).

9.4.1 Pilot Phase: Additional Research Arm G

The IDMC reviewed safety data, in the context of data from the control arm, when the first 30 patients allocated to Arm G had been on trial for at least 18 weeks.

Furthermore, an additional review of safety was performed when 30 patients with newly-diagnosed non-metastatic disease, allocated to Arm G, had been on trial for at least 18 weeks. Both of these milestones were successfully completed.

9.4.2 Activity Stages I-III: Additional Research Arm G

The same principles were applied to this new comparison as to the original research comparisons. The notable difference was in the accrual rate to this comparison which was anticipated to be higher. There were two reasons for this. First, STAMPEDE initially started recruitment slowly in only a limited number of pilot sites. As more sites have been activated, including internationally, accrual has increased. At the time of adding Arm G (Protocol version 8.0), monthly accrual to the trial was averaging around 60 patients/month (over 700 patients/year). Second, there was an equal allocation ratio for the abiraterone arm compared to the control arm. It was this different allocation ratio which meant that the number of control arm events required to trigger the intermediate analyses was lower for the assessment of abiraterone than the assessment of the original research arms. This is shown in the table below.

Table 23: Guidelines for stopping accrual to additional research Arms G and H

Activity Stage	Sig Level	Power	Targeted HR	Number of control arm events	Consider discontinuation if HR(observed) is…
I	0.50	95%	0.75	~75	>1.00
II	0.25	95%	0.75	~142	>0.92
III	0.10	95%	0.75	~221	>0.89

9.4.3 Efficacy Stage IV: Additional Research Arm G

The analysis of Efficacy Stage IV for this comparison will be performed when around 267 deaths have been observed in the control arm. This will give 90% power to detect the targeted hazard ratio of 0.75 at a one-sided significance level of 0.025.

9.4.4 Sample Size For Additional Research Arm G

Up to around 1,800 patients were targeted join the abiraterone comparison, with half allocated to the research arm G; the observed allocation was 1,917. Consideration was given to ceasing further randomisations to Arm G if it was not showing sufficient evidence of activity at the interim analyses.

The original plan intended for accrual to be halted either when 1,500 patients had been recruited or after 3 years, whichever was the sooner, providing the accrual rate remained above 50 patients/months.

The total number of patients joining this comparison depended not just on observed accrual and event rates, but also the length of time that the original research arms co-recruited alongside this additional research arm; it was originally assumed that this would be for approximately 1 year, but it was closer to 1.5 years. The sample size calculations and projected durations are fairly robust to changes in the length of co-recruitment with the original research arms and future co-recruitment with any further research arms which the Trial Management Group may introduce. Many scenarios are detailed in the Statistical Design Document.

In Protocol version 11.0 in Sep-2013, the target sample size for the "abiraterone comparison" was increased from around 1,500 patients to around 1,800 patients, with note that the efficacy analysis remains unchanged and is still to be triggered by around 267 control arm deaths. This increase in sample size was primarily because of an increase in the proportion of non-metastatic patients joining the comparison; this related to the activation of Arm H which only recruits patients with newly-diagnosed metastatic disease and thereby reduces the numbers of metastatic patients randomised to the "abiraterone comparison". Non-metastatic patients have a lower event rate than the metastatic patients and maintaining the same overall sample size would lead to a delay in time to the primary analysis. The increase in sample size was achievable because recruitment rates to the trial had been substantially higher than the anticipated 50 patients/month for the 6 months preceding the increase.

9.5 SAMPLE SIZE ISSUES AND TRIAL STAGES: ADDITIONAL RESEARCH ARM H

This is the "M1l RT comparison" and includes patients allocated to research Arm H (SOC + RT) and newly-diagnosed M1 patients with no contraindication to RT allocated to the control Arm A whilst Arm H was open to recruitment. Suitability for allocation to the comparison was assessed before randomisation to ensure comparability with contemporaneous control arm patients.

9.5.1 Pilot Phase: Additional Research Arm H

The IDMC reviewed safety data, in the context of data from the control arm, when the first 30 patients allocated to Arm H had been on trial for around six months.

9.5.2 Activity Stages I-III: Additional Research Arm H

The same principles were applied to this new comparison as to previous comparisons and an equal allocation ratio of control arm patients to patients allocated to Arm H was employed; as for Arm G. The number of control arm events required to trigger the intermediate analyses are the same as for the "abiraterone comparison" (see Table 23).

9.5.3 Efficacy Stage IV: Additional Research Arm H

The analysis of Efficacy Stage IV for this comparison will be performed when around 267 deaths have been observed in the relevant control arm patients. This will give 90% power to detect the targeted hazard ratio of 0.75 at one-sided significance level of 0.025.

9.5.4 Sample Size For Additional Research Arm H

Consideration was given to ceasing further randomisations to Arm H if it was not showing sufficient evidence of activity on the intermediate primary outcome measure (FFS), just as for the other research arms. This research comparison is relevant to around 60% of patients joining STAMPEDE. At the point of the scientific approval, accrual was averaging around 80 patients per month to the trial. If accrual to the trial was slower at 70 patients per month, then accrual to this comparison could be

between 18 and 42 patients per month, depending on which other trial arms are open to recruitment at the time.

We are targeting a 25% relative improvement in overall survival following local radiotherapy to the prostate in this patient group. This is the same size of effect targeted with the other research arms in STAMPEDE. This relative improvement can be further justified in the light of MRC PR07 which demonstrated an improvement of this magnitude for adding radiotherapy to ADT in locally-advanced disease, with a hazard ratio for overall survival of 0.77 (95% CI 0.61 to 0.98). In that trial, fewer than half of the deaths were from prostate cancer, whereas in newly-diagnosed metastatic patients nearly all men will die of their disease. Therefore, it is relevant to note the relative benefit of radiotherapy in PR07 in terms of prostate cancer-specific survival, where the hazard ratio was 0.54 (95% CI 0.27 to 0.78). Long-term survival-based data, with a median follow-up of ~10 years, were presented orally at the American Society of Clinical Oncology 2012 which confirmed these findings.(8)

We anticipated that around 1250 patients were required over 4 years to observe 267 control arm deaths after 5.25 years. This assumed that (i) recruitment was constantly 70 pts/m to the trial overall; (ii) the original research arms stopped accrual within 6 months after activation of the RT arm; (iii) the abiraterone arm stops accrual around 24 months after activation of the RT arm; and (iv) a further new research arm with an equal allocation ratio was introduced 18 months after activation of the RT arm. In Protocol version 13.0, we reflected on these four points: (i) recruitment to the trial has been faster; (ii) the original research arms completed accrual 2 months after activation of the RT arm; (iii) the abiraterone arm stopped accrual 12 months after activation of the RT arm; and (iv) Arm J was activated 18 months after activation of the RT arm, Arm H.

Of patients joining STAMPEDE during this time, 60% have been eligible for the “M1l RT comparison”. Prior to randomisation, a RT schedule must be nominated: Weekly or Daily. We have observed that around half of patients in the comparison are nominated for RT with the Daily schedule and half for the Weekly schedule, primarily chosen by trial site with patient groups nominated for each schedule observed to be comparable at baseline. There will likely be interest to know the effect of each RT schedule when the main results are reported. This will be explored by “within schedule” comparisons of patients randomised to research vs control (arms H vs A) within each nominated RT schedule.

Therefore, in Protocol version 13.0, the target sample size was increased from 1,250 patients up to around 1,800 patients, resulting in an approximate increase in the split by planned RT schedule from 625 to 900 in each “within schedule” analysis. A FFS analysis “within schedule” will be carried out at the time of the “main analysis”; predicted to have ~300 control arm FFS events by schedule (FFS “within schedule” analysis parameters: target HR=0.75, power 90%, 1-sided α=0.015). For either of the RT schedules showing evidence of an effect on FFS, a comparative “within schedule” analysis will be carried out on survival when ~199 control arm deaths are observed in that schedule comparison. This is a closed test with OS only formally compared within schedule if there is an advantage in FFS for that RT schedule at the main analysis. Thus, extending recruitment enables a secondary analysis of the impact of RT on survival by planned “RT schedule” to happen within around 18 months from the first main analysis.

All sample scenarios are documented in the Trial Master File.

All patients joining the trial will be starting long-term ADT for the first time. The focus of this comparison will be on the newly-diagnosed, metastatic patients (with no contraindications to RT), which is the largest subgroup of patients in the trial and the group of patients at highest risk of death

from prostate cancer. Patients with non-metastatic disease will be excluded from this particular comparison as there are already randomised data demonstrating the survival benefit from radiotherapy in patients with locally-advanced disease. Radiotherapy is now mandatory in node negative patients; it is also recommended in the node-positive, non-metastatic (N+ M0) group. Relapsing patients are also excluded from this comparison.

For the control arm of the whole trial, we constructed sample size scenarios median failure-free survival being 18, 24 or 30 months and constructed sample size scenarios around each of these options; the event rate would depend on the patient mix. We now know that around 60% of patients have M1 disease at trial entry and we have reported that FFS at 24 months is 51% across the whole of the control arm.(2)

For the updated sample size calculation for this comparison, we based our estimates on the subgroup of patients with newly-diagnosed M1 disease in the control arm. Therefore, we estimate median FFS to be 1 year and estimate that median overall survival will be around 3.5 years.

9.6 SAMPLE SIZE ISSUES AND TRIAL STAGES: ADDITIONAL RESEARCH ARM J

This is the “enzalutamide + abiraterone comparison” and included patients allocated to research Arm J (SOC + enzalutamide + abiraterone) and patients contemporaneously allocated to the control Arm A.

9.6.1 Pilot Phase: Additional Research Arm J

The IDMC first reviewed safety data for this combination when the first 50 patients allocated to Arm J had been on trial around 6 weeks (i.e. to the first follow-up visit).

Furthermore, an additional review of safety was performed when these 50 Arm J patients had been on trial for around 6 months. Safety is routinely reviewed at regular intervals and additional safety reviews will be performed if the IDMC raises any concerns.

Direct comparison will be available with contemporaneously randomised patients on Arm A (hormones alone). Contextual data will be provided from Arm G (hormones plus abiraterone). Indicative safety data may also be available on the combination from other studies in CRPC.

9.6.2 Activity Stages I-II: Additional Research Arm J

The principles of intermediate analyses were applied to this new comparison as to previous comparisons, but some of the details were different, and an equal allocation ratio of control arm patients to patients allocated to Arm J was employed; as for Arms G and H. Owing to the expected accrual rate to the trial (>100 pts/m) and the expected slower event rate, only two activity stages were planned before accrual completed. These are set out in **Table 24**.

Table 24: Guidelines for stopping accrual to the additional research Arm J

ACTIVITY STAGE	SIG LEVEL	POWER	TARGETED HR	NUMBER OF CONTROL ARM EVENTS	CONSIDER DISCONTINUATION IF HRJ(OBSERVED) IS...
I	0.40	95%	0.70	~66	>0.957
II	0.12	95%	0.70	~139	>0.869

9.6.3 Efficacy Stage III: Additional Research Arm J

The analysis of the final Efficacy Stage for this comparison will be performed when around 267 deaths have been observed in the control arm. This would give 90% power to detect the targeted hazard ratio of 0.75 at a one-sided significance level of 0.025.

9.6.4 Sample Size For Additional Research Arm J

Consideration was given to ceasing further randomisations to Arm J if it was not showing sufficient evidence of activity on the intermediate primary outcome measure (FFS), just as for the other research arms.

The patient mix for this comparison is likely to represent a more favourable prognosis on average than in the original research trial's other arms, due to concurrent recruitment of M1 but not M0 patients, to Arm H.

We anticipate that around 1,800 patients are required within 3.5 years to observe ~267 control arm deaths within 6 years. This time will be dependent on the observed overall survival. The default scenario assumes that (i) recruitment is constantly 70pts/m to the trial overall, (ii) the M1| RT arm H accrues throughout and (iii) a further new research arm with an equal allocation ratio is introduced 18 months after activation of Arm J. The stopping date for Arm G is no longer an assumption.

Variations on these factors are documented in a Statistical Design Document. If accrual rates to the trial are at 150pts/m (as observed during Summer 2013), accrual of around 1,800 patients to the comparison could be achieved within 2 years. These sample scenarios will also be documented in the Trial Master File.

Updating the standard-of-care to include docetaxel has minimal impact on the projected time to maturity of the "enzalutamide + abiraterone comparison".

9.6.5 Further Sample Size Issues For Additional Research Arm J

Careful consideration will be given to the implications of any emerging data from the "abiraterone comparison". This has no effect on recruitment to the "enzalutamide + abiraterone comparison" because the recruitment target was reached before any data are available from the "abiraterone comparison".

Indirect comparisons to understand the contribution from each agent may be possible if this research arm is demonstrably superior to the standard-of-care. These plans will be developed and documented elsewhere, but a higher number of patients will help with the power to the indirect comparison.

9.7 SAMPLE SIZE ISSUES AND TRIAL STAGES: ADDITIONAL RESEARCH ARM K

This is the “metformin comparison” and includes patients allocated to research Arm K (SOC + metformin) and the equivalent non-diabetic patients with no contraindication to metformin contemporaneously allocated to the control Arm A whilst Arm K is open to recruitment. Suitability for allocation to the comparison is assessed before randomisation to ensure comparability with contemporaneous control arm patients

9.7.1 Implementation: Additional Research Arm K

The implementation of the MAMS principles are different in this comparison for the following reasons:

- Although all non-diabetic patients will be eligible for allocation to the “metformin comparison”, the timing of the analyses will be driven only by the M1 patients.

- Failure-free survival will not be used as the intermediate primary outcome measure; overall survival will be used as both the intermediate and definitive primary outcome measure. This is because we are not convinced that any comment on metformin’s usefulness should be determined from an ability to act on a PSA-driven outcome measure. Furthermore, treatment with metformin is intended to continue throughout long-term hormone therapy which may include going well beyond an FFS event, particularly in M1 patients.

- The target HR is 0.80 for overall survival (a 20% relative improvement). This is a smaller relative improvement in survival than targeted for previous comparisons because of metformin’s known low toxicity profile, the low cost of the drug and the potential positive effects on metabolic parameters and morbidity; a smaller impact may still have clinical benefit.

9.7.2 Outcome Measures: Additional Research Arm K

Table 25 lists the outcome measures for this comparison and can be compared with the outcome measures for the other comparisons in Table 22.

Table 25: Trial outcome measures by stage for the "metformin comparison"

COMPARISON STAGE	PRIMARY OUTCOME MEASURES	SECONDARY OUTCOME MEASURES
Pilot phase	Safety*	Feasibility<br>Metabolic effects including:<br>:: Changes in BMI<br>:: Changes in haemoglobin A1c (HbA1c)<br>:: Changes in waist circumference<br>:: New diagnosis of diabetes mellitus<br>:: Cardiovascular event: major adverse cardiac events‡
Activity Stage (AS) I	Overall survival	Metabolic effects<br>Toxicity<br>Symptomatic skeletal events (SSE)<br>Failure-free survival† (FFS)
Efficacy Stage (ES) II	Overall survival	Metabolic effects<br>Toxicity<br>Symptomatic skeletal events (SSE)<br>Failure-free survival† (FFS)<br>Quality-of-life<br>Cost effectiveness<br>Correlative outcomes⁂

* Based on toxicity
‡MACE; nonfatal MI, nonfatal stroke, & death from CV causes
†Including biochemical failure (see Section 6.1.2 and Appendix J)
⁂Plasma lipid and fasting triglyceride levels, fasting plasma glucose
Sarcopenia and/or radiological progression free survival (rPFS)
Plasma insulin
AMP Kinase

Note: All arms are unblinded so primary outcome measures for this comparison are objectively measured with caution to be taken around interpretation of more subjective secondary outcome measures such as symptomatic skeletal events

9.7.3 Pilot Phase: Additional Research Arm K

The IDMC will review safety data for this comparisons when the first 50 patients allocated to Arm K have been on trial around 12 months. Furthermore, analyses will be conducted on metabolic parameters (see Table 25). If there is harm observed in metabolic effects, or any serious concerns regarding the toxicity profile, recruitment would be stopped; there are no formal criteria to guide this.

Safety is routinely reviewed at regular intervals and additional safety reviews will be performed if the IDMC raises any concerns.

9.7.4 Activity Stage I: Additional Research Arm K

The principles of intermediate analyses will be applied to this new comparison as to previous comparisons, but some of the details will be different, and an equal allocation ratio of control arm patients to patients allocated to Arm K is employed; as for Arms G, H and J. Owing to the expected accrual rate to the trial overall (>100 pts/m) and the interim primary outcome being overall survival, only one intermediate activity stage is planned before accrual is completed; this is set out in the Table 26.

Although analyses are triggered by events in M1 patients, they will include all patients in the "metformin comparison"; this will have high power. A separate subgroup analysis in M1 patients (conventionally-powered) and M0 patients (limited power) will then look at consistency of effect;

there will be few deaths in M0 patients at this time. The IDMC recommendation will be based on the totality of the available data, including safety, metabolic and compliance data.

Table 26: Guidelines for stopping accrual to the additional research Arm K

Activity Stage	Sig Level	Power	Targeted HR	Number of control arm events	Consider discontinuation if HR_K (observed) is…
I	0.40	90%	0.80	~104 M1 deaths	>0.965

9.7.5 Efficacy Stage II: Additional Research Arm K

The analysis of the final Efficacy Stage for this comparison will be performed when around 374 deaths have been observed for M1 patients randomised contemperaneously to the control arm. This would give 85% power to detect the targeted hazard ratio of 0.80 at a one-sided significance level of 0.025.

As with the intermediate activity, this analysis will include all patients in the comparison, with a separate subgroup analysis in M1 and M0 patients looking at consistency of effect. At this time point we predict <60 control arm M0 deaths will be observed. Further subgroup analyses, defined by the stratification factors, are planned to check for consistency of effect at intermediate and final analyses.

9.7.6 Sample Size For Additional Research Arm K

Consideration will be given to ceasing further randomisations to Arm K if it is not showing sufficient evidence of improvement on overall survival at the intermediate analysis.

We anticipate that around 1,800 patients, including around 1,100 M1 patients, are required over 3 years to observe ~374 control arm M1 deaths over around 8 years. This number and time will be dependent on the observed overall survival. The default scenario assumes (i) recruitment is constantly 100pts/m to the trial overall, (ii) co-recruitment throughout of the equivalent of one other research arm, and (iii) the majority of metastatic patients will also have docetaxel but non-metastatic patients will not.

Variations on these factors are documented in a Statistical Design Document. If accrual rates to the trial are at 150pts/m (as observed during summer 2013), accrual of around 1,800 patients to the comparison could be achieved within 2 years. These sample scenarios will also be documented in the Trial Master File.

Updating the standard-of-care to permit first-line use of docetaxel was assumed within the sample size scenarios and is reflected in the projected time to maturity of the “metformin comparison”.

9.7.7 Further Sample Size Issues For Additional Research Arm K

Careful consideration will be given to the emerging data from the "abiraterone comparison" when this reports in 2017.

Analyses for the “metformin comparison” will be timed from randomisation. The point of randomisation compared to the start of hormone therapy may differ, depending on the planned use of docetaxel. This practical information will be reviewed by the TMG and IDMC.

9.8 SAMPLE SIZE ISSUES AND TRIAL STAGES: ADDITIONAL RESEARCH ARM L

This is the "transdermal oestradiol comparison" and includes patients allocated to research Arm L (transdermal oestradiol ± RT ± docetaxel) and the equivalent, eligible patients contemporaneously allocated to the control Arm A (SOC).

The phase III evaluation of the clinical efficacy of transdermal oestradiol will ultimately be based on the relevant data from this comparison within STAMPEDE and the PATCH trial, combined using an individual patient data meta-analysis. The overall evaluation is based on a non-inferiority design.

9.8.1 Implementation And Outcome Measures: Additional Research Arm L

The transdermal oestradiol evaluation is based on the following approach.

9.8.1.A Earlier Stages In The PATCH Trial

- The early stages of the PATCH trial already demonstrated the safety and early activity of transdermal oestradiol in comparison to LHRH therapy (see **Appendix I**)(42). The pilot phase (completed in 2010, n=254) showed the rates of cardiovascular events in the transdermal oestradiol and LHRH arms were similar, and the castration rates were equivalent. These results were confirmed by longer-term data including nearly 900 patients enrolled up to Oct-2015.

- A pre-planned, confidential interim analysis undertaken in Jun-2013, based on progression-free survival, at the end of the Phase II component of the PATCH trial, led the PATCH IDMC to recommend further recruitment for an extension to Phase III. That analysis included 638 patients with 206 PFS events, and reviewed data against a pre-specified non-inferiority margin hazard ratio of 1.25 with a 1-sided alpha 0.25.

9.8.1.B STAMPEDE And PATCH Meta-analysis

- To assess the clinical efficacy of transdermal oestradiol, the relevant data from the STAMPEDE "transdermal oestradiol comparison" will be combined with that data from all patients recruited into PATCH; the data from STAMPEDE will not be analysed alone.

- As the eligibility criteria with respect to the timing of start of ADT differs between the STAMPEDE "transdermal oestradiol comparison" and the PATCH trial (see **Section 4.4.2**), the "transdermal oestradiol comparison" will undergo an initial Pilot Phase to assess castration rates and safety among those patients on Arm L. This will also include a safety review of patients receiving transdermal oestradiol in combination with docetaxel. The data will be reviewed by the PATCH IDMC when there are 30 patients in Arm L who have been followed up for at least 18 weeks. A feasibility review will also be performed at the same time.

- There will be an additional safety review for transdermal oestradiol used in combination with docetaxel, based in the first instance on data from the PATCH trial. This analysis will primarily assess cardiovascular events, but will also review other toxicities including neutropenia. This will be carried out when there are 30 research patients from the PATCH trial who have received both transdermal oestradiol and docetaxel as part of their first-line treatment and been followed up for at least 12 weeks (expected date around May-2017). These results will be made available to the STAMPEDE TMG and TSC, pending approval by the PATCH IDMC, and will inform whether an additional safety review of patients on transdermal oestradiol with docetaxel is required within both STAMPEDE and PATCH.

- The pre-planned Activity Stage II, on intermediate primary outcome measure progression-free survival, will take place based on combined data from the STAMPEDE "transdermal oestradiol comparison" patients and PATCH patients.

- The same approach will be used at the final Efficacy Stage, with progression-free and overall survival as definitive co-primary outcome measures (see PATCH Protocol v10.0 for further details). The rationale for choosing progression-free survival as both the intermediate primary outcome measure and as part of the definitive co-primary outcome measure for the "transdermal oestradiol comparison" is outlined in Section 9.8.3.

Table 27 summarises the outcome measures for each stage of this research comparison. The target sample size for the meta-analysis of the "transdermal oestradiol comparison" is approximately 2,000 patients, with around 500 to be recruited through the STAMPEDE "transdermal oestradiol comparison". By Feb-2017, around 1,200 patients had been recruited directly to the PATCH trial.

Table 27: Trial outcome measures by stage for the "transdermal oestradiol comparison"

COMPARISON STAGE	DATA SOURCE(S)	PRIMARY OUTCOME MEASURES	SECONDARY OUTCOME MEASURES
Pilot phase (completed 2010)	PATCH trial	Cardiovascular morbidity and mortality	Castration rates<br>Other toxicities<br>Metabolic effects
Activity Stage I (completed 2013)	PATCH trial	Progression-Free Survival*	Cardiovascular and other toxicities<br>Castration rates<br>Metabolic effects
Activity Stage II+$	PATCH and STAMPEDE trials	Progression-Free Survival*	Cardiovascular & other toxicities
Efficacy Stage III$	PATCH and STAMPEDE trials	Progression-Free Survival*<br>Overall survival	Cardiovascular & other toxicities<br>Prostate cancer specific survival<br>Quality-of-life

* Defined as the earliest among biochemical failure, clinical progression (local progression, lymph node progression, distant metastases), or death from any cause (see Section 9.8.3).

+ In addition, there is Pilot Phase to assess castration rates and safety among Arm L patients within STAMPEDE, since the eligibility criteria with respect to timing of start of ADT differs between the transdermal oestradiol comparison within STAMPEDE and the PATCH trial (see Section 4.4.2).

$ The timing of these analyses is determined by when a pre-specified number of events for the primary outcome measure have been observed in the control arms for the PATCH and STAMPEDE trials combined. Please see the PATCH Protocol v10.0 for further details.

9.8.2 Additional Use of Outcome Data from the "transdermal oestradiol comparison"

Patients allocated to the "transdermal oestradiol comparison" may provide additional consent to participate in translational sub-studies, see Section 4.6 for details. Subsequent correlative analysis using outcome data from these patients will be undertaken by the STAMPEDE team and collaborators, overseen by the STAMPEDE BRG and other STAMPEDE oversight committees.

9.8.3 Definition of PFS and Use As Co-primary OM: Additional Research Arm L

Note that the definition of progression-free survival (PFS) used within the "transdermal oestradiol comparison" analyses differs slightly to that of failure-free survival used for other research comparisons within STAMPEDE. This is because it includes death from any cause as an event- i.e. both PCa deaths and non-PCa deaths (see **Appendix D** for further details of the definition of progression). Progression-free survival is hence defined as time from randomisation to the first of: biochemical failure, clinical progression or death from any cause.

The use of PFS rather than FFS for the "transdermal oestradiol comparison" has no practical impact on STAMPEDE. The rationale for choosing PFS as part of the co-primary outcome measure for the "transdermal oestradiol comparison" is to capture any potential effects on survival due to the different toxicity profiles between transdermal oestradiol and LHRH.

Although PFS and survival are co-primary endpoints, their respective primary analyses will be triggered at different timepoints particularly because PFS is likely to contain a relatively low proportion of deaths as the contributing first PFS event.

9.9 FURTHER NOTES ON TRIAL DESIGN

9.9.1 Overall Sample Size

Given the adaptive nature of the study, there is no formal overall sample size target, but the numbers of patients required for each comparison are detailed in **Sections 9.4-9.8**. To date, more than 8,000 patients have been recruited overall and at least 10,000 patients will join the trial.

9.9.2 Factorial Design

We note here that we have not employed a factorial design in the original design of this trial because we anticipate the possibility of synergy between SOC, zoledronic acid and docetaxel and between SOC, zoledronic acid and celecoxib.

It would not be possible to assess any such interactions reliably in a factorial trial (see the Statistical Design Document for further details).

9.10 INTERIM MONITORING AND ANALYSES

The accumulating data will be reviewed at regular intervals (approximately annually) by an Independent Data Monitoring Committee (IDMC), including pre-specified formal intermediate analyses of activity data (see also **Section 16**). These analyses will be performed by the trial team at CTU. Only patients randomised contemporaneously, and eligible for that comparison, will be included in the comparison of each research arm against control e.g. patients allocated to the control arm prior to Protocol version 12.0 will not contribute to the "enzalutamide + abiraterone comparison" (Arm A vs Arm J). For the "transdermal oestradiol comparison", the relevant STAMPEDE data will only be analysed as a meta-analysis in combination with the PATCH trial. Therefore, interim data from this comparison will be reviewed by the PATCH IDMC.

The IDMC will be asked to give advice on whether the accumulating data from the trial justifies continuing recruitment of further patients or further follow-up; guidelines for discontinuation of accrual for the relevant Activity Stages, together with results from any other relevant trials will aid

them in this. A decision to discontinue recruitment, either in all patients or in selected subgroups, will be made only if the result is likely to convince a broad range of clinicians including those entering patients into the trial and the general clinical community. The intermediate stopping guidelines apply to the intermediate primary outcome measure.

To stop accrual early for benefit in any comparison would require convincing data in terms of the definitive primary outcome measure, overall survival. For example, this could be p<0.001 as proposed by Haybittle-Peto.(55, 56) The use of such a guideline for stopping for benefit has a minimal impact on the operating characteristics.

If a decision is made to continue without change, the IDMC will advise on the frequency of future reviews of the data on the basis of accrual and event rates. The IDMC will make recommendations to the Trial Steering Committee (TSC, see **Section 16**) as to whether the trial should continue in its present form. While the trial is ongoing the accumulating data will generally remain confidential, unless the TSC and IDMC agree that the data should be made public.

9.11 OUTLINE ANALYSIS PLAN

Analyses will be performed on an intention-to-treat basis. The standard unadjusted log-rank approach will be applied to analyses of intermediate and definitive primary outcome measures. The impact of potential confounders including the stratification factors used at randomisation will be considered in a Cox proportional hazard model. Flexible parametric models will be used to calculate the absolute differences between the arms to show treatment differences over time and to estimate restricted mean "survival" times (RMST). The estimated difference in restricted means survival time (RMST) will be used preferentially to compare treatment arms if the proportional hazards assumptions required for hazard ratios cannot be supported. The χ^2 test or Mann-Whitney test will be implemented for categorical data comparisons, including toxicity, as appropriate. Where relevant the primary outcome measure(s) (see **Section 9.2**) will be considered for all arms of the trial at each phase, but the main emphasis will be placed on the comparison of the research arms that have continued to recruit throughout the trial.

In the "transdermal oestradiol comparison," a meta-analysis approach will be used to combine data from the STAMPEDE and PATCH trials. The analysis will also take into account the change in randomisation ratio partway through the PATCH trial (from 2:1 for transdermal oestradiol versus LHRH before Feb-2011, to 1:1 thereafter). In addition, as the comparison uses a non-inferiority design, sensitivity analyses will be conducted based on a number of pre-defined definitions for the per-protocol population.

9.11.1 Pilot / Safety Phases

The Pilot Phase randomised patients between all the trial arms so that the results from these patients can be included in the main trial. Feasibility is considered in terms of acceptability of the trial randomisation and reported toxicities and adherence to trial medication. Centres participating in the Pilot Phase for the original research arms were required to keep an anonymised log of all patients assessed for trial eligibility (see Protocol version 2.0) so that the number of patients who did not participate in the study and the number of eligible patients who chose to not participate in the study could be summarised (reasons for non-participation were collected where the patients was willing). The anonymised logs are no longer needed for new research arms (since Protocol version 8.0).

For the patients who are randomised, we shall describe the incidence of expected and unexpected severe toxicities and adverse events/reactions (see **Section 11**) to decide whether to continue with research arms beyond the Pilot Phase.

9.11.2 Activity And Efficacy Stages

The approach to analysis of these stages is summarised within the sample size calculations (see earlier subsections of **Section 9**). Each research arm will be compared in a pairwise fashion against the contemperaneously recruited control arm.

Full details are available in the Statistical Analysis Plan. See **Figure 4** for an overview of the schema of progress.

10 MONITORING AND QUALITY ASSURANCE

10.1 MONITORING AT CTU

Data provided to the CTU will be checked for missing or unusual values (range checks) and consistency over time. If missing or questionable data are identified, staff at the CTU will request that the data be clarified. The exact procedures for data clarification and the amendment of CRFs will be described in the trial Data Management Plan and instructions will be sent to all STAMPEDE institutions as soon as they have been approved to participate in the trial. The CTU will also send reminders for any overdue data.

Anonymised copies of the initial patient's consent form and any subsequent re-consent should be sent to the STAMPEDE team at the CTU.

10.2 DIRECT ACCESS TO DATA

Collaborating institutions should be aware that direct access to patient data by CTU staff may be required for trial-related monitoring or audit. Patient consent for this will be obtained as part of the general trial consent process.

10.3 VISITS TO INVESTIGATOR SITES

A selection of institutions will be visited at least once during the course of the STAMPEDE trial. The CTU will give the responsible investigator adequate notice of the monitoring visit to allow adequate time, space and staff for these visits. The standard operating procedures (SOPs) for monitoring are available from the CTU.

After the monitoring visit the monitor will complete a site visit report. This report may be circulated to the TMT for comment. Once the TMT have reviewed the report and agreed on any recommendations the monitor will finalise the report and send a copy to the Principal Investigator (PI) at the site. A copy will be kept in the CTU STAMPEDE Trial Master File.

10.4 CONFIDENTIALITY

All information collected during the course of the research will be kept strictly confidential. In addition, all procedures for handling, processing, storage and destruction of data are compliant with the Data Protection Act 1998. No individual patients will be identified when results from the trial are published.

Patients will be asked for permission for information about their health status to be obtained from the Office of National Statistics (ONS) or via NHS Digital (formerly HSCIC) or similar or national equivalent by CTU, if necessary. In addition, patients will be asked for permission to inform their GP of their involvement in the STAMPEDE trial.

11 SAFETY REPORTING

ICH GCP requires that both investigators and sponsors follow specific procedures when reporting adverse events/reactions in clinical trials. These procedures are described in this section of the protocol. Further information on the expected toxicities for the trial interventions (docetaxel, zoledronic acid, abiraterone, radiotherapy, enzalutamide, metformin and transdermal oestradiol) can be found in Appendix C.

11.1 SAFETY REPORTING DEFINITIONS

The safety reporting definitions from ICH GCP apply in this trial protocol. These definitions are given in Table 28.

Table 28: Event Terms and Definitions

TERM	DEFINITION
Adverse Event (**AE**)	Any untoward medical occurrence in a patient or clinical trial patient to whom a medicinal product has been administered including occurrences which are not necessarily caused by or related to that product.
Adverse Reaction (**AR**)	Any untoward and unintended response to an investigational medicinal product related to any dose administered.
Unexpected Adverse Reaction (**UAR**)	An adverse reaction, the nature or severity of which is not consistent with the information about the medicinal product in question set out in the summary of product characteristics (or Investigator brochure) for that product.
Serious Adverse Event (**SAE**) or Serious Adverse Reaction (**SAR**) or Suspected Unexpected Serious Adverse Reaction (**SUSAR**)	Respectively any adverse event, adverse reaction or unexpected adverse reaction that:<br>• results in death<br>• is life-threatening*<br>• requires hospitalisation or prolongation of existing hospitalisation**<br>• results in persistent or significant disability or incapacity<br>• consists of a congenital anomaly or birth defect<br>• Other important medical condition***

Clarifications and Exceptions

* The term 'life-threatening' in the definition of 'serious' refers to an event in which the patient was at risk of death at the time of the event; it does not refer to an event which hypothetically might have caused death if it were more severe.

** Hospitalisation is defined as an inpatient admission, regardless of length of stay, even if the hospitalisation is a precautionary measure for continued observation. Hospitalisations for a pre-existing condition (including elective procedures that have not worsened) do not constitute an SAE.

*** Medical judgement should be exercised in deciding whether an AE/AR is serious in other situations. Important AE/ARs that are not immediately life-threatening or do not result in death or hospitalisation but may jeopardise the patient or may require intervention to prevent one of the other outcomes listed in the definition above, should also be considered serious.

Pregnancy occurring in a STAMPEDE patient's partner during the patient's participation in the trial must be reported to the CTU within the same timelines as an SAE and classified as an 'other important medical condition' on the SAE CRF. The outcome of a pregnancy should be followed up carefully and any abnormal outcome to the mother or child should be reported.

Patients who develop any new primary carcinomas should have the event reported on a SAE CRF as "other important medical condition" with the exception of non-melanoma skin cancer (e.g. basal cell carcinomas and squamous cell carcinomas) which do not require reporting.

11.1.1 Trial-Specific Exemptions

Events that fulfil the definition of serious e.g. result in hospital admission, but are due to disease progression or death as a result of disease progression are not considered to be SAEs. Do not complete an SAE CRF in this instance, instead details should be reported on the STAMPEDE Progression Log or Death Form only.

The following situations that fulfil the definition of an SAE are **excluded** from expedited notification on an SAE CRF and should be reported only on the STAMPEDE Follow-Up CRF:

- Elective hospitalisation and surgery for treatment of locally-advanced or metastatic prostate cancer or its complications
- Elective hospitalisation to simplify treatment or procedures

Elective hospitalisation for pre-existing conditions that have not been exacerbated by trial treatment do not need to be reported.

Furthermore, given the solid accumulated evidence on its safety profile (13, 57), hospitalisation or prolongation of hospitalisation for the following SOC docetaxel-related events is exempted from reporting:

- Febrile neutropenia
- Thrombocytopenia

However, it is expected that investigators continue to report the abovementioned expected reactions via the MHRA Yellow Card Scheme (https://yellowcard.mhra.gov.uk/).

The exemption does **not** apply for events resulting in death; these should be reported as per procedures detailed in Section 11.2.1.D.

11.2 INSTITUTION/INVESTIGATOR RESPONSIBILITIES

All non-serious AEs/ARs, whether expected or not, should be recorded on the Toxicity (symptoms) CRF linked to the Follow-Up CRF and sent to the CTU within one month of the corresponding Follow-Up CRF being due. SAEs/SARs should be notified to the CTU as described below.

The severity (i.e. intensity) of all AEs/ARs (serious and non-serious) in this trial should be should be graded using Common Terminology Criteria for Adverse Events (CTCAE) v4.0[4].

The complete CTCAE v4 .0 can be found at: http://evs.nci.nih.gov/ftp1/CTCAE/CTCAE_4.03_2010-06-14_QuickReference_5x7.pdf

4 ctep.cancer.gov/reporting/index.html

Any questions concerning this process should be directed to the STAMPEDE CTU team in the first instance.

11.2.1 Investigator Assessment

11.2.1.A Seriousness

When an AE/AR occurs the investigator responsible for the care of the patient must first assess whether the event is serious using the definitions given in Table 28. If the event is serious and not exempt from expedited reporting, then an SAE CRF must be completed and the CTU notified.

11.2.1.B Causality

The Investigator must assess the causality of all serious events/reactions in relation to the trial therapy using the definitions in Table 29. There are 5 categories: unrelated, unlikely, possibly, probably and definitely related. If the causality assessment is unrelated or unlikely to be related the event is classified as a SAE. If the causality is assessed as either possibly, probably or definitely related then the event is classified as a SAR.

Table 29: Assigning type of SAE through causality

RELATIONSHIP	DESCRIPTION	EVENT TYPE
Unrelated	There is no evidence of any causal relationship	Unrelated SAE
Unlikely	There is little evidence to suggest there is a causal relationship (e.g. the event did not occur within a reasonable time after administration of the trial medication). There is another reasonable explanation for the event (e.g. the patient's clinical condition, other concomitant treatment).	Unrelated SAE
Possibly	There is some evidence to suggest a causal relationship (e.g. because the event occurs within a reasonable time after administration of the trial medication). However, the influence of other factors may have contributed to the event (e.g. the patient's clinical condition, other concomitant treatments).	SAR
Probably	There is evidence to suggest a causal relationship and the influence of other factors is unlikely.	SAR
Definitely	There is clear evidence to suggest a causal relationship and other possible contributing factors can be ruled out.	SAR

11.2.1.C Expectedness

If the event is a SAR the Investigator must assess the expectedness of the event. Please refer to Appendix C Table 6 when recording expectedness. This summary table compiles all the recognised undesirable toxicites listed in the reference information i.e. the Summary of Product Characteristics or Investigator Brochure for all research treatments. If the suspected drug reaction is a listed toxicity in this summary table, it will be consider 'expected'. If a SAR is not a recognised adverse reaction, it should be recorded as unexpected and therefore it becomes a SUSAR. It should be noted that a recognised toxicity may still be considered as 'unexpected' if the severity or duration is worse than that described in the reference information.

11.2.2 Notification

Arm A: SAEs occurring in patients randomised to the control Arm A (SOC) must be reported until 30 days after last ADT injection or progression (whichever is sooner)

All Research Arms
Investigators must notify the CTU of all SAEs occurring from the time of randomisation until 30 days after the last administration of research treatment. For patients not on research treatment (i.e. research treatment has been stopped, or was never started) but who remain on ADT alone, SAEs should be reported until progression and second line treatment has been started. Therefore, post-progression SAEs only need reporting if trial treatment is ongoing or has stopped in the past 30 days.

SARs and SUSARs must be notified to the CTU indefinitely for all arms (i.e. no matter when they occur after randomisation).

Notification checklist

Before sending the SAE CRF please check that the information provided meets **all** of the following minimum criteria required for initial processing and review:

1. At least two patient identifiers
2. Indication of why the event was serious
3. Grade severity of event/reaction according to CTCAE version 4.0
4. Assessment of causality in relation to all treatment (trial and SOC)
5. Assessment of expectedness (refer to list of expected toxicities in **Appendix C Tables 6 and 7**)
6. Provide the date of last administration for all treatments (minimum month/year) i.e. hormone therapy, docetaxel and where applicable trial treatment(s).
7. Ensure SAE CRF is signed (if not by a clinician, by a site trial team member in the first instance)

SAE REPORTING
Fax to 020 7670 4818 within 24 hours of becoming aware of the event
Or send via **encrypted** email to mrcctu.stampede@ucl.ac.uk

The STAMPEDE trial team will confirm receipt of the SAE report to the main point of contact via email. Contact the STAMPEDE trial team if receipt is not received within 24 hours.
Follow-Up: Patients must be followed-up until clinical recovery is complete or stabilised. Follow-Up should continue after completion of protocol treatment if necessary. Follow-Up information can be updated on the original SAE CRF by ticking the box marked 'follow-up' and faxing to the CTU as information becomes available. Extra, annotated information and/or copies of test results may be provided separately. The patient must be identified by trial number, date of birth and initials only. The patient's name should not be used on any correspondence.

11.3 CTU RESPONSIBILITIES

Medically qualified staff at the CTU and/or the Chief Investigator (or a medically qualified delegate) will review all SAE reports received. The causality assessment given by the local Investigator at the hospital cannot be overruled and in the case of disagreement, both opinions will be provided in any subsequent reports.

The CTU is undertaking the duties of trial sponsor and is responsible for the reporting of SUSARs and other SARs to the regulatory authorities (through the MHRA to competent authorities in other European member states) and the UK research ethics committees; and in addition has sponsor oversight for reporting in other countries in which the trial is taking place.

The CTU will also keep all investigators informed of any safety issues that arise during the course of the trial.

12 ETHICAL CONSIDERATIONS AND APPROVAL

12.1 ETHICAL CONSIDERATIONS

12.1.1.A Randomisation

This is a randomised trial therefore neither the patients nor their physicians will be able to choose the patients' treatment. Treatment will be allocated randomly using a computer-based algorithm. This is to ensure that the groups of patients receiving each of the different treatments are as similar as possible.

All patients, with the exception of those allocated to transdermal oestradiol (Arm L), will receive standard hormone treatment. All patients, including those allocated to Arm L, may also receive other standard-of-care treatments which may include prostate radiotherapy and/or docetaxel. Use of radiotherapy and/or docetaxel will be unaffected by trial participation and is left to the discretion of the treating clinician and patient. Patients may be randomised to receive additional treatment (metformin) given with standard-of-care treatments, or an alternative form of hormone treatment (transdermal oestradiol). An even allocation ratio is being currently being used which means all eligible patients have an equal chance of being randomised to the control or research arms.

Through the introduction of a "transdermal oestradiol comparison" into the STAMPEDE trial platform, sufficient data will be collected to evaluate this treatment approach more rapidly. By undertaking a meta-analysis using data collected in both PATCH and STAMPEDE, fewer patients overall are allocated the control arm i.e. more patients gain access to novel treatments.

12.1.1.B Evaluation of Novel Therapeutic Strategies

The newer treatment options are being assessed in a detailed and systematic fashion in this trial. There is some evidence to suggest that the newer treatment options may have advantages over standard treatment alone with regards to clinical outcome, but this is not confirmed and toxicity may be increased. This trial will follow a large group of men who have been randomly allocated to either the standard treatment(s) or the novel treatment strategies in order to measure the benefits of these approaches. The patients will also be followed-up for toxicity and safety issues, so that any benefits can be weighed against any negative aspects including the impact treatments have on other aspects of medical health e.g. cardiovascular disease, as well as quality-of-life and value for money (health economic analysis).

12.1.1.C Additional Tests and Hospital Visits

Patients participating in the trial will have some additional hospital visits and some extra blood samples taken compared to patients who are not participating in the trial, with the amount varying according to the allocated treatment and stage of disease. Sometimes the blood samples can be taken when the patient is attending hospital for treatment anyway. On some of the trial arms, the patient may have to make additional visits to the hospital for the blood sample to be taken, although in some cases it may be possible for the blood sample to be taken in the GP's surgery.

12.1.1.D Facilitating Participant Feedback From Investigations and Additional Analyses

For participants who choose to take part in additional sub-studies, biological samples including blood, saliva and remaining stored FFPE tumour samples will be used in research projects. These projects will enable the study of genetic factors and other biomarkers that can help identify individuals who serve to benefit most from the treatments tested in STAMPEDE and to further understand why and how treatment resistance develops. All samples will be link-anonymised and only made accessible to approved collaborators granted access by the STAMPEDE oversight

committees. We will make every effort to protect the confidentiality of this information and make sure personal identities are protected.

From Protocol version 16.0, patients may opt to receive feedback regarding genetic results that may arise from analyses of research analyses of genetic material extracted from any of the biological samples collected as part of the trial e.g. saliva, FFPE tumour blocks or circulating tumour DNA extracted from blood. Only results which are of established clinical relevance and for which testing would be available under standard NHS genetic testing guidelines will be fed back. Any genetic analysis undertaken as part of additional research associated with STAMPEDE does not replace clinically indicated investigations as only a proportion of STAMPEDE patient will undergo prospective testing and therefore it cannot be guaranteed that results will be fed back in a timely fashion.

This change has been made in response to emerging data that demonstrates a small proportion of men may have genetic faults in genes such as BRCA2. This has implications for both patients and potentially their biological relatives. For patients and their treating clinician, knowledge of this information may facilitate access into further clinical trials and may potentially impact on the choice of treatment following progression i.e. metastatic castrate-resistant prostate cancer.

Any patient who consents to receive feedback and in whom a known pathogenic mutation of clinical significance is detected on testing of research samples collected as part of STAMPEDE will be told of this. Patients will be recommended to undergo genetic counselling accessed via clinical genetics services and consider confirmatory testing. This is necessary to determine if the defect is germline (inherited) and ensures access to appropriate ongoing support. If confirmed as a germline (inherited) abnormality, this will enable biological relatives to also access appropriate genetic counselling and testing if they wish.

However, patients and STAMPEDE investigators are informed that any genetic analysis undertaken as part of additional research associated with STAMPEDE does not replace clinically indicated investigations as only a proportion of STAMPEDE patient will undergo prospective testing and therefore it cannot be guaranteed that results will be fed back in a timely fashion. Therefore participation in the additional research conducted on biological samples collected as part of STAMPEDE should not impact on an clinicians decision to recommend genetic screening.

The introduction of the “metformin comparison” means that all patients, not known to be diabetic, will be screened for diabetes prior to trial entry. This is to enable the effect of metformin to be studied in non-diabetic patients. All patients in whom screening bloods are abnormal will be referred for confirmatory tests and further management according to local guidelines e.g. via their GP. Screening is expected to lead to a small proportion of potential trial participants receiving a new diagnosis of diabetes but will ensure appropriate management of both conditions.

12.1.1.E Considering the Impact of Emerging Data

If new information emerges during the course of the trial which may affect the treatment or follow-up of patients who have joined the trial, information will be provided through by the trial team to all Principal Investigators. PIs therefore have the duty to inform the patients in their care of any new information emerging using any appropriate channel (e.g. letter, communication at follow-up clinic, etc).

12.2 ETHICAL APPROVAL

The protocol has a Favourable Opinion from an appropriate Research Ethics Committee, according to national guidelines. Additionally, each site must also obtain management permission for research (Local R&D approval or equivalent) from the relevant host organisations before patients can be entered into the trial. The patient's informed consent to participate in the trial should be obtained after a full explanation has been given of the treatment options, including the conventional and generally accepted methods of treatment. Patient information sheets and patient consent forms are available on the STAMPEDE website (www.stampedetrial.org).

The right of the patient to refuse to participate in the trial without giving reasons must be respected. After the patient has entered the trial, the clinician must remain free to give alternative treatment to that specified in the protocol, at any stage, if he feels it to be in the best interest of the patient. However, the reason for doing so should be recorded and the patient will remain within the trial for the purpose of follow-up and data analysis according to the treatment option to which he has been allocated. Similarly, the patient must remain free to withdraw at any time from the protocol treatment without giving reasons and without prejudicing his further treatment.

A statement of MRC policy on ethical considerations in clinical trials of cancer therapy, including the question of informed consent, is available from the MRC Head Office web site (http://www.mrc.ac.uk). In addition, the MRC and the Wellcome Trust framework on the feedback of health-related findings in research is readily available (https://www.mrc.ac.uk/documents/pdf/mrc-wellcome-trust-framework-on-the-feedback-of-health-related-findings-in-researchpdf/) and has been used when developing the trial specific processes.

13 REGULATORY APPROVAL

This trial has been approved in the UK by the MHRA and will be conducted under a CTA (Ref: 00316/0026/001-0001) in the UK.

The trial has been approved in Switzerland by Swissmedic (Ref: 2009 DR3235).

14 INDEMNITY

University College London holds insurance against claims from participants for injury caused by their participation in this clinical trial. Participants may be able to claim compensation if they can prove that UCL has been negligent. However, as this clinical trial is being carried out in a hospital, the hospital continues to have a duty of care to the participant of the clinical trial. University College London does not accept liability for any breach in the hospital's duty of care, or any negligence on the part of hospital employees. This applies whether the hospital is an NHS Trust or otherwise.

Participants may also be able to claim compensation for injury caused by participation in this clinical trial without the need to prove negligence on the part of University College London or another party. Participants who sustain injury and wish to make a claim for compensation should do so in writing in the first instance to the Chief Investigator, who will pass the claim to the managing organisation's Insurers, via the managing organisation's office.

Hospitals selected to participate in this clinical trial must provide clinical negligence insurance cover for harm caused by their employees and a copy of the relevant insurance policy or summary can be provided on request.

15 FINANCE

STAMPEDE is funded by Cancer Research UK's Clinical Research Committee (formerly the Clinical Trials Advisory Awards Committee; CTAAC). It is also funded by the MRC through the MRC Clinical Trials Unit at UCL. The trial has National Institute for Health Research Clinical Research Network (NIHR CRN) approval and, therefore, local NCRN funds may be available at each centre to support entry of patients into this trial.

Funding arrangements for research arms now closed to recruitment can be found in **Protocol version 13.0**

Standard therapies including **ADT**, **prostate radiotherapy** and **docetaxel** will be administered as per routine clinical care using local NHS supplies.

Abiraterone is manufactured by Janssen Pharma PV (pharmaceutical companies of Johnson & Johnson). They have agreed to provide free drug and funds to distribute drug to participating sites and to help support the conduct and management of the trial.

Enzalutamide is manufactured by Astellas Pharma. They have agreed to provide free drug and funds to distribute drug to participating sites and to help support the conduct and management of the trial.

Metformin will be administered using local NHS supplies.

Transdermal oestradiol will be administered as Progynova TS 100 patches, manufactured by Bayer who have agreed to supply these patches at a trial-specific discounted price. All accredited STAMPEDE centres will be able to order Progynova patches through AAH Pharmaceuticals Ltd wholesalers at the discounted rate.

Biomarker-Screening Pilot will be funded Clovis Oncology who will fund all sample analysis and help support the coordination of sample retrieval including site reimbursement through an educational grant to the MRC CTU at UCL.

16 TRIAL COMMITTEES

16.1 TRIAL MANAGEMENT GROUP (TMG)

A Trial Management Group (TMG) has been formed comprising the Chief Investigator, other co-investigators and members of the CTU. The membership of the TMG may be expanded if other groups of trialists wish to participate. It will also be amended during the trial if other circumstances require e.g. retirement.

The TMG will be responsible for the day-to-day running and management of the trial. They will meet by teleconference at least 3-monthly and in person as needed. The TMG members are detailed in **Appendix F**.

Further details of TMG functioning are provided in the TMG charter (available on request).

16.2 TRIAL STEERING COMMITTEE (TSC)

A Trial Steering Committee (TSC) has been formed to provide overall supervision for the trial and provide advice through its independent chair. The ultimate decision for the continuation of the trial lies with the TSC. The TSC will meet regularly.

Further details of TSC functioning are provided in the TSC charter (available on request).

16.3 INDEPENDENT DATA MONITORING COMMITTEE (IDMC)

An Independent Data Monitoring Committee (IDMC) has been formed. The IDMC will be the only group who sees the confidential, accumulating data to the trial. Reports to the IDMC will be produced by the CTU. The IDMC will meet within 6 months of the trial opening with the frequency of meetings dictated by the IDMC. The IDMC will consider data in accordance with the analysis plan (see **Section 9**) and will be advisory to the TSC. The IDMC can recommend premature closure or reporting of the trial, or that recruitment to any research arm is discontinued.

From Protocol version 8.0 onwards, any recommendation from the IDMC to stop recruitment to one or more trial arms will be acted upon immediately, pending ratification from the TSC. As this period between meetings should be very short, sites would not be notified until after the TSC have made a decision. IDMC recommendations based on emerging safety issues will be discussed with sites promptly.

Further details of IDMC functioning and the procedures for interim analysis and monitoring are provided in the IDMC charter (available on request).

Data from the “transdermal oestradiol comparison” are viewed by the PATCH IDMC, in meta-analysis with PATCH, rather than by the STAMPEDE IDMC. Recommendations of any actions relating to STAMPEDE would be made to the STAMPEDE TSC.

16.4 TRIAL EXPERT PANELS

The trial has two established translational expert groups chaired by TMG members the Biological Research Group (BRG) and the Metabolic Translational Group (MTG). Both groups input and provide expert oversight of relevant translational aspects of the trial and associated sub-studies.

Figure 5: Diagram of relationships between trial committees

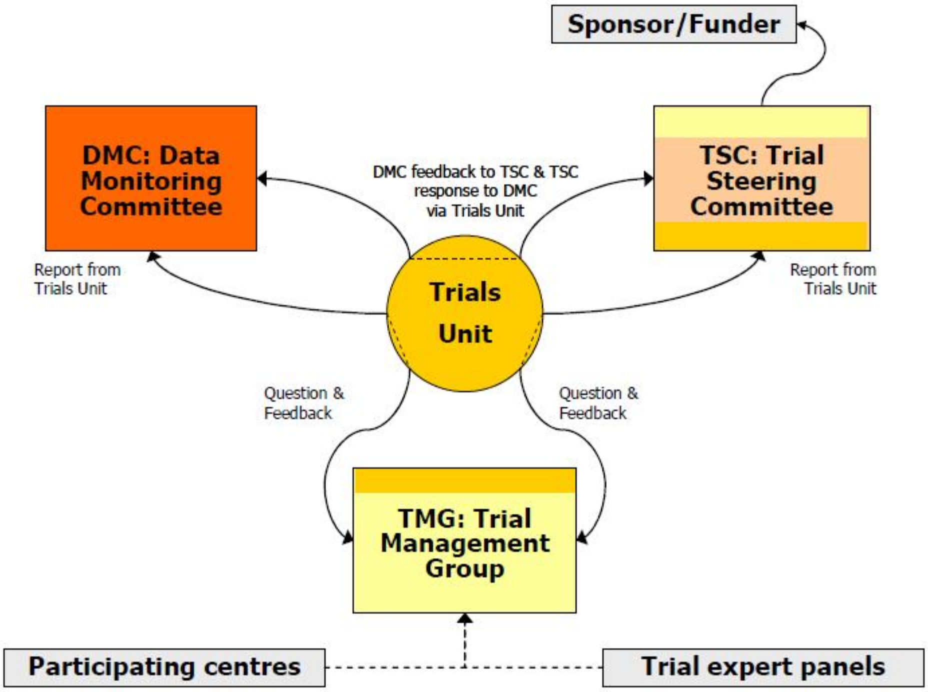

17 ANCILLARY STUDIES

17.1 QUALITY-OF-LIFE

A quality-of-life (QL) study is being performed to assess the impact of each treatment arm on the quality of patient's lives. Initial participation in this study was limited to the first 700 patients recruited (this was reached in Sep-2008) patients. The QL study re-opened from the implementation of Protocol version 8.0.

The EORTC QLQ-C30 with the prostate-specific module QLQ PR25 will be used. Key items for assessment are pain reduction for patients with metastatic disease and urinary symptoms for patients with locally-advanced disease. In addition specific hypotheses will be generated for each of the research arms. The EuroQol (EQ-5D) will be used in the study as a generic measure of health-related quality-of-life which can be linked to public preferences. These data will be used to calculate quality-adjusted life-years as part of the economic evaluation (see **Section 17.2**). Patients recruited into the QL study, should continue to complete QL data for five years after randomisation or until progression, whichever is sooner. Questionnaires should be self-administered, although it is recommended that a key person (e.g. research nurse) at each centre be responsible for the data collection to optimise compliance and completeness of the data.

The QL and the HE questionnaires should be completed by the patient without conferring with friends or relatives and all questions should be answered even if the patient feels them to be irrelevant.

The responsible person should check each questionnaire for its completeness, ensuring that the correct date of completion and patient identifiers are present. The research nurse should approach patients at appropriate clinical visits to complete a questionnaire. If no clinical visit is scheduled for the patient (with a window of 4 weeks around the expected date) the nurse should organise the completion of the questionnaire, by post or by a visit to the patient at home (or in a hospice).

17.2 HEALTH ECONOMICS

A health economics (HE) sub-study will be performed. Core resource use information will be collected, using CRFs on days in hospital (by speciality) and outpatient visits. Data collected on concomitant medication will also be used in the economic analysis. Information on patients' use of primary care and community-based services will be collected as additional questions in the QL questionnaire. Costs will be calculated on the basis of representative UK unit costs at the point of analysis. Health outcomes will be assessed in terms of quality-adjusted life years (QALYs). Quality adjustments will be based on patients' responses to the EQ-5D health status measure which will be administered at baseline and each point of follow-up as part of the QL questionnaire. A cost-effectiveness analysis will compare all regimens that continue to recruit into their final Efficacy Stage. For further details please refer to **Appendix G**.

17.3 TRANSLATIONAL SUB-STUDIES

Samples obtained from consenting STAMPEDE participants are analysed as part of separate translational sub-studies. These are conducted through collaborations with other academic and

industry partners. All applications for collaboration and sample accesss are reviewed by the STAMPEDE oversight committees and are overseen by the STAMPEDE BRG. For further details of each ongoing substudy please see **Section 4.6**. For details regarding sample collection please refer to the **Sample collection and handling manual**.

18 PUBLICATIONS

The results from different centres will be analysed together and published as soon as possible. Individual clinicians must not publish data concerning their patients that are directly relevant to questions posed by the study until the TMG has published its report. The TMG together with the STAMPEDE collaborators will form the basis of the writing committee and decide on the nature of publications. For the "transdermal oestradiol comparison", as the efficacy analyses will based on relevant data from the STAMPEDE and PATCH trials, TMGs for the two studies will form the writing committee. Any release, of efficacy or safety data, presentation or publication will be agreed with the TSC according to the terms of their charter.

All publications will acknowledge the participating centres and clinicians, and these will be detailed in an appendix to the main report. Papers will have named authors determined by the TMG according to the following principles:

- To be as inclusive as possible where this is practicable
- To ensure that there is justification for anyone to be named as an author

Reasons for nomination for authorship may include: trial design; grant holding; day-to-day trial oversight (TMG membership); analysis; discussion and interpretation of data; representation for key groups; active participation at large recruiting sites.

It should be accepted that the people qualifying for authorship will vary over time. In addition, key positions will vary depending on the nature of the publication: clinical lead for clinical papers, statistician lead for methodology papers, translational papers may be led by authors not on the main TMG if appropriate (e.g., the bone sub-study). In the event of any dispute related to authorship or data release, the TSC will be responsible for making the executive decision.

In the manuscript, a full list of sites and the number of patients recruited will be provided. In the presentations, this list of sites will also be shown. The term "the STAMPEDE investigators" will clearly be stated and relevant names included in the presentation credits.

A detailed **Publication Plan** is documented separately.

19 PROTOCOL AMENDMENTS

19.1 PROTOCOL

19.1.1 Amendments Made To Sections In Protocol Version 1.0 (May-2004)

Administrative changes such as typos, word change etc.

Name additions/changes to:

TMG members

TSC members

IDMC members

'General Information' Section – additional information re. Abridged version of protocol

Section 1.2 – Figure 1, Celecoxib duration amended

Section 1.3 – Figure 2, addition of cardiovascular assessment form, name and timings amended

Section 2.3 – Docetaxel information updated

Section 2.4 – Additional text re dose and duration justification for Celecoxib use.

Section 3 – Title change and content updated

Section 4.2 – New exclusion criteria added

Section 4.3.1 – New investigations added and additional text re testosterone measurements and additional text re. prior celecoxib treatment

Section 6.1.4 – Celecoxib duration amended

Section 6.1.5 – Additional text re. Co-administration of docetaxel and bisphosphonates

Section 6.1.6 – Celecoxib duration amended

Section 6.2.2 – additional docetaxel information

Section 6.2.3 – addition of CV event history

Section 11 – Safety reporting updated

Section 12.1 – Additional text re. the collection of blood for genetic and serum marker studies

Section 15 – Additional information re. Central Subvention for docetaxel arms

19.1.2 Amendments Made To Sections In Protocol Version 1.1 (May-2005)

Section 6.2 Administration and Dose Modifications, subsection 6.2.1 Zoledronic Acid

19.1.3 Amendments Made To Sections In Protocol Version 2.0 (Jun-2005)

General Information section – SAE reporting fax number and timeframe added.

Section 1.2 – Addition of anti-androgen use for M0 patients as a method of HT

Section 1.2 – Increase in amount of blood needed & addition tissue sample request.

Section 1.3 Trial Documentation updated to include new table detailing trial documentation ahead of accreditation, the inclusion of the radiotherapy forms and correct case report form timings

Section 2.1 – Addition of anti-androgen use for M0 patients as a method of HT

Section 4.1.3 – Inclusion criteria Vii "Normal testosterone prior to hormone treatment" removed.

Section 4.1.3 - φ note has been omitted and moved to section 4.2 (see number 8)

Section 4.2 – Exclusion criteria added to exclude patients with active peptic ulceration, gastrointestinal bleeding and inflammatory bowel disease.

Section 4.2 – Exclusion Criteria added to exclude patients with planned major dental work

Section 4.3.1 - All blood test timelines changed from 14 days to 28 days.

Section 4.3.1 – Hormone Therapy pre-randomisation deadline extended from 4 weeks to 12 weeks.

Section 4.3.1 – Additional information regarding the use of NSAIDs and cox-2-inhibitors before coming on to the STAMPEDE study and once commenced on study treatment

Section 4.3.2 – Updated to ask for all vitamins and minerals the patient is taking to be recorded.

Section 4.3.3 – Updated to include the extra blood required and the request for consent of patients' tissue samples.

Section 6.1.1 – Addition of anti-androgen use for M0 patients as a method of HT

Section 6.1.6 – Addition of the calcium & vitamin name "calcichew".

Section 6.6.2 – asking also to collect vitamins and minerals under concomitant medication.

Section 6.6.3 – New section to inform investigators that patient's, who they wish to give radiotherapy to, are also eligible for STAMPEDE

Section 6.6.4 – New section to detail what data is being collected on the radiotherapy given to patients.

Section 7.1; figure 4 – Addition of radiotherapy form and in note, addition of AA alone

Section 7.1.2 – omission of repeated scans and x-rays at 24 weeks, also omitted in note under figure 4.

Chapter 11 – Safety reporting section updated

Section 17.3 – Increase in amount of blood needed & additional tissue sample request.

19.1.4 Amendments Made To Section In Protocol Version 3.0 (Jul-2006)

Front Cover - NCRN logo added for accuracy

Front Cover - Clarification that protocol developed with NCRI rather than on behalf of

Front Cover - Clarification that it is a 6 arm trial

General Information section - MRC CTU staff section updated

Section 1.2 – Statistics section updated.

Section 1.2 - Additional research paragraph updated to reflect additional studies and for clarification of terms

Section 1.2 - Blood collection volume changed to reflect new technique used

Section 1.3 (figure 3) - Table showing case report form schedule updated to reflect clarification of follow-up schedule and addition of new CRF (End of Treatment)

Section 2.2 - AS changed to HT (clarification of terms)

Section 2.3 - Updated in information in regard to use of docetaxel added to reflect up to date practice

Section 2.5 - Sub-headings numbered for consistency

Section 3.0 - Information in regard to the Pilot Phase now written in past tense as Pilot Phase has now been completed

Section 4.1.1 - Inclusion criteria extended so that patients who fulfil 2 out of the three of the first inclusion criteria can be eligible.

Section 4.3.1 - Change in time scales by which baseline investigations need to be completed.

Section 4.3.1 - Clarification that chest X-ray is only required if chest is not included in the CT

Section 4.3.1 - Removal of 12 week timeline for baseline PSA test to be performed. (Stipulation that it must be performed before start of HT)

Section 4.3.2 – Information added in regard to time allowed from randomisation to start of treatment

Section 4.3.3 - Additional research paragraph updated to reflect additional studies and for clarification of terms

Section 4.3.3 - Blood collection volume changed to reflect new technique used

Sections 6.1.2-6.1.6 - Androgen Suppression replaced with hormone therapy for consistency of terms

Section 6.2.2 - '(Taxotere)' Removed for consistency

Section 6.2.2 _ information added in regard to the need to closely monitor liver function prior to docetaxel administration

Section 7.1 - Page number reference updated

Section 7.1.1 - PSA measurement timings updated to accurately reflect follow-up schedule

Section 7.3 (Table 4) - Table and key updated to accurately reflect follow-up schedule and to include information about new CRFs and removal of withdrawal CRF

Section 8 - Rewording for clarification of definition of trial withdrawal

Section 8.1 - Instruction that withdrawal from trial treatment should be recorded on End of Treatment Form rather than withdrawal form

Section 8.1 - Information updated to emphasise that trial treatment must be discontinued following a progression

Section 8.2- Information added in regard to patient transfers

Section 8.3 - Instruction that withdrawal from trial completely must be notified in writing to the MRC CTU rather than included on withdrawal form

Section 9 and Summary – Target event numbers updated to reflect the slightly revised numbers obtained by using –nstage- which is the new, recommended program for MAMS trials

Sections 11.1 and 11.2 - Form numbers removed to allow for future changes in numbering

Section 11.2 – Reference to toxicity grading website added

Section 11.2.1 - Reference to table in appendix G added

Section 12.2 - 'Suggested' removed from 'Suggested patient information sheets'

Section 13 - CTA reference added

Section 17.3 - Information added to reflect new blood collection method for DNA analysis and in regard to additional translational studies for which funding has recently been approved

19.1.5 Amendments Made To Protocol Version 4.0 (Dec-2007)

General Information Section - Randomisation and SAE reporting details sections clarified

Section 1.2 and throughout protocol - Efficacy Stages 1-111 renamed to Activity Stages 1-111 for accuracy and clarity

Section 1.2 - Follow schedule corrected

Section 4.1.2 - Inclusion criteria widened to include high risk relapsing patients, that would not have met the previous PSA based criteria

Section 4.1.3 - Note added to reference location of WHO performance status definitions

Section 4.2 - Notes added to reference locations of toxicity gradings and NYHA classifications

Section 4.3.1 - Timings of baseline scan information changed to accurately reflect most common current practice

Section 6.1.1 - Information about use of LHRH antagonists to ensure that the protocol accurately reflects current and future practice

Section 6.1.1 - Information about suggested duration of hormone therapy added to ensure that the protocol accurately reflects current practice

Section 6.2.2 - Additional information added about the timing of liver function tests prior to docetaxel administration added for clarity

Section 6.6.4 - Information on radiotherapy data collection added

Section 7.1.1 - Erroneous information about the timing of PSA measurements removed

Figure 3 - Moved to new section in protocol for clarity and extended to include current information on data collection

Figure 3b - Added to describe how extent of data collection during follow-up should change, post treatment and post progression

Figure 4 - Notes added to explain the changes in data collected at follow-up and to information that the quality-of-life study will be applicable to the first 700 patients randomised only

Figure 4 - Note added to include palliative radiotherapy CRF

Section 11.3 - SAE reporting information updated

Section 19 - Protocol amendments list updated

19.1.6 Amendments Made To Protocol Version 5.0 (Aug-2008)

1. General Information Section – Randomisation phone line number updated – non UK extension added
2. Section 3 – Information about QL study removed to reflect closure of QL study after first 700 patients
3. Section 4.2 – Exclusion criteria clarified to explain that only patients with severe poor cardiovascular history should be excluded
4. Section 4.3.1 – Information on co-administration of NSAIDS with celecoxib changed based on clinical advice.
5. Section 5 - Randomisation phone line number updated – non UK extension added
6. Section 6.2.1. – Information added to clarify that patients who develop an osteonecrosis of the jaw should stop zoledronic acid treatment
7. Section 6.2.3 – 'severe' text added to accurately reflect which patients should be excluded based on their cardiovascular history

8. Section 7.1.2 – Definition of disease progression extended for clarity

9. Figure 3 – Updated to include reference to newly created skeletal related event form

10. Figure 4 – Previous error in table amended to show that the 4th Zoledronic Acid form that is submitted contains information about 3 cycles rather than 2 as previously indicated

11. Table 4 – 'Other important medical condition' added to definition of serious in the SAE section, to accurately reflect SAE form and current practice

12. Section 11.1 – Information added on reporting or pregnancies

13. Section 17 - Information about QL study removed to reflect closure of QL study after first 700 patients

19.1.7 Amendments Made To Protocol Version 6.0 (Jul-2009)

1. General Information Section – Trial Pharmacist removed and changes of:

Co-Investigator

Patient Representatives

Trial Manager

Data Manager

General Information Section - Coordinating Centre – address change

General Information Section – change of Sponsor address

Section 1.1 – ratio of patients randomised to the investigational arms updated

Section 1.2 – figure 1b added to clarify trial design from Apr-2011 onwards

Section 1.2 – paragraph added to explain trial changes after the second activity analysis

Section 1.2 – wording added to clarify that QL data only collected for first 700 patients randomised

Section 1.3 – SSA Favourable Opinion removed from list of trial documentation required ahead of site accreditation

Section 2.1 – Amount of men diagnosed with prostate cancer annually updated

Section 2.4 –note added to explain completion of recruitment to celecoxib- containing arms

Section 2.5.2 - note added to explain completion of recruitment to celecoxib- containing arms

Section 3 – SSA Favourable Opinion removed

Section 4.2 – Exclusion criterion xiii greyed out

Section 4.3.1 – paragraph removed regarding potential randomisation to celecoxib-containing arms

Section 5 – Randomisation instructions expanded to exclude public holidays or dates when notice has been given by the CTU

Section 6.1.4 – formatting changed to grey font to reflect recruitment completion for arm D

Section 6.1.6 - formatting changed to grey font to reflect recruitment completion for arm F

Section 6.2.3 – recruitment note added

Section 6.6.3 – radiotherapy statement changed to reflect data from recent trials

Section 7.1.2 – removal of reference to SRE- specific CRF

Section 7.3 – Figure 3 - Addition of Bone Density Risk Factor Form and BMD sub-study assessment forms to summary of timing table

Section 7.3 – Figure 4 – Weeks added to timings of assessments post 2 years

Section 7.3- Figure 4 – note added to explain recruitment completion for arms D and F

Section 12.1 – Wording changed to reflect change to randomisation allocation ratio

Section 12.1 – Addition of statement regarding new information emerging during the trial

Section 12.2 – Reference to SSA removed

Section 16.3 – Statement added regarding actioning IDMC recommendation ahead of TSC ratification

19.1.8 Amendments Made To Protocol Version 7.0 (Jul--2011)

1. General Information Section- SAE reporting fax number corrected

2. Section 11- SAE reporting fax number corrected

19.1.9 Amendments Made To Protocol Version 7.1 (Jul-2011)

Throughout protocol – numbering has been updated in some sections new accommodate new information that has been added.

General Information Section – contact details updated

General Information Section – Funding information updated to include involvement from additional company

General Information Section – Wording on compliance and regulations updated to reflect current MRC CTU standard wording

General Information Section – Abbreviations list updated

Section 1.1 – The number of investigational agents being studied updated from three to four

Section 1.1 – Information regarding celecoxib updated to reflect that recruitment to these arms was discontinued in Apr-2011

Section 1.1 – Information about new IMP, Abiraterone inserted

Section 1.1 – Sample size and trial duration information updated to reflect changes brought about by additional trial arm

Section 1.2 – Summary information updated to reflect the discontinuation of recruitment to celecoxib arms and the addition of abiraterone

Figures 1a, b and c - Updated to reflect the discontinuation of recruitment to celecoxib arms and the addition of abiraterone

Section 1.2 – Information on trial stages updated to reflect changes brought about by additional trial arm

Section 1.2 – Information updated regarding the re-opening of the quality-of-life sub-study from implementation of protocol version 8.0

Section 2.1 – Wording related to hormone therapy updated for clarity

Section 2.1 - Updated to reflect the discontinuation of recruitment to celecoxib arms and the addition of abiraterone

Section 2.2 – Updated references added

Section 2.3 – Updated references added

Section 2.5 – Section added to give background information on new IMP, abiraterone

Section 2.6.1 – Updated references added

Section 2.7 – Section added to give information regarding radiotherapy which is to be given as part of standard care following recently published trial data.

Section 3 – Wording updated regarding selection of investigators to reflect current MRC CTU practice

Section 4.1 – Inclusion criteria updated with new criterion regarding radiotherapy use

Section 4.1 - Inclusion criteria updated with new criterion regarding contraceptive use

Section 4.1 – Wording of inclusion and exclusion criteria updated for clarity

Section 4.1 – Exclusion criteria updated with new criterion regarding acceptable liver function for trial entry

Section 4.1 – Exclusion criteria updated with specifics related to blood pressure levels

Section 4.1 - Exclusion criteria updated with new criterion regarding concomitant medications

Section 4.1 - Exclusion criteria updated with new criterion regarding prior treatment with abiraterone

Section 4.1 - Exclusion criteria updated with new criterion regarding prior treatment with chemotherapy

Section 4.1 - Exclusion criteria updated with new criterion regarding prior treatment with zoledronic acid

Section 4.3 – Wording updated to reflect that patients who initially fail screening can be re-screened at a later date

Section 4.3.2 – Wording updated regarding prior anti-androgen and LHRH use updated for clarity

Section 5.1 – Co-enrolment guidelines information updated to describe newly created co-enrolment CRF

Section 6.1 – Trial treatment information updated to reflect the fact that anti-androgens alone will be no longer permitted as hormone therapy

Section 6.1.1 – Updated to describe patients for whom radiotherapy should be given as standard practice

Section 6.1.1 a and b - Sections added to give information regarding radiotherapy treatment

Section 6.1.1-6.1.6 – References to further sections updated

Section 6.1.7 – Section added to describe abiraterone treatment

Section 6.2.4 - Section added to describe abiraterone treatment

Section 6.6 - Section added to give information regarding radiotherapy treatment

Section 7.1.1 – Reference to blood being taken at patient's home removed as this does not occur in practice

Section 7.1.2 – Wording updated regarding the reporting of biochemical failures for clarity

Section 7.1.2 – Wording updated regarding skeletal-related events for clarity

Section 7.1.3 – Section added to describe additional assessments required related to abiraterone treatment

Section 7.1.4 – Section added to provide information on when treatment should commence

Figure 4 – Updated for clarity regarding return of BMD sub-study forms, the addition the co-enrolment CRF and the description of the re-opening of the QoL Sub-study.

Figure 5 – Updated with reference to abiraterone and co-enrolment form

Section 7.3 - Wording on trial closure updated to reflect current MRC CTU standard wording

Section 8.1 – Additional criteria for definition of progression added for clarity

Section 8.1 – Definition of progression for abiraterone patients added.

Section 9 – Statistical information updated to describe the addition of the new trial arm

Section 11 – Safety reporting wording updated for clarity

Section 11 – SAE reporting fax number updated

Section 12 – Ethical information updated to describe the unequal randomisation allocation ratio

Section 12 – Ethical information updated to describe that the visit schedule will vary according to trial arm

Section 12.2 – Wording updated to reflect international participation in the trial

Section 13 – Wording updated to reflect international participation in the trial

Section 14 – Wording updated to reflect international participation in the trial

Section 15 - Updated to reflect the discontinuation of recruitment to celecoxib arms and the addition of abiraterone

Section 16 – Reference to trial committee charters added for information

Section 17.1 – Information added to reflect re-opening of quality-of-life sub-study

Section 17.2 – Timing of health economics analysis updated to previous error

Section 18 – Information on publication policy expanded for clarity

Section 19 – Information regarding amendments to protocol appendices moved to the separate appendices document

Section 20 – References extensively updated

19.1.10 Amendments Made To Protocol Version 8.0 (Sep-2011)

Throughout protocol – numbering, sections headings, tables, figures and bibliographical references have been updated in some sections to accommodate new information that has been added

Throughout protocol – Androgen Deprivation Therapy has replaced Hormone Therapy as deemed more representative of the type of hormone therapy used in the study

General Information Section – New staff members of the MRC CTU and Co-Investigators added and contact details updated

General Information Section – Abbreviations list updated

Section 1.1 – Information regarding the new research radiotherapy treatment inserted

Section 1.1 – Information regarding docetaxel updated

Section 1.2 – Wording updated to reflect the addition of the new research comparison arm

Section 1.3 – Additional criteria for the re-accreditation of participating centres (for protocol version 9.0 only)

Section 2.1.1 – Wording updated to clarify the use of anti-androgen in trial patients

Section 2.1.2 – Information added to describe the rationale for the RT comparison arm

Section 2.8 – Information added to describe research RT treatment to prostate for patients with newly diagnosed metastatic disease

Section 3.1 – Information added to describe RT Quality Assurance procedures and centre accreditation

Section 4.1.1 to 4.1.3 – Wording updated to clarify inclusion criteria for all patients groups (newly diagnosed non-metastatic, metastatic and relapsing patients)

Section 4.2 – Clarification added on cardiovascular exclusion criteria

Section 4.2 – New exclusion criterion added concerning patients with prior exposure to hormone therapy

Section 4.2 – New exclusion criterion added to reflect the addition of the new RT comparison arm

Section 4.4.1 – Clarification added regarding pre-randomisation checks

Section 4.4.2 – Clarification added regarding permissible hormone therapy duration prior to randomisation

Section 4.4.5 – Information added regarding starting research radiotherapy treatment

Section 4.4.6 – Information updated on concomitant medications

Section 5 – Clarification regarding randomisation allocation added to reflect the addition of the new RT research arm

Section 6.1.8 – Information added to describe the administration of research radiotherapy

Section 6.2.1 – Clarification added regarding the measurement of serum creatinine levels prior to the administration of zoledronic acid

Section 6.2.3 – Clarification regarding the completion of recruitment to the celecoxib containing arms

Section 6.25 – Information added regarding the administration of research radiotherapy treatment

Section 6.6 – Clarification incorporated to describe the administration of standard-of-care radiotherapy

Section 7.1.4 – Information added regarding data collection and non-administration of standard radiotherapy

Section 7.2 – Section updated to include new treatment specific CRFs and timing of CRFs

Section 8.1 – Clarification added for the criteria to stop treatment for patients randomised to arm G

Section 8.2 – Section expanded to include additional details on study patient transfer to different centres

Section 8.3 – Additional sentence inserted to reinforce the importance of compliance with follow-up assessments

Section 9.1 – Additional paragraph inserted to clarify the method of randomisation and allocation distribution in the light of the introduction of the new RT arm

Section 9.4 – Wording updated to clarify the assessment of safety data

Section 9.5.4 – Wording updated concerning the end of randomisations to arm G

Section 9.6 to 9.6.4 – Section added describing sample size issues and trial stages for arm H

Section 9.8 – Clarification on intermediate stopping guidelines

Section 9.9 – Clarification on the outline analysis plan

Section 11 – Information on safety reporting updated to reflect the addition of the research RT comparison arm

Section 11 – Clarification added regarding arm A safety reporting timelines

Section 12.1 – Clarification added regarding the Principal Investigator's responsibilities

Section 14 – Indemnity section updated to reflect current MRC policy

Section 16 – Clarification regarding TMG membership

Section 17.3 – Section on Bone Mineral Density sub-study removed

Section 19 – Information regarding amendments to protocol appendices moved to the separate appendices document

Section 20 – References updated

19.1.11 Amendments Made To Protocol Version 9.0 (Oct-2012)

Throughout protocol – numbering, sections headings, tables, figures and bibliographical references have been updated in some sections to accommodate the completion of recruitment to original research arms B, C and E.

Throughout protocol – Tenses have been changed to reflect activities that were in the future and which have now been passed.

Section 1 – Figure added and clarifications added to each figure

Section 2 – Previous reference 8 removed

Section 4 – Clarification of acceptable alternatives to bone scans

Section 6.2.5 – Correction of an error defining the PTV: the wording has been reordered

Table 4 – Dose-volume objectives corrected: order swapped

Table 5- Correction CRFs names

Section 17.3.2 – Clarification that DNA may be extracted

19.1.12 Amendments Made To Protocol Version 10.0 (Apr-2013)

Throughout protocol – numbering, sections headings, tables, figures and bibliographical references have been updated in some sections

Throughout protocol – typos have been corrected

Section 4 –Clarification of exclusion criteria V (now V and VI)

Section 6 – Timing of orchidectomy prior to randomisation extended to 12 weeks

Section 6 – Clarification of hypokalaemia, blood pressure and fluid retention management

Section 9 – Statistical considerations amended in light of the recruitment extension for the abiraterone comparison

Section 14 - Section updated to reflect the changes in the structure of the MRC CTU (now MRC CTU at UCL) and indemnity arrangements

19.1.13 Amendments Made To Protocol Version 11.0 (Sep-2013)

Throughout protocol – numbering, sections headings, tables, figures and bibliographical references have been updated in some sections

Throughout protocol – typos have been corrected

Co-investigators list updated to reflect the addition of the “enzalutamide + abiraterone comparison” lead

Section 1.2 – Enzalutamide added as trial treatment

Section 1.2 – Protocol version 12.0 added to the list of amendments

Section 2.10 – Rationale for the combination of enzalutamide and abiraterone

Section 4.2 – Eligibility criteria amended to reflect the addition of enzalutamide + abiraterone arm

Section 4.4.2 – Wording clarified

Section 6.8 – Clarification regarding end of trial treatment after starting trial therapy

Section 6.10 – Section added to describe enzalutamide and abiraterone treatment for the new research arm (Arm J)

Section 6.11.4.A – Section added to describe the management of toxicities from trial abiraterone

Section 6.11.4.B - Section added to describe the management of toxicities from trial enzalutamide

Section 9.1.4 – Section added to describe the statistical considerations concerning the introduction of Arm J

Section 9.3 – Principles and assumption for the introduction of Arm J added

Section 9.7 and sub-sections – Sample size issues and trial stages for Arm J

Section 9.9 – Details on interim monitoring and analyses for Arm J added

Section 11.2.1.D – Wording clarified regarding safety reporting requirements for control arm

Section 12.1 – Wording clarified

Section 15 – Details on funding for the “enzalutamide + abiraterone comparison” added

Section 19 - Amendments made to protocol updated

Reference list updated

19.1.14 Amendments Made To Protocol Version 12.0 (Jan-2014)

Throughout protocol – typos have been corrected

Section 4.4.2. Wording clarified

Section 4.3. Wording clarified for eligibility to M1l RT comparison

Section 6.10. Addition of use of dexamethasone post-biochemical progression for Arm J patients

Section 6.11.4.A. Correction of CTCAE version

Section 6.11.4.C. Clarification on enzalutamide dose modification to be in line with current SmPC

Section 9.6. Sample size increase for M1l RT comparison

Section 11. Correction of safety reporting timelines for Arm A patients

Section 17. Addition of saliva samples collection for DNA analysis

Table 4, 5 and 6. Clarification on Case Report Forms and Follow-up schedule

19.1.15 Amendments Made To Protocol Version 13.0 (Feb-2015)

Throughout protocol – typos have been corrected

Throughout protocol – clarification on the new definition of standard-of-care

Table of contents updated to reflect any changes to the protocol

Section 1.1. Wording added throughout section to include reference to survival results from "original comparisons"

Section 2.1.1. Section improved to include reference to survival results from "original research comparisons"

Section 2.1.2. Section improved to include reference to survival results from "original research comparisons".

Section 2.1.3. Additional section added to describe the role of docetaxel for men with M0 or M1 disease

Section 2.9. Clarification on treatment completion and primary results for "original research comparisons"

Section 4.2. Clarification of Exclusion criteria XIII and XVI

Section 4.4.2. Clarification on HT prior to randomisation

Section4.4.3. New section to clarify standard-of-care docetaxel treatment prior to randomisation

Section 4.4.7. Clarification on concomitant medication and contra-indicated concomitant medications

Section 4.5. Clarification provided on tissue block collection

Section 6. Inclusion of docetaxel into the standard-of-care

Section 6.2.3 New section to describe standard-of-care docetaxel administration

Section 6.11. Improvement throughout sections and sub-sections for abiraterone and enzalutamide-related toxicity management

Section 6.12. Section improved throughout to incorporate clearer details on concomitant medications and drug-to-drug interactions

Section 7.1.4. New section to describe data collection for standard-of-care docetaxel

Section 9.7.4. Clarification provided about implications for "enzalutamide+ abiraterone comparison" following change of standard-of-care treatment

Section 11.2.1.D Clarification on SAE notification timelines to reflect change in standard-of-care treatments (addition of docetaxel)

Figure 1. Figure updated to reflect change in standard-of-care

Figure 2. Figure updated to reflect trial history and recruitment over time

Figure 3. Figure updated to reflect changes in standard-of-care and recruiting arms

Table 1. Table updated to remove repetition

Table 13. Table updated to include new CRF to report standard-of-care docetaxel treatment

Table 15. Table updated to include only active trial treatments

19.1.16 Amendments Made To Protocol Version 14.0 (Oct-2015)

Throughout protocol – typos have been corrected

Throughout protocol – clarification on the new definition of standard-of-care

Table of contents updated to reflect any changes to the protocol

Section 1. Wording added throughout section to include reference "metformin comparison"

Section 2. Section updated to include reference "metformin comparison"

Section 4.2. Exclusion criteria review to reflect Arm J closure and instruction of "metformin comparison"

Section 4.3. Clarification of comparison specific eligibility (M1l RT and metformin)

Section 4.5.7. Clarification on concomitant medication and contra-indicated concomitant medications

Section 6. Treatment sections improved throughout

Section 6.11. Section updated to include details on metformin treatment

Section 6.12. Amendment throughout sections and sub-sections for metformin treatment

Section 6.13. Amendment throughout sections and sub-sections for metformin treatment

Section 6.13. Improvement throughout sections and sub-sections for abiraterone and enzalutamide treatment

Section 7.0. Amendment throughout sections and sub-sections to include assessment and procedures specific to "metformin comparison"

Section 9.0. Section updated and streamlined to capture statistical considerations on each comparison

Section 9.0. Details on "metformin comparison" added

Section 11. Safety processes updated and clarified

Section 16.0 Membership to oversight groups updated

Section 11.2.1.D Clarification on SAE notification timelines to reflect change in standard-of-care treatments (addition of docetaxel)

19.1.17 Amendments Made To Protocol Version 15.0 (Mar-2016)

Throughout protocol – re-structure of the treatment-related information for ease of use

Throughout protocol – clarification on the definition of standard-of-care

Throughout protocol – typos have been corrected

Addition of TMG members

Table of contents updated to reflect any changes to the protocol

New section for summary of trial added in table format

Section 1. Revised format for the summary of treatment groups, with the new transdermal oestradiol arm also added

Section 2. Clarification regarding research treatments that have previously reported or completed recruitment, section updated to include the "transdermal oestradiol comparison"

Section 3. New sections added for the "transdermal oestradiol comparison" and future planned biomarker-selected comparisons

Section 4.1.4. Change in definition of adequate renal function

Section 4.3. New section added for the biomarker-screening pilot, selection criteria removed for "research RT comparison"

Section 4.4.1. Change in definition of adequate renal function

Section 4.4.2. New section added for the patient selection criteria specific to the "transdermal oestradiol comparison"

Section 4.5. Screening procedure tables and figure added for clarification.

Section 4.5.1. New section added for biomarker-screening pilot investigations prior to randomisation.

Section 5.1.1. New section added for the biomarker-screening pilot registration.

Section 6. New sections added for the "transdermal oestradiol comparison"

Section 7. Amendment throughout sections and sub-sections to include assessment and procedures specific to "transdermal oestradiol comparison"

Section 7.1.4.B. Section added on cardiovascular outcomes for the "transdermal oestradiol comparison"

Table 18. Table added to clarify follow-up assessments

Section 8. Section updated for "transdermal oestradiol comparison"

Section 9. Section updated for "transdermal oestradiol comparison"

Section 12.1.1.D. Section added on participant feedback from investigations and additional analyses

Section 15. Section updated for "transdermal oestradiol comparison" and biomarker-screening pilot

20 REFERENCES

1. James ND, Sydes MR, Clarke NW, Mason MD, Dearnaley DP, Spears MR, et al. Addition of docetaxel, zoledronic acid, or both to first-line long-term hormone therapy in prostate cancer (STAMPEDE): survival results from an adaptive, multiarm, multistage, platform randomised controlled trial. Lancet. 2016;387(10024):1163-77.
2. James ND, Sydes MR, Mason MD, Clarke NW, Anderson J, Dearnaley DP, et al. Celecoxib plus hormone therapy versus hormone therapy alone for hormone-sensitive prostate cancer: first results from the STAMPEDE multiarm, multistage, randomised controlled trial. The lancet oncology. 2012;13(5):549-58.
3. Cancer Research UK. CancerStats Key Facts: Prostate Cancer. Cancer Research UK. 2011.
4. Sharifi N, Gulley JL, Dahut WL, Sharifi N, Gulley JL, Dahut WL. An update on androgen deprivation therapy for prostate cancer. Endocrine-Related Cancer. 2010;17(4):R305-15.
5. Widmark A, Klepp O, Solberg A, Damber JE, Angelsen A, Fransson P, et al. Endocrine treatment, with or without radiotherapy, in locally advanced prostate cancer (SPCG-7/SFUO-3): an open randomised phase III trial. Lancet. 2009;373(9660):301-8.
6. Warde PR, Mason MD, Sydes MR, Gospodarowicz MK, Swanson GP, Kirkbride P, et al. Intergroup randomized phase III study of androgen deprivation therapy (ADT) plus radiation therapy (RT) in locally advanced prostate cancer (CaP) (NCIC-CTG, SWOG, MRC-UK, INT: T94-0110; NCT00002633). J Clin Oncol. 2010;28(18s Supplement: Proceedings of ASCO 2010):Abstr CRA4504.
7. Warde P, Mason M, Ding K, Kirkbride P, Brundage M, Cowan R, et al. Survival Benefit with Combined Androgen Deprivation and Radiation Therapy in Locally Advanced Prostate Cancer – Results of a Phase III Trial. The Lancet. 2011 - in press.
8. Mason M, Sydes M, Parulekar W, Parmar M, Anderson J, Barber J, et al. Final analysis of intergroup randomized phase III study of androgen deprivation therapy (ADT) + radiation therapy (RT) in locally advanced prostate cancer (CaP) (NCIC-CTG, SWOG, MRC-UK, INT: T94-0110). National Cancer Research Institute (NCRI) Cancer Conference 2012. 2012;2012.
9. Warde P, Mason M, Ding K, Kirkbride P, Brundage M, Cowan R, et al. Combined androgen deprivation therapy and radiation therapy for locally advanced prostate cancer: a randomised, phase 3 trial. The Lancet. 2011;378:2104-11.
10. Nicholas D James MRS, Noel W Clarke, David P Dearnaley, Malcolm D Mason, Christopher C Parker, Alastair W S Ritchie, J. Martin Russell, Francesca Schiavone, Gerhardt Attard, Johann S de Bono, Alison Birtle,Daniel S Engeler,Tony Elliott, David Matheson, Joe O'Sullivan, Delia Pudney, Narayanan Srihari, Jan Wallace, Jim Barber, Isabel Syndikus, Mahesh K B Parmar, Matthew R Sydes. Failure-free survival and the impact of radiotherapy in patients with newly diagnosed non metastatic prostate cancer: Data from patients in the control arm of the STAMPEDE trial (MRC PR08, CRUK/06/019). JAMA Oncology. 2015.
11. Sweeney CJ, Chen YH, Carducci M, Liu G, Jarrard DF, Eisenberger M, et al. Chemohormonal Therapy in Metastatic Hormone-Sensitive Prostate Cancer. N Engl J Med. 2015;373(8):737-46.
12. Vale CL, Burdett S, Rydzewska LH, Albiges L, Clarke NW, Fisher D, et al. Addition of docetaxel or bisphosphonates to standard of care in men with localised or metastatic, hormone-sensitive prostate cancer: a systematic review and meta-analyses of aggregate data. The lancet oncology. 2016;17(2):243-56.
13. James ND, Sydes MR, Clarke NW, Mason MD, Dearnaley DP, Spears MR, et al. Addition of docetaxel, zoledronic acid, or both to first-line long-term hormone therapy in prostate cancer

(STAMPEDE): survival results from an adaptive, multiarm, multistage, platform randomised controlled trial. Lancet. 2015.
14. Mason MD, Clarke NW, James ND, Dearnaley DP, Spears MR, W.S.R. A, et al. Adding celecoxib with or without zoledronic acid for hormone-naive prostate cancer: long-term survival results from an adaptive, multi-arm, multi-stage, platform, randomised controlled trial. 2017. In press.
15. Attard G, Richards J, de Bono JS, Attard G, Richards J, de Bono JS. New strategies in metastatic prostate cancer: targeting the androgen receptor signaling pathway. Clinical Cancer Research. 2011;17(7):1649-57.
16. Fizazi K, Scher HI, Molina A, Logothetis CJ, Chi KN, Jones RJ, et al. Abiraterone acetate for treatment of metastatic castration-resistant prostate cancer: final overall survival analysis of the COU-AA-301 randomised, double-blind, placebo-controlled phase 3 study. The lancet oncology. 2012;13(10):983-92.
17. Rathkopf DE, Smith MR, de Bono JS, Logothetis CJ, Shore ND, de Souza P, et al. Updated interim efficacy analysis and long-term safety of abiraterone acetate in metastatic castration-resistant prostate cancer patients without prior chemotherapy (COU-AA-302). European urology. 2014;66(5):815-25.
18. Flanigan RC, Salmon SE, Blumenstein BA, Bearman SI, Roy V, McGrath PC, et al. Nephrectomy followed by interferon alfa-2b compared with interferon alfa-2b alone for metastatic renal-cell cancer. N Engl J Med. 2001;345(23):1655-9.
19. Mickisch GH, Garin A, van Poppel H, de Prijck L, Sylvester R. Radical nephrectomy plus interferon-alfa-based immunotherapy compared with interferon alfa alone in metastatic renal-cell carcinoma: a randomised trial. Lancet. 2001;358(9286):966-70.
20. Attard G, Cooper CS, de Bono JS. Steroid hormone receptors in prostate cancer: a hard habit to break? Cancer cell. 2009;16(6):458-62.
21. Chen G, Wang X, Zhang S, Lu Y, Sun Y, Zhang J, et al. Androgen receptor mutants detected in recurrent prostate cancer exhibit diverse functional characteristics. The Prostate. 2005;63(4):395-406.
22. Taplin ME, Bubley GJ, Ko YJ, Small EJ, Upton M, Rajeshkumar B, et al. Selection for androgen receptor mutations in prostate cancers treated with androgen antagonist. Cancer Res. 1999;59(11):2511-5.
23. Veldscholte J, Ris-Stalpers C, Kuiper GG, Jenster G, Berrevoets C, Claassen E, et al. A mutation in the ligand binding domain of the androgen receptor of human LNCaP cells affects steroid binding characteristics and response to anti-androgens. Biochemical and biophysical research communications. 1990;173(2):534-40.
24. Zhao XY, Malloy PJ, Krishnan AV, Swami S, Navone NM, Peehl DM, et al. Glucocorticoids can promote androgen-independent growth of prostate cancer cells through a mutated androgen receptor. Nature medicine. 2000;6(6):703-6.
25. Attard G, Reid AH, Auchus RJ, Hughes BA, Cassidy AM, Thompson E, et al. Clinical and biochemical consequences of CYP17A1 inhibition with abiraterone given with and without exogenous glucocorticoids in castrate men with advanced prostate cancer. The Journal of clinical endocrinology and metabolism. 2012;97(2):507-16.
26. Richards J, Lim AC, Hay CW, Taylor AE, Wingate A, Nowakowska K, et al. Interactions of abiraterone, eplerenone, and prednisolone with wild-type and mutant androgen receptor: a rationale for increasing abiraterone exposure or combining with MDV3100. Cancer Res. 2012;72(9):2176-82.
27. Scher HI, Fizazi K, Saad F, Taplin ME, Sternberg CN, Miller K, et al. Increased survival with enzalutamide in prostate cancer after chemotherapy. N Engl J Med. 2012;367(13):1187-97.
28. Tombal B, Borre M, Rathenborg P, Werbrouck P, Heidenreich A, Iversen P, et al. Enzalutamide monotherapy: Phase II study results in patients with hormone-naive prostate cancer. Journal of Clinical Oncology. 2013;31(Supplement 6):Abstract 18.

29. Efstathiou E, Titus MA, Tsavachidou A, Hoang A, Karlou M, Wen S, et al. MDV3100 effects on androgen receptor (AR) signaling and bone marrow testosterone concentration modulation: A preliminary report. . 2011 ASCO Annual Meeting, 2011 J Clin Oncol 2011.
30. Locke JA, Guns ES, Lubik AA, Adomat HH, Hendy SC, Wood CA, et al. Androgen levels increase by intratumoral de novo steroidogenesis during progression of castration-resistant prostate cancer. Cancer Res. 2008;68(15):6407-15.
31. Tran C, Ouk S, Clegg NJ, Chen Y, Watson PA, Arora V, et al. Development of a second-generation antiandrogen for treatment of advanced prostate cancer. Science. 2009;324(5928):787-90.
32. Scher HI, Beer TM, Higano CS, Anand A, Taplin ME, Efstathiou E, et al. Antitumour activity of MDV3100 in castration-resistant prostate cancer: a phase 1-2 study. Lancet. 2010;375(9724):1437-46.
33. Smith MR, Finkelstein JS, McGovern FJ, Zietman AL, Fallon MA, Schoenfeld DA, et al. Changes in body composition during androgen deprivation therapy for prostate cancer. The Journal of clinical endocrinology and metabolism. 2002;87(2):599-603.
34. Eriksson A, Attvall S, Bonnier M, Eriksson JW, Rosander B, Karlsson FA. Short-term effects of metformin in type 2 diabetes. Diabetes, obesity & metabolism. 2007;9(4):483-9.
35. Bailey CJ. Treating insulin resistance in type 2 diabetes with metformin and thiazolidinediones. Diabetes, obesity & metabolism. 2005;7(6):675-91.
36. Ohira M, Miyashita Y, Ebisuno M, Saiki A, Endo K, Koide N, et al. Effect of metformin on serum lipoprotein lipase mass levels and LDL particle size in type 2 diabetes mellitus patients. Diabetes research and clinical practice. 2007;78(1):34-41.
37. Wulffele MG, Kooy A, de Zeeuw D, Stehouwer CD, Gansevoort RT. The effect of metformin on blood pressure, plasma cholesterol and triglycerides in type 2 diabetes mellitus: a systematic review. Journal of internal medicine. 2004;256(1):1-14.
38. Lubik AA, Gunter JH, Hendy SC, Locke JA, Adomat HH, Thompson V, et al. Insulin increases de novo steroidogenesis in prostate cancer cells. Cancer Res. 2011;71(17):5754-64.
39. Venkateswaran V, Haddad AQ, Fleshner NE, Fan R, Sugar LM, Nam R, et al. Association of diet-induced hyperinsulinemia with accelerated growth of prostate cancer (LNCaP) xenografts. J Natl Cancer Inst. 2007;99(23):1793-800.
40. Noto H, Goto A, Tsujimoto T, Noda M. Cancer risk in diabetic patients treated with metformin: a systematic review and meta-analysis. PloS one. 2012;7(3):e33411.
41. Rothermundt C, Hayoz S, Templeton AJ, Winterhalder R, Strebel RT, Bartschi D, et al. Metformin in chemotherapy-naive castration-resistant prostate cancer: a multicenter phase 2 trial (SAKK 08/09). European urology. 2014;66(3):468-74.
42. Langley RE, Cafferty FH, Alhasso AA, Rosen SD, Sundaram SK, Freeman SC, et al. Cardiovascular outcomes in patients with locally advanced and metastatic prostate cancer treated with luteinising-hormone-releasing-hormone agonists or transdermal oestrogen: the randomised, phase 2 MRC PATCH trial (PR09). The lancet oncology. 2013;14(4):306-16.
43. Byar DP. Proceedings: The Veterans Administration Cooperative Urological Research Group's studies of cancer of the prostate. Cancer. 1973;32(5):1126-30.
44. Hedlund PO, Henriksson P. Parenteral estrogen versus total androgen ablation in the treatment of advanced prostate carcinoma: effects on overall survival and cardiovascular mortality. The Scandinavian Prostatic Cancer Group (SPCG)-5 Trial Study. Urology. 2000;55(3):328-33.
45. Gilbert DC, Duong T, Kynaston HG, Alhasso AA, Cafferty FH, Rosen SD, et al. Quality-of-life outcomes from the Prostate Adenocarcinoma: TransCutaneous Hormones (PATCH) trial evaluating luteinising hormone-releasing hormone agonists versus transdermal oestradiol for androgen suppression in advanced prostate cancer. BJU international. 2016.
46. Smith DC, Redman BG, Flaherty LE, Li L, Strawderman M, Pienta KJ. A phase II trial of oral diethylstilbesterol as a second-line hormonal agent in advanced prostate cancer. Urology. 1998;52(2):257-60.

47. de Bono JS, Logothetis CJ, Molina A, Fizazi K, North S, Chu L, et al. Abiraterone and increased survival in metastatic prostate cancer. New England Journal of Medicine. 2011;364(21):1995-2005.
48. Ryan CJ, Smith MR, de Bono JS, Molina A, Logothetis CJ, de Souza P, et al. Abiraterone in metastatic prostate cancer without previous chemotherapy. N Engl J Med. 2013;368(2):138-48.
49. Lipska KJ, Bailey CJ, Inzucchi SE. Use of metformin in the setting of mild-to-moderate renal insufficiency. Diabetes care. 2011;34(6):1431-7.
50. Salpeter SR, Greyber E, Pasternak GA, Salpeter EE. Risk of fatal and nonfatal lactic acidosis with metformin use in type 2 diabetes mellitus: systematic review and meta-analysis. Archives of internal medicine. 2003;163(21):2594-602.
51. Kouloulias V, Giraud J-Y, Davis BJ, Dusserre A, Zurlo A, Bolla M. Quality assurance of the 22961 EORTC trial. A phase III study of the optimal combination of hormonal adjuvant treatment by LHRH analogue and radiation therapy for the management of locally advanced prostate cancer: the dummy run. 2004(0167-8140 (Print)).
52. Royston P, Parmar MKB, Qian W. Novel designs for multi-arm clinical trials with survival outcomes with an application in ovarian cancer. Statistics in Medicine. 2003;22(14):2239-56.
53. Royston P, Barthel FMS, Parmar MKB, Choodari-Oskooei B, Isham V. Designs for clinical trials with time-to-event outcomes based on stopping guidelines for lack of benefit. Trials. 2011;12(1):81.
54. Royston P. nstage: MAMS trial sample size calculator. MRC Clinical Trials Unit, London2009.
55. Haybittle JL. Repeated assessment of results in clinical trials of cancer treatment. The British journal of radiology. 1971;44(526):793-7.
56. Peto R, Pike MC, Armitage P, Breslow NE, Cox DR, Howard SV, et al. Design and analysis of randomized clinical trials requiring prolonged observation of each patient. I. Introduction and design. British journal of cancer. 1976;34(6):585-612.
57. Schweizer MT, Huang P, Kattan MW, Kibel AS, de Wit R, Sternberg CN, et al. Adjuvant leuprolide with or without docetaxel in patients with high-risk prostate cancer after radical prostatectomy (TAX-3501): important lessons for future trials. Cancer. 2013;119(20):3610-8.

STAMPEDE

Systemic Therapy in Advancing or Metastatic Prostate Cancer: Evaluation of Drug Efficacy

A multi-arm multi-stage randomised controlled trial

Version: 17.0

Date: 19-Oct-2017

MRC CTU AT UCL ID:	PR08
ISRCTN #:	ISRCTN78818544
NCT #:	NCT00268476
EUDRACT #:	2004-000193-31
CTA #:	00316/0026/001-0001
MREC #:	04/MRE07/35

Authorised by:

Name:	PROFESSOR NICHOLAS D JAMES
Role:	CHIEF INVESTIGATOR
Signature:	

Name:	MATTHEW SYDES
Role:	TRIAL STATISTICIAN

GENERAL INFORMATION

This document was constructed using the MRC CTU at UCL Protocol Template Version 4.0. It describes the STAMPEDE trial, coordinated by the Medical Research Council (MRC) Clinical Trials Unit (CTU) at University College London (UCL), and provides information about procedures for entering patients into it. The protocol should not be used as an aide-memoire or guide for the treatment of other patients. Every care has been taken in drafting this protocol, but corrections or amendments may be necessary. These will be circulated to the registered investigators in the trial, but sites entering patients for the first time are advised to contact the Cancer And Other Non-Infectious Diseases Group, MRC CTU at UCL, London, UK, to confirm they have the most up-to-date version.

COMPLIANCE

The trial will be conducted in compliance with the approved protocol, the Declaration of Helsinki 1996, the principles of Good Clinical Practice (GCP), Commission Directive 2005/28/EC with the implementation in national legislation in the UK by Statutory Instrument 2004/1031 and subsequent amendments, the UK Data Protection Act (DPA number: Z6364106), and the National Health Service (NHS) Research Governance Framework for Health and Social Care (RGF). International sites will comply with the principles of GCP as laid down by the ICH topic E6 (Note for Guidance on GCP), Commission Directive 2005/28/EC (the European Directive 2001/20/EC [where applicable]) and applicable national regulations.

SPONSOR

Medical Research Council, 2nd Floor, David Phillips Building, Polaris House, North Star Avenue, Swindon, SN2 1FL, UK.

On 01-Aug-2013, the MRC CTU became part of University College London (UCL). The MRC maintains sponsorship for the trial however UCL is the legal entity responsible for the running of the trial. This responsibility is delegated to the coordinating trial unit, the MRC CTU at UCL.

FUNDING

Cancer Research UK's Clinical Research Committee (formerly the Clinical Trials Advisory Awards Committee), Medical Research Council, and educational grants from Novartis, Sanofi-Aventis, Pfizer, Janssen Pharma NV, Astellas, Clovis Oncology.

AUTHORISATIONS AND APPROVALS

The following persons are authorised to sign the final protocol and protocol amendments for the sponsor: Professor Nicholas James (Chief Investigator) and Matthew Sydes (Trial Statistician).

TRIAL REGISTRATION

This trial has been registered with the ClinicalTrials.gov Clinical Trials Register, where it is identified as NCT00268476.

REGISTRATION AND RANDOMISATION
Call MRC CTU at UCL, Monday to Friday 0900-1700
Excluding public holidays or dates when notice has been given by the Unit.
Tel: +44 (0) 20 7670 4777

SAE REPORTING
Fax to 020 7670 4818 within 24 hours of becoming aware of the event
Or send via **encrypted** email to mrcctu.stampede@ucl.ac.uk

TRIAL ADMINISTRATION

COORDINATING SITE

MRC Clinical Trials Unit at UCL<br>90 High Holborn<br>2nd Floor<br>London WC1V 6LJ<br>UK	Switchboard:	020 7670 4700
	Fax:	020 7670 4818
	Email:	mrcctu.stampede@ucl.ac.uk
	Website:	http://www.stampedetrial.org/

MRC CTU AT UCL STAFF

Trial Manager:	Mazna Anjum	Tel:	0207 670 4772
Trial Manager:	Michelle Buckner	Tel:	0207 670 4840
Trial Manager:	Joanna Calvert	Tel:	0207 670 4916
Trial Manager:	Claire Murphy	Tel:	0207 670 4620
Trial Manager:	Christopher Wanstall	Tel:	0207 670 4882
Trial Manager:	Arlen Wilcox	Tel:	0207 670 4822
Data Manager:	Carly Au	Tel:	0207 670 4794
Data Manager:	Lina Bergstrom	Tel:	0207 670 4693
Data Manager:	Danielle Johnson	Tel:	0207 670 4604
Data Manager:	Saba Khan	Tel:	020 670 4865
Data Manager:	Tasmin Philips	Tel:	0207 670 4947
Data Manager:	Peter Vaughan	Tel:	0207 670 4655
Clinical Project Manager:	Claire Amos	Tel:	0207 670 4771
Clinical Project Manager:	Nafisah Atako	Tel:	0207 670 4896
Statistician:	Chris Brawley	Tel:	0207 670 4677
Statistician:	Fiona Ingleby	Tel:	0207 670 4696
Senior Trial Statistician:	Matthew Sydes	Tel:	0207 670 4798
Transdermal Oestradiol Lead:	Duncan Gilbert	Tel:	Please contact STAMPEDE team
Clinical Research Fellow	Clare Gilson	Tel:	Please contact STAMPEDE team
Director, MRC CTU at UCL:	Max Parmar	Tel:	Please contact STAMPEDE team

CHIEF INVESTIGATOR

Prof Nicholas James

Clinical Oncology, The Medical School
University of Birmingham
Birmingham, B15 2TJ
UK

Tel: 0121 371 3615 (Secretary)

Email: n.d.james@bham.ac.uk

CO-INVESTIGATORS AND TMG MEMBERS

Prof Paul Abel<br>Imperial College	London, UK	Co-CCI "Transdermal oestradiol comparison"
Dr Gerhardt Attard<br>Oncologist	London, UK	CCI "Enzalutamide+Abiraterone comparison"
Dr Simon Chowdhury<br>Oncologist	London, UK	
Prof Noel Clarke<br>Urologist	Manchester, UK	TMG Vice-Chair<br>Co-CCI "Metformin comparison"
Mr William Cross<br>Urologist	Leeds, UK	
Prof David Dearnaley<br>Oncologist	London, UK	
Prof Silke Gillessen<br>Oncologist	St Gallen, Switzerland	Co-CCI "Metformin comparison"
Prof Rob Jones<br>Oncologist	Glasgow, UK	
Dr Zafar Malik<br>Oncologist	Liverpool, UK	
Prof Malcolm Mason<br>Oncologist	Cardiff, UK	TMG Vice-Chair
Prof Ruth Langley<br>Oncologist	London & Brighton, UK	Co-CCI "Transdermal oestradiol comparison"
Dr Chris Parker<br>Oncologist	London, UK	CCI "M1|RT comparison"
Mr Alastair Ritchie<br>Surgeon	Edinburgh, UK	
Dr Martin Russell<br>Oncologist	Glasgow, UK	
Prof George Thalmann<br>Urologist	Bern, Switzerland	
Robin Millman<br>Patient representative	UK	
David Matheson,<br>Patient representative	UK	
Prof Mark Sculpher<br>Health Economics Advisor	York, UK	
Dr Amanda Adler<br>Diabetologist	Cambridge, UK	Diabetologist "Metformin comparison"

SAAK Coordinating Centre

Corinne Schär	Bern, Switzerland

Trial Advisory Groups

Biological Research Group	Prof Malcolm Mason (Chair)
	Prof David Waugh (Co-Chair)
Metabolic Translational Group	Prof Noel Clarke (Chair)
Clinical Safety Group	Prof Noel Clarke (Chair)

SUMMARY OF TRIAL

SUMMARY INFORMATION TYPE	SUMMARY DETAILS
Acronym	STAMPEDE
Long Title of Trial	Systemic Therapy in Advancing or Metastatic Prostate Cancer: Evaluation of Drug Efficacy: A multi-arm multi-stage randomised controlled trial
Version	17.0
Date	19-Oct-2017
MRC CTU at UCL ID	PR08
NCT #	NCT00268476
EudraCT #	2004-000193-31
Study Design	Multi-arm multi-stage platform randomised controlled trial
Type of Participants to be Studied	Men starting long-term hormone therapy for metastatic or high-risk non-metastatic prostate cancer
Setting	Tertiary care
Interventions to be Compared	Various - see comparison-specific tables
Study Hypothesis	Various - see comparison-specific tables
Definitive Primary Outcome Measure	Overall survival (unless stated)
Intermediate Primary Outcome Measure	Failure-free survival (unless stated)
Secondary Outcome Measure(s)	Toxicity<br>Symptomatic skeletal events<br>Quality-of-life<br>Cost-effectiveness
Randomisation	Minimisation using a random element across a number of stratification factors
Number of Participants	See comparison-specific tables
Duration	See comparison-specific tables
Sponsor	Medical Research Council
Funders	Cancer Research UK<br>Medical Research Council<br>Astellas<br>Clovis Oncology<br>Janssen<br>Novartis<br>Pfizer<br>Sanofi-Aventis

SUMMARY INFORMATION TYPE	SUMMARY DETAILS
**"Original comparisons"**	
Type of Participants to be Studied	Men starting long-term hormone therapy for metastatic or high-risk non-metastatic prostate cancer
Control Arm	Arm A: Standard-of-care (SOC)<br>Androgen-deprivation therapy (ADT) ± prostate RT ± docetaxel
Interventions to be Compared	Arm B: SOC + zoledronic acid<br>Arm C: SOC + docetaxel<br>Arm D: SOC + celecoxib<br>Arm E: SOC + zoledronic acid + docetaxel<br>Arm F: SOC + zoledronic acid + celecoxib
Allocation ratio	2 control arm : 1 research arm [2A:1B:1C:1D:1E:1F]
Study Hypothesis	Research interventions will improve survival over SOC
Definitive Primary Outcome Measure	Overall survival
Intermediate Primary Outcome Measure	Failure-free survival
Number of Participants	Sufficient for 400 control arm definitive primary outcome measure events (in practice, >3000 patients)
Duration	10 years
Status	Primary results published (1, 2)
**"Abiraterone comparison"**	
Type of Participants to be Studied	Men starting long-term hormone therapy for metastatic or high-risk non-metastatic prostate cancer
Control arm	Arm A: Standard-of-care (SOC)
Intervention to be Compared	Arm G: SOC + abiraterone
Allocation ratio	1 control arm : 1 research arm [1A:1G]
Study Hypothesis	Addition of abiraterone to SOC will improve survival over SOC alone
Definitive Primary Outcome Measure	Overall survival
Intermediate Primary Outcome Measure	Failure-free survival
Number of Participants	Around 1,800 patients for 267 control arm definitive primary outcome measure events
Duration	6 to 8 years
Status	Primary results published (3)
**"M1|RT comparison"**	
Type of Participants to be Studied	Men starting long-term hormone therapy for newly-diagnosed metastatic prostate cancer with no contraindication to prostate radiotherapy
Control arm	Arm A: Standard-of-care (SOC)
Intervention to be Compared	Arm H: SOC + radiotherapy to the prostate (RT)

SUMMARY INFORMATION TYPE	SUMMARY DETAILS
Allocation ratio	1 control arm : 1 research arm [1A:1H]
Study Hypothesis	Addition of RT to SOC will improve survival over SOC alone
Definitive Primary Outcome Measure	Overall survival
Intermediate Primary Outcome Measure	Failure-free survival
Number of Participants	Around 1,800 patients for 267 control arm definitive primary outcome measure events
Duration	6 to 8 years
Status	In follow-up
**"Enzalutamide + abiraterone comparison"**	
Type of Participants	Men starting long-term hormone therapy for metastatic or high-risk non-metastatic prostate cancer
Control Arm	Arm A: Standard-of-care (SOC)
Interventions to be Compared	Arm J: SOC + enzalutamide + abiraterone
Allocation ratio	1 control arm : 1 research arm [1A:1J]
Study Hypothesis	Addition of enzalutamide, in combination with abiraterone, to SOC will improve survival over SOC alone
Definitive Primary Outcome Measure	Overall survival
Intermediate Primary Outcome Measure	Failure-free survival
Number of Participants	Around 1,800 patients for 267 control arm definitive primary outcome measure events
Duration	6 to 8 years
Status	In follow-up
**"Metformin comparison"**	
Type of Participants to be Studied	Non-diabetic men, with no contraindication to metformin, starting long-term hormone therapy for metastatic or high-risk non-metastatic prostate cancer
Control arm	Arm A: Standard-of-care (SOC)
Intervention to be Compared	Arm K: SOC + metformin
Allocation ratio	1 control arm : 1 research arm [1A:1K]
Study Hypothesis	Addition of metformin to SOC will improve survival over SOC alone
Definitive Primary Outcome Measure	Overall survival
Intermediate Primary Outcome Measure	Overall survival
Number of Participants	Around 1,800 patients, including around 1,100 M1 (metastatic) patients, for 374 control arm definitive primary outcome measure events among

SUMMARY INFORMATION TYPE	SUMMARY DETAILS
	M1 patients
Duration	10 years
Status	Recruiting
**"Transdermal oestradiol comparison"**	
Type of Participants to be Studied	Men starting long-term hormone therapy for metastatic or high-risk non-metastatic prostate cancer, having had no more than one 4-week (or one-month) LHRH (Luteinizing hormone releasing hormone) injection & 8 weeks of anti-androgens
Control arm	Arm A: Standard-of-care (SOC)
Intervention to be Compared	Arm L: Transdermal oestradiol ± RT ± docetaxel
Allocation ratio	1 control arm : 1 research arm [1A:1L]
Study Hypothesis	Transdermal oestradiol will be non-inferior to standard hormone therapy, while having fewer side-effects and improved quality-of-life
Definitive Primary Outcome Measures	Co-primary endpoints of progression-free survival and overall survival
Intermediate Primary Outcome Measure	Progression-free survival
Number of Participants	Around 500 to include within a meta-analysis with the PATCH trial, which will include around 2,000 patients overall
Duration	4 to 6 years
Status	Recruiting

Figure 1: Recruiting arms of the STAMPEDE trial from Protocol version 17.0

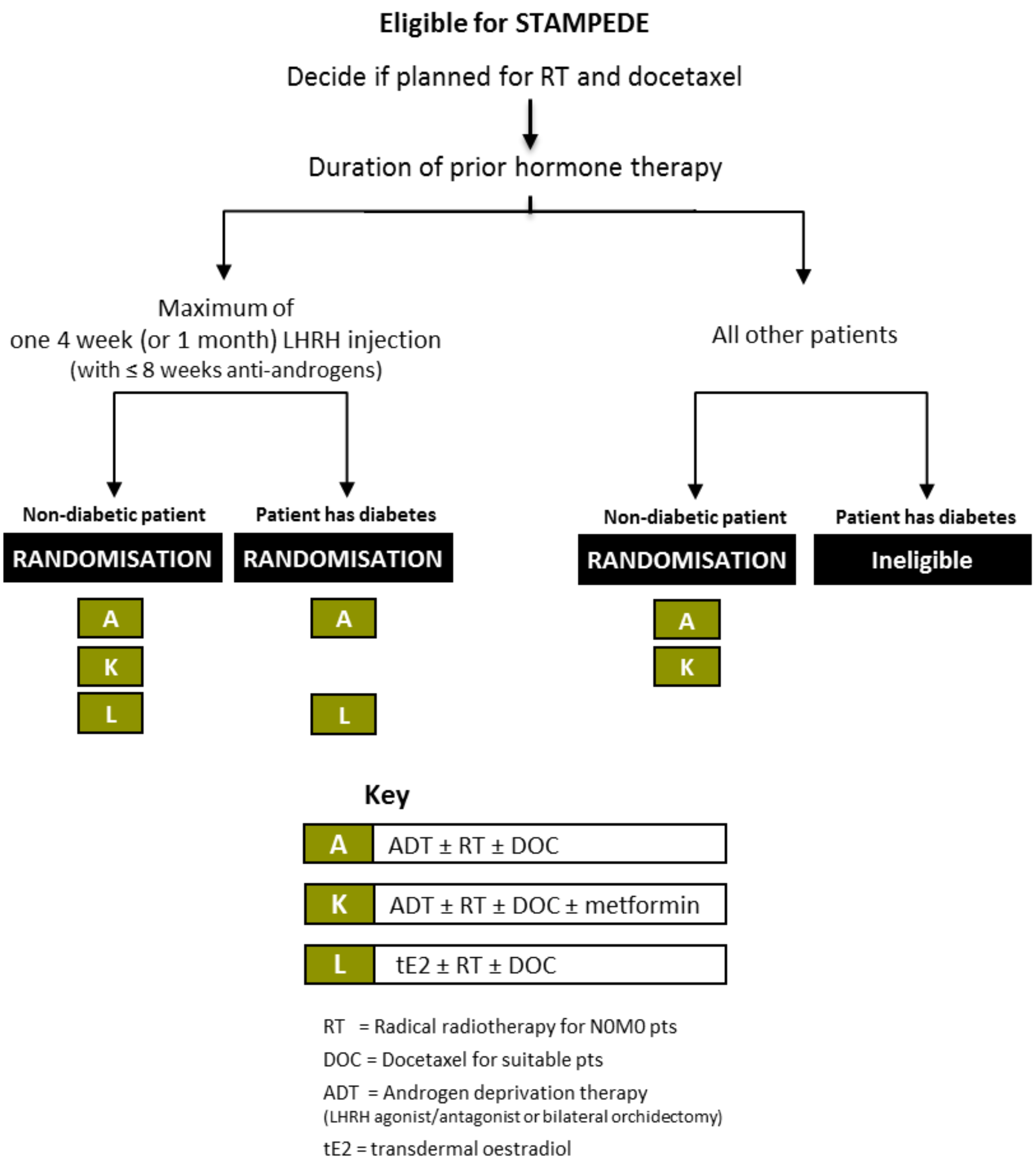

Figure 2: Arms of the STAMPEDE trial open to recruitment over time

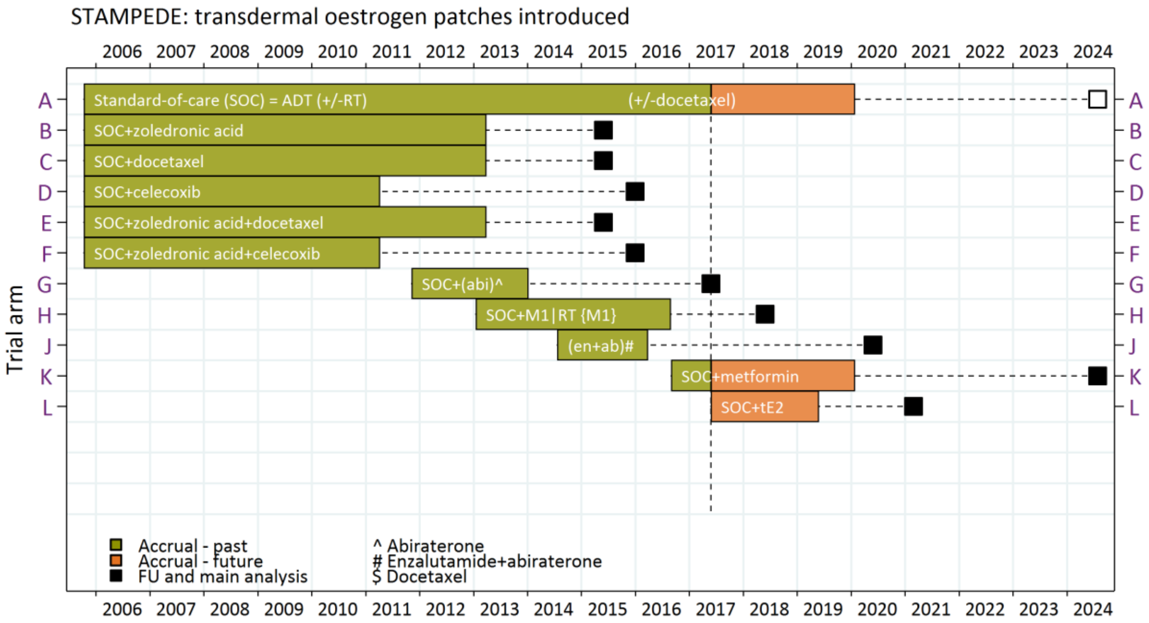

Note: dotted line represents activation of this protocol version

Table 1a: Schedule of Assessments

ALL PATIENTS	PRE-REGISTRATION [a]	PRIOR TO RANDOMISATION
Confirmation of metastatic status to determine eligibility for registration & biomarker screening	X	
Full staging		X [b]
Biochemistry and Haematology [c]	X	X
Pre-ADT PSA (can be obtained up to 6 months before randomisation)		X
ECG		X
BP		X
FFPE tumour block sent for biomarker-screening [a]		X [a]

[a] Only patients participating in biomarker-screening, see **Section 4.3.1** for details and the **Biomarker-Screening Manual** available via the website.

[b] Cross-sectional imaging of pelvis and abdomen (e.g. CT or MRI), Bone Scan (or equivalent e.g. whole body MRI, choline-PET-CT, PSMA-CT-PET), Chest X-ray (**only** if chest was not included in cross sectional imaging)

[c] Full blood count, urea and electrolytes, liver function tests, creatinine or estimated glomerular filtration rate

Table 1 b: Patients randomised before 5th Sep 2016

ARM A/G/J	BASELINE	ASSESSMENT WEEK											ALL FURTHER FU VISITS [a]	PROGRESSION	END OF TRT	PRIOR TO 2ND LINE TRT
		4-6	12	18	24	36	48	60	72	84	96	104				
Baseline bloods [1]	X															
Blood collection cell-free DNA Streck™ tubes [2]							X[b]		X[b]	X[b]				X	X	X
Saliva sample [3]	Any time point															
FFPE block [3]	At the point of request															
PSA	X	X	X	X	X	X	X	X	X	X	X	X	X			
Waist circumference	X	X	X	X	X	X	X	X	X	X	X	X	X			
Weight	X	X	X	X	X	X	X	X	X	X	X	X	X			
Height	X															
QL + HE [3,4]	X	X	X	X	X	X	X	X	X	X	X	X	X			

ARM-SPECIFIC TESTING		BASELINE	ASSESSMENT WEEK											ALL FURTHER FU VISITS [a]
			4-6	12	18	24	36	48	60	72	84	96	104	
**G&J**	Blood pressure		X[c]	X[c]	X	X	X	X	X[c]	X[c]	X[c]	X[c]	X[c]	X[c]
**G&J**	LFTs & potassium		X[d]	X[d]	X	X	X	X	X[d]	X[d]	X[d]	X[d]	X[d]	X[d]

[a] Follow-up visits after year 2 need to be carried out every 6 months for the first 5 years. At year 6 and onwards visits should be every 12 months whilst follow-up continues.
[b] Sample only required for patient with metastatic disease at trial entry (M1)
[c] For patients receiving research abiraterone blood pressure should be checked every 2 weeks until 12 weeks on treatment, then monthly until 12 months on treatment. Then every 2-months providing measurements have been stable and normal. It is acceptable to review documented patient-monitored BP values or those obtained via the patients GP.
[d] For patients receiving research abiraterone, liver function tests and serum potassium monitoring is required 2-weekly in the first 12-weeks, then monthly until 12 months on treatment. For patients who have not experienced toxicity following 12 months of treatment this may be reduced to every 2 months, to continue whilst receiving research abiraterone. Increased monitoring is required in patients experiencing toxicity; see **Tables 7,8,9** for details.

[1] Phosphate, Magnesium, Albumin, calcium and testosterone pre-ADT if available
[2] Only patients participating in sequential blood sampling sub-study, see the **Sample Collection & Handling Manual** available via the STAMPEDE website for details
[3] Only for patients that have consented to participate in the relevant sub-study
[4] 1st 700 pts and from Protocol version 8.0 onwards all patients who have consented to participate in the Quality of life (QL) sub-study. QL and Health-economic data collection is to continue until 5 years post randomisation or progression, whichever is sooner.

Table 1c: Patients randomised after 5th Sep 2016

ARM A/K/L	BASELINE	ASSESSMENT WEEK											ALL FURTHER FU VISITS [a]	PROGRESSION	END OF TRT	PRIOR TO 2ND LINE TRT
		4-6	12	18	24	36	48	60	72	84	96	104				
Baseline bloods [1]	X															
Blood collection cell-free DNA Streck™ tubes [2]	X [b]						X [b]		X [b]	X [b]				X	X	X
Saliva sample [3]	Any time point															
FFPE block [3]	At the point of request															
PSA	X	X	X	X	X	X	X	X	X	X	X	X	X			
Waist circumference	X	X	X	X	X	X	X	X	X	X	X	X	X			
Weight	X	X	X	X	X	X	X	X	X	X	X	X	X			
Height	X															
QL & HE [3,4]	X	X	X	X	X	X	X	X	X	X	X	X	X			
HbA1c	X				X [c]		X [c]					X [c]	X [c]			
Fasting Glucose	X				X [c]		X [c]					X [c]				
Fasting Triglycerides	X				X [c]		X [c]					X [c]				
Lipid profile	X				X [c]		X [c]					X [c]	X [c]			

ARM-SPECIFIC TESTING		BASELINE	ASSESSMENT WEEK											ALL FURTHER FU VISITS [a]
			4-6	12	18	24	36	48	60	72	84	96	104	
**K**	Renal function [5]	X				X		X		X	X	X	X	X
**L**	Testosterone & Oestradiol [6]		X	X		X		X		X		X	X	X

[a] Follow up visits after year 2 need to be carried out every 6 months for the first 5 years. At year 6 and onwards visits should be every 12 months.
[b] Sample only required for patient with metastatic disease at trial entry (M1)
[c] If missed, samples can be obtained +/-12 weeks of the scheduled FU visit, maintaining 10-12 weeks in between the tests due at week 24 and 48 weeks.
[1] Phosphate, Magnesium, Albumin, calcium and testosterone pre-ADT if available
[2] Only patients participating in sequential blood sampling sub-study, see the **Sample Collection & Handling Manual** available via the STAMPEDE website for details
[3] Only for patients that have consented to participate in the relevant sub-study
[4] 1st 700 pts and from Protocol v8.0 onwards all patients who have consented to participate in the Quality of life (QL) sub-study. Data collection is to continue until 5 years or progression, whichever is sooner.
[5] Monitoring of renal function (Creatinine or estimated GFR) required at least every 6 months (week 24, week 104 etc.) while receiving metformin.
[6] Hormone tests are required whilst the patient is still receiving research transdermal oestradiol. Note that additional tests may be necessary as detailed in **Section 6.2.8.B** and **Section 6.2.8.C.**

CONTENTS

GENERAL INFORMATION 2

SUMMARY OF TRIAL 7

CONTENTS 16

ABBREVIATIONS & GLOSSARY 21

1 LAY SUMMARY 25

2 BACKGROUND 28
2.1 INTRODUCTION AND SETTING 28
2.1.1 Long-term Androgen Deprivation Therapy 28
2.1.2 Role Of Radiotherapy For Men With M0 Disease 28
2.1.3 Role Of Docetaxel For Men With M0 Or M1 Disease 29
2.2 RATIONALE 29
2.3 DESIGN 29
2.4 PREVIOUSLY-REPORTED RESEARCH TREATMENTS 30
2.5 RATIONALE FOR RESEARCH TREATMENTS UNDER EVALUATION 30
2.5.1 Steroid Synthesis Inhibitors 30
2.5.2 Radiotherapy To The Prostate For Patients With Newly-Diagnosed Metastatic Disease 31
2.5.3 Combination Of Steroid Synthesis Inhibitors And Androgen Receptor Signalling Inhibitor 31
2.5.4 Metformin 32
2.5.5 Transdermal Oestradiol 33

3 SELECTION OF INSTITUTIONS AND INVESTIGATORS 36
3.1 COMPARISON-SPECIFIC SITE ACCREDITATION 36
3.1.1 "Transdermal Oestradiol Comparison" 36
3.2 FUTURE PLANNED BIOMARKER-SELECTED COMPARISONS 36
3.3 REQUIRED TRIAL DOCUMENTATION 37

4 SELECTION OF PATIENTS 38
4.1 GENERAL INCLUSION CRITERIA 38
4.1.1 High-Risk Newly-Diagnosed Non-Metastatic Node-Negative Disease 38
4.1.2 Newly-Diagnosed Metastatic Or Node-Positive Disease 38
4.1.3 Previously Radically Treated, Now Relapsing (Prior Radical Surgery And/Or Radiotherapy) 38
4.1.4 For All Patients 38
4.2 GENERAL EXCLUSION CRITERIA 39
4.3 BIOMARKER-SCREENING PILOT 40
4.3.1 Selection Criteria For Patient Registration 40
4.4 COMPARISON-SPECIFIC SELECTION CRITERIA 40
4.4.1 For Randomisation To Include The "Metformin Comparison" 40
4.4.2 For Randomisation To Include The "Transdermal Oestradiol Comparison" 41
4.5 SCREENING PROCEDURES 42
4.5.1 Investigation Prior to Registration (for patients participating in biomarker-screening pilot) 42
4.5.2 Investigations Prior To Randomisation 42
4.5.3 Additional Baseline Investigations 43

4.5.4 Androgen Deprivation Therapy Prior To Randomisation 43
4.5.5 Standard-Of-Care (SOC) Radiotherapy 44
4.5.6 Standard-Of-Care (SOC) Docetaxel 44
4.5.7 Starting Trial Treatment 45
4.5.8 Concomitant Medications 45
4.6 ADDITIONAL DETAILS FOR PATIENTS JOINING SUB-STUDIES 46
4.6.1 Germline DNA Analysis (Saliva Samples) 46
4.6.2 Circulating Tumour-DNA Analysis (Sequential Blood Samples) 46
4.6.3 Tissue Sample Analysis (FFPE Blocks) 47
4.6.4 Biomarker-Screening Pilot 47
4.6.5 Informed Consent For Genetic Screening 48

5 REGISTRATION AND RANDOMISATION 49
5.1 TRIAL ENROLMENT: DEFINITIONS AND PROCESS 49
5.1.1 Registration 49
5.1.2 Randomisation 49
5.2 CO-ENROLMENT GUIDELINES 50

6 TREATMENT OF PATIENTS 51
6.1 STANDARD-OF-CARE (SOC) 51
6.1.1 Hormone Therapy 51
6.1.2 Standard-Of-Care (M0) RT 52
6.1.3 Standard-Of-Care Docetaxel 52
6.2 RESEARCH TREATMENTS 53
6.2.1 Required Timelines When Starting Research Treatment 53
6.2.2 Research Abiraterone + Prednisolone (relevant to arms G & J) 53
6.2.3 Abiraterone + Prednisolone: Administration And Management Of Toxicities 54
6.2.4 Research Enzalutamide + Abiraterone + Prednisolone (Arm J) 59
Enzalutamide: Administration, Dose Modification And Management Of Toxicities 60
6.2.5 60
6.2.6 Research Metformin (Arm K) 62
6.2.7 Metformin: Administration, Dose Modifications And Management Of Toxicities 62
6.2.8 Research Transdermal Oestradiol (Arm L) 65
6.2.9 Transdermal Oestradiol: Administration, Dose Modifications And Management Of Toxicities66
6.3 CONCOMITANT MEDICATIONS AND DRUG INTERACTION 67
6.3.1 Abiraterone: Interaction With Medicinal Products And Other Forms Of Interaction 67
6.3.2 Enzalutamide: Interaction With Medicinal Products And Other Forms Of Interaction 67
6.3.3 Metformin: Interaction With Medicinal Products And Other Forms Of Interaction 70
6.3.4 Transdermal Oestradiol: Drug Interactions 70
6.4 TRIAL PRODUCTS 71
6.5 TREATMENT DATA COLLECTION 71
6.6 MEASURES OF COMPLIANCE/ADHERENCE 71
6.7 ADMINISTRATION OF STANDARD RADIOTHERAPY TO M0 PATIENTS 71
6.7.1 Treatment Details 71

7 ASSESSMENTS AND PROCEDURES 73
7.1 SCHEDULE FOR ASSESSMENTS 73
7.1.1 Follow-Up Schedules 73
7.1.2 PSA, Testosterone And Oestradiol Measurements 73
7.1.3 Assessment Of Treatment Failure (Definition Of Progression) 74
7.1.4 Additional Metabolic And Cardiovascular Outcomes 75

7.1.5 Additional Safety Assessments 76
7.2 DATA COLLECTION PROCEDURES 77
7.2.1 Data Collection For SOC Hormone Therapy 77
7.2.2 Data Collection For Standard Docetaxel 77
7.2.3 Data Collection And Non-Administration Of Standard Radiotherapy 78
7.2.4 Data Collection For Palliative Radiotherapy 78
7.2.5 Data Collection for Research (M1) Radiotherapy 78
7.2.6 Data Collection for Additional Treatments Given for Disease Progression 78
7.3 FOLLOW-UP PROCEDURE 79
7.3.1 Follow-Up Telephone Consultations 79
7.4 TRIAL CLOSURE 79

8 STOPPING OF TREATMENT OR FOLLOW-UP 83
8.1 STOPPING RESEARCH INTERVENTIONS 83
8.1.1 Stopping Trial Treatment: Abiraterone, Enzalutamide + Abiraterone 83
8.1.2 Stopping Trial Treatment: Metformin 83
8.1.3 Stopping Trial Treatment: Transdermal Oestradiol 83
8.2 PATIENT TRANSFERS 84
8.3 EARLY CESSATION OF TRIAL PARTICIPATION 84

9 STATISTICAL CONSIDERATIONS 86
9.1 METHOD OF RANDOMISATION 86
9.2 OUTCOME MEASURES 86
9.3 SAMPLE SIZE: PRINCIPLES 87
9.4 SAMPLE SIZE ISSUES AND TRIAL STAGES: ADDITIONAL RESEARCH ARM G 89
9.4.1 Pilot Phase: Additional Research Arm G 89
9.4.2 Activity Stages I-III: Additional Research Arm G 89
9.4.3 Efficacy Stage IV: Additional Research Arm G 89
9.4.4 Sample Size For Additional Research Arm G 89
9.5 SAMPLE SIZE ISSUES AND TRIAL STAGES: ADDITIONAL RESEARCH ARM H 90
9.5.1 Pilot Phase: Additional Research Arm H 90
9.5.2 Activity Stages I-III: Additional Research Arm H 90
9.5.3 Efficacy Stage IV: Additional Research Arm H 90
9.5.4 Sample Size For Additional Research Arm H 90
9.6 SAMPLE SIZE ISSUES AND TRIAL STAGES: ADDITIONAL RESEARCH ARM J 92
9.6.1 Pilot Phase: Additional Research Arm J 92
9.6.2 Activity Stages I-II: Additional Research Arm J 92
9.6.3 Efficacy Stage III: Additional Research Arm J 93
9.6.4 Sample Size For Additional Research Arm J 93
9.6.5 Further Sample Size Issues For Additional Research Arm J 93
9.7 SAMPLE SIZE ISSUES AND TRIAL STAGES: ADDITIONAL RESEARCH ARM K 93
9.7.1 Implementation: Additional Research Arm K 94
9.7.2 Outcome Measures: Additional Research Arm K 94
9.7.3 Pilot Phase: Additional Research Arm K 95
9.7.4 Activity Stage I: Additional Research Arm K 95
9.7.5 Efficacy Stage II: Additional Research Arm K 95
9.7.6 Sample Size For Additional Research Arm K 95
9.7.7 Further Sample Size Issues For Additional Research Arm K 96
9.8 SAMPLE SIZE ISSUES AND TRIAL STAGES: ADDITIONAL RESEARCH ARM L 96
9.8.1 Implementation And Outcome Measures: Additional Research Arm L 96
9.8.2 Additional Use of Outcome Data from the “transdermal oestradiol comparison” 98

9.8.3 Definition of PFS and Use As Co-primary OM: Additional Research Arm L 98
9.9 FURTHER NOTES ON TRIAL DESIGN 99
9.9.1 Overall Sample Size 99
9.9.2 Factorial Design 99
9.10 INTERIM MONITORING AND ANALYSES 99
9.11 OUTLINE ANALYSIS PLAN 100
9.11.1 Pilot / Safety Phases 100
9.11.2 Activity And Efficacy Stages 100

10 MONITORING AND QUALITY ASSURANCE 101
10.1 MONITORING AT CTU 101
Central monitoring of consent 101
10.1.1 101
10.2 DIRECT ACCESS TO DATA 101
10.3 VISITS TO INVESTIGATOR SITES 101
10.4 CONFIDENTIALITY 101

11 SAFETY REPORTING 102
11.1 SAFETY REPORTING DEFINITIONS 102
11.1.1 Defining treatment for the purposes of safety reporting 103
11.1.2 Safety data collection in STAMPEDE 103
11.2 SAFETY PROCESSES: EXEMPTIONS 104
11.2.1 Exemptions: Trial-specific SAE reporting exemptions 104
11.3 SITE INVESTIGATOR RESPONSIBILITIES 105
11.3.1 Investigator Assessment 105
11.3.2 Notification responsibilities 106
Event 108
11.3.3 Follow Up 108
11.4 CTU RESPONSIBILITIES 108

12 ETHICAL CONSIDERATIONS AND APPROVAL 110
12.1 ETHICAL CONSIDERATIONS 110
12.2 ETHICAL APPROVAL 112

13 REGULATORY APPROVAL 113

14 INDEMNITY 114

15 FINANCE 115

16 TRIAL COMMITTEES 116
16.1 TRIAL MANAGEMENT GROUP (TMG) 116
16.2 TRIAL STEERING COMMITTEE (TSC) 116
16.3 INDEPENDENT DATA MONITORING COMMITTEE (IDMC) 116
16.4 TRIAL EXPERT PANELS 117

17 ANCILLARY STUDIES 118
17.1 QUALITY-OF-LIFE 118
17.2 HEALTH ECONOMICS 118
17.3 TRANSLATIONAL SUB-STUDIES 118

17.4 DISEASE VOLUMETRIC ANALYSIS SUB-STUDY 119

18 PUBLICATIONS 120

19 PROTOCOL AMENDMENTS 121
19.1 PROTOCOL 121
19.1.1 Amendments Made To Sections In Protocol Version 1.0 (May-2004) 121
19.1.2 Amendments Made To Sections In Protocol Version 1.1 (May-2005) 121
19.1.3 Amendments Made To Sections In Protocol Version 2.0 (Jun-2005) 121
19.1.4 Amendments Made To Section In Protocol Version 3.0 (Jul-2006) 122
19.1.5 Amendments Made To Protocol Version 4.0 (Dec-2007) 123
19.1.6 Amendments Made To Protocol Version 5.0 (Aug-2008) 123
19.1.7 Amendments Made To Protocol Version 6.0 (Jul-2009) 124
19.1.8 Amendments Made To Protocol Version 7.0 (Jul--2011) 124
19.1.9 Amendments Made To Protocol Version 7.1 (Jul-2011) 125
19.1.10 Amendments Made To Protocol Version 8.0 (Sep-2011) 126
19.1.11 Amendments Made To Protocol Version 9.0 (Oct-2012) 127
19.1.12 Amendments Made To Protocol Version 10.0 (Apr-2013) 128
19.1.13 Amendments Made To Protocol Version 11.0 (Sep-2013) 128
19.1.14 Amendments Made To Protocol Version 12.0 (Jan-2014) 128
19.1.15 Amendments Made To Protocol Version 13.0 (Feb-2015) 129
19.1.16 Amendments Made To Protocol Version 14.0 (Oct-2015) 129
19.1.17 Amendments Made To Protocol Version 15.0 (Mar-2016) 130
19.1.18 Amendments Made To Protocol Version 16.0 (Oct-2017) 130

20 REFERENCES 132

ABBREVIATIONS & GLOSSARY

ABBREVIATION	EXPANSION
AA	Anti-androgen
AAH	Amalgamated Anthracite Holdings
ACE	Angiotensin-Converting Enzyme
ACTH	Adrenocorticotropic hormone
ADT	Androgen deprivation therapy
AR	Androgen receptor
AS	Activity Stage
AUC	Area under the plasma concentration–time curve
BID	Twice a day (bis in die)
BP	Blood pressure
BRG	Biological Research Group
BSA	Body surface area
CCI	Comparison Chief Investigator
CF	Consent Form
CI	Chief Investigator
CI	Confidence interval
Co-CCI	Comparison Co-Chief Investigator
Cox-2	Cyclooxygenase 2
CRF	Case Report Form
CRN	Clinical Research Network
CRUK	Cancer Research UK
CRPC	Castration Resistant Prostate Cancer
CT	Computerised tomography
CTA	Clinical Trials Authorisation
CTAAC	Clinical Trials Advisory and Awards Committee
CTC	Common Toxicity Criteria
CTU	Clinical Trials Unit
CTV	Clinical Tumour Volume
CXR	Chest X-ray
DAB	Dual Androgen Blockade
DHT	Dihydrotestosterone
DNA	Deoxyribonucleic Acid

ABBREVIATION	EXPANSION
DPA	Data Protection Act
ES	Efficacy Stage
IB	Investigator Brochure
ICH	International Conference on Harmonization
ECG	Electro cardiogram
FBC	Full Blood Count
FFS	Failure-Free Survival
FFPE	Formalin Fixed Paraffin Embedded
GCP	Good Clinical Practice
GFR	Glomerular Filtration Rate
GP	General Practitioner
HbA1c	Glycated haemaglobin
Hb	Haemoglobin
HE	Health Economics
HES	Hospital Episode Statistics
Hr	Hour
HR	Hazard Ratio
HSCIC	Health & Social Care Information Centre
HT	Hormone Therapy
IDMC	Independent Data Monitoring Committee
IM	Intramuscular
IMRT	Intensity Modulated Radiation Therapy
IR	Immediate-Release
ISRCTN	International Standard Randomised Controlled Trial Number
IU	International Units
IV	Intravenous
LFTs	Liver Function Tests
LHRH	Luteinising Hormone Releasing Hormone
LREC	Local Research Ethics Committee
m	Month
mcg	Microgram
MHRA	Medicine and Healthcare Products Regulatory Agency
min	Minutes
MRC	Medical Research Council

ABBREVIATION	EXPANSION
MREC	Multi-Centre Research Ethics Committee
MRI	Magnetic resonance imaging
M0	Non-metastatic
M1	Metastatic
NCI	National Cancer Institute (USA)
NHS	National Health Service
N0	Node-negative
N+	Node-positive
NSAID	Non-Steroidal Anti-inflammatory Drugs
OD	Once per day (omne in die)
ONS	Office for National Statistics
OS	Overall Survival
PFS	Progression-free survival
PI	Principal Investigator
PIS	Patient Information Sheet
po	Orally (per orum)
PSA	Prostate Specific Antigen
pts	Patients
PTV	Planned Tumour Volume
QALY	Quality-adjusted Life Years
qds	Four times each day (quater die sumendus)
QL	Quality-of-life
R&D	Research and Development
RECIST	Response Evaluation Criteria In Solid Tumours
SAE	Serious Adverse Event
SAR	Serious Adverse Reaction
sc	Under skin (sub-cutaneous)
SmPC	Summary of Product Characteristics
SOC	Standard-of-Care
SR	Sustained-Release
SSA	Site Specific Assessment
STAMPEDE	Systemic Therapy in Advancing and Metastatic Prostate Cancer: Evaluation of Drug Efficacy
SUSAR	Suspected Unexpected Serious Adverse Reactions

ABBREVIATION	EXPANSION
SWOG	South West Oncology Group
tE2	Transdermal Oestradiol
TMG	Trial Management Group
TMT	Trial Management Team
TURP	Trans-Urethral Resection of Prostate
TSC	Trial Steering Committee
UCL	University College London
ULN	Upper Limit of Normal
U+E	Urea and Electrolytes
WHO	World Health Organisation

TERM	DEFINITION
ADT	Androgen deprivation therapy given in the form of LHRH agonists/antagonists (abbreviated to LHRH) or alternatively, transdermal oestradiol
Hormone Therapy	Refers to all forms of hormone therapy given in the first line setting and includes LHRH, anti-androgens, transdermal oestradiol, GnRH Agonists and antagonists. This term does not include novel AR-targeted agents such as abiraterone or enzalutamide.
PSA nadir	For trial purposes, this refers to the lowest PSA value detected between randomisation and week 24 on trial. This is used to derive the PSA progression value.
Protocol Research treatment	Additional treatments patients allocated to research arms (B-K) receive as part of the STAMPEDE protocol e.g. metformin for patients allocated to arm K, or alternative in the case of transdermal oestradiol (arm L)
Protocol standard-of-care (SOC) treatment	Standard forms of background treatment permitted as part of the STAMPEDE protocol which include licenced ADT (e.g. LHRH analogues) given in the setting of hormone-naïve prostate cancer and first-line use of docetaxel
Non-protocol treatments	All prostate cancer treatments given following disease progression in the management of CRPC
Prednisolone	In Swiss sites this maybe referred to as prednisone.

1 LAY SUMMARY

Prostate cancers depend upon the male hormone testosterone for their growth. Lowering testosterone levels (either by removing all or part of both testes, or by giving anti- male hormone treatment) slows the growth of prostate cancers. This type of treatment is called hormone treatment or androgen deprivation therapy (ADT) and is often used when prostate cancers have spread outside the prostate gland. Although hormone treatment is usually successful at stopping the cancer growing for a period of time, the cancer will begin to grow again in most men. In addition, standard hormone treatment with injections (LHRH Analogues) can cause a range of side-effects which may become serious and affect quality-of-life, particularly since some men could remain on treatment for a decade or longer.

The overall aim of this trial, which is called STAMPEDE, is to assess novel approaches for the treatment of men with prostate cancer who are starting long-term ADT for the first time. Since opening to accrual in Oct-2005, the trial has tested many ways of treating prostate cancer and some results are now already known. More than 10,000 men will join the trial with answers becoming available throughout the trial. The trial will also look at the effects each treatment has on quality-of-life, and which treatment provides the greater value for money for the health service.

New patients joining the trial from Protocol version 16.0 onwards may be eligible to join one of two treatment comparisons, metformin (treatment group K; the “metformin comparison”) and transdermal oestradiol (treatment group L; the “transdermal oestradiol comparison”). A computer program will be used to allocate which treatment each participant receives, using a chance process.

Table 2: Summary of treatment groups currently open to recruitment (Protocol version 17.0)

Treatment being tested	Treatment group	Summary	From Protocol version
Metformin	Arm K	This anti-diabetic medication is proposed to have both anti-cancer effects and may help prevent the adverse metabolic effects of long-term ADT. STAMPEDE will investigate whether adding metformin to the current standard-of-care for non-diabetic men can improve all-cause survival.	15.0
Transdermal oestradiol	Arm L	This is a form of hormone treatment which can suppress testosterone as effectively as standard ADT and has been shown to avoid some of the side-effects. For example, treatment with transdermal oestradiol does not appear to cause the bone to thin, a common problem with standard forms of ADT which might lead to the bones becoming fragile (osteoporosis) and more likely to break. It may also help to avoid some of the side effects and therefore improve overall quality of life compared with standard forms of ADT. STAMPEDE will investigate whether transdermal oestradiol can treat the cancer as well as current standard forms of ADT.	16.0

Further results are expected in the next few years from other treatments tested in STAMPEDE, which have completed recruitment. These include treatments currently used in different settings, including abiraterone and enzalutamide, both currently used when hormone treatment is no longer

effective and the cancer has started to grow again, termed castrate resistant prostate cancer (CRPC). Prostate radiotherapy, which is a treatment used in localised prostate cancer, has also been tested as an additional treatment for men with cancer that has spread to other parts of the body (metastatic prostate cancer). The results relating to these questions are expected in the next few years.

Table 3: Summary of treatment groups closed to recruitment; results awaited

TREATMENT BEING TESTED	TREATMENT GROUP	SUMMARY	FROM PROTOCOL VERSION
Prostate radiotherapy	Arm H	This is treatment with high-energy x-rays targeted to the prostate gland. This treatment is now mandatory within STAMPEDE for patients with cancer that is confined to the prostate gland as large trials have shown it improves life expectancy. We are not certain whether we should give radiotherapy to the prostate if the cancer has already spread and so we are investigating this in STAMPEDE.	9.0
Enzalutamide (given with abiraterone)	Arm J	Enzalutamide is another novel hormone treatment, similar to abiraterone, which is also used in advanced prostate cancer, when standard hormone therapy has stopped working. Enzalutamide works by blocking androgen receptors and this may complement abiraterone. STAMPEDE is testing whether this treatment combination is a more effective way of controlling prostate cancer growth for longer and improving life expectancy.	12.0

In the past STAMPEDE also tested whether adding docetaxel chemotherapy, zoledronic acid, celecoxib, alone or in combination, was benefical in controlling prostate cancer growth and improving life expectancy. Recruitment has now been completed to all of these original treatment groups and the results have been presented. For further information relevant to these treatment groups, refer to the STAMPEDE website where you can see earlier versions of the protocol and find summaries of the results and links to the scientific publications.

Table 4: Summary of treatment (groups) closed to recruitment; results reported

Treatment tested	Treatment group	Summary of rationale and results	Protocol version Added
Zoledronic acid	Arm B	Prostate cancer cells can spread to bones and weaken them. Zoledronic acid is a drug that reduces bone destruction and hardens bones cells.<br>The results of STAMPEDE show that the addition of zoledronic acid does not prolong life expectancy. These results were compared with data from other similar trials that have tested this treatment, these data also support the findings of STAMPEDE.	1.0
Docetaxel	Arm C	Docetaxel is a type of chemotherapy which stops cells replicating. It has been used to treat advanced prostate cancer for some time, and is used in the treatment of lung, breast and ovarian cancer.<br>The results of STAMPEDE show that the addition of docetaxel to hormone treatment does improve survival, most markedly in men with metastatic disease, and delays time to progression for men with locally-advanced and metastatic disease.<br>The results of STAMPEDE were combined with other similar trials testing docetaxel and the results of the meta-analysis support this effect.<br>Docetaxel may now be given as part of standard treatment to all suitable men entering STAMPEDE (from Protocol version 14.0).	1.0
Celecoxib	Arm D	Celecoxib is an aspirin-like drug that is used to treat arthritis. It slows down the growth of cancer cells in the laboratory. STAMPEDE tested whether the addition of celecoxib could delay the growth of prostate cancer cells. Recruitment stopped early as a planned intermediate analysis failed to demonstrate sufficient effect of this drug. The final results were presented at GU ASCO 2016, a major international congress, and show that alone, celecoxib does not improve life expectancy.	1.0
Abiraterone	Arm G	This is a novel hormone treatment which works by inhibiting steroid hormone synthesis so blocks prostate cancer cells from generating their own male hormones. This is thought to be a major way in which prostate cancer cells resume growth following anti-hormonal therapies. The results of STAMPEDE have shown that the addition of abiraterone with prednisone improves survival and time to progression or relapse when used earlier, for men with locally-advanced or metastatic disease.	8.0

Note that the combination of docetaxel and zoledronic acid was assessed in Arm E and, whilst beneficial overall, did not provide additional benefit over docetaxel. The combination of celecoxib and zoledronic acid was assessed in Arm F. No benefit was seen overall, however an improvement in life-expectancy was observed in the group of patients who had metastatic disease at trial entry who received both celecoxib and zoledronic acid (4).

2 BACKGROUND

2.1 INTRODUCTION AND SETTING

Prostate cancer is a major health problem world-wide and accounts for nearly one fifth of all newly-diagnosed male cancers. In the UK, approximately 46,700 men were diagnosed with prostate cancer in 2014 and over 11,000 men died from the disease(5).

2.1.1 Long-term Androgen Deprivation Therapy

The initial (first-line) treatment for locally-advanced or metastatic prostate cancer is based on androgen deprivation therapy (ADT) achieved either surgically with bilateral orchidectomy, or medically with LHRH agonists or antagonists (6). Long-term use of oral anti-androgens is permitted only when given with LHRH agonists, to achieve dual androgen blockade (previously termed maximum androgen blockade - MAB).

ADT produces responses in up to 95% of patients but it is not curative and disease recurs in virtually all patients treated with ADT as sole therapy, with a median time to progression of 18-24 months (6). Data from the control arm in STAMPEDE has shown that for men with newly-diagnosed metastatic disease, treated with ADT alone, the time to progression is just 11 months. Such progressive disease is referred to as castrate resistant prostate cancer (CRPC).

Another important issue with ADT is the numerous associated side-effects, particularly with prolonged use. Since patients continue on LHRH after disease progression (with additional agents added), many men remain on treatment for a decade or longer, particularly as life expectancy for men with prostate cancer should continue to improve as the number of effective treatments increases. The adverse effects of ADT using LHRH analogues include osteoporosis (leading to an increased risk of fracture), adverse metabolic effects, cognitive decline, sexual dysfunction, hot flushes, physical deterioration and fatigue.

2.1.2 Role Of Radiotherapy For Men With M0 Disease

Two randomised trials, SPCG7 (7) and NCIC PR.3 / MRC PR07 (8-10) have tested the question of whether ADT alone combined with radiotherapy is the best treatment for patients with high-risk localised prostate cancer (N0M0). Both trials demonstrated an improvement in overall and disease specific survival from the addition of radiotherapy to ADT. The size of this overall survival benefit is substantial (hazard ratio 0.68 in SPCG7 and 0.77 in PR07). As these two mature, large, well-conducted randomised trials have demonstrated benefit, we now mandate that radiotherapy be standard for patients with N0M0 disease (i.e. no nodal or metastatic spread). Patients with node-negative M0 prostate cancer will only be allowed to enter the trial if standard radiotherapy is planned. Any patients with N0M0 disease for whom radiotherapy is contra-indicated should be discussed with the STAMPEDE team prior to inclusion. For patients with node-positive, M0 disease there are no randomised data on whether radiotherapy is indicated or not. However the NCIC PR.3 / MRC PR07 trial included patients with unknown nodal status who received whole pelvic radiotherapy (11) and demonstrated a large overall benefit. Additionally, non-randomised data from the STAMPEDE control arm suggests that the benefit observed in patients with N0M0 disease can be extended to those with pelvic nodal involvement. Therefore the STAMPEDE TMG recommends that pelvic nodal radiotherapy be considered for patients with node-positive, M0 disease at the discretion of the treating clinician (12).

2.1.3 Role Of Docetaxel For Men With M0 Or M1 Disease

The primary analysis of the "original comparisons" has shown docetaxel to significantly prolong survival (HR 0.78; 95% CI 0.66-0.93)(1). This is in support of the results of the CHAARTED trial which showed docetaxel improved survival in men with metastatic disease(13, 14). There was no evidence of heterogeneity in STAMPEDE in the treatment effect across any patient subgroups and median survival was improved by 10 months, from 71 to 81 months. In a well powered and pre-planned sub-group analysis of men with metastatic disease at randomisation the treatment effect was most apparent with a median survival benefit of 15 months. As a result the STAMPEDE TMG recommends that docetaxel should be strongly considered in all men with metastatic disease at presentation who are commencing ADT for the first time and are fit enough to receive chemotherapy.

Survival data for men without metastases at diagnosis is less mature but a statistically significant improvement in failure-free survival is seen, therefore, docetaxel may also be considered for men with high-risk non-metastatic disease who are commencing ADT for the first time and are fit enough to receive chemotherapy. Therefore, docetaxel is now permitted as part of the standard-of-care for all men entering STAMPEDE at the discretion of the treating clinician and patient.

2.2 RATIONALE

There are increasing numbers of treatments which are used post-relapse of first-line ADT in patients with CRPC, but there has been limited evidence as to which is associated with the best response, how they may be combined or sequenced or whether any of them might have a role as first-line treatment. An alternative approach is to investigate the addition of new drugs as part of first-line therapy in patients starting ADT. At this point, patients should be fitter and better able to tolerate treatment than when they have CRPC, and there is the possibility of having a larger and longer-lasting effect. This is the rationale for the evaluation of docetaxel, abiraterone, abiraterone and enzalutamide.

The increasing and widespread use of ADT in prostate cancer management has led to growing awareness of the adverse effects of LHRH. An alternative approach for improving long-term outcomes in patients is therefore to mitigate some of these side-effects. Many of these side-effects can affect quality-of-life as well as result in significant morbidities and potentially life-threatening consequences, particularly with prolonged treatment and in patients with exisiting co-morbidities.

For these reasons, metformin is being evaluated within the trial as a re-purposed treatment for prostate cancer, because of its potential anti-cancer effects (based on pre-clinical and epidemiological evidence) and the expectation that it may counteract the metabolic effects of long-term ADT. Similarly, transdermal oestradiol, another novel re-purposed treatment approach, is being evaluated as an alternative form of ADT which may be as effective or more effective than LHRH in treating prostate cancer but with fewer side-effects.

2.3 DESIGN

STAMPEDE (also known as MRC PR08) is an innovative, multi-arm multi-stage, multi-centre, randomised controlled trial. It initially assessed the effects of a bisphosphonate (zoledronic acid), a cytotoxic chemotherapeutic agent (docetaxel) and a cyclooxygenase (Cox-2) inhibitor (celecoxib), as single agents or combinations, in patients commencing long-term ADT for locally advancing or metastatic prostate cancer. For these questions, each comparison was divided into five stages such

that, for each investigational arm, safety and activity data were generated in the first four stages; an investigational arm could only proceed to the fifth and final stage of recruitment, where it would be assessed for effect on overall survival, if shown to be sufficiently safe and active at all prior activity stages. Patient data from all arms and all stages are, however, included in the final analyses of the primary outcome measure, even if the investigational arm did not proceed to the final stage. Of note, a second, pre-planned interim analysis failed to demonstrate sufficient activity for celecoxib and this agent was removed from trial recruitment in Apr-2011; patients remaining on celecoxib treatment reverted to standard care. Results for all of these "original comparisons" have now been reported(2, 15).

Since the start of the trial, a number of new research arms have been added to STAMPEDE over time to evaluate: abiraterone, a steroid synthesis inhibitor; prostate radiotherapy for patients with newly-diagnosed metastatic disease; enzalutamide, an inhibitor of androgen receptor signalling, given with abiraterone; and metformin, an anti-diabetic medication. In Protocol version 16.0, a new research arm is added for transdermal oestradiol, to be given as an alternative form of ADT.

2.4 PREVIOUSLY-REPORTED RESEARCH TREATMENTS

Data have been reported on zoledronic acid, docetaxel, celecoxib and the combination of zoledronic acid with docetaxel or with celecoxib. As such the rationale for these treatments, along with their design and details of treatment administration are no longer covered within the Protocol.

2.5 RATIONALE FOR RESEARCH TREATMENTS UNDER EVALUATION

2.5.1 Steroid Synthesis Inhibitors

Note: recruitment to both the abiraterone containing comparisons has now been completed as the required target accrual was reached.

Recent evidence suggests that an important mechanism for escape from tumour control by androgen ablation is the intracellular conversion of steroid precursors to androgenic steroids by prostate cancer cells. A key enzyme in this process is CYP17, which therefore represents a logical target for therapy in CRPC(16). Abiraterone acetate (3β-acetoxy-17-(3-pyridyl)androsta-5,16-diene, code CB7630; JNJ-212082) is rapidly converted in vivo to abiraterone (JNJ-589485; formerly code named CB7598). It is a selective, irreversible inhibitor of 17α-hydroxylase/C17,20-lyase (cytochrome P450c17 [CYP17]), an enzyme that is critical in the production of androgens in the testes, adrenal glands and prostate tumour tissue. Inhibition of CYP17 inhibits the conversion of pregnenolone or progesterone into dehydroepiandrosterone (DHEA) or androstenedione, respectively, each of which is a precursor of testosterone. The pharmacodynamic effect is a more effective androgen depletion than can be induced by surgical castration, or medically by gonadotropin releasing (GnRH) hormone analogues used as first-line hormone therapy in prostate cancer.

Approximately 2,280 prostate cancer patients participated in the two Phase 3 RCTs (COU-AA-301 and COU-AA-302), with approximately 1,335 patients receiving abiraterone acetate at 1000mg daily dose continuously, in these studies. These studies have demonstrated abiraterone to prolong survival when given post-docetaxel (HR 0.65) and pre-docetaxel (HR 0.82). As a result it is now approved use in the USA and Europe in CRPC (17, 18).

Side-effects with abiraterone acetate are modest with the main adverse effects being elevated transaminases (usually mild), hypokalaemia and hypertension due to secondary hyperaldosteronism and fluid retention (preventable by low doses of glucocorticoids). In order to prevent secondary hyperaldosteronism, it is recommended that prednisolone (or prednisone) 10mg daily be administered in the CRPC setting. Within more recent studies in earlier stage patients, lower doses (typically 5mg of prednisone/prednisolone) are being used due to concerns about side effects of long-term exposure to glucocorticoid. Within the STAMPEDE trial, we suggest prednisolone/ prednisone dose of 5mg OD, which may be increased to 5mg BID at the investigator's discretion if there are any concerns about monitoring or risks for the patient with 5mg OD.

We hypothesise that abiraterone may be more active still, when given up-front in combination with first-line ADT, by preventing or delaying the development of castrate refractory disease.

2.5.2 Radiotherapy To The Prostate For Patients With Newly-Diagnosed Metastatic Disease

Note: recruitment completed to the radiotherapy arm in Sep-2016 as the revised recruitment target sample was reached. Treatment has been completed in all patients and the results will be reported when the data has matured. See Protocol version 15.0 or older for details on the rationale.
(7, 11, 19, 20)

2.5.3 Combination Of Steroid Synthesis Inhibitors And Androgen Receptor Signalling Inhibitor

The most common form of disease progression for men on single-agent abiraterone or enzalutamide is a rise in PSA. This would suggest that the mechanism driving resistance is increased PSA transcription resulting from reactivation of the androgen receptor (AR), or another steroid signalling pathway(21).

The primary pharmacodynamic effect of enzalutamide is inhibition of androgen binding to the AR, AR nuclear translocation in the presence of androgen and AR:chromatin association. In multiple prostate cancer cell lines that specifically model CRPC (LNCaP/AR, VCaP, W741C LNCaP), the consequences of enzalutamide treatment include inhibition of AR-induced gene transcription, reduced cell proliferation, increased cell death by apoptosis and tumour regression.

In a mouse xenograft model of CRPC using prostate cancer cells that overexpress the AR (LNCaP/AR), enzalutamide inhibits tumour growth and reduces tumour size. A major human metabolite of enzalutamide, N-desmethyl enzalutamide, demonstrates key primary pharmacodynamics of similar potency to the parent molecule, while the carboxylic acid derivative metabolite has no known pharmacodynamic effect.

The question under investigation is: can progression be delayed (and survival extended) by using a combination of abiraterone and enzalutamide given up-front in combination with first-line ADT?

2.5.3.A Supplementing Abiraterone And Prednisolone With Enzalutamide

Several studies have shown that the AR can become promiscuously activated by very low levels of androgens or other steroid metabolites and drugs that bind the AR (22-25). It is known that very low levels of androgens can persist in patients treated with abiraterone acetate (26). Drugs that bind the AR, may include co-administered glucocorticoids. Furthermore, AR mutations of the sort previously described in CRPC, can be activated by cortisol and other glucocorticoids at levels much lower than those reported in patients treated with abiraterone and prednisolone at a dose of 5mg bid (25, 27). Moreover, abiraterone binds the AR and, although weak antagonism of wild-type and most

previously described AR mutations are observed (27), a similar mechanism to that described with classical anti-androgens, such as bicalutamide, could lead to change-of-function AR mutations associated with AR activation following abiraterone binding. Therefore, concomitant treatment with an androgen receptor signalling inhibitor could prevent "promiscuous" AR activation in patients treated with abiraterone. Enzalutamide is an androgen receptor signalling inhibitor and is approved for use on its own in the treatment of advanced CRPC (28), and there is evidence of activity for hormone-naïve prostate cancer (29).

2.5.3.B Supplementing Enzalutamide With Abiraterone And Prednisolone

Enzalutamide in combination with ADT is both effective and well tolerated in CRPC(28). However, recent studies have suggested that intra-tumoral testosterone levels increase in patients treated with enzalutamide (30). The implications of this finding are that the increase in intra-tumoral testosterone could be associated with up-regulation of enzymes involved in steroid biosynthesis(31). Although enzalutamide has a high affinity for the AR, this is several-fold lower than both the natural ligands testosterone and DHT (32), which means that enzalutamide would be out-competed at the AR ligand-binding domain if and when androgen levels rise. In vitro, a ten-fold rise in intra-cellular androgen was sufficient to prevent inhibition of AR by 30uM of enzalutamide(27); these levels are representative of the plasma levels of enzalutamide active metabolites, which can be achieved with enzalutamide 160mg po daily (33).

A strategy for preventing the rise in intra-cellular androgens in patients treated with enzalutamide would be inhibition of CYP17A1. Abiraterone is currently the only CYP17A1 inhibitor with proven efficacy. It therefore seems logical to use the combination of enzalutamide and abiraterone to both block a rise of intra-cellular androgens and prevent promiscuous activation of the AR.

2.5.3.C Summary Of Rationale For This Combination

To date, investigation has focussed on patients with CRPC but there is a strong rationale for the combination of enzalutamide and abiraterone in the hormone treatment-naïve setting in which STAMPEDE is focused.

STAMPEDE is already evaluating abiraterone plus conventional ADT but we will not assess the combination of conventional ADT plus enzalutamide; other trials by industry and other cooperative groups will address that question. The inclusion of an arm with ADT and enzalutamide in STAMPEDE was therefore considered to be a duplication of effort and was not supported by the Trial Management Group.

The combination of enzalutamide and abiraterone is a novel approach and offers considerable promise in delaying progression – it therefore represents an attractive addition to the comparisons under investigation in STAMPEDE, and one that is unlikely to be replicated in other planned trials of this size.

2.5.4 Metformin

All men joining STAMPEDE are planned for long-term ADT, a treatment associated with an increased risk of insulin resistance, hyperglycaemia, dyslipidaemia and obesity. Over 50% of men receiving long-term ADT will develop Metabolic Syndrome (34) resulting in increased cardiovascular morbidity and mortality. Obesity and high bind insulin C-peptide levels, indicating insulin resistance are independent predictors of increased prostate cancer-specific mortality and the presence of metabolic syndrome and diabetes in men treated with ADT is associated with shorter survival.

Metformin, which in non-diabetic individuals has been shown to lower the incidence of diabetes, counteracts some of these side-effects of ADT, including insulin insensitivity, hyperinsulinaemia and diabetes. It also reduces the levels of cholesterol, LDLs and triglycerides by inhibiting the fatty acid synthesis via activation of Adenosine Monophosphate Activated Kinase (AMPK) and decreases the platelet aggregation factor 1, platelet aggregation, vascular adhesion molecules, CRP and leptin (35-38). Through mitigation of the cardiovascular and metabolic consequences of ADT, metformin is proposed to reduce treatment-associated morbidity and improve all-cause mortality.

In addition, recent data has emerged consolidating the knowledge that cancer progression is linked integrally with metabolic modulators and that modification of this process by metformin has an important effect on cancer progression and survival. Pre-clinical data has shown that metformin is an important stimulator of AMPK which acts as the cellular “masterswitch” for energy regulation. AMPK acts to inhibit the effects of elevated insulin levels which promote metastasis, tumour growth and treatment resistance. Insulin increases mRNA and protein expression of steroidogenic enzymes leading to the up-regulation of intracellular testosterone levels, secreted androgens, thereby activating the AR (39). Metformin also influences the PI3K-AKT pathway and has an anti-proliferative effect via inhibitor of mTOR as well as targeting cancer stem cells. In vitro, metformin has been shown to inhibit androgen-induced IGF-IR up-regulation through disruption of androgen signalling (40).

Evidence in support of this includes a systematic review and meta-analysis of 13,008 men with type 2 diabetes mellitus (T2DM) and concurrent cancer which has shown improved survival in men treated with metformin compared with other anti-diabetic agents. In a systematic review of observational data from over 1 million men, there was a significant association seen between metformin and decreased risk of death from any cancer. Another systematic review found that the use of metformin in diabetic patients was associated with a significantly lower risk of cancer incidence and cancer mortality (41). In a large retrospective cohort study of 3837 diabetic men with prostate cancer, metformin was associated with a decreased risk of prostate cancer specific mortality (HR=0.76 [0.64-0.89]) and death (HR=0.76 [0.70-0.82]). In a prospective non-randomised phase II study in non-diabetic CRPC patients, 36% of patients receiving metformin were progression-free at 3 months and >50% had a prolongation of their PSA doubling time (42).

In summary, meformin is proposed to mitigate many of the adverse side effects of long-term ADT as well as having multiple potential anti-cancer effects and therefore STAMPEDE will evaluate re-purposing this treatment as a novel therapeutic approach in the management of high risk locally-advanced or metastatic prostate cancer.

2.5.5 Transdermal Oestradiol

2.5.5.A Background & Rationale

ADT with LHRH analogue injections suppresses testosterone to castrate levels, but also depletes oestradiol, since around 80% of oestradiol in men is derived by aromatisation from testosterone. Thus men who are treated with LHRH will have toxicities caused by low levels of both testosterone and oestrogen. The LHRH-associated toxicities which are due to low testosterone include loss of libido, erectile dysfunction and decrease in muscle mass. Other toxicities associated with LHRH such as osteoporosis, increased fracture risk, hot flushes, memory loss, dyslipidemia and increased body fat deposition are thought to be due to oestradiol deficiency. In particular, the adverse effect of LHRH on bone health has been well documented. Oestradiol deficiency prolongs the life-span of bone-resorptive osteoclasts, with the resulting imbalance between osteoclasts and bone-forming osteoblasts increasing the rate of bone thinning. This may lead to osteoporosis and increased risk of fracture, with the rate of fracture increasing with duration of LHRH.

Transdermal oestradiol is a potential alternative to LHRH that may avoid some treatment-related side-effects, therefore improving quality-of-life, which would be advantageous if shown to be equally effective at prolonging survival. Exogenous administration of oestradiol suppresses androgen production through a negative feedback loop involving the hypothalamic-pituitary axis, whilst avoiding the fall in oestradiol associated with castrate levels of testosterone(43). This, in turn, mitigates the toxicities of LHRH associated with oestradiol deficiency. Oral oestrogen was previously used for ADT before the development of LHRH, but discontinued as first-line treatment due to increased thromboembolic toxicity, attributable to first-pass hepatic metabolism (44).

Parenteral administration (e.g. intravenous, intramuscular or transdermal oestradiol) avoids first-pass hepatic metabolism, mitigating the cardiovascular risk, as supported by results so far from the ongoing PATCH (Prostate Adenocarcinoma TransCutaneous Hormones [MRC PR09; ISRCTN70406718]) trial and previous studies evaluating parenteral oestradiol in the form of intramuscular polyestradiol phosphate (43, 45).

To date, there are a number of encouraging results from the PATCH trial demonstrating the safety and early activity of transdermal oestradiol compared to LHRH agonists in men with advanced hormone-naïve prostate cancer (see **Appendix I** for further details). In particular, similar rates of cardiovascular events have been observed in the transdermal oestradiol and LHRH arms, as well as equivalent rates of testosterone suppression (based on around 900 patients enrolled up to Oct-2015) (43). Transdermal oestradiol has been shown to avoid the loss in bone mineral density associated with LHRH, and results in improved metabolic profiles and quality-of-life compared to LHRH(46). Furthermore, a pre-planned, confidential, interim analysis of the PATCH trial in Jun-2013 based on progression-free survival (PFS) led to the trial being extended to phase III; that analysis included n=638 participants with 206 PFS events, reviewed against a pre-specified non-inferiority margin hazard ratio of 1.25 and 1-sided alpha 0.25. The phase III evaluation of clinical efficacy for transdermal oestradiol will be based on progression-free and overall survival as co-primary outcome measures.

Demonstrating that transdermal oestradiol is an equally effective approach to ADT would provide a globally important alternative (to LHRH), with the potential to reduce treatment-associated morbidity and improve quality-of-life. In addition, there is a possibility that transdermal oestradiol may improve overall survival compared to standard hormone therapy. First, transdermal oestradiol may reduce treatment-associated morbidity and could potentially benefit overall survival. Second, up to 30% of men with castrate-resistant prostate cancer respond to oral oestrogen as post-relapse therapy, suggesting oestradiol may potentially have additional direct anti-tumour effects(47).

2.5.5.B Meta-Analysis With PATCH Trial

To further assess the clinical efficacy of transdermal oestradiol, the relevant data from the “transdermal oestradiol comparison” within STAMPEDE will be combined with data from patients recruited into PATCH i.e. the “transdermal oestradiol comparison” within STAMPEDE is not sufficiently powered to form a stand-alone analysis. The evaluation of transdermal oestradiol will be based on a non-inferiority approach (in contrast to the other comparisons within STAMPEDE which are superiority questions), to test the hypothesis that transdermal oestradiol is at least as effective as standard hormone therapy, but with fewer side-effects.

Recruitment of patients to the “transdermal oestradiol comparison” through STAMPEDE enables the transdermal oestradiol research question to be answered more quickly than via PATCH alone. It also reduces the number of patients allocated standard treatment alone in both trials, thereby increasing the proportion of patients receiving a novel treatment approach and improving trial efficiency.

As of Feb-2017, nearly 1,200 patients had been recruited directly to the PATCH trial (also coordinated by MRC CTU at UCL) for the phase III evaluation of clinical efficacy of transdermal oestradiol. The overall recruitment target for the transdermal oestradiol evaluation is approximately 2,000 patients (including initially around 500 to be recruited through STAMPEDE).

3 SELECTION OF INSTITUTIONS AND INVESTIGATORS

Centres who wish to participate in the STAMPEDE trial should be registered with the MRC CTU at UCL for this purpose. Before any patients are registered or randomised, the CTU must receive a completed and signed Investigator Statement. The STAMPEDE Investigator Statement is signed by the Principal Investigator for that institution (download from http://www.stampedetrial.org/). The return of the Investigator Statement will be taken as confirmation of agreement to adhere to the trial protocol. In addition, a fully-signed model agreement is also required before recruitment can begin.

In compliance with the principles of GCP, all institutions participating in the trial will complete a delegation log and forward this to the CTU. Each person working on the STAMPEDE trial must sign off a section of this log indicating their responsibilities. CTU must be notified of any changes to trial personnel and/or their responsibilities. An up-to-date copy of this log must be stored in the Investigator Site file at the institution and also at CTU.

The Clinical Trial Authorisation (CTA) for the STAMPEDE trial requires that the Medicines and Healthcare Products Regulatory Agency (MHRA) be supplied with the names and addresses of all participating investigators/institutions. Trial staff at the CTU will perform this task; hence, it is vital to receive full contact details for all investigators prior to their entering patients.

Finally, before a patient is entered into the trial, and any trial-related procedures are conducted, written informed consent must be obtained. Approved patient information sheets and informed consent forms are supplied as templates.

Only a limited number of centres participated in the initial Pilot Phase of the original trial; this was to ensure that safety and feasibility data were collected expediently. Subsequent stages of the trial are open to any centre that wishes to participate and has fulfilled the requirements described above. In addition for some comparisons, there will be additional criteria required prior to accreditation, see **Sections 3.1** and **3.2**.

Following substantial amendments and future comparisons opening, sites will be notified of relevant documents and training required and if and when they are able to participate. Further accreditation packs may be circulated as a result to update trial documentation.

3.1 COMPARISON-SPECIFIC SITE ACCREDITATION

3.1.1 "Transdermal Oestradiol Comparison"

Only UK centres participating in STAMPEDE will be accredited for the "transdermal oestradiol comparison", since treatment with transdermal oestradiol is administered using Progynova TS 100mcg/24 hours transcutaneous oestradiol patches (see **Section 6.2.8**) which are currently unavailable in Switzerland.

3.2 FUTURE PLANNED BIOMARKER-SELECTED COMPARISONS

Initially, only UK centres participating in STAMPEDE will be accredited for the biomarker-selected comparisons, which are in development and will be incorporated in the next version of the protocol.

Sites wishing to gain accreditation for this future comparison should participate in the biomarker-screening pilot, described from Protocol version 16.0 onwards.

Full details of the accreditation procedure for the future biomarker-selected comparisons will be available in the next version of the protocol. Centres participating in the biomarker-screening pilot will need to complete a feasibility assessment and will be provided with additional training prior to activation of the pilot. An additional agreement signed by the Head of Histopathology Service with contact details of a designated secretarial or technical person at each site will be required from each site participating in biomarker testing.

Please refer to the **Biomarker-screening manual** for further details.

3.3 REQUIRED TRIAL DOCUMENTATION

Table 5 presents a summary of the required trial documentation for participating centres. Templates are provided on the STAMPEDE website www.stampedetrial.org.

Table 5: Trial documentation required for participating centres

TRIAL DOCUMENTATION	TIMING
R&D approval (or local equivalent; including IRMER approval)	Before centre participation
Signed Investigator Statement	Before centre participation
Signature list & delegation of responsibilities	Before centre participation
Trial personnel contact details	Before centre participation
PIS, GP & CF on local paper	Before centre participation
Signed Clinical Trial Agreement between Trust and Sponsor (or Variation, if applicable)	Before centre participation
Site initiation training	Before centre participation
Signed Pharmacy Pack acknowledgment	Before centre participation
Signed Pathology Agreement (for sites participating in biomarker-screening)	Before centre participation in biomarker screening

4 SELECTION OF PATIENTS

4.1 GENERAL INCLUSION CRITERIA

Participants must fulfil both of the criteria in Section 4.1.1 **or at least** one criterion in Section 4.1.2 **or** at least one criterion in Section 4.1.3. Additionally, all patients must fulfil the criteria in Section 4.1.4.

4.1.1 High-Risk Newly-Diagnosed Non-Metastatic Node-Negative Disease

Both:

- At least two of: T category T3/4, PSA≥40ng/ml or Gleason sum score 8-10
- Intention to treat with radical radiotherapy (unless there is a contra-indication; exemption can be sought in advance of consent, after discussion with CTU)

OR

4.1.2 Newly-Diagnosed Metastatic Or Node-Positive Disease

At least one of:

- Stage T_{any} N+ M0
- Stage T_{any} N_{any} M+

OR

4.1.3 Previously Radically Treated, Now Relapsing (Prior Radical Surgery And/Or Radiotherapy)

At least one of:

- PSA ≥4ng/ml and rising with doubling time less than 6 months
- PSA ≥20ng/ml
- N+
- M+

AND

4.1.4 For All Patients

I. Histologically confirmed prostate adenocarcinoma
II. Intention to treat with long-term androgen deprivation therapy
III. Treating clinician and patient should have decided if docetaxel is to be part of the standard-of-care prior to randomisation
IV. Fit for all protocol treatment[1] and follow-up, WHO performance status 0-2[2]
V. Have completed the appropriate investigations prior to randomisation
VI. Adequate haematological function: neutrophil count >$1.5x10^9$/l and platelets >$100x10^9$/l
VII. Adequate renal function, defined as GFR >30ml/min/$1.73m^2$
VIII. Serum potassium ≥3.5mmol/L
IX. Written informed consent
X. Willing and expected to comply with follow-up schedule
XI. Using effective contraceptive method if applicable

[1] Medical contraindications to the trial medications are given in Section 6
[2] For WHO performance status definitions see Appendix A

4.2 GENERAL EXCLUSION CRITERIA

Patients must not fulfil any of the criteria, below. In addition, see **Sections 4.4.1** and **4.4.2** for comparison-specific criteria.

I. Prior systemic therapy for locally-advanced or metastatic prostate cancer except as listed in **Section 4.1.3**
II. Metastatic brain disease or leptomeningeal disease
III. Abnormal liver functions consisting of any of the following:
   - Serum bilirubin ≥1.5 x ULN (except for patients with Gilbert's disease, for whom the upper limit of serum bilirubin is 51.3µmol/l or 3mg/dl)
   - Aspartate aminotransferase (AST) or alanine aminotransferase (ALT) ≥2.5 x ULN
IV. Any other previous or current malignant disease which, in the judgement of the responsible clinician, is likely to interfere with STAMPEDE treatment or assessment
V. Any surgery (e.g. TURP) performed within the past 4 weeks
VI. Patients with significant cardiovascular disease, including:
   - Severe/unstable angina
   - Myocardial infarction less than 6 months prior to randomisation
   - Arterial thrombotic events less than 6 months prior to randomisation
   - Clinically significant cardiac failure requiring treatment (NYHA II-IV)[3]
   - Cerebrovascular disease (e.g. stroke or transient ischaemic episode) less than 6 months prior to randomisation
   - Patients with uncontrolled hypertension defined as systolic BP greater or equal than 160mmHg or diastolic BP greater or equal than 95mmHg[4]
   - Or any other significant cardiovascular disease that in the investigator's opinion means the patient is unfit for any of the study treatments.
VII. Prior chemotherapy for prostate cancer (excluding patients receiving docetaxel as part of the new SOC)
VIII. Prior exposure to long-term hormone therapy before randomisation (unless as described in **Section 4.5.4**)
IX. Prior exposure to systemic treatment for prostate cancer (excluding hormone therapy) e.g. abiraterone and enzalutamide.

[3] NYHA classifications can be found in **Appendix A**
[4] Based on representative values, as judged by the investigator

4.3 BIOMARKER-SCREENING PILOT

4.3.1 Selection Criteria For Patient Registration

In preparation for the introduction of biomarker-selected comparisons, a Biomarker-Screening Pilot is being undertaken, described in Protocol version 16.0 and onwards. This will be undertaken in a limited number of sites. Participation in the pilot will help to facilitate site accreditation for recruitment to the future biomarker-selected comparisons see Section 3.2.

All patients who fulfil the criteria below should be **registered without delay** in order to proceed to biomarker-screening.

- Registered at a site participating in biomarker-screening
- Confirmed metastatic disease (M1)
- Recent FFPE tumour sample available for prompt transfer (sample must have been obtained within 8 months prior to date of registration)
- Written informed consent provided for the STAMPEDE trial and for biomarker-screening
- If hormone therapy has started, FFPE tumour blocks should be **sent within**:
    - **8 weeks** of the patient starting LHRH
    - **10 weeks** of starting anti-androgens

  This is to allow for a turnaround time for biomarker analysis results of 4 weeks.

Once eligibility is confirmed complete the registration CRF and contact the CTU to proceed to trial registration, see Section 5.1.

At the point of registration three samples are required to complete the biomarker-screening.

- Expedited retrieval of FFPE tumour block.
- Baseline blood sample collected using cell free DNA Streck™ tubes
- Saliva sample

Please refer to the Biomarker-screening manual for further details.

Registration will occur **before** the patient is randomised. In the pilot phase, results of biomarker-screening are **not** required prior to randomisation as recruitment has not yet been activated to the "rucaparib comparison". Therefore patient can be randomised straight after being registered. For further details on results and feedback to patients, please see Section 4.6.5.

Patients who participate in the pilot will continue to be allocated to any of the current open arms for which they are eligible (arm A, L and K). When recruitment is activated to biomarker-selected comparisons, the results of biomarker-screening will determine eligibility and will be required prior to randomisation. The screening pilot will inform the randomisation process for future biomarker-selected comparisons.

4.4 COMPARISON-SPECIFIC SELECTION CRITERIA

4.4.1 For Randomisation To Include The "Metformin Comparison"

Patients with known diabetes mellitus are not eligible for randomisation to the "metformin comparison".

All non-diabetic patients require an HbA1c to be performed prior to randomisation (ideal timeline: within 8 weeks prior to randomisation), to confirm their non-diabetic status.

In addition, an assessment of renal function is required to determine glomerular filtration rate (GFR). The method used to determine glomerular filtration rate may vary according local practice. Equations that either estimate glomerular filtration rate (eGFR) or creatinine clearance (CrCl) may be used and the same threshold value applies.

In summary, additional inclusion criteria specifically for the "metformin comparison" are:

- HbA1c <48mmol/mol (equivalent to <6.5%)
- Adequate renal function, defined as GFR ≥45ml/min/1.73m^2 *
- No history of lactic acidosis or pre-disposing conditions
- Not current or previous treatment with metformin
- No contra-indications to metformin

*Except Switzerland, please refer to SAKK appendix for local guidance

Note that if the patient is known to be diabetic or the patient is found to have diabetes mellitus (i.e. HbA1c is 6.5% or higher) following screening, the patient is only eligible for randomisation if they meet all of the selection criteria for the “transdermal oestradiol comparison” (randomisation between Arms A and L only; see **Section 4.4.2**).

All patients with abnormal baseline HbA1c (i.e. 6.0% or higher) should be informed and referred to their GP for further management.

Where possible, the screening bloods, including HbA1c, should be performed prior to commencing SOC docetaxel. This is to reduce the likelihood of corticosteroid-related hyperglycaemia impacting on eligibility for the "metformin comparison".

4.4.2 For Randomisation To Include The “Transdermal Oestradiol Comparison”

Patients who have any of the following are not eligible for the “transdermal oestradiol comparison”:

- >8 weeks of anti-androgen use
- >1 dose of monthly or 4 weekly LHRH agonist/antagonist
- Prior LHRH agonist injection with a stated duration of effect greater than 1 month
- >12 weeks since first dose of any hormone therapy
- Bilateral orchidectomy
- Cyproterone acetate started prior to randomisation
- Known porphyria
- Any history of deep vein thrombosis or pulmonary embolism confirmed radiologically
- Known thrombophilic disorder (e.g. Protein C, Protein S, antithrombin deficiency)

Note that patients unsuitable for the “transdermal oestradiol comparison” will only be eligible for randomisation if they meet all of the selection criteria for the “metformin comparison” and therefore may be allocated to control (arm A) or metformin (arm K) only (see **Section 4.4.1**).

Patients presenting with relapsed disease who fulfil criteria in **Section 4.1.3** are also eligible for the “transdermal oestradiol comparison” providing the neo-adjuvant or adjuvant hormone therapy previously received adheres to criteria outlined in **Section 4.5.4.**

4.5 SCREENING PROCEDURES

Table 6: Summary of initial required screening and baseline investigations

TIMEPOINT	PATIENTS PARTICIPATING IN BIOMARKER-SCREENING PILOT
PRE-REGISTRATION	✓ Confirmation of metastatic disease<br>✓ FBC, U&Es, LFTs, Creatinine or estimated GFR<br>✓ Retrieval of recent FFPE tumour block (within 8 months of registration)
AT REGISTRATION	✓ Transfer of FFPE tumour block<br>✓ Blood collection (cell free DNA Streck™ tubes)<br>✓ Saliva sample
**TIMEPOINT**	**ALL PATIENTS**
PRIOR TO RANDOMISATION	✓ Bloods: FBC, U&Es, LFTs, Creatinine, , PSA (including pre-treatment PSA within 6 months of randomisation)<br>✓ Cardiac: ECG, BP<br>✓ Imaging: Bone scan, CT or MRI pelvis and abdomen, CXR if chest not included in CT

Key: FBC= Full blood count, LFT= liver function test, U&E= Urea and electrolytes, GFR=glomerular filtration rate, BP= blood pressure

4.5.1 Investigation Prior to Registration (for patients participating in biomarker-screening pilot)

Sufficient screening investigations must have been completed to ensure that patient fulfil all of the selection criteria for the Biomarker-Screening Pilot, see **Section 4.3.1** prior to registration. In addition, confirmation of adequate organ function should be obtained through baseline bloods which at a minimum include FBC, U&Es and LFTs. Metastatic disease should be confirmed radiologically but all the required screening scans may not have been completed. All remaining screening investigations e.g. additional blood and imaging, may be completed following registration prior to randomisation (See **Table 1** and **Table 6**).

Please refer to the **Biomarker-screening manual** for further details relating to sample requirements.

4.5.2 Investigations Prior To Randomisation

All patients should have the following examinations performed to confirm eligibility prior to randomisation.

The following standard imaging is required and the latest available scans that reflect the patient's current disease status should be used:

- CT or MRI of pelvis and abdomen
- Bone Scan (or equivalent e.g. whole body MRI, choline-PET-CT, PSMA-CT-PET)
- Chest X-ray (only if chest was not included in CT or MRI which would be preferable)

Any additional imaging such as CT-PET scanning can be performed according to local practice but, for the purposes of the trial, the recorded stage should be the CT stage only; additional information on the CT-PET stage will also be collected.

The following bloods and additional measurements are required prior to randomisation:

- ECG

- Pre-hormone treatment PSA (this must be obtained within 8 months of randomisation)
- HbA1c
- Full blood count
- Urea and Electrolytes
- Liver function tests
- Serum creatinine
- Systolic and diastolic blood pressure

Patients who initially fail to meet the trial eligibility criteria can be re-screened at a later date. Of note, for patients receiving standard-of-care docetaxel at the point of screening, it is acceptable to use a full blood count measurement prior to chemotherapy to confirm eligibility.

Prior to randomisation:

- Check details of any prior treatments for prostate cancer
- Check any contraindications to radiotherapy or research treatment
- Check concomitant medications

4.5.3 Additional Baseline Investigations

The following blood tests are required at baseline (within 4 weeks before or after randomisation):

- Testosterone (pre-ADT, if available)
- Serum corrected calcium
- Phosphate
- Magnesium
- Albumin
- Fasting glucose (mandatory)
- Fasting triglycerides (mandatory)
- Lipid profile (fasting or non-fasting; total cholesterol, LDL and HDL) (mandatory)

The following additional procedures are required and **mandatory** at baseline:

- Waist circumference measurement
- Weight and height

A trial screening log will be *available* to all centres; copies are *not required* at this time (unless a specific issue is identified at a given site by the STAMPEDE Trial Team).

4.5.4 Androgen Deprivation Therapy Prior To Randomisation

From Protocol version 16.0, patients can potentially be randomised to the “transdermal oestradiol comparison” and it would be preferable for these patients to have had as little exposure to ADT as possible.

Within the separate PATCH trial, patients are randomised within 8 weeks after starting anti-androgens and cannot have received an LHRH injection. This approach is also favoured in STAMPEDE, but patients who have received a single 4-week (or 1-month) LHRH injection remain eligible for randomisation to the “transdermal oestradiol comparison”, as shown in **Figure 3**.

For all other comparisons, if ADT has already started prior to randomisation, the first LHRH injection must have been given within 12 weeks prior to randomisation. Additionally, if anti-androgens are being used, these must have started within 14 weeks prior to randomisation. A PSA level must have been taken prior to starting long-term hormone treatment. Note that baseline testosterone

measurements will not be required in patients who have already commenced hormone manipulation prior to randomisation.
Figure 3 illustrates the maximum duration of ADT allowed pre-randomisation.

Note that anti-androgen monotherapy is not permitted as a form of long-term hormone therapy. It is accepted that sites participating in the Biomarker-Screening Pilot are unlikely to meet the "preferred timeline for randomisation".

Figure 3: Maximum duration of hormone therapy allowed prior to randomisation and the latest point of randomisation

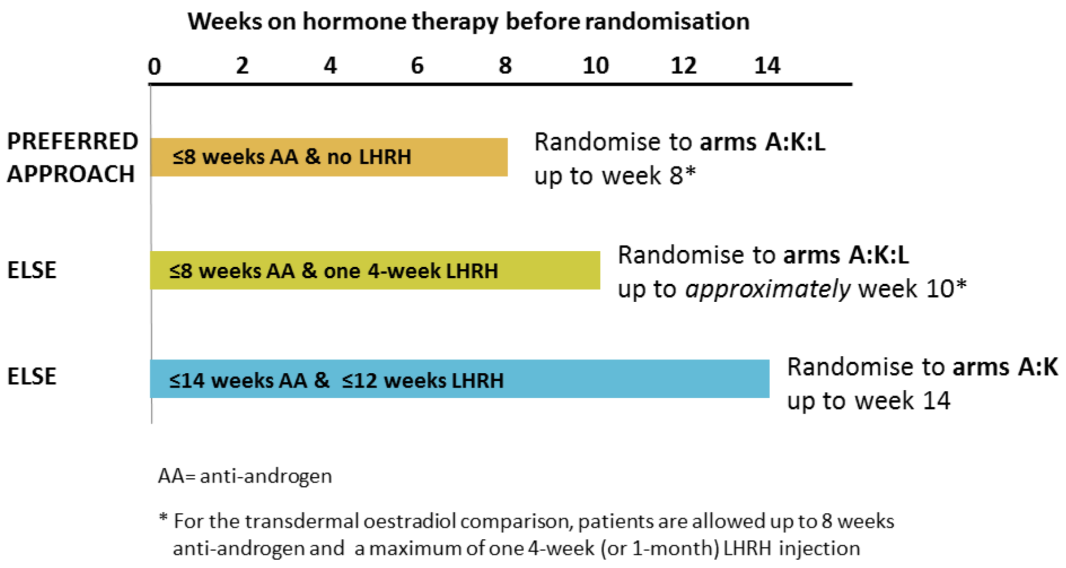

Permitted prior hormone therapy for now-relapsing disease: Any patients now presenting with relapsed disease, previously treated with adjuvant or neo-adjuvant hormone therapy alongside their radical surgery or radiotherapy, must have completed that period of hormone therapy **at least 12 months** before joining STAMPEDE and it must have been **no longer than 12 months in duration**.

4.5.5 Standard-Of-Care (SOC) Radiotherapy

The treating clinician and patient must have decided, prior to randomisation, whether radiotherapy is to be given as part of standard-of-care (SOC).

4.5.6 Standard-Of-Care (SOC) Docetaxel

The treating clinician and patient must have decided, prior to randomisation, whether docetaxel is to be given as part of standard-of-care (SOC). SOC docetaxel treatment should start within 14 weeks after starting hormone therapy (ideal timeline: within 8-10 weeks). Patients can have already started docetaxel treatment when randomised providing this is within 14 weeks after starting hormone therapy.

For those planned for SOC docetaxel and subsequently randomised to receive transdermal oestradiol (Arm L), it is recommended that docetaxel treatment commences *after* patients have

been established on transdermal oestradiol for around 4 weeks, when most patients are likely to have completed the induction period (see **Section 6.2.8**).

4.5.7 Starting Trial Treatment

4.5.7.A Metformin

For all patients allocated to metformin, treatment should start as soon as possible after randomisation. Investigators should aim that this is at least within 4 weeks post-randomisation and within 12 weeks of starting hormone therapy (see **Section 6.2.7**). Metformin can be given in combination with SOC docetaxel.

4.5.7.B Transdermal Oestradiol

For all patients allocated to transdermal oestradiol, treatment should start as soon as possible after randomisation (and ideally within 1 week after randomisation). It is not necessary to wait for completion of the 4-week (or 1-month) duration of the LHRH injection, if this was previously given prior to randomisation. For those prescribed bicalutamide or flutamide prior to randomisation, this treatment should be discontinued before treatment with transdermal oestradiol can commence (no washout period is needed).

4.5.8 Concomitant Medications

From Protocol version 15.0 onwards the trial requires the reporting to CTU of information regarding planned or actual long-term (>6 months) use of the following concomitant medications of classes of interest.

- Statins
- Metformin
- Aspirin
- Bisphosphonates or denosumab
- Opiate pain killers
- ACE inhibitors or angiotension II antagonists

This information is of interest both in terms of baseline use and ongoing use through the trial; as such it should be recorded on the Randomisation CRF and will be collected at each follow-up assessment (see **Table 1**).

Caution should be exercised when starting any concomitant medications that may result in a worsening of renal function e.g. initiating anti-hypertensive therapies such as ACE inhibitors, diuretics such as frusemide, or starting a non-steroidal anti-inflammatory drug (NSAID). Please refer to **Table 18** for more information on drugs which may require additional monitoring of renal function.

All concomitant medications should be continued throughout the trial unless the responsible clinician decides otherwise. If patients continue to require medication for the management of docetaxel-related toxicities, please discuss this with the trial team. See **Section 6.3** for more information on concomitant medications and their use with abiraterone, enzalutamide and transdermal oestradiol.

4.6 ADDITIONAL DETAILS FOR PATIENTS JOINING SUB-STUDIES

All patients joining STAMPEDE are asked for additional, optional consent to provide samples for three ongoing sub-studies. Since Mar-2016, all consent for additional research is recorded on a separate form to consent for the trial.

For details regarding sample collection, please refer to the **Sample collection and handling manual**.

For details relating to samples obtained for biomarker-screening please refer to the **Biomarker-screening manual**.

4.6.1 Germline DNA Analysis (Saliva Samples)

In collaboration with Professor Ros Eeles, Institute of Cancer Research, London, DNA is being extracted from saliva samples provided by consenting participants enrolled in STAMPEDE.
The aims of this sub-study are to examine the germline (inherited) genetic changes present in men with high-risk localised or metastatic prostate cancer. The aim is to determine the prevalance of germline genetic aberrations present pre-diagnosis and to correlate prostate cancer risk SNP genetic profiles, identified in Genome-wide Association Studies (GWAS) and other sequence variants from next generation sequencing (NGS), with duration of response to ADT and the experimental treatments tested in STAMPEDE.

All patients joining the trial are asked to consent to provide a saliva sample from which germline DNA can be extracted. This has been the case since Protocol version 15.0 and replaces the blood spot collection method used in previous versions of the protocol. Preliminary data has shown saliva to be a feasible method to collect sufficient DNA to conduct analyses of germline (inherited) genetic changes.

All patients who consent to part A (donation of saliva) on the Additional Research Consent Form version 1.0 onwards are eligible for this sub-study. Saliva samples should be provided after randomisation and all consenting patients from all arms can participate. See the **Sample collection and handling manual** for further details.

Patients who previously joined the trial prior to Protocol version 13.0 and who consented to provide a blood spot (Consent Form version 4.0 part K) can also be retrospectively approached to provide a saliva sample. These should be collected from patients randomised to the trial from **Nov-2011 onwards** (when recruitment to the abiraterone comparison was activated) who have received the REC approved letter explaining the need for additional saliva sample collection as the DNA extraction using the blood spot method did not work as well as anticipated.

4.6.2 Circulating Tumour-DNA Analysis (Sequential Blood Samples)

This sub-study is being conducted in collaboration with Dr Gerhardt Attard, Institute of Cancer Research, London. The aims of this analysis include to identify molecular subgroups with differential treatment effects and, through sequential sampling, identify molecular changes associated with disease progression to explore resistance mechanisms and early detection of treatment failure.

From Protocol version 14.0 (activated from Jan-2014 onwards), sequential blood samples were collected from patients within the “enzaluatmide and abiraterone” comparison, i.e. allocated to arm A or J between 29-Jul-2014 and 31-Mar-2016. From Protocol version 16 onwards, **all patients** joining the trial will be asked to donate sequential blood samples from which genetic material shed by the tumour cells can be extracted, enabling tumour DNA analysis.

The sampling schedule is different for M0 and M1 patients and is detailed in the **Sample collection and handling manual**. Sequential samples are required in order to detect genetic changes within tumours over time. The most important sampling timepoint is at progression, as it is hoped this can inform the potential mechanisms of treatment resistance. From Protocol version 16.0 the sampling schedule has been updated and now includes a baseline sample, obtained as soon as possible after consent is provided. The aim of this additional sampling point is to explore the genetic changes initially present i.e. at the point at which treatment is first started.

From Protocol version 16.0 onwards (activated Sept-2016), all patients allocated to all arms should be asked if they are willing to provide additional consent to participate in this sub-study which is recorded on part B of the Additional Research Consent Form.

4.6.3 Tissue Sample Analysis (FFPE Blocks)

As the clinical outcome data matures for several of the treatments comparisons evaluated within STAMPEDE, correlative analysis of the archival formalin-fixed paraffin-embedded (FFPE) tumour tissue will be undertaken, aiming to identify predictive and prognostic biomarkers. Targeted next-generation sequencing (tNGS) of FFPE tumour samples from selected, consenting STAMPEDE patients will be performed in order to explore the prevalance of genomic abberations and examine the predictive and prognostic effect of molecular sub-groups. FFPE blocks are currently being collected at selected STAMPEDE sites to support different projects.

All patients joining the trial have been asked to consent for the use of remaining tissue samples e.g. those obtained at prostate biopsy or following surgery, for use in additional research. These samples are usually stored as FFPE tissue blocks at the hospital where the procedure was performed. Randomising sites will be asked to assist in the retrieval of tissue samples stored in pathology stores or referring hospitals when these are required for additional translational sub-studies.

All patients who consent to part C on the additional research consent form are eligible for ongoing sub-studies involving FFPE tumour block analysis. For patients who previously joined the trial prior to 2016, consent for use of remaining samples was provided on the main consent form. Research teams at randomising sites will be required to provide an anonymised copy of the consent form when requesting samples from pathology departments and facilitate the transfer of samples to the trial designated laboratories. Further details on the sample processing and transfer can be found in the **Sample collection and handling manual.**

4.6.4 Biomarker-Screening Pilot

From Protocol version 16.0 onwards (activated Sept-2016), a Biomarker-Screening Pilot will be activated in selected sites.Patients participating in biomarker-screening will be required to register prior to randomisation, see **Section 4.3** for details on the eligibility criteria for registration and see **Section 5** for details on registration and randomisation.

At the point of registration three samples are required to complete the biomarker-screening.

- Expedited retrieval of FFPE tumour block
- Baseline blood sample collected using cell free DNA Streck™ tubes
- Saliva sample

Please refer to the **Biomarker-screening manual** for further details

The aim of this pilot is to assess the feasibility of rapid pre-randomisation biomarker-screening which will be required for planned future biomarker-selected comparisons. The first biomarker-selected comparison has successsfully received independent peer-review through CRUK and is in the late stages of development.

When recruitment is activated to biomarker-selected comparisons, the results of biomarker-screening will be required prior to randomisation and will determine eligibility. The screening pilot will activate before randomisation to biomarker-selected comparisons and inform this process. Therefore patients who participate in the pilot will continue to be allocated to any of the current open arms for which they are eligible (arm A, L and K).

4.6.5 Informed Consent For Genetic Screening

For patients joining the trial from Protocol version 16.0 onwards (activated Sept-2016), the consent process has been updated. The trial participants are asked to provide explict informed consent if they wish to receive feedback of any results that arise from research analyses of genetic material extracted from any of the biological samples collected as part of the trial e.g. saliva, FFPE tumour blocks or circulating tumour DNA extracted from blood.

Only results which are of established clinical relevance and for which testing would be available under standard NHS genetic testing guidelines will be fed back e.g. pathogenic BRCA1/2 mutations. Any genetic analysis undertaken as part of additional research associated with STAMPEDE does not replace clinically indicated investigations as only a proportion of STAMPEDE patient will undergo prospective testing and therefore it cannot be guaranteed that results will be fed back in a timely fashion.

STAMPEDE investigators are recommended to refer all patients in whom a clinically relevant genetic result is detected during research analyses to a clinical geneticist. This is to facilitate access to genetic couselling and the required confirmatory testing, this is also necessary in order to offer appropriate advice to biological relatives in the event of detecting a germline (inherited) genetic abnormality. The list of clinically relevant gene mutations to be fed back will be based on current clinical guidelines. The STAMPEDE Biological Research Group will review this periodically to ensure it remains current and oversee this process.

In the information provided to STAMPEDE patients who joined the trial prior to Protocol version 16.0, it was stated that any subsequent genetic results would not be linked to them or their families and therefore results will not be provided in this instance. Going forward, the changes incorporated in Protocol version 16.0 will enable patients who may undergo analyses on genetic material extracted from FFPE tissue, saliva or circulating tumour DNA (extracted from blood) to opt to receive clinically relevant information.

5 REGISTRATION AND RANDOMISATION

5.1 TRIAL ENROLMENT: DEFINITIONS AND PROCESS

5.1.1 Registration

Currently, registration is only required for patients participating in the Biomarker-Screening Pilot. This is initially being activated in a proportion of STAMPEDE sites.

For sites participating in the Biomarker-Screening Pilot, all patients who are eligible to participate in the Biomarker-Screening Pilot must be registered prior to randomisation. Once registered to participate in the pilot, patients can currently be randomised without waiting for the results of the biomarker-screening providing all the required screening information is known.

See **Section 4.3.1** for details on selection criteria, please confirm all criteria are met and complete the Registration CRF prior to contacting the STAMPEDE trial team at CTU.

All participants in the Biomarker-Screening Pilot will be allocated a registration number which relates specifically to the biomarker-screening process. The registration number will be used to identify the patient until the point of randomisation when this will be linked and replaced by the trial number.

Once recruitment is activated to biomarker-selected comparisons, biomarker-screening will be implemented. Registration will be then required for all patients participating in biomarker-screening and patients will only be eligible for randomisation once the results of biomarker-screening are known.

5.1.2 Randomisation

All other patients **not** participating in the Biomarker-Screening Pilot may proceed immediately to randomisation. Eligibility will be confirmed during the randomisation process and patients will be allocated to any of the open research arms for which they are suitable (see **Section 4.4**).

To enter a patient into STAMPEDE (either to register or randomise), the relevant forms should be completed carefully, and CTU contacted by phone:

REGISTRATION & RANDOMISATIONS
Call MRC CTU at UCL, Monday to Friday 0900-1700
Excluding public holidays or dates when notice has been given by CTU.
Tel: +44 (0) 20 7670 4777

A trial number and treatment will be allocated and given over the phone or by return fax. In addition, a letter confirming these details will be sent. The trial number will be the primary way in which the patient will be identified and should be used in all correspondence. Centres should send a letter to the patient's GP to inform them of their trial participation and treatment allocation. The GP letter is supplied as a template and can be downloaded from the trial website www.stampedetrial.org.

5.2 CO-ENROLMENT GUIDELINES

Ideally, patients should not be participating in any other clinical trial of prostate cancer treatment when they enter STAMPEDE and should not enter any other trials until a failure-free survival (FFS) event has been experienced and reported. After this point, the patient may be entered into further, second-line treatment studies. The primary outcome measure of STAMPEDE is overall survival and follow up reports must continue after co-enrolment. Participation in post-progression studies must be reported to CTU on the Co-enrolment CRF; details of any interventional treatments received for progression in such studies should be reported on the Additional Treatment Log.

Data sharing agreements with “down-stream” trials are encouraged to improve data quality in both trials and to reduce costs to both organisations.

6 TREATMENT OF PATIENTS

6.1 STANDARD-OF-CARE (SOC)

The standard-of-care for this patient group is **androgen deprivation therapy** (ADT) as per local practice (see **Section 6.1.1**). For some patient groups, this should now be supplemented with standard radiotherapy (see **Section 6.1.2**). From Protocol version 14.0 onwards the standard-of-care includes permitted use of docetaxel for all suitable patients (see **Section 6.1.3**).

In summary, SOC treatment is defined as being **one** of the following combinations:

- ADT alone
- ADT + Radiotherapy
- ADT + Docetaxel
- ADT + Radiotherapy + Docetaxel

6.1.1 Hormone Therapy

Patients will be randomised either to the control arm (Arm A) or to one of the actively recruiting research arms for which the patient is eligible.

With the exception of those allocated to transdermal oestradiol (Arm L), all patients will receive ADT as per local practice to achieve castrate levels of testosterone. Please see **Section 4.5.4** for more information on ADT timing before randomisation.

Patients allocated to Arm L will go on to receive transdermal oestradiol in place of standard ADT methods.

The method of planned or current long-term standard-of-care ADT must be specified for each patient prior to randomisation.

The permitted methods of ADT are:

6.1.1.A Bilateral Orchidectomy

Operations should be performed by appropriately trained surgeons. A total or sub-capsular orchidectomy may be performed. Patients having a bilateral orchidectomy are required to adhere to the same timelines as specified in **Section 4.5.4** unless there is a strong clinical reason not to do so.

6.1.1.B LHRH Agonists

LHRH agonists used according to local practice. The prophylactic use of anti-androgens to prevent tumour “flare” is recommended.

6.1.1.C LHRH Antagonists

LHRH antagonists used according to local practice. The use of prophylactic use of anti-androgens to prevent tumour “flare” is not necessary.

6.1.1.D Dual Androgen Blockade

Long-term use of anti-androgens alongside LHRH agonists, according to local practice. Note this was previously referred to as maximum androgen blockade.

6.1.1.E Others
Other methods of ADT should be discussed with the STAMPEDE trial team. The planned duration of ADT should be **at least 2 years**.

6.1.2 Standard-Of-Care (M0) RT

6.1.2.A N0M0 Patients
Investigators should give standard radiotherapy (RT) to patients with node negative, non-metastatic disease (N0M0), in accordance with data from the PR07 and SPCG trials. If there is an intention to omit radiotherapy (e.g. RT is contraindicated for the patient) in patients with N0M0 disease this must be discussed with the STAMPEDE trial team before randomisation to confirm eligibility. See Section 6.7 for further details of radiotherapy administration.

6.1.2.B N+M0 Patients
The benefit of radiotherapy in this group is at present uncertain with no firm data to either support or refute its use. However, the PR07 trial included some node-positive patients as cross sectional imaging was not a part of the baseline assessment in this trial, which did include whole pelvis radiotherapy (11). For patients with node-positive, non-metastatic disease, radiotherapy is therefore recommended in suitable cases (12).

6.1.2.C Planned use of SOC RT
Investigators will be asked to state their intention with regards to planned radiotherapy in this group at randomisation. Intention to give radiotherapy (or not) for **all** patients must be stated at randomisation to ensure that there is no bias towards particular combinations of systemic therapy with radiotherapy.

Standard-of-care radiotherapy is not a core part of the trial, therefore we intend to collect minimal data about the radiotherapy administered. It is accepted that some patients will develop progressive disease before radiotherapy can be administered and if this occurs the reasons for non-delivery of treatment must be recorded on the Radiotherapy Detail CRF.

Suitability for radiotherapy is assessed by the treating clinicians. Any patient who has had a previous, definite diagnosis of inflammatory bowel disease is at increased risk of disease re-activation following radiotherapy, and the risks of this must be balanced against the potential benefits of radiotherapy on an individual basis.

6.1.3 Standard-Of-Care Docetaxel
Investigators are strongly encouraged to consider giving docetaxel as part of the standard-of-care for patients with newly-diagnosed metastatic disease, based on the survival benefit demonstrated by both STAMPEDE in the primary analysis of the "original comparisons" and CHAARTED (13) (14, 15). Investigators may also consider giving docetaxel to patients with high-risk locally-advanced disease, given both the significant improvement in failure-free-survival and consistency of effect for prostate cancer-specific survival shown by STAMPEDE.

The treating clinician and patient must have decided, prior to randomisation, if docetaxel is to be given. Chemotherapy treatment may have started when the patient is randomised. For patients allocated to receive transdermal oestradiol (Arm L) who have not already started docetaxel prior to randomisation, it is recommended that docetaxel commences around 4 weeks after starting research treatment (see Section 6.2.8). As with standard radiotherapy, minimum data collection will be required, however the start and end dates of docetaxel treatment are needed to ensure the appropriate timelines are met (see Section 4.5.6). A SOC Docetaxel Treatment CRF should be

completed for all patients confirming whether docetaxel was given or not, regardless of being planned.

Docetaxel is given according to local protocols as a standard non-trial treatment. The regime used previously within STAMPEDE was 75mg/m^2 Day 1 as 1hr IV infusion, plus prednisolone 5mg BID for 21 days repeated every 3 weeks for a maximum of 6 cycles. The STAMPEDE TMG would suggest prednisolone could be omitted and data on the use of co-prescribed steroid will be collected on the SOC Docetaxel Treatment CRF (please see Table 19 for more details).

6.2 RESEARCH TREATMENTS

6.2.1 Required Timelines When Starting Research Treatment

Allocated treatment should start promptly after randomisation. Please refer to Section 4.5.7 for more information on starting of research treatment.

6.2.2 Research Abiraterone + Prednisolone (relevant to arms G & J)

Note: recruitment has now closed to all research comparisons containing abiraterone; that is Arm G (SOC + abiraterone) and Arm J (SOC + enzalutamide + abiraterone).

Please note that for some patients treatment with abiraterone may continue until all categories of disease progression or up to a maximum duration of 2 years.

Arm G (SOC + abiraterone) patients who have now reached their maximum duration of 2 years on trial treatment include:

- All N0M0 patients
- All N+M0 patients receiving radical radiotherapy

Arm J (SOC + enzalutamide + abiraterone) patients who have now reached their maximum duration of 2 years on trial treatment include:

- N0M0 patients starting treatment over 2 years ago
- N+M0 patients receiving radical radiotherapy and starting treatment over 2 years ago

All such patients should have reported permanent stopping of research abiraterone on an End of Research Treatment CRF.

Please see sections below for more information.

Abiraterone will be administered as a single 1000mg daily oral dose (4 tablets to be taken together once a day) together with prednisolone or prednisone 5mg daily to prevent secondary mineralocorticoid excess. Abiraterone absorption is increased by food. The tablets should be taken at least 2 hours after food, swallowed whole with some water. No food should be eaten for 1 hour afterwards.

Prednisolone (prednisone in Switzerland) should be taken as a single dose with food in the morning.

Trial treatment must stop if other systemic treatments are initiated at any time for disease progression control (including chemotherapy, radium-223 etc).

Anti-androgens (e.g.. bicalutamide) should not be given in combination with abiraterone due to the risk of toxicity; as such patients on, or planned for dual androgen blockade (DAB), at randomisation should not continue with their anti-androgen use if allocated to receive abiraterone, additionally anti-androgens started whilst on abiraterone treatment should trigger abiraterone to be stopped. In patients with **M1 disease**, treatment with abiraterone will continue from randomisation until all categories of disease progression have occurred, consistent with the COU-AA-301 and COU-AA-302 trials (48, 49) i.e., abiraterone would be given for these patients until a composite of:

- PSA progression (as defined in **Section 7.1.3.A**)
- Radiological progression (appearance of new lesions or progression of existing lesions) **and**
- Clinical progression (defined as new cancer-related symptoms)

It is accepted that these flexible criteria for stopping treatment with abiraterone are open to the investigator's interpretation and discretion. Patients might continue treatment beyond the first failure-free survival (FFS) event; the first FFS event must be reported as per the other arms; all categories of disease progression (PSA, radiological and clinical) need to be reported once.

In patients **with N0M0 disease or N+M0 disease undergoing radical radiotherapy**, treatment would continue until the earliest of 2 years or all categories of disease progression as defined for M1 patients. ADT can be discontinued in this group at 2 years at the discretion of the local investigator (see **Section 6.1.1**).

For patients with **N+M0 disease not planned for radical radiotherapy**, or who do not receive planned prostate RT, treatment will continue as for patients with M1 disease until all categories of disease progression.

If a patient allocated to receive abiraterone develops only biochemical failure, the responsible clinician might switch from abiraterone + prednisolone 5mg od to abiraterone and dexamethasone 0.5mg od.

See **Section 7.1.3** for further information on the trial definition of progression.

6.2.3 Abiraterone + Prednisolone: Administration And Management Of Toxicities

Abiraterone absorption is increased by food therefore should be taken on an empty stomach without food. The tablets should be taken at least 2 hours after food, swallowed whole with some water. No food should be eaten for 1 hour afterwards.

Prednisolone (prednisone in Switzerland) should be taken as a single dose with food in the morning.

6.2.3.A Abiraterone Contraindications

- Unusual or allergic reaction to past abiraterone acetate treatment
- Uncontrolled hypertension
- Uncontrolled heart failure
- Abnormal liver function or active or chronic liver disease

See **Table 16** for details on drugs that may interact with abiraterone.

6.2.3.B Abiraterone Special Warnings And Required Monitoring Whilst on Treatment

:: Hypokalaemia

Abiraterone may cause hypokalaemia due to secondary mineralocorticoid excess, this can be counteracted by co-prescription of prednisolone. Regular monitoring of serum potassium levels are required whilst receiving treatment with abiraterone. The Investigator Brochure states that monitoring should be performed 2-weekly for the first 12 weeks and then every month or as per protocol whilst receiving abiraterone (50).

When abiraterone is used routinely in the licenced setting (CRPC), it is common practice to prescribe the next course of treatment for 8 weeks to patients who have been on abiraterone for over 12 months with no abnormalities, having checked that the potassium is normal (or >3mmol/L and in line with previous results) prior to writing the prescription.

The STAMPEDE protocol requires continued monthly monitoring for patients who experience hypokalaemia related to research abiraterone. For patients who have been monitored appropriately with no evidence of hypokalaemia, the frequency of monitoring may be reviewed after 12 months on treatment and, at the discretion of the investigator, may be reduced to every 2 months if judged appropriate. This is consistent with the approach adopted in the LATITUDE trial in which abiraterone was evaluated in high-risk metastatic hormone-naïve prostate cancer (51). Treatment should always be interrupted presence of symptoms (constipation, palpitations, fatigue, muscle weakness or spasm, tingling or numbness), see **Table 8.**

:: Hepatic Impairment

Abiraterone treatment can be associated with increased liver enzymes and hepatotoxicity therefore regular monitoring of liver function tests (LFTs) is required whilst on treatment. LFTs (ALT or AST and bilirubin). The Investigator Brochure states that monitoring should be performed 2-weekly for the first 12 weeks and then every month or as per protocol (50) whilst receiving abiraterone.

When abiraterone is used routinely, it is common practice to prescribe the next course of treatment for 8 weeks to patients who have been on abiraterone for over 12 months with no abnormalities, having checked that the liver function tests are normal, or no worse than grade 1 prior to writing the prescription (51). This is acceptable provided those with grade 1 abnormalities are monitored more frequently and treatment is interrupted if they increase to grade 2, see **Table 9.** The STAMEPDE protocol requires monthly monitoring in the first 12-months on treatment with research abiraterone. For patients who have been monitored appropriately with no evidence of liver function abnormality, the frequency of monitoring may be reviewed after 12 months on treatment and, at the discretion of the investigator, reduced to every 2 months if judged appropriate, consistent with the approach adopted in the LATITUDE trial (51).

If clinical symptoms or signs suggestive of hepatotoxicity develop, serum transaminases, in particular serum alanine aminotransferase (ALT), should be measured immediately. See **Table 9** for the management of abiraterone induced hepatotoxicity.

:: Blood pressure management

Abiraterone may cause hypertension. Regular monitoring of blood pressure is required whilst receiving treatment. The Investigator Brochure states that monitoring should be performed 2-weekly for the first 12 weeks and then every month whilst receiving abiraterone. STAMPEDE Investigators will be required to ensure monthly blood pressure monitoring is performed and reviewed in the first 12-months on treatment, it is acceptable for this to be documented self-monitoring or via the GP providing this is reviewed at each follow-up. After 12 months on treatment, it is acceptable for blood pressure monitoring to be performed every 2-months and reviewed at each

follow-up visit, providing blood pressure has been well controlled. For the management of abiraterone induced hypertension see **Table 7.**

:: Cardiovascular history
Abiraterone acetate should be used with caution in patients with a history of cardiovascular disease. The safety of abiraterone acetate in patients with left ventricular ejection fraction <50% or New York Heart Association (NYHA) Class III or IV heart failure has not been established. Before treatment with abiraterone acetate, hypertension must be controlled and hypokalaemia must be corrected.

Caution is required in treating patients whose underlying medical conditions might be compromised by increases in blood pressure, hypokalaemia, or fluid retention, e.g. those with heart failure, recent myocardial infarction, or ventricular arrhythmia.

:: Renal Impairment
No dose adjustments are required in renal impairment; however caution is advised if patients develop severe renal impairment as there is limited clinical data in this population. Systemic exposure to abiraterone after a single oral 1000mg dose did not increase in patients with end-stage renal disease on dialysis.

6.2.3.C Abiraterone Undesirable Effects
The most common adverse drug reactions observed in the integrated safety data for those patients who received 1000mg abiraterone acetate plus prednisone or prednisolone in clinical studies (n=1,070) were fatigue, arthralgia, peripheral oedema, back pain, bone pain, nausea, constipation, hypokalemia and anaemia.

The adverse events graded as 3 or 4 and which occurred in more than 5% of patients were fatigue, peripheral oedema, anaemia and back pain see **Appendix C**.

6.2.3.D Abiraterone Overdose
Human experience of overdose with abiraterone is limited. There is no specific antidote to abiraterone acetate. In the event of an overdose, administration of abiraterone acetate should be stopped and general supportive measures undertaken, including monitoring for cardiac arrhythmias, liver function and electrolytes.

6.2.3.E Management Of Specific Toxicities From Prednisolone
The co-administration of prednisolone/prednisone 5mg once daily is required whilst receiving abiraterone to prevent secondary mineralocorticoid excess and 5 mg once daily is used in this trial.

Prednisolone/prednisone dose increase of up to 5mg BID is recommended to manage mineralocorticoid-related toxicities (e.g., hypokalaemia, hypertension, peripheral oedema) see **Table 7, Table 8** and **Table 10**.

Patients experiencing serious symptoms of Cushing's syndrome (e.g., weight gain, muscle loss) can decrease or discontinue (temporarily or permanently) steroids at the investigator's discretion but should be closely monitored for symptoms of secondary mineralocorticoid excess. It should be noted that weight gain and muscle loss are also associated with ADT.

Table 7: Management of hypertension associated with abiraterone (given alone or in combination with enzalutamide)

TOXICITY EVENT	ACTION
Grade 1-2	Management as per investigator with anti-hypertensive treatment and increase frequency of blood pressure monitoring to at least weekly. Follow local guidance for selection of anti-hypertensives but avoid thiazide diuretics to minimise risk of serum potassium derrangment. Calcium channel antagonists or beta blockers are often preferred.<br>As with other symptoms of minerlocorticoid excess, consider increasing prednisolone dose to 5mg BID.
Grade 3-4	Withhold abiraterone and enzalutamide. Adjust or add anti-hypertensive medications to mitigate the toxicity. When hypertension resolves to Grade ≤1, resume both enzalutamide and abiraterone at full dose with prednisolone 5mg bid.

A cardiologist's opinion should be considered if blood pressure control is not achieved within 4 weeks.

Table 8: Management of hypokalaemia associated with abiraterone (given alone or in combination with enzalutamide)

TOXICITY EVENT	ACTION
Grade 1<br>(LLN- 3.0mmol/L)	Supplement with oral potassium and monitor closely and increase prednisolone dose to 5mg BID.<br>Exclude and manage other causes of hypokalemia.
Grade 2<br>(<LLN – 3.0mmol/L and symptomatic)	**Pause abiraterone.**<br>Supplement with oral potassium and monitor closely and increase prednisolone dose to 5mg BID.<br>Exclude and manage other causes of hypokalemia.<br>Re-start abiraterone with close monitoring, discontinue if recurs.
Grade 3<br>(<3.0–2.5mmol/L)<br><br>or Grade 4 (<2.5mM) life-threatening	Abiraterone will be **permenently discontinued** and the patients will be hospitalized for intravenous potassium replacement and cardiac monitoring. After the return of serum potassium to normal, prednisolone will be discontinued. The patient can continue on enzaluatamide alone. If hypokalaemia persists consider a dose reduction of enzalutamide to 120mg once a day.

Table 9: Management of Abnormal Liver Function Tests (LFTs) associated with abiraterone (given alone or in combination with enzalutamide)

TOXICITY EVENT	ACTION
**Grade 1** increases in AST, ALT or bilirubin (e.g. increase in AST or ALT from ULN to 2.5X ULN; increase in total bilirubin from ULN to 1.5X ULN)	The frequency of LFT monitoring should be increased to at least weekly, if the investigator judges that the laboratory abnormalities are potentially related to study medication.<br>No dose reduction is required.<br>Providing LFTs are stable for 4 weeks, resume monthly checks
**Grade 2** increases in AST, ALT or bilirubin (e.g. increase in AST or ALT to >2.5-5X ULN; increase in total bilirubin from >1.5-3X ULN)	Withhold abiraterone, enzalutamide and all other concomitant medications that are potentially hepatotoxic.<br>The frequency of LFT monitoring should be increased to at least weekly until the liver function tests return to baseline value or grade 1 when all trial medication can be re-started.<br>No dose reduction is required after one episode providing this resolved within 4 weeks but should be considered if Grade 2 derangements recurs.
**Grade 3** increases in AST, ALT or bilirubin (e.g. increase in AST or ALT to >5X ULN; increase in total bilirubin to >3X ULN),	Withhold abiraterone and enzalutamide and all other concomitant medications that are potentially hepatotoxic.<br>At least weekly monitoring is required until the LFTs return to baseline value or Grade 1.<br>Enzaluatmide can be re-started with no dose reduction. See below for abiraterone re-challange.
**Grade 4** increases in AST, ALT or bilirubin (e.g. increase in AST or ALT to >20x ULN; increase in total bilirubin to >10x ULN)	Patients must discontinue abiraterone and enzalutamide immediately.<br>At least weekly monitoring is required until the LFTs return to baseline value or grade 1 and then prednisone can be discontinued and the investigator can consider restarting enzalutamide.<br>Abiraterone should not be re-introduced.
**RE-CHALLENGE**	**ACTION**
Recurrent grade 2 derangement	Reduce to 750mg once LFTs return to grade 1
If study treatment resumption is considered for patients who have experienced Grade 3 increases in AST, ALT, or bilirubin	Resume study treatment with abiraterone dose reduction to 750mg when grade 3 toxicities resolve to grade 1 or baseline.
If Grade 3 or higher increases in AST, ALT or bilirubin recur after the first dose reduction	Hold study medication and all other concomitant medications that are potentially hepatotoxic. At least weekly LFT monitoring is required, starting immediately regardless of study schedule and continued until a return to baseline values or Grade 1.
If study treatment resumption is considered for patients who have experienced Grade 3 increases in AST, ALT, or bilirubin with the first dose reduction	Resume study treatment with abiraterone dose reduction to 500mg when AST, ALT or bilirubin returns to baseline value or grade 1.

An opinion from a hepatologist should be considered if there are any concerns or liver function derangement shows no improvement within 2 weeks of discontinuation of abiraterone.

Table 10: Management of fluid retention/oedema associated with abiraterone (given alone or in combination with enzalutamide)

TOXICITY EVENT	ACTION
Grade 1-2	Increase prednisolone dose to 5mg bid.
Grade 3-4	Withhold abiraterone.<br>Consider addition of mineralocorticoid receptor antagonist eplerenone until resolution of symptoms. Enzalutamide can be continued. When fluid retention/oedema returns to baseline or resolves to ≤Grade 1, resume abiraterone at full dose with prednisone 5mg bid, if symptoms do not resolve abiraterone should not be re-started and enzalutamide should be dose reduced to 120 mg per day.

Table 11: Management of diarrhoea (associated with abiraterone or enzalutamide)

TOXICITY EVENT	ACTION
Grade 1-2	Symptomatic management.
Grade 3-4	Withhold abiraterone.<br>If no improvement reduce dose of enzalutamide to 120 mg per day. Once resolved to Grade 1, recommence abiraterone at 750 mg per day.

6.2.4 Research Enzalutamide + Abiraterone + Prednisolone (Arm J)

Note: recruitment has now closed to Arm J (SOC + enzalutamide + abiraterone).

Please note that for some patients treatment with enzalutamide + abiraterone may continue until all categories of disease progression or up to a maximum duration of 2 years.

Arm J (SOC + enzalutamide + abiraterone) patients who have now reached their maximum duration of 2 years on trial treatment include:

- N0M0 patients starting treatment over 2 years ago
- N+M0 patients receiving radical radiotherapy and starting treatment over 2 years ago

Please see sections below for more information.

Abiraterone as described in **Section 6.2.2**.

Prednisolone/Prednisone as described in **Section 6.2.2**.

Enzalutamide will be administered as a 160mg oral dose (four capsules), taken together at the same time every day, with or without food.

Trial treatment must stop if other systemic treatments are initiated at any time for disease progression control (including chemotherapy, radium-223 etc).

Anti-androgens (i.e. bicalutamide) should not be given in combination with enzalutamide (as with abiraterone) due to the risk of toxicity; as such patients on, or planned for MAB, at randomisation should not continue with their anti-androgen use if allocated to receive enzalutamide + abiraterone, additionally anti-androgens started whilst on enzalutamide (+abiraterone) treatment should trigger

enzalutamide (+abiraterone) to be stopped. See Table 16 and Table 17 for further details on drugs that may interact with abiraterone and enzalutamide respectively.

In patients with **M1 disease**, treatment with both abiraterone and enzalutamide will continue until all categories of progression have occurred, consistent with the approach taken for abiraterone (see Section 6.2.2) i.e. abiraterone and enzalutamide will be given until a composite of:

- PSA progression (as defined in Section 7.1.3.A)
- Radiological progression (appearance of new lesions or progression of existing lesions) **and**
- Clinical progression (defined as new cancer-related symptoms).

It is accepted that these flexible criteria for stopping treatment with abiraterone and enzalutamide are open to the investigator's interpretation and discretion. Patients may continue treatment beyond the first failure-free survival (FFS) event; the first FFS event must be reported as per the other arms.

In patients with **N0M0 disease or N+M0 disease undergoing radical radiotherapy**, treatment would continue for 2 years or all categories of disease progression as defined for M1 patients, whichever is the sooner. ADT can be discontinued in this group at 2 years at the discretion of the local investigator (see Section 6.1.1).

For patients with **N+M0 disease not planned for radical radiotherapy**, or who do not receive planned prostate RT, treatment will continue as for patients with M1 disease until all categories of disease progression.

If a patient develops PSA progression only whilst on abiraterone and enzalutamide, the local investigator might consider switching from from abiraterone + prednisolone 5mg od to abiraterone and dexamethasone 0.5mg OD.

See Section 7.1.3 for further information on the definition of progression.

6.2.5 Enzalutamide: Administration, Dose Modification And Management Of Toxicities

Enzalutamide can be taken with or without food.

6.2.5.A Enzalutamide Contraindications

The full induction potential of enzalutamide may not occur until approximately 1 month after the start of treatment, when steady-state plasma concentrations of enzalutamide are reached, although some induction effects may be apparent earlier. Monitoring should continue for at least the first month of treatment and dose adjustments considered. Given the long half-life of enzalutamide (5.8 days), effects on enzymes may persist for one month or longer after stopping enzalutamide. A gradual dose reduction of the concomitant medicinal product may be necessary when stopping enzalutamide treatment. See Table 17 for further details on specific drug interactions with enzalutamide.

6.2.5.B Enzalutamide Special Warnings And Precautions For Use

:: History of seizures

Caution should be used in administering enzalutamide to patients with a history of seizures or other predisposing factors including, but not limited to, underlying brain injury, stroke, primary brain tumours or brain metastases or alcoholism. In addition, the risk of seizure may be increased in patients receiving concomitant medications that may lower the seizure threshold. Enzalutamide should be **permanently discontinued** in patients who have a seizure while on treatment.

:: Hepatic impairment

A hepatic impairment study showed that the composite AUC of enzalutamide plus N-desmethyl enzalutamide after administration of a single dose of enzalutamide was similar in patients with baseline mild, moderate or severe hepatic impairment (Child-Pugh Class A, B or C respectively) relative to patients with normal hepatic function, and no starting dose adjustment is needed.

:: Renal impairment

No dose adjustment are required in renal impairment; however caution is advised if patients develop severe renal impairment as there is limited clinical data in this population.

6.2.5.C Enzalutamide Overdose

There is no antidote for enzalutamide. In the setting of an overdose, stop treatment with enzalutamide and initiate general supportive measures taking into consideration the $t^{1/2}$ of 5.8 days. Patients may be at increased risk of seizures following an overdose.

6.2.5.D Management Of Specific Toxicities Due To Abiraterone And Enzalutamide

The safety monitoring and toxicity management plan described below takes into account AEs based on the reported clinical safety data of abiraterone and enzalutamide given separately. There are limited reported data on the safety and toxicity of the combination of enzalutamide and abiraterone however the recommendations summarised here have been updated in light of the experience gained in STAMPEDE as recommended by the STAMPEDE TMG.

:: Seizures

If any patient suffers a seizure whilst on treatment, enzalutamide should be permanently discontinued immediately. Abiraterone and prednisolone can be continued providing there are no abiraterone-specific toxicities.

Table 12: Management of arthralgia & muscle pain (associated with enzalutamide)

Toxicity event	Action
Grade 1-2	Symptomatic management
Grade 3-4	Reduce dose of enzalutamide to 120 mg /day

Table 13: Management of fatigue (associated to enzalutamide)

Toxicity event	Action
Grade 1-2	Consider a dose reduction to 120 mg/day
Grade 3-4	Pause enzalutamide for 1 week or until the toxicity grade improves to grade 2 or lower severity. Re-started at a reduced dose (120mg/day or 80mg/day) in consultation with the study team.

6.2.6 Research Metformin (Arm K)

Metformin will be given as a daily dose in addition to standard-of-care treatment. The dose is 850mg OD. If tolerated, this should be increased to the target dose of 850mg BID after 4-6 weeks i.e. at the first follow-up visit.

In the case of **M0 patients**, if ADT is stopped after a minimum of 2 years, metformin should continue for a minimum of 3 years following randomisation and for a further 12 months after the administration of the last LHRH (whichever is longer). This is to allow for the delay in testosterone levels returning to normal following stopping ADT. If ADT is not stopped, then metformin should continue as it the case for M1 patients. In the event that ADT is stopped and then re-started for relapsed disease, if ADT is restarted whilst patients remain on metformin (i.e. within 12 months of the last administration of LHRH) then metformin should continue whilst on ADT. If metformin is stopped 12 months after the last administration of LHRH it should not be re-started in the event of relapse.

For **M1 patients** metformin should continue whilst on ADT. Treatment should continue post-progression providing it is judged to be in the patients best interest. Metformin can be given together with any additional treatments started for progression, excluding other IMPs i.e. investigators may choose to stop metformin treatment post-progression in order to enable patients to participate in another clinical trial evaluating treatments for CRPC.

In the event of stopping research treatment, unless a patient states otherwise, consent is assumed for continued recording trial data.

6.2.7 Metformin: Administration, Dose Modifications And Management Of Toxicities

The starting dose for metformin is 850mg once daily. If tolerated this should be increased to the target dose of 850mg twice daily after 4-6 weeks. Metformin should be taken around the same time each day and treatment tolerance is best if taken with or after food. For twice daily dosing, the minimum time between doses should be 8 hours, doses should not be taken closer together if forgotten or missed. If metformin is well tolerated and it is desirable to make a dose modification outside of the trial follow-up schedule, it is acceptable to conduct a telephone consultation. If metformin 850mg OD is not well tolerated, consider switching to 750mg SR OD or alternatively, reduce to 500mg OD.

6.2.7.A Metformin Special Warnings And Required Monitoring Whilst on Treatment

:: Renal impairment
Metformin is not nephrotoxic, but is exclusively excreted by the kidneys. Therefore treatment should only be started in participants with stable renal function. From protocol version 16.0 the renal threshold has been revised in light of updated FDA guidance and published prescribing recommendations. Metformin should be only started when the GFR ≥45min/ml/1.73m^2. Renal function should be monitored **at least every 6 months** in participants with stable renal function, whilst on metformin. Additional monitoring is required in any patient at risk of deteriorating renal function (see Table 12). In line with published prescribing recommendations, if the GFR falls to between 30-45 min/ml/1.73m^2 a **dose reduction** is required to ensure the maximum 24hr dose is 1000mg or less and monitoring of renal function is required at least 3 monthly (52). Metformin should be **permanently stopped** if the GFR falls to ≤30ml/min/1.73m^2.

See Table 14 for situations when metformin treatment should be paused due to the risk of deterioration in renal function.

Table 14: Situations when metformin treatment should be paused

SITUATIONS	RISK FACTOR
Iodinated contrast agents	If the GFR<60 ml/min/1.73m^2 metformin should be paused for 24 hours prior to receiving contrast and re-started 48 hours post-administration.
Anaesthesia (peridural; spinal or general)	Pause metformin 48 hours prior to procedure and re-start no earlier than 48 hours following procedure, providing oral intake re-established and renal function is stable and at baseline.
Surgery	Pause metformin 48 hours prior to procedure and re-start no earlier than 48 hours following procedure, providing oral intake re-established and renal function is stable and at baseline.
Dehydration<br>e.g. nausea, vomiting or diarrhoea	Pause metformin and re-start only when oral intake is re-established and renal function is stable and at baseline.
Obstructive uropathy<br>e.g. urinary retention or ureteric obstruction	Pause metformin and re-start only when renal function confirmed to be stable and at baseline.

:: Treatment breaks
It is anticipated that metformin treatment will be paused for approximately 72 hours around the time of contrast enhanced CT scans (see Table 14) and may need to be paused during episodes of inter-current illness. If metformin is paused for 6 days or less this information does not need to be recorded and no additional action is needed. Treatment pauses of 7 days or more should be recorded by updating the Metformin Treatment Log.

If metformin treatment is paused for more than 2 weeks, investigators may consider re-starting at 850mg once daily for the first 4 weeks before escalating to full dose providing tolerance is acceptable. It is suggested that, providing patients have a sufficient supply of labelled IMP metformin tablets, a telephone consultation may be sufficient to assess tolerance and advice regarding dose modification in order to limit hospital visits.

If treatment is paused for more than 3 months or >50% of doses are missed for any reason the trial team should be informed as metformin may need to be discontinued.

6.2.7.B Management Of Specific Toxicities From Metformin

:: Gastrointestinal disturbance

Gastrointestinal disturbances are very common with metformin and include nausea, vomiting, diarrhoea, abdominal pain and loss of appetite. These are most common when first starting treatment (occur in >1/10 individuals).

If toxicities occur we recommend a dose reduction and/or a switch to a sustained release (SR) preparation if available (see **Table 15**).

Table 15: Management of metformin related gastrointestinal toxicity

TOXICITY EVENT	ACTION
Grade 1	• Ensure metformin is taken with or after food.<br>• Consider switching to 750 mg BID SR preparation if available.<br>Or, if unavailable consider:<br>• 1 week treatment pause, re-start at reduced dose 850mg once daily. Attempt an escalation after 1 month but if necessary, remain at 850mg OD.<br>And, if unable to tolerate 850mg OD or sustained release preparations are not available<br>• Consider dose reduction to 500mg OD (SR or IR if not available)
Grade 2<br>or higher	Reduce to 500mg OD SR (or IR if not available); re-attempt dose escalation after minimum of 1 week if symptoms improve, aiming to continue at the maximum tolerated dose<br>If grade 2 toxicity persists:<br>• Pause treatment for 2 weeks, and re-start at 850mg sustained release or if not available 500mg OD.<br>• And re-attempt a dose escalation 2 months later.<br>• And continue at the maximum tolerated dose providing symptoms ≤ grade 1.

If toxicities occur whilst on the initial starting dose of 850mg OD then the following dose modifications should be made:

- Switch to 750mg SR OD
- Alternatively, reduce to 500mg OD

If toxicities persist, consider a 1 week treatment pause, before re-starting at one of the dose-modified regimes and attempt an dose escalation after a minimum of 1 month.

Other possible metformin related toxicites included taste disturbance, skin reactions and B12 deficiency resulting in megaloblastic anaemia (see **Appendix C, Table 7**). Any patient who experiences anaemia whilst taking metformin should have haematinics including vitamin B12 measured and replaced if deficient.

:: Lactic acidosis

This is a very rare (3/100,000 patient years), but serious metabolic consequence. Reported cases have occurred primarily in diabetic patients with significant renal impairment who are also dehydrated. It is unclear whether this is due to the underlying diabetes or metformin. This is supported by a meta-analysis demonstrating similar rates of lactic acidosis in people with diabetes

taking metformin compared with diabetic patients not taking metformin (53). This evidence suggests this side effect may be a complication of diabetes and may not be associated with metformin treatment. The risk factors for lactic acidosis are: renal impairment, prolonged fasting or malnutrition, excessive alcohol intake, hepatic insufficiency or any condition associated with hypoxia e.g. cardiac or respiratory failure or circulatory shock due to any cause.

The risk of lactic acidosis should be considered in the event of non-specific symptoms such as muscle cramps, abdominal pain and/or severe weakness or lethargy. Any patient with a suspected metabolic acidosis requires immediate discontinuation of metformin and evaluation. Lactic acidosis is characterised by metabolic acidosis (decreased blood pH, high lactate above 5mmol/L and an increased anion gap and lactate/pyruvate ratio). The most effective way to remove lactate and metformin is haemodialysis.

:: Metformin overdose

Hypoglycaemia has not been reported with metformin doses of up to 85g although lactic acidosis has occurred in such circumstances. Patients should be urgently assessed in the event of an overdose and hospital admission considered. The management of metformin overdoses should be as per standard clinical care by the local team. The most effective way to remove lactate and metformin is haemodialysis.

6.2.8 Research Transdermal Oestradiol (Arm L)

Patients randomised to receive transdermal oestradiol may also receive SOC radiotherapy (**Section 6.1.2**) and SOC docetaxel (**Section 6.1.3**) as clinically appropriate, as has been done in the PATCH trial. It is recommended that patients commence SOC docetaxel (where planned) after they have been on transdermal oestradiol for around 4 weeks when most will have completed the induction period; radiotherapy, if used, would follow later.

Transdermal oestradiol is delivered as Progynova TS 100mcg/24 hours transcutaneous oestradiol patches according to the following dose regimen which has been shown within the PATCH trial to be sufficient for achieving castrate levels of testosterone.

6.2.8.A Induction Regimen

Four Progynova TS 100 transdermal oestradiol patches to be changed twice weekly (e.g. Monday and Thursday) for four weeks. A confirmatory testosterone and oestradiol sample should be taken at 4 weeks with the sample drawn the **day before** the patches are changed.

6.2.8.B Maintenance Regimen

If the patient has achieved a testosterone value of ≤1.7nmol/L at 4 weeks then treatment is changed to a **maintenance regimen** of **three** patches changed twice weekly. The oestradiol level should also be monitored at the 4 week time point, with castrate levels of testosterone typically achieved with a plasma oestradiol level ≥500pmol/L.

If a patient's testosterone is >1.7nmol/L at four weeks then they should remain on the induction regimen for another 4 week period, with monitoring of testosterone and oestradiol samples taken at around the week 8 time point, the day before patches are changed. Once the patient achieves a castrate level of testosterone≤1.7nmol/L, they can be reduced to the maintenance regimen.

6.2.8.C Monitoring Hormone Levels

Oestradiol and testosterone levels should continue to be monitored throughout follow-up, while the patient remains on transdermal oestradiol treatment, to assess for evidence of compliance and to also ensure the patient is on the appropriate dose. See **Section 7.1.2** and **Table 1** for when these

values are required, noting also that the samples can be taken at the same time as scheduled PSA measurements.

A repeat blood test should be carried out within 4 weeks if, at any time, the patient's oestradiol level is found to be <300pmol/L or >2000pmol/L, with particular attention paid to the day that the patches are changed compared to when the blood sample is drawn (should be the day before changing patches). If the patient continues to have out of range oestradiol levels, and/or persistent testosterone >1.7nmol/L, then a member of the CTU team should be contacted for advice.

If the maintenance patch dose is changed at any time (for example, reducing from 3 to 2 patches changed twice weekly), then additional oestradiol and testosterone tests are required around 4 weeks after dose modification.

6.2.9 Transdermal Oestradiol: Administration, Dose Modifications And Management Of Toxicities

Consecutive patches should be applied to different sites. It is recommended that patches are placed on dry, intact and hairless skin and on areas where little wrinkling occurs, at the following sites only:

- Shoulder girdle
- Back
- Upper arms
- Buttocks

Patches should not be placed on or near the breast area, or on areas of the body where there are large amounts of subcutaneous fat, particularly around the abdomen, as this could affect absorption. Please note that these recommendations are mainly based on studies in women using the patches.

To apply the patch, remove the protective liner and press on to the skin immediately, holding for at least 30 seconds to ensure proper adhesion. If necessary, tape can be used to fix the patch in place. If applied correctly, the patient can bath or shower as normal, however the patches might come off in very hot water or in a sauna.

Dermatitis can be a common side-effect of using the patches, especially in the induction period, which can usually be controlled by alternating the site of patch application. Patients should be advised that if patches become dislodged they should not put on extra patches, but apply their next set of patches when they are next due to be applied.

We expect patients to remain on the prescribed dose, and any potential dose modifications other than those indicated in Section 6.2.8 should be discussed with the CTU team.

If a patient has a cardiovascular event (see Section 7.1.4.B), discontinuation of treatment with transdermal oestradiol may be considered at the discretion of the treating clinician.

6.3 CONCOMITANT MEDICATIONS AND DRUG INTERACTIONS

6.3.1 Abiraterone: Interaction With Medicinal Products And Other Forms Of Interaction

Details on drug interactions are described in **Appendix C** and **Table 16** provides a summary on the main interactions.

:: Anti-androgens
Abiraterone is steroid synthesis inhibitor and should **not** be given together with any other anti-androgens given the risk of toxicity. Cyproterone acetate should be discontinued 10 days and finasteride stopped 48 hours before commencing abiraterone. Concomitant use of dutasteride, bicalutamide, flutamide and tamoxifen are all **contraindicated.**

6.3.2 Enzalutamide: Interaction With Medicinal Products And Other Forms Of Interaction

Details on drug interactions are described in **Appendix C** and **Table 17** provides a summary on the main interactions.

:: Anti-androgens
Enzalutamide is potent androgen receptor antagonist and should **not** be given together with any other anti-androgens given the risk of toxicity.

Cyproterone acetate should be discontinued 10 days and finasteride stopped 48 hours before commencing enzalutamide. Concommitent use of dutasteride, bicalutamide, flutamide and tamoxifen are all **contraindicated.**

Table 16: Drugs which may interact with abiraterone

DRUGS WHICH MAY INCREASE ABIRATERONE LEVELS			
**Substrate**	**Clinical Use**	**Drug**	**Recommendation**
CYP3A4 inhibitors	Macrolide antibiotics	Clarithromycin	Avoid or hold abiraterone if short term use unavoidable given increased risk of abiraterone toxicity
	Anti-fungals	Ketoconazole<br>Itraconazole<br>Voriconazole	Avoid or hold abiraterone if short term use unavoidable given increased risk of abiraterone toxicity
**DRUGS WHICH MAY REDUCE ABIRATERONE LEVELS**			
**Substrate**	**Clinical Use**	**Drug**	**Recommendation**
CYP3A4	Anti-epileptics*	Phenytoin<br>Carbamazepine<br>Phenobarbital<br>Primadone	Contraindicated
	Anti-depressants	St Johns Wart	Contraindicated
	Anti-TB	Rifampicin<br>Rifabutin	Contraindicated
	Anti-retroviral	Atazanavir<br>Saquinavir<br>Ritonavir<br>Indinavir<br>Nelfanavir	Contraindicated. Seek specialist advice and discuss with trial team
**DRUGS WHICH MAY ACCUMULATE WHEN GIVEN WITH ABIRATERONE**			
**Substrate**	**Clinical Use**	**Drug**	**Recommendation**
CYP2D6	Cardiac	Metoprolol<br>Propranolol<br>Propafenone<br>Flecanide	Monitoring required as drug levels may increase with abiraterone use
	Anti-depressants	Desipramine<br>Venlafaxine<br>Citalopram	Monitoring required as drug levels may increase with abiraterone use
	Anti-psychotics	Haloperidol<br>Risperidone	Monitoring required as drug levels may increase with abiraterone use
	Analgesia	Tramadol<br>Codeine<br>Oxycodone	Monitoring required as drug levels may increase with abiraterone use
	Alpha blockers	Tamsulosin	Monitoring required as drug levels may increase with abiraterone use
	Anti-diabetic	Repaglinide	Monitoring required as drug levels may increase with abiraterone use

Table 17: Drugs which may interact with enzalutamide

DRUGS WHICH MAY INCREASE ENZALUTAMIDE LEVELS			
**Substrate**	**Clinical Use**	**Drug**	**Recommendation**
CYP2C8 inhibitors	Lipid-lowering	Gemfibrozil	Avoid, if no alternatives, reduce enzalutamide dose to 80mg
**DRUGS WHICH MAY DECREASE ENZALUTAMIDE LEVELS**			
**Substrate**	**Clinical Use**	**Drug**	**Recommendation**
CYP2C8 inducers	Anti-TB	Rifampicin<br>Rifabutin	Avoid and switch to an alternative if possible
CYP3A4 inducers	Anti-epileptics	Phenytoin<br>Carbamazepine<br>Phenobarbital	Contraindicated
	Anti-depressant	St Johns Wart	Contraindicated
	Anti-retrovirals	Atazanavir<br>Saquinavir<br>Ritonavir<br>Indinavir<br>Nelfanavir	Contraindicated. Seek specialist advice and discuss with trial team
**ENZALUTAMIDE MAY REDUCE DRUG LEVELS**			
**Substrate**	**Clinical Use**	**Drug**	**Recommendation**
CYP2C19	Gastric protection	Omeprazole	Omeprazole AUC reduced by 70%<br>Consider increasing dose of omeprazole for same therapeutic effect
CYP3A4	Analgesia	Fentanyl*<br>Alfentanil*<br>Tramadol	Monitor closely and consider alteratives
	Immunosuppressants	Sirolimus*<br>Tacrolimus*<br>Cyclosporine*	Monitor closely
	Anti-migraine	Ergotamine	Monitor closely
	Cardiac	Nifedipine<br>Ivabradine	Monitor closely, consider alternatives as clinical effect may be reduced
CYP2C9	Anti-epileptics	Phenytoin*	Contraindicated
	Anti-coagulants	Warfarin*	Warfarin AUC reduced by 56%<br>Consider switching to low molecular heparin, increase INR monitoring if this is not possible
**DRUGS WHICH MAY ACCUMULATE WHEN GIVEN WITH ENZALUTAMIDE**			
**Substrate**	**Clinical Use**	**Drug**	**Recommendation**
p-gp		Colchicine*<br>Dabigatran*<br>Digoxin*	Monitor closely

*narrow therapeutic index

6.3.3 Metformin: Interaction With Medicinal Products And Other Forms Of Interaction

Metformin does not interact with any of the other treatments for prostate cancer and can be continued during all further treatments started on progression.

Caution is needed however when initiating potential nephrotoxic drugs as metformin is renal excreted therefore may accumulate if renal function deteriorates, see Table 18 for details.

As metformin is being given as an IMP in the context of a clinical trial, continued use will not be permitted if patients participate in other interventional clinical trials for prostate cancer (i.e. CRPC setting). Investigators should use their discretion and discuss discontinuing metformin with the trial team if it is felt to be in the patient's best interest.

Table 18: Drugs which require additional monitoring of renal function

Clinical use	Drug	Recommendation
Anti-hypertensives and other cardiac disease	ACE inhibitors/angiotension II receptor blockers e.g. ramipril, lisinopril, Irbesartan	Monitor renal function until confirmed to be stable and providing GFR remains >45ml/min/m^2 . Repeat test if necessary
	Diuretics e.g Frusemide, budesonide	
Antibiotics	Aminoglycoside antibiotics e.g Gentamicin or amikacin	Hold metformin during treatment and re-start providing renal function confirmed to be stable and GFR remains >45ml/min/m^2
Analgesia	NSAIDS e.g. Ibuprofen, diclofenac, naproxen	Avoid if possible If no alternative increase renal monitoring to until confirmed to be stable and providing GFR remains >45ml/min/m^2

If the renal function declines to GFR<45ml/min/m^2 a dose reduction is required and the frequency of monitoring of renal function must increase. See Section 6.2.7.A.

6.3.4 Transdermal Oestradiol: Drug Interactions

Tamoxifen should not be prescribed for patients receiving transdermal oestradiol.
The metabolism of oestrogens may be increased by concomitant use of substances known to induce drug metabolising enzymes, specifically cytochrome P450 enzymes, such as anticonvulsants (e.g. phenobarbital, phenytoin, carbamezapine) and anti-infectives (e.g. rifampicin, rifabutin, nevirapine, efavirenz). Ritonavir and nelfinavir, although known as strong inhibitors, by contrast exhibit inducing properties when used concomitantly with steroid hormones. Herbal preparations containing St. John's wort (Hypericum Perforatum) may induce the metabolism of oestrogens.

With transdermal administration, the first-pass effect in the liver is avoided and, thus, transdermally applied oestrogens might be less affected than oral hormones by enzyme inducers. Oestradiol levels are already monitored as part of trial follow-up while patients are on transdermal oestradiol. As a precaution, we recommend monitoring the drug levels of the above concomitant medications among patients receiving transdermal oestradiol.

6.4 TRIAL PRODUCTS

Details of the procedures for obtaining the drugs within the trial, dispensing and disposal of unused drug are given in **Appendix B**. Arrangements for free or discounted drugs are given in the Finance section (**Section 15**).

6.5 TREATMENT DATA COLLECTION

Data will be recorded on case report forms (CRFs); the top copy/original should be sent to CTU for data entry and a copy kept at the local centre. Up-to-date versions of all CRFs can be found on the trial website (http://www.stampedetrial.org/) and centres will be notified of any changes throughout the course of the trial. The type of data to be recorded is detailed in the Assessments and Procedures section (**Section 7**).

6.6 MEASURES OF COMPLIANCE/ADHERENCE

Date of treatment, dose, delays and reasons for delays or dose modifications of all trial treatments will be recorded. The estimated number of abiraterone tablets and enzalutamide capsules taken in a given time period will also be recorded as well as any dose reductions.

Oestradiol levels will be collected for patients in the transdermal oestradiol arm and used to assess compliance to treatment (see **Section 6.2.8**).

Evidence of compliance with safety monitoring is required for patients on research abiraterone or metformin treatment, as described in **Section 6.2.3.B** and **Section 6.2.7.A** e.g. potassium, LFT and blood pressure monitoring for patients receiving abiraterone. Site investigators should document in the patient's medical records the date of the blood test or review of blood pressure measurements and confirmation that the results were known to be within acceptable limits and if not, the toxicity should be graded according to CTCAE and the action described. This should be available at on-site monitoring visits and used to verify the information provided on the follow-up CRF and treatment logs.

6.7 ADMINISTRATION OF STANDARD RADIOTHERAPY TO M0 PATIENTS

6.7.1 Treatment Details

Standard radiotherapy will be given to appropriate patients in each of the trial arms, following a period of neo-adjuvant ADT therapy, as is generally standard in UK practice. For patients with negative nodes on axial imaging, clinicians may choose between irradiating prostate and seminal vesicles alone or including the pelvic nodes in addition. Additional staging tests such as pelvic node sampling may be considered in making this decision. Conformal or intensity modulated radiotherapy should be used in all patients. Where patients have good clinical evidence that nodes are free of tumour or patients for whom nodal radiotherapy is contra-indicated (e.g. significant bowel disease), treatment may be given to the prostate gland and seminal vesicles only. The recommended dose is 74Gy in 37 fractions to the prostate and seminal vesicles or the equivalent using hypo-fractionated schedule, 60Gy in 20 fractions. Alternative dosing schedules are permitted but must be agreed with the STAMPEDE Trial Management Group.

6.7.1.A Standard-Of-Care RT Timing In M0 patients

If receiving docetaxel as part the standard-of-care (permitted from Protocol version 14.0), the patient must have sufficiently recovered from any docetaxel toxicity before RT can begin. In all other patients not receiving SOC docetaxel, SOC RT may be started sooner (2-6 months post-randomisation) consistent with the data from the MRC PR07 trial (11).

7 ASSESSMENTS AND PROCEDURES

7.1 SCHEDULE FOR ASSESSMENTS

7.1.1 Follow-Up Schedules

An individualised form with a follow-up schedule will be provided for each randomised patient. A detailed follow-up schedule is given in **Table 1** and **Table 20**.

Note that for the transdermal oestradiol arm, the first follow-up visit post-randomisation can be scheduled at 4 instead of 6 weeks to coincide with the 4-week hormone tests (see **Section 6.2.8A**).

See **Table 1** for a summary of required investigations at each follow-up visit.

7.1.2 PSA, Testosterone And Oestradiol Measurements

All patients should have PSA measured prior to starting ADT and at every subsequent trial follow-up visit, regardless of allocated treatment arm. For patients who do not have a scheduled hospital visit, it is acceptable for arrangements to be made for blood samples to be drawn at their GP surgery.

For arm L patients, oestradiol and testosterone levels should continue to be monitored while the patient remains on transdermal oestradiol treatment; see **Table 1** for when these measurements should be obtained. These samples could be taken at the same time as the PSA tests, unless additional tests are required as detailed in **Sections 6.2.8.B** and **6.2.8.C**. It is also preferable for samples to be taken the day before the oestradiol patches are changed, to allow consistent measurements of testosterone and oestradiol with respect to the pharmacokinetic profile of the patches.

7.1.3 Assessment Of Treatment Failure (Definition Of Progression)

It is not proposed to routinely assess patients for response. However, in order that objective progression can be assessed, it is necessary to have imaging taken at time of best response as judged by the treating clinician.

All patients should have baseline radiological examinations as detailed in **Section 4.5.2**. In addition, it is recommended all patients should have scans or X-rays repeated at 24 weeks (and whenever clinically appropriate) if they were abnormal at baseline, particularly if they have a low PSA value on entry in to the trial making biochemical assessment of treatment failure difficult.

The following outcomes should be reported on the Progression log:

- Biochemical failure
- Local progression
- Lymph node progression
- Progression or development of new distant metastases, defined as lymph nodes outside the pelvis, bone or organ involvement
- Skeletal-related events confirmed as progression (see below)

7.1.3.A Biochemical Failure

For the purposes of the STAMPEDE trial, a unique threshold PSA value for biochemical failure is calculated, referred to as the **PSA progression value**.

This value is derived for each patient based on their **PSA nadir,** defined as the lowest PSA value reported between randomisation and 24 weeks on trial. Please refer to the PSA progression value calculator on the STAMPEDE website.

The exact method for deriving the progression value for a patient depends on the value of their PSA nadir, and how this compares to their pre-treatment PSA value (i.e. the extent of the fall in PSA from the starting point).

The PSA progression value is calculated in one of three ways:

A. If the lowest recorded PSA value in the 24 weeks following randomisation is more than 4ng/ml and more than 50% of the pre-treatment PSA level then the patient fulfils the criteria for immediate treatment failure.

B. For patients whose PSA nadir in the 24 weeks following randomisation is less than or equal to 50% of the pre-treatment PSA level but remains above 4ng/ml, biochemical failure will be defined as a rise of 50% above the nadir level.

C. For patient whose PSA nadir is less than or equal to 4ng/ml, biochemical failure is defined as at least a 50% rise above the nadir value that is also above 4ng/ml.

Confirming biochemical failure: the timing of assessments needs to be considered because spurious rises in PSA can occur e.g. following procedures involving the urinary tract. For this reason, any isolated rise in PSA should be confirmed before reporting biochemical failure.

In the case that the raised PSA value reaches the progression value, a confirmatory PSA test should be performed between one week and 3 months later. Biochemical failure is confirmed if the second

value is around the same level or higher i.e. the trend is confirmed. The date of PSA progression should be provided as the date of the **first** raised PSA that fulfilled the trial definition of progression. Only the first instance of biochemical failure needs to be reported.

A confirmatory PSA is not required if there are other signs of progression e.g. progression of cancer related symptoms (clinical progression) or new radiological progression.

Second line treatment commenced specifically for biochemical failure should not start until the trial definition for biochemical failure has been met. However, if second line treatment does start before the trial definition is met then report the closest PSA value prior to the treatment start date as the progression value. This is not required if second line treatment is being started for other signs of progression e.g. clinical or radiological.

Testosterone levels: are only required when reporting biochemical progression whilst receiving hormone treatment to confirm the diagnosis of castrate resistant prostate cancer. Testosterone levels are not required when reporting biochemical progression in patients not receiving hormone therapy e.g. patients who presented with non-metastatic disease have relapsed following completion of treatment.

See **Appendix E** for further details on the trial definition of biochemical failure.

7.1.3.B Local, Lymph Node And Metastatic Failure

For each of local, lymph node and distant metastases progression, **both** the following should be reported:

1. Date of first clinical/symptomatic progression
2. Date of first objective/radiological progression

7.1.3.C Skeletal-related Events

Skeletal-related events (SREs) are defined as:

- Pathological Fracture
- Spinal cord compression
- Requirement for RT to bone (e.g. for pain or impending fracture)
- Requirement for surgery (e.g. for prevention or management of fracture)

SREs are a secondary outcome measure and a disease event of interest. SREs may represent disease progression but can also occur due to treatment-related effects e.g. osteoporotic fracture due to treatment-related bone-mineral density loss. From Protocol version 15.0 information regarding SREs will be collected at each follow-up visit. All SREs should be investigated further to establish whether or not the patient has progressed and, if confirmed as progression, a Progression Log should be completed to record this along with an Additional Treatment Log to give details of any treatment received (e.g. palliative RT or surgery)

The summary of timing of Case Report Forms can be viewed in **Table 19**.

7.1.4 Additional Metabolic And Cardiovascular Outcomes

A number of metabolic and cardiovascular (CVS) outcomes are being assessed in the “metformin comparison” and “transdermal oestradiol comparison” as outlined below. From protocol version 17.0 onwards, a metabolic profile (lipids, glucose and HbA1c) will be measured for all patients randomised from Sept-5-2016 onwards to capture data on metabolic and cardiovascular outcomes

for both comparisons. See **Table 1** for a schedule of assessments, please note it is permitted to obtain these measurements within 12 weeks of the scheduled follow-up visit.

The summary of timing of Case Report Forms can be viewed in **Table 19**.

7.1.4.A Cardiovascular Outcomes: Transdermal Oestradiol Comparison

Cardiovascular morbidity and mortality was the primary outcome measure for the first stage in the PATCH trial (completed in 2010), which showed similar rates of CVS events in patients receiving transdermal oestradiol compared to those receiving LHRH injections(43). These results have been confirmed by longer-term data within the trial (see **Appendix I**). Continued monitoring of CVS outcomes will be undertaken by the PATCH IDMC for both the PATCH trial, as well as for the patients in STAMPEDE allocated to transdermal oestradiol together with their contemporaneous controls.

While Arm L patients are undergoing treatment with transdermal oestradiol, the majority of these CVS events will fall under the definitions of Serious Adverse Events (see **Section 11**). Once a patient has a cardiovascular event, the discontinuation of treatment with transdermal oestradiol may be considered at the discretion of the treating clinician and the patient switched to standard of care hormone therapy.

An increased risk of venous thromboembolism has been observed when docetaxel is used in combination with certain agents for the treatment of prostate cancer. As yet, there are limited safety data available on docetaxel use in combination with transdermal oestradiol from the PATCH trial. Therefore, the rate of CVS events will be closely monitored among patients within Arm L who are receiving docetaxel as part of their first-line treatment. For more details see **Appendix I**.

7.1.5 Additional Safety Assessments

Medical review and PSA measurements are repeated for all patients across all research arms (including the control arm) and follow the trial FU schedule. Patients have FU assessments every 6 weeks for 6 months (apart from the transdermal oestradiol arm where the first assessment is at 4 weeks instead of 6 to coincide with the hormone tests), every 12 weeks up to 2 years, six-monthly up to 5 years and annually thereafter). In addition, there are arm-specific assessments as outlined below and summarised in **Table 1**. The summary of the timing of Case Report Forms also can be viewed in **Table 19**.

7.1.5.A Additional Safety Assessment: Abiraterone

Due to the risk of liver toxicity and secondary hyperaldosteronism with abiraterone, all patients require regular monitoring of **potassium, liver function tests and blood pressure** whilst receiving research abiraterone. Monitoring should be performed 2-weekly in the first 12 weeks of treatment, then **monthly until 12-months on treatment** and then, providing treatment is well tolerated, **2-monthly thereafter** whilst treatment with research abiraterone continues, see **Section 6.2.3.B.**

Investigators are responsible for ensuring blood tests are performed at the required frequency and need to review and document the results. It is acceptable for blood pressure to be self-monitored at the required frequency by trial participants or via the GP, providing this is reviewed at each follow-up by investigators.

Confirmation that potassium and liver functions test have been performed regularly and blood pressure control reviewed will be required at each follow-up visit. Any abnormalities should be graded according to CTCAE version 4.0 and recorded on the toxicity section of the follow-up CRF; any

abnormalities fulfilling the criteria for a SAE (e.g. requiring hospital admission) should also be reported on a SAE CRF (see **Section 11**).

7.1.5.B Additional Safety Assessment: Metformin

Patients with normal and stable renal function receiving metformin require monitoring of **renal function (U&Es) every 6 months** whilst on treatment. More frequent monitoring is required in patients with declining renal function, or when initiating new potentially nephrotoxic medications or at times of intercurrent illness (see **Section 6.2.7.A**). Changes in renal function (eGFR, graded according to CTCAEv.4) are recorded on the Follow-Up CRF. It is acceptable for bloods sampling to be arranged via the GP at the patient's home or local hospital.

7.1.5.C Additional Safety Assessment: Transdermal Oestradiol

Hormone levels are monitored while patients are on transdermal oestradiol, and if oestradiol levels are found to be >2000pmol/L with confirmed repeat test, please contact CTU for advice (see **Section 6.2.8.C**).

7.2 DATA COLLECTION PROCEDURES

Treatment-related data are collected on Treatment Specific Forms or Logs. It is important that any treatment given for progressive disease is recorded on the Additional Treatment Log. This should be updated with any subsequent changes e.g. treatment for CRPC. The summary of timing of Case Report Forms can be viewed in **Table 19**.

7.2.1 Data Collection For SOC Hormone Therapy

Information relating to SOC hormone therapy is recorded on the SOC Hormone Therapy Log unless it is a treatment change for disease progression. The SOC Hormone Therapy Log should be updated with any changes in long-term hormone therapy e.g. if anti-androgens are being added to LHRH for dual androgen blockade in the **absence of progression**. If however, anti-androgens are being added as an additional treatment for progressive disease, then this should be recorded on the Additional Treatment Log. SOC hormone therapy only refers to LHRH or anti-androgens; if second-generation AR-targeted treatments such as abiraterone or enzalutamide are used as second-line treatments for progression this should only be recorded on the Additional Treatment Log. Corresponding details of the progression event should be reported on the Progression Log.

If a patient allocated to receive transdermal oestradiol switches to receiving SOC Hormone Therapy i.e. LHRH in the **absence of progression**, then this information should be recorded on the SOC Hormone Therapy Log. However, any changes in hormone therapy initiated to treat disease progression should be recorded on the Additional Treatment Log e.g. switching from transdermal oestradiol to LHRH due to progressive disease.

7.2.2 Data Collection For Standard Docetaxel

The decision to use docetaxel as part of the standard-of-care must be made before randomisation and should be recorded on the Randomisation CRF. The date of the first cycle should be recorded at the time of randomisation; this can be a planned date when randomisation occurs prior to docetaxel commencing but must be within 12 weeks of starting ADT (see **Section 6.2.7**). All further details should be recorded on the SOC Docetaxel Treatment CRF upon completion of the final cycle.

If a patient does not receive the planned docetaxel, this must also be recorded on the SOC Docetaxel Treatment CRF, together with the reason why.

7.2.3 Data Collection And Non-Administration Of Standard Radiotherapy

There are CRFs to be completed for ALL patients regardless of being planned for, or subsequently receiving, primary radiotherapy. Where radiotherapy is not received a reason should be provided on the Radiotherapy Detail CRF whether this is standard-of-care radiotherapy for patients (on any research arm) or research RT to the prostate for Arm H patients.

All radiotherapy and acute side effects details should be recorded on the Radiotherapy Detail and Radiotherapy Acute Toxicity CRFs upon completion of the RT schedule; any RT late side effects should be recorded on the Follow-Up CRF under the section for RTOG Toxicities.

If RT is not given, this should be stated on the Radiotherapy Detail CRF together with the reason for non-administration of the treatment in those instances where RT was planned and not given (for example, due to early metastatic progression or patient refusal).

7.2.4 Data Collection For Palliative Radiotherapy

Details of any radiotherapy given for progressive disease should be recorded on the Additional Treatment Log.

This includes palliative RT for SREs e.g. bone pain and spinal cord compression (note that these should also be reported as SREs on the Follow-Up CRF and only reported on the Progression Log and Additional Treatment Log if confirmed as progression), as well as salvage RT to the prostate.

7.2.5 Data Collection for Research (M1) Radiotherapy

Arm H only: all radiotherapy and acute side-effects details will be recorded on the Radiotherapy Detail and Radiotherapy Acute Toxicity CRFs upon completion of the RT schedule; any RT late side effects will be recorded on the Follow-Up CRF under the section for RTOG Toxicities.

In those cases where RT is not given (for example, due to early metastatic progression or patient refusal), this should be stated on the Radiotherapy Detail CRF together with the reason for non-administration of the treatment.

7.2.6 Data Collection for Additional Treatments Given for Disease Progression

All treatments given for disease progression are recorded on the Additional Treatment Log. This should be updated with all subsequent changes to treatment. Only treatments for progressive disease need to be recorded; details of supportive treatments such as pain killers or bone-strengthening agents e.g. zoledronic acid, given to relieve symptoms, does not need to be provided.

In some scenarios, SOC hormone therapies such as LHRH or anti-androgens may be given as a treatment for progressive disease. For example, LHRH may be re-started on relapse for patients with M0 disease who discontinued hormone therapy and commenced surveillance. In addition, patients allocated to trandermal oestradiol may switch to LHRH on progression. Historically, some patients progressing on LHRH will have commenced anti-androgens (dual androgen blockade) as a treatment for progression. In all cases, if treatment is being started for disease progression, treatment data are collected on the Additional Treatment Log and the details of the progression event recorded on the Progression Log.

Please note that any change in ADT which are solely a change in the patient's long-term hormone therapy, and not for disease progression, should be reported on the SOC Hormone Therapy Log only and not on the Additional Treatment Log.

7.3 FOLLOW-UP PROCEDURE

Every effort should be made to follow-up all patients who have been randomised. Patients should, if possible, remain under the care of an oncologist or urologist for the duration of the trial. If care of a patient is returned to the GP, it is the responsibility of the responsible clinician who obtained the patient's consent to participate in the trial to ensure that all relevant data collection forms are completed. Nurse-led follow-up is permitted and should be conducted in line with local practice and procedures.

If the patient moves away from the local area, arrangements should be made for trial follow-up to be undertaken by their new local centre. Details of other participating centres can be obtained from the STAMPEDE Trial Team. Information on patient transfer procedures is detailed in **Section 8.2.** If the responsible clinician moves, appropriate arrangements should be made to arrange for trial follow-up to continue at the centre.

All efforts should be made to preserve the initial patient's consent for long-term survival information to be flagged through national registries, for example NHS Digital (previously the Health and Social Care Information Centre); Office of National Statistics (ONS) in England/Wales; General Register Office in Scotland; Hospital Episode Statistics (HES) or Public Health England.

Please see **Section 8** for more information on early stopping of follow-up.

7.3.1 Follow-Up Telephone Consultations

In certain circumstances it may be appropriate to replace hospital visits with telephone consultations providing that it is still possible to collect all the necessary follow-up information. Situations where this may be considered include at the point where patients would normally be discharged from oncology or urology services. In these instances, it is acceptable to alternate appointments with telephone consultations providing the required blood results are available to the research team. All necessary information required to complete the Follow-Up CRF is still required. All details on the telephone consultation must be recorded in the patients' notes as per in person assessments.

Other circumstances where it may be appropriate to use telephone consultations is when assessing treatment tolerance and advising regarding dose modifications, providing that all the required safety monitoring has been adhered to. For example, when re-commencing metformin after a treatment break it may be appropriate to confirm tolerance and advise regarding dose escalation over the phone.

7.4 TRIAL CLOSURE

For the purpose of complying with UK Clinical Regulations introduced in May-2004, each comparison will only be considered 'closed' when follow-up has ceased. This will be reviewed for each comparison separately after the point of the primary analysis and, if appropriate, a later, updated analysis. Longer term outcome data may be sought via site research teams and/or through linkage with national registers where possible and adequate consent has been obtained.

Table 19: Summary of timing of case report forms

CASE REPORT FORMS	TIMING OF ASSESSMENT AND CRF
**Registration***	
Registration	At registration. For patients participating in the Biomarker-Screening Pilot, when FFPE tumour sample has been retrieved and is ready to be sent to the Sponsor's designated laboratory.
Biomarker Test Request Form	As soon as possible after registration.
Blood Form	As soon as possible after registration.
Saliva Pathology	As soon as possible after registration.
**Baseline**	
Randomisation	At randomisation
Baseline	At randomisation
Cardiovascular Assessment	At randomisation
Bone Density Risk Factor	At randomisation
Saliva Pathology	At randomisation or any point on trial. When saliva sample has been taken and sent to Sponsor's designated laboratory.
**Treatment**	
SOC Docetaxel Treatment	To be completed for all patients 20 weeks after randomisation.
SOC Hormone Therapy Log	To be completed every time there is a change in SOC hormone therapy to report (including when Arm L patients switch to SOC HT pre-progression). To be sent in with the corresponding Follow-Up CRF.
Abiraterone and Enzalutamide Treatment Log	To be completed when treatment is first started and subsequently every time there is a dose change, treatment pause and re-start.<br>To be sent in with the corresponding Follow-Up CRF.
Metformin Treatment	To be completed when treatment is first started and subsequently every time there is a dose change, treatment pause and re-start.<br>To be sent in with the corresponding Follow-Up CRF.
Transdermal Oestradiol Treatment Log**	To be completed when treatment is first started and subsequently when reporting change in dose or type of patch.
RT Detail	To be completed for all patients:<br>Upon completion of SOC RT<br>If planned RT is no longer to be given (at 10 months after randomisation)<br>Arm H patients when research RT completed<br>Arm A patients with newly-diagnosed M1 disease at 3 months to confirm RT was not given
RT Acute Toxicity	For all patients who receive primary RT
**Assessments**	
Follow-Up	To be completed every 6 weeks for 6 months, then every 12 weeks until 2 years, then every 6 months until 5 years and annually thereafter*. (See Table 1 for more information)

CASE REPORT FORMS	TIMING OF ASSESSMENT AND CRF
Toxicity	Required at each follow-up and in the event that treatment is changed due to toxicity.
Transdermal Oestradiol Treatment Hormone Results Log	To be completed whenever there are testosterone and oestradiol test results while arm L patients on transdermal oestradiol.
End of Research Treatment	To be completed when (each) allocated research treatment is permanently stopped or in the event that allocated research treatment is never started (in each case a reason for stopping/never starting should be provided).
Progression Log	To be completed at the occurrence of each progression event (PSA, local, nodal, distant metastases) and for each method of detection (clinical/symptomatic and objective/radiological).<br>Skeletal-related events confirmed as progression should also be reported here.
Additional Treatment Log	To be completed each time a patient who has progressed starts or completes any additional treatment for progression.
Serious Adverse Event	To be completed following any Serious Adverse Event having confirmed none of the trial specific exemptions are met
Death	At Death
**Administration**	
Patient Transfer Confirmation Form	To be completed when a patient is transferred to a different hospital for the administration of trial treatment and follow-up
Co-enrolment	To be completed when a patient is co-enrolled in any other clinical trial. Please see **Section 5.2** for more information

* For centres participating in the biomarker-screening only
** For the transdermal oestradiol arm, the 6-week follow-up form can be completed at the same time as the 4-week visit for the hormone tests (see Section 6.2.8.A)

Table 20: Schedule for completion of treatment and outcome forms by arm

Timing From Randomisation			Treatment Log$
Years	Months	Weeks	(if required)
**6-Weekly**			
-	-	6*	G, J, K, L
-	-	12	G, J, K, L
-	-	18	G, J, K, L
-	6	24	G, J, K, L
**12-Weekly**			
-	9	36	G, J, K, L
1	12	48	G, J, K, L
-	15	60	G, J, K, L
-	18	72	G, J, K, L
-	21	84	G, J, K, L
-	-	96	G, J, K, L
**6-Monthly**			
2	24	104	G, J, K, L
	30	130	G, J, K, L
3	36	156	G, J, K, L
	42	182	G, J, K, L
4	48	208	G, J, K, L
	54	234	G, J, K, L
5	60	260	G, J, K, L
**Annual**			
6	72	-	G, J, K, L
7	84	-	G, J, K, L
Etc.	-	-	G, J, K, L

Key:

G = SOC + abiraterone
J = SOC + enzalutamide + abiraterone
K = SOC + metformin
L = Transdermal oestradiol ± RT ± docetaxel

Notes:

* For the transdermal oestradiol arm, the 6-week follow-up form can be completed at the same time as the 4-week visit for the hormone tests (see Section 6.2.8.A)

$ For patients in Arm L on transdermal oestradiol, the hormone tests results are to be reported on the Transdermal Oestradiol Treatment Hormone Results Log

8 STOPPING OF TREATMENT OR FOLLOW-UP

Patients should be given every encouragement to adhere to their allocated protocol treatment and follow-up schedule, in order to reduce bias. However, a patient has the right to withdraw consent for participation in any aspect of this trial at any time.

8.1 STOPPING RESEARCH INTERVENTIONS

A patient may stop **any trial treatment** for the following reasons:

- Unacceptable toxicity
- Intercurrent illness which prevents further treatment
- Patient refusal
- Any alteration in the patient's condition which justifies the discontinuation of treatment in the clinician's opinion

8.1.1 Stopping Trial Treatment: Abiraterone, Enzalutamide + Abiraterone

For **patients randomised to Arm G or J**, trial treatment should also be discontinued for the following reasons:

- Disease progression whilst on therapy (please refer to **Section 7.1.3**)
- Intention to commence a new anti-cancer treatment due to evidence of relapse

As detailed in **Section 7.1.3**, the disease event for stopping treatment may be after the first reportable Failure-Free Survival event.

In all cases reason for permanent stopping of research treatment should be recorded on the End of Research Treatment CRF.

8.1.2 Stopping Trial Treatment: Metformin

For **patients randomised to Arm K,** treatment duration is detailed in **Section 6.2.6**.

Reasons for early stopping of metformin can be:

- Decline in renal function (metformin must be stopped if GFR <30ml/min/1.73m^2, see **Section 6.2.7.A**
- Decline in performance status (WHO PS >2)
- Unacceptable toxicity
- Patient refusal
- Intercurrent illness preventing continued metformin treatment
- Investigator decision e.g. administration of IMP within a CTIMP in CRPC setting

If metformin is paused for more than 3 months or >50% of doses are missed please discuss with the trial team as treatment is likely to need to be stopped.

8.1.3 Stopping Trial Treatment: Transdermal Oestradiol

For **patients randomised to Arm L**, treatment with transdermal oestradiol may be discontinued for the following main reasons:

- Unacceptable toxicity
- Patient refusal

- Intercurrent illness
- Investigator decision
- Cardiovascular event (see **Section 7.1.4B**)

In addition, upon evidence of disease progression and at the investigator's discretion, a switch to LHRH analogues is appropriate to facilitate the addition of further therapies where concurrent treatment with transdermal oestradiol is untested. For patients who are on the 3 patch maintenance dose and have castrate levels of testosterone, there is currently no evidence that increasing the number of patches further once the patient has progressed would be beneficial and is therefore not recommended.

In the event of stopping research treatment, unless a patient states otherwise, consent is assumed for continued recording of trial data.

8.2 PATIENT TRANSFERS

For patients moving away from the area and planning to transfer care, every effort should be made for the patient to be followed-up at another participating trial centre. The patient will need to sign a new consent form. Once this has been done, the new trial centre will take over responsibility for the patient ongoing participation in the trial, until this has been done, responsibility for the patient lies with the original trial centre.

To document the transfer process the main contact person at both the current and receiving hospitals should complete and sign the Patient Transfer Confirmation form.

A fully completed form must be returned to CTU prior to the patient transfer and any outstanding data queries for the patient should be completed prior to transfer.

On receipt of the completed transfer form a member of the STAMPEDE team will confirm the database has been updated and request confirmation of the name of the patient's new clinician. Photocopies of the following documents may then be sent to the new hospital to complete the transfer and copies must be also retained at the original site for monitoring purposes:

- Consent form
- Completed CRFs
- Any documentation relating to the patient's participation in STAMPEDE (patient names must be removed from any documentation).

8.3 EARLY CESSATION OF TRIAL PARTICIPATION

If a patient explicitly withdraws consent to have any further trial data recorded their decision must be respected and CTU must be informed in writing. All communication surrounding the early cessation of trial participation should be noted in the patient's records. Please note data for the patient prior to this decision will still be required.

In the majority of cases, patients continue to give permission for their data and information on their health to continue to be collected via clinical notes and national registries. Any information on the follow-up status, however minimal, would be helpful. Investigators are encouraged to faciliate

ongoing collection of follow-up data for example, through considering telephone consultations (see **Section 7.3.1**).

Early cessation of trial participation should not be undertaken lightly and the site must consider the implications for the trial and the patient in reaching such a decision. Without long-term data, the efficacy of trial treatments would be less reliable and could lead to inconclusive results. The early stopping of trial treatment should not lead to the early cessation of trial participation and in such cases follow-up assessments should be continued as per trial protocol.

Patients can change their minds about withdrawal at any time and re-consent to participate in the trial. Follow-Up data should be collected only from the point of when consent was re-instated.

9 STATISTICAL CONSIDERATIONS

9.1 METHOD OF RANDOMISATION

Patients will be randomised centrally using a computerised algorithm developed and maintained by CTU. Randomisation will be performed using the method of minimisation over a number of clinically important stratification factors with an additional random element. To decrease determinability, the factors are not listed here but can be found in the Statistical Analysis Plan.

Protocol version 16.0 introduced a new allocation, Arm L: transdermal oestradiol. Allocation to Arm K remains available only to non-diabetic patients with no contraindication to metformin.

See **Appendix H** for the allocation weighting of each arm by previous protocol version; this also shows allocation weighting for previously closed research arms.

9.2 OUTCOME MEASURES

The overall, definitive primary outcome measure for each comparison in the trial is overall survival (all-cause mortality), unless otherwise stated. The design of the trial is such that it is important to have additional intermediate primary outcome measures to assess activity in each research arm as the trial progresses.

For comparisons involving research arms B to J the intermediate primary outcome measure is failure-free survival; this and other outcome measures are listed in **Table 21.**

Table 21: Trial Outcome Measures by Comparison Stage (Arms B-J)

COMPARISON STAGE	PRIMARY OUTCOME MEASURE	SECONDARY OUTCOME MEASURES
Pilot phase	Safety*	Feasibility
Activity Stages (AS)	Failure-free survival (FFS)†	Overall survival (OS)<br>Toxicity<br>Symptomatic skeletal events (SSE)
Efficacy Stage (ES)	Overall survival	Quality-of-life<br>Cost effectiveness<br>Failure-free survival†<br>Toxicity<br>Symptomatic skeletal events (SSE)

*Based on toxicity
†Including biochemical failure (see Section 7.1.3 and Appendix E)

For the "metformin comparison" the intermediate and definitive primary outcome measure are the same, being overall survival; see **Table 24** for full details of all outcome measures for that comparison.

For the "transdermal oestradiol comparison", overall survival and progression-free survival are the definitive co-primary outcome measures, and the intermediate primary outcome measure is

progression-free survival (PFS); see **Table 26.** The rationale for choosing progression-free survival rather than failure-free survival as the outcome measure for this comparison is outlined in **Section 9.8.2**.

The reasons for different emphases in each recruitment stage are explained in **Section 9.3**.

9.3 SAMPLE SIZE: PRINCIPLES

The design is a multi-arm multi-stage, multi-centre, platform, randomised controlled trial. There are a number of stages for each research arm: a Pilot/Feasibility/Safety Phase, Activity Stages and a final Efficacy Stage. Full details of the methodology underlying the trial design are given by Royston et al. (54, 55) The original sample size calculations were performed using the stage2 (version 1.2.0, Mar-2002) and stagen (version 1.1.1, May-2004) programs, both implemented in Stata (Stata Corp, TX) and updated using the later nstage program (version 1.0.3, Jun-2007; version 2.1.0, Jun-2009; version 3.0.1, Sep-2014). (56)

For each of the comparsions, other than transdermal oestradiol, we have adequately powered each comparison to detect an appropriate improvement in overall survival at the final Efficacy Stage, with high power at each of the planned interim Activity Stages on the intermediate primary outcome. For example, in a cohort with 2 years median FFS and 4 years median survival a target HR of 0.75 for research arm relative to control would translate into an absolute improvement in FFS of 10%, from approximately 50% to 60% at two years and OS of 10%, from approximately 50% to 60% at four years.

The “transdermal oestradiol comparison” is powered only for contributing to a meta-analysis of patients from the STAMPEDE “transdermal oestradiol comparison” and the PATCH trial. It will assess non-inferiority of transdermal oestradiol in terms of overall and progression-free survival which are co-primary outcome measures.

As each comparison is powered to detect a difference in relative improvement, the analyses will be performed when the pre-planned number of events has been reported in the control arm, rather than after a certain number of patients have been recruited to the comparison or a certain amount of time has elapsed. Further details of the sample size calculations and varying assumptions for each research comparison are summarised in the relevant **Sections 9.4-9.8** and detailed in a separate Statistical Design Document which are available on request.

As with all trials, changes in both the standard-of-care and second-line therapies over time are possible which improve outcomes and thus will affect the observed control arm event rates and associated reporting timelines. In particular, from Protocol version 8.0, standard-of-care RT was mandated for all patients with N0 M0 disease and no RT contraindication (this is likely to improve outcomes for this subgroup) and docetaxel permitted from Protocol version 14.0. Further agents are starting to be licensed for patients with castrate-refractory disease which may also improve survival rates. Improved FFS rates would delay the intermediate analyses, for comparisons where FFS is the intermediate primary outcome measure; whilst improved survival rates would delay the definitive analyses. Similarly, improved PFS rates could delay both the time of intermediate and definitive analysis for the "transdermal oestradiol comparison". For each comparison event rates are estimated based on data which are publicly available at the time of design. The Statistical Design Document for arms A-K includes models where median survival is varied around such estimated rates.

Figure 4: Schema of progress of STAMPEDE through the trial*

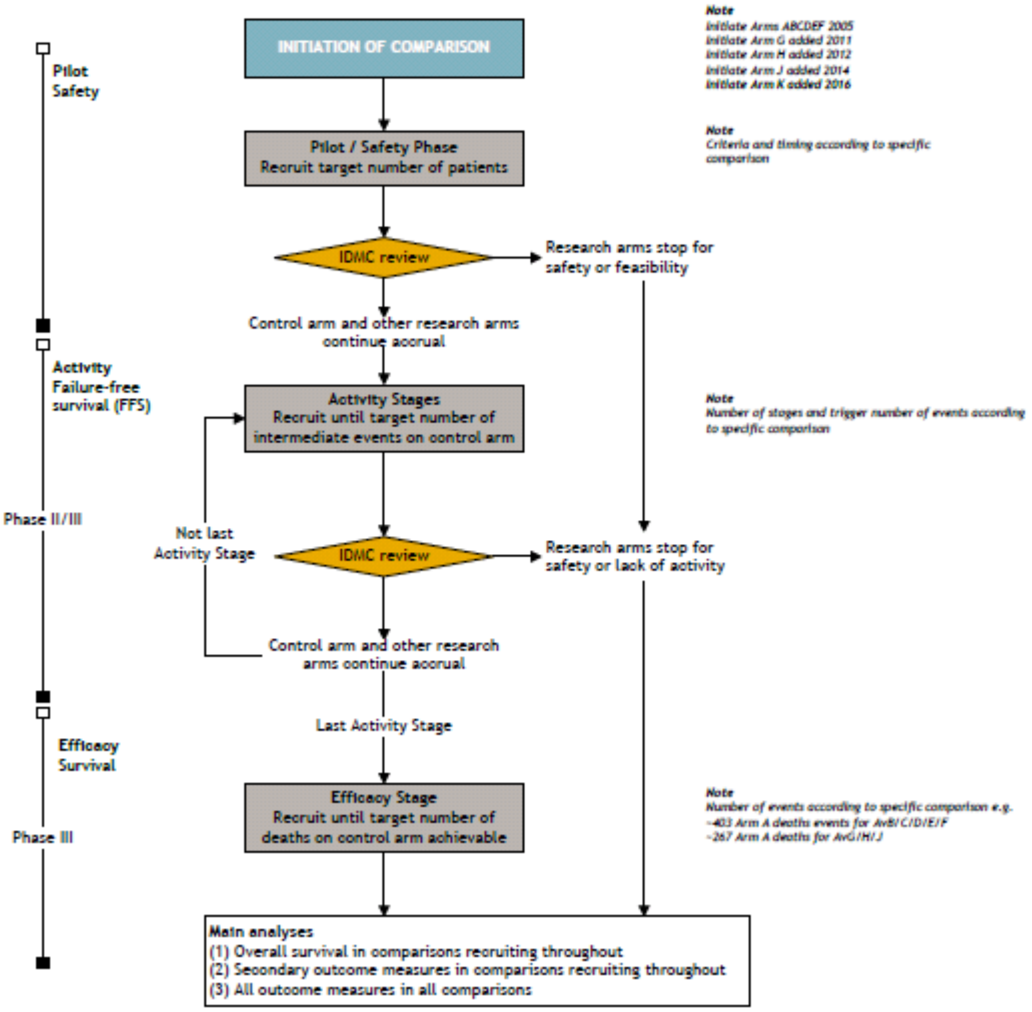

Key
FFS: Failure-free survival
HR: Hazard ratio
IDMC: Independent Data Monitoring Committee
Pts: Patients

Notes
Exact accrual depends on many factors including accrual rate, event rate and arms recruiting in each stage

* Except for the “transdermal oestradiol comparison”

9.4 SAMPLE SIZE ISSUES AND TRIAL STAGES: ADDITIONAL RESEARCH ARM G

This is the "abiraterone comparison" and includes patients allocated to research Arm G (SOC+abiraterone) and patients contemporaneously allocated to the control Arm A (SOC).

9.4.1 Pilot Phase: Additional Research Arm G

The IDMC reviewed safety data, in the context of data from the control arm, when the first 30 patients allocated to Arm G had been on trial for at least 18 weeks.

Furthermore, an additional review of safety was performed when 30 patients with newly-diagnosed non-metastatic disease, allocated to Arm G, had been on trial for at least 18 weeks. Both of these milestones were successfully completed.

9.4.2 Activity Stages I-III: Additional Research Arm G

The same principles were applied to this new comparison as to the original research comparisons. The notable difference was in the accrual rate to this comparison which was anticipated to be higher. There were two reasons for this. First, STAMPEDE initially started recruitment slowly in only a limited number of pilot sites. As more sites have been activated, including internationally, accrual has increased. At the time of adding Arm G (Protocol version 8.0), monthly accrual to the trial was averaging around 60 patients/month (over 700 patients/year). Second, there was an equal allocation ratio for the abiraterone arm compared to the control arm. It was this different allocation ratio which meant that the number of control arm events required to trigger the intermediate analyses was lower for the assessment of abiraterone than the assessment of the original research arms. This is shown in the table below.

Table 22: Guidelines for stopping accrual to additional research Arms G and H

Activity Stage	Sig Level	Power	Targeted HR	Number of control arm events	Consider discontinuation if HR(observed) is…
I	0.50	95%	0.75	~75	>1.00
II	0.25	95%	0.75	~142	>0.92
III	0.10	95%	0.75	~221	>0.89

9.4.3 Efficacy Stage IV: Additional Research Arm G

The analysis of Efficacy Stage IV for this comparison will be performed when around 267 deaths have been observed in the control arm. This will give 90% power to detect the targeted hazard ratio of 0.75 at a one-sided significance level of 0.025.

9.4.4 Sample Size For Additional Research Arm G

Up to around 1,800 patients were targeted join the abiraterone comparison, with half allocated to the research arm G; the observed allocation was 1,917. Consideration was given to ceasing further randomisations to Arm G if it was not showing sufficient evidence of activity at the interim analyses.

The original plan intended for accrual to be halted either when 1,500 patients had been recruited or after 3 years, whichever was the sooner, providing the accrual rate remained above 50 pts/m.

The total number of patients joining this comparison depended not just on observed accrual and event rates, but also the length of time that the original research arms co-recruited alongside this additional research arm; it was originally assumed that this would be for approximately 1 year, but it was closer to 1.5 years. The sample size calculations and projected durations are fairly robust to changes in the length of co-recruitment with the original research arms and future co-recruitment with any further research arms which the Trial Management Group may introduce. Many scenarios are detailed in the Statistical Design Document.

In Protocol version 11.0 in Sep-2013, the target sample size for the "abiraterone comparison" was increased from around 1,500 patients to around 1,800 patients, with note that the efficacy analysis remains unchanged and is still to be triggered by around 267 control arm deaths. This increase in sample size was primarily because of an increase in the proportion of non-metastatic patients joining the comparison; this related to the activation of Arm H which only recruits patients with newly-diagnosed metastatic disease and thereby reduces the numbers of metastatic patients randomised to the "abiraterone comparison". Non-metastatic patients have a lower event rate than the metastatic patients and maintaining the same overall sample size would lead to a delay in time to the primary analysis. The increase in sample size was achievable because recruitment rates to the trial had been substantially higher than the anticipated 50 patients/month for the 6 months preceding the increase.

9.5 SAMPLE SIZE ISSUES AND TRIAL STAGES: ADDITIONAL RESEARCH ARM H

This is the “M1|RT comparison” and includes patients allocated to research Arm H (SOC+RT) and newly-diagnosed M1 patients with no contraindication to RT allocated to the control Arm A whilst Arm H was open to recruitment. Suitability for allocation to the comparison was assessed before randomisation to ensure comparability with contemporaneous control arm patients.

9.5.1 Pilot Phase: Additional Research Arm H

The IDMC reviewed safety data, in the context of data from the control arm, when the first 30 patients allocated to Arm H had been on trial for around six months.

9.5.2 Activity Stages I-III: Additional Research Arm H

The same principles were applied to this new comparison as to previous comparisons and an equal allocation ratio of control arm patients to patients allocated to Arm H was employed; as for Arm G. The number of control arm events required to trigger the intermediate analyses are the same as for the “abiraterone comparison” (see Table 22).

9.5.3 Efficacy Stage IV: Additional Research Arm H

The analysis of Efficacy Stage IV for this comparison will be performed when around 267 deaths have been observed in the relevant control arm patients. This will give 90% power to detect the targeted hazard ratio of 0.75 at one-sided significance level of 0.025.

9.5.4 Sample Size For Additional Research Arm H

Consideration was given to ceasing further randomisations to Arm H if it was not showing sufficient evidence of activity on the intermediate primary outcome measure (FFS), just as for the other research arms. This research comparison is relevant to around 60% of patients joining STAMPEDE. At the point of the scientific approval, accrual was averaging around 80 patients per month to the trial. If accrual to the trial was slower at 70 patients per month, then accrual to this comparison could be

between 18 and 42 patients per month, depending on which other trial arms are open to recruitment at the time.

We are targeting a 25% relative improvement in overall survival following local radiotherapy to the prostate in this patient group. This is the same size of effect targeted with the other research arms in STAMPEDE. This relative improvement can be further justified in the light of MRC PR07 which demonstrated an improvement of this magnitude for adding radiotherapy to ADT in locally-advanced disease, with a hazard ratio for overall survival of 0.77 (95% CI 0.61 to 0.98). In that trial, fewer than half of the deaths were from prostate cancer, whereas in newly-diagnosed metastatic patients nearly all men will die of their disease. Therefore, it is relevant to note the relative benefit of radiotherapy in PR07 in terms of prostate cancer-specific survival, where the hazard ratio was 0.46 (95% CI 0.34 to 0.61) after a median follow-up time of 8 years (57).

We anticipated that around 1250 patients were required over 4 years to observe 267 control arm deaths after 5.25 years. This assumed that (i) recruitment was constantly 70 pts/m to the trial overall; (ii) the original research arms stopped accrual within 6 months after activation of the RT arm; (iii) the abiraterone arm stops accrual around 24 months after activation of the RT arm; and (iv) a further new research arm with an equal allocation ratio was introduced 18 months after activation of the RT arm. In Protocol version 13.0, we reflected on these four points: (i) recruitment to the trial has been faster; (ii) the original research arms completed accrual 2 months after activation of the RT arm; (iii) the abiraterone arm stopped accrual 12 months after activation of the RT arm; and (iv) Arm J was activated 18 months after activation of the RT arm, Arm H.

Of patients joining STAMPEDE during this time, 60% have been eligible for the “M1|RT comparison”. Prior to randomisation, a RT schedule must be nominated: Weekly or Daily. We have observed that around half of patients in the comparison are nominated for RT with the Daily schedule and half for the Weekly schedule, primarily chosen by trial site with patient groups nominated for each schedule observed to be comparable at baseline. There will likely be interest to know the effect of each RT schedule when the main results are reported. This will be explored by “within schedule” comparisons of patients randomised to research vs control (arms H vs A) within each nominated RT schedule.

Therefore, in Protocol version 13.0, the target sample size was increased from 1,250 patients up to around 1,800 patients, resulting in an approximate increase in the split by planned RT schedule from 625 to 900 in each “within schedule” analysis. A FFS analysis “within schedule” will be carried out at the time of the “main analysis”; predicted to have ~300 control arm FFS events by schedule (FFS “within schedule” analysis parameters: target HR=0.75, power 90%, 1-sided α=0.015). For either of the RT schedules showing evidence of an effect on FFS, a comparative “within schedule” analysis will be carried out on survival when ~199 control arm deaths are observed in that schedule comparison. This is a closed test with OS only formally compared within schedule if there is an advantage in FFS for that RT schedule at the main analysis. Thus, extending recruitment enables a secondary analysis of the impact of RT on survival by planned “RT schedule” to happen within around 18 months from the first main analysis.

All sample scenarios are documented in the Trial Master File.

All patients joining the trial will be starting long-term ADT for the first time. The focus of this comparison will be on the newly-diagnosed, metastatic patients (with no contraindications to RT), which is the largest subgroup of patients in the trial and the group of patients at highest risk of death from prostate cancer. Patients with non-metastatic disease will be excluded from this particular comparison as there are already randomised data demonstrating the survival benefit from

radiotherapy in patients with locally-advanced disease. Radiotherapy is now mandatory in node negative patients; it is also recommended in the node-positive, non-metastatic (N+ M0) group. Relapsing patients are also excluded from this comparison.

For the control arm of the whole trial, we constructed sample size scenarios median failure-free survival being 18, 24 or 30 months and constructed sample size scenarios around each of these options; the event rate would depend on the patient mix. We now know that around 60% of patients have M1 disease at trial entry and we have reported that FFS at 24 months is 51% across the whole of the control arm.(58)

For the updated sample size calculation for this comparison, we based our estimates on the subgroup of patients with newly-diagnosed M1 disease in the control arm. Therefore, we estimate median FFS to be 1 year and estimate that median overall survival will be around 3.5 years.

9.6 SAMPLE SIZE ISSUES AND TRIAL STAGES: ADDITIONAL RESEARCH ARM J

This is the "enzalutamide + abiraterone comparison" and included patients allocated to research Arm J (SOC + enzalutamide + abiraterone) and patients contemporaneously allocated to the control Arm A.

9.6.1 Pilot Phase: Additional Research Arm J

The IDMC first reviewed safety data for this combination when the first 50 patients allocated to Arm J had been on trial around 6 weeks (i.e. to the first follow-up visit).

Furthermore, an additional review of safety was performed when these 50 Arm J patients had been on trial for around 6 months. Safety is routinely reviewed at regular intervals and additional safety reviews will be performed if the IDMC raises any concerns.

Direct comparison will be available with contemporaneously randomised patients on Arm A (hormones alone). Contextual data will be provided from Arm G (hormones plus abiraterone). Indicative safety data may also be available on the combination from other studies in CRPC.

9.6.2 Activity Stages I-II: Additional Research Arm J

The principles of intermediate analyses were applied to this new comparison as to previous comparisons, but some of the details were different, and an equal allocation ratio of control arm patients to patients allocated to Arm J was employed; as for Arms G and H. Owing to the expected accrual rate to the trial (>100 pts/m) and the expected slower event rate, only two activity stages were planned before accrual completed. These are set out in Table 23.

Table 23: Guidelines for stopping accrual to the additional research Arm J

Activity Stage	Sig Level	Power	Targeted HR	Number of control arm events	Consider discontinuation if HRJ(observed) is...
I	0.40	95%	0.70	~66	>0.957
II	0.12	95%	0.70	~139	>0.869

9.6.3 Efficacy Stage III: Additional Research Arm J

The analysis of the final Efficacy Stage for this comparison will be performed when around 267 deaths have been observed in the control arm. This would give 90% power to detect the targeted hazard ratio of 0.75 at a one-sided significance level of 0.025.

9.6.4 Sample Size For Additional Research Arm J

Consideration was given to ceasing further randomisations to Arm J if it was not showing sufficient evidence of activity on the intermediate primary outcome measure (FFS), just as for the other research arms.

The patient mix for this comparison is likely to represent a more favourable prognosis on average than in the original research trial's other arms, due to concurrent recruitment of M1 but not M0 patients, to Arm H.

We anticipate that around 1,800 patients are required within 3.5 years to observe ~267 control arm deaths within 6 years. This time will be dependent on the observed overall survival. The default scenario assumes that (i) recruitment is constantly 70pts/m to the trial overall, (ii) the M1|RT arm H accrues throughout and (iii) a further new research arm with an equal allocation ratio is introduced 18 months after activation of Arm J. The stopping date for Arm G is no longer an assumption.

Variations on these factors are documented in a Statistical Design Document. If accrual rates to the trial are at 150pts/m (as observed during Summer 2013), accrual of around 1,800 patients to the comparison could be achieved within 2 years. These sample scenarios will also be documented in the Trial Master File.

Updating the standard-of-care to include docetaxel has minimal impact on the projected time to maturity of the “enzalutamide + abiraterone comparison”.

9.6.5 Further Sample Size Issues For Additional Research Arm J

Careful consideration will be given to the implications of any emerging data from the "abiraterone comparison". This has no effect on recruitment to the “enzalutamide + abiraterone comparison” because the recruitment target was reached before any data are available from the "abiraterone comparison".

Indirect comparisons to understand the contribution from each agent may be possible if this research arm is demonstrably superior to the standard-of-care. These plans will be developed and documented elsewhere, but a higher number of patients will help with the power to the indirect comparison.

9.7 SAMPLE SIZE ISSUES AND TRIAL STAGES: ADDITIONAL RESEARCH ARM K

This is the “metformin comparison” and includes patients allocated to research Arm K (SOC + metformin) and the equivalent non-diabetic patients with no contraindication to metformin contemporaneously allocated to the control Arm A whilst Arm K is open to recruitment. Suitability for allocation to the comparison is assessed before randomisation to ensure comparability with contemporaneous control arm patients

9.7.1 Implementation: Additional Research Arm K

The implementation of the MAMS principles are different in this comparison for the following reasons:

- Although all non-diabetic patients will be eligible for allocation to the "metformin comparison", the timing of the analyses will be driven only by the M1 patients.

- Failure-free survival will not be used as the intermediate primary outcome measure; overall survival will be used as both the intermediate and definitive primary outcome measure. This is because we are not convinced that any comment on metformin's usefulness should be determined from an ability to act on a PSA-driven outcome measure. Furthermore, treatment with metformin is intended to continue throughout long-term hormone therapy which may include going well beyond an FFS event, particularly in M1 patients.

- The target HR is 0.80 for overall survival (a 20% relative improvement). This is a smaller relative improvement in survival than targeted for previous comparisons because of metformin's known low toxicity profile, the low cost of the drug and the potential positive effects on metabolic parameters and morbidity; a smaller impact may still have clinical benefit.

9.7.2 Outcome Measures: Additional Research Arm K

Table 24 lists the outcome measures for this comparison and can be compared with the outcome measures for the other comparisons in Table 21.

Table 24: Trial outcome measures by stage for the "metformin comparison"

COMPARISON STAGE	PRIMARY OUTCOME MEASURES	SECONDARY OUTCOME MEASURES
Pilot phase	Safety*	Feasibility<br>Metabolic effects including:<br>:: Changes in BMI<br>:: Changes in haemoglobin A1c (HbA1c)<br>:: Changes in waist circumference<br>:: New diagnosis of diabetes mellitus<br>:: Cardiovascular event: major adverse cardiac events‡
Activity Stage (AS) I	Overall survival	Metabolic effects<br>Toxicity<br>Symptomatic skeletal events (SSE)<br>Failure-free survival† (FFS)
Efficacy Stage (ES) II	Overall survival	Metabolic effects<br>Toxicity<br>Symptomatic skeletal events (SSE)<br>Failure-free survival† (FFS)<br>Quality-of-life<br>Cost effectiveness<br>Correlative outcomes ₳

*Based on toxicity
‡MACE; nonfatal MI, nonfatal stroke, & death from CV causes
†Including biochemical failure (see Section 6.1.2 and Appendix J)
₳Plasma lipid and fasting triglyceride levels, fasting plasma glucose
Sarcopenia and/or radiological progression free survival (rPFS)
Plasma insulin
AMP Kinase

Note: All arms are unblinded so primary outcome measures for this comparison are objectively measured with caution to be taken around interpretation of more subjective secondary outcome measures such as symptomatic skeletal events

9.7.3 Pilot Phase: Additional Research Arm K

The IDMC will review safety data for this comparisons when the first 50 patients allocated to Arm K have been on trial around 12 months. Furthermore, analyses will be conducted on metabolic parameters (see **Table 24**). If there is harm observed in metabolic effects, or any serious concerns regarding the toxicity profile, recruitment would be stopped; there are no formal criteria to guide this.

Safety is routinely reviewed at regular intervals and additional safety reviews will be performed if the IDMC raises any concerns.

9.7.4 Activity Stage I: Additional Research Arm K

The principles of intermediate analyses will be applied to this new comparison as to previous comparisons, but some of the details will be different, and an equal allocation ratio of control arm patients to patients allocated to Arm K is employed; as for Arms G, H and J. Owing to the expected accrual rate to the trial overall (>100 pts/m) and the interim primary outcome being overall survival, only one intermediate activity stage is planned before accrual is completed; this is set out in the **Table 25**.

Although analyses are triggered by events in M1 patients, they will include all patients in the "metformin comparison"; this will have high power. A separate subgroup analysis in M1 patients (conventionally-powered) and M0 patients (limited power) will then look at consistency of effect; there will be few deaths in M0 patients at this time. The IDMC recommendation will be based on the totality of the available data, including safety, metabolic and compliance data.

Table 25: Guidelines for stopping accrual to the additional research Arm K

Activity Stage	Sig Level	Power	Targeted HR	Number of control arm events	Consider discontinuation if HR_K (observed) is…
I	0.40	90%	0.80	~104 M1 deaths	>0.965

9.7.5 Efficacy Stage II: Additional Research Arm K

The analysis of the final Efficacy Stage for this comparison will be performed when around 374 deaths have been observed for M1 patients randomised contemperaneously to the control arm. This would give 85% power to detect the targeted hazard ratio of 0.80 at a one-sided significance level of 0.025.

As with the intermediate activity, this analysis will include all patients in the comparison, with a separate subgroup analysis in M1 and M0 patients looking at consistency of effect. At this time point we predict <60 control arm M0 deaths will be observed. Further subgroup analyses, defined by the stratification factors, are planned to check for consistency of effect at intermediate and final analyses.

9.7.6 Sample Size For Additional Research Arm K

Consideration will be given to ceasing further randomisations to Arm K if it is not showing sufficient evidence of improvement on overall survival at the intermediate analysis.

We anticipate that around 1,800 patients, including around 1,100 M1 patients, are required over 3 years to observe ~374 control arm M1 deaths over around 8 years. This number and time will be dependent on the observed overall survival. The default scenario assumes (i) recruitment is constantly 100pts/m to the trial overall, (ii) co-recruitment throughout of the equivalent of one other research arm, and (iii) the majority of metastatic patients will also have docetaxel but non-metastatic patients will not.

Variations on these factors are documented in a Statistical Design Document. If accrual rates to the trial are at 150pts/m (as observed during summer 2013), accrual of around 1,800 patients to the comparison could be achieved within 2 years. These sample scenarios will also be documented in the Trial Master File.

Updating the standard-of-care to permit first-line use of docetaxel was assumed within the sample size scenarios and is reflected in the projected time to maturity of the "metformin comparison".

9.7.7 Further Sample Size Issues For Additional Research Arm K

Careful consideration will be given to the emerging data from the "abiraterone comparison" when this reports in 2017.

Analyses for the "metformin comparison" will be timed from randomisation. The point of randomisation compared to the start of hormone therapy may differ, depending on the planned use of docetaxel. This practical information will be reviewed by the TMG and IDMC.

9.8 SAMPLE SIZE ISSUES AND TRIAL STAGES: ADDITIONAL RESEARCH ARM L

This is the "transdermal oestradiol comparison" and includes patients allocated to research Arm L (transdermal oestradiol ± RT ± docetaxel) and the equivalent, eligible patients contemporaneously allocated to the control Arm A (SOC).

The phase III evaluation of the clinical efficacy of transdermal oestradiol will ultimately be based on the relevant data from this comparison within STAMPEDE and the PATCH trial, combined using an individual patient data meta-analysis. The overall evaluation is based on a non-inferiority design.

9.8.1 Implementation And Outcome Measures: Additional Research Arm L

The transdermal oestradiol evaluation is based on the following approach.

9.8.1.A Earlier Stages In The PATCH Trial

- The early stages of the PATCH trial already demonstrated the safety and early activity of transdermal oestradiol in comparison to LHRH therapy (see **Appendix I**)(43). The pilot phase (completed in 2010, n=254) showed the rates of cardiovascular events in the transdermal oestradiol and LHRH arms were similar, and the castration rates were equivalent. These results were confirmed by longer-term data including nearly 900 patients enrolled up to Oct-2015.

- A pre-planned, confidential interim analysis undertaken in Jun-2013, based on progression-free survival, at the end of the Phase II component of the PATCH trial, led the PATCH IDMC to recommend further recruitment for an extension to Phase III. That analysis included 638 patients with 206 PFS events, and reviewed data against a pre-specified non-inferiority margin hazard ratio of 1.25 with a 1-sided alpha 0.25.

9.8.1.B STAMPEDE And PATCH Meta-analysis

- To assess the clinical efficacy of transdermal oestradiol, the relevant data from the STAMPEDE "transdermal oestradiol comparison" will be combined with that data from all patients recruited into PATCH; the data from STAMPEDE will not be analysed alone.

- As the eligibility criteria with respect to the timing of start of ADT differs between the STAMPEDE "transdermal oestradiol comparison" and the PATCH trial (see **Section 4.4.2**), the "transdermal oestradiol comparison" will undergo an initial Pilot Phase to assess castration rates and safety among those patients on Arm L. This will also include a safety review of patients receiving transdermal oestradiol in combination with docetaxel. The data will be reviewed by the PATCH IDMC when there are 30 patients in Arm L who have been followed up for at least 18 weeks. A feasibility review will also be performed at the same time.

- There will be an additional safety review for transdermal oestradiol used in combination with docetaxel, based in the first instance on data from the PATCH trial. This analysis will primarily assess cardiovascular events, but will also review other toxicities including neutropenia. This will be carried out when there are 30 research patients from the PATCH trial who have received both transdermal oestradiol and docetaxel as part of their first-line treatment and been followed up for at least 12 weeks (expected date around May-2017). These results will be made available to the STAMPEDE TMG and TSC, pending approval by the PATCH IDMC, and will inform whether an additional safety review of patients on transdermal oestradiol with docetaxel is required within both STAMPEDE and PATCH.

- The pre-planned Activity Stage II, on intermediate primary outcome measure progression-free survival, will take place based on combined data from the STAMPEDE "transdermal oestradiol comparison" patients and PATCH patients.

- The same approach will be used at the final Efficacy Stage, with progression-free and overall survival as definitive co-primary outcome measures (see PATCH Protocol v10.0 for further details). The rationale for choosing progression-free survival as both the intermediate primary outcome measure and as part of the definitive co-primary outcome measure for the "transdermal oestradiol comparison" is outlined in **Section 9.8.3**.

Table 26 summarises the outcome measures for each stage of this research comparison. The target sample size for the meta-analysis of the "transdermal oestradiol comparison" is approximately 2,000 patients, with around 500 to be recruited through the STAMPEDE "transdermal oestradiol comparison". By Feb-2017, around 1,200 patients had been recruited directly to the PATCH trial.

Table 26: Trial outcome measures by stage for the "transdermal oestradiol comparison"

COMPARISON STAGE	DATA SOURCE(S)	PRIMARY OUTCOME MEASURES	SECONDARY OUTCOME MEASURES
Pilot phase (completed 2010)	PATCH trial	Cardiovascular morbidity and mortality	Castration rates<br>Other toxicities<br>Metabolic effects
Activity Stage I (completed 2013)	PATCH trial	Progression-Free Survival*	Cardiovascular and other toxicities<br>Castration rates<br>Metabolic effects
Activity Stage II+$	PATCH and STAMPEDE trials	Progression-Free Survival*	Cardiovascular & other toxicities
Efficacy Stage III$	PATCH and STAMPEDE trials	Progression-Free Survival*<br>Overall survival	Cardiovascular & other toxicities<br>Prostate cancer specific survival<br>Quality-of-life

* Defined as the earliest among biochemical failure, clinical progression (local progression, lymph node progression, distant metastases), or death from any cause (see Section 9.8.3).

+ In addition, there is Pilot Phase to assess castration rates and safety among Arm L patients within STAMPEDE, since the eligibility criteria with respect to timing of start of ADT differs between the transdermal oestradiol comparison within STAMPEDE and the PATCH trial (see Section 4.4.2).

$ The timing of these analyses is determined by when a pre-specified number of events for the primary outcome measure have been observed in the control arms for the PATCH and STAMPEDE trials combined. Please see the PATCH Protocol v10.0 for further details.

9.8.2 Additional Use of Outcome Data from the "transdermal oestradiol comparison"

Patients allocated to the "transdermal oestradiol comparison" may provide additional consent to participate in translational sub-studies, see **Section 4.6** for details. Subsequent correlative analysis using outcome data from these patients will be undertaken by the STAMPEDE team and collaborators, overseen by the STAMPEDE BRG and other STAMPEDE oversight committees.

9.8.3 Definition of PFS and Use As Co-primary OM: Additional Research Arm L

Note that the definition of progression-free survival (PFS) used within the "transdermal oestradiol comparison" analyses differs slightly to that of failure-free survival used for other research comparisons within STAMPEDE. This is because it includes death from any cause as an event- i.e. both PCa deaths and non-PCa deaths (see **Appendix D** for further details of the definition of progression). Progression-free survival is hence defined as time from randomisation to the first of: biochemical failure, clinical progression or death from any cause.

The use of PFS rather than FFS for the "transdermal oestradiol comparison" has no practical impact on STAMPEDE. The rationale for choosing PFS as part of the co-primary outcome measure for the "transdermal oestradiol comparison" is to capture any potential effects on survival due to the different toxicity profiles between transdermal oestradiol and LHRH.

Although PFS and survival are co-primary endpoints, their respective primary analyses will be triggered at different timepoints particularly because PFS is likely to contain a relatively low proportion of deaths as the contributing first PFS event.

9.9 FURTHER NOTES ON TRIAL DESIGN

9.9.1 Overall Sample Size

Given the adaptive nature of the study, there is no formal overall sample size target, but the numbers of patients required for each comparison are detailed in **Sections 9.4-9.8**. To date, more than 8,000 patients have been recruited overall and at least 10,000 patients will join the trial.

9.9.2 Factorial Design

We note here that we have not employed a factorial design in the original design of this trial because we anticipate the possibility of synergy between SOC, zoledronic acid and docetaxel and between SOC, zoledronic acid and celecoxib.

It would not be possible to assess any such interactions reliably in a factorial trial (see the Statistical Design Document for further details).

9.10 INTERIM MONITORING AND ANALYSES

The accumulating data will be reviewed at regular intervals (approximately annually) by an Independent Data Monitoring Committee (IDMC), including pre-specified formal intermediate analyses of activity data (see also **Section 16**). These analyses will be performed by the trial team at CTU. Only patients randomised contemporaneously, and eligible for that comparison, will be included in the comparison of each research arm against control e.g. patients allocated to the control arm prior to Protocol version 12.0 will not contribute to the "enzalutamide + abiraterone comparison" (Arm A vs Arm J). For the "transdermal oestradiol comparison", the relevant STAMPEDE data will only be analysed as a meta-analysis in combination with the PATCH trial. Therefore, interim data from this comparison will be reviewed by the PATCH IDMC.

The IDMC will be asked to give advice on whether the accumulating data from the trial justifies continuing recruitment of further patients or further follow-up; guidelines for discontinuation of accrual for the relevant Activity Stages, together with results from any other relevant trials will aid them in this. A decision to discontinue recruitment, either in all patients or in selected subgroups, will be made only if the result is likely to convince a broad range of clinicians including those entering patients into the trial and the general clinical community. The intermediate stopping guidelines apply to the intermediate primary outcome measure.

To stop accrual early for benefit in any comparison would require convincing data in terms of the definitive primary outcome measure, overall survival. For example, this could be $p<0.001$ as proposed by Haybittle-Peto.(59, 60) The use of such a guideline for stopping for benefit has a minimal impact on the operating characteristics.

If a decision is made to continue without change, the IDMC will advise on the frequency of future reviews of the data on the basis of accrual and event rates. The IDMC will make recommendations to the Trial Steering Committee (TSC, see **Section 16**) as to whether the trial should continue in its present form. While the trial is ongoing the accumulating data will generally remain confidential, unless the TSC and IDMC agree that the data should be made public.

9.11 OUTLINE ANALYSIS PLAN

Analyses will be performed on an intention-to-treat basis. The standard unadjusted log-rank approach will be applied to analyses of intermediate and definitive primary outcome measures. The impact of potential confounders including the stratification factors used at randomisation will be considered in a Cox proportional hazard model. Flexible parametric models will be used to calculate the absolute differences between the arms to show treatment differences over time and to estimate restricted mean "survival" times (RMST). The estimated difference in restricted means survival time (RMST) will be used preferentially to compare treatment arms if the proportional hazards assumptions required for hazard ratios cannot be supported. The χ^2 test or Mann-Whitney test will be implemented for categorical data comparisons, including toxicity, as appropriate. Where relevant the primary outcome measure(s) (see **Section 9.2**) will be considered for all arms of the trial at each phase, but the main emphasis will be placed on the comparison of the research arms that have continued to recruit throughout the trial.

In the "transdermal oestradiol comparison," a meta-analysis approach will be used to combine data from the STAMPEDE and PATCH trials. The analysis will also take into account the change in randomisation ratio partway through the PATCH trial (from 2:1 for transdermal oestradiol versus LHRH before Feb-2011, to 1:1 thereafter). In addition, as the comparison uses a non-inferiority design, sensitivity analyses will be conducted based on a number of pre-defined definitions for the per-protocol population.

9.11.1 Pilot / Safety Phases

The Pilot Phase randomised patients between all the trial arms so that the results from these patients can be included in the main trial. Feasibility is considered in terms of acceptability of the trial randomisation and reported toxicities and adherence to trial medication. Centres participating in the Pilot Phase for the original research arms were required to keep an anonymised log of all patients assessed for trial eligibility (see Protocol version 2.0) so that the number of patients who did not participate in the study and the number of eligible patients who chose to not participate in the study could be summarised (reasons for non-participation were collected where the patients was willing). The anonymised logs are no longer needed for new research arms (since Protocol version 8.0).

For the patients who are randomised, we shall describe the incidence of expected and unexpected severe toxicities and adverse events/reactions (see **Section 11**) to decide whether to continue with research arms beyond the Pilot Phase.

9.11.2 Activity And Efficacy Stages

The approach to analysis of these stages is summarised within the sample size calculations (see earlier subsections of **Section 9**). Each research arm will be compared in a pairwise fashion against the contemperaneously recruited control arm.

Full details are available in the Statistical Analysis Plan. See **Figure 4** for an overview of the schema of progress.

10 MONITORING AND QUALITY ASSURANCE

10.1 MONITORING AT CTU

Data provided to the CTU will be checked for missing or unusual values (range checks) and consistency over time. If missing or questionable data are identified, staff at the CTU will request that the data be clarified. The exact procedures for data clarification and the amendment of CRFs will be described in the trial Data Management Plan and instructions will be sent to all STAMPEDE institutions as soon as they have been approved to participate in the trial. The CTU will also send reminders for any overdue data.

10.1.1 Central monitoring of consent

Anonymised copies of the initial patient's consent form (including the additional research consent) and any subsequent re-consent should be sent to the STAMPEDE team at the CTU as soon as possible to enable central monitoring of consent. The dates and signatures should be visible on the copies sent to the CTU however the name of the patient in block capitals must be omitted. Any queries resulting after central monitoring will be redirected to sites for clarification.

The original un-anonymised consent forms should be kept at site in the ISF.

10.2 DIRECT ACCESS TO DATA

Collaborating institutions should be aware that direct access to patient data by CTU staff may be required for trial-related monitoring or audit. Patient consent for this will be obtained as part of the general trial consent process.

10.3 VISITS TO INVESTIGATOR SITES

A selection of institutions will be visited at least once during the course of the STAMPEDE trial. The CTU will give the responsible investigator adequate notice of the monitoring visit to allow adequate time, space and staff for these visits. The standard operating procedures (SOPs) for monitoring are available from the CTU.

After the monitoring visit the monitor will complete a site visit report. This report may be circulated to the TMT for comment. Once the TMT have reviewed the report and agreed on any recommendations the monitor will finalise the report and send a copy to the Principal Investigator (PI) at the site. A copy will be kept in the CTU STAMPEDE Trial Master File.

10.4 CONFIDENTIALITY

All information collected during the course of the research will be kept strictly confidential. In addition, all procedures for handling, processing, storage and destruction of data are compliant with the Data Protection Act 1998. No individual patients will be identified when results from the trial are published.

Patients will be asked for permission for information about their health status to be obtained from the Office of National Statistics (ONS) or via NHS Digital (formerly HSCIC) or similar or national equivalent by CTU, if necessary. In addition, patients will be asked for permission to inform their GP of their involvement in the STAMPEDE trial.

11 SAFETY REPORTING

The principles of GCP require that both investigators and sponsors follow specific procedures when reporting adverse events/reactions in clinical trials. These procedures are described in this section of the protocol and in **Section 7**. Further information on the expected toxicities for the investigational medicinal products (IMPs) (LHRH analogues, docetaxel, zoledronic acid, abiraterone, enzalutamide, metformin and transdermal oestradiol) can be found in **Appendix C**.

11.1 SAFETY REPORTING DEFINITIONS

The definitions of the EU Directive 2001/20/EC Article 2 based on the principles of GCP apply to this trial protocol. These definitions are given in **Table 27**.

Table 27: Event Terms and Definitions

TERM	DEFINITION
Adverse Event (**AE**)	Any untoward medical occurrence in a patient or clinical trial patient to whom a medicinal product has been administered including occurrences which are not necessarily caused by or related to that product.
Adverse Reaction (**AR**)	Any untoward and unintended response to an investigational medicinal product related to any dose administered.
Unexpected Adverse Reaction (**UAR**)	An adverse reaction, the nature or severity of which is not consistent with the information about the medicinal product in question set out in the summary of product characteristics (or Investigator brochure) for that product.
Serious Adverse Event (**SAE**) or Serious Adverse Reaction (**SAR**) or Suspected Unexpected Serious Adverse Reaction (**SUSAR**)	Respectively any adverse event, adverse reaction or unexpected adverse reaction that:<br>• results in death<br>• is life-threatening*<br>• requires hospitalisation or prolongation of existing hospitalisation**<br>• results in persistent or significant disability or incapacity<br>• consists of a congenital anomaly or birth defect<br>• Other important medical condition***

Clarifications and Exceptions

*The term 'life-threatening' in the definition of 'serious' refers to an event in which the patient was at risk of death at the time of the event; it does not refer to an event which hypothetically might have caused death if it were more severe.

**Hospitalisation is defined as an inpatient admission, regardless of length of stay, even if the hospitalisation is a precautionary measure for continued observation. Hospitalisations for a pre-existing condition (including elective procedures that have not worsened) do not constitute an SAE.

***Medical judgement should be exercised in deciding whether an AE/AR is serious in other situations. Important AE/ARs that are not immediately life-threatening or do not result in death or hospitalisation but may jeopardise the patient or may require intervention to prevent one of the other outcomes listed in the definition above, should also be considered serious.

STAMPEDE is an adaptive platform protocol in which research treatments are given in addition to standard-of-care (SOC) therapies, or as alternatives in the case of transdermal oestradiol.

11.1.1 Defining treatment for the purposes of safety reporting

- **Protocol research treatments** are the IMPs under investigation in STAMPEDE i.e. the additional or alternative treatments patients allocated to research arms (B-L) receive as part of the STAMPEDE protocol:
    - Arm B: zoledronic acid
    - Arm C: docetaxel
    - Arm D: celecoxib
    - Arm E: docetaxel + zoledronic acid
    - Arm F: zoledronic acid + celecoxib
    - Arm G: abiraterone
    - Arm J: abiraterone & enzalutamide
    - Arm K: metformin
    - Arm L: transdermal oestradiol

  Note, the research treatment in arm H (prostate RT) is not an IMP, but safety reporting requirements to the CTU are the same.

- **Protocol SOC treatments** are standard forms of background treatment permitted as part of the STAMPEDE protocol
    - Licenced ADT (e.g. LHRH analogues) given in the setting of castrate-sensitive prostate cancer.
    - Docetaxel given in castrate-sensitive prostate cancer

  Please note standard forms of ADT e.g. LHRHa given in the setting of CRPC is not considered protocol treatment

- **Non-protocol treatments** are all prostate cancer treatments given following disease progression in the management of CRPC. This may include a “protocol research treatment” which is provided to a participant following disease progression.

11.1.2 Safety data collection in STAMPEDE

Safety data is collected in three ways:

- **Adverse Events** (AEs) are collected systematically for all patients and recorded on the Toxicity CRF required at each follow-up and in the event that treatment is changed due to toxicity. Adverse events are collected whilst trial treatment is ongoing, please refer to the CRF completion guidance available on the STAMPEDE website for further details. The purpose of safety data collected in this way is to enable comparative analysis of toxicity between the control and research group in each comparison therefore all toxicity data needs to be captured on this form.

- **Serious Adverse Event** (SAE) are AEs that fulfil the definition of serious as detailed in **Table 27.** SAEs are reported using the SAE CRF. There are two types of SAEs: these are defined based on their causal relationship to treatment. If the event is judged to be possibly, probably or definitely related to treatment, it is categorised as a Serious Adverse Reaction (SARs). If the event is judged unlikely or unrelated to treatment, it is classed as an unrelated SAE, see **Table 28.** The purpose of expedited SAE data collection is to meet regulatory requirements and to enable central monitoring.

- **Other important medical conditions** including pregnancy occurring in a STAMPEDE patient's partner during the patient's participation in the trial. This must be reported to the CTU within the same timelines as an SAE and the outcome of a pregnancy should be followed up carefully and any abnormal outcome to the mother or child should be reported. Patients who develop any new primary carcinomas should have the event reported on a SAE CRF with the exception of non-melanoma skin cancer (e.g. basal cell carcinomas and squamous cell carcinomas) which do not require reporting.

11.2 SAFETY PROCESSES: EXEMPTIONS

11.2.1 Exemptions: Trial-specific SAE reporting exemptions

The following events which may fulfil the definition of "serious" are exempted from regulatory reporting and therefore do not require an SAE CRF to be completed. They may still require reporting as an AE on the toxicity form, or using an alternative CRF e.g. progression log as appropriate.

- **Serious adverse events** unrelated to STAMPEDE research treatment i.e. unrelated SAEs occurring more than 30 days after stopping STAMPEDE research treatment

- **Serious adverse events** unrelated to protocol SOC ADT i.e. unrelated SAEs occurring more than 30 days after the last exposure to ADT (please note this is assumed to be 30 days after the expiration date of a depot preparation)

- **Serious adverse events** unrelated to protocol treatment (research or SOC) occurring after disease progression, providing research treatment has stopped more than 30 days previously.

- **Non-fatal progression events:** events that fulfil the definition of serious e.g. result in hospital admission, but are due to disease progression are exempt from reporting as an SAE, instead details should be provided on the progression log.

- **Death as a result of disease progression** is also not considered to be a SAE. Do not complete an SAE CRF, instead details should be reported on the STAMPEDE Death Form.

- **Elective hospitalisation** and surgery for treatment of locally-advanced or metastatic prostate cancer or its complications. Instead, record this as a non-trial inpatient admission on the follow-up form under Non-Trial visits.

- **Elective hospitalisation** to simplify treatment or procedures. If related to prostate cancer, record until non-trial inpatient admission on the follow-up form, if unrelated e.g. pre-existing conditions that have not been exacerbated by trial treatment, do not report.

11.3 SITE INVESTIGATOR RESPONSIBILITIES

All toxicities experienced, both non-serious and serious, occurring whilst protocol treatment is ongoing and up to 30 days after discontinuation, should be recorded in the participants medical notes and on the Toxicity CRF linked to the Follow-Up CRF. The toxicity CRF should be sent to the CTU within one month of the corresponding Follow-Up CRF being due.

In addition, if an AE meeting the definition of serious (see **Table 27**) occurs then it is the responsibility of the site investigator to determine if it requires reporting to the CTU, see **Figure 5** for guidance on when it is necessary to complete a SAE CRF.

11.3.1 Investigator Assessment

11.3.1.A Seriousness

When an adverse event occurs the investigator or delegates must first assess whether the event is serious using the definitions given in **Table 27**. If the event is serious and does not meet any of the exemption criteria then it must be reported using the SAE CRF and submitted to the CTU; see **Section 11.2.1** for details on exemptions.

11.3.1.B Grading severity of adverse event

The severity (i.e. intensity) of all AEs/ARs (serious and non-serious) in this trial should be graded using Common Terminology Criteria for Adverse Events (CTCAE) v4.0[4].

The complete CTCAE v4 .0 can be found at: http://evs.nci.nih.gov/ftp1/CTCAE/CTCAE_4.03_2010-06-14_QuickReference_5x7.pdf. Any questions concerning this process should be directed to the CTU team in the first instance.

Please note, prior to September 2016 all events were graded according to CTCAE version 3.0. Please ensure the correct version of the CTCAE grading system is referred to and the correct corresponding version of the CRF.

11.3.1.C Causality

The Investigator must assess the causality of all SAEs in relation to protocol treatment using the definitions in **Table 28**. There are 5 categories: unrelated, unlikely, possibly, probably and definitely related. If the causality assessment is unrelated or unlikely to be related the event is classified as unrelated, therefore an unrelated SAE. If the causality is assessed as either possibly, probably or definitely related then the event is classified as related and therefore a SAR. The assessment of causality determines whether the event requires notification to meet regulatory requirements, see **Section 11.3.2.A** for details.

4 ctep.cancer.gov/reporting/index.html

Table 28: Assigning type of SAE through assessment of causality and expectedness

CAUSAL RELATIONSHIP	DESCRIPTION	EVENT TYPE		
		N/A	EXPECTED	UNEXPECTED
Unrelated	There is no evidence of any causal relationship	**Unrelated SAE**	**No assessment required as unrelated to treatment**	
Unlikely	There is little evidence to suggest there is a causal relationship (e.g. the event did not occur within a reasonable time after administration of the trial medication). There is another reasonable explanation for the event (e.g. the patient's clinical condition, other concomitant treatment).	**Unrelated SAE**		
Possibly	There is some evidence to suggest a causal relationship (e.g. because the event occurs within a reasonable time after administration of the trial medication). However, the influence of other factors may have contributed to the event (e.g. the patient's clinical condition, other concomitant treatments)	**Must assess expectedness to categorise reactions**	**SAR**	**SUSAR**
Probably	There is evidence to suggest a causal relationship and the influence of other factors is unlikely.		**SAR**	**SUSAR**
Definitely	There is clear evidence to suggest a causal relationship and other possible contributing factors can be ruled out.		**SAR**	**SUSAR**

11.3.1.D Expectedness

If there is at least a possible involvement of the protocol research treatment the investigator must assess the expectedness of the event, i.e. expectedness must be assessed for all SARs. An unexpected adverse reaction is one not previously reported or one that is more frequent or more severe than previously reported in the reference safety information. The definition of an unexpected adverse reaction (UAR) is given in **Table 27.** If a SAR is assessed as being unexpected, it becomes a SUSAR.

The reference safety information will either be the current Summary of Product Characteristics (SPC) or Investigator Brochure. The reference safety information for all protocol research treatment and common forms of SOC ADT are summarised in **Appendix C.**

11.3.2 Notification responsibilities

STAMPEDE investigators are responsible for notifying the CTU of all serious events that do not fulfil any of the trial-specific exemptions (see **Section 11.2.1**). All unrelated SAE, SARs and SUSARs must be notified within 24 hours of the investigator being made aware of the event. The notification period differs according to whether the event is classified as an unrelated SAE, SAR or SUSAR. See **Table 28** for details on event classification.

11.3.2.A Unrelated SAEs

All unrelated SAEs must be reported up until STAMPEDE protocol treatment has been discontinued for 30 days. In the case of LHRH analogue this is assumed to be 30 days after the depot expiration date e.g. up to 8 weeks after a 4-week depot or 16 weeks after the last administration of a 12-week depot. Providing research treatment has been stopped, unrelated SAEs are also exempt from reporting **after disease progression**, see **Section 11.2.1** for details on exemptions.

11.3.2.B SARs and SUSARs

All reactions judged by the investigator as possibly, probably or definitely related to protocol research or SOC treatment are reportable whilst follow-up continues.

11.3.2.C Other important medical conditions

All other important medical conditions are reportable regardless of when they occur following randomisation whilst follow-up continues.

It should be noted that docetaxel, abiraterone and enzalutamide may be given as non-trial treatments in the management of CRPC. It is not necessary to report safety data to the STAMPEDE trial team relating to this non-trial use, instead the yellow card system should be used to notify the regulatory authorities of adverse drug reactions if appropriate (https://yellowcard.mhra.gov.uk/)

See **Figure 5** which provides an overview of when SAE, SAR and SUSARs are required to be reported to the STAMPEDE Trial team.

Figure 5: SAE reporting flowchart

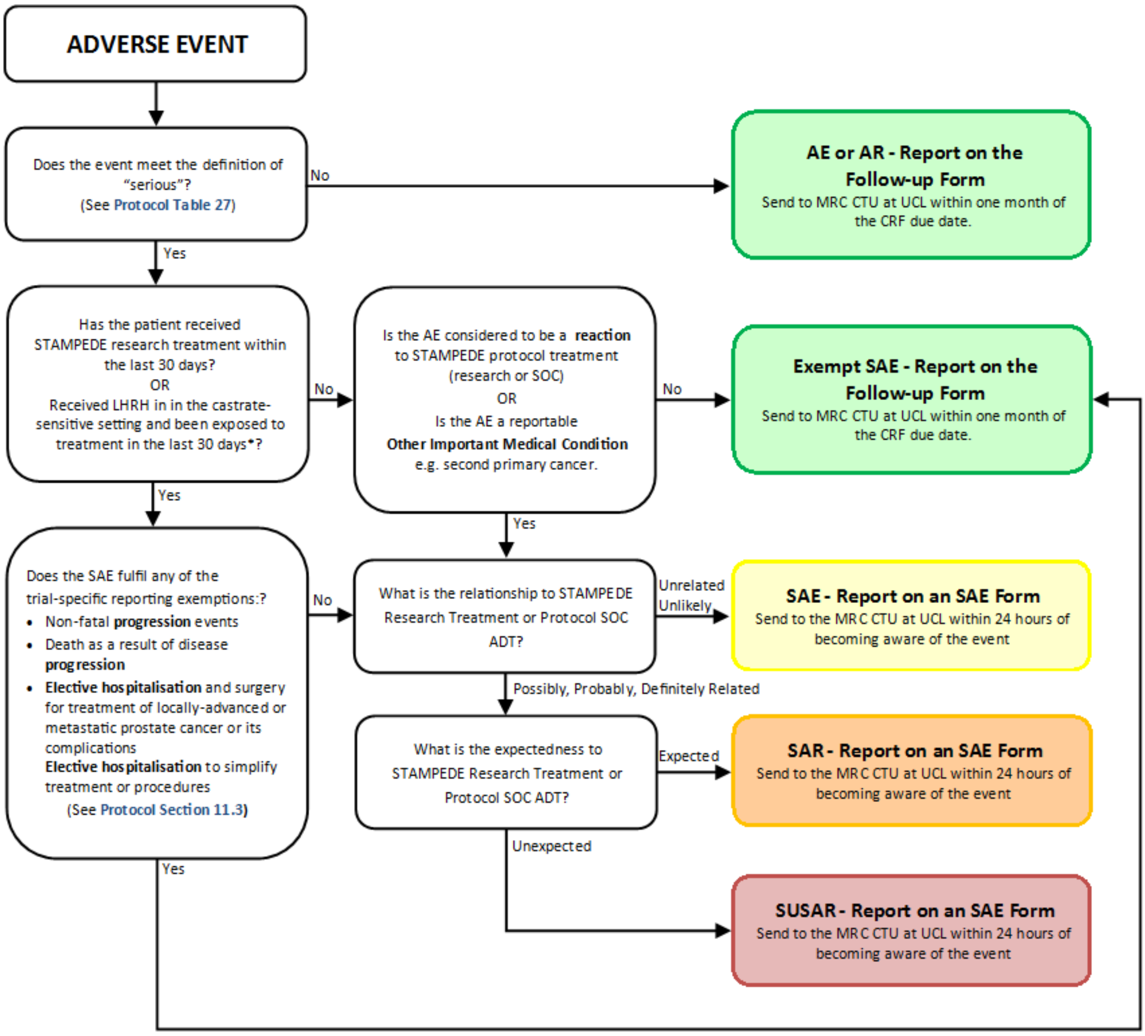

**Exposure to LHRHa is assumed to be until the depot expiration date, therefore unrelated SAEs are reportable up until 8 weeks after the administration of a 4-week depot or 16 weeks after the administration of a 12-week depot.*

Box 1: SAE report notification checklist

Before sending the SAE CRF please check that the event does not meet any of the exemption criteria, see Section 11.2.1. Once confirmed, please ensure that the information provided meets **all** of the following minimum criteria required for initial processing and review:

1. At least **two** patient identifiers
2. Indication of why the event was **serious**
3. **Grade** severity of event/reaction according to CTCAE version 4.0
4. Assessment of **causality** in relation to research protocol treatment and SOC protocol treatment – *confirm that this is a reportable event*
5. Assessment of **expectedness if possibly related to treatment**. Please refer to list of expected toxicities in Appendix C
6. Provide the **date of last administration** for all trial treatments (minimum month/year) – *if reporting an unrelated SAE confirm that this is still reportable*
7. **Signature** (if not by a clinician, by a site trial team member in the first instance)

SAE REPORTING
Fax to 020 7670 4818 within 24 hours of becoming aware of the event
Or send via **encrypted** email to mrcctu.stampede@ucl.ac.uk

11.3.3 Event Follow Up

Patients must be followed-up until clinical recovery is complete or stabilised. Follow-up should continue after completion of protocol treatment if necessary. Follow-Up information can be updated on the original SAE CRF by ticking the box marked “follow-up” and faxing to the CTU as information becomes available. Extra, annotated information and/or copies of test results may be provided separately. The patient must be identified by trial number, date of birth and initials only. The patient’s name should not be used on any correspondence.

11.4 CTU RESPONSIBILITIES

The STAMPEDE trial team will confirm receipt of the SAE report to the main point of contact via email. Please contact the STAMPEDE trial team if receipt is not received within 24 hours.

At least one medically qualified person at the CTU, or comparison chief-investigator or another appropriate TMG member will review all SAE reports received. The causality assessment given by the

local Investigator at the hospital cannot be overruled and in the case of disagreement, both opinions will be provided in any subsequent reports.

The CTU is undertaking the duties of trial sponsor and is responsible for the reporting of SUSARs and other SARs to the regulatory authorities (through the MHRA to competent authorities in other European member states) and the UK research ethics committees; and in addition has sponsor oversight for reporting in other countries in which the trial is taking place.

The CTU will also keep all investigators informed of any safety issues that arise during the course of the trial.

12 ETHICAL CONSIDERATIONS AND APPROVAL

12.1 ETHICAL CONSIDERATIONS

12.1.1.A Randomisation

This is a randomised trial therefore neither the patients nor their physicians will be able to choose the patients' treatment. Treatment will be allocated randomly using a computer-based algorithm. This is to ensure that the groups of patients receiving each of the different treatments are as similar as possible.

All patients, with the exception of those allocated to transdermal oestradiol (Arm L), will receive standard hormone treatment. All patients, including those allocated to Arm L, may also receive other standard-of-care treatments which may include prostate radiotherapy and/or docetaxel. Use of radiotherapy and/or docetaxel will be unaffected by trial participation and is left to the discretion of the treating clinician and patient. Patients may be randomised to receive additional treatment (metformin) given with standard-of-care treatments, or an alternative form of hormone treatment (transdermal oestradiol). An even allocation ratio is being currently being used which means all eligible patients have an equal chance of being randomised to the control or research arms.

Through the introduction of a "transdermal oestradiol comparison" into the STAMPEDE trial platform, sufficient data will be collected to evaluate this treatment approach more rapidly. By undertaking a meta-analysis using data collected in both PATCH and STAMPEDE, fewer patients overall are allocated the control arm i.e. more patients gain access to novel treatments.

12.1.1.B Evaluation of Novel Therapeutic Strategies

The newer treatment options are being assessed in a detailed and systematic fashion in this trial. There is some evidence to suggest that the newer treatment options may have advantages over standard treatment alone with regards to clinical outcome, but this is not confirmed and toxicity may be increased. This trial will follow a large group of men who have been randomly allocated to either the standard treatment(s) or the novel treatment strategies in order to measure the benefits of these approaches. The patients will also be followed-up for toxicity and safety issues, so that any benefits can be weighed against any negative aspects including the impact treatments have on other aspects of medical health e.g. cardiovascular disease, as well as quality-of-life and value for money (health economic analysis).

12.1.1.C Additional Tests and Hospital Visits

Patients participating in the trial will have some additional hospital visits and some extra blood samples taken compared to patients who are not participating in the trial, with the amount varying according to the allocated treatment and stage of disease. Sometimes the blood samples can be taken when the patient is attending hospital for treatment anyway. On some of the trial arms, the patient may have to make additional visits to the hospital for the blood sample to be taken, although in some cases it may be possible for the blood sample to be taken in the GP's surgery.

12.1.1.D Facilitating Participant Feedback From Investigations and Additional Analyses

For participants who choose to take part in additional sub-studies, biological samples including blood, saliva and remaining stored FFPE tumour samples will be used in research projects. These projects will enable the study of genetic factors and other biomarkers that can help identify individuals who serve to benefit most from the treatments tested in STAMPEDE and to further understand why and how treatment resistance develops. All samples will be link-anonymised and only made accessible to approved collaborators granted access by the STAMPEDE oversight

committees. We will make every effort to protect the confidentiality of this information and make sure personal identities are protected.

From Protocol version 16.0, patients may opt to receive feedback regarding genetic results that may arise from analyses of research analyses of genetic material extracted from any of the biological samples collected as part of the trial e.g. saliva, FFPE tumour blocks or circulating tumour DNA extracted from blood. Only results which are of established clinical relevance and for which testing would be available under standard NHS genetic testing guidelines will be fed back. Any genetic analysis undertaken as part of additional research associated with STAMPEDE does not replace clinically indicated investigations as only a proportion of STAMPEDE patient will undergo prospective testing and therefore it cannot be guaranteed that results will be fed back in a timely fashion.

This change has been made in response to emerging data that demonstrates a small proportion of men may have genetic faults in genes such as BRCA2. This has implications for both patients and potentially their biological relatives. For patients and their treating clinician, knowledge of this information may facilitate access into further clinical trials and may potentially impact on the choice of treatment following progression i.e. metastatic castrate-resistant prostate cancer.

Any patient who consents to receive feedback and in whom a known pathogenic mutation of clinical significance is detected on testing of research samples collected as part of STAMPEDE will be told of this. Patients will be recommended to undergo genetic counselling accessed via clinical genetics services and consider confirmatory testing. This is necessary to determine if the defect is germline (inherited) and ensures access to appropriate ongoing support. If confirmed as a germline (inherited) abnormality, this will enable biological relatives to also access appropriate genetic counselling and testing if they wish.

However, patients and STAMPEDE investigators are informed that any genetic analysis undertaken as part of additional research associated with STAMPEDE does not replace clinically indicated investigations as only a proportion of STAMPEDE patient will undergo prospective testing and therefore it cannot be guaranteed that results will be fed back in a timely fashion. Therefore participation in the additional research conducted on biological samples collected as part of STAMPEDE should not impact on an clinicians decision to recommend genetic screening.

The introduction of the “metformin comparison” means that all patients, not known to be diabetic, will be screened for diabetes prior to trial entry. This is to enable the effect of metformin to be studied in non-diabetic patients. All patients in whom screening bloods are abnormal will be referred for confirmatory tests and further management according to local guidelines e.g. via their GP. Screening is expected to lead to a small proportion of potential trial participants receiving a new diagnosis of diabetes but will ensure appropriate management of both conditions.

12.1.1.E Considering the Impact of Emerging Data

If new information emerges during the course of the trial which may affect the treatment or follow-up of patients who have joined the trial, information will be provided through by the trial team to all Principal Investigators. PIs therefore have the duty to inform the patients in their care of any new information emerging using any appropriate channel (e.g. letter, communication at follow-up clinic, etc.)

12.2 ETHICAL APPROVAL

The protocol has a Favourable Opinion from an appropriate Research Ethics Committee, according to national guidelines. Additionally, each site must also obtain management permission for research (Local R&D approval or equivalent) from the relevant host organisations before patients can be entered into the trial. The patient's informed consent to participate in the trial should be obtained after a full explanation has been given of the treatment options, including the conventional and generally accepted methods of treatment. Patient information sheets and patient consent forms are available on the STAMPEDE website (www.stampedetrial.org).

The right of the patient to refuse to participate in the trial without giving reasons must be respected. After the patient has entered the trial, the clinician must remain free to give alternative treatment to that specified in the protocol, at any stage, if he feels it to be in the best interest of the patient. However, the reason for doing so should be recorded and the patient will remain within the trial for the purpose of follow-up and data analysis according to the treatment option to which he has been allocated. Similarly, the patient must remain free to withdraw at any time from the protocol treatment without giving reasons and without prejudicing his further treatment.

A statement of MRC policy on ethical considerations in clinical trials of cancer therapy, including the question of informed consent, is available from the MRC Head Office web site (http://www.mrc.ac.uk). In addition, the MRC and the Wellcome Trust framework on the feedback of health-related findings in research is readily available (https://www.mrc.ac.uk/documents/pdf/mrc-wellcome-trust-framework-on-the-feedback-of-health-related-findings-in-researchpdf/) and has been used when developing the trial specific processes.

13 REGULATORY APPROVAL

This trial has been approved in the UK by the MHRA and will be conducted under a CTA (Ref: 00316/0026/001-0001) in the UK.

The trial has been approved in Switzerland by Swissmedic (Ref: 2009 DR 3235).

14 INDEMNITY

University College London holds insurance against claims from participants for injury caused by their participation in this clinical trial. Participants may be able to claim compensation if they can prove that UCL has been negligent. However, as this clinical trial is being carried out in a hospital, the hospital continues to have a duty of care to the participant of the clinical trial. University College London does not accept liability for any breach in the hospital's duty of care, or any negligence on the part of hospital employees. This applies whether the hospital is an NHS Trust or otherwise.

Participants may also be able to claim compensation for injury caused by participation in this clinical trial without the need to prove negligence on the part of University College London or another party. Participants who sustain injury and wish to make a claim for compensation should do so in writing in the first instance to the Chief Investigator, who will pass the claim to the managing organisation's Insurers, via the managing organisation's office.

Hospitals selected to participate in this clinical trial must provide clinical negligence insurance cover for harm caused by their employees and a copy of the relevant insurance policy or summary can be provided on request.

15 FINANCE

STAMPEDE is funded by Cancer Research UK's Clinical Research Committee (formerly the Clinical Trials Advisory Awards Committee; CTAAC). It is also funded by the MRC through the MRC Clinical Trials Unit at UCL. The trial has National Institute for Health Research Clinical Research Network (NIHR CRN) approval and, therefore, local NCRN funds may be available at each centre to support entry of patients into this trial.

Funding arrangements for research arms now closed to recruitment can be found in **Protocol version 13.0**

Standard therapies including **ADT**, **prostate radiotherapy** and **docetaxel** will be administered as per routine clinical care using local NHS supplies.

Abiraterone is manufactured by Janssen Pharma PV (pharmaceutical companies of Johnson & Johnson). They have agreed to provide free drug and funds to distribute drug to participating sites and to help support the conduct and management of the trial.

Enzalutamide is manufactured by Astellas Pharma. They have agreed to provide free drug and funds to distribute drug to participating sites and to help support the conduct and management of the trial.

Metformin will be administered using local NHS supplies.

Transdermal oestradiol will be administered as Progynova TS 100 patches, manufactured by Bayer who have agreed to supply these patches at a trial-specific discounted price. All accredited STAMPEDE centres will be able to order Progynova patches through AAH Pharmaceuticals Ltd wholesalers at the discounted rate.

Biomarker-Screening Pilot will be funded by Clovis Oncology who will fund all sample analysis and help support the coordination of sample retrieval including site reimbursement through an educational grant to the MRC CTU at UCL.

16 TRIAL COMMITTEES

16.1 TRIAL MANAGEMENT GROUP (TMG)

A Trial Management Group (TMG) has been formed comprising the Chief Investigator, other co-investigators and members of the CTU. The membership of the TMG may be expanded if other groups of trialists wish to participate. It will also be amended during the trial if other circumstances require e.g. retirement.

The TMG will be responsible for the day-to-day running and management of the trial. They will meet by teleconference at least 3-monthly and in person as needed. The TMG members are detailed in **Appendix F**.

Further details of TMG functioning are provided in the TMG charter (available on request).

16.2 TRIAL STEERING COMMITTEE (TSC)

A Trial Steering Committee (TSC) has been formed to provide overall supervision for the trial and provide advice through its independent chair. The ultimate decision for the continuation of the trial lies with the TSC. The TSC will meet regularly.

Further details of TSC functioning are provided in the TSC charter (available on request).

16.3 INDEPENDENT DATA MONITORING COMMITTEE (IDMC)

An Independent Data Monitoring Committee (IDMC) has been formed. The IDMC will be the only group who sees the confidential, accumulating data to the trial. Reports to the IDMC will be produced by the CTU. The IDMC will meet within 6 months of the trial opening with the frequency of meetings dictated by the IDMC. The IDMC will consider data in accordance with the analysis plan (see **Section 9**) and will be advisory to the TSC. The IDMC can recommend premature closure or reporting of the trial, or that recruitment to any research arm is discontinued.

From Protocol version 8.0 onwards, any recommendation from the IDMC to stop recruitment to one or more trial arms will be acted upon immediately, pending ratification from the TSC. As this period between meetings should be very short, sites would not be notified until after the TSC have made a decision. IDMC recommendations based on emerging safety issues will be discussed with sites promptly.

Further details of IDMC functioning and the procedures for interim analysis and monitoring are provided in the IDMC charter (available on request).

Data from the “transdermal oestradiol comparison” are viewed by the PATCH IDMC, in meta-analysis with PATCH, rather than by the STAMPEDE IDMC. Recommendations of any actions relating to STAMPEDE would be made to the STAMPEDE TSC.

16.4 TRIAL EXPERT PANELS

The trial has two established translational expert groups chaired byTMG members the Biological Research Group (BRG) and the Metabolic Translational Group (MTG). Both groups input and provide expert oversight of relevant translational aspects of the trial and associated sub-studies.

Figure 6: Diagram of relationships between trial committees

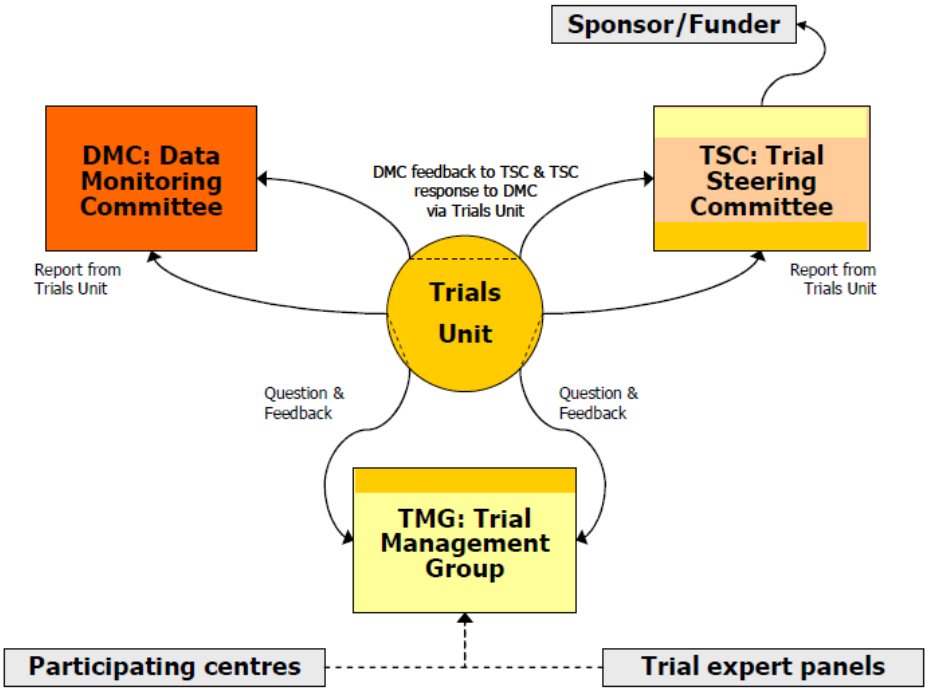

17 ANCILLARY STUDIES

17.1 QUALITY-OF-LIFE

A quality-of-life (QL) study is being performed to assess the impact of each treatment arm on the quality of patient's lives. Initial participation in this study was limited to the first 700 patients recruited (this was reached in Sep-2008) patients. The QL study re-opened from the implementation of Protocol version 8.0.

The EORTC QLQ-C30 with the prostate-specific module QLQ PR25 will be used. Key items for assessment are pain reduction for patients with metastatic disease and urinary symptoms for patients with locally-advanced disease. In addition specific hypotheses will be generated for each of the research arms. The EuroQol (EQ-5D) will be used in the study as a generic measure of health-related quality-of-life which can be linked to public preferences. These data will be used to calculate quality-adjusted life-years as part of the economic evaluation (see Section 17.2). **Patients recruited into the QL study, should continue to complete QL data for five years after randomisation or until progression, whichever is sooner.** Questionnaires should be self-administered, although it is recommended that a key person (e.g. research nurse) at each centre be responsible for the data collection to optimise compliance and completeness of the data.

The QL and the HE questionnaires should be completed by the patient without conferring with friends or relatives and all questions should be answered even if the patient feels them to be irrelevant.

The responsible person should check each questionnaire for its completeness, ensuring that the correct date of completion and patient identifiers are present. The research nurse should approach patients at appropriate clinical visits to complete a questionnaire. If no clinical visit is scheduled for the patient (with a window of 4 weeks around the expected date) the nurse should organise the completion of the questionnaire, by post or by a visit to the patient at home (or in a hospice).

17.2 HEALTH ECONOMICS

A health economics (HE) sub-study will be performed. Core resource use information will be collected, using CRFs on days in hospital (by speciality) and outpatient visits. Data collected on concomitant medication will also be used in the economic analysis. Information on patients' use of primary care and community-based services will be collected as additional questions in the QL questionnaire. Costs will be calculated on the basis of representative UK unit costs at the point of analysis. Health outcomes will be assessed in terms of quality-adjusted life years (QALYs). Quality adjustments will be based on patients' responses to the EQ-5D health status measure which will be administered at baseline and each point of follow-up as part of the QL questionnaire. A cost-effectiveness analysis will compare all regimens that continue to recruit into their final Efficacy Stage. For further details please refer to **Appendix G**.

17.3 TRANSLATIONAL SUB-STUDIES

Samples obtained from consenting STAMPEDE participants are analysed as part of separate translational sub-studies. These are conducted through collaborations with other academic and

industry partners. All applications for collaboration and sample accesss are reviewed by the STAMPEDE oversight committees and are overseen by the STAMPEDE BRG. For further details of each ongoing substudy please see **Section 4.6**. For details regarding sample collection please refer to the **Sample collection and handling manual** available via the website.

17.4 DISEASE VOLUMETRIC ANALYSIS SUB-STUDY

Baseline imaging obtained from STAMPEDE participants are accessed and analysed as part of the trial data collection. Collection and analysis will be undertaken in collaboration with partners on the TMG, initially, in ordered to determine disease volume. For details partaking to retrospective imaging centralisation and image handling, please refer to the individual sub study Working practices available from CTU. All subsequent applications for collaboration and imaging access are reviewed by the STAMPEDE oversight committees following the usual processes.

18 PUBLICATIONS

The results from different centres will be analysed together and published as soon as possible. Individual clinicians must not publish data concerning their patients that are directly relevant to questions posed by the study until the TMG has published its report. The TMG together with the STAMPEDE collaborators will form the basis of the writing committee and decide on the nature of publications. For the "transdermal oestradiol comparison", as the efficacy analyses will based on relevant data from the STAMPEDE and PATCH trials, TMGs for the two studies will form the writing committee. Any release, of efficacy or safety data, presentation or publication will be agreed with the TSC according to the terms of their charter.

All publications will acknowledge the participating centres and clinicians, and these will be detailed in an appendix to the main report. Papers will have named authors determined by the TMG according to the following principles:

- To be as inclusive as possible where this is practicable
- To ensure that there is justification for anyone to be named as an author

Reasons for nomination for authorship may include: trial design; grant holding; day-to-day trial oversight (TMG membership); analysis; discussion and interpretation of data; representation for key groups; active participation at large recruiting sites.

It should be accepted that the people qualifying for authorship will vary over time. In addition, key positions will vary depending on the nature of the publication: clinical lead for clinical papers, statistician lead for methodology papers, translational papers may be led by authors not on the main TMG if appropriate (e.g., the bone sub-study). In the event of any dispute related to authorship or data release, the TSC will be responsible for making the executive decision.

In the manuscript, a full list of sites and the number of patients recruited will be provided. In the presentations, this list of sites will also be shown. The term "the STAMPEDE investigators" will clearly be stated and relevant names included in the presentation credits.

A detailed **Publication Plan** is documented separately.

19 PROTOCOL AMENDMENTS

19.1 PROTOCOL

19.1.1 Amendments Made To Sections In Protocol Version 1.0 (May-2004)

Administrative changes such as typos, word change etc.

Name additions/changes to:

TMG members

TSC members

IDMC members

'General Information' Section – additional information re. Abridged version of protocol

Section 1.2 – Figure 1, Celecoxib duration amended

Section 1.3 – Figure 2, addition of cardiovascular assessment form, name and timings amended

Section 2.3 – Docetaxel information updated

Section 2.4 – Additional text re dose and duration justification for Celecoxib use.

Section 3 – Title change and content updated

Section 4.2 – New exclusion criteria added

Section 4.3.1 – New investigations added and additional text re testosterone measurements and additional text re. prior celecoxib treatment

Section 6.1.4 – Celecoxib duration amended

Section 6.1.5 – Additional text re. Co-administration of docetaxel and bisphosphonates

Section 6.1.6 – Celecoxib duration amended

Section 6.2.2 – additional docetaxel information

Section 6.2.3 – addition of CV event history

Section 11 – Safety reporting updated

Section 12.1 – Additional text re. the collection of blood for genetic and serum marker studies

Section 15 – Additional information re. Central Subvention for docetaxel arms

19.1.2 Amendments Made To Sections In Protocol Version 1.1 (May-2005)

Section 6.2 Administration and Dose Modifications, subsection 6.2.1 Zoledronic Acid

19.1.3 Amendments Made To Sections In Protocol Version 2.0 (Jun-2005)

General Information section – SAE reporting fax number and timeframe added.

Section 1.2 – Addition of anti-androgen use for M0 patients as a method of HT

Section 1.2 – Increase in amount of blood needed & addition tissue sample request.

Section 1.3 Trial Documentation updated to include new table detailing trial documentation ahead of accreditation, the inclusion of the radiotherapy forms and correct case report form timings

Section 2.1 – Addition of anti-androgen use for M0 patients as a method of HT

Section 4.1.3 – Inclusion criteria Vii "Normal testosterone prior to hormone treatment" removed.

Section 4.1.3 - φnote has been omitted and moved to section 4.2 (see number 8)

Section 4.2 – Exclusion criteria added to exclude patients with active peptic ulceration, gastrointestinal bleeding and inflammatory bowel disease.

Section 4.2 – Exclusion Criteria added to exclude patients with planned major dental work

Section 4.3.1 - All blood test timelines changed from 14 days to 28 days.

Section 4.3.1 – Hormone Therapy pre-randomisation deadline extended from 4 weeks to 12 weeks.

Section 4.3.1 – Additional information regarding the use of NSAIDs and cox-2-inhibitors before coming on to the STAMPEDE study and once commenced on study treatment

Section 4.3.2 – Updated to ask for all vitamins and minerals the patient is taking to be recorded.

Section 4.3.3 – Updated to include the extra blood required and the request for consent of patients' tissue samples.

Section 6.1.1 – Addition of anti-androgen use for M0 patients as a method of HT

Section 6.1.6 – Addition of the calcium & vitamin name "calcichew".

Section 6.6.2 – asking also to collect vitamins and minerals under concomitant medication.

Section 6.6.3 – New section to inform investigators that patient's, who they wish to give radiotherapy to, are also eligible for STAMPEDE

Section 6.6.4 – New section to detail what data is being collected on the radiotherapy given to patients.

Section 7.1; figure 4 – Addition of radiotherapy form and in note, addition of AA alone

Section 7.1.2 – omission of repeated scans and x-rays at 24 weeks, also omitted in note under figure 4.

Chapter 11 – Safety reporting section updated

Section 17.3 – Increase in amount of blood needed & additional tissue sample request.

19.1.4 Amendments Made To Section In Protocol Version 3.0 (Jul-2006)

Front Cover - NCRN logo added for accuracy

Front Cover - Clarification that protocol developed with NCRI rather than on behalf of

Front Cover - Clarification that it is a 6 arm trial

General Information section - MRC CTU staff section updated

Section 1.2 – Statistics section updated.

Section 1.2 - Additional research paragraph updated to reflect additional studies and for clarification of terms

Section 1.2 - Blood collection volume changed to reflect new technique used

Section 1.3 (figure 3) - Table showing case report form schedule updated to reflect clarification of follow-up schedule and addition of new CRF (End of Treatment)

Section 2.2 - AS changed to HT (clarification of terms)

Section 2.3 - Updated in information in regard to use of docetaxel added to reflect up to date practice

Section 2.5 - Sub-headings numbered for consistency

Section 3.0 - Information in regard to the Pilot Phase now written in past tense as Pilot Phase has now been completed

Section 4.1.1 - Inclusion criteria extended so that patients who fulfil 2 out of the three of the first inclusion criteria can be eligible.

Section 4.3.1 - Change in time scales by which baseline investigations need to be completed.

Section 4.3.1 - Clarification that chest X-ray is only required if chest is not included in the CT

Section 4.3.1 - Removal of 12 week timeline for baseline PSA test to be performed. (Stipulation that it must be performed before start of HT)

Section 4.3.2 – Information added in regard to time allowed from randomisation to start of treatment

Section 4.3.3 - Additional research paragraph updated to reflect additional studies and for clarification of terms

Section 4.3.3 - Blood collection volume changed to reflect new technique used

Sections 6.1.2-6.1.6 - Androgen Suppression replaced with hormone therapy for consistency of terms

Section 6.2.2 - '(Taxotere)' Removed for consistency

Section 6.2.2 _ information added in regard to the need to closely monitor liver function prior to docetaxel administration

Section 7.1 - Page number reference updated

Section 7.1.1 - PSA measurement timings updated to accurately reflect follow-up schedule

Section 7.3 (Table 4) - Table and key updated to accurately reflect follow-up schedule and to include information about new CRFs and removal of withdrawal CRF

Section 8 - Rewording for clarification of definition of trial withdrawal

Section 8.1 - Instruction that withdrawal from trial treatment should be recorded on End of Treatment Form rather than withdrawal form

Section 8.1 - Information updated to emphasise that trial treatment must be discontinued following a progression

Section 8.2- Information added in regard to patient transfers

Section 8.3 - Instruction that withdrawal from trial completely must be notified in writing to the MRC CTU rather than included on withdrawal form

Section 9 and Summary – Target event numbers updated to reflect the slightly revised numbers obtained by using –nstage- which is the new, recommended program for MAMS trials

Sections 11.1 and 11.2 - Form numbers removed to allow for future changes in numbering

Section 11.2 – Reference to toxicity grading website added

Section 11.2.1 - Reference to table in appendix G added

Section 12.2 - 'Suggested' removed from 'Suggested patient information sheets'

Section 13 - CTA reference added

Section 17.3 - Information added to reflect new blood collection method for DNA analysis and in regard to additional translational studies for which funding has recently been approved

19.1.5 Amendments Made To Protocol Version 4.0 (Dec-2007)

General Information Section - Randomisation and SAE reporting details sections clarified

Section 1.2 and throughout protocol - Efficacy Stages 1-111 renamed to Activity Stages 1-111 for accuracy and clarity

Section 1.2 - Follow schedule corrected

Section 4.1.2 - Inclusion criteria widened to include high risk relapsing patients, that would not have met the previous PSA based criteria

Section 4.1.3 - Note added to reference location of WHO performance status definitions

Section 4.2 - Notes added to reference locations of toxicity gradings and NYHA classifications

Section 4.3.1 - Timings of baseline scan information changed to accurately reflect most common current practice

Section 6.1.1 - Information about use of LHRH antagonists to ensure that the protocol accurately reflects current and future practice

Section 6.1.1 - Information about suggested duration of hormone therapy added to ensure that the protocol accurately reflects current practice

Section 6.2.2 - Additional information added about the timing of liver function tests prior to docetaxel administration added for clarity

Section 6.6.4 - Information on radiotherapy data collection added

Section 7.1.1 - Erroneous information about the timing of PSA measurements removed

Figure 3 - Moved to new section in protocol for clarity and extended to include current information on data collection

Figure 3b - Added to describe how extent of data collection during follow-up should change, post treatment and post progression

Figure 4 - Notes added to explain the changes in data collected at follow-up and to information that the quality-of-life study will be applicable to the first 700 patients randomised only

Figure 4 - Note added to include palliative radiotherapy CRF

Section 11.3 - SAE reporting information updated

Section 19 - Protocol amendments list updated

19.1.6 Amendments Made To Protocol Version 5.0 (Aug-2008)

1. General Information Section – Randomisation phone line number updated – non UK extension added
2. Section 3 – Information about QL study removed to reflect closure of QL study after first 700 patients
3. Section 4.2 – Exclusion criteria clarified to explain that only patients with severe poor cardiovascular history should be excluded
4. Section 4.3.1 – Information on co-administration of NSAIDS with celecoxib changed based on clinical advice.
5. Section 5 - Randomisation phone line number updated – non UK extension added
6. Section 6.2.1. – Information added to clarify that patients who develop an osteonecrosis of the jaw should stop zoledronic acid treatment
7. Section 6.2.3 – 'severe' text added to accurately reflect which patients should be excluded based on their cardiovascular history

8. Section 7.1.2 – Definition of disease progression extended for clarity

9. Figure 3 – Updated to include reference to newly created skeletal related event form

10. Figure 4 – Previous error in table amended to show that the 4th Zoledronic Acid form that is submitted contains information about 3 cycles rather than 2 as previously indicated

11. Table 4 – 'Other important medical condition' added to definition of serious in the SAE section, to accurately reflect SAE form and current practice

12. Section 11.1 – Information added on reporting or pregnancies

13. Section 17 - Information about QL study removed to reflect closure of QL study after first 700 patients

19.1.7 Amendments Made To Protocol Version 6.0 (Jul-2009)

1. General Information Section – Trial Pharmacist removed and changes of:

Co-Investigator

Patient Representatives

Trial Manager

Data Manager

General Information Section - Coordinating Centre – address change

General Information Section – change of Sponsor address

Section 1.1 – ratio of patients randomised to the investigational arms updated

Section 1.2 – figure 1b added to clarify trial design from Apr-2011 onwards

Section 1.2 – paragraph added to explain trial changes after the second activity analysis

Section 1.2 – wording added to clarify that QL data only collected for first 700 patients randomised

Section 1.3 – SSA Favourable Opinion removed from list of trial documentation required ahead of site accreditation

Section 2.1 – Amount of men diagnosed with prostate cancer annually updated

Section 2.4 –note added to explain completion of recruitment to celecoxib- containing arms

Section 2.5.2 - note added to explain completion of recruitment to celecoxib- containing arms

Section 3 – SSA Favourable Opinion removed

Section 4.2 – Exclusion criterion xiii greyed out

Section 4.3.1 – paragraph removed regarding potential randomisation to celecoxib-containing arms

Section 5 – Randomisation instructions expanded to exclude public holidays or dates when notice has been given by the CTU

Section 6.1.4 – formatting changed to grey font to reflect recruitment completion for arm D

Section 6.1.6 - formatting changed to grey font to reflect recruitment completion for arm F

Section 6.2.3 – recruitment note added

Section 6.6.3 – radiotherapy statement changed to reflect data from recent trials

Section 7.1.2 – removal of reference to SRE- specific CRF

Section 7.3 – Figure 3 - Addition of Bone Density Risk Factor Form and BMD sub-study assessment forms to summary of timing table

Section 7.3 – Figure 4 – Weeks added to timings of assessments post 2 years

Section 7.3- Figure 4 – note added to explain recruitment completion for arms D and F

Section 12.1 – Wording changed to reflect change to randomisation allocation ratio

Section 12.1 – Addition of statement regarding new information emerging during the trial

Section 12.2 – Reference to SSA removed

Section 16.3 – Statement added regarding actioning IDMC recommendation ahead of TSC ratification

19.1.8 Amendments Made To Protocol Version 7.0 (Jul--2011)

1. General Information Section- SAE reporting fax number corrected

2. Section 11- SAE reporting fax number corrected

19.1.9 Amendments Made To Protocol Version 7.1 (Jul-2011)

Throughout protocol – numbering has been updated in some sections new accommodate new information that has been added.

General Information Section – contact details updated

General Information Section – Funding information updated to include involvement from additional company

General Information Section – Wording on compliance and regulations updated to reflect current MRC CTU standard wording

General Information Section – Abbreviations list updated

Section 1.1 – The number of investigational agents being studied updated from three to four

Section 1.1 – Information regarding celecoxib updated to reflect that recruitment to these arms was discontinued in Apr-2011

Section 1.1 – Information about new IMP, Abiraterone inserted

Section 1.1 – Sample size and trial duration information updated to reflect changes brought about by additional trial arm

Section 1.2 – Summary information updated to reflect the discontinuation of recruitment to celecoxib arms and the addition of abiraterone

Figures 1a, b and c - Updated to reflect the discontinuation of recruitment to celecoxib arms and the addition of abiraterone

Section 1.2 – Information on trial stages updated to reflect changes brought about by additional trial arm

Section 1.2 – Information updated regarding the re-opening of the quality-of-life sub-study from implementation of protocol version 8.0

Section 2.1 – Wording related to hormone therapy updated for clarity

Section 2.1 - Updated to reflect the discontinuation of recruitment to celecoxib arms and the addition of abiraterone

Section 2.2 – Updated references added

Section 2.3 – Updated references added

Section 2.5 – Section added to give background information on new IMP, abiraterone

Section 2.6.1 – Updated references added

Section 2.7 – Section added to give information regarding radiotherapy which is to be given as part of standard care following recently published trial data.

Section 3 – Wording updated regarding selection of investigators to reflect current MRC CTU practice

Section 4.1 – Inclusion criteria updated with new criterion regarding radiotherapy use

Section 4.1 - Inclusion criteria updated with new criterion regarding contraceptive use

Section 4.1 – Wording of inclusion and exclusion criteria updated for clarity

Section 4.1 – Exclusion criteria updated with new criterion regarding acceptable liver function for trial entry

Section 4.1 – Exclusion criteria updated with specifics related to blood pressure levels

Section 4.1 - Exclusion criteria updated with new criterion regarding concomitant medications

Section 4.1 - Exclusion criteria updated with new criterion regarding prior treatment with abiraterone

Section 4.1 - Exclusion criteria updated with new criterion regarding prior treatment with chemotherapy

Section 4.1 - Exclusion criteria updated with new criterion regarding prior treatment with zoledronic acid

Section 4.3 – Wording updated to reflect that patients who initially fail screening can be re-screened at a later date

Section 4.3.2 – Wording updated regarding prior anti-androgen and LHRH use updated for clarity

Section 5.1 – Co-enrolment guidelines information updated to describe newly created co-enrolment CRF

Section 6.1 – Trial treatment information updated to reflect the fact that anti-androgens alone will be no longer permitted as hormone therapy

Section 6.1.1 – Updated to describe patients for whom radiotherapy should be given as standard practice

Section 6.1.1 a and b - Sections added to give information regarding radiotherapy treatment

Section 6.1.1-6.1.6 – References to further sections updated

Section 6.1.7 – Section added to describe abiraterone treatment

Section 6.2.4 - Section added to describe abiraterone treatment

Section 6.6 - Section added to give information regarding radiotherapy treatment

Section 7.1.1 – Reference to blood being taken at patient's home removed as this does not occur in practice

Section 7.1.2 – Wording updated regarding the reporting of biochemical failures for clarity

Section 7.1.2 – Wording updated regarding skeletal-related events for clarity

Section 7.1.3 – Section added to describe additional assessments required related to abiraterone treatment

Section 7.1.4 – Section added to provide information on when treatment should commence

Figure 4 – Updated for clarity regarding return of BMD sub-study forms, the addition the co-enrolment CRF and the description of the re-opening of the QoL Sub-study.

Figure 5 – Updated with reference to abiraterone and co-enrolment form

Section 7.3 - Wording on trial closure updated to reflect current MRC CTU standard wording

Section 8.1 – Additional criteria for definition of progression added for clarity

Section 8.1 – Definition of progression for abiraterone patients added.

Section 9 – Statistical information updated to describe the addition of the new trial arm

Section 11 – Safety reporting wording updated for clarity

Section 11 – SAE reporting fax number updated

Section 12 – Ethical information updated to describe the unequal randomisation allocation ratio

Section 12 – Ethical information updated to describe that the visit schedule will vary according to trial arm

Section 12.2 – Wording updated to reflect international participation in the trial

Section 13 – Wording updated to reflect international participation in the trial

Section 14 – Wording updated to reflect international participation in the trial

Section 15 - Updated to reflect the discontinuation of recruitment to celecoxib arms and the addition of abiraterone

Section 16 – Reference to trial committee charters added for information

Section 17.1 – Information added to reflect re-opening of quality-of-life sub-study

Section 17.2 – Timing of health economics analysis updated to previous error

Section 18 – Information on publication policy expanded for clarity

Section 19 – Information regarding amendments to protocol appendices moved to the separate appendices document

Section 20 – References extensively updated

19.1.10 Amendments Made To Protocol Version 8.0 (Sep-2011)

Throughout protocol – numbering, sections headings, tables, figures and bibliographical references have been updated in some sections to accommodate new information that has been added

Throughout protocol – Androgen Deprivation Therapy has replaced Hormone Therapy as deemed more representative of the type of hormone therapy used in the study

General Information Section – New staff members of the MRC CTU and Co-Investigators added and contact details updated

General Information Section – Abbreviations list updated

Section 1.1 – Information regarding the new research radiotherapy treatment inserted

Section 1.1 – Information regarding docetaxel updated

Section 1.2 – Wording updated to reflect the addition of the new research comparison arm

Section 1.3 – Additional criteria for the re-accreditation of participating centres (for protocol version 9.0 only)

Section 2.1.1 – Wording updated to clarify the use of anti-androgen in trial patients

Section 2.1.2 – Information added to describe the rationale for the RT comparison arm

Section 2.8 – Information added to describe research RT treatment to prostate for patients with newly diagnosed metastatic disease

Section 3.1 – Information added to describe RT Quality Assurance procedures and centre accreditation

Section 4.1.1 to 4.1.3 – Wording updated to clarify inclusion criteria for all patients groups (newly diagnosed non-metastatic, metastatic and relapsing patients)

Section 4.2 – Clarification added on cardiovascular exclusion criteria

Section 4.2 – New exclusion criterion added concerning patients with prior exposure to hormone therapy

Section 4.2 – New exclusion criterion added to reflect the addition of the new RT comparison arm

Section 4.4.1 – Clarification added regarding pre-randomisation checks

Section 4.4.2 – Clarification added regarding permissible hormone therapy duration prior to randomisation

Section 4.4.5 – Information added regarding starting research radiotherapy treatment

Section 4.4.6 – Information updated on concomitant medications

Section 5 – Clarification regarding randomisation allocation added to reflect the addition of the new RT research arm

Section 6.1.8 – Information added to describe the administration of research radiotherapy

Section 6.2.1 – Clarification added regarding the measurement of serum creatinine levels prior to the administration of zoledronic acid

Section 6.2.3 – Clarification regarding the completion of recruitment to the celecoxib containing arms

Section 6.25 – Information added regarding the administration of research radiotherapy treatment

Section 6.6 – Clarification incorporated to describe the administration of standard-of-care radiotherapy

Section 7.1.4 – Information added regarding data collection and non-administration of standard radiotherapy

Section 7.2 – Section updated to include new treatment specific CRFs and timing of CRFs

Section 8.1 – Clarification added for the criteria to stop treatment for patients randomised to arm G

Section 8.2 – Section expanded to include additional details on study patient transfer to different centres

Section 8.3 – Additional sentence inserted to reinforce the importance of compliance with follow-up assessments

Section 9.1 – Additional paragraph inserted to clarify the method of randomisation and allocation distribution in the light of the introduction of the new RT arm

Section 9.4 – Wording updated to clarify the assessment of safety data

Section 9.5.4 – Wording updated concerning the end of randomisations to arm G

Section 9.6 to 9.6.4 – Section added describing sample size issues and trial stages for arm H

Section 9.8 – Clarification on intermediate stopping guidelines

Section 9.9 – Clarification on the outline analysis plan

Section 11 – Information on safety reporting updated to reflect the addition of the research RT comparison arm

Section 11 – Clarification added regarding arm A safety reporting timelines

Section 12.1 – Clarification added regarding the Principal Investigator's responsibilities

Section 14 – Indemnity section updated to reflect current MRC policy

Section 16 – Clarification regarding TMG membership

Section 17.3 – Section on Bone Mineral Density sub-study removed

Section 19 – Information regarding amendments to protocol appendices moved to the separate appendices document

Section 20 – References updated

19.1.11 Amendments Made To Protocol Version 9.0 (Oct-2012)

Throughout protocol – numbering, sections headings, tables, figures and bibliographical references have been updated in some sections to accommodate the completion of recruitment to original research arms B, C and E.

Throughout protocol – Tenses have been changed to reflect activities that were in the future and which have now been passed.

Section 1 – Figure added and clarifications added to each figure

Section 2 – Previous reference 8 removed

Section 4 – Clarification of acceptable alternatives to bone scans

Section 6.2.5 – Correction of an error defining the PTV: the wording has been reordered

Table 4 – Dose-volume objectives corrected: order swapped

Table 5- Correction CRFs names

Section 17.3.2 – Clarification that DNA may be extracted

19.1.12 Amendments Made To Protocol Version 10.0 (Apr-2013)

Throughout protocol – numbering, sections headings, tables, figures and bibliographical references have been updated in some sections

Throughout protocol – typos have been corrected

Section 4 –Clarification of exclusion criteria V (now V and VI)

Section 6 – Timing of orchidectomy prior to randomisation extended to 12 weeks

Section 6 – Clarification of hypokalaemia, blood pressure and fluid retention management

Section 9 – Statistical considerations amended in light of the recruitment extension for the abiraterone comparison

Section 14 - Section updated to reflect the changes in the structure of the MRC CTU (now MRC CTU at UCL) and indemnity arrangements

19.1.13 Amendments Made To Protocol Version 11.0 (Sep-2013)

Throughout protocol – numbering, sections headings, tables, figures and bibliographical references have been updated in some sections

Throughout protocol – typos have been corrected

Co-investigators list updated to reflect the addition of the “enzalutamide + abiraterone comparison” lead

Section 1.2 – Enzalutamide added as trial treatment

Section 1.2 – Protocol version 12.0 added to the list of amendments

Section 2.10 – Rationale for the combination of enzalutamide and abiraterone

Section 4.2 – Eligibility criteria amended to reflect the addition of enzalutamide + abiraterone arm

Section 4.4.2 – Wording clarified

Section 6.8 – Clarification regarding end of trial treatment after starting trial therapy

Section 6.10 – Section added to describe enzalutamide and abiraterone treatment for the new research arm (Arm J)

Section 6.11.4.A – Section added to describe the management of toxicities from trial abiraterone

Section 6.11.4.B - Section added to describe the management of toxicities from trial enzalutamide

Section 9.1.4 – Section added to describe the statistical considerations concerning the introduction of Arm J

Section 9.3 – Principles and assumption for the introduction of Arm J added

Section 9.7 and sub-sections – Sample size issues and trial stages for Arm J

Section 9.9 – Details on interim monitoring and analyses for Arm J added

Section 11.2.1.D – Wording clarified regarding safety reporting requirements for control arm

Section 12.1 – Wording clarified

Section 15 – Details on funding for the “enzalutamide + abiraterone comparison” added

Section 19 - Amendments made to protocol updated

Reference list updated

19.1.14 Amendments Made To Protocol Version 12.0 (Jan-2014)

Throughout protocol – typos have been corrected

Section 4.4.2. Wording clarified

Section 4.3. Wording clarified for eligibility to M1|RT comparison

Section 6.10. Addition of use of dexamethasone post-biochemical progression for Arm J patients

Section 6.11.4.A. Correction of CTCAE version

Section 6.11.4.C. Clarification on enzalutamide dose modification to be in line with current SmPC

Section 9.6. Sample size increase for M1|RT comparison

Section 11. Correction of safety reporting timelines for Arm A patients

Section 17. Addition of saliva samples collection for DNA analysis

Table 4, 5 and 6. Clarification on Case Report Forms and Follow-up schedule

19.1.15 Amendments Made To Protocol Version 13.0 (Feb-2015)

Throughout protocol – typos have been corrected

Throughout protocol – clarification on the new definition of standard-of-care

Table of contents updated to reflect any changes to the protocol

Section 1.1. Wording added throughout section to include reference to survival results from "original comparisons"

Section 2.1.1. Section improved to include reference to survival results from "original research comparisons"

Section 2.1.2. Section improved to include reference to survival results from "original research comparisons".

Section 2.1.3. Additional section added to describe the role of docetaxel for men with M0 or M1 disease

Section 2.9. Clarification on treatment completion and primary results for "original research comparisons"

Section 4.2. Clarification of Exclusion criteria XIII and XVI

Section 4.4.2. Clarification on HT prior to randomisation

Section4.4.3. New section to clarify standard-of-care docetaxel treatment prior to randomisation

Section 4.4.7. Clarification on concomitant medication and contra-indicated concomitant medications

Section 4.5. Clarification provided on tissue block collection

Section 6. Inclusion of docetaxel into the standard-of-care

Section 6.2.3 New section to describe standard-of-care docetaxel administration

Section 6.11. Improvement throughout sections and sub-sections for abiraterone and enzalutamide-related toxicity management

Section 6.12. Section improved throughout to incorporate clearer details on concomitant medications and drug-to-drug interactions

Section 7.1.4. New section to describe data collection for standard-of-care docetaxel

Section 9.7.4. Clarification provided about implications for "enzalutamide+ abiraterone comparison" following change of standard-of-care treatment

Section 11.2.1.D Clarification on SAE notification timelines to reflect change in standard-of-care treatments (addition of docetaxel)

Figure 1. Figure updated to reflect change in standard-of-care

Figure 2. Figure updated to reflect trial history and recruitment over time

Figure 3. Figure updated to reflect changes in standard-of-care and recruiting arms

Table 1. Table updated to remove repetition

Table 13. Table updated to include new CRF to report standard-of-care docetaxel treatment

Table 15. Table updated to include only active trial treatments

19.1.16 Amendments Made To Protocol Version 14.0 (Oct-2015)

Throughout protocol – typos have been corrected

Throughout protocol – clarification on the new definition of standard-of-care

Table of contents updated to reflect any changes to the protocol

Section 1. Wording added throughout section to include reference "metformin comparison"

Section 2. Section updated to include reference "metformin comparison"

Section 4.2. Exclusion criteria review to reflect Arm J closure and instruction of "metformin comparison"

Section 4.3. Clarification of comparison specific eligibility (M1|RT and metformin)

Section 4.5.7. Clarification on concomitant medication and contra-indicated concomitant medications

Section 6. Treatment sections improved throughout

Section 6.11. Section updated to include details on metformin treatment

Section 6.12. Amendment throughout sections and sub-sections for metformin treatment

Section 6.13. Amendment throughout sections and sub-sections for metformin treatment

Section 6.13. Improvement throughout sections and sub-sections for abiraterone and enzalutamide treatment

Section 7.0. Amendment throughout sections and sub-sections to include assessment and procedures specific to “metformin comparison”

Section 9.0. Section updated and streamlined to capture statistical considerations on each comparison

Section 9.0. Details on “metformin comparison” added

Section 11. Safety processes updated and clarified

Section 16.0 Membership to oversight groups updated

Section 11.2.1.D Clarification on SAE notification timelines to reflect change in standard-of-care treatments (addition of docetaxel)

19.1.17 Amendments Made To Protocol Version 15.0 (Mar-2016)

Throughout protocol – re-structure of the treatment-related information for ease of use

Throughout protocol – clarification on the definition of standard-of-care

Throughout protocol – typos have been corrected

Addition of TMG members

Table of contents updated to reflect any changes to the protocol

New section for summary of trial added in table format

Section 1. Revised format for the summary of treatment groups, with the new transdermal oestradiol arm also added

Section 2. Clarification regarding research treatments that have previously reported or completed recruitment, section updated to include the “transdermal oestradiol comparison”

Section 3. New sections added for the “transdermal oestradiol comparison” and future planned biomarker-selected comparisons

Section 4.1.4. Change in definition of adequate renal function

Section 4.3. New section added for the biomarker-screening pilot, selection criteria removed for “research RT comparison”

Section 4.4.1. Change in definition of adequate renal function

Section 4.4.2. New section added for the patient selection criteria specific to the “transdermal oestradiol comparison”

Section 4.5. Screening procedure tables and figure added for clarification.

Section 4.5.1. New section added for biomarker-screening pilot investigations prior to randomisation.

Section 5.1.1. New section added for the biomarker-screening pilot registration.

Section 6. New sections added for the “transdermal oestradiol comparison”

Section 7. Amendment throughout sections and sub-sections to include assessment and procedures specific to “transdermal oestradiol comparison”

Section 7.1.4.B. Section added on cardiovascular outcomes for the “transdermal oestradiol comparison”

Table 18. Table added to clarify follow-up assessments

Section 8. Section updated for “transdermal oestradiol comparison”

Section 9. Section updated for “transdermal oestradiol comparison”

Section 12.1.1.D. Section added on participant feedback from investigations and additional analyses

Section 15. Section updated for “transdermal oestradiol comparison” and biomarker-screening pilot

19.1.18 Amendments Made To Protocol Version 16.0 (Oct-2017)

Summary of trial- Table 1: Schedule of Assessments has been added

Abbreviations & Glossary- new terms have been added

Section 1- Table 4: Abiraterone information updated as results of primary analysis published

Section 4.3 - Biomarker timelines redefined, the length of prior hormone therapy has increased to reflect change in turnaround time for testing

Section 4.6 - Biomarker screening information updated

Section 6.2 – Clarification on safety monitoring required for patients receiving trial abiraterone added . Abiraterone overdose information altered for clarity.

Section 6.3.4 - Drug interactions updated to specify that tamoxifen is contraindicated in combination with abiraterone, enzalutamide and transdermal oestradiol.

Section 6.5 – Detail on requirements at site to demonstrate compliance with per-protocol required safety monitoring added

Section 7 – Schedule for assessments updated, removal of table 19

Section 7.1 - Clarification on additional safety monitoring required for patients receiving trial abiraterone added

Section 7.4 - Table 20 QoL information removed and added to Table 1: Schedule for Assessments

Section 10.1.1- Central monitoring of consent information added

Section 11 – Re-structured and re-worded for clarity on reporting requirements for safety data captured on the SAE CRF. Explanation provided for exempted events and definitions added. Table 28 and Box 1 updated and Figure 1 added.

Section 11.2- Updated SAE exceptions, SAE flow chart added for clarity

Section 11.3 - Update of investigator assessments and notification checklist for expedited safety reporting

Section 11.4 - Update of wording of CTU responsibilities

Section 17.4 - Sub-study information added to include Disease Volumetric sub-study

20 REFERENCES

1. James ND, Sydes MR, Clarke NW, Mason MD, Dearnaley DP, Spears MR, et al. Addition of docetaxel, zoledronic acid, or both to first-line long-term hormone therapy in prostate cancer (STAMPEDE): survival results from an adaptive, multiarm, multistage, platform randomised controlled trial. Lancet. 2016;387(10024):1163-77.

2. Mason MD, Clarke NW, James ND, Dearnaley DP, Spears MR, W.S.R. A, et al. Adding celecoxib with or without zoledronic acid for hormone-naive prostate cancer: long-term survival results from an adaptive, multi-arm, multi-stage, platform, randomised controlled trial. 2017. In press. DOI: 10.1200/JCO.2016.69.0677.

3. James ND, de Bono JS, Spears MR, Clarke NW, Mason MD, Dearnaley DP, et al. Abiraterone for Prostate Cancer Not Previously Treated with Hormone Therapy. N Engl J Med. 2017;377(4):338-51.

4. Mason MD, Clarke NW, James ND, Dearnaley DP, Spears MR, Ritchie AWS, et al. Adding Celecoxib With or Without Zoledronic Acid for Hormone-Naive Prostate Cancer: Long-Term Survival Results From an Adaptive, Multiarm, Multistage, Platform, Randomized Controlled Trial. J Clin Oncol. 2017;35(14):1530-41.

5. Cancer Research UK. CancerStats Key Facts: Prostate Cancer. Cancer Research UK. 2011.

6. Sharifi N, Gulley JL, Dahut WL, Sharifi N, Gulley JL, Dahut WL. An update on androgen deprivation therapy for prostate cancer. Endocrine-Related Cancer. 2010;17(4):R305-15.

7. Widmark A, Klepp O, Solberg A, Damber JE, Angelsen A, Fransson P, et al. Endocrine treatment, with or without radiotherapy, in locally advanced prostate cancer (SPCG-7/SFUO-3): an open randomised phase III trial. Lancet. 2009;373(9660):301-8.

8. Warde PR, Mason MD, Sydes MR, Gospodarowicz MK, Swanson GP, Kirkbride P, et al. Intergroup randomized phase III study of androgen deprivation therapy (ADT) plus radiation therapy (RT) in locally advanced prostate cancer (CaP) (NCIC-CTG, SWOG, MRC-UK, INT: T94-0110; NCT00002633). J Clin Oncol. 2010;28(18s Supplement: Proceedings of ASCO 2010):Abstr CRA4504.

9. Warde P, Mason M, Ding K, Kirkbride P, Brundage M, Cowan R, et al. Survival Benefit with Combined Androgen Deprivation and Radiation Therapy in Locally Advanced Prostate Cancer – Results of a Phase III Trial. The Lancet. 2011 - in press.

10. Mason M, Sydes M, Parulekar W, Parmar M, Anderson J, Barber J, et al. Final analysis of intergroup randomized phase III study of androgen deprivation therapy (ADT) + radiation therapy (RT) in locally advanced prostate cancer (CaP) (NCIC-CTG, SWOG, MRC-UK, INT: T94-0110). National Cancer Research Institute (NCRI) Cancer Conference 2012. 2012;2012.

11. Warde P, Mason M, Ding K, Kirkbride P, Brundage M, Cowan R, et al. Combined androgen deprivation therapy and radiation therapy for locally advanced prostate cancer: a randomised, phase 3 trial. The Lancet. 2011;378:2104-11.

12. Nicholas D James MRS, Noel W Clarke, David P Dearnaley, Malcolm D Mason, Christopher C Parker, Alastair W S Ritchie, J. Martin Russell, Francesca Schiavone, Gerhardt Attard, Johann S de Bono, Alison Birtle,Daniel S Engeler,Tony Elliott, David Matheson, Joe O'Sullivan, Delia Pudney, Narayanan Srihari, Jan Wallace, Jim Barber, Isabel Syndikus, Mahesh K B Parmar, Matthew R Sydes. Failure-free

survival and the impact of radiotherapy in patients with newly diagnosed non metastatic prostate cancer: Data from patients in the control arm of the STAMPEDE trial (MRC PR08, CRUK/06/019). JAMA Oncology. 2015.

13. Sweeney CJ, Chen YH, Carducci M, Liu G, Jarrard DF, Eisenberger M, et al. Chemohormonal Therapy in Metastatic Hormone-Sensitive Prostate Cancer. N Engl J Med. 2015;373(8):737-46.

14. Vale CL, Burdett S, Rydzewska LH, Albiges L, Clarke NW, Fisher D, et al. Addition of docetaxel or bisphosphonates to standard of care in men with localised or metastatic, hormone-sensitive prostate cancer: a systematic review and meta-analyses of aggregate data. The lancet oncology. 2016;17(2):243-56.

15. James ND, Sydes MR, Clarke NW, Mason MD, Dearnaley DP, Spears MR, et al. Addition of docetaxel, zoledronic acid, or both to first-line long-term hormone therapy in prostate cancer (STAMPEDE): survival results from an adaptive, multiarm, multistage, platform randomised controlled trial. Lancet. 2015.

16. Attard G, Richards J, de Bono JS, Attard G, Richards J, de Bono JS. New strategies in metastatic prostate cancer: targeting the androgen receptor signaling pathway. Clinical Cancer Research. 2011;17(7):1649-57.

17. Fizazi K, Scher HI, Molina A, Logothetis CJ, Chi KN, Jones RJ, et al. Abiraterone acetate for treatment of metastatic castration-resistant prostate cancer: final overall survival analysis of the COU-AA-301 randomised, double-blind, placebo-controlled phase 3 study. The lancet oncology. 2012;13(10):983-92.

18. Rathkopf DE, Smith MR, de Bono JS, Logothetis CJ, Shore ND, de Souza P, et al. Updated interim efficacy analysis and long-term safety of abiraterone acetate in metastatic castration-resistant prostate cancer patients without prior chemotherapy (COU-AA-302). European urology. 2014;66(5):815-25.

19. Flanigan RC, Salmon SE, Blumenstein BA, Bearman SI, Roy V, McGrath PC, et al. Nephrectomy followed by interferon alfa-2b compared with interferon alfa-2b alone for metastatic renal-cell cancer. N Engl J Med. 2001;345(23):1655-9.

20. Mickisch GH, Garin A, van Poppel H, de Prijck L, Sylvester R. Radical nephrectomy plus interferon-alfa-based immunotherapy compared with interferon alfa alone in metastatic renal-cell carcinoma: a randomised trial. Lancet. 2001;358(9286):966-70.

21. Attard G, Cooper CS, de Bono JS. Steroid hormone receptors in prostate cancer: a hard habit to break? Cancer cell. 2009;16(6):458-62.

22. Chen G, Wang X, Zhang S, Lu Y, Sun Y, Zhang J, et al. Androgen receptor mutants detected in recurrent prostate cancer exhibit diverse functional characteristics. The Prostate. 2005;63(4):395-406.

23. Taplin ME, Bubley GJ, Ko YJ, Small EJ, Upton M, Rajeshkumar B, et al. Selection for androgen receptor mutations in prostate cancers treated with androgen antagonist. Cancer Res. 1999;59(11):2511-5.

24. Veldscholte J, Ris-Stalpers C, Kuiper GG, Jenster G, Berrevoets C, Claassen E, et al. A mutation in the ligand binding domain of the androgen receptor of human LNCaP cells affects steroid binding characteristics and response to anti-androgens. Biochemical and biophysical research communications. 1990;173(2):534-40.

25. Zhao XY, Malloy PJ, Krishnan AV, Swami S, Navone NM, Peehl DM, et al. Glucocorticoids can promote androgen-independent growth of prostate cancer cells through a mutated androgen receptor. Nature medicine. 2000;6(6):703-6.

26. Attard G, Reid AH, Auchus RJ, Hughes BA, Cassidy AM, Thompson E, et al. Clinical and biochemical consequences of CYP17A1 inhibition with abiraterone given with and without exogenous glucocorticoids in castrate men with advanced prostate cancer. The Journal of clinical endocrinology and metabolism. 2012;97(2):507-16.

27. Richards J, Lim AC, Hay CW, Taylor AE, Wingate A, Nowakowska K, et al. Interactions of abiraterone, eplerenone, and prednisolone with wild-type and mutant androgen receptor: a rationale for increasing abiraterone exposure or combining with MDV3100. Cancer Res. 2012;72(9):2176-82.

28. Scher HI, Fizazi K, Saad F, Taplin ME, Sternberg CN, Miller K, et al. Increased survival with enzalutamide in prostate cancer after chemotherapy. N Engl J Med. 2012;367(13):1187-97.

29. Tombal B, Borre M, Rathenborg P, Werbrouck P, Heidenreich A, Iversen P, et al. Enzalutamide monotherapy: Phase II study results in patients with hormone-naive prostate cancer. Journal of Clinical Oncology. 2013;31(Supplement 6):Abstract 18.

30. Efstathiou E, Titus MA, Tsavachidou A, Hoang A, Karlou M, Wen S, et al. MDV3100 effects on androgen receptor (AR) signaling and bone marrow testosterone concentration modulation: A preliminary report. . 2011 ASCO Annual Meeting, 2011 J Clin Oncol 2011.

31. Locke JA, Guns ES, Lubik AA, Adomat HH, Hendy SC, Wood CA, et al. Androgen levels increase by intratumoral de novo steroidogenesis during progression of castration-resistant prostate cancer. Cancer Res. 2008;68(15):6407-15.

32. Tran C, Ouk S, Clegg NJ, Chen Y, Watson PA, Arora V, et al. Development of a second-generation antiandrogen for treatment of advanced prostate cancer. Science. 2009;324(5928):787-90.

33. Scher HI, Beer TM, Higano CS, Anand A, Taplin ME, Efstathiou E, et al. Antitumour activity of MDV3100 in castration-resistant prostate cancer: a phase 1-2 study. Lancet. 2010;375(9724):1437-46.

34. Smith MR, Finkelstein JS, McGovern FJ, Zietman AL, Fallon MA, Schoenfeld DA, et al. Changes in body composition during androgen deprivation therapy for prostate cancer. The Journal of clinical endocrinology and metabolism. 2002;87(2):599-603.

35. Eriksson A, Attvall S, Bonnier M, Eriksson JW, Rosander B, Karlsson FA. Short-term effects of metformin in type 2 diabetes. Diabetes, obesity & metabolism. 2007;9(4):483-9.

36. Bailey CJ. Treating insulin resistance in type 2 diabetes with metformin and thiazolidinediones. Diabetes, obesity & metabolism. 2005;7(6):675-91.

37. Ohira M, Miyashita Y, Ebisuno M, Saiki A, Endo K, Koide N, et al. Effect of metformin on serum lipoprotein lipase mass levels and LDL particle size in type 2 diabetes mellitus patients. Diabetes research and clinical practice. 2007;78(1):34-41.

38. Wulffele MG, Kooy A, de Zeeuw D, Stehouwer CD, Gansevoort RT. The effect of metformin on blood pressure, plasma cholesterol and triglycerides in type 2 diabetes mellitus: a systematic review. Journal of internal medicine. 2004;256(1):1-14.

39. Lubik AA, Gunter JH, Hendy SC, Locke JA, Adomat HH, Thompson V, et al. Insulin increases de novo steroidogenesis in prostate cancer cells. Cancer Res. 2011;71(17):5754-64.

40. Venkateswaran V, Haddad AQ, Fleshner NE, Fan R, Sugar LM, Nam R, et al. Association of diet-induced hyperinsulinemia with accelerated growth of prostate cancer (LNCaP) xenografts. J Natl Cancer Inst. 2007;99(23):1793-800.

41. Noto H, Goto A, Tsujimoto T, Noda M. Cancer risk in diabetic patients treated with metformin: a systematic review and meta-analysis. PloS one. 2012;7(3):e33411.

42. Rothermundt C, Hayoz S, Templeton AJ, Winterhalder R, Strebel RT, Bartschi D, et al. Metformin in chemotherapy-naive castration-resistant prostate cancer: a multicenter phase 2 trial (SAKK 08/09). European urology. 2014;66(3):468-74.

43. Langley RE, Cafferty FH, Alhasso AA, Rosen SD, Sundaram SK, Freeman SC, et al. Cardiovascular outcomes in patients with locally advanced and metastatic prostate cancer treated with luteinising-hormone-releasing-hormone agonists or transdermal oestrogen: the randomised, phase 2 MRC PATCH trial (PR09). The lancet oncology. 2013;14(4):306-16.

44. Byar DP. Proceedings: The Veterans Administration Cooperative Urological Research Group's studies of cancer of the prostate. Cancer. 1973;32(5):1126-30.

45. Hedlund PO, Henriksson P. Parenteral estrogen versus total androgen ablation in the treatment of advanced prostate carcinoma: effects on overall survival and cardiovascular mortality. The Scandinavian Prostatic Cancer Group (SPCG)-5 Trial Study. Urology. 2000;55(3):328-33.

46. Gilbert DC, Duong T, Kynaston HG, Alhasso AA, Cafferty FH, Rosen SD, et al. Quality-of-life outcomes from the Prostate Adenocarcinoma: TransCutaneous Hormones (PATCH) trial evaluating luteinising hormone-releasing hormone agonists versus transdermal oestradiol for androgen suppression in advanced prostate cancer. BJU international. 2016.

47. Smith DC, Redman BG, Flaherty LE, Li L, Strawderman M, Pienta KJ. A phase II trial of oral diethylstilbesterol as a second-line hormonal agent in advanced prostate cancer. Urology. 1998;52(2):257-60.

48. de Bono JS, Logothetis CJ, Molina A, Fizazi K, North S, Chu L, et al. Abiraterone and increased survival in metastatic prostate cancer. New England Journal of Medicine. 2011;364(21):1995-2005.

49. Ryan CJ, Smith MR, de Bono JS, Molina A, Logothetis CJ, de Souza P, et al. Abiraterone in metastatic prostate cancer without previous chemotherapy. N Engl J Med. 2013;368(2):138-48.

50. Janssen Research & Development L. ZYTIGA® (abiraterone acetate) Investigator Brochure. 13 ed2017.

51. Fizazi K, Tran N, Fein L, Matsubara N, Rodriguez-Antolin A, Alekseev BY, et al. Abiraterone plus Prednisone in Metastatic, Castration-Sensitive Prostate Cancer. N Engl J Med. 2017;377(4):352-60.

52. Lipska KJ, Bailey CJ, Inzucchi SE. Use of metformin in the setting of mild-to-moderate renal insufficiency. Diabetes care. 2011;34(6):1431-7.

53. Salpeter SR, Greyber E, Pasternak GA, Salpeter EE. Risk of fatal and nonfatal lactic acidosis with metformin use in type 2 diabetes mellitus: systematic review and meta-analysis. Archives of internal medicine. 2003;163(21):2594-602.

54. Royston P, Parmar MKB, Qian W. Novel designs for multi-arm clinical trials with survival outcomes with an application in ovarian cancer. Statistics in Medicine. 2003;22(14):2239-56.

55. Royston P, Barthel FMS, Parmar MKB, Choodari-Oskooei B, Isham V. Designs for clinical trials with time-to-event outcomes based on stopping guidelines for lack of benefit. Trials. 2011;12(1):81.

56. Royston P. nstage: MAMS trial sample size calculator. MRC Clinical Trials Unit, London2009.

57. Mason MD, Parulekar WR, Sydes MR, Brundage M, Kirkbride P, Gospodarowicz M, et al. Final Report of the Intergroup Randomized Study of Combined Androgen-Deprivation Therapy Plus Radiotherapy

Versus Androgen-Deprivation Therapy Alone in Locally Advanced Prostate Cancer. J Clin Oncol. 2015;33(19):2143-50.

58. James ND, Sydes MR, Mason MD, Clarke NW, Anderson J, Dearnaley DP, et al. Celecoxib plus hormone therapy versus hormone therapy alone for hormone-sensitive prostate cancer: first results from the STAMPEDE multiarm, multistage, randomised controlled trial. The lancet oncology. 2012;13(5):549-58.

59. Haybittle JL. Repeated assessment of results in clinical trials of cancer treatment. The British journal of radiology. 1971;44(526):793-7.

60. Peto R, Pike MC, Armitage P, Breslow NE, Cox DR, Howard SV, et al. Design and analysis of randomized clinical trials requiring prolonged observation of each patient. I. Introduction and design. British journal of cancer. 1976;34(6):585-612.
